# IL-17 production by tissue-resident MAIT cells is locally induced in children with pneumonia

**DOI:** 10.1038/s41385-020-0273-y

**Published:** 2020-02-28

**Authors:** Bingtai Lu, Ming Liu, Jun Wang, Huifeng Fan, Diyuan Yang, Li Zhang, Xiaoqiong Gu, Junli Nie, Zhenjun Chen, Alexandra J. Corbett, Michael J. Zhan, Shengbo Zhang, Vanessa L. Bryant, Andrew M. Lew, James McCluskey, Hai-bin Luo, Jun Cui, Yuxia Zhang, Yifan Zhan, Gen Lu

**Affiliations:** 1 Department of Respiratory Medicine, Guangzhou Institute of Pediatrics, Guangzhou Women and Children’s Medical Centre, State Key Laboratory of Respiratory Diseases, Guangzhou Medical University, Guangzhou, Guangdong China; 2 School of Life Sciences, Sun Yat-sen University, Guangzhou, Guangdong China; 3 School of Pharmaceutical Sciences, Sun Yat-sen University, Guangzhou, Guangdong China; 4 Department of Immunology and Microbiology, The Peter Doherty Institute for Infection and Immunity, University of Melbourne, Melbourne, VIC Australia; 5 The Melbourne Medical School, University of Melbourne, Melbourne, VIC Australia; 6 Walter and Eliza Hall Institute of Medical Research, Parkville, VIC Australia; 7 Department of Medical Biology, University of Melbourne, Parkville, VIC Australia

## Abstract

Community-acquired pneumonia (CAP) contributes substantially to morbidity and mortality in children under the age of 5 years. In examining bronchoalveolar lavages (BALs) of children with CAP, we found that interleukin-17 (IL-17) production was significantly increased in severe CAP. Immune profiling showed that mucosal-associated invariant T (MAIT) cells from the BALs, but not blood, of CAP patients actively produced IL-17 (MAIT17). Single-cell RNA-sequencing revealed that MAIT17 resided in a BAL-resident PLZF^hi^CD103^+^ MAIT subset with high expression of hypoxia-inducible factor 1α (HIF-1α), reflecting the hypoxic state of the inflamed tissue. CAP BALs also contained a T-bet^+^ MAIT1 subset and a novel DDIT3^+^ (DNA damage-inducible transcript 3-positive) MAIT subset with low expression of HIF1A. Furthermore, MAIT17 differed from T-helper type 17 (Th17) cells in the expression of genes related to tissue location, innateness, and cytotoxicity. Finally, we showed that BAL monocytes were hyper-inflammatory and elicited differentiation of MAIT17. Thus, tissue-resident MAIT17 cells are induced at the infected respiratory mucosa, likely influenced by inflammatory monocytes, and contribute to IL-17-mediated inflammation during CAP.

## Graphical abstract

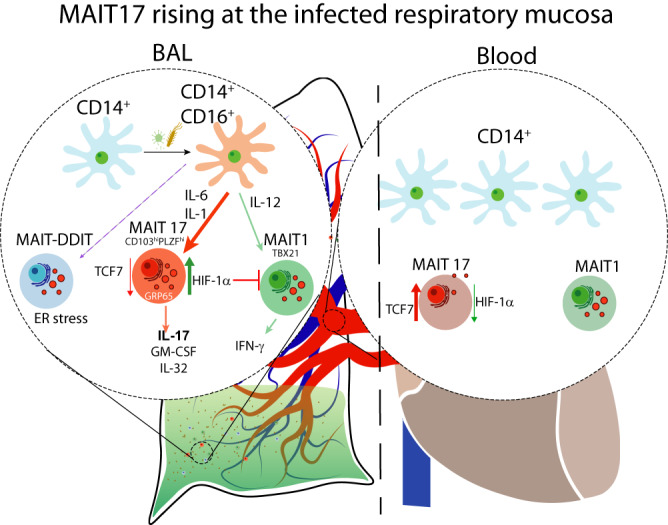

## Introduction

Mucosal-associated invariant T (MAIT) cells are innate T cells that are abundant in mucosal sites and comprise about 5% of human blood T cells^1^. Their semi-invariant T cell receptors (TCRs) recognize microbial riboflavin metabolite-based antigens presented on major histocompatibility complex class I-related protein-1 (MR1)^2,3^. MAIT cell-deficient MR1^−/−^ mice show impaired control of bacterial and viral infections with *Legionella longbeachae*^4^, *Mycobacterium bovis* Bacillus Calmette–Guérin^5^, *Francisella tularensis* live vaccine strain^6^, and influenza A^7^. There have been many reports intimating a role of MAIT cells in anti-microbial immunity in humans. Patients with active tuberculosis^5^, intensive care unit-acquired infections^8^, and HIV infections^9,10^ have decreased numbers of MAIT cells in the blood. MAIT cells are also implicated in the pathogenesis of human diseases^11^ and mucosal immune response to bacterial infections^12^. For example, *Mr1*^−/−^ mice showed significantly less gastric pathology during *Helicobacter pylori* infection^13^. It has not been resolved whether immune defense and immunopathology mediated by MAIT cells can be distinguished molecularly.

Human MAIT cells are known for their ability to produce interferon-γ (IFN-γ) and interleukin-17 (IL-17)^1^, although MAIT cells at mucosal sites have higher potential for IL-17 production than their circulating counterparts^14–16^. In general, molecular and cellular basis for the tissue difference in IL-17 production by MAIT cells remains to be unraveled. IL-17 expression by mouse CD4^+^ T-helper type 17 (Th17) cells is regulated by certain transcription factors, including RAR-related orphan receptor γt (RORγt)^17^, RORA^18^, basic leucine zipper ATF-like transcription factor (BATF)^19^, and signal transducer and activator of transcription 3 (STAT3)^20^, as well as other factors such as hypoxia-inducible factor-1 (HIF-1)^21^ and the aryl hydrocarbon receptor (AhR)^22^. Circulating human MAIT cells express RORγt and display a mixed IFN-γ and IL-17 expression pattern upon in vitro TCR stimulation^1^. In individuals with loss-of-function mutations in STAT3 but intact RORγt expression, MAIT cells have impaired IL-17 expression^23^. Overall, the transcriptional machinery governing IL-17 expression in MAIT cells (MAIT17) and how these cells are induced at mucosal sites have not been specified.

Community-acquired pneumonia (CAP) is an immune-mediated lung disease caused by a wide variety of microbial pathogens. As a substantial cause of morbidity and mortality in children under 5 years of age, CAP remains a major public health burden, particularly in developing countries^24–26^. Severe CAP is associated with acute respiratory and cardiovascular failure, multiple organ dysfunction, and high mortality^27^. The proportions of CD4^+^ T cells that secrete IL-17A and IL-22 are increased in bronchoalveolar lavages (BALs) in adult CAP patients compared to healthy controls^28^. The contribution by MAIT cells to IL-17 production and to CAP (both adult and pediatric) has not been fully defined. We opine that this may be important, because IL-17 as well as having a role in defending against infections has been cogently linked to immunopathology in both mice and humans, including respiratory infections^29–31^.

In this study, we examined the inflammatory mediators at both systemic and local pulmonary levels in a cohort of children hospitalized with CAP. We found that BAL IL-17 levels correlated with disease severity and that MAIT cells in BALs, but not in blood, were primed for IL-17 production. Bulk RNA-sequencing (RNAseq) analysis revealed that BAL MAIT cells expressed higher levels of transcription factors that promote IL-17 production, while blood MAIT cells expressed higher levels of negative regulators of IL-17 production, like TCF7^32^. Single-cell RNAseq (scRNAseq) showed that MAIT17 cells are encompassed within a population of cells with high expression of PLZF, CD103, and HIF-1α. They also uniquely expressed the pathogenic Th17 marker GPR65, cytotoxic molecule IL-32, lymphotoxin Β, and granulysin. In addition, BAL MAIT cells contained two additional subsets: a T-bet^+^ MAIT1 subset and a novel MAIT subset that expresses the DNA damage-inducible transcript 3 (DDIT3; also termed CHOP), a pro-apoptotic transcription factor elicited during endoplasmic reticulum stress. Finally, we showed that BAL monocytes actively secreted inflammatory cytokines and elicited MAIT17 differentiation. These findings would be consistent with a pathogenetic pathway whereby pulmonary infection recruits and stimulates monocytes to promote MAIT17 differentiation during CAP.

## Results

### Levels of IL-17 and other inflammatory cytokines were higher in the BALs of children with CAP and corresponded with CAP severity

We recruited 187 hospitalized children with CAP at the Respiratory Clinic of a tertiary pediatric hospital in Southern China. CAP severity was classified according to high-resolution computed tomography (HRCT) scores (HRCT-CAP) ranging from 1 to 6 as specified in Supplementary Fig. 1A, B. Children with severe CAP were those who had HRCT scores ≥4. Severe and not-severe (HRCT scores <4) CAP were defined for 92 and 95 children, respectively (Supplementary Table 1). Non-CAP control subjects were those without a concurrent infection but required surgical removal of inhaled foreign objects. Children with severe CAP had significantly prolonged fever, cough, and hospitalization. Around 80% of CAP patients were diagnosed with at least one known pathogen, with adenovirus and *Mycoplasma pneumoniae* being the most common. Infections with multiple pathogens were more common in children with severe CAP.

**Fig. 1:**
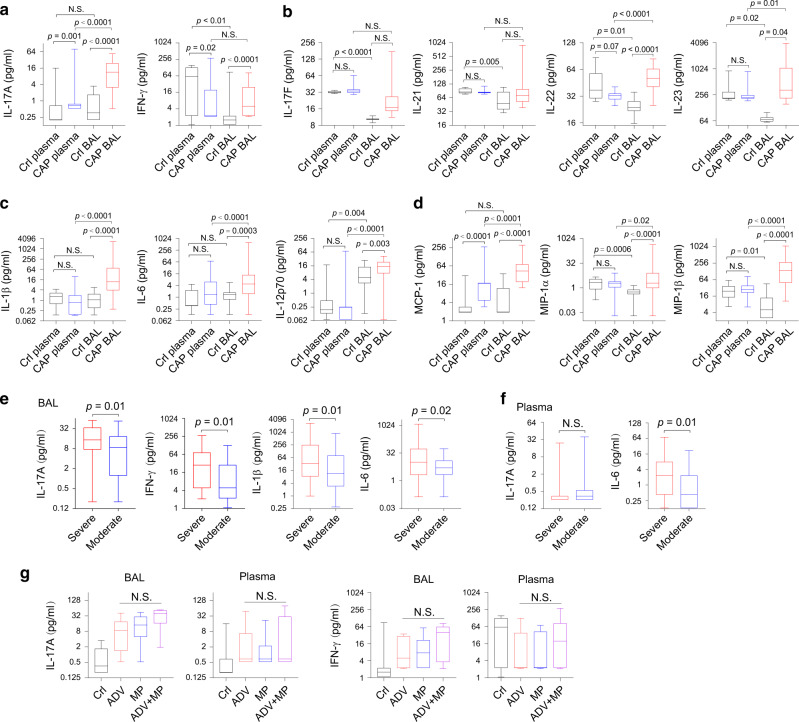
IL-17 and related cytokines are prominently presented in BAL fluids. **a**, **c**–**g** Concentrations of cytokines from plasma and BAL fluid as determined by Bio-Plex are shown as the range and min to mix boxes with horizontal lines within representing median. **a** Concentrations of IL-17A and IFN-γ. Cytokines from paired plasma and BAL samples of CAP (plasma and BAL; *n* = 70) and non-CAP control subjects (Crl plasma and Crl BAL; *n* = 16). **b** Th17-associated cytokine levels in paired plasma and BAL samples from CAP patients and control subjects (CAP, *n* = 27; controls, *n* = 9). **c**, **d** Levels of monocyte-derived cytokines and chemokines. **e** BAL cytokine levels and disease severity (severe *n* = 51, moderate *n* = 41). **f** Plasma cytokine levels and disease severity (severe *n* = 35, moderate *n* = 35). **g** Levels of IL-17A or IFN-γ in BAL or plasma in controls (Crl; *n* = 16) or in CAP patients infected with adenovirus (ADV; *n* = 15) or *Mycoplasma pneumoniae* (MP; *n* = 14) or both (*n* = 9). *P* values were calculated by paired *t* test (plasma vs. BAL, **a**–**d**) or unpaired *t* test (CAP vs. control, **a**–**d**); unpaired *t* test (severe vs. moderate, **e**, **f**); and Kruskal–Wallis test for **g**. NS, not significant.

We first evaluated the concentrations of inflammatory mediators in matched plasma and BAL samples from CAP (*n* = 70) and non-CAP control subjects (*n* = 16). Compared with plasma cytokine concentrations, we found that the concentrations of IL-17A and IFN-γ were significantly higher in the BALs in children with CAP (Fig. 1a). Other IL-17-related cytokines, IL-22 and IL-23, measured in a separate blood and BAL-matched cohort (CAP, *n* = 27; control, *n* = 9) were also significantly elevated in BALs of CAP patients (Fig. 1b). Levels of several innate cytokines, including those influencing T cell differentiation (IL-1β, IL-6, IL-12p70) (Fig. 1c) and chemotaxis (monocyte chemotactic protein-1 [MCP-1], macrophage inflammatory protein-1α [MIP-1α], and MIP-1β), were also significantly higher in the BAL than in the plasma (Fig. 1d). Compared with BAL in CAP patients, BAL in control subjects had markedly lower concentrations of most inflammatory mediators.

We then determined whether the levels of BAL inflammatory mediators corresponded with CAP severity. In an expanded cohort including unmatched BAL and plasma samples, we found that compared with BALs of children with non-severe CAP, BALs of children with severe CAP had significantly increased concentrations of IL-17A, IFN-γ, IL-1β, and IL-6 (Fig. 1e). Plasma IL-6 was also significantly higher in severe than in not-severe CAP (Fig. 1f). For those patients with identifiable pathogens, we did not detect significant differences between the types of pathogen (whether adenovirus or *Mycoplasma pneumoniae*) with the cytokine levels in the BAL (Fig. 1g). Overall, IL-17 and other inflammatory cytokines were elevated in CAP BALs, compared with CAP plasma or control BALs.

### MAIT and Th17 cells are prominent sources of IL-17 in the BALs of children with CAP

**Fig. 2:**
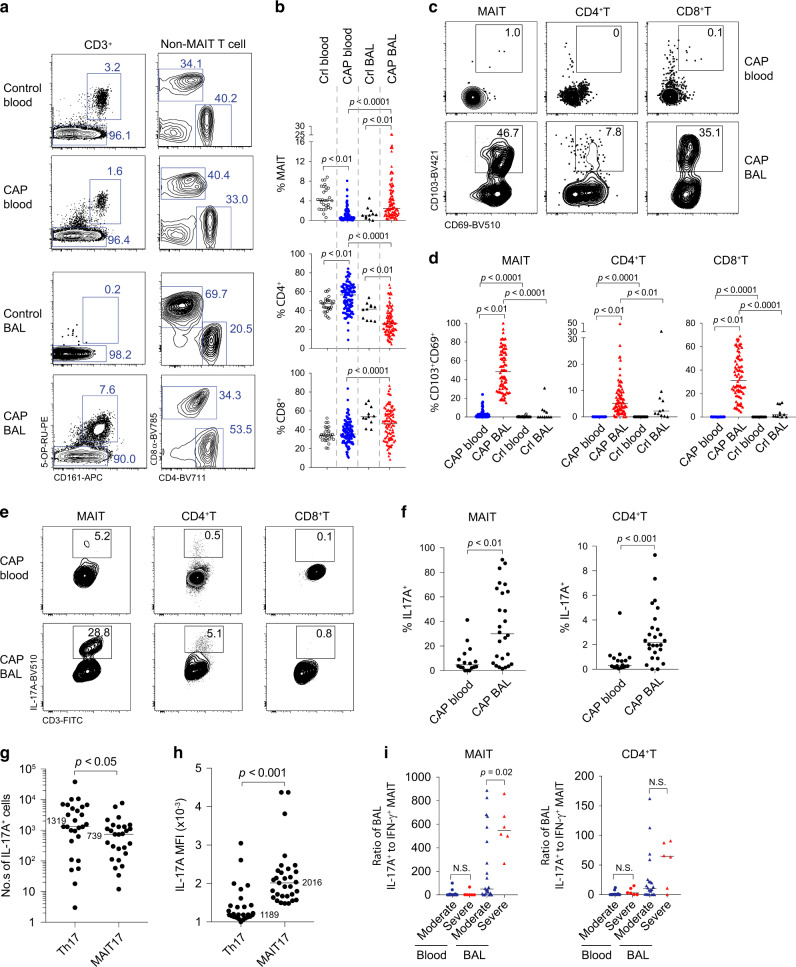
BAL MAIT cells are prominent IL-17 producing cells. CD3^+^ T cells from blood of control (*n* = 29) and CAP subjects (*n* = 110), and from BAL of control (*n* = 11) CAP subjects (*n* = 100) were analyzed for T cell subsets by flow cytometry. **a** CD3^+^ T cells were first gated for MAIT by MR1-5-OP-RU tetramer and CD161 staining (left). Non-MAIT CD3^+^ T cells were then gated as CD4^+^ and CD8^+^ (right). **b** Frequency of T cell subsets as a proportion of total CD3^+^ T cells in the blood and BAL of CAP and control (Crl) subjects; *p* values from unpaired *t* tests are shown. **c**, **d** Frequency of T cells expressing the tissue-resident markers CD103 and CD69 are shown; *p* values from unpaired *t* tests are shown. **e** Intracellular IL-17A staining was performed for T cells. FACS plots show IL-17A^+^ cells within indicated subsets. **f** Data showing frequency of IL-17A-producing cells in the indicated subsets from blood (*n* = 21) and BAL (*n* = 27) of CAP patients. **g** Data showing the number of IL-17A-producing MAIT and CD4^+^ T cells in the BAL. **h** Data showing mean fluorescent intensity (MFI) of IL-17A within the IL-17-producing cells. **i** The correlation of the ratio of IL-17A^+^ to IFN-γ^+^ T cells to disease severity. Horizontal lines represent median and *p* values from Mann–Whitney *U* tests are shown. NS, not significant.

To identify the cellular sources of IL-17, we examined the T cell compartment from CAP patients (110 blood and 100 BAL samples). MAIT cells were identified by the expression of CD3, CD161, and reactivity to MR1-5-OP-RU tetramer, or by the expression of CD3, TCRVα7.2, and CD161. A similar proportion of MAIT cells was identified with either staining method (Supplementary Fig. 2A), thus affording a level of cross-validation. In blood, the proportions of MAIT cells within the CD3^+^ T cell compartment were significantly lower in CAP patients than those in control subjects. In contrast, in BAL, this was reversed, that is, MAIT cell proportions were significantly higher in CAP patients than in control subjects (Fig. 2a, b). Compared to the control BALs, the number of MAIT cells was significantly increased in the BALs of CAP patients (Supplementary Fig. 2B). The abundance of blood MAIT cells did not significantly alter between acute infection and at discharge (Supplementary Fig. 2C).

Phenotypically, around 50% of MAIT cells in the BALs of CAP patients expressed the tissue-resident markers CD69 and CD103, whereas these markers were barely detectable in blood MAIT cells of CAP patients or BAL MAIT cells of the controls (Fig. 2c, d). We further analyzed the CD69 and CD103 expression of MAIT subsets, and found similar expression levels in the CD8^+^ and CD8^lo^CD4^lo^ populations (Supplementary Fig. 2D). For conventional T cells, we observed reduced CD4^+^ T and increased CD8^+^ T cells in the BALs (Fig. 2a, b). Larger proportions of conventional CD8^+^ T cells (median 34%) expressed tissue-resident markers CD69 and CD103 compared to CD4^+^ T cells (median 7%) in the BALs.

Next, we determined IL-17 expression by BAL T cells. To do this, we stimulated the cells ex vivo with phorbol myristate acetate (PMA) and ionomycin. In the BALs of CAP patients, 30% of MAIT cells and 2% of conventional CD4^+^ T cells produced IL-17A (Fig. 2e, f). Although the total number of Th17 cells was more abundant than the MAIT17 cells, IL-17 was expressed at significantly increased levels in MAIT17 cells as indicated by the mean fluorescent intensity (Fig. 2g, h). In contrast, in the blood of CAP patients, 5% of MAIT cells and <1% of conventional T cells expressed IL-17A (Fig. 2e, f). Of note, there was a lower frequency of IFN-γ^+^ cells in BAL MAIT cells than blood MAIT cells in CAP patients (Supplementary Fig. 2E, F). In addition, we found that MAIT cells from control blood contained a high proportion of IFN-γ^+^ cells but a low proportion of IL-17A^+^ cells (Supplementary Fig. 2G, H). There were no sufficient numbers of MAIT cells from control BALs for functional evaluation.

Despite the known fact that blood MAIT cell frequencies increase with age in early life^33^, the frequency of BAL IL-17A^+^ MAIT cells significantly declined with age. There was no significant change in IFN-γ^+^MAIT cells in CAP patients with increasing age (Supplementary Fig. 2I). The frequency of IL-17A^+^ or IFN-γ^+^ T cell subsets in BALs did not significantly distinguish between severe and not-severe CAP (Supplementary Fig. 2J). This was potentially due to limited study subjects and large age differences. However, we found that MAIT cells in severe CAP subjects had a significantly increased IL-17^+^ to IFN-γ^+^ ratio when compared to not-severe CAP (Fig. 2i). Of note, we observed no difference in cytokine production by T cells, whether the type of pathogen was adenovirus or mycoplasma (Supplementary Fig. 2K). Together, MAIT17 skewing may be reflective of immunopathology.

### BAL MAIT cells express high levels of IL-17-related cytokines and transcription factors

**Fig. 3:**
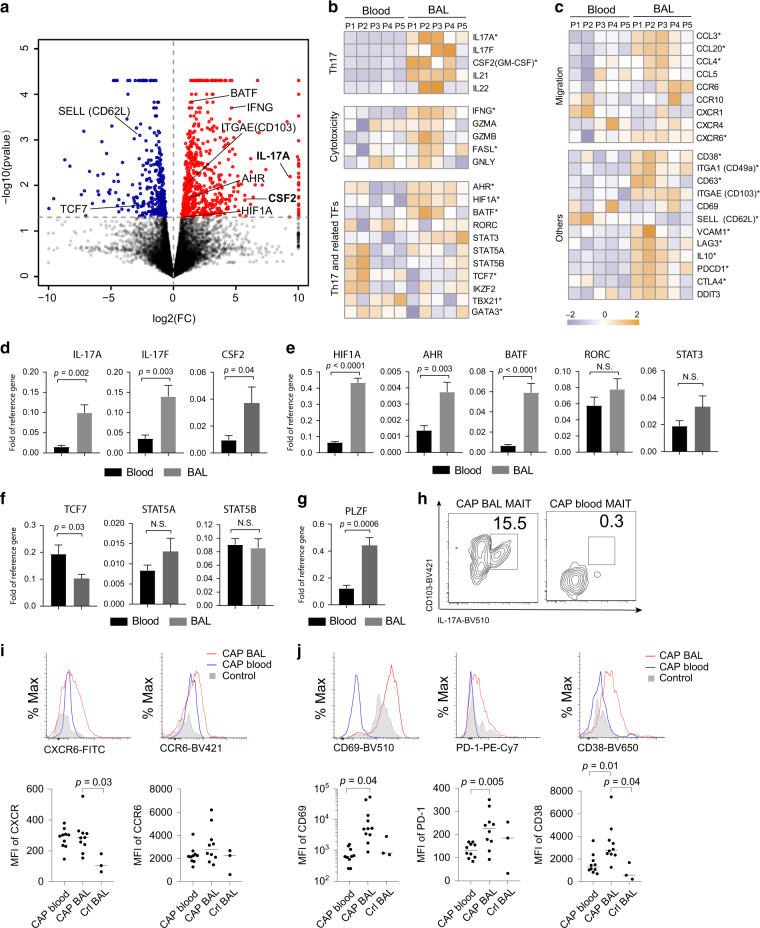
BAL MAIT cells express Th17-related cytokines and transcriptional factors. **a** A volcano plot showing differential expression of genes in BAL vs. blood MAIT cells from CAP patients (*n* = 5). Red and blue points mark the genes with significantly increased or decreased expression respectively in BAL compared to blood samples (FDR < 0.05). The *x*-axis shows log_2_ fold changes in expression and the *y*-axis the log_10_ *p* value of genes being differentially expressed. **b**, **c** Heat map shows log_2_ gene expression of various clusters of interest: Th17 cytokines, IFN-γ and cytotoxic molecules, Th17 and related transcriptional factors, chemokines/chemokine receptors, and others, including tissue residency and activation. * indicates *p* < 0.05 by *t* test. **d**–**g** Comparison of the expression levels of Th17-related genes and transcription factors between blood and BAL by RT-PCR (*n* = 8). *P* values were calculated with the unpaired *t* test. **h** FACS plots show correlation of CD103 expression to IL-17A production by MAIT cells from BAL and blood of CAP patients. **i**, **j** Histograms showing expression of indicated markers by MAIT cells from BAL (*n* = 10) and blood (*n* = 10) of CAP patients. Controls (*n* = 3) were MAIT cells from control BAL. NS, not significant.

To provide further insight into why blood and BAL MAIT cells differ in the ability to produce IL-17, we performed bulk RNAseq using MAIT cells isolated from blood and BALs from five CAP patients. Compared to blood MAIT cells, BAL MAIT cells significantly upregulated 483 genes and down-regulated 233 genes (≥2 fold; Fig. 3a and Table S2). At the transcriptional level, BAL MAIT cells expressed significantly higher levels for IL-17-related genes, for example, *IL17A*, *CSF2*, and IL-21 (*p* < 0.05; multivariate power analysis using multivariate analysis of variance)^34^ as well as *IFNG* (Fig. 3b). Among several transcription factors promoting Th17 differentiation, expression of *HIF1A*, *AHR*, *BATF*, and *STAT3* was significantly higher in BAL MAIT cells while expression of *RORC* was not (Fig. 3b). Of note, *TCF7, a* transcription factor suppressing Th17 differentiation was highly expressed in blood MAIT cells (Fig. 3b). Blood and BAL MAIT cells also showed differences in expression of many genes related to chemokine/chemokine receptors, T cell activation, and exhaustion (Fig. 3c). In an independent cohort (*n* = 8), we performed quantitative polymerase chain reaction (qPCR) to validate differences on IL-17-related cytokines and regulatory genes. Compared with blood MAIT cells, BAL MAIT cells from CAP patients expressed markedly higher levels of IL-17A, IL-17F, and CSF2 (Fig. 3d) and Th17-promoting transcriptional factors HIF1A, AHR, and BATF (Fig. 3e). Differences in RORC and STAT3 between blood and BALs were not significant. Among the three tested Th17-suppressing transcriptional factors, we observed significantly decreased expression of TCF7 in BAL MAIT cells compared with blood (Fig. 3f). *PLZF* regulates CD103 expression^35^; its increase in BAL MAIT cells is consistent with the finding that IL-17-producing MAIT cells express CD103 (Fig. 3g, h). Flow cytometric analysis also confirmed higher expression of chemokine receptors CXCR6, and markers of cell activation (CD38, CD69, PD-1) by BAL MAIT cells in comparison with blood MAIT cells from CAP patients (Fig. 3i, j). Although high expression of CD69 was observed on the surface of BAL MAIT cells detected by flow cytometry (Fig. 3j), the levels of CD69 messenger RNA (mRNA) were not upregulated (Fig. 3c). This discrepancy in protein and mRNA level may be due to the transient expression and rapid degradation of CD69 mRNA^36,37^.

### Tissue-resident MAIT cells with an IL-17-promoting gene signature is identified as one of the three subsets of MAIT cells in the BALs

**Fig. 4:**
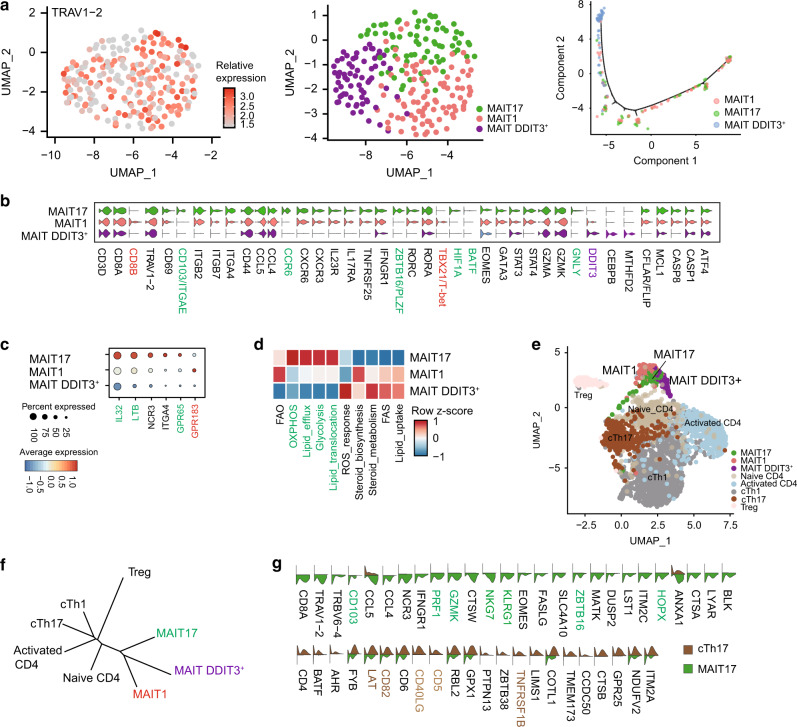
MAIT cells in BALs of CAP children contain distinct clusters. MAIT and CD4^+^ T cells isolated from the BALs of children with CAP were analyzed by single-cell RNA-sequencing (scRNAseq). **a**, Left panel: two-dimensional uniform manifold approximation and projection (2D-UMAP) plot displaying T cells expressing *TRAV1-2*; middle panel: unsupervised graphic clustering partitioned those cells into MAIT17, MAIT1, and *DDIT3*^+^ MAIT; right panel: pseudotime cell trajectory of MAIT cells. **b** Violin plots displaying expression of marker genes across MAIT cell clusters. **c** Dot plot showing expression of *IL-32*, *LTB*, *NCR3*, *ITGA4*, *GPR65*, and *GPR183* among MAIT cell clusters. Dot size reflects the percentage of cells expressing the gene, while color intensity reflects the average gene expression level. **d** Heat map plotting of metabolic expression programs among MAIT subsets. **e** 2D-UMAP plot displaying three MAIT cell clusters and five CD4^+^ T. **f** Unrooted distance tree showing transcriptional similarities between CD4^+^T subsets and MAIT cell clusters. **g** Violin plots displaying differentially expressed genes between MAIT17 and Th17.

Accumulating evidence indicates that MAIT cells also exhibit lineage differentiation and functional specialization^38^. Using ROR-γt and T-bet reporter mouse models, MAIT1 and MAIT17 cells have been identified in rodents^38^. However, alignment with their human counterparts is not straightforward. We performed scRNAseq on MAIT cells enriched from the BALs of children with CAP. MAIT cells were defined by co-expression of *TRAV1-2* (TCRVα7.2) and CD161 (Fig. 4a, left panel); 228 high-quality single-cell transcriptomes of MAIT cells were identified. Using unsupervised graphic clustering, those cells were partitioned into three subsets (Fig. 4a, middle and right panel). MAIT17 cells expressed Th17-associated genes (*IL23R*^+^*IL17RA*^+^*RORC*^+^*RORA*^+^*HIF1A*^+^*STAT3*^+^) and the tissue-resident marker CD103; MAIT1 cells, although also expressing *RORC*, expressed *TBX21*/*T-bet*, *STAT4*, and CD8B (Fig. 4b and Supplementary Fig. 4A); and a novel MAIT subset characterized by high expression of *DDIT3* showed low expression of *RORC* (Fig. 4b). We also noted that MAIT17 cells had higher expression of *PLZF*, a transcription factor that has been reported to regulate CD103 expression in natural killer T cells^35^ (Fig. 4b). Intracellular staining of PLZF protein showed that MAIT cells, but not conventional T cells, expressed PLZF (Supplementary Fig. 4B).

Moreover, MAIT17 cells preferentially expressed *IL32* and *LTB* (lymphotoxin B), and the pathogenic Th17 cell marker *GPR65*^30^ (Fig. 4c). MAIT1 cells expressed GRP183, an oxysterol-sensing molecule involved in cell migration and inflammation^39^ (Fig. 4c). Metabolic gene set analysis showed that MAIT17 cells were enriched in genes encoding glycolysis, oxidative phosphorylation, and lipid efflux, while the remaining MAIT subsets were enriched in genes encoding lipid and steroid responses (Fig. 4d). DDIT3^+^ MAIT cells were characterized by high expression of genes related to reactive oxygen species (ROS) responses and expression of genes that would promote cell survival, for example, decrease in CASP8 and CFLAR (encoding FLIP) (Fig. 4b, d).

As CD4^+^ Th17 cells were also present in the BALs of pediatric and adult CAP patients^28^, we analyzed the functional difference of MAIT17 and Th17 cells in BAL. Analysis of differentially expressed genes (DEGs) between MAIT17 and Th17 cells showed that MAIT17 were PLZF^+^ and CD103^+^ in the BAL (Fig. 4e, f). MAIT17 cells also highly expressed characteristic “innateness” markers from innate lymphocytes (*ZBTB16*/*PLZF*, *NKG7*, *KLGR1*, *NCR3*, and *HOPX*)^40^, cytotoxicity (*GZMK* and *PRF1*), and chemotaxis *CCL4* and *CCL5* (Fig. 4g). In contrast and as expected, Th17 cells expressed T cell activation-related genes *CD40LG*, *CD5*, *CD82*, *LAT*, and *TNFRSF1B* (Fig. 4g). Differences in transcription factors that positively regulate IL-17 production were unremarkable between MAIT and Th17 cells (Fig. 4G), perhaps suggesting functional convergence under the same inflammatory milieu.

### Inflammatory cytokines derived from the BAL CD14^+^ monocytes promote pathogenic MAIT17 differentiation

**Fig. 5:**
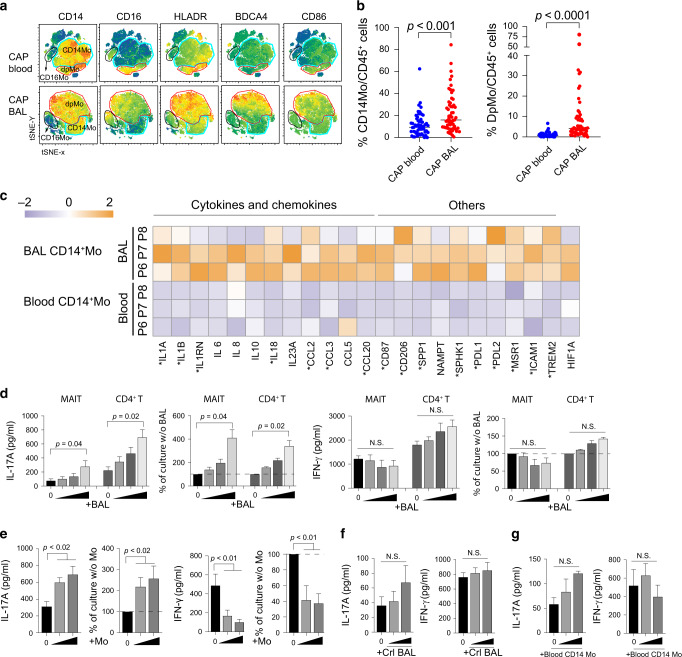
BAL fluid and BAL monocytes of CAP patients are Th17 promoting. **a** tSNE analysis of concatenate innate cells (depleted of granulocytes and T, B, and NK cells) showing the monocyte/macrophage phenotypes in blood and BAL of children with CAP (*n* = 10). **b** Proportions of CD14^+^ and CD14^+^CD16^+^ double positive (DpMo) monocytes (blood *n* = 55 and BAL *n* = 57) within the live CD45^+^ population were shown for blood and BALs. Horizontal lines represent the median; *p* values were calculated using the unpaired *t* test. **c** Heat map shows log_2_ gene expression of Th17-inducing cytokines and T cell activation. * indicates *p* < 0.05 by *t* test. **d** Effect of CAP BALs on differentiation of T cells to MAIT17 or Th17 cells. MAIT and CD4^+^ T cells isolated from buffy coat of normal subjects were stimulated with CD3/28 beads and co-cultured with BAL supernatant from CAP patients for 3 days (*n* = 4). Cytokine concentrations and levels as a percentage of those cultured without BALs are shown. It should be noted the maximum level of IL-17 contamination by BAL itself was <10 pg/ml and thus insubstantial. **e** Effect of BAL CD14^+^ monocytes on differentiation of MAIT17. MAIT cells extracted from buffy coats were co-cultured with CD3/CD28 beads and BAL CD14^+^ monocytes for 3 days (*n* = 4). **f** Effect of control BALs on the differentiation of MAIT to MAIT17 cells (*n* = 8). **g** Effect of blood CD14^+^ monocytes on the differentiation of MAIT to MAIT17 cells (*n* = 6), *p* values were calculated with unpaired *t* test (**e**–**g**).

Classically, pathogenic Th17 differentiation is driven by inflammatory cytokines (e.g., IL-1, IL-6, and IL-23) derived from myeloid cells;^29^ the BALs contained high levels of such cytokines in CAP patients (Fig. 1b, c). Hence, we investigated whether monocytic cells might be responsible for MAIT17 induction. We observed that CD14^+^ monocytes, particularly CD14^+^CD16^+^ subset were significantly enriched in the BALs than in blood (Fig. 5a, b). *T*-distributed stochastic neighbor embedding (tSNE) analysis of concatenate monocytic populations (depleted of granulocytes and T, B, natural killer (NK) cells) using CD11c, CD14, CD163, BDCA-4, HLA-DR, CD16, CD86, and FcεRIα parameters revealed that BAL CD14^+^ cells showed significantly increased expression of CD16, HLA-DR, BDCA-4, and CD86 compared with their blood counterparts (Fig. 5a, b), indicating a hyper-inflammatory state of the BAL CD14^+^ monocytes. Next, bulk RNAseq also revealed higher expression of IL-17-promoting cytokines in the CD14^+^ monocytes in the BALs than in blood (Fig. 5C).

The above findings reveal a possible interaction of BAL monocytes and the differentiation of MAIT17 cells. We showed that BAL fluids from CAP patients strongly promoted IL-17 secretion by MAIT and CD4^+^T cells in vitro upon TCR stimulation with anti-CD3/CD28, even though the cells were derived from the blood of normal subjects (Fig. 5D). Of note, IFN-γ production was not promoted by BAL fluids in T cells (Fig. 5D). To delineate the possible cytokines in BALs promoting MAIT17 defferentiation, we added neutralizing antibodies (Abs) to IL-1β and IL-23 to MAIT cell cultures with BALs. We found that Abs to IL-1β and IL-23 reduced IL-17A production by MAIT cells upon TCR stimulation (Supplementary Fig. 4C). Finally, we showed that BAL CD14^+^ monocytes isolated from BALs of CAP patients directly increased IL-17 but suppressed IFN-γ production by blood MAIT cells from healthy subjects (Fig. 5e). Overall, these findings indicate that BAL CD14^+^ monocytes and their products favored induction of MAIT17 and Th17 in children with CAP. BAL fluid from control patients and CD14^+^ monocytes from CAP blood, were less potent to promote IL-17 production by MAIT cells (Fig. 5f, g).

## Discussion

Here we showed that IL-17 level was elevated locally in padiatric CAP patient and associated with disease severity. Investigation of IL-17 producers identified that BAL MAIT cells but blood MAIT cells were the major contributors. Unique expression of several transcription factors (e.g., HIF1A, BATF, and AHR) that promote IL-17 production by BAL MAIT cells with high expression of tissue-resident markers differeniate them from blood counterparts. ScRNAseq further partitioned BAL MAIT cells into three functional subsets, including a novel DDIT3^+^ subset. Finally, we provided evidence that induction of MAIT17 at the infected respiratory mucosa is likely the consequence of heightened inflammation, exemplified with high local concentration of inflammatory cytokines and monocytes.

Characterization of MAIT cells of blood and BALs showed a decrease in the abundance of blood MAIT cells in CAP patients compared to control subjects. This agrees with decreased numbers of MAIT cells in the blood, observed in patients with active tuberculosis^5^, intensive care unit-acquired infections^8^, and HIV infections^9,10^. On the other hand, the frequency of MAIT in the BAL seems to be more variable. A recent study showed that the frequencies of MAIT in the BAL of pulmonary tuberculosis patients were significantly decreased^41^. In contrast, Lezmi et al.^42^ reported that BAL MAIT frequencies were elevated in children with asthma and MAIT17 was further increased in severe cases. Here we showed that there was an increase in the abundance of MAIT cells in CAP BALs over control BALs. We reason that abundance of MAIT cells at the affected sites could be determined by multiple factors, such as age, genetics, and disease causes/status. At the cellular level, abundance of MAIT cells at the affected sites could also be determined by recruitment, proliferation, and cell death. These aspects remain to be illustrated.

The cellular and molecular basis for the difference in IL-17 production by blood and tissue MAIT cells is less clear. One possibility is that functional subsets of MAIT cells may distribute diversely^38,43,44^. Interestingly, we also noted that blood MAIT cells, while lower in IL-17 production, showed “readiness” for IFN-γ production, as MAIT cells of CAP patients as well as control subjects contained large proportion of IFN-γ-producing cells upon stimulation with PMA and ionomycin.

Although MAIT17 also differed from Th17 cells in multiple genes regulating tissue residency, innateness, and cytotoxicity, key transcriptional programs that govern IL-17 expression were shared between MAIT17 and Th17 cells. RORγt is highly expressed by human MAIT cells^1^ and typifies MAIT17 cells^38^. STAT3 is also required for human MAIT cells to produce IL-17, independent of RORγt^23^. Apart from RORγt and STAT3, the BAL MAIT cells of CAP patients also had robust expression of other Th17-associated molecules such as AHR, HIF1A, and BATF. Furthermore, we show that blood MAIT cells of CAP patients expressed higher levels of TCF7, which has been shown to suppress Th17 differentiation^32,45,46^. From these findings, one could infer that blood MAIT cells are less differentiated and that a set of molecular factors co-ordinate BAL MAIT cells to differentiate into a MAIT17 phenotype.

Our scRNAseq analysis of BAL MAIT cells also revealed a cohort of MAIT cells with high DDIT3 (CHOP) expression. DDIT3 is a key player in the ER stress response^47^. Notably, DDIT3^+^ MAIT cells expressed higher levels of ROS response genes. A recent study showed that DDIT3 negatively regulates CD8^+^ T cell responses^48^. The functional relevance of DDIT3 expression by the MAIT subset deserves future investigation.

We suggest that MAIT17 induction is likely promoted by monocytes activated in situ, that is, BAL monocytes (or their products in BAL fluid) but not blood monocytes. Our investigation of BAL myeloid cells revealed that CD14^+^ monocytes (most with CD16 expression) highly expressed genes supporting MAIT17 and Th17 differentiation. A group of cytokines supporting Th17 differentiation is highly expressed by BAL monocytes but not blood monocytes. We also found that BAL monocytes promoted differentiation of MAIT17 but suppressed MAIT1. As HIF-1α suppressed IL-12 production by dendritic cells to hamper Th1 differentiation^49^, it is interesting to note that BAL monocytes expressed higher levels of HIF1A. Overall, our findings indicate that BAL CD14^+^ monocytes and their products favored induction of MAIT17 and Th17. Thus, it is plausible that the highly inflammatory environment with elevated concentrations of MCP-1 and MIP-1 α/β could recruit monocytes to the inflammatory tissues. In turn, accumulated inflammatory monocytes and their derived cytokines subsequently promote MAIT17 differentiation locally. Despite the different etiological pulmonary infections mediating the local activation of monocytes, the overall levels of IFN-γ and IL-17 and the T cell subsets producing these cytokines did not significantly change. Although the pathogen-sensing mechanisms differed at early infection, we speculate that these infections finally resulted in a similar profile of inflammatory cytokines, which then shape the adaptive system. Neither adenovirus nor mycoplasma synthesize riboflavin, the source of the vitamin-based MAIT cell antigens. Thus, in this setting MAIT cells were likely activated through commensal microorganisms or co-infecting bacteria in combination with inflammatory cytokines.

Th17 cells have been suggested to contribute to CAP pathogenesis in adult patients^28^. Here we showed that MAIT17 cells are a major contributors of IL-17 in children with CAP, particularly in early childhood. However, while involvement of MAIT cells in anti-microbial immunity and pathogenesis of human diseases is implicated^11^, direct evidence that MAIT cells mediate immune defense and immunopathology in human diseases including CAP is still lacking. Of note, scRNAseq analysis showed that MAIT17 from CAP patient BAL expressed GRP65. GPR65 is a G protein-coupled receptor for the glycosphingolipids including psychosine^50^. It is considered to be a pathogenic Th17 marker by promoting excess granulocyte–macrophage colony-stimulating factor secretion in patients with spondyloarthritis^51^ and has been found to regulate Th17 genes^30^. BAL MAIT17 cells also showed higher expression of IL-32 and lymphotoxin B, mediators linked to protection against pathogens and immunopathology^52,53^. Together, we speculate that MAIT17 cells at the infection sites could contribute to pathology. Therefore, these disease-associated biomarkers may aid to further delineate the role of human MAIT cells in disease settings.

In summary, MAIT cells are increasingly appreciated as participants in immune protection and the pathogenesis of human infectious and non-infectious diseases^11^. IL-17 production is a characteristic and yet enigmatic feature of MAIT cells. Here we demonstrated using multiple approaches that human MAIT17 cells only become highly functional at the interface of respiratory infection. The induction of MAIT17 is likely instructed by local pathogen-activated monocytes and derived cytokines. An unresolved issue is whether tissue MAIT17 represents expansion of local-resident or circulating populations. In either case, induced MAIT17 cells likely contribute to BAL inflammation and disease severity in children with CAP. As such, we envisage that pathogenic MAIT17 may be a propitious target for patients with severe CAP.

## Methods

### Ethical clearance

The study was approved by the Medical Ethics Committee of Guangzhou Women and Children’s Medical Center (2016111853), and its implementation was according to the International Ethical Guidelines for Research Involving Human Subjects as stated in the Declaration of Helsinki. Informed written consent was obtained from legal guardians of the patients.

### Participants

The study was carried out from September 2016 to May 2019 in 187 hospitalized children with CAP who underwent flexible fiber-optic bronchoscopy for investigation and/or treatment purposes in a respiratory clinic of the Guangzhou Women and Children’s Medical Center. CAP severity was classified according to HRCT scoring system as specified in Supplementary Fig. 1. Non-CAP control subjects were those without a current infection but requiring bronchoscopy treatments for removal of inhaled foreign objects. Control peripheral blood samples were obtained from age- and sex-matched healthy children who underwent growth assessments or health checks. Samples from patients and controls were utilized for analysis of cytokines, cell phenotype, RNAseq, and scRNAseq as shown in Supplementary Fig. 3.

### Flexible bronchoscopy

The criteria of flexible bronchoscopy for CAP children were as follows: (1) presence of lesions of unknown etiology on the chest radiography; (2) recurrent pneumonia; (3) persistent pulmonary infiltrates or consolidation; (4) the need to investigate hemoptysis, persistent unexplained cough, dyspnea, localized wheeze, or stridor. BAL was carried out in the most affected area identified radiologically and/or endoscopically. Warm sterile saline was instilled at 2–3 ml/kg body weight for each affected lobe and recovered by aspiration into a suction trap under negative pressure of 6.65–13.3 kPa (50–100 mmHg). BAL recovery rate was >40%. The BAL samples were collected immediately after flexible bronchoscopy and stored in 4 °C during transportation.

### Pathogen detection

Twenty-one respiratory pathogens were retrieved from the electronic medical records: mastadenovirus (ADV), bocavirus (BOV), rhinovirus (RHV), respiratory syncytial virus (RSV), ifluenza virus A & B (IAV, IBV), parainfluenza virus (PIV), cytomegalovirus (CMV), enterovirus (EV), *Mycoplasma pneumoniae* (MP), *Moraxella catarrhalis* (MC), *Staphylococcus aureus* (SA), *Streptococcus pneumoniae* (SP), *Acinetobacter baumannii* (AB), *Haemophilus influenzae* (HI), *Stenotrophomonas maltophilia* (SM), *Pseudomonas aeruginosa* (PA), *Klebsiella pneumoniae* (KP), *Chlamydia pneumoniae* (CP), *Candida albicans* (CA), and *Aspergillus* spp. (AS). Pathogen detection was performed at the central hospital diagnostic laboratories on blood, throat swab, sputum, or BAL samples. Bacterial and fungal species were cultured in Bactec9120 auto microbial culturing hood (BD) for 16–18 h and characterized by VITEK Compact System (Biomerieux, Marcy-l'Étoile, France). Viral pathogens were detected by Taqman qPCR^54^ and/or Pneumoslide IgM ELISA Kit for ADV, BOV, IAV, IBV, RSV, EV, PIV, RHV MP, and CP (Vircell, Granada, Spain).

### Cytokine assay

Cytokines in plasma and BAL supernatant were measured using “Bio-Plex Pro™ Human Cytokine Standard 27-Plex, Group I-kit” or “Bio-Plex Pro™ Human Th17 Cytokine Panel 15-Plex” from BioRad (Hercules, CA) with magnetic bead-based multiplex immunoassay (LX1000; Luminex, Austin, TX), according to the manufacturer’s instructions. For results under the limits of detection, the lowest value of the standard for each of the cytokines was used for statistics analysis.

### Flow cytometry

Peripheral blood mononuclear cells (PBMCs) were isolated from venous blood samples by Ficoll density gradient centrifugation. BAL samples were first filtered through double-layered gauze before Ficoll centrifugation. Cell pellets from BAL and blood were washed twice with phosphate-buffered saline (PBS) containing 0.5% bovine serum albumin (BSA) and 0.5 mM ethylenediaminetetraacetic acid (EDTA) and stained with various Ab combinations. For MAIT cell counting, 5 × 10^5^ APC beads (BD Biosciences, San Jose, CA) were added to the samples before flow cytometry analyzed. Data were acquired on FACS Aria SORP (BD Biosciences, San Jose, CA) and analyzed by the Flowjo 10.4 software. Staining and gating of immune populations were illustrated in the text. Abs used in this study were CD161 APC, CD3 FITC, MR1-5-OP-RU tetramer PE, TCRVα 7.2 PE, MR1-6-FP tetramer PE, CD103 BV421, CD69 BV510, CD4 BV711, CD8α BV785, IL-17A BV510, IFN-γ PE-Cy7, IFN-γ FITC, CD45 FITC, CD11c-FITC,HLA-DR BV421, CD16 BV650, CD86 BV711, CD14 BV785, FcεRIα APC-Cy7,CD163- PE/Dazzle™ 594, TCR α/β APC, CD19 APC, TCRVα 7.2 BV421, CD3 BV711, IL-17A APC-Cy7, and T-bet BV605. All of these Abs were purchased from BioLegend (San Diego, CA). RORγT PE was purchased from BD Biosciences. PLZF AF488 was purchased from Thermo Fisher. Human MR1-5-OP-RU and control MR1-6-FP tetramers have been previously described^3^. tSNE was performed on compensated concatenate populations using default parameters: iterations 1000, Perplexity 20, Eta (learning rate) 200, and Theta 0.5.

### Cell stimulation and intracellular staining

PBMCs from BAL or venous blood were cultured on V-bottom 96-well plates in RPMI-1640 (Gibco) medium (R10) containing 10% fetal bovine serum (Gibco), 2 mM glutamine (Gibco), 0.05 mM 2-mercaptoethanol (Sigma-Aldrich), 100 U/ml penicillin/streptomycin (Gibco), and 100 μM non-essential amino acids (Gibco). A combination of 40 ng/ml phorbol 12-myristate 13-acetate (PMA, Sigma-Aldrich, St Louis, MO), 1 μM/ml Ionomycin (Sigma-Aldrich), and 2 μM/ml monensin (Sigma-Aldrich) were used to stimulate the PBMCs for 4–6 h. Intracellular staining was performed using eBioscience™ Intracellular Fixation and Permeabilization Buffer Set (Invitrogen) following the manufacturer’s protocols. Briefly, stimulated cells were washed with PBS containing 0.5% BSA and 0.5 mM EDTA before being stained with surface markers. Cells were then washed and fixed at room temperature for 20–30 min, stained for intracellular cytokines permeabilization buffer at 4 °C for 1 h, and washed with PBS before analysis by flow cytometry.

### MAIT differentiation mediated by fluid or monocytes from BAL

T cells were enriched by EasySep™ Human T Cell Isolation Kit (Stemcell) from PBMCs. MAIT, CD4^+^ T cells were purified from those PBMCs by FACS sorting. Ten randomly pooled CAP BAL with a total 50 ml volume or 10 ml control BAL were concentrated by an Amicon® Pro Purification System with 10 kDa Amicon® Ultra-0.5 Device (Millipore) to a final volume of 5 or 1 5 × 10^5^ T cells were cultured in IP10 medium (10% fetal bovine serum + IMDM (Gibco) and stimulated with 1:1 anti-CD3/CD28 beads (Gibco). For differentiation, 0, 5, 10, and 20 μl concentrated BAL fluids (0, 5, and 10 μl for control BAL) were added to the FACS-sorted T cells. For CD14^+^ monocyte-MAIT co-culture assays, 10^5^ blood MAIT cells were cultured with 1 or 2 × 10^5^ FACS-sorted CD14^+^ monocytes pooled from BALs or peripheral blood of CAP patients in the presence of anti-CD3/CD28 beads in IP10 medium for 3 days. For cytokines neutralization assays, 0, 10, and 20 ng/ml IL-1β (Invivogen) or IL-23 (Mabtech) neutralization Abs were added to 10^5^ blood. MAIT cells were cultured with anti-CD3/CD28 beads and 10 μl concentrated BAL fluids in IP10 medium for 3 days (*n* = 4). Cytokines in the culture supernatants (IL-17A and IFN-γ) were measured by LEGENDplex™ bead-based immunoassays (BioLegend).

### Bulk RNAseq analysis

The quality of pair-ended raw data was evaluated with *fastQC*. For qualified datasets, adaptor trimming was performed with *fastp*^55^. We applied the *HISAT-StringTie-Ballgown* pipeline for transcript-level expression analysis^56^. Briefly, the trimmed reads were mapped to human genome (*hg19*) with *HISAT2* and the transcript assembly and quantification were performed with *StringTie*^57^. The R package of *ballgown* was applied for differential expression analysis^58^. Genes with absolute log 2 fold change over 1.0 and adjusted *p* value below 0.05 were designated as DEGs.

### Isolation of scRNAseq dataset for MAIT and CD4^+^ T cell subsets

Single-cell transcriptomes for MAIT and CD4^+^ T cells were isolated from scRNAseq dataset (GSE124885). Briefly, BAL samples from five CAP children were mixed for isolation of T cells (CD45^+^CD14^−^CD16^−^CD3^+^CD19^−^), followed by single-cell RNA library construction (5′ library kit from 10x GENOMICS) and sequencing on the Illumina HiSeq X Ten platform. After cell barcoding and reverse transcription, we also split part of the complementary DNA to enrich TCR transcripts for paired single-cell TCR-seq [v(d)j sequencing]. Raw data pre-processing was performed with Cell Ranger v2.1.1 pipeline (10x GENOMICS) with default setting. The pre-processed 5′ expression data was analyzed further with the R toolkit Seurat (v.3.0)^59^. Genes expressed in <3 cells and cells with <200 genes were removed. After further filtering, only cells with unique gene counts between 200 and 2500 and percentage of UMI aligning to mitochondrial genes (%-MT) below 5% were kept. The raw gene expression reads for each cell were normalized and scaled for dimensional reduction (principal component analysis) and graph-based clustering (*K*-nearest neighbor) based on the Euclidean distance in the space of selected PCs. Uniform manifold approximation and projection (UMAP) was performed for data visualization. We annotated each cell cluster based on the expression of canonical markers. MAIT and CD4^+^ T cell subsets were isolated from the whole T cell Seurat object for further analysis. Based on the results of single-cell TCR-seq, only T cells harboring TRAV1.2 within their TCRα chain were designated as MAIT cells.

### Metabolic gene set expression analysis

Metabolic gene set (Table S4) expression level for each cell were obtained by running *AddModuleScore* function within Seurat. The average gene set expression value for each cluster was scaled (*z*-score) among clusters for heat map plotting.

### Distance tree plotting

Genes with zero average normalized expression value in each cluster were removed. The remaining 15,884 genes were used for Euclidean distance determination and unrooted tree plotting with R package ape^60^.

### Construction of single-cell trajectories

Single-cell trajectory analysis was performed with the Monocle package (v.2.8.0) (Trapnell et al.^61^). The normalized expression values for MAIT cells were used to create the starting subject CellDataSet class. The DEGs across cell clusters were identified by performing a function differential GeneTest (XX [expressed_genes,], full Model Formula Str = ‘~cluster’, cores = 1) and subsequently used as genes to define cell progress. Data dimensionality was reduced using the DDRTree approach incorporated in the reduceDimension function. Cells were then ordered in pseudotime with the orderCells function. The trajectory was visualized in two-dimensional space by running the plot_cell_trajectory function in Monocle2.

### Quantitative reverse transcription-PCR

Cells were directly reverse-transcribed using Single-Cell Sequence Specific Amplification Kit (Vazyme, P621-01) according to the manufacturer’s instructions. Briefly, 1000 flow-sorted MAIT cells were subjected to a 5 μl PCR reaction mix containing 2.5 μl of 2× Reaction Mix, 0.5 μl Primer Assay Pool (0.1 μM), and 0.1 μl RT/Taq enzyme. Primers used in reverse transcription-PCR (RT-PCR) are shown in Table S5. The reaction mix were quickly freezed in a −80 °C freezer for 2 min. Quantitative RT-PCR was conducted with the Applied Biosystems™ QuantStudio™ 6 Flex Real-Time PCR System, using ChamQ Universal SYBR qPCR Master Mix (Vazyme, Q711-02). The expression of mRNA encoding detected genes was normalized relative to that of the mRNA encoding the internal standard *RPL13a* (ribosomal protein L13a).

### Statistics

Prism 7.0 (GraphPad Software) was used for statistical analysis. Unpaired *t* test was used for two groups’ analysis and one-way analysis of variance for three or more groups’ analysis. Non-parametric data were analyzed by Mann–Whitney *U* test or Kruskal–Wallis test. For all analyses, two-tailed *p* values were calculated. *P* values for multiple comparisons were adjusted by the Benjamini-Hochberg method. All data points are shown with central lines indicating medians, unless stated otherwise.

## Supplementary information

Supplementary Information

## References

[1] Dusseaux M (2011). Human MAIT cells are xenobiotic-resistant, tissue-targeted, CD161hi IL-17-secreting T cells. Blood.

[2] Kjer-Nielsen L (2012). MR1 presents microbial vitamin B metabolites to MAIT cells. Nature.

[3] Corbett AJ (2014). T-cell activation by transitory neo-antigens derived from distinct microbial pathways. Nature.

[4] Wang H (2018). MAIT cells protect against pulmonary Legionella longbeachae infection. Nat. Commun..

[5] Chua WJ (2012). Polyclonal mucosa-associated invariant T cells have unique innate functions in bacterial infection. Infect. Immun..

[6] Meierovics A, Yankelevich WJ, Cowley SC (2013). MAIT cells are critical for optimal mucosal immune responses during in vivo pulmonary bacterial infection. Proc. Natl Acad. Sci. USA.

[7] Wilgenburg BV (2018). MAIT cells contribute to protection against lethal influenza infection in vivo. Nat. Commun..

[8] Grimaldi D (2014). Specific MAIT cell behaviour among innate-like T lymphocytes in critically ill patients with severe infections. Intens. Care Med..

[9] Cosgrove C (2013). Early and nonreversible decrease of CD161++ /MAIT cells in HIV infection. Blood.

[10] Leeansyah E (2013). Activation, exhaustion, and persistent decline of the antimicrobial MR1-restricted MAIT-cell population in chronic HIV-1 infection. Blood.

[11] Toubal, A., Nel, I., Lotersztajn, S. & Lehuen A. Mucosal-associated invariant T cells and disease. *Nat. Rev. Immunol.* **19**, 643–657 (2019). 10.1038/s41577-019-0191-y 31308521

[12] Le Bourhis L (2010). Antimicrobial activity of mucosal-associated invariant T cells. Nat. Immunol..

[13] D’Souza C, Chen Z, Corbett AJ (2018). Revealing the protective and pathogenic potential of MAIT cells. Mol. Immunol..

[14] Gibbs A (2017). MAIT cells reside in the female genital mucosa and are biased towards IL-17 and IL-22 production in response to bacterial stimulation. Mucosal Immunol..

[15] Sobkowiak MJ (2019). Tissue-resident MAIT cell populations in human oral mucosa exhibit an activated profile and produce IL-17. Eur. J. Immunol..

[16] Serriari NE (2014). Innate mucosal-associated invariant T (MAIT) cells are activated in inflammatory bowel diseases. Clin. Exp. Immunol..

[17] Ivanov II (2006). The orphan nuclear receptor RORgammat directs the differentiation program of proinflammatory IL-17+ T helper cells. Cell.

[18] Yang XO (2008). T helper 17 lineage differentiation is programmed by orphan nuclear receptors ROR alpha and ROR gamma. Immunity.

[19] Schraml BU (2009). The AP-1 transcription factor Batf controls T(H)17 differentiation. Nature.

[20] Chen Z (2006). Selective regulatory function of Socs3 in the formation of IL-17-secreting T cells. Proc. Natl Acad. Sci. USA.

[21] Dang EV (2011). Control of T(H)17/T(reg) balance by hypoxia-inducible factor 1. Cell.

[22] Veldhoen M, Hirota K, Christensen J, O’Garra A, Stockinger B (2009). Natural agonists for aryl hydrocarbon receptor in culture medium are essential for optimal differentiation of Th17 T cells. J. Exp. Med..

[23] Wilson RP (2015). STAT3 is a critical cell-intrinsic regulator of human unconventional T cell numbers and function. J. Exp. Med..

[24] Nair GB, Niederman MS (2014). Year in review 2013: critical care-respiratory infections. Crit. Care.

[25] Rudan I (2010). Causes of deaths in children younger than 5 years in China in 2008. Lancet.

[26] Shi, T. et al. Global and regional burden of hospital admissions for pneumonia in older adults: a systematic review and meta-analysis. *J. Infect. Dis.* jiz059. 10.1093/infdis/jiz059. [Epub ahead of print] (2019). 10.1093/infdis/jiz053 30849172

[27] Steel HC, Cockeran R, Anderson R, Feldman C (2013). Overview of community-acquired pneumonia and the role of inflammatory mechanisms in the immunopathogenesis of severe pneumococcal disease. Mediat. Inflamm..

[28] Paats MS (2013). T helper 17 cells are involved in the local and systemic inflammatory response in community-acquired pneumonia. Thorax.

[29] Stockinger B, Omenetti S (2017). The dichotomous nature of T helper 17 cells. Nat. Rev. Immunol..

[30] Gaublomme JT (2015). Single-cell genomics unveils critical regulators of Th17 cell pathogenicity. Cell.

[31] Mukherjee S, Allen RM, Lukacs NW, Kunkel SL, Carson WFt (2012). STAT3-mediated IL-17 production by postseptic T cells exacerbates viral immunopathology of the lung. Shock.

[32] Mielke LA (2019). TCF-1 limits the formation of Tc17 cells via repression of the MAF-RORgammat axis. J. Exp. Med..

[33] Walker LJ, Tharmalingam H, Klenerman P (2014). The rise and fall of MAIT cells with age. Scand. J. Immunol..

[34] D’Amico EJ, Neilands TB, Zambarano R (2001). Power analysis for multivariate and repeated measures designs: a flexible approach using the SPSS MANOVA procedure. Behav. Res. Methods Instrum. Comput..

[35] Mao AP (2016). Multiple layers of transcriptional regulation by PLZF in NKT-cell development. Proc. Natl Acad. Sci. USA.

[36] Lopez-Cabrera M (1993). Molecular cloning, expression, and chromosomal localization of the human earliest lymphocyte activation antigen AIM/CD69, a new member of the C-type animal lectin superfamily of signal-transmitting receptors. J. Exp. Med..

[37] Santis AG, Lopez-Cabrera M, Sanchez-Madrid F, Proudfoot N (1995). Expression of the early lymphocyte activation antigen CD69, a C-type lectin, is regulated by mRNA degradation associated with AU-rich sequence motifs. Eur. J. Immunol..

[38] Salou M (2019). A common transcriptomic program acquired in the thymus defines tissue residency of MAIT and NKT subsets. J. Exp. Med..

[39] Emgard J (2018). Oxysterol sensing through the receptor GPR183 promotes the lymphoid-tissue-inducing function of innate lymphoid cells and colonic inflammation. Immunity.

[40] Gutierrez-Arcelus M (2019). Lymphocyte innateness defined by transcriptional states reflects a balance between proliferation and effector functions. Nat. Commun..

[41] Malka-Ruimy C (2019). Mucosal-associated invariant T cell levels are reduced in the peripheral blood and lungs of children with active pulmonary tuberculosis. Front. Immunol..

[42] Lezmi, G. et al. Evidence for a MAIT-17-high phenotype in children with severe asthma. *J. Allergy Clin. Immunol*. **174**, 1714–1716 (2019). 10.1016/j.jaci.2019.08.003 31425779

[43] Magalhaes I (2015). Mucosal-associated invariant T cell alterations in obese and type 2 diabetic patients. J. Clin. Invest..

[44] O’Brien A (2019). Obesity reduces mTORC1 activity in mucosal-associated invariant T cells, driving defective metabolic and functional responses. J. Immunol..

[45] Laurence A (2007). Interleukin-2 signaling via STAT5 constrains T helper 17 cell generation. Immunity.

[46] Thornton AM (2010). Expression of Helios, an Ikaros transcription factor family member, differentiates thymic-derived from peripherally induced Foxp3^+^ T regulatory cells. J. Immunol..

[47] Song B, Scheuner D, Ron D, Pennathur S, Kaufman RJ (2008). Chop deletion reduces oxidative stress, improves beta cell function, and promotes cell survival in multiple mouse models of diabetes. J. Clin. Invest..

[48] Cao Y (2019). ER stress-induced mediator C/EBP homologous protein thwarts effector T cell activity in tumors through T-bet repression. Nat. Commun..

[49] Hammami A, Abidin BM, Heinonen KM, Stager S (2018). HIF-1alpha hampers dendritic cell function and Th1 generation during chronic visceral leishmaniasis. Sci. Rep..

[50] Im DS, Heise CE, Nguyen T, O’Dowd BF, Lynch KR (2001). Identification of a molecular target of psychosine and its role in globoid cell formation. J. Cell Biol..

[51] Al-Mossawi MH (2017). Unique transcriptome signatures and GM-CSF expression in lymphocytes from patients with spondyloarthritis. Nat. Commun..

[52] Ribeiro-Dias F, Saar Gomes R, de Lima Silva LL, Dos Santos JC, Joosten LA (2017). Interleukin 32: a novel player in the control of infectious diseases. J. Leukoc. Biol..

[53] Pikor NB (2015). Integration of Th17- and lymphotoxin-derived signals initiates meningeal-resident stromal cell remodeling to propagate neuroinflammation. Immunity.

[54] Chen Y (2016). Rapid and combined detection of *Mycoplasma pneumoniae*, Epstein–Barr virus and human cytomegalovirus using AllGlo quadruplex quantitative PCR. J. Med. Microbiol..

[55] Chen S, Zhou Y, Chen Y, Gu J (2018). fastp: an ultra-fast all-in-one FASTQ preprocessor. Bioinformatics.

[56] Pertea M, Kim D, Pertea GM, Leek JT, Salzberg SL (2016). Transcript-level expression analysis of RNA-seq experiments with HISAT, StringTie and Ballgown. Nat. Protoc..

[57] Kim D, Langmead B, Salzberg SL (2015). HISAT: a fast spliced aligner with low memory requirements. Nat. Methods.

[58] Frazee AC (2015). Ballgown bridges the gap between transcriptome assembly and expression analysis. Nat. Biotechnol..

[59] Stuart T, Satija R (2019). Integrative single-cell analysis. Nat. Rev. Genet..

[60] Popescu AA, Huber KT, Paradis E (2012). ape 3.0: New tools for distance-based phylogenetics and evolutionary analysis in R. Bioinformatics.

[61] Trapnell, C. et at. The dynamics and regulators of cell fate decisions are revealed by pseudotemporal ordering of single cells. *Nat Biotechnol.* **32**, 381–386 (2014). 10.1038/nbt.2859 PMC4122333 24658644

