# Supplementary material for: IL-17 production by tissue-resident MAIT cells is locally induced in children with pneumonia
Source: Mucosal Immunol. 2020 Feb 28;13(5):824–35. doi: 10.1038/s41385-020-0273-y (PMC7434594; doi:10.1038/s41385-020-0273-y)
Supplement: Supplementary file 1 — Supplementary Information [file 41385_2020_273_MOESM1_ESM.pdf]

Supplementary Materials for

IL-17 production by tissue resident MAIT cells is locally induced in children with pneumonia

Bingtai Lu#, Ming Liu#, Jun Wang#, Huifeng Fan#, Diyuan Yang, Li Zhang, Xiaoqiong Gu, Junli Nie, Zhenjun Chen, Alexandra J. Corbett, Michael J Zhan, Shengbo Zhang, Vanessa L. Bryant, Andrew M. Lew, James McCluskey, Hai-bin Luo, Jun Cui, Yuxia Zhang*, Yifan Zhan*, Gen Lu*

Correspondence: yuxia.zhang@gwcmc.org (Y.X.Z), zhan@WEHI.edu.au (Y.F.Z) or lugen5663330@sina.com (G.L)

This PDF file include：

Figure S1. HRCT scoring and clinical presentation clinical presentation criteria for CAP severity

Figure S2. Cytokine-producing cells and link to disease status and pathogen

Figure S3. Flow chart showing sampling numbers and type of analysis

Figure S4. Flow cytometry validation of transcription factors and cytokines neutralization assays for MAIT cells

Table S1. Moderate and severe HRCT-CAP characteristics

Table S2. Gene expression by MAIT cells from blood and BAL of CAP patients

Table S3. Gene expression by CD14+ Monocytes from blood and BAL of CAP patients

Table S4. Metabolic gene sets of MAIT cell subsets from CAP patient BAL

Table S5. Supplemental materials

A. Representative images of HRCT scores for children with CAP

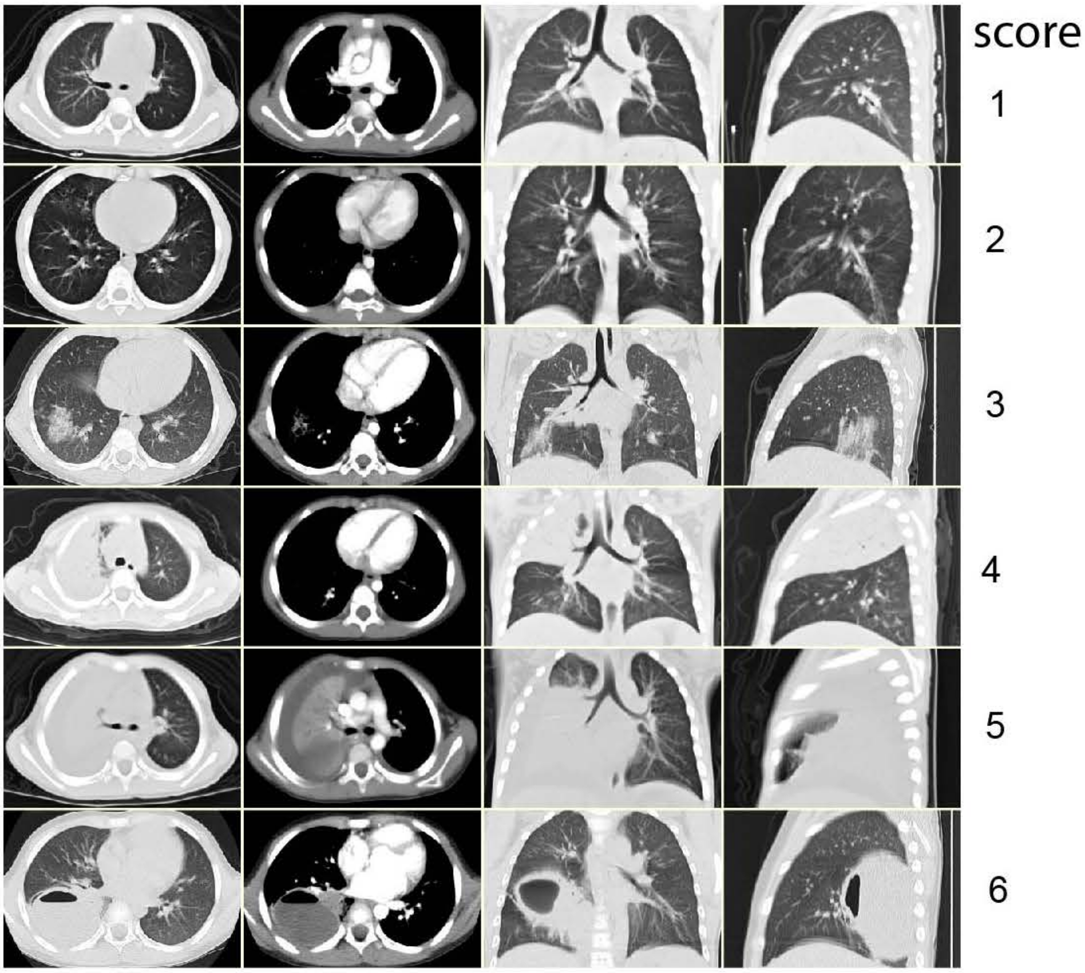

B. HRCT Scoring Criteria for CAP severity

HRCT findings	Score
Single localized, patchy, clouding opacity, tracheitis image	1
Bilateral localized, patchy, clouding opacity image	2
Consolidation chest image, less than 1 lobe	3
Bilateral diffuse infiltration or consolidation image, less than 2 lobes	4
Massive diffuse infiltration or consolidation, more than 2 lobes with or without mild/moderate pleural effusion	5
Lung abscesses or massive pleural effusion	6
If any of the following patters appear, add 1 to each of the above score<br>Mosaic sign, ground glass opacity, bronchiectasis, multiple pulmonary nodules or cloudy dense areas	

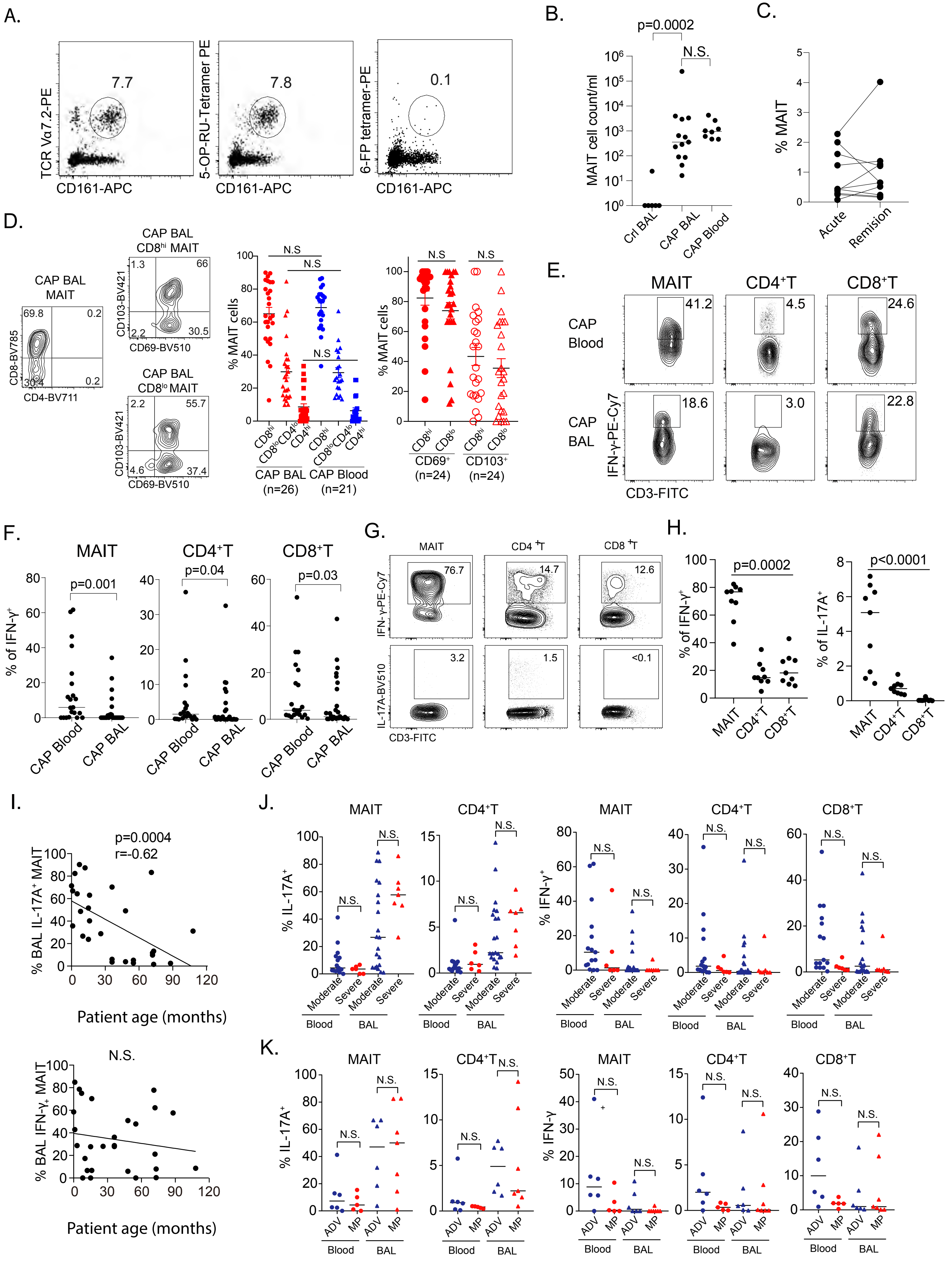
A.
TCR Vα7.2-PE
5-OP-RU-Tetramer PE
6-FP tetramer-PE
CD161-APC
7.7
7.8
0.1
B.
p=0.0002
N.S.
MAIT cell count/ml
Crl BAL
CAP BAL
CAP Blood
C.
% MAIT
Acute
Remision
D.
CAP BAL MAIT
CD8-BV785
CD4-BV711
69.8
0.2
30.4
0.2
CAP BAL CD8hi MAIT
CD103-BV421
CD69-BV510
1.3
66
2.2
30.5
CAP BAL CD8lo MAIT
2.2
55.7
4.6
37.4
% MAIT cells
N.S
CD8hi
CD8lo
CD4lo
CD4hi
CAP BAL (n=26)
CAP Blood (n=21)
CD69+ (n=24)
CD103+ (n=24)
E.
MAIT
CD4+T
CD8+T
CAP Blood
CAP BAL
IFN-γ-PE-Cy7
CD3-FITC
41.2
4.5
24.6
18.6
3.0
22.8
F.
MAIT
CD4+T
CD8+T
% of IFN-γ+
p=0.001
p=0.04
p=0.03
CAP Blood
CAP BAL
G.
MAIT
CD4+T
CD8+T
IFN-γ-PE-Cy7
IL-17A-BV510
CD3-FITC
76.7
14.7
12.6
3.2
1.5
<0.1
H.
% of IFN-γ+
% of IL-17A+
p=0.0002
p<0.0001
MAIT
CD4+T
CD8+T
I.
p=0.0004
r=-0.62
% BAL IL-17A+ MAIT
Patient age (months)
N.S.
% BAL IFN-γ+ MAIT
Patient age (months)
J.
MAIT
CD4+T
MAIT
CD4+T
CD8+T
% IL-17A+
% IFN-γ+
N.S.
Moderate
Severe
Blood
BAL
K.
MAIT
CD4+T
MAIT
CD4+T
CD8+T
% IL-17A+
% IFN-γ
N.S.
ADV
MP
Blood
BAL

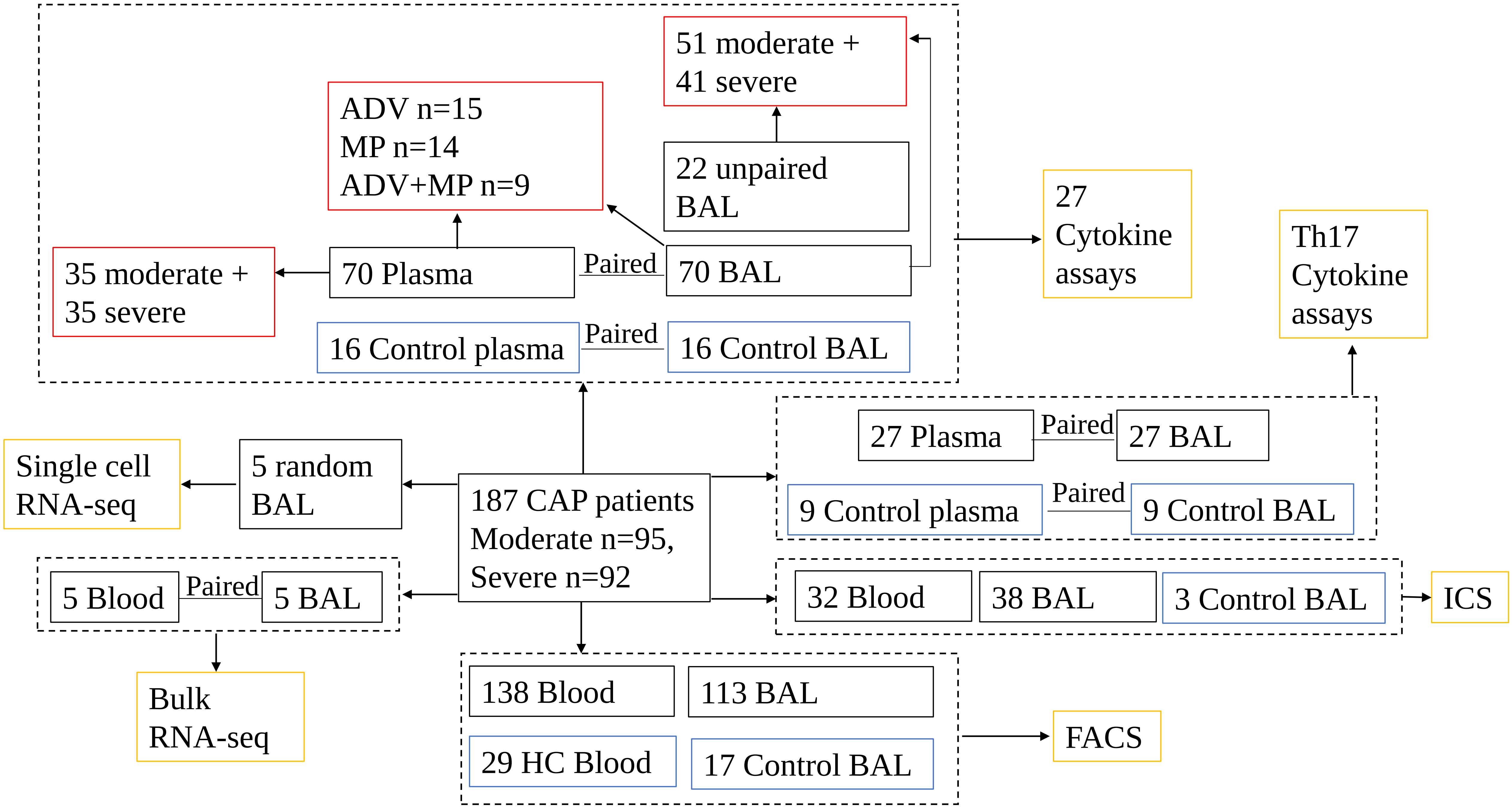

51 moderate +
41 severe
ADV n=15
MP n=14
ADV+MP n=9
22 unpaired BAL
27 Cytokine assays
Th17 Cytokine assays
35 moderate +
35 severe
70 Plasma
Paired
70 BAL
16 Control plasma
Paired
16 Control BAL
27 Plasma
Paired
27 BAL
Single cell RNA-seq
5 random BAL
187 CAP patients
Moderate n=95,
Severe n=92
9 Control plasma
Paired
9 Control BAL
5 Blood
Paired
5 BAL
32 Blood
38 BAL
3 Control BAL
ICS
Bulk RNA-seq
138 Blood
113 BAL
29 HC Blood
17 Control BAL
FACS

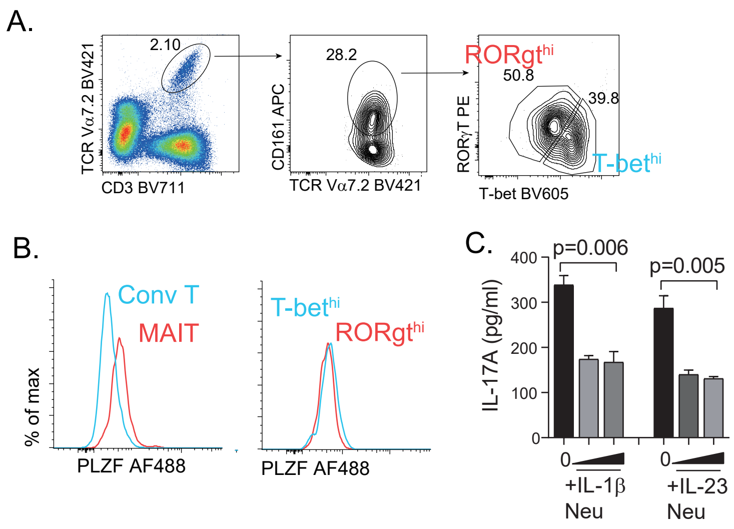
A.
2.10
TCR Vα7.2 BV421
CD3 BV711
28.2
CD161 APC
TCR Vα7.2 BV421
RORgthi
50.8
39.8
RORγT PE
T-bethi
T-bet BV605
B.
Conv T
MAIT
% of max
PLZF AF488
T-bethi
RORgthi
PLZF AF488
C.
p=0.006
p=0.005
IL-17A (pg/ml)
400
300
200
100
0
0
+IL-1β
Neu
0
+IL-23
Neu

sFigure 4

Supplemental Table 1: CAP clinical characteristics

Parameters<br>HRCT-CAP	Moderate CAP<br>Median<br>(SD; Range)	Severe CAP<br>Median<br>(SD; Range)	Non-CAP control<br>Median<br>(SD; Range)	P of unpaired *t*-Test<br>(Moderate vs Severe)
Number	95	92	22	
Demographics				
Gender (M/F), (%)	59 (62.1%)/36(38.9%)	58(63.0%)/34(37.0%)	10(45.5%)/12(55.6%)	n.s.*
Age (Months)	36 (33.4; 1-144)	25 (35.0; 1-157)	24 (25.3; 8-84)	<0.0001
Common Clinical				
Duration of fever (days)	6.0 (12.6; 0-92)	7.0 (10.6; 0-60)	N/A	0.006
Duration of cough (days)	30 (72.6; 0-365)	20 (45.2; 0-365)	N/A	0.05
Length of stay (days)	9.0 (6.8; 0-38)	16 (11.3; 0-73)	9 (11.3; 1-18)	<0.000
Pathogens (No., %)				
Multi-Infection	28(29.4%)	31(41.6%)	0	
AdV+MP†	7(7.3%)	8(8.7%)	0	
AdV†	26(27.3%)	22(23.9%)	0	
Adv only	8(8.4%)	5(5.4%)	0	
Mycoplasma†	35(36.8%)	38(41.3%)	0	
MP only	25(26.3%)	24(26.1%)	0	
Bacterial†	23(24.2%)	26(28.2%)	0	
Viral†	37(38.9%)	43(46.7%)	0	
No-Pathogen-detected	17(17.9%)	18(19.6%)	22(100.0%)	
Treatment (No., %)				
Antibiotics	69(72.6%)	83(90.2%)	1(4.0%)	
Macrolide	38(40.0%)	37(40.2%)	1(4.0%)	
Glucocorticoid	73(76.8%)	38(41.3%)	0(0%)	
†: Pathogens or pathogencombinations % are not exclusive and count towards multi-infections				

*: p value calculated by Fisher's exact test

Table S2. Gene expression by MAIT cells from blood and BAL of CAP patients

GeneID	GeneNam	Locus	Blood_MA	Blood_MA	Blood_MA	Blood_MA	Blood_MA	BAL_MAIT	BAL_MAIT	BAL_MAIT	BAL_MAIT	BAL_MAIT	adj_p_val	log2FC
ENSG0000(	ENGASE	17:790749	18.8982	23.2582	4.01392	2.95225	3.86815	18.0192	8.39616	6.60949	2.98719	7.702	0.5796	-0.30502
ENSG0000(	MAT2B	5:1635031	265.749	220.075	238.397	205.711	166.536	203.679	224.333	169.039	218.422	100.841	0.47785	-0.24624
ENSG0000(	ZNF134	19:576142	6.99744	10.2957	5.7726	5.13418	5.13215	5.1071	3.96433	11.9586	2.98702	7.41317	0.5935	-0.25793
ENSG0000(	LIN52	14:740847	12.6392	9.48767	18.8378	12.202	10.2738	3.16168	8.08887	9.5547	11.1771	13.0512	0.2033	-0.50988
ENSG0000(	VRK3	19:499764	62.5735	71.3498	87.879	82.3422	76.3289	63.3848	49.3376	48.0255	44.8635	54.5242	0.06985	-0.60789
ENSG0000(	RIC8B	12:106357	10.8096	7.8867	3.05638	5.76902	4.77147	4.25085	4.55034	4.09448	10.9515	6.75714	0.84225	-0.12731
ENSG0000(	CTD-2532	8:6584275	1.76268	0	0.579178	0	0.304405	0	0	2.2314	2.32099	0.588375	0.41215	1.29874
ENSG0000(	ADGRF3	2:2630817	0.504525	0.463897	0.223084	0.554719	0.116365	0.542677	0.247363	0.120047	1.0192	0.386515	0.7346	0.430553
ENSG0000(	FAM98A	2:3358365	13.7052	7.56536	9.2573	6.2187	10.8641	4.02259	14.23	6.73391	3.73613	13.1514	0.31715	-0.5003
ENSG0000(	REV3L	6:1112990	8.04984	8.12158	1.77663	2.0465	2.941	5.84647	5.48226	1.53408	4.33156	5.5242	0.72735	0.174741
ENSG0000(	MBNL3	X:1322030	10.4292	5.05383	4.50829	6.6603	13.4372	3.35508	7.90357	3.31656	1.11135	10.9621	0.2724	-0.75283
ENSG0000(	ZNF565	19:361820	2.27657	3.92379	4.10076	1.38187	1.92972	0	1.84569	1.38659	0	1.46315	0.2716	-1.66198
ENSG0000(	GOLGA8A	15:343790	40.0703	63.4218	10.4312	14.0217	18.3834	75.3365	58.2267	20.689	17.1668	52.75	0.117	0.730512
ENSG0000(	SYT11	1:1558595	7.24604	4.60565	4.78645	3.56441	3.63912	6.27229	7.06005	12.0822	4.7449	8.73484	0.0859	0.622941
ENSG0000(	RP11-345.	16:294257	24.2096	9.73041	11.0387	12.8886	8.20275	15.2147	4.5291	8.90326	16.389	0	0.6059	-1.24529
ENSG0000(	TXNDC15	5:1348738	18.8582	17.5406	28.3566	24.1369	23.9496	44.7598	44.8448	34.028	17.8169	16.4365	0.2406	0.446416
ENSG0000(	CTD-2012	16:675178	0	0.504643	0.288698	0.470547	0.340346	0	0.292419	0	1.37999	0	0.78505	0.480049
ENSG0000(	FBXO36	2:2297638	0.690577	0.386945	1.10048	0.220286	0.097425	0	0.657407	0.594887	0	0.680182	0.78015	-0.45519
ENSG0000(	RP11-110I	11:118994	0.436485	1.01335	1.23283	1.66683	1.27314	0	0.149412	1.01848	1.87891	2.06576	0.93635	-0.11554
ENSG0000(	HDAC2	6:1139330	52.0246	53.074	39.5219	31.3551	32.3365	68.7758	49.8008	33.2655	35.2466	33.7464	0.89985	0.051351
ENSG0000(	ITGB1BP1	2:9206764	100.599	61.3374	100.908	82.3496	67.6874	103.253	123.544	109.866	69.3445	31.0125	0.9052	0.043986
ENSG0000(	MEF2D	1:1564637	10.7773	8.52445	10.3954	4.59047	11.8913	9.7221	12.5248	18.9452	5.6116	26.1906	0.3029	0.416056
ENSG0000(	SH2D3C	9:1277383	49.4416	42.3344	37.7348	39.29	38.8731	20.0588	25.4	30.262	25.8665	55.8053	0.2093	-0.4138
ENSG0000(	NCF2	1:1834608	5.36093	23.0305	0.540809	3.19711	0.772986	44.1482	1.25924	2.61779	10.7661	0.770305	0.447	0.945757
ENSG0000(	SPIN4	X:6334322	0.498234	0.199445	0.388798	1.20221	1.12252	0.266453	0.627817	1.13686	0.630788	1.78467	0.939	0.119058
ENSG0000(	FEN1	11:616804	21.9126	34.6637	12.1669	19.2704	15.9899	23.7804	28.1262	16.1626	2.61993	20.7222	0.7142	-0.27064
ENSG0000(	LSM2	6:3179739	85.3804	56.8238	92.0016	73.3285	65.8215	104.156	140.616	116.179	73.9911	24.1302	0.4412	0.268721
ENSG0000(	GALE	1:2379559	9.88886	6.33609	11.5571	10.6223	9.3159	22.8248	11.4749	7.58452	12.3195	8.2988	0.57155	0.269459
ENSG0000(	AGER	6:3218096	4.13225	11.5805	5.10993	4.77683	4.28307	24.9208	8.20181	3.6859	3.55484	4.79423	0.4737	0.515197
ENSG0000(	TMEM2	9:7168336	12.7081	26.9543	9.01946	11.4452	10.2963	8.67478	6.97317	11.1993	12.542	15.0318	0.8726	-0.08098
ENSG0000(	IREB2	15:784374	11.2132	7.17179	4.71582	2.79645	3.63156	8.75476	6.26253	6.16499	3.17086	8.83858	0.9095	0.049118
ENSG0000(	TTC3	21:370732	47.8945	53.6574	34.2024	26.4915	59.5386	44.8022	42.6678	20.7465	25.8339	72.7898	0.232	-0.40404
ENSG0000(	RAD51AP1	12:453879	2.89985	9.15765	1.20512	1.88076	1.11224	7.72812	9.94355	4.61835	13.0319	5.50511	0.04915	1.45878
ENSG0000(	ANKLE1	19:172318	0.543816	1.91824	0.137445	0.612989	0.213348	2.5247	1.59406	0.643391	0	0.583819	0.5819	0.685045
ENSG0000(	TXNDC5	6:7881516	26.5099	45.0408	9.98931	16.1511	8.83782	14.8315	20.7822	5.48725	48.4313	9.92315	0.93825	0.05996
ENSG0000(	C2CD4D	1:1518378	6.20716	1.00598	6.0289	5.23236	8.47779	1.98782	1.52401	8.08382	7.94762	5.96571	0.9742	0.024761
ENSG0000(	RCC1	1:2850594	7.15481	6.72384	5.41891	7.44863	6.23389	2.33598	5.10898	3.18016	0.022355	8.66011	0.3855	-0.91137
ENSG0000(	GLYR1	16:480320	21.2528	21.4841	3.19794	4.79524	7.18623	21.6977	22.3983	5.58955	4.91427	9.81785	0.8774	0.091685
ENSG0000(	SF3B2	11:660507	75.8369	82.5985	35.5919	34.7707	58.5744	73.2373	56.2018	46.6799	35.1938	82.6184	0.91375	0.033225
ENSG0000(	ACBD4	17:450233	12.5621	13.2674	4.34745	11.2204	7.36562	14.6218	7.3509	6.89936	16.3589	10.6438	0.7187	0.241611
ENSG0000(	MFSD8	4:1279178	6.50836	13.034	5.93183	6.84899	3.6026	15.1008	19.9257	10.19	13.2769	5.6389	0.3378	0.565707
ENSG0000(	ASAH2B	10:507393	1.68023	0.861852	0.843781	0.744197	0.853002	1.10411	1.25968	1.94803	1.32577	1.98225	0.30805	0.727002
ENSG0000(	NHP2	5:1781309	119.711	98.4954	226.595	257.122	171.424	106.928	142.723	191.069	109.379	31.7861	0.09315	-0.64761
ENSG0000(	PUM1	1:3093150	36.4466	57.3003	21.1683	19.0078	20.1536	7.49451	14.1461	18.2502	11.2511	39.3939	0.00835	-0.88661
ENSG0000(	HOMER2	15:828369	3.63027	1.84994	1.29527	2.71516	0.843045	2.86795	2.24146	1.46326	0	1.53956	0.7046	-0.31321
ENSG0000(	MYO9B	19:170757	14.8828	20.8727	6.21043	4.99937	10.2686	21.7912	26.8241	10.0268	11.2897	22.6053	0.16295	0.75687
ENSG0000(	RP11-498	17:818784	0.997632	0.658273	1.19313	0.548337	0.989627	0.215842	1.28901	0.563438	0	2.32708	0.8223	-0.24676
ENSG0000(	GINS4	8:4152920	4.20278	3.44105	0.981043	2.13286	0.448982	12.2475	4.28339	2.75707	2.02451	4.03853	0.2332	1.01329
ENSG0000(	PSMA7	20:621367	314.194	284.628	473.348	450.492	562.637	529.306	522.82	611.588	403.258	315.476	0.6869	0.137797
ENSG0000(	RHOT2	16:668085	147.512	257.37	72.7771	133.362	60.8293	167.879	221.467	83.8738	104.762	82.5445	0.9603	-0.01621
ENSG0000(	METTL10	10:124461	22.3241	14.1558	24.2857	28.7219	22.8296	27.0285	18.4709	19.7802	26.4262	9.04763	0.8213	-0.20137
ENSG0000(	TRBV5-5	7:1424825	7.24911	13.3713	1.67359	0	0.129297	21.865	12.3904	4.39539	0	0.574794	0.68175	0.837945
ENSG0000(	LIX1L	1:1459113	4.97472	9.14505	7.19382	3.12447	10.684	2.96322	4.63354	11.4203	8.34486	21.5236	0.8577	0.327596
ENSG0000(	RACK1	5:1812369	415.7	373.945	638.838	474.742	637.113	312.569	352.71	501.27	420.553	193.394	0.1903	-0.49869
ENSG0000(	SH3BP5L	1:2488104	6.79386	2.80861	1.80626	1.24462	2.60373	4.4897	4.26393	3.6785	3.06944	5.24841	0.3681	0.492264
ENSG0000(	CHKB	22:505688	116.32	115.008	49.4848	84.1095	66.1388	149.039	131.265	39.2909	88.1675	62.8152	0.64	0.169302
ENSG0000(	PURA	5:1400708	1.41808	1.38983	0.822428	0.743664	1.96234	1.79904	3.28011	1.6383	0.990115	3.19322	0.1992	0.567225
ENSG0000(	UPF3A	13:114281	23.4863	27.2476	36.1443	31.6436	41.297	38.8184	43.2644	28.8706	29.7419	33.4201	0.86455	0.063242
ENSG0000(	TGFB3	14:755748	0.768927	0	0.892811	0	0.419637	0.49501	1.89516	1.16266	0.509681	0.873511	0.27915	1.09555
ENSG0000(	RP11-118	12:922853	0	0	3.16024	2.6061	0.363244	0	0	0.3699	0	0.862735	0.5502	-1.17846
ENSG0000(	PTDSS2	11:448267	6.5945	6.17323	5.78229	6.41333	6.18037	8.17632	8.18355	6.50623	9.11031	8.57907	0.47365	0.295069
ENSG0000(	GSDMB	17:399045	6.56955	18.4186	7.27005	3.14979	4.97108	28.7206	26.1224	7.456	5.53929	16.7636	0.03985	1.15074
ENSG0000(	DOCK7	1:6245429	2.22899	2.38026	1.96704	1.09604	1.02047	1.20316	0.166308	0	2.82241	1.56242	0.2834	-0.65575
ENSG0000(	HSPBAP1	3:1227400	11.8171	13.5149	10.025	8.19684	8.78908	9.05951	13.4425	7.67369	11.6472	7.37858	0.8933	-0.06787
ENSG0000(	LDHB	12:216353	475.26	765.059	512.797	456.897	451.634	461.538	423.919	378.484	371.2	207.886	0.2493	-0.42008
ENSG0000(	RP4-635A	1:1852923	0.552528	2.23407	1.95061	0.545497	0.238388	0.625455	3.20478	0.23484	0.178315	0.814254	0.7848	-0.12981
ENSG0000(	FBXW8	12:116910	2.49331	3.7818	2.31765	1.75348	2.54469	3.76907	6.51835	3.02742	1.59877	4.98786	0.5727	0.400364
ENSG0000(	CEBPZ	2:3716781	34.3974	36.1227	19.5332	24.2921	18.4447	21.902	26.1886	18.059	20.9109	20.4015	0.7092	-0.17822
ENSG0000(	AC113189	17:756187	3.06598	4.16444	3.01146	3.92528	2.17433	1.67815	5.64742	2.62981	6.39182	0.830977	0.7677	0.318653
ENSG0000(	HBA1	16:176679	0.725614	0.44081	111.794	1847.42	1.23536	2.8941	5.43349	33.0853	586.169	0.853508	0.2265	-1.94073
ENSG0000(	IL13RA1	X:1187275	0.711357	3.66125	0.030136	0.08585	0.071818	8.52974	0.052827	0.035773	0	0.152649	0.6966	0.98482
ENSG0000(	CTB-31O2	19:187487	0.175558	0.68858	0.580623	2.05895	0.271137	0.293689	0.583665	0.990919	0.974959	0.080412	0.85085	-0.34088
ENSG0000(	CDC25A	3:4815714	0.689985	0.206226	0	0.089594	0.046331	1.75374	0.441445	0.907098	0	0.516652	0.10125	2.15401
ENSG0000(	PGAM4	X:7791065	2.30053	2.13071	2.31648	4.33404	1.03031	3.17552	4.01335	3.01152	2.26049	0.582444	0.5615	0.894334
ENSG0000(	ASB7	15:100553	8.85593	8.75796	7.35656	6.70813	5.5609	3.35427	3.54692	5.34629	2.57849	8.31039	0.3135	-0.90433
ENSG0000(	ATXN7	3:6386455	9.91893	22.996	8.81406	4.47824	6.49273	14.5242	9.30077	14.4783	5.43233	18.9454	0.8171	0.105174
ENSG0000(	CEND1	11:787103	0.181036	0.567678	0	0	0	2.78193	0.621084	2.90787	0	0.25419	0.2665	3.26449
ENSG0000(	ETF1	5:1385060	18.6744	20.8455	14.1289	16.467	14.3585	16.2032	30.3895	18.1635	23.5393	14.0311	0.46995	0.284273
ENSG0000(	XXcos-LU(	3:5022629	2.0947	4.55001	0.376583	0	0	6.02645	2.14725	0	0.510886	0.47498	0.7797	0.46647
ENSG0000(	NOP14-A:	4:2934898	10.0657	7.52075	7.95565	4.9146	5.22606	20.2339	12.4406	13.7106	16.94	9.00361	0.2398	1.02492
ENSG0000(	PPP1R14B	11:642444	49.0712	20.8079	25.2807	19.9318	43.4653	96.36	66.6837	51.3884	34.2456	18.0673	0.11945	0.632296
ENSG0000(	AC002310	16:305240	0	0	0	0	0	0	0	0	0	0.628391	0.25575	3.34132
ENSG0000(	C2orf76	2:1193022	18.5368	12.908	13.5839	12.3993	9.03114	13.6256	24.2798	14.1051	9.68959	3.25217	0.96525	0.031293
ENSG0000(	BAG6	6:3163902	9.06891	16.632	15.144	12.6063	30.6046	21.0248	23.4812	29.613	15.76	25.9527	0.426	0.567277
ENSG0000(	PAN2	12:562716	21.4376	19.846	6.44915	6.28117	4.3565	9.31011	7.80602	2.36717	3.39046	6.09216	0.16615	-0.88614
ENSG0000(	RP11-66N	14:235117	10.2436	14.2925	5.86792	4.23934	3.0235	12.357	6.55631	6.0325	0.907778	8.04635	0.9769	0.052213
ENSG0000(	BRCA1	17:430442	3.7787	1.44934	0.585687	1.03286	0.915421	5.5392	1.32076	3.89606	2.8104	3.69064	0.1085	1.0326
ENSG0000(	TRBV28	7:1426707	12.384	38.692	26.7805	34.9769	32.3649	43.972	28.816	122.52	69.421	33.4347	0.2486	1.28847
ENSG0000(	TTC31	2:7447195	28.0602	47.181	22.4637	24.6331	13.4739	12.9983	27.6888	23.2686	22.5236	29.0767	0.56965	-0.23908
ENSG0000(	RP11-114	8:2318927	0.637314	0.488258	0.689514	0.19184	0.427428	0	0.087413	1.51853	0.065409	0	0.8099	-0.38253

ENSG0000	CTC-523E	19:349490	1.27161	1.18941	0.180296	0.090729	0.900483	1.46306	1.70877	0.179241	0	2.59154	0.53965	0.677001
ENSG0000	PDCL	9:1227983	8.50291	14.9226	10.645	11.4596	12.3618	12.5309	11.3066	10.0099	13.4457	10.9448	0.90095	-0.04505
ENSG0000	CASD1	7:9450921	17.0221	12.8912	3.51079	3.63918	4.71348	13.7859	8.9706	5.61457	1.93039	8.81611	0.95035	-0.02681
ENSG0000	C10orf111	10:150953	0.545131	1.71598	0.537546	0.13231	0.052273	0.208712	0	0.057134	0	0.278892	0.2084	-2.41386
ENSG0000	BRD3	9:1340254	7.41348	9.57964	16.9489	10.8181	17.1615	4.55591	7.87862	12.1003	7.87047	24.1552	0.37485	-0.40098
ENSG0000	LBX2	2:7449751	0.566202	1.45419	0.417038	0.340128	0.863414	0.5124	1.67774	0.270546	0.126852	1.02035	0.73545	-0.35322
ENSG0000	PPP2R5D	6:4298449	22.0071	23.5564	11.9239	11.1024	11.8155	16.2037	12.6022	14.441	4.92241	19.6669	0.75905	-0.24132
ENSG0000	ADD3-AS	10:109940	0.163739	0	0.084349	1.74474	0.478209	0.929199	0.498373	0	0.60417	0	0.9026	-0.21416
ENSG0000	RPL36	19:567494	325.659	343.443	700.776	492.165	242.327	344.33	397.935	488.624	285.743	42.1413	0.3589	-0.50035
ENSG0000	RN7SL288	13:461259	0	0	0	0	0.158978	0	0	2.25083	5.52697	1.56362	0.2736	5.495
ENSG0000	STX12	1:2777318	11.3285	10.3944	9.4009	10.2331	10.3013	20.505	17.014	13.0304	9.73803	16.781	0.3681	0.498193
ENSG0000	CCL22	16:573587	0	0.476264	0	0	0.454063	0	2.35168	0	0	0.16802	0.42745	1.39916
ENSG0000	ARMCX5	X:1025991	6.20742	6.02863	6.931	6.75041	5.28337	5.43357	7.88607	4.46095	2.61669	2.70652	0.42085	-0.57774
ENSG0000	PELO	5:5267519	5.7168	7.09838	9.92712	7.49056	6.66099	24.4966	40.571	15.0053	22.5621	18.7869	0.0138	2.19459
ENSG0000	RPS2P5	12:118149	202.925	207.351	167.355	239.09	309.03	156.977	171.44	91.5096	167.114	37.3384	0.1086	-0.88132
ENSG0000	METTL6	3:1538127	10.6657	8.39707	12.1916	11.2953	10.5718	11.7798	14.8809	12.7173	8.39138	4.96181	0.99895	0.001305
ENSG0000	ZBTB11-A	3:1016488	1.48248	0.666614	1.39421	2.11564	1.5155	0.787534	1.16362	1.49223	0	0.933014	0.696	-0.78418
ENSG0000	RP4-803A	1:3297994	0.103987	0	0	1.28586	0	0	0	2.67463	0	0.097804	0.3525	1.21166
ENSG0000	FBXW9	19:126880	4.59321	1.61471	7.05121	11.5167	4.89003	4.1273	4.58795	4.22325	7.25893	2.36083	0.5208	-0.38828
ENSG0000	SETD2	3:4701642	11.8004	10.2942	3.41844	3.74961	6.4512	6.82974	9.6079	5.81474	8.67803	14.9045	0.53145	0.247145
ENSG0000	ZNF780B	19:400282	2.28868	2.70824	2.99397	2.40191	0.471313	2.01903	3.03337	1.02498	3.93001	1.54297	0.94845	0.064953
ENSG0000	OLMALIN	10:100373	2.86817	0.236064	2.57406	2.12954	1.35931	0.733546	0.087648	2.66605	0.39945	2.68592	0.4604	-0.5706
ENSG0000	ZNF674-A	X:4654549	0.977383	2.07931	2.07001	9.08595	1.53971	2.13717	5.13087	5.79944	7.37144	3.02806	0.4962	0.517427
ENSG0000	DNM3	1:1718414	0.2942	1.59317	0	1.73265	0.085982	1.79001	0.175896	0	0.791863	0.120217	0.8366	-0.27278
ENSG0000	RP11-83N	9:1361078	1.10404	0.057779	1.01481	1.5016	0.840526	0	0	0	0	1.00954	0.27165	-2.35
ENSG0000	CNFN	19:423870	0.104878	0.476262	0.390583	0.651408	0.625416	0	0	0.358011	0	0	0.2341	-3.56562
ENSG0000	ZNF518A	10:961297	6.55018	8.62861	4.68047	12.8446	6.03076	14.2318	9.50014	6.21949	5.50205	18.1163	0.4986	0.384988
ENSG0000	COIL	17:569381	15.2431	19.9952	14.1339	14.8126	17.2228	17.4241	10.7134	13.8651	4.65355	15.0514	0.2226	-0.44968
ENSG0000	RP11-435	16:284563	6.96399	6.44407	1.7081	0.905908	0.86933	1.37816	1.02848	1.06979	3.75009	1.05601	0.5219	0.494072
ENSG0000	THUMPD3	3:8980590	15.4828	43.3977	37.9596	47.1497	32.903	42.8258	74.3939	58.9691	104.174	68.9352	0.0712	1.23384
ENSG0000	HAUS7	X:1534447	22.6866	29.445	34.7747	27.9837	38.3581	27.6864	36.9138	21.5488	11.0523	26.0776	0.3173	-0.39877
ENSG0000	SMIM14	4:3949875	7.51086	4.38809	25.4181	15.8656	13.5191	4.81988	2.00295	4.18429	4.97185	10.0299	0.0028	-2.00943
ENSG0000	MIF-AS1	22:238564	2.8319	1.34283	7.59849	8.5744	2.40261	1.85268	2.32503	4.00243	6.05788	1.85798	0.64405	-0.5319
ENSG0000	AP3M1	10:739951	21.4712	24.0985	7.37952	10.1853	8.59789	22.2415	15.0034	17.8099	6.71549	14.1322	0.9892	-0.00637
ENSG0000	SLC16A3	17:822283	5.95182	2.18385	13.9913	16.4073	18.2784	4.79606	1.42856	30.4477	29.7817	30.4941	0.46125	0.774245
ENSG0000	GTPBP1	22:387057	15.73	13.5607	8.9727	7.96795	12.6985	9.68118	30.2382	19.606	2.78222	22.7399	0.73195	0.313595
ENSG0000	IFT88	13:205670	7.46109	5.77981	2.9278	1.27795	2.09031	9.43413	3.11556	2.56756	1.48732	2.75499	0.9711	0.02552
ENSG0000	ATF7IP2	16:103264	127.882	73.5744	127.715	201.75	61.048	52.3686	41.9371	77.4872	142.865	59.9008	0.18685	-0.63486
ENSG0000	USP44	12:955165	0	1.34418	0.283536	0.52185	0.49915	0	0	0.018094	0	0.428304	0.1052	-3.82195
ENSG0000	COL9A3	20:628162	0.073112	0.965899	1.00761	3.87055	0.578648	4.6277	0	0.970136	0	0.085331	0.80345	-0.27109
ENSG0000	HNRNPU	1:2448353	216.847	225.25	78.0506	54.8767	89.024	152.224	137.307	89.4274	65.5366	136.865	0.5679	-0.22035
ENSG0000	TBCB	19:361111	178.148	160.166	253.54	239.442	253.33	283.941	256.556	215.485	190.28	126.068	0.8721	-0.06452
ENSG0000	AARSD1	17:429505	28.8399	37.4054	43.6061	46.243	37.8048	26.7095	27.949	34.5351	29.9913	30.3489	0.29675	-0.3526
ENSG0000	NDUFS1	2:2061148	18.8514	19.763	21.3553	21.4121	14.0709	31.8497	16.8381	24.6511	29.1454	17.4745	0.72305	0.246378
ENSG0000	GPATCH8	17:443952	16.2728	24.5755	13.508	11.8474	15.8719	16.3629	17.6656	11.2447	20.5836	25.0413	0.8607	0.069748
ENSG0000	SPHK1	17:763094	1.46552	0.059113	1.25004	1.92732	1.04818	32.7962	6.62372	8.80348	1.69964	0.54005	0.01115	2.91496
ENSG0000	DCLRE1A	10:113834	9.40451	1.8901	1.14615	2.75535	1.07468	5.13005	2.58964	1.97056	3.82015	4.47797	0.6087	0.284465
ENSG0000	INTS5	11:626468	11.9012	21.1361	9.51729	5.81232	11.0392	5.64834	5.72063	7.28418	4.24983	17.0268	0.4595	-0.616
ENSG0000	ZNF638	2:7127656	44.7857	49.574	31.0495	40.5208	40.9487	43.1628	25.5353	24.6666	23.8642	62.8588	0.75585	-0.12218
ENSG0000	LINC0129	2:7491814	0.975444	2.66954	2.89494	1.36168	0	0.084803	3.00159	2.44221	1.85948	0	0.95735	-0.04849
ENSG0000	TTBK2	15:427387	4.71496	4.87081	4.27387	1.75425	6.92839	7.19625	4.09455	2.39552	0.43085	12.1025	0.9565	-0.03847
ENSG0000	CDC42EP2	11:653148	0.440066	1.84688	0.667135	1.21456	0.674551	0	0.035227	0.30201	0	0.240794	0.31975	-2.9036
ENSG0000	APLP2	11:130069	32.6732	51.3155	17.1074	8.124	20.1323	74.1394	36.6083	19.3025	15.6193	31.476	0.46475	0.289231
ENSG0000	SLC25A51	9:3751089	8.20787	12.5656	8.47491	8.45885	9.33686	17.2808	3.78254	2.05952	0.821999	6.09675	0.46725	-0.75429
ENSG0000	NBPF26	1:1207239	8.71713	8.46686	1.879	2.70146	3.8075	6.67001	6.4127	2.65603	4.0882	9.23322	0.874	-0.11989
ENSG0000	PSMD12	17:673379	17.9397	22.2797	20.23	11.5351	25.442	37.3782	22.4245	22.5017	8.51509	18.5844	0.8936	-0.05661
ENSG0000	CENPT	16:678067	71.5297	103.263	33.2942	51.0088	28.0936	91.067	77.1041	46.4247	37.3381	49.6389	0.8979	0.082497
ENSG0000	CCDC74A	2:1315276	0.82936	0.992499	0.765198	0	0.31423	5.3162	1.91258	3.21792	4.38642	0.419449	0.0299	2.57788
ENSG0000	CLPTM1	19:449545	10.2159	7.95016	31.1233	24.6861	36.6724	3.75101	5.24419	35.722	23.7015	58.2789	0.80875	0.093637
ENSG0000	BTBD6P1	1:2387052	0.479128	0.423929	0.722142	1.75537	0.153501	0	0	0	0	0.152394	0.2348	-4.52279
ENSG0000	SELPLG	12:108622	392.881	436.104	524.365	482.477	598.208	324.604	292.629	355.923	355.552	542.192	0.27895	-0.42896
ENSG0000	LPCAT1	5:1456479	76.9765	36.4972	28.3823	22.6655	42.2863	19.2102	22.3189	20.1934	11.5378	36.9804	0.01305	-0.9534
ENSG0000	CFAP97	4:1851596	40.7052	51.6004	62.0582	46.6411	105.745	27.6977	32.9608	39.3338	54.3401	121.022	0.52775	-0.34342
ENSG0000	PLK3	1:4480022	11.5207	38.2249	11.4881	15.3718	8.88896	49.998	37.3119	46.6368	24.5314	36.1088	0.00065	1.27255
ENSG0000	NUDT21	16:564291	19.5655	21.8954	16.4876	11.2543	11.1349	20.6186	26.2568	22.9724	17.733	11.0081	0.8171	0.144545
ENSG0000	FBXL12	19:981026	6.27038	5.05287	23.6325	20.049	18.3713	5.2868	2.82795	13.3758	19.5799	18.8773	0.58085	-0.26472
ENSG0000	RP11-78C	17:198965	0.919493	1.09783	0.422168	0.484596	0.902361	2.52038	0.716885	1.86155	3.3055	1.82441	0.50365	1.50754
ENSG0000	RP11-203	14:204464	0.497136	0.106646	0.875872	1.342	0.340991	1.08547	0.949875	0.630901	2.12711	0.274249	0.6209	0.646002
ENSG0000	LPCAT4	15:343586	17.5837	10.6285	8.57915	7.18078	9.65961	13.6644	28.6669	17.0497	2.50653	22.891	0.1697	0.640872
ENSG0000	MBD6	12:575207	3.24659	2.12239	2.42754	1.73296	4.05135	3.29704	2.67881	3.97535	3.59106	4.96957	0.81965	0.149377
ENSG0000	CAPN5	11:770669	1.18421	2.61608	0.265357	0.498264	0.855561	0	0	1.45516	0	1.28135	0.3721	-1.12351
ENSG0000	SGK1	6:1341692	6.53105	14.8744	2.62295	23.4523	3.0539	67.0149	5.33662	15.7887	29.6511	7.37403	0.00815	1.35468
ENSG0000	TWISTNB	7:1969546	8.90609	7.96074	10.5799	13.2497	20.9108	6.00733	13.609	9.05161	10.2192	18.3662	0.65505	-0.3082
ENSG0000	SP140	2:2301213	25.3034	24.228	16.7536	18.5907	22.0252	45.2501	62.9117	32.7732	14.8168	36.1329	0.1822	0.741415
ENSG0000	RN7SL521	7:1491027	0.201907	1.02413	0.169857	0	0.334416	0.759239	0.516362	0.348624	0	2.09126	0.39985	1.88211
ENSG0000	MAFB	20:406858	1.04085	14.5103	0.186889	0.391898	2.53156	32.2531	0.617452	2.49386	3.84009	2.0866	0.1067	1.19532
ENSG0000	LTN1	21:289281	10.9847	11.0458	5.06837	3.35323	8.70701	14.5651	4.03164	3.96535	6.31931	13.2325	0.91195	-0.05968
ENSG0000	ZNF524	19:555913	26.2502	22.8869	16.5893	17.3966	29.7049	42.4485	45.3632	34.2327	17.2738	14.9935	0.49175	0.33951
ENSG0000	PIM1	6:3717020	149.824	86.2281	300.683	351.295	276.407	97.6928	132.478	297.116	188.963	208.125	0.2802	-0.37416
ENSG0000	RP5-994D	1:4276726	3.55999	1.5723	4.65121	2.32528	2.17213	4.20711	1.78963	2.77963	7.11402	1.61336	0.8121	0.366276
ENSG0000	LIPE-AS1	19:423971	6.06012	6.97915	9.20751	13.62	1.80469	7.60533	4.806	7.14765	5.44302	0.37681	0.6994	-0.60714
ENSG0000	SNAPC4	9:1363755	1.46453	3.095	0.371599	0.277366	0.470515	1.28839	1.31578	0.023748	0.012643	1.21842	0.45255	-0.57063
ENSG0000	AC104532	19:590484	1.00827	1.04817	0.442349	0.106218	0.24893	0.282813	0.490294	0.076938	0.240231	0	0.3518	-2.05017
ENSG0000	HSD17B4	5:1194524	33.7771	29.9828	17.6878	21.5866	18.3344	37.9804	8.27037	8.83019	11.0034	13.9247	0.69825	-0.18007
ENSG0000	PSMB2	1:3559954	51.8147	44.6385	55.8477	62.1642	65.7966	86.7523	74.0991	61.4468	62.3131	19.4184	0.90885	0.048223
ENSG0000	STAT2	12:563415	36.9987	57.5536	18.8426	24.8136	18.4854	35.7234	56.7916	34.1758	35.2564	39.9158	0.34435	0.347271
ENSG0000	DNAJC4	11:642066	28.8304	45.2137	30.934	30.8719	28.728	48.8197	31.0921	24.8181	33.8435	21.6144	0.98775	0.011391
ENSG0000	ARHGEF5	7:1441860	2.12279	2.57911	0.233944	0.711671	1.61531	2.05852	1.13888	0.855192	2.22452	1.87559	0.89515	0.123015
ENSG0000	N4BP1	16:485387	10.3761	12.547	5.95905	3.59201	9.3209	21.0017	23.4829	12.2539	8.92106	19.5965	0.10355	0.763599
ENSG0000	TMEM258	11:616804	120.923	79.4966	205.072	181.557	59.1962	150.853	133.508	146.527	143.465	14.5575	0.7514	-0.18561
ENSG0000	FAAP100	17:815398	15.9044	18.9074	8.60992	11.0593	8.73763	10.4458	8.49084	11.4872	14.3855	17.0861	0.96235	0.018962

ENSG0000	RP11-574	10:737855	3.55524	1.57039	1.47894	0.513335	0.490955	1.66009	1.87304	1.91851	0.566714	1.82103	0.5578	0.649098
ENSG0000	FAM174B	15:926029	0.629412	3.41537	0.086683	0.483078	0	11.5287	5.19751	5.55959	0	1.39229	0.0464	2.67492
ENSG0000	ZBTB3	11:627483	1.28899	3.55446	3.99649	1.27362	2.49706	2.94178	2.21696	0.233141	0	3.90414	0.52425	-0.58957
ENSG0000	RP11-482	7:12703-3	0.196977	0.073536	0	0	0	3.28625	0.037467	0.056666	0	0.194963	0.27335	5.74192
ENSG0000	KIAA1191	5:1763460	30.7525	32.9145	36.5103	27.4488	36.5129	14.0937	33.2009	33.2731	13.4589	42.5656	0.4218	-0.26789
ENSG0000	RP11-745	12:661165	0.210892	0.167515	0.344653	0.389819	0.192357	0.434164	0.764706	0.470053	0.167416	0	0.759	0.399516
ENSG0000	ECI1	16:223939	20.7251	24.3585	31.1439	17.8242	35.4727	33.2939	57.5612	36.0328	10.9377	11.8687	0.9423	0.033744
ENSG0000	NCF4	22:368473	34.0532	38.2219	8.94616	12.7631	13.6802	106.691	41.3863	30.9077	20.4221	28.7313	0.0188	1.04568
ENSG0000	NCR3	6:3158612	141.639	16.3648	386.607	756.931	529.059	34.0099	17.6246	194.815	295.322	76.0243	0.0261	-1.47143
ENSG0000	LIM2	19:513799	2.70085	0.10879	3.62845	1.48873	3.96526	0	0	0	0	0.282953	0.25805	-6.27075
ENSG0000	RP11-737	18:291699	0.253776	0.200143	0	0	0	0	1.89858	0	0	0.530054	0.30665	2.81189
ENSG0000	XRCC2	7:1526447	0.259735	0.643242	0.224669	0	0.163917	0.568916	1.17782	0.340046	0	0.668669	0.56465	0.97891
ENSG0000	SOCS7	17:383522	0.329178	0.336547	0.761293	0.0681	0.642257	0.065153	0.455083	0.910989	0	2.08521	0.6764	0.453911
ENSG0000	ZCCHC2	18:625230	2.44732	1.88637	2.0281	1.57571	3.51994	6.64338	5.35538	0.952924	3.42232	5.69387	0.14595	0.935445
ENSG0000	RBM6	3:4994000	63.2718	63.9657	41.4419	29.1036	30.2777	37.4271	32.4842	36.8986	35.2418	75.072	0.82235	0.11148
ENSG0000	PYM1	12:559014	72.7123	76.0038	86.5336	77.6492	58.0554	68.8197	73.3401	53.542	41.7736	25.7771	0.5147	-0.52353
ENSG0000	RPS27L	15:631217	151.549	97.137	138.698	102.006	34.6846	395.794	237.049	147.894	108.056	12.0577	0.293	0.658157
ENSG0000	MRPS30	5:4480892	54.6076	40.0749	49.6448	44.9434	45.7946	32.9864	28.0375	29.6185	8.43567	32.9051	0.02425	-0.87694
ENSG0000	RP5-1092	1:2823610	2.48023	2.8655	4.28698	1.98745	3.96015	0	3.41264	4.31541	0	6.49414	0.88285	-0.25946
ENSG0000	GS1-114I	7:3042452	7.1257	5.43469	2.07137	5.59259	1.24447	6.48617	3.15828	0.825714	4.80899	2.65804	0.77465	0.539259
ENSG0000	ZFP69B	1:4045010	1.85163	1.18741	1.82914	1.10452	0.804249	2.50559	0	0.843622	0	2.21326	0.7995	-0.34564
ENSG0000	STAT1	2:1908807	79.8904	108.522	28.0009	42.3912	30.2599	118.869	150.19	102.096	101.826	159.61	0.0059	1.11835
ENSG0000	LINC0016	11:130002	1.06193	0.775198	0.213438	0.227195	0.034454	0	0	0.220737	1.55145	0	0.9838	-0.02112
ENSG0000	RTCA	1:1002662	33.9864	13.6088	24.9557	20.3843	21.0225	46.0356	39.6245	35.5492	27.6605	21.3769	0.17735	0.489826
ENSG0000	MFHAS1	8:8783353	2.76077	2.23892	1.76376	2.90703	4.33115	5.78648	3.01189	4.12613	3.58782	9.0035	0.1111	0.707381
ENSG0000	ZNF638-I	2:7127656	1.58677	2.14046	0	0.528528	0.193634	0.606139	0.853665	0.408689	1.23998	0.305767	0.9122	-0.16072
ENSG0000	NPEPL1	20:586512	38.1018	47.6638	9.39624	13.6016	13.1529	44.8271	43.9468	11.7666	5.49758	22.8829	0.9853	0.017958
ENSG0000	R3HDM1	2:1355314	7.59052	8.68987	4.42309	2.67784	7.13422	6.6636	7.85282	5.87134	10.1488	12.8681	0.2218	0.519202
ENSG0000	NNT	5:4357159	45.3826	39.7822	12.3178	26.5217	10.6874	28.3751	15.1894	13.6261	12.2222	19.2692	0.41305	-0.32465
ENSG0000	TIMM23	10:459724	49.651	40.6427	57.7249	73.9738	43.5974	54.4429	53.8463	68.856	66.4164	29.3544	0.86205	0.056001
ENSG0000	PYROXD1	12:214376	15.784	12.2913	6.2792	8.90192	4.28677	7.73896	14.7069	12.5404	9.35575	9.98927	0.6558	0.246843
ENSG0000	HIPK3	11:332566	11.6751	6.99133	2.93026	3.02647	5.22385	5.80216	3.91735	6.02994	2.79953	9.05775	0.9669	-0.01741
ENSG0000	RBM47	4:4042326	1.0367	6.55009	0.273841	0.021772	0.068228	11.564	0.099716	0.696217	5.62997	0.130508	0.0577	1.51422
ENSG0000	REPS1	6:1387959	22.4041	18.8458	21.6315	25.4239	24.613	19.8233	17.4536	22.3941	32.9842	32.6186	0.652	0.16493
ENSG0000	RP11-823	12:893472	1.74689	4.85206	0.867928	2.0607	1.09045	4.81471	1.62791	0.842491	1.53621	4.04442	0.9159	0.155389
ENSG0000	TAF9	5:6935098	75.0199	67.6886	86.4733	75.7404	48.9744	89.2825	96.6813	84.0399	59.7794	28.6962	0.9178	0.046397
ENSG0000	PAK2	3:1967398	23.9873	21.4612	7.50729	8.36685	13.9828	22.7783	15.1729	12.9364	9.71544	23.0883	0.86635	0.056525
ENSG0000	ABCF2	7:1512078	36.0736	19.4414	48.2351	34.3298	40.4404	18.4427	18.4769	24.3308	19.8964	34.7307	0.038	-0.75288
ENSG0000	AC026202	3:5122219	0	0	0.209728	2.35586	0.073881	0	0.071081	0	0	0	0.3044	-5.42296
ENSG0000	EVI2B	17:310084	44.5205	52.9753	68.061	62.5292	116.058	68.3693	54.818	95.5441	107.415	131.887	0.65475	0.217059
ENSG0000	CANT1	17:789917	4.41368	4.96426	1.72641	4.34588	4.20651	4.61643	5.51903	2.98152	2.41706	5.92855	0.78755	0.148909
ENSG0000	MEPCE	7:1004008	5.74312	3.67873	3.28923	4.31444	9.98935	5.74093	4.44086	6.15766	3.45755	15.3619	0.60925	0.310238
ENSG0000	LYRM1	16:206102	26.8122	21.1501	24.1355	26.4636	18.2427	30.026	45.6404	25.5692	27.2144	5.31957	0.7734	0.195387
ENSG0000	MBTD1	17:511774	2.49514	3.6109	1.15917	0.83266	1.43445	8.99846	1.54017	0.62024	0.842792	3.85283	0.2595	0.812708
ENSG0000	TAF9BP1	3:2571894	0.328832	0.089923	0.248827	1.01028	0.46467	0	1.20258	2.61382	4.27767	5.23756	0.1365	2.69568
ENSG0000	CCR4	3:3295157	5.05114	15.424	0.622306	0.759112	0.058741	9.99362	7.34327	4.39458	11.4454	2.76666	0.07255	0.903103
ENSG0000	RP11-325	16:569320	0.639827	0.899321	0.159198	0.561413	0.118315	1.80707	0.598436	1.72627	3.24497	0.727231	0.16585	1.91582
ENSG0000	ATG4A	X:1080916	21.8268	16.9751	15.8885	17.5855	13.7126	13.6278	6.81913	11.2898	13.3637	9.51556	0.1102	-0.66716
ENSG0000	CLCN3	4:1696126	5.16068	7.32144	1.93258	2.12023	2.79135	7.65754	4.42825	2.30596	12.8466	8.17001	0.1059	0.780871
ENSG0000	TET3	2:7398640	0.554198	1.4159	0.407732	0.21635	0.444339	0.42344	1.63117	0.768412	0.293342	1.57738	0.46805	0.508482
ENSG0000	PLXNA1	3:1269885	1.42743	1.58665	0.794143	0.281173	1.42673	1.63417	0.753344	0.898991	2.08398	2.45511	0.4305	0.412487
ENSG0000	NDUFAB1	16:235810	99.7079	80.6406	157.818	141.139	71.9246	133.978	139.559	138.603	106.386	26.5962	0.9078	-0.04104
ENSG0000	AEN	15:886212	18.95	17.9926	16.0034	17.6449	7.5499	10.225	15.3332	22.6816	9.58755	10.8108	0.44625	-0.28104
ENSG0000	NCOA5	20:460619	12.7792	19.1299	8.91086	4.2798	9.58178	9.18538	11.824	10.7406	2.97208	14.4605	0.5599	-0.23669
ENSG0000	TFPT	19:541027	26.5289	21.5358	41.4617	27.2619	39.1169	41.567	33.9064	32.0811	21.8889	13.8166	0.8147	-0.25265
ENSG0000	PI4K2A	10:975843	10.4708	5.04601	10.5795	8.76319	17.0759	8.7523	4.70359	7.21111	9.46387	11.1938	0.32635	-0.39664
ENSG0000	AP000580	11:776599	0.511487	0.436105	0.928166	0	0.390304	1.57258	0.667939	0.348335	1.41055	0.955843	0.3628	1.44503
ENSG0000	RP11-288	11:934784	0.183686	0.117445	0	0.177746	0	0.153355	0.276614	1.3378	0	0.348492	0.1697	2.71921
ENSG0000	USP19	3:4910804	19.928	28.6968	11.1369	8.73337	12.6531	9.48797	12.4855	10.289	7.30701	16.3821	0.1245	-0.5615
ENSG0000	TBKBP1	17:476940	1.95792	1.6988	1.38203	1.97238	2.07488	1.47045	2.53129	1.21071	0.89191	2.13913	0.851	-0.15344
ENSG0000	CBLB	3:1056554	44.668	26.9671	31.085	44.5689	33.8598	23.403	38.0774	44.1415	57.1547	60.7892	0.9152	0.045701
ENSG0000	G2E3	14:304379	10.8784	7.43672	5.16183	4.78254	3.34036	13.8949	6.87239	4.81521	12.47	11.6794	0.3658	0.4012
ENSG0000	PRKAR2B	7:1070446	0.607534	0	0.402887	1.77917	1.64119	1.05859	0.057278	0.202974	0	0.657702	0.4071	-1.36463
ENSG0000	RP11-277	12:104123	0.148677	1.63126	1.44028	8.80609	0.34231	1.97335	21.3085	4.01207	10.5152	0.335042	0.34905	1.7574
ENSG0000	ZNF69	19:118877	2.86287	0.984266	0.82958	0.375502	1.18305	1.47359	0.523428	0.259727	0	0.560298	0.47715	-1.08103
ENSG0000	MVD	16:886519	50.3924	22.1481	40.6854	48.4851	41.1007	47.4602	85.7851	51.9487	39.0905	31.8914	0.78005	0.089559
ENSG0000	CTSO	4:1559241	23.3003	17.7574	20.7761	15.9344	13.0056	10.1412	3.64367	7.40793	10.2227	11.9651	0.00535	-1.1514
ENSG0000	TMEM183	1:2030073	46.3846	52.2703	33.6061	44.1983	24.4523	30.6833	44.5133	36.3141	29.9221	25.7845	0.50485	-0.25839
ENSG0000	LINC0086	1:1496063	38.9306	24.1995	17.6866	24.2117	24.2642	23.8588	24.965	13.5075	13.7272	17.0247	0.31795	-0.60786
ENSG0000	RP5-1021	14:737151	1.63741	0.541076	2.52934	0.616538	0.823112	2.37928	2.10044	3.37182	6.93538	0.40899	0.3161	1.30744
ENSG0000	POLR2H	3:1843617	43.7367	27.353	43.0114	39.0221	30.3709	47.7748	34.1585	32.791	27.4164	25.6326	0.65935	-0.15351
ENSG0000	LAMTOR5	1:1103471	1.17621	0.826313	0.727758	1.83562	0.559691	0	1.82709	0	2.51501	0	0.9434	-0.05375
ENSG0000	PIGG	4:499209-	24.9089	25.8629	12.9249	22.844	9.11151	18.496	17.8114	14.6644	18.8125	18.3211	0.9422	0.026063
ENSG0000	CCNDBP1	15:431062	195.596	135.647	203.112	183.322	183.352	244.773	182.781	158.647	148.408	117.592	0.79845	-0.09991
ENSG0000	RP4-761J	12:666325	23.616	26.5208	13.4188	22.864	12.1817	11.274	17.2741	11.0833	12.52	30.5677	0.99645	-0.00223
ENSG0000	RP11-178	17:598930	1.36376	0.31324	0.53468	0.738079	0.303829	1.34448	0.839272	0.088997	0.462139	0.007727	0.91	-0.15023
ENSG0000	FAM96B	16:669320	102.376	49.0134	38.6194	38.299	24.0063	119.543	132.137	46.3324	25.9445	19.1186	0.46285	0.316438
ENSG0000	GPATCH2	14:761317	10.5565	10.1106	8.00502	5.88531	5.61181	29.1885	5.68579	10.4775	13.0917	11.0781	0.38095	0.603568
ENSG0000	RP11-297	16:303781	5.27865	9.47038	1.34771	3.44105	0	10.2512	3.40421	2.23644	0.639253	2.90383	0.82535	0.325679
ENSG0000	NKIRAS1	3:2389165	4.01552	3.41186	3.72149	3.35287	4.53549	2.6943	2.22489	3.01031	0.024058	2.05028	0.2427	-0.95894
ENSG0000	SLC25A53	X:1040992	6.42706	6.74819	5.71525	5.76718	5.84202	11.324	6.45362	8.45445	6.16361	6.45144	0.7979	0.283054
ENSG0000	BRD2	6:3293462	71.3866	52.2813	76.6912	49.0698	79.6328	52.0271	78.9036	67.9727	43.4472	111.964	0.9998	-0.00016
ENSG0000	PRH1-PRI	12:108249	1.03987	1.08568	2.98745	1.33006	0.575248	1.72223	0	2.33765	0.73173	0.990037	0.93595	-0.12678
ENSG0000	RP11-127	5:1775548	0.614938	0.60753	0.832896	0	0.633122	0	1.52334	0.251553	0	1.00747	0.97475	0.036734
ENSG0000	TMEM179	11:627711	78.382	68.0453	141.704	127.535	93.1272	119.405	91.9229	108.534	77.4714	35.1737	0.49535	-0.29098
ENSG0000	C5orf24	5:1347587	11.0005	11.9815	7.78112	6.88296	6.34249	12.8276	8.11933	5.90841	17.1206	8.35853	0.9374	0.050142
ENSG0000	RP11-206	1:585988-	1.79091	2.62662	0.643722	0.413596	0.265647	0.597298	2.25537	0.63552	0.617756	1.01327	0.8717	-0.30502
ENSG0000	PPP1R9B	17:501337	1.52159	0.946615	0.971537	0.487427	2.24182	1.42324	1.12041	1.30644	0.08225	4.88926	0.5123	0.497984
ENSG0000	SERPINF1	17:176195	1.11328	11.3392	0.91209	0.187844	0.783185	0.083981	4.02476	6.80226	0	0.636913	0.92485	-0.10103
ENSG0000	RP11-147	17:682054	6.42257	8.08637	4.19168	1.64608	3.53947	5.27236	10.0526	7.16889	7.84788	6.73969	0.24125	0.678388
ENSG0000	TMEM140	7:1349865	23.0946	23.8367	30.509	20.3904	28.9531	44.5896	52.5218	47.9034	45.0842	26.3948	0.05305	0.7426

ENSG0000	RBM8A	1:1459113	100.499	110.122	268.449	241.598	214.937	225.876	254.59	305.27	220.135	105.616	0.96105	0.036088
ENSG0000	SENP6	6:7560150	48.0274	39.2351	15.2314	15.6361	29.2653	19.6843	30.6487	19.3001	16.1877	32.1766	0.4804	-0.28615
ENSG0000	ANXA3	4:7855151	1.1975	0	0	2.56255	0.125339	0	0	0	0.627039	0	0.2938	-2.87267
ENSG0000	OTUD6B-	8:9105990	30.6288	24.3188	34.52	34.2391	28.9468	18.3509	24.3989	25.0082	48.7876	27.2602	0.70825	-0.1331
ENSG0000	NRL	14:240801	3.66377	2.80935	1.72811	4.29961	3.00428	6.55854	4.36394	5.5163	4.0587	1.71137	0.49055	0.464379
ENSG0000	ACCS	11:440659	30.0821	35.6706	10.6892	13.8018	5.64459	11.2072	9.96077	13.231	11.5796	16.5905	0.15355	-0.58211
ENSG0000	COA1	7:4358275	50.0209	55.2577	63.335	49.7216	23.4383	84.0591	73.6604	87.4505	70.0185	26.7683	0.43225	0.534201
ENSG0000	ZNF408	11:467008	8.23496	11.9779	12.6759	11.2437	10.474	7.26894	7.93933	6.40262	11.6227	11.6333	0.70405	-0.16938
ENSG0000	MICB	6:3149488	6.78758	8.08926	9.43775	11.6642	9.49216	3.68722	9.86752	13.5304	8.79545	14.5308	0.7876	0.11959
ENSG0000	IRF2BP1	19:458836	3.53926	4.64751	3.65275	3.156	10.7296	0.587021	3.59932	6.84563	2.83731	12.8756	0.69415	0.222095
ENSG0000	OSBPL2	20:622319	11.0929	9.60763	8.96993	10.9885	7.95371	8.31382	8.49315	7.24029	10.0234	15.2955	0.7825	-0.10013
ENSG0000	CTC-523E	19:347885	1.12844	0.536292	0.3753	0.013612	0.287385	0	0	0.560838	0	0.534028	0.59205	-1.23776
ENSG0000	CCND3	6:4193493	279.726	287.515	229.447	415.659	292.633	118.127	147.721	192.665	167.641	148.165	0.00485	-0.95793
ENSG0000	RP11-111	15:404880	1.28477	1.08191	0.450792	0.612698	0.720869	0.312693	0.389136	0.43274	1.12341	0.264779	0.99645	-0.00533
ENSG0000	MEIG1	10:149594	1.94906	0.897504	1.37955	0	0	0	0.740238	0	0	0	0.30095	-2.83747
ENSG0000	THAP11	16:678067	72.7866	67.115	113.469	91.3034	107.57	61.4561	59.6814	53.0575	61.8006	53.1183	0.2743	-0.62227
ENSG0000	RHOT1	17:321412	33.2696	17.5762	10.7595	14.7003	7.47646	15.9179	14.7784	7.95313	7.43153	11.1634	0.2609	-0.45254
ENSG0000	SYK	9:9080178	0.82416	11.7197	0.922609	2.49113	1.31628	10.7028	0.727288	0.833151	3.0339	0.927852	0.8681	-0.11565
ENSG0000	DHTKD1	10:120689	3.15437	5.58814	2.7679	3.59922	2.11404	0.927962	4.25997	3.1947	4.83448	4.8603	0.88705	-0.06571
ENSG0000	GPR22	7:1071689	0.21792	0.223283	0.071902	0.491106	0.068503	0.806779	0.169269	1.04009	0	0.330809	0.4662	1.03525
ENSG0000	TMEM161	19:191191	27.8997	32.7983	27.4866	18.877	24.9245	31.5152	25.567	10.4503	4.50678	31.7163	0.26135	-0.45881
ENSG0000	ANKHD1-	5:1404018	5.27896	6.87103	3.48797	0.513682	4.37789	3.25455	4.50152	4.70957	3.38186	10.2355	0.6116	0.470634
ENSG0000	KRT86	12:522325	0	0	0.139341	0.276181	0.046577	5.04338	6.89945	2.10085	1.14154	0	0.06025	5.04898
ENSG0000	GRHL1	2:9951692	0.254909	0	0.200513	1.05761	0.142895	0.975899	0.468898	0	4.74675	0.057617	0.16615	2.18104
ENSG0000	IGKV1-12	2:8904022	0	18.5901	0	0	1.11074	0	0	0	0	0.266438	0.19045	-4.95675
ENSG0000	SAYSD1	6:3910406	14.4011	9.44918	15.445	15.3209	15.1492	17.9144	19.7914	10.7152	16.8955	11.9252	0.78805	0.1163
ENSG0000	RP11-440	4:705747-	5.14491	5.77639	3.52291	6.90775	2.52986	2.67926	4.11664	0.917609	3.67538	2.13344	0.3667	-0.82318
ENSG0000	C1orf132	1:2078015	0.26994	0.361964	0.16924	0.04438	0.186044	0.612572	0.537913	0.022416	1.00177	0.380117	0.2898	1.21652
ENSG0000	EEFSEC	3:1280516	12.2598	14.1475	19.4731	17.0239	18.1611	13.2611	12.7663	10.1052	13.615	21.7001	0.9132	-0.22081
ENSG0000	ST13	22:408245	73.4071	89.9538	74.8503	48.6728	71.4956	113.976	87.501	71.3673	44.1083	37.3054	0.7378	-0.12577
ENSG0000	THBS4	5:7999131	0.032204	0.772674	0	0.255439	0	1.83435	0.14791	0.04615	0	0.37824	0.2512	1.35878
ENSG0000	TRIM21	11:438489	44.0873	33.6413	63.2748	50.7941	50.3984	63.7038	101.746	56.4657	38.5149	60.3891	0.3036	0.339528
ENSG0000	APBB1	11:639512	34.6241	87.9279	22.7124	23.8289	28.1211	25.6237	25.752	27.8178	19.6559	33.1861	0.23405	-0.44739
ENSG0000	SMG1	16:188030	9.80041	11.5571	3.19271	4.97663	4.17193	6.48698	10.5801	6.69191	4.63263	14.3487	0.72845	0.145662
ENSG0000	NTMT1	9:1296088	30.6206	34.1163	61.906	43.5521	43.0538	37.704	48.0785	57.8785	29.4554	32.3762	0.8223	-0.11846
ENSG0000	CDK5R1	17:324866	2.49654	6.10052	0.527769	1.07038	1.07744	0	0.961283	0.317128	2.09969	2.85341	0.60235	-0.68822
ENSG0000	RP11-423	5:1778094	49.8124	45.1885	36.1086	33.2465	25.6679	33.0516	42.8707	23.1091	13.16	16.2729	0.2781	-0.48472
ENSG0000	HIP1R	12:122834	14.085	21.0759	6.83927	7.09907	4.93488	16.1942	12.0438	5.58065	3.63495	12.0885	0.59335	-0.23502
ENSG0000	RIN3	14:925137	12.7354	24.1459	6.35022	10.782	10.8569	14.385	22.8244	8.45278	3.66733	10.1166	0.8815	-0.11536
ENSG0000	ROGDI	16:479696	13.7521	18.4482	12.2681	13.5471	11.7258	19.0129	30.363	19.0681	24.6895	13.6176	0.172	0.585324
ENSG0000	ZNF442	19:123459	0.757089	3.21951	0.06712	0.010481	0.114943	0.051064	0	0.00509	0	0.722354	0.1744	-2.69733
ENSG0000	MGEA5	10:101784	61.4444	79.3853	36.6289	37.759	34.2889	62.1533	63.2597	51.934	50.2255	116.12	0.23005	0.420384
ENSG0000	MRPL36	5:1798385	80.3919	54.4624	107.639	99.4768	63.0748	109.273	143.731	101.556	49.5296	19.3022	0.84285	-0.07768
ENSG0000	MAPK8	10:483066	4.0614	3.06487	23.9206	16.5121	24.8927	12.467	8.62168	25.9771	7.84761	29.8092	0.85535	-0.12954
ENSG0000	Metazoa_	10:690881	0.297435	0	0	1.75555	0	0	0.658173	0.942695	2.53085	0	0.55895	1.3128
ENSG0000	COPB2	3:1390054	46.1524	35.3405	28.5872	31.8271	21.8236	33.8289	21.6886	24.3888	16.0542	39.1156	0.28465	-0.39953
ENSG0000	SRRM1	1:2463171	89.6132	127.846	113.068	81.5377	252.659	115.646	106.042	93.0218	85.7454	183.081	0.4732	-0.36285
ENSG0000	IFT46	11:118436	3.64143	3.53199	6.04714	9.29559	4.67348	9.44183	1.41013	3.22139	9.12325	3.00735	0.8734	-0.21095
ENSG0000	BRCC3	X:1550616	16.1329	13.6122	12.6742	17.0896	15.2647	16.3628	14.6995	12.6706	21.3979	12.8697	0.9747	0.014363
ENSG0000	CTD-2196	16:235220	0.123046	1.16282	0.089007	0.288348	0.046267	0	0.388816	0.704407	5.52786	2.8348	0.2193	2.63844
ENSG0000	TOPBP1	3:1335981	11.1519	15.5251	1.47406	1.85919	3.57458	9.66591	11.1834	3.71871	4.41235	7.75314	0.6988	0.160259
ENSG0000	RFX3	9:3218296	3.64024	5.22053	1.91239	1.94433	2.37327	1.49219	2.89409	3.21748	5.14879	4.70084	0.79225	0.146075
ENSG0000	PSMG2	18:124388	68.7174	67.9739	54.9894	53.0692	41.3591	95.5636	95.5287	39.9837	42.9124	23.129	0.8259	0.085748
ENSG0000	IDS	X:1494769	59.9175	34.8244	57.7905	59.1908	45.1969	51.7111	50.3341	85.2367	95.3674	90.0495	0.463	0.441653
ENSG0000	RP11-613	9:3751089	0.23692	0	0.279619	0.059008	0.103544	1.88107	0.670704	0.940734	0	0	0.40095	1.35026
ENSG0000	FAM221A	7:2368012	8.1264	5.59987	1.95097	4.83171	4.74625	7.2359	4.22021	1.10825	8.77709	2.94282	0.96345	-0.03602
ENSG0000	SPCS3	4:1763082	56.344	28.4817	30.8075	30.0409	35.2173	46.1977	45.3945	41.0071	58.8934	27.7099	0.88745	0.112503
ENSG0000	NUP153	6:1761503	2.73104	1.89175	2.36411	1.94101	4.21627	2.93202	1.94354	4.36734	4.99742	8.81056	0.1917	0.720289
ENSG0000	CSNK1A1	5:1494921	87.5143	76.7916	63.4101	63.3661	63.5471	93.5793	88.7856	62.3369	67.8793	71.4003	0.97065	-0.01468
ENSG0000	TGFBR2	3:3060650	28.9938	74.1025	13.531	13.8404	15.1724	25.4033	19.2255	22.9268	10.2209	34.3521	0.2362	-0.44123
ENSG0000	FRMD4B	3:6916998	10.3702	8.99411	1.80248	5.64649	8.77861	34.911	20.4306	15.9809	24.7367	17.9623	0.00385	1.66566
ENSG0000	SH3BGRL3	1:2627917	955.653	889.462	1646.78	1513.5	1750.87	1350.64	1278.03	1626.23	1530.33	818.87	0.9499	-0.05279
ENSG0000	TMEM229	14:674470	12.5295	10.7389	3.54818	2.29023	5.70947	0.813443	4.68504	3.7248	4.36031	12.9946	0.6789	-0.35336
ENSG0000	PTP4A1	6:6352176	54.7992	37.2668	32.3185	48.5433	21.7026	45.9164	27.6579	25.152	46.8053	21.7395	0.4949	-0.24373
ENSG0000	NAT9	17:747705	33.8909	45.2667	24.7694	34.8791	17.6656	21.5555	36.928	17.5074	28.7309	26.905	0.5162	-0.20753
ENSG0000	RP11-571	12:577623	2.87032	1.1846	2.19525	1.136	0.811403	0.158507	0.772575	1.90841	0	0.661586	0.65745	-0.6856
ENSG0000	CERS4	19:820673	11.2019	6.60674	9.32842	10.0931	10.734	5.98484	11.1972	13.1851	13.82	11.9853	0.56175	0.311183
ENSG0000	HNRNPM	19:844456	209.025	233.232	236.349	225.372	305.915	227.934	172.44	241.59	154.705	326.417	0.64295	-0.16227
ENSG0000	TMEM127	2:9624851	17.9804	19.4177	8.22983	6.76467	10.2361	14.3607	16.457	8.69787	2.88087	26.5498	0.56245	-0.23047
ENSG0000	PIR	X:1538479	0.639432	0	0	0.373661	0	1.99114	1.36336	0	0	0	0.4519	1.53669
ENSG0000	RP11-983	12:530061	0.957138	1.7652	0.500825	0.461752	0.32111	1.02263	0.085941	0.483589	0	0.674535	0.4624	-0.84069
ENSG0000	TATDN1	8:1244884	30.2171	29.9113	31.5067	24.184	16.3945	65.4558	44.6426	27.9495	29.5149	9.37989	0.57485	0.46779
ENSG0000	PIGU	20:345605	14.2556	11.8697	13.2115	14.6822	15.4382	20.5899	17.4305	14.7278	13.7792	14.431	0.74355	0.177162
ENSG0000	HDAC10	22:502451	8.03836	8.65621	2.62919	3.13007	3.54697	11.0414	5.27277	1.0556	0.985949	4.06624	0.4923	-0.42012
ENSG0000	SMARCA5	4:1435134	2.77202	1.43246	1.39648	2.63224	6.27242	1.69612	7.68618	1.84794	0	7.03066	0.6828	-0.58902
ENSG0000	OR10G2	14:216338	0.770697	0.619919	0.989949	0	0.553009	0	0	3.2503	0	3.43537	0.61925	1.20645
ENSG0000	LRP12	8:1044892	3.12982	1.07666	0.691951	0.947267	0.854153	3.87762	0.571025	2.36296	2.44771	2.83727	0.15065	1.03846
ENSG0000	SMN2	5:7004961	22.7414	35.8073	27.0562	28.1252	13.0338	20.3227	32.9438	28.8943	27.4412	11.4133	0.8074	0.14352
ENSG0000	MSANTD2	11:124766	8.66718	10.3694	4.99906	3.12455	5.88571	4.31294	2.39232	6.36028	2.75843	8.87666	0.30855	-0.60932
ENSG0000	CYB5R3	22:426178	37.9973	51.1285	46.2007	79.3529	46.8589	65.9424	80.9826	50.2883	80.281	54.4991	0.5091	0.229878
ENSG0000	TPRKB	2:7372910	57.0868	42.7087	56.2325	45.8401	29.1549	82.9004	62.1337	48.433	38.722	7.45189	0.90465	0.053731
ENSG0000	ATP2B1	12:895611	33.9553	60.3296	39.8741	25.5595	32.9223	30.5015	47.8396	43.3297	41.5461	58.9801	0.9746	-0.02527
ENSG0000	SLC27A3	1:1537280	15.0321	15.3226	14.1465	5.31347	13.9982	20.5798	22.7356	9.99674	1.753	20.8029	0.63695	0.282243
ENSG0000	RP11-108	1:1673993	3.33565	1.01836	1.14	2.83195	3.03624	2.81224	0.202516	1.97874	5.15529	2.54223	0.8742	0.23873
ENSG0000	RP1-223E	6:1357452	0.812679	0.407097	0	1.39804	0.094809	0.523115	0	0	0	0	0.68035	-0.90816
ENSG0000	C6orf226	6:4289026	25.4577	32.2364	43.0719	33.34	21.0391	27.1778	26.595	18.667	16.0391	11.028	0.17905	-0.72294
ENSG0000	LCK	1:3225123	627.783	668.778	746.297	664.039	691.618	498.568	590.776	498.271	505.878	667.853	0.42305	-0.32544
ENSG0000	RFK	9:7638551	14.497	11.7806	5.68622	6.05673	6.40724	6.37326	15.8431	9.56625	4.50706	7.07439	0.89365	0.057227
ENSG0000	TMEM115	3:5034732	23.6803	18.7623	27.3667	17.8299	31.603	24.042	28.8436	29.0075	11.8725	27.1487	0.9702	-0.036
ENSG0000	RP11-293	12:716094	1.25391	0.630593	1.00959	0	0.705438	0	1.21759	0.785138	0.997331	0	0.5492	-0.88393
ENSG0000	RP11-802	11:680390	6.71532	11.709	2.16298	3.08173	2.07516	16.722	5.99045	1.44244	2.27439	4.35582	0.7196	0.309929

ENSG0000	RP5-882C	17:441918	0.533126	1.66184	1.171	4.32541	0.833106	0.410362	1.3626	0.757609	0.504317	1.10454	0.4562	-1.06087
ENSG0000	TAF5	10:103367	5.62233	7.2995	3.35078	3.62591	2.88734	5.65478	5.52716	1.87014	1.84502	3.31494	0.76845	-0.29598
ENSG0000	RP11-130	2:2066666	1.41951	0.443908	0.034571	0	0.628289	2.13557	0	0.36296	0	0.326997	0.8397	0.473854
ENSG0000	CHST2	3:1431193	5.22645	11.7746	2.04298	4.27914	3.62841	4.68652	2.36151	1.91018	6.44589	3.65869	0.472	-0.40169
ENSG0000	TRAV12-3	14:219654	19.6515	26.2468	5.15794	2.70326	1.66057	42.2373	24.9385	3.75111	8.66673	3.21757	0.39355	0.658578
ENSG0000	ZNF667-A	19:564393	0.53617	5.30877	0	0	0.176476	0	0	1.62202	0	0	0.46445	-1.43736
ENSG0000	AC016747	2:6106587	2.97561	1.81562	1.47162	2.09111	0.843436	1.41913	1.36483	3.80849	0	0.664981	0.8557	-0.23757
ENSG0000	NMUR1	2:2315231	1.55991	1.92306	3.65719	1.23068	6.32615	1.54591	3.50552	1.10744	0	7.911	0.68905	-0.29238
ENSG0000	HLA-DMB	6:3293462	13.7649	31.9273	27.4649	26.6052	19.9928	128.399	24.2044	29.4836	21.2423	12.8309	0.54515	0.78823
ENSG0000	RUNDC1	17:429805	9.63345	10.2576	12.0386	14.0179	8.97474	6.16641	10.6535	10.6159	5.44948	15.5	0.49065	-0.26203
ENSG0000	MCM4	8:4796018	17.0472	23.6958	2.2679	3.73411	3.05365	38.7211	13.3639	8.68228	2.97063	13.1086	0.2151	0.564661
ENSG0000	PDZD8	10:117239	3.25041	3.27235	1.55968	1.34733	2.42888	1.51321	3.68329	1.39467	0.82415	3.95441	0.59455	-0.30771
ENSG0000	BCL10	1:8526624	6.20134	17.744	13.8976	15.5298	11.5448	6.91216	13.3004	13.7216	9.77738	14.2053	0.60235	-0.21464
ENSG0000	TIMM50	19:394804	34.4877	24.5425	38.6832	43.7734	36.6485	51.969	40.2851	38.4317	22.9539	18.1528	0.70915	-0.1365
ENSG0000	ZEB2-AS1	2:1443840	1.08473	1.66586	0.833131	0	0.995699	0.16343	0.549843	0.08283	3.46016	0.372509	0.83095	0.225212
ENSG0000	RP11-722	8:3336083	2.00935	0	0.50012	0.681856	0.777697	0	0	0	2.94188	2.99296	0.62845	0.856124
ENSG0000	PLPP3	1:5649474	0	0.782135	0	0.771844	0.177356	8.09565	0	0	0	0.025046	0.3058	2.07984
ENSG0000	INPP5D	2:2330599	110.907	124.472	36.1531	39.2238	39.5251	57.6282	77.8799	33.5096	29.6233	59.2404	0.2961	-0.34963
ENSG0000	RABGEF1	7:6662888	15.6167	15.0299	10.0424	15.0014	7.81152	10.1727	10.2534	12.756	8.7456	8.91446	0.4657	-0.48109
ENSG0000	AHI1	6:1352835	3.75004	13.9562	1.4263	4.52642	1.00491	25.2872	52.3967	10.8097	13.0728	14.1123	0.01235	2.20334
ENSG0000	METTL2B	7:1284558	10.5564	8.65722	9.59267	8.54888	12.4532	15.0687	15.8735	13.9908	11.1892	14.465	0.3932	0.407166
ENSG0000	DDX5	17:644996	1759.96	1790.4	984.564	1393.4	573.364	893.616	864.959	935.392	1169.6	1185.61	0.6416	-0.3013
ENSG0000	SMIM4	3:5249533	6.84973	8.80947	12.5128	10.5653	3.65752	16.6732	15.0712	11.0967	5.73779	2.72968	0.75485	0.29376
ENSG0000	HSCB	22:286877	16.4462	11.0779	12.3903	12.1852	9.29622	30.8216	22.54	11.5251	7.41041	5.81208	0.6573	0.297745
ENSG0000	RP11-455.	2:1896742	0.211346	0	0.397512	0	0.606428	0	0	0	0	2.51294	0.7219	0.964742
ENSG0000	AIFM2	10:700527	2.02924	2.41493	1.30257	1.86045	1.72616	4.57615	2.58558	1.95777	0	2.29566	0.80255	0.248832
ENSG0000	AL513122.	9:1211781	0.350878	0	0	0	1.42599	6.48671	2.53488	0.90368	0.000328	0	0.2419	3.98583
ENSG0000	TWNK	10:100969	4.94929	2.72781	4.5952	4.41501	4.79047	0.2676	1.256	3.69473	0	5.66345	0.45565	-0.99243
ENSG0000	MIR4453	4:1525362	1.9908	1.73223	1.00302	1.65558	0.908739	5.79712	4.87427	1.52614	0	3.1956	0.24995	1.0636
ENSG0000	SLC25A19	17:752713	11.861	13.5211	24.6281	18.2391	16.8647	28.6012	28.6111	8.82133	12.6224	13.7477	0.9743	-0.01724
ENSG0000	SC5D	11:121292	9.01171	4.13343	9.14631	20.0535	6.01189	12.4485	8.00799	20.3028	39.1822	13.3051	0.0483	0.909734
ENSG0000	AC002064	7:9033522	0.704348	0	0	0	0	2.59418	0	0	0.726889	0.196286	0.3209	2.65157
ENSG0000	RP11-114	X:4856801	1.42554	1.34886	0.918103	0.134088	0.474415	0	0.760454	0.448373	1.51931	0.126748	0.757	-0.47362
ENSG0000	PBXIP1	1:1549440	199.175	152.865	102.834	109.919	68.2641	171.531	124.891	76.6543	86.3319	109.25	0.61965	-0.16699
ENSG0000	IL11RA	9:3463813	6.91193	29.8321	3.6584	7.64145	3.69989	8.98965	12.456	10.6063	2.73223	7.83623	0.75735	-0.2228
ENSG0000	NHSL2	X:7191081	8.12127	6.21522	23.4878	19.0363	27.4099	3.85387	2.65883	18.8231	10.7473	18.0053	0.0588	-0.81786
ENSG0000	BLM	15:907173	11.9845	6.9782	2.38872	3.92477	2.59194	8.84864	6.5821	6.76821	12.4809	5.44737	0.12515	0.796635
ENSG0000	KIF3B	20:322776	3.00917	4.14089	3.16972	1.64267	3.74409	5.78084	3.26177	3.0047	1.8761	7.26724	0.4354	0.325954
ENSG0000	UBP1	3:3327702	8.24944	7.159	4.07687	2.05932	5.8759	4.5756	6.8686	6.97415	0.637497	11.1491	0.8974	0.056333
ENSG0000	HBEGF	5:1403328	0.197582	3.22157	0.281735	0.957265	0	0.586829	0.97681	0.758276	0	0.398406	0.65925	-0.77485
ENSG0000	CCP110	16:195238	6.60662	6.86425	2.55117	1.56097	2.16301	6.28737	5.38746	2.60621	7.77326	5.43377	0.172	0.595899
ENSG0000	EMSY	11:764449	12.4025	14.0013	9.18185	10.6034	10.0282	15.3212	12.8163	11.4191	9.726	12.6837	0.98755	0.010622
ENSG0000	LDAH	2:2068401	11.8298	12.1883	4.29645	2.32435	4.98205	7.40113	9.99902	6.99431	4.05798	9.14312	0.78485	0.133424
ENSG0000	ZNF213-A	16:311045	7.01609	8.48775	15.6874	15.5353	10.9938	6.21108	4.25235	11.9769	26.7461	15.051	0.7639	0.188789
ENSG0000	ACAA1	3:3803920	60.2975	101.667	73.3351	77.3182	60.5787	99.1325	87.4008	73.5196	55.0191	78.777	0.88975	0.04352
ENSG0000	FN1	2:2153604	0.406897	1.71942	0	0	0.025608	1.49895	2.07058	0.396936	0	0.491758	0.3425	0.863321
ENSG0000	ZNF782	9:9681647	2.08597	2.72814	0.312521	0.507535	1.0024	3.16832	0.907058	2.43333	0.53194	0.820433	0.8426	-0.16313
ENSG0000	ATP6AP2	X:4057937	133.891	124.905	98.1386	107.77	62.5391	148.151	96.3543	92.8362	79.6218	72.8349	0.62795	-0.1514
ENSG0000	ACP6	1:1476296	8.05398	4.52009	12.9528	15.4156	7.62576	3.22235	2.41559	5.92337	8.10353	7.61748	0.2139	-0.78581
ENSG0000	RP13-270	18:324748	0.255976	0.928434	1.06624	2.9065	0.038991	0	1.90235	2.73272	0.068431	0.953296	0.89305	0.194182
ENSG0000	RNF187	1:2284861	60.0698	47.1328	29.306	36.4774	38.102	56.7149	72.4637	44.9668	30.7107	31.8876	0.5817	0.188312
ENSG0000	SLC26A2	5:1499607	1.08272	0.96242	0.482394	0.691772	0.900761	1.15663	0.538517	1.68626	1.33966	2.63423	0.4626	0.735302
ENSG0000	LAYN	11:111540	0.710273	1.80555	0	0.084286	0.139297	13.6538	38.2533	7.37348	10.4803	3.16448	0.0009	4.80605
ENSG0000	TNFAIP8L	1:1511566	52.9146	51.2081	71.4138	38.2835	64.511	45.3856	22.103	22.9461	18.051	19.2671	0.10685	-1.1887
ENSG0000	RPUSD4	11:126202	12.3162	10.2727	14.5266	12.248	9.94647	19.1268	12.9156	10.7328	9.4816	11.7013	0.9754	0.041992
ENSG0000	RP11-81H	12:683328	3.39981	5.22551	11.3	4.39025	1.16609	9.76476	5.42082	16.54	0	1.44031	0.47815	0.547618
ENSG0000	SNX3	6:1082112	147.363	120.327	147.535	159.314	131.794	217.471	146.214	106.741	177.377	51.7619	0.7339	-0.10797
ENSG0000	AP003774	11:643250	1.36647	0.258995	1.5549	0.855111	0.168648	6.56523	2.02324	1.89373	2.26155	0.068362	0.5532	1.46984
ENSG0000	CD28	2:2037064	45.1811	34.7804	16.8574	18.4947	14.0448	26.4795	14.8086	15.56	18.5501	11.5526	0.13715	-0.52177
ENSG0000	XRCC4	5:8305284	13.5051	17.889	11.1011	10.513	10.7194	22.8412	15.7216	9.73967	10.0266	6.53685	0.93105	0.068336
ENSG0000	TRMT2B	X:1010093	9.37455	13.0239	8.03102	7.87693	7.85224	9.10705	13.9317	12.2871	5.79716	7.06519	0.97365	-0.0152
ENSG0000	ZNF318	6:4329569	2.28013	5.56829	2.29841	0.617168	2.55052	2.24072	1.92631	1.62216	0.438292	5.16592	0.4119	-0.49184
ENSG0000	HLF	17:552650	0.059171	0.123173	0.0752	0.054648	0.057127	1.34381	3.57323	2.70509	1.1829	0.516591	0.00005	4.64945
ENSG0000	CH17-189	1:1491760	7.70786	3.11194	8.06242	9.62088	3.92614	7.67757	3.65265	3.77343	6.50598	4.32225	0.6233	-0.47088
ENSG0000	LINC0101	6:2769403	0.910953	2.9912	1.35671	1.74769	1.24135	0	1.11375	1.52464	1.9567	1.89144	0.83805	-0.26266
ENSG0000	PTPN2	18:127854	65.7041	57.9938	78.6428	78.1662	56.6096	70.1682	60.1605	58.6589	36.2721	53.7027	0.33475	-0.30983
ENSG0000	CDYL2	16:805979	0.70397	1.68205	0.82321	0.335477	0.933559	2.04029	1.8454	4.17882	0.60158	3.29389	0.0405	1.14389
ENSG0000	BRIX1	5:3490526	43.9095	42.7617	56.307	50.4242	46.9123	46.3721	57.9161	39.9624	23.4531	29.9815	0.4047	-0.35274
ENSG0000	PDIA4	7:1490030	97.0517	71.8363	115.209	92.4769	99.8537	64.8051	85.2927	80.2493	77.3914	76.4266	0.24345	-0.35297
ENSG0000	PHBP9	10:100229	0.056685	0.157527	1.85732	1.58738	0.782596	0	0	1.38142	3.77861	1.01004	0.81235	0.507473
ENSG0000	GBAP1	1:1551950	4.87842	8.72377	3.52019	5.42196	3.49024	6.99582	5.43748	2.72946	1.26025	4.95388	0.51325	-0.55997
ENSG0000	DAD1	14:225649	280.647	194.778	367.567	313.846	242.723	335.493	359.962	340.623	258.668	73.1658	0.7774	-0.09191
ENSG0000	SGK494	17:284557	0.329887	0.155624	1.08383	0.341441	0.068879	1.17669	0.013544	0.432952	0.52918	1.48262	0.53735	0.699797
ENSG0000	SLC2A9	4:9771152	1.29888	3.13097	0.610274	1.14668	0.671796	0	2.59092	0.909991	0	0.125794	0.44595	-0.74839
ENSG0000	PAAF1	11:738726	10.5052	12.3606	11.4632	12.8976	14.9152	15.9666	13.2684	9.95741	9.05053	13.6089	0.94825	-0.04294
ENSG0000	PRPF38B	1:1086923	37.954	42.6986	46.6105	37.4543	54.876	46.3523	52.2818	54.4549	46.0253	108.477	0.3741	0.334068
ENSG0000	RNF113A	X:1198704	83.0299	51.6357	114.219	70.28	102.509	96.3794	84.9404	67.0429	41.7726	56.5807	0.6164	-0.35613
ENSG0000	CTD-2583	19:577696	1.54664	0.612536	2.80217	1.51373	0.400634	2.29064	0.951932	1.04555	2.20068	4.10013	0.64205	0.601493
ENSG0000	RP11-386.	9:7025896	0.367251	1.81911	0.453826	0.101273	0.028336	1.29651	0.977486	0.873228	0.240402	2.64147	0.4849	1.11549
ENSG0000	PTPN4	2:1197596	79.9495	33.1814	86.9009	80.0127	107.324	16.6526	44.2321	31.8435	24.9438	37.6805	0.00305	-1.33512
ENSG0000	ARL17B	17:462747	13.0689	18.8534	15.809	15.3152	17.8204	22.0061	20.9993	11.4139	12.8918	18.6204	0.8348	-0.15889
ENSG0000	RBBP8	18:226994	10.2975	7.66618	0.687654	0.39946	1.44869	13.5363	19.0759	5.90984	10.6307	4.3731	0.0021	1.72867
ENSG0000	STK11IP	2:2195978	16.365	16.3453	3.77218	7.89875	4.68796	14.6461	6.4779	3.30829	0.996147	14.2158	0.4737	-0.29592
ENSG0000	GNG5	1:8449832	151.504	156.522	188.081	164.62	120.612	288.324	295.552	277.648	162.46	52.5038	0.43065	0.346772
ENSG0000	BCL7A	12:122007	0.483742	4.20214	0	0.330077	0.223151	0	0	0.851989	0	1.10793	0.36715	-1.53849
ENSG0000	BCKDHB	6:8010664	10.7563	18.8359	16.4071	20.1804	10.4875	7.30551	7.23523	7.65336	17.2575	7.29128	0.29705	-0.57329
ENSG0000	RP11-273	8:1429817	0.179704	1.43029	0.186265	2.17802	0.622464	0	0	0	0	2.55204	0.7088	-0.67221
ENSG0000	FUT8-AS1	14:654105	0.899969	1.35663	2.42783	1.26844	0.896835	0.039575	1.99432	3.06729	0.134794	1.8089	0.99495	0.000153
ENSG0000	UXT-AS1	X:4765179	0.817212	0	0.50017	0.2774	0.228188	0.30765	0	0.559405	0	0.164543	0.60775	-0.8261
ENSG0000	GLIPR1	12:754806	33.6829	20.4271	34.6559	18.5695	26.4364	64.2704	66.3601	52.5968	50.8296	51.2918	0.14795	0.791711
ENSG0000	CD53	1:1108731	718.259	531.843	535.296	541.058	573.435	770.471	658.634	533.439	492.49	429.399	0.9523	-0.02138

ENSG0000	RCAN2	6:4622073	0	0.826646	0	0	0	0	2.90122	2.8315	0	1.09446	0.3301	3.09991
ENSG0000	FMO4	1:1713142	1.79314	1.62915	0.946137	1.61483	0.959904	1.88742	2.2707	1.42534	0.127715	1.80723	0.75205	0.26893
ENSG0000	TGOLN2	2:8531801	30.8146	34.7958	19.2313	10.0448	21.6999	26.7948	19.4428	22.2056	21.1457	44.3495	0.91815	0.036567
ENSG0000	NFATC3	16:679873	40.3547	62.1954	24.6672	18.8896	21.3454	24.8318	25.1628	20.2657	14.8182	34.9527	0.1192	-0.64042
ENSG0000	C11orf58	11:159664	212.666	152.751	208.913	208.201	175.809	230.487	205.219	165.177	170.202	82.8599	0.54955	-0.19154
ENSG0000	IL12A	3:1599133	2.4127	2.03182	1.66863	2.55905	1.38946	0	2.67439	0.675234	0	0.875069	0.34295	-1.17264
ENSG0000	PITPNM1	11:674917	31.982	28.982	9.84919	10.1282	16.7679	24.2555	39.361	20.7943	11.9698	27.9629	0.3551	0.321467
ENSG0000	PDE6B	4:625583-	11.6938	7.40936	15.9924	12.7148	7.47011	1.78687	4.22753	7.88308	3.5126	5.99931	0.1502	-1.16321
ENSG0000	MSANTD3	9:1004271	12.6917	7.84882	6.39737	2.63469	8.93547	23.0857	10.8023	5.31921	3.67649	10.0503	0.59595	0.359085
ENSG0000	MICAL2	11:120940	37.8372	7.01942	40.6948	25.1446	17.3514	28.5887	24.6844	30.9263	20.7946	19.8184	0.959	-0.0325
ENSG0000	BAZ2A	12:565219	12.0941	12.6189	24.2179	11.4925	33.9882	13.2878	10.1	16.4966	15.9388	55.6559	0.7172	-0.16808
ENSG0000	AC009495	2:1657948	3.8212	1.92729	1.7699	1.4551	3.84768	1.15969	5.17513	0.409079	3.22848	2.51637	0.4161	0.024979
ENSG0000	RP11-385	1:2091730	1.32788	2.74282	1.39863	0.580589	0.754139	1.04486	0	0.852477	0.204577	0	0.3694	-1.68284
ENSG0000	MRRF	9:1222376	20.9907	31.5544	21.0824	26.04	16.0897	24.6361	25.1805	18.6614	6.70251	14.82	0.537	-0.40603
ENSG0000	TXNDC9	2:9899726	60.8561	48.6074	60.8051	48.4182	31.2684	67.8828	71.5769	43.9151	52.0853	16.9112	0.94255	0.053938
ENSG0000	RANBP3	19:590484	3.4738	4.34081	13.9992	10.3481	25.7648	5.05113	6.25458	14.3539	10.8185	26.2587	0.9683	-0.02231
ENSG0000	STRADB	2:2013772	14.3513	15.9782	9.12149	11.2369	7.73203	14.2152	7.38221	2.73182	3.9253	4.68288	0.2144	-0.85613
ENSG0000	CIAO1	2:9626613	69.1978	55.8002	78.1735	55.999	73.6119	64.6068	48.7792	55.8903	76.1344	70.4487	0.74585	-0.12369
ENSG0000	RAB11FIP5	2:7294203	2.46804	2.58366	1.25018	1.012	2.0134	1.19952	0.896261	1.7878	0.646827	1.99453	0.6043	-0.51383
ENSG0000	CDK16	X:4721785	11.5019	13.2373	11.0186	10.7576	9.94001	14.053	18.3761	13.7112	7.42959	20.5132	0.52715	0.252465
ENSG0000	CTC-559E	19:197573	4.04974	9.02694	8.62404	7.24634	5.16067	2.01773	8.62653	3.51938	8.92339	5.45896	0.7871	-0.21728
ENSG0000	RBMS1	2:1602721	55.2896	51.2284	61.5513	59.2433	66.7884	50.9327	72.53	49.4663	51.0131	95.5528	0.984	0.007424
ENSG0000	RP11-265	11:120940	4.28393	0	0.029068	0	0	1.43657	0.336611	0	1.18127	0	0.2306	10.227
ENSG0000	TRBV5-6	7:1425000	9.50287	31.3039	6.43707	5.53042	1.04116	23.7325	7.4136	6.6686	42.6665	2.67251	0.26675	0.899551
ENSG0000	LRP5L	22:253514	3.57103	5.74278	4.21123	3.60001	1.98744	0.269224	4.97237	7.16718	0	4.78807	0.97115	-0.03314
ENSG0000	SEMA4A	1:1561473	6.28854	11.3615	3.65418	8.26895	5.40004	14.0421	12.6607	6.34367	0.10298	5.06235	0.79195	0.110136
ENSG0000	PLA2G15	16:682453	5.83811	4.10498	6.55567	5.15391	4.37703	10.5627	6.43226	8.37269	6.47897	8.50865	0.13435	0.629678
ENSG0000	MATR3	5:1389467	188.886	160.559	42.799	62.3498	46.5792	131.447	113.894	73.5081	48.0695	71.9265	0.54755	-0.22952
ENSG0000	TMEM101	17:440111	18.7211	15.695	29.9409	23.1004	25.1804	23.6906	24.7016	30.5741	33.2247	14.1275	0.6708	0.163554
ENSG0000	RAPGEF1	9:1315767	16.6714	19.1629	7.40928	6.23495	9.51383	26.5231	14.6973	16.8039	9.97101	28.3582	0.22415	0.58548
ENSG0000	TMEM51	1:1515253	0	0.704374	0	0	0.205438	13.3914	0.922457	0	0	0.340734	0.1275	4.05834
ENSG0000	PEA15	1:1602021	2.28458	1.37563	2.24409	3.28493	4.85433	4.70165	1.7043	4.14821	4.7654	4.3593	0.7308	0.181223
ENSG0000	CPSF4	7:9932589	12.2498	19.4977	12.0004	16.6407	15.6069	5.47379	13.0194	7.43449	0.12318	12.3884	0.2026	-1.02322
ENSG0000	ZNF239	10:435563	2.80056	6.65225	1.09988	1.11083	1.87371	0.396014	1.36721	1.22977	0	3.37821	0.45295	-0.92955
ENSG0000	GAL3ST4	7:1001592	0.546326	3.69677	0.684042	0	0.187751	2.7033	0.507421	0	0	0.299517	0.787	-0.33806
ENSG0000	ZNF649	19:518880	7.23181	4.17464	3.98587	4.48463	3.30352	1.13804	5.99745	4.97767	6.64228	4.24013	0.89815	0.067965
ENSG0000	GIMAP4	7:1505672	450.866	519.988	565.304	409.197	746.78	344.723	226.908	269.923	223.675	289.056	0.00455	-1.07154
ENSG0000	EIF3CL	16:283795	48.4482	48.1543	50.8192	43.2628	38.274	43.2354	32.8567	50.9479	42.5978	45.8166	0.88975	-0.11546
ENSG0000	RAP1B	12:686108	389.976	334.789	344.791	374.946	236.493	306.996	277.484	264.215	301.376	143.058	0.2103	-0.4492
ENSG0000	TRIP6	7:1008671	4.23128	3.68628	1.03627	1.72625	0.570115	5.6497	8.51796	2.20003	2.84023	2.30887	0.1458	0.919203
ENSG0000	RNASEK	17:685386	239.683	255.857	294.788	301.654	335.712	383.253	360.353	407.348	341.931	119.967	0.9279	0.064009
ENSG0000	CCNY	10:352470	21.0671	20.5874	6.08467	2.86172	7.37471	17.3436	15.2811	5.20535	5.82142	9.48419	0.7136	-0.14241
ENSG0000	STOML1	15:739789	7.73529	6.87051	6.8759	4.43125	6.15065	8.45912	8.86726	3.61025	2.33788	6.62818	0.72	-0.22029
ENSG0000	RP11-109	1:3786069	2.70639	2.06628	3.04995	3.09861	0.806319	1.23268	1.62995	0.559042	0.678916	0.835547	0.99585	0.005138
ENSG0000	ZRANB1	10:124917	5.51252	5.05682	1.25533	1.57575	2.04568	0.816102	2.74111	2.29886	0.025943	3.37502	0.332	-0.71136
ENSG0000	IFT81	12:110124	1.28372	1.85132	0.426503	0.389942	1.09724	2.03478	0	2.0626	0	0.491254	0.99545	-0.00511
ENSG0000	IL22	12:682482	0	0	0	0.740246	0.039152	0	13.326	15.9143	0	0.050092	0.2257	5.21829
ENSG0000	ELMOD3	2:8534208	20.2672	17.7376	17.1773	11.4273	11.1116	13.5586	15.0721	12.029	33.8881	19.0695	0.45015	0.334297
ENSG0000	DISP3	1:1147916	0.098077	0	0	0	0.009604	0	0	0	1.65971	0.468751	0.24705	4.60322
ENSG0000	PCK2	14:240801	12.969	7.9548	7.57344	9.7618	6.40107	16.0016	24.0316	15.4435	5.34343	14.2644	0.3397	0.704124
ENSG0000	PABPC1P1	4:3982286	0.174253	1.0689	0	0	0	1.81911	1.57572	0	0	0.172242	0.57515	1.54912
ENSG0000	GID8	20:629381	9.94447	8.2137	8.78419	5.79426	8.9504	8.91458	9.45049	9.72248	8.72196	10.4076	0.66695	0.157722
ENSG0000	RAB8B	15:631894	54.7991	30.9671	12.4988	10.3808	21.6428	38.8708	29.8633	26.1564	12.9604	25.992	0.5925	0.262752
ENSG0000	ALKBH4	7:1024268	11.5733	13.7685	27.2262	20.5514	24.9746	10.5414	5.49846	9.10832	5.67804	12.3139	0.18795	-1.2726
ENSG0000	VPS54	2:6389214	12.9605	10.5191	2.93654	1.57991	2.53316	10.1922	8.69994	4.0227	2.19216	7.11221	0.68355	0.222822
ENSG0000	UBE2K	4:3969804	16.1024	19.2052	24.2436	25.7033	31.052	14.0408	34.0396	37.3414	53.4788	34.4847	0.7741	0.188834
ENSG0000	WASH5P	19:60950-	11.134	10.9274	7.03959	7.56899	2.64298	13.8761	9.3718	7.70065	3.70792	10.794	0.67695	0.382462
ENSG0000	ZNHIT2	11:651164	16.8211	21.406	36.6185	28.6057	43.616	24.9273	29.9283	24.8824	17.623	11.8753	0.24705	-0.53108
ENSG0000	NAF1	4:1631087	2.3265	1.64911	2.40656	5.92383	2.12242	0.045692	3.17242	1.18806	0.930483	2.71452	0.38675	-0.91612
ENSG0000	CYSTM1	5:1401746	8.58919	4.20253	7.04338	15.4444	7.23287	30.3265	12.4016	23.1212	21.3489	3.88974	0.4067	1.08033
ENSG0000	THAP12	11:763499	41.7099	29.7217	11.7675	5.31706	13.7238	17.4965	20.2949	7.79205	4.83046	18.9478	0.1729	-0.53522
ENSG0000	ADAM12	10:126012	15.0915	1.26804	6.18271	5.39802	9.5228	2.78856	0.248366	6.69625	8.14911	10.0903	0.1992	-0.64324
ENSG0000	BBS9	7:3312924	9.37729	5.1701	2.69665	4.25523	4.90914	8.32921	3.77179	3.80249	7.29182	11.3602	0.70025	0.206439
ENSG0000	HMBOX1	8:2889039	21.1171	28.1457	9.94692	12.1644	4.63922	18.7155	12.9955	13.177	13.9499	12.5195	0.8696	-0.0707
ENSG0000	TOMM70	3:1003634	15.7363	13.9884	5.67277	3.19099	5.20838	9.41805	9.26471	6.71598	2.86513	11.5699	0.7521	-0.127
ENSG0000	GGA2	16:234635	26.6824	37.5478	20.4972	23.4416	14.0687	38.9857	44.7304	36.3861	40.1244	30.4985	0.2433	0.460728
ENSG0000	NFYC-AS1	1:4069037	2.30818	1.18783	1.34585	2.35547	0.729564	0.576629	2.67402	1.64714	5.24992	3.04239	0.6498	0.80411
ENSG0000	ADCY9	16:395338	0.778291	0.972223	1.01242	0.712139	0.817849	0	1.10884	0.480021	0.285876	1.15491	0.4889	-0.57021
ENSG0000	CPEB2	4:1500267	1.50055	1.08152	0.491907	1.55964	0.921653	3.99062	1.22695	1.57326	1.80879	2.26142	0.21875	0.790553
ENSG0000	ALG13	X:1116658	26.634	18.3642	21.3378	27.4143	10.4422	17.5093	22.6056	17.0124	17.1601	12.378	0.5525	-0.258
ENSG0000	SEMA4F	2:7465422	3.33081	2.68687	1.64718	4.23679	3.66457	2.38947	2.00782	2.61548	2.43719	5.72918	0.41735	-0.43671
ENSG0000	AMZ2	17:682466	100.26	59.7364	67.1747	66.8334	59.3596	96.9721	83.4675	77.342	55.6401	54.0088	0.65545	0.145156
ENSG0000	GINS3	16:582950	2.63611	6.92203	1.36823	3.24759	2.40874	1.37013	1.34533	1.28698	0	1.97004	0.12575	-1.47472
ENSG0000	DXO	6:3196980	31.096	20.8013	22.5412	21.2669	15.7696	11.4415	25.2689	23.3609	18.8758	27.8398	0.838	-0.095
ENSG0000	SMUG1	12:540582	17.3113	18.8795	30.5539	38.1256	25.5609	23.9303	12.4957	39.2022	30.6939	14.4636	0.7744	-0.14293
ENSG0000	VPS26B	11:134068	35.6277	39.1254	26.1448	21.9834	25.8188	34.9834	33.0467	21.9795	18.5974	30.3524	0.7888	-0.11336
ENSG0000	HIST1H4J	6:2782410	17.3013	8.03559	19.6	22.802	10.0726	15.1209	20.191	10.9595	23.6539	3.14402	0.91025	-0.09697
ENSG0000	KIFC1	6:3339153	5.15836	10.9886	0	1.14102	0.289024	7.06348	5.47193	1.67699	5.83734	1.67057	0.3792	0.643923
ENSG0000	NPIPB2	16:119273	1.88412	2.57611	0.094066	1.54263	0.915086	1.6546	2.02334	0.195217	0.623361	0.518529	0.76025	-0.46679
ENSG0000	USPL1	13:306176	14.4522	15.6069	17.1033	9.59462	16.2232	9.03626	11.0415	20.4795	10.1531	30.4418	0.9217	0.040161
ENSG0000	FTH1P3	2:2738119	0.130184	1.11819	0.18884	0	0.579799	1.25059	4.004	1.41367	0	0.346191	0.3153	1.71601
ENSG0000	RP11-727.	11:831569	3.14736	1.99247	1.66307	3.81443	1.42413	3.2663	1.95447	2.0464	3.17983	6.45188	0.58725	0.432785
ENSG0000	UBA7	3:4980520	66.8625	129.227	29.5038	79.3941	20.6182	58.7321	60.4366	28.5945	37.9669	38.3885	0.0675	-0.57759
ENSG0000	KLHDC1	14:496931	1.33625	3.82269	1.38203	2.54837	1.24666	1.89862	0.598476	1.21985	0.094741	2.39984	0.1852	-0.94343
ENSG0000	ETV5	3:1860463	0.035781	0.201143	0	0	0	3.37876	0	0.988752	0.799327	0	0.0085	5.15002
ENSG0000	SPCS2	11:749492	102.573	84.1711	164.934	126.579	150.492	126.088	155.407	121.168	101.049	88.0211	0.64885	-0.19787
ENSG0000	NBPF19	1:1493906	4.75	13.8808	3.70788	3.56587	5.7963	7.943	7.74882	4.82981	6.6252	10.7385	0.7793	0.17707
ENSG0000	MYSM1	1:5865473	8.0714	7.38741	4.0732	2.59003	3.51477	2.74603	11.9489	3.52596	6.99596	7.213	0.83205	0.155464
ENSG0000	DYNLT3	X:3734927	41.7946	19.9702	38.0413	55.3503	23.495	30.8755	27.0476	38.8585	40.372	26.9805	0.8051	-0.11947
ENSG0000	DHX58	17:421014	6.03287	9.80041	10.9746	8.28541	6.75361	7.56926	17.7143	25.9775	4.92701	13.3284	0.08845	0.796616
ENSG0000	CH507-24	21:622896	0.544374	0.815754	1.51657	0.449458	0.344905	1.91146	1.2182	2.82409	0	0.901118	0.10165	1.54839

ENSG0000(	SLC25A26	3:6613360	28.5559	24.629	52.5893	65.2912	47.4085	39.5823	38.7521	58.0518	52.4295	12.4988	0.79775	-0.19138
ENSG0000(	CDK7	5:6923479	21.5722	15.473	29.6229	40.6964	14.8775	33.2227	21.126	27.0563	29.0909	12.8625	0.85965	-0.069
ENSG0000(	RP11-356	6:1378236	28.6576	14.9532	26.673	43.8723	10.5866	23.8402	27.0153	17.5002	41.262	37.7608	0.7303	0.233801
ENSG0000(	RPL27A	11:868241	697.66	555.903	1382.69	965.552	414.522	631.585	653.531	847.567	647.575	65.7348	0.20085	-0.57357
ENSG0000(	LA16c-OS	16:185747	0.783948	1.67063	0.205337	0.132316	0	0	0	0	0	0.408264	0.283	-1.9846
ENSG0000(	PPAN	19:101062	56.2887	48.1321	82.7435	52.0121	88.8765	65.543	61.8804	66.1392	35.6266	69.4554	0.83745	-0.12136
ENSG0000(	PLD2	17:480709	4.34246	5.38918	2.70559	2.60518	1.6771	12.1837	2.90155	4.58428	3.58752	3.40668	0.14325	0.690469
ENSG0000(	DEK	6:1822386	102.152	105.608	63.0482	58.3131	140.588	133.4	96.6748	90.4038	73.3324	126.94	0.9952	-0.00272
ENSG0000(	CD276	15:736839	0.325954	0	0.022541	0	0	2.26035	1.61138	0.094447	2.80919	0.345181	0.07175	5.0423
ENSG0000(	SOCS2-AS	12:935424	3.38333	0.582692	4.05197	4.14493	4.13476	4.78179	1.12528	1.518	0.331146	1.47923	0.463	-0.94615
ENSG0000(	RP11-345.	15:256772	0.47184	1.48587	0	0	0.152339	2.4043	2.88951	0.69477	0	2.33304	0.3005	2.05582
ENSG0000(	POLR2C	16:574281	21.6368	18.7468	43.7545	30.5386	24.9053	20.5593	22.0286	30.3723	34.3831	16.0584	0.7198	-0.22784
ENSG0000(	DUSP12	1:1617497	43.7795	26.7917	51.3379	42.171	35.835	49.2876	40.2076	40.3742	47.0306	23.2555	0.94135	0.031131
ENSG0000(	DUS2	16:679873	12.0527	14.3976	16.4583	24.5906	12.239	6.22407	11.8499	13.7129	11.8334	9.1558	0.51945	-0.51364
ENSG0000(	PDE4D	5:5896903	8.26429	3.88012	5.55319	10.302	7.30457	12.4083	12.6424	15.5623	20.3748	16.5944	0.0333	1.08448
ENSG0000(	EXTL3	8:2860046	3.65317	2.91775	1.36668	1.12297	1.90575	1.85778	1.77828	1.2738	2.27637	3.58968	0.99005	-0.00644
ENSG0000(	PDE7A	8:6564473	65.2309	74.6405	16.7484	12.5944	16.471	38.822	33.8178	21.9613	26.0943	57.747	0.8617	-0.06147
ENSG0000(	SRP9	1:2257778	230.558	194.079	213.321	240.605	111.39	208.334	218.482	174.589	273.842	61.7536	0.94365	-0.02565
ENSG0000(	LSM8	7:1181840	38.3643	32.9329	30.2083	34.6097	23.1178	52.8669	41.1651	43.4748	21.5906	21.3099	0.6198	0.197348
ENSG0000(	GNAI3	1:1095486	7.7398	7.07902	5.32592	5.89535	4.50788	8.4349	8.49793	5.58346	7.46068	3.66453	0.73765	0.134682
ENSG0000(	NOTCH2	1:1199115	3.45771	4.35372	1.76282	1.63884	3.41019	4.02502	4.20706	3.97195	6.5864	10.3352	0.12075	0.944163
ENSG0000(	ZBED4	22:498538	6.02031	3.77727	1.51724	1.50166	2.22542	2.25758	2.02029	2.2036	1.71872	6.52098	0.778	-0.14677
ENSG0000(	UHRF1BP1	12:100028	12.1653	4.63778	3.42827	5.50835	4.93496	2.16507	8.52414	4.59982	7.68444	4.67186	0.54665	-0.28759
ENSG0000(	CRTC2	1:1539476	28.3127	37.3942	14.685	11.9868	14.292	28.2187	15.7917	16.2164	4.23221	24.4336	0.558	-0.2296
ENSG0000(	RP11-4B1	18:769746	0.175556	0.44181	0.06787	0	0	0	0.553002	0	1.54029	0.053621	0.4325	1.89426
ENSG0000(	PP14571	2:2404356	0	0	0	0	0	0	0	0	2.06694	0	0.0066	inf
ENSG0000(	BRK1	3:1011559	138.482	97.9867	201.765	176.904	150.194	170.836	152.269	178.22	158.979	56.2827	0.6893	-0.13034
ENSG0000(	HNRNPH3	10:682826	74.1189	101.746	64.4293	55.3927	64.3912	66.3414	90.8335	73.2318	71.5916	102.721	0.6311	0.164733
ENSG0000(	DDA1	19:172921	17.1929	15.8569	21.9181	18.8111	18.0234	28.0228	19.499	27.8071	22.9386	14.1942	0.95775	0.049513
ENSG0000(	TBCEL	11:121024	3.47112	2.06737	1.10456	1.00868	1.04132	1.44914	3.41265	1.04933	0.985667	2.547	0.6743	0.2445
ENSG0000(	NME3	16:170618	53.7571	41.943	67.7636	55.5467	58.9135	71.0205	83.0981	64.8187	62.2878	26.5146	0.9266	0.049404
ENSG0000(	CNOT9	2:2185685	16.2621	12.3988	39.4064	22.7617	21.0932	22.749	18.6183	32.9641	28.2825	31.0385	0.8917	0.087166
ENSG0000(	RP11-17A	11:228137	0.893085	1.55619	1.67659	0.428188	2.13015	3.71547	0.4794	0.87096	0	1.44578	0.92455	-0.12503
ENSG0000(	HIST1H3J	6:2789038	0.205872	0	0.677897	0.866692	0	0.152567	0.79024	2.38873	0.170797	0.196082	0.44765	1.19941
ENSG0000(	INSL3	19:178165	4.14337	2.44516	1.30801	0.065583	2.97367	2.9552	1.57481	0.78975	0	4.54537	0.95845	-0.08109
ENSG0000(	RP11-548	2:8590427	0.892978	0.640546	0.805553	1.02454	0.695421	0	0.920953	0	0	0.109109	0.2882	-2.19179
ENSG0000(	CAPN10-/	2:2405826	0.477141	1.57856	0.425316	3.37681	0.127718	2.80849	1.32063	3.15722	1.99544	1.09077	0.2158	0.835368
ENSG0000(	FEZ1	11:125445	20.3242	0.612486	9.35888	11.004	15.414	1.62148	0	5.38685	14.3479	5.22135	0.30205	-0.95925
ENSG0000(	FLYWCH2	16:288318	11.6902	15.7358	28.03	17.7981	22.2885	12.8576	30.2832	23.5235	8.58893	6.14812	0.4796	-0.36531
ENSG0000(	CCDC88A	2:5528231	1.46902	6.97081	0.483861	0.614912	1.41089	3.83841	3.42943	2.25272	1.62908	4.70315	0.4292	0.673104
ENSG0000(	ASPH	8:6105715	4.42255	3.78199	3.03871	0.901082	2.67481	8.1474	1.47709	1.80968	0.58549	4.9918	0.72785	0.19636
ENSG0000(	HERC2P3	15:203794	6.11583	7.15252	2.0411	1.81327	4.27098	3.8835	3.93384	1.62098	2.95887	4.72537	0.7344	-0.27434
ENSG0000(	RAPGEF6	5:1314239	16.9864	19.5383	5.33322	5.28014	8.75423	11.9365	7.50859	10.1699	10.5959	25.6561	0.59485	0.207505
ENSG0000(	FBXL3	13:769906	24.1493	42.8842	7.37473	12.0968	6.43105	23.1559	18.5151	20.1169	16.7024	15.8195	0.83305	0.122797
ENSG0000(	COPS2	15:491060	18.4418	18.8265	15.3966	9.26197	15.2148	12.7406	23.9586	15.9776	12.9553	13.5924	0.8196	-0.12186
ENSG0000(	CTD-2555	14:643880	0.751219	1.20924	1.21408	0.48049	0.446537	2.5671	1.66367	1.91441	5.27189	0.307764	0.2237	1.54542
ENSG0000(	MCM3AP-	21:462292	1.50805	4.44814	1.47659	1.85034	0.834947	1.76386	2.00976	2.31515	0.042734	1.81325	0.9084	-0.10244
ENSG0000(	GPR68	14:912325	20.5923	5.28019	65.5467	27.7022	137.627	10.6098	26.3313	54.9696	26.0313	149.566	0.8874	-0.07154
ENSG0000(	PVT1	8:1277945	25.153	26.1282	38.8772	71.6825	10.2446	36.9811	20.3343	39.7065	37.2861	17.1855	0.77595	-0.14943
ENSG0000(	MAPK9	5:1802331	16.5514	15.0578	2.20243	2.26165	3.22061	5.30724	15.1957	4.62928	2.48536	5.30578	0.36655	-0.44424
ENSG0000(	CYB561A3	11:612994	42.3841	54.0018	27.3711	48.4299	31.924	57.4645	27.7951	27.9445	28.7593	46.951	0.89655	-0.08352
ENSG0000(	FAM177A1	14:350449	31.7277	17.1309	34.8746	55.8672	30.222	49.2791	74.2533	72.5011	68.3443	21.3267	0.36875	0.610687
ENSG0000(	B9D1	17:193375	0.86545	1.96	0.931909	1.92755	0.958196	0	1.36764	0	2.32073	0.124053	0.3408	-0.7588
ENSG0000(	RP11-481.	16:582950	0.642413	0.834481	1.05422	0.975877	1.38147	0	2.6392	2.61591	0	1.05272	0.9369	0.134508
ENSG0000(	CYB5R4	6:8385964	11.1066	10.5987	5.7608	5.57698	4.00385	10.2087	9.4107	9.78845	8.21383	7.90343	0.9675	-0.02594
ENSG0000(	SESTD1	2:1791016	0.877246	4.43396	1.2854	0.261321	0.105989	4.23915	2.80161	0.024332	0.047059	0.978445	0.92635	0.084235
ENSG0000(	FRS3	6:4177017	2.22937	2.12391	1.59442	0.348808	0.94985	4.39279	1.29599	1.93045	1.19416	1.99949	0.61515	0.671968
ENSG0000(	WDFY1	2:2238557	22.8631	15.9594	7.80432	16.0298	9.7756	13.8621	14.6456	10.1547	4.59041	17.5099	0.48095	-0.27294
ENSG0000(	PSRC1	1:1092795	1.25052	2.10914	0.348048	0	0.217052	2.18869	2.06341	0.657621	0	1.7005	0.3618	0.852262
ENSG0000(	TMEM45B	11:129815	0.32229	0.910985	0.356439	1.22313	0	0	0	0	0	0.277273	0.2634	-4.00032
ENSG0000(	UFM1	13:383498	55.1578	44.5386	25.7984	40.0638	16.5244	49.9915	38.8761	37.8011	52.4395	21.7405	0.43145	0.239545
ENSG0000(	UBR1	15:429428	9.63923	10.4751	4.5952	7.50752	5.33835	5.68633	9.93676	7.4889	3.38247	13.0272	0.7038	-0.1536
ENSG0000(	UBXN2B	8:5841126	9.01133	9.98179	8.02313	9.27642	10.9551	13.2341	8.21087	13.5957	11.9463	14.0213	0.65665	0.275945
ENSG0000(	PRPF31	19:541027	42.7885	59.9431	85.1081	83.4491	97.4838	32.3832	60.7938	75.529	67.2978	82.5383	0.56795	-0.23973
ENSG0000(	RP11-7O1	1:4394436	0.400399	0	1.11178	0.097177	0.481504	0	0.308108	0.493623	2.05107	0.378933	0.59945	0.728214
ENSG0000(	ISG15	1:1001137	166.597	134.379	367.074	397.071	302.173	347.767	1996.96	996.184	391.772	110.726	0.0061	1.20149
ENSG0000(	PASK	2:2411060	3.63916	11.4653	4.16203	12.0156	2.076	5.64583	2.37225	5.09605	17.3694	5.06687	0.72905	0.287234
ENSG0000(	GLCE	15:691605	4.84767	8.22844	0.99131	7.19906	1.47795	1.43514	1.47257	2.23847	0	1.42918	0.2111	-1.8327
ENSG0000(	ARRB2	17:471048	24.5526	16.1714	7.97538	5.94551	13.3946	20.6593	15.8856	7.86403	12.321	19.4223	0.6669	0.186541
ENSG0000(	RP11-38M	7:1344162	0.07361	0	0	0	0	0	0	0	4.80474	0	0.484	6.39179
ENSG0000(	NGDN	14:234696	64.8506	80.3394	96.5445	93.6243	74.2891	70.3091	77.9892	53.6325	58.6448	47.5765	0.183	-0.43082
ENSG0000(	NAA38	17:785668	75.1061	37.6046	110.938	116.56	65.342	99.2153	90.3633	77.9957	55.1409	13.3128	0.6606	-0.41047
ENSG0000(	EXOSC2	9:1306937	12.7487	16.7401	21.4887	21.9151	18.803	12.5028	12.9413	16.0179	6.91412	9.12303	0.11125	-0.67788
ENSG0000(	GLB1L2	11:134331	0.36375	0.894486	0.698438	0.581525	0.454784	0	2.39799	0.796415	0.607825	0.306802	0.773	0.33034
ENSG0000(	KIAA0100	17:284557	16.887	17.4676	5.27119	4.45853	10.4837	11.825	12.1734	6.75446	9.89231	14.7808	0.8518	0.105965
ENSG0000(	ITPK1	14:929369	16.2596	18.3233	3.36231	1.68874	8.86742	7.85167	18.2935	7.66916	4.05794	13.8471	0.83515	0.095883
ENSG0000(	RP11-196	16:310744	1.98921	0.509422	2.47854	1.62263	1.37483	1.04632	7.41853	2.92207	0.17551	1.7507	0.6183	-0.70163
ENSG0000(	TOR3A	1:1790813	41.8339	28.4656	38.1376	49.3438	30.6966	45.7632	41.6979	31.2563	38.8424	32.7469	0.8754	0.048542
ENSG0000(	CGGBP1	3:8805194	61.1909	57.4662	16.4572	21.186	33.9605	48.4229	51.3675	31.539	37.3456	57.4116	0.5968	0.243933
ENSG0000(	MRFAP1L1	4:6707700	152.824	133.446	171.069	142.584	150.521	145.305	171.595	107.333	109.713	141.116	0.5698	-0.18552
ENSG0000(	CTC-303L	5:9415296	0.28106	0.663815	0	0	0.055544	5.37309	4.84838	1.04676	4.51114	0.37763	0.0836	4.23924
ENSG0000(	FBXO2	1:1164836	13.0128	2.23477	2.41896	2.71504	8.72945	10.4987	23.2299	8.3264	1.10247	8.9557	0.5892	0.62473
ENSG0000(	KLHL6-AS	3:1834875	0	0.249665	0	0	0	0.11619	0.470537	0.207442	1.34349	0.110388	0.2889	3.43966
ENSG0000(	METTL21A	2:2072396	24.4992	22.7534	27.2301	24.5081	18.2279	5.40268	6.8337	18.9596	12.2397	10.2687	0.0868	-1.19336
ENSG0000(	PDGFB	22:392233	9.7154	2.75665	11.9089	7.19699	10.4014	0	0.80467	1.48114	4.98608	8.86921	0.05825	-1.50297
ENSG0000(	NCK2	2:1057448	60.6935	75.7437	20.8794	10.1861	24.4778	37.2876	23.1849	14.062	11.8767	24.4488	0.09175	-0.65674
ENSG0000(	CTB-5E10	19:137645	1.52407	1.15215	0.502135	0.060019	2.5913	2.29445	2.06148	1.56227	1.00235	1.14928	0.8062	0.306745
ENSG0000(	FAM188A	10:157781	10.7761	10.6444	4.10174	5.8918	4.62681	7.29032	9.27515	7.44961	11.4938	7.86962	0.4878	0.319781
ENSG0000(	SRSF4	1:2914774	97.9516	106.98	54.6465	44.9257	78.9939	76.4494	79.4946	62.5322	33.184	79.3848	0.5552	-0.1933
ENSG0000(	ZYX	7:1433810	58.4052	93.3223	33.2362	26.6588	51.6766	80.855	116.572	99.5668	65.271	141.256	0.00745	0.966013
ENSG0000(	RP11-471	14:903965	3.83571	2.32187	0.588224	2.10146	0	1.12863	0	1.11413	0.266766	0.536475	0.53435	-0.92748

ENSG0000	ZFP36L2	2:4322240	332.166	466.351	243.559	456.354	362.287	107.01	183.507	225.993	299.82	328.164	0.0976	-0.68196
ENSG0000	GPNMB	7:2323596	0.40082	0.955024	0.451642	0	0	181.91	6.07341	1.21059	0.077608	1.4799	0.00975	6.52208
ENSG0000	SBF2	11:975476	2.98483	1.69045	2.23311	3.01795	2.90832	0.480689	1.30288	2.10129	0.038124	4.28764	0.42325	-0.81974
ENSG0000	SLC39A6	18:361085	18.43	19.0386	15.9391	12.8802	11.5007	11.8929	22.4529	14.6521	24.2278	14.2316	0.674	0.154024
ENSG0000	MRPL15	8:5413520	43.3625	33.2546	38.6082	42.8673	36.773	66.5654	63.525	33.132	48.2509	18.1891	0.5469	0.221249
ENSG0000	CRTAM	11:122838	15.0137	17.1775	13.7272	7.71098	20.6027	14.3744	37.8474	39.4224	6.08418	23.7399	0.05825	0.758795
ENSG0000	DPY30	2:3186505	121.99	89.4323	152.202	116.488	106.956	152.969	151.798	121.425	97.2676	30.3905	0.80115	-0.11134
ENSG0000	PABPC1	8:1006858	178.834	215.233	187.906	150.365	352.902	175.514	146.234	234.389	199.804	419.377	0.7258	0.141802
ENSG0000	CD2	1:1167543	235.841	300.767	346.201	287.365	343.339	921.48	881.328	717.589	557.709	496.158	0.00575	1.19012
ENSG0000	ZZEF1	17:400444	5.31138	3.98465	3.21812	2.74013	2.80355	5.23709	3.90898	3.00624	1.4489	8.23182	0.61875	0.211394
ENSG0000	IKZF5	10:122930	13.7796	11.7897	5.24602	10.0361	5.50959	7.53472	9.60222	10.6749	11.6813	8.73113	0.95865	-0.02253
ENSG0000	CD27	12:643900	300.119	339.543	421.47	348.925	449.258	203.454	265.021	126.178	196.917	110.657	0.02135	-1.09141
ENSG0000	SMC5	9:7025896	20.0283	23.6111	5.36024	7.4727	13.0709	28.4613	21.725	6.9447	9.32467	20.0861	0.15155	0.497999
ENSG0000	RPL12P16	2:2030148	5.30034	5.30868	2.37836	2.80611	0.807552	5.08845	3.86841	2.4919	0.294133	0.097996	0.81475	-0.50008
ENSG0000	ACTR1A	10:102461	38.4983	37.2672	34.2036	37.6483	35.2539	47.3449	29.9416	26.0993	21.8489	35.5442	0.4218	-0.25854
ENSG0000	MICU1	10:723673	19.1487	22.1091	11.2232	11.5141	17.4082	22.927	17.6986	14.5712	4.9426	22.3318	0.769	-0.11033
ENSG0000	RGS14	5:1773578	91.4604	100.999	79.6404	92.9571	89.2533	59.4419	67.0114	52.2112	51.9064	80.3985	0.0671	-0.57407
ENSG0000	GMDS-AS	6:2245747	8.23741	7.6771	4.01037	8.15733	3.10663	8.18408	10.4437	7.65979	4.64944	5.74756	0.65445	0.281797
ENSG0000	NBPF1	1:1656231	6.52226	15.7466	5.86753	5.84076	3.54816	1.49746	4.37511	3.19056	5.27187	5.57907	0.2183	-0.88207
ENSG0000	DTNB	2:2537719	12.6784	20.7644	11.1118	6.43469	7.74952	10.7688	14.1274	8.80841	11.0223	17.5008	0.7968	0.110064
ENSG0000	GBGT1	9:1331529	0.117439	0.822417	0.163342	0	0.284961	3.59087	0.841874	0.678998	0	0.694741	0.0933	2.10494
ENSG0000	LGALS3BP	17:789712	50.7558	64.489	62.0784	62.4896	37.5603	107.793	26.3059	43.7908	66.0353	56.4445	0.76815	0.095659
ENSG0000	GEMIN7	19:450714	46.2146	40.7737	83.4226	70.5977	52.1818	42.2567	49.0669	45.7012	38.8231	14.0385	0.38865	-0.72867
ENSG0000	QSER1	11:328931	0.630767	0.696585	0.478082	0.133984	0.916063	0.615493	0.972455	0.203362	0.009377	1.28147	0.9686	0.027234
ENSG0000	RP11-429	20:612524	0.23379	0.814615	0.267894	1.06179	0.339969	0	0.147185	0	0	0.176283	0.4337	-3.21788
ENSG0000	ABHD18	4:1279178	6.49573	6.15264	5.90252	3.81437	2.37277	10.5854	8.79064	5.97289	9.15034	5.77501	0.39635	0.711404
ENSG0000	PMS1	2:1897703	32.233	24.3255	18.2612	25.6122	11.0012	17.4426	11.1269	12.4969	4.38335	16.3746	0.2737	-0.67343
ENSG0000	CDKN2D	19:105664	77.2203	74.0797	141.894	122.313	123.947	47.4682	48.9566	62.4443	64.4533	31.3184	0.00295	-1.10732
ENSG0000	ETFDH	4:1586666	13.1469	13.9513	11.3692	12.0766	8.913	14.1141	12.2799	17.234	19.4952	11.6895	0.7414	0.221611
ENSG0000	TRMT1L	1:1851180	11.8063	12.3491	4.40933	3.23585	4.28641	7.54285	11.6226	5.78516	2.10181	8.95547	0.9354	-0.03469
ENSG0000	SLC2A1-A	1:4292537	0.262309	0.029349	0.435232	0	0.14362	0	0.436952	0	3.82908	0.591684	0.3274	2.44975
ENSG0000	CTC-499J	5:1112236	0.481654	1.68023	0	0.398761	0	0	0.797684	0	0.599306	0	0.7177	-0.73934
ENSG0000	TMX3	18:686736	18.8436	15.2747	4.34338	10.2782	4.69928	13.5797	11.4395	8.18	11.5768	11.6447	0.79525	0.098605
ENSG0000	TCEAL4	X:1035762	12.1378	13.1994	2.71928	1.34549	9.86543	7.28093	10.4567	2.51105	0.591489	4.64116	0.27725	-0.6692
ENSG0000	AGPAT4	6:1611299	10.0426	8.98913	6.08176	20.1011	9.33998	4.25057	4.97798	2.56083	17.2588	1.68293	0.42555	-0.87181
ENSG0000	ENTPD7	10:996595	0.398775	0.624752	0.476159	0.3689	0.302145	0.881368	1.47189	0.322992	1.28466	0.140456	0.51395	0.948341
ENSG0000	PUM3	9:2717501	31.5144	20.1614	27.1424	25.2022	28.4578	45.2472	29.828	19.7315	36.278	28.1936	0.42935	0.291279
ENSG0000	DCBLD1	6:1172882	1.04161	1.38638	1.20899	0.757408	0.83726	1.02378	3.25196	3.6493	2.9015	2.81956	0.26495	1.27299
ENSG0000	ZNF396	18:353666	0.81039	0.16277	1.55684	0.755453	0.633164	1.70401	0.341341	0.924011	0.029234	0.719883	0.8849	-0.20445
ENSG0000	BCL2	18:631233	9.44622	12.4264	8.06668	18.843	22.3226	9.18186	12.0453	9.39993	21.9352	14.2933	0.64695	-0.20678
ENSG0000	TMOD1	9:9750117	2.89572	1.44899	0.683719	0.414695	0.854207	2.91614	0.494819	0.276062	0	1.0539	0.986	-0.03774
ENSG0000	TIMM17A	1:2019554	54.5852	36.0928	46.0599	52.0646	38.0711	71.5596	75.4642	44.8531	65.1014	19.4246	0.3604	0.29947
ENSG0000	TBC1D25	X:4853945	6.06451	8.57589	8.22438	7.54835	8.92418	5.15224	5.01893	6.93852	1.82292	10.2496	0.18465	-0.56887
ENSG0000	RP3-477C	20:354551	11.3877	4.8882	11.3923	13.0193	8.91633	16.5223	7.04429	6.43225	3.12021	3.67403	0.6672	-0.51206
ENSG0000	ADAM28	8:2429403	0.194538	5.02872	0.52852	2.45503	0.495115	6.15313	0	1.06455	0	0.439416	0.78605	-0.32149
ENSG0000	TMEM68	8:5569642	13.9343	9.7566	15.1974	9.5488	8.35465	17.1775	17.4975	13.7554	9.16267	8.74897	0.7602	0.17063
ENSG0000	PMM2	16:867456	16.423	20.041	23.8869	32.4985	20.6851	19.1495	12.9049	13.0227	8.997	22.2535	0.16685	-0.67053
ENSG0000	ANKRD35	1:1458665	0.145387	0	0.17639	0.387134	0.061266	1.45464	1.12496	2.14666	0	0.328472	0.3826	2.44049
ENSG0000	AC011997	2:1976931	0.136299	0.548774	0	0.082015	0.104193	0.713745	2.72448	0.779146	0	0	0.3263	2.56058
ENSG0000	TXNRD1	12:104215	47.7613	26.1095	19.8646	29.9095	19.2285	33.5255	20.7004	22.1198	30.1395	19.0526	0.44915	-0.29865
ENSG0000	FOXJ2	12:803270	6.20072	6.25845	2.5243	1.96035	3.54461	2.50872	1.64583	4.255	0	7.93287	0.3869	-0.50328
ENSG0000	RP1-228H	1:4046431	0.853433	1.34914	0.324896	0.083285	0.64549	0	0.616517	0.220135	0	1.17107	0.50155	-0.78308
ENSG0000	TSC1	9:1328913	6.74706	9.4582	5.78805	7.52443	7.89263	6.29721	3.46923	10.1435	4.57182	12.5893	0.81545	-0.14847
ENSG0000	MYL5	4:672435-	3.94225	0.523118	2.99118	4.7331	1.53211	8.21828	5.36596	2.56495	2.91831	1.11739	0.40335	0.701218
ENSG0000	RP11-124	10:172142	0.107714	0	0.295934	0.277887	0	0	0.269385	2.30997	0.37042	0.100196	0.33865	2.10093
ENSG0000	HEXA	15:722411	61.1862	60.0684	75.7143	90.4283	51.0772	69.0172	55.2074	48.6455	80.5711	59.07	0.7991	-0.11246
ENSG0000	MTCH1	6:3696814	50.1021	64.6971	42.6517	50.635	45.688	56.2878	68.7986	55.9095	85.3619	55.978	0.28795	0.357393
ENSG0000	TAL1	1:4721628	0	0	0.726972	1.32486	0	0	0	0	0	0	0.05425	#NAME?
ENSG0000	TRPV2	17:164145	64.3753	47.7794	59.6243	74.6465	60.7132	38.2053	57.9165	60.3313	40.3139	58.5383	0.39515	-0.24846
ENSG0000	TRAV40	14:222025	1.1445	4.86589	0	0	0.306743	1.00059	0.757989	0	0	0	0.46685	-1.94999
ENSG0000	RP11-138	17:476494	4.14405	4.92179	3.17549	7.00492	9.5353	1.56209	4.51304	8.33315	3.18754	2.42861	0.73135	-0.65472
ENSG0000	SEC11C	18:591394	122.875	69.2423	146.708	154.657	98.6744	106.589	186.798	148.945	173.509	24.0797	0.586	0.201089
ENSG0000	LDLRAP1	1:2554357	36.9969	270.484	41.9284	27.5207	39.2643	12.5865	12.2741	13.9232	7.35739	33.3399	0.00005	-2.36654
ENSG0000	CLOCK	4:5539595	3.09203	2.16452	0.986611	1.19754	2.34906	0.552846	1.56017	2.30269	1.05547	2.96243	0.6025	-0.44131
ENSG0000	TSTA3	8:1436126	68.9947	47.2518	85.9459	53.5351	77.7221	108.291	106.761	88.6024	78.9556	38.5446	0.4042	0.28435
ENSG0000	C15orf61	15:672010	5.36854	4.76671	10.3946	11.6772	4.49023	4.4939	7.76186	5.52057	6.03079	2.50449	0.52475	-0.44171
ENSG0000	E2F3	6:2040190	5.37505	4.48028	4.90505	3.18793	4.17792	2.58961	2.21866	3.14756	0.371098	5.04037	0.1864	-0.82992
ENSG0000	STK38	6:3649389	85.7604	121.827	41.4554	29.0208	36.6859	30.5724	27.1082	28.5597	14.0459	29.6407	0.00015	-1.28433
ENSG0000	MTCH2	11:476173	26.9813	30.8118	35.0724	34.2525	33.3749	47.1204	42.2633	37.3402	15.9511	17.9977	0.5919	-0.18168
ENSG0000	RP11-390	17:395669	1.56503	2.73791	0.630416	1.45175	2.68827	4.20382	2.80857	4.12732	0	4.08804	0.45555	0.82888
ENSG0000	AP3B1	5:7800052	17.0483	22.8055	6.7074	4.31351	9.36334	10.6039	17.1813	8.14615	4.17926	15.2525	0.6368	-0.17024
ENSG0000	CTC-432N	5:1314239	0	0.251845	1.29466	1.54211	0.480105	0	0.370393	2.94084	3.63256	3.95725	0.9302	0.111771
ENSG0000	LINC0135	1:5893364	1.1111	1.59199	1.04813	1.27792	0.443447	3.95928	5.16282	1.46124	0.690407	1.88629	0.10045	1.49753
ENSG0000	CLEC5A	7:1419078	0.14735	0.515724	0	0	0	6.09885	0	0	0	0	0.3454	3.14061
ENSG0000	LINC0158	14:499275	8.42702	5.29979	4.23988	2.3521	4.14078	7.92659	13.473	5.44041	2.88592	9.9388	0.1159	0.77105
ENSG0000	AC093838	2:1314928	7.00994	9.7462	2.82673	1.8858	2.3171	8.40561	7.76475	5.72308	3.34342	7.01916	0.25165	0.506139
ENSG0000	RP11-804	11:608359	0	0.106518	0.046669	0	0	0	0	0	0	0	0.51635	-1.45857
ENSG0000	MASTL	10:271101	2.46432	5.06434	1.5224	3.74425	0.920425	2.43874	6.11704	2.11679	0.734974	2.62377	0.9496	0.075831
ENSG0000	NET1	10:541255	1.63673	10.0258	0.512074	1.55736	0.339392	0.984942	2.09398	1.13952	0	0.47672	0.18055	-1.51806
ENSG0000	NCOA7	6:1257811	21.7122	15.8758	16.4068	20.4488	33.9838	10.649	20.5811	14.6803	30.5121	32.0105	0.75855	-0.1958
ENSG0000	RP11-121	16:183179	7.36378	6.73826	0.5126	0.973017	1.32921	1.44575	1.60718	1.11423	0.919266	3.13031	0.35205	-1.07322
ENSG0000	B3GALT2	1:1931220	5.30993	0.380462	6.22237	4.38318	3.83013	1.75991	0.440442	8.62913	8.30052	7.85242	0.77175	0.299772
ENSG0000	ZFYVE1	14:729694	9.12109	6.21363	3.34471	3.59167	3.87056	4.48512	10.6578	3.2094	7.52711	6.70604	0.60125	0.231752
ENSG0000	TBC1D24	16:247505	1.05504	0.90218	1.33804	1.27724	1.31365	1.25406	0.993382	2.47905	1.01466	3.3728	0.49815	0.681665
ENSG0000	TPR	1:1862962	66.9782	100.904	58.886	43.6458	77.0354	99.8438	80.1637	45.1578	53.8523	93.5702	0.9837	0.008898
ENSG0000	RAB27A	15:552029	44.8283	25.8464	30.1621	37.4354	29.1937	78.4794	93.4742	74.3236	39.7477	38.7177	0.1944	0.733515
ENSG0000	TTC13	1:2309062	20.6018	24.1022	9.22629	10.615	5.53651	16.3026	11.8882	8.79352	11.0393	13.0691	0.5413	-0.21554
ENSG0000	UNC93B1	11:679911	15.6943	25.6293	17.4842	15.4823	21.4993	24.6053	20.9929	24.6473	11.3365	19.2334	0.9853	-0.00715
ENSG0000	F2R	5:7640324	17.2275	8.23167	22.3307	16.916	10.0401	29.09	18.4504	43.5748	26.3897	29.5013	0.16715	0.916484
ENSG0000	ACOT4	14:735917	4.94976	0.869874	5.19479	4.81286	8.42063	7.91179	4.75508	8.44129	2.13818	3.22977	0.96155	0.027103
ENSG0000	AC027612	2:9158949	3.47616	1.79127	1.9331	0.095687	0.657644	11.4193	3.65629	3.40049	0	1.03136	0.1413	1.45576

ENSG0000	DNAH17	17:783786	0.276436	0.523553	0.105971	0.186034	0.064725	1.50271	0.444818	0.475385	0.016079	0.469497	0.191	1.37746
ENSG0000	ZGPAT	20:636578	56.7019	65.3875	77.9285	55.4532	68.392	62.4357	50.8329	45.7656	25.2621	69.6403	0.59355	-0.4412
ENSG0000	WDYHV1	8:1234167	8.74041	10.9556	5.96686	9.97235	4.5819	7.17284	12.0184	7.06103	3.06851	3.46222	0.6703	-0.28754
ENSG0000	SNHG21	15:827505	15.1928	19.4934	29.7829	26.8888	14.8743	12.3633	19.7794	12.7038	11.0934	12.7759	0.39565	-0.62506
ENSG0000	CTD-2287	5:1160838	1.27989	1.56832	1.04869	1.53126	0.652713	0.294634	2.16505	0.961816	3.11922	1.80188	0.76835	0.454196
ENSG0000	MTUS1	8:1757643	0.907998	4.18205	0.719462	0.053467	2.24139	3.83032	0.972663	0.537103	0.179708	1.96707	0.87145	-0.09889
ENSG0000	IGKV1-17	2:8911734	0	0.890906	0.492932	1.89076	0.756069	0	0	0	0	0.600159	0.3507	-1.66874
ENSG0000	RANBP6	9:6011042	10.5195	10.2964	3.49523	3.66698	3.1831	15.186	3.94698	4.54925	4.25131	4.7391	0.91185	0.047586
ENSG0000	RAB3A	19:181931	0.260734	0.636939	0.518941	0.31679	1.99447	0	0.61869	0.349705	0	2.32271	0.8589	-0.21278
ENSG0000	RP11-755	11:662448	0.401312	0.084189	0	0	0.10293	1.57969	0.20362	0.219634	0	0	0.3038	1.68821
ENSG0000	GPR34	X:4151493	0.329496	0.280329	0.25253	0	0.063694	22.9598	24.2301	9.33215	1.48306	6.25382	0.0026	6.16389
ENSG0000	AC005076	7:8715142	1.50778	0.63853	0.049129	0.585029	0.523448	0	0.209946	0	0	0.795615	0.36725	-1.71229
ENSG0000	CTPS1	1:4097933	23.3334	18.382	10.164	22.8669	9.32144	16.1606	13.7536	11.7307	6.14062	12.0567	0.1595	-0.53412
ENSG0000	ADAM17	2:9473657	6.25269	5.27942	2.50866	2.94535	2.40533	2.72273	3.61957	5.06938	2.37819	5.0153	0.928	-0.07161
ENSG0000	NAPRT	8:1435734	54.2634	47.417	52.3308	33.2117	40.0718	72.8257	44.8849	57.5534	38.7181	54.141	0.69625	0.239189
ENSG0000	FLVCR2	14:755748	2.26666	1.84792	0.177879	0.254846	0.72881	24.3206	8.89146	3.7805	0	1.643	0.0787	2.83709
ENSG0000	RP11-799	17:351478	0	3.5163	0	0	0	0	0	0	0	0	0.15755	#NAME?
ENSG0000	CKS2	9:8931119	53.0845	50.412	41.5898	44.9991	15.1428	110.03	159.18	59.6325	44.1598	10.5288	0.03455	0.897058
ENSG0000	RP3-333H	22:393499	0.176924	0.91402	0.268675	0.691401	0	0	0.824981	0.5785	0	0.339612	0.961	0.055122
ENSG0000	CMB9-55F	11:777577	0.556618	0.725151	0	0	0.356243	0	1.18247	0.743704	0	2.24949	0.3959	1.46235
ENSG0000	DNAJB14	4:9989624	23.1181	14.6711	13.4028	13.6095	14.5246	32.0606	26.379	16.5577	17.0577	32.652	0.50885	0.305461
ENSG0000	SCRN2	17:478316	59.8588	48.8832	88.394	80.7268	43.2199	50.9485	37.886	59.998	72.2368	44.7911	0.47525	-0.23187
ENSG0000	SMG1P7	16:702195	0.954159	3.09313	0.933117	0.687725	0.808588	0.074658	2.99854	1.09435	0.08597	2.44178	0.78975	0.289497
ENSG0000	GTF2IRD2	7:7509257	15.2216	25.114	23.0092	22.9245	17.0429	20.8884	19.9172	27.5391	25.1309	20.1752	0.85575	0.128686
ENSG0000	TUG1	22:309706	8.96981	16.1525	9.62524	6.09235	8.21551	8.64609	7.32912	12.3391	6.88764	33.4704	0.4789	0.276202
ENSG0000	FAM20B	1:1790258	2.59766	3.2157	1.01473	1.06817	1.15808	2.3474	1.77219	1.35204	0.13081	2.16328	0.25545	-0.70684
ENSG0000	PTEN	10:878591	16.1754	16.6694	9.45947	8.38341	11.0435	11.7908	15.3893	12.9797	26.431	14.4995	0.38795	0.433618
ENSG0000	FAM122B	X:1347695	5.60797	8.48633	5.96045	3.48434	6.77787	9.92665	5.14425	6.80757	1.43571	13.7468	0.6099	0.254577
ENSG0000	ZNF324	19:584670	11.4524	12.9427	5.62157	4.18138	5.6639	3.07713	7.51842	2.99776	2.77714	12.7878	0.56375	-0.43104
ENSG0000	POLR2L	11:818901	98.8568	72.9277	227.939	278.267	124.744	131.487	146.619	174.418	181.768	30.7326	0.4433	-0.36471
ENSG0000	RCHY1	4:7547903	15.6159	11.1765	17.862	13.9027	15.1383	20.8279	8.55939	16.0694	17.1123	16.9632	0.8159	-0.10587
ENSG0000	DYNC2LI1	2:4377403	3.42798	2.66919	3.0403	4.53571	3.04061	2.38489	4.86665	0.978167	0	2.47357	0.4543	-0.73485
ENSG0000	AKAP7	6:1311356	19.6212	10.8237	14.183	7.60629	9.43406	2.79108	9.20619	7.25929	17.6786	4.40173	0.42305	-0.52966
ENSG0000	CDK2AP1	12:123152	39.9028	15.938	40.0954	33.7921	36.0495	44.9103	41.2599	26.646	28.0321	10.943	0.80255	-0.18789
ENSG0000	LL0XNC01	X:1027691	0.762261	2.99758	0.86731	0.213616	0.555835	1.45856	2.30125	1.0257	0	1.66867	0.87085	0.263379
ENSG0000	ATP5G1	17:488748	81.5501	67.5069	139.123	158.265	92.4387	132.673	114.958	106.822	128.504	23.8412	0.8421	-0.13618
ENSG0000	RPL10P12	12:686108	0.226161	1.60421	1.07487	0	0.176522	0	3.24198	0.780717	0.502204	0	0.8309	0.446589
ENSG0000	GALNT6	12:513512	1.75238	6.53727	1.69281	0.49046	1.63212	11.197	18.8355	4.56676	10.4424	10.6718	0.0002	2.1463
ENSG0000	MAST1	19:127630	1.19431	0	0.509254	0.464084	0.969621	0	0	0	2.77683	2.53561	0.5539	0.776914
ENSG0000	HABP4	9:9645020	11.0004	18.0554	12.7067	6.1866	13.9236	17.995	15.5603	9.89186	9.11931	18.3213	0.60555	0.215391
ENSG0000	USF1	1:1610392	1.53108	3.71478	2.38302	3.8818	4.08184	2.35935	2.63187	2.49535	6.39401	4.96685	0.6421	0.309359
ENSG0000	RP11-70L	9:2180254	0.178457	1.46546	0	0	0.021917	3.52798	0.510413	0	0	0.026085	0.6819	1.39679
ENSG0000	TMEM251	14:931849	27.1743	18.9287	25.6628	26.2282	16.3521	37.5347	27.8375	31.1925	38.8662	12.7204	0.5587	0.424426
ENSG0000	TTC1	5:1600091	82.9636	67.5862	81.6829	70.1137	69.6288	133.004	123.466	42.6001	58.7525	26.2324	0.8907	0.059065
ENSG0000	ADAM9	8:3899686	2.01181	1.2783	0.486568	0.618019	0.265654	11.0452	1.63496	0.7055	1.97632	0.69667	0.00875	1.82677
ENSG0000	RP11-196	16:310431	0.678237	0	0.592472	0	1.21939	4.93215	0.3939	0.219243	0	1.19353	0.428	1.22875
ENSG0000	RFT1	3:5290357	6.01354	8.00555	11.215	15.0883	8.27007	9.82659	7.84584	16.3103	15.8293	8.54532	0.88135	-0.10329
ENSG0000	CXCR2	2:2181252	5.20433	1.25371	0.9543	1.22141	0.791248	2.84373	0	1.12284	0	0.534915	0.20275	-1.19496
ENSG0000	HBG1	11:524808	0	0	0	38.6879	0.018005	0	0	0.8581	0	0	0.2886	-6.78627
ENSG0000	HIST2H2A	1:1498510	80.2914	22.2695	152.009	107.312	106.665	46.6057	48.0877	45.6796	63.3921	17.9546	0.70525	-0.78004
ENSG0000	MLYCD	16:838991	2.56828	4.60988	2.63157	0.672928	3.20975	3.12078	3.61237	2.95074	1.22459	3.92462	0.8535	-0.09971
ENSG0000	HLA-DMA	6:3293462	18.5104	68.4592	41.8124	25.6586	27.341	187.169	61.1355	88.2702	36.6315	26.1719	0.2932	1.06178
ENSG0000	PSMC1P1	3:6863570	2.72911	2.25551	1.54002	1.78455	0.695466	3.97575	3.04744	0.752136	1.22943	0.54852	0.75265	0.243128
ENSG0000	SKI	1:2228694	1.03528	1.16813	1.13906	0.144221	3.45645	1.16899	1.92882	4.28871	1.48062	10.6703	0.04735	1.37928
ENSG0000	RP13-270	18:306680	1.98702	4.86978	1.99223	2.67788	2.61947	2.91327	5.26202	1.77362	0.231332	0.560716	0.8263	-0.49642
ENSG0000	RP11-359	1:1625602	1.45759	0	1.45879	0	0.141448	0	0.032271	0	0	0	0.2762	-6.54903
ENSG0000	SAP18	13:211405	182.898	163.448	255.032	229.458	176.808	247.013	237.488	205.496	178.841	57.628	0.7443	-0.14632
ENSG0000	FUT2	19:486959	0.675197	0.010652	1.14705	0.184433	0.443326	0.234475	0	0	2.9517	0.598557	0.2814	1.31005
ENSG0000	IL7	8:7866604	1.86783	3.95136	0.228075	1.94512	0.31124	2.26964	5.51342	0.153835	0.354876	1.21006	0.86445	0.181584
ENSG0000	AVL9	7:3249542	5.65218	2.94174	3.3353	7.77742	3.24145	2.85938	5.1945	4.79439	5.01092	5.45281	0.7249	-0.25906
ENSG0000	MRPL33	2:2777171	208.912	132.286	203.712	183.833	42.9023	266.107	284.639	173.779	157.583	18.1965	0.80585	0.178623
ENSG0000	RP11-35C	15:986489	0.122613	0.323198	0	1.5867	0	0	0	0	0	0	0.13835	#NAME?
ENSG0000	PMS2P7	7:7296969	3.18302	3.39225	3.09916	4.87999	2.33033	2.8581	3.63012	2.29904	4.89334	3.15028	0.8483	0.320102
ENSG0000	TTN-AS1	2:1785211	18.0713	22.5246	9.6148	16.2275	4.1384	10.5364	19.9716	10.346	21.9085	11.3118	0.7829	0.212311
ENSG0000	FALEC	1:1505157	0.673189	0.426299	0.503983	1.56802	0.159415	0.715532	0.534521	0.956401	0	0.734736	0.9277	-0.15231
ENSG0000	RBM24	6:1728134	2.14514	0	0.768974	0.676204	1.25775	0	0	0	0	1.33236	0.2789	-2.48669
ENSG0000	CEACAM1	19:423971	1.74009	1.86624	0.73661	1.50195	0.329776	2.58718	3.64352	1.20346	5.1724	3.41963	0.17235	1.12467
ENSG0000	SGTB	5:6558967	3.86548	5.91027	1.06613	1.42163	0.9294	6.28838	3.49272	3.40566	2.2399	1.84211	0.69665	0.351019
ENSG0000	THOC6	16:302261	37.9763	51.5001	30.6623	34.2478	29.118	37.6652	53.3054	33.4036	33.0866	30.5513	0.9647	0.017416
ENSG0000	POLR2J	7:1024649	64.2587	73.1008	59.8223	91.2559	46.2742	90.2908	88.2836	52.947	54.9485	15.1557	0.6524	-0.23652
ENSG0000	IGHV4-61	14:106639	0	0.290446	0	2.85134	0.194444	0	0	0	0	0	0.00095	#NAME?
ENSG0000	ARHGAP1	6:1295761	10.0788	3.69644	4.50383	4.03305	6.89758	23.8368	15.1978	11.2268	8.60925	19.7182	0.0011	1.34605
ENSG0000	IL17RB	3:5384657	0.856317	0.199296	0.055528	1.53592	0.097757	2.75771	1.46819	1.52748	1.86743	0.194084	0.3165	1.53456
ENSG0000	NDUFB2	7:1405184	238.743	124.306	311.231	328.264	191.012	326.499	295.236	278.966	236.836	52.7027	0.8946	-0.05943
ENSG0000	ATAD3A	1:1512150	10.7633	12.6012	5.80951	9.0856	6.39325	9.21994	14.3769	8.66299	5.37064	4.10401	0.74495	-0.14709
ENSG0000	PRRT2	16:298061	0.346349	1.25203	0.086203	1.00885	0.5679	0	0.655629	1.17803	0	1.79913	0.97175	-0.03641
ENSG0000	BRI3BP	12:124993	2.22074	2.38862	2.53292	2.18132	2.6892	4.35701	5.13129	2.68998	2.45329	5.01052	0.1334	0.655762
ENSG0000	R3HCC1	8:2327011	39.3155	38.7071	65.2784	60.2013	50.772	54.4719	54.047	51.7581	26.2712	30.1583	0.36715	-0.31874
ENSG0000	ZNF292	6:8715115	23.0481	26.4541	23.128	14.8127	23.5627	20.8301	24.6851	15.5235	24.5145	40.8284	0.5803	0.321371
ENSG0000	CORO7	16:431476	120.112	148.158	32.808	47.6054	56.7057	57.8559	98.1832	45.0937	24.0343	67.0744	0.1219	-0.50714
ENSG0000	C2orf68	2:8559854	11.3043	16.0784	17.639	16.0212	12.5811	14.9162	8.55984	20.1739	15.8319	15.3792	0.8515	-0.10738
ENSG0000	OTUD7B	1:1499378	0.724354	0.487342	0.944937	0.469331	0.779672	0.916089	1.1686	0.822008	3.02469	1.18959	0.25545	0.967229
ENSG0000	RP11-46F	1:3623189	0.384216	0.564297	0.083627	0	0.159306	1.33948	1.91623	0.541738	0	0	0.36135	1.49761
ENSG0000	ZNF16	8:1449303	4.40617	4.78689	3.54773	4.65441	3.46888	0	3.50913	2.10309	6.2926	4.66447	0.6244	-0.34523
ENSG0000	RAB11FIP1	8:3785861	9.44304	6.2766	7.48537	8.98226	12.8847	12.9539	9.27606	15.4929	13.8451	18.7785	0.10045	0.590596
ENSG0000	ZNF211	19:576303	24.7546	21.7852	16.2894	15.7635	8.7375	13.73	8.20709	19.1832	12.9869	17.2624	0.6228	-0.20041
ENSG0000	ACP5	19:115593	3.46573	3.51109	22.0907	18.3813	13.3454	22.6027	13.8991	42.3414	48.7747	23.6312	0.0373	1.3041
ENSG0000	RP11-381	5:1378143	1.83285	0.39213	0.390885	0.521067	0.668993	1.01374	0.334693	1.30523	0	0.564477	0.94565	-0.08687
ENSG0000	SCRIB	8:1437909	3.63475	3.08431	1.60018	1.98828	4.53049	3.22079	4.02913	2.92192	0	7.18652	0.58715	0.457382
ENSG0000	CTD-3214	19:762973	1.25731	3.01538	0.102592	0.84495	0.055143	0	0.612105	0.288986	0	0.558309	0.37595	-1.68555
ENSG0000	WRAP53	17:766177	7.51409	11.3029	8.69993	7.54514	6.8911	8.69013	15.5655	4.01359	10.5447	5.10505	0.91105	0.09625

ENSG000C	FSTL3	19:676364	1.3709	1.04786	2.07173	1.74581	1.3308	2.59622	1.49485	0.227892	2.23255	2.34147	0.80845	-0.19294
ENSG000C	ZNF75D	X:1352489	6.44082	7.17392	3.37262	3.39824	2.77131	2.90142	3.63807	3.64844	0.760088	4.08651	0.55655	-0.32281
ENSG000C	TMEM109	11:609131	108.671	79.0524	150.801	132.025	156.004	64.5626	97.2461	98.8199	97.601	123.359	0.45895	-0.3016
ENSG000C	AC002075	7:9359157	0.37176	0.240542	3.11423	0.729333	1.46187	0	0.567431	2.32692	0.372755	0	0.68765	-0.84838
ENSG000C	PAXBP1	21:327281	29.4149	28.6431	6.73644	5.07965	7.60972	24.4218	15.4651	10.8262	11.6139	15.8593	0.96035	-0.02454
ENSG000C	MAPKAPK	12:111839	21.0144	19.0203	24.9178	25.7639	16.6587	22.424	22.6557	26.3829	18.8736	19.3815	0.90545	-0.06685
ENSG000C	CTA-29F1	22:467618	79.9299	26.8884	41.4171	57.9641	17.4884	47.9635	20.3304	24.9123	18.7836	14.5508	0.06025	-0.84623
ENSG000C	WDCP	2:2401008	3.57807	1.73149	2.57113	2.28414	2.3962	1.96535	1.7669	1.77843	5.97945	5.71751	0.5311	0.399599
ENSG000C	GPD2	2:1564352	1.62353	2.72954	0.816005	0.87322	1.07177	0.502795	2.47512	2.36241	0	1.88911	0.94245	-0.04426
ENSG000C	TOE1	1:4532916	8.47358	8.05321	12.265	11.1656	8.59216	4.26684	4.61414	12.3614	3.63411	12.245	0.6059	-0.44336
ENSG000C	C1D	2:6804112	57.0078	42.7461	39.72	52.9163	17.0908	41.5744	51.6987	43.8817	32.7595	5.97478	0.6799	-0.18375
ENSG000C	ZNF665	19:531592	2.58582	3.24822	1.39478	2.31268	0.574712	2.51501	0	1.14357	0	0.349397	0.24565	-1.60513
ENSG000C	SNPH	20:122605	0.833517	1.7519	0	0.212052	0.47547	0.837261	0.303024	0.009843	0	0.700943	0.73525	-0.87379
ENSG000C	RP11-46C	16:892152	2.1376	1.58636	2.3891	0.442009	1.28416	2.74203	1.35154	3.68534	0	1.59006	0.7642	0.235043
ENSG000C	CLINT1	5:1577857	73.3372	49.3506	40.3587	58.2972	48.2152	21.7698	25.8857	46.5342	34.473	51.1733	0.0233	-0.76234
ENSG000C	ESCO2	8:2773331	2.72423	5.44437	0.361072	0.80767	0.297906	6.43562	7.4585	0.376846	0.416066	1.66423	0.8355	0.239688
ENSG000C	ZMIZ1	10:790690	3.00479	4.25551	1.06608	0.975541	1.75711	5.40671	7.18885	5.37147	0.189537	10.1223	0.01065	1.40934
ENSG000C	FAM104A	17:731649	50.5387	38.619	48.5822	46.2281	32.7592	52.4405	46.2148	44.5007	26.7963	36.1865	0.67875	-0.17844
ENSG000C	RP11-690	2:2317324	1.51662	1.98322	0.400164	1.31637	0.272606	0.641924	1.21333	0	0	0	0.35795	-1.65745
ENSG000C	WDR90	16:649310	1.25086	5.0229	0.55816	0	0.609303	5.31265	2.3782	1.36714	0	1.66608	0.27665	0.862941
ENSG000C	PITRM1	10:306633	20.3454	31.0113	17.615	18.9436	9.03096	25.9728	18.5852	11.9297	25.2629	14.1041	0.8572	0.092467
ENSG000C	CTB-193M	16:156432	5.83773	5.88406	5.20797	4.0539	3.12325	6.54045	4.84718	2.53753	1.11856	6.14944	0.8957	-0.2201
ENSG000C	RP11-192	17:284557	0.476072	0.982725	0.208637	0	0.106686	0.278527	1.53509	0.864735	0	0.553481	0.5262	0.945422
ENSG000C	OAT	10:124397	40.3798	27.9399	30.2714	37.7212	24.5575	46.6657	26.6873	19.3273	22.4021	14.065	0.38345	-0.29352
ENSG000C	CTB-13F3	7:1078909	0.162769	0	2.73333	1.89927	0.250172	0	0.373984	0.20264	0	0.228637	0.18305	-2.74717
ENSG000C	BRWD1-A	21:391841	0.966049	0.52714	0.295515	0	0.520767	0	0	0	0	0.071802	0.25635	-4.8238
ENSG000C	SMARCD2	17:638320	77.6518	75.8305	27.149	14.6178	37.9931	62.9179	80.1896	27.0747	16.3398	36.6541	0.73365	-0.12077
ENSG000C	NHLRC1	6:1812141	0.573186	0	0.570344	0.843152	0.781845	0.72079	1.64864	0	0	0.729024	0.9322	-0.14453
ENSG000C	DAAM1	14:591886	4.18516	5.05568	3.67737	6.54864	7.86191	4.00357	6.76086	4.2907	9.12636	9.5717	0.651	0.343848
ENSG000C	ARPC1A	7:9932589	61.6741	63.9413	41.3079	36.5026	42.8249	93.8625	55.3414	37.3005	23.9858	32.084	0.94345	-0.08252
ENSG000C	PDIA3P1	1:1471727	1.65081	0.734685	1.09389	0.68807	0.560757	2.26463	0.790579	1.08273	0.19666	1.9495	0.702	0.552079
ENSG000C	ZNF331	19:535209	15.5596	18.0247	20.4132	41.5559	13.2584	7.05987	18.1765	15.7589	49.0572	20.7075	0.8735	-0.06638
ENSG000C	GPR146	7:996985-	3.88684	5.72597	0.85021	3.75241	1.41846	0	1.86362	0.396753	7.91913	1.11823	0.84215	-0.26274
ENSG000C	UTP15	5:7355233	6.92535	4.27177	3.91779	3.84523	2.25728	6.15215	5.47482	5.03621	3.98401	5.39747	0.8139	0.158745
ENSG000C	GPHN	14:662128	5.25833	4.3266	1.3328	2.7066	1.46546	5.03621	3.27853	0.740913	1.63651	2.22274	0.56365	-0.38801
ENSG000C	ZNF692	1:2488500	46.2364	72.5396	25.4239	21.2205	17.109	56.9946	36.4034	16.2602	24.5661	23.144	0.5752	-0.19445
ENSG000C	ATP11B	3:1827934	22.7743	17.6076	5.5306	9.20778	7.51238	14.6652	11.4028	15.4008	13.7641	19.4465	0.71385	0.190998
ENSG000C	LINC0089	X:1366395	10.4076	5.50615	10.896	7.69257	4.60247	109.286	65.1351	92.0943	57.5159	24.1409	0.00065	3.16128
ENSG000C	TMEM55A	8:9099379	6.5353	6.51892	12.1262	14.6175	12.8126	10.0828	3.03396	6.13496	4.25544	5.7505	0.0112	-1.15658
ENSG000C	FAF2	5:1764476	12.1241	12.636	6.4454	6.72552	10.019	13.5405	7.47635	12.6312	15.1767	17.4404	0.2657	0.386245
ENSG000C	IRF5	7:1289376	15.7345	19.8233	4.51632	9.18171	2.83724	46.1823	31.0925	4.87154	2.7577	7.56139	0.13005	0.636516
ENSG000C	SYNE1	6:1516566	58.7599	56.0909	19.7765	22.408	22.3148	33.7282	23.0223	23.3868	27.4543	38.9693	0.4964	-0.28429
ENSG000C	GOLGA7	8:4149039	113.912	76.821	98.9115	105.658	73.9747	71.182	93.6432	95.5145	110.218	44.5606	0.3964	-0.28388
ENSG000C	IGSF9B	11:133908	1.79538	2.17393	0.745045	0.656352	0.714398	0.10635	0.235123	3.01245	0	0.310261	0.6571	-0.54582
ENSG000C	ZC3H3	8:1434376	12.383	19.7971	6.66316	3.29088	11.671	9.42373	4.03765	11.1249	9.29177	17.0251	0.83295	0.09147
ENSG000C	THEMIS2	1:2787254	91.8728	114.298	133.238	66.5369	198.702	86.1947	27.5261	56.135	14.8474	91.1355	0.00195	-1.14316
ENSG000C	TSC22D1	13:444321	5.83238	2.05092	5.31525	4.20708	4.54301	1.95447	3.41913	5.15956	0.971525	6.21538	0.68975	-0.23955
ENSG000C	SNHG1	11:628519	27.3792	21.9162	21.2222	28.3617	14.4128	11.9699	34.712	26.6241	13.0263	25.8032	0.7788	0.132411
ENSG000C	ATP6V0D1	16:674380	85.3869	109.411	85.4098	88.1924	86.1307	137.479	100.124	83.4473	77.3754	68.3254	0.91815	-0.03154
ENSG000C	AC009505	2:1057448	0.751921	2.31032	0.139351	0.389812	0.178888	0.815534	1.01118	0	0.167396	0.366905	0.59605	-0.58896
ENSG000C	DESI2	1:2446529	9.20482	7.45853	5.98123	4.4296	8.63085	7.95221	8.15611	8.62386	2.30431	11.2845	0.9192	-0.06334
ENSG000C	CTC-260E	19:200779	0.51399	0.019595	0	0	0.063778	2.2465	0.574387	0	0	0.947645	0.31315	2.84605
ENSG000C	MYO1E	15:591050	1.35943	2.089	0	0	0.008121	7.64487	17.6993	0.544515	2.73074	1.48556	0.0533	2.84526
ENSG000C	AC011380	5:1405582	0.261324	0.530209	0	0	0	2.51827	0.067919	0	0	0.491548	0.35885	2.13258
ENSG000C	ZNF747	16:305240	3.35648	4.18805	1.45323	1.08905	2.82558	4.33019	3.32783	3.83624	1.49702	4.19143	0.68735	0.411017
ENSG000C	DARS	2:1359066	78.2873	55.3412	77.437	76.9416	80.1846	98.7291	58.7175	66.8186	88.7721	65.6396	0.98465	0.006024
ENSG000C	CTC-529I	17:159765	0.57124	1.10385	0	0	0.622193	0	0.636464	0.225807	0	0.971751	0.87895	0.27277
ENSG000C	RFX1	19:139615	2.59863	1.26578	1.47592	0.674547	1.13424	0.521499	1.76932	1.51508	0.607715	1.50534	0.4545	-0.64003
ENSG000C	CSE1L	20:490462	31.2733	31.0149	8.09852	10.0242	9.21969	12.0191	12.4472	8.72195	10.3914	16.3543	0.17625	-0.48656
ENSG000C	SLC52A2	8:1443522	43.8223	37.1506	34.9428	45.5619	29.3668	61.7021	49.9301	49.6184	26.0283	38.3415	0.6978	0.172151
ENSG000C	TRIM68	11:459867	7.42156	8.97763	9.97088	5.0783	5.94024	7.26458	7.21141	5.86875	4.62171	11.276	0.9372	-0.0325
ENSG000C	H3F3BP1	13:714379	0.317473	0	0.062907	0.165208	0	0.27998	1.56375	0.268145	0	0	0.3819	2.17344
ENSG000C	CTNND1	11:577125	0.656343	2.46827	0.269253	1.20644	0.22498	1.6241	0.368907	1.39991	0	0.16022	0.7035	-0.40535
ENSG000C	DBH-AS1	9:1336363	0.023647	2.37331	0.768633	0	0.024553	5.07398	3.01133	4.80027	1.55551	5.24847	0.10665	2.73386
ENSG000C	ZNF107	7:6466608	4.14779	4.01194	1.73383	1.82408	1.84993	2.56209	2.22604	1.45482	3.18109	5.50318	0.90165	0.069389
ENSG000C	PLEKHA3F	19:415210	0.721311	0	0.23766	0	0.256655	0	2.66661	0.337741	0	0.063625	0.3603	1.34382
ENSG000C	CLYBL	13:996066	8.99584	13.3076	12.8514	8.81069	10.8102	5.85397	7.08669	7.51769	9.87684	3.74097	0.3344	-0.61731
ENSG000C	NNT-AS1	5:4357159	5.73953	5.30492	3.68515	2.77097	5.84718	12.163	4.56153	2.59141	1.27952	4.93107	0.7821	-0.27886
ENSG000C	DDX10	11:108665	16.2007	11.7935	9.98224	11.6578	10.3044	9.61665	10.8732	8.56277	7.27813	13.3291	0.5563	-0.22876
ENSG000C	MT1H	16:566698	0	0	0	0	0	4.88495	0	0.638959	0	0	0.05625	inf
ENSG000C	LRRC34	3:1697934	1.17266	0	0.161193	0.374192	0.339213	4.40324	4.16189	0.046073	4.60878	0.874783	0.00705	2.99347
ENSG000C	ANAPC5	12:121308	141.164	154.507	114.518	140.201	91.5117	83.7506	120.059	94.7984	109.961	104.841	0.36745	-0.28114
ENSG000C	MIEF2	17:182605	5.49564	3.38044	7.32001	7.65767	5.44463	3.42218	2.0275	3.81501	6.55622	9.2313	0.6582	-0.22185
ENSG000C	GIMAP1-(	7:1507165	1.69485	1.84263	9.32707	1.01888	2.34222	3.25219	3.86804	42.5983	1.09672	16.729	0.73805	-0.65911
ENSG000C	ANXA5	4:1216679	144.609	130.795	80.1521	78.5495	77.6034	363.066	222.951	192.462	151.074	95.2862	0.003	0.979476
ENSG000C	OTUD5	X:4892202	56.1289	74.7193	26.7369	35.6798	38.6085	51.9598	65.5335	47.4934	32.2112	42.2754	0.9988	0.000494
ENSG000C	CD99	X:2691178	451.606	448.116	625.283	503.239	709.405	559.43	947.575	808.938	654.52	336.461	0.5674	0.217469
ENSG000C	RAPGEF2	4:1591041	1.23154	1.8893	1.31407	2.49879	1.31895	1.04379	0.942308	2.70252	0.396317	4.13991	0.7575	0.163943
ENSG000C	CTA-292E	22:288006	4.6961	1.63579	10.4435	16.3307	8.26596	2.5604	7.57022	8.14975	6.70034	6.78417	0.7496	-0.40134
ENSG000C	CIPC	14:770980	4.10397	2.81198	1.66517	2.79448	1.53007	2.72752	2.1216	1.83809	3.65996	3.31453	0.94765	-0.04103
ENSG000C	REV1	2:9899726	11.1459	15.0846	7.70979	10.5681	7.27438	7.88409	10.6339	7.59014	4.90213	16.713	0.6005	-0.30872
ENSG000C	ZNF700	19:119250	5.41061	3.17676	3.38218	5.38224	2.89461	15.4329	8.31609	5.34027	8.57297	9.91385	0.0408	1.13841
ENSG000C	MAGEE1	X:7642772	1.22127	0.505872	0.502348	1.42021	0.571049	3.35967	0.956404	0.806963	2.39342	0.325518	0.282	0.919946
ENSG000C	GNA13	17:650107	26.9319	24.1057	9.86649	7.5851	11.3938	16.1311	21.3859	15.4534	15.2422	26.7727	0.44235	0.257746
ENSG000C	CD52	1:2627917	4155.14	2871.8	4901.32	3971.09	1813.25	4951.12	4474.09	3523.61	3498.88	552.386	0.9672	-0.08834
ENSG000C	G0S2	1:2096613	13.4947	2.11872	0.337208	15.1391	2.62733	8.22318	0.112008	41.2404	44.8287	12.3351	0.0906	1.77504
ENSG000C	CMTR1	6:3743321	11.4125	12.8832	2.83606	2.74963	1.92714	7.74693	12.2766	3.49017	3.50197	4.28444	0.9454	-0.02787
ENSG000C	MRPL43	10:100969	74.9733	66.3454	106.064	91.5069	117.763	90.4674	79.3296	99.3953	82.0434	45.3794	0.5122	-0.25889
ENSG000C	DMTF1	7:8715142	42.1114	60.1016	19.3106	24.7815	11.1348	24.9466	30.328	28.1126	53.6374	40.2974	0.7424	0.146138
ENSG000C	BEST1	11:619498	2.44987	3.19409	2.18666	6.81247	0.950692	1.32441	0.502303	1.48279	3.39381	4.868	0.6321	-0.65658
ENSG000C	PLPP5	8:3822521	34.0521	27.0819	40.7792	65.4237	33.0794	76.8118	37.7321	39.8062	54.3102	44.3515	0.7274	0.261759

ENSG0000C	PSMG4	6:3224260	23.6432	21.4455	32.7241	24.4068	20.1379	15.1655	35.868	21.0158	21.091	9.17909	0.52535	-0.39608
ENSG0000C	PAM16	16:431476	23.9476	17.6662	29.1994	28.9701	12.4317	21.9117	38.7471	15.7725	21.3998	8.34826	0.8908	-0.11229
ENSG0000C	RP11-147	17:681257	4.75187	8.34353	2.84429	1.66074	5.71006	7.88026	7.852	5.86253	9.43613	14.3121	0.0731	0.979484
ENSG0000C	PSENEN	19:357180	36.1075	25.3476	67.5579	77.0213	42.912	53.1536	53.4659	79.1502	57.5855	19.7435	0.98705	0.02111
ENSG0000C	RGL1	1:1836360	0.03463	0.066582	0.069452	0	0	6.80232	0	0.312899	0.083075	0.009932	0.3057	5.49411
ENSG0000C	RP11-90P	8:3810524	7.26583	4.69849	7.18506	8.66994	5.06507	8.37894	12.2442	6.08698	5.5135	0	0.9526	-0.07884
ENSG0000C	TMED8	14:773350	1.17633	1.51494	0.374818	0.194614	0.634424	1.46608	0.726992	1.09023	2.2289	1.81504	0.0963	0.91352
ENSG0000C	EIF4G1	3:1843144	33.4329	33.4976	17.3817	12.1595	33.7405	24.4521	30.3731	21.5763	6.21305	42.0875	0.73415	0.158905
ENSG0000C	EEF1DP7	17:636224	0.547162	3.01945	0.258575	0	0	0	3.16103	0.093147	0	0.438555	0.9695	-0.07719
ENSG0000C	ASPM	1:1970841	1.62188	6.79056	0.061446	0.086272	0.096342	2.68986	3.81317	1.15261	1.8645	2.44636	0.41505	0.670071
ENSG0000C	UTY	Y:1324837	12.9879	25.3659	15.5518	30.0774	8.36187	15.1992	14.0605	17.2055	32.7478	20.7686	0.8331	0.190949
ENSG0000C	LINC0033	1:2202398	1.82229	22.9896	1.49727	2.82542	9.17707	0.067259	27.9383	2.43932	5.45591	4.61624	0.8924	0.122317
ENSG0000C	HUS1B	6:485132-	1.424	0.502996	2.46704	1.16095	0.848799	0	0.606194	0.569789	0	0.070558	0.1691	-2.60414
ENSG0000C	DNAJC14	12:557432	14.3674	17.0744	10.3061	7.15296	6.27784	12.3436	2.86567	9.81082	14.5117	11.6089	0.8894	-0.11213
ENSG0000C	TRIM73	7:7534401	1.04606	1.23073	0.686386	0.634586	0.561017	0.819276	0.541601	1.60337	0.019354	0.523725	0.94365	0.075309
ENSG0000C	AC002310	16:305693	0	1.31649	0	0	0	0.708493	1.43681	1.21712	2.6907	1.42598	0.3125	2.04265
ENSG0000C	GOLM1	9:8602614	1.07915	4.47146	0.855895	2.02855	0.84697	3.49756	1.22363	0.445297	0	1.46384	0.60855	-0.51651
ENSG0000C	GINS2	16:851695	5.11044	8.05248	0.894515	2.19286	1.24017	4.71003	7.70582	5.98107	0	2.91025	0.7737	0.345059
ENSG0000C	ZNF800	7:1273467	26.5611	24.6895	29.219	20.2115	39.5416	25.5228	31.6592	26.2591	28.791	60.7518	0.9843	-0.00715
ENSG0000C	ZNF253	19:198658	2.61834	10.109	2.87725	2.35956	3.34708	4.6075	3.60272	1.28317	5.49115	6.93006	0.9104	-0.07001
ENSG0000C	DARS2	1:1737995	7.95622	7.80755	4.2327	4.25535	2.26813	4.06741	6.3953	6.75351	4.62536	4.00511	0.9399	-0.04454
ENSG0000C	RP11-462	10:329003	0	0	0	0	0	0	2.15207	0	0	0.102629	0.1222	inf
ENSG0000C	SMG6-IT1	17:205415	0	0.244052	0	0	0	1.95817	0.984855	0	0	0	0.365	3.61732
ENSG0000C	ULK4	3:4119483	9.12228	5.71348	6.6602	2.66754	2.74358	7.37559	5.6453	9.04059	10.4362	6.38216	0.40385	0.648975
ENSG0000C	PHF2	9:9357640	2.41166	4.94956	3.13883	1.88692	3.93505	4.31031	3.22804	3.11846	5.29537	6.8046	0.3871	0.518607
ENSG0000C	RPS28P7	11:818798	0.423933	0.811676	1.79975	1.27863	0	1.15521	1.33535	0	0.443258	0	0.7205	-0.7162
ENSG0000C	LINC0068	X:320989-	1.29559	1.13189	0.115476	0.88331	0.227374	2.88357	3.70532	0.121836	0	0.430349	0.5842	0.922282
ENSG0000C	MVK	12:109553	1.61627	0.940981	6.2876	7.21674	7.95506	2.85838	1.62428	5.63604	8.86529	3.54974	0.93875	-0.06186
ENSG0000C	SETD4	21:360345	10.9007	12.0531	10.4787	6.33787	6.13572	5.10596	11.4547	8.68533	0	5.63685	0.38115	-0.67006
ENSG0000C	ZFY-AS1	Y:2935280	0	0	1.6947	0.269095	0.214523	1.44564	1.59053	0.58152	0	0.230695	0.772	0.513164
ENSG0000C	MORF4L1I	1:2201482	2.10538	2.1521	0.660943	0.536048	0.40562	0.946942	1.76931	0.282665	0.334781	0.447184	0.6735	-0.53759
ENSG0000C	CTD-2186	5:3217447	4.12231	1.61812	3.38452	2.91025	1.42133	8.10048	1.41869	0.659183	2.9772	0.707847	0.81735	-0.03946
ENSG0000C	CCDC53	12:102012	52.2599	47.8488	58.8232	68.3905	43.4188	90.1903	65.7312	49.9075	90.1848	18.0125	0.4606	0.257415
ENSG0000C	TMPRSS3	21:423718	3.9709	2.80935	3.74982	2.45515	1.18028	12.5873	15.2922	9.66097	1.25825	4.4468	0.0395	1.58385
ENSG0000C	PRR29-AS	17:639960	4.80108	0	1.20439	3.85112	4.51163	0.548578	0	1.6298	0	0.528582	0.11355	-2.49616
ENSG0000C	ATP5H	17:750325	141.812	134.922	198.532	234.198	99.791	296.672	199.372	227.319	194.94	37.1908	0.5582	0.241686
ENSG0000C	TUBA1A	12:491847	140.379	157.876	120.841	198.944	77.6254	92.1095	99.8282	126.99	155.569	74.1303	0.27945	-0.38787
ENSG0000C	PRKAG2-/	7:1515561	2.44473	1.39102	2.59543	3.35432	3.39889	1.47386	0.500896	2.31288	2.46259	1.22605	0.6479	-0.79504
ENSG0000C	RP11-490	1:3125956	0.255307	0.242832	2.28828	0.488053	0.978043	0.332475	0.193566	1.65877	0	1.52697	0.9681	-0.0695
ENSG0000C	TCP11L2	12:106237	10.2375	14.9088	7.15532	7.9204	11.9847	0.048456	2.46228	5.06461	7.79504	3.56543	0.1022	-1.2847
ENSG0000C	GIGYF1	7:1006795	1.4956	1.47099	7.29976	1.45633	5.23577	1.60741	1.45497	7.35229	3.46712	11.683	0.28795	0.474039
ENSG0000C	AKAP13	15:853805	93.173	93.5358	92.9946	86.0537	108.656	103.282	124.475	111.224	102.061	201.583	0.2732	0.47482
ENSG0000C	AC241585	1:1451640	8.2106	10.0534	13.4293	6.13577	9.44123	2.2661	6.01885	14.9697	4.82521	4.48035	0.73445	-0.53063
ENSG0000C	RP11-259	17:462595	0.325132	0.525929	0.182986	0.643416	0.361002	0.308475	1.06328	0.148225	2.25422	0.812846	0.1473	3.29617
ENSG0000C	PPM1D	17:606001	7.61707	10.8724	6.41487	4.51956	4.80447	5.54641	7.67618	5.20577	2.221	6.32939	0.28265	-0.61655
ENSG0000C	IFITM1	11:307630	811.465	962.532	2209.25	2614.98	1871.2	653.387	1836.69	2541.71	2086.4	601.663	0.84155	-0.15589
ENSG0000C	TMCC1	3:1296477	2.4138	2.10836	1.83522	1.5962	1.92574	4.39176	1.83113	1.82991	3.39498	2.239	0.64195	0.346321
ENSG0000C	AC110615	4:7591365	9.90239	8.95841	3.08351	2.69771	1.86696	5.95286	4.64746	1.15601	1.72497	3.40489	0.8705	-0.2256
ENSG0000C	KTN1	14:554992	52.5718	44.7704	27.3274	30.7356	39.8874	60.6537	64.5939	36.6721	44.4231	51.1765	0.5887	0.350196
ENSG0000C	AF213884	4:1025008	5.95428	2.01687	4.80346	6.31377	0.845589	0	1.79663	1.92739	1.75015	0.339244	0.49575	-1.78631
ENSG0000C	RP11-504	1:2239927	2.34624	3.9579	6.1497	7.84525	1.63763	0.082386	5.39109	4.42007	4.03257	4.32083	0.6019	-0.42441
ENSG0000C	SMURF1	7:9887793	2.13322	2.68173	0.821376	0.419505	0.910634	2.1682	3.03988	1.5806	0.261821	3.02745	0.71235	0.352054
ENSG0000C	HCG4P3	6:3000101	2.74072	1.94678	0.833298	1.10086	1.35667	0.441439	1.51078	0.873907	0	2.38462	0.687	-0.63095
ENSG0000C	NRF1	7:1296117	9.17088	8.418	7.19939	8.43377	5.49798	3.17797	7.08613	8.28263	8.63822	6.4899	0.538	-0.24336
ENSG0000C	ERVK3-1	19:582285	66.046	53.9934	67.5911	59.8926	43.6676	57.0115	79.8575	79.8958	54.9657	36.3108	0.82405	0.094972
ENSG0000C	PPP1R15B	1:2043778	13.7898	12.7498	5.56124	7.42014	6.22597	5.97256	9.12573	11.4758	2.90274	14.7854	0.7286	-0.14105
ENSG0000C	MCL1	1:1505485	132.511	157.984	58.2909	102	50.0248	96.535	135.86	137.926	110.282	115.046	0.40585	0.268065
ENSG0000C	ERGIC3	20:355420	55.1365	46.4528	76.5672	66.5167	58.0204	78.7676	58.9157	74.2942	102.256	25.7018	0.57785	0.17059
ENSG0000C	RNASEH2I	13:508621	1.17436	0.980585	0.387961	0.869202	0.130999	2.59201	0.857241	0	0	0.116312	0.97755	0.0298
ENSG0000C	SET	9:1286834	78.8214	53.4835	168.237	95.9484	136.543	45.4307	68.6451	149.501	98.0006	126.459	0.53205	-0.23337
ENSG0000C	KCTD12	13:768801	0.177463	0.880398	0.143668	0.09206	0.088148	3.63092	0.063689	0.510342	0.265766	0.114693	0.3324	1.68563
ENSG0000C	GNL3	3:5249533	90.1111	74.654	96.2061	88.0327	67.4553	83.0016	87.8851	45.3754	49.8924	40.7051	0.47435	-0.45968
ENSG0000C	GVINP1	11:671353	9.97007	12.8883	2.54386	0.830199	4.95784	9.36368	7.1187	4.3855	1.24228	11.2746	0.9312	-0.03478
ENSG0000C	RNASEH1	2:3531812	19.5691	22.989	23.992	23.9817	29.2112	28.314	23.2055	16.2071	23.2226	24.5348	0.7757	-0.11861
ENSG0000C	NAAA	4:7591365	41.6182	79.1199	76.4968	69.058	34.0198	32.673	50.4974	61.9056	49.1713	29.6245	0.36865	-0.45732
ENSG0000C	CHKA	11:680390	2.89487	7.43908	3.35151	3.19933	2.86438	3.66818	5.94643	4.15619	5.03145	2.73591	0.86505	0.175407
ENSG0000C	TEN1	17:759415	12.921	9.45269	21.9702	20.9732	12.5247	12.7843	15.3258	15.284	3.13007	5.88791	0.42775	-0.66934
ENSG0000C	PHF20	20:357771	55.5771	60.4006	83.3747	74.5716	90.07	67.6535	50.167	46.7465	53.0327	97.8606	0.47665	-0.38353
ENSG0000C	PBX2	6:3218473	1.09538	0.856554	0.304127	0.371485	0.715833	2.02088	1.11842	1.02776	0	1.8933	0.62215	0.781174
ENSG0000C	SPATA24	5:1393965	3.42782	6.64176	2.44627	3.24456	0.893913	3.27158	6.24336	3.05534	5.71091	1.25032	0.67155	0.411357
ENSG0000C	ZNF233	19:442075	0.402034	2.7421	0.07957	0.163758	0	1.08517	0.701057	0.297177	0	0.359716	0.7716	-0.39511
ENSG0000C	TNIK	3:1710613	15.9052	15.7247	17.7246	24.4926	39.5713	9.46539	10.338	23.8756	32.0863	44.995	0.94635	-0.05856
ENSG0000C	ANKRD28	3:1566723	9.29173	12.3752	1.3729	6.29331	2.77042	12.0746	19.7699	11.6199	19.7241	7.97504	0.006	1.2611
ENSG0000C	RNFT1	17:598930	44.5877	29.6118	26.4976	30.7612	15.1123	33.297	18.0595	15.9669	25.814	21.2068	0.42835	-0.36589
ENSG0000C	APP	21:258805	1.65987	3.34781	0.99753	4.12898	0.553416	2.00411	1.05936	0.013158	4.78034	1.41285	0.64265	-0.35959
ENSG0000C	CTD-2589	5:612271-	0.44829	0.300361	0.364422	0.184663	0	0	1.95947	0	0	1.25291	0.57485	1.16418
ENSG0000C	LCAT	16:679397	0.894219	2.42623	0.294258	0.364535	0.613296	2.05799	2.21218	0.125307	0.461031	3.40188	0.3718	0.809071
ENSG0000C	RP11-367	6:2479732	6.3479	7.04695	8.86245	15.2295	3.35578	4.64023	9.29956	7.73742	7.7917	2.18431	0.8178	-0.35953
ENSG0000C	TNFRSF10	8:2313558	1.20966	5.18977	0.274882	0.35988	0.020122	0.746822	0.082815	0.301984	0	0.570753	0.2557	-2.15444
ENSG0000C	UQCRFS1	19:292053	48.9865	41.2468	56.0827	66.443	48.9297	58.1346	49.3274	42.023	33.4137	13.16	0.19205	-0.42095
ENSG0000C	SAMD4B	19:393423	7.85717	8.16805	4.38067	4.42659	5.19856	3.1558	7.51665	6.51994	4.8721	14.8121	0.73055	0.196228
ENSG0000C	SPRY2	13:803359	0.417622	0	0.872199	0.619631	0.742123	4.88811	4.04469	3.86948	0.625826	5.8906	0.1178	2.70895
ENSG0000C	ZNF131	5:4301441	39.9139	38.8362	38.2058	33.8836	36.9507	39.4839	32.8869	43.4438	32.8859	42.9416	0.75985	-0.13083
ENSG0000C	PPP1R35	7:1004352	153.584	155.499	113.642	62.0639	101.267	268.411	218.845	95.4315	56.0891	56.5709	0.802	0.101203
ENSG0000C	PNMA3	X:1530564	0.153734	0.681912	0.049228	0.478223	0.131584	2.09879	0.053525	0.344182	0.02911	0.818744	0.58255	1.11645
ENSG0000C	CCDC96	4:6909241	1.39866	0.48905	1.71113	0.354549	1.00131	0	0.324515	0.904749	0	1.29731	0.54785	-0.93554
ENSG0000C	PMS2P4	7:6729560	3.92099	6.26102	4.72418	2.95346	2.6682	3.12163	2.42691	1.31704	5.82783	4.08371	0.961	-0.07294
ENSG0000C	LRRC8C	1:8952483	6.40554	11.3097	1.49215	2.55401	3.35594	5.73483	3.11266	3.56465	3.64646	6.2155	0.64835	-0.25402
ENSG0000C	NTPCR	1:2329506	21.5428	12.707	18.5445	18.9383	12.6499	16.1764	16.3716	17.4126	22.8354	6.30049	0.85045	-0.14965
ENSG0000C	CAD	2:2721738	7.72654	7.13214	2.33738	3.15522	3.80776	4.80363	3.266	4.31681	5.87653	7.00217	0.63465	0.200929
ENSG0000C	FAM49B	8:1298323	220.04	180.885	172.534	162.203	114.475	246.921	212.759	134.706	179.605	71.5603	0.8568	0.054241

ENSG000C	TMEM263	12:106955	23.5865	23.5358	11.4657	12.5198	10.2442	26.1452	20.3727	16.5588	6.93445	8.60526	0.83555	-0.08314
ENSG000C	CHI3L2	1:1111871	8.88155	38.868	14.5923	15.3471	5.64604	17.8287	4.06006	12.4755	19.7156	12.4377	0.8139	-0.25908
ENSG000C	DYNLL1	12:120469	224.983	166.41	211.8	183.553	139.202	471.627	398.998	311.871	201.493	56.6382	0.12035	0.558452
ENSG000C	VAMP3	1:7771268	25.9941	22.2821	18.7115	14.9159	18.7263	17.2177	20.4115	12.569	14.3422	20.7937	0.45845	-0.27591
ENSG000C	EIF2S2P4	2:1707230	0.959182	0.831196	0.171489	0.457044	0.418576	1.7741	1.96825	0.389454	0.374459	0.130663	0.69655	0.817618
ENSG000C	AC007383	2:2059895	11.7295	8.52131	11.3161	8.10001	6.31282	17.476	11.8051	6.66175	14.2007	7.38527	0.823	0.252468
ENSG000C	NEMP2	2:1904083	4.47652	10.4	2.62802	4.31907	1.69765	2.08591	3.38093	5.46731	0.006589	3.03326	0.3525	-0.80104
ENSG000C	MLH1	3:3699333	19.6397	19.0036	16.7983	19.3801	10.5628	41.6926	25.8415	13.3956	25.9992	19.6542	0.0895	0.582904
ENSG000C	HIPK2	7:1395615	1.56327	2.70859	0.837733	0.72162	2.76832	2.46924	0.70055	1.02084	0.74293	4.91443	0.8382	0.115847
ENSG000C	TUBA1C	12:491847	50.3517	85.4946	43.7121	68.3423	33.7405	81.6973	93.4257	60.9562	62.6121	28.2232	0.6305	0.388968
ENSG000C	PTPRN2	7:1575390	0.614452	2.1189	0.799153	1.56057	0.17814	2.11603	2.28814	2.3189	1.50196	1.44129	0.2458	0.824397
ENSG000C	SLC25A13	7:9612021	6.13777	4.52505	1.8964	2.36575	2.49405	2.60242	5.3893	1.77366	3.26545	5.02667	0.9257	0.081548
ENSG000C	INO80C	18:354438	17.8777	18.1823	20.6687	18.068	17.3246	15.3049	33.8411	22.6446	25.6526	10.8182	0.6861	0.19241
ENSG000C	FAM120B	6:1702822	15.7646	15.8693	8.18438	6.55817	5.60571	11.1628	6.89772	7.55279	2.59571	9.96198	0.10205	-0.67906
ENSG000C	SMIM13	6:1109403	4.012	4.27715	2.93524	3.3482	3.01068	5.14821	6.3513	2.08821	1.96384	1.49682	0.77725	-0.22435
ENSG000C	HERC2	15:281110	9.91439	11.2481	5.78291	6.41036	3.19119	7.45411	6.68501	5.02882	6.34097	6.86568	0.9409	-0.04551
ENSG000C	CSTF2T	10:509913	6.59669	6.35051	11.77	4.55903	10.0209	7.95728	6.51225	15.2039	7.08158	13.2682	0.5249	0.242092
ENSG000C	RP3-508I1	22:387057	0.57987	1.13548	0.158533	0.100716	0	0.525031	1.40232	0.083717	0	0.20319	0.8932	0.212963
ENSG000C	LMO7	13:755494	3.10513	10.9516	2.05146	3.48663	3.31562	0.777805	0.866585	5.63721	6.50841	11.1518	0.79525	0.21905
ENSG000C	DNAJC15	13:430232	10.544	11.179	12.4719	5.98799	6.32779	14.4333	24.2002	13.4012	6.32277	5.18702	0.8358	0.181471
ENSG000C	DHX30	3:4780290	37.0032	46.0293	29.4875	33.9991	21.6926	34.5359	19.8386	28.9241	16.1332	32.6041	0.20115	-0.41839
ENSG000C	MAML1	5:1797328	5.99766	7.68436	3.12155	0.834821	4.89787	4.36007	7.24824	3.6259	0.672262	11.4136	0.7726	0.138046
ENSG000C	NUCB2	11:172081	98.3653	176.195	116.195	90.2077	135.141	82.2619	104.854	72.8012	58.3386	75.6713	0.0342	-0.68677
ENSG000C	AC130469	19:852078	9.55682	4.27865	10.8585	13.2567	7.81543	10.5313	8.50031	5.81686	8.60912	15.1448	0.97115	0.04629
ENSG000C	CD274	9:5450502	2.78877	3.37307	1.87721	1.48621	0.917096	5.83807	4.81867	7.07665	2.2727	2.95114	0.03875	1.16042
ENSG000C	MRTO4	1:1921050	10.3919	12.761	17.5368	19.4344	18.8385	11.9735	19.5325	19.5407	15.8723	7.63992	0.8653	-0.09377
ENSG000C	CPSF7	11:614026	7.55694	9.35319	3.55012	3.90674	3.78789	4.42513	6.27855	3.58096	3.74166	7.38433	0.7755	-0.21074
ENSG000C	FKBP1B	2:2401008	0.90986	2.15004	0.581859	0	1.24104	4.27154	10.4113	8.31445	0.058799	3.73004	0.0613	2.46007
ENSG000C	ARL3	10:102673	6.25866	2.80414	9.5595	7.93684	8.08603	13.8496	7.87914	11.2502	10.8847	4.52749	0.41295	0.340334
ENSG000C	KATNBL1	15:341406	32.1118	25.6995	20.6124	29.2825	16.9407	51.6286	39.2492	21.6106	33.8463	31.0725	0.24445	0.505655
ENSG000C	NBAS	2:1516690	6.50662	7.75584	2.49894	2.30157	4.17681	6.99122	1.9577	2.4523	2.7534	7.00996	0.8609	0.093054
ENSG000C	TNRC6C	17:779592	6.5222	8.79986	5.34063	2.80547	6.24998	3.69941	5.56727	2.50053	1.04025	11.8088	0.6126	-0.43113
ENSG000C	MCC	5:1130220	0.729512	0.517214	0.158264	0.176013	0.287781	1.05641	0.32021	0.443073	0	0.811355	0.7299	0.260133
ENSG000C	RBM34	1:2351311	89.0789	74.9298	90.0997	85.9931	98.4896	120.279	107.714	79.8992	45.7058	56.2401	0.6861	-0.25029
ENSG000C	RREB1	6:7107596	1.90715	2.5805	1.23975	1.13837	1.57913	2.21408	1.41084	1.49415	0.954945	3.87776	0.6838	0.214131
ENSG000C	PRKACB	1:8407806	52.291	53.2683	21.1503	29.123	17.1869	22.7514	38.9044	33.8028	23.0244	31.7821	0.58535	-0.17272
ENSG000C	TRAPPC1C	21:440123	31.1902	20.1441	20.1665	24.7587	24.5468	22.3293	12.9166	15.9199	14.1594	26.1284	0.2459	-0.52019
ENSG000C	TMF1	3:6897521	23.7173	22.7625	12.2652	11.9646	18.0198	27.6487	27.5119	9.96389	22.4473	27.808	0.6535	0.290107
ENSG000C	DKK2	4:1069218	1.51529	0	1.09705	1.25869	2.59602	0	0	0	0.673714	0.690718	0.1975	-2.18163
ENSG000C	CD5	11:611023	45.9767	165.977	39.6846	33.6394	37.7714	52.6593	54.4522	37.1353	37.5763	78.7997	0.50985	-0.22566
ENSG000C	EPS8L2	11:644232	18.8579	3.78511	16.5001	9.95658	17.2756	19.0408	20.4162	18.3826	16.2619	10.6607	0.68	0.319624
ENSG000C	WDR20	14:102080	16.6636	21.8188	19.4985	19.395	17.3797	7.22619	19.2795	18.65	28.5038	16.9468	0.9231	-0.06563
ENSG000C	ADHFE1	8:6642902	39.5177	42.482	40.2536	39.011	20.4009	18.9289	34.1597	22.5523	22.809	21.367	0.14635	-0.62263
ENSG000C	TSR3	16:133360	92.934	69.9267	98.4439	72.7793	140.414	87.3044	154.724	69.871	70.1996	41.0876	0.3641	-0.32053
ENSG000C	DNAJC21	5:3492959	27.9468	26.5561	21.7431	10.1644	31.9819	44.9097	40.7091	29.84	16.1202	33.6081	0.2464	0.419244
ENSG000C	HNRNPF	10:433856	183.394	170.281	195.106	159.749	172.409	141.281	172.766	161.764	132.191	144.016	0.38425	-0.3078
ENSG000C	UMPS	3:1247303	20.2871	18.8894	29.4781	36.5367	25.4785	7.15066	20.1315	23.8637	12.7521	18.6816	0.0812	-0.69014
ENSG000C	AC000123	7:1273467	0.152915	0.145019	0.168266	0	0.179661	0.323533	0.184382	0.325477	0.584844	1.00636	0.2503	1.71025
ENSG000C	HDDC3	15:909299	33.965	19.5112	58.7326	39.3851	43.7019	45.2583	31.9487	44.1672	27.9229	29.2817	0.84355	-0.14976
ENSG000C	TRIM23	5:6558967	7.51702	11.5803	2.22143	4.61586	1.68665	8.74624	5.76822	4.42957	3.8628	4.29788	0.97115	-0.02568
ENSG000C	MROH8	20:371012	0.724744	1.25574	0.883002	1.88568	0.560383	2.46432	0.249542	1.54986	0	0.022807	0.57125	-0.60362
ENSG000C	TMEM57	1:2543085	3.46782	5.25378	1.5949	1.16901	3.7154	2.48554	2.39597	2.42028	1.15723	4.69226	0.6046	-0.29082
ENSG000C	ADGRA3	4:2234507	3.96455	2.63456	0.655322	0.305505	0.7157	0.332164	0	0.122579	1.58717	0.171387	0.0957	-1.65342
ENSG000C	NCS1	9:1301725	1.09244	0.225322	0.029263	0.111702	0.693057	1.1567	10.9909	0.348343	3.47216	0.522977	0.03925	2.6519
ENSG000C	DYNC1LI1	3:3252597	17.7915	14.1687	10.7847	9.97642	12.618	16.7753	23.2886	19.71	7.87407	10.0447	0.45645	0.296709
ENSG000C	HSD11B1	1:2096613	1.11613	0	0.22018	0	0	10.1992	0	0	0	0.169272	0.2679	2.85391
ENSG000C	RP11-428.	6:5029971	0.450742	0.200068	1.08146	0	0.54	0	0.618633	1.07299	0	0	0.8529	-0.27277
ENSG000C	RPL26L1	5:1729547	30.4983	21.6521	34.9647	31.2225	18.6111	60.7684	28.4324	18.5657	17.9866	5.25351	0.74955	-0.16213
ENSG000C	RP11-97C	3:3126915	4.07881	4.83419	2.8734	2.87458	0.50445	2.28602	7.8161	5.60164	4.18263	5.86871	0.3915	1.07147
ENSG000C	C17orf53	17:441419	0.298581	0.535074	0.615591	0	0.146428	0.494052	0.81471	0.128262	0	2.48779	0.26335	1.28023
ENSG000C	CYLD	16:506936	56.0999	63.8325	13.3435	21.294	15.1293	50.8693	51.3205	34.4819	22.0585	38.7364	0.7937	0.09688
ENSG000C	PAICSP1	9:3751089	0.310967	0.172396	0	0	0	0.290456	0.242921	0	1.59726	0	0.3431	2.48247
ENSG000C	RGS2	1:1928090	117.498	103.464	167.942	651.14	63.8276	54.2967	34.0022	106.85	222.389	70.1194	0.02415	-1.13005
ENSG000C	ZYG11B	1:5272646	1.33782	1.39145	0.693073	0.748183	1.09695	1.2017	0.953168	1.47402	0.416259	2.21219	0.73625	0.219606
ENSG000C	SLC35A3	1:9996838	8.85457	7.96448	4.86647	3.89025	2.48489	6.40354	4.43708	4.93613	8.3225	3.85502	0.9616	0.03012
ENSG000C	CDCA7L	7:2154321	12.234	54.5384	1.34841	9.16999	1.41066	2.10518	27.2926	4.07217	7.28716	5.743	0.24725	-0.60773
ENSG000C	AGPAT3	21:438651	5.50159	6.33892	2.23447	1.01654	5.27746	5.76735	10.1618	3.67265	1.53535	14.0309	0.43065	0.368474
ENSG000C	LAMP1	13:113297	6.18149	2.78756	3.35657	3.39531	6.23535	8.23422	4.36417	7.02775	4.49733	7.21722	0.38675	0.464116
ENSG000C	MTX1	1:1551950	18.2368	22.6923	26.6659	29.4928	31.5976	19.4261	24.7954	22.8527	15.654	9.69729	0.59665	-0.54087
ENSG000C	KIR3DL1	19:548164	0.577718	2.23288	2.20211	13.187	0.368574	3.77081	1.54074	0.068583	0	0.059474	0.32705	-2.00582
ENSG000C	ZNF766	19:522695	18.4563	23.3553	38.4783	22.5847	14.8897	10.7284	11.9654	24.1035	17.9322	27.482	0.23605	-0.57207
ENSG000C	TIGD7	16:326374	2.62334	4.43018	2.53722	1.14807	1.49307	2.52814	2.13485	1.65524	0.141053	3.96346	0.997	-0.00375
ENSG000C	RAPH1	2:2033282	0.330184	0.16814	0.151724	0.765185	0.165345	0.442509	2.00491	0.015004	0	0	0.7748	0.353169
ENSG000C	NUP54	4:7611465	23.6238	26.1893	24.5056	25.3977	20.3124	24.9104	23.5235	22.6757	26.152	13.679	0.8102	-0.08051
ENSG000C	UBXN2A	2:2392728	11.6501	10.2799	6.96908	7.23524	7.40097	10.2226	19.1619	8.85795	13.3544	8.17554	0.54125	0.339475
ENSG000C	SFXN5	2:7294203	1.85763	2.32369	0.8141	1.46338	1.13714	3.50372	2.43203	1.7538	1.41911	1.95215	0.66915	0.214273
ENSG000C	KCTD5	16:268247	21.9411	16.8253	18.8265	11.5946	19.9296	27.3636	28.9801	23.475	19.2056	35.0332	0.14325	0.533035
ENSG000C	LLGL1	17:182255	0.480909	0.733409	0.61474	0.407493	2.09672	0.218045	0.955802	1.50885	0	2.65137	0.99525	0.007116
ENSG000C	STAP1	4:6755872	5.42579	8.49983	2.06043	5.25084	1.92799	5.07943	2.37637	4.37	2.46315	1.76297	0.4873	-0.41657
ENSG000C	PRAF2	X:4907115	44.4114	51.0639	89.8722	58.6925	112.117	30.8503	57.5847	91.8073	83.8634	72.923	0.9571	-0.02885
ENSG000C	MYL4	17:472004	0.30784	0	0	0.775875	0.069293	0	0	0.601506	2.3419	0	0.2689	1.66568
ENSG000C	PRORSD1I	2:5528231	0.885943	1.90664	2.44728	0.964919	0.628678	1.29475	0.987228	2.12951	3.03279	2.34066	0.55565	0.685504
ENSG000C	GATA6	18:221694	0.801343	0	1.9727	0.172784	0.594435	0	0	0.463402	0	0	0.2922	-2.73481
ENSG000C	SLC25A30	13:453334	1.07395	0.192366	0.20455	0.32031	0.462935	0.597685	2.8781	0.313886	0.786104	1.46309	0.36075	1.25285
ENSG000C	GAPVD1	9:1252617	14.9655	15.926	14.1657	8.31096	9.7735	9.31263	13.0075	9.51011	9.93264	20.8424	0.445	-0.29301
ENSG000C	CDKL3	5:1341147	1.57768	0.500454	1.5845	1.00827	0.291822	3.15148	1.87084	2.18889	0	0.025487	0.6432	0.585722
ENSG000C	CTA-14H9	6:2652706	0.609755	0.615636	0.615894	0	0.720897	1.7543	0.299367	2.14011	1.86726	0.599004	0.4508	1.42098
ENSG000C	ADPRM	17:106724	9.22921	27.3172	12.6311	7.74242	7.04552	14.5561	15.4723	12.8364	7.55041	5.27591	0.82975	-0.17242
ENSG000C	TMEM245	9:1090151	8.6848	10.5858	3.58085	3.34168	4.37517	7.4114	8.97712	4.16903	6.63974	14.0254	0.6195	0.210493
ENSG000C	NUDT19	19:326919	1.70907	2.10668	28.4868	16.5408	16.4842	0.891121	1.40553	18.3299	22.2763	23.3539	0.8485	-0.08351
ENSG000C	ABHD11-/	7:7373503	0.379653	0	0.490404	0.103303	0	0	0.809886	2.14204	2.17274	0	0.1748	2.47016

ENSG0000	CHUK	10:100150	18.6733	11.9373	6.24737	6.71891	3.56064	14.6447	4.61014	3.84603	8.53292	6.01883	0.49715	-0.31592
ENSG0000	RP11-632	6:1179075	18.7403	10.7091	7.7014	7.45504	18.2575	13.5534	18.8378	2.91365	3.49478	4.28322	0.4219	-0.71274
ENSG0000	LNPEP	5:9681402	9.8934	12.4323	4.68678	5.08327	4.57741	5.30722	11.972	6.58735	5.10297	11.6133	0.8982	-0.07629
ENSG0000	RAB7A	3:1287261	153.595	156.642	132.737	122.077	122.437	184.917	136.88	98.7336	123.442	128.328	0.8148	-0.07814
ENSG0000	RP11-400	17:425361	5.20494	1.77193	9.88265	2.96239	10.5222	2.28828	1.54771	4.29562	3.1791	14.2624	0.8459	-0.27116
ENSG0000	FOS	14:752787	1310.08	719.171	737.356	2762.58	135.345	84.9168	26.6087	360.282	2118.22	694.585	0.40405	-0.67307
ENSG0000	U47924.6	12:686711	0.920438	1.01869	2.3265	4.02815	2.93997	4.31395	4.89561	2.21086	4.6842	5.24712	0.3033	0.86658
ENSG0000	CYBRD1	2:1715222	0.761245	2.01445	0.090091	0.362708	0.110544	0	0.053545	0	0	0.440032	0.1429	-3.16559
ENSG0000	USP46	4:5259097	1.59867	1.48267	0.858465	1.70402	1.2855	2.58724	1.54337	1.27583	2.5668	2.50987	0.60095	0.348732
ENSG0000	HNRNPAB	5:1782045	67.8088	58.3999	87.8362	71.9014	96.3528	58.3387	85.3949	112.765	62.2683	90.5827	0.89185	0.066774
ENSG0000	BROX	1:2226680	22.5178	24.6673	31.878	25.8683	12.8785	21.4654	17.9923	23.0741	29.8668	23.6902	0.89245	-0.05897
ENSG0000	DDX46	5:1347587	29.1188	32.0676	24.7424	23.7969	48.1169	30.6279	37.7361	24.3792	22.3228	47.5787	0.8376	-0.09728
ENSG0000	RP11-162.	18:767947	0.700187	0.447181	0.885847	0.116236	0.643092	0.471468	0.227841	0.317548	0.243646	0.389974	0.6502	-0.82409
ENSG0000	PEX19	1:1602157	19.6924	22.4889	19.1267	22.3474	11.0031	17.7774	14.6297	14.1945	26.894	10.7022	0.71735	-0.18678
ENSG0000	C9orf72	9:2754654	9.42469	7.97255	5.3537	8.8745	1.65735	4.31785	2.3611	4.40534	2.28304	4.58745	0.14605	-0.78942
ENSG0000	RPSAP6	10:741511	0.051718	0	0	0.662269	0.164928	0	1.08265	0.967638	0	0.758641	0.4406	1.63543
ENSG0000	CRNKL1	20:200343	21.1335	11.6338	15.5043	14.4569	16.0516	29.8034	13.916	12.9568	12.4328	15.1668	0.9922	-0.00374
ENSG0000	RPS18	6:3324953	1194.26	975.143	2939.95	2068.58	925.044	837.292	974.871	1649.67	1302.41	160.885	0.19445	-0.70445
ENSG0000	NDUFA7	19:830212	71.318	64.3501	119.81	131.236	53.9351	79.7638	88.0683	104.293	79.1459	12.2172	0.66825	-0.33475
ENSG0000	MMD	17:553926	15.2229	7.14838	6.3381	11.4591	6.75345	16.1277	11.8589	21.0731	10.672	9.44794	0.2962	0.558558
ENSG0000	AC021224	18:320183	5.32396	5.82269	0.906931	1.37995	0.796058	5.22928	3.48216	0.387507	0.612229	0.467992	0.7894	-0.51219
ENSG0000	NAA40	11:639389	10.372	11.4499	8.0976	12.0217	6.9592	9.38019	5.38335	7.28523	2.20936	10.6905	0.1661	-0.58297
ENSG0000	SOCS4	14:550272	2.40162	2.52558	1.81992	1.70378	3.32053	3.00265	5.04692	3.45996	3.31218	6.29062	0.2388	0.588307
ENSG0000	FAM46C	1:1176059	9.82422	3.46626	14.491	31.0558	3.4456	15.7654	14.0308	12.3894	16.7412	7.29422	0.99375	0.002426
ENSG0000	DMXL2	15:514140	0.799384	3.15058	0.370232	0	0.185459	6.3834	0.017491	0.784594	3.00636	0.702767	0.1361	1.40986
ENSG0000	SELENOT	3:1505419	119.524	84.5459	153.392	138.004	76.3247	159.774	167.089	148.859	93.4951	37.7361	0.8357	-0.12159
ENSG0000	PYGO2	1:1549570	26.7126	37.6423	7.30934	7.41558	8.03401	12.7868	22.258	14.6325	22.98	21.7211	0.73835	0.203884
ENSG0000	IDE	10:924516	12.3119	12.4518	4.60811	4.16963	2.45055	6.31292	5.36807	2.81162	2.10855	5.27996	0.0176	-1.02645
ENSG0000	PIH1D1	19:494462	79.1379	100.799	123.693	115.898	117.109	93.7901	101.267	127.28	76.6427	64.5735	0.71395	-0.27754
ENSG0000	SDCBP2	20:130990	1.47422	1.85709	1.11414	3.2032	0.743547	1.97653	2.33511	3.47567	3.01246	1.57466	0.656	0.563099
ENSG0000	VDR	12:478415	0.69812	2.52312	0.008489	0.440657	0.088615	7.7501	11.3141	8.86196	1.49124	4.84637	0.0004	2.82483
ENSG0000	NMRAL1	16:446167	57.3655	40.2652	81.8239	64.9357	94.5296	61.2978	45.5165	39.996	58.308	39.7466	0.49275	-0.50259
ENSG0000	BDNF-AS	11:275068	0.431537	0.832001	0.884413	0	0.253517	0	0	0	0	0.101384	0.00005	-4.53237
ENSG0000	TTF2	1:1170603	12.7639	12.2388	4.45935	9.70782	3.70059	21.8767	14.5291	7.15779	7.45299	9.32111	0.6742	0.362501
ENSG0000	RP11-932	15:306169	0.379562	0.417121	0.246703	0	0	2.0898	0	0	0	1.04128	0.481	1.64601
ENSG0000	ACOT7	1:6264268	14.5504	19.1256	5.17125	11.8013	10.9817	52.9526	78.2694	46.1197	20.7191	33.5167	0.0013	1.83836
ENSG0000	REXO2	11:114391	96.7994	76.3549	82.9533	70.2968	67.5086	81.7301	118.727	65.8122	68.0501	29.1216	0.90275	-0.05526
ENSG0000	NPW	16:200992	0.157836	0	0	0.5112	0.133406	1.05595	3.05287	1.08638	0	0.359993	0.25395	2.26492
ENSG0000	RAE1	20:573510	35.5268	32.2593	39.7141	31.5773	47.0285	35.9374	31.3468	30.5817	34.8415	38.1144	0.58095	-0.19317
ENSG0000	MAP4K4	2:1016968	16.3411	30.8802	4.83463	1.91862	5.94483	8.57667	11.149	5.1271	2.76498	18.7414	0.25655	-0.51475
ENSG0000	CBX7	22:391201	9.94138	17.0066	5.85599	3.73083	4.82288	3.26084	12.2092	7.68215	8.21181	12.6773	0.78365	0.120188
ENSG0000	KIAA1586	6:5704653	7.76135	4.23374	3.63532	2.62961	6.77014	5.7395	3.75211	4.56836	7.52891	6.21873	0.8697	0.08562
ENSG0000	NDUFAF4I	15:491060	1.08587	1.36313	1.31496	0.297246	0.112641	3.2339	0	0.614179	2.06768	0.545514	0.66255	0.704234
ENSG0000	ZNF222	19:440253	4.7323	4.44895	7.24962	4.98584	3.15045	7.75963	2.84096	2.60915	10.865	3.51109	0.8122	0.164409
ENSG0000	RP11-667	17:203010	0.544959	0.08762	0.845282	1.63565	0	0.409052	1.67987	0	0	0.339618	0.7259	-0.48233
ENSG0000	CXCR6	3:4591789	240.33	18.4028	353.311	193.286	365.73	597.687	579.162	660.807	382.948	405.596	0.01335	1.07867
ENSG0000	RP11-656	1:4047721	0.378922	0	0	0.232003	0.086208	0	0.266606	0	7.17902	0.717352	0.16025	3.79738
ENSG0000	RP11-403	16:664272	0.081007	0	0.397223	1.00513	0.311051	0.494121	0.204585	0.432799	0.830006	0.129431	0.956	0.113689
ENSG0000	CTB-96E2	17:283575	0.203874	0	0.540554	0.32583	0.130074	0	0	0.010897	0.829343	0	0.7815	-0.48618
ENSG0000	SBDSP1	7:7282942	28.7692	20.5968	39.9872	44.8731	30.7716	44.3626	29.7074	32.6915	30.63	22.8342	0.8762	-0.08987
ENSG0000	MTOR	1:1110653	5.63131	7.23603	3.39365	2.16472	4.09079	6.13789	6.3556	4.74783	1.42165	8.83115	0.33645	0.451105
ENSG0000	NKILA	20:576483	0.202305	0.451183	0	0	0.161832	0.762519	0.401433	1.30996	0	0.197106	0.1855	2.22544
ENSG0000	HCG27	6:3119519	0.206255	0.403554	0.726239	0.691111	0.223775	0	0.251538	0.901603	0	1.07445	0.96785	0.045531
ENSG0000	CRLF1	19:185717	1.1806	0.497161	0.943981	1.36635	1.02748	2.82389	0.057514	1.16165	0	1.01113	0.90625	-0.14663
ENSG0000	NOP10	15:343417	253.395	193.978	331.113	298.17	211.305	623.822	365.834	310.859	285.473	53.2029	0.5163	0.231193
ENSG0000	TRAP1	16:361172	27.4673	38.8148	41.6708	43.8228	38.5062	13.0966	40.3088	24.4147	22.8616	28.6074	0.2341	-0.5823
ENSG0000	ITK	5:1570858	62.9677	126.793	18.591	30.511	15.5825	61.4394	65.1662	52.0265	47.4775	41.9872	0.5904	0.235994
ENSG0000	C3orf62	3:4926860	1.81603	2.53831	1.62555	1.03943	1.6953	3.62952	1.27155	1.9443	0.642434	3.71057	0.80475	0.265467
ENSG0000	PATJ	1:6174247	12.5312	10.6581	3.39815	2.33121	2.91507	5.29793	4.19733	5.92854	6.29165	5.38384	0.5431	-0.27204
ENSG0000	PKP3	11:392613	0.289974	0	0.370508	0	0.060951	0	0	1.42778	1.84967	0.848763	0.0583	2.89538
ENSG0000	TBRG1	11:124622	30.4838	35.0631	46.6827	44.4568	43.8908	39.6016	53.8366	47.8604	51.4909	46.7851	0.98065	-0.01742
ENSG0000	SERINC2	1:3140956	0.109503	0	0	0.109272	0	0	0.263899	0.952976	0.632886	0.605704	0.13045	4.25052
ENSG0000	GNAS	20:588189	359.078	331.934	294.621	194.062	503.988	472.34	484.684	414.526	371.824	532.747	0.3051	0.377712
ENSG0000	GPKOW	X:4911338	31.7939	31.5962	46.5597	39.2939	52.4215	38.484	28.911	30.4704	35.7187	30.8349	0.4263	-0.26875
ENSG0000	ZNF580	19:556350	14.0257	24.8647	6.4591	7.20479	6.43973	12.3944	13.6528	9.20442	5.93889	9.22127	0.87	-0.1431
ENSG0000	LRRC75B	22:245404	0.435095	0.457984	0.374276	0.749871	0.191115	5.89773	1.06443	4.2432	5.11804	0.766652	0.0017	2.90999
ENSG0000	CXCR3	X:7161591	41.7054	79.0532	35.5047	21.8615	42.6494	150.949	244.79	156.01	81.3571	176.731	0.00005	1.85646
ENSG0000	MROH6	8:1435661	1.47286	0.583826	0.444245	0.931304	1.61495	6.67518	2.15289	0.271519	0	1.40481	0.2712	1.07277
ENSG0000	EFCAB14	1:4667403	74.8007	46.7754	34.4112	27.4659	52.5243	47.3212	34.4627	29.8537	19.8963	54.1238	0.3457	-0.38037
ENSG0000	COPS7B	2:2317324	21.2231	35.635	22.3377	28.538	24.1439	33.74	28.1058	28.4333	49.8405	27.0815	0.55635	0.324253
ENSG0000	RNF114	20:499363	54.1518	62.0041	77.4009	57.7223	52.4379	46.1213	62.6207	51.2492	48.6001	56.3978	0.52485	-0.21285
ENSG0000	RP11-373	2:2842594	0.067233	0	0.154711	0	0.724963	6.37789	0.733579	0.63057	2.20489	0.46303	0.26135	3.1723
ENSG0000	PZP	12:914883	1.96794	0.12812	0.284483	0.540528	0.665235	0.65811	0	0.146525	0.066597	0.204247	0.0737	-1.89028
ENSG0000	EHHADH	3:1851628	0.667581	0.221952	0.121446	0.261578	0.068613	1.73694	0.318741	0.545909	0	1.07836	0.1612	1.53904
ENSG0000	RP11-16E	15:312160	0.412388	0.098699	0.152972	0.725304	0.607025	0	0.073516	0	2.34298	0	0.65735	0.554601
ENSG0000	SLC31A2	9:1131509	5.39691	17.3459	7.32198	4.8566	7.03719	44.6631	4.79948	5.04045	10.2865	8.82717	0.3796	0.723834
ENSG0000	C17orf80	17:731649	11.8625	15.0029	4.84456	7.27843	3.5782	2.22793	3.1552	4.67243	8.49873	4.09225	0.3348	-0.82607
ENSG0000	SDHC	1:1613142	98.4705	85.6062	198.734	172.278	98.2713	82.0639	72.9637	135.546	173.296	47.5575	0.29355	-0.39021
ENSG0000	RP11-139	2:8743952	9.37414	3.46438	0.316322	1.12133	0.245956	9.68016	5.07209	6.40133	18.9246	0.192697	0.3561	1.81447
ENSG0000	TUSC2	3:5031778	28.8862	26.0513	37.4488	33.4457	27.6111	31.4119	29.9451	30.7808	17.7822	16.5847	0.41535	-0.47576
ENSG0000	B3GNT8	19:414253	12.9063	1.16425	24.2825	41.6824	29.7679	8.97424	2.5491	9.10074	11.0456	12.4962	0.00245	-1.36816
ENSG0000	CHRNB1	17:742010	14.7285	10.4397	25.2083	28.4006	20.2272	10.9878	17.6229	18.6926	25.17	14.335	0.7562	-0.21435
ENSG0000	SARNP	12:557432	120.03	93.8358	101.639	102.873	116.387	184.131	211.146	123.16	131.132	44.5652	0.59825	0.360191
ENSG0000	CHEK2	22:286877	4.40558	6.79055	3.08671	2.34005	5.02608	6.75632	5.57207	3.20198	0	2.40587	0.4621	-0.47105
ENSG0000	AGAP7P	10:461096	1.14288	2.56374	1.39148	0.757244	1.10154	1.58207	1.42061	1.15516	1.23003	2.10798	0.60505	0.084553
ENSG0000	ATXN7L3E	12:745378	9.28103	11.135	3.65227	5.25429	4.80462	4.70773	7.37383	6.72367	4.61105	6.33817	0.58675	-0.1857
ENSG0000	ZNF763	19:119250	0.720673	2.62609	1.75324	2.71766	0.324708	0	0.533071	1.43081	4.44645	2.65411	0.94625	0.073733
ENSG0000	ADAM19	5:1572601	3.48912	3.87633	6.98167	7.46831	3.85076	16.5324	24.1039	44.1078	30.2078	32.9378	0.01035	2.43191
ENSG0000	FYB	5:3910523	125.03	435.72	139.964	84.903	137.883	238.822	201.65	168.608	130.267	281.84	0.6469	0.179166
ENSG0000	RP11-388	16:311801	0	0	0	1.7837	0.813309	0.478255	0	1.98293	1.26157	1.20495	0.52435	1.46625
ENSG0000	MZT2B	2:1301513	136.088	91.9466	237.755	197.774	173.264	160.959	174.025	236.533	131.64	37.5476	0.53455	-0.28957

ENSG0000	FXYD7	19:351245	1.06823	1.62833	3.36387	12.4077	4.84009	0	0.516098	1.16718	12.8054	3.039	0.8625	-0.33519
ENSG0000	FCER1G	1:1612152	31.6364	127.655	20.0979	74.8651	37.7863	642.862	36.0199	48.8911	140.978	49.2579	0.0003	1.62732
ENSG0000	PIK3R5	17:887891	46.0987	55.1426	18.754	22.6517	22.4798	17.323	21.6222	27.0972	18.7093	33.3613	0.13355	-0.49243
ENSG0000	SCPEP1	17:569781	8.93157	31.6887	14.0902	11.6712	7.82525	46.2605	9.39298	6.47712	13.1388	10.0365	0.47875	0.283898
ENSG0000	ZBED3	5:7707207	0.851092	0.370867	1.03444	0.618378	0.863684	1.55317	0.859154	0.206142	0.443064	0.187356	0.50045	-0.70517
ENSG0000	MOB1A	2:7415252	76.069	64.9263	36.6546	32.5423	30.9174	60.9104	49.3375	53.3449	58.4651	35.6929	0.65235	0.156165
ENSG0000	HAPLN3	15:888772	22.4333	80.7844	11.3255	13.9033	10.2764	53.4263	101.131	53.6749	39.7768	39.0965	0.02165	1.12061
ENSG0000	SARAF	8:3006301	380.444	564.568	435.058	463.986	271.391	331.764	399.475	446.638	686.171	273.391	0.9923	0.003871
ENSG0000	ABCB8	7:1510284	18.3004	24.4045	20.8853	16.2089	15.1132	22.4244	20.345	18.0256	25.6871	24.5958	0.6321	0.182148
ENSG0000	SNX29	16:119767	4.57215	7.77413	3.48908	2.35812	5.27219	4.07613	5.47007	5.17085	11.2362	6.25229	0.8424	0.150419
ENSG0000	ASPRV1	2:6996008	0.61831	0.783467	0.406249	0	0.598256	0	0	0	0	0.581101	0.40895	-2.07058
ENSG0000	SLAMF7	1:1607390	96.2047	18.6985	128.686	119.516	107.42	81.3969	47.4537	88.0801	57.3816	81.524	0.15035	-0.50443
ENSG0000	C2orf74	2:6106587	31.7308	25.8064	35.3128	30.2557	39.5318	41.6032	29.8517	8.35984	23.913	16.0448	0.44955	-0.44161
ENSG0000	ATP6V1G2	6:3152871	10.9809	11.7607	18.0397	15.6615	29.2957	6.52541	5.1204	23.1403	9.61977	24.5842	0.71335	-0.25204
ENSG0000	MAP2K1	15:663868	25.6647	30.7633	46.0913	73.3306	42.993	28.9178	29.1739	49.2619	57.6041	52.5451	0.8729	-0.07772
ENSG0000	RP11-274I	7:1286511	2.01919	1.9052	1.27668	0.099094	1.23388	1.88179	0.665497	0.862797	0.016297	2.34396	0.8571	-0.22396
ENSG0000	METTL22	16:862168	10.4081	11.2808	15.4759	14.1216	11.3954	13.9057	10.8031	8.46702	6.32636	20.886	0.77435	-0.13354
ENSG0000	RP11-95M	6:1376733	0.913019	0	0.340115	1.77753	0.293956	0.733295	0	0	0	0.214848	0.52385	-1.96207
ENSG0000	ACVR1	2:1577364	4.48317	7.09125	2.13579	3.74455	1.81455	9.29329	6.8457	3.1687	3.59563	6.81195	0.20685	0.579026
ENSG0000	HEXIM2	17:451485	11.0408	2.73826	11.2163	9.4988	13.728	6.86078	6.85181	6.30744	1.21398	5.9636	0.3059	-0.92069
ENSG0000	RAB3GAP1	2:1350522	4.00292	12.5782	1.65248	3.30497	2.94361	9.45531	9.1144	7.62871	10.7825	10.8981	0.1912	0.83529
ENSG0000	CD248	11:663128	1.57882	11.6153	1.49123	1.04569	1.15826	6.71774	8.71831	1.81382	0	4.77816	0.63775	0.456186
ENSG0000	NCF1	7:7477396	9.02573	39.7022	8.72988	13.9046	8.95385	48.0592	8.62221	11.9942	15.5819	5.64716	0.93375	0.052813
ENSG0000	TRBV13	7:1425358	2.82921	8.08431	1.68295	0	0.115092	3.64962	8.19129	0.478654	0	8.97882	0.5901	1.21541
ENSG0000	ARHGAP2	2:6867960	138.065	130.697	128.135	93.1452	115.574	81.4073	73.6668	56.0265	38.9471	90.4685	0.01345	-0.79923
ENSG0000	PCBP1	2:6996008	84.318	66.1051	138.79	73.3289	238.951	85.1086	100.506	121.846	58.3193	170.967	0.6124	-0.19775
ENSG0000	PRR12	19:495916	0.496563	0.240717	0.258386	0.342499	1.11535	0.634451	0.957955	0.360891	0.031129	2.49754	0.3582	1.02151
ENSG0000	FDXR	17:748624	8.39683	9.40428	6.2196	7.36296	3.85354	17.1482	6.90512	12.4597	13.4202	5.79066	0.17235	0.701471
ENSG0000	MRE11	11:944155	11.4343	12.9936	3.90433	3.52527	5.15321	14.1057	11.7081	3.22314	3.02878	6.31296	0.7439	-0.21246
ENSG0000	SCRN3	2:1743957	9.33707	10.2674	8.21084	10.4446	4.36289	7.66421	4.6981	6.56532	22.0122	7.80002	0.848	0.148089
ENSG0000	DDX3Y	Y:1290410	69.1956	70.7996	17.5549	27.3926	14.857	33.3281	23.6152	26.6534	28.0365	31.6873	0.16535	-0.43529
ENSG0000	LGALS4	19:388016	0.197706	1.34145	0.345431	0.156841	0.446564	0	0.940408	0.136646	0	1.55318	0.711	0.384428
ENSG0000	RDX	11:110174	10.8545	8.85697	10.5965	11.1714	22.2186	11.0192	19.4819	16.4657	10.3315	27.036	0.50195	0.298365
ENSG0000	GIT2	12:109900	58.2407	66.2776	59.7499	44.4727	39.521	52.0306	37.6599	54.9482	44.6971	60.8124	0.49375	-0.26834
ENSG0000	SSX2IP	1:8464370	5.27848	2.86116	3.72698	2.40531	6.74281	2.1431	2.44812	0.605759	0.646347	3.89391	0.03415	-1.47182
ENSG0000	ZNF436	1:2335944	2.68368	1.84666	0.864108	1.5799	0.619723	0	3.13561	2.11709	0.245997	2.64314	0.9431	0.059291
ENSG0000	CTD-3195	17:371462	0	0	0	0	0	0.616228	0.937879	0.34183	0	0	0.1354	inf
ENSG0000	RP4-800G	7:1492621	3.13032	10.7765	1.01484	0.434368	0.78292	3.51716	1.3187	2.1761	0	2.44041	0.4484	-0.7838
ENSG0000	RP13-36G	X:7394432	0.431224	0.210672	0.48292	0.400273	0.190994	0.15221	0.746522	0.482559	0	0.911136	0.71465	0.464649
ENSG0000	ELL2	5:9588509	2.6493	1.29707	0.616239	1.16454	1.28231	2.89732	1.09567	1.31662	4.47631	1.22635	0.60775	0.593462
ENSG0000	RP11-254I	2:1003909	1.34327	3.34104	2.15774	3.80861	4.11238	0	1.01094	1.10681	0.865911	2.37441	0.3289	-1.51925
ENSG0000	RP11-449I	7:996985-	2.98007	3.18784	0.317027	3.04121	0.099005	0	1.62729	0.883697	1.76098	0.136741	0.65115	-0.87561
ENSG0000	RP11-465I	1:1059733	0.863741	3.89608	0.082242	1.06049	0.213974	1.93237	8.3384	2.63748	0.335421	1.29211	0.37195	1.29218
ENSG0000	CASP12	11:104885	0.623299	0.08787	0.45512	0	0.121418	0	0	0.696695	2.92156	0	0.15985	1.89515
ENSG0000	PAIP1	5:4352626	44.2425	27.735	21.9279	15.0114	24.9016	33.6719	47.0934	22.063	28.4154	18.7869	0.56005	0.220744
ENSG0000	P2RY11	19:101062	4.65385	2.19043	8.96173	4.86557	11.2324	18.4348	7.82541	15.7664	4.01674	17.6458	0.37615	1.03857
ENSG0000	ARHGAP1	4:1477320	18.4685	3.98281	12.0148	21.8891	10.6867	5.13127	3.59568	3.0097	12.3699	7.29963	0.10695	-0.84016
ENSG0000	C12orf57	12:694297	105.02	67.217	98.4109	75.512	53.1178	164.736	155.657	127.251	106.505	22.6465	0.3594	0.438453
ENSG0000	VCPKMT	14:501086	16.7636	20.4481	17.5758	15.5285	12.4622	17.7338	17.2144	11.0806	19.1042	10.6456	0.81485	-0.11718
ENSG0000	GS1-259H	7:9954630	0.940503	1.92302	0.710693	0.78125	1.25173	0	0	1.54381	0	0.577397	0.3699	-1.32525
ENSG0000	DMAP1	1:4421345	26.1149	37.2923	31.2306	30.0412	34.989	33.4245	29.0565	24.4191	31.6468	29.5216	0.8887	-0.05368
ENSG0000	C11orf63	11:122882	2.63394	4.77929	1.97201	1.03027	5.39508	1.98163	6.16029	4.42776	5.40909	4.6081	0.36045	0.602068
ENSG0000	RP3-337H	6:4350696	0.354149	0.306482	0.074016	0.637347	0.41474	1.38446	1.46127	0	0	0	0.7414	0.583753
ENSG0000	NDUFV2P	19:532239	3.9606	3.62917	0.367032	5.24262	1.15452	8.75565	5.18816	0.305556	4.41459	0.388285	0.57255	0.426569
ENSG0000	DHX37	12:124946	2.42458	1.41519	1.93366	0.941044	2.21898	0.769498	2.26321	2.27463	0.883897	3.93556	0.92585	0.104496
ENSG0000	XXbac-B4	22:180765	2.49755	2.78924	6.02621	5.3779	5.68925	3.2825	5.55572	3.52985	17.0207	9.50358	0.5446	0.822201
ENSG0000	LIN9	1:2262311	3.88496	4.10767	1.81185	3.1644	1.06378	4.03195	2.24997	1.29944	0	0.959176	0.46205	-0.74265
ENSG0000	MIOS	7:7552461	16.5499	15.1475	7.19969	6.17052	3.90016	10.311	12.6615	8.96116	2.87	6.99068	0.49795	-0.2997
ENSG0000	RWDD4	4:1836396	19.748	11.5714	16.6782	17.7083	13.805	17.6853	20.5126	15.2858	15.7016	8.77316	0.8829	-0.06353
ENSG0000	SLAIN2	4:4834132	11.4345	9.1893	4.511	4.22504	6.62249	5.85441	7.5032	6.43653	6.10235	13.5613	0.91305	-0.04186
ENSG0000	DMTN	8:2204899	2.89282	0.800433	1.02491	1.35885	2.71277	2.10668	2.6904	4.48578	0	4.74502	0.41765	0.584378
ENSG0000	ETV6	12:116498	5.80085	7.01607	2.57304	1.51476	2.11303	3.65771	5.1123	4.91652	9.29894	4.1027	0.263	0.578564
ENSG0000	DDR1	6:3087642	1.1444	1.45722	0.343033	0.276075	1.43485	0.109151	0.683659	0.095875	0	0.762373	0.00705	-1.9398
ENSG0000	MMS22L	6:9714216	1.90878	3.24768	0.425377	0.74626	0.534726	0.847555	2.76444	0.62124	0	1.51	0.70325	-0.29804
ENSG0000	FAM89A	1:2310189	9.68368	7.56343	5.86544	3.63088	9.85141	10.6329	11.6229	6.53061	4.49872	8.74843	0.8317	0.130579
ENSG0000	AL022067.	6:1060454	4.56325	0	5.87267	5.76528	7.71465	15.2565	11.5672	5.62864	0.26011	8.91196	0.8074	0.375174
ENSG0000	GGA3	17:752365	14.5432	18.048	5.77385	2.91241	6.84694	6.77936	8.27786	7.47148	4.96692	13.1988	0.88935	-0.12102
ENSG0000	CPQ	8:9664522	1.93577	11.0003	4.05425	4.90631	12.0432	3.44892	5.10763	3.18478	0	7.23285	0.2867	-0.72118
ENSG0000	SUGCT	7:4013497	0	0.48959	0.602147	0.980057	0.159042	0	0	0	0	0	0.11485	#NAME?
ENSG0000	RP11-108	15:340840	0.343584	1.21734	0.122658	0.638857	0.132399	2.05588	0.396356	0.695792	0	1.24905	0.52165	1.13591
ENSG0000	MRPL35	2:8619935	14.1994	18.4578	20.617	18.0189	12.5436	23.061	18.8114	30.6003	20.0431	7.73502	0.9876	0.011128
ENSG0000	KIAA0141	5:1419238	20.2188	32.8073	12.6492	14.641	12.3053	35.3491	17.903	14.0381	11.6564	20.3572	0.7998	-0.11407
ENSG0000	RP3-330M	6:4318217	0.799376	0.818245	0	0.681755	0.09079	0.91602	1.75582	0.047647	1.97071	0.1052	0.62475	1.05244
ENSG0000	SOCS5	2:4669895	1.46086	2.4437	2.16071	3.0708	1.59936	2.38389	1.21057	1.1357	4.78631	1.85729	0.9868	0.01112
ENSG0000	BACH2	6:8992652	1.81533	13.1017	1.96652	6.745	1.87606	2.98771	1.76789	0.863725	1.71823	3.48246	0.2874	-1.31214
ENSG0000	BZW1	2:2006782	165.822	108.254	81.3645	115.834	82.6056	170.944	147.719	112.406	112.712	77.044	0.99655	-0.00182
ENSG0000	MT-ND4	MT:10469-	283.225	253.101	2154.56	1833.85	3810.32	399.23	218.693	1969.37	2335.68	4015.66	0.93465	-0.12918
ENSG0000	BBS10	12:763444	1.74712	1.95396	0.415749	0.825049	0.360331	3.70282	1.04739	2.05437	2.45958	1.02412	0.14225	1.03277
ENSG0000	LENG8-AS	19:544448	3.92152	0.846177	2.5443	2.19272	0.73433	3.83897	4.16037	3.40329	0	1.78218	0.889	0.155899
ENSG0000	RP4-705D	20:167148	0.66572	0.437422	0.353195	1.35018	0.08189	0.773341	0.25567	0	0	0.422964	0.47215	-1.04553
ENSG0000	FAM228B	2:2407652	6.94771	4.63908	5.54213	5.47922	5.53519	2.89957	7.72474	3.20412	2.8812	10.9511	0.9702	-0.0216
ENSG0000	RP11-381	3:1085498	0	0	0	0	0	0	0	0	2.94683	0	0.1674	inf
ENSG0000	ZSCAN31	6:2832469	0.35167	0.036351	0.582104	0.409317	0.466949	0	0.893612	1.40683	0	0.085448	0.61055	0.371258
ENSG0000	NDUFB7	19:145660	208.313	118.966	298.64	280.208	184.296	256.376	243.529	218.406	141.353	26.7116	0.16735	-0.47726
ENSG0000	PSMB6	17:479614	180.704	137.679	266.252	279.401	203.686	280.478	221.056	221.193	242.825	66.4428	0.81095	-0.07826
ENSG0000	RPS6KA2	6:1664093	1.28965	1.69611	1.89125	1.66522	0.620542	0	1.56402	2.31156	0.869228	1.49425	0.6922	-0.32954
ENSG0000	SULT1B1	4:6972116	0.576426	2.98047	0.274343	0.585508	0.193841	0	0	0.031534	1.19748	0.003636	0.2153	-2.2466
ENSG0000	ZNF740	12:531576	2.80877	6.6697	1.4338	1.21912	1.26322	3.66602	2.31996	0.84584	1.57431	3.54695	0.8711	-0.17644
ENSG0000	HIST1H1D	6:2623426	39.9966	58.9649	5.82434	13.7628	23.3859	37.4775	55.7583	3.16825	3.35908	0.999229	0.3536	-0.66725
ENSG0000	TENM4	11:786528	0	0	0	0.104017	0.11765	0.003023	0	0.012252	2.44555	0.204577	0.24735	5.0195
ENSG0000	TRIB3	20:362834	16.7691	8.6752	14.5306	11.7191	18.5979	26.2003	21.7242	23.304	6.08101	16.4099	0.3676	0.358685

ENSG0000	ATXN7L3	17:441918	4.00698	4.85523	0.810037	0.179347	1.74509	4.51651	5.49553	2.48987	0	2.92191	0.77945	0.28785
ENSG0000	UCHL3	13:755494	41.9775	38.8765	49.5971	49.7114	25.3494	70.9208	45.987	44.778	37.6858	20.5189	0.8137	0.10608
ENSG0000	ZNF554	19:281987	2.13549	1.54988	1.95934	1.38009	1.73194	0.251066	0.049386	1.54546	4.23108	1.36114	0.94535	-0.07454
ENSG0000	VAC14	16:706874	25.6384	40.7219	15.6853	19.5247	16.716	31.8365	15.4706	16.3035	21.5905	16.8535	0.95815	-0.02203
ENSG0000	ZDHHC6	10:112374	62.7729	68.929	41.239	52.1256	30.5808	84.1665	58.5298	47.88	38.3763	32.8299	0.8706	0.07578
ENSG0000	RFC2	7:7423149	19.7496	17.53	25.7896	31.7764	19.6334	24.5668	26.0173	30.5733	27.6461	11.9654	0.9987	0.000923
ENSG0000	C8orf44-S	8:6666759	0.858394	0	0.278254	0.409834	0.610231	0	0.532667	2.71263	0.292297	1.47642	0.7745	-0.50366
ENSG0000	RP11-434	2:8721133	0.674	0.120202	0.561856	0.783452	0.277419	2.89627	0.734533	0	3.78908	1.70081	0.2922	1.83687
ENSG0000	CEBPD	8:4773690	207.935	53.8474	370.934	275.105	630.802	173.505	72.3391	161.347	122.584	146.404	0.0011	-1.19812
ENSG0000	C2orf88	2:1898796	0.12434	0.251994	0.816804	1.69009	0.015515	0.236492	0	0.0823	0	0.146987	0.0259	-2.89504
ENSG0000	CEP97	3:1017239	3.69858	2.15876	1.38416	1.67857	3.31962	1.79355	4.69168	2.53234	4.8499	3.33003	0.68375	0.262089
ENSG0000	HMGN2P4	15:454305	0	0	0.308176	2.24302	0.311304	1.70227	0.590361	0.179788	0	0.191947	0.6973	-0.49433
ENSG0000	AHSA2	2:6117741	25.3617	41.4347	9.57866	4.68295	8.56212	29.2147	23.164	11.7617	5.85448	23.4677	0.97195	0.02281
ENSG0000	TIMM8A	X:1013456	4.0007	3.23363	5.21497	3.87739	3.39053	0.408055	4.88093	2.97502	8.54976	2.85022	0.93675	0.061117
ENSG0000	UBR5-AS1	8:1022393	2.74508	1.88346	1.4332	0.654347	1.37764	1.23604	1.51524	2.42462	0.731134	2.66466	0.91145	0.143736
ENSG0000	FAM117A	17:497009	30.2857	19.7812	18.292	12.8797	23.991	9.38865	19.487	11.1075	15.4434	29.2278	0.63015	-0.332
ENSG0000	CLEC1B	12:994813	0.426133	0.456221	0.800271	1.52067	0.361705	1.69386	0	0	0	1.3407	0.83625	-0.37841
ENSG0000	LPCAT3	12:697089	25.6839	36.3633	18.4723	21.3868	15.8759	39.3245	28.4048	15.942	14.1891	43.7845	0.76585	0.177712
ENSG0000	TOR2A	9:1275793	40.7353	27.1742	53.9472	64.8378	73.5442	63.7614	39.6045	37.559	39.0597	45.5603	0.5206	-0.28725
ENSG0000	DGKH	13:420400	0.847517	1.25078	0.348539	0.283558	0.265134	0.667327	1.17101	0.246429	0	1.22459	0.80265	-0.19091
ENSG0000	LARP4	12:503262	12.5932	10.5995	7.40334	6.53357	10.7657	12.6372	15.1712	7.164	6.35255	14.7294	0.97815	0.013566
ENSG0000	NT5C3A	7:3301411	47.5095	37.0591	39.2897	53.674	26.3972	34.2651	93.7621	71.7734	55.5999	23.994	0.13785	0.506173
ENSG0000	REXO4	9:1333763	16.5547	14.7334	18.5901	21.2045	22.7812	19.3233	13.0978	18.7356	23.0882	20.6029	0.9804	0.008677
ENSG0000	DEGS1	1:2241757	83.3021	70.0365	77.0208	101.959	54.9461	87.7817	107.803	85.7726	77.4118	70.6219	0.72305	0.109759
ENSG0000	VPS26A	10:691235	45.1581	55.4905	22.0457	26.5135	19.0666	54.5808	38.7223	18.8488	27.0651	20.5383	0.9552	-0.01639
ENSG0000	MED14OS	X:4064830	1.93895	3.58408	2.58205	1.79523	1.11368	4.7504	0.634754	0.730515	1.87645	1.53579	0.86205	-0.26996
ENSG0000	SLC7A4	22:209991	0.137162	0	0.135783	0.881886	0.019535	0.555234	3.52776	0	0	0.427885	0.45775	1.53706
ENSG0000	TOMM6	6:4177017	170.451	132.187	214.173	182.537	182.855	254.641	217.206	171.718	146.214	33.3592	0.61315	-0.19131
ENSG0000	EEF1B2	2:2061148	198.312	200.574	324.878	229.354	166.383	161.324	192.1	207.927	154.75	41.0823	0.2114	-0.5637
ENSG0000	NAE1	16:668028	65.8918	53.3463	54.3366	63.4245	47.0344	38.2663	41.1946	32.1608	24.3255	23.895	0.0092	-0.83459
ENSG0000	CDK5RAP3	17:479415	96.6262	95.924	101.387	113.172	97.91	130.262	66.2851	70.9689	120.888	120.496	0.56935	0.193672
ENSG0000	PIK3IP1	22:312815	116.683	375.591	188.175	236.233	108.213	117.438	126.501	227.784	266.733	174.871	0.8327	-0.08222
ENSG0000	LRWD1	7:1024649	17.3524	13.4097	20.5369	15.6331	16.0491	21.2332	18.3056	24.9917	11.068	12.745	0.95845	0.02704
ENSG0000	NLRP6	11:278364	2.39775	6.70643	0.965452	0.466126	1.39077	4.81091	1.12618	1.22878	1.20087	3.39088	0.8071	0.252842
ENSG0000	RP5-1024	1:5319642	1.2131	2.13382	0.936181	1.32295	0.842856	1.92505	0.698788	0.968908	1.21003	0.261136	0.8852	0.176
ENSG0000	ZNF587B	19:577696	1.4237	0.620997	1.35066	1.04043	0.904012	1.52101	2.12301	0.990906	0.543866	0.762097	0.9651	0.040987
ENSG0000	WIPF3	7:2980648	0	0	0.012671	0.145141	0	1.521	0.043947	4.35914	0	0.433944	0.19925	5.42971
ENSG0000	KIF22	16:297907	48.1051	89.1448	67.6752	55.7256	46.7882	67.8295	98.2699	48.4244	63.6684	52.8874	0.75145	0.103056
ENSG0000	RP11-640	17:320078	0.381473	0.367645	0.988939	0.917198	0.493417	0.488134	0.841476	0.779558	0.459438	1.24159	0.90935	0.201433
ENSG0000	SNRPN	15:248236	231.38	216.171	362.755	259.258	320.75	163.502	194.985	218.409	196.6	165.018	0.1322	-0.58729
ENSG0000	PFDN1	5:1401746	77.5473	83.7656	116.986	108.615	80.4386	83.2388	67.6659	63.651	96.9892	30.0363	0.24375	-0.44011
ENSG0000	GDPD1	17:592204	1.6664	0.247195	2.72225	3.93418	2.08896	0.69138	0	1.32806	5.47825	3.00808	0.88315	-0.15615
ENSG0000	DDX12P	12:941867	1.37764	3.2659	0.351614	0.248379	0.364303	1.61809	0.687242	0.26624	0	0.494161	0.5608	-0.74805
ENSG0000	RP5-1085	20:328566	3.04975	5.77375	8.19725	1.82181	4.16185	2.87593	4.64654	8.57818	8.68664	10.8488	0.3176	0.541656
ENSG0000	GNAQ	9:7771608	3.30546	4.38659	0.832039	0.950541	2.06305	3.08367	1.1153	1.46594	0.586128	2.70465	0.3116	-0.48203
ENSG0000	RPS6	9:1937545	1025.32	1006.62	1778.95	1322.12	1260.05	1015.62	818.399	1102.39	975.216	209.031	0.16485	-0.60792
ENSG0000	SDHAF3	7:9711658	67.5337	38.8154	75.8093	75.649	29.4247	46.6079	37.3215	47.7025	105.598	7.90894	0.7345	-0.15598
ENSG0000	RPRD2	1:1503630	9.29532	18.4454	4.74028	4.9994	5.4157	4.40153	12.8287	4.85399	5.69897	10.885	0.7462	-0.19704
ENSG0000	CABLES2	20:623886	3.69859	4.48193	1.16319	0.055174	1.33742	4.40041	2.68657	0.677735	0.014406	3.66515	0.97545	0.023964
ENSG0000	CTD-2649	16:219502	0.431707	0.698526	0.696633	0.52585	0.449312	0.027454	0.567311	0.521201	2.9192	1.85147	0.69825	1.06094
ENSG0000	CTD-2369	19:103048	5.03381	5.56709	4.89309	1.09026	4.40751	1.94118	3.42603	2.47392	0.316692	0	0.8337	0.358754
ENSG0000	ZNF562	19:960467	2.35242	1.02522	2.14336	2.36171	3.31194	1.77777	2.65444	3.60364	1.54003	4.05987	0.82475	0.137446
ENSG0000	FGFBP3	10:919065	2.81728	3.17865	2.99511	1.45168	3.63342	5.66884	2.36665	2.29982	0.364241	5.20796	0.83	0.189555
ENSG0000	MRPS18C	4:8345593	33.0415	23.0404	50.0395	26.4181	18.8567	58.1019	41.4944	41.445	32.021	8.25704	0.5241	0.293855
ENSG0000	RP11-342	1:4276726	0.50304	0.7494	0.469861	0.994443	0.547021	3.59748	0.772545	0.261732	0	0	0.8579	0.250841
ENSG0000	MFSD1	3:1586968	37.8835	35.1965	23.4836	39.7859	21.7621	67.8847	26.6811	28.401	30.0849	19.1606	0.8009	0.088775
ENSG0000	FOXRED2	22:364871	1.59345	1.95774	1.34567	0.591924	1.04591	0.901834	0.129603	1.0607	0	0.409921	0.19055	-1.40344
ENSG0000	ELOVL5	6:5326739	55.0042	57.9994	44.385	38.362	24.6349	51.951	51.0609	53.3529	40.0213	43.61	0.7895	0.089431
ENSG0000	AC004453	7:4437912	0.956155	0.72733	1.17589	1.83718	0	0.675729	1.02159	0.491812	0.375153	0	0.52905	-0.90461
ENSG0000	SNRK	3:4328651	23.2749	32.3385	11.7513	12.7999	13.2188	5.20877	7.57869	8.37489	11.9004	21.9922	0.07765	-0.71963
ENSG0000	YARS	1:3277523	122.498	54.8097	92.5689	99.4739	95.308	97.033	138.253	119.732	63.4932	67.5587	0.74885	0.11139
ENSG0000	CLK1	2:2008530	178.382	160.28	139.129	125.905	113.008	138.793	127.445	137.945	164.941	142.244	0.38805	0.320652
ENSG0000	MDGA1	6:3763067	0.004195	1.03154	0.030466	0.81627	0.540636	0.101635	0	0.600462	1.94897	2.09192	0.6403	0.497097
ENSG0000	LINC0040	13:742314	0.553503	7.31772	0.523335	1.59354	0.021041	0	0	0	0	0.012428	0.24585	-9.98307
ENSG0000	RP11-325	15:650015	0.194712	0	0.612237	0	1.37245	0	0.146808	0	0	0	0.1597	-4.25164
ENSG0000	NDUFAF8	17:812392	77.0806	46.1776	115.049	130.091	60.5982	87.6379	104.573	77.8858	53.8527	18.4536	0.2233	-0.50338
ENSG0000	NDUFA6	22:420855	94.4437	70.7803	104.253	101.989	94.4207	133.04	131.383	109.206	78.6014	29.9232	0.93325	0.029756
ENSG0000	ST20-AS1	15:798335	1.36364	1.49165	1.35025	1.65891	0.7159	1.27014	0.792051	1.82699	0.058867	1.04116	0.85315	-0.2857
ENSG0000	GIPR	19:456682	0.60389	0.923118	0.562496	0.888687	0.402863	1.50255	1.55667	0.989881	0.964852	1.42171	0.19625	0.885055
ENSG0000	RP11-267	12:120498	0	0	4.69063	0	0.907733	0	0	2.44168	0	1.62174	0.9376	-0.1895
ENSG0000	HIRIP3	16:299923	19.068	24.8891	11.8787	12.9502	21.037	23.0763	22.2271	22.6307	30.8986	16.4619	0.36995	0.474499
ENSG0000	CYTH1	17:786740	197.087	216.631	134.738	148.704	124.934	121.658	97.9013	97.8833	84.7395	129.267	0.1105	-0.49614
ENSG0000	ITPR1	3:4493347	4.18689	4.70646	1.26938	3.32021	1.19894	6.48799	3.14273	3.07653	11.7358	3.82283	0.0494	0.901219
ENSG0000	RP11-73M	14:103553	0.987551	2.03318	0	0.627189	0.756286	1.00043	1.90862	0.229575	0	1.85811	0.8445	0.297506
ENSG0000	STMN3	20:636397	7.06056	29.8577	8.59853	3.94625	8.94393	6.87479	7.88634	6.64683	9.18574	18.5156	0.80805	-0.11909
ENSG0000	BGLAP	1:1562129	0.710817	0.401293	0.356668	0.506925	1.06123	3.34697	4.24246	0.118861	0	1.41285	0.3011	1.35676
ENSG0000	PERP	6:1380885	11.0151	3.73145	8.24399	6.53982	7.09729	19.0685	13.1948	19.5131	20.033	14.7049	0.0016	1.18842
ENSG0000	CREG1	1:1675296	16.5889	14.987	8.91498	8.42456	6.87177	69.7083	15.2119	8.83802	16.1509	5.63111	0.01625	1.03064
ENSG0000	TAF4B	18:262259	2.86645	4.45447	0.667151	0.367658	1.3873	1.51345	2.9529	1.45728	0	1.37456	0.61315	-0.40979
ENSG0000	RP11-246	8:5749288	0	0	0	0	0	4.17204	0	0	0	0	0.12705	inf
ENSG0000	C4orf3	4:1192964	82.3339	94.2737	114.214	104.984	78.5325	106.657	67.836	96.1649	100.749	41.8515	0.8375	-0.07848
ENSG0000	C12orf45	12:104986	7.74876	5.32772	8.56083	7.62429	4.94229	13.0015	8.22428	10.1886	7.06811	1.03186	0.7375	0.232368
ENSG0000	CCDC85B	11:658901	125.188	71.5611	167.259	138.943	310.705	114.036	199.075	131.065	77.9959	117.806	0.13955	-0.4984
ENSG0000	METTL26	16:634426	86.0181	85.0598	146.709	128.251	131.576	96.5185	123	118.556	86.3452	49.135	0.26815	-0.39704
ENSG0000	ZNF93	19:199009	1.53574	2.09763	0.803268	3.49263	0.599584	2.05243	1.31895	1.82243	1.60624	0.867398	0.82235	-0.1498
ENSG0000	ARID4B	1:2351311	51.2741	92.166	60.4299	45.9302	67.5818	68.3502	105.083	45.1826	59.8441	124.672	0.8944	0.071058
ENSG0000	GS1-166A	7:1659362	0.723648	0.667897	0.484915	0.258759	0.113926	1.30517	0.359277	1.47921	0	1.40413	0.5514	1.25562
ENSG0000	RP11-468	14:241718	1.83644	3.57119	2.47451	1.97808	1.5211	1.07167	2.56763	3.92638	4.73293	4.88961	0.6109	0.595483
ENSG0000	CBL	11:119206	2.51825	3.69955	2.81834	3.35015	4.9837	2.27245	3.52779	8.13496	1.54181	12.348	0.5395	0.40186
ENSG0000	PPHLN1	12:422384	68.1809	69.9218	89.0215	94.6447	91.4295	52.1448	70.5386	78.9808	64.673	65.3512	0.41725	-0.38888
ENSG0000	PRMT7	16:683109	32.3553	29.0633	34.2686	39.3826	24.4804	18.5815	18.1139	16.7165	30.2377	25.3248	0.17075	-0.54469

ENSG0000	AC010468	5:1111922	0.721823	0.444745	0.64937	0.464633	0	0.353248	0.463305	0.211689	0.165955	0	0.6191	-0.88342
ENSG0000	C19orf71	19:350627	0.065957	0.129651	0	0	0	0	0.86102	0.246237	1.39218	0.230137	0.1865	4.12452
ENSG0000	CTD-2562	15:753467	0.499527	0.693203	0.458408	0	0.16909	1.62605	0.480227	0.944345	0	0.154955	0.59965	0.793448
ENSG0000	TEPSIN	17:812282	12.4094	11.9518	4.26223	2.45388	4.2715	11.978	7.96475	4.61592	3.3392	5.01747	0.6567	-0.21459
ENSG0000	SPAG5-AS	17:284557	3.22448	3.47909	1.37534	1.19454	0.435153	0.186049	1.97456	0.536902	0.802531	0.533393	0.276	-1.3025
ENSG0000	L3MBTL3	6:1300136	4.86042	5.49894	2.55497	0.684371	1.98894	5.47073	4.29868	0.719368	0	3.33955	0.76005	-0.28315
ENSG0000	GGCX	2:8553916	10.6236	5.24049	10.515	18.0436	3.90292	9.89382	10.4963	8.13725	8.01866	3.34681	0.6889	-0.38997
ENSG0000	CYTOR	2:8743952	78.762	45.593	54.9391	61.2897	20.525	248.448	266.051	186.213	159.592	38.7248	0.00035	1.80663
ENSG0000	KIAA0825	5:9415296	1.371	1.15135	0.236141	0.45024	0.493501	0.696092	4.91973	0.232704	0.609021	0.448215	0.30365	1.36636
ENSG0000	GTDC1	2:1439370	30.2636	11.6713	25.6671	32.2673	21.3692	32.1279	26.4795	31.0754	37.6613	33.2611	0.4653	0.333273
ENSG0000	RETN	19:766904	0.570019	2.02039	0.350821	0.912019	0.086424	30.6198	1.33621	6.06563	0	0	0.1391	3.28357
ENSG0000	TPBG	6:8236320	0.812482	0	1.27211	0.880932	0.45094	0	0	0.690064	0.088645	1.8122	0.85685	-0.35823
ENSG0000	CASP1P2	11:105041	0.638567	0.941495	0.476139	0.228813	0.390856	0.975314	1.39614	0.138475	0	0.494996	0.92995	0.132547
ENSG0000	SH2D3A	19:675215	29.4574	30.1435	15.879	23.6673	22.9414	9.3644	22.2248	15.8829	17.0393	22.0186	0.296	-0.49567
ENSG0000	XIAP	X:1238597	8.02659	2.20061	2.95366	3.85907	4.29803	1.53115	5.06905	3.93377	3.18277	7.32385	0.4966	-0.35825
ENSG0000	DRAP1	11:659192	149.47	130.715	158.038	131.887	228.074	296.302	429.183	246.01	147.084	128.64	0.1303	0.51539
ENSG0000	MAP3K14	17:451909	6.91578	10.8016	2.49977	2.20795	3.22372	6.09958	4.81795	9.04926	0.682803	5.79449	0.87495	0.173735
ENSG0000	RP11-552	17:758970	0.166789	0.39862	0.578036	0.327549	0.447706	1.49665	0.798975	0.465407	0.333233	0.432516	0.56955	0.575036
ENSG0000	ULK1	12:131894	3.29767	4.13585	1.66877	2.07536	3.24867	2.50465	4.63163	1.84802	0.05326	6.71992	0.71435	0.208238
ENSG0000	MBOAT1	6:2010214	4.20088	1.43442	1.67027	4.55215	4.44226	1.20155	0.985121	4.92958	2.6497	4.51264	0.75655	-0.20656
ENSG0000	SNIP1	1:3753444	2.81197	3.71498	3.70949	1.48663	2.91583	4.96396	3.36587	4.21808	1.46694	3.29318	0.9949	-0.00281
ENSG0000	RFC3	13:338180	4.86588	7.88566	3.57129	3.38294	2.12099	6.20504	7.98076	4.27997	2.22013	1.81743	0.82075	0.154602
ENSG0000	EIF3E	8:1082012	128.116	101.458	156.545	125.796	108.182	150.09	137.385	138.247	121.56	36.403	0.8245	-0.0693
ENSG0000	RPAP1	15:415171	10.1444	9.59703	6.44083	7.54365	8.00721	6.93335	5.42548	5.77673	5.53582	11.7636	0.65275	-0.17296
ENSG0000	SLCO2B1	11:750991	0	0	0	0.00718	0	15.8142	2.4065	0.201385	0.75932	0.046626	0.2232	11.0573
ENSG0000	PCBD2	5:1349049	5.5834	6.02958	7.03951	6.52076	5.45849	6.11692	9.21338	6.41901	4.40457	5.24199	0.97635	0.018544
ENSG0000	PCP2	19:762973	0.547636	1.79878	0.828224	0.138207	0.579803	0	0.780368	0.262364	0	0.526532	0.3893	-1.24215
ENSG0000	PELI2	14:561173	1.19839	1.82972	1.1269	0.908241	1.36318	0	0	0.779763	0.107901	0.506959	0.0868	-2.57563
ENSG0000	NRIR	2:6828513	0	0.168779	0.089757	0.17817	0	0	0.706204	1.01675	0.065789	0.41411	0.22555	2.39158
ENSG0000	SKP1	5:1341147	618.303	514.298	643.409	480.669	386.506	705.641	627.76	572.334	464.653	143.754	0.87545	-0.12375
ENSG0000	CTC-487M	5:1127074	0.882759	1.87329	3.45147	2.04694	4.38069	0.913901	3.93645	2.13893	0.327604	6.37381	0.97835	0.036854
ENSG0000	NR2C1	12:950202	23.2204	29.5345	8.32261	16.741	6.20237	19.258	19.691	9.50357	22.6647	12.2463	0.75625	0.11865
ENSG0000	TSPY26P	20:321864	0.391091	1.73719	0.929132	0.013271	0.915731	0	0	0.331471	0	0.507515	0.2956	-2.45398
ENSG0000	IL18BP	11:719985	8.25404	16.1236	11.9905	9.52028	24.1314	23.2554	2.23194	2.94282	8.81458	18.0969	0.6023	-0.41178
ENSG0000	SLTM	15:587711	87.5637	88.7115	77.6846	54.0569	105.94	109.528	100.22	73.5132	96.1616	152.823	0.63985	0.189607
ENSG0000	CLIP1	12:122271	26.0951	33.5857	14.6246	13.1323	24.5111	34.3698	42.6539	23.2855	24.7572	68.1709	0.26475	0.474791
ENSG0000	RP11-227	3:9849760	1.73228	1.32048	0	0.469989	0	1.09914	0.388344	0.041046	0	0.192061	0.66275	-0.87717
ENSG0000	TRAV4	14:217361	32.8722	31.6337	54.5235	55.6032	24.247	42.6887	39.0384	31.8071	45.7072	33.5302	0.9058	0.059094
ENSG0000	GNAI1	7:8013395	0.025811	5.80877	0	0	0.253833	0	0	0	0.012117	0	0.26435	-9.58512
ENSG0000	IRF8	16:858988	8.08044	18.3698	5.68779	14.9552	8.73194	34.7206	21.2655	19.0096	22.8677	9.50139	0.0317	0.916664
ENSG0000	ALDH2	12:111686	1.07816	9.46092	0.272378	2.01195	0.346519	45.5961	2.18477	4.49341	2.35063	0.648652	0.1152	2.05301
ENSG0000	MS4A1	11:604557	0.294601	88.5515	5.60204	29.5977	11.1491	0	0	2.75073	0.105382	3.68588	0.0144	-4.64886
ENSG0000	BRPF3	6:3614669	4.14346	4.58695	1.30664	1.60036	2.16074	1.83929	1.48538	1.82374	0.049839	4.45924	0.50345	-0.45508
ENSG0000	HIST2H2B	1:1498844	11.0007	16.4359	18.7811	17.979	9.50747	2.45476	9.73026	11.0271	16.8677	10.294	0.2644	-0.54377
ENSG0000	AAMDC	11:778211	8.83944	10.0332	10.0288	26.9528	13.2269	18.2223	9.91065	9.40039	6.96851	5.72594	0.62665	-0.50819
ENSG0000	XXbac-BP	6:3074292	1.48629	2.52933	3.45941	4.09214	2.66227	4.67044	2.17765	3.2755	9.19587	2.5717	0.65725	0.666578
ENSG0000	A1BG	19:583451	5.85998	5.4453	9.931	6.32719	4.24132	16.9845	19.3113	27.5321	18.497	4.22335	0.2961	1.38471
ENSG0000	CCDC82	11:963527	36.8755	42.3811	20.6001	19.2034	37.9728	36.5445	33.6346	29.2321	24.379	35.101	0.94255	0.04285
ENSG0000	DAB2	5:3928426	0	0.745587	0.331471	0.647249	0.493219	3.57049	0.136197	1.11724	0	0.149066	0.1214	1.27316
ENSG0000	PPT2-EGF	6:3214835	2.92414	1.44186	2.99569	3.58615	1.86822	1.47351	2.22811	2.35746	2.02738	1.1475	0.4459	-0.71367
ENSG0000	POC1A	3:5207525	2.18311	3.32515	0.672634	1.34329	1.87465	7.42012	1.52129	1.93564	2.83767	2.85323	0.3488	0.835261
ENSG0000	FIG4	6:1096913	7.91896	13.8067	9.46677	9.86282	4.29904	18.0985	6.32934	4.40302	2.85412	5.64172	0.65115	-0.20534
ENSG0000	CDS2	20:512678	11.685	9.40455	14.5917	8.35428	9.17874	10.0977	17.2567	17.4524	10.8571	12.9702	0.91765	0.044447
ENSG0000	C16orf58	16:314830	30.7498	30.4012	7.01236	13.7247	7.503	18.3706	14.9196	10.1168	13.0951	12.3774	0.35135	-0.38395
ENSG0000	SUMO1	2:2022061	176.288	169.497	189.963	195.014	122.508	202.438	177.056	145.167	151.643	52.5134	0.40625	-0.26647
ENSG0000	MRPL19	2:7564678	16.2726	12.2725	16.8008	11.9018	14.4459	21.3817	21.5394	13.3436	15.5829	8.13341	0.94095	-0.04685
ENSG0000	CTB-109A	5:1572601	0.273	0.095058	0	0.447801	0.386921	0	0.902812	0.763682	0.057863	0.898592	0.4539	1.09819
ENSG0000	PCBP4	3:5195531	8.16768	4.43253	5.74302	9.42127	8.22087	10.0651	14.3524	8.61706	14.7308	14.3245	0.405	0.735055
ENSG0000	VSIG1	X:1080449	1.36452	2.80454	0.738987	0.121748	0.307172	1.10665	0	0.10366	0	0.588198	0.18065	-1.49323
ENSG0000	KCTD20	6:3635409	17.7676	16.4843	7.1895	8.86744	6.0696	15.4452	6.24136	10.2668	3.5807	11.3178	0.3066	-0.43245
ENSG0000	POMZP3	7:7654935	3.54819	2.97732	3.57406	3.93577	3.62447	7.28372	3.23411	5.47893	3.19273	4.00974	0.68075	0.328463
ENSG0000	AKT1	14:104769	100.87	96.5908	22.5293	6.3607	25.7749	108.588	150.724	26.837	11.7838	46.2015	0.49585	0.260657
ENSG0000	HK2	2:7483265	3.2727	2.12685	1.32665	0.219208	0.511389	7.64075	0.54777	1.82863	0	0.895025	0.5521	0.482723
ENSG0000	LINC0161	12:919849	8.04151	6.47621	5.91648	6.58589	2.09359	2.07476	2.93623	3.207	7.57738	4.3166	0.46695	-0.604
ENSG0000	MTMR14	3:9649432	34.7443	42.2803	57.5656	62.6946	47.8667	30.4826	18.0074	47.8661	35.6528	45.2796	0.1648	-0.48227
ENSG0000	GCC1	7:1275806	13.0973	10.8363	6.87754	3.6088	6.53183	6.31497	7.17473	9.20509	0.455503	7.79754	0.7773	-0.45739
ENSG0000	LARP4B	10:806913	10.9472	12.2064	4.84528	3.39979	5.58313	5.70379	8.33336	7.20735	7.53648	13.2677	0.865	0.060672
ENSG0000	GRWD1	19:484457	13.2836	16.1087	29.7946	18.4218	26.3681	13.5187	14.1844	15.5697	7.69494	17.718	0.17775	-0.91208
ENSG0000	DSE	6:1162541	1.00074	2.24535	0.220847	1.29028	0.314705	5.13558	0.877392	2.45898	1.43615	3.79485	0.5054	1.1099
ENSG0000	SYS1-DBN	20:453619	0.630513	0	2.28098	2.73372	0.807555	0	0	1.35608	0	0	0.2665	-2.39771
ENSG0000	ARHGAP3	11:128965	0.857132	2.95154	0.348972	0.025485	1.5613	0	0	0	0.314972	0.362839	0.1162	-3.29521
ENSG0000	RP11-493	12:473537	0.298386	0.287001	0	0	0	0.899577	5.22589	0	0	0	0.39965	3.423
ENSG0000	NANOS3	19:138620	0.635103	0	0.375577	0	0	0	2.37119	0.49152	0	0.379644	0.41615	1.5728
ENSG0000	BCL11B	14:991692	6.24804	12.5462	15.3049	6.82851	17.4882	9.29265	7.71062	14.2471	10.6414	33.213	0.67355	0.16052
ENSG0000	ZER1	9:1287244	18.2589	25.9292	10.0146	13.2058	7.10114	6.94047	13.3487	6.68632	6.51173	11.4362	0.02015	-0.93409
ENSG0000	RP11-311	16:695650	2.93278	4.89378	3.67035	0.102448	4.78728	5.9314	3.16838	4.55922	1.35285	14.6497	0.66935	0.58751
ENSG0000	RP11-73M	14:103553	0.920558	0.56561	0.291312	0.894812	0.234415	2.58082	0	1.71118	0	0.925648	0.51195	0.946918
ENSG0000	RALGPS1	9:1269147	1.52891	2.93343	6.38518	2.76264	4.15696	5.09226	7.8367	3.94714	6.11212	8.95908	0.1833	0.847156
ENSG0000	MIR222HG	X:4574521	0.677533	0.876882	0.423368	0.167377	0	3.38125	1.40746	2.55737	0.059169	2.94596	0.0666	2.34251
ENSG0000	RP11-266	3:9749943	0.388368	2.50891	0.898465	1.95311	0.440484	0	0	0.324721	0.408956	0.18717	0.1911	-2.2786
ENSG0000	RP11-162	9:1213389	0.361196	0.600754	0.092315	0.635782	0	0.649576	0	1.43645	0	0.230231	0.72835	0.653126
ENSG0000	EEPD1	7:3615314	9.46823	17.844	7.90243	13.6268	3.57597	18.974	6.71845	9.76798	4.19588	1.46504	0.6846	-0.34381
ENSG0000	RPS3AP47	15:431062	0.615739	0.660525	1.3153	0.766353	0.210055	0.756087	0.378648	1.04479	0.17762	0	0.7384	-0.43969
ENSG0000	AFF1	4:8687620	11.4944	19.0521	3.92341	8.79205	10.7351	18.7133	21.005	5.36842	20.6503	18.7184	0.2652	0.686056
ENSG0000	STIM1	11:385431	11.4191	13.1245	19.3636	30.9566	32.2004	3.65032	5.7656	26.8283	24.7027	55.2662	0.7622	0.09988
ENSG0000	USP25	21:157300	31.6202	32.1767	6.86959	6.82248	7.85345	19.0997	20.919	10.3236	20.5759	15.2586	0.82825	0.082977
ENSG0000	SUOX	12:559971	1.09252	2.80307	0.687146	0.93411	0.329694	7.14683	4.31988	1.27503	0	1.35212	0.24395	1.07345
ENSG0000	DMC1	22:385189	1.78381	1.25544	0.864348	0.352181	0.586135	0	0.449579	1.1262	0	0.468868	0.19015	-2.56039
ENSG0000	SDCCAG3	9:1364019	8.41004	10.2115	4.00916	2.43184	6.99466	14.779	6.63336	8.54678	3.31632	13.0775	0.4024	0.616785
ENSG0000	ZDHHC17	12:767635	10.1521	10.8401	4.86671	4.07706	3.65442	4.70022	6.90085	4.78543	3.86597	7.34514	0.40915	-0.34718
ENSG0000	SYVN1	11:651206	1.933	1.68303	3.89852	4.10477	7.47934	2.11664	2.05022	3.32674	6.26484	11.0267	0.97485	0.030492

ENSG0000	CWF19L2	11:107326	10.696	16.5349	8.94878	7.75374	12.6017	12.4098	13.6773	11.1227	5.37546	16.4606	0.88335	0.056768
ENSG0000	PPP1CB	2:2875163	22.9559	25.0461	49.9088	24.4813	29.4927	16.6167	34.9677	68.8234	44.2674	52.596	0.19545	0.488095
ENSG0000	AKAP5	14:643880	0.119087	0	0.44262	0.388473	0.092078	1.15632	4.39816	0.676687	0.660954	0.670128	0.0334	2.91593
ENSG0000	AC099850	17:587558	0.89762	1.48185	0.777574	1.22776	0.223055	0	0	0.766281	0	1.09113	0.52145	-1.17798
ENSG0000	AIMP2	7:6009244	21.7488	22.8771	31.1981	39.3109	31.2909	37.5044	21.8684	24.1331	48.7395	11.0843	0.9586	-0.05362
ENSG0000	CLMP	11:123069	0.083415	0.345199	0.076366	0	0	1.86241	0.319199	0.071214	0.221958	0.103385	0.04455	2.88793
ENSG0000	JCHAIN	4:7062874	0	87.9943	2.60384	500.682	21.7849	0	1.17167	0.424203	1806.45	5.45152	0.26655	1.56477
ENSG0000	AC017002	2:1111959	1.24001	0.954415	0.176361	0	0.030054	19.702	40.7786	6.13728	8.65894	1.51243	0.03595	5.19906
ENSG0000	CTD-2373	5:9040999	0.806423	0.453603	0.872916	0.413807	0	0	0	0	0	0.818207	0.4959	-1.17465
ENSG0000	ENO1-AS:	1:8861001	1.05012	0.92911	0.400299	1.58762	0.491763	1.27186	0.963923	0.329971	1.51788	0.126458	0.95455	-0.06748
ENSG0000	ZNF34	8:1447731	4.34943	8.98097	5.71178	9.07668	4.91295	3.52564	3.45779	3.84239	13.6248	4.5999	0.80085	-0.13445
ENSG0000	RNF34	12:121400	66.8427	73.1947	81.4642	62.6731	66.1533	72.7579	80.2688	58.9416	63.6079	73.1295	0.86815	0.060601
ENSG0000	KCTD17	22:370517	8.88638	8.26165	1.24142	3.83834	3.93214	17.8209	14.1711	4.9754	4.22458	5.41487	0.15695	0.814254
ENSG0000	DNAJC9-/	10:731103	0.984656	2.1258	0.25844	0.996626	0.19996	1.97642	0.110885	0.500104	0.77098	0.496095	0.45435	1.00204
ENSG0000	EIF2AK1	7:6009244	56.8865	55.2727	37.1411	28.8178	31.0773	38.3506	39.1039	23.1873	21.9603	29.388	0.03935	-0.712
ENSG0000	ARL8B	3:5122219	24.3163	21.7408	13.9519	20.1979	13.4789	46.7019	34.215	20.1743	24.8448	15.3732	0.1729	0.583241
ENSG0000	SEMA6A	5:1164436	1.70524	1.99696	0.496817	2.06372	0.486181	2.71799	0.278865	0.189697	3.00124	0.544668	0.9179	0.096409
ENSG0000	S100Z	5:7685000	1.82212	3.03897	0.130841	0.393243	0.077044	3.48145	0.89273	0.591357	0	0.585504	0.98725	0.017656
ENSG0000	MYO1F	19:852078	311.294	167.734	206.242	228.703	198.042	291.176	148.359	122.152	118.683	222.769	0.28445	-0.44268
ENSG0000	NTHL1	16:203981	16.2862	13.7174	20.5119	21.2946	24.1755	9.01637	12.4535	9.66614	12.3436	6.71766	0.33375	-0.97846
ENSG0000	ATP6V1C1	8:1030201	17.7892	17.7541	9.3808	9.8596	10.1054	39.9069	16.3337	16.9865	15.6375	9.12385	0.31455	0.445945
ENSG0000	FAM193B	5:1775197	27.3727	43.1813	10.4005	6.53149	13.0506	32.8098	32.17	17.4033	13.6838	30.3078	0.23245	0.521366
ENSG0000	VAV3	1:1075711	7.8161	8.38086	2.2805	1.31494	3.01265	5.90394	7.72723	1.306	4.94259	3.96408	0.6736	0.213741
ENSG0000	HSD17B12	11:435564	24.19	18.2366	5.88174	10.0147	9.62725	24.1008	20.7898	8.66782	13.5388	16.2812	0.45575	0.297261
ENSG0000	LLNLR-24!	19:582285	2.23209	2.53954	2.87731	2.22982	1.69855	3.38027	4.26463	1.91371	2.75885	1.61141	0.88685	0.240269
ENSG0000	CYP1B1	2:3792318	0.100476	2.52002	0	0	0.118227	1.58026	0	0	0	0.037387	0.70045	-0.55528
ENSG0000	ENTPD4	8:2329718	22.0171	39.1054	21.6839	23.2994	21.0591	17.0745	17.3323	23.4185	22.9737	41.0541	0.5797	-0.22402
ENSG0000	CAMKV	3:4985798	0	0	0	0	0.034594	2.94045	2.39814	0	0	0	0.2903	6.82536
ENSG0000	HEXDC	17:823892	51.7727	70.4439	14.6942	10.3793	15.1147	76.0035	81.2784	31.3468	19.4337	31.0777	0.3802	0.530901
ENSG0000	BIRC2	11:102347	54.3733	48.7133	20.9171	28.5885	11.0119	34.5199	36.0385	35.4276	30.9366	25.5948	0.63535	0.155672
ENSG0000	ASMTL-AS	X:1401768	1.02415	2.85106	0.70337	2.61252	1.05079	2.67242	2.87169	0.846855	2.33266	0.515568	0.8283	0.185663
ENSG0000	MRPL12	17:817033	39.1097	25.3492	62.4004	54.9418	84.3225	47.0069	48.8691	72.7515	40.1236	20.7823	0.453	-0.32563
ENSG0000	CTBP1	4:1210119	99.0899	67.8918	68.7752	53.0205	149.485	69.1007	70.5595	75.9191	46.9811	147.636	0.5646	-0.2115
ENSG0000	ZNF770	15:349783	12.8469	14.6892	68.0172	51.7799	101.259	14.3034	14.6303	40.2535	39.1002	82.833	0.46845	-0.66276
ENSG0000	SOCS3	17:783567	39.6532	16.5522	9.59383	23.4839	14.1612	15.6657	33.62	8.71489	9.77355	19.663	0.57485	-0.22362
ENSG0000	CCDC40	17:800366	0	0	0	0	0.228971	0	1.50947	0.3257	2.56877	0	0.2374	3.98162
ENSG0000	PPCS	1:4243032	145.737	101.311	169.027	163.979	118.134	222.736	139.516	131.479	150.185	104.883	0.8071	0.086234
ENSG0000	MRPS14	1:1749991	41.7801	35.8997	58.5939	43.7713	40.2708	52.4748	57.3768	51.3999	45.5182	15.6643	0.9557	-0.07118
ENSG0000	GOT1	10:993968	17.0654	19.501	22.8692	21.0384	20.6022	18.4498	19.3083	16.2852	10.3314	13.1975	0.30255	-0.41182
ENSG0000	ZMPSTE24	1:4025810	28.9685	20.1305	12.1216	17.3471	11.1968	35.6637	28.903	15.3931	19.502	22.1534	0.2496	0.408867
ENSG0000	GALNS	16:888137	12.6677	13.7275	10.1035	14.7604	9.34762	15.4483	14.3944	8.55801	2.61744	12.1036	0.6464	-0.31801
ENSG0000	SH3PXD2A	10:103594	0.287511	0.479762	0.155619	0	0.122325	0.92151	1.0491	0.12153	0.814659	0.704239	0.2091	1.93309
ENSG0000	RP11-166I	17:570782	0.272643	1.90048	1.89125	1.48438	0.234576	1.68789	0.072799	0	2.64467	0.190996	0.83665	-0.2824
ENSG0000	PPIAP29	6:2479732	1.41004	1.36107	0	0.998785	0.596511	0.293495	1.17568	0	0.42911	0	0.291	-1.6946
ENSG0000	RAD23B	9:1072831	5.96012	4.8344	4.62882	14.4546	4.30481	6.37322	4.08953	2.14987	19.049	5.04388	0.82865	0.096277
ENSG0000	DBF4	7:8783356	21.5348	18.9614	12.4543	15.3489	19.8171	19.7704	22.4993	24.5855	15.748	15.7734	0.99305	-0.00369
ENSG0000	MBNL1	3:1522438	145.629	130.829	64.7521	122.038	83.7129	68.102	77.9039	59.3938	119.434	86.57	0.3422	-0.44713
ENSG0000	UTP18	17:512605	46.6835	37.8161	33.9256	34.0644	41.2758	48.9388	48.8367	29.2389	21.0118	26.9034	0.6656	-0.14624
ENSG0000	KIFC2	8:1444660	6.75445	4.49784	1.54539	3.56088	3.527	4.59428	6.76685	3.32951	3.59594	4.22639	0.70395	0.203408
ENSG0000	RP5-997D	1:3471273	0	0.814195	0.348393	0	0.107364	0	0.048238	1.08857	0	1.48425	0.61555	1.28027
ENSG0000	GFM2	5:7462740	19.2235	14.4394	6.04714	7.94169	4.68151	16.1432	13.2447	8.61499	13.9332	10.5758	0.6585	0.468065
ENSG0000	RP11-180I	12:127150	2.96768	3.28151	3.08341	2.66116	1.05862	4.74879	0.772834	3.32823	8.06476	2.04213	0.7236	0.649986
ENSG0000	CENPB	20:378385	1.25182	0.955244	0.688893	0.191023	1.78283	0.748073	1.58939	2.05604	0.028356	3.64885	0.1871	0.789483
ENSG0000	FNBP1P1	2:7412067	0.680587	0.917424	0.324119	0.703376	0.27995	0	0.887624	0.377657	0	0.396043	0.71265	-0.78268
ENSG0000	LYRM2	6:8943316	20.0601	20.9414	37.7031	24.9865	30.1748	36.2715	41.1466	45.1019	29.4407	42.5205	0.48715	0.395341
ENSG0000	CPSF3L	1:1292375	56.4612	76.7402	74.4938	86.2853	65.9208	57.9175	64.0735	65.8305	49.1786	69.8967	0.40265	-0.26115
ENSG0000	VSTM4	10:486848	0.226768	0.254498	0.045332	4.23176	0.048073	1.61274	2.11323	0	7.19335	0.079105	0.27835	1.14574
ENSG0000	CXCL2	4:7409703	1.29574	1.02607	0.428637	0.845535	1.31941	4.67332	0	1.50974	15.1414	3.39408	0.11575	2.27333
ENSG0000	IGLVI-70	22:219910	0	0	0	10.8654	0	0	0	0	0	0	0.11665	#NAME?
ENSG0000	ELAVL1	19:795857	18.0182	24.126	13.7498	7.66851	26.7225	16.082	20.9168	23.5741	6.6813	36.785	0.6605	-0.2095
ENSG0000	RP1-168L1	6:1663831	0.544756	0.432481	0	0.331141	0.211015	1.85024	0.160604	0	0.766806	0.092456	0.60485	0.919413
ENSG0000	SNHG11	20:384465	11.5526	18.339	16.6326	24.4653	9.76172	14.8267	8.91333	6.7646	15.7639	14.9079	0.4745	-0.34231
ENSG0000	MPLKIP	7:4012602	6.85599	5.50329	9.13016	7.23195	6.607	7.3569	7.21892	6.33092	6.04233	2.73553	0.37625	-0.30661
ENSG0000	SCARNA2	1:1091001	7.07973	1.38305	1.18273	0.583634	0.000509	4.75634	6.88748	3.01187	0	1.46953	0.8443	0.546907
ENSG0000	AP000253	21:316535	0.072268	1.32149	1.34172	0.131888	0	0	0.833136	0	0.341234	0.523741	0.6916	-0.75993
ENSG0000	NCBP3	17:380216	9.13394	9.39723	8.3319	4.21212	7.59026	6.40568	10.0859	9.62274	6.65159	11.6807	0.89395	-0.06261
ENSG0000	TOB1-AS1	17:508622	7.26801	4.25652	4.99484	2.44948	5.09355	2.46807	3.30179	2.19607	1.38586	2.38821	0.4093	-1.083
ENSG0000	RP11-567I	2:1478445	0.156505	0.575509	0.550471	0	0.093547	0	1.74118	0.275326	0.565585	1.19192	0.3309	1.59011
ENSG0000	TRBV6-1	7:1423282	37.3714	46.6749	159.695	79.9425	91.3086	48.2988	32.1859	90.8165	89.6228	69.2939	0.57715	-0.22808
ENSG0000	ARID1A	1:2669213	20.1878	22.2545	13.8035	5.20211	30.1981	17.6683	20.8725	16.4038	10.1749	44.8025	0.7313	0.149364
ENSG0000	VDAC1	5:1339719	71.1447	62.9855	60.4035	56.5352	68.9929	146.206	84.3453	91.3791	74.756	51.6587	0.1631	0.451337
ENSG0000	DPP7	9:1371105	328.611	336.31	184.931	176.86	184.012	460.688	447.861	235.997	175.208	184.741	0.49695	0.231304
ENSG0000	LLGL2	17:755250	45.0481	31.2856	24.3206	15.0676	40.491	45.2965	41.8018	20.0709	15.4896	66.5733	0.60965	0.217303
ENSG0000	COQ6	14:738518	17.3473	21.5473	22.9727	28.4197	16.1805	21.847	18.3877	23.652	30.2563	16.5809	0.8939	0.075956
ENSG0000	SNAPC3	9:1542270	13.5744	18.135	9.3435	7.61945	6.68504	10.5463	17.6917	8.54096	10.8455	8.73248	0.85075	-0.16362
ENSG0000	HSPA8	11:123057	751.56	818.886	655.624	598.605	1046.88	679.054	1008.86	801.043	716.89	882.686	0.8078	0.106514
ENSG0000	GTF3C2	2:2732584	30.8418	28.7689	20.4032	29.1275	15.3978	5.96042	21.3203	18.9414	13.4208	18.5146	0.1659	-0.67687
ENSG0000	ERICH1	8:614745-	32.2386	49.4939	40.6324	40.6635	59.0326	58.8736	70.0296	32.1759	26.1724	48.51	0.85825	-0.06831
ENSG0000	RP11-542I	16:859352	6.27152	3.51084	2.53405	5.78676	1.90454	5.99024	7.17198	4.7484	0	6.0119	0.767	0.213131
ENSG0000	MAP4K2	11:647849	18.7039	35.8867	5.81346	8.23135	9.47628	5.10882	14.3553	7.89974	5.6633	11.2394	0.05055	-0.87157
ENSG0000	GPX4	19:110392	144.208	156.277	179.846	130.75	129.634	368.635	254.7	238.488	160.501	27.0906	0.22535	0.405907
ENSG0000	SWT1	1:1851570	2.94503	6.06136	1.38334	1.12354	0.942614	1.90466	2.49826	2.28203	1.79107	1.68211	0.62975	-0.27129
ENSG0000	CMTR2	16:712813	26.6814	19.3526	9.18307	6.86268	10.0524	12.3282	8.4432	11.0283	2.42111	19.4838	0.1826	-0.50572
ENSG0000	MPV17L2	19:181931	9.99247	6.70861	16.0794	17.8865	16.76	13.1082	7.61153	15.169	0	14.6966	0.42195	-0.56458
ENSG0000	ACOT13	6:2466703	7.18751	5.56513	9.35792	6.13998	7.46457	14.6033	7.33439	9.00904	10.0159	3.59542	0.78665	0.319899
ENSG0000	GNS	12:647134	15.5906	20.7243	9.31435	8.17566	21.8682	25.337	6.11033	9.40969	8.22546	27.4574	0.69565	-0.17269
ENSG0000	ZNF791	19:123791	10.3412	10.6246	11.7845	8.99356	5.59344	14.152	12.9464	15.9664	12.5727	30.7485	0.1729	0.599505
ENSG0000	LIPE	19:423971	2.18261	1.69279	0.559909	2.07736	0.906321	0.106768	0.75199	0.2166	0.240573	0.506002	0.0051	-1.94727
ENSG0000	EEF1A1P3	13:281385	0.984893	1.49064	0.453136	0.755824	0.379312	0	3.76799	0	0	4.01397	0.6436	0.922175
ENSG0000	MACF1	1:3908131	87.2303	80.7794	24.0225	30.2715	65.8435	78.857	56.3743	23.1898	42.7924	92.5004	0.9203	0.049182
ENSG0000	NXPH4	12:572167	0.162356	0.513298	0	0	0	2.43199	0.448075	0.283529	0.206963	2.12949	0.2828	3.2719

ENSG0000	PPFIBP2	11:751329	0.646085	3.05994	0.203173	0.024631	0.366746	2.16566	0	0	3.95016	0.299435	0.67065	0.397162
ENSG0000	SENP2	3:1855824	6.47985	3.49352	2.51892	6.90463	4.1664	3.53739	6.51916	6.67123	4.21633	10.6301	0.7784	0.134492
ENSG0000	SLC9B2	4:1030198	14.3638	3.58121	8.75903	13.1761	6.95439	6.92769	10.075	16.5731	5.62997	3.90538	0.74565	-0.20428
ENSG0000	ABCA2	9:1370072	10.0182	4.50399	2.5423	4.54452	9.537	12.2778	21.4593	2.70605	5.04547	12.7563	0.3339	0.689902
ENSG0000	PPP4R1	18:954679	15.4203	14.7433	5.57437	8.78398	5.41082	7.44864	7.19929	7.84595	1.83752	9.16493	0.50165	-0.27434
ENSG0000	INAFM1	19:472744	3.94525	3.6835	7.1599	4.3845	7.32528	10.4647	5.84792	6.52337	1.743	3.24102	0.8743	-0.12112
ENSG0000	RAD54L2	3:5154114	4.50828	4.73667	2.07092	3.36071	3.7107	2.59785	4.88671	1.85593	3.61678	8.87616	0.9043	-0.07824
ENSG0000	ARID5B	10:619012	10.9041	24.7775	7.65818	6.40068	9.55027	29.0108	28.3642	14.7455	17.1913	25.1311	0.02775	0.953983
ENSG0000	CACNA1C	12:194605	2.25714	1.2694	0.748524	0.738157	0.338047	0	0.087307	0.889801	0	0.029656	0.24395	-2.21207
ENSG0000	CCDC58	3:1223595	12.6772	10.4926	2.03021	5.24849	7.47806	19.2864	14.2031	4.27432	6.2373	3.42497	0.6358	0.361032
ENSG0000	ACOD1	13:769484	0	0	0.016403	0	0.250492	0.6948	0.015799	2.13045	2.04971	0.674916	0.20945	4.38664
ENSG0000	RP11-6F2.	3:1570816	0.911175	0.119193	0.000139	0	0.067204	1.07949	0.421258	0	0	0.191949	0.9383	0.139537
ENSG0000	RP11-23J1	12:470757	12.28	13.7717	25.8509	40.9481	18.7633	7.39886	8.92696	15.5936	10.256	6.07126	0.27005	-1.11732
ENSG0000	RP11-148I	7:1510284	0.517327	0.938598	0.264312	0.778524	0.362328	1.64112	1.57056	0.586611	0.245132	0.455787	0.6062	0.664044
ENSG0000	ZNF320	19:528618	3.4738	1.59786	1.5447	2.37465	1.60595	2.64645	0.836558	1.49283	1.56366	2.57576	0.88405	-0.10166
ENSG0000	NRP1	10:331774	0	0.476679	0	0	0	1.80234	1.38167	0	0	0	0.3623	2.58968
ENSG0000	DHRS7B	17:211233	8.48055	14.376	12.2758	10.1223	7.8853	31.6031	13.5355	6.39327	23.9832	4.65796	0.2835	0.582356
ENSG0000	RP11-370I	12:481983	2.38815	1.29024	1.70597	0.337941	0.832746	0.79653	1.73001	1.60513	2.18168	3.4186	0.4106	0.564824
ENSG0000	RP11-18H	3:1200948	0.82573	0.517454	0.327617	0	0.292015	2.4136	0	0.76196	0	0.919698	0.45715	1.13131
ENSG0000	ARCN1	11:118436	67.5609	81.4008	30.7746	24.4169	29.5589	30.9366	45.7438	42.1633	31.1627	42.528	0.8444	-0.10113
ENSG0000	STAG3L3	7:7296969	22.7319	50.6328	32.6695	28.4813	16.4139	26.7342	39.9538	26.1364	48.1959	59.6495	0.50355	0.422583
ENSG0000	RP11-278	12:326791	0.454289	0.04427	0.355841	0	0.543438	1.43173	0.234675	0.943431	0	0.829757	0.4638	1.2394
ENSG0000	ZNF559	19:932377	25.8232	30.2811	16.5712	30.318	8.88112	17.4068	7.51028	8.60763	7.73434	13.744	0.0232	-0.94058
ENSG0000	MSRB2	10:230955	12.0155	6.9052	6.48671	4.33395	8.80936	22.646	1.76582	4.6177	10.7035	2.95111	0.95455	0.031843
ENSG0000	PIP5K1A	1:1511979	7.72491	8.00228	7.33693	13.2042	4.40274	4.45338	5.81799	10.8084	3.62345	8.51529	0.56345	-0.27778
ENSG0000	FAM209A	20:564685	0.858492	1.41643	1.00274	0.350693	0.109996	0	1.40743	1.79791	0	0.935048	0.8949	0.173924
ENSG0000	NKIRAS2	17:419667	18.4661	21.7457	27.0517	32.2271	33.0662	19.9093	20.3954	33.3129	21.414	29.2574	0.7945	-0.16051
ENSG0000	CEP120	5:1233448	13.8592	8.96113	2.06906	2.00924	2.79444	5.7448	7.34949	2.50003	2.14465	4.80682	0.34565	-0.41072
ENSG0000	CSTF1	20:563923	25.2849	30.0197	29.8097	22.4769	21.4224	31.3435	30.8594	24.7093	3.0143	28.7214	0.35485	-0.36393
ENSG0000	LOXL1-AS	15:739080	5.79915	2.97067	12.6139	11.9073	15.7058	7.1073	7.08146	6.70381	2.92157	11.1012	0.25585	-0.72451
ENSG0000	SMIM3	5:1507779	2.89975	4.57166	4.54883	14.0207	9.43597	6.38728	7.27081	3.16095	7.87478	3.64315	0.4129	-0.39002
ENSG0000	PDCD1LG2	9:5510569	0.476924	1.47728	0.334325	0.481883	0.181144	2.90342	1.6732	1.45413	3.24417	0.355216	0.33005	1.82599
ENSG0000	PPIB	15:641517	546.097	340.499	671.228	670.302	488.62	551.622	736.467	632.507	552.7	200.522	0.82015	-0.08422
ENSG0000	RHOC	1:1127011	51.6151	16.6641	77.7155	66.6632	108.558	72.9835	84.1269	84.7112	78.1113	39.5022	0.7807	0.100882
ENSG0000	PDPR	16:701136	3.36561	2.55615	1.88359	1.73467	1.98233	2.33781	2.3027	3.10707	1.45512	5.65407	0.6723	0.176745
ENSG0000	GDPD3	16:301048	1.44353	1.72876	1.09614	1.09529	1.52081	6.55238	2.69806	1.53084	0.705719	3.73242	0.30635	1.0908
ENSG0000	PI16	6:3694826	1.85279	8.60136	1.00078	3.78159	0.096265	1.80843	0	0.441156	21.894	0.181369	0.4079	1.06179
ENSG0000	TEX2	17:641472	3.78996	1.37238	1.24168	3.80592	1.80103	0.78926	1.78455	2.37026	2.34545	2.30744	0.6863	-0.26041
ENSG0000	STAMBP	2:7382891	25.1264	23.5701	23.673	15.0704	25.4834	30.5877	49.3962	26.4549	24.5364	23.066	0.4687	0.297588
ENSG0000	TRDMT1	10:171422	5.9473	9.02246	1.80426	3.95414	2.99294	9.77404	5.01834	4.12614	3.73131	3.26852	0.902	0.073213
ENSG0000	SNX11	17:481033	13.228	14.1563	17.1319	16.1095	11.6	18.2361	11.4711	18.7059	17.4349	16.5235	0.72385	0.130107
ENSG0000	MKS1	17:582054	8.89518	14.1651	13.0956	9.54456	6.51707	10.6179	0.039844	3.62086	9.53245	7.81113	0.22975	-0.70647
ENSG0000	YTHDC2	5:1135136	12.8246	16.7258	8.91743	5.50519	9.8943	16.841	11.38	8.34545	9.41792	27.3651	0.5842	0.282678
ENSG0000	GUSBP11	22:236084	11.2326	23.0495	6.82285	6.01854	4.89313	7.61872	14.8154	5.36687	3.61604	9.63575	0.878	-0.12579
ENSG0000	VPS18	15:404894	12.148	11.143	6.46165	7.11535	7.14851	9.89193	15.6727	7.15717	0	8.50295	0.64745	-0.23957
ENSG0000	UBE2G1	17:426925	36.2156	35.755	38.157	34.4841	38.795	52.7202	32.0126	34.3226	25.8841	33.1966	0.62055	-0.20023
ENSG0000	MED15	22:204959	39.6517	86.0279	16.9314	19.2343	20.536	37.4525	45.5534	21.4825	16.3482	33.6719	0.56685	-0.19094
ENSG0000	TOP3A	17:182714	9.09599	10.2625	5.32712	5.4447	4.95882	6.32968	8.70923	5.6195	3.08419	11.0707	0.7369	-0.154
ENSG0000	BTF3	5:7349840	366.806	330.362	436.011	373.483	339.708	426.271	392.686	415.162	343.043	98.6022	0.7176	-0.12714
ENSG0000	VPS16	20:283531	25.0898	26.9634	21.9755	28.6154	22.9252	31.1255	29.6124	23.9433	9.28579	22.6858	0.88355	-0.07481
ENSG0000	DIMT1	5:6230616	21.7998	21.3604	20.8524	20.8356	16.6207	30.9715	20.1837	14.8741	13.8878	15.0327	0.7978	-0.14088
ENSG0000	BICD2	9:9271136	12.1349	11.5651	4.33511	2.83062	5.07366	6.57455	3.6017	4.45812	0.519685	8.08444	0.15285	-0.66545
ENSG0000	RP11-629	8:1335731	0.565162	1.64685	0.840916	0.91189	0.718154	0	2.23597	1.86615	0	1.17925	0.9042	0.224756
ENSG0000	ZNF106	15:424128	34.6635	37.1458	14.3887	10.9458	22.1797	35.1989	40.0948	13.5252	8.78266	41.0153	0.93095	0.057517
ENSG0000	PRR7	5:1774021	12.0495	11.2369	15.8552	31.8455	18.3305	2.1173	9.26056	11.4738	6.22919	7.74629	0.25725	-1.32512
ENSG0000	HMGB1P5	3:2238181	96.5415	86.3134	77.2772	84.2425	83.7621	114.789	134.167	75.9891	78.1465	54.885	0.73475	0.173274
ENSG0000	ATXN2	12:111452	20.8708	30.8533	7.66957	7.07511	10.4981	8.38845	8.59422	9.06231	11.7621	20.459	0.3855	-0.31439
ENSG0000	CWF19L1	10:100229	20.5506	15.0816	13.3647	19.6862	15.855	15.6075	20.019	14.3547	4.00015	18.6681	0.30425	-0.42147
ENSG0000	PIK3C2A	11:170743	6.21817	6.187	2.00853	2.07099	1.6203	2.58026	3.66388	3.28483	2.52625	3.75206	0.98985	0.01321
ENSG0000	RRN3P3	16:223459	6.73616	10.473	2.88991	2.55071	2.93397	8.27646	9.35713	2.1005	2.26706	5.11194	0.8576	0.209458
ENSG0000	OSGEPL1	2:1896742	11.482	11.3505	12.5026	12.5542	10.7559	6.64309	7.41635	9.61407	13.8969	8.59569	0.54055	-0.34055
ENSG0000	ZNF512	2:2758296	19.1068	29.5965	10.7651	8.18841	6.73564	15.5961	13.7883	13.9761	12.229	18.9045	0.921	0.071325
ENSG0000	RPA2	1:2789152	141.471	118.587	214.943	184.171	158.855	90.0964	102.819	119.565	112.948	56.8383	0.0169	-0.78963
ENSG0000	SEC61G	7:5475224	166.115	99.2707	220.864	180.304	72.2638	213.881	200.826	168.089	133.514	20.2643	0.89375	-0.0491
ENSG0000	PON2	7:9540486	6.42598	4.45153	6.99747	3.21591	2.39765	4.38759	4.67284	8.49068	3.96603	4.13238	0.7414	0.171691
ENSG0000	MALT1	18:586700	18.5107	22.497	5.63546	10.1719	5.60723	10.5635	13.4138	8.48359	7.22078	10.7606	0.36885	-0.37761
ENSG0000	TES	7:1162092	129.407	128.151	117.279	131.558	125.01	72.095	54.0078	56.4392	66.0246	105.975	0.0044	-0.88139
ENSG0000	AC068831	15:909022	10.0994	8.76045	3.26298	5.19052	4.05255	15.3395	9.96388	6.04634	8.09119	4.41645	0.48625	1.19282
ENSG0000	PTPN9	15:754632	17.8113	11.2864	7.99088	11.5969	8.73518	14.705	12.2361	13.6051	9.9037	17.5872	0.7565	0.145762
ENSG0000	PDS5B	13:325864	12.9883	12.7934	5.09767	5.54138	7.46746	6.26615	7.10711	8.81549	10.6259	17.4207	0.74915	0.132214
ENSG0000	ACTG1P1	3:1393898	0.07142	0	0.032147	0	0.073649	1.2768	0	0	1.50831	0	0.2553	4.1637
ENSG0000	YWHAZP3	10:231369	7.00052	3.35315	0.337022	0.596475	0.1398	4.84115	3.15153	0.323131	0.372766	0.251384	0.8773	-0.29723
ENSG0000	RP11-368I	16:295951	0.506228	0.172994	0.058283	0.139368	0.056367	0	1.81769	0.791142	0.140806	0.209164	0.35575	1.53689
ENSG0000	CTRL	16:678932	1.8151	2.71476	1.31554	1.18682	0.832686	1.10464	0.664009	0.998331	0.689213	0.781255	0.4594	-0.67929
ENSG0000	RP11-111I	7:4290872	9.01971	7.01826	16.6448	12.0158	12.6879	8.09502	10.8888	17.5424	1.89327	6.90725	0.86535	-0.43987
ENSG0000	COMMD2	3:1497384	24.5378	28.8545	35.5296	31.158	23.8481	41.1107	29.951	38.307	21.63	25.8456	0.94545	0.051477
ENSG0000	BDH1	3:1975097	13.7323	17.395	39.2466	22.9087	22.5765	6.30098	8.32947	14.3118	6.49533	11.6832	0.0047	-1.44511
ENSG0000	APTR	7:7765765	5.1628	4.49257	4.77452	8.90338	3.4702	11.3234	13.0106	4.90275	16.518	3.38252	0.41	0.85537
ENSG0000	FAM122A	9:6870541	5.29416	8.88523	5.51979	4.52955	4.97374	5.27869	5.31247	3.97437	1.39388	3.17983	0.18745	-0.61799
ENSG0000	MGA	15:416212	5.69625	7.26222	5.31342	4.63759	6.30623	4.56645	6.07148	4.13285	7.02304	13.735	0.82495	-0.1119
ENSG0000	PPP1R16B	20:388057	12.9969	11.3451	4.36964	5.71481	3.64564	16.8383	19.7165	16.097	7.14212	25.1887	0.00145	1.07242
ENSG0000	TRAV39	14:222025	1.48572	7.0527	0.599513	0	0	11.6831	5.00337	0.860768	4.72663	0	0.49215	1.36527
ENSG0000	MMP19	12:557432	0	0.047738	0	0.011105	0.223351	1.9488	0	0.289213	0.937221	0.494228	0.0473	3.48545
ENSG0000	SLC7A7	14:227732	1.44579	8.99129	0.306414	0.660059	1.53267	21.8201	1.29662	1.08417	0.056977	0.932179	0.17875	0.986449
ENSG0000	RPS11	19:494963	454.921	389.344	1005.36	721.299	410.16	500.027	587.605	841.059	596.638	111.985	0.5885	-0.20384
ENSG0000	USP5	12:685212	29.1773	30.8911	9.42572	12.8332	10.1649	7.66328	22.2618	10.367	7.17194	14.148	0.1832	-0.51655
ENSG0000	IKZF4	12:560076	0.909784	1.00039	0.197669	0.299546	0.098157	1.95614	1.92879	1.15104	0	0.884387	0.1072	1.28935
ENSG0000	GPR89A	1:1456079	27.748	31.6254	25.4578	21.7067	19.6367	29.7311	30.1274	14.8445	18.5147	22.6652	0.9964	0.002049
ENSG0000	RP11-688	14:994811	0.311911	0	0.758613	0	0.465682	0	0.127439	0	1.64088	0.333262	0.78155	0.794892
ENSG0000	EEF1G	11:625596	421.683	501.382	1116.35	773.841	936.196	325.013	294.333	612.862	654.076	287.295	0.1053	-0.79002
ENSG0000	AC098614	3:2763297	2.0576	2.42521	1.51529	0	1.75821	2.36045	1.24099	3.35163	0	3.77785	0.7701	0.410663

ENSG0000C	SLC25A20	3:4885693	57.5696	39.9528	35.512	48.7068	57.4014	44.7615	51.4876	34.5783	38.2125	49.4324	0.6173	-0.16309
ENSG0000C	GRAMD4	22:465760	5.90919	11.009	0.730915	0.59979	0.968234	12.108	15.3572	4.12158	2.41992	7.65268	0.0719	1.09863
ENSG0000C	RNU2-63I	2:8801635	5.71628	0	1.00415	0.733167	0	0	0	0	0	0	0.0118	#NAME?
ENSG0000C	CEP89	19:328759	10.919	8.12268	6.75374	4.47001	7.06598	5.23319	11	6.16931	5.90543	8.948	0.6687	-0.21661
ENSG0000C	CLTC	17:596185	29.4582	39.2646	9.62799	7.92682	14.6635	33.5998	29.8015	19.0251	15.654	35.858	0.57965	0.341227
ENSG0000C	ATF1	12:507637	25.5196	37.0164	14.1237	12.3562	12.2274	27.6497	23.9947	17.3224	15.7085	14.8879	0.8885	0.052442
ENSG0000C	GDPGP1	15:902299	1.14548	2.4613	3.12386	3.23519	1.93152	2.22287	0.859815	1.31429	0.51856	1.78431	0.1796	-1.2869
ENSG0000C	CLIC3	9:1369946	76.9118	40.9906	82.484	130.795	91.007	137.565	146.306	78.4086	133.487	50.6003	0.52755	0.241615
ENSG0000C	C22orf39	22:193306	26.188	21.5317	31.3251	29.3224	18.8558	25.1778	28.0795	20.1519	39.5222	16.6225	0.9855	-0.01522
ENSG0000C	GPR132	14:105049	8.77927	14.657	10.5982	12.8538	29.5913	14.7311	23.871	34.5775	25.9886	63.5789	0.0712	0.8495
ENSG0000C	ZNF287	17:165513	6.05401	7.47888	13.0874	9.9111	7.55706	14.4908	8.53468	9.62378	11.3973	3.82334	0.83835	0.118986
ENSG0000C	ATG10	5:8197202	9.72668	6.43273	10.7735	7.93813	7.49885	7.62012	1.56315	8.86935	12.2082	8.54515	0.7872	-0.1924
ENSG0000C	C6orf48	6:3183460	39.0058	46.1714	172.281	151.276	95.4466	41.8502	58.7711	177.601	115.034	47.2947	0.5383	-0.23802
ENSG0000C	PALLD	4:1683567	0.472636	1.58577	0.707298	0.019058	0.094406	4.20908	0.345568	1.03689	1.07837	0.439493	0.2311	1.33075
ENSG0000C	SRPRB	3:1337840	25.539	14.2263	30.4164	28.2295	27.2111	18.2268	31.957	28.7884	21.5155	16.5331	0.79035	-0.09007
ENSG0000C	RP11-192	17:284557	0.133224	1.13275	0.426643	1.31352	0.071492	0.021776	0.082834	1.21594	1.93515	0.884432	0.61145	0.771726
ENSG0000C	BCL2L13	22:176288	14.7087	12.0225	16.6797	15.8456	16.3467	13.8234	9.59126	11.306	10.864	29.0839	0.59155	-0.21553
ENSG0000C	RP1-40E1	6:3118373	0.10762	0	0.772234	0.001165	0	0	0	0.240719	0	0.184599	0.4272	-2.09877
ENSG0000C	CRK	17:142068	9.88534	7.82633	17.2566	12.0373	12.6009	11.0098	11.964	16.3214	12.695	27.749	0.5283	0.246132
ENSG0000C	DLEU1	13:499825	10.4476	6.93032	11.5142	11.8027	11.2463	3.94605	13.2296	8.81314	14.9882	7.17838	0.86075	-0.18152
ENSG0000C	EDC3	15:745984	14.7591	15.8818	7.80114	9.48123	7.91131	9.91147	12.8146	5.18034	7.37878	9.98476	0.5505	-0.38431
ENSG0000C	IQCE	7:2558971	2.56566	2.46271	2.56636	0.678135	3.90881	3.25482	6.60818	1.30377	3.07798	3.75412	0.463	0.477823
ENSG0000C	FAM133B	7:9256079	24.4021	26.7435	30.8772	23.7372	39.7605	30.6571	31.0615	27.6679	26.7253	37.1968	0.95465	0.02352
ENSG0000C	SMPD3	16:683109	1.35568	5.3551	0.899193	0.428884	0.72166	1.72325	6.45879	1.90616	0	3.18617	0.70405	0.420266
ENSG0000C	ANGPTL6	19:100861	1.2325	1.31373	1.29783	1.74157	1.19051	7.33955	2.82767	0.940812	1.02949	0.529947	0.4866	0.752085
ENSG0000C	RPARP-AS	10:102449	12.5284	19.8536	29.9733	27.5757	11.9401	5.73546	16.653	24.3185	19.8917	16.6088	0.52685	-0.3157
ENSG0000C	ERCC6L2	9:9575923	19.3313	19.3857	8.1554	11.581	10.6757	8.60116	9.46544	7.52514	8.11534	20.2103	0.7184	-0.21544
ENSG0000C	ERICH6B	13:454648	0.847992	1.47511	0.063268	0.165513	0	0.365442	0.570519	0.139744	0.265301	0	0.63555	-0.75569
ENSG0000C	SDR39U1	14:244265	85.5983	144.293	164.944	154.362	108.648	53.8198	63.0804	101.062	99.526	112.11	0.2308	-0.57901
ENSG0000C	LIG3	17:349278	7.77185	8.62632	6.45246	2.93235	3.07111	2.18004	8.25379	3.14759	1.84122	4.18946	0.1662	-1.06141
ENSG0000C	MT-ATP8	MT:8365-!	83.9276	57.3309	219.351	420.454	591.456	109.467	64.384	190.897	404.022	176.957	0.68455	-0.40818
ENSG0000C	APOC4-A	19:449422	0	0	0	0	0	31.254	0	1.18652	0	0	0.05015	inf
ENSG0000C	MTF2	1:9307923	24.1565	29.7699	42.0853	36.5684	31.3498	37.1321	28.6921	28.927	23.7407	54.0869	0.48895	-0.29464
ENSG0000C	TMEM242	6:1572893	10.8136	4.62342	17.8699	17.3721	9.15611	13.7077	11.8405	10.2219	11.9382	4.91265	0.59225	-0.28498
ENSG0000C	LONRF3	X:1189746	2.4268	0.625455	3.49848	3.19103	2.51396	0.978522	0.303678	3.9503	1.34849	1.73254	0.59035	-0.59728
ENSG0000C	TAS2R14	12:108249	0.682321	0	0.432445	1.00398	0.504672	0.019405	2.71602	0.708785	1.11538	1.29602	0.43755	1.14328
ENSG0000C	RP11-849	11:718655	4.02792	7.40521	4.66146	3.9696	3.35149	2.80027	5.7171	4.15127	10.5081	3.18957	0.7087	0.238799
ENSG0000C	VAMP2	17:814047	102.327	152.316	110.718	105.733	172.257	74.4034	128.204	89.0468	100.032	210.715	0.62895	-0.18536
ENSG0000C	EIF4EP1	20:554440	1.15811	2.26334	0	0	0	0.923966	0.895719	0	0	0	0.75	-0.76254
ENSG0000C	SLC36A1	5:1514370	0.72615	1.80077	1.72002	4.28119	0.963066	3.1918	0.291914	0.846695	0.41246	1.79823	0.136	-1.1565
ENSG0000C	TFIP11	22:264838	39.5967	29.2768	21.0383	25.3998	19.3906	29.2686	24.6261	24.2142	9.72254	30.2091	0.636	-0.17909
ENSG0000C	MRPL39	21:255856	23.9822	33.2509	32.1034	30.5685	15.516	38.431	28.66	33.1844	17.0243	8.08294	0.78735	-0.11366
ENSG0000C	SHF	15:450926	0.606089	0.4256	0.813409	0.313268	0.619904	0.528317	0.240563	0.024033	1.74421	0.747194	0.4064	0.645657
ENSG0000C	RHOH	4:4019105	104.8	130.83	148.096	141.7	111.022	135.567	124.87	109.446	79.4193	88.2222	0.2127	-0.39138
ENSG0000C	GLO1	6:3867592	85.6047	54.6018	62.786	61.227	48.2353	64.9695	53.6029	25.7651	65.5466	22.3059	0.22065	-0.39545
ENSG0000C	AC093375	2:1560115	0.05529	0.059884	0.499793	1.43348	0	0	0	0	0	0	0.0018	#NAME?
ENSG0000C	TRBV5-4	7:1424629	15.4475	21.7544	8.4512	7.29695	1.516	43.7725	17.5096	8.37479	16.8102	7.72869	0.23845	0.878249
ENSG0000C	ANKEF1	20:998608	2.84279	2.05742	1.14752	1.69325	1.64271	2.31485	0.637539	0.941234	1.17576	1.70018	0.28055	-0.86494
ENSG0000C	SERPINH1	11:755620	6.70284	4.1934	2.47361	2.65291	5.2238	18.345	6.92832	3.11028	11.3052	11.4133	0.0072	1.27382
ENSG0000C	PHGDH	1:1196597	4.27984	13.6636	1.97274	2.54508	4.56812	9.37168	12.7989	8.1093	4.5634	7.23592	0.25295	0.610851
ENSG0000C	RP11-708.	3:4737908	2.1062	1.27469	3.19857	1.78022	1.5329	5.44994	1.20579	6.25191	0	3.79906	0.4159	0.642342
ENSG0000C	YIPF3	6:4350696	60.896	63.7682	60.4814	36.1824	70.4455	63.6402	81.2649	69.4603	46.5507	65.5279	0.718	0.143496
ENSG0000C	S100A8	1:1533900	247.816	169.559	17.5716	150.996	33.8045	306.018	10.441	94.72	135.42	34.8688	0.91845	0.044427
ENSG0000C	RP11-543	12:307735	3.58296	4.19609	1.34098	1.74766	0.284336	2.78743	7.71971	2.92928	1.44202	0	0.78445	0.59125
ENSG0000C	TTC22	1:5477971	7.775	6.19945	7.34672	7.37168	9.56813	6.28657	4.79877	3.22458	4.51353	7.55488	0.23595	-0.57085
ENSG0000C	RP11-706	X:3891437	5.8734	8.60964	2.99544	7.84741	4.68979	2.7603	5.36668	1.7983	6.61091	9.12675	0.8616	-0.21409
ENSG0000C	WDR60	7:1588565	8.19261	8.35253	7.86272	9.77187	10.4121	10.6939	7.66988	5.89213	4.70034	6.25979	0.63755	-0.43906
ENSG0000C	ARHGEF4(	14:210167	1.06913	1.99932	0.505514	0.075212	0.073996	0.971909	1.50495	1.1998	2.25624	0.386214	0.50025	0.759387
ENSG0000C	KCTD6	3:5849211	15.0793	13.6702	12.7465	12.4084	10.1009	6.36252	2.17074	8.62095	5.59024	7.27744	0.0411	-1.06851
ENSG0000C	ZNHIT3	17:364866	57.3872	28.5133	91.4771	88.2332	54.2756	48.4223	63.2102	75.7565	73.1515	25.2683	0.80295	-0.12456
ENSG0000C	MIR646HC	20:600878	0.428746	0.439198	0.619009	0.538479	0.766073	0	0	0	0.029232	0.1434	0.0824	-4.72068
ENSG0000C	TAF15	17:355747	20.37	27.6586	8.84287	13.2458	6.43592	18.77	21.7778	6.56362	17.8785	15.0653	0.86445	0.133162
ENSG0000C	CD300E	17:746098	1.11941	9.43202	0.322266	0.011537	0.311348	7.39336	0.045591	0.820666	3.04585	0.44641	0.7297	0.22359
ENSG0000C	PRDM10	11:129899	1.56429	1.67763	0.637292	0.323936	0.701023	1.26071	1.18828	0.493474	0.042682	1.5799	0.90945	0.096995
ENSG0000C	TPST1	7:6620519	0.78803	0.288584	1.38682	0.545682	1.83212	3.04649	0.016835	0.537454	0	0.158835	0.7931	-0.29289
ENSG0000C	DLGAP4-/	20:363063	0.443695	0.027884	1.49587	0.289483	0.44601	0.255718	0.828318	1.47365	0	0.194004	0.8855	-0.18567
ENSG0000C	ABHD15	17:295605	19.3402	4.99496	17.7387	20.4268	13.9227	22.1208	14.3442	15.7358	24.0541	26.2835	0.625	0.419602
ENSG0000C	AJAP1	1:4654731	0	0.473613	0.802466	0	0.08188	0	0	0	1.95725	0.513181	0.54785	1.25642
ENSG0000C	CSGALNA(	8:1940416	4.03908	37.4428	0.975278	3.68762	0.623018	6.81702	12.6133	6.60902	12.7016	6.33353	0.921	0.047572
ENSG0000C	LCMT1-AS	16:250855	1.87152	0.614407	0.993899	0	0.930551	0	1.42804	0.524069	0	0.673584	0.5987	-0.65603
ENSG0000C	PNRC2P1	1:1177780	1.81531	1.43347	1.26649	1.90497	0.071357	1.05084	1.37924	0.505311	0.755526	0.082442	0.72775	-0.54095
ENSG0000C	DST	6:5645798	0.36274	0.606052	0	0.366253	0.01182	0	1.663	0.622492	0	0.333774	0.2262	1.10645
ENSG0000C	CDKL5	X:1842558	0.316773	0.718998	0.619903	0.848818	0.473646	0	0	0.452159	2.30306	0.331716	0.7754	0.283341
ENSG0000C	AP2A2	11:924893	4.12804	6.19662	2.35207	4.68337	3.93729	4.21971	4.42601	3.77511	2.71745	5.9609	0.38285	-0.4093
ENSG0000C	ACOT2	14:735676	14.3809	15.3135	20.7842	19.8599	18.4277	20.7301	20.9802	18.3663	13.4802	13.0613	0.9938	0.003203
ENSG0000C	RP11-571	4:9906725	0.540781	1.08239	0.342047	0.513255	0.279527	0	0	0.248159	0	0.660668	0.3939	-1.60528
ENSG0000C	RP11-89H	12:478415	0	0	0	0	0	1.83051	0.428113	0.419758	0	0	0.12655	inf
ENSG0000C	QRICH2	17:762740	0.374095	0.637794	0.032993	0.721466	0.493335	0.826552	1.80064	0	0	0.21006	0.8073	0.281555
ENSG0000C	MAN2B2	4:6575174	15.799	15.2614	6.05883	4.40811	7.79462	8.82878	9.80696	7.36979	2.10597	13.8922	0.50935	-0.24064
ENSG0000C	SLC43A1	11:574845	13.0628	5.3685	11.6987	12.4255	9.34111	11.8851	11.4865	12.3585	16.1435	14.3253	0.4145	0.339463
ENSG0000C	NCK1-AS1	3:1368186	9.84836	3.52258	11.191	8.73215	3.74078	8.47919	9.56778	6.77083	8.45857	3.54193	0.9724	0.029214
ENSG0000C	SERTAD2	2:6463162	3.83561	7.22212	6.10756	3.65958	12.6759	6.02742	4.10275	5.62217	2.1533	12.5038	0.65	-0.32923
ENSG0000C	HTATIP2	11:203636	69.7701	55.6048	67.0966	68.8954	54.8902	77.3174	74.8717	48.3168	34.1308	35.7089	0.4668	-0.24117
ENSG0000C	MPEG1	11:591714	3.01854	24.4965	0.379605	0.328927	0.432339	12.7304	0.061786	0.348138	0.332555	0.26255	0.16755	-1.0274
ENSG0000C	WDR33	2:1277010	24.2346	24.9501	22.1449	16.2915	20.5523	29.585	29.1958	21.8618	28.0693	28.7257	0.52	0.238325
ENSG0000C	SPTLC1	9:9203199	36.8398	38.7224	18.508	33.4645	16.7468	39.5765	32.215	16.7549	34.9132	21.1508	0.94045	0.026755
ENSG0000C	EIF4E	4:9887168	43.5436	40.5855	54.6831	48.3074	25.6961	57.2158	62.3654	32.5066	35.2298	15.851	0.8704	-0.06948
ENSG0000C	GTF2IP4	7:7315493	31.485	33.9996	10.8238	9.72391	16.2694	23.7756	18.865	14.2044	5.70202	22.9061	0.6547	-0.28703
ENSG0000C	SLC35G1	10:938939	3.95315	4.78436	2.20567	4.07374	2.13817	3.94323	2.01415	3.20942	2.96199	5.6651	0.8693	0.176486
ENSG0000C	PRR4	12:108249	14.9116	23.9858	15.175	20.8684	10.0418	14.9772	28.0502	12.7683	17.2282	10.0868	0.9721	-0.02896
ENSG0000C	H2AFJ	12:147743	79.7373	45.5159	41.3702	57.2807	51.8095	93.9373	90.1214	57.494	62.6399	16.4711	0.86715	0.07708

ENSG0000	ALOX12P2	17:685386	0.494408	0	0	0	0	1.26018	0	0.550904	0	0.846228	0.1926	2.57809
ENSG0000	REEP6	19:148142	3.34774	2.47012	2.94753	2.44594	3.16957	2.48106	2.09765	3.57896	3.68316	2.34276	0.99575	-0.0032
ENSG0000	ACBD5	10:271952	17.3356	9.57971	8.11094	8.2096	10.1072	14.1665	18.7158	7.06458	10.5466	12.7563	0.62895	0.200206
ENSG0000	STK25	2:2413562	74.6123	90.3419	41.6075	47.81	52.5489	75.8461	95.8464	42.4956	59.0881	63.9298	0.9149	0.033719
ENSG0000	RPL10A	6:3546840	392.184	328.907	1136.2	814.291	509.813	340.989	401.706	682.729	543.288	112.964	0.10195	-0.66266
ENSG0000	SPOPL	2:1385018	2.46457	2.08786	1.13055	0.961034	1.14787	3.73553	3.53156	2.53465	0.835241	2.82513	0.16225	0.74652
ENSG0000	SH3GL1P1	17:320078	1.59746	3.28562	0.710011	0.30551	0.628986	0	2.77409	0.47403	0.52208	3.94262	0.84895	0.249075
ENSG0000	MPP6	7:2457326	1.52774	3.45529	2.96326	0.611194	1.86342	0.762433	1.10576	2.28434	4.65988	3.73286	0.8714	0.12882
ENSG0000	RP11-244	1:3497435	1.28018	1.20036	0.662945	1.18456	1.8441	0.843152	1.43619	1.74325	1.45279	1.06272	0.6586	-0.65127
ENSG0000	RP5-1160	1:1095486	0.514467	1.02145	0.220066	0.588895	0.306429	0	0.05257	0.122722	1.11606	0.901701	0.92275	0.146244
ENSG0000	TIAL1	10:119571	11.2988	17.9265	10.4518	8.48425	12.8422	17.8913	28.5309	15.758	8.01265	27.1948	0.19845	0.624459
ENSG0000	FRS2	12:694703	4.69999	1.35905	9.09895	8.14051	19.4053	2.95171	1.1755	20.3021	9.93926	15.4457	0.9532	0.058177
ENSG0000	USP37	2:2184190	3.86943	3.37275	1.51163	1.16153	1.78229	6.23727	4.52231	2.17209	7.99379	3.10362	0.1411	0.9007
ENSG0000	SYNGR3	16:198420	1.77483	0.606638	5.15012	2.27154	4.70157	0	12.3384	7.60777	13.6114	8.88288	0.0829	1.49202
ENSG0000	MAP2K7	19:790384	1.38088	2.88149	1.34311	1.10553	2.42304	2.31989	2.79818	1.95572	0.878264	4.82821	0.59575	0.397936
ENSG0000	ABHD11	7:7373609	10.2668	7.25757	17.8508	16.9626	16.3571	14.0665	11.0949	16.9715	8.82554	17.8439	0.92565	-0.04043
ENSG0000	FAM208B	10:568483	8.30845	9.08955	3.03989	4.13261	3.26534	2.75756	5.74358	6.59434	12.6717	9.69109	0.2996	0.532104
ENSG0000	PHOSPHC	2:1696944	4.26761	0.624358	3.24079	4.0586	2.78701	3.97849	6.21583	3.2831	0.172465	0.690757	0.98095	-0.02356
ENSG0000	CROCC	1:1673993	12.9314	11.6267	4.56766	5.79085	4.26249	7.01596	14.1454	4.64453	5.64641	6.38263	0.9245	0.051615
ENSG0000	CCDC159	19:112961	27.5847	34.2506	41.5665	33.8962	36.5486	41.6902	35.4659	35.0685	18.3138	24.7943	0.6845	-0.20235
ENSG0000	OGFRL1	6:7122145	3.65445	2.52118	0.710337	1.212	1.51987	2.7262	2.85891	1.49882	0.195329	2.43259	0.8762	0.080906
ENSG0000	VIPR1	3:4248929	5.37926	13.9457	2.84656	6.47884	3.40726	0.462586	0	1.10686	4.73349	3.39321	0.1688	-1.33816
ENSG0000	KLHL24	3:1836355	12.3797	15.5392	14.1831	17.1797	8.93648	12.2062	16.0597	6.21834	33.4654	19.8957	0.55755	0.274693
ENSG0000	WDR37	10:104953	8.42577	10.9622	5.53005	7.79222	5.23901	3.43619	5.14021	3.89981	5.18028	5.83173	0.04915	-0.78489
ENSG0000	FSIP1	15:396000	1.50922	0.189937	1.94013	1.19862	0.202873	0.382084	0.483478	1.29542	2.10647	0.725343	0.97855	0.036808
ENSG0000	SBDS	7:6698767	72.6588	51.009	91.4744	168.513	67.4908	60.842	62.8536	66.0432	112.447	36.6045	0.3058	-0.40559
ENSG0000	RP1-283E	1:1702729	1.82873	7.89015	0.710324	1.48796	2.92547	1.18594	2.58437	1.26634	2.896	3.39538	0.99485	0.008607
ENSG0000	NUDT18	8:2210687	12.826	11.0694	7.69629	10.3069	14.3583	8.91914	6.91075	9.507	9.15606	9.71012	0.5689	-0.31887
ENSG0000	NCEH1	3:1726302	2.45495	1.82616	0.683072	0.871734	0.856636	7.18827	1.27697	0.046626	1.90147	1.27935	0.25945	0.748967
ENSG0000	RPS16	19:394332	324.481	309.332	657.744	554.431	302.891	329.912	440.908	614.206	446.597	87.3371	0.5936	-0.20281
ENSG0000	FLOT1	6:3072770	57.2078	57.809	53.4678	44.9608	77.9417	97.48	63.7507	56.5057	51.135	65.0925	0.563	0.183173
ENSG0000	TRAV29D\	14:221632	17.9129	61.5643	8.87092	0.540921	1.05661	43.3044	106.457	10.092	5.42675	1.39682	0.1838	0.912577
ENSG0000	ZNF717	3:7570964	1.96974	0.952516	2.79571	4.08539	1.42656	4.00301	0.531652	1.64252	0	1.71956	0.51555	-0.66268
ENSG0000	CPSF2	14:921219	8.51275	4.69656	3.71556	2.82926	5.44472	10.1414	7.08053	5.07031	4.17029	8.89236	0.8062	0.135563
ENSG0000	FBXO33	14:393976	11.991	11.6826	5.65127	8.7176	6.45007	9.79232	10.6525	9.22237	11.3479	12.3311	0.38115	0.317997
ENSG0000	ETFA	15:760598	130.812	83.8587	107.725	123.03	137.196	115.754	127.828	92.2445	111.21	60.7988	0.65575	-0.17067
ENSG0000	EIF5	14:103333	189.848	144.841	254.336	145.602	455.147	292.495	482.104	260.337	140.794	371.389	0.8864	0.074114
ENSG0000	CLP1	11:576380	8.70157	9.24539	14.2803	16.9682	8.866	9.12941	8.52262	13.9187	6.15213	14.914	0.65385	-0.21629
ENSG0000	RP11-349	X:1235144	0.798859	1.04174	0.390308	0.510017	0.129131	1.14949	0.838564	0	0.175587	0	0.83215	-0.3433
ENSG0000	ZNF610	19:522695	1.80013	0.772784	0.132789	0	0.24727	2.0062	0	0	0	1.04534	0.9252	-0.14747
ENSG0000	MTBP	8:1203807	2.49549	1.94136	1.23856	1.56221	0.621611	1.31002	1.94528	1.53142	7.04861	1.68858	0.4543	0.814333
ENSG0000	HIST2H4A	1:1498326	8.01417	3.83459	13.2451	14.5755	7.15875	7.33486	3.05155	12.7815	13.5026	6.68836	0.8892	-0.22711
ENSG0000	RP11-186	10:121296	0.467098	0.300887	0.486794	0.175369	0.404794	0.6047	1.44205	0.066119	0	1.10041	0.64525	0.574417
ENSG0000	MINA	3:9782203	18.4783	18.8622	16.3558	17.873	12.8047	6.40787	5.59201	4.86224	17.8899	8.85039	0.06815	-0.92532
ENSG0000	C11orf98	11:626468	133.382	111.858	206.225	173.815	216.161	218.232	213.161	181.18	132.05	63.4211	0.83805	-0.10134
ENSG0000	PAF1	19:393858	38.3809	40.6681	33.222	19.3476	34.186	20.2123	38.8944	30.8575	12.5609	23.7672	0.1961	-0.47744
ENSG0000	ANGEL1	14:767621	8.40476	5.93137	3.88227	3.33707	2.99764	4.33753	1.33083	2.48329	4.95959	7.9273	0.48415	-0.347
ENSG0000	S100A12	1:1533737	19.0866	10.0703	0	16.1317	1.51614	2.47702	0.697207	4.7906	0.850354	2.19737	0.28805	-1.75558
ENSG0000	AC006547	22:200170	0.675649	2.94473	0.710703	0.838712	0.504412	1.42503	1.29354	0.07878	1.32846	0.944942	0.9946	0.007823
ENSG0000	DNM2	19:107180	74.3127	100.539	14.5306	18.1198	24.5875	71.9455	59.373	45.6388	30.557	47.7296	0.6882	0.169062
ENSG0000	REG4	1:1197940	0	3.79267	0.26154	0.658217	0.160318	0	0	0	0	0	0.00385	#NAME?
ENSG0000	RP11-371	14:931849	0.121251	0.162502	0.279127	0.149511	0.847124	0.24537	0.31812	0.330957	0.58567	0	0.8593	-0.23753
ENSG0000	FOXP4	6:4149485	1.19146	0.494387	0.296563	0.062784	0.734618	2.39691	1.27426	1.13158	0.867345	2.02413	0.1989	1.19814
ENSG0000	METTL25	12:822236	10.4754	7.74216	6.64491	5.40915	9.41436	6.47934	4.13264	4.22204	6.74823	5.57876	0.529	-0.48235
ENSG0000	MKRN1	7:1404530	65.6328	102.9	49.4676	50.7602	32.6624	52.9701	44.2093	27.2483	41.0373	38.6338	0.0874	-0.58727
ENSG0000	CHRAC1	8:1405112	21.8495	22.2494	23.2897	27.0823	18.5443	27.2793	30.8918	30.5777	7.99826	16.1972	0.7305	-0.13255
ENSG0000	CTD-3099	19:525885	0.890231	1.66601	1.47621	0.285834	0.281713	0.616988	0.160522	0.343414	0.944396	1.24536	0.6947	-0.62713
ENSG0000	MED10	5:6371880	95.3435	97.327	142.252	164.153	114.96	86.0914	126.464	121.412	143.103	94.1103	0.78465	-0.08873
ENSG0000	CTD-2207	19:734894	8.67165	10.371	2.10717	2.50745	5.74548	1.39491	0	0.486355	0.090084	2.12978	0.1328	-2.60374
ENSG0000	GOLIM4	3:1680086	3.01422	0.532362	4.33898	3.18884	5.65904	12.4217	8.69517	12.4605	16.0411	15.8205	0.001	1.92315
ENSG0000	UBE2G2	21:447685	49.3933	41.7705	48.5186	45.6189	46.7881	29.4668	31.4033	37.936	38.3228	56.2275	0.2694	-0.34868
ENSG0000	SEC22B	1:1201508	10.5447	8.03527	12.419	7.51185	9.00329	10.1526	7.69729	15.3316	8.2324	10.0488	0.7642	0.102939
ENSG0000	PERM1	1:966496-	0.04962	0.263648	0.218345	0	0.179334	2.81213	1.19599	0.498763	3.46361	0.292949	0.0805	3.81693
ENSG0000	SIPA1L3	19:379072	1.82742	3.52993	1.53733	1.33256	1.58364	1.8496	1.67655	1.36673	1.93149	2.75073	0.7642	-0.18525
ENSG0000	AC006129	19:415312	0.480747	0.532211	1.17628	0.575065	0.263516	1.59544	0.687115	0	0.608416	0.106809	0.93225	-0.1367
ENSG0000	PACRGL	4:2069628	4.70658	2.83212	6.35669	9.15325	4.59174	10.1609	3.11308	8.77999	4.7947	2.33812	0.8045	0.106839
ENSG0000	STAP2	19:432404	1.58406	1.70191	1.85073	0.523341	3.14062	1.4879	3.22243	0.927603	3.03823	1.64907	0.76195	0.254252
ENSG0000	MCFD2	2:4689927	13.9549	14.7698	13.84	13.7828	16.8315	14.1398	8.00103	12.0526	9.07033	24.4136	0.6722	-0.3398
ENSG0000	CTD-2262	15:853805	8.66753	2.04774	3.07902	4.6782	1.00564	1.707	0	0.798461	4.33581	0.847621	0.48195	-1.10887
ENSG0000	SMYD4	17:177948	9.98002	13.6222	6.43679	6.10269	4.80702	2.76852	7.92433	9.35893	8.98606	15.8363	0.86605	0.154067
ENSG0000	RP11-574	10:737443	10.0096	8.89951	2.07489	5.65657	0.368588	1.11357	1.58588	0.747108	7.26215	1.15543	0.96735	-0.06698
ENSG0000	ALAD	9:1133863	13.4203	19.7531	11.8137	7.72984	10.1536	16.393	4.08147	10.7873	14.1629	12.0871	0.8481	-0.07826
ENSG0000	RUBCN	3:1976713	11.3685	15.498	7.2756	5.80341	9.0736	18.4228	19.7133	8.03256	7.07117	15.3087	0.7498	0.17103
ENSG0000	CASC4	15:442887	26.8837	16.0954	9.00399	8.57007	8.79794	20.6673	15.7858	11.0922	8.90806	12.7247	0.96985	0.012769
ENSG0000	ORMDL1	2:1897703	164.269	214.707	144.936	169.982	71.8516	159.107	192.602	170.088	162.964	106.489	0.92435	-0.04125
ENSG0000	JADE1	4:1288096	15.9368	18.6004	12.516	14.187	8.89798	7.56486	10.2804	8.16547	10.2932	17.9653	0.20095	-0.58973
ENSG0000	RTN3	11:636814	34.6465	46.7073	24.9259	28.0888	33.964	58.2205	41.5381	20.1569	36.5346	30.8868	0.73645	0.109618
ENSG0000	ZNF710	15:900013	3.01786	3.24657	0.794338	0.359884	1.00318	5.94699	2.28752	0.750556	1.58219	1.94186	0.62715	0.461852
ENSG0000	ERAL1	17:287559	24.7596	34.1452	35.3644	31.5229	26.6147	28.8052	15.5332	17.2114	8.91399	17.7745	0.0457	-0.8359
ENSG0000	RP5-855D	8:213185-	5.62479	1.66612	3.05295	3.30049	2.23869	7.3159	6.21307	3.60482	7.72659	6.93038	0.5395	1.17071
ENSG0000	ARMCX4	X:1014182	2.22381	2.44552	1.1105	1.27003	0.588725	2.82658	0.483582	1.72742	1.39915	0.880916	0.91345	0.120254
ENSG0000	RP11-156	17:418485	2.81299	2.96415	1.02004	1.29098	1.78702	1.05446	2.11078	3.43792	5.11871	5.87126	0.10205	0.822454
ENSG0000	FHAD1	1:1524727	0	0	0	0	0	3.67688	0.085202	0	0	0	0.03635	inf
ENSG0000	CXCR2P1	2:2180349	0	0	0.377621	2.17649	0.137697	1.69238	0	0	0	0	0.7357	-1.06254
ENSG0000	MCM6	2:1358396	14.4375	22.3824	12.0563	12.203	7.94853	30.5001	31.6338	27.5296	16.0216	22.6792	0.01265	0.894656
ENSG0000	CTD-3222	19:164790	0.278813	0.389497	0.312868	0.550763	0.043749	3.25832	0.561355	0.447108	0.542727	0.237178	0.27525	1.6204
ENSG0000	RAD51B	14:678197	17.3826	15.6108	15.7393	13.4921	9.80097	8.26652	16.987	11.3839	14.4889	9.71165	0.71645	-0.19843
ENSG0000	VPS13D	1:1223006	7.13644	9.00174	3.55379	2.59722	7.09393	3.06638	5.85083	5.1197	3.3193	13.6677	0.94055	0.039905
ENSG0000	NTRK1	1:1568062	1.66497	0.133574	0.459253	1.40692	0.09102	2.60636	2.12384	4.89798	2.13771	1.50748	0.06845	1.73511
ENSG0000	LINC0141(	9:6280146	0.548212	0.139537	0.221021	0.23488	0.291341	2.1825	0.170114	0.587355	0	0.27429	0.72185	0.54226
ENSG0000	CTD-3088	16:113598	0.44733	1.55134	0.058546	0.170447	0.037939	0	0.657286	0.799628	0.029844	0	0.68135	-0.46486

ENSG0000	LARP1B	4:1280612	6.24053	1.86482	3.28393	3.60332	4.25422	5.28694	7.22144	6.49682	7.52172	4.3181	0.28205	0.658181
ENSG0000	LY6G6F	6:3168694	0	0	0	2.17393	0.088895	0	0	0	0	0	0.15695	#NAME?
ENSG0000	FAM13B	5:1378143	10.7199	14.4842	2.46864	5.54128	4.24102	13.714	5.04354	4.89624	5.6167	9.87408	0.7745	0.132741
ENSG0000	RELA	11:656535	35.7337	29.1575	18.1936	15.5339	37.7097	12.9594	27.3306	26.4971	26.3544	47.0555	0.672	0.154381
ENSG0000	SAMD3	6:1300136	245.037	120.657	205.828	226.516	175.136	82.4907	97.6666	76.8212	45.2343	65.3458	0.0002	-1.39889
ENSG0000	NEDD4L	18:580443	1.24807	2.37732	0.822179	0.142579	0.850304	0	1.48827	0.040452	2.62235	0.758386	0.62995	0.359548
ENSG0000	HAGHL	16:720580	9.23491	5.21973	11.132	13.2966	16.7843	11.8877	9.32309	7.41189	15.3806	12.0055	0.99005	0.010253
ENSG0000	VTI1B	14:676197	29.6215	39.3042	48.5062	35.0636	36.7806	58.1954	61.6846	38.6933	34.6006	20.116	0.9685	-0.02568
ENSG0000	RRBP1	20:176136	16.5461	14.2804	5.13819	4.59564	17.9044	27.9161	22.9296	5.43623	10.1084	21.6144	0.8425	0.124611
ENSG0000	C2orf48	2:1014138	0.348726	2.47062	0	0	0	0.726614	1.97817	0.604071	0	0.212334	0.84525	0.371708
ENSG0000	SLC35D1	1:6699933	5.30758	4.28481	2.24293	1.38016	2.25537	1.77036	4.398	2.44797	2.34461	5.52516	0.9705	0.015168
ENSG0000	NR1H2	19:503119	89.909	98.3211	116.552	93.5519	97.4099	94.3548	113.818	126.007	89.0986	113.816	0.80435	0.080318
ENSG0000	TGDS	13:945740	16.8754	17.0821	21.5085	17.9152	16.3707	8.4194	9.37084	13.613	14.6577	16.8347	0.15935	-0.56525
ENSG0000	PHF10	6:1697021	21.7002	25.3487	11.7377	8.58733	13.2913	17.9458	22.4568	15.4888	15.493	16.8844	0.73755	0.160712
ENSG0000	LRIG2	1:1130732	2.93611	2.89003	1.84145	3.37107	1.54256	5.91141	3.19893	3.11599	3.7203	4.08463	0.39465	0.541374
ENSG0000	LINC0087	3:7203529	0.248483	5.98944	0	0	0.365484	0	0	0	0	0.112499	0.23725	-5.95122
ENSG0000	TRMT61A	14:103529	7.62274	10.711	3.79629	8.76768	2.19491	4.01344	9.79672	3.62883	2.0527	2.20311	0.13745	-0.70949
ENSG0000	ZNF778	16:892177	1.59384	2.24618	6.91711	1.57079	2.21861	2.0284	2.20744	2.02377	1.44711	1.24568	0.3405	-1.31009
ENSG0000	ZNF347	19:531078	0.498095	1.13159	0.57025	0.903693	0.324253	0.097098	0.495047	0.205824	0	0.781584	0.1449	-1.62671
ENSG0000	PQLC1	18:799024	10.4533	11.1286	4.99123	2.93848	11.2015	14.4226	14.2926	5.58039	4.72704	13.6789	0.5513	0.292077
ENSG0000	USP21	1:1611594	31.7528	41.9594	11.3489	10.5353	13.39	15.9373	24.1444	12.7805	13.8343	19.9733	0.4198	-0.33746
ENSG0000	FDX1	11:110429	22.9116	13.9895	23.2777	21.0644	18.9596	20.2774	17.398	20.5245	26.858	8.24999	0.8029	-0.08476
ENSG0000	BMS1P4	10:736448	0.66574	0.487104	0.545883	0.464185	0.486406	0	1.24522	0.994266	1.73484	0.233846	0.76355	0.598057
ENSG0000	KIAA0319I	1:3543348	20.2529	22.0931	11.99	14.2762	9.38831	15.2236	10.9412	20.5809	10.0726	24.3188	0.89555	-0.0495
ENSG0000	AC241377	1:1209131	3.1028	3.63268	3.06749	2.81821	4.47741	0.989899	2.08926	2.8533	2.28417	1.85527	0.6506	-0.69181
ENSG0000	RP11-960	16:817382	0	0.187513	0.2988	0.834976	0.505399	0	0	0	2.18425	0.066928	0.56975	0.692013
ENSG0000	CIB1	15:902299	124.267	171.974	192.256	221.574	193.783	234.546	229.575	258.097	256.618	152.762	0.4035	0.289877
ENSG0000	PMPCA	9:1364019	52.0517	69.8571	49.9866	55.1225	38.1214	46.4395	47.6229	42.454	47.1762	44.1359	0.541	-0.21764
ENSG0000	TM6SF1	15:831074	1.28755	1.72043	1.42156	0.974001	0.442765	13.2156	4.89561	3.87924	0	0.992882	0.0707	1.96649
ENSG0000	ACVR1B	12:519516	0.165052	0.674773	0.468086	0.447271	0.41545	1.27738	1.00573	0.363704	1.2772	1.73208	0.12055	1.42324
ENSG0000	TXNDC11	16:116684	46.2483	44.3485	27.0077	30.6185	27.4733	37.1194	31.4706	29.7735	13.4741	31.1118	0.96685	-0.01739
ENSG0000	TLE4	9:7957177	40.8846	39.8668	137.727	81.5646	166.689	19.9498	15.747	58.676	80.7264	152.447	0.2961	-0.54466
ENSG0000	ZNF252P	8:1449735	3.21893	7.53076	1.29653	3.2497	2.37326	2.33899	3.32205	4.40595	2.85173	3.60896	0.9296	-0.06527
ENSG0000	UNC13D	17:758272	69.665	57.8266	17.6422	22.155	25.7449	44.8369	39.0115	22.2366	19.7893	42.8998	0.5057	-0.20402
ENSG0000	RP11-473	8:1298323	0.274131	0.533524	0.190722	0.258676	0.207908	0	0.309871	0.108592	1.43619	0.242893	0.6544	0.67206
ENSG0000	TSPAN4	11:842807	4.38339	9.28123	2.82248	4.14366	5.91141	45.3139	8.77846	8.71229	4.92004	3.59761	0.07195	1.30299
ENSG0000	NAP1L1	12:760365	66.7221	84.7877	68.6705	40.2842	101.871	98.0451	116.054	94.9517	66.877	77.5161	0.4316	0.298909
ENSG0000	RP11-415	3:1626515	0.098534	0.511902	0.150949	0	0	0.345084	0.37513	0.490114	0	0.62728	0.3693	1.51833
ENSG0000	C6orf52	6:1067141	1.53312	1.63203	0.338755	0	0	7.40022	0	0	0	0	0.59945	1.03201
ENSG0000	YWHAG	7:7632679	0.840108	0.529262	2.319	0.857117	2.67623	3.21482	1.76711	4.2664	0.837593	5.27064	0.24925	0.887609
ENSG0000	RP11-338	10:841384	3.29573	0.931545	0.661515	2.31712	0.286212	0.737773	0.728602	0.900427	3.0953	0	0.8334	-0.29722
ENSG0000	NFKBIL1	6:3152871	6.7138	10.2287	6.78352	7.17205	6.86524	7.37813	3.60693	4.45822	12.8	4.65452	0.95325	-0.07333
ENSG0000	RP11-680	11:941884	0.435916	0.722824	0.136666	0.827936	0.164389	0.561821	0.857457	0.33627	0.63272	0.538266	0.7727	0.555751
ENSG0000	AC092580	2:7736437	0.083935	0	0.757515	0	0	0	0.991432	0.763895	0	0.653944	0.3395	1.43873
ENSG0000	RP11-727	11:628322	2.38832	2.4587	4.31016	1.3862	1.70241	5.00747	2.34514	1.81759	2.40071	2.05055	0.8919	0.168485
ENSG0000	ANKRD20	18:141790	2.86778	1.82872	1.49066	0	2.64041	0.071008	0.748072	1.1206	0	3.56739	0.54695	-0.57119
ENSG0000	TRAF2	9:1368819	24.2622	30.0163	30.1611	26.0045	26.3169	18.5636	33.864	29.8248	18.0587	30.8743	0.87935	-0.05386
ENSG0000	HIST1H2B	6:2619951	0.885705	0.761563	2.87145	0.71565	0.774371	0	3.34339	0.444594	0	0.123274	0.71135	-0.67534
ENSG0000	APOA1	11:116835	0.361102	0.89306	0.611714	0.435029	0.527114	0.226528	2.18804	1.20929	0	1.03949	0.6845	0.51032
ENSG0000	PTGDS	9:1369775	1.00461	1.52407	0.531244	0.983523	0.344437	0	0.371221	0.434919	0.767784	0	0.12075	-1.43328
ENSG0000	RP11-449	17:761363	0.863494	2.26144	0.074314	1.02256	0.306742	0	1.03198	0.121891	1.65112	0.208596	0.64285	-0.70357
ENSG0000	TMEM9B-	11:894729	2.52657	3.32131	0.978092	0.295401	0.460451	0.833856	0.181328	0.790708	0.089584	1.19161	0.49745	-1.00515
ENSG0000	LENG8	19:544448	5.06847	6.98605	10.2818	8.78057	8.91344	7.04819	8.00999	16.4011	10.8121	27.9503	0.1968	0.650304
ENSG0000	ACTG1P17	15:827258	0.4182	0.528726	0.83869	0.553565	0.283193	0	0.194594	0	0	0.367829	0.3993	-1.78614
ENSG0000	ZNF675	19:235256	15.3752	17.3177	6.4554	7.45751	4.21761	4.63829	11.2977	2.75812	13.7766	5.69655	0.39385	-0.38308
ENSG0000	SIT1	9:3564929	128.425	75.6993	91.9359	70.8236	97.733	154.429	100.518	124.821	76.2313	80.9123	0.59285	0.175082
ENSG0000	VILL	3:3798805	15.0525	15.9232	7.70187	10.6308	8.56684	6.07562	14.809	9.87641	1.64066	7.9162	0.2908	-0.5524
ENSG0000	RP11-638	14:100207	6.79584	2.37488	0.872851	2.99966	0.595382	3.2043	2.37942	1.37233	0.280521	0	0.6037	-0.84038
ENSG0000	NDRG3	20:366517	18.3294	24.2801	18.4518	17.7156	20.9359	18.7842	12.6897	18.8294	13.176	31.5695	0.594	-0.18158
ENSG0000	TPPP3	16:673898	0.375203	5.20503	0.30912	1.23768	0.425447	0	0	0	6.41642	0.293628	0.8852	0.312827
ENSG0000	PDIA6	2:1072164	137.351	99.2981	114.203	124.544	160.345	184.488	187.131	114.609	171.113	150.065	0.396	0.289235
ENSG0000	EVI5L	19:783023	2.01189	1.99395	0.360733	0.082938	0.435748	1.89399	1.78959	0.236746	1.05238	0.451542	0.6996	0.289814
ENSG0000	CAPG	2:8539474	34.5516	51.6846	18.7995	38.4256	12.4978	357.932	261.357	203.023	417.667	163.635	0.00005	3.26264
ENSG0000	RP11-268	18:217049	0	0.469098	0.132695	0.359263	0	0	0.275441	1.45294	0	0.472699	0.37295	1.74167
ENSG0000	BPNT1	1:2200574	6.92277	5.73785	11.1903	5.73526	6.63557	5.21238	8.30483	3.08812	6.0266	6.66968	0.32665	-0.45998
ENSG0000	DCTN6	8:3009539	16.9257	23.8843	36.9141	33.8356	22.261	23.7404	24.7796	29.9789	35.6505	9.09253	0.9364	-0.14542
ENSG0000	MR1	1:1810334	28.0659	26.9084	43.9637	36.5964	26.9481	44.7172	43.1928	31.5902	46.3583	35.9823	0.642	0.174736
ENSG0000	NDUFS6	5:1801399	49.2792	40.471	87.2464	83.137	96.5954	73.1889	77.4164	80.4851	59.4843	20.9375	0.48545	-0.25326
ENSG0000	HGSNAT	8:4314045	7.34541	11.9079	6.71445	8.06491	4.96496	6.95815	8.72378	8.28939	4.5147	13.3344	0.89405	-0.07078
ENSG0000	DHRS4-A	14:239387	10.8293	11.5539	23.1445	18.1046	22.3978	6.94973	10.8993	10.9248	11.3188	18.538	0.20005	-0.72036
ENSG0000	IFI27L2	14:941277	55.4496	27.5725	68.0364	81.1467	30.9654	148.202	117.815	119.414	76.7678	19.0043	0.07215	0.77352
ENSG0000	CLPB	11:722906	4.17486	1.84705	3.799	2.08092	3.82906	7.96786	2.44532	1.58036	4.70651	2.44328	0.61245	0.332383
ENSG0000	DNAJC16	1:1552681	4.36645	6.72914	6.50446	3.529	3.37765	0.362941	2.11498	6.19015	3.29489	6.73887	0.4114	-0.51829
ENSG0000	SIRT7	17:819119	75.4437	81.2502	73.9024	79.2064	74.5946	83.1665	78.7926	72.3255	55.2123	91.1144	0.92185	0.033652
ENSG0000	CFL1	11:658230	1717.51	1857.83	1723.03	1496.8	1990.61	1751.6	1980.4	1871.66	1776.84	962.916	0.82675	-0.10901
ENSG0000	GSAP	7:7695983	27.6056	8.58844	8.62695	11.2982	7.25078	7.96368	3.13065	4.66104	4.47094	7.72118	0.0723	-1.32381
ENSG0000	KMT2E-A	7:1050134	23.5735	9.815	11.3364	17.138	5.62757	4.85356	17.981	6.86293	14.6773	4.07782	0.70175	-0.51223
ENSG0000	SPATA33	16:896444	2.89971	5.18765	4.8079	4.87433	4.81109	4.35999	6.50065	4.10022	5.35107	6.05344	0.79065	0.175414
ENSG0000	CD300C	17:745411	0.631783	6.70782	0.028625	0	0.315437	7.4397	0	1.01901	4.37055	0	0.4359	1.02635
ENSG0000	RP5-1050	17:496799	0	0	0	0	0	0	0.459647	0.361991	0.230141	0.139898	0.54475	1.23302
ENSG0000	CTD-2026	17:474505	1.20538	2.80532	3.03236	3.00767	1.35991	0.235563	1.37183	1.82836	5.99985	1.80311	0.99745	-0.00275
ENSG0000	ZFP69	1:4047721	3.8711	5.31133	3.11905	5.5867	3.86085	0.127612	1.74015	0.333717	3.89114	1.90636	0.04185	-1.4384
ENSG0000	PABPC1L	20:449100	14.8347	21.0786	10.7798	8.4124	3.39766	13.2886	13.0905	4.20725	8.42695	16.2109	0.95965	0.031626
ENSG0000	ALDH5A1	6:2440290	3.58707	3.08857	3.27408	0.685554	1.95348	1.78355	1.50809	1.14929	1.55326	2.74921	0.3751	-0.79994
ENSG0000	FBXO25	8:406427-	14.5724	10.3811	9.74949	10.0381	10.87	14.5877	11.4217	8.10797	6.80832	5.14975	0.47955	-0.33915
ENSG0000	C1orf162	1:1114737	50.4119	146.681	23.4249	26.8399	22.7228	140.901	19.1935	32.2873	35.5957	11.6474	0.8156	-0.10619
ENSG0000	ITM2A	X:7936038	343.029	331.008	314.471	183.312	429.177	490.731	310.664	328.585	323.983	277.722	0.82975	0.075683
ENSG0000	AF131215	8:1089604	1.41066	0.386971	0.556994	0.443139	0.660377	1.63635	1.32975	0.998666	0.519346	3.11905	0.62415	1.05239
ENSG0000	RPL15	3:2389165	888.771	697.824	1677.82	1447	1196.59	801.216	794.899	1188.35	1074.18	275.117	0.3973	-0.54972
ENSG0000	DNAJC8	1:2819945	160.285	140.352	173.647	143.016	132.358	189.691	200.101	123.83	100.482	69.1747	0.56105	-0.19136
ENSG0000	CES1	16:558028	1.22732	4.59453	1.82789	2.8247	1.26301	0	3.53449	0	2.11686	0.959498	0.82885	-0.27682

ENSG000C	LRPAP1	4:3506375	62.0252	52.958	118.979	80.7051	60.1476	130.726	82.0933	99.9966	77.3657	41.2731	0.94125	0.033235
ENSG000C	TRIM65	17:758803	15.8032	13.3938	9.13365	6.60337	12.4697	3.91415	3.07016	8.82953	6.66359	10.9477	0.02135	-1.01791
ENSG000C	FAM234A	16:234545	19.6226	36.3184	11.705	17.0582	9.63302	9.21525	8.73782	10.7523	11.7573	12.7313	0.0321	-0.78231
ENSG000C	MCF2L	13:112894	1.20372	3.76069	0.690134	0.353747	0.49921	0	0	0.884048	0	1.13877	0.13245	-1.69294
ENSG000C	TIMM9	14:583959	24.2834	21.3575	31.0112	22.2699	22.4486	20.2436	22.9308	17.2983	12.9367	6.76878	0.3401	-0.60979
ENSG000C	PDLIM7	5:1774833	6.10652	8.16581	4.00259	2.63947	5.17981	22.6529	21.6057	11.4182	8.22639	17.5767	0.03225	1.59011
ENSG000C	SWAP70	11:966407	1.29573	7.92202	0.762247	0.858184	1.26896	6.2244	0.502767	0.688791	0.045038	1.85598	0.6015	-0.38312
ENSG000C	AF127936.	21:148188	1.20176	0.299515	0.7736	0.600728	0.837682	0.007637	0.239124	0.415753	0	0.828399	0.4516	-1.62076
ENSG000C	PRPF4	9:1132215	16.4282	17.5387	16.9228	19.5203	12.4482	6.56135	14.0048	14.918	8.41218	13.4211	0.42445	-0.53628
ENSG000C	ACOT9	X:2370225	25.8805	17.8986	29.064	28.9503	17.9039	28.7396	41.3147	24.437	19.92	18.9273	0.8574	0.074774
ENSG000C	RLF	1:4016137	4.93356	6.54866	1.86515	1.82582	2.58819	5.15354	6.15735	2.6099	3.39894	5.72104	0.3632	0.38113
ENSG000C	AKNA	9:1143341	18.2548	19.862	21.8919	23.1832	32.9222	20.7247	18.9828	28.1151	31.769	59.0792	0.33725	0.38773
ENSG000C	TMOD2	15:517230	1.38506	2.27972	0.623337	0.316173	1.11873	0.085085	0.354063	0.731046	0.463717	1.34447	0.30115	-1.18435
ENSG000C	KAT2B	3:2004002	19.0775	12.0946	11.3038	13.2404	19.9011	26.8197	31.8364	15.3426	20.7965	34.0986	0.07475	0.780206
ENSG000C	RP11-126I	1:1513406	0.686111	0	0.634526	0.484627	0	1.06128	0.384114	0	2.27627	0	0.7603	0.471251
ENSG000C	DNAJC9	10:731103	17.667	28.0167	18.9005	18.8698	16.5311	25.7055	36.735	20.227	16.6018	17.1458	0.77475	0.166929
ENSG000C	INO80D	2:2059895	5.19033	6.804	4.48746	3.68342	10.9818	9.32808	7.82366	7.94773	9.78371	15.0593	0.56725	0.418532
ENSG000C	AC073410	2:2023769	0.101008	1.30618	0	0	0	3.58038	0	0	0	0	0.70325	1.40257
ENSG000C	VARS	6:3177751	32.8771	29.6912	9.26835	12.0351	10.5037	17.6011	16.9603	13.7966	10.1473	18.498	0.42265	-0.29786
ENSG000C	RAB11FIP4	17:313916	10.8959	13.0814	12.4921	11.9604	20.0096	17.8208	8.85534	14.2711	7.09436	22.7884	0.7704	-0.14297
ENSG000C	PLGRKT	9:5357972	53.335	41.7691	73.6298	56.3939	46.884	67.4801	60.1279	67.1451	59.1681	16.9708	0.94415	-0.02722
ENSG000C	BLOC1S4	4:6716054	58.4438	62.2878	112.41	90.3852	88.3763	49.2403	64.6413	57.8616	51.754	37.6399	0.0262	-0.73503
ENSG000C	ACTL6A	3:1795628	35.5634	40.59	23.6313	27.6485	23.4988	45.2666	34.3806	29.0057	9.76323	21.8181	0.8066	-0.10069
ENSG000C	AC004471	22:190362	0.538905	0.231613	0.080266	0.20641	0.145269	0.346628	0.563639	0.164362	0.201312	0.486235	0.64935	0.553048
ENSG000C	SON	21:335039	245.518	299.828	324.465	352.154	442.846	438.417	460.364	165.69	232.268	251.245	0.8618	-0.21597
ENSG000C	LGALS9B	17:204493	0	2.80026	0.123441	0.018439	0.293536	0.033553	4.45249	0.032142	0	0.044684	0.91835	0.14409
ENSG000C	RBM48	7:9252877	16.7081	22.0292	32.5143	23.4368	25.1752	14.9123	21.8573	14.1412	15.5853	23.5013	0.23435	-0.47301
ENSG000C	STARD3NI	7:3817822	59.5106	30.0975	63.0396	48.3926	29.7652	55.3248	39.1436	44.6146	44.8133	22.9608	0.5992	-0.17739
ENSG000C	TMEM189	20:500811	2.06312	5.56308	1.91257	0.919893	2.70036	6.5491	2.7019	4.24534	2.08951	1.61268	0.76215	0.454867
ENSG000C	XPO4	13:207773	4.18657	6.17075	3.15451	3.09463	1.36727	6.0726	5.8189	1.61434	3.36359	3.51309	0.8497	0.176551
ENSG000C	FAM71E1	19:504667	0.176079	0	0.066001	0.001033	0.14182	0.220613	0	0	3.42066	0.68253	0.03155	3.6291
ENSG000C	VAMP5	2:8558440	77.5922	63.0441	146.141	148.282	103.482	150.35	92.6341	123.776	89.899	38.1065	0.54465	-0.21545
ENSG000C	RP11-402.	4:1125135	0.305401	0.687063	0.350746	0.462952	0.238886	0.134105	0.450884	0.125109	0.324259	0.149284	0.623	-0.68123
ENSG000C	KIF20A	5:1381397	1.0069	3.59921	0	0.316694	0.012701	0	1.32234	0	0	1.8845	0.65155	-0.80543
ENSG000C	ZNF322	6:2663438	3.93812	4.56893	2.12222	0.797279	2.45332	4.97398	3.18797	1.3234	2.08107	2.51635	0.32095	-0.62626
ENSG000C	LRFN3	19:359353	0.326266	0.206484	1.72289	0.438901	3.33842	0.071715	0.279377	0.961332	0	8.18485	0.84305	0.428597
ENSG000C	SLC25A27	6:4665291	0.12913	0	0	0	0	2.44514	0	0	0	0	0.1709	4.15444
ENSG000C	TSPAN33	7:1291448	2.06332	9.14848	0.289397	0.665219	1.14417	11.1918	8.55854	1.3503	0	1.78475	0.3945	0.75955
ENSG000C	SNHG8	4:1182787	57.4918	35.1434	95.7624	138.684	42.7876	68.5843	51.4352	68.4372	73.0078	27.7535	0.38965	-0.37372
ENSG000C	NMNAT1	1:9922112	2.01785	2.36548	3.09134	2.56859	2.75475	0.768868	2.98076	2.10734	4.46838	2.05944	0.72225	-0.33141
ENSG000C	HBB	11:522546	0.431197	0	230.791	3737.14	0.540447	0.392538	0.053628	13.1701	667.329	0.915958	0.1429	-2.38821
ENSG000C	ZNF431	19:211420	6.75378	9.40994	1.99356	3.64085	1.68357	4.61638	4.76297	3.41025	3.28357	2.98706	0.6197	-0.41931
ENSG000C	CAMKMT	2:4436194	9.83959	12.3373	10.3112	11.4584	9.1623	10.8808	14.9146	6.91325	6.57651	8.30665	0.917	-0.06858
ENSG000C	RN7SL1	14:495709	0	0	0	0	0	74.348	0	0	0	0	0.85525	0.345858
ENSG000C	CCDC7	10:324460	6.72989	20.5433	3.26441	6.4619	3.29916	20.4491	14.4019	1.19501	8.75009	7.10433	0.73465	0.312121
ENSG000C	GBA2	9:3569694	38.3362	34.0992	12.7225	11.1653	17.3783	36.5363	26.2703	10.0168	8.29167	26.2004	0.97185	-0.01992
ENSG000C	TMEM265	16:306977	2.67153	1.0622	2.21728	3.86156	6.00128	2.66863	5.53196	4.78385	10.1797	12.0945	0.3943	1.24624
ENSG000C	NAPB	20:233745	3.99325	5.81359	2.96822	2.44681	2.30298	5.65349	7.00534	1.8298	4.58874	5.81269	0.3835	0.448497
ENSG000C	NLRP2	19:549235	4.63414	0.955455	3.37857	2.6618	3.08583	5.6032	5.2957	3.49646	8.96303	7.75683	0.0416	1.08021
ENSG000C	KIF5B	10:320090	29.8939	31.6387	22.5729	22.357	26.0746	38.0959	42.4064	23.6088	28.2609	49.9814	0.23665	0.364166
ENSG000C	ALDH9A1	1:1656622	52.3858	41.4988	51.4116	59.8477	50.4159	33.3326	34.2604	36.1107	59.1537	44.284	0.35515	-0.29052
ENSG000C	CD55	1:2073215	48.9482	65.1206	56.34	62.7075	48.5717	11.9511	50.7827	28.5093	61.007	22.3257	0.0459	-0.76557
ENSG000C	RPL29P11	3:3699333	2.0443	1.0416	1.68906	1.77957	0.397111	1.71279	1.75914	0.640274	1.14082	0.149098	0.888	0.210021
ENSG000C	TMEM110	3:5281297	1.3549	1.7846	2.13713	0.540048	2.86308	4.54305	2.25126	1.0743	3.14868	4.51651	0.65405	0.628013
ENSG000C	AC006942	19:497669	0.122066	1.24647	0.441416	0.717543	0.097019	0	0.288018	1.26236	1.99533	0.276473	0.6901	0.785892
ENSG000C	GSPT2	X:5174343	7.69451	9.85277	8.48661	3.28242	9.91763	6.16858	6.27879	8.26069	1.56304	8.41191	0.4688	-0.36362
ENSG000C	SERPINE1	7:1011270	0.14135	0	0.021843	0.111249	0	1.61349	0	0.380211	2.30848	0.622489	0.02035	4.20977
ENSG000C	RP11-141(	16:222062	0	1.78761	0.660369	0.547729	1.13399	0.299239	1.69537	1.31529	0	0.807044	0.92615	0.118222
ENSG000C	IL1R2	2:1019918	1.58757	3.078	0	0.427512	0	21.0866	78.5146	2.02331	0	0.6792	0.01685	4.62475
ENSG000C	CSF3R	1:3646604	9.55363	28.0082	1.24378	2.0672	0.760617	39.5821	2.18027	2.01285	0.96521	1.56448	0.81835	0.16531
ENSG000C	KLRC4-KLI	12:103637	69.0609	82.8205	41.6837	35.6856	56.98	62.4123	134.208	67.7924	16.0873	119.204	0.54545	0.584548
ENSG000C	JRK	8:1426574	2.57581	3.6929	1.53993	1.39505	1.20983	1.06149	3.3905	2.26045	0.148388	5.59353	0.9715	-0.02904
ENSG000C	METTL3	14:214590	42.9248	65.8049	50.0457	61.0635	39.7018	38.3476	33.9323	55.9779	51.8709	61.8164	0.8292	-0.09756
ENSG000C	FAM66C	12:812363	1.89944	2.58554	1.60403	0.651789	0.932815	0.038803	1.7572	0.372911	0.328309	1.12445	0.1687	-1.2601
ENSG000C	VBP1	X:1551970	82.702	51.3959	52.8769	56.7273	49.2997	61.1158	90.8865	52.9351	42.6874	24.196	0.99775	-0.00095
ENSG000C	RP11-148(	8:1149400	0.812237	0	0.790072	0.311895	0.075939	0.470566	0	0	0.641914	0.092752	0.72225	-0.64297
ENSG000C	ERLEC1	2:5353267	42.8019	24.4729	26.3975	34.3323	26.0129	39.735	27.4117	24.708	27.5596	19.4345	0.587	-0.21611
ENSG000C	CARD8	19:481706	63.0837	55.2008	62.5112	64.6904	36.5096	58.6286	40.3545	50.8032	38.7072	58.4518	0.36	-0.39431
ENSG000C	RP11-672I	18:813273	1.54075	0.607988	0.320558	1.11307	0.557885	4.62088	0.456447	0.855074	1.74692	0	0.61915	0.900784
ENSG000C	DEF8	16:899479	22.9279	18.4215	35.6294	34.1233	44.9923	41.3297	22.6218	19.1085	51.3526	34.5344	0.90475	0.069056
ENSG000C	STRN3	14:308745	1.37035	2.32068	4.99342	3.35613	4.34386	1.3618	1.37672	4.56254	2.97519	7.0131	0.7876	-0.16683
ENSG000C	MTMR11	1:1499286	1.13553	6.74432	0.293971	0.079051	0.098069	0	0.088266	0	1.12954	0.225289	0.146	-2.41623
ENSG000C	SQRDL	15:455871	92.0451	60.846	111.27	111.658	91.2253	105.93	95.6392	103.909	102.399	60.278	0.8992	-0.04648
ENSG000C	AC093673	7:1433796	13.9111	6.98128	15.112	16.2253	8.68524	14.5199	26.4318	18.0004	24.6178	11.113	0.2318	0.617396
ENSG000C	AKR1C3	10:503535	2.83666	3.35398	5.9534	7.9064	2.72422	0	0	0	0	0.932526	0.2204	-4.90588
ENSG000C	RP11-467I	2:7093588	1.74558	1.33013	2.55768	0.464943	1.00486	0	0.845902	1.03624	0	0.427686	0.2936	-1.65835
ENSG000C	CCT2	12:695853	99.4535	98.9153	120.65	122.083	151.002	90.6597	103.4	104.022	84.2194	85.8613	0.3208	-0.31275
ENSG000C	GTF2A1	14:811754	2.42943	4.06647	1.67376	2.20695	2.59459	3.32123	9.44183	2.94085	0.927909	6.35155	0.39475	0.546087
ENSG000C	IFNLR1	1:2415415	0.320322	0.485998	0.582862	1.17496	0.734246	0.010172	1.61137	1.11237	1.46058	2.04776	0.2961	0.941808
ENSG000C	RP11-96D	16:679873	0	0	0.398035	0.809691	2.001	0.474005	2.288	0.921018	2.77974	2.56696	0.67065	1.42054
ENSG000C	LCN10	9:1367381	0.132578	2.00085	0.248108	0.318421	0.152889	0.504689	0.334773	0.082855	0	0.03294	0.49625	-0.76751
ENSG000C	RPH3AL	17:212388	2.13153	1.80583	0.855498	1.24346	3.40696	0	2.86294	1.46566	0	2.59007	0.18705	-0.98413
ENSG000C	SPP1	4:8797564	0	0.152747	0	0	0	232.724	0.096275	0.028546	0	0.044892	0.22165	11.4206
ENSG000C	C2	6:3189778	0	0	0.065487	0	0.068338	10.9506	0	0	3.07066	0	0.0813	6.42108
ENSG000C	URB1-AS1	21:323931	9.15581	6.01418	12.222	5.06249	6.20955	4.86508	6.63804	5.30967	6.09533	3.83035	0.51285	-0.5559
ENSG000C	IGLV2-11	22:227924	0.380193	87.0317	0.792386	1184.31	0.397636	0	0	0.10536	0.529612	0.128576	0.0323	-10.299
ENSG000C	C1orf21	1:1843870	16.6349	4.46448	9.65262	7.96837	12.0881	9.40991	1.88747	1.99799	1.37304	5.64098	0.2124	-1.47301
ENSG000C	MMAA	4:1456182	3.36291	8.41704	2.88278	4.1464	3.65389	12.1385	6.14308	2.64281	3.65176	2.55866	0.94245	0.068678
ENSG000C	HOXB-AS:	17:485408	0.859148	6.68258	3.71901	3.35083	2.42684	0.520349	15.7703	0.622883	2.79301	5.19062	0.756	0.4641
ENSG000C	FTSJ3	17:638194	30.8855	24.2755	22.3539	17.6482	21.4228	7.78039	13.907	19.7285	15.634	29.0555	0.56225	-0.43781
ENSG000C	HIST1H3E	6:2622419	2.32819	0.598342	1.33096	0	0.336064	0	1.65705	2.27858	2.31046	2.00611	0.6353	0.902957

ENSG0000(	FLJ37035	10:125683	1.77964	0.78178	2.72231	0.883532	2.1317	0	0	0	0	0.026312	0.00055	#NAME?
ENSG0000(	CPM	12:688081	1.67449	5.68551	0.232335	0	0.056517	27.2966	1.7311	0.072512	1.01561	0.154976	0.174	1.99149
ENSG0000(	ATG4D	19:105438	24.2547	17.0521	33.0058	26.8091	31.3854	22.4434	24.9657	18.9539	6.56202	29.4997	0.4563	-0.41025
ENSG0000(	RP11-85G	10:272431	0.044232	0.060894	0	0.378969	0.26558	0	0.135543	0.043394	2.76354	0.15719	0.22945	2.36128
ENSG0000(	RPS14P4	2:1111959	0.327437	0.417502	0	0	0	1.01619	1.99881	0	0	0	0.36715	2.38864
ENSG0000(	XXbac-BP	6:3281276	0.075507	0.603406	0.073391	1.3539	0.084199	0	0.077293	0.009785	3.48842	0.065173	0.6579	-1.39262
ENSG0000(	LINC0126(	20:446564	1.38284	0.513136	3.24368	3.01471	1.22598	0.478037	2.9397	5.1054	0.263128	1.15735	0.895	-0.24118
ENSG0000(	RP11-109	17:355481	0	1.75172	0.285663	0	0.09879	0	0	0.179152	0	0.743615	0.74435	-0.54126
ENSG0000(	POMC	2:2516085	20.9139	6.45193	25.0077	15.2035	13.2244	20.393	14.8694	21.4258	10.86	6.11816	0.6607	-0.2591
ENSG0000(	ATP2A1-A	16:288784	1.2268	0.515347	1.63314	1.79557	0.093186	0	0.446798	0.316099	1.6485	0.348168	0.6177	-0.8764
ENSG0000(	MED12	X:7111855	8.01753	6.19351	1.37555	0.713107	3.46625	2.63609	3.35194	2.79405	2.03212	5.06972	0.87355	-0.07643
ENSG0000(	ZNF273	7:6487017	3.28315	4.01583	1.76658	0.852844	1.31612	5.90453	4.18762	1.83527	1.75104	0.849279	0.58845	0.461956
ENSG0000(	RP11-649	17:752713	2.31113	0.562661	0.445048	0.095772	0.568442	3.31721	0	1.47095	3.19722	2.17425	0.36195	1.2191
ENSG0000(	DUSP28	2:2404794	14.1951	12.0293	22.7105	16.5904	20.1327	20.9244	17.6917	17.6165	8.77247	15.4573	0.8482	-0.24131
ENSG0000(	ZNF460	19:572672	1.90109	4.42133	0.407722	5.68338	1.12895	0	0.448074	1.57679	2.8559	3.85999	0.5643	-0.63362
ENSG0000(	RN7SL301	17:361101	0	0	0.149556	0	0	0	0	2.1063	0	0	0.26235	3.96862
ENSG0000(	MRPL18	6:1597898	74.6758	62.1922	88.1068	75.922	68.9177	94.1915	68.649	63.5824	63.1139	36.8104	0.47855	-0.24021
ENSG0000(	IGSF6	16:215972	2.09715	5.23501	0.301324	0.478155	2.46678	34.8304	0.857042	1.78674	10.4536	2.9572	0.11495	2.23949
ENSG0000(	RP11-430	11:453553	0.776121	1.54772	3.58844	1.1352	0.367946	0	0.743172	5.83417	0	0.780275	0.87575	0.232813
ENSG0000(	SCLT1	4:1288096	12.2781	9.95746	3.07526	4.09736	3.90713	9.31984	24.2866	9.52846	1.67247	7.62424	0.42005	0.725031
ENSG0000(	IGHV1-3	14:106005	0.258912	1.86603	1.22358	0.664739	0.718212	0.574516	3.94735	5.87571	4.42971	1.53751	0.4986	1.92979
ENSG0000(	CHAC2	2:5353267	10.9398	6.38999	7.20879	8.50294	4.51111	11.9329	12.1628	6.2837	7.57671	2.25919	0.9082	0.18302
ENSG0000(	B9D2	19:412624	10.8881	15.3953	19.42	31.1636	21.827	23.1781	24.6588	15.9212	21.8907	4.70437	0.8609	-0.23954
ENSG0000(	PCSK7	11:117199	77.6265	95.7625	48.722	59.8804	38.7008	56.0845	48.1983	37.115	39.8102	68.3169	0.1659	-0.48906
ENSG0000(	CYTH4	22:372820	33.3681	42.3318	32.543	33.0518	42.1266	30.547	31.9337	36.1033	27.3066	62.6943	0.94745	0.025344
ENSG0000(	HIST1H2A	6:2789038	7.52786	1.86334	10.0125	2.78522	0.825112	4.91134	5.85284	5.18487	6.07935	1.91447	0.9207	0.017836
ENSG0000(	SPATA9	5:9564675	0.17108	0.120776	0.148326	0.181049	0.253472	2.19258	0	0	0.231249	0.164388	0.30305	1.43799
ENSG0000(	LINC0131	22:191713	1.37482	2.73585	0.843956	1.52405	1.822	2.5373	5.11472	3.77713	0	3.22296	0.2953	0.762463
ENSG0000(	RP11-181	1:2050469	0.696554	1.4459	0.395705	0.561173	0.972745	0	0.354475	0.353197	0	1.17122	0.41955	-1.07446
ENSG0000(	ZFAT	8:1344777	1.68164	1.6606	1.54219	1.1247	1.39773	0.054599	2.31147	4.67521	1.45498	2.32187	0.31515	0.684004
ENSG0000(	BCL2L14	12:120498	1.15454	0	0	0	0	2.92483	12.3696	0.239438	0.202336	0.289074	0.0076	3.69644
ENSG0000(	BORCS7	10:102845	32.4386	31.6838	39.2047	63.7963	44.7109	22.1542	31.0114	19.5079	53.6995	15.6269	0.1373	-0.64247
ENSG0000(	GNPDA1	5:1419917	20.6194	16.0954	26.0558	31.4535	31.9853	18.0868	7.14422	12.4036	11.155	14.6764	0.00595	-1.0518
ENSG0000(	RP11-144	3:1013242	1.23089	3.85475	0	0	0	0.468514	1.969	0	3.23008	0	0.87085	0.411571
ENSG0000(	RP11-632	15:325198	4.06293	6.5178	4.68548	6.60871	5.29051	0.848525	2.75316	3.5739	3.58472	6.64842	0.4613	-0.64859
ENSG0000(	UBE2E1	3:2380402	3.93168	2.90384	5.32449	17.7618	3.70091	7.39996	5.32685	5.68682	21.8442	3.35365	0.48475	0.410031
ENSG0000(	SGMS2	4:1078245	0.864938	2.93504	0	0	0	0	0.355796	0.071443	1.97693	0.006238	0.9329	-0.11239
ENSG0000(	AGBL3	7:1349865	0.433565	2.44879	0.509987	0.773647	0.375727	1.15755	0.203185	1.09253	0.016666	0.136055	0.63475	-0.61387
ENSG0000(	RP11-677	11:124766	1.37246	0.471751	0.096937	0.25599	0	0.446063	0.664351	0	0	0	0.60065	-1.03542
ENSG0000(	AGO2	8:1405201	7.29394	5.03816	5.19643	4.65585	4.18843	4.54783	10.4987	11.4739	10.6954	17.5215	0.1237	0.886802
ENSG0000(	SHPRH	6:1457935	8.85559	12.3244	4.27367	3.21693	4.59258	7.81048	7.81017	3.70819	5.0663	11.5919	0.80575	-0.12394
ENSG0000(	MRPL22	5:1549410	54.1291	42.6683	83.4027	56.3969	50.8964	100.077	90.6475	54.7454	50.4339	22.4076	0.89415	0.053218
ENSG0000(	OLR1	12:101583	0	0.15777	0.197249	0	0.372888	102.738	2.82615	1.70102	5.3525	2.8381	0.00985	7.08234
ENSG0000(	LEPR	1:6542058	1.08668	0.65165	0.249389	0.423927	0.134325	0	0.886822	1.39414	0	1.03842	0.64195	0.500854
ENSG0000(	CTC-534A	5:6558967	10.0563	2.77975	8.90298	11.8462	3.55612	4.80677	1.92382	9.52857	3.07041	4.42556	0.57775	-0.68105
ENSG0000(	CCDC117	22:287726	11.2375	12.7692	4.43883	4.32886	5.21523	5.79153	10.3416	7.81322	0.618534	9.68266	0.43015	-0.41885
ENSG0000(	RP11-326	11:318639	0.778912	2.0168	0.263979	0.421493	0.531274	1.32032	0.807574	0.749509	0.387291	0	0.82385	-0.30739
ENSG0000(	SENP8	15:718183	1.14456	0.625146	1.25499	0.577016	0.959324	3.19462	0	2.10883	2.58354	0.063128	0.6757	0.663448
ENSG0000(	GTF2A2	15:595948	61.4577	50.9033	81.1384	80.292	28.9129	84.3733	68.5837	84.7867	69.6291	24.827	0.8004	0.126624
ENSG0000(	RP11-262	9:6285699	0	5.98931	13.9048	0.161115	0.161647	0.411027	7.08625	9.86994	0.128193	0.114061	0.85115	-0.27309
ENSG0000(	COMMD3	10:223159	105.712	87.6524	90.266	103.92	54.5953	173.589	115.224	83.1919	79.6664	34.6304	0.7939	0.133014
ENSG0000(	CD86	3:1220553	1.71096	13.2374	0.127033	0	1.23446	26.513	6.21208	2.33911	0	1.80841	0.3782	1.1196
ENSG0000(	RP11-289	1:1452330	0.045443	0.347506	1.50746	1.45039	2.10621	0.340222	0.295244	1.86608	1.40776	1.34476	0.973	-0.04899
ENSG0000(	LRRC25	19:183911	3.42764	24.6093	1.10293	0.498179	2.71489	17.4009	0.087482	3.36472	1.74262	0.41114	0.6246	-0.38986
ENSG0000(	LRRC47	1:3778557	17.4505	21.1069	17.6203	15.5341	24.9867	21.3441	24.381	17.3141	17.4911	25.2574	0.68935	0.143579
ENSG0000(	PCMTD1	8:5181757	25.6758	38.1068	16.9652	17.8275	24.4653	13.2891	11.5249	17.9699	28.7328	24.7225	0.50425	-0.33587
ENSG0000(	FUNDC1	X:4452363	20.6073	15.6202	18.7645	16.7272	19.2365	14.635	15.8833	12.0845	13.4621	8.39121	0.274	-0.50913
ENSG0000(	TAF12	1:2858932	72.1089	54.1907	67.7143	69.4738	46.6639	79.802	53.5698	51.3212	43.7321	19.9034	0.33645	-0.33448
ENSG0000(	ALKBH2	12:109088	11.6384	26.0398	23.0331	17.1739	17.4174	4.83757	8.75115	8.02392	2.08599	8.49698	0.05145	-1.63816
ENSG0000(	C12orf4	12:448772	11.441	9.94609	3.16176	3.93124	2.68038	11.2419	6.61928	5.94176	8.21309	5.92046	0.6278	0.222081
ENSG0000(	IRGQ	19:435430	1.71017	1.00607	0.542651	0.309322	0.93373	2.7618	1.61966	2.12785	2.07992	2.70331	0.3591	1.16176
ENSG0000(	RAB11FIP1	X:7394432	0.872	1.72785	6.52887	2.45366	5.80206	0.685763	4.38092	3.72111	5.32478	15.9404	0.68285	0.631449
ENSG0000(	TPP2	13:102597	38.4254	42.5347	8.81775	14.4947	10.4262	21.5533	15.1255	13.5201	28.4888	15.8085	0.98945	-0.00501
ENSG0000(	CTD-2192	19:123791	1.08654	1.29806	1.50945	0.049197	0.662924	3.4465	1.10675	0.3831	0.273534	2.43746	0.4807	1.27484
ENSG0000(	ZNF845	19:533306	6.71317	9.47755	1.80811	3.81838	1.37434	1.32808	3.29806	2.18987	2.06703	2.58072	0.14755	-1.04226
ENSG0000(	MFSD4A	1:2055688	0.711295	2.46826	0.678303	0.545209	0.5594	0.013909	1.43521	0.118923	0	0.82733	0.254	-1.14682
ENSG0000(	MED6	14:705812	33.7775	41.7028	26.176	28.826	22.202	23.8224	45.4944	37.2644	14.9052	20.3613	0.7152	-0.15231
ENSG0000(	CARNMT1	9:7495296	11.4219	13.2996	3.49716	4.20208	5.12448	5.85895	6.02325	7.00982	0.814755	8.04562	0.35735	-0.40465
ENSG0000(	RP11-190	3:1194976	0.315317	0.203225	0.225845	0	0	0	0.476198	0	2.49809	0.414633	0.17795	2.45558
ENSG0000(	E2F2	1:2350642	3.48255	4.13893	0	0.511078	0.436989	0.595124	0.852222	0.316492	0	1.95997	0.3954	-1.29187
ENSG0000(	MORN3	12:121648	4.98618	3.92002	5.18097	7.11013	2.70088	1.59685	1.33012	4.18501	8.91202	2.2822	0.7725	-0.30488
ENSG0000(	MYBL1	8:6656217	54.0046	18.823	30.863	53.6557	55.9518	6.70913	6.93091	9.03935	37.7671	31.5246	0.10825	-1.18537
ENSG0000(	RP5-1056	1:1959697	0	0.407392	0.293068	0	0.309897	1.77136	1.39721	4.10804	1.89322	2.09024	0.0631	3.53796
ENSG0000(	RPP21	6:3023403	34.5665	37.0025	68.7455	51.3262	44.1225	60.8492	57.7334	52.9496	35.6051	30.4438	0.93515	-0.06189
ENSG0000(	AP3S1	5:1158281	47.9643	32.4053	55.6255	61.0944	38.7067	50.3416	55.6397	61.3752	34.463	18.6939	0.77215	-0.14971
ENSG0000(	ZNF561-A	19:960467	3.61306	3.05235	3.8163	4.69416	3.49072	1.61576	3.1643	2.61071	0.593342	1.53777	0.205	-0.98971
ENSG0000(	ZNF598	16:199765	6.39522	3.35373	1.77892	1.22314	5.96871	4.94684	6.27189	2.90761	0	5.14694	0.90725	-0.08067
ENSG0000(	MAN2B1	19:126432	34.4771	69.084	28.2234	29.7943	32.5282	66.4672	28.0297	29.5462	15.3739	45.0033	0.8528	-0.16758
ENSG0000(	IL18R1	2:1023115	42.4133	8.54221	20.2505	44.486	13.997	29.0763	20.3027	13.3397	23.5337	24.8515	0.5172	-0.2411
ENSG0000(	ZNF19	16:714475	0.784583	0.658625	1.88564	0.729562	0.691529	0.734416	0	0.963939	0	0.665098	0.10825	-1.27722
ENSG0000(	AC093616	2:8770098	4.72809	4.57008	3.04428	3.74773	2.0387	3.92576	6.12799	5.83083	3.46737	4.83329	0.22525	0.996753
ENSG0000(	AK4P1	17:310084	0.072613	0	0	0	0	3.5299	0	0	0	1.07218	0.33455	6.05779
ENSG0000(	TUBB1	20:590192	0.143809	0.298006	2.22571	7.0085	0.628806	0	0	0	0	0	0.04145	#NAME?
ENSG0000(	GOPC	6:1172882	25.9044	17.7593	7.27156	5.57726	8.20037	12.7438	10.9589	6.72373	4.20345	14.1447	0.33385	-0.38655
ENSG0000(	RP11-134	12:142165	2.04261	0.322387	2.55438	2.74391	3.19787	0	0.687205	3.01202	0	3.85063	0.7803	-0.43343
ENSG0000(	KRT72	12:525855	1.84445	3.88397	0.427332	2.05051	0.089067	0	0	0	0	0	0.11915	#NAME?
ENSG0000(	AC013264	2:1969670	27.3083	16.3573	33.1314	20.3129	17.3641	12.1809	10.9415	6.52609	13.2697	2.63675	0.4261	-1.33466
ENSG0000(	CTD-3096	18:531039	0.613712	0.602215	2.21872	0.145005	0	3.25764	0.223709	0.832232	0	0.872756	0.7008	0.571166
ENSG0000(	NSMAF	8:5858350	45.5987	42.4517	14.6669	17.279	7.80607	43.6843	24.1515	16.5827	15.432	17.267	0.9562	0.018063
ENSG0000(	MFN2	1:1198018	17.2328	19.999	7.77083	5.20432	6.8085	10.0616	8.79407	8.78494	2.10792	13.8977	0.23845	-0.4486
ENSG0000(	RNF207	1:6181268	2.97591	3.40707	0.227399	1.8956	0.584821	10.3165	3.13729	0.791692	6.53552	2.38203	0.13295	1.49353

ENSG0000(	PRELID1	5:1773011	171.304	124.858	207.422	241.472	228.899	293.812	273.748	230.654	177.211	114.766	0.86265	0.068046
ENSG0000(	MRPL57	13:211766	43.7653	32.2799	54.6999	48.4809	35.2686	52.4345	36.9354	38.7384	29.7675	14.1118	0.30655	-0.35318
ENSG0000(	ZNF529-A	19:365106	2.77506	0.52737	1.42364	1.16517	1.25992	1.74317	1.9508	0.206397	0.152082	1.50489	0.7138	-0.40921
ENSG0000(	USP22	17:208146	6.87111	10.789	8.31933	7.35685	11.4699	6.28884	8.6153	18.1204	10.8672	28.2246	0.21895	0.475052
ENSG0000(	RP11-554	10:661871	0.372956	1.36237	0.16188	0.67197	0	0	0.827866	0.470917	0	0.513771	0.93445	-0.13554
ENSG0000(	TMEM169	2:2159428	1.43732	1.58998	0.186294	1.3907	0.398678	0	0.98792	0.374185	2.77066	1.26028	0.9979	-0.00415
ENSG0000(	RP11-731	3:1368186	0.190906	0.450967	0.188087	0.132802	0.079597	0	0	0.170514	1.88164	0.208605	0.64715	0.922669
ENSG0000(	EXOC3	5:441497-	9.91125	13.523	11.9756	7.47544	9.50315	11.8443	10.9428	10.3359	1.39597	12.3328	0.5925	-0.29489
ENSG0000(	FHIT	3:5974930	2.17066	26.3601	1.02162	0.699012	1.0538	0	0.517698	0.271997	0	0.306289	0.0126	-5.09672
ENSG0000(	TCTEX1D4	1:4480022	0.878331	0.270313	0	0.531301	0	1.0132	0	0	2.91026	0.095902	0.57565	1.51017
ENSG0000(	CEBPE	14:231173	0	0.370171	0	0	0.07599	0.072034	0.801563	1.4854	1.46844	0	0.1517	3.42601
ENSG0000(	C19orf66	19:100861	76.9136	66.9373	64.4297	54.6406	70.6052	85.1231	139.226	75.6819	44.574	68.358	0.4509	0.232375
ENSG0000(	SLC46A3	13:287000	20.6331	20.2312	7.0671	11.1637	5.94132	11.4077	10.268	8.26102	11.5178	11.4648	0.63315	-0.21254
ENSG0000(	PIKFYVE	2:2082662	3.78566	5.20214	1.34537	1.55035	1.48682	2.11932	2.42353	1.06531	2.10524	3.38368	0.44205	-0.34764
ENSG0000(	C1QL3	10:164369	0.794564	0.653863	1.32808	0.389981	0.39294	1.49354	1.08063	1.52197	0	1.56273	0.6366	0.815254
ENSG0000(	CKMT2-A	5:8094769	6.10048	4.32386	6.48114	5.45837	4.67608	3.69893	5.81446	3.86365	4.14389	4.61538	0.77705	-0.26574
ENSG0000(	EHMT1	9:1376056	35.6598	43.213	24.2969	15.9145	66.5276	28.3177	36.2708	16.582	21.6565	57.675	0.5946	-0.29336
ENSG0000(	GSN	9:1211781	3.93153	19.0458	1.99337	5.3655	1.72313	26.363	3.8061	5.55255	4.53423	1.83602	0.66205	0.460904
ENSG0000(	POLR2J2	7:1024824	22.1966	39.6088	23.5332	26.2172	19.2886	37.0241	30.0575	29.4769	25.3435	20.2616	0.9155	0.187358
ENSG0000(	SCYL3	1:1696620	7.68597	7.09659	4.81427	4.6545	5.09348	12.3737	4.12916	9.39356	3.55529	8.83702	0.9043	-0.09473
ENSG0000(	RP11-701	15:248236	0	1.08527	0.160355	0.224093	0.351359	0	1.31775	0	0	0.386363	0.9432	-0.00182
ENSG0000(	AC010894	2:1744873	0.554179	1.21552	0.435246	0.370346	0	0	0	0	1.14604	0.193502	0.80435	-0.40616
ENSG0000(	C1orf50	1:4276726	25.3289	18.3917	32.8659	18.7756	20.3762	36.3682	23.3334	33.7623	45.748	8.20296	0.57045	0.303333
ENSG0000(	NBL1	1:1959697	0.848274	1.41349	0.995192	0.454363	0.88209	5.15584	42.5317	14.9481	3.10442	4.54727	0.0059	3.79536
ENSG0000(	RP11-110	12:520228	0.091956	0.382627	0.319571	0.83831	0.467137	0.051249	3.0907	3.02162	5.16963	0	0.05675	3.12845
ENSG0000(	LINC0153	19:366854	4.0767	7.69826	2.84051	2.93612	2.34962	7.36207	6.93615	5.6711	9.4822	4.40296	0.3021	0.974897
ENSG0000(	C18orf32	18:494772	89.7039	57.2093	127.488	112.768	61.5172	126.214	133.959	86.5172	107.939	26.4442	0.95585	0.039034
ENSG0000(	THUMPD2	2:3973605	10.9639	8.10481	6.79798	11.0143	10.3517	10.2152	11.8876	5.2746	6.07209	5.75802	0.64535	-0.24365
ENSG0000(	KAT7	17:497009	19.5589	23.3396	10.1245	11.904	11.3364	6.88071	16.9592	16.9563	7.62984	13.5113	0.24975	-0.58841
ENSG0000(	SMARCA2	9:1980289	51.3266	53.8107	21.3674	24.7863	40.5848	42.9222	31.2389	29.1414	32.86	82.9567	0.35915	0.365621
ENSG0000(	CFL1P1	10:877515	2.04108	4.0407	3.31238	1.51695	1.17805	3.11012	2.92512	1.89135	0	0.554227	0.53935	-0.60886
ENSG0000(	COX10	17:140694	17.9237	16.6004	17.9733	18.5454	18.7709	16.0338	8.58959	14.1017	17.5853	18.9376	0.4787	-0.32541
ENSG0000(	ATL3	11:636240	7.73611	6.95051	4.18977	3.43912	4.89923	3.49707	5.89873	6.18393	1.44731	8.43104	0.52515	-0.24843
ENSG0000(	NDUFAF7	2:3716781	21.3126	15.1888	16.0667	13.1933	9.83093	26.776	9.43182	15.9658	10.2698	13.288	0.98385	-0.01572
ENSG0000(	CHURC1	14:649143	64.608	77.7653	77.9477	70.4513	58.3711	63.0351	69.615	89.3279	52.1652	67.2736	0.83715	-0.0983
ENSG0000(	EOGT	3:6897521	4.19074	4.28343	0.855578	1.58939	1.68403	3.60148	5.60995	1.85263	4.12447	3.07638	0.44035	0.594374
ENSG0000(	LINC0062	1:1209131	25.0584	12.8232	15.2954	12.855	16.6021	16.4388	23.4588	11.374	11.7755	9.06892	0.67495	-0.32133
ENSG0000(	KATNB1	16:577357	25.5215	20.8451	23.1075	24.1615	20.4112	28.4826	42.1029	17.224	22.202	23.0384	0.6758	0.143807
ENSG0000(	VNN2	6:1327438	16.6848	29.4452	7.50941	12.7996	5.9336	13.9709	4.94691	11.2447	5.82479	6.64293	0.1297	-0.68004
ENSG0000(	ZDHHC23	3:1139479	0.264007	0.470601	0.061327	0.389661	0.080892	0.845119	0.983576	0.13163	0	0.827355	0.3685	0.940603
ENSG0000(	C10orf35	10:696302	13.9044	4.19322	12.2677	12.5389	9.48399	4.57615	8.92141	8.69319	0.869141	5.4088	0.16645	-0.97352
ENSG0000(	FAU	11:651206	285.381	172.173	380.609	383.751	222.945	275.733	307.61	358.666	232.849	47.2873	0.6116	-0.30246
ENSG0000(	RP11-295	10:135869	5.76119	13.2696	4.94622	4.59805	3.37749	2.09904	8.31767	6.32496	11.9234	11.1482	0.46875	0.499763
ENSG0000(	SCYL1	11:655250	25.3153	24.8811	25.5234	21.9202	20.0937	35.963	16.1718	16.531	18.5226	20.9209	0.8165	-0.08511
ENSG0000(	IGKV1D-3	2:8991398	0	7.68659	0	0.503291	1.92943	0	0	0	0	0.136094	0.09975	#NAME?
ENSG0000(	TRPC4AP	20:349754	66.5299	63.3108	30.0681	29.2916	20.7077	46.4064	47.2335	33.3428	24.3676	34.8852	0.673	-0.14397
ENSG0000(	JSRP1	19:225225	1.26655	1.72252	4.00871	4.5038	2.28813	0.102937	1.88901	0.729473	3.32895	1.29504	0.4098	-0.83584
ENSG0000(	ZNF496	1:2472974	6.57047	5.97012	10.4147	6.51211	17.0091	8.64211	10.4641	7.15604	4.78108	12.972	0.67585	-0.338
ENSG0000(	B3GAT3	11:626152	43.372	46.0213	39.3602	46.3316	36.1076	53.5025	53.6276	52.7568	38.0924	44.775	0.5966	0.178541
ENSG0000(	ZDHHC9	X:1298032	1.05022	2.83478	1.96365	2.17923	2.10571	3.26581	0.063661	1.83375	2.1505	2.47969	0.8429	-0.15587
ENSG0000(	MIGA1	1:7777962	3.52826	3.2545	2.93296	1.35974	2.30709	1.72349	1.41945	3.06312	3.60581	4.59211	0.99765	0.001461
ENSG0000(	PREB	2:2712378	75.0538	68.3724	86.6184	86.4061	64.9452	79.659	75.5594	70.4189	64.4083	67.4097	0.6937	-0.12517
ENSG0000(	UBAP1L	15:650927	1.34522	2.77995	0.786554	1.47287	0.507863	0.969882	0.926737	1.73027	0.005488	1.53505	0.7843	-0.2527
ENSG0000(	ZNF485	10:436064	2.44703	2.60958	3.17576	3.42561	2.63457	0	3.61829	2.26217	1.28774	1.84541	0.6063	-0.52769
ENSG0000(	TNFSF4	1:1731837	0.599155	0	0.366125	0.456135	0.340811	2.60262	3.36236	7.18849	2.34038	2.83017	0.0185	3.49763
ENSG0000(	FAR1	11:136686	9.15857	7.18706	1.88125	4.39465	2.74744	7.12236	7.92669	5.2787	7.08452	8.30529	0.21515	0.601063
ENSG0000(	SUN1	7:816614-	26.7661	44.2569	9.45442	17.0391	5.4351	16.8983	22.5787	15.0815	21.9048	11.2444	0.70885	-0.20674
ENSG0000(	GMPS	3:1558705	32.5264	21.4105	22.3048	11.1782	15.2912	26.2948	26.4509	19.9141	20.0078	17.4925	0.96425	-0.0189
ENSG0000(	MCAM	11:119206	0.891578	0.803653	0.331774	0.906949	0.055836	1.50944	2.0907	3.02838	4.47058	3.03352	0.0734	2.23075
ENSG0000(	CCDC34	11:273308	2.65736	2.46319	4.0242	1.97123	5.75042	9.23986	3.8823	3.43373	1.34154	1.52132	0.85785	0.159293
ENSG0000(	CDC42EP3	2:3760013	67.724	28.7677	51.8198	35.5946	78.0391	49.6488	39.5832	35.5893	28.4204	58.315	0.36245	-0.58639
ENSG0000(	ZNF585A	19:370078	30.9101	20.8346	48.0252	31.6705	15.6435	36.1136	39.4653	34.075	44.3483	9.85289	0.8832	0.13947
ENSG0000(	TRMO	9:9790448	13.1165	19.6571	28.1035	21.0363	26.2467	11.6881	16.6612	24.7144	21.3278	21.3075	0.6467	-0.17358
ENSG0000(	TRAV27	14:221479	6.94032	21.1828	3.60146	0.284103	0.667573	23.7501	17.3768	2.3132	0	0.409553	0.4673	0.388329
ENSG0000(	AFG3L2	18:123076	20.3748	19.4976	12.9486	20.9886	12.5018	23.8858	22.4301	11.6827	14.0294	15.7363	0.981	-0.0102
ENSG0000(	MELK	9:3657286	2.44428	8.22321	0.451326	0.566895	0.28989	0.383597	4.83671	3.26795	0	2.03619	0.91495	-0.10466
ENSG0000(	LMNTD2	11:537526	0.495873	5.03722	1.31671	0.24777	0.697017	2.53627	2.90199	0.172506	0	0.699499	0.73785	-0.4686
ENSG0000(	TBXA2R	19:359450	0.505332	3.91339	1.12839	0.607689	0.283363	0	0	0	0	0.394647	0.25335	-3.71066
ENSG0000(	GNB2	7:1006735	16.0161	13.5285	21.729	17.1777	49.2078	11.3569	20.3542	37.9826	20.8758	47.1724	0.66345	0.198985
ENSG0000(	RP11-229	9:1370072	4.85822	1.93324	1.08322	1.73107	1.06953	2.32191	1.5281	0.695752	2.8682	0.222662	0.8151	-0.44552
ENSG0000(	RP11-395	2:1278865	6.53637	6.37988	5.38454	7.01382	5.72562	12.4182	4.85854	3.34871	6.17324	2.72858	0.90695	-0.07888
ENSG0000(	HIST1H1E	6:2615635	46.838	41.5274	18.5038	8.35177	23.8523	45.0551	41.814	15.0562	5.02792	0.51845	0.32065	-0.56721
ENSG0000(	CCR7	17:405537	45.8418	867.833	93.8497	46.5668	23.6664	11.346	22.6798	74.4706	37.5485	61.034	0.00325	-2.18161
ENSG0000(	ASUN	12:269051	29.0606	27.5575	20.3038	15.2791	21.9802	17.408	17.6754	15.5828	13.0666	23.2964	0.7652	-0.1094
ENSG0000(	KAT8	16:311157	18.5074	29.6856	14.7935	15.6723	22.2282	9.30005	15.1467	17.951	8.09862	17.3569	0.2339	-0.55293
ENSG0000(	TRAV5	14:217491	8.28688	16.8648	9.49719	17.9844	9.5871	9.31562	22.6917	9.33246	26.3616	20.4075	0.3918	0.755644
ENSG0000(	CDC42SE2	5:1312454	125.429	116.156	173.086	116.976	95.1381	58.4585	87.156	113.723	145.779	120.916	0.38185	-0.29945
ENSG0000(	LTF	3:4643564	4.06664	0	0	0.84123	0	0	0.327681	0	0	0	0.33325	-3.65959
ENSG0000(	FCF1	14:747131	8.15581	6.41401	1.93638	0.576098	2.21135	5.49891	5.48739	2.3977	1.36054	3.08539	0.6915	-0.22614
ENSG0000(	MAPK1	22:217544	39.6268	28.0258	38.2905	35.4683	33.5118	31.3467	27.058	38.2816	20.0289	36.3964	0.53735	-0.31064
ENSG0000(	CORO1A	16:301828	680.235	645.363	1020.43	1010.24	1118.98	618.405	756.772	1221.72	1018.57	836.848	0.89025	-0.06434
ENSG0000(	SYNJ1	21:326287	3.54461	4.91525	0.998988	1.47455	1.05562	3.39626	4.03623	3.0588	1.41313	4.01341	0.1197	0.907462
ENSG0000(	AC005578	19:424633	0.222876	0	0.635885	0	0.216855	1.04823	0	0.289444	0.820839	0.170994	0.59805	0.979247
ENSG0000(	SESN3	11:951505	1.19441	6.04425	0.409623	0.115571	0.678196	0.873603	1.32819	0.847106	0.073653	2.70522	0.61305	-0.52902
ENSG0000(	PDK4	7:9558349	0.280109	7.6216	0	0	0.073081	7.86928	0.017439	0	0	0.130377	0.902	0.248544
ENSG0000(	SRGAP2	1:2061861	11.2072	9.60921	3.20579	2.53208	3.34368	17.9728	15.3105	5.51425	10.6707	5.8792	0.10595	0.970759
ENSG0000(	LINC0085	17:432118	0.307889	1.15697	1.4113	0.129678	0.039956	0.851759	1.18315	0.858948	2.0809	0.864319	0.2783	0.866149
ENSG0000(	CD47	3:1080432	105.389	98.8594	113.436	94.0516	52.924	80.9467	110.452	89.3386	41.8447	59.1676	0.36305	-0.43047
ENSG0000(	USP24	1:5506635	4.62329	7.19925	1.60085	1.16312	2.25826	3.48748	4.49007	4.46244	0.921989	4.77808	0.51955	0.284079
ENSG0000(	UNQ6494	9:8963981	0.47674	0	0	0	0.018787	15.478	1.75574	1.78092	0	0.271527	0.223	5.47832
ENSG0000(	FCER1A	1:1592897	0	12.854	0.613003	0	0.482961	0	0	0	0	0	0.01805	#NAME?

ENSG0000C	HCG11	6:2652344	1.04591	0.377339	0.621994	0.100893	0.561368	0	0.353148	0.459999	0	1.47457	0.51215	-0.56945
ENSG0000C	ERV3-1	7:6497177	5.53893	6.4398	4.39961	5.29877	4.03797	10.6635	6.93611	2.71486	2.25721	7.69809	0.6994	0.370051
ENSG0000C	RPS2	16:196205	1332.46	1392.83	3172.03	1873.84	3256.01	985.931	1089.86	2028.64	1383.43	629.58	0.09865	-0.86184
ENSG0000C	STRIP2	7:1294344	0.441444	0.401284	0	0.049099	0.045449	0.014185	2.7094	0.394956	0.571995	0.128179	0.121	1.95162
ENSG0000C	SLC33A1	3:1558210	15.7995	12.8062	8.69175	10.815	5.01481	6.95575	10.0496	11.0817	11.1974	10.7884	0.68115	-0.17001
ENSG0000C	MFGE8	15:888986	3.91624	3.42491	4.68445	2.38523	4.69674	3.83381	3.80402	4.64331	1.44875	1.47413	0.55025	-0.4073
ENSG0000C	GP9	3:1290607	0	0	2.13138	15.1196	1.05872	0	0	0	0	0	0.10155	#NAME?
ENSG0000C	GGH	8:6301507	7.50849	10.6982	0.896416	5.05923	1.14462	14.5795	7.8422	1.28882	7.45816	1.47542	0.4824	0.483324
ENSG0000C	GOLGA2	9:1282558	6.44271	6.13265	7.91895	9.85905	14.1348	7.59838	10.6492	9.31225	3.07908	17.9247	0.9744	0.024989
ENSG0000C	RP11-288	2:3683992	0.59261	0.667603	0.503372	0.708775	0.563283	0.626109	0.829784	0.927926	0.542039	2.05771	0.3884	0.612577
ENSG0000C	FCF1P2	3:4824109	2.45209	0.28152	2.05988	0.472176	2.1744	0.3722	0.654783	1.68889	2.18321	1.392	0.88355	-0.18772
ENSG0000C	MRPS31P	13:526000	6.2681	3.26154	3.4812	3.68344	2.52776	4.60565	4.51357	5.37943	4.94058	2.49872	0.8893	0.230292
ENSG0000C	POLR2G	11:627615	200.28	131.236	290.955	299.243	189.529	247.463	184.05	270.929	357.118	76.2593	0.9211	0.036737
ENSG0000C	BLK	8:1149400	71.2754	9.7487	40.2754	57.2602	20.7761	39.2224	1.84277	43.5068	78.8855	40.7914	0.81265	0.094077
ENSG0000C	PLXNB2	22:502749	0.59271	4.78319	0.014785	0	0.314558	11.2779	1.16631	0.978819	1.33805	0.883559	0.14665	1.57319
ENSG0000C	CTB-58E1	17:387024	4.79627	4.40756	6.89613	5.21442	8.13873	1.34405	10.4855	8.78685	6.85605	4.59563	0.96905	0.070505
ENSG0000C	U1	1:1485226	14.3218	4.79947	6.14844	9.37733	2.37039	0	1.13117	0	8.71617	1.4528	0.47425	-1.15464
ENSG0000C	ABCA11P	4:386173-	0.850487	1.91648	2.1447	1.16089	0.938941	0.029552	0.70016	1.07759	3.17118	1.22375	0.9374	-0.11626
ENSG0000C	AKR1B1	7:1344423	79.9253	85.0517	127.775	94.788	96.5175	90.8371	77.1241	73.2779	61.1281	36.3731	0.06665	-0.57326
ENSG0000C	INCENP	11:621239	1.12697	0.390981	2.46352	3.08407	6.71165	0.902953	0.156784	2.76533	12.2594	9.10894	0.27405	0.933695
ENSG0000C	RP11-463	14:518261	2.04133	0.589332	1.017	1.47119	0.340282	0	3.41284	0.36	2.03462	2.06444	0.5744	0.734066
ENSG0000C	CRELD2	22:499002	50.8854	38.2359	59.6785	59.8953	97.7937	52.049	83.6372	70.0715	73.8329	71.213	0.79125	0.116322
ENSG0000C	STAMBPL1	10:888797	29.2103	38.1627	18.5652	10.734	12.9054	56.0901	39.4264	18.069	13.8904	17.0223	0.4866	0.431062
ENSG0000C	ZNF175	19:515712	0.901325	4.48835	0.538468	0.231396	0.516288	0	1.64343	0.420395	0.199247	1.73786	0.42715	-0.80713
ENSG0000C	TMEM129	4:1715951	31.1436	33.5828	19.798	23.6798	19.914	22.713	34.4434	14.2511	9.80597	22.6609	0.34845	-0.34352
ENSG0000C	CTC-378H	19:420861	4.2612	1.31872	2.50558	1.72553	2.26653	5.45883	0.819892	0.13399	0.156235	0.964053	0.6332	-0.83585
ENSG0000C	PREP	6:1052775	30.5269	32.6645	13.4691	15.1665	16.7041	27.153	20.6915	17.3924	8.9774	16.9658	0.37885	-0.30189
ENSG0000C	RP13-977	12:132190	0.834722	0	0.337746	1.02783	0.330882	0	0	0.353124	2.15477	0.694596	0.64355	0.688304
ENSG0000C	PDCD4-A	10:110869	3.60367	5.34548	1.42349	2.83054	1.62318	0.665106	0.743219	1.47833	0.783171	1.48426	0.22395	-1.37756
ENSG0000C	ATRAID	2:2719958	138.781	103.011	203.744	189.616	101.718	148.756	138.657	141.836	180.107	51.0467	0.6345	-0.19027
ENSG0000C	DENND3	8:1411172	5.61013	6.83283	2.3869	10.6887	3.78717	11.0489	4.24369	8.32823	5.11467	7.68294	0.5473	0.240742
ENSG0000C	VSIG10	12:118063	0.603415	0.857485	1.09398	3.18279	0.774484	2.21167	2.7607	0.269969	0.648954	0.419601	0.71345	-0.34715
ENSG0000C	SACM1L	3:4555539	49.3533	39.733	20.2909	42.3355	14.32	27.2637	32.7826	26.3093	17.7234	25.7465	0.3394	-0.33742
ENSG0000C	TTC9	14:706417	7.0071	9.34523	1.92687	0.688117	4.22521	1.18642	0.979642	1.23758	1.37519	2.8501	0.0035	-1.60239
ENSG0000C	RBAK	7:4973987	3.71759	4.03818	0.810442	0.366044	1.15607	4.83239	1.98812	0.600771	2.32286	3.06623	0.67555	0.314656
ENSG0000C	TRPV1	17:356544	0.545039	1.4559	0.457471	0.779135	0.362007	1.49014	1.66893	0.830104	1.73076	1.47474	0.27795	1.10916
ENSG0000C	MPC2	1:1679167	59.3553	50.7136	75.112	67.4424	57.6833	101.618	85.0783	79.713	82.2779	25.866	0.6869	0.213397
ENSG0000C	PECR	2:2159428	12.4269	18.8206	14.0094	10.4247	8.87519	16.7908	11.5914	6.20146	6.70811	9.11615	0.4458	-0.41239
ENSG0000C	FOLR2	11:722166	0.498019	1.16888	0	0.04876	0	7.32281	0	0	0	0.339131	0.22695	2.25766
ENSG0000C	INTS6-AS	13:513357	7.95556	10.2743	10.1594	12.2578	4.0612	4.94141	6.85153	4.77213	4.73051	15.2454	0.817	-0.28943
ENSG0000C	DUX4L50	9:6381774	3.11347	0.798732	0.692097	1.13981	2.37966	5.6924	10.1358	7.09734	3.08208	14.0202	0.2552	2.1939
ENSG0000C	RYR1	19:384336	2.23753	1.19496	0.413947	0.046407	0.654025	3.55457	5.29884	0.607638	0.528897	1.37938	0.1596	1.23029
ENSG0000C	KANK3	19:830212	7.44119	3.91309	1.77912	2.07451	2.48153	6.31481	4.9481	3.55193	1.68608	2.83638	0.87805	0.180419
ENSG0000C	CHCHD3	7:1327848	47.5444	49.5805	40.4832	61.3349	39.8447	54.5601	52.9238	50.2671	24.1308	29.3151	0.53925	-0.20955
ENSG0000C	RP11-467	12:316471	1.35367	2.47578	1.6594	1.28503	1.39007	3.63218	0.887752	5.01023	5.63606	3.0802	0.4182	1.17644
ENSG0000C	RUSC1-AS	1:1553087	1.50471	1.74336	2.75573	2.36521	0.937962	4.91034	2.27174	0.716	1.68674	4.80833	0.63775	0.461352
ENSG0000C	DHRSX	X:2219515	14.6809	18.6896	18.2952	22.9316	21.0011	25.2751	11.4922	17.0696	7.56062	16.189	0.56985	-0.35595
ENSG0000C	SPATA5L1	15:454023	11.4687	12.6309	8.09805	10.7246	9.04093	9.16187	7.56683	11.2917	4.2458	9.93753	0.5121	-0.33121
ENSG0000C	GIMAP5	7:1507165	309.499	322.051	403.411	216.092	445.833	264.295	202.4	150.61	160.365	205.557	0.15815	-0.76544
ENSG0000C	UBE2W	8:7342000	8.04565	5.65568	8.30062	3.88032	6.0984	12.8572	17.8882	16.4968	3.61217	8.57146	0.31775	0.613058
ENSG0000C	GTF2IP12	4:1194079	0.862495	0.569381	0.376802	0.877181	0.239804	0.683586	0.613643	1.94703	0.018562	0.41709	0.83805	0.305884
ENSG0000C	AL021546	12:120438	0.733404	0	0.186532	0.210455	0.285675	0.170406	0.081405	0.326899	0.095442	0.110547	0.55615	-1.83791
ENSG0000C	ZDHHC1	16:673944	0.217377	0.899025	0.465374	0.736595	0.517279	0	0	0.3445	0	2.01405	0.84785	-0.2363
ENSG0000C	CHST10	2:1003918	2.34233	1.60832	2.95848	2.48635	2.79587	0	1.86077	3.16135	0	4.61161	0.51915	-0.48054
ENSG0000C	AKT2	19:402303	4.31471	7.82221	4.49756	1.81296	6.17254	4.15477	5.67724	5.61252	1.24787	10.7028	0.5562	-0.22318
ENSG0000C	GALNT2	1:2300579	4.00347	4.89035	3.67991	2.17241	6.51329	7.7935	9.75554	6.56563	17.89	14.1722	0.0061	1.29658
ENSG0000C	ZMYM4	1:3526896	13.6954	16.9319	6.00968	2.72965	13.4958	5.1191	15.7933	2.91176	1.38286	14.296	0.6163	-0.42453
ENSG0000C	ZNF77	19:293321	2.53049	2.46516	1.57067	2.72838	2.78254	0.107983	2.82476	8.07424	0	4.26677	0.78445	0.244004
ENSG0000C	BCL2L12	19:496595	18.2044	23.9865	24.5435	26.1591	29.5777	26.01	18.0968	24.4137	19.6904	9.91087	0.75805	-0.32642
ENSG0000C	RP11-517	6:1669991	0.81392	0.628809	0.096311	0.230568	0.085406	1.59205	0.954461	0.159267	0.453388	0.196087	0.6884	0.838828
ENSG0000C	KDELR1	19:483825	13.3107	9.48105	9.84509	10.126	21.5807	10.1888	15.6538	16.051	21.9683	23.5007	0.363	0.417146
ENSG0000C	EIF3J	15:445370	34.001	40.6305	17.1485	25.2466	25.06	23.3018	43.3753	20.7109	20.1245	19.0885	0.6938	-0.17933
ENSG0000C	ZHX3	20:410288	2.09884	3.61233	1.7588	1.51463	3.93916	1.7124	4.73896	0.06852	0.520345	1.11713	0.2228	-1.19093
ENSG0000C	HMGB1P1	8:1230025	0.900251	0.907434	0.370307	0	0.891493	0.902844	1.8339	0.548103	0.255466	1.70049	0.5234	0.836529
ENSG0000C	GPR137B	1:2361425	4.68149	8.07737	7.82725	3.07275	4.26278	41.1692	44.9782	17.3503	20.8166	10.96	0.0006	2.24646
ENSG0000C	RP11-133	15:402781	1.19785	1.81225	0.103802	0.56585	0.414304	1.57084	0.407348	0	0	0.515461	0.6833	-0.60091
ENSG0000C	IFNG-AS1	12:679894	23.2358	3.43499	3.60319	12.0829	3.85935	0	0.088513	0.787297	5.64078	1.55204	0.1918	-2.26268
ENSG0000C	S1PR1	1:1012368	60.5699	80.6339	137.115	62.5945	96.3893	12.2379	1.48631	15.0078	21.9611	38.0911	0.00005	-2.40904
ENSG0000C	VPS13A	9:7717675	8.28939	9.74124	6.38458	5.32399	6.57206	9.95888	9.98305	5.70746	7.55538	12.165	0.7186	0.206576
ENSG0000C	SNX27	1:1516120	4.62344	7.2554	4.15682	1.10613	2.92315	6.53827	3.70154	3.31782	1.80413	7.17047	0.76725	0.136181
ENSG0000C	SCFD2	4:5287298	8.49501	9.85926	9.01401	15.0058	15.5318	7.69582	9.71762	5.41387	12.4334	17.1548	0.547	-0.29529
ENSG0000C	USP30-AS	12:109023	13.5983	7.00085	28.0995	43.0817	35.3865	28.8613	22.9308	22.3426	16.4741	13.1963	0.5573	-0.44582
ENSG0000C	SLC4A10	2:1614160	96.9377	0.650336	36.6441	48.5044	36.3652	16.9401	0.238281	36.0559	29.6198	27.7744	0.0076	-1.0056
ENSG0000C	SMU1	9:3304176	10.583	12.9036	9.13133	4.24822	6.25591	11.6376	8.78144	7.60858	7.31616	8.57649	0.9331	-0.02709
ENSG0000C	PRPF4B	6:4021266	19.189	24.9829	10.4403	14.4258	17.9942	29.7201	27.2161	23.7547	20.2908	34.2456	0.27725	0.466362
ENSG0000C	IKBKGP1	X:1546399	18.4479	14.9807	10.8042	10.7547	12.6327	12.6191	18.928	8.69576	8.91179	10.9632	0.8701	-0.28088
ENSG0000C	CANX	5:1796785	120.331	81.1035	56.6296	47.8979	43.4783	88.4343	66.0423	55.6394	52.1061	48.9332	0.60365	-0.16649
ENSG0000C	DCPS	11:126283	38.1492	24.9338	54.408	57.9492	64.8181	37.7182	49.4573	35.4462	34.2454	21.0746	0.2384	-0.49887
ENSG0000C	DAP	5:1067922	32.3407	38.6824	37.2895	44.6895	37.3337	62.0164	29.4068	34.7298	31.4084	27.0194	0.7161	-0.1206
ENSG0000C	SH3BGR	21:394458	1.32132	2.35032	2.67117	0.684353	1.59504	2.43918	1.38967	1.54829	0	1.48668	0.8396	-0.18117
ENSG0000C	TRAPPC11	4:1836592	13.7215	14.2281	3.67259	6.4878	3.08033	7.10682	4.12003	5.90262	7.31856	8.33745	0.99355	0.003896
ENSG0000C	CTD-2207	5:5267519	0	0	0	0	0	0	3.64905	0	0	1.55851	0.14175	inf
ENSG0000C	GM2A	5:1511810	1.23534	2.5657	1.08595	1.96969	1.73244	14.4489	4.17929	1.33758	2.62462	3.16085	0.38845	1.37228
ENSG0000C	PPP2R5E	14:633713	11.7534	11.7409	4.22816	5.25469	8.93286	7.73799	10.4129	6.3534	7.03361	16.7873	0.91275	0.047821
ENSG0000C	TLR10	4:3877223	0.321612	4.28675	0.223702	0.851034	0.355968	0	0	0	0	0.066772	0.2306	-6.8222
ENSG0000C	LL21NC02	21:449328	0.093924	0.895456	0.392403	0.088793	0.426364	0.245854	0	0	0.772122	0.49116	0.8763	-0.27874
ENSG0000C	ZNF614	19:520127	4.61388	4.44199	2.63115	1.78113	1.27142	3.31864	3.75262	1.38825	3.75601	2.90469	0.97875	0.014132
ENSG0000C	HARS2	5:1406646	24.8321	28.848	22.3657	24.1661	18.0716	16.7562	20.0477	22.6634	17.6777	26.3817	0.824	-0.14741
ENSG0000C	GPS2	17:730699	161.689	203.321	146.344	119.728	153.027	217.822	207.859	112.029	116.065	85.7401	0.864	-0.09429
ENSG0000C	RP11-680	12:532952	0.089857	0.2364	0.365193	0.276889	0.07241	2.05032	1.15401	1.61748	1.95657	1.49861	0.02755	2.99152
ENSG0000C	CTD-2017	14:101761	0.60637	1.63448	0.439258	0.650577	0	1.00894	0.874193	0.126237	0	0.993213	0.97765	-0.04549

ENSG0000	ANKRD12	18:910262	125.928	172.386	108.857	60.0149	111.025	309.098	216.875	135.883	118.406	273.523	0.2654	0.700403
ENSG0000	CTD-2201	5:7999131	0.098776	0.683912	0	0	0	3.16181	0.067101	0	0	0.179665	0.30545	2.15571
ENSG0000	RP11-248.	14:507197	1.41212	0.247677	1.68441	1.16969	0.823756	1.1441	2.74493	0.593864	0	1.81598	0.8472	0.285309
ENSG0000	TM9SF3	10:965181	42.8968	37.7966	10.7975	7.97253	10.9681	32.3902	24.5949	19.8387	11.6262	24.5796	0.9859	-0.00639
ENSG0000	EEF1B2P6	7:1316619	2.51072	1.12711	0.895291	0.318663	0.197866	1.02739	1.43207	0.593444	0.515064	0.041296	0.73845	-0.58265
ENSG0000	MAPK13	6:3612780	19.3882	19.2309	17.2117	16.7447	25.1996	13.7402	13.6834	15.8603	18.8338	11.0458	0.26005	-0.47304
ENSG0000	EI24	11:125569	54.1521	52.0915	54.42	47.4621	55.5279	28.7373	42.6484	45.6601	46.9675	67.7786	0.57905	-0.28427
ENSG0000	ZFP37	9:1129971	0.631894	0.033603	0.59711	0.805848	0.325775	1.29067	0.901541	0.048318	0	1.28397	0.91155	0.16112
ENSG0000	AC006369	2:3760013	6.3635	1.70143	4.20384	7.78569	2.49954	4.26441	1.39079	2.37678	3.94554	1.5551	0.5965	-0.67598
ENSG0000	RPLP0P6	2:3840671	7.40834	3.86761	3.96526	4.78248	3.64542	6.40873	6.3979	2.10335	3.06958	0.940501	0.65435	-0.35769
ENSG0000	CDT1	16:888032	3.31399	7.57994	0.317067	0.813218	1.16459	6.49306	10.267	5.20383	6.59481	4.59464	0.0239	1.53602
ENSG0000	RPRD1A	18:359843	9.71304	21.0739	8.84962	8.42667	4.16457	3.70774	12.9617	5.05467	4.59455	8.78641	0.3055	-0.67501
ENSG0000	RP11-705	12:965856	3.58575	4.75955	1.81664	1.25165	2.05677	2.22599	3.67977	3.76473	3.48955	3.08487	0.60595	0.308647
ENSG0000	PIGC	1:1718414	48.1646	51.2586	64.4746	44.961	33.8161	32.8938	34.4802	31.6269	49.9369	23.3716	0.181	-0.48786
ENSG0000	TEX264	3:5154114	62.9266	67.8634	89.1557	80.399	99.7336	55.9514	78.3972	65.646	84.5362	48.7276	0.49595	-0.27203
ENSG0000	AC002398	19:357180	0	0	0.253706	0.008181	0.703006	0	0	0	0	0	0.2211	-2.0709
ENSG0000	RAP1GDS1	4:9825168	29.4654	26.6112	13.8279	20.3918	14.7395	29.0071	18.0038	14.2052	19.6467	23.8988	0.78835	-0.09115
ENSG0000	MARK3	14:103385	72.7741	68.7879	56.6083	54.1886	32.0468	69.7239	43.7135	48.2884	59.2079	70.2035	0.84505	0.063022
ENSG0000	COPG2	7:1304860	1.62095	0.328538	0.501634	0.703292	0.423456	1.09637	2.05573	0.750519	0	0.711866	0.73755	0.522676
ENSG0000	MTHFD1L	6:1508655	3.42088	6.65187	4.13026	2.15297	1.78497	8.5528	10.4284	6.44147	2.36342	7.3651	0.145	0.841317
ENSG0000	PRSS23	11:867910	1.09863	8.87325	5.91442	4.20919	2.88388	0	0	0	0	0	0.11045	#NAME?
ENSG0000	LRTOMT	11:719985	1.10502	3.62632	1.75777	1.51945	1.80443	4.77486	1.81173	4.08588	4.09936	2.87915	0.11895	1.04188
ENSG0000	PSME1	14:241361	751.665	584.237	843.959	732.676	666.183	815.593	765.621	828.047	620.191	245.341	0.7357	-0.12262
ENSG0000	LINC0029	2:7922424	1.46151	0.374065	2.29724	1.84192	1.69215	2.25966	0.934507	1.95486	3.57948	2.24941	0.66675	0.505008
ENSG0000	NOSIP	19:495557	207.592	349.36	372.653	338.432	388.735	163.836	149.077	205.072	176.867	128.151	0.00215	-1.0196
ENSG0000	MBD2	18:541516	39.0432	27.8831	4.79515	3.03567	12.2666	53.9127	44.93	9.06829	5.58548	13.5997	0.2093	0.60753
ENSG0000	CUL7	6:4303761	2.68326	1.99699	1.08308	0.694071	0.827781	3.32696	2.43271	0.666003	2.80383	2.60645	0.5673	0.706854
ENSG0000	RP11-16F	11:966043	0.392436	0.184127	0.60854	0.615698	0.095221	0.21921	0.372895	0.292839	0.130096	0	0.6526	-0.78788
ENSG0000	NADK2	5:3619259	5.80904	5.63423	3.58639	2.63376	2.47898	10.9848	2.18228	1.65464	2.85805	2.97597	0.88835	-0.0704
ENSG0000	PDHA1	X:1934389	42.6284	34.8879	50.3981	51.2008	47.6647	40.4767	56.3899	55.3983	31.0059	42.3124	0.49725	-0.26781
ENSG0000	KRR1	12:754806	39.6405	21.0858	34.8967	31.9382	43.0974	38.5451	47.5808	39.5938	21.9951	29.1023	0.94715	-0.0434
ENSG0000	TIPRL	1:1681789	20.3668	11.0381	15.8175	14.5831	12.4463	18.8786	20.7748	16.7269	11.8581	9.67613	0.92165	0.051518
ENSG0000	SAFB	19:558699	113.925	151.738	68.5469	51.9316	61.6971	92.0605	78.226	54.5545	46.3394	75.1819	0.44345	-0.33074
ENSG0000	GCLM	1:9388520	9.13864	4.09472	12.3329	5.56103	10.724	9.99467	7.98665	9.74411	9.49086	11.1046	0.9929	-0.0038
ENSG0000	MTHFS	15:798335	58.4821	66.674	83.9412	58.1393	62.5085	76.9311	86.8956	77.4789	12.389	23.3374	0.4137	-0.38266
ENSG0000	COCH	14:308745	0.173701	1.1185	0	0.908321	0.368825	0	0	0	0	0.644943	0.23795	-1.23022
ENSG0000	PRCP	11:828235	24.2602	19.5195	16.4978	25.063	23.7324	33.2243	10.4649	15.3252	23.1371	19.9125	0.5878	-0.2287
ENSG0000	MED31	17:657814	25.9903	18.1552	19.9246	17.5961	6.79417	30.5851	37.8845	32.9549	16.6767	11.9165	0.3764	0.636329
ENSG0000	CARS	11:300092	40.3605	51.392	29.0607	30.6839	31.048	41.2904	58.8619	48.6599	25.5433	33.7616	0.67175	0.150132
ENSG0000	BX322557	21:452880	1.43742	1.36879	0.457746	0.227864	2.4514	3.38369	0.644505	1.42698	0	2.79939	0.6568	0.736296
ENSG0000	BRMS1L	14:358192	1.53895	2.76225	1.69535	1.80998	1.68711	2.99766	1.22819	1.59317	1.49416	1.35663	0.67915	-0.30162
ENSG0000	LL0XNC01	X:4926984	0.459503	2.93567	1.18177	0.417004	0.410567	0.908673	5.39075	2.12827	4.20092	8.03131	0.11455	1.91987
ENSG0000	RP11-192	17:284557	1.04322	0.901987	0.502919	0.348355	0.876518	0	2.69909	0.278799	0	1.48375	0.7914	0.39374
ENSG0000	RP11-175	1:2023486	1.27887	0.970975	0.184513	1.07947	0	0	0	1.77774	0	0.110108	0.71795	-0.67765
ENSG0000	EOMES	3:2771594	156.694	18.9203	143.008	153.151	170.677	38.1762	13.2392	63.774	41.3873	102.933	0.0003	-1.37966
ENSG0000	ZNF234	19:441415	4.62089	6.56327	3.54715	4.27159	3.5587	0.135974	4.38793	2.41598	0.738948	9.60857	0.22125	-0.63168
ENSG0000	RP11-401	7:1030997	0.510318	0.708761	1.03344	0	0	0	0	0	0	0.821286	0.65095	-1.05505
ENSG0000	RPL12P11	20:414021	0.345653	0.15036	0	0	0	2.71058	0.17697	0	0	0.104284	0.40185	2.66447
ENSG0000	MAPRE3	2:2684774	1.34209	0.481568	0.163438	1.04544	0.919265	0	0.089004	0.433564	2.3106	0.931866	0.8741	0.153055
ENSG0000	PYGM	11:647463	0.557344	1.63186	0.680822	0.558282	0.441763	0	0	1.20929	0.774372	0.662442	0.7114	-0.47185
ENSG0000	ANKS1B	12:986451	0.431865	1.50654	0.382142	0	0.106692	1.83874	3.6797	1.63223	3.61942	0.750583	0.0041	2.44526
ENSG0000	TRAF5	1:2113266	32.939	26.1012	12.3163	16.2697	12.9114	23.2492	21.568	18.0969	18.917	31.9416	0.61755	0.170189
ENSG0000	RP11-645	16:217940	2.28451	5.3435	0.204681	0.214032	0.69835	0	2.45442	0.487439	0	2.40418	0.8008	-0.52756
ENSG0000	FANCA	16:897071	24.0483	16.6558	17.8902	27.2141	19.2682	29.2611	27.1171	22.4314	15.8531	23.4413	0.4499	0.347825
ENSG0000	BRF2	8:3778419	6.62888	6.65863	5.00926	4.03178	4.8154	4.8574	2.26223	3.48226	1.18697	3.84278	0.12385	-0.85014
ENSG0000	RP11-296	1:1734177	2.3357	2.21779	1.21147	2.72933	1.11467	3.31986	3.96043	3.91738	3.26887	2.63469	0.18115	0.917879
ENSG0000	SPAG9	17:509621	4.82618	5.00808	3.43715	1.85633	3.11722	7.41757	4.28889	3.72604	2.41981	11.4503	0.28995	0.42397
ENSG0000	RP11-412	12:106954	5.22443	2.40231	5.59182	5.1142	3.83306	3.24204	0.659095	1.76015	4.98528	1.63658	0.32175	-0.83467
ENSG0000	HLA-DQA	6:3262817	0.666992	24.354	5.77815	8.09876	1.67223	51.6365	18.9724	55.499	13.1675	1.99244	0.01075	1.89316
ENSG0000	RP11-54C	5:1517423	1.5606	0.507475	2.62451	2.93685	1.07301	0	2.12247	1.56523	1.90719	0.105847	0.60065	-0.71323
ENSG0000	RP11-783	11:642066	1.71837	1.03822	0.251907	0.172339	0.404305	0.045756	1.55189	1.34611	0.326794	0.484861	0.87655	0.228684
ENSG0000	ZNF223	19:440253	3.10453	2.34976	0.83771	1.13066	0.881772	1.44086	0.312476	0.091405	0.143825	0.901518	0.2632	-1.2514
ENSG0000	CDC42SE1	1:1510477	65.6752	106.473	111.228	97.4097	59.9929	56.8995	29.3347	86.8232	46.7916	66.0131	0.1346	-0.79376
ENSG0000	TM9SF2	13:995014	61.3824	63.261	27.0399	31.982	20.7442	58.0555	55.9404	38.7337	45.1127	37.3958	0.35235	0.284209
ENSG0000	CRAMP1	16:161232	2.12859	2.82809	1.18784	0.676524	2.20245	1.21676	2.20785	1.2608	0.624646	4.71642	0.9198	0.089829
ENSG0000	FSD1L	9:1054477	0.251396	0.590018	0.039545	0	0.109698	0.491697	1.53414	0.206507	0	0.640707	0.1185	1.61535
ENSG0000	SMARCD3	7:1512324	6.30745	10.6361	6.45708	8.59296	6.67696	10.5403	5.67925	12.4173	19.3913	15.1541	0.38745	0.761998
ENSG0000	INPP4A	2:9844485	50.8819	56.2943	14.1256	13.8614	14.2877	38.9294	29.9961	16.152	10.5073	31.1877	0.4707	-0.35618
ENSG0000	ERC1	12:912076	6.11717	16.7144	2.55816	3.79752	5.54615	42.8855	18.1231	1.09319	17.874	8.68503	0.16555	1.02281
ENSG0000	RPN2	20:371012	184.587	165.482	218.56	206.985	202.333	213.662	168.567	140.894	164.793	202.508	0.51705	-0.20607
ENSG0000	EGR2	10:628119	0.10832	1.20363	0.5529	1.08576	0.093909	13.7962	7.86923	45.4145	5.72337	12.3893	0.02025	4.96434
ENSG0000	RP3-426I6	1:2777318	0	0.724572	0	0	0.134541	0	1.98347	0.551247	0	0.581573	0.35695	2.06422
ENSG0000	ISOC1	5:1290947	31.6143	24.6101	35.2445	31.6672	20.5061	40.6298	36.735	26.6604	35.2477	13.9563	0.786	0.088822
ENSG0000	AC024560	3:1975782	11.2744	13.6364	10.1103	9.4264	6.29061	14.3374	14.2127	19.648	5.30017	16.8171	0.35775	0.470691
ENSG0000	WDR76	15:438269	4.21853	5.76776	2.31297	2.3732	3.44309	1.42968	4.60146	6.31648	0.276824	4.79475	0.81135	-0.16329
ENSG0000	CEBPB	20:501845	31.9656	46.6811	14.8432	16.5522	15.3091	105.874	125.915	30.1384	24.0827	10.9983	0.00675	1.16518
ENSG0000	TM7SF3	12:269715	43.2314	28.252	22.5756	22.3207	26.1714	34.6888	20.8331	23.3726	29.0318	36.7721	0.7046	0.110237
ENSG0000	GCNT4	5:7502534	0.201927	0.90048	0.793694	0.377023	0.597526	0.037799	0.03112	0.120481	0.816248	0.787301	0.1692	-1.07911
ENSG0000	CTD-2341	14:859347	6.30826	1.03781	4.65358	5.00966	2.07109	2.62591	1.20359	4.55762	3.26761	3.1648	0.8643	-0.28484
ENSG0000	SLC17A9	20:629526	1.74796	2.53687	4.26962	8.34914	1.61739	2.74585	2.829	5.09136	0	2.23669	0.5079	-0.59164
ENSG0000	AUTS2	7:6959891	5.86124	5.1282	4.14587	4.35398	9.13579	14.6347	16.9159	9.75685	5.41126	53.8261	0.00065	1.67861
ENSG0000	ZNF576	19:435963	11.3525	5.08079	15.9201	7.17587	19.1744	14.1055	11.9579	17.5011	8.29804	12.2289	0.9161	-0.0802
ENSG0000	NAA16	13:413112	16.0645	33.9094	9.51626	15.4079	12.5523	9.15231	9.84541	6.48375	8.07704	15.869	0.073	-0.88715
ENSG0000	ZFAND4	10:456155	20.854	15.0584	7.38393	9.23563	5.54684	6.41104	6.39731	7.25178	10.2973	7.3813	0.41005	-0.45586
ENSG0000	PLEKHA8P	12:451730	11.6315	12.9106	5.43865	10.9814	5.93115	16.1059	7.70848	2.22634	0.418422	5.45081	0.6078	-0.50212
ENSG0000	RP3-467N	6:1008814	2.70296	1.71223	3.50341	5.7154	0.847103	0	1.53196	3.54767	4.10575	1.39146	0.6407	-0.37802
ENSG0000	USB1	16:579946	41.7037	40.869	43.0105	52.9387	73.5141	48.1464	38.0961	53.3498	52.7146	91.4236	0.70585	0.131021
ENSG0000	NPIPA1	16:149115	12.6719	14.5936	2.24029	3.38982	3.09585	12.9837	8.43134	3.29555	3.13027	7.38577	0.735	0.218466
ENSG0000	MOB1B	4:7090232	1.21194	2.09136	0.405534	0.71882	0.427745	1.29766	2.13103	1.60625	0.634846	1.16301	0.7168	0.367637
ENSG0000	VPS53	17:503170	6.76378	7.73047	2.72211	1.74989	2.54206	11.9308	9.71751	2.35855	2.59739	4.49788	0.56235	0.302629
ENSG0000	PSMC6	14:527071	79.5142	57.6801	58.059	53.8438	59.3848	66.1119	91.1174	62.9578	44.5933	46.3416	0.91925	0.0327

ENSG0000	TRBV10-3	7:1425442	15.8011	27.4038	10.6617	19.4784	8.21555	10.4247	14.9178	245.502	12.3974	3.2943	0.0149	2.08538
ENSG0000	RNF40	16:306977	29.5133	22.6951	17.282	18.7562	19.3588	18.9988	21.653	10.06	13.937	20.7167	0.4104	-0.46547
ENSG0000	PGBD2	1:2489061	12.8381	14.6191	10.2686	10.4121	6.43364	9.61615	4.77095	7.93116	6.39948	6.25968	0.1717	-0.67018
ENSG0000	TMSB10	2:8482164	2723.55	3190.27	2341.37	1974.32	1158.05	4149.76	3183.55	2298.9	1884.3	327.435	0.94975	-0.02548
ENSG0000	COX14	12:501119	77.0158	55.9286	141.428	151.455	53.5426	128.219	113.781	115.629	100.159	15.5818	0.85615	-0.10007
ENSG0000	TGIF2P1	1:2443526	0.412812	0.306763	0.200971	0	0	0	2.45262	0	0	0.693134	0.4604	1.68574
ENSG0000	DAXX	6:3331855	88.9526	102.9	125.101	110.106	120.878	58.7936	92.4817	115.032	41.0512	114.809	0.147	-0.45512
ENSG0000	TCEAL8	X:1032529	60.9024	66.8745	56.9812	42.8201	41.704	46.5387	32.4936	20.6813	49.9769	7.73319	0.05145	-0.7665
ENSG0000	LRRC20	10:702989	1.08974	1.01209	0.975808	0.236346	0.669942	0	0	1.26803	0.127643	0.691155	0.4042	-0.94617
ENSG0000	MON2	12:624668	12.1121	10.9568	3.23643	4.28316	2.9821	5.9682	12.881	5.82779	9.12558	9.89585	0.2895	0.513396
ENSG0000	RP11-363	9:1945320	0.174271	0.922175	1.73658	0.18003	0.59936	0.987525	1.2725	1.88269	0.780714	1.6889	0.49305	0.773105
ENSG0000	RP4-533D	1:4604013	0	0	0	1.20614	0	2.48749	0.809925	1.67481	0	0	0.47715	2.13335
ENSG0000	CTD-2600	16:481649	0.814109	0.903109	0.250771	0.058995	0.413548	1.0493	0.354916	1.31543	0	2.17263	0.53585	0.9906
ENSG0000	TIMM23B	10:498182	11.399	13.0785	7.67344	11.0246	6.00836	9.24468	5.03531	11.0573	9.69634	9.97534	0.87875	-0.11023
ENSG0000	PCDHGB5	5:1413305	0.017452	0	0.030943	0	0	0	0	0	1.32962	0	0.1513	5.50163
ENSG0000	ZDHHC24	11:664350	14.1562	7.6337	16.1566	16.9577	19.3807	19.5704	13.5468	12.8142	16.6454	12.5795	0.9456	-0.04646
ENSG0000	GFOD2	16:676745	8.08288	4.6713	7.22089	11.6603	10.6535	12.9422	5.81095	7.90192	8.82083	11.2163	0.8896	0.070883
ENSG0000	RP11-367.	4:6909241	0	2.32715	1.08242	0.60556	1.04251	0.071693	0	0.548838	0.041414	2.70969	0.91275	-0.25906
ENSG0000	IL1R1	2:1020645	0.608597	0.690982	0	0	0.237231	7.33977	5.65488	1.53414	0.337777	0.846575	0.01485	3.65899
ENSG0000	FLI1	11:128629	56.2985	56.1954	42.999	33.9705	41.4709	30.9891	36.4801	29.4647	24.9122	41.8191	0.02145	-0.81382
ENSG0000	SSH1	12:108778	1.92578	3.50772	1.68473	1.77062	3.39778	2.92774	5.11951	2.58554	1.34269	5.71412	0.8542	0.12924
ENSG0000	EIF4ENIF1	22:313995	8.95191	12.4012	6.05379	2.87406	4.67228	9.93429	5.05511	6.04865	6.99702	11.1599	0.86035	0.153844
ENSG0000	RP11-435	9:9544297	0.248307	0	0	0.383081	0.944458	0	0	0	0	0	0.1331	#NAME?
ENSG0000	SP100	2:2304159	179.226	185.572	126.407	135.433	153.567	169.801	277.543	160.662	101.264	208.843	0.64855	0.147942
ENSG0000	MACROD1	11:639746	10.0374	7.74584	6.90431	6.0565	9.16247	3.9827	5.48349	4.31884	1.8868	2.55378	0.14885	-1.17927
ENSG0000	CEP170	1:2431244	3.73526	10.4157	1.31144	1.88334	1.79729	13.7367	4.70374	3.20847	1.01418	3.95392	0.38635	0.537059
ENSG0000	PDCD4	10:110869	278.842	206.562	270.471	204.212	273.519	217.949	225.755	290.63	331.783	422.556	0.7838	0.117497
ENSG0000	ZNF813	19:534677	1.62861	1.58511	0.358675	0.404668	0.237748	0.517347	0.21828	0.099931	0.323875	0.567747	0.23765	-1.5974
ENSG0000	FCHSD2	11:728143	12.8103	16.7688	4.27705	2.42074	4.47954	12.3614	9.19259	4.78839	1.43378	5.8618	0.7294	-0.28729
ENSG0000	ZNF585B	19:370078	4.76551	5.04553	4.36609	4.39215	1.83454	1.22864	2.53932	2.18185	6.38462	3.12943	0.77675	-0.20156
ENSG0000	RABL3	3:1206866	14.6696	12.7572	8.12517	7.64577	7.58428	15.8422	10.162	8.79773	2.39975	8.78633	0.79745	-0.13275
ENSG0000	TIMELESS	12:564163	7.3605	6.77299	1.48388	3.92763	2.29542	9.88873	6.19948	3.23394	2.43332	5.92561	0.6162	0.268036
ENSG0000	ESCO1	18:215292	17.646	14.0943	7.5231	7.79903	8.96979	23.1262	21.0666	9.41269	4.97585	18.7657	0.1418	0.526739
ENSG0000	RP11-314	20:130990	0.34946	1.12841	0.08545	0.680084	0.089813	0.80857	0.858127	0	1.29157	0.440285	0.7094	0.713755
ENSG0000	LRRC24	8:1445179	2.28829	3.90588	0.268084	0.897218	0.388507	4.7872	1.88195	0.192076	0.513674	1.40442	0.92345	0.152572
ENSG0000	EXD2	14:691830	3.72156	4.29909	1.41636	3.23531	2.64946	1.61147	1.0904	1.91161	7.83001	3.78186	0.7207	0.241703
ENSG0000	LINC0064	21:335039	23.2838	18.1282	24.6123	18.673	23.1165	22.5671	26.2251	21.5875	21.7172	26.3189	0.9365	0.066669
ENSG0000	TM7SF2	11:650842	9.34455	5.88585	15.9277	13.7913	9.42436	10.693	7.81994	7.05383	7.93146	10.1417	0.5841	-0.40595
ENSG0000	LENG9	19:544448	0.715183	0	0.101958	0.409655	0.839917	1.74096	0	0.544847	0	0.579172	0.8409	0.428689
ENSG0000	VPS36	13:524126	31.8025	19.8792	18.4626	13.3343	17.3373	28.5512	23.5963	13.9068	16.2173	24.1873	0.9886	-0.00576
ENSG0000	OSBP2	22:306937	0.844688	0.608635	0.434998	0.793781	0.369014	2.18501	1.88206	0	1.0506	0.030106	0.7754	0.193139
ENSG0000	RP11-600	14:103525	0.345679	0.757027	0.099719	0	0.039928	0	0.498271	0	2.23683	0.898866	0.1963	1.87207
ENSG0000	MAGEH1	X:5545210	27.0307	30.3304	34.8385	34.8093	21.7367	31.226	58.8289	22.7786	9.09984	14.1983	0.6309	-0.1895
ENSG0000	KCP	7:1288303	0.086044	0	0.170165	0	0.22748	0	0.409673	0.028676	4.20964	0.114431	0.07655	3.39225
ENSG0000	URB1	21:322918	1.46062	1.89472	0.869182	0.663004	1.10116	0.490381	1.19466	1.80184	0.117416	1.57261	0.51525	-0.33225
ENSG0000	NOL4L	20:324430	17.4263	35.4255	8.42486	6.35945	9.58106	12.9272	13.6696	8.74147	2.27574	12.3109	0.1631	-0.63542
ENSG0000	RABL2B	22:507569	16.8846	22.0171	23.0116	25.96	13.8859	13.6832	13.4524	19.6434	17.465	22.1223	0.62805	-0.23416
ENSG0000	GAR1	4:1098155	15.4342	19.3566	14.1637	12.4718	14.392	5.34166	19.9385	14.8558	0.957522	11.3671	0.31435	-0.58297
ENSG0000	LRRC14	8:1445179	21.4856	32.4482	9.38498	16.3175	9.7504	19.372	17.8778	5.45638	10.906	15.3471	0.2713	-0.46345
ENSG0000	CHIC2	4:5337764	70.7738	62.2865	67.667	68.1382	50.9556	66.7175	86.035	96.6618	73.9795	37.3293	0.75425	0.162884
ENSG0000	SELENOH	11:577125	122.944	89.713	118.651	120.119	74.7119	192.426	125.057	109.571	114.232	48.0138	0.82315	0.125492
ENSG0000	XKR8	1:2793499	22.1187	12.0424	23.6196	14.9762	22.9126	10.0342	7.75853	13.9518	12.1154	19.7029	0.16815	-0.58433
ENSG0000	RPS6KB1	17:598930	18.1975	14.5486	15.2014	13.4277	14.5391	26.1941	9.38949	17.2218	7.25864	14.1663	0.66695	-0.22015
ENSG0000	CLEC7A	12:101167	1.57357	10.3298	1.0187	1.28785	0.723549	33.7013	4.02041	1.94435	15.7847	2.77116	0.06455	2.24825
ENSG0000	1-Sep	16:303781	224.194	252.67	364.782	264.244	408.424	265.752	261.397	368.234	317.007	413.146	0.8092	0.084294
ENSG0000	ST6GALN	17:765653	0.518117	0.308863	0.881905	0.616856	0.496048	0	2.19448	2.12693	2.67266	0.978077	0.1252	1.48365
ENSG0000	AK3	9:4709558	22.2637	20.8042	21.4588	30.2743	25.6744	23.2047	37.4864	26.2757	26.3645	38.4325	0.64275	0.158883
ENSG0000	FNDC10	1:1598011	1.48753	5.7466	2.46284	0	3.74981	2.32164	1.25295	0.838229	0.695908	2.0628	0.23595	-0.86858
ENSG0000	FAM114A	5:1539901	16.1664	9.85024	13.2417	9.63698	9.21314	12.2136	9.46336	15.7117	11.8404	17.2434	0.8594	0.115437
ENSG0000	RP11-267	8:9437195	0.466014	0.558762	0.541176	0.482809	0.826548	0	0	0.395806	0	1.2628	0.6439	-1.16538
ENSG0000	ADCY3	2:2478973	7.66901	6.01487	1.64271	0.48336	2.74811	17.3221	18.3433	9.15462	7.24445	13.8623	0.0106	2.14454
ENSG0000	RP11-108	1:1673993	9.68845	0	2.40924	3.6424	3.33774	6.00658	0	1.03932	2.96797	0.638666	0.6206	-0.82544
ENSG0000	LA16c-35	16:130554	0.937853	1.41126	0.705013	1.49506	0.715042	0.294789	1.61994	2.04877	1.16447	1.37199	0.74905	0.332705
ENSG0000	SNHG7	9:1367125	4.96892	6.25817	8.27314	8.80956	8.82879	8.81292	9.2064	12.7466	11.5495	8.58932	0.59285	0.426198
ENSG0000	ANKRD36	2:9749266	42.0985	49.9904	19.3211	35.0334	18.8031	50.6391	55.3514	14.7667	21.0475	89.5417	0.4385	0.307163
ENSG0000	SLC2A1	1:4292537	25.0656	20.2536	45.8416	48.2037	40.0098	23.0249	26.5233	33.2778	5.0848	49.5351	0.07375	-0.68112
ENSG0000	USP18	22:181498	12.0948	9.84284	20.4468	19.6505	13.4878	11.0228	110.66	48.1452	19.1419	10.5693	0.00135	1.32993
ENSG0000	AC005618	5:1413262	0.126646	0.204529	0	0	0.17781	0	0	2.37358	0	0.096165	0.26775	2.68443
ENSG0000	HMGB3P2	5:1796785	0.224269	0.845522	0.591287	1.39071	0.683632	0	1.16709	0	0	0.12476	0.3356	-1.80978
ENSG0000	HERC1	15:636086	18.6393	34.3527	6.44369	6.50039	14.3647	16.9762	28.4527	9.34878	28.701	30.3603	0.1544	0.884018
ENSG0000	HLA-DOB	6:3281276	0.308403	2.99262	2.62774	3.05519	1.72142	1.40891	0	0.459184	0	0.409904	0.2114	-2.28075
ENSG0000	PPFIA1	11:702706	11.5665	16.4613	5.22451	6.9666	7.95689	21.6431	10.2762	7.76029	4.48283	15.5361	0.73585	0.136874
ENSG0000	FAM32A	19:161853	131.251	125.441	142.511	153.002	100.823	125.987	126.336	92.2889	101.411	55.193	0.1973	-0.41841
ENSG0000	IPO13	1:4394693	8.76271	5.85947	2.99562	5.76824	5.85718	7.17846	5.37552	4.14392	10.5875	7.20557	0.467	0.320478
ENSG0000	IARS2	1:2200941	37.2335	36.5684	13.0863	25.2853	10.3602	17.9413	20.312	12.746	21.5757	15.7032	0.25805	-0.36519
ENSG0000	TRAV26-1	14:221233	13.8508	23.4423	12.9386	3.92464	0.57345	14.7395	12.3865	5.46012	0	7.43137	0.64545	-0.36409
ENSG0000	NPLOC4	17:815568	35.3699	37.1995	9.40588	6.61835	10.693	27.463	28.1684	13.1213	14.576	19.8438	0.94555	-0.0237
ENSG0000	HIST1H2B	6:2615814	0.605608	0.241715	0.93039	1.19229	0.145395	0.321105	0	0.251743	0.000614	0	0.097	-3.84643
ENSG0000	ASMTL	X:1401768	25.9602	14.8025	34.8226	35.0121	31.7013	30.6324	28.6996	26.3975	46.7891	21.8645	0.79015	0.101085
ENSG0000	PM20D1	1:2058280	2.43668	0.892259	1.18078	2.22207	0.955607	3.57505	2.18834	0.082507	3.16059	2.0659	0.5065	0.535509
ENSG0000	USP7	16:889209	28.6765	31.3184	7.8711	5.40602	14.0649	18.368	20.7472	13.9122	10.4285	23.1271	0.76685	-0.11746
ENSG0000	PPM1K	4:8825761	12.7848	12.2299	19.8671	30.9139	23.6412	11.93	36.412	22.5205	27.67	37.4028	0.61505	0.227091
ENSG0000	MRPS10	6:4220680	36.9342	39.0952	33.2045	43.0086	33.3471	37.4875	21.5484	35.7651	14.4705	22.1903	0.1143	-0.53424
ENSG0000	RBBP9	20:184865	1.95444	0.791943	1.33423	1.28045	1.50313	2.28313	2.85776	0.926725	0.655077	0.608144	0.96265	-0.02541
ENSG0000	PTGES3L-	17:429505	0.449464	0.175842	0.108741	0.11053	3.02523	0	0.201375	2.91748	0.198016	1.22572	0.9304	0.135747
ENSG0000	TMEM198	12:557432	9.41372	22.5498	8.55581	7.52472	4.20976	0.046051	2.88428	7.3007	13.2493	5.85193	0.44075	-0.6067
ENSG0000	SNRNP35	12:123457	43.2108	51.8708	54.2424	36.8858	84.4019	64.6252	69.413	45.0883	25.8109	44.0457	0.6219	-0.19344
ENSG0000	NIP7	16:691055	14.7345	14.6528	21.2235	26.8148	19.77	23.7895	22.1645	23.3266	36.3881	19.4499	0.80255	0.271623
ENSG0000	RP11-582	12:270225	0.80765	3.75443	1.67159	1.53126	1.232	2.72474	2.02386	5.34795	9.18145	5.31617	0.3204	1.42914
ENSG0000	WDR82	3:5225442	24.5442	27.2515	15.152	17.819	20.644	13.0657	12.7439	18.0403	12.0537	32.2513	0.31745	-0.37623
ENSG0000	SUPT6H	17:286483	11.9709	12.484	3.23415	0.810895	6.33405	8.48838	8.00242	6.37435	1.35543	11.1254	0.94755	0.047527

ENSG0000	LSR	19:352483	4.77356	17.0713	3.40925	19.1614	6.73315	5.32881	11.5374	8.8493	17.75	11.0696	0.76295	0.14892
ENSG0000	CDC37L1-	9:4676599	3.40823	1.22634	2.49884	3.61629	0.621568	0.874014	2.09988	0.269552	0	0	0.3032	-1.82763
ENSG0000	ATE1	10:121740	12.5844	12.512	7.91963	6.37065	6.18194	7.74841	5.87798	2.4216	5.53285	7.86074	0.20365	-0.74938
ENSG0000	TRIM56	7:1010854	8.61655	17.2929	4.54121	1.54462	7.99018	11.4891	18.7247	8.51276	6.79202	17.5168	0.71515	0.167611
ENSG0000	UBE2J2	1:1253908	50.0171	56.5046	78.5011	61.5407	71.4315	52.9365	57.7219	69.4989	30.8824	52.5671	0.21505	-0.39627
ENSG0000	RP11-353	2:9681223	0.642285	0	0.783614	2.05141	0.360336	0	1.16886	0	0	0.760597	0.5404	-0.97013
ENSG0000	PTGES3P1	1:8910428	3.83476	5.96507	1.37888	2.59192	1.86592	9.29453	3.91133	3.176	0.354182	3.97843	0.82655	0.492946
ENSG0000	TBC1D3L	17:379779	3.15571	6.03568	0.916385	0.825324	1.81113	5.08868	5.23781	1.85758	0	5.10081	0.74515	0.397562
ENSG0000	MAT2A	2:8553916	72.5324	97.7825	38.3962	39.2939	38.5586	67.6126	103.237	58.9429	36.6971	69.9549	0.5217	0.21089
ENSG0000	FAM19A2	12:617082	7.40387	1.87564	0.441934	0.755777	0.086584	4.88322	1.01563	0.328482	0	0	0.7277	-0.54336
ENSG0000	CMKLR1	12:108288	0.394779	2.68327	0.496524	0.247128	0.216047	5.96649	0	0.102485	0	0.010428	0.6942	0.480459
ENSG0000	GALNT11	7:1520256	38.9666	26.6196	46.5489	50.291	36.1501	24.985	22.4664	26.1415	50.4918	32.114	0.2202	-0.38336
ENSG0000	ANKHD1	5:1404018	49.0148	53.9562	35.8027	29.936	41.5519	46.182	50.044	44.2732	26.3595	75.7475	0.9707	-0.01469
ENSG0000	OSGIN1	16:838991	0.212358	0.863276	0.367104	0.851152	0.195175	1.15812	0	0	0	0.275819	0.5304	-0.81235
ENSG0000	BIVM	13:102799	13.9379	2.14219	7.84631	10.1002	5.65368	12.4138	7.71743	10.8917	1.84928	10.2763	0.97915	-0.01944
ENSG0000	N4BP2	4:4004291	6.85137	9.54002	2.2653	4.00308	3.71971	4.03333	5.24858	3.74068	2.01192	7.7668	0.4248	-0.36569
ENSG0000	PHF5A	22:414597	8.87457	4.88621	52.6372	41.6344	38.8125	10.5569	6.76314	53.5023	34.6659	19.9196	0.55665	-0.25229
ENSG0000	PSMD2	3:1842987	140.026	155.184	92.8487	128.757	88.7668	78.6146	99.9796	79.6137	78.7399	110.487	0.2001	-0.36797
ENSG0000	AP000688	21:360345	0.067186	0.046522	0.369136	0	0.567663	1.23954	0.329069	1.02988	0	0.6883	0.3487	1.40684
ENSG0000	SPRYD3	12:530061	19.6466	19.3995	15.7421	11.749	25.9952	14.1564	18.7415	25.2195	9.47658	32.4322	0.9703	0.030656
ENSG0000	ZNF581	19:556350	10.8348	11.6699	11.4049	7.37112	16.044	7.07261	15.2294	19.2258	15.1734	8.47173	0.72895	0.230858
ENSG0000	PGM1	1:6344076	24.5049	15.6294	28.1362	30.0829	30.7268	33.8795	26.2013	28.7087	19.3211	27.9914	0.98065	0.009444
ENSG0000	RP11-107	2:1443840	0.196966	0.385843	0.0793	0.572547	0.171588	0.119784	0.707424	0.108921	2.20496	0.410737	0.4571	1.38133
ENSG0000	AC005796	19:178712	0	0	0	0	0.09931	0	0	0	1.83702	0.238661	0.2525	4.10951
ENSG0000	NSRP1	17:295605	27.0824	23.5241	47.5166	38.7423	61.8593	33.9914	45.1548	40.7847	41.2297	78.5619	0.70665	0.22634
ENSG0000	LINC0017	7:6620519	0.619985	0.480343	0.35108	0	0.279271	1.70466	1.32768	0.215279	0	1.73025	0.29265	1.22753
ENSG0000	NMRK1	9:7506057	30.9258	19.0582	31.6166	34.9306	15.4375	64.1277	29.5627	51.054	90.8431	25.9011	0.0165	0.951013
ENSG0000	MGST2	4:1396657	20.1877	5.13131	3.70889	5.25107	3.90274	27.7937	13.4434	2.75297	3.65447	1.4561	0.586	0.428566
ENSG0000	TMEM80	11:644232	33.9623	35.4245	49.9934	24.7798	34.7432	54.504	41.8301	36.5215	27.0708	42.5675	0.7709	0.138464
ENSG0000	FAM210B	20:563589	9.38689	16.9957	7.52762	7.63082	6.45669	5.37466	8.41411	7.26084	9.54399	8.45553	0.7071	-0.17469
ENSG0000	RP5-1009	20:380835	2.48936	0.281435	0.829921	0	1.45127	0	0.055719	0.129926	0	1.24873	0.45475	-1.72447
ENSG0000	C16orf62	16:195552	23.8636	24.1123	14.4657	14.4297	14.9272	21.1637	9.93198	7.65683	10.8813	20.3721	0.35485	-0.40194
ENSG0000	PSMD7	16:742967	100.992	112.164	122.133	115.253	156.817	149.701	139.406	123.145	119.521	88.3267	0.99695	-0.0012
ENSG0000	ANO9	11:417932	19.664	24.5213	10.3522	11.7565	11.3313	25.8064	18.8342	13.6742	6.85007	27.3923	0.49575	0.299712
ENSG0000	FCGRT	19:495068	18.9327	87.4499	34.2142	32.1437	41.4559	98.9483	27.5807	18.8424	21.0649	16.5857	0.60115	-0.21243
ENSG0000	PTGIR	19:466204	7.14058	10.0338	0.500813	0.456417	0.212316	6.27627	5.22098	0.100339	0.142028	0.494826	0.3996	-0.89012
ENSG0000	CNN2	19:102658	412.404	593.419	596.072	376.85	708.02	254.014	230.098	309.438	197.952	465.913	0.01695	-0.90114
ENSG0000	RP11-660	11:714281	36.7489	27.9101	66.487	57.6025	33.3224	41.3503	20.3982	32.6108	37.5933	7.81682	0.3233	-0.69791
ENSG0000	PARP10	8:1439771	125.194	112.109	33.2284	35.0918	41.2071	129.093	268.158	95.4161	49.1486	102.734	0.037	0.799106
ENSG0000	RENBP	X:1539352	0.655642	2.70788	0.524262	0	0.530976	8.98945	1.55578	0.815811	0.314261	1.58386	0.06905	1.85128
ENSG0000	PGM2	4:3782663	19.9371	17.441	13.4912	11.4731	13.5714	23.7234	15.9058	11.6895	11.8597	13.6634	0.8311	-0.08383
ENSG0000	RP11-461	16:361172	1.32385	4.17307	0.376238	0.259354	0.207786	0.69532	0	0	1.59004	0.981886	0.7176	-0.67047
ENSG0000	NDUFS8	11:680306	104.537	93.339	183.509	178.704	150.612	175.82	121.255	147.698	132.213	38.2058	0.34655	-0.32826
ENSG0000	ZNF689	16:305859	5.5892	7.75503	8.8141	7.70543	7.57799	1.78073	2.9725	8.80445	11.7742	13.7974	0.8547	0.083527
ENSG0000	SFI1	22:313995	40.1364	73.1295	18.3648	16.5029	12.7951	36.5694	26.1817	26.4184	24.5658	27.3027	0.93995	-0.04493
ENSG0000	SELENON	1:2580017	1.20386	4.65071	0.594154	0.856273	1.66193	2.48424	1.5914	1.69574	1.80155	3.21796	0.88195	0.176542
ENSG0000	FUOM	10:133355	12.5751	5.32097	18.3917	28.5979	12.0542	33.1697	19.2988	28.3425	24.0881	5.43201	0.4661	0.407964
ENSG0000	MARCKS	6:1138573	0.452521	3.17141	0.226728	0.868565	0.866212	4.93058	0.02482	2.54795	9.25342	1.12744	0.0156	1.65651
ENSG0000	ALG11	13:520123	14.5385	9.06818	13.851	7.37539	22.0194	29.9306	13.9583	6.46237	11.3374	20.9026	0.872	0.076358
ENSG0000	BMI1	10:223159	26.9458	22.1931	14.6676	11.8669	11.1938	12.9176	13.5466	15.0886	10.2733	15.9111	0.5234	-0.36323
ENSG0000	NLRC3	16:336509	28.5505	28.7528	8.85662	8.46332	13.5547	22.4379	14.972	14.4924	12.9828	29.3275	0.91205	0.060311
ENSG0000	CD3D	11:118338	610.46	407.884	862.737	838.946	416.859	903.779	745.286	985.627	811.329	156.528	0.58405	0.21079
ENSG0000	HLA-DRB5	6:3251734	27.6072	123.131	46.5914	44.3712	10.0199	266.052	126.543	178.162	88.9473	20.4098	0.00015	1.46445
ENSG0000	C10orf11	10:754305	0.400129	1.6223	0	0	0	6.66205	0	0	0	0.347	0.1609	2.15403
ENSG0000	CPOX	3:9849760	12.6922	13.8355	6.22885	5.39515	5.85717	7.33473	9.14319	11.8054	16.2888	9.53638	0.80535	0.316533
ENSG0000	RPL10P16	19:126432	37.1223	11.151	62.4864	12.0298	7.12356	32.9883	9.88299	38.1977	8.89797	1.51745	0.75075	-0.52017
ENSG0000	RP11-574	1:1602157	0.818202	1.298	0.677903	3.22419	1.16515	2.43487	1.58654	2.73202	4.49805	0.343939	0.59415	0.688822
ENSG0000	AL928768	14:105703	0.134801	7.57658	0	6.39729	0.310874	0	0	1.09185	0.14231	0.26127	0.1047	-3.5867
ENSG0000	HBG2	11:524808	0.054337	0	0	126.209	0.031143	0	0	10.9475	0	0	0.3296	-3.13338
ENSG0000	KDM1B	6:1815532	0.857282	1.7747	0.590792	0.021209	0.478265	0.540748	0.368168	0.689958	0	1.13341	0.74145	-0.4552
ENSG0000	TRGV8	7:3833034	30.6197	6.28854	76.3587	30.5078	30.0494	4.75011	33.4571	41.1027	15.6493	20.0528	0.303	-0.62294
ENSG0000	STAG3L5P	7:1003077	27.6053	31.1132	8.05846	13.8911	6.7738	23.5938	13.6157	8.94327	14.0095	2.80248	0.64555	-0.41503
ENSG0000	GOSR1	17:304773	17.4358	19.1156	33.4773	34.193	36.1262	21.3535	25.9472	30.349	19.3788	50.6865	0.85545	-0.09443
ENSG0000	CTB-63M	5:1663823	1.43127	1.55347	4.52039	6.43186	3.17591	3.53597	3.31991	1.60589	2.75337	0.482513	0.7841	-0.57232
ENSG0000	MARS2	2:1976931	4.0167	4.1651	3.22942	2.85974	3.34124	0.630159	1.47125	3.54463	0.017544	5.53194	0.27105	-0.80795
ENSG0000	RPL15P3	6:1251410	21.8832	14.2149	34.4587	46.8884	12.7215	17.1309	14.6146	17.0345	29.3924	2.10244	0.8845	-0.20679
ENSG0000	SURF1	9:1333517	58.394	50.6294	85.124	73.5937	87.361	66.9443	56.8257	70.7766	60.4932	44.0166	0.4832	-0.29396
ENSG0000	AMPD2	1:1095486	50.0945	41.1881	27.4791	49.103	27.2699	17.374	29.4512	28.0384	33.7952	43.1838	0.5335	-0.32323
ENSG0000	AC025335	17:791332	1.37445	4.64037	1.8371	3.09382	1.96919	0.431038	2.40141	4.49375	3.11671	1.92461	0.99135	0.007608
ENSG0000	ANP32A	15:687785	43.488	40.9679	9.39366	5.86761	33.1815	32.8751	50.7876	14.0355	9.73097	18.325	0.32145	-0.49793
ENSG0000	MST1P2	1:1664562	0.152013	0.74679	1.08746	0	0.404328	0.150198	0.292745	0.273106	0.319354	0.349426	0.7383	-0.7634
ENSG0000	ZNF416	19:575715	2.54185	3.7854	1.30385	1.45612	1.50255	2.1936	2.54551	3.83469	0	2.90363	0.89585	0.138994
ENSG0000	EVL	14:999714	84.0143	189.996	92.6108	92.1007	147.537	174.592	171.474	163.749	86.263	298.148	0.1153	0.547144
ENSG0000	YIPF6	X:6849832	13.3952	10.9221	13.2229	10.0033	9.21045	22.5	35.5412	14.2481	6.01374	10.8588	0.5356	0.522385
ENSG0000	UBA52P6	9:2180254	0.337463	0.554642	0	0.339468	0	0	0.534095	0	2.77118	0.9045	0.26085	2.2747
ENSG0000	PIN1	19:983525	158.079	108.169	221.184	219.967	201.166	194.088	212.693	206.073	169.814	62.0048	0.5967	-0.1757
ENSG0000	EXOSC3	9:3751089	34.4017	34.0554	37.2092	22.7681	35.9906	52.7922	60.7798	35.0134	40.6871	24.5721	0.64835	0.332048
ENSG0000	CLSTN1	1:9651731	14.1839	17.591	8.80232	6.70191	18.5744	8.92055	12.8563	15.3437	11.6556	30.1657	0.7138	0.374705
ENSG0000	YTHDF2	1:2873662	71.0856	79.9265	91.6955	95.2991	80.751	46.895	64.2633	53.3582	63.9982	78.2623	0.10765	-0.52758
ENSG0000	CTU1	19:510976	15.0721	9.07591	15.1408	4.71434	27.1994	10.2092	9.89776	8.10678	4.81392	5.69197	0.1036	-0.93716
ENSG0000	DUSP8P5	10:737305	3.51493	10.0316	7.307	6.47132	3.18835	1.2955	4.26631	5.89768	5.9599	13.3383	0.97375	0.027518
ENSG0000	SEPHS2	16:304436	60.7649	53.7454	73.4627	55.4062	62.765	62.7149	42.8151	46.9294	73.4369	43.4156	0.3122	-0.33556
ENSG0000	RP11-96D	16:682645	0.90955	1.26838	0.075475	0.100602	0.108373	0.175583	2.47293	0.125821	0	0.371813	0.8823	0.293241
ENSG0000	SEPHS1	10:133174	18.2454	22.5904	9.97287	10.7971	11.3643	22.8781	14.9857	10.9915	14.9459	12.8856	0.6899	0.160528
ENSG0000	ZNF517	8:1447988	0.66126	1.4493	0.68589	0.045845	0.792566	0.850058	1.53749	1.60623	0	0.913406	0.72015	0.34596
ENSG0000	GCSAM	3:1120863	0.603183	11.3859	1.53726	0.565385	0.128313	0	5.15645	2.42996	0	0.72819	0.46285	-0.7236
ENSG0000	SMC6	2:1766381	17.1768	16.7699	5.3299	4.14653	11.4179	14.5546	17.7728	4.72673	7.95365	19.8518	0.7581	-0.13236
ENSG0000	KLF13	15:313268	35.5387	46.7589	15.4447	18.6623	48.4194	26.3437	56.6856	20.9919	21.0447	34.3224	0.9608	-0.04633
ENSG0000	BMS1	10:427828	8.53888	10.7827	5.32901	4.9353	7.1712	6.77155	7.42021	5.14488	3.32502	6.84799	0.3299	-0.34202
ENSG0000	HSH2D	19:161116	32.8927	36.4683	45.0069	98.9763	30.9328	30.6924	94.4482	51.993	92.296	31.6617	0.6505	0.307119
ENSG0000	CENPQ	6:4946337	9.51547	9.79619	3.07795	2.47255	3.15119	2.52838	5.67944	2.39758	0.641782	1.53009	0.18165	-0.95971

ENSG0000	EFNA1	1:1551274	0.100186	2.79381	0.846657	0.490804	1.07853	0	0	0.88133	0	0	0.342	-2.37884
ENSG0000	GLT8D1	3:5249533	55.7402	51.8579	51.6694	54.0415	32.5444	44.4258	26.778	30.6793	33.7523	29.3244	0.26615	-0.60753
ENSG0000	TRIM8	10:102644	40.7847	36.0261	31.6468	39.5261	33.5258	26.605	42.8235	35.1576	22.3316	38.0325	0.4919	-0.23244
ENSG0000	ALDH16A1	19:494462	19.1727	21.8666	7.34835	5.76126	9.47479	12.3902	13.1458	13.8447	5.84472	14.4523	0.91415	-0.1109
ENSG0000	LL22NC03	22:222839	1.18097	2.39162	0.260793	0.045616	0.233076	0.989392	0.540338	0.206084	0.170115	0.981916	0.4837	-0.27833
ENSG0000	MYO15B	17:755880	9.14743	18.2861	2.35208	0.873048	1.16372	8.27542	4.96554	3.13777	3.56876	1.64661	0.74175	-0.19119
ENSG0000	COX20	1:2448353	41.0625	36.893	34.807	36.7257	20.7136	45.3892	43.2798	39.1375	35.3136	17.5109	0.853	0.186171
ENSG0000	ITPKA	15:414933	1.4929	0.891344	2.94522	0.844624	3.88326	0	1.52205	1.32223	0.536874	0.801786	0.27925	-1.17948
ENSG0000	COASY	17:425361	51.1024	59.8702	47.0805	51.3968	41.404	27.1863	37.8317	21.9388	39.1447	35.4142	0.1063	-0.56993
ENSG0000	SNRPB	20:246163	254.466	226.258	394.422	417.426	321.986	326.507	407.573	462.02	392.916	119.095	0.8647	0.060039
ENSG0000	LINC0098	12:922853	5.39362	1.89871	4.68441	7.03717	2.30317	1.9297	0.06221	2.85955	4.47853	3.6857	0.32645	-0.70788
ENSG0000	ZNF623	8:1436360	4.32713	3.22236	3.28766	4.46124	2.22266	0.173943	1.76413	1.05714	2.18533	2.6372	0.11325	-1.26433
ENSG0000	CTSK	1:1507962	0.789974	4.52061	0.941119	1.4487	0.461754	0	3.6081	2.14958	2.34713	3.44985	0.4722	0.563213
ENSG0000	CEP128	14:804769	6.54105	6.7858	2.03692	2.58077	2.51291	5.36831	8.06916	2.80401	0.777338	5.91415	0.91815	-0.06848
ENSG0000	RP11-45M	17:170425	0.387509	1.00056	0.366264	0.058305	1.49302	0	1.23295	1.73562	0.031852	6.84289	0.43205	1.52016
ENSG0000	JMJD1C	10:631672	45.8896	47.5329	14.4546	14.675	29.8282	23.8846	43.0667	16.2459	19.5744	48.3993	0.8489	0.114354
ENSG0000	ZNF853	7:6615616	2.94812	3.7964	2.39813	0.438709	4.09862	0.832751	4.28351	2.26302	3.06486	4.0402	0.7888	0.145599
ENSG0000	CD1C	1:1582897	0.430202	18.4446	0.957192	2.17772	0.387501	0	0	0	0	0.072879	0.20405	-8.29562
ENSG0000	TRIM16	17:155654	6.10295	6.93805	3.51353	1.53135	1.19011	5.27223	5.78543	4.89441	1.74802	2.7239	0.6605	0.210608
ENSG0000	A2M	12:906517	9.69702	0.713625	3.25857	3.94204	3.83876	8.27776	0.139799	4.17642	5.80956	9.73236	0.6414	0.473628
ENSG0000	FAM126A	7:2288937	7.54756	4.77551	2.78533	0.668358	1.82916	12.3179	18.0601	3.87516	0.96454	3.74961	0.1021	1.06376
ENSG0000	GNG11	7:9392169	0	0.192721	2.34632	6.7588	0.345228	0	0.144855	0	0	0	0.0134	-6.07335
ENSG0000	EIF1B	3:3980891	58.31	85.7967	55.3404	42.5742	43.5782	95.4315	86.3768	81.552	35.0144	37.3017	0.49125	0.240447
ENSG0000	SYTL2	11:856942	100.823	25.4704	33.9025	55.9301	39.0674	58.742	53.2511	27.428	52.3393	63.9874	0.771	0.097819
ENSG0000	GABPB2	1:1510705	2.29272	2.59607	3.47129	1.4269	2.34341	2.54764	3.54455	3.25385	0.461322	4.7716	0.85415	0.098642
ENSG0000	LPP	3:1881512	17.7566	16.3355	7.97874	8.20346	5.90517	8.55999	12.3083	1.92397	13.0632	9.97615	0.5886	-0.41213
ENSG0000	RP11-758	17:596185	3.50978	0	4.47808	2.25579	2.73203	4.34819	4.2674	1.20102	1.10693	2.38021	0.9048	-0.14698
ENSG0000	RP11-403	1:1451640	12.7126	22.7504	13.9374	11.8921	11.8494	3.79057	10.6614	8.2214	8.38165	12.8002	0.13615	-0.94176
ENSG0000	SFT2D3	2:1277010	4.741	7.24557	2.94817	5.15158	0	0.12393	3.58513	0.293865	6.28982	0	0.8215	0.638847
ENSG0000	TMBIM4	12:661165	107.466	111.514	157.567	83.2084	94.9339	200.701	257.258	190.223	78.2668	56.9203	0.7623	0.139211
ENSG0000	NOTCH1	9:1364944	1.85938	3.48192	0.732999	0.288962	1.82824	3.73855	7.31105	3.84527	0.409158	8.01462	0.02015	1.35469
ENSG0000	IP6K2	3:4868800	101.921	94.4476	167.895	109.498	95.6792	88.0962	103.036	127.089	105.164	105.009	0.58955	-0.17221
ENSG0000	RP11-156	1:2449693	4.37335	5.35117	1.27934	1.74039	0.159396	0.586144	0.909	0.89505	1.5929	0.077331	0.4712	-1.55033
ENSG0000	RP5-821D	22:418312	6.88562	5.7758	12.1133	6.2687	6.53485	2.5254	2.56618	13.1596	1.21621	8.1596	0.70605	-0.39186
ENSG0000	WDR34	9:1285525	8.12576	13.0471	3.93631	4.44758	5.88711	16.4076	15.8131	18.1957	4.64294	12.2304	0.43105	0.862876
ENSG0000	INSIG1	7:1552977	32.9465	31.3671	27.2326	37.1148	17.1454	48.1063	54.9339	52.173	53.7019	23.2226	0.07155	0.592702
ENSG0000	NAA15	4:1392661	9.36741	10.4119	9.38101	6.24089	8.59257	11.1588	7.71789	6.31932	10.8519	19.3062	0.8924	0.085547
ENSG0000	NDUFA3	19:541027	133.271	76.3344	176.788	169.221	76.604	146.255	148.055	102.209	67.0615	16.9741	0.61955	-0.52953
ENSG0000	ACADSB	10:123008	7.08174	7.89036	10.4709	5.84727	3.14418	4.71332	2.25231	2.40535	0.576824	4.19172	0.00365	-1.83759
ENSG0000	AC009948	2:1784139	4.12307	5.62348	5.23429	6.43883	2.69993	3.38555	9.49304	8.0122	8.78929	6.78823	0.49315	0.570857
ENSG0000	SPDL1	5:1695836	11.5912	12.5939	5.40294	7.01966	5.59771	4.15868	11.8543	5.35536	11.2067	5.67188	0.6958	0.173654
ENSG0000	CCDC157	22:303319	4.20719	2.25699	1.3849	2.34085	1.519	3.88017	3.95078	0.78999	3.30792	1.15397	0.96525	0.041524
ENSG0000	URI1	19:299236	34.7182	27.4597	12.9288	10.063	16.7797	26.939	24.956	18.5626	10.819	24.0099	0.976	-0.01094
ENSG0000	EPPK1	8:1438573	0.204536	3.75199	0.141239	0.316147	0.313408	0.06668	0.089978	0.063958	0.470258	1.30184	0.1742	-1.08692
ENSG0000	COA7	1:5268445	1.29328	1.10127	1.39857	1.94302	1.20937	0	2.31532	1.40416	0	0.658887	0.3504	-1.06673
ENSG0000	NUBP2	16:177320	62.8478	71.5226	96.3814	107.325	83.6594	73.0938	95.2131	84.3938	74.9717	46.0849	0.69785	-0.24364
ENSG0000	RP11-85K	14:350449	0.184424	0.402354	0.182775	0.074043	0.271869	0.271642	1.22852	0.041386	2.19928	1.29834	0.1638	2.13923
ENSG0000	TNNC1	3:5245110	0.335245	0	0.134509	1.47803	0.34452	0.587632	0	1.62225	1.59216	0.3482	0.4565	0.994301
ENSG0000	SLC27A4	9:1283406	7.10696	5.98857	2.7681	5.76212	4.39994	6.33188	5.68547	4.05144	10.3374	9.17573	0.30765	0.630828
ENSG0000	MAP3K6	1:2735518	8.30294	18.2353	4.5444	9.15491	4.60304	12.9466	11.0996	7.97096	9.54092	6.96529	0.73575	0.242452
ENSG0000	NACA	12:567124	473.814	483.041	536.551	453.346	471.943	614.628	593.081	486.085	413.421	92.0024	0.7246	-0.14355
ENSG0000	UNC45B	17:351478	0.353184	0.090808	0.550362	0.625329	0.58561	0	0.479578	0.220761	0.437884	0.245409	0.53125	-0.70401
ENSG0000	STYX	14:527301	23.6786	16.6238	5.24733	5.76832	4.81029	4.83481	11.2014	4.77999	10.465	7.77348	0.64635	-0.16453
ENSG0000	TMEM259	19:999795	190.163	255.465	37.9649	67.2172	47.7178	136.17	220.102	62.363	60.3906	68.513	0.76505	-0.10689
ENSG0000	ZNF599	19:347580	5.09777	8.537	8.72649	3.63029	6.65502	0	2.76502	4.60909	4.55044	2.90633	0.3327	-1.12741
ENSG0000	WDR74	11:628322	41.3147	33.3068	43.2984	37.8908	33.9686	36.7919	39.2053	35.6078	32.1121	18.0868	0.41145	-0.27639
ENSG0000	IFT80	3:1602256	10.7775	15.161	4.62774	5.01433	2.11448	5.62778	8.72392	5.11894	6.03993	7.94487	0.8532	-0.11255
ENSG0000	AC068533	7:6607579	1.73222	0.548914	0.671556	0	1.95586	4.44569	2.58443	0.1049	0	0.593914	0.96795	-0.07179
ENSG0000	LRR1	14:495709	9.66889	13.5902	10.7417	11.7006	6.99999	12.7164	16.4662	15.2804	12.3048	6.637	0.7719	0.293862
ENSG0000	CCL8	17:343190	0	0	0	0	0	6.72387	0.887378	0.450683	0	0	0.04545	inf
ENSG0000	LYSMD3	5:9051561	13.3665	7.03245	4.15555	4.07465	5.11744	14.8205	8.36357	8.41121	8.12785	6.02383	0.3933	0.405346
ENSG0000	MAD2L1B	6:4362953	22.6969	27.6393	33.3606	24.9313	31.3958	54.8887	68.3843	37.9495	30.749	26.8373	0.1328	0.549255
ENSG0000	MTX2	2:1762693	18.2184	11.0377	20.9965	21.5932	15.2945	18.4083	18.4747	17.7801	28.7345	15.7514	0.58655	0.227734
ENSG0000	CCDC69	5:1511810	60.1342	69.272	35.2477	35.0277	50.4485	60.9135	52.4755	34.3838	29.3515	46.9878	0.4909	-0.22958
ENSG0000	H2AFV	7:4482679	178.503	179.232	171.311	140.831	134.903	234.623	182.693	179.058	207.435	90.5873	0.673	0.154647
ENSG0000	METTL17	14:209897	60.8531	69.4546	46.7901	66.2852	32.1486	97.4312	88.8895	58.3217	77.3558	56.9701	0.1353	0.507371
ENSG0000	ST3GAL3	1:4365015	3.58059	6.72388	3.99399	1.66643	4.68907	1.26961	6.32392	4.53661	0	4.94989	0.6451	-0.2902
ENSG0000	RP11-385	6:4747724	2.52746	1.24172	5.14152	1.25031	0.733088	0	0.248607	2.57531	0	1.33385	0.5697	-0.90691
ENSG0000	TMEM163	2:1344557	0.247964	0.030002	0.17669	1.95293	0.922322	0	0	1.18513	0	0	0.60775	-1.4011
ENSG0000	PI4KB	1:1512800	43.3304	43.8231	33.5219	46.5035	34.7167	26.4074	35.3745	29.4154	25.184	50.969	0.48135	-0.23843
ENSG0000	BORCS8	19:191455	16.7589	17.4717	20.6918	22.263	17.9877	27.6841	13.9898	16.7923	17.3036	9.34666	0.83075	-0.15474
ENSG0000	SUSD1	9:1120417	7.09878	7.01719	3.66078	3.49876	6.71281	1.19212	1.74331	1.80512	2.06099	6.5508	0.02845	-1.1834
ENSG0000	SCRN1	7:2992010	17.3705	2.0344	3.19999	4.89246	7.35782	1.8528	0	2.99677	2.62485	10.5606	0.0774	-1.1505
ENSG0000	ZNF394	7:9947284	36.9232	46.5194	40.1893	56.4952	32.5665	40.9525	33.1286	28.589	29.2684	34.5643	0.31545	-0.38221
ENSG0000	SLC27A2	15:498582	3.56979	1.84918	0.718011	0.766771	1.35317	31.7084	51.347	14.2735	0	7.0076	0.00325	3.66971
ENSG0000	TRAV38-1	14:222025	3.63213	20.8335	0.343948	1.59121	0.241897	6.33558	8.16996	0	0	0	0.6948	-0.89322
ENSG0000	RPL13A	19:494462	651.277	652.071	1625.86	949.419	1381.67	592.256	638.5	1230.04	747.204	326.739	0.21845	-0.61992
ENSG0000	RP11-849	17:836556	0.90431	2.62538	1.21669	2.20301	0.147135	0	2.40453	0.474156	0.273879	0.935354	0.64335	-0.71973
ENSG0000	DUS1L	17:820575	160.369	206.184	20.8741	10.5952	32.348	112.274	226.827	22.5838	12.6013	33.8165	0.62185	-0.24599
ENSG0000	SMAD3	15:669841	11.8286	9.8298	7.35372	6.43953	8.66668	2.50834	3.67199	7.46349	5.37193	17.0056	0.3518	-0.38818
ENSG0000	ENG	9:1278150	2.6485	5.01554	1.23241	1.586	2.37899	28.5592	4.12087	2.54999	4.60367	3.74938	0.0191	1.68791
ENSG0000	MSH5	6:3173994	3.28093	5.5216	2.40457	3.99362	1.78616	7.92062	2.66749	2.40709	5.18961	3.73782	0.6922	0.248619
ENSG0000	ZFYVE28	4:2269581	7.20942	5.17341	11.4948	9.84712	13.6299	25.7452	23.7175	26.1921	33.9805	31.6379	0.0025	1.54596
ENSG0000	TMEM106	17:432118	3.36208	2.81315	1.22001	3.12337	1.41172	6.52987	8.82142	3.74321	3.68095	3.81455	0.1419	1.06394
ENSG0000	FKBP2	11:642410	76.8905	43.6876	145.026	134.63	172.488	107.865	120.565	145.327	113.895	75.3863	0.75645	-0.11018
ENSG0000	QRICH1	3:4902970	34.2753	40.331	82.8164	58.0792	108.183	29.7349	39.6813	73.984	46.7622	119.34	0.5352	-0.39243
ENSG0000	SNAPC1	14:616810	7.19398	4.4555	5.00396	7.51706	4.50272	3.40362	14.0952	5.85697	6.68815	5.26768	0.81955	0.337856
ENSG0000	ITPRIPL1	2:9632533	15.9637	7.44338	11.4105	9.2957	14.6247	12.3531	22.2982	19.6248	11.7251	20.2157	0.3241	0.468133
ENSG0000	TRPM2	21:443501	3.10322	1.02821	0.473134	1.09785	1.29046	4.46074	4.3638	2.24546	1.03673	2.93949	0.18595	1.27646
ENSG0000	LRRK1	15:100849	0.090834	1.40571	0.207176	0.131843	0.067549	1.86904	0.077376	0	0	0.657978	0.74085	0.331712
ENSG0000	EMG1	12:697089	3.38132	3.87915	22.7827	18.847	14.6938	5.53025	2.73235	27.0989	9.46483	9.54673	0.65405	-0.37827

ENSG0000	UBE2D2	5:1395264	89.8389	92.9692	36.4156	31.7687	36.0609	93.3561	116.949	42.7436	30.0042	31.701	0.843	0.082423
ENSG0000	PPP5C	19:463469	28.8161	25.2742	18.6829	25.1265	22.5152	29.6291	35.1509	26.31	16.3557	23.9672	0.84225	0.067968
ENSG0000	AP4M1	7:1000927	5.89448	6.15269	6.5728	8.62491	7.06767	20.7192	7.3105	9.92179	5.93808	8.7305	0.5527	0.473287
ENSG0000	LZIC	1:9922112	20.4818	23.9045	14.9506	22.7637	18.0092	19.918	32.7707	22.823	17.4734	9.86004	0.86065	0.106885
ENSG0000	RP11-401	17:670704	1.03676	0.542435	1.08829	2.14286	0.871097	0.775479	0.409773	0.984587	1.2318	0.659122	0.65735	-0.4998
ENSG0000	ZNF285	19:443265	1.23956	0.399439	0	1.8455	0.015338	0	0	0	0	0.097383	0.0223	-5.60471
ENSG0000	ZNF530	19:575998	2.54512	1.42946	0.579855	3.29928	1.36349	0	2.08588	0.912637	2.88863	1.22692	0.67915	-0.41841
ENSG0000	CALCOCC	12:537085	78.8461	101.182	60.2424	49.6696	48.7635	40.2451	37.6971	49.7216	46.8027	74.408	0.23875	-0.37914
ENSG0000	CPE	4:1653611	0.081982	1.07977	0	0	0	0	2.29822	0	0	0	0.55115	1.51436
ENSG0000	LRBA	4:1502645	14.3064	10.5848	6.1128	4.85642	9.22167	10.2451	14.5766	5.88535	5.33609	9.19433	0.5602	0.300986
ENSG0000	MYO5B	18:497821	0.150072	0.133231	0.085108	0.029277	0.063651	4.2266	0.791218	0.300074	0.583499	0.845054	0.0118	3.5446
ENSG0000	AC009961	2:1596707	1.23564	4.00019	1.49988	0.360163	0.726099	1.06902	2.44356	0.568796	2.21048	0.745782	0.9682	-0.0494
ENSG0000	CTC-429P	19:164790	3.01717	3.62094	5.73602	2.69103	2.19637	1.39296	6.03992	1.71411	3.75785	1.09563	0.9412	-0.08125
ENSG0000	LTB4R	14:242998	12.9447	8.21657	14.3582	11.3426	14.9935	13.7663	8.07869	5.24098	11.6627	25.2713	0.91755	-0.07905
ENSG0000	AC009299	2:1610962	1.25353	0.48231	0.102605	0.160286	0.372486	0.987331	0	0.516711	0	0.220758	0.6939	-0.54895
ENSG0000	ARL5B	10:186594	2.42132	3.40246	0.629727	0.423426	0.876712	2.85234	3.09824	2.39823	2.72625	2.61676	0.0961	0.902919
ENSG0000	ADAM10	15:585875	44.147	26.8523	9.30022	11.6858	8.24215	34.4345	11.2806	13.7065	16.3127	14.843	0.225	-0.59732
ENSG0000	TIMM29	19:109287	24.4963	27.9924	9.55361	23.5426	11.0457	25.074	19.7616	7.59907	20.9236	5.60241	0.5474	-0.2915
ENSG0000	CEP95	17:644996	18.63	32.9559	10.7618	11.715	9.11717	20.7686	11.6875	13.7567	14.6121	16.8109	0.95325	-0.03174
ENSG0000	CROCCP2	1:1661896	9.80848	15.2287	9.21323	9.31942	3.67396	8.68458	6.2728	6.22705	2.62871	3.24218	0.2143	-0.79166
ENSG0000	LIMA1	12:501757	9.01781	10.8845	2.24936	4.48951	4.19956	20.2893	14.2166	5.67743	4.95177	10.6639	0.0777	0.810138
ENSG0000	AP001442	21:258805	0	0.794258	0	0	0	0	0	0	2.47441	0	0.23225	1.9762
ENSG0000	FAM209B	20:565332	0.624958	1.74583	0.131734	0.534317	0.071429	5.32332	0.143981	0.787882	0	1.29917	0.4497	1.31881
ENSG0000	AC091729	7:1152070	2.32601	0.726907	3.24911	2.75729	1.44849	0.098937	0.159088	3.13863	3.87007	1.86734	0.90845	-0.16713
ENSG0000	DUT	15:483123	90.6403	82.4355	105.79	92.3321	72.6121	126.052	108.732	87.1218	64.6853	35.1602	0.65005	-0.18914
ENSG0000	C7orf50	7:996985-	30.998	35.8509	101.636	67.1982	64.1726	59.6711	45.9507	57.3516	77.3344	24.4561	0.6479	-0.19417
ENSG0000	ZNF57	19:290089	1.93855	3.6121	4.00816	6.50065	2.99369	0	2.05658	4.61801	0	3.13135	0.32095	-1.08361
ENSG0000	PLEKHN1	1:966496-	0.346252	0.383504	0.654194	1.85706	1.47364	3.10096	1.83309	1.80165	0.365905	5.77704	0.16625	1.28406
ENSG0000	GBP2	1:8910613	150.515	122.824	98.1853	83.385	112.292	193.494	215.311	158.434	89.4795	153.929	0.11995	0.5227
ENSG0000	SMPDL3A	6:1227890	0.348693	1.68801	0	0.043484	0.245049	19.1126	0.017536	0.635785	0.155226	0.273512	0.04045	3.10281
ENSG0000	MCM3	6:5226400	43.319	67.3879	33.6171	31.1866	35.0091	31.2455	38.8178	27.2782	15.44	48.318	0.1687	-0.43999
ENSG0000	JAKMIP1	4:6026198	58.8864	20.9776	73.5447	25.5412	56.5214	48.1405	39.5448	47.3918	18.1153	51.753	0.55605	-0.20423
ENSG0000	C6orf203	6:1070282	22.8688	22.4649	27.5816	18.253	22.8331	14.8741	25.599	23.0783	9.1545	11.2898	0.4676	-0.40438
ENSG0000	MTFR2	6:1362310	5.49167	3.61419	0.503066	1.89814	1.08031	8.97754	2.27228	4.4173	0	2.26556	0.6837	0.492069
ENSG0000	RBM5	3:4994000	56.9643	71.4666	44.7411	41.8533	35.2844	34.0735	72.9267	46.1011	47.4974	77.7796	0.62405	0.189479
ENSG0000	RP11-706	X:3817527	15.2504	21.3957	28.6622	20.9108	10.0146	16.4574	10.3033	19.5227	13.3569	10.2186	0.4552	-0.51583
ENSG0000	CAPN2	1:2237015	200.357	130.956	113.983	128.906	92.3938	115.931	142.588	89.1481	92.0525	99.1601	0.36395	-0.29542
ENSG0000	H3F3B	17:757764	1238.64	744.742	1889.57	1570.97	1331.41	1886.53	1741.39	1578.06	994.793	803.416	0.78555	-0.15175
ENSG0000	SDHD	11:112024	117.84	118.036	152.554	153.53	78.0443	157.616	114.224	110.815	161.155	37.2964	0.866	-0.07385
ENSG0000	CXCR4	2:1361143	157.705	155.704	487.69	2244.2	452.238	131.574	182.99	469.037	720.228	317.683	0.3188	-0.97569
ENSG0000	ABCA5	17:691472	6.31845	3.64184	8.89116	4.26371	11.2449	7.19094	5.22183	11.0234	9.94982	24.7706	0.4143	0.631294
ENSG0000	RNF5P1	8:3860066	0.22606	0.847164	0.516029	0.113394	0	0.100484	1.73464	0.089847	0.118396	0	0.91885	0.14763
ENSG0000	MEN1	11:648035	42.2513	61.1766	31.9804	21.1993	31.1488	18.5097	25.282	26.832	14.1672	34.4983	0.04775	-0.65205
ENSG0000	PPP1R3E	14:231853	3.8467	6.07084	2.15495	1.42222	2.07797	6.41924	3.95128	5.60791	7.84843	7.79372	0.232	1.09992
ENSG0000	FABP4	8:8143943	0	0	0	0	0	5.56916	4.60653	5.06101	0	2.9008	0.00005	inf
ENSG0000	PKD1	16:203981	8.17645	11.7724	1.36794	1.05513	3.47248	1.67357	8.50968	1.30751	2.57208	3.62231	0.6653	-0.35943
ENSG0000	LOXL3	2:7452664	0.138102	0.599044	0.039199	0.027972	0.064241	6.70454	0.268166	0.620604	0.232417	0.119278	0.03925	3.29033
ENSG0000	GAN	16:813149	0.481104	0.472941	0.625701	0.702043	0.565762	0.828909	0.851961	0.778416	1.02807	1.50748	0.2801	0.612068
ENSG0000	SCML1	X:1773744	0.541857	11.4523	0.146445	0.520003	0.24781	0	0.234266	0.23963	0.068685	2.16634	0.0446	-2.49872
ENSG0000	AC012358	2:5528231	2.11757	1.50915	2.31396	0.574423	0.529353	0	1.14393	0.775466	0	0.463156	0.0309	-1.28584
ENSG0000	HNRNPH1	5:1796141	55.5661	66.7995	173.82	181.196	90.7523	55.7084	74.29	196.456	236.556	186.803	0.52505	0.278624
ENSG0000	SRBD1	2:4538867	12.7138	11.6938	7.88569	13.9755	7.76997	8.85691	7.73393	4.00723	6.00608	7.49192	0.12055	-0.68894
ENSG0000	BOLA2B	16:301929	51.6867	30.8108	71.9651	66.7534	39.8056	83.0286	86.7666	61.9485	34.6748	16.8905	0.7932	-0.38402
ENSG0000	MPZ	1:1613047	0.155596	0.047796	0.093385	0.628166	0.229794	0	0.192956	0.403599	1.19206	0.526929	0.17615	1.33563
ENSG0000	NPL	1:1827892	4.91821	6.53111	5.29946	2.35245	2.78033	30.1034	8.57675	1.73585	9.2819	1.9646	0.0294	1.3307
ENSG0000	HLA-DPA	6:3306456	17.2372	69.0782	98.5904	94.6323	114.568	97.2488	78.3818	156.936	125.884	120.793	0.29335	0.475468
ENSG0000	IRF2BPL	14:770245	2.09683	1.62773	2.52838	2.05031	2.19047	2.60494	1.82723	2.6	1.46402	3.38914	0.8495	0.158593
ENSG0000	KB-1572G	22:236084	2.55347	3.48201	1.23134	0.931992	0.384239	2.29686	1.5276	1.40316	3.53093	1.78866	0.4001	0.782617
ENSG0000	LYPLAL1	1:2190866	13.3735	9.08754	16.5249	13.2478	4.61116	25.6084	12.3095	19.2069	16.6124	4.13041	0.26355	0.542342
ENSG0000	NARFL	16:720580	35.8197	46.908	20.6895	35.0337	20.6147	36.969	38.8155	18.5822	8.86876	25.9288	0.4928	-0.35437
ENSG0000	CTC-523E	19:348378	14.4133	21.7081	18.6499	20.7245	13.7047	4.21707	9.81918	13.3029	5.01917	4.57805	0.23195	-1.24597
ENSG0000	KBTBD4	11:474659	17.068	22.2629	19.9003	21.9225	18.1812	11.1186	17.568	13.1173	6.03748	18.8815	0.46365	-0.69884
ENSG0000	TTI2	8:3337082	12.9688	10.4849	19.1317	10.8583	16.6471	11.5615	12.2644	13.7709	16.3223	13.5331	0.84875	-0.11458
ENSG0000	RP11-549.	5:9361806	0.362345	1.30641	0.086459	0.062679	0.045638	0.636606	0.405883	1.69391	0.316032	0.616748	0.51565	1.06869
ENSG0000	NAB1	2:1906467	3.39877	3.26592	1.81369	4.42106	3.09653	3.72575	11.2511	7.29259	4.46595	6.79599	0.06705	0.986038
ENSG0000	CABYR	18:241389	0.227382	0.993594	0	0.656936	0.267955	2.8917	0	1.23305	0	0.324946	0.29205	0.963209
ENSG0000	OSBPL5	11:308711	4.18431	4.45797	2.96562	1.05768	7.0223	3.2716	3.40226	2.57782	0	2.92488	0.42555	-0.62374
ENSG0000	VAMP7	X:1558812	37.8413	39.5088	20.474	21.156	13.3555	22.5547	19.5829	12.9811	14.1738	15.2664	0.0561	-0.6417
ENSG0000	AC093627	7:135852-	0.960896	3.11263	0.460574	1.15942	0.469362	1.5209	2.61918	0.87115	0.322365	0	0.88195	-0.17499
ENSG0000	DCTD	4:1828900	73.8554	77.3163	73.3164	76.1796	59.0181	68.9547	60.6598	81.3287	76.3856	52.2904	0.72355	-0.1089
ENSG0000	STK24	13:981425	49.7547	61.5213	21.0954	8.25331	30.488	35.3453	45.2264	29.3961	28.252	53.1434	0.7828	0.09621
ENSG0000	OGG1	3:9749943	18.3007	16.4009	15.3825	19.3445	20.2639	40.1007	18.2011	16.8565	6.78303	15.5255	0.91685	0.055503
ENSG0000	TTL	2:1124821	3.35239	4.81668	1.8342	0.706155	1.09549	3.911	6.6252	2.73752	0.094632	5.40976	0.63335	0.286498
ENSG0000	FAM215A	17:439144	0.384973	1.80194	0.083759	0.456538	0.141874	0	0.38959	0	0	0.092426	0.22825	-2.73387
ENSG0000	IGHV3-33	14:106359	0	0.896527	1.35571	30.1841	0.174279	0	0	1.00368	0	1.11933	0.78705	-0.40655
ENSG0000	AC011893	2:1357878	0.172676	0.049069	0	0	0	1.52779	0.312509	0.522789	0	0	0.37295	3.6304
ENSG0000	PPP4C	16:300759	155.705	161.32	208.459	165.31	187.298	171.074	191.266	202.936	158.831	168.404	0.93545	-0.02354
ENSG0000	H1FX-AS1	3:1293147	0.888994	0.012561	0.195907	0.535317	0.340946	2.60858	0.966872	0.068845	0	0.082242	0.66235	0.621027
ENSG0000	TRMT10B	9:3751089	10.0168	9.85576	13.2446	13.9736	11.5414	11.7485	4.90866	11.9122	5.77892	11.4933	0.6122	-0.36005
ENSG0000	SNX5	20:179415	98.4071	66.7093	74.3876	64.4922	63.5137	158.526	103.921	68.1263	53.5266	50.6621	0.59725	0.178757
ENSG0000	F5	1:1695141	3.59254	10.8723	0.004216	0.080387	0.131572	2.53744	9.3833	1.54663	2.83646	1.97107	0.2767	1.02351
ENSG0000	UQCRQ	5:1328611	224.593	130.055	311.971	312.699	151.014	352.768	265.239	219.309	214.506	59.2672	0.7292	-0.17651
ENSG0000	GLTSCR1L	6:4274695	3.34785	2.59434	2.89761	1.78151	2.74245	2.52926	1.75953	2.01315	2.13405	5.98549	0.8687	-0.07266
ENSG0000	LA16c-36	16:326374	2.54751	0	3.75879	0	0.46991	2.02494	2.03309	0	0	0.204205	0.1606	#NAME?
ENSG0000	RP11-31F	1:1129118	0.397048	0.106843	0.355302	0.099806	0.084691	0	0.069076	0.474747	0.368307	1.40198	0.5757	0.987574
ENSG0000	BSG	19:567209	381.447	215.874	251.635	172.194	243.98	378.48	355.549	253.508	204.228	153.02	0.9161	0.035864
ENSG0000	UBE2Q2P1	15:845078	2.56613	1.1173	4.02875	5.59358	6.89805	0	10.6234	6.58693	6.57928	5.73707	0.47065	0.690651
ENSG0000	ARMCX5-	X:1025991	8.80865	11.8896	4.16159	4.44566	5.48003	7.33602	2.7238	2.7753	10.6482	2.11126	0.75785	-0.22448
ENSG0000	ATP5E	20:590192	84.1755	55.4667	69.9129	66.3541	19.7432	135.409	109.339	80.4172	66.0974	11.4636	0.1278	0.541744
ENSG0000	TTC27	2:3262803	16.7635	16.2284	7.32644	14.486	7.52777	13.2092	6.87423	8.01836	9.08966	10.5186	0.3916	-0.34553
ENSG0000	PVR	19:445370	1.34199	0.146754	0.470918	0.083937	0.2259	7.26187	2.96841	0.405068	0	0.571273	0.2064	2.10189

ENSG0000	CDH23	10:713969	2.52576	1.3553	1.49658	1.79803	0.875826	3.01289	0.00737	1.6741	3.32458	1.19831	0.74635	0.247719
ENSG0000	IL3RA	X:1336615	0.172217	1.567	0.122431	1.17206	0.285898	12.2164	0.160954	0.167572	0.486121	0.162506	0.42145	1.93684
ENSG0000	RP11-333	4:3982286	1.1822	2.654	0.132273	0	0.136216	3.69944	4.23668	0.888592	0	0.590465	0.44555	1.40108
ENSG0000	SEMA4C	2:9685971	2.36283	2.08121	2.88435	1.12893	3.7176	0.252882	0.529688	0.657577	0.308625	1.81307	0.038	-1.92707
ENSG0000	AC012358	2:5517479	0.518544	0.184116	0.556549	0.86651	0.238677	1.07418	0	0.071965	2.05191	0.22145	0.65405	0.610986
ENSG0000	FCRL2	1:1577457	0	6.15856	0.369577	2.17876	0.356607	0	0	0.783814	0	0.009606	0.15955	-3.30325
ENSG0000	C9orf139	9:1370072	0.4148	2.15317	0.621404	0.561125	0.080249	4.72366	3.68002	0.868076	0	0.924726	0.31575	1.54737
ENSG0000	GTF2F1	19:637956	17.7743	17.7584	21.1291	12.7174	28.3125	12.679	29.6458	21.1318	17.5883	27.8413	0.68945	0.147173
ENSG0000	TMEM184	22:382192	15.2638	15.9156	16.1825	21.7937	27.9243	8.29304	7.90021	15.7205	16.2545	29.9816	0.3538	-0.34645
ENSG0000	YWHAE	17:134427	95.1075	76.8624	28.6283	19.0341	25.7184	103.514	88.756	47.7847	38.8548	29.7051	0.28135	0.368989
ENSG0000	RP11-348	10:110869	1.16112	1.98025	1.3004	1.0116	0.176117	0	1.75834	0.373901	0	2.10406	0.87905	-0.23075
ENSG0000	NBPF3	1:2144012	2.09688	0.80746	3.18193	4.01996	1.79585	0	3.31955	2.934	4.70175	3.34373	0.9246	-0.0679
ENSG0000	PPP1R12A	12:795749	134.603	123.217	52.0815	38.8661	65.5766	163.375	150.98	57.8867	58.9159	122.099	0.25845	0.385516
ENSG0000	E4F1	16:222356	21.7905	27.7655	24.2379	21.2013	24.7249	12.0099	27.9706	17.1613	20.8221	25.715	0.54725	-0.21193
ENSG0000	MROH1	8:1441480	6.14414	6.76294	2.54434	2.50765	2.39529	4.12551	2.73932	3.73348	6.60587	5.28081	0.65815	0.252888
ENSG0000	ZHX2	8:1227813	11.8353	6.90132	8.25936	5.07444	10.3765	2.30647	6.47754	5.96802	4.61246	14.0761	0.377	-0.35724
ENSG0000	BTBD10	11:133880	21.4837	12.9558	10.9457	15.2835	9.83955	20.5368	24.5325	13.6743	1.6248	12.2155	0.91305	0.041442
ENSG0000	EIF4G2	11:107833	396.401	327.127	122.797	179.058	112.895	265.622	180.675	175.292	188.483	172.095	0.7654	-0.11716
ENSG0000	ASRGL1	11:623369	3.83455	7.45093	3.68013	4.51793	3.05697	5.27945	2.01651	1.93664	0.873643	1.7237	0.1823	-1.17328
ENSG0000	IGKV1-27	2:8921342	0	1.81125	0	0.92402	0.236129	0	0	0	0	0	0.00605	#NAME?
ENSG0000	MMEL1	1:2586490	3.38737	6.19821	2.27287	2.35571	1.61873	2.11145	2.80166	1.84887	1.33773	0.75362	0.47695	-0.81045
ENSG0000	HIPK1-AS	1:1139239	3.01739	0.61279	1.53896	4.38166	0.599724	0	0	1.57174	0.389758	1.45262	0.24315	-1.56826
ENSG0000	THG1L	5:1577311	2.95644	2.09395	5.00202	7.56552	3.3178	1.87384	3.52705	2.93967	2.26356	3.9614	0.21185	-0.69807
ENSG0000	KBTBD7	13:411329	3.18521	6.69749	2.52404	1.45499	2.86678	3.23651	1.81815	2.426	6.0364	5.22059	0.8811	0.094103
ENSG0000	NEDD1	12:969072	21.1592	22.1569	7.15017	6.64814	7.07594	15.0797	16.2995	11.3284	10.2574	9.7599	0.8966	-0.0533
ENSG0000	FLNB	3:5800839	8.8187	15.2677	3.43964	5.71109	4.34059	3.17512	3.01509	2.03906	2.92503	4.91008	0.2583	-0.75487
ENSG0000	DEPTOR	8:1198674	1.43615	0	1.12546	0.457468	1.55583	0.276155	0.242542	0.530702	0.036354	0	0.2373	-2.26379
ENSG0000	RRP12	10:973563	1.0921	0.858204	4.51338	4.66833	3.31807	1.44192	0.242659	4.63595	4.08173	4.04409	0.9208	0.069715
ENSG0000	DLAT	11:112024	14.7437	13.0534	8.74395	9.11286	6.51498	8.77669	12.2172	7.73097	0.124991	6.04412	0.47735	-0.65559
ENSG0000	CCDC57	17:821014	29.8416	30.5283	44.6882	46.3328	20.5101	43.9352	33.2539	36.1803	29.8891	9.8342	0.76665	-0.1822
ENSG0000	GNB1L	22:197567	15.8764	16.7107	25.8471	16.6612	27.5504	22.4482	13.751	16.987	25.045	11.0448	0.72955	-0.19742
ENSG0000	PURB	7:4487629	1.63146	1.56928	0.923924	0.435561	1.42629	1.92854	1.03521	1.66584	0.303625	3.3276	0.5555	0.333267
ENSG0000	AGAP6	10:499821	8.39247	9.24212	7.38725	6.72718	4.24134	13.6227	6.41095	7.92227	7.23737	17.6762	0.323	0.50649
ENSG0000	LINC0133	5:7504771	0.101269	2.51103	0.130106	1.33058	0.338617	0	0	0	0.438917	0.221321	0.2778	-2.38191
ENSG0000	ABCB7	X:7505317	6.85212	13.6346	10.181	14.1999	7.35993	11.7896	5.34635	11.6903	9.03683	9.64763	0.8084	-0.10689
ENSG0000	MREG	2:2159428	3.84486	2.48793	8.41905	1.87932	3.92249	6.48719	6.25125	12.3928	7.60904	11.6656	0.208	0.958647
ENSG0000	NRM	6:3068804	5.07237	3.10661	3.78458	4.16804	7.93551	16.989	7.34959	3.54333	3.61759	6.69771	0.4135	0.542041
ENSG0000	TMEM60	7:7779372	37.5667	25.5632	30.4756	22.2299	20.3701	61.5814	41.9863	28.9674	45.0158	15.7935	0.18815	0.51537
ENSG0000	ZNF527	19:373710	3.28871	1.70746	3.22984	1.17614	1.60046	1.18508	1.77162	4.05464	4.39073	2.26255	0.57885	0.362279
ENSG0000	SKAP2	7:2666706	8.68544	14.9163	13.7975	11.518	7.62141	14.8809	15.6623	9.94325	11.7725	13.9548	0.9009	0.052723
ENSG0000	HIST1H4A	6:2602167	3.07677	1.18706	0.197459	0.918626	0	2.89582	0.179841	0.203089	0	0	0.5888	-0.77459
ENSG0000	CATIP	2:2182703	0.039026	0	0.037987	0.490122	0	0	0.016736	0.535263	2.10081	0	0.36955	2.33459
ENSG0000	RALGPS2	1:1787243	2.33307	4.62884	0.655856	0.747408	1.11103	0.022462	0	0.22624	2.71737	0.458432	0.3965	-0.82099
ENSG0000	EIF4EBP3	5:1404018	7.05807	10.547	20.0955	29.4134	12.6582	17.8488	6.73133	9.76377	19.3956	4.76008	0.82535	-0.37542
ENSG0000	DDI2	1:1561749	2.69457	3.27536	1.90235	1.04733	1.74026	4.30761	5.79564	3.67638	2.34327	4.55383	0.11445	0.978336
ENSG0000	KYAT3	1:8893577	10.1104	11.9464	13.0589	9.92402	10.6372	10.8463	17.8866	18.0153	36.7884	8.94963	0.2082	0.703956
ENSG0000	AES	19:305290	481.074	544.905	472.138	349.817	613.507	243.333	209.494	298.859	222.553	217.946	0.00505	-1.01873
ENSG0000	AC010150	2:2573375	0	0.282599	0	0	0	0.680196	0.565693	0.273682	0	0.602856	0.20755	3.13105
ENSG0000	CTC-529I	17:159765	1.36043	2.41282	1.14126	0	0.198155	0.449772	3.41588	0.17039	0.881508	0.722211	0.84065	0.288407
ENSG0000	ZNF341	20:337289	2.43222	4.01576	0.624271	1.3629	1.72721	6.11789	2.05233	1.89686	1.84665	3.04752	0.3866	0.584941
ENSG0000	ABHD4	14:225958	12.05	6.22947	11.7521	10.2946	8.81897	9.06495	2.93421	11.228	5.63614	11.2468	0.2704	-0.42606
ENSG0000	EXO1	1:2418479	3.21456	4.57275	0.591357	0	0.20872	0.375879	0.30378	0	1.59416	1.25617	0.3146	-1.13958
ENSG0000	CCDC84	11:118998	14.1872	14.5111	19.5294	13.6609	9.04031	28.9342	24.8703	13.9684	8.21509	20.3952	0.58375	0.414756
ENSG0000	MRFAP1	4:6640090	87.3821	91.1139	107.331	116.043	126.612	95.9726	118.737	118.062	56.1978	113.129	0.68485	-0.13164
ENSG0000	MAL	2:9502519	11.1279	96.5615	15.7386	12.4475	4.58909	0.333606	2.50271	31.9285	29.2615	4.65507	0.18205	-0.73812
ENSG0000	LOH12CR	12:123554	0.432043	0.773021	1.66086	0.549584	0.157194	2.33798	0	0.654823	0	0.064123	0.85975	-0.33577
ENSG0000	APOBEC3	22:390140	10.2526	3.55551	13.2956	5.8371	22.3017	9.05232	15.8243	17.7171	10.8511	37.0117	0.4986	0.644424
ENSG0000	RP13-104	17:647496	1.1381	3.26126	1.14888	0.983731	0.904893	2.62886	3.43062	0.982149	3.20769	3.3282	0.61255	0.809313
ENSG0000	PHBP19	12:510936	1.56929	2.17208	0.103926	0	0.056188	0	3.1049	0.568028	0	2.11094	0.77235	0.679516
ENSG0000	RNF123	3:4967400	14.9268	16.9671	5.4718	6.60993	6.16408	6.40493	7.28633	6.42898	8.05983	9.31649	0.84175	-0.19026
ENSG0000	IGKV1-6	2:8896626	0	0.408234	0	133.08	0	0	0	0	0	0	0.00005	#NAME?
ENSG0000	FAM185A	7:1027489	5.69666	10.0473	6.3443	9.75472	4.92115	8.0184	5.2861	4.74395	4.85876	4.36289	0.29265	-0.66142
ENSG0000	RP11-395	4:4016667	0.790664	1.25149	0.38036	0.764855	0.479038	0.890281	1.31572	0.134667	1.40391	1.25575	0.7134	0.484725
ENSG0000	LSG1	3:1945840	16.9002	25.6618	19.2572	14.6184	19.3582	22.2937	12.9022	19.3115	20.4634	20.8096	0.9192	0.038318
ENSG0000	UBTD2	5:1722096	1.83027	4.01667	0.918832	0.114418	1.20096	5.00624	4.95019	2.1276	5.77922	2.63423	0.05605	1.41446
ENSG0000	CTD-2184	15:518296	0.357508	0	0	0	0.145587	0	0	0	1.23209	1.62193	0.32945	2.8246
ENSG0000	AIM1L	1:2632185	0.035995	2.23441	0.619736	0	0.801975	0	7.53661	1.86046	0	1.18372	0.1813	1.63191
ENSG0000	CCDC17	1:4562004	0.471203	0.934193	0.165151	0	0.016534	2.06068	0.30885	0	0.903219	0.966346	0.10565	1.73761
ENSG0000	MAZ	16:298061	65.758	66.3724	59.7092	35.9121	56.1552	53.9405	70.9472	63.1193	39.2891	59.8748	0.90575	0.084412
ENSG0000	RP11-876	16:108649	0.325835	0	0.195828	0	0.112814	2.71971	0	0	0	0.242622	0.4002	2.18025
ENSG0000	TMEM182	2:1027369	3.25734	4.21796	3.25363	1.98289	1.78081	1.44163	2.54888	0.906301	2.33645	1.08126	0.54585	-0.58878
ENSG0000	GSTM3	1:1097122	1.07754	3.33573	8.32148	1.31143	1.74601	0.52342	3.14235	6.62582	0	1.3399	0.58795	-0.57516
ENSG0000	SEC23B	20:185074	40.4708	22.7768	47.3439	54.5412	31.7105	24.2103	21.9201	26.9408	31.403	32.9104	0.1079	-0.56516
ENSG0000	TOR1A	9:1298129	69.627	64.9322	75.3222	71.457	64.483	75.7008	47.3818	65.1343	74.7341	61.5078	0.6611	-0.13724
ENSG0000	CBWD1	9:121037-	26.7059	38.6992	27.1745	19.4028	23.0228	40.2815	36.6239	34.3287	23.3309	22.5433	0.61105	0.216559
ENSG0000	RP5-1021	14:738518	0	0.938863	0	0	0	0.718277	0	0	2.32218	0	0.53645	1.97703
ENSG0000	IGHV3-7	14:106062	0	4.62253	0	542.138	0.369695	0	0	0.287811	0.126243	0.228748	0.0577	-9.27809
ENSG0000	RELT	11:733762	6.88637	7.67043	5.71122	6.92924	5.01039	11.198	7.88465	5.82678	9.71793	10.4969	0.28445	0.478622
ENSG0000	ESYT1	12:561181	196.055	197.053	81.6284	109.872	70.2014	73.6121	64.5597	63.9895	95.9358	122.101	0.15265	-0.45134
ENSG0000	FGF7P3	9:3981752	0	0	0	3.21632	0.755492	0.593924	0.26818	0	0	0.628456	0.48625	-1.08123
ENSG0000	RP11-115	12:115953	0	0.219862	0	0	0	0.38017	0.415965	0.177289	1.38289	0.095181	0.20475	3.72804
ENSG0000	CEP152	15:487129	4.64364	7.58347	1.20428	0.667557	1.55181	9.93698	4.58768	1.83168	4.58766	4.91088	0.26155	0.623703
ENSG0000	DAPK3	19:395845	23.0051	15.6288	24.6207	18.6513	32.8485	13.556	11.3715	18.9261	22.9495	26.8037	0.57115	-0.2344
ENSG0000	PRKCA	17:663026	1.98893	3.70487	2.66224	3.14875	0.803495	0.771057	2.99249	1.79667	3.57905	1.87905	0.9735	0.032348
ENSG0000	NBPF25P	1:1455723	0.995628	2.33971	0.831324	0.469518	1.47844	1.40609	1.32771	1.06296	0.720971	2.15195	0.92625	0.162101
ENSG0000	AC116366	5:1324106	1.12976	0.94009	0.887937	0.587952	0.298041	1.20372	0.602066	0.547465	0.633289	1.0807	0.996	0.007808
ENSG0000	CTB-4E7.1	5:1570858	1.82291	2.9929	0.929076	0.485979	0.295603	3.32356	6.96665	2.5684	0	2.92933	0.24115	1.40476
ENSG0000	GLRX5	14:955322	78.1788	70.0244	103.306	78.6509	80.3679	60.4997	83.4105	68.6699	60.9749	23.6558	0.168	-0.51072
ENSG0000	RP11-51F	17:636224	14.1258	12.3312	13.9444	9.53449	18.749	9.87562	6.18181	7.74705	4.5765	12.1681	0.5408	-0.7854
ENSG0000	THAP9-AS	4:8281866	37.3857	28.3052	23.029	20.9913	14.1058	48.2121	38.9584	19.3251	23.6105	13.9541	0.68725	0.208427
ENSG0000	ZC3H7B	22:413015	1.60837	2.55793	1.16059	1.90026	1.27475	1.12902	2.52371	1.82165	1.67352	1.97979	0.8473	0.114546

ENSG0000	RCN2	15:769316	58.5776	27.4695	44.9152	44.4001	37.4218	37.2506	45.6051	38.5281	20.1473	21.681	0.25585	-0.42388
ENSG0000	ZSCAN25	7:9961694	9.2743	9.06828	7.9328	7.29815	6.89638	4.47516	4.27015	2.71888	5.19268	12.2334	0.16825	-0.611
ENSG0000	CLK2	1:1552628	16.5645	16.423	9.96234	6.49633	16.734	15.8355	12.7896	14.8658	10.4527	24.1192	0.6342	0.198187
ENSG0000	EIF3G	19:101062	177.862	167.677	303.639	205.227	346.542	159.837	167.392	190.827	135.513	95.6032	0.0806	-0.71962
ENSG0000	ZFR	5:3235434	35.9718	33.5401	35.8639	23.7403	50.8053	28.4739	38.051	26.8168	20.4865	77.9111	0.6975	-0.1602
ENSG0000	SLC7A6OS	16:682645	18.9317	25.0555	16.3089	16.281	22.768	12.0354	21.8897	19.2593	26.6138	25.2629	0.9709	0.017671
ENSG0000	PLCG1	20:410288	56.0452	43.3496	5.38589	16.1478	7.74018	17.7046	26.6488	7.36043	10.5811	13.1383	0.2532	-0.6379
ENSG0000	C8orf82	8:1445179	19.989	14.9201	3.32849	8.14559	6.66166	32.4509	39.1695	10.479	7.3372	6.09005	0.55045	0.569368
ENSG0000	ORC4	2:1478445	20.4306	23.1407	17.437	10.8908	8.12938	18.8048	11.6832	6.36179	8.36965	5.19877	0.10295	-0.94628
ENSG0000	RP11-478	12:110372	1.98989	0.578004	0.214707	0.551742	0	0	1.27611	0.324044	0.25471	0.636476	0.7709	-0.46323
ENSG0000	MAGI3	1:1133907	0.876155	0.97664	0.101016	0.037022	0.086785	0	0.489047	0.085663	2.02938	0.406619	0.5406	0.868435
ENSG0000	MAPK7	17:193375	5.66292	4.93822	2.21331	1.95759	4.86986	2.36715	2.17486	2.67654	1.05896	3.98442	0.0952	-0.84229
ENSG0000	DLEU2	13:499825	14.9686	7.94992	6.03011	5.23434	2.53201	18.8618	15.6644	10.3414	20.5789	7.36046	0.18095	1.15478
ENSG0000	CTD-2201	5:4301441	6.56212	3.87021	9.55965	10.3617	3.66514	3.24025	2.59051	6.62028	7.60792	3.76245	0.6107	-0.48292
ENSG0000	TUBD1	17:598594	13.0131	7.69863	24.7441	23.5866	15.9977	4.11239	9.81818	18.7916	20.43	9.97092	0.31545	-0.47549
ENSG0000	HCFC1	X:1539475	2.93235	4.78429	4.65997	1.84245	9.56357	3.51714	4.36042	6.26729	2.83185	18.5474	0.2488	0.591476
ENSG0000	ARL11	13:496282	0.253717	0.536277	0.13901	0.245966	0.111756	3.74209	0	0	1.25445	0.041655	0.34785	1.96116
ENSG0000	ZNF792	19:349563	5.34516	5.20415	2.53458	3.4276	2.64591	4.90471	1.71809	2.45704	2.85755	7.25732	0.88605	-0.07178
ENSG0000	SPATA13	13:239796	24.4284	32.3343	20.2005	21.8727	27.6576	13.7233	25.0973	20.5241	17.5764	51.9761	0.80215	-0.14969
ENSG0000	ARHGEF6	X:1366655	28.9684	32.6689	9.79003	8.33757	10.8439	15.167	16.5457	15.6296	9.95381	20.992	0.6954	-0.13342
ENSG0000	C17orf51	17:215247	0.266767	1.2145	0.348107	1.08219	0.930505	0.605412	0	0.529865	0	1.36027	0.62355	-0.72531
ENSG0000	ZNF681	19:237391	1.08293	1.04393	0.673179	0.021487	0.27535	0.908598	0.773301	0.004346	0	0.505818	0.6433	-0.49042
ENSG0000	ZMYND8	20:472092	9.97341	20.6898	9.5737	10.2141	8.25828	11.4118	7.06522	7.88715	10.9967	16.9441	0.43235	-0.31642
ENSG0000	NBPF12	1:1469387	5.38751	9.06253	3.59651	3.66539	2.09503	4.51946	3.52985	3.01505	2.81963	5.78076	0.67445	-0.22897
ENSG0000	NDUFA2	5:1406387	79.0018	51.1393	130.026	139.17	71.6748	128.996	78.62	99.2111	92.7667	21.7143	0.78925	-0.23617
ENSG0000	SIGLEC12	19:514913	0.938658	2.63788	0	0	0	12.3509	0.936676	1.14309	0	0	0.3012	2.04976
ENSG0000	KLHL15	X:2398371	2.04221	2.54041	0.700626	0.312855	0.611348	0.837182	1.78779	1.7964	1.5165	1.0613	0.63265	0.292441
ENSG0000	LPGAT1	1:2117434	7.29601	3.81073	3.80023	2.59114	3.24296	6.56018	3.89988	3.73587	3.18895	6.98692	0.93435	0.047588
ENSG0000	GBF1	10:102245	7.75254	8.79543	2.33211	3.0531	4.35496	5.0173	7.17658	3.21425	2.95874	7.87416	0.77905	-0.11158
ENSG0000	NUDT13	10:731103	0.663265	1.54443	0.609033	0.201857	0.538138	0	0	1.48396	0	0.69286	0.60285	-0.75258
ENSG0000	RN7SL589	4:2269581	0.383995	0.959447	0.426801	0	0	0	2.38611	0	5.23129	1.23323	0.22115	2.68872
ENSG0000	SH3TC1	4:8182071	7.80115	13.9834	0.352101	0.074267	0.68025	18.9314	13.6935	7.86775	10.6732	8.4828	0.09	1.52808
ENSG0000	CCAR2	8:2260463	50.8823	63.7613	15.9868	14.5599	13.8516	42.811	41.6297	20.6594	4.90652	25.0549	0.56455	-0.29001
ENSG0000	NAPSA	19:503119	1.03579	3.36891	2.19437	0	2.82119	2.46117	2.18243	0.842684	0	4.6426	0.93405	-0.09273
ENSG0000	CCDC94	19:424633	28.9848	32.4102	28.4993	30.2385	34.3585	37.8751	29.1184	31.0142	16.4266	21.7702	0.62845	-0.2042
ENSG0000	VLDLR	9:2422701	0.399516	0	0.143844	0.735556	0.289561	1.43705	0	1.81985	0	0.988002	0.39125	1.16794
ENSG0000	FTH1P8	X:1480522	3.04603	2.99082	7.65313	15.4622	4.45113	10.6162	1.87554	8.54032	23.5915	0.925555	0.54815	0.434679
ENSG0000	RHBDD2	7:7584260	53.2302	68.2159	68.7817	83.6726	66.5093	72.3369	111.731	73.4357	173.409	71.8293	0.1089	0.536147
ENSG0000	BHLHE40-	3:4896808	1.17332	0	0.806176	1.13351	1.31903	5.05509	1.75376	8.15969	2.35648	1.17472	0.10315	1.94145
ENSG0000	CCL5	17:358689	1238.6	421.633	4261.27	1880.63	3131.64	2001.98	4425.83	3875.81	2193.86	1749.68	0.631	0.340329
ENSG0000	IKZF3	17:397577	32.7548	15.9338	28.6159	27.4417	46.9097	32.5981	26.4103	31.5088	26.1575	71.8222	0.93355	0.055173
ENSG0000	TCF25	16:898735	97.8131	106.88	122.041	115.775	166.158	119.385	141.925	96.2949	101.937	124.986	0.8386	-0.06434
ENSG0000	MTIF2	2:5523659	10.4328	17.322	9.43368	11.53	14.0634	8.82988	5.81856	4.83309	5.39929	11.6939	0.061	-0.81661
ENSG0000	KB-226F1.	22:239669	0.594864	0.258879	0.05345	1.20877	0	0	0	0.524782	0	0	0.44765	-1.89566
ENSG0000	FAM13A-/	4:8870978	0.713517	3.48363	0.218091	1.24725	0.423433	0.667413	1.4621	0.310623	1.30758	2.97441	0.9788	0.031933
ENSG0000	PSAT1	9:7829714	8.14475	6.00708	3.09089	1.74286	2.7311	17.2091	22.5933	15.7255	8.39472	5.24712	0.0019	1.79446
ENSG0000	MPDU1	17:756187	37.7923	26.7205	21.6658	29.1322	25.7879	61.2774	54.1085	39.9683	15.4397	21.5123	0.7084	0.363645
ENSG0000	RP11-305	9:9110495	1.10957	0.211704	0.126918	0.736955	0.830232	7.78764	7.31879	1.17232	5.44894	0.73412	0.1054	2.89511
ENSG0000	ATP5SL	19:414313	37.7696	29.3686	63.5957	55.2695	52.7428	30.4446	36.1722	32.9967	44.5256	27.7268	0.14205	-0.53269
ENSG0000	RP11-850.	18:542683	0.451128	0	0.357779	0.317311	0.395494	0	0.029565	1.41928	0	2.31709	0.5432	1.32684
ENSG0000	KCNN4	19:437665	5.35614	20.2235	1.2559	3.08729	0.553095	35.7187	20.4665	7.69313	11.0251	6.18331	0.0088	1.47056
ENSG0000	ELL3	15:437269	1.03886	1.60488	1.25495	0.936292	0.981843	0	1.24176	0	1.9692	1.42427	0.6283	-0.45804
ENSG0000	SMIM19	8:4241647	60.1643	40.7851	51.3393	59.0398	22.4915	101.121	67.5619	45.3123	81.4067	10.5133	0.5164	0.347498
ENSG0000	PSMB4	1:1513995	42.7685	29.5433	175.629	189.872	126.094	51.7238	65.117	189.568	153.291	68.3529	0.72055	-0.13813
ENSG0000	RABGGTA	14:242655	26.9446	21.5971	23.4585	31.6574	21.5117	49.8638	23.9594	21.7862	6.51511	28.4648	0.99195	-0.00355
ENSG0000	UMAD1	7:7636517	23.1619	34.3972	18.2739	24.3765	15.8186	34.6099	18.7529	17.1854	20.1841	15.6156	0.7583	-0.18865
ENSG0000	NPIPB15	16:743778	3.32723	8.99088	2.18641	3.91675	1.69911	5.00702	5.3556	3.18228	4.28448	3.14173	0.877	0.131382
ENSG0000	CNTRL	9:1210748	25.6652	52.5905	19.9723	13.7088	17.1198	37.8493	37.5065	18.3348	30.7117	30.5048	0.6283	0.203809
ENSG0000	TRBV7-6	7:1424921	3.35276	9.78144	7.3554	1.09344	1.03824	3.28442	101.312	3.72588	0	0.55712	0.2774	2.13638
ENSG0000	SERAC1	6:1581095	1.39196	2.1801	0.632929	1.10217	1.35061	0.231937	2.8662	0.426307	1.87599	1.91479	0.79685	0.168249
ENSG0000	RP11-19P.	17:274567	1.14102	0.666295	0	0.011752	1.08477	2.3192	2.86655	0.774759	0.020344	1.51162	0.3436	1.66521
ENSG0000	NUS1	6:1176755	9.05506	8.72421	4.18784	5.12856	5.47273	3.95556	4.42214	8.75912	6.55131	6.16875	0.72725	-0.15592
ENSG0000	MRM3	17:757096	20.7648	18.4721	31.8154	32.8811	30.0815	17.5495	13.0412	13.5877	7.40287	14.374	0.2176	-1.12715
ENSG0000	DPH3	3:1625797	19.6712	12.1332	13.5871	13.6888	10.4131	28.8957	20.6118	18.4595	8.06592	9.25	0.6063	0.226541
ENSG0000	CDC5L	6:4438752	12.2515	15.4734	9.83604	11.9847	16.6609	11.3118	15.0333	11.8169	12.2118	14.5124	0.8635	-0.05706
ENSG0000	CENPBD1	16:899697	4.37659	4.55703	6.22964	1.42173	5.31888	1.01005	1.21113	9.73901	5.2962	6.47977	0.75795	0.185246
ENSG0000	TBX21	17:477332	64.0237	29.1312	58.1159	64.9022	101.604	32.2165	55.5282	45.9691	21.082	51.6182	0.0285	-0.74248
ENSG0000	ZDHHC20	13:213725	24.1699	25.0051	3.85965	1.78347	4.55606	20.9467	10.3498	10.505	5.89128	11.9426	0.9271	0.039519
ENSG0000	2-Mar	19:841326	34.2446	26.3044	48.3418	58.8408	45.0577	36.6652	32.5091	32.2789	81.9492	23.9636	0.9329	0.031533
ENSG0000	OAS2	12:112906	38.7783	47.8298	24.9895	27.2725	28.6731	23.5033	82.8826	46.3838	22.8456	47.7474	0.5524	0.330592
ENSG0000	DDX43	6:7336855	1.9554	0.66705	2.10703	3.50197	2.79911	1.69609	2.74825	1.52205	2.227	1.33999	0.93455	-0.12704
ENSG0000	HAR1B	20:630954	2.20293	0.782496	4.53908	4.2829	3.12291	0	0	0	2.9058	1.29691	0.4423	-1.72623
ENSG0000	ARHGEF11	1:1569348	3.82068	19.3177	1.63501	2.71376	0.989199	1.65663	0.438388	0.48652	3.11755	1.60529	0.0302	-1.71235
ENSG0000	PRELID2	5:1454717	0.023795	0	0	0.4353	0.067756	2.29757	4.11981	0.686224	0	0	0.06735	2.79924
ENSG0000	CD164	6:1093665	236.947	193.564	106.364	155.744	79.6187	183.38	192.271	148.847	191.262	128.809	0.5911	0.193768
ENSG0000	FBF1	17:758970	12.6668	11.0017	5.85051	5.93857	5.41819	9.98596	4.58971	11.6228	4.30225	6.3273	0.9501	-0.05984
ENSG0000	JARID2	6:1524629	7.95809	10.0175	5.16697	3.03175	6.46712	6.02413	9.99802	6.40992	2.81416	13.0692	0.701	0.158419
ENSG0000	SP110	2:2301213	76.965	154.068	104.954	89.2713	122.569	114.282	201.551	112.44	74.9736	156.736	0.4492	0.28258
ENSG0000	RP11-326	18:320183	2.71057	6.61064	3.16343	8.43432	1.16908	0	4.29887	0.574472	2.72923	1.48735	0.4452	-1.02819
ENSG0000	PAXBP1-A	21:327281	0.28787	1.36878	0.549659	0.183657	0.137477	0.647896	0.035051	0	2.23583	0.718057	0.6591	0.617865
ENSG0000	AC093642	2:2418813	0.107285	2.61147	0	0	0.068121	0	2.61094	0.115179	0	0.039642	0.93335	0.143113
ENSG0000	TOP2A	17:403885	7.6715	20.9385	0.169419	0.170478	0.864706	13.9566	12.7314	3.15212	6.21346	6.8467	0.1652	0.865879
ENSG0000	TMEM168	7:1127623	7.8333	5.13647	3.81258	2.69345	1.40799	6.49468	2.81228	6.98465	1.57444	4.78215	0.99375	-0.00334
ENSG0000	DYNLRB1	20:345164	119.955	140.751	127.891	124.297	118.034	192.382	163.916	161.638	125.468	35.8568	0.8964	0.04534
ENSG0000	EHMT2	6:3187975	33.2355	33.1888	14.6711	9.23149	22.1399	15.5958	24.549	13.2406	1.35386	25.6245	0.42615	-0.33512
ENSG0000	EFCAB11	14:891248	0.849452	3.77755	1.07806	1.43728	1.22998	4.69115	5.67684	2.45311	0	1.96664	0.35355	0.852797
ENSG0000	GDI2P2	1:7108132	0.183684	0.239914	0.332415	0.762183	0.072126	0.15725	0.072092	0.455603	0.093612	0.081671	0.6477	-0.82023
ENSG0000	CA13	8:8522058	1.68757	3.39287	0.962768	1.68361	1.039	0	2.12583	1.55013	0	0.699175	0.4111	-0.94155
ENSG0000	GOLPH3	5:3212470	94.4949	76.4803	64.5995	69.722	47.9488	83.5955	72.2119	68.7355	27.0373	56.3369	0.4216	-0.24317
ENSG0000	AC131056	17:361832	12.2228	6.0819	6.06812	8.81981	0.935348	6.27151	23.859	8.18359	1.84456	0.547958	0.88935	0.181444
ENSG0000	POU2AF1	11:111352	1.69371	2.46771	0.288007	2.06079	0.315194	1.18082	1.18399	0.203163	2.7639	0.643577	0.9996	0.00027

ENSG0000(	DPEP2	16:679873	16.5664	31.5956	35.87	33.7768	17.1681	25.6231	5.64277	8.11881	8.20105	10.4722	0.17085	-1.28032
ENSG0000(	NDUFV1	11:676068	135.605	151.553	182.655	228.599	193.041	123.528	173.509	153.436	203.003	160.808	0.72825	-0.12702
ENSG0000(	ZGRF1	4:1125135	2.05064	1.45561	0.735583	2.33878	0.719208	5.14297	2.78779	0.546746	1.74126	3.12747	0.18055	1.11808
ENSG0000(	SNX10	7:2629189	39.0102	18.869	12.7849	15.8393	18.6814	80.258	28.6565	26.7617	27.0671	22.0555	0.0302	0.761498
ENSG0000(	HSF4	16:671541	1.07292	0.838195	0.985192	1.18846	0.555061	0.638979	2.18213	0.878555	0.560406	0.810742	0.8074	-0.161
ENSG0000(	PCNT	21:463241	13.5101	26.4441	18.3095	24.5471	14.2685	9.69051	16.3389	5.53308	3.31214	24.6836	0.1122	-0.87825
ENSG0000(	HN1L	16:161232	9.92612	7.90523	7.56711	11.7451	9.61507	4.78417	7.06713	12.6063	9.70418	14.6574	0.90655	-0.05866
ENSG0000(	SNRPA	19:406915	53.1668	62.3235	43.0973	31.2103	38.4615	69.2711	65.4965	49.2622	47.0781	33.9915	0.6182	0.21162
ENSG0000(	SLC20A2	8:4241647	9.44422	12.4578	6.00382	6.10593	4.68151	6.28323	12.5208	8.49832	5.82518	10.643	0.7457	0.233554
ENSG0000(	WDR19	4:3918240	3.05528	3.4837	2.44263	2.57219	0.643899	2.4721	1.35773	1.22454	5.65533	2.58911	0.6333	0.417686
ENSG0000(	ACSS1	20:250062	47.1037	40.5128	35.9187	39.4118	25.5997	20.3252	22.9901	22.042	20.0693	31.6139	0.0187	-0.74972
ENSG0000(	LAX1	1:2037651	32.5182	18.5456	22.6204	24.4829	17.6137	11.5088	25.2397	21.6419	33.3925	32.6442	0.8191	0.084554
ENSG0000(	RP4-576H	20:151675	0.11985	0.61876	0	0	0	0.567071	0	1.00761	0	0.24502	0.41245	1.4361
ENSG0000(	GRB2	17:753180	66.7123	100.058	57.4697	37.9536	58.9115	76.3587	57.7131	56.2206	45.0741	84.1826	0.87105	-0.06422
ENSG0000(	CD3EAP	19:453796	4.0336	7.0138	15.7404	7.00681	13.4032	0.721587	5.7323	6.47199	7.54982	8.02211	0.48525	-0.71156
ENSG0000(	TPGS2	18:362977	43.1789	39.9682	39.3412	31.6766	42.6272	57.8198	49.5148	42.7013	21.0353	27.7391	0.7043	-0.15638
ENSG0000(	RBM12B-/	8:9321330	1.84703	1.57287	0.455583	0.585775	0.600316	0	4.94022	0.89215	1.93734	0.721296	0.6153	0.78227
ENSG0000(	DNAJB4	1:7797917	7.86661	7.02497	3.78453	4.48132	3.52198	4.24014	6.40506	4.40314	7.11354	4.68207	0.54345	-0.31121
ENSG0000(	RP11-958	16:149115	2.47268	2.27678	0.514227	0.381993	3.83391	0.677049	1.37896	1.77062	0.054303	6.01004	0.92035	-0.18806
ENSG0000(	RP11-156	17:469231	0.783621	0.002236	4.21406	2.62747	1.45272	0	0	0.635091	0	0	0.26715	-3.7473
ENSG0000(	PRR3	6:3054137	5.29278	7.99781	4.92546	3.7191	5.66865	3.87017	6.42945	5.18286	8.07036	6.45011	0.87045	0.110558
ENSG0000(	NOTCH4	6:3219076	2.18409	0.158696	1.15095	2.28718	1.34123	0.190465	0	1.90721	0.034147	0.587846	0.41805	-1.35443
ENSG0000(	EIF1AD	11:659965	8.63433	7.41137	22.2693	18.9644	21.1584	18.8513	14.4279	28.2155	8.92927	20.7226	0.9437	0.040751
ENSG0000(	ZNF516	18:763581	0.834197	0.798432	0.393088	0.604358	0.262763	0.862634	0	0.499716	1.52552	0.307882	0.6554	0.565273
ENSG0000(	RP11-521	10:503055	0.976163	0.675501	0.65193	0.948714	0.252246	0.217198	2.2252	0.991254	4.59268	1.37767	0.35425	1.5135
ENSG0000(	DNAJB5	9:3498964	0.491333	0.729205	0.066539	0.587061	0.463196	1.2145	1.00553	0.622054	2.78647	0.368598	0.1038	1.52351
ENSG0000(	RP11-251	7:1055302	0.571704	0.967909	0.81204	0.488881	0.186051	0	0.086898	0	0.631448	1.14491	0.8778	-0.24273
ENSG0000(	RP4-666F	1:1150996	0.645061	0.560673	1.13284	1.29027	0	0	0.652757	0.499557	0	0.116515	0.4428	-1.29242
ENSG0000(	ADAT1	16:755285	37.8781	18.2321	21.8158	18.6729	16.8366	20.5755	22.1122	10.5202	4.22137	26.7842	0.60345	-0.4238
ENSG0000(	ORC6	16:466896	3.44016	4.7521	0.795129	1.85988	1.07603	6.67979	4.24523	4.22066	6.88083	2.26115	0.0715	1.08916
ENSG0000(	PSMD11	17:324239	30.8611	37.1322	60.9629	37.7789	75.677	49.0821	62.2848	56.4994	43.426	69.2401	0.7876	-0.1017
ENSG0000(	MELTF	3:1969886	0.984916	0	0.706756	0	1.14049	4.1352	0.340225	0.405298	2.95346	0.960167	0.1995	1.7565
ENSG0000(	EIF2B2	14:750029	11.9094	15.3052	36.3291	19.6984	24.0796	16.5401	21.9013	33.1261	30.5541	25.2836	0.85	0.104096
ENSG0000(	MYL12B	18:326190	527.995	398.654	632.347	587.939	454.747	816.801	766.591	745.195	703.426	174.846	0.44305	0.278276
ENSG0000(	ME1	6:8321038	11.4387	0.719139	9.15706	8.84772	7.07381	3.09018	0	2.59315	3.16491	7.77454	0.12605	-1.36318
ENSG0000(	PRDX5	11:643180	168.367	86.3461	246.788	238.504	203.74	231.726	255.51	265.592	221.899	94.416	0.69875	0.123758
ENSG0000(	CDK5	7:1510538	9.63288	12.7587	14.2858	16.4399	13.9411	16.5955	13.0352	14.5188	0	3.08582	0.5226	-0.55833
ENSG0000(	SEL1L	14:814715	12.2709	8.3592	3.97175	4.68388	4.24545	7.73773	8.73823	6.20425	3.52021	9.76075	0.9693	-0.0171
ENSG0000(	CYBB	X:3734927	3.10674	13.6079	0.264807	0.552272	0.251413	34.0101	0.085816	0.609771	0.019737	0.289657	0.68445	0.948917
ENSG0000(	HNRNPA1	12:542309	317.332	423.265	487.409	376.506	613.914	355.702	406.663	451.688	311.701	459.333	0.67185	-0.19555
ENSG0000(	CCHCR1	6:3114243	8.80257	17.8207	7.99611	9.62338	6.90196	6.96546	11.4162	6.51092	2.44552	14.0432	0.5144	-0.29811
ENSG0000(	GRK2	11:672664	176.003	207.662	15.8566	10.0233	27.9952	151.525	159.109	29.6157	16.4933	58.6568	0.7703	-0.11076
ENSG0000(	CSF2RA	X:1268799	3.01783	7.1745	0	0.100556	0.211642	19.3752	1.41837	0.662357	1.21745	0.053872	0.24535	1.09723
ENSG0000(	IFIH1	2:1622670	14.0217	12.5085	7.40095	10.473	10.2642	19.3126	30.4651	18.68	5.97784	20.0811	0.07255	0.759892
ENSG0000(	C12orf49	12:116710	8.73068	7.36629	17.3654	13.8874	12.4316	11.5318	15.1638	11.7991	13.9932	8.12916	0.73485	-0.20025
ENSG0000(	HYAL3	3:5027902	2.3872	1.17941	1.96599	4.93521	2.79372	10.1443	1.43041	2.39815	0.120869	2.22314	0.86585	0.165813
ENSG0000(	SHFM1	7:9648162	66.6161	64.1149	79.0214	83.1882	38.9245	120.143	123.294	101.154	101.305	20.6072	0.4822	0.324612
ENSG0000(	STT3A	11:125569	40.4967	29.1111	48.8303	36.3247	31.7179	26.2115	27.1568	40.608	39.2507	41.437	0.6576	-0.21781
ENSG0000(	CKLF-CM	16:665525	3.53801	1.54173	2.60398	4.92207	3.6416	9.83795	10.558	6.38452	9.67323	2.84184	0.21095	1.01503
ENSG0000(	SLC19A1	21:454051	6.1386	11.3474	3.14846	3.8322	3.26055	6.0678	7.9573	2.60767	0.994878	5.15113	0.3168	-0.66797
ENSG0000(	TGIF1	18:341160	23.1122	15.5128	80.5201	35.5153	36.1441	14.8008	19.3581	53.9039	26.6691	18.996	0.134	-0.61708
ENSG0000(	RN7SL559	1:2797034	0.742678	0.345067	0	0	0	0.312673	0	0.287705	0.954055	0.178318	0.53415	1.27606
ENSG0000(	RAB40B	17:826549	2.15247	3.10032	4.07481	2.39758	3.31955	1.6773	2.39908	3.23635	2.26153	2.04063	0.2995	-0.66454
ENSG0000(	RP1-197B	12:476612	1.08646	0.382589	0	1.86713	0.248704	2.47494	0.414221	0.599651	0	0.26424	0.9032	0.210797
ENSG0000(	RP11-413	13:423391	0	0	0	0	0	0	1.66081	0.69828	0	0.400362	0.0126	inf
ENSG0000(	CDKN1B	12:127150	7.716	5.47573	21.3105	41.4368	19.7179	7.97815	13.9785	25.5098	28.3075	19.6444	0.9804	0.011149
ENSG0000(	CTB-50E1	19:423971	1.56785	3.50404	1.81338	1.88061	1.54895	1.99981	0.795535	1.48017	4.46709	0.262905	0.9714	-0.05804
ENSG0000(	PLOD1	1:1193420	5.26546	6.4605	2.07549	4.51591	7.04295	8.36157	2.86408	5.97666	3.97699	11.8833	0.359	0.419939
ENSG0000(	CAMK2G	10:738125	4.16893	8.88063	18.6289	15.0975	24.765	3.02479	8.23304	11.5007	8.56062	31.8329	0.51405	-0.33737
ENSG0000(	AHCYL1	1:1099846	19.3207	13.0996	6.98163	2.95172	15.3718	18.9594	11.5941	11.0076	6.32182	22.66	0.777	0.113874
ENSG0000(	NUBP1	16:107437	21.1422	25.2116	37.9175	28.1034	33.5792	42.0649	36.3796	40.3787	27.7881	29.3836	0.62375	0.184926
ENSG0000(	LMAN2	5:1773315	159.929	125.507	287.704	264.251	308.12	157.373	218.578	235.914	237.818	190.853	0.50765	-0.22179
ENSG0000(	RP11-100	15:325198	3.93245	5.2102	4.04159	10.7728	2.45201	6.651	0.996621	0.883024	4.44214	2.88291	0.6667	-0.67074
ENSG0000(	TMEM209	7:1301647	14.73	15.3203	10.1992	12.8147	7.67361	7.38272	8.14013	8.91722	2.33632	14.1104	0.03665	-0.7568
ENSG0000(	GPX7	1:5260237	32.8794	21.8563	28.0957	34.5412	33.3089	25.8589	20.3578	15.3832	25.5096	12.2423	0.1135	-0.63603
ENSG0000(	DDX17	22:384680	28.6652	43.3943	37.4327	23.8022	50.9506	48.548	54.9253	61.741	41.1054	135.903	0.23135	0.455492
ENSG0000(	RP11-18B	9:1056945	0	0	0	0	0	0	0	0	2.65909	0.073429	0.1519	inf
ENSG0000(	TMEM244	6:1298312	0.688555	0.682918	0	0	0	0	3.82577	0.069529	0	0.959061	0.33015	2.31413
ENSG0000(	SYS1	20:453619	17.5901	12.0539	25.5482	17.9199	23.5476	22.5225	26.5008	25.062	30.6497	25.9104	0.1298	0.55344
ENSG0000(	MAN1B1	9:1370869	30.5401	29.5279	33.7995	32.4029	23.2628	24.4436	21.9918	24.4436	26.8735	31.8776	0.55505	-0.18846
ENSG0000(	SERINC4	15:437269	1.07463	0.836171	1.53983	0.80451	1.20972	0.997242	1.20357	1.82019	0	2.80702	0.8481	0.201267
ENSG0000(	PKMYT1	16:296394	6.0774	14.2305	0.161334	0.070662	0.514106	12.8661	19.1567	7.26032	3.05418	7.21567	0.1081	1.23779
ENSG0000(	LINC0094	12:126726	2.2966	0	0.516997	1.34302	3.06678	0.471307	0.37709	1.62663	0	1.37068	0.44715	-0.9111
ENSG0000(	PPT1	1:4007270	95.8088	109.392	77.5065	74.5253	62.2279	228.991	87.0717	79.0517	112.654	82.496	0.11575	0.486887
ENSG0000(	SLC35B2	6:4425409	26.4719	21.7197	36.4025	29.212	35.3446	23.1959	27.8642	22.6644	38.7962	32.4684	0.90365	-0.04513
ENSG0000(	GRB10	7:5059006	0.119671	2.17952	0.169969	0.5341	0.08621	0.137825	0	0.554913	1.44938	0.349726	0.58135	-0.40235
ENSG0000(	LUZP1	1:2301944	32.4847	32.6239	33.7253	40.4021	89.7885	41.9878	56.7667	52.258	75.5202	132.282	0.33045	0.622602
ENSG0000(	ZNF597	16:336509	1.6608	0.942799	0.48608	0.891841	0.835089	2.40056	0.723302	1.12323	0.169959	0.995486	0.9396	0.10799
ENSG0000(	C1orf122	1:3780294	36.1957	21.1562	39.2036	47.2502	38.6642	66.8006	49.4118	39.9698	38.9446	12.7891	0.945	0.046613
ENSG0000(	ORMDL2	12:557432	45.5888	33.8228	86.3255	67.9738	49.3179	110.419	86.7306	100.3	80.773	37.0161	0.597	0.450768
ENSG0000(	MGAT4A	2:9861910	74.1671	95.1358	127.198	163.37	59.3029	59.4372	25.9716	82.1125	115.262	55.4843	0.27925	-0.67906
ENSG0000(	BUB3	10:123154	108.974	127.822	124.993	111.163	93.7538	129.689	176.663	115.562	146.249	83.5048	0.793	0.097701
ENSG0000(	SH3RF1	4:1690942	0	0	0.225898	0.27933	0	0	0	0	1.95526	0	0.55005	1.68278
ENSG0000(	UBTF	17:441918	29.907	35.5889	19.6402	13.5996	33.9936	24.1712	27.9466	17.4573	11.7494	48.2997	0.6435	-0.20093
ENSG0000(	GOLGA6L	15:823551	0.98785	0.586506	0.444668	0.587884	0.864624	0.051521	0.400581	0.190514	1.4476	1.62574	0.99795	-0.00322
ENSG0000(	IGLV4-69	22:219910	0	2.3584	0	4.44325	0.097306	0	0	0	0	0	0.02045	#NAME?
ENSG0000(	GPAT2	2:9601055	1.80587	0.04347	2.5743	0.216594	1.61858	0.041415	1.304	3.07243	0.785896	1.35502	0.92985	-0.08331
ENSG0000(	IGHA1	14:105707	0.526084	454.649	0.98277	259.399	20.3662	0	1.06893	3.05886	11.7775	6.41585	0.00005	-4.74671
ENSG0000(	PLA2G7	6:4665291	0.692989	4.11974	0.139634	0	0.091613	60.4737	0.865815	0.108682	0.283591	0.058751	0.03735	3.62507
ENSG0000(	FAM189B	1:1552472	1.03096	1.22862	0.756277	1.62772	3.39854	0	0.284047	2.05806	0.158254	3.15701	0.70655	-0.40634
ENSG0000(	MYH10	17:841313	0.449885	0.99628	0.533388	0.388344	0.168018	0.270406	0.066771	0.549129	0	0.020613	0.21195	-1.37009

ENSG0000	GHRLOS	3:1024802	3.01034	2.26796	1.914	0.52726	1.71711	2.22055	0.277835	0.727135	1.97946	9.04266	0.6313	0.574022
ENSG0000	PNPLA2	11:818901	17.4094	27.9905	33.8245	25.9256	44.2309	10.7568	31.8517	34.3935	22.1057	45.1726	0.92305	-0.0774
ENSG0000	EP400	12:131949	6.59546	11.5888	2.84486	3.66559	5.87093	7.70602	7.12819	4.28643	1.42331	13.326	0.81635	0.112365
ENSG0000	DLGAP3	1:3486543	0.165926	0.287836	0.186783	0.395852	0.387965	0.311969	0.490396	0.798749	0.131696	0.324339	0.88595	0.283177
ENSG0000	BBX	3:1075229	79.8514	84.4116	124.367	68.5364	140.891	38.4067	107.186	62.1294	49.8213	209.75	0.434	-0.27826
ENSG0000	AC144449	2:1495696	1.49318	0.257183	0.177556	0.372736	0.101671	0.283195	0	0	0.858567	0.997232	0.93185	0.160086
ENSG0000	USP53	4:1191927	3.21552	3.31039	1.21465	1.77958	1.13309	1.31329	1.8301	1.41912	0.732846	1.39373	0.30255	-0.63185
ENSG0000	NR2C2AP	19:191455	19.9634	17.1883	44.2904	31.308	26.0905	37.4421	29.8708	36.4967	27.4368	20.8132	0.87235	0.086079
ENSG0000	DONSON	21:335039	9.70717	9.3015	4.43584	2.93526	2.85218	17.7389	9.48056	6.19451	6.93717	7.21928	0.18525	0.841498
ENSG0000	NANS	9:9805673	30.0625	29.5949	43.4878	42.1698	51.8609	48.51	38.1821	34.8074	50.4547	17.5263	0.8784	-0.10804
ENSG0000	CTD-2286	18:790692	0.347913	0	0.263961	0.986905	0.405391	0.999415	0	0	0.073436	0.936246	0.98425	-0.00256
ENSG0000	SLC37A1	21:424724	7.48723	3.64181	10.4147	13.3	8.7407	2.45133	5.38503	10.6897	6.38169	17.1256	0.82335	-0.11296
ENSG0000	RP11-530	12:795749	0.597241	1.03665	0.058478	0	0.189146	1.7834	0.067524	0.780083	0	1.18661	0.47235	1.15639
ENSG0000	DPYD	1:9707774	25.9732	35.2891	9.64287	13.1828	6.53189	10.13	8.30747	12.2299	10.9641	9.64293	0.0526	-0.76638
ENSG0000	RP11-51J	8:3009539	31.2818	18.7166	24.4346	45.2735	13.9213	6.25013	8.20956	9.81484	13.1182	7.50061	0.27145	-1.3993
ENSG0000	KIR2DL4	19:548035	0	0	0.719385	12.7343	0	5.91006	66.6309	4.60042	0	0.717247	0.22765	2.26598
ENSG0000	RNF130	5:1799116	15.4178	40.6598	17.8277	14.9325	19.281	132.895	7.97078	8.4243	20.097	6.95515	0.24715	0.560642
ENSG0000	IFFO1	12:653837	38.273	47.947	17.1277	21.4659	15.7732	26.0685	32.1319	20.8395	15.7865	30.635	0.84105	-0.08247
ENSG0000	RP11-817	12:321041	0.275055	0.724235	0	0.831991	0.340593	0.893623	0	0.836631	0	0.665217	0.94635	0.138979
ENSG0000	HNRNPA3	2:1769905	30.7385	28.7434	14.7166	17.7897	28.8215	42.1449	46.0311	26.034	32.1061	46.3431	0.29305	0.454542
ENSG0000	DNAJB2	2:2192792	3.5096	3.33786	2.39108	1.72147	2.89665	4.8044	2.90144	2.25265	3.34282	4.4145	0.6721	0.253742
ENSG0000	HMGXB3	5:1499607	9.95697	12.8567	3.91634	6.85146	4.30343	8.35379	5.7586	5.23917	0.815746	6.79495	0.37795	-0.45225
ENSG0000	TMEM230	20:506823	130.674	106.273	179.398	174.529	120.634	121.071	111.107	100.582	171.431	65.6184	0.3282	-0.34026
ENSG0000	MAP3K4	6:1609903	11.11	9.56957	3.97713	3.02846	4.18994	5.65997	7.05058	5.64464	11.1214	7.56495	0.2963	0.546802
ENSG0000	PM20D2	6:8914604	5.20984	3.85507	2.11617	1.52737	2.5972	1.67441	3.40351	2.12869	2.49423	4.37547	0.77345	-0.14203
ENSG0000	AKR7A2	1:1930396	48.2163	42.9621	81.8753	55.2174	97.1644	39.1254	48.7048	46.01	37.323	29.1424	0.0235	-0.76226
ENSG0000	RP11-809	8:1411172	0	1.0142	0.536374	1.59043	0.434909	0	0.212953	0.817893	6.54596	0.826862	0.3722	1.60352
ENSG0000	PLK1	16:236776	7.57626	21.1447	0.746379	0.696001	0.440572	11.0938	4.99885	2.86944	15.5082	4.65072	0.34325	0.550181
ENSG0000	LSS	21:461850	5.12296	8.41542	3.44639	2.29168	3.134	8.38191	9.07411	5.43921	4.09726	8.61114	0.24465	0.500567
ENSG0000	MTHFD1	14:643880	34.0608	29.2354	23.0683	18.2746	17.2825	39.8117	35.9392	13.3375	16.4845	22.5833	0.9698	0.024072
ENSG0000	NEK2	1:2116586	4.51234	7.89465	0.228189	0.423733	0.878044	13.4314	7.6625	1.22683	0	1.08213	0.2759	0.87097
ENSG0000	MT-RNR1	MT:647-1	9.15528	10.5091	475.289	1211.64	779.153	16.9786	13.7987	1145.43	1636.54	265.571	0.62475	0.360631
ENSG0000	RP1-39G2	1:4025642	10.0199	9.25285	6.218	6.24202	3.45296	18.2468	21.252	9.01779	7.19947	5.14473	0.1396	0.853253
ENSG0000	HIST1H4E	6:2620455	10.4973	0.995104	5.59968	3.78916	2.03725	7.06254	2.06535	2.39992	3.47009	0.594188	0.4956	-0.5493
ENSG0000	SAP30BP	17:756268	75.3503	85.0361	96.6723	99.9377	93.6738	88.9456	133.369	83.9752	96.3575	70.3287	0.96165	0.025302
ENSG0000	IGHMBP2	11:689038	9.7202	10.2833	8.0973	11.7297	13.5824	7.4364	7.49212	9.71549	15.958	18.023	0.70225	0.149966
ENSG0000	BBS7	4:1218244	6.53216	6.16604	3.36511	4.67343	1.99877	3.25097	6.89879	11.1521	9.4022	3.93992	0.25405	0.720009
ENSG0000	SELENOS	15:101270	119.343	67.0987	101.26	115.896	84.0076	151.432	195.171	101.966	139.084	39.9786	0.31505	0.34965
ENSG0000	IFI6	1:2766606	50.9875	42.8258	57.1113	57.325	78.5493	90.5042	793.562	288.637	41.5474	38.6307	0.001	1.94489
ENSG0000	RP11-340	12:121190	0.45005	0	0	0	0	0	0	0	3.65742	0.236861	0.065	3.63783
ENSG0000	RNF6	13:260947	41.8346	34.7661	12.8723	22.3946	9.28378	18.9723	17.0542	18.2579	19.666	15.4209	0.37555	-0.29215
ENSG0000	PTRH2	17:596185	17.4688	13.9952	23.1083	15.3707	18.7595	23.3966	22.0431	15.345	8.52467	6.35375	0.83105	-0.29288
ENSG0000	SMAD2	18:478089	49.513	41.9744	30.416	33.1104	26.5748	30.0264	31.9394	29.064	3.99092	24.3727	0.17125	-0.67529
ENSG0000	UGGT1	2:1280911	5.49097	7.77756	2.65449	2.41024	2.70741	7.02749	2.25218	3.61301	0.318783	4.75504	0.4043	-0.39589
ENSG0000	MED23	6:1315731	15.0596	18.1445	3.91585	3.57837	2.44896	10.7135	6.43535	4.56574	3.93476	5.6278	0.3043	-0.46874
ENSG0000	MFSD3	8:1445090	10.1847	11.1517	11.0103	8.56402	13.5773	10.3383	19.8375	4.47934	6.76519	14.849	0.9176	-0.05383
ENSG0000	MANF	3:5138504	34.214	27.637	20.6071	23.1231	25.4341	53.2784	58.6015	17.6045	20.069	10.0659	0.5993	0.219606
ENSG0000	CNTLN	9:1713498	0.954878	1.74852	0.059122	0.035618	0.034301	4.96018	1.96792	0.927775	0	0.951109	0.19025	1.81216
ENSG0000	RING1	6:3320849	2.9081	3.40137	2.96751	2.79392	3.4549	2.9681	4.23047	3.43283	3.35149	3.4337	0.8188	0.193177
ENSG0000	NIPA1	15:227578	2.3977	3.09349	5.4327	2.63556	1.59374	3.29757	3.40115	9.31503	3.89649	5.15392	0.3719	0.585939
ENSG0000	GSE1	16:851695	4.59427	4.42534	4.51047	2.36676	6.67545	5.57082	3.91217	6.42791	2.82341	16.9299	0.2762	0.565128
ENSG0000	PSMD5	9:1208154	16.9797	11.4722	6.89505	7.79706	5.52007	10.9313	13.612	9.30328	8.67608	7.94558	0.84075	0.079691
ENSG0000	RP11-452	16:314495	0.192478	0.501272	1.00765	0.305511	0.226435	0	0.290567	0.558306	0	0.365237	0.67735	-0.67862
ENSG0000	AC007773	19:323900	0.535403	0.357697	0.391422	0.069877	0.4014	0	0.853178	0	0.140668	0.371574	0.8122	-0.45192
ENSG0000	RP11-894	14:103553	1.01516	1.41936	0.027871	0.960105	0.206233	2.51327	0.732721	1.02679	0.198456	2.13566	0.5412	0.86389
ENSG0000	RNF139	8:1244747	43.7229	37.978	30.0571	52.8321	20.2019	42.1289	31.2885	31.9942	35.6895	22.5222	0.59705	-0.1752
ENSG0000	ARHGAP2	17:453939	43.7051	61.6058	10.3371	15.5625	12.9532	29.9037	37.1897	20.649	11.1751	28.8723	0.59435	-0.18342
ENSG0000	KMT2A	11:118436	15.5839	28.1615	7.63674	2.60435	16.8712	21.5592	30.0542	11.6135	6.28664	42.9892	0.423	0.533542
ENSG0000	AF131215.	8:1089604	0.382416	0.867376	0.264729	0.092327	0.476195	1.33861	0.456	0.422851	0	1.35582	0.71595	0.657905
ENSG0000	IGLC7	22:229225	0	2.79567	0.235516	0	0.345477	0	0	0	0	0.154577	0.26285	-3.60412
ENSG0000	RAB35	12:120095	12.1718	8.83985	2.06617	3.20481	5.36886	10.0709	13.9331	7.78111	1.22812	8.26969	0.69895	0.240736
ENSG0000	CTD-2165	5:1462001	0.263955	2.05424	0.110984	0	0	0.33124	3.34324	0.025932	2.38578	0.4241	0.45865	1.65497
ENSG0000	ZNF284	19:440253	0.511655	0.785437	1.41895	0.596268	0.892742	0.816348	0.568048	0.364893	0.015511	2.28919	0.80025	-0.36169
ENSG0000	AC005300	22:171787	0.196744	0.626997	0	0	0	1.80016	2.58951	0	0	0.200831	0.3825	2.47236
ENSG0000	RP11-20B	17:288977	7.36663	1.98724	5.10539	7.04267	4.77403	1.96692	1.97922	5.7326	4.29936	5.62784	0.8521	-0.18033
ENSG0000	DDX19A	16:702896	22.3768	20.0317	20.9188	18.5767	13.7516	14.4608	11.2914	15.6318	11.6774	17.2649	0.3223	-0.47559
ENSG0000	UQCRFS1F	22:397430	6.51715	5.27128	5.08071	9.41264	3.65098	9.63136	6.49094	2.45735	3.59405	0.748081	0.54645	-0.4375
ENSG0000	CAPS	19:590484	7.00222	11.4205	3.8389	2.70822	5.13056	10.6567	8.57001	9.26797	5.93618	15.2894	0.4395	0.812808
ENSG0000	AF001548.	16:156432	1.60075	3.75754	14.7635	4.73229	8.92269	2.76434	0.973112	6.62038	2.15453	10.3188	0.7652	-0.6272
ENSG0000	VEZF1	17:579715	21.9712	14.3953	43.6311	28.661	55.23	7.94536	11.7733	33.0228	24.4659	73.3449	0.42095	-0.32611
ENSG0000	IL1B	2:1128297	3.10795	9.29741	1.31904	1.18664	3.22502	6.14447	0.022514	51.5746	32.4159	12.884	0.00015	2.67109
ENSG0000	TRAM2-A	6:5257730	0.570225	0.929072	0.633164	0.668643	0.614134	0.824815	0	0	0.953315	0.391311	0.60395	-0.71348
ENSG0000	P2RX5-TA	17:366289	1.14218	1.23071	1.77796	2.10067	2.42557	0.586293	1.05616	2.3663	0.112203	3.09586	0.8178	-0.42091
ENSG0000	CSPP1	8:6696409	6.74722	9.43609	3.38499	4.7737	5.34111	6.95776	5.33283	3.41489	5.2087	6.52136	0.9094	-0.09565
ENSG0000	MIR503HG	X:1345433	0.107406	0	0	0	0	0	2.08622	0.982286	0	0.410178	0.257	4.66513
ENSG0000	S100A2	1:1535611	0.648937	0.264034	0.291211	0.324598	0.788413	0	0.991297	0.128444	2.58149	1.53378	0.1564	1.6112
ENSG0000	PQLC2	1:1931232	4.04508	1.60608	6.97773	7.5055	5.51658	8.98296	5.0496	11.9664	7.77605	4.62067	0.36265	0.519307
ENSG0000	RPS17	15:825367	283.724	239.62	485.334	326.231	228.225	294.62	357.105	318.687	201.657	42.5641	0.22295	-0.48519
ENSG0000	SMOX	20:412097	0.673833	0	1.0494	1.14318	0.294929	5.35052	2.33749	1.21735	8.41274	7.2302	0.0045	2.93629
ENSG0000	NUDT4	12:933778	16.5461	21.6431	7.73484	8.18809	8.14574	12.0947	14.8702	13.789	12.6067	12.5641	0.96555	0.039858
ENSG0000	FITM2	20:443028	0.864475	1.08235	1.51602	0.659868	1.07049	1.46882	0.545295	0.933826	2.42255	2.00399	0.52195	0.437708
ENSG0000	RPL21P10	14:776724	0.126929	0.079995	0.867764	0.117419	0.188287	0	0	0	0	0.330503	0.42445	-1.68121
ENSG0000	TLE3	15:700477	6.45135	7.98605	3.54195	1.58238	5.74877	10.5692	13.5926	8.18872	4.3413	13.4025	0.08265	0.896336
ENSG0000	SEPT7P1	14:350449	0.631194	1.02691	0.25028	0	0	0	0	0	3.39966	0.239445	0.6484	1.10123
ENSG0000	UFL1	6:9635837	15.4664	18.3732	10.8338	9.32055	11.6172	12.8648	12.9625	11.6077	13.9307	17.41	0.9848	-0.00595
ENSG0000	RN7SL587	14:775059	0.411486	0.773771	0.157459	1.19889	0	0	0.93214	0	0	0	0.4955	-1.58319
ENSG0000	TRANK1	3:3682681	9.73688	6.13474	8.46067	16.1023	17.0505	6.78658	15.0902	9.33182	10.8649	23.7516	0.93235	0.077484
ENSG0000	RP11-307	1:1550338	0	0	0.164001	0.343292	0	0	0	1.59454	3.41508	0	0.3398	3.35787
ENSG0000	IGKV3D-1	2:9017280	0	9.55323	0.099054	0	0.097436	0	0	0	0	0	0.00105	#NAME?
ENSG0000	AKAP8L	19:153533	105.204	123.43	69.5739	64.0353	64.125	100.939	104.559	67.119	54.2914	84.1042	0.83595	-0.07637
ENSG0000	CUX1	7:1018159	14.8315	25.4189	8.59556	5.67013	10.0304	13.5147	8.19967	2.9307	9.14288	9.71723	0.279	-0.5934

ENSG0000(	RP11-102	3:1362503	0.447495	0	0	0.175545	0.074361	0	1.35341	0.154706	2.58472	0.183788	0.2327	2.78375
ENSG0000(	PDGFA	7:497257-	0	0	0	0.118684	0.365292	0.264325	2.6188	2.79456	0	1.12507	0.13835	3.6221
ENSG0000(	RPL41	12:561165	1135.6	711.842	1520.24	1068.57	608.128	1103.58	1307.92	1096.06	756.238	107.539	0.63055	-0.17197
ENSG0000(	IRF1	5:1324816	93.1381	72.4987	70.7527	67.2436	107.358	134.995	173.91	98.6944	37.6352	189.64	0.5614	0.261811
ENSG0000(	SLC35C1	11:458040	9.27673	6.65833	10.1084	11.3913	11.8249	1.5136	15.1127	18.6504	12.6421	14.9039	0.60865	0.282099
ENSG0000(	C9orf16	9:1281602	99.8214	83.1163	181.108	168.501	151.564	110.536	145.947	183.045	116.941	35.9592	0.37635	-0.29623
ENSG0000(	ACSL4	X:1096236	9.56272	11.3687	2.16636	1.18984	2.17007	10.3912	10.6565	3.86648	1.95753	6.73535	0.2879	0.409765
ENSG0000(	CTB-129C	5:1802932	0.898705	0.58329	0.121016	0	0.366851	0	0.974418	0.528496	0	0	0.74725	-0.50401
ENSG0000(	TCF7L2	10:112950	1.17764	0.425361	1.81449	0.84833	5.97248	3.89489	0.87305	0.651448	3.30876	2.17288	0.674	-0.26285
ENSG0000(	CH17-472	1:1204898	3.26818	4.4993	0.725884	0.45535	2.14027	5.39556	4.88621	4.64661	5.69874	14.244	0.0167	1.73791
ENSG0000(	TFF3	21:423116	0	0	0	1.01168	0	0.943396	1.89141	0.654024	0.372006	0	0.43095	1.48634
ENSG0000(	ZEB1	10:311878	10.1009	18.3357	8.22353	6.40914	14.0975	4.58366	11.8999	4.88369	26.544	26.5512	0.9723	0.017576
ENSG0000(	DOT1L	19:210098	1.32153	2.1097	2.55013	1.97416	4.28121	3.49649	5.54016	3.41374	1.615	7.42091	0.4671	0.618374
ENSG0000(	HSPA1L	6:3180961	2.73218	0.776826	2.64518	1.12291	3.5159	3.0164	1.70326	0.551277	1.32982	4.04549	0.87205	-0.14613
ENSG0000(	AMMECR1	2:1278616	4.2743	3.72412	2.96191	2.14333	2.11685	1.68689	0.974412	3.84601	0.540336	4.96064	0.40935	-0.46539
ENSG0000(	RP11-644	12:556815	0.545601	1.04476	0.558375	0.052124	0.761679	0.91399	1.88559	2.01755	0.064574	2.21817	0.4815	1.26671
ENSG0000(	EIF3F	11:797025	88.4318	114.598	201.774	155.709	190.34	68.7203	77.091	158.114	154.1	84.0106	0.1314	-0.51137
ENSG0000(	RAB14	9:1211781	28.7417	34.2321	11.1136	16.811	10.6716	24.8946	29.9897	18.8583	11.312	19.0376	0.78035	0.099579
ENSG0000(	SEC14L2	22:303319	2.55927	8.25399	2.57874	1.09789	0.295745	7.68503	4.66116	1.47948	3.30611	2.06538	0.80545	0.275876
ENSG0000(	ZFP90	16:685300	8.62594	5.04109	8.40453	2.6827	6.6035	5.02863	6.95543	7.61157	4.08045	17.8172	0.3949	0.546604
ENSG0000(	POGLUT1	3:1194689	18.8786	17.1087	18.9838	13.0706	18.0108	11.1157	10.4428	16.2904	5.04425	24.2915	0.3353	-0.38338
ENSG0000(	UBR7	14:931849	17.177	23.0284	10.0636	11.9486	10.7742	9.01825	13.3891	11.4497	13.5486	14.3982	0.6362	-0.24079
ENSG0000(	CTD-2017	19:240334	1.65489	2.21424	0.587167	0.245595	0.375519	1.05592	0.941382	0	0.189178	0.404712	0.5749	-0.9289
ENSG0000(	IKBIP	12:986134	13.3203	9.58759	15.718	14.4449	18.9203	15.557	26.7078	16.6228	22.7256	6.93895	0.6342	0.245754
ENSG0000(	CCDC47	17:637028	26.2929	26.741	19.3852	29.5427	28.7669	29.3838	27.2237	36.1241	24.5843	43.9769	0.6066	0.250861
ENSG0000(	AMT	3:4941677	5.98191	8.42657	0.338706	3.7877	0.465027	1.56532	3.70942	1.03601	2.02267	0.867082	0.66475	-0.30631
ENSG0000(	KDM7A	7:1400847	3.0682	3.22531	2.89549	1.72157	2.7666	8.00431	3.29277	3.83422	3.94407	7.88115	0.03625	0.798836
ENSG0000(	HES4	1:998961-	1.19599	11.785	0.500199	1.60405	1.24931	8.08225	43.3009	7.5687	6.58951	2.83717	0.07065	1.96647
ENSG0000(	WDR25	14:100333	9.16008	10.4045	6.48072	5.46966	8.20308	7.91103	8.61608	11.8268	2.36588	4.48476	0.81395	-0.21005
ENSG0000(	CLDN12	7:9033522	0.494328	1.22354	0.401999	2.54716	0.251325	0	0.692544	0	0.024938	0.564081	0.0059	-2.52021
ENSG0000(	POLD3	11:744917	9.42884	10.5794	5.34387	2.80921	7.67633	18.2978	14.6134	6.42695	5.38195	11.0015	0.3114	0.459647
ENSG0000(	KMT2D	12:489951	5.31537	7.05638	1.83798	0.779179	7.66605	2.88371	9.4375	3.64404	1.38852	11.2695	0.82795	0.229379
ENSG0000(	GPAT4	8:4152920	24.9996	29.9437	14.1008	17.3333	15.0287	27.0606	31.7018	26.0468	15.0291	23.7361	0.8388	-0.089
ENSG0000(	IL32	16:306529	1718.08	1266.61	1682.96	3024.25	3597.27	1526.88	2094.13	1108.25	2677.39	933.84	0.3213	-0.43283
ENSG0000(	LYSMD1	1:1511566	0.227939	0.27625	0.832699	0	0.485076	0.226012	0.636424	1.37258	0	1.23984	0.56435	0.862508
ENSG0000(	RPL3P4	14:989728	13.4514	9.06198	6.01349	4.40899	4.52676	7.48559	8.83667	2.46535	3.00117	1.01299	0.44915	-0.69893
ENSG0000(	CTD-2647	8:2889039	18.8564	32.6947	9.76938	1.97986	5.03306	36.0482	45.0561	10.0028	4.76515	7.65723	0.5609	0.693358
ENSG0000(	IGHV1-69	14:106714	0	2.26464	0.994632	13.794	0.392833	0	0	0	0	0	0.00325	#NAME?
ENSG0000(	PUS10	2:6094022	7.44332	8.46118	4.40222	8.41471	3.78817	6.07669	4.58987	5.8261	6.25588	3.00866	0.7444	-0.29628
ENSG0000(	BACH1	21:291934	6.37565	10.846	1.31192	1.21888	4.13295	12.7658	11.403	2.1183	4.06179	4.7182	0.38985	0.536474
ENSG0000(	KDELC1	13:102784	4.64667	0.791582	2.06274	4.39693	2.36695	3.95859	2.60058	1.88582	10.2263	2.09256	0.44395	0.522606
ENSG0000(	ENO3	17:494565	5.23501	8.71562	7.14267	10.4454	6.25558	16.8872	6.96106	3.53937	0.203013	10.8053	0.8838	-0.09518
ENSG0000(	IDUA	4:958886-	18.7355	21.7222	11.5237	7.18996	6.77192	14.3028	31.8855	15.6876	19.5905	12.5442	0.4925	0.507439
ENSG0000(	RP11-571	4:5337764	0.359735	1.29932	1.10506	1.20291	0	0	0.944493	0	4.34594	0.492693	0.6903	0.736779
ENSG0000(	RGP1	9:3574928	3.61912	2.33056	1.72739	0.886971	2.0521	1.84125	1.52722	2.92602	1.20009	7.13102	0.73665	0.191383
ENSG0000(	MPPE1	18:116889	49.0604	64.8851	36.8668	40.5812	17.9459	49.5091	47.2967	30.3132	24.6285	29.081	0.5486	-0.29191
ENSG0000(	IFI44	1:7864979	44.0082	22.9743	38.6004	47.6104	30.6482	58.3695	253.112	88.799	69.2338	64.0606	0.00045	1.68258
ENSG0000(	HINT1	5:1311590	310.53	189.238	295.063	286.106	215.281	313.608	298.441	283.674	246.286	50.6248	0.71735	-0.13753
ENSG0000(	TBXAS1	7:1395615	15.1845	35.6748	14.0503	10.5083	22.3608	33.7233	2.92786	16.9589	14.8678	25.2722	0.88	-0.07831
ENSG0000(	STARD3	17:396370	50.4261	41.2154	46.9688	35.8059	41.2089	89.2332	47.9186	62.1507	61.4259	74.0122	0.0423	0.629163
ENSG0000(	KIAA0040	1:1751569	18.6635	21.199	17.0066	8.27798	40.01	11.8787	18.5353	21.9737	15.1344	36.1263	0.4514	-0.3791
ENSG0000(	DCUN1D1	3:1829380	11.2353	8.4415	5.09021	3.11226	4.7455	13.9495	6.1211	6.62303	11.3841	5.88865	0.35535	0.36047
ENSG0000(	RP11-336	10:568483	0.275026	1.20081	0	0.879565	0	1.44171	1.1772	0.244636	3.92031	0.576504	0.3789	1.69978
ENSG0000(	NIPSNAP3	9:1047641	0.19613	0.799512	0.499087	0.893361	0.159083	2.32463	0	1.0606	0	0.533196	0.7559	0.398352
ENSG0000(	IGLV3-12	22:227718	0	0	0	3.65164	0	0	0	0	0	0	0.0012	#NAME?
ENSG0000(	ZNF793	19:374971	0.928581	1.22835	0.771684	1.2315	0.314074	0.662964	1.2213	0.441835	0	0.386116	0.3516	-0.91919
ENSG0000(	MT1E	16:566254	4.45151	6.50303	6.5737	4.10407	7.52163	10.1866	6.10454	5.2828	1.66795	1.47801	0.5988	-0.40479
ENSG0000(	CPNE8	12:386468	0.405582	1.79024	0.736653	0.154798	0.693785	5.11518	0.272353	0.54972	0	1.26352	0.5687	0.555216
ENSG0000(	STAT6	12:570888	71.1575	86.5998	32.6462	34.1713	32.9631	38.6185	32.4591	28.3058	14.8234	49.5027	0.0962	-0.62194
ENSG0000(	ST8SIA1	12:220461	0.99304	3.87731	0.743521	0.057616	0.1431	1.2767	1.93547	2.44934	5.12372	4.2275	0.29815	1.3156
ENSG0000(	CTC-559E	19:197573	0.338702	1.06245	0.460554	0	0.684439	0	0.183937	0.698114	0	0.870233	0.69885	-0.60496
ENSG0000(	RP11-602	4:1098150	0.422377	0.801352	0.669964	0.414864	1.34543	0.761182	0.688075	0.181019	0	0.221921	0.4835	-1.24202
ENSG0000(	FAM91A1	8:1237684	10.6379	9.86244	2.72673	4.50164	2.72221	7.07531	7.63215	3.19307	4.67313	6.02472	0.7138	-0.13784
ENSG0000(	RNF121	11:719287	6.36768	6.77126	10.0493	7.32098	5.74713	13.369	15.8714	3.60283	8.06261	5.55567	0.64155	0.285189
ENSG0000(	DDX50	10:689012	37.6958	48.4262	31.8074	35.9083	22.8644	13.0235	35.1433	27.103	25.8478	24.3076	0.23185	-0.40915
ENSG0000(	TONSL	8:1444287	1.1055	2.08316	0.522113	0.424909	0.631218	1.36352	1.15305	0	2.35595	1.17221	0.65325	0.466011
ENSG0000(	FAM127B	X:1350509	15.4129	25.9684	21.9033	16.8787	29.0491	26.0575	49.0098	38.5916	26.1364	30.8783	0.25105	0.530186
ENSG0000(	MRC2	17:626260	0.616596	0.61907	0.045396	0	0.013073	2.12918	1.06628	0.342437	0	0.321227	0.14875	1.57015
ENSG0000(	CASC15	6:2166477	0	3.10165	0	0	0.014584	0.418072	0	0	0	0	0.27515	-2.81164
ENSG0000(	C17orf75	17:322661	16.1311	18.377	16.5551	13.5486	18.3084	20.3284	31.8438	16.2809	16.5527	9.84947	0.85475	0.164109
ENSG0000(	ZNF699	19:929151	4.67233	4.12313	2.709	1.19669	2.02512	0.824106	5.19159	2.13516	8.43102	2.14687	0.61985	0.588079
ENSG0000(	TPGS1	19:489175	56.7393	21.0076	22.7323	55.7345	74.508	41.6555	45.067	14.7094	25.6759	22.5936	0.0867	-0.83678
ENSG0000(	DGAT1	8:1442915	37.2973	38.8619	16.8827	9.91417	20.541	33.9983	33.2813	16.6974	9.77782	23.7591	0.797	-0.25506
ENSG0000(	FAM76A	1:2772597	2.05678	2.08852	14.903	6.75802	5.08296	1.84578	2.22547	14.2923	10.3446	8.82945	0.7984	0.120898
ENSG0000(	CYP27A1	2:2187817	1.64984	2.13816	0	0.229996	0.355571	50.4101	1.09944	0	2.90518	1.33057	0.00995	3.76412
ENSG0000(	TSPAN18	11:447193	1.97287	2.6037	1.29573	0.595893	6.04447	0.046124	0	0.656298	0.137622	2.32138	0.10505	-1.74185
ENSG0000(	POMT1	9:1315029	29.612	27.3901	27.8074	34.8157	19.2474	5.00446	14.1887	10.9899	23.2073	16.1617	0.13285	-0.8381
ENSG0000(	HLA-E	6:3048946	1067.15	1153.97	2589.99	2296.83	1900.18	800.201	1247.44	1774	1691.8	1793.31	0.67665	-0.3498
ENSG0000(	EMC3	3:9958716	49.1904	32.4501	65.3366	51.2455	56.8806	75.7116	74.5508	55.4014	34.53	32.6652	0.58625	-0.21831
ENSG0000(	MICD	6:2997080	0	2.21447	0	0	0	0	1.39352	0	0	0	0.64175	-0.58238
ENSG0000(	RSAD1	17:504787	41.9969	37.5066	55.9371	72.2917	42.5032	15.1544	41.4561	30.8639	13.5448	33.1421	0.0042	-0.92679
ENSG0000(	RP11-87H	9:4099224	3.09765	2.5685	2.3093	2.03382	1.09758	4.23974	3.41394	4.25518	5.72213	4.47353	0.4808	0.994527
ENSG0000(	ATP13A3	3:1944026	14.0368	10.7164	2.13593	1.21231	2.85566	10.3052	14.4532	4.88902	1.72981	7.14648	0.20885	0.534819
ENSG0000(	LRRFIP1	2:2376275	101.689	130.451	93.0774	73.5668	146.784	128.951	145.404	115.183	80.5805	187.824	0.93615	0.027833
ENSG0000(	SIMC1	5:1762383	7.46501	7.59435	4.03563	3.3366	3.48798	8.48481	4.87431	3.28427	4.03428	6.56729	0.88865	0.073205
ENSG0000(	ACRBP	12:663807	2.70757	2.21787	3.06832	3.51688	0.839547	3.20036	0.078658	1.87817	0	1.44454	0.31645	-1.20142
ENSG0000(	C7orf31	7:2513469	2.24639	3.03986	0.522224	0.090921	1.16208	0.333124	2.61725	0.91295	1.03987	1.42183	0.6776	-0.28685
ENSG0000(	RP4-728D	1:3556981	5.2645	3.12538	6.29905	9.59484	3.39096	13.8162	2.66047	3.82901	5.60582	0.262572	0.80665	-0.20295
ENSG0000(	VPS28	8:1444236	136.726	130.393	177.608	169.429	187.059	148.373	192.802	178.996	152.554	81.8334	0.7201	-0.13144
ENSG0000(	TSSK4	14:241718	0.991891	0	0.740574	0.139987	0.245843	0.734654	0.934517	0.06546	0	0.431353	0.95005	0.078628
ENSG0000(	XRN2	20:213027	45.1491	45.7233	29.3736	31.0654	32.8632	22.6859	28.3277	35.0806	24.1705	41.3721	0.39265	-0.27788

ENSG0000	MAPKBP1	15:417744	2.7642	4.18536	0.843691	1.14258	1.19943	3.23916	0.45441	0.185646	0.2643	2.28726	0.12835	-0.92225
ENSG0000	RN7SL363	15:908559	0	0	0.149785	1.79386	0	0	0	0	0	0	0.00475	#NAME?
ENSG0000	PLK2	5:5845398	2.3583	1.84343	0.267739	3.90467	0.17217	0	1.40606	0	1.85997	0.075431	0.317	-1.00995
ENSG0000	TXNDC12	1:5202013	89.5592	60.5955	103.787	97.2811	67.2645	99.7686	89.0823	68.0413	83.0073	27.9493	0.6577	-0.19733
ENSG0000	RP11-127	5:1776112	4.74241	12.234	1.09039	2.9435	1.71274	9.58827	2.32184	0.640885	2.39087	3.80911	0.89925	-0.13132
ENSG0000	AC112497	X:1567493	0.513807	0.955985	1.22272	0	0.444662	0	0.195566	1.42684	0	1.01835	0.8996	-0.21729
ENSG0000	ZNF680	7:6451988	5.96775	4.54221	2.37736	2.55918	1.67632	3.22649	1.0713	3.28206	5.01909	2.86842	0.92665	-0.05209
ENSG0000	TRBV23-1	7:1426459	26.4743	58.9683	23.8332	43.6644	18.4657	77.7423	21.5808	21.7386	86.7407	78.7507	0.03895	1.08489
ENSG0000	TASP1	20:133893	8.77103	25.7024	6.54808	11.6898	7.62446	6.80146	6.43358	7.55088	3.99788	6.61689	0.06325	-0.92666
ENSG0000	JAM3	11:134068	0.496636	4.10863	0.448592	0.910287	0.86873	0	2.11056	0	0	2.40112	0.4939	-0.93502
ENSG0000	KIAA1841	2:6106587	1.53505	3.28507	0.579477	0.935192	0.654922	3.5639	3.75046	0.818528	0.04169	2.61591	0.6702	0.397119
ENSG0000	PSD4	2:1131573	21.4444	24.8602	10.5116	12.5928	21.8813	16.5841	18.3764	26.2919	12.1106	51.3034	0.2692	0.507266
ENSG0000	PITRM1-A	10:306633	0.336416	0.396256	0.057914	0	0.082534	0.642837	0.797043	0.56599	0.615468	0.309987	0.20775	1.78475
ENSG0000	RPL32	3:1279647	579.678	693.443	1015.21	672.249	327.902	605.644	596.797	600.033	455.195	42.2533	0.17005	-0.56898
ENSG0000	FASTKD3	5:7830377	11.1451	13.1329	9.38235	15.978	7.2692	9.00936	12.5149	7.71445	3.23355	9.60504	0.5758	-0.38482
ENSG0000	BAK1	6:3354004	47.8441	41.823	31.6217	49.3565	33.1575	25.7301	70.973	51.0637	81.1833	35.5874	0.22545	0.408477
ENSG0000	CDRT4	17:154360	2.91285	5.03067	0.459823	2.25859	0.479074	3.44718	1.26215	2.03832	0.106602	3.4028	0.9334	0.080982
ENSG0000	DCP2	5:1129767	21.0042	24.6212	18.4644	20.3467	22.61	22.6039	32.7292	13.5085	23.2761	21.1496	0.80845	-0.15836
ENSG0000	PCCA	13:100089	14.0395	5.83728	11.7632	9.98346	8.40143	1.55841	11.3965	4.16588	9.09601	13.17	0.5197	-0.36694
ENSG0000	AKIRIN1	1:3899122	52.2556	47.1108	60.79	76.0239	46.4437	56.2058	40.834	45.2466	48.0457	43.7363	0.2768	-0.34833
ENSG0000	ZNF507	19:323455	4.56583	4.0027	1.37319	2.12395	1.64349	0.8654	2.96261	3.05601	5.1615	4.55841	0.86795	0.080989
ENSG0000	MYCBPAP	17:505083	1.52927	1.38386	0.369864	1.11302	0.123794	0	0	1.10454	0.029707	0.290275	0.1982	-1.28726
ENSG0000	GNA15	19:313619	3.48938	3.64355	0.622574	6.34328	2.30675	20.1236	10.6513	14.5646	30.3415	16.3266	0.004	2.53239
ENSG0000	PHF8	X:5393667	8.52366	9.91607	4.46392	5.02034	7.86782	3.32446	6.46537	6.40243	8.33025	15.1828	0.824	0.088046
ENSG0000	ZFP1	16:751484	7.39991	7.48233	2.47661	1.15317	2.92548	6.46831	4.28271	6.94519	5.93406	2.97213	0.4554	0.577759
ENSG0000	CTD-2047	17:802608	0.404468	0.192088	0.086735	0.281165	0.41277	0.181399	3.08655	0.744515	1.10849	1.04194	0.0866	2.11832
ENSG0000	NASP	1:4558384	75.1327	92.4436	75.3619	72.6111	118.814	102.621	118.856	91.8809	43.0621	116.394	0.7981	0.086876
ENSG0000	DNAJC2	7:1032974	38.5979	39.2544	37.7428	35.6377	52.5344	51.5344	57.9768	33.917	35.0847	70.2459	0.7539	0.220853
ENSG0000	VEZT	12:952177	23.5719	20.8308	18.8731	23.9333	12.0783	10.8706	23.279	13.1311	11.1674	17.0144	0.258	-0.41752
ENSG0000	ITPRIPL2	16:191139	0.656821	1.0982	0.05405	0.321149	0.118998	6.04894	2.70134	0.240673	0.556474	1.14045	0.1902	2.21984
ENSG0000	GGT1	22:245404	23.0909	7.54215	9.15475	16.9505	12.6454	29.1243	6.74988	19.7108	19.4675	18.7176	0.3689	0.549678
ENSG0000	RP11-57H	10:112823	0.678749	0.581558	0.24962	0.13501	0.316141	0	1.82281	0.209651	0.700314	0.751114	0.3552	0.854719
ENSG0000	IQCK	16:195552	1.02874	2.61733	1.55616	1.94178	0.434193	1.74844	0.944988	2.03151	0	1.12802	0.46715	-0.62514
ENSG0000	KCNH3	12:495391	1.34389	1.12623	0.352095	0.556259	0.771068	0	0	0.139282	0	0.439863	0.11155	-2.65118
ENSG0000	RPL21P11	16:915640	32.0052	25.1398	8.86352	7.96986	1.9712	19.7434	25.9124	4.65196	4.44342	0.294464	0.7426	-0.586
ENSG0000	ITCH	20:343632	0.130419	1.46642	0.006931	0	1.64372	2.05683	1.2313	0	0.060224	1.95187	0.62825	-1.16686
ENSG0000	ZNF616	19:521130	2.04443	2.61477	1.83624	1.87765	1.31423	2.55399	1.10302	2.139	0	1.63776	0.46215	-0.6316
ENSG0000	MBD3	19:157359	15.8518	18.9642	6.84533	4.28829	13.5419	16.8284	24.4629	9.81407	2.47139	19.2675	0.86805	0.238159
ENSG0000	MYOM2	8:2045039	3.80048	38.7627	5.15741	68.288	0.819205	0.304452	2.49691	0.081564	0.063798	0	0.0011	-5.75528
ENSG0000	ZNF133	20:182882	6.44494	6.57558	6.55868	7.42922	6.47553	1.3547	7.49473	3.84438	0.053545	9.20364	0.27325	-0.69194
ENSG0000	FXR1	3:1806021	32.1128	28.1731	19.3615	16.2125	28.1085	22.903	33.2809	18.1955	16.9969	29.0244	0.93915	-0.04025
ENSG0000	IDO1	8:3990227	0	0.840798	0	0	0	17.4186	3.6118	4.37859	0	0.528243	0.22795	5.00646
ENSG0000	PLEKHG4	16:672775	3.75502	9.51756	1.18489	0.52042	1.26066	0.757494	1.61777	3.67202	2.13138	3.17855	0.7479	-0.20431
ENSG0000	RP11-402.	3:1112927	0.648047	1.3585	1.78374	1.97031	1.54056	3.47974	1.95579	2.60455	1.02832	7.66609	0.3884	1.04464
ENSG0000	ZNF391	6:2737461	0.258981	0.902525	0.386036	0.090365	0.114504	0	0	1.56659	1.79547	0.780352	0.4154	1.32885
ENSG0000	SMARCE1	17:406249	169.924	156.198	153.31	121.033	153.812	179.412	140.59	124.148	97.5579	85.9678	0.4028	-0.30441
ENSG0000	RGCC	13:414575	46.2228	222.218	71.8754	48.6424	38.7385	227.067	400.604	232.372	137.314	48.8409	0.00065	1.31408
ENSG0000	CKAP5	11:467430	13.683	14.6804	6.75431	2.85316	15.945	8.04646	9.79306	7.51393	3.35356	23.9204	0.702	-0.22604
ENSG0000	ING4	12:665027	59.588	86.2726	73.6383	73.0547	43.8171	74.662	71.397	63.0924	65.4185	47.1772	0.84795	-0.05969
ENSG0000	SSTR3	22:372042	0.262535	5.56817	0.106235	0	0.104757	0.742383	1.50874	0.940696	0	0.347532	0.77755	-0.69247
ENSG0000	TARSL2	15:101653	10.0262	11.9002	6.03841	5.51363	7.06042	15.9725	12.1291	8.59488	7.74398	10.0091	0.33075	0.413606
ENSG0000	APOO	X:2383335	12.6939	7.10762	12.1527	12.8099	10.9911	30.9392	13.6744	8.69428	15.4639	4.27947	0.49955	0.399118
ENSG0000	AD001527	19:361111	0.433542	0.394656	1.5367	3.66656	0.774001	0	0.213117	0.718522	0	0.587196	0.2381	-2.09949
ENSG0000	ZRSR2	X:1579047	25.8247	26.1535	30.1931	32.245	32.3375	24.2224	27.8943	25.1944	21.4275	30.5758	0.60775	-0.19807
ENSG0000	C15orf39	15:751956	1.17185	1.35544	1.89167	0.481865	3.34066	0.903709	0.993686	2.27456	1.17402	6.63976	0.477	0.471215
ENSG0000	RP5-1091	X:7118355	9.22878	7.81205	14.523	9.93102	19.3402	7.96602	12.4947	12.0508	3.92168	4.63182	0.3944	-0.62032
ENSG0000	RP11-421	12:269715	1.056	1.55608	0.260111	0.858336	0.11309	1.01055	1.97832	0.444316	0.936184	0.175458	0.80605	0.425603
ENSG0000	BCLAF1	6:1362568	41.9153	48.7435	32.6729	17.4163	50.4971	35.5568	48.7676	29.171	34.9379	78.4728	0.8583	-0.06816
ENSG0000	CFLAR-AS	2:2011161	0.497051	0.242912	0.112284	0	0.147791	0.200937	1.95224	0.564899	2.93004	0.441432	0.0252	2.86606
ENSG0000	KRBA2	17:836556	1.65991	3.58303	2.43851	1.64452	1.86299	0.673131	2.19636	1.39874	0	4.41169	0.76195	-0.5551
ENSG0000	HS3ST3B1	17:143010	8.23996	16.3151	4.72785	2.9	4.24768	18.0535	11.0335	11.2868	9.79463	13.1353	0.1044	0.818467
ENSG0000	ABCA10	17:691472	0.153371	0	0	0.013953	0.070022	0	0	0	3.10821	0.193881	0.0206	4.14353
ENSG0000	MDFIC	7:1149221	39.6229	24.573	18.4511	38.341	37.8251	34.7823	44.7069	27.115	42.3166	28.2658	0.90705	0.043069
ENSG0000	PTCD1	7:9932589	5.25563	6.12227	11.5529	12.8238	16.3237	1.15405	4.02277	5.92049	9.53144	19.0816	0.58075	-0.60283
ENSG0000	C3orf33	3:1557626	3.77852	2.62424	4.83334	5.42515	3.14818	2.70602	2.98835	4.82835	2.25373	2.66438	0.57845	-0.38827
ENSG0000	DCAF15	19:139311	2.36769	3.81767	0.758174	1.66733	1.26746	2.99645	3.5444	0.278038	0.399178	1.53069	0.64535	-0.3542
ENSG0000	PUDP	X:7048919	7.12947	6.90959	6.33528	7.30725	6.73379	5.58541	6.41901	6.81811	6.78146	8.65268	0.69635	-0.19025
ENSG0000	GLMP	1:1562829	16.2962	10.2216	18.3089	23.3026	13.3729	55.5732	6.05322	19.0484	16.0585	10.9949	0.525	0.366591
ENSG0000	FLT3LG	19:494462	73.0739	86.1915	76.6112	76.0172	95.0087	51.1511	64.7814	89.8307	62.4625	44.8621	0.42965	-0.38969
ENSG0000	SGSM2	17:205415	23.2234	32.7882	5.67845	5.50356	6.35575	21.5472	22.8367	10.252	3.84507	19.0297	0.71125	0.27023
ENSG0000	PMS2P3	7:7549362	9.39985	13.7694	3.45756	1.96132	5.49858	4.1038	13.6751	5.75052	0.361047	15.2125	0.8062	0.17611
ENSG0000	RP11-274	7:1286536	3.50136	6.19209	2.90598	1.79422	3.44709	2.42007	5.84155	3.93281	1.372	7.13233	0.74145	0.231427
ENSG0000	ZNF417	19:577696	7.85193	5.63081	2.60277	3.72939	1.91671	4.65203	2.90894	4.53412	0.988788	5.1999	0.79845	-0.22496
ENSG0000	RP11-262	9:6283774	2.22576	0.126528	0.967811	1.10073	0.247696	2.00194	0.867893	1.01063	0	0.108472	0.9344	-0.13225
ENSG0000	ZNF461	19:366371	4.18323	2.23359	3.53217	4.30108	1.76625	0.13365	3.69873	4.15182	5.69888	1.85279	0.83975	-0.12907
ENSG0000	LGALS3	14:551241	29.2148	36.6528	8.71602	31.0959	22.342	439.031	152.31	135.375	130.533	32.0768	0.00005	2.7339
ENSG0000	WRAP73	1:3630766	36.3016	34.9742	31.7495	27.14	28.9475	27.093	32.8647	33.2295	35.1594	32.1591	0.9648	-0.01851
ENSG0000	GGTA1P	9:1213389	0.212793	1.66108	1.80232	0.553371	0.052667	4.06753	0.814985	0	0.34039	0.066172	0.93495	0.170018
ENSG0000	ADCK2	7:1405184	15.1352	8.57103	8.45692	12.3777	12.316	10.1605	13.9847	9.26898	12.5167	19.102	0.89655	0.154932
ENSG0000	AC009950	2:2301213	20.8114	5.65862	8.57784	16.961	17.7885	37.0569	40.1662	11.3947	0.446082	41.1202	0.95665	0.047408
ENSG0000	ZKSCAN5	7:9950465	3.0209	1.45588	1.71876	0.746782	1.34621	1.93041	3.57719	1.38955	1.32594	3.12327	0.59815	0.355188
ENSG0000	PRDM8	4:8018327	6.31574	7.55809	2.0327	1.18358	3.45328	3.40648	7.20927	3.29197	1.23763	4.72728	0.9287	-0.04997
ENSG0000	SYNCRIP	6:8550549	37.6573	37.7729	23.9482	29.4436	42.888	47.4037	50.0762	28.9553	29.3311	51.5793	0.85325	0.084226
ENSG0000	AP006621	11:777577	4.0805	5.96364	4.71094	8.7896	3.06954	10.2314	4.11739	0.231513	0	1.56481	0.44995	-0.95796
ENSG0000	NOS3	7:1509909	2.94366	4.90936	1.71785	2.15713	0.757579	0	1.24171	1.13066	0.809916	0.376029	0.1231	-1.63218
ENSG0000	RP11-930	8:4165321	1.35477	1.8875	1.62936	0.847401	1.71556	2.69748	0.653942	1.67136	2.02462	1.87268	0.89405	0.308088
ENSG0000	RP11-182.	15:845078	0.252736	0	0	0.249224	0	0	1.95696	0.629043	0	0.272421	0.27095	2.7307
ENSG0000	RP11-109	17:354594	2.08527	0.04046	0.31454	0.664137	0.94318	1.49269	0.873098	0.582314	0.062659	0.837278	0.97175	-0.04945
ENSG0000	ZNF783	7:1492621	5.1524	5.08112	4.35365	1.22559	4.73524	4.9944	3.8438	3.19324	6.3993	9.71556	0.33555	0.465485
ENSG0000	CTD-2203	5:3342402	1.03228	0.61084	0.245525	2.35201	0.245597	0.368576	0.755673	0.177906	0	0.105529	0.3201	-1.74366
ENSG0000	ECHS1	10:133362	77.2601	68.3255	95.5514	85.0814	87.1859	117.934	95.1604	81.0839	65.7809	32.3392	0.6647	-0.14406

ENSG0000(	RP11-147I	17:682466	2.08442	1.21059	3.27838	1.87076	2.37268	0	0.31573	1.61444	0	1.84516	0.3585	-1.45239
ENSG0000(	ZNF8	19:582285	0.525364	1.08146	0.4475	0.799474	0.604506	0.415694	0.735144	0.139936	0.111169	0.895063	0.85655	-0.31438
ENSG0000(	RP11-362I	3:1697934	0.760412	0	0.334394	0	0.747461	0.112461	0.487739	0.847203	1.58259	0.243698	0.72005	0.753241
ENSG0000(	PADI4	1:1730819	2.36362	5.06862	1.21222	1.55705	0.04768	0.524566	0.201547	0	0	0	0.10415	-4.05371
ENSG0000(	PTPN22	1:1137618	116.1	39.0704	45.396	72.8085	23.9432	72.0568	123.374	50.8824	71.5354	63.293	0.2674	0.486775
ENSG0000(	KIAA2013	1:1191959	66.0792	65.4762	15.0897	6.21864	30.6637	42.439	65.2795	24.6505	6.32047	38.5971	0.90185	-0.0465
ENSG0000(	PIK3C2B	1:2043778	2.10666	0.946363	0.808276	2.02093	1.60122	1.2357	1.00451	2.56198	1.84575	2.84268	0.8619	0.17691
ENSG0000(	PPFIBP1	12:275234	1.60726	3.79711	0.048465	0.050698	0.271918	0	0.894794	0.614806	0	0.851029	0.1838	-1.30598
ENSG0000(	ARL8A	1:2021228	6.04368	3.90228	0.774422	0.486488	1.48868	6.36865	7.21837	1.60954	0.245275	1.94037	0.65395	0.365924
ENSG0000(	RC3H1	1:1739312	4.70302	4.3099	1.97915	2.69703	1.70944	2.16883	3.5204	2.19996	0.57087	3.71037	0.26505	-0.51718
ENSG0000(	GCAT	22:378079	0.723075	3.81995	3.54558	2.24588	0.466439	0	0.797914	0.35015	0.066666	0.567122	0.0115	-2.68596
ENSG0000(	LINC0101:	6:2987966	7.62627	8.4931	3.22607	6.46369	3.9906	16.7712	8.55221	1.65022	0	6.10384	0.9911	0.01247
ENSG0000(	ACTR3	2:1138899	152.438	143.074	72.1093	45.7031	58.0712	165.868	170.954	109.706	68.6183	109.927	0.71045	0.167318
ENSG0000(	PKM	15:721990	199.83	281.679	94.9027	81.3549	109.829	252.423	261.125	162.991	165.908	164.076	0.21285	0.395251
ENSG0000(	CPPED1	16:126597	12.8966	16.7768	8.16285	8.14442	5.95483	29.5665	7.13219	11.0001	10.652	8.65561	0.49595	0.324438
ENSG0000(	INPP1	2:1898796	18.1859	11.2122	17.5711	13.6568	14.8698	34.8144	23.6344	14.3553	29.7246	12.165	0.25715	0.589604
ENSG0000(	CDV3	3:1335737	49.4825	49.5149	24.6927	16.4926	26.2642	38.002	49.3321	34.3881	23.8979	49.9063	0.5417	0.19749
ENSG0000(	RP11-253I	12:124742	0.171948	0.327225	0.362529	0	0.127406	0	1.59306	1.38946	5.54942	1.88952	0.06125	3.55241
ENSG0000(	GGT5	22:242196	0	0	0	0	0.006794	3.20222	0	0	0	0	0.46905	8.30014
ENSG0000(	C17orf100	17:665171	0.696221	1.02489	0.491785	0.957334	0.287885	1.62176	0.268822	1.75832	0	0	0.68485	-0.69765
ENSG0000(	AP2A1	19:497669	4.92749	4.9259	3.50764	3.86565	12.0211	3.2418	4.5234	6.35212	2.50507	16.0116	0.95235	0.033116
ENSG0000(	COPZ1	12:542309	128.593	84.6923	126.129	127.34	121.797	96.3218	100.326	142.908	154.081	68.3989	0.9486	-0.05845
ENSG0000(	MTFP1	22:303319	34.6685	25.3829	98.1266	83.8623	64.638	29.8484	31.2392	65.8025	34.4151	17.7154	0.1818	-0.7942
ENSG0000(	SUMO4	6:1492179	0.695232	4.58904	0.338964	0	0.221116	3.4587	4.22337	1.17892	0	2.386	0.58535	1.07764
ENSG0000(	HPS1	10:984161	76.5685	87.9033	81.4452	84.6766	94.9376	83.5404	68.7031	67.561	80.965	75.8015	0.56405	-0.24352
ENSG0000(	HPCAL1	2:1030288	47.6125	67.6527	79.9471	88.1876	90.9568	45.0948	31.4646	58.2011	54.1016	88.5745	0.26935	-0.37446
ENSG0000(	ATG16L1	2:2332100	25.9137	18.2654	17.9787	24.4414	12.7074	21.194	33.2187	26.4769	33.6777	25.9098	0.18845	0.473748
ENSG0000(	CA10	17:516303	0	0	0	0	0	2.89441	1.37723	2.44473	0	7.54489	0.0001	inf
ENSG0000(	RFX3-AS1	9:3526722	1.84386	1.01121	0.667851	0.281115	0.582388	0.658748	4.22659	3.96162	0.48914	1.68552	0.28505	1.46532
ENSG0000(	CEP55	10:934966	6.12626	14.2372	0.263804	0.121956	0.142532	19.6388	10.3518	2.43674	1.30704	2.9425	0.2272	0.9185
ENSG0000(	TIMM10B	11:647468	22.4708	16.7416	27.9921	29.2479	24.1015	26.5423	25.0052	34.8446	20.7523	22.863	0.95285	-0.02899
ENSG0000(	RP11-484I	3:1129904	0.382851	0.144353	0.927036	2.4361	0.923549	0	1.58868	0.430749	0	0.746318	0.54385	-1.15881
ENSG0000(	CSRNP2	12:510612	6.04587	3.69741	2.64631	2.23533	2.59874	1.99649	7.07144	4.19408	1.81079	5.66065	0.7033	0.177222
ENSG0000(	STRADA	17:637028	16.1937	18.9857	5.76145	9.98088	7.19942	11.7471	12.0073	7.28133	3.45204	11.5352	0.62105	-0.3463
ENSG0000(	TNFSF12	17:754889	38.6593	27.3106	29.6761	29.8059	46.5786	51.1197	35.4069	32.4274	13.4781	30.2896	0.68665	-0.18422
ENSG0000(	FBRS	16:306584	10.3264	15.4522	3.62663	3.93624	7.83853	13.4422	20.8698	9.28698	4.00908	17.465	0.0802	0.846505
ENSG0000(	SLC40A1	2:1895605	1.35932	17.2561	1.3651	3.16155	0.050333	3.3966	0	0.118986	0	0.286294	0.0753	-2.75333
ENSG0000(	RAB30	11:828235	3.80557	4.77202	1.66668	1.88038	1.69006	0.417221	3.00821	5.72827	4.23855	5.2569	0.6796	0.262109
ENSG0000(	RARA-AS1	17:403091	0.996573	0.68631	0.583383	0.302533	0.482852	1.9922	0.595568	0.614685	7.49369	0.222003	0.2137	2.07807
ENSG0000(	ARF3	12:489034	9.65148	8.20332	9.31588	3.85764	11.0191	20.1831	12.5491	14.0889	12.3302	12.9888	0.44095	0.718666
ENSG0000(	NKG7	19:513716	1033.32	225.5	2245.18	1858.63	2472.68	628.435	842.054	1282.26	479.168	456.455	0.0195	-1.18453
ENSG0000(	UBD	6:2955551	0	0	0	0.050233	0	13.5692	0.526594	0	0	0.054345	0.2243	6.99527
ENSG0000(	PRMT9	4:1476173	7.96898	4.19765	4.38573	6.78404	3.62229	6.62638	2.51737	2.7767	2.02107	4.65512	0.4336	-0.58318
ENSG0000(	MS4A6A	11:601720	16.7647	55.8854	3.55807	3.13544	1.70704	133.603	3.88323	6.32434	5.52961	2.02612	0.27335	0.949374
ENSG0000(	RP11-147I	17:681257	0.336694	1.47283	0.609648	0.43134	1.92479	0.190743	1.58597	0.549413	0	3.31765	0.98715	0.014417
ENSG0000(	PDXP	22:376346	14.7685	16.8035	47.2508	36.5121	36.2089	21.3239	21.8908	18.8363	14.126	24.6611	0.116	-0.65892
ENSG0000(	TANK	2:1610962	91.8623	51.129	52.4145	71.0578	43.8148	134.139	131.343	115.812	117.623	51.532	0.01155	0.865924
ENSG0000(	AC144652	7:1552959	2.54796	1.10713	0.842165	0.93731	0.232569	0	0.408549	1.25101	0.598619	1.02383	0.69145	-0.66499
ENSG0000(	SYTL1	1:2734201	218.162	264.922	163.804	153.647	149.525	164.369	168.442	120.021	71.4644	102.118	0.0922	-0.5768
ENSG0000(	BIN1	2:1270480	106.435	121.052	241.04	147.004	214.332	66.8078	92.8522	145.894	94.081	104.918	0.02495	-0.74749
ENSG0000(	MGAT4B	5:1797975	16.0209	20.8445	11.0729	8.35219	16.07	19.2287	22.8081	11.3047	4.55824	17.4671	0.89115	0.100063
ENSG0000(	DHRS3	1:1256790	7.56234	11.4986	52.7768	48.3604	48.9783	9.97461	1.1158	21.3766	43.2371	29.8253	0.1153	-0.64653
ENSG0000(	GALNT4	12:894197	3.58439	1.81044	0.896722	1.52122	2.30702	2.69336	0.935107	1.08843	1.93221	7.22713	0.8693	0.17912
ENSG0000(	IL23R	1:6709216	67.1275	3.25971	29.5807	18.6515	15.2226	26.1355	4.0158	18.3073	13.2925	23.5502	0.08655	-0.6415
ENSG0000(	MEI1	22:416994	66.5907	58.8243	144.698	171.322	91.507	48.9654	68.0008	113.371	147.105	53.547	0.57015	-0.28524
ENSG0000(	RP11-57G	10:678495	0.879125	0	0.783473	0.908606	0	0	0	0.23762	0	0	0.12615	-3.37191
ENSG0000(	TMEM120	12:121712	4.46784	4.69099	14.6239	22.5532	5.43224	6.16823	3.59743	3.84863	25.7363	4.91526	0.95265	0.065247
ENSG0000(	LRRC58	3:1203245	3.83677	4.32594	1.79117	1.67829	2.59658	2.6782	3.44593	2.48422	4.85861	4.98294	0.39755	0.337986
ENSG0000(	JUP	17:417546	0.307227	2.19872	0.353314	0.459247	0.153077	1.87449	0.011716	0	0.947859	0.144954	0.89475	0.127208
ENSG0000(	KDM4C	9:6716494	30.333	30.1923	9.06759	17.3781	7.54972	17.2165	10.5368	13.8303	6.82729	13.177	0.07975	-0.71076
ENSG0000(	TMEM261	9:7796489	18.0596	11.414	29.9992	30.3625	15.0363	14.61	14.1076	21.418	15.3794	3.26744	0.0997	-0.70773
ENSG0000(	SCAMP4	19:190521	8.73727	13.0815	4.45249	5.11296	18.3107	14.3996	12.2703	6.95584	12.6729	20.3662	0.50865	0.410032
ENSG0000(	TRIM59	3:1602256	7.77267	7.43582	12.5165	12.0125	15.0202	9.42178	18.7588	22.3523	26.5176	31.6358	0.19605	0.869639
ENSG0000(	PEX7	6:1368225	2.93191	2.38208	5.15639	4.41107	2.59092	1.64961	4.21331	0.305766	0	1.04217	0.2501	-1.46353
ENSG0000(	PRCC	1:1567421	57.3788	62.6042	25.8228	26.9725	30.1715	34.9219	48.2481	25.6616	13.4905	38.8529	0.52435	-0.33982
ENSG0000(	ALG10	12:340222	1.78158	0.781747	2.24091	0.771319	1.58816	0	2.71728	2.29405	0	1.47794	0.7358	-0.40903
ENSG0000(	SRM	1:1105458	78.8349	85.0778	165.84	130.854	243.625	78.174	95.6161	117.87	92.3428	84.9763	0.07335	-0.62366
ENSG0000(	CSTB	21:437725	48.923	35.0564	52.0856	49.4113	49.4391	135.198	48.3266	56.8354	71.7095	18.2019	0.17965	0.471477
ENSG0000(	KIF5C	2:1488664	2.67104	0.179655	0.673063	1.1913	1.85087	5.30036	2.19645	1.19837	2.49925	3.53511	0.14875	1.04197
ENSG0000(	ZNF22	10:449976	55.8183	42.9061	70.7567	57.187	70.3306	27.1775	44.8303	50.1978	41.8975	40.8203	0.10775	-0.54824
ENSG0000(	ATP1B3	3:1418761	90.4214	75.8214	78.9731	79.206	73.8186	152.882	145.978	96.4872	117.359	40.1179	0.15755	0.438441
ENSG0000(	CCDC124	19:179330	91.5835	64.9515	122.145	112.84	117.336	104.868	100.484	97.0053	91.1542	33.1748	0.371	-0.33187
ENSG0000(	ZNF142	2:2186379	5.75598	4.53011	3.21254	3.64372	4.90536	3.75404	1.87681	6.45144	0.797991	9.83117	0.9009	-0.0803
ENSG0000(	GPBP1	5:5717394	131.969	120.408	59.2512	92.2098	58.2475	101.154	87.8326	72.304	79.0235	79.866	0.777	-0.09016
ENSG0000(	HDLBP	2:2412272	50.0877	52.7622	16.2058	20.089	24.5249	61.9667	33.5106	28.9433	19.0179	53.3024	0.6829	0.235532
ENSG0000(	UBR5	8:1022393	14.0536	7.00024	4.00589	6.25241	4.83285	9.22857	11.7007	9.43668	5.26608	10.9293	0.15045	0.834345
ENSG0000(	SETD7	4:1394959	3.78942	0.646216	3.80738	8.24187	3.14543	2.00396	0.416666	3.13034	7.20614	3.61892	0.7312	-0.36015
ENSG0000(	SLC22A17	14:233463	0.35449	2.01316	0.083499	0.137491	0.369756	0	0	0.373944	0	1.13295	0.53445	-0.7659
ENSG0000(	RP11-29G	13:755494	0.728192	1.14971	0.860039	1.53772	1.66129	0.181314	0.473379	0.113167	0.071467	2.86956	0.7176	-0.58897
ENSG0000(	SLPI	20:452522	0.182738	0.060459	0	0	0	1.12213	0	0.719815	0.795921	0	0.1894	3.67322
ENSG0000(	RTP4	3:1873683	16.8191	10.3902	29.4887	14.3851	19.091	30.0739	50.7038	23.5124	11.8769	8.40137	0.2941	0.431375
ENSG0000(	C3orf14	3:6156156	3.82142	9.58097	0.459542	2.67315	0.514041	25.8132	39.6597	13.4255	21.285	2.97707	0.0224	2.62451
ENSG0000(	GLB1L	2:2192297	0.64054	0.644617	1.10389	1.53326	0.041414	0	1.77342	0.784247	0	0.088249	0.7646	-0.36891
ENSG0000(	RNF144B	6:1838734	0.3766	2.29556	0.254544	0.812494	0.32132	1.82372	0.171818	1.11151	4.37023	0.95201	0.1596	1.15811
ENSG0000(	STX3	11:597550	2.50678	5.40684	1.51811	3.10702	2.27093	4.70917	0.066771	1.90229	3.289	1.87079	0.8597	-0.17307
ENSG0000(	ASGR2	17:710132	1.6078	2.1031	0	0	0	9.26831	0	0.209452	0	0.185464	0.44555	1.32585
ENSG0000(	ADA	20:445317	113.396	76.0495	137.955	141.968	113.401	166.296	81.0773	102.816	129.776	85.5274	0.86495	-0.05515
ENSG0000(	TMEM42	3:4476171	15.0005	17.071	29.8877	37.8795	21.2862	18.5776	17.0539	24.0486	15.7581	12.7873	0.3181	-0.49555
ENSG0000(	EIF4A2	3:1867817	299.606	271.68	261.915	291.437	188.774	303.644	237.624	229.255	224.801	203.625	0.7734	-0.10778
ENSG0000(	RP11-543I	1:2111734	3.00651	3.16074	12.1844	9.74542	3.53681	2.17303	3.46398	6.1826	5.07351	0.599145	0.6843	-0.79085
ENSG0000(	AC005301	22:166018	0.353981	1.65439	0.796039	1.52376	0.068557	0.105602	0	0.437559	0	0.129421	0.0779	-2.34521

ENSG0000	CEP112	17:655285	1.1906	0.255337	1.25986	0.641012	0.612125	5.89846	0.535864	2.34	2.4267	4.06083	0.00355	2.24879
ENSG0000	GRAP2	22:399010	35.8823	59.634	49.323	38.1905	41.9081	35.5478	92.5439	56.268	36.3551	63.0505	0.73335	0.142002
ENSG0000	MYCBP2-/	13:769906	5.6769	9.63824	6.22696	8.05647	10.8532	4.07379	11.9123	6.4032	8.55127	15.6005	0.996	0.005446
ENSG0000	VWA8	13:415668	3.69234	3.58331	1.04106	1.14611	1.3322	0.916118	1.1787	0.788831	4.08052	2.13075	0.7983	0.170281
ENSG0000	PTGES3P2	2:2573375	4.6029	2.32541	2.75909	2.06967	1.91877	7.35611	7.77535	7.79218	0	2.66368	0.54645	1.03971
ENSG0000	ZNF552	19:577696	3.84209	3.12052	4.326	3.81311	3.41611	2.92016	2.35796	5.03434	3.07611	5.73429	0.8143	0.222148
ENSG0000	GDAP2	1:1178634	6.81932	10.0202	5.95922	4.96343	5.15429	5.67917	6.68839	4.27343	5.18713	2.32385	0.2703	-0.61305
ENSG0000	CITF22-92	22:460136	0.082337	0	0.488563	1.14232	0.361422	0	0.589459	2.66565	0	0	0.7572	0.494457
ENSG0000	CIZ1	9:1281660	23.6512	25.8823	17.7007	12.7138	26.8199	17.5762	11.8153	18.2339	25.3279	30.6538	0.9438	0.025788
ENSG0000	CTD-3148	19:494462	2.42944	2.09451	0.726559	0.273087	0.348758	2.17919	0.138732	0.338451	0.387175	1.8967	0.93385	-0.14794
ENSG0000	HSD17B6	12:567124	0.435219	0.419449	0.518216	0	1.031	0.619855	1.21182	0.028345	0	0.404619	0.7973	-0.30514
ENSG0000	RP11-356	6:1376733	0	0	0.281015	0	0	0	2.17296	2.2925	2.10759	0.381332	0.19875	4.4559
ENSG0000	POM121	7:7287936	7.75606	12.856	7.56332	3.28803	12.7345	9.7093	7.07345	6.08391	2.26302	23.2158	0.99945	0.000359
ENSG0000	GALR2	17:760387	0.202719	0.573533	0.054508	0.584133	0.29835	0	5.45942	0.956147	0	0	0.45315	1.52368
ENSG0000	ABCG1	21:421996	19.4594	5.4153	19.5794	10.9936	8.06297	52.737	8.85214	19.7612	24.4108	52.0796	0.00235	1.22912
ENSG0000	MTMR6	13:252462	13.0574	9.94964	5.26182	7.93785	4.50404	6.488	5.84401	8.56211	11.9952	7.97515	0.9708	0.012928
ENSG0000	HIST2H2B	1:1498501	2.32278	1.58203	7.63276	4.76856	4.3338	1.00083	0.919365	5.18952	4.47319	5.23157	0.7895	-0.52335
ENSG0000	SRGAP2B	1:1446271	1.77556	3.80559	1.11982	0.709157	0.71365	2.22255	3.28775	1.98459	2.5522	2.30623	0.29295	0.936371
ENSG0000	ANKRD44	2:1969670	28.6503	37.1416	16.8386	27.9303	16.9318	25.7556	29.7762	21.792	28.327	30.4637	0.80995	0.110398
ENSG0000	HSD17B7	1:1627907	23.9356	18.0998	28.917	22.9876	18.9738	13.8754	25.4819	22.3418	32.2094	19.099	0.9987	0.000646
ENSG0000	DNM1L	12:326791	19.5435	11.0693	8.06653	16.6015	8.27033	10.1412	11.9347	16.5198	3.6618	15.9353	0.8823	-0.06181
ENSG0000	POLR2B	4:5696336	42.0873	38.9614	16.8429	16.5506	15.9634	28.1962	27.7573	24.2614	13.2313	33.4708	0.6994	0.146399
ENSG0000	GOLGA8B	15:345252	67.5328	97.0765	25.4025	46.3203	44.2357	106.893	92.7281	45.3224	57.3442	101.635	0.4857	0.556934
ENSG0000	MTRR	5:7830377	43.4956	28.8057	14.9789	20.6647	14.5131	32.921	26.2358	9.39871	28.0731	16.0143	0.8669	-0.05924
ENSG0000	TRAV34	14:222025	2.29818	2.2954	0.577996	0	0.25238	14.2493	5.12149	0	0	0.143414	0.3861	1.80289
ENSG0000	YAE1D1	7:3956637	12.6063	8.06721	19.8191	10.7749	12.8919	28.131	9.14681	16.2626	6.19857	4.70435	0.95775	-0.0296
ENSG0000	C19orf70	19:567494	61.8063	37.7595	113.795	130.183	63.7614	88.6774	87.2529	108.524	97.021	23.9209	0.91965	-0.07777
ENSG0000	FARS2	6:5261043	23.5198	23.0652	31.2722	36.4422	28.822	16.5868	22.6059	19.2113	68.0492	19.3891	0.87025	0.061325
ENSG0000	SPRED2	2:6531085	1.19247	0.192756	0.216846	0.367745	0.562103	2.32883	0.542709	1.1578	0.990728	2.80149	0.17145	1.4515
ENSG0000	C12orf29	12:879800	16.2422	9.47374	9.80005	7.60478	4.84057	5.46603	11.4318	5.8936	15.1043	5.03635	0.5386	-0.4052
ENSG0000	XAF1	17:675544	92.6464	89.9087	65.8421	78.8138	83.8095	125.378	238.024	82.0968	36.2796	133.417	0.35175	0.380788
ENSG0000	UBE2E3	2:1809672	26.1018	25.7992	24.4789	25.4348	37.0608	25.2307	21.2834	12.4796	25.7287	27.3214	0.3073	-0.38975
ENSG0000	TRPC3	4:1218790	0	0	0.060263	0	0.03353	0	1.35143	1.7306	0	0.851339	0.1462	5.74564
ENSG0000	MAEA	4:1289850	81.7715	72.9995	110.229	111.85	127.032	61.5624	55.2164	68.5628	71.6486	117.18	0.1131	-0.49379
ENSG0000	RP11-596	14:498611	8.8248	4.20681	12.5095	3.46326	6.70172	5.40991	1.78786	6.00241	12.9469	1.16654	0.8718	-0.33951
ENSG0000	AP001432	21:372085	2.31361	2.08485	0.672102	0.644766	0.441455	2.96172	2.59204	1.80653	2.85691	2.42988	0.31625	1.29616
ENSG0000	RABGGTB	1:7572434	13.7894	22.1442	12.9472	15.3637	9.68728	22.073	17.1618	21.4378	17.8482	17.8275	0.54965	0.406817
ENSG0000	CCNI2	5:1327474	1.91195	2.46303	1.63239	3.47054	2.32799	2.04033	3.02528	6.14491	0	1.58919	0.8057	0.261637
ENSG0000	UNC119	17:284557	34.5081	34.3639	31.5094	54.5992	28.7627	51.1857	25.4757	34.4134	50.5764	29.9466	0.9466	0.089559
ENSG0000	TRG-AS1	7:3834157	11.1395	5.00484	13.9424	16.2122	11.4273	16.056	43.7645	19.8532	26.331	18.9986	0.1346	1.16159
ENSG0000	QPCTL	19:456874	0.864101	0.529603	1.34965	2.26538	0.927724	0.707372	2.33444	2.48821	2.61312	1.11738	0.63535	0.515127
ENSG0000	MST1R	3:4988700	0.293225	0	0	0	0	0	2.37325	0.486449	0	0	0.00595	3.63189
ENSG0000	RPF2	6:1109820	13.7177	11.721	22.558	19.9896	15.1758	21.3678	22.7678	21.7272	8.22887	9.17052	0.931	-0.03897
ENSG0000	PPIH	1:4265842	34.4784	26.302	35.2216	35.1814	35.3152	31.1815	33.0077	24.8858	28.105	9.56938	0.37645	-0.40993
ENSG0000	ITPA	20:320886	66.1747	62.4093	68.0719	103.09	77.6989	55.2768	104.283	73.5566	83.0107	32.909	0.70895	-0.14215
ENSG0000	AC005562	17:305734	0.627965	0.790129	1.14251	0.121281	1.31912	0.52096	3.00024	1.37882	0.082071	4.69285	0.2565	1.32565
ENSG0000	ZNF653	19:114513	2.11903	1.51262	0.270414	0	0.652284	1.74748	1.94788	0.205591	0.354229	0.909635	0.8478	0.198014
ENSG0000	RP5-864K	1:3883819	0.570696	0.948535	0.917012	0.328501	0.611285	1.14375	2.9578	0.347784	2.99901	0.496234	0.24525	1.29877
ENSG0000	TMEM14E	3:1522438	0	0.10106	0.113233	0	0	0	1.95417	0	0	0.53146	0.3591	3.62683
ENSG0000	NKTR	3:4254796	40.444	75.8007	29.1229	25.5451	27.0248	43.8289	53.4393	29.3052	33.6804	88.9056	0.67135	0.182881
ENSG0000	YWHAQ	2:9583971	397.69	225.82	297.461	356.41	293.002	255.916	223.961	235.102	300.958	224.177	0.24745	-0.42798
ENSG0000	ANKRD13	1:7026058	15.0879	15.1322	8.46184	16.9982	6.40042	7.46128	5.63688	10.8984	21.5339	10.6169	0.7908	-0.10015
ENSG0000	RPL23AP8	22:507569	18.0993	10.1142	19.1842	20.6852	12.817	17.3739	10.2815	9.06862	14.3165	8.8319	0.4773	-0.49428
ENSG0000	TRMT61B	2:2875163	22.979	9.61553	17.313	14.301	16.8377	13.0713	8.93393	8.88677	5.91317	9.68688	0.4411	-0.78161
ENSG0000	PPP2CA	5:1341147	88.6056	72.7067	78.2007	53.8771	68.4156	70.9054	86.8157	63.6933	53.1045	58.8821	0.5605	-0.32951
ENSG0000	TRBV30	7:1428125	17.1085	40.9586	55.8163	15.6234	9.07773	47.7925	17.9991	22.4246	13.4883	7.20529	0.55385	-0.36137
ENSG0000	RP11-394	12:961943	0.338919	0.454162	0.082517	0.709817	0.170126	2.04567	0.508005	0.540364	1.54822	0	0.33215	1.3994
ENSG0000	KIF1C	17:499794	2.74244	2.00334	0.333846	0.107628	1.20715	4.60769	3.00452	1.23831	0.53949	2.75724	0.3077	0.664808
ENSG0000	LDOC1	X:1411757	0.659989	4.15579	0.804266	0.552422	2.63118	0	1.73322	0.635869	0	3.59028	0.7992	-0.52542
ENSG0000	TRIP13	5:795605-	4.32351	5.2546	0.372453	1.19817	0.548343	7.53509	2.50149	1.48243	4.53898	2.55996	0.644	0.751267
ENSG0000	MRPL54	19:376266	61.9308	56.4211	107.518	116.988	35.556	84.8562	130.126	132.778	124.03	9.30184	0.4635	0.302068
ENSG0000	LLPH	12:661165	37.3965	27.3073	35.5103	44.5375	29.6687	69.6208	47.2912	31.7919	36.8354	10.6696	0.92125	0.104432
ENSG0000	NSUN2	5:6599238	55.3545	66.844	38.1936	45.418	21.9073	41.23	42.8076	39.9303	36.4462	38.3173	0.82255	-0.07595
ENSG0000	SIDT1-AS	3:1135322	0.363217	0.460911	0.623623	0.208008	0	0	0.091437	0.170782	0	0.371581	0.60875	-0.91146
ENSG0000	CTD-2006	19:119876	12.6282	9.72183	18.8462	12.8254	6.3093	13.0857	16.375	9.41688	18.2735	5.00842	0.9979	-0.00192
ENSG0000	TCF4	18:552221	2.23393	5.40833	2.23499	3.6256	2.98895	1.6122	2.77735	0.136225	0.269312	0.987642	0.0007	-1.55661
ENSG0000	TESC	12:117038	19.6349	16.4167	14.1613	12.3004	11.6239	54.1802	87.5811	46.6838	14.397	24.7136	0.00295	1.48719
ENSG0000	AEBP1	7:4410436	0.848045	5.3199	0.070744	0.470167	0.498602	0	0.037624	1.13355	0	0.384206	0.194	-1.78606
ENSG0000	SATB1	3:1715716	77.9611	83.7451	90.399	73.102	61.1929	24.7013	23.1263	50.3773	82.271	66.9967	0.05375	-0.75405
ENSG0000	SPTLC2	14:775059	4.3614	3.77417	5.80134	6.81038	3.13627	2.91668	2.87062	5.54788	6.6359	6.83955	0.7527	-0.21103
ENSG0000	SGK223	8:8317735	0.624841	3.10551	0.30331	0.450549	0.742778	0.470886	0.614185	0.492828	0	2.29279	0.75155	-0.38945
ENSG0000	GIT1	17:295605	27.688	30.3989	5.92435	4.22142	9.03269	28.7404	27.3715	12.5084	6.29765	16.833	0.9089	0.135151
ENSG0000	MAN2A1	5:1096893	9.15621	11.9316	5.43833	8.52911	6.21775	5.51296	4.56049	4.99173	23.8755	9.82709	0.5463	0.258523
ENSG0000	RASGEF1A	10:431945	2.60387	0.234239	1.2883	1.30956	2.52526	2.35724	1.12414	3.1657	0	3.45377	0.91125	0.09196
ENSG0000	EBLN3P	9:3707985	12.8052	15.709	10.3699	16.8907	10.7611	11.3562	8.6762	9.42241	19.9389	22.8091	0.8818	0.051446
ENSG0000	VPS37C	11:611302	0.166111	0.522329	0.292746	0.185652	0.357241	0	0.617711	1.01539	0	0.890645	0.66035	0.646111
ENSG0000	MEFV	16:324202	0.570598	4.0785	0	0.107179	0.112297	1.23385	0.09467	0.135256	0	0	0.1457	-2.09987
ENSG0000	UBE2S	19:553853	36.8204	25.9456	24.6209	20.754	30.0465	45.3917	67.3123	30.9254	29.4716	15.7839	0.6207	0.28563
ENSG0000	TMED1	19:107180	29.0662	12.5404	57.5376	59.1185	51.9461	33.2953	36.2729	29.4436	32.8425	22.4119	0.50785	-0.55059
ENSG0000	CTNNBIP1	1:9848275	5.59212	3.1832	1.89664	1.63439	2.31944	7.59961	6.50206	2.33073	0.702006	1.54832	0.78985	0.249128
ENSG0000	TTC12	11:113314	3.75785	5.30706	2.80048	2.86772	2.18718	7.07952	2.44021	6.39589	7.0751	2.74729	0.21185	0.631248
ENSG0000	GPM6B	X:1377103	1.77018	3.9182	0.497154	0.392342	0.925816	4.58944	4.55124	1.19522	1.09363	1.92705	0.3252	0.771639
ENSG0000	HN1	17:751352	97.3043	95.5151	69.0367	77.069	68.1762	122.724	142.391	126.649	68.1884	48.1252	0.6024	0.293905
ENSG0000	TRBV11-1	7:1424076	0.845012	2.31757	1.04691	2.47374	1.33047	0	4.88663	0.566897	13.8868	1.11839	0.51675	1.52688
ENSG0000	KTN1-AS1	14:554992	0	0.649497	0.023957	0	0.315426	0.020866	0	0	4.17584	0	0.33975	2.68703
ENSG0000	METTL24	6:1101801	0.7423	0	0.775799	1.45688	0.082295	0	0	0.42631	0.799343	0.907276	0.7706	-0.49804
ENSG0000	ZFP36L1	14:687876	39.3187	62.1593	241.61	173.098	347.579	58.2126	39.1393	341.131	170.468	632.644	0.6296	0.254065
ENSG0000	HIF1A	14:616810	46.805	54.0728	27.8003	51.4665	20.8069	80.6175	80.7498	62.8511	73.9218	54.9922	0.0502	0.798892
ENSG0000	RNF7	3:1417382	39.1523	41.9105	66.6861	55.2436	40.4973	64.5712	70.6181	78.5546	51.7913	15.2755	0.8368	0.095879
ENSG0000	RP11-384	17:179728	0.425452	0.282332	0.55821	1.18419	0	0	0.916291	0.604397	0.380578	0.553105	0.94565	0.100784
ENSG0000	CHD7	8:6067877	23.8141	33.2434	12.1049	9.12012	11.2814	15.3098	8.53776	5.7627	16.3834	23.632	0.49385	-0.35696

ENSG0000	RAD51C	17:586925	22.3947	29.4818	39.8909	34.912	37.1153	36.9337	43.1364	33.7719	14.3634	21.5855	0.7158	-0.22204
ENSG0000	CLEC10A	17:707453	0.256183	5.22188	1.14479	0	0.062875	7.48613	0	1.49768	0	0.813964	0.65415	0.595455
ENSG0000	RNASEH2I	13:508621	68.3342	68.2688	56.4746	33.9121	40.5639	90.3916	71.7118	44.6408	20.8138	17.3324	0.57355	-0.20849
ENSG0000	RP11-379	3:1258272	0.562266	0.272637	0.292085	0.317077	0.185801	1.56221	1.29188	0.413001	0	0.202013	0.30515	0.56005
ENSG0000	ANKRD22	10:888221	0.356926	0.023392	0.299429	0.720027	1.04505	16.2356	1.46644	1.70938	0	0.700534	0.3255	3.19003
ENSG0000	FOXN3	14:891248	73.0003	97.9458	50.0292	27.5521	43.7851	54.5573	67.6711	37.1239	44.8663	68.7417	0.84265	-0.10759
ENSG0000	RABEP2	16:288784	9.48718	7.14519	6.47293	5.1685	4.53084	6.3086	11.6205	3.39661	2.02219	10.5797	0.9722	-0.0238
ENSG0000	BCORP1	Y:1945543	5.24014	8.1814	4.1681	2.39153	2.45897	1.11163	0.932628	2.5409	0.067261	5.00532	0.0405	-1.38126
ENSG0000	RP11-216	1:1537890	0.479589	0.771817	0	0	0.141235	0.479472	0.264922	0	1.35104	0.83333	0.52035	1.25697
ENSG0000	ATP1A1	1:1163726	20.1039	18.5804	56.6179	52.6848	46.0419	25.3113	19.2429	93.4468	54.3246	119.365	0.0985	0.606527
ENSG0000	NVL	1:2242196	19.4885	18.8742	15.9266	20.8874	13.719	33.3206	19.3657	14.4478	11.4845	18.2723	0.86385	0.065356
ENSG0000	TSFM	12:577623	32.2624	25.8341	48.1815	49.0165	39.9236	36.6155	40.402	24.6848	6.6729	17.1371	0.13435	-0.80919
ENSG0000	BATF2	11:649879	0.025045	0	0	0	0	4.75501	0.031939	0	0	0	0.3369	7.43074
ENSG0000	RP11-343	16:567087	0.424787	0.135915	0	0	0.45837	1.46202	0.354642	0.257599	2.32262	0.155632	0.25085	2.28007
ENSG0000	ZCCHC17	1:3129698	37.7397	28.5865	63.0779	50.5838	74.6482	32.82	43.5521	29.1129	37.7203	30.6601	0.09765	-0.59812
ENSG0000	RP11-473	16:306529	16.4307	28.1927	0.410387	8.48503	0.109165	7.12148	9.61327	0	0	0.334451	0.3287	-1.74793
ENSG0000	S100A13	1:1536187	0.972908	1.21033	1.41726	1.94005	0.44347	2.14246	1.04183	0	0	1.26571	0.72035	-0.41941
ENSG0000	DSCC1	8:1198339	1.84264	1.38	0.53627	0	0.337373	2.3429	4.5769	1.08582	0	0.781233	0.5806	1.2852
ENSG0000	SH3PXD2E	5:1723249	0	0.038256	0.093499	0	0.00568	1.93765	0	0.792697	0	0.069814	0.105	4.06324
ENSG0000	PSMD4	1:1512547	173.232	149.934	243.698	233.409	218.326	203.013	173.282	188.396	187.449	116.883	0.4319	-0.25198
ENSG0000	MAPK12	22:502451	0.280202	0.052226	0.17808	0	0.012048	3.16808	7.89204	1.24293	3.12842	0.849541	0.00035	4.51156
ENSG0000	DOK4	16:574281	2.63213	0.96018	0.949191	1.45032	1.78358	3.33527	1.32103	1.63896	6.767	1.74756	0.27785	0.99034
ENSG0000	FAM126B	2:2009089	7.00782	9.15512	4.20657	5.83652	2.77168	3.73836	8.3835	5.48908	2.24784	8.52941	0.69115	-0.29211
ENSG0000	RP5-839B	20:998608	0	0	0	0	0	11.1376	0	0	0	0.589377	0.1158	inf
ENSG0000	AFMID	17:781873	0.614912	1.33849	1.3418	2.38106	1.95543	0	0	0.628134	1.40439	0.422882	0.07445	-1.72822
ENSG0000	PPOX	1:1611664	27.0415	31.2409	37.4471	52.0721	20.7249	18.3049	26.8794	29.8149	49.8069	21.8893	0.76345	-0.14475
ENSG0000	MITD1	2:9899726	104.414	83.4044	68.9337	100.517	46.4871	109.599	85.4193	85.6628	80.7709	56.2865	0.8573	0.114011
ENSG0000	RP11-416.	1:3908131	0.203007	1.1643	0.892301	0.716166	0.701784	4.03577	0.894265	1.96991	0	2.5695	0.3682	1.36586
ENSG0000	RPS2P46	17:194178	4.58871	3.56543	13.6198	12.7724	6.64163	2.00481	3.07085	4.91381	6.146	0.589221	0.34435	-1.36449
ENSG0000	SAXO2	15:822628	0.329711	1.27856	0.379307	0	0.021197	0	0	0.108107	2.30477	0	0.68625	0.597156
ENSG0000	MLLT1	19:621037	1.91025	3.62359	1.17165	1.5496	4.62143	3.2745	6.71741	4.10309	2.06536	5.34706	0.28865	0.744317
ENSG0000	KIDINS220	2:8725277	13.2508	12.0375	4.09326	4.47054	4.27186	4.3704	6.1951	3.84689	17.362	11.376	0.34665	0.389129
ENSG0000	AP4E1	15:509086	2.72683	3.33529	0.800365	1.35412	1.16474	2.57981	1.1135	1.89902	3.54004	2.06346	0.67595	0.235567
ENSG0000	MT-ATP6	MT:8365-!	592.068	597.868	1339.33	1628.94	2818.51	1049.47	637.901	1673.99	2411.32	2149.96	0.88105	0.177555
ENSG0000	C6orf136	6:3064703	9.59332	14.0442	14.4578	11.7849	14.6631	9.33926	14.2359	8.17868	13.7082	15.2428	0.8354	-0.13314
ENSG0000	ZNF670	1:2469455	0.460621	2.96692	1.91432	0	0.337169	2.17415	1.87134	1.02403	0	1.40252	0.9248	0.117217
ENSG0000	RP1-101A	20:465578	1.35482	0.331627	0.244766	0	0.513528	0.949205	0	1.26189	0	0.771565	0.80375	0.438029
ENSG0000	KIAA0907	1:1559130	5.28031	8.71	4.41882	3.44253	3.12466	9.19445	10.1502	5.87469	4.83067	13.8564	0.20785	0.601744
ENSG0000	PKIA-AS1	8:7842610	0	0.905305	0	0.060796	0	0	0	0	2.63854	0.642858	0.4092	1.82585
ENSG0000	SPATA2	20:499033	2.45086	6.28357	0.5867	0.840602	1.21931	3.51	1.67448	0.8668	0.898272	1.87495	0.7389	-0.19592
ENSG0000	EP300	22:410917	12.2088	13.669	4.22398	2.15912	7.22483	10.0011	10.873	6.27496	10.0389	18.1396	0.2353	0.426331
ENSG0000	RP11-543	14:996045	0.189599	0.063822	0.287981	0.523074	0.114338	0.969775	1.22925	0	0	1.42941	0.424	1.50271
ENSG0000	LIPT1	2:9899726	15.2339	14.0259	25.8999	13.0413	17.7777	3.22581	8.92366	10.5984	19.7016	10.5458	0.4436	-0.63848
ENSG0000	TIMMDC1	3:1194985	67.2818	44.4339	83.4394	81.9279	47.9772	88.1372	62.5355	47.1748	80.9578	30.5352	0.5699	-0.19432
ENSG0000	MOSPD3	7:1006121	17.3204	16.985	42.9611	56.0814	47.6547	38.11	19.4999	43.0194	57.4241	45.0269	0.73865	0.131106
ENSG0000	IPMK	10:581915	2.41419	3.35077	0.516501	0.717299	0.695812	1.3661	0.899176	2.38543	1.36393	1.36563	0.9381	0.047036
ENSG0000	CTD-2410	5:1341147	0	0.137905	0	0	0.149293	1.31917	0	0.632527	0	0	0.4219	2.5436
ENSG0000	RP11-291	12:105533	1.36686	9.96235	2.68532	1.94166	0.742453	43.6097	86.5373	69.7531	3.91005	5.39929	0.00005	3.7076
ENSG0000	CSNK1D	17:822283	68.0691	72.6062	41.9204	36.7372	65.6028	69.7694	85.9467	54.8597	42.4958	84.1	0.66385	0.154879
ENSG0000	SMIM20	4:2574742	33.6066	16.8039	40.5025	35.5821	14.3997	25.0312	27.3884	38.5064	22.4622	5.25524	0.7958	-0.223
ENSG0000	HCN3	1:1552628	0.267398	2.0634	0.234594	0	0.189678	0.026781	0.443254	1.71941	2.13778	0.287872	0.4658	1.18955
ENSG0000	2-Sep	2:2412272	109.756	100.307	63.2442	90.2361	39.6884	111.825	105.252	97.1756	86.8697	76.1483	0.4235	0.267956
ENSG0000	ALS2	2:2017005	3.67502	3.05227	1.53762	2.00733	0.992654	5.99208	2.65377	1.21737	2.788	1.24877	0.71655	0.215045
ENSG0000	LIN7B	19:491143	4.42146	6.10715	10.5284	11.7	4.69629	11.6548	10.0027	8.98281	4.94255	4.18585	0.9668	0.027212
ENSG0000	FAM120A	9:9344649	44.7107	26.2263	29.7277	40.6351	27.2864	58.4969	28.2609	32.5454	57.779	30.6301	0.60005	0.289105
ENSG0000	C16orf95	16:870835	1.48034	1.69925	2.36786	2.45797	2.70799	0.636007	7.08893	1.01406	0	1.19356	0.6885	-0.4248
ENSG0000	SERPINB8	18:639493	5.10894	8.71116	7.74114	4.63707	8.57982	7.96264	0.513207	5.89514	10.8463	8.15082	0.8761	-0.08943
ENSG0000	ZSCAN32	16:336509	4.35042	5.51077	6.71987	5.78115	7.14141	11.0789	8.33835	2.57846	6.15821	9.1394	0.80435	0.157382
ENSG0000	TMX1	14:512401	52.9879	29.3995	33.1826	30.9497	19.2759	64.03	66.0077	29.6058	29.2509	22.5942	0.4241	0.286957
ENSG0000	SLK	10:103967	16.6152	12.2275	4.01249	3.89448	7.57282	15.8119	13.7077	3.60786	3.46235	16.0883	0.49745	0.246324
ENSG0000	APOL4	22:361891	0.013754	0	0	0	0	4.88215	0	0	0	0.537146	0.293	8.61074
ENSG0000	SGSM1	22:248061	0.73375	0.353232	0.203949	0.06228	0.388106	1.79026	0	0.059706	0	0.203548	0.45295	1.08141
ENSG0000	RASA4	7:1024824	20.6074	22.324	24.3384	13.6083	22.8424	15.4072	18.8402	18.346	13.2433	21.3143	0.44165	-0.52966
ENSG0000	SRR	17:205415	5.61634	4.6926	5.62993	4.1033	2.96187	8.38108	8.12845	6.32891	2.74656	2.77816	0.87695	0.183289
ENSG0000	CTD-2081	5:5388029	0.289018	0	0.087821	0	0.103819	0	0	0.317299	2.74778	0.244862	0.11385	2.94116
ENSG0000	S100A11	1:1519945	436.641	427.144	408.395	393.104	466.029	1115.76	792.465	818.609	580.018	207.355	0.04195	0.688935
ENSG0000	IL21R-AS1	16:274021	1.10983	0.748728	1.04464	3.37115	0.528125	0.251461	0.526971	0.760105	0.836494	2.32007	0.7693	-0.47842
ENSG0000	RP11-315	1:1459113	3.45636	2.21948	1.47822	4.01988	1.47698	1.29077	3.48545	0.92838	0	0.969501	0.2817	-1.03997
ENSG0000	UNC50	2:9860857	43.3444	37.9966	47.7628	46.9887	26.6687	44.2218	58.6104	52.5784	38.7897	25.6669	0.62965	0.202272
ENSG0000	AC139100	18:801479	0.076894	0.598738	0.328781	0	0.131559	2.48182	0	0.145662	7.11211	1.7747	0.1831	3.57878
ENSG0000	TRAV38-2	14:222025	7.1944	32.8385	1.84055	0	0.733104	32.2234	37.0822	8.23577	0.981994	2.18364	0.60155	1.00922
ENSG0000	OAZ2	15:646875	32.3832	37.578	46.4423	45.4627	34.478	46.7547	28.8419	26.8396	44.4062	25.7349	0.44275	-0.25569
ENSG0000	CPXM1	20:279406	0	2.05906	0.133062	0.262187	0.077487	2.42007	1.16949	0	0	0.02536	0.8016	0.493062
ENSG0000	PARL	3:1838293	48.0645	47.3341	64.2982	71.7635	49.6863	39.1067	49.7534	39.1269	47.4158	19.3465	0.11635	-0.53784
ENSG0000	SYF2	1:2522267	74.4624	85.7922	83.0997	80.4448	85.6706	117.689	105.504	66.2546	80.383	44.6431	0.9813	-0.00778
ENSG0000	HNRNPC	14:212091	706.414	495.777	729.252	560.735	635.901	720.715	604.111	628.726	527.893	403.362	0.7199	-0.13926
ENSG0000	RP11-21K	12:128793	0.49993	1.37918	0.582878	1.11591	0.494237	1.47381	1.23698	1.70727	0.029946	3.24062	0.6527	0.799007
ENSG0000	HIST1H2B	6:2781504	0.173603	0	0.989034	0.989148	0.25949	2.48601	0	0	0	0	0.8393	-0.27921
ENSG0000	RP3-465N	1:2524223	5.68679	4.47922	1.45585	4.81761	1.03864	0.766605	3.25305	2.24638	5.67327	3.73795	0.98125	0.033126
ENSG0000	SLC4A5	2:7419856	0.255806	1.97172	0	0.179363	0.156699	0.772514	0	0.76644	0.424818	0.069048	0.7147	-0.46614
ENSG0000	SH3BP5-A	3:1525418	4.29727	7.14427	2.72895	3.63306	2.05514	4.83118	5.09729	4.27865	0.547026	4.9446	0.84425	0.159131
ENSG0000	ATG3	3:1125325	73.3547	56.9743	68.4016	68.527	64.2147	77.6927	80.29	71.901	66.086	50.5712	0.8236	0.084091
ENSG0000	SCO2	22:505235	27.3116	26.2251	44.5017	31.7132	41.1972	33.8062	61.7012	40.8489	8.45201	24.4285	0.8201	-0.20446
ENSG0000	RBM42	19:356290	93.2158	85.0494	74.1688	60.4814	92.0419	82.1213	115.325	78.7175	53.5306	85.6892	0.90965	-0.03712
ENSG0000	RP5-1180	1:1111173	4.1431	3.92152	2.1776	1.18651	1.4641	1.60781	0.665008	2.78697	0	2.51873	0.901	0.18003
ENSG0000	HAX1	1:1542725	108.83	94.7495	114.071	101.845	105.614	101.563	126.124	114.901	45.8037	65.8796	0.4267	-0.26182
ENSG0000	GCFC2	2:7564678	17.4397	12.1798	15.4111	16.6254	9.80459	10.366	10.4427	10.1988	15.8111	11.9678	0.7462	-0.18653
ENSG0000	GPRIN1	5:1765425	0.745102	0.438573	0.335121	0	0.261934	0.137327	1.26637	0.903779	3.60145	0.203107	0.3301	2.02328
ENSG0000	ZNF549	19:575273	4.28081	2.52445	3.07246	2.39289	2.15236	1.25123	1.65241	1.18324	5.50238	6.04456	0.9132	0.086091
ENSG0000	UBE2Q2	15:758432	38.3207	32.9875	20.9352	28.7663	17.3919	31.6655	17.6171	19.4944	17.3014	16.0681	0.22755	-0.42205
ENSG0000	JOSD1	22:386855	5.10986	3.49134	6.42739	6.12982	8.07579	8.06109	8.22579	8.78293	5.42577	16.7573	0.30285	0.462358

ENSG0000C	KIAA1524	3:1085498	1.4334	3.9358	1.54501	1.58704	1.56634	0.06828	3.20754	4.72233	0	3.87097	0.9999	0.000182
ENSG0000C	BET1L	11:127203	29.9783	23.8433	29.4748	15.9975	29.5598	20.9013	19.3634	22.816	20.4127	37.8024	0.64485	-0.1515
ENSG0000C	RP11-134	1:2014835	0.030572	1.91789	1.03061	0.089513	0.921512	0	2.13955	0.230211	0	0.904991	0.84975	-0.33382
ENSG0000C	MYLK-AS1	3:1235855	1.04755	1.75231	3.66064	1.28769	0.887	4.75212	0.557629	0.136635	0	0.448751	0.6239	-0.67485
ENSG0000C	TSN	2:1217371	42.1883	43.3215	42.4742	35.4549	25.1675	44.2687	37.9867	30.9749	29.9552	25.9347	0.4051	-0.28637
ENSG0000C	PPP4R2	3:7288807	17.7241	14.2993	8.51539	11.832	12.2009	13.1757	17.8412	10.9838	8.98468	20.3327	0.9055	-0.07052
ENSG0000C	IGHV4-4	14:106011	0	33.4576	1.52888	1.53764	0.216256	0	0.413728	0.468196	0.733636	0.7109	0.25175	-3.44815
ENSG0000C	TGM5	15:432325	0	0	0	0	0	0	0	0	2.55708	0	0.1103	inf
ENSG0000C	P3H1	1:4274633	19.6563	23.5789	12.0627	17.4418	6.60205	21.8737	22.0847	15.9464	15.4435	13.8983	0.39615	0.325776
ENSG0000C	MNT	17:238405	1.46397	0.956737	0.722535	0.870408	1.72572	0.426728	2.25396	1.13028	0.48272	3.93762	0.83575	-0.15117
ENSG0000C	SLC22A5	5:1321848	2.56533	2.63235	1.80436	2.22881	1.9464	1.83989	0.606356	1.20496	0.64207	3.69513	0.4139	-0.69417
ENSG0000C	MTG1	10:133379	38.9261	43.3804	67.3548	86.6859	70.8336	48.5951	97.4353	61.756	84.4524	99.8796	0.53425	0.305383
ENSG0000C	STX16	20:586512	44.2672	50.0136	23.7308	17.85	16.813	33.6859	28.6109	48.9762	19.5926	60.2202	0.59765	0.204162
ENSG0000C	RDH14	2:1855472	30.7822	26.4182	33.5008	29.1266	34.4258	17.3428	36.2703	20.1391	13.2016	12.8221	0.12755	-0.62753
ENSG0000C	NAP1L4	11:294443	149.653	189.601	128.564	91.8376	127.913	140.358	177.866	126.069	80.5407	138.299	0.85335	-0.0527
ENSG0000C	NOL10	2:1057076	12.6924	11.8154	5.65444	3.90233	8.60692	12.6943	13.7639	11.464	6.80578	10.9528	0.51925	0.293307
ENSG0000C	EPB41L4A	5:1121424	0.750994	1.20416	0.06858	0.010111	4.27507	0.259193	0.191809	0.198542	0	3.91889	0.16045	-1.5899
ENSG0000C	EFTUD2	17:448463	40.1196	37.2354	27.6446	35.6353	17.7114	29.4107	18.3601	17.1463	16.1851	21.9708	0.0596	-0.63537
ENSG0000C	ATP2A2	12:110280	3.66856	5.30464	1.7443	1.83213	2.40331	2.44905	6.39532	2.80527	2.35279	6.83191	0.36455	0.436
ENSG0000C	NKD1	16:505483	0.012282	0.265998	0.022215	0.815534	0.716705	0	0.001894	0.644449	0.227579	4.37437	0.2699	1.78182
ENSG0000C	RPS8	1:4473981	319.405	313.525	771.186	469.787	307.13	305.024	331.703	580.677	372.583	79.5729	0.2432	-0.43676
ENSG0000C	MRPL34	19:172921	117.487	96.4558	160.125	133.764	95.2359	210.391	105.128	107.878	86.9146	19.7995	0.56445	-0.29341
ENSG0000C	KB-1732A	8:1028055	0.652035	0.259254	0	0	0.509089	0	0.228399	1.00034	0	2.09029	0.54335	1.08748
ENSG0000C	RP11-326	11:287304	6.7003	9.07921	4.66648	8.00965	4.35468	3.68051	6.39802	6.03116	7.44218	9.3895	0.8655	0.14124
ENSG0000C	FAAHP1	1:4643212	5.12177	0.054774	0.59607	7.38885	2.18066	0	1.90492	1.16078	2.31384	1.07551	0.2501	-1.12683
ENSG0000C	C5orf15	5:1339555	40.032	38.5806	26.0244	43.0456	16.1284	40.542	25.9152	25.2848	26.1959	12.0985	0.37375	-0.29266
ENSG0000C	RP11-334.	9:1313940	3.60494	6.13973	5.74637	3.38843	5.99207	12.4556	8.33904	5.4025	3.91435	10.5025	0.2882	1.15382
ENSG0000C	RP11-460	7:6503835	0.766149	0	1.43484	1.30773	0	0	0	0	0	0	0.17065	#NAME?
ENSG0000C	SLC38A6	14:607347	5.96609	13.2379	4.78039	5.9272	2.57404	4.50041	3.29797	3.8492	8.94404	4.1216	0.49065	-0.39387
ENSG0000C	ABCD1	X:1537248	2.70032	0.737845	2.14215	2.16313	4.56642	4.46098	1.58066	1.40312	1.29193	7.13685	0.62735	0.326331
ENSG0000C	SLC35A2	X:4890317	18.4762	10.488	16.2505	16.4757	12.9458	12.3469	20.1376	15.8192	13.0642	21.7821	0.756	-0.13079
ENSG0000C	FAM65A	16:675178	2.72417	3.48921	1.10999	3.22416	3.11129	4.3432	1.49514	2.83453	0	5.62897	0.41645	-0.4589
ENSG0000C	ZNF815P	7:5823159	5.97885	11.0337	8.10568	8.54973	3.85667	10.0866	6.88728	3.84033	7.01571	2.8664	0.6547	-0.27973
ENSG0000C	PPP1R14A	19:382440	0	6.38976	0.709566	7.59377	0.753782	4.21425	0	2.22738	0	0.347411	0.46635	-1.26174
ENSG0000C	NR1D2	3:2389165	18.223	7.89927	10.2673	5.66722	8.97149	1.88724	3.04655	11.9056	9.22377	13.9205	0.65255	-0.51222
ENSG0000C	RP11-294	15:217178	15.3849	8.92294	13.4639	10.0519	1.7673	38.1025	32.3522	62.3251	36.8968	9.79332	0.43215	1.72705
ENSG0000C	RP11-545	9:1287244	9.44209	8.26823	12.8547	13.049	3.62372	28.9376	13.0951	14.9638	2.99495	1.30532	0.8762	0.256784
ENSG0000C	DLGAP5	14:551481	8.26276	15.1386	0	0	0.0611	2.75878	8.14612	1.95453	2.07484	2.52288	0.9564	-0.05455
ENSG0000C	PXN	12:120201	56.9703	55.2913	34.0977	32.6953	50.7987	5.6307	7.65451	17.0865	12.4581	40.7484	0.00015	-1.56973
ENSG0000C	DNAJB6	7:1573353	195.02	150.143	191.54	150.471	128.331	224.904	212.357	153.792	212.881	81.4213	0.7726	0.094184
ENSG0000C	FAM175A	4:8345593	3.14826	3.19134	3.6384	2.18256	2.27494	5.26505	2.71359	0.524925	5.0238	2.36639	0.87145	0.122191
ENSG0000C	PI4KAP2	22:214729	11.361	16.508	4.97437	3.20332	6.27153	6.78928	16.4609	6.236	7.2909	12.404	0.68615	0.249394
ENSG0000C	FOSB	19:453796	65.8984	18.6437	10.123	10.6698	1.68253	3.13992	2.22431	10.7746	12.2772	58.7959	0.8008	-0.25423
ENSG0000C	DOC2GP	11:676126	9.39938	1.99817	2.79815	2.592	3.52057	3.81805	4.00076	0.892651	0	5.19185	0.65265	-0.6474
ENSG0000C	TMEM171	5:7312029	7.19669	0	22.436	28.654	19.0248	0	0	4.29979	2.27321	5.2345	0.0978	-2.75457
ENSG0000C	RBFOX2	22:357387	0.583595	0	1.06885	0	1.42255	1.87716	0	0.685363	0	1.9326	0.73105	0.359665
ENSG0000C	RP11-463.	14:518261	2.80874	2.40836	2.12544	3.47405	1.4899	1.29513	1.00939	2.38354	2.61654	0.570522	0.625	-0.57158
ENSG0000C	CALR	19:129385	75.2044	65.5206	59.0303	61.4811	56.6955	65.9556	87.7605	86.7324	58.6927	41.0318	0.89125	0.058795
ENSG0000C	RP11-394	5:1493713	0.575725	1.51125	0.540869	0	0	0.58068	1.68522	1.70915	0	0.084083	0.68035	0.739308
ENSG0000C	ECHDC2	1:5289590	34.3965	46.3379	34.8608	46.3454	29.9637	43.0276	38.8842	21.9548	31.7185	34.9745	0.62415	-0.19231
ENSG0000C	ARHGAP8	22:446685	0.515121	1.17457	0.401054	0.334488	0.137358	0	0.666383	0	0.41607	0.301386	0.5657	-0.81831
ENSG0000C	FMC1	7:1393394	24.4552	28.6031	45.8931	34.6686	13.0754	33.9138	24.7459	29.5894	22.1685	6.23705	0.7056	-0.38181
ENSG0000C	RNF166	16:886964	203.069	177.02	171.955	196.763	137.037	208.697	156.69	125.148	125.102	139.364	0.39215	-0.27243
ENSG0000C	ANKS6	9:9873132	8.95041	5.33732	0.820357	0.504692	1.20377	3.87119	4.59503	1.38042	0.761355	3.20492	0.64295	-0.44186
ENSG0000C	FBXL18	7:5431334	1.23377	0.995137	0.800402	0.729384	2.11237	0.257373	2.26068	2.15021	0	3.98831	0.7664	0.385665
ENSG0000C	ATP6V1H	8:5371555	27.9991	36.2989	21.5709	32.5275	19.0593	31.6229	27.2686	20.9481	28.9974	23.6833	0.89365	0.048899
ENSG0000C	AC007566	7:9244745	0.884837	1.35288	0	0.146135	0.910048	2.83632	1.34559	0.289205	0	0	0.878	0.262114
ENSG0000C	HIST1H1B	6:2786684	4.04745	3.86608	2.10813	0.125027	2.24179	11.0778	1.94736	7.63842	3.88837	0.956726	0.4526	1.05052
ENSG0000C	MGMT	10:129467	28.8206	24.4184	38.0207	36.5484	48.8729	37.7471	29.6121	20.9628	27.6105	20.0922	0.29275	-0.40252
ENSG0000C	RP11-203.	9:1278664	30.7757	16.5893	7.92236	14.6941	6.11989	55.1236	18.3549	9.23137	19.6747	33.3095	0.42715	0.7616
ENSG0000C	LINC0154	21:449328	1.46594	2.83773	2.28112	1.39442	0.919297	2.09826	3.06931	0.28761	2.01494	0.850783	0.9547	-0.0683
ENSG0000C	NECAP2	1:1644067	100.451	122.743	79.2873	91.8942	79.9743	98.3591	88.8915	57.1848	68.9835	54.9041	0.3472	-0.33594
ENSG0000C	PCBP2	12:534416	51.5562	61.3167	41.1803	25.4837	74.7196	59.615	68.072	59.2996	60.9073	87.1871	0.3977	0.356767
ENSG0000C	MRPS31	13:407291	51.0929	45.4615	51.7648	53.9551	43.24	43.3532	48.7625	34.1491	35.4534	24.932	0.26785	-0.38196
ENSG0000C	CHMP2A	19:585515	168.744	142.563	202.536	200.902	292.835	230.251	188.758	208.298	172.876	127.839	0.67715	-0.12951
ENSG0000C	RALBP1	18:947500	26.5735	28.1539	66.8053	58.6354	103.265	38.3128	37.192	41.2101	51.705	67.4705	0.42335	-0.40405
ENSG0000C	STAT5B	17:421991	39.7489	51.6757	17.3132	15.0473	17.3573	24.2122	22.2692	20.6017	28.1244	31.5417	0.6633	-0.14596
ENSG0000C	SAMM50	22:439237	57.0178	43.4196	55.577	55.5676	56.9443	37.3431	57.4949	34.6171	43.6858	23.4553	0.20585	-0.44926
ENSG0000C	HSPA9	5:1385548	102.005	92.623	71.1578	71.4035	70.4604	86.2398	119.329	92.6475	66.7656	91.0857	0.6262	0.152611
ENSG0000C	RP11-121.	7:3832606	3.56094	0.311583	0.888487	1.02464	1.07052	2.28507	1.08829	0.392412	0.042242	3.42077	0.8634	0.130023
ENSG0000C	SUCLA2	13:479364	35.6522	43.0838	25.1937	38.9458	21.6311	30.6371	23.9944	18.5271	25.4645	14.5793	0.11605	-0.5381
ENSG0000C	HSD17B7F	10:383563	4.05583	2.98057	7.07073	7.10615	3.2919	5.69792	4.81492	7.46489	3.21756	5.81777	0.88705	0.099485
ENSG0000C	RP3-423B.	1:3755624	1.1123	0.471199	0.288352	0.715719	0	0.650912	0.294542	0	0.371248	0	0.6578	-0.91003
ENSG0000C	RP13-890	17:451485	0	1.33847	0.193633	0.614029	0	0.736804	2.93174	0.276561	0	0.879285	0.6193	1.06284
ENSG0000C	SH2D1B	1:1623952	0	1.24775	1.28516	9.30594	2.40585	1.46303	5.73105	0.025305	0	0.547743	0.51135	-0.87496
ENSG0000C	POLA1	X:2469391	3.21433	3.05266	0.76763	1.19898	1.46643	2.10933	1.67166	1.25677	3.22106	2.20231	0.74425	0.201776
ENSG0000C	CH17-472	1:1201970	11.009	3.58575	29.1936	16.0637	28.6052	4.29244	5.99594	13.8297	2.89815	11.463	0.0711	-1.74524
ENSG0000C	ZNF540	19:374971	2.48292	2.66011	1.88031	1.69428	0.83901	1.78705	1.7825	1.85075	0	1.94531	0.614	-0.43484
ENSG0000C	SSR2	1:1560090	185.461	101.571	319.257	289.624	355.898	158.41	192.438	251.802	185.034	118.409	0.1225	-0.51997
ENSG0000C	UNG	12:109097	29.5588	19.6425	29.7363	26.3677	36.96	25.1261	24.3588	20.3562	11.8607	26.0614	0.1983	-0.48917
ENSG0000C	DTX2P1	7:7695983	1.96518	2.74271	0.706408	0.665749	0.681145	1.41846	3.41542	0.972563	1.19396	1.06699	0.8621	0.269726
ENSG0000C	PIGW	17:364866	3.10697	6.92011	4.48914	6.90601	2.53067	2.0954	7.06439	3.59493	6.37078	3.60072	0.9102	-0.11004
ENSG0000C	OSCP1	1:3641582	0.302849	0.154074	0	0	0.177658	3.23063	0	0	0	0	0.3115	2.06004
ENSG0000C	ZNF75A	16:326374	22.5224	44.032	32.1912	22.549	20.9814	17.325	25.996	21.7724	32.7134	39.1717	0.94495	-0.04469
ENSG0000C	RP11-442	1:2864322	0.789421	0.259966	0.378189	0.903458	0.456808	2.14207	0.074467	0.686114	0	0	0.93115	-0.14507
ENSG0000C	RP11-337	1:1471727	3.03527	3.81214	3.18054	2.80844	4.57283	5.14675	1.819	3.30454	1.65053	4.42446	0.9418	-0.12956
ENSG0000C	IFNAR1	21:333244	12.4004	16.6111	13.4161	11.5142	14.7307	22.3636	13.3792	11.8588	18.1284	12.5875	0.9444	-0.05834
ENSG0000C	RP5-1009	20:380835	1.25727	0	1.60024	1.45159	1.01294	0	0.914502	0	0	0.132789	0.2492	-2.51279
ENSG0000C	WNT10A	2:2188803	0.500071	1.28957	0.369137	0.976211	0.104996	3.97117	2.93891	2.04144	0	0.699333	0.40025	1.49858
ENSG0000C	SPRY1	4:1233967	16.6656	4.47626	21.6849	28.8128	18.7934	5.84756	19.5915	32.0559	15.549	29.0362	0.94285	-0.05794
ENSG0000C	GTF2F2	13:451205	18.015	15.645	14.5544	13.7432	14.9839	23.5203	26.2166	15.4113	10.4699	8.77573	0.7141	0.145255

ENSG0000C	RGPD2	2:8774808	5.18341	6.80693	0.339133	0.583957	0.31215	1.74801	9.57567	3.05937	5.56783	1.10014	0.6736	0.43066
ENSG0000C	KLHL6	3:1834875	3.1091	11.4204	0.655569	1.29612	0.517033	7.06331	5.45873	4.44671	5.2421	8.96621	0.0558	1.00265
ENSG0000C	UFC1	1:1611527	129.816	70.3991	145.47	157.708	134.391	139.761	102.764	116.451	123.008	39.8	0.32415	-0.31445
ENSG0000C	SOX13	1:2040731	1.98796	0.06439	0.521684	0.256192	1.86085	0.232218	0	0.953731	0	2.95563	0.80685	-0.23768
ENSG0000C	OTUD6B	8:9107019	4.41574	8.95628	3.36124	3.43886	2.00849	4.12535	8.78375	3.44519	1.59166	4.66496	0.8151	-0.14334
ENSG0000C	HPF1	4:1697294	26.5403	27.1451	41.9778	25.1335	56.8347	26.1753	31.8388	28.7796	21.928	25.0687	0.2814	-0.43169
ENSG0000C	ZDHHC20	6:2970812	1.59933	0.640594	0.802706	1.89399	0.417922	0	1.43815	0.605566	0	0.160232	0.4987	-1.1542
ENSG0000C	ADARB2	10:117731	1.04877	0	0.852773	1.11047	0.153942	6.27486	0	2.42582	16.7269	4.87562	0.00985	3.46007
ENSG0000C	JMJD8	16:680223	70.8761	53.6155	60.6696	53.3618	59.8332	55.5478	66.8248	43.1984	41.3049	50.7375	0.56815	-0.30922
ENSG0000C	DMKN	19:354972	2.96448	0	1.76869	2.66967	3.11596	0	0	2.33137	3.12696	1.44302	0.7058	-0.23666
ENSG0000C	RP11-454I	12:511869	0.22522	0.445508	2.06391	0	0.713183	0	0.651281	1.08046	0.076254	1.55372	0.96025	-0.10218
ENSG0000C	STK17A	7:4358275	115.756	101.376	90.6796	87.6562	69.9338	138.541	114.071	88.8873	93.5742	69.1304	0.7959	0.095439
ENSG0000C	MFSD9	2:1027158	6.72233	3.13163	6.15642	7.64654	3.19092	4.63849	3.01712	6.33306	1.08591	8.61876	0.3442	-0.51205
ENSG0000C	SETMAR	3:3700813	7.59779	14.7834	5.64088	4.96594	6.56205	18.2466	7.45397	6.06253	3.1529	2.79096	0.97335	-0.04114
ENSG0000C	AC092580	2:7725800	112.381	26.1546	379.397	224.089	51.7025	308.795	407.04	228.916	103.648	18.0355	0.4637	0.329487
ENSG0000C	JKAMP	14:594290	33.5307	34.0174	31.4502	47.1893	16.6059	26.8955	50.0686	33.8321	48.5156	15.0691	0.78175	0.113873
ENSG0000C	LHPP	10:124461	10.7771	9.07086	14.2999	12.287	23.1078	7.19505	10.5495	9.49644	9.66337	16.0323	0.641	-0.4166
ENSG0000C	MYL6	12:561507	1742.45	971.944	1776.77	1856.85	1428.25	2722.45	2374.26	2121.33	1795.03	389.58	0.61495	0.224271
ENSG0000C	ZNF548	19:573634	9.45802	11.4776	4.77084	5.48824	3.37508	2.89277	1.85836	5.26803	4.77108	7.11726	0.17205	-0.92489
ENSG0000C	ATP8B3	19:178207	1.60718	4.2114	1.05221	0.790865	0.741563	2.26701	1.31262	0.935993	2.09624	2.76588	0.80295	0.220574
ENSG0000C	SGSM3	22:403464	110.606	144.64	49.7087	54.2318	43.5101	92.1273	63.4955	39.0219	24.4693	43.0307	0.23635	-0.55321
ENSG0000C	RP4-635E	1:1101234	3.32421	5.34238	8.60226	6.5654	4.98585	0.81977	4.67385	16.7955	18.7288	25.1123	0.29165	1.2642
ENSG0000C	ASGR1	17:717343	0.982114	4.22739	0.937313	0.576326	0.191493	12.9459	0.050734	1.26784	0	0.443713	0.2818	1.15043
ENSG0000C	PRDM1	6:1060454	21.0953	19.3767	23.927	51.9001	51.225	15.9006	32.5741	30.5718	36.1417	44.6914	0.53845	-0.30004
ENSG0000C	C20orf194	20:324930	0.704156	0.586493	0.546368	0.331134	0.60388	0.121521	2.4334	1.42094	0.401458	2.58478	0.09095	1.15399
ENSG0000C	ZBED6CL	7:1502439	2.8306	3.89751	2.22842	0.608167	2.21279	3.33755	4.8671	4.33059	0.038336	4.65488	0.68665	0.493251
ENSG0000C	MAMSTR	19:487127	0.611714	0.504619	1.10166	0.633819	0.358993	1.62085	0.881614	1.07847	3.79226	1.65535	0.0965	1.67697
ENSG0000C	PGAM5	12:132687	6.60062	12.6013	8.54494	7.36262	6.53162	3.21516	13.4394	5.7071	6.63655	8.01661	0.58965	-0.30295
ENSG0000C	IGLV1-40	22:224097	0	1.01786	1.90966	12.884	2.71984	0	0	0	0	0.472672	0.2419	-4.62373
ENSG0000C	OSER1	20:441964	30.7522	26.1289	33.0337	37.2404	19.9698	29.5923	44.1725	30.6293	17.0938	9.4667	0.6098	-0.19722
ENSG0000C	PIGQ	16:566994	14.902	20.5423	9.08362	9.62939	12.2494	6.02783	12.1325	9.3317	14.4152	17.6563	0.9626	-0.01928
ENSG0000C	RPL22	1:6181268	90.4979	107.109	155.449	92.9529	109.031	107.692	90.2746	110.529	72.3011	35.6748	0.3076	-0.41013
ENSG0000C	DFFB	1:3857266	5.12769	4.12723	5.4441	4.54365	3.1016	6.67741	3.98738	1.89328	0.085023	5.1372	0.5829	-0.31598
ENSG0000C	PLCB2-AS	15:402781	0.12732	0.186542	0.126916	0	0.11449	0	0.160841	0.140018	0.243785	0.303315	0.55115	1.03046
ENSG0000C	BSCL2	11:626902	32.599	32.6552	31.342	18.5578	30.6756	64.6757	26.1602	21.3775	20.9709	31.8482	0.67975	0.18027
ENSG0000C	CMTM3	16:666038	111.986	66.7728	96.8829	108.525	122.493	133.033	124.907	98.4998	124.035	107.271	0.7667	0.087458
ENSG0000C	RP11-535.	11:758034	0	0	0.344989	0	0	0	0	0.138144	0.723465	0	0.22275	2.31663
ENSG0000C	LIN37	19:357180	23.8259	24.1737	36.8001	41.8553	47.2758	27.6469	24.9163	35.6636	27.4863	27.2044	0.7601	-0.26042
ENSG0000C	AC007349	7:3678100	0.222732	0	0.653916	2.23021	0.223353	0	0	0.045082	3.10148	0	0.9895	0.000981
ENSG0000C	MPHOSPH	16:821477	47.7781	29.415	59.7796	61.992	31.4571	50.1403	40.7108	55.3244	55.5094	18.2751	0.9121	-0.03899
ENSG0000C	CDKN1A	6:3667645	2.52272	8.73845	2.71044	27.3461	2.818	58.7757	28.848	22.7318	38.2313	8.39716	0.00255	1.76317
ENSG0000C	PARP8	5:5066589	74.8854	37.7494	48.4604	59.8477	40.8234	24.7192	43.323	48.9278	41.8173	95.886	0.3103	-0.31792
ENSG0000C	RP11-378.	1:2224772	1.61088	2.09852	1.62995	1.51543	0.357537	1.10329	0.210225	0.116714	0	0.82415	0.07035	-2.34744
ENSG0000C	SSH3	11:673034	2.56517	2.69207	2.08328	2.434	2.90416	2.63655	1.55964	1.02624	0.844198	2.05567	0.2227	-0.96812
ENSG0000C	AC017104	2:2313889	4.23555	1.9253	1.05155	1.65766	1.58837	3.18572	9.30076	0.7949	0	0.764681	0.8907	0.154557
ENSG0000C	COX6C	8:9901326	501.393	346.858	522.338	450.971	122.084	686.525	678.904	415.527	406.101	48.7422	0.69255	0.215843
ENSG0000C	TNFRSF10	8:2318927	9.16182	10.3649	34.8615	30.2032	22.6188	7.52774	10.5518	20.7677	19.1484	32.28	0.388	-0.33506
ENSG0000C	MLKL	16:746718	21.0437	29.3685	13.0861	17.2411	13.401	27.8717	37.9042	14.0361	10.8454	14.7595	0.67275	0.170874
ENSG0000C	RPS20	8:5606729	404.824	400.117	937.712	553.67	232.038	497.721	508.549	702.607	470.789	66.7948	0.5724	-0.21036
ENSG0000C	RPPH1	14:203430	3.67655	3.01575	15.6252	0.733259	1.7585	2.57705	0	1.38537	0	0.380849	0.18955	-2.51368
ENSG0000C	SPRYD7	13:499127	2.10203	0.932468	1.84072	1.8755	2.33646	2.6109	1.92615	3.22952	2.09241	1.79197	0.6399	0.256344
ENSG0000C	RP11-226I	13:769906	1.91248	5.50336	0.187783	0.572584	0.394253	0	1.52684	0.822599	0.916145	0.92704	0.787	-0.34499
ENSG0000C	CYB561D2	3:5034732	49.8136	47.4246	90.9718	101.041	64.3483	68.1608	62.0226	82.3889	85.2766	49.6408	0.97105	-0.02363
ENSG0000C	UBE2B	5:1343711	40.6038	50.7606	59.3799	54.5498	34.1187	46.7025	81.515	60.1682	77.623	20.4427	0.4996	0.311734
ENSG0000C	RORC	1:1518002	80.0572	4.44008	49.4463	95.1882	42.2466	37.9723	8.133	32.9582	47.9917	46.1347	0.08545	-0.642
ENSG0000C	CD36	7:8031257	3.44674	22.5167	1.04063	0.206711	0.316658	33.79	0.26712	0.560657	7.52958	0.319873	0.26605	0.856345
ENSG0000C	ZNF280C	X:1302027	0.963472	1.59128	1.31632	0.680833	0.446735	1.43275	1.45333	1.03677	0.473912	1.75131	0.82835	0.193382
ENSG0000C	RORA-AS	15:604791	1.71748	1.07673	0.132271	0.466357	0	0.255582	1.83604	0.422395	0.213185	0.235981	0.9709	-0.04457
ENSG0000C	PEX16	11:459096	37.756	31.2202	62.2948	55.0543	64.5836	28.9501	34.126	45.2895	42.9553	28.1411	0.16765	-0.48439
ENSG0000C	RP5-1050I	17:496799	0.61475	0.193447	0	0.198028	0.167936	1.78469	0.812956	0	0.092268	0.025909	0.6346	0.961004
ENSG0000C	B4GAT1	11:663453	5.81458	7.04756	11.0629	8.73543	10.7098	2.03966	5.18523	3.88435	0	4.24487	0.0431	-1.56332
ENSG0000C	NSA2	5:7462740	16.3163	21.441	21.6139	19.0603	19.8703	13.9882	27.872	19.2926	14.8728	8.71996	0.6906	-0.36327
ENSG0000C	RP11-386	12:491277	56.5995	45.451	20.3502	34.7437	13.7503	83.5827	37.3347	18.4319	16.4432	6.67682	0.8163	0.21556
ENSG0000C	AF146191.	4:1897803	0.586909	1.05431	0.75762	0.6507	0.443518	0	0	0.244486	0	0.517644	0.21875	-2.06662
ENSG0000C	CH17-270	1:1459113	0.639989	0.254225	0.26828	0.43292	0	0	0	0.392297	0	0	0.49485	-1.50899
ENSG0000C	TRBV11-3	7:1425548	1.53541	1.72071	5.73974	1.05636	2.68946	0	4.0375	6.87448	0	2.53775	0.93825	0.136806
ENSG0000C	LINC0032	13:234665	0	0	0	0	0	0	0	0	6.6832	0	0.11285	inf
ENSG0000C	PTGDR	14:522677	24.2944	5.57736	19.4656	19.1463	12.5046	5.75881	3.78539	10.3559	5.22937	12.1396	0.03575	-1.28284
ENSG0000C	TLE1	9:8158368	80.4922	5.41356	55.4752	99.2319	56.7877	7.87342	1.01594	32.7028	43.1466	44.4676	0.0086	-1.17822
ENSG0000C	RP11-73E	14:347527	3.53722	2.76907	2.15166	5.77277	3.27214	0.387402	4.48512	1.62513	1.85861	3.22417	0.69535	-0.61752
ENSG0000C	DIABLO	12:122203	41.6356	50.0822	75.2498	68.686	58.2697	54.985	70.0371	58.5003	62.7452	26.0036	0.85005	-0.09471
ENSG0000C	PHF12	17:289052	8.54012	9.7997	10.159	6.97451	11.2636	3.31305	8.94276	6.57005	3.87089	20.1679	0.7409	-0.13797
ENSG0000C	EHBP1	2:6267385	7.39343	7.90134	5.07544	10.0145	5.32575	8.68841	6.53739	4.15132	9.23793	10.9239	0.93685	-0.04959
ENSG0000C	EIF3A	10:119029	42.2294	41.7863	19.6367	12.3643	40.6532	42.4079	40.0555	26.8362	18.1115	76.0853	0.7607	0.105327
ENSG0000C	RPRD1B	20:380335	6.63108	9.46024	3.44957	2.02529	5.0148	9.99386	6.14887	5.24788	5.23449	6.5633	0.8404	0.09088
ENSG0000C	FDFT1	8:1179557	120.34	122.932	124.957	116.2	118.209	89.5501	138.623	157.354	163.189	70.2843	0.92335	0.030209
ENSG0000C	CTD-2647	8:2906727	0.517395	1.60122	0	0.492176	0.091355	0	0.386877	0.779692	0.093844	0	0.85085	-0.28667
ENSG0000C	RNF144A-	2:6912276	0.245355	0.457145	0.464757	0.544189	0.469019	0.430168	0.342674	0	0	0.528479	0.46745	-0.91677
ENSG0000C	BEX5	X:1021537	2.27618	0.382516	5.31225	3.38619	8.04733	0	1.2096	2.69034	0	1.9061	0.17315	-1.79649
ENSG0000C	MINK1	17:483338	14.637	21.994	2.03877	1.69471	3.02951	9.74891	10.3088	4.55133	1.14603	6.32723	0.41365	-0.43232
ENSG0000C	FAM134B	5:1647303	12.4204	51.2861	10.7844	21.2647	16.0424	7.93248	24.6203	16.4526	32.1736	33.2346	0.914	0.056973
ENSG0000C	MAP3K3	17:636224	25.8632	31.3583	8.86744	4.94478	9.73053	14.807	10.3788	10.0142	4.02721	15.3897	0.4749	-0.70283
ENSG0000C	POLR2J3	7:1024824	71.6791	102.162	120.703	103.844	76.0664	91.5236	83.0033	114.447	80.1544	64.5539	0.5862	-0.25125
ENSG0000C	NRBP1	2:2742778	68.5553	75.0963	59.0174	52.3811	65.455	79.5542	83.3139	63.7272	48.2057	47.4053	0.9109	-0.03429
ENSG0000C	CLMN	14:951819	0.06675	1.79543	0.002868	0.600512	0.368622	5.9072	0.02752	0.082794	0	0.1134	0.54435	0.833026
ENSG0000C	HPS5	11:182786	11.0149	7.034	4.07885	8.60347	3.69224	3.47765	6.16767	9.61226	4.15438	5.51473	0.9033	-0.05402
ENSG0000C	SLC9A9	3:1432652	21.0333	23.4027	13.0127	16.4756	5.6332	20.8592	14.093	22.119	12.5463	11.2686	0.94735	0.047006
ENSG0000C	CENPE	4:1031058	3.83019	4.39973	0.231328	0.157331	0.425462	4.32064	6.66497	0.862102	1.35589	4.01072	0.2968	0.756859
ENSG0000C	CUTA	6:3341039	216.908	186.708	301.179	318.528	366.848	272.839	221.372	272.616	185.689	113.487	0.27775	-0.46098
ENSG0000C	CACNB1	17:391732	3.69905	4.72343	2.14879	2.41209	2.33555	3.84623	7.46731	4.5234	0.593004	3.77286	0.6624	0.226212
ENSG0000C	SND1-IT1	7:1276521	0.673824	1.64084	0	0.442337	0.132379	0.402787	0.696114	0.991059	0	1.02513	0.926	0.156965

ENSG0000	GSK3B	3:1198213	5.06882	5.08438	2.51878	1.56646	3.69115	3.50624	3.12713	2.33427	3.25127	8.85388	0.8495	0.109567
ENSG0000	POMK	8:4309350	2.11261	3.44727	0.331578	0.514489	0.723509	2.30717	0.967965	0.067206	0.687886	1.41387	0.80415	-0.37209
ENSG0000	KLRB1	12:959455	384.325	19.5525	621.27	448.634	304.443	166.421	34.465	364.774	433.695	66.4751	0.13575	-0.75911
ENSG0000	SOX4	6:2152873	0.795395	6.22815	0.694628	0.726356	0.079723	4.45508	3.06401	1.18067	2.74418	0.938904	0.35095	0.697393
ENSG0000	TMEM39B	1:3201382	0.479755	0.944546	11.2888	14.3735	15.7387	1.88241	1.76715	11.7445	9.42794	15.6819	0.91355	-0.13708
ENSG0000	DNAJC24	11:313698	4.74774	5.53624	4.48994	5.56243	1.93989	5.08813	3.72756	5.33184	5.74313	2.99688	0.9498	-0.03692
ENSG0000	HIST1H1C	6:2605578	96.7649	43.6833	58.3022	78.2875	51.2477	77.7759	64.1012	15.8548	38.7951	5.85538	0.0732	-0.80677
ENSG0000	ROMO1	20:356260	162.261	98.872	230.748	197.115	69.6294	262.954	252.033	201.46	143.739	27.3837	0.9564	0.064143
ENSG0000	HERPUD1	16:569320	68.7554	79.2038	114.667	152.299	86.9055	111.284	150.165	121.285	115.698	79.4415	0.9793	0.008789
ENSG0000	ZNF568	19:369163	5.99296	4.01062	6.09706	6.62225	5.40798	5.5145	3.66142	4.38822	3.79175	4.09104	0.41975	-0.45754
ENSG0000	CCDC88C	14:912713	37.2882	43.7105	17.1326	11.0734	20.5906	37.286	26.8761	21.7318	8.92833	36.2532	0.90295	0.057081
ENSG0000	TNRC18	7:5274368	1.86211	2.25333	0.565416	0.517838	1.64144	3.36074	2.35406	0.855212	0.679552	2.35056	0.2598	0.573929
ENSG0000	RIN1	11:663302	0.657535	1.27028	0	1.08667	0.777797	4.58222	0.393565	1.12113	0	0.854094	0.47465	0.967433
ENSG0000	RP11-407	14:392302	2.31379	1.87366	1.41114	2.19913	0.328719	0	1.18656	1.16549	0	0.3315	0.36345	-1.55114
ENSG0000	KCNK5	6:3918897	1.03313	0.54191	0.172041	0.260583	0.046573	0.08362	1.5315	1.55682	0	3.24079	0.35015	1.56884
ENSG0000	HCG4P5	6:2994125	8.95926	2.27561	0.121479	0.465655	0.309144	2.28095	1.32286	0.148783	0.077333	0.324647	0.42485	-1.69278
ENSG0000	PSMD1	2:2310568	44.2895	42.6801	46.8015	46.5187	28.7261	53.4751	36.6022	27.5163	36.4441	45.792	0.72925	-0.11515
ENSG0000	AMIGO1	1:1095041	3.30807	7.2768	2.29146	0.768612	3.84998	3.41366	0	0.316212	0	2.82692	0.184	-1.53668
ENSG0000	TPRA1	3:1275730	27.3986	23.3098	28.9357	30.4784	15.3627	40.5363	25.5705	24.5468	7.79052	24.3059	0.75655	-0.13447
ENSG0000	RUFY4	2:2180349	0.191028	0.867531	0.029653	0.384123	0.064005	3.91415	0	0.394501	0	0.342845	0.15875	1.64093
ENSG0000	MCRIP2	16:636816	24.7378	16.2068	27.9468	29.2653	31.1196	22.9313	23.9515	29.0409	24.2456	3.52678	0.42195	-0.44761
ENSG0000	ARPC5L	9:1248621	123.854	76.2554	125.127	149.905	173.987	113.681	111.499	123.235	108.875	94.108	0.44875	-0.25805
ENSG0000	LSMEM1	7:1124229	1.03717	0	2.08731	1.41698	0.183549	1.35935	0	0	0	0.712586	0.438	-1.32455
ENSG0000	RGPD5	2:1097927	2.92999	2.19361	0.994183	1.47114	0.835025	3.29311	2.47937	1.76813	1.93298	3.01797	0.00025	2.72043
ENSG0000	AC006449	17:387024	1.53866	2.10095	2.54786	0.784123	1.21527	1.84426	2.43728	0.695721	2.82721	0.225484	0.5476	2.60124
ENSG0000	TAF6L	11:627711	22.4092	19.1057	12.7864	11.6638	10.1406	17.1886	16.5462	11.1963	5.61507	8.51393	0.656	-0.45112
ENSG0000	SERPING1	11:575973	0.446361	3.37744	0.412089	0.125586	0.248295	53.29	5.47289	0.657245	0	3.93099	0.00655	3.60771
ENSG0000	NRN1	6:5997998	0.325303	0.541329	3.04979	1.70016	0.569712	0.040523	0	0.132069	0	0.035542	0.06145	-5.45026
ENSG0000	IGKV1-33	2:8926649	0	10.6589	0	1.14748	5.8873	0	0	0	0	1.6236	0.3984	-1.56835
ENSG0000	ZNF790	19:367975	3.95476	5.08683	3.2534	3.24717	3.00671	1.94058	2.66261	1.43056	1.63576	3.35651	0.342	-0.69956
ENSG0000	BTF3L4	1:5202013	36.817	40.2065	38.4017	47.5288	27.5141	24.2426	25.3068	26.6379	35.148	25.132	0.41585	-0.47996
ENSG0000	MIB2	1:1613757	42.3252	39.4814	37.6522	47.1077	49.9434	28.9611	29.2763	29.2971	25.5902	19.8861	0.20685	-0.75003
ENSG0000	FANCM	14:451359	1.94551	4.50092	3.07353	2.89762	1.59556	3.23967	3.46663	0.846633	0	5.80798	0.4582	-0.60673
ENSG0000	HCAR3	12:122687	0.584375	1.31417	0	0	0.220686	2.78912	0	0.642165	0	0	0.77405	0.685394
ENSG0000	THAP5	7:1084704	3.96012	2.13327	22.0292	11.946	14.063	6.90905	5.0272	24.0066	14.818	15.5078	0.8891	0.115059
ENSG0000	TMEM63A	1:2258100	48.18	71.7207	17.8353	14.3315	15.2651	5.69176	12.8468	6.07335	6.09056	15.9399	0.00005	-1.88188
ENSG0000	RP11-34F	13:499825	1.67257	0.088996	0.06435	0.276821	0.020402	0	0.01911	0	0	0	0.2345	-6.98281
ENSG0000	LDB1	10:102107	15.9661	17.5054	12.4267	6.80422	22.8543	20.8083	24.4785	11.6124	6.01865	34.1511	0.5902	0.230098
ENSG0000	TRIP4	15:643877	30.3744	18.296	30.8104	29.6718	21.1699	15.8702	27	18.4779	13.3601	27.3961	0.25625	-0.40651
ENSG0000	RP11-421	1:1009896	10.6178	22.2039	12.5717	10.2255	2.73575	2.76437	4.85257	1.05137	7.93569	3.53153	0.181	-1.47082
ENSG0000	GTF2E1	3:1206866	9.23909	10.2655	8.78388	6.88578	5.95325	5.10276	2.56471	8.62936	7.54077	8.06892	0.6101	-0.33184
ENSG0000	TMEM19	12:716094	6.57745	7.17613	6.24217	9.1126	5.00044	6.38404	9.95515	6.24287	6.00876	3.32838	0.74255	-0.24718
ENSG0000	RP11-148	8:1149400	2.32221	0.375464	0.382292	1.94945	0	2.71186	0	0.420028	4.15006	0.938173	0.5417	0.931987
ENSG0000	ZNF429	19:213971	7.22129	13.5258	10.8891	17.5196	9.18699	1.27075	9.38439	4.84226	4.42681	10.8495	0.1582	-1.03377
ENSG0000	DDX11	12:310207	4.75018	10.73	0.137806	1.11752	1.12618	10.2302	11.808	1.68827	0.217216	4.28657	0.32825	0.809497
ENSG0000	PPARGC1	5:1497302	0.564365	1.70122	0.5881	0.389402	0.434994	1.14925	0.771587	0.54821	0.304641	1.69154	0.905	0.145763
ENSG0000	CASP4	11:104942	133.429	66.5262	131.088	148.032	135.719	143.965	107.199	127.449	108.257	71.5352	0.6049	-0.17027
ENSG0000	SIAH1	16:481649	9.53126	16.2667	8.21511	12.1568	9.0926	26.5154	14.8474	11.7221	10.4963	8.08875	0.5651	0.347706
ENSG0000	IRF7	11:612552	56.6865	74.4729	51.7522	53.1245	45.9862	79.1237	283.849	149.632	69.6399	86.2486	0.00195	1.14773
ENSG0000	RP11-382	8:2244081	0.725093	0.348899	0.397914	0	0.277264	0	0	0.297458	0	0.681454	0.8962	-0.2203
ENSG0000	CCND2-A	12:424876	0.378147	0.727552	1.24316	0	0.093354	0	0.99871	0	0	0.119658	0.6327	-1.26861
ENSG0000	EHBP1L1	11:655760	15.141	11.2153	2.54514	2.3858	6.62118	14.7889	15.9547	8.98663	4.38546	11.6682	0.3626	0.663096
ENSG0000	ZC3H11A	1:2037956	54.7197	70.9496	12.6446	15.0307	16.4281	36.761	25.2078	18.7582	23.7141	31.9921	0.5967	-0.18495
ENSG0000	RP1-47M	6:1076972	1.25738	3.17135	0.422899	0.534211	0.859659	0	3.22421	1.0915	1.08594	4.3556	0.5006	0.574674
ENSG0000	DENND2D	1:1111871	264.77	248.679	187.543	185.426	238.541	172.026	98.615	110.284	135.12	189.215	0.12515	-0.60322
ENSG0000	RP11-95D	2:9936359	0.424257	0.019479	0.736223	0.407583	0.72685	0	0.375492	1.69781	1.6756	1.06226	0.2138	0.959424
ENSG0000	GNPNAT1	14:527751	3.72294	2.94927	2.95872	3.00104	2.43577	1.39881	2.44775	2.8392	0.611879	1.51766	0.11755	-1.07316
ENSG0000	ARNTL	11:132766	49.8034	21.5794	20.2095	23.7813	21.9321	37.0019	27.1476	23.5958	29.095	36.0286	0.5307	0.201821
ENSG0000	RASGRP1	15:384881	32.7904	47.533	18.749	6.43675	14.1336	19.271	18.9923	23.3955	12.3517	26.2694	0.40345	-0.27801
ENSG0000	KHSRP	19:641334	5.25853	4.67175	3.18941	3.01593	8.81903	4.49273	4.1227	4.21943	1.84258	12.9532	0.87685	0.125686
ENSG0000	MT-CO3	MT:8365-	1222.72	1225.02	2330.16	3284.4	5832.18	1297.56	991.396	2829.57	4466.35	2779.98	0.8818	-0.22998
ENSG0000	NFAT5	16:695650	11.0341	9.2621	2.87375	1.46302	3.91929	11.6209	10.7584	5.52453	7.87136	8.09521	0.33345	0.592245
ENSG0000	HES1	3:1941361	0.942755	1.14942	0.226731	0.680438	0.280888	0.325346	2.19778	0.12817	1.09684	0.235268	0.85835	0.360141
ENSG0000	RP11-452	16:314830	0.98527	0.967186	0.745493	3.68506	0.449527	0	0	0	0.103794	0.598499	0.1762	-3.09115
ENSG0000	GIN1	5:1030859	8.11454	4.98472	4.92055	6.98218	3.52787	10.4202	4.01824	5.73488	6.46946	3.72834	0.68885	-0.33244
ENSG0000	SKIV2L	6:3195208	15.3459	11.3067	13.4262	20.2137	13.8525	14.3228	9.31434	20.0344	24.1644	31.3184	0.1813	0.744009
ENSG0000	TMEM102	17:742010	16.7962	18.385	17.6507	12.9282	30.4383	10.5601	12.8651	15.714	11.4279	30.7235	0.64555	-0.29534
ENSG0000	LYN	8:5587981	13.0758	22.6122	11.466	9.34889	11.3611	10.3031	2.56528	4.87935	6.13634	7.87672	0.0195	-1.25898
ENSG0000	RNF157	17:761363	11.5698	7.09769	13.822	5.4971	18.1675	4.76355	4.5813	7.67488	8.23411	14.9027	0.2174	-0.473
ENSG0000	DEPDC1B	5:6059691	3.65171	7.17243	0.157455	0	0.079628	8.86391	5.13905	2.24598	3.35709	1.74748	0.36095	1.14987
ENSG0000	HIST1H2B	6:2625165	0.859976	0.270745	1.01632	0.225067	0.158616	2.9985	1.34244	1.00691	3.77262	0.433607	0.25505	1.94252
ENSG0000	MDC1	6:3069980	5.56452	4.25414	3.59149	1.29738	3.23512	4.22875	3.5768	2.73189	4.93182	6.11624	0.34755	0.537952
ENSG0000	DRG1	22:313995	48.1522	41.5286	65.9065	75.3354	45.9829	52.1171	43.7738	47.8994	51.5349	19.198	0.509	-0.3788
ENSG0000	ABHD14A	3:5195531	43.1758	40.1598	102.335	93.992	72.142	35.8589	35.599	53.2527	64.2105	16.8252	0.2338	-0.79242
ENSG0000	HLA-DOA	6:3300417	0.475401	3.39558	0.566325	0.730642	0.479729	0	2.07011	1.97114	2.18001	0.225318	0.7948	0.259327
ENSG0000	ITGB3BP	1:6344076	28.3396	17.6515	13.3669	14.0431	9.54308	24.0294	26.266	17.0061	8.5153	8.35159	0.8686	0.15748
ENSG0000	ZCWPW1	7:1004008	3.18272	4.92459	4.48237	2.95601	5.68846	0.173807	2.97329	3.56239	0.101218	4.44148	0.32615	-0.84048
ENSG0000	RP11-513	9:1937545	1.34687	3.04105	3.70975	2.03946	1.60396	1.6427	2.3814	3.95077	0	4.30663	0.89335	0.188523
ENSG0000	FAM212B	1:1114833	0.375891	0.1785	0	0.44939	0.260616	0.069858	0.011038	0.970223	2.25911	0.137268	0.28565	1.66816
ENSG0000	ADSL	22:403464	56.4339	68.0736	53.947	70.147	51.0508	58.4256	56.3492	66.6364	84.8197	34.1355	0.95155	-0.03278
ENSG0000	LATS1	6:1496581	6.22135	8.9045	7.50005	7.73608	5.67653	3.7041	3.90548	6.39394	7.12963	12.118	0.44205	-0.35593
ENSG0000	C12orf60	12:148035	1.98878	0.8408	3.48028	1.12004	0.381005	0	2.06303	1.01128	0	0.066395	0.3523	-1.1673
ENSG0000	ZNF615	19:519913	4.70141	4.40588	3.28468	1.41964	1.07979	1.43647	1.53751	1.11035	0	3.06098	0.0741	-1.29628
ENSG0000	HS6ST1	2:1282367	2.70015	3.53175	0.368155	0.265627	1.26834	1.76751	0.347966	0.512335	0	1.38059	0.3146	-1.12491
ENSG0000	ZNF879	5:1790237	6.40108	6.88334	2.96503	1.83816	1.85245	1.49378	4.93767	4.01626	6.03439	3.41775	0.83795	0.109497
ENSG0000	LNX2	13:275459	3.14022	3.11037	1.68167	2.94536	1.37604	3.0486	1.06644	1.4874	2.7117	2.15798	0.6036	-0.24009
ENSG0000	PROB1	5:1393905	0.841856	0.077916	0.128321	0	0.310415	0	2.85829	0	1.87292	0.646072	0.19955	1.9811
ENSG0000	RP11-468	14:241718	0.214959	0.045779	0.976996	0.613487	0.107817	0	0.050375	0	0	0.200429	0.26145	-2.94722
ENSG0000	HCG25	6:3324953	14.7725	10.8087	3.36145	2.00686	0.280943	3.34793	4.67186	1.80204	1.49	2.69976	0.9442	-0.10856
ENSG0000	AC073283	2:4689927	0.304807	0.829381	0.367794	0	0	1.10562	0.14022	0.670191	0	1.0146	0.52205	1.14818
ENSG0000	MYO7A	11:771282	0.069665	0.287059	0.031122	0	0.008249	8.84859	14.3023	10.4748	2.17814	12.5626	0.0007	6.8277

ENSG0000C	CD3G	11:118344	88.6132	96.9155	231.856	151.207	258.983	106.16	146.879	212.375	198.968	282.501	0.8664	0.072028
ENSG0000C	RP1-130H	22:302851	1.11151	1.09603	1.46304	1.62659	1.23648	0.419207	0.334029	0.474382	2.18876	0.179124	0.82775	-0.28119
ENSG0000C	PSMD6-A	3:6400402	5.51586	15.606	0.595893	0.028074	0.092002	3.52885	3.57426	1.02654	0.128047	0.755057	0.5996	-1.1593
ENSG0000C	FAM89B	11:655704	120.839	110.731	107.353	50.3563	237.013	173.892	168.756	106.557	65.091	94.0772	0.72085	-0.16441
ENSG0000C	PGD	1:1039859	20.4922	26.54	20.2097	17.1757	18.8619	39.8894	29.8988	18.7885	12.908	15.1115	0.77325	0.115578
ENSG0000C	SUMF2	7:5606400	32.1702	48.7753	70.9783	66.6614	73.9865	49.8533	56.4069	70.3889	60.8045	64.2345	0.97155	-0.01116
ENSG0000C	C22orf15	22:237630	1.16202	2.39209	0.476353	0.456961	0.344971	2.95675	1.30459	0	0	1.87696	0.70935	0.385412
ENSG0000C	RP11-264	16:289739	2.85515	4.0635	0.857348	1.19533	0.736825	1.64163	1.51306	1.15223	0.997964	0.960033	0.6163	-0.58079
ENSG0000C	DAPK1	9:8749722	0.457839	4.80648	0.205424	1.12846	0.138885	7.23007	0	0.946857	5.94105	0.276211	0.0984	1.42841
ENSG0000C	PTPRE	10:127907	14.3363	11.2198	13.2336	15.3686	16.238	18.5939	11.1547	14.5172	5.71062	18.0147	0.7594	-0.12646
ENSG0000C	CCDC183	9:1367913	0.804428	0.57998	0.044576	0.130295	0.222671	0.234307	0.337926	0.460944	0.685914	0.576081	0.79195	0.262039
ENSG0000C	TSPYL2	X:5308236	23.0627	45.8317	26.5667	10.8766	21.8323	59.464	39.5988	34.2339	30.3869	30.2735	0.12625	0.575273
ENSG0000C	AC006129	19:415451	4.12255	9.28722	2.25756	1.86313	2.71449	6.29488	1.62379	2.17274	0	3.33335	0.7155	-0.56539
ENSG0000C	COPS6	7:1000889	105.726	75.0089	151.93	152.538	142.081	132.738	108.956	138.179	113.542	75.0468	0.57725	-0.17622
ENSG0000C	RP11-731	14:105643	0	3.97443	0.437126	14.0349	2.75866	0	0	0	0	0.895198	0.303	-5.30449
ENSG0000C	PSMC5	17:638194	172.503	172.349	207.775	181.885	210.739	190.773	156.519	175.03	161.428	131.094	0.5407	-0.22017
ENSG0000C	AC055733	3:1308505	0	0	0	0	0	2.794	0	0	0	0	0.21705	inf
ENSG0000C	C11orf54	11:936616	14.4158	13.5636	11.3929	17.3486	11.578	17.0242	13.8499	12.6732	6.56098	10.6865	0.69105	-0.28398
ENSG0000C	ZBTB41	1:1971536	2.12555	1.89573	0.752134	0.370851	0.563398	2.48206	1.26179	1.33356	0.993263	1.87969	0.7111	0.238531
ENSG0000C	RP11-35G	18:576303	6.32135	7.85698	8.46299	13.5804	10.585	2.35913	4.4756	4.76869	8.4636	7.04649	0.10635	-0.85365
ENSG0000C	FOXM1	12:194605	1.79186	4.6135	0.431577	0.184223	0.286006	6.33393	2.08114	0.25345	0.497297	3.9399	0.45285	0.875832
ENSG0000C	RP11-872.	14:965008	0.727978	0.716224	0.239248	0	1.00021	1.83217	0	0.830549	0	1.27364	0.7914	0.445809
ENSG0000C	DIXDC1	11:111927	0.183784	1.12799	0.223247	0	0.166255	1.69751	1.01431	0.749509	0	0.673067	0.2737	1.10516
ENSG0000C	RAB11A	15:656113	76.7969	85.5955	99.3183	48.8042	86.4958	100.975	178.711	123.704	143.57	39.2862	0.74505	0.201998
ENSG0000C	MYO6	6:7574919	0.554501	0.338968	0.045252	0.004592	0.115242	2.29822	0.596047	1.17601	0.073322	0.919875	0.01945	2.34478
ENSG0000C	EPB41	1:2888709	31.6701	56.79	14.8877	17.5999	114.667	15.0413	34.1464	10.7725	19.6131	72.3513	0.29645	-0.75462
ENSG0000C	MFSD10	4:2930560	73.3515	66.417	50.6078	50.7923	72.8069	79.1451	118.416	75.1199	66.7038	93.0019	0.19575	0.463591
ENSG0000C	DYNC2H1	11:103109	0.980686	0	0.099294	0.202399	0.79244	2.66747	0.386528	0.506153	0.439584	2.34491	0.1067	1.82561
ENSG0000C	AFTPH	2:6452430	30.8026	23.0025	13.0859	14.5791	17.2256	21.2643	17.2393	21.2323	29.8295	28.4484	0.33095	0.382996
ENSG0000C	IFNG	12:679894	19.2478	9.8385	2.45144	3.50389	1.29857	99.8179	272.054	222.564	77.55	27.8846	0.0002	4.61734
ENSG0000C	TRIM25	17:568879	2.36071	3.92484	2.46402	1.58173	3.01702	6.72102	8.34692	5.35428	2.61804	8.41902	0.00635	1.1323
ENSG0000C	MUSTN1	3:5281297	1.83495	1.09587	0.016956	3.4362	0.341045	0.061424	0	4.24668	0	1.13348	0.8494	0.312081
ENSG0000C	TP53INP2	20:345605	0.124977	0.026178	0.239626	0.860463	0.133457	0.439346	0.53603	0.720707	0.657257	0.367207	0.63405	0.642983
ENSG0000C	ELOVL6	4:1100458	1.58662	0.176817	2.7537	1.88727	3.50451	3.85708	1.92648	3.66881	5.56084	7.69735	0.1006	0.820755
ENSG0000C	SRSF5	14:697268	675.258	837.614	397.633	695.315	431.814	336.094	405.696	278.778	416.165	342.632	0.06595	-0.74391
ENSG0000C	AC000123	7:1273467	0.70308	0.363685	0.194296	0.132736	1.18087	2.20802	2.6937	0	0	1.81709	0.43435	1.30117
ENSG0000C	CTB-51J2	7:7402778	0.319561	0.346354	0.168503	0.223519	0.168697	0.791282	0	0.094218	0.34183	0.807923	0.6266	0.952607
ENSG0000C	ZNF428	19:435963	22.8725	20.5699	43.6713	26.0483	50.1668	11.2683	15.5105	34.3628	22.7944	20.2342	0.21505	-0.62412
ENSG0000C	MXD1	2:6989355	12.2272	10.5284	7.99397	18.7614	6.51339	8.17856	2.21655	5.55434	13.0873	5.64844	0.22465	-0.94374
ENSG0000C	AC005519	14:742854	1.16181	1.08747	0.748673	0	0.120862	0.591932	0	0.032654	0	0.300688	0.3363	-1.69426
ENSG0000C	DDX11L2	2:1135885	0.135363	0	2.63849	3.25405	0.276664	0.008368	0.594817	3.3675	0	0.211229	0.7425	-0.62153
ENSG0000C	TIMM13	19:238977	43.3774	39.267	83.3842	80.7206	74.0643	55.2072	75.0506	60.409	68.674	18.7032	0.49475	-0.39983
ENSG0000C	WHRN	9:1144020	2.72706	3.85104	11.0677	23.409	8.09303	2.33427	14.2993	27.6724	13.6948	25.6041	0.2669	0.775061
ENSG0000C	SHARPIN	8:1440986	79.9984	81.0848	80.765	79.1403	95.066	67.6317	90.9495	65.2451	60.0489	66.9362	0.65515	-0.23435
ENSG0000C	SPRN	10:133379	1.27847	0.942277	0.947697	0.805905	0.516051	1.01586	1.3462	1.45083	0	1.74104	0.8326	0.323237
ENSG0000C	MYCL	1:3989542	0.100989	3.11868	0.236272	0.524747	0	3.85498	0	0	0	0.061367	0.99235	-0.00186
ENSG0000C	ZNF414	19:850967	12.5406	33.2771	3.24575	4.47167	6.08697	13.7892	13.8101	3.95011	3.29989	9.11212	0.39105	-0.58731
ENSG0000C	SMAD7	18:489198	9.51142	5.06322	8.17737	5.18756	8.81061	8.07786	13.5855	9.25699	15.2082	26.3503	0.02855	0.936322
ENSG0000C	KIR2DL3	19:547244	0.113005	2.66601	1.39409	19.1944	1.41117	4.35472	13.2691	0.804033	0	2.19549	0.63915	-0.50461
ENSG0000C	TMA16	4:1634944	15.178	13.0027	16.7078	11.2917	17.2122	16.6862	18.6233	17.2624	19.797	9.75673	0.82665	0.093304
ENSG0000C	RPS25	11:118998	1084.18	964.183	1846.27	1234.29	512.199	872.786	1049.32	1408.5	753.527	121.09	0.44795	-0.39871
ENSG0000C	CLN8	8:1755777	3.85191	12.5271	9.62199	6.27708	7.32626	8.35837	6.99319	9.80466	6.13249	9.98016	0.9311	-0.06158
ENSG0000C	SRPK2	7:1050134	19.591	26.9126	19.7797	14.0212	27.6861	18.0377	19.5796	13.0238	17.7005	39.1417	0.5143	-0.32921
ENSG0000C	RP11-299.	3:1224162	19.518	22.9306	15.3501	12.1898	6.42381	24.8556	15.9802	13.6056	0.556567	11.3033	0.7138	-0.37374
ENSG0000C	PPARD	6:3534255	4.93621	12.2956	1.18381	2.10399	1.03853	14.255	2.01233	0.990315	0.267609	4.1846	0.9075	0.070548
ENSG0000C	RP11-498	17:819323	0.258151	0.701949	0	0.118906	0	0	2.27599	0.266324	0.733331	0.057603	0.1849	2.12855
ENSG0000C	ACAD9	3:1288795	14.2553	20.3749	14.2961	19.4655	11.9753	24.5339	7.94493	15.8814	23.3669	17.9631	0.67135	0.164797
ENSG0000C	RP11-45M	17:170425	0.151569	1.15239	0.114378	0	0.05932	0	2.31938	0	0	0.491598	0.70155	1.09098
ENSG0000C	DLGAP1-/	18:349603	1.47641	0.478823	0.953254	1.50316	0.117421	0	0.698665	2.42429	1.19218	2.4303	0.699	0.584508
ENSG0000C	RN7SL834	2:2304159	1.05442	0.654427	0	0	0	0	1.86438	0.331229	0	0.370754	0.6161	1.12499
ENSG0000C	DCP1A	3:5328342	26.3175	29.8998	37.0364	23.9257	34.2999	38.7988	27.9895	25.8561	24.0874	47.4161	0.7403	-0.12179
ENSG0000C	RP11-16B	15:618347	0.130416	0.828742	0.507215	0.133354	0.10582	0.562607	0	0.663343	0	0	0.80325	-0.38441
ENSG0000C	RP5-1180	12:360663	2.47106	0.620569	2.80054	0.435961	1.74976	1.58621	0.706092	2.03792	0	4.85188	0.9638	-0.08375
ENSG0000C	CWC27	5:6476892	31.4999	37.2218	30.197	27.4052	42.8878	34.6623	34.7732	21.9575	20.6487	32.021	0.374	-0.3138
ENSG0000C	SPDYA	2:2875163	2.0132	1.25023	1.73779	2.73503	0.276858	3.11421	1.78569	1.00527	1.66676	2.22441	0.78425	0.297294
ENSG0000C	IGLV5-45	22:223759	0	115.257	0.29509	33.0568	0.205635	0	0	0.104758	0	0.764089	0.02505	-6.51573
ENSG0000C	RFTN1	3:1626515	17.4726	15.4587	10.0388	18.2086	14.7516	18.3454	25.9439	20.951	8.0541	27.948	0.60175	0.325998
ENSG0000C	BTLA	3:1124639	6.26732	8.40027	3.11151	2.34422	4.68798	8.58493	5.74155	9.53805	9.98335	2.47952	0.3104	0.652403
ENSG0000C	RP11-21J	18:910262	39.255	25.8219	19.8848	30.7395	13.6463	32.3313	42.1155	30.3045	33.7511	0.560227	0.77415	0.442244
ENSG0000C	MAN1B1-	9:1370849	1.55488	2.66657	1.14794	1.58702	1.82188	0	3.36759	2.46649	3.56538	0.909885	0.6465	0.324334
ENSG0000C	RNF168	3:1964687	7.44051	8.46477	9.08697	4.2559	10.5535	9.55127	8.08331	8.45096	3.52137	16.6116	0.85925	-0.0751
ENSG0000C	ZNF621	3:4052487	2.11009	4.55999	3.87013	1.62937	2.06334	1.71041	4.68306	7.84356	0.398708	9.08003	0.47605	0.496405
ENSG0000C	UIMC1	5:1769050	18.6632	20.6104	13.9281	20.0735	11.7116	22.914	15.6794	17.4487	17.112	13.0575	0.7428	0.148509
ENSG0000C	FIZ1	19:555913	3.83047	3.41779	1.28522	0.326865	2.30581	2.41389	5.43895	1.63212	0.861474	4.37447	0.72005	0.465169
ENSG0000C	COQ8B	19:406915	14.7158	14.6937	11.2942	13.9317	12.3668	15.1862	19.8802	11.7541	16.9019	14.9666	0.73975	0.224697
ENSG0000C	MDM1	12:682724	20.0696	13.6778	5.90871	8.59863	5.10096	15.3111	11.6111	8.37053	9.91152	8.25138	0.5702	0.26355
ENSG0000C	AP001462	11:647789	0.854764	0	0.138297	0.536673	0.257551	0	0.966389	0.302609	0	0.360737	0.9375	-0.10154
ENSG0000C	CTD-2013	3:6897521	5.28154	4.86221	1.88286	4.86404	2.01035	6.67645	5.00689	1.76728	3.6493	2.69689	0.9678	0.043922
ENSG0000C	RRNAD1	1:1567218	1.25927	1.32321	5.75199	1.70367	6.08903	0.368289	1.34224	3.36417	5.11938	7.03682	0.7007	0.311471
ENSG0000C	CTD-2554	19:377358	0.222661	0.489021	0.027522	1.62747	0.349912	0	0.121233	0	1.70718	1.28521	0.77545	0.566533
ENSG0000C	FOCAD	9:2065830	8.25824	4.59686	2.26256	2.4741	2.18225	5.93241	4.6154	1.79904	2.05699	5.04528	0.9026	-0.05294
ENSG0000C	FAM179B	14:449243	5.46696	6.41718	3.16988	3.10305	1.68634	4.63684	4.85103	5.08663	3.81514	4.46454	0.82515	0.189959
ENSG0000C	B4GALT3	1:1611664	42.5523	44.055	39.2115	61.3852	49.5949	48.3764	56.1257	42.8942	49.4755	50.1096	0.96425	0.017786
ENSG0000C	CTD-2547	16:252579	0.035061	0.772741	0	0.181083	0.081549	0	0.496303	0.501884	2.02319	0.696915	0.29925	1.99902
ENSG0000C	RPS16P5	6:5326739	0.247846	0.482398	0.646602	0.478462	0	0.21745	0.755205	0	3.22194	0	0.51355	1.28256
ENSG0000C	MSL2	3:1361489	14.6102	11.4059	4.98839	4.22793	10.2373	2.44286	10.1621	7.05561	4.6407	11.4618	0.2221	-0.45925
ENSG0000C	ZSWIM4	19:137954	0.631026	0.203267	0.039368	0.014081	0.186169	0.15012	0.925787	0.262549	0.267695	0.695484	0.39925	1.23292
ENSG0000C	RP11-80H	3:5830624	9.60879	11.1426	17.3043	15.1168	15.4416	16.6368	11.8224	15.1941	4.81276	13.4924	0.65835	-0.24564
ENSG0000C	OSGIN2	8:8990185	7.31249	7.40004	3.86305	3.12799	1.98297	4.9464	4.86288	4.92948	4.98012	4.03227	0.96405	0.029202
ENSG0000C	GMNN	6:2477493	20.9038	24.658	6.80385	9.96437	8.38143	40.8701	34.1867	23.9252	7.48587	7.3924	0.10355	0.778994
ENSG0000C	RP11-474	2:6812293	1.60683	2.41431	0.812985	0	0.97254	1.60001	0.042159	0.876432	1.04016	0.994294	0.8555	-0.27723

ENSG0000C	ZNF333	19:146191	7.78007	5.42445	6.07346	5.64731	2.37736	1.68514	4.22204	2.8755	13.8066	6.78007	0.8909	0.08366
ENSG0000C	CCL3L3	17:361832	36.5295	33.6379	124.568	168.67	11.5511	53.8507	195.2	320.642	118.457	20.7475	0.04505	0.918004
ENSG0000C	CCDC71	3:4916253	0.05497	0.539076	0.515388	4.37541	0.579034	0	0	0.692354	2.44213	0.280925	0.75745	-0.67987
ENSG0000C	PAFAH2	1:2595976	13.5604	9.48671	28.0364	20.2707	20.997	8.42784	4.71547	9.90724	7.47581	22.0469	0.0133	-0.97961
ENSG0000C	LAMA2	6:1288831	1.65388	1.49941	0.29148	0.128954	0.373821	0	0.084754	0	0	0.195836	0.1211	-4.63568
ENSG0000C	C16orf52	16:220074	1.12919	0.740405	1.04287	0.424834	1.4987	0.686621	2.89104	1.48231	0.457786	2.72322	0.37895	0.646385
ENSG0000C	KRT18P61	17:606774	1.04314	1.3058	0	0.452802	0.029972	0	1.57328	0	0	0	0.72955	-0.93729
ENSG0000C	EFHC1	6:5236142	4.45402	11.1975	2.38198	3.57635	2.45783	3.12087	4.42849	5.3811	6.31837	3.50226	0.91805	0.073614
ENSG0000C	SCN1B	19:350306	0.811702	0.514067	0.07826	0.681247	0.767878	1.0748	0.190358	0.081639	0.801012	1.53763	0.7574	0.407642
ENSG0000C	CCDC120	X:4905357	0.480677	0.431637	1.08467	0.352564	1.16826	0	0	0.97817	0	1.14456	0.48375	-0.78197
ENSG0000C	DNAJC3	13:956771	10.4607	6.15656	17.5079	14.3171	17.3301	13.1957	10.2829	15.4233	21.162	25.2854	0.5338	0.221261
ENSG0000C	RPS4Y1	Y:2841485	115.19	136.032	175.582	139.562	164.858	110.698	128.428	131.959	125.28	42.7632	0.1778	-0.48605
ENSG0000C	BACE2	21:411678	0.378969	2.86902	0	0.503784	0.344538	0	0	0	0.065972	0.056586	0.0855	-5.37159
ENSG0000C	TMCO1	1:1657242	162.126	71.5245	104.268	126.57	93.5546	245.902	204.093	139.774	139.2	63.5515	0.23805	0.455135
ENSG0000C	PLCXD1	X:276321-	4.48827	5.69773	2.23981	0.656704	3.99987	4.89428	3.54234	3.7154	3.81495	3.74596	0.72	0.245851
ENSG0000C	HMGA1P4	9:1286631	2.0689	0.991046	1.87447	5.12495	0.276944	0	1.28957	0.712296	0.908605	0	0.3672	-1.5966
ENSG0000C	PYCARD	16:312014	61.1667	37.5378	71.6717	83.7643	65.5335	93.015	56.5292	72.5188	46.9984	16.2206	0.474	-0.26449
ENSG0000C	RP11-157I	13:736860	3.75815	7.56936	1.01852	0.978445	0.125107	0	0	0.272649	2.69806	0.272247	0.49175	-1.71479
ENSG0000C	RBMXL1	1:8893577	3.37275	4.64748	4.71701	3.05685	4.21448	0.700417	4.97262	3.95718	3.00789	6.0515	0.7389	-0.25675
ENSG0000C	LOXL1	15:739080	1.67187	3.9436	1.7548	0.211149	1.10642	0	2.41358	0.354679	7.28656	2.129	0.58595	0.761959
ENSG0000C	JAK1	1:6474509	155.633	148.981	77.1134	79.1505	123.212	84.0811	65.5402	53.1734	80.3292	118.194	0.09805	-0.5705
ENSG0000C	ALS2CR12	2:2012882	1.45205	0.497226	0.283492	1.12045	0.279701	0.682644	1.17137	0	0.919488	0.389222	0.74825	-0.27247
ENSG0000C	GALNT12	9:9880769	7.99286	4.26052	2.6865	0.670463	3.32781	5.26855	3.65362	2.24816	2.01884	4.86843	0.9207	-0.05493
ENSG0000C	NBN	8:8993333	15.4616	14.4735	9.15946	6.59911	12.6744	13.2428	20.7124	5.62887	9.04662	18.8439	0.7228	0.179472
ENSG0000C	ANKIB1	7:9224623	4.26381	7.43827	4.48	4.97934	7.13678	4.46433	5.55513	7.54734	5.53576	13.3065	0.4689	0.288255
ENSG0000C	KLHL11	17:418535	3.08249	5.23262	10.0394	9.50274	11.482	5.08547	6.16325	12.071	9.90615	12.7016	0.78585	0.113213
ENSG0000C	NFX1	9:3329051	19.1518	25.3188	13.2461	18.4202	7.08268	16.6438	11.0525	15.8836	13.542	16.0402	0.5266	-0.22025
ENSG0000C	RP11-792,	7:6662888	6.61004	3.07261	7.49307	7.4463	3.09837	7.92026	3.48819	6.765	4.20872	1.66121	0.86995	-0.26604
ENSG0000C	HIGD2A	5:1763654	97.2555	97.4823	172.135	155.918	189.518	199.634	193.797	170.855	107.781	60.3183	0.8744	-0.09502
ENSG0000C	PRPS2	X:1279135	16.0015	22.3672	12.649	10.1668	10.9531	18.0022	20.087	17.4177	12.0883	15.8379	0.6946	0.14303
ENSG0000C	RP11-187(	9:4147995	0.822734	0.621968	2.329	0.185651	0.958149	0.325083	1.61015	1.53468	0	0.8269	0.91395	-0.1952
ENSG0000C	INPP5E	9:1364286	9.69974	11.2527	4.133	6.77053	10.5987	8.19845	4.08691	10.3943	11.8837	16.6815	0.36105	0.424453
ENSG0000C	CEP72	5:612271-	4.02335	1.99094	1.88998	3.55339	1.74845	2.00999	0.725317	1.54198	3.61621	3.60547	0.96595	-0.04034
ENSG0000C	RP11-259I	3:3724178	0.916194	0.301383	0.376481	1.42522	0.143677	0	0	0.171669	1.86555	0.271044	0.89025	-0.31362
ENSG0000C	RP5-827C	1:2343721	0.206124	0.336461	0	1.08479	0.153199	4.53514	0	0.380885	0	0.083934	0.54675	1.39714
ENSG0000C	RP6-91H8	14:711411	0.381224	0.947506	1.50424	5.41274	2.57353	0	0.667209	2.5391	0.262116	0.766815	0.3856	-1.40795
ENSG0000C	RP11-589I	17:685386	2.75752	1.49262	0.518174	1.41893	0.694035	0	2.42613	1.16123	3.33694	0	0.8941	0.208107
ENSG0000C	UAP1L1	9:1370775	6.5212	6.13733	2.1101	3.32645	3.56148	12.009	14.2027	7.57256	6.34548	8.28421	0.02695	1.09558
ENSG0000C	RP11-345I	1:1659324	0.641536	11.4375	1.28729	1.29486	4.07988	0.119184	1.02692	2.34531	0.78342	4.20812	0.4526	-0.9883
ENSG0000C	AAMP	2:2182641	44.4827	56.9043	84.2854	88.0008	87.0771	50.1998	52.9177	74.1045	68.5179	78.583	0.5975	-0.17518
ENSG0000C	ERMP1	9:5629024	18.9462	7.9388	3.77774	3.88602	4.14089	9.14939	8.73175	6.76661	5.72503	10.6188	0.95845	-0.02224
ENSG0000C	RP4-816N	12:194605	2.3091	1.95064	1.79351	4.58061	0.921424	0.20776	1.75925	3.6837	5.34388	0.926206	0.6544	0.55823
ENSG0000C	IBTK	6:8216998	9.37685	10.7672	2.8673	3.70837	4.91542	4.25676	7.24666	4.76604	3.05366	8.98561	0.5425	-0.21982
ENSG0000C	ALPK1	4:1122745	0.565917	4.25675	0.125769	0.259693	0.153873	3.29046	1.53191	0.617996	0	1.26552	0.94755	0.075788
ENSG0000C	PTK2B	8:2731148	57.1181	67.2954	23.9838	22.8728	30.9245	43.4423	61.2772	34.0037	27.2045	63.5803	0.5349	0.181888
ENSG0000C	RP11-783I	11:642444	0	0	0	0	0	0	0.491679	0	1.03611	0.141885	0.11405	5.35475
ENSG0000C	ZFYVE26	14:677018	1.32388	2.62694	0.872605	1.43604	0.778581	2.70217	2.23215	1.01264	2.90124	2.26197	0.5447	0.684659
ENSG0000C	HECA	6:1391351	21.3525	23.3549	8.03629	4.84916	9.39659	10.6534	12.4316	16.7292	10.6896	21.0308	0.74	0.116097
ENSG0000C	HSPD1	2:1974865	88.042	89.4655	66.061	56.1264	98.6499	140.788	93.0536	65.5526	93.5866	101.917	0.48165	0.355625
ENSG0000C	TRERF1	6:4222493	3.46031	4.95529	1.51657	0.798931	2.51181	2.41897	4.494	2.35162	1.46108	5.85329	0.6371	0.231369
ENSG0000C	MIR3945H	4:1848445	0.140344	0	0.239625	0.221356	0.061424	5.08196	1.39708	1.77866	6.30178	0.971103	0.0724	4.60243
ENSG0000C	ASNS	7:9785211	14.8239	24.1452	11.0805	13.4492	8.88592	32.4269	23.0137	22.1231	13.8991	20.1273	0.1339	0.65382
ENSG0000C	AC017035	2:1969670	0.989341	1.08761	0.178091	0.736977	0	0	1.01729	0.126693	0	0.146767	0.56885	-1.09908
ENSG0000C	CLCN2	3:1843359	0.64451	0	0.617894	1.93777	0.257444	1.97365	1.54036	1.06282	0.851925	1.31942	0.05845	1.40762
ENSG0000C	FLJ22447	14:611875	2.25348	0.535435	0.509483	0	0.242819	1.20924	1.02904	0	0	0.64574	0.9457	-0.1092
ENSG0000C	MFSD14B	9:9437455	24.4033	37.0763	14.1093	14.6652	10.9161	22.4415	25.9649	25.2198	13.3924	22.8429	0.705	0.132978
ENSG0000C	ACBD6	1:1802302	20.9864	39.9386	26.5961	25.8546	27.4255	18.3709	21.2691	23.805	21.3893	18.7296	0.3432	-0.36836
ENSG0000C	RP11-66H	16:109444	1.76435	1.31208	0.348907	0	0.096553	1.79858	0	0.308855	0	0.376793	0.89235	-0.22355
ENSG0000C	TMTC4	13:100603	4.59061	5.14572	1.5231	2.97332	2.38993	5.30828	4.32497	6.76906	11.3004	7.47776	0.03795	1.08848
ENSG0000C	SCAMP1-,	5:7834236	26.0016	15.54	41.5431	40.4719	15.4225	16.4182	18.0396	16.0511	20.5894	17.6995	0.11915	-0.64509
ENSG0000C	PLEKHA3	2:1784804	8.63395	6.13785	7.74251	9.57498	9.14227	6.81352	4.38351	8.75121	6.98174	11.5155	0.75685	-0.25395
ENSG0000C	INTS1	7:1470276	15.2653	15.2637	2.21715	2.00583	4.76415	9.95306	8.05957	3.99253	2.21313	8.37998	0.74595	-0.15116
ENSG0000C	ZFYVE16	5:8040801	5.26417	5.16099	0.592053	1.8731	2.51151	8.07139	3.00798	0.433554	9.92325	2.97516	0.16295	0.777993
ENSG0000C	TRAPPC8	18:318291	13.6127	11.892	4.49163	5.3194	5.9235	11.4657	5.42722	6.36345	11.9152	12.0085	0.3475	0.371343
ENSG0000C	RP11-548I	2:2186077	0.69211	1.40948	0.766885	0.088178	0.855498	0	3.02734	1.18347	6.71054	3.53064	0.33615	2.08407
ENSG0000C	URB2	1:2296262	2.10153	2.80043	1.4292	1.43905	1.03718	2.15196	1.35851	1.7694	0	1.34969	0.6227	-0.40565
ENSG0000C	CD80	3:1194985	0.180153	0.307481	0	0	0.034673	1.25613	4.51625	0.884195	3.28098	0.407939	0.0219	4.47342
ENSG0000C	DNAJA2	16:469553	43.6677	42.7767	35.5503	26.0969	29.0553	52.8509	47.8769	30.0835	24.2702	23.7196	0.8865	-0.04824
ENSG0000C	ENPP5	6:4615918	3.27622	2.47844	4.04758	5.47215	5.12247	0	0	0	1.52334	1.1618	0.10575	-3.13086
ENSG0000C	C14orf119	14:230585	59.4515	55.9035	92.494	64.9377	60.1979	102.521	77.0692	68.0935	47.1292	22.1714	0.8209	-0.13524
ENSG0000C	TBC1D13	9:1287872	12.5898	15.0531	6.53365	5.66361	10.455	10.1059	5.43011	6.11464	2.28282	13.358	0.19975	-0.52017
ENSG0000C	RSAD2	2:6840569	5.84811	4.22931	4.89972	5.95874	4.38942	9.45205	104.715	41.085	10.3867	12.563	0.00005	2.69046
ENSG0000C	FGFR1	8:3841113	10.5453	15.6906	6.92245	4.82601	6.1108	3.3809	1.31135	7.56131	2.82957	4.17303	0.00345	-1.36527
ENSG0000C	PMFBP1	16:720442	0.508334	1.5861	0.316727	0	0.15759	0.920412	1.1058	0	0	0.871131	0.8457	0.26198
ENSG0000C	CARF	2:2028747	2.37	4.9827	0.917366	2.90741	1.15224	1.19673	1.36726	3.02924	0.979055	3.60087	0.2822	-0.73837
ENSG0000C	ARFIP2	11:647468	24.8963	34.4292	33.2527	30.1117	29.6409	22.3815	16.5767	22.4571	16.0925	32.9008	0.3225	-0.54848
ENSG0000C	FAM86DP	3:7541506	3.63627	5.42905	3.53714	4.721	2.4209	2.6307	4.9608	1.40241	3.66968	2.53976	0.59105	-0.46444
ENSG0000C	BRD7	16:502461	39.963	29.7644	24.6362	16.2616	42.886	51.4027	41.7303	28.8132	19.9348	46.393	0.79295	-0.15094
ENSG0000C	ACTN4	19:386476	12.877	5.35434	29.8615	34.1333	53.6387	18.4131	21.6074	78.9643	63.3706	104.59	0.0492	1.06179
ENSG0000C	CTD-2528	19:377285	0.182921	0.408454	0.582208	0.90024	0.577474	1.63589	1.03837	0.831038	0	0.836649	0.75395	0.59791
ENSG0000C	ANKS3	16:469650	9.46697	14.2997	10.4932	10.8781	8.3661	11.1953	11.9393	13.6053	10.2261	15.7994	0.6352	0.249378
ENSG0000C	PRPSAP1	17:763094	34.5492	31.7995	29.1524	33.4563	25.5382	36.4482	35.2223	26.5892	34.6438	22.5241	0.79985	-0.09087
ENSG0000C	TM2D1	1:6168104	36.2798	36.2902	36.1616	39.8179	14.0006	48.7206	59.1388	40.3678	15.8988	17.9966	0.67605	0.177413
ENSG0000C	RP11-730I	11:121447	0.670254	1.66928	0.224663	1.65674	0.251832	0	0	0.089348	0.262432	0.239039	0.20465	-2.33799
ENSG0000C	SMIM1	1:3772787	0.178082	0.30129	0.684626	0.638891	0	0	0.647326	0.162932	0.698453	0	0.72705	-0.48805
ENSG0000C	APOM	6:3163902	0.665311	2.0463	2.15698	3.55656	1.58419	2.90257	1.79641	0.588684	3.01654	1.0939	0.954	-0.08216
ENSG0000C	AGPAT1	6:3214835	11.2291	9.92987	18.2848	12.3659	20.7675	12.6183	4.69858	19.5704	12.7616	32.1302	0.76005	0.144196
ENSG0000C	HINT2	9:3581295	73.627	46.3123	91.9957	95.666	49.2224	82.5361	98.3216	63.9423	92.1715	18.2332	0.88135	-0.06694
ENSG0000C	KIF2C	1:4473981	4.45641	6.83404	1.028	2.34949	0.290908	10.1857	4.20249	2.88394	0.066752	2.3141	0.7838	0.45225
ENSG0000C	HS1BP3	2:2056044	8.88669	5.82863	4.8052	3.51007	7.54449	10.3273	5.00782	8.85066	7.68375	5.05245	0.69005	0.215636
ENSG0000C	CCNL1	3:1571465	261.634	216.619	116.775	188	103.25	216.7	189.334	164.921	135.807	193.304	0.96695	0.01233

ENSG0000	LL22NC03	22:223032	4.87944	6.48767	5.32226	4.7349	3.26711	2.80016	3.76563	4.79125	6.8996	0.902696	0.7221	-0.29358
ENSG0000	RPL34	4:1086205	670.854	495.852	1682.03	921.937	290.317	625.509	598.304	763.638	554.883	48.6273	0.12525	-0.6536
ENSG0000	RPS24P8	3:4515977	0	0	0.967195	0.830684	0.295702	0	0.509118	0	0.543298	0	0.48145	-1.04211
ENSG0000	RAB18	10:275041	66.9602	28.8693	66.2148	64.1769	46.7034	38.1405	35.8436	39.9553	37.4697	27.3886	0.0738	-0.77706
ENSG0000	SMC2-AS1	9:1040800	0	0	0	0	0	1.94813	0.709742	0	0	0.369114	0.0189	inf
ENSG0000	HECTD4	12:112160	5.35884	5.10503	2.77856	3.63911	10.9172	5.01264	6.76171	3.33545	3.43588	19.8996	0.2416	0.721981
ENSG0000	PLAC8	4:8309004	256.88	197.006	215.155	253.103	242.568	62.4407	9.71058	65.6776	50.1436	32.9718	0.00005	-2.42868
ENSG0000	CDHR3	7:1058767	1.59854	5.91866	0.404669	0.041885	0.31941	0.331512	1.25624	0.513325	0.352943	0.412092	0.13595	-1.21543
ENSG0000	MATN2	8:9786883	12.5947	0.291849	6.62683	8.53767	2.64712	0	0	0.92084	2.07314	3.81651	0.15455	-2.16393
ENSG0000	ZFP28	19:565389	0.568532	0.050489	0.591132	0	0.085626	1.12329	2.34358	0.507971	0	0.859416	0.1323	1.65695
ENSG0000	IKZF2	2:2125813	11.5365	12.1302	5.62891	4.97595	6.78937	4.1413	9.4079	6.46483	8.43617	9.10517	0.73665	-0.21414
ENSG0000	OSBPL11	3:1255288	5.30667	4.69449	1.45643	0.914761	1.40299	3.74602	3.20102	2.01425	1.77411	3.40328	0.93035	0.041202
ENSG0000	RP11-44I1	16:485387	0	0.160362	0	0	0	1.52659	0.191055	0	0.963078	0	0.29635	4.1573
ENSG0000	BCO2	11:112024	0.772599	0.37171	0.24665	1.06956	0.688386	2.19472	0	0.377146	0	0.817665	0.9593	0.054315
ENSG0000	APRT	16:888093	221.923	180.555	433.584	454.407	389.794	206.772	233.637	303.611	262.01	100.092	0.0573	-0.66667
ENSG0000	FAF1	1:5043702	31.0558	37.4771	34.3992	20.0368	30.0697	37.0514	23.8986	25.4579	28.237	42.4797	0.8604	-0.08094
ENSG0000	AFG3L1P	16:899725	11.3965	21.3338	9.41776	21.3212	8.15916	11.8014	18.7228	14.0134	13.4922	21.3636	0.80675	0.135867
ENSG0000	CH507-9B	21:507345	48.5417	41.3841	54.858	44.6921	47.5274	59.6537	49.5604	44.972	35.4844	24.3406	0.7373	-0.18285
ENSG0000	NUMA1	11:719985	55.9288	69.1705	19.0226	11.7904	36.4773	41.2162	33.1068	18.9405	12.4356	69.3064	0.97185	0.025569
ENSG0000	PRPF6	20:639811	45.8767	59.63	39.7933	35.6509	40.6731	28.0485	35.8592	31.9199	24.0982	53.9835	0.2586	-0.36264
ENSG0000	RP11-157.	6:1528363	2.02301	0.568918	0.514945	0	0.34695	2.06479	1.74933	0	0.739411	0.157647	0.7769	0.484896
ENSG0000	PHLDB1	11:118606	0.106059	0.853905	0.438949	0	0.310872	0	0	1.98051	0	0.752909	0.4497	0.90358
ENSG0000	MYO5A	15:523072	9.35606	5.8566	2.1213	1.66806	4.63349	9.52377	4.9779	3.69596	3.82599	10.4841	0.7337	0.1617
ENSG0000	TNPO1P1	10:843285	1.4522	3.69631	0.551623	0	0.290479	0.727955	3.96898	0	0	3.78264	0.7728	0.570936
ENSG0000	L2HGDH	14:502375	2.27007	0.814185	0.93209	1.91734	0.75099	0.523114	0.618498	0.890924	0.780931	1.06467	0.30405	-0.94218
ENSG0000	CDR2	16:223459	32.7477	57.9546	13.3892	6.32719	13.1611	13.1855	22.9063	10.5207	17.2722	25.3239	0.40315	-0.42135
ENSG0000	AHCTF1	1:2468390	17.2392	19.7013	3.27146	2.32026	7.93162	3.53874	8.36213	5.73769	2.77731	7.81321	0.0708	-1.06377
ENSG0000	VCAN	5:8347146	3.8503	17.5135	0.129612	0.867817	1.66729	6.8143	0.842593	4.26681	0.282801	0.414552	0.6395	-0.45036
ENSG0000	POLR2F	22:379430	107.091	73.4968	153.515	149.321	102.805	149.522	108.552	123.594	82.0636	29.6527	0.52785	-0.32361
ENSG0000	NEU1	6:3185765	27.5312	18.8362	25.0183	29.0121	20.5109	55.9404	24.0234	28.046	26.598	14.8349	0.4503	0.276905
ENSG0000	NOMO2	16:184173	41.046	51.7636	14.9466	26.1194	21.2098	40.2373	31.5228	19.6717	24.0953	33.8495	0.44145	-0.386
ENSG0000	LITAF	16:115477	231.325	205.996	568.968	479.112	594.991	197.997	122.793	219.432	235.834	346.398	0.00415	-1.08941
ENSG0000	RP11-188	9:1376056	0.316316	0.878159	0.074836	0.04961	0.024493	5.05911	0.27551	0.257958	0.741893	0	0.1626	2.3399
ENSG0000	ADAMTS6	5:6514873	0.49827	1.04357	0.454144	0.829062	0.125723	0	0.241346	0.758471	0.136097	0.111611	0.2109	-1.08405
ENSG0000	RP11-533	1:1798816	3.53407	2.42979	5.07794	1.06852	1.2659	4.22063	3.40991	2.00075	0	1.94921	0.80165	-0.35802
ENSG0000	BOLA3	2:7413539	50.9688	27.3927	89.5158	92.2277	44.5275	88.0046	58.5745	85.1248	54.102	15.6154	0.84465	-0.1081
ENSG0000	GGT7	20:348447	1.20173	4.74757	0.905001	0.385757	0.685385	1.62135	0.515912	1.78141	0.286434	1.87627	0.70445	-0.42999
ENSG0000	RP11-206	1:585988-	1.29579	0.735572	0.78372	1.31886	0.89308	0.947256	1.2611	1.48864	0.389487	2.61197	0.5285	0.560107
ENSG0000	CTD-3092	15:304707	6.41658	5.0887	5.72879	3.62101	4.9805	4.43828	1.70257	2.14225	1.80845	6.46382	0.6109	-0.51811
ENSG0000	AC145110	8:2974830	0	0	0	0	0.011387	0	5.58174	0	0	0	0.46425	8.794
ENSG0000	MFSD5	12:532512	28.7957	26.2059	51.0834	45.801	67.5773	15.0826	38.7217	55.5669	39.6352	70.3334	0.84405	-0.06652
ENSG0000	DIP2A	21:464588	26.6941	39.0627	24.3218	25.4693	22.2131	33.2362	16.1854	22.0945	20.664	37.1004	0.8297	-0.07647
ENSG0000	GMCL1	2:6982964	27.4387	20.6285	11.9117	10.8031	9.33059	13.3162	8.89579	13.6809	13.3062	13.2753	0.2757	-0.39474
ENSG0000	RP11-467	7:7695983	0.577683	0.670909	0.13588	0.596241	0.385716	0	0	1.08937	0	0.985477	0.97675	0.038753
ENSG0000	RP11-344	1:1215183	3.70415	1.20888	3.42732	2.43039	1.66583	2.32751	1.34051	2.45688	7.31914	0.846204	0.8562	0.266971
ENSG0000	CCDC180	9:9715656	0.724051	1.00806	0.375308	0.492685	0.896883	0	0.884153	0.130062	0	2.96984	0.84625	0.150635
ENSG0000	KLHL5	4:3904503	3.46907	5.60871	1.32482	2.23653	2.67126	4.93887	2.90493	4.70454	3.81948	6.209	0.39575	0.439086
ENSG0000	MRPL53	2:7447195	31.8535	20.7798	54.1434	34.4615	23.2432	44.0901	36.3953	31.0395	36.7931	7.01651	0.8893	-0.16078
ENSG0000	RP11-111	11:763813	0.335497	1.05786	1.23104	0	0	0.498606	0	0	0.300918	0.887072	0.8599	-0.39206
ENSG0000	ZNF192P1	6:2816178	0.15123	1.16656	0	0.319481	0.192264	1.03138	0	1.14232	0	0.514374	0.7498	0.386957
ENSG0000	PPP6R3	11:684607	18.3163	26.4884	17.5873	15.3594	15.8218	12.6363	21.4974	20.2893	26.6494	43.7544	0.4905	0.271728
ENSG0000	KIFAP3	1:1699213	19.7934	19.7847	7.64436	12.2661	10.4293	18.8891	11.4202	14.8891	6.70497	15.6961	0.9133	-0.04166
ENSG0000	PLEKHJ1	19:210098	98.4875	91.6184	131.626	164.765	142.596	100.316	149.79	99.4412	129.238	69.0443	0.6154	-0.26759
ENSG0000	RP11-715	18:253752	0.406845	0	0.515171	1.72309	0.042843	1.7004	0	0	0	0.858145	0.9004	-0.31029
ENSG0000	HPS6	10:102065	14.2791	11.5632	9.13274	9.42676	8.60375	14.2277	9.82588	9.6771	5.85502	8.71472	0.7331	-0.14756
ENSG0000	TIGD2	4:8911149	2.51038	0.840212	1.66203	1.48367	1.37567	0.393865	5.87608	3.08242	0	3.02436	0.47075	0.778366
ENSG0000	FOXO3	6:1085598	7.51506	6.21732	11.8144	6.14722	15.0737	5.16315	4.33133	8.16154	3.78203	22.8197	0.3296	-0.46898
ENSG0000	ASF1A	6:1188134	67.7058	50.8127	71.958	58.4293	32.367	53.0702	55.8635	49.6564	50.5785	27.1322	0.57345	-0.20817
ENSG0000	LINC0093	12:825452	0.503143	0.244129	0.096574	0	0	4.10734	0	0.060883	0.161059	0.020358	0.0528	2.515
ENSG0000	CTDNEP1	17:724001	19.9662	20.8642	11.4886	11.6268	31.4184	18.5886	15.7908	27.8901	9.07255	38.9189	0.89175	0.182924
ENSG0000	HINFP	11:119121	18.3463	19.773	19.2594	19.0128	13.1475	21.5289	11.7948	19.0673	13.7327	21.4947	0.7433	-0.15049
ENSG0000	CDIPT	16:298583	54.363	48.9399	68.8159	69.0997	76.615	45.3448	47.0752	57.8466	62.1176	78.4654	0.72125	-0.11489
ENSG0000	MRPL51	12:649188	131.927	98.8542	162.132	157.126	149.73	206.611	202.224	177.902	126.511	41.701	0.85695	0.078828
ENSG0000	CDPF1	22:462440	13.2819	15.3003	39.0049	24.0413	20.4311	15.0513	18.1767	24.8425	7.64372	14.807	0.11815	-0.67845
ENSG0000	PRDM15	21:417982	2.7882	4.22135	0.568169	2.50658	1.00611	1.66917	2.11441	0.822932	0.048414	1.85027	0.32015	-0.66108
ENSG0000	SORL1	11:121447	36.7316	29.2382	33.7792	23.1763	37.0659	16.348	4.65117	14.6641	25.9331	48.8758	0.0312	-0.85885
ENSG0000	PPP2R2D	10:131900	23.8337	30.2201	22.5324	19.7285	15.4058	21.1009	23.9088	18.6488	8.88545	18.1314	0.3325	-0.43806
ENSG0000	RP3-402G	22:502009	0	0	0	0	0	0.894354	0	0	0	0.008671	0.72285	0.871643
ENSG0000	ECHDC3	10:117423	0.331742	2.94053	0	0	0.135854	1.49983	0	0.289031	0	0.121862	0.6753	-0.64252
ENSG0000	CEMP1	16:247505	3.66248	3.34073	0.453202	0.798017	0.991888	0.234433	3.09387	1.66342	4.46724	3.19157	0.54775	0.84538
ENSG0000	CTSB	8:1184252	96.5986	144.97	72.5207	86.8595	58.735	1221.18	176.223	141.086	139.398	122.329	0.0034	1.83043
ENSG0000	SCCPDH	1:2467240	21.2627	9.38906	17.0297	15.7011	17.039	37.7514	21.9948	15.0846	6.79168	9.26854	0.7419	0.128746
ENSG0000	GPALPP1	13:449392	18.2866	14.4656	8.907	7.52489	9.52547	17.9889	14.4015	11.1246	9.85945	9.66781	0.76545	0.17823
ENSG0000	MBTPS2	X:2183963	4.00985	3.78139	1.50472	2.37746	1.37118	3.18626	3.47801	0.519109	3.30637	2.04332	0.86615	-0.09595
ENSG0000	NDUFB4	3:1205963	150.976	142.228	168.41	172.92	115.532	201.392	173.098	180.606	126.556	37.8416	0.6913	-0.13725
ENSG0000	ACSL6	5:1318069	2.04014	2.29979	1.54735	3.65989	1.20603	0	0.078776	1.53297	2.10396	2.45988	0.2219	-0.98965
ENSG0000	PSMC1	14:902564	122.578	118.558	135.346	109.082	121.802	179.259	143.368	119.655	72.7692	103.541	0.73915	-0.12991
ENSG0000	TESK1	9:3560530	12.4232	10.2114	5.53629	2.6622	8.68196	13.459	12.715	7.97238	5.64741	15.3467	0.53015	0.415595
ENSG0000	DAB1	1:5692920	6.17876	0.639363	2.59397	5.89867	2.31263	2.76921	0	0	4.17505	1.07355	0.3914	-1.14576
ENSG0000	PAQR3	4:7877634	4.22799	3.82142	1.31153	1.93441	1.25777	5.08779	3.91951	2.38104	4.35955	2.91303	0.3173	0.602218
ENSG0000	HLX	1:2208327	0.247864	1.62507	0.106379	0.182884	0.209111	1.31131	0	0	0	0	0.67755	-0.73076
ENSG0000	RP11-365	1:2242196	0.226848	0	0.134742	0.085524	0	0	0.433737	0.757782	1.09725	0.458106	0.16785	2.797
ENSG0000	DEPDC5	22:317539	6.29171	7.58212	3.2941	3.11302	1.6625	5.17689	4.55116	5.80199	4.40511	5.33695	0.7222	0.177038
ENSG0000	ATP7A	X:7791065	1.16212	1.47034	0.424203	0.707379	0.581633	1.50431	0.967635	1.23627	0.159121	2.19827	0.58305	0.391777
ENSG0000	ZBTB6	9:1229080	1.18257	0.439126	1.07824	0.651528	1.16832	3.11731	1.22598	1.74892	0	2.64787	0.30215	0.728605
ENSG0000	SEC23A	14:390319	26.2568	31.6248	13.1134	14.0094	6.22369	11.9481	14.9124	10.927	19.2062	8.75502	0.37475	-0.31212
ENSG0000	GPRIN3	4:8923638	9.9572	4.99206	7.11037	5.85963	8.54402	8.42066	11.0549	12.0162	12.1795	17.8986	0.31765	0.386138
ENSG0000	ABHD2	15:890870	2.78663	5.75693	2.52582	2.15025	1.38609	1.92257	4.4687	3.52611	1.69616	5.82697	0.9754	-0.01648
ENSG0000	AC034220	5:1321848	0.835482	0.971913	1.46506	1.1997	0.327095	1.91228	0	0.648821	0.261987	0.963875	0.7565	-0.33483
ENSG0000	RHEBP2	10:476897	0.476634	1.31356	0.212143	0.607642	0.486845	1.0063	0.763129	0.294133	0.328571	0.198825	0.9403	-0.1345
ENSG0000	SMIM2-AS	13:441063	0	0	0.352437	0.607197	1.52763	0	0.921017	0	0	0	0.3914	-1.95505

ENSG0000(	AVEN	15:338662	15.2482	5.4614	2.46577	0.491034	3.54107	9.42792	12.3918	1.11944	0	2.93772	0.92005	-0.08603
ENSG0000(	EYA3	1:2797034	3.87224	4.27921	4.2894	2.83748	2.78921	4.14936	0.99289	3.1296	1.88528	6.20911	0.47385	-0.39228
ENSG0000(	CHTF18	16:784973	8.32894	18.2844	3.00133	4.41986	3.67788	6.43885	10.8635	5.78196	7.09968	5.57449	0.7769	0.219078
ENSG0000(	BNIP3L	8:2637925	30.6212	44.5156	30.7453	40.2312	22.1221	64.3077	31.1683	38.8952	34.8074	23.3713	0.8437	0.102704
ENSG0000(	PPM1B	2:4416762	58.3124	58.5336	36.2358	53.5648	23.9271	38.4074	40.1224	39.694	44.7067	35.3758	0.58275	-0.17284
ENSG0000(	GPR155	2:1744315	5.90465	9.2875	1.95172	0.725166	1.40902	3.76626	7.98966	4.6864	1.88127	6.03115	0.46865	0.356025
ENSG0000(	ZNF669	1:2470999	4.96878	4.1824	5.24251	8.35114	4.12489	0.295023	4.1373	4.45927	4.28153	4.22689	0.1824	-0.80179
ENSG0000(	SLC25A16	10:684779	2.19628	1.90353	1.45058	1.58439	1.05481	4.04822	1.85856	2.3098	1.19088	3.43733	0.55135	0.372482
ENSG0000(	NOG	17:565936	0.062016	12.8813	1.69466	1.82319	0.433427	0	0	0	0	0.210357	0.016	-6.00551
ENSG0000(	AC003075	7:1727983	2.2954	3.6591	2.64322	5.07455	0.565691	7.17753	5.24099	8.77979	9.2516	0.149511	0.5356	1.09185
ENSG0000(	RP11-843I	12:321041	0.244446	0.462857	0.078597	1.50007	0	0	0.083958	0	0	1.06781	0.85935	-0.47953
ENSG0000(	MEGF8	19:423526	0.125973	0.130496	0.257508	0.071106	0.360042	0.084433	0.710941	1.03349	0.200622	0.410978	0.06865	1.49447
ENSG0000(	CNN3	1:9489694	0.229296	0.753823	0	0	0.219799	0	0	0.114498	3.85288	0.298394	0.1362	2.5837
ENSG0000(	RPS29	14:495709	885.712	685.389	1632.18	1108.86	203.882	849.928	983.87	824.857	475.807	40.9074	0.64715	-0.52434
ENSG0000(	RP11-264I	16:289508	0.764697	0.743786	0.181863	0.248388	0	1.14885	1.16808	0.104407	0.542337	0.057053	0.41415	1.02218
ENSG0000(	CD101	1:1170017	1.62478	9.95347	3.04851	1.87232	3.0637	4.75752	20.3622	17.2358	8.61618	17.0319	0.0015	2.03417
ENSG0000(	PGM3	6:8306766	4.10445	2.9081	5.30561	2.84317	2.93598	3.12457	5.26534	5.58976	6.07267	5.59193	0.6694	0.248283
ENSG0000(	ZNF213	16:311045	2.99385	2.99469	2.08808	0.830086	1.32894	4.22053	1.75765	2.78341	0.435641	5.52958	0.628	0.47738
ENSG0000(	RPL10	X:1543899	949.038	1011.52	1875.2	1639.73	1721.93	644.397	934.631	1151.74	1228.27	276.13	0.11215	-0.79704
ENSG0000(	RP3-395M	1:2581559	4.11001	3.76982	2.73879	3.39696	1.50592	3.52859	3.44779	5.49428	0	0.506953	0.76585	-0.30166
ENSG0000(	FGF5	4:8026659	0	0	0	0	0	0	0	0.002074	3.51776	0	0.11875	inf
ENSG0000(	ARL6IP4	12:122920	252.222	239.97	219.961	237.346	268.476	321.711	310.667	190.13	211.739	99.5988	0.8421	-0.087
ENSG0000(	RAB4A	1:2292582	19.957	15.3525	18.5095	15.6619	15.2718	18.8564	19.3081	13.3636	13.7831	7.24136	0.1831	-0.62283
ENSG0000(	SLC12A2	5:1280837	2.82355	1.89423	2.65706	1.71372	2.53697	1.77976	0.483055	3.32976	1.61042	5.20087	0.8852	-0.06846
ENSG0000(	KIAA0895I	16:671701	1.2653	0.347966	0.168778	0	0.735889	3.3669	1.24638	1.26467	0	3.65826	0.0357	2.25122
ENSG0000(	WWTR1	3:1495172	0.576826	3.04178	0.112104	0	0.016718	0	5.00059	0.106915	0	0.927575	0.5983	0.615092
ENSG0000(	RN7SL449	17:572558	0	0	0	0.432079	0	0	0.825023	0	3.03817	0.220088	0.2354	3.61762
ENSG0000(	FPR3	19:517451	0	0.77634	0	0	0	12.0534	0	1.15836	0.152582	0	0.2699	4.12636
ENSG0000(	CD8A	2:8678460	168.746	180.978	260.198	336.443	280.791	188.305	499.272	506.862	283.872	355.008	0.1683	0.565495
ENSG0000(	CBWD5	9:6566880	29.9076	22.3791	17.0047	23.3896	17.8766	34.7607	25.5743	19.1217	39.4641	26.6354	0.30575	0.526573
ENSG0000(	F8A3	X:1554569	23.9701	39.5293	32.5779	24.7552	39.9575	24.6865	33.0423	31.1645	21.5572	29.8282	0.9302	-0.07134
ENSG0000(	PPIL3	2:2008709	22.6792	20.776	30.7798	24.2019	11.2201	23.014	32.9455	28.8718	18.81	13.8779	0.6873	0.16063
ENSG0000(	VMA21	X:1513965	13.2143	9.51378	11.6156	8.31844	8.07947	19.1314	11.9392	19.2152	22.7071	12.3821	0.1466	0.67614
ENSG0000(	SNX8	7:2251769	3.73858	6.48011	2.91105	4.50535	2.75105	20.5826	10.6929	6.51035	9.21175	3.08336	0.1912	1.05581
ENSG0000(	SDCCAG8	1:2432560	13.5767	31.682	15.8487	11.8166	11.5637	28.5823	11.8101	16.0537	14.5536	18.9184	0.8315	0.095358
ENSG0000(	NSFP1	17:463728	15.8461	20.0471	6.10542	10.5549	4.54423	8.56495	14.4895	3.49354	5.48968	6.82399	0.59675	-0.54935
ENSG0000(	GTF3C4	9:1325929	1.019	1.00807	0.635915	0.557933	0.834426	0.176436	0.57532	0.799267	0.23111	1.50212	0.60425	-0.51818
ENSG0000(	SUSD3	9:9305868	18.4029	88.2052	36.3179	21.0491	20.4432	61.0578	114.901	69.8506	57.439	29.3785	0.03245	0.884845
ENSG0000(	MAPKAP1	9:1254373	41.6323	45.458	37.388	30.0796	35.6341	27.1118	30.6198	33.8511	14.4073	37.9353	0.1436	-0.53964
ENSG0000(	TMEM39A	3:1194294	10.8629	9.34033	11.0426	6.55314	7.29652	8.83629	6.52583	8.64455	19.3713	13.3585	0.7728	0.145308
ENSG0000(	CLN3	16:284563	40.4865	47.0368	54.3905	51.3921	47.4428	52.394	60.6491	51.2851	38.9869	65.3698	0.8708	-0.07628
ENSG0000(	HGS	17:816811	69.9367	107.075	33.7079	49.5648	31.9803	54.6992	58.0384	33.016	22.7805	38.6784	0.36395	-0.49582
ENSG0000(	DPY19L3	19:323900	1.85345	2.07516	0.310026	0.299896	0.328738	3.9736	1.12603	1.50807	0.154509	1.28834	0.43995	0.599678
ENSG0000(	FAM184A	6:1188134	1.052	5.96815	0.480865	0.061196	0.543673	2.41657	4.09505	1.54673	3.14103	1.68997	0.22975	1.16937
ENSG0000(	RP11-62J1	7:1282976	0.709113	0.788036	1.14885	0	0.97509	0	1.1932	1.08868	0	0.227193	0.68005	-0.7728
ENSG0000(	TXN	9:1102438	209.225	117.274	192.867	181.14	89.2314	409.111	405.071	238.307	264.695	23.8245	0.04175	0.724712
ENSG0000(	VMO1	17:478528	0	0.317641	0.546909	0	0.127612	2.18293	1.42509	0.818781	0	1.14561	0.1205	2.46947
ENSG0000(	IGHV2-5	14:106037	0	3.28542	0.302815	0	0.100496	0	0	0	0	0.129409	0.2581	-3.84973
ENSG0000(	NKRF	X:1195744	4.62769	6.34938	2.02835	1.14286	2.60096	2.74693	5.08978	3.76601	3.67962	2.84803	0.89895	0.198062
ENSG0000(	FTL	19:489653	3344.01	2914.04	4893.74	3403.73	5255.31	13208.2	2381.2	2591.3	4514.04	1376.34	0.8222	0.189654
ENSG0000(	ADAMTS1	19:858024	1.4089	4.99093	0.715744	1.00589	2.7355	0	1.33026	0	5.94136	3.02583	0.8489	0.154941
ENSG0000(	E2F8	11:191820	0.641141	2.88286	0	0	0.048561	1.98991	0.894732	0.761399	0	0.372838	0.8895	0.182341
ENSG0000(	FAM134A	2:2191718	12.5493	18.3294	7.01698	4.75359	12.174	15.0144	12.6191	10.4891	3.77935	14.5802	0.7871	-0.18787
ENSG0000(	RP11-24C	3:4843216	0	0.697567	0.220603	1.00965	0	0.937999	0.805353	0.31955	0.343278	0.218377	0.74185	0.503091
ENSG0000(	TSC22D4	7:1004566	20.8211	21.0046	24.4432	18.033	50.2993	25.2279	37.7672	31.7441	15.9971	63.302	0.43085	0.329284
ENSG0000(	PLD4	14:104924	0	9.91159	2.02757	2.37234	0.84909	1.97923	0	0	0.33702	0.204611	0.14355	-2.32938
ENSG0000(	SCD5	4:8262953	2.26863	0.606499	6.48444	2.44522	4.70489	1.1297	0	2.61057	0	3.24085	0.3113	-1.40379
ENSG0000(	CDH24	14:230470	1.05701	0.521404	0.122487	0	0.2531	0	2.10535	0.128523	0.071174	1.51083	0.49105	0.806482
ENSG0000(	SECTM1	17:823210	1.97068	9.36133	0.607641	0.059004	0.735815	28.6242	4.01731	7.42991	0.402053	0.460141	0.029	1.70977
ENSG0000(	PDE6G	17:816504	2.31952	1.88955	2.2215	10.5948	3.09921	3.38388	1.78661	1.92912	0	0.36321	0.084	-1.61993
ENSG0000(	C1orf56	1:1510477	3.55008	6.37581	32.9667	53.4074	11.001	1.37471	2.36247	17.2745	49.446	22.6122	0.76225	-0.35271
ENSG0000(	PJA2	5:1093347	26.4629	33.3592	7.7769	12.5024	9.09102	16.2757	17.922	13.2961	12.3548	20.0793	0.66375	-0.14988
ENSG0000(	AC015971	2:8650343	0.631756	0.430315	0.114041	0.053	0.117202	0	0	0.050588	2.07084	0.325435	0.57305	0.955728
ENSG0000(	PPP6R2	22:503433	22.7805	24.8377	20.0869	20.0448	18.5793	22.6395	25.4115	19.0968	14.1539	24.0539	0.99465	-0.00332
ENSG0000(	IKBKB	8:4227130	26.3725	36.844	10.2486	9.5008	13.5232	27.4907	32.3022	17.8165	10.0486	21.6669	0.36995	0.341117
ENSG0000(	LINC0086:	10:873427	2.07595	1.13685	1.46844	1.45145	1.18765	1.3409	1.06447	1.33211	2.4923	1.15782	0.93725	0.061866
ENSG0000(	FUT7	9:1370072	14.173	8.06596	2.02414	5.33582	2.611	4.7791	6.04046	3.10615	5.19314	1.05412	0.5665	-0.69763
ENSG0000(	PPP4R3B	2:5554729	51.5577	44.4208	15.1206	25.7902	20.6192	24.3209	29.8583	25.9464	19.5337	29.8173	0.44035	-0.28959
ENSG0000(	CTD-2035	5:4301441	1.05949	0.891913	1.2866	1.51343	0	0	0	0	0.324374	0	0.2712	-3.39023
ENSG0000(	FBXO46	19:457106	7.2058	12.1056	1.6644	1.41469	2.09237	4.10472	4.69647	1.85737	0.739245	3.43996	0.2032	-0.82445
ENSG0000(	KCNQ5	6:7262179	0.299748	1.40708	0.202644	0.256514	0.095527	0.33174	0.005528	0	0	0.383128	0.14715	-1.99218
ENSG0000(	ZNF574	19:419983	14.3161	20.2128	14.7677	18.4144	14.5527	4.53838	17.7727	10.8611	7.74839	16.9868	0.0921	-0.63599
ENSG0000(	MRPL42	12:934674	60.0398	40.4592	56.1811	60.7244	36.3117	66.0707	51.3804	58.698	45.8312	23.8278	0.87225	-0.06686
ENSG0000(	FTSJ1	X:4847602	9.12072	11.0639	59.4098	53.3271	47.2513	9.87025	7.45004	38.9897	35.4581	35.8642	0.3201	-0.50997
ENSG0000(	CDK2AP2	11:675064	162.774	166.292	391.847	277.881	464.679	156.195	279.526	282.711	274.301	171.707	0.3155	-0.35043
ENSG0000(	FMNL3	12:495682	1.9716	2.93763	0.549295	0.323146	1.03589	2.56273	3.64627	2.47878	1.62253	7.86475	0.23635	1.2436
ENSG0000(	CACNB2	10:181406	0.322645	1.95397	1.05752	0.089875	0.160536	0.855476	2.27929	0.280253	0.271593	0.565119	0.90145	0.113497
ENSG0000(	SNX18	5:5451775	1.33584	3.034	1.31264	2.07829	1.33869	6.88921	2.27381	1.85368	3.07323	3.76203	0.34535	0.715456
ENSG0000(	VPS33A	12:122203	13.0939	16.4153	15.84	9.71195	11.9224	24.9631	16.4764	13.7595	15.6872	11.3265	0.61995	0.257339
ENSG0000(	RP11-285I	1:2258100	5.50766	21.2931	2.98966	2.8375	6.61216	1.90492	2.27076	2.20715	0.625374	5.32215	0.3555	-1.14973
ENSG0000(	PF4V1	4:7385318	0	0	0.283046	1.39424	0.088908	0	0	0	0	0	0.00365	#NAME?
ENSG0000(	RP1-151F:	6:1676434	0.197722	0	0.52279	0.28509	0.111951	1.09584	3.26526	0.679787	0.123229	1.79774	0.16745	2.43626
ENSG0000(	ATAD2B	2:2374866	10.7526	9.24512	2.5416	3.74743	3.66212	10.1898	9.35238	2.615	5.55937	9.32331	0.66215	0.297624
ENSG0000(	RP1-111D	6:1492179	0.629939	0	0.17624	0.713454	0	3.89992	0.147985	0.430716	0	0.02305	0.38395	1.57538
ENSG0000(	RPL18AP7	3:3849612	0	0.465258	0.393528	1.47301	0.242797	0	0	0.086818	0	0.364421	0.2508	-2.16857
ENSG0000(	CXCL8	4:7374050	8.15662	9.15989	0.376331	1.52349	0.895167	2.27918	0	10.6792	23.7669	1.87453	0.2104	1.08082
ENSG0000(	ASB1	2:2384267	15.1736	10.1436	19.7337	26.3535	21.9418	26.9075	20.8731	19.6893	7.553	19.2282	0.8611	-0.09756
ENSG0000(	TTLL5	14:755748	8.1435	9.75029	2.73607	2.69957	3.38936	2.55775	6.51931	0.859436	5.5932	2.89771	0.55585	-0.46788
ENSG0000(	LINC0155(	14:979256	15.9415	47.685	26.9688	19.995	16.6758	21.0411	8.92127	8.67671	15.2966	14.9901	0.0725	-0.94127
ENSG0000(	LRRC28	15:992513	5.6349	11.691	5.16904	4.80391	6.2776	5.8551	4.75956	8.73051	7.36145	3.9842	0.5826	-0.28826
ENSG0000(	AIDA	1:2226680	6.74311	7.83773	4.88765	5.33443	6.96474	4.42009	6.37262	7.60381	8.69836	8.3224	0.89105	0.112112

ENSG000C	ZNF594	17:517953	2.24219	3.40645	0.97744	0.778418	0.725631	3.14212	1.77847	1.37507	0.425496	2.55795	0.95365	0.088237
ENSG000C	ZFP64	20:520516	5.5974	6.034	3.64287	4.04802	4.71461	4.88629	3.77856	4.0996	3.58766	4.31068	0.72685	-0.18131
ENSG000C	ZSCAN21	7:1000497	10.0373	6.76358	13.9646	10.6671	12.7723	5.32408	3.46909	9.16927	10.7117	11.5613	0.5103	-0.45794
ENSG000C	BCDIN3D	12:497911	4.16055	4.57531	6.39623	4.20457	4.74349	3.86345	5.13925	2.50601	0	4.07487	0.30585	-0.70296
ENSG000C	ANXA2P2	9:3362427	3.89624	2.53862	1.34468	2.05506	0.778009	3.73855	3.6399	1.81635	2.03674	0.37205	0.81785	0.164779
ENSG000C	SDHAP3	5:1572221	2.54308	1.30837	0.870305	1.39025	0.737254	1.78046	1.39471	1.14308	3.10425	0.748643	0.80035	0.311968
ENSG000C	TFAP2A-A	6:1039318	0.466016	0	0	0	0	2.98876	0	0	0	0.195097	0.3539	2.62779
ENSG000C	BTG1	12:919849	185.436	319.592	190.481	249.596	194.793	110.871	143.633	184.55	205.606	169.706	0.29735	-0.56229
ENSG000C	SEC14L1P:	11:438808	0.720784	1.33401	1.68385	0.37253	0.480136	1.04543	0	0.926763	3.72069	2.178	0.6969	0.821546
ENSG000C	C7orf73	7:1356624	42.1628	46.7376	40.9779	41.0952	21.9619	47.4887	37.9698	40.6524	32.8953	10.197	0.62845	-0.1617
ENSG000C	TMEM217	6:3721217	1.17324	0.097594	0.804107	0	0.847551	5.93332	1.65156	1.59796	0	0	0.25415	1.46842
ENSG000C	AC025442	5:6613997	1.14292	2.04015	1.83699	1.72936	2.09798	2.40634	1.17805	1.20681	3.60253	0.951193	0.7848	-0.38269
ENSG000C	MYO19	17:364866	15.8008	15.0667	7.921	6.3454	5.79988	7.34611	9.71915	6.33569	11.9041	12.3054	0.93045	0.054551
ENSG000C	RRAGC	1:3883819	3.26011	3.95933	3.04441	3.39878	6.3267	7.54771	7.90727	6.35739	4.15796	8.6688	0.53435	0.716728
ENSG000C	RP5-894A	7:1415292	0.497569	2.02485	0	0.345085	0.25447	0	0.229348	0	0	0.376905	0.26605	-2.31197
ENSG000C	FCRLB	1:1617215	1.19332	1.29869	1.69715	2.80305	2.46836	3.77238	1.032	0.900768	0	0.602076	0.5102	-0.72463
ENSG000C	APITD1-C	1:1042988	0.943151	1.07467	0.826407	0.120792	0.254962	0.084916	2.44452	0.449785	0	0	0.4979	-0.82485
ENSG000C	MRPL23	11:194727	28.4972	26.8622	56.6608	55.3816	43.9657	37.333	45.4573	49.2839	37.1869	6.25285	0.434	-0.37931
ENSG000C	F8	X:1548357	2.8593	1.65915	6.46117	7.11661	4.26357	4.21962	5.33421	8.12063	15.0617	5.26155	0.51405	0.703665
ENSG000C	ARPC1B	7:9932589	882.943	925.739	899.739	1136.65	1283.07	1231.91	945.16	757.754	860.436	607.972	0.5066	-0.30126
ENSG000C	WBP1LP2	7:1075795	0.600517	0.755867	0.528337	0.604393	0.287478	1.18996	0	0.596013	0	0.772974	0.8895	-0.19857
ENSG000C	CREBBP	16:372505	7.90549	7.45823	9.95336	4.86249	23.9523	19.71	9.04384	11.9044	7.55141	44.035	0.26525	0.515518
ENSG000C	UBE2A	X:1195744	57.2323	54.5477	35.1405	27.8118	34.2179	79.98	87.405	57.6425	30.4451	30.8838	0.17995	0.458408
ENSG000C	ERF	19:422301	0.525802	0.512327	2.84744	1.49971	7.31905	0.901189	1.66431	4.40174	1.04043	8.33933	0.8525	0.180216
ENSG000C	RNF8	6:3735397	22.4861	16.276	24.1478	10.9815	21.4837	33.7852	9.83348	13.3164	8.05869	16.7883	0.3674	-0.39529
ENSG000C	SH2D1A	X:1239592	137.895	61.9263	144.313	88.8864	81.4865	81.9316	57.7066	87.6	48.8329	47.5066	0.0625	-0.76156
ENSG000C	ERCC1	19:453796	25.8718	27.6628	50.4628	41.6391	46.8664	53.866	26.3749	48.4453	30.809	34.0546	0.9061	-0.07701
ENSG000C	MAP1LC3/	20:345468	6.72875	3.44797	2.58292	4.75546	3.51156	17.9235	10.3431	13.4481	17.8882	5.78929	0.00515	1.82613
ENSG000C	PDHX	11:348530	15.5897	18.7618	10.0535	14.0397	9.18366	21.4	10.0111	7.59232	3.02974	7.8132	0.31875	-0.52912
ENSG000C	MCPH1-A	8:6403550	0.552184	0.79763	0	0	0.247991	3.79721	0.664691	1.11361	2.39747	0	0.04915	2.60322
ENSG000C	IGHM	14:105851	12.1673	650.553	47.0528	3388.01	41.1871	3.91836	3.0893	5.48498	6819.82	47.7972	0.2611	1.02286
ENSG000C	CLUHP3	16:317005	11.3612	10.6278	20.3874	22.9215	15.6924	13.3908	16.6815	29.3459	25.0821	44.8009	0.14645	0.621491
ENSG000C	TFEB	6:4168397	9.59413	22.8731	2.5633	2.53805	5.3511	15.9516	11.7967	5.26108	1.16933	5.26897	0.7249	-0.2143
ENSG000C	SURF4	9:1333614	131.194	79.1167	97.7152	95.7998	97.7715	184.467	204.183	144.069	115.303	143.245	0.0698	0.599017
ENSG000C	CTD-2583	19:577696	0.187654	0	0.502543	0	0	0	0	0.062583	1.24941	0.5608	0.43745	1.98734
ENSG000C	CCNB1IP1	14:203113	4.06903	5.43486	6.46155	5.04944	4.89539	3.30392	9.65728	13.5518	5.30283	5.6257	0.3356	0.550908
ENSG000C	VTA1	6:1421471	55.956	52.2817	45.1763	36.5356	32.658	44.8284	53.5549	36.9389	39.2063	20.473	0.564	-0.22965
ENSG000C	GOLGA5	14:927942	26.2521	16.733	11.2432	13.1518	9.00578	9.89797	14.9015	9.91985	7.99714	10.1348	0.36645	-0.32938
ENSG000C	CTC-490E	19:407716	1.89847	1.54487	0.799571	1.03452	1.28458	1.34842	1.81966	1.41335	1.97487	1.13063	0.79605	0.286702
ENSG000C	TBK1	12:644515	37.6958	15.0365	9.49837	15.6957	6.87308	21.3492	19.8531	14.0005	10.9491	14.0184	0.9429	0.024461
ENSG000C	RP4-635A	1:1851570	0.617001	0.177268	0.461697	0.738403	0.207817	0	0	0	1.58921	0	0.8571	-0.34807
ENSG000C	DUSP11	2:7376218	43.7445	31.2757	61.7835	48.0907	41.2937	34.408	49.2279	45.0131	47.0009	21.1945	0.6048	-0.17541
ENSG000C	SMG7-AS:	1:1834608	1.4038	0.380542	0.473481	0.186112	0.249205	0.171875	0	0	0.833647	0.309916	0.5914	-0.90161
ENSG000C	MPG	16:77006-	35.0944	35.5705	54.5655	49.9942	41.4359	47.7149	58.3928	60.0005	47.9936	29.8479	0.82045	0.096124
ENSG000C	SMYD2	1:2142811	37.2471	30.6232	32.6905	33.7692	25.0674	18.6965	24.7709	18.5675	18.875	21.4595	0.1001	-0.62411
ENSG000C	TNFSF12-1	17:754889	0.45099	0.092428	1.47924	0.601217	1.32276	0.190756	0.007726	0.549541	3.8606	1.15896	0.58595	1.08491
ENSG000C	NUDT22	11:642066	58.9557	62.2523	66.1055	74.8143	78.7913	89.2759	73.0982	65.0694	70.9594	34.9819	0.92585	-0.08085
ENSG000C	CAPN1	11:651776	67.8918	68.8363	34.1832	34.6824	40.3092	41.9311	43.3361	34.4605	34.2458	46.6196	0.5347	-0.19396
ENSG000C	P2RY13	3:1510856	2.26426	10.6186	0	0.292717	0.03606	5.10591	0.024372	1.33027	0.108027	0	0.8024	-0.84761
ENSG000C	AC006486	19:422301	0.327523	0	0.249903	0.062492	0.02569	1.92444	0.021614	0.543657	0	0.032617	0.457	1.83945
ENSG000C	DHRS9	2:1690647	0	1.20531	0.381024	1.68478	0.011662	0	0.02132	0	0	0	0.235	-6.99267
ENSG000C	G6PC3	17:440707	8.47504	10.6001	8.2142	8.09435	10.8572	15.6129	8.39219	8.94936	4.26042	7.52109	0.9116	-0.06696
ENSG000C	LINC0011(	2:1102115	50.706	58.2624	78.3131	70.7414	31.4696	85.8894	97.681	57.0761	34.0468	15.2433	0.86425	-0.08423
ENSG000C	ZCCHC4	4:2531278	10.7954	8.19875	7.33988	4.11704	6.04829	10.1723	2.92324	8.31246	12.2673	5.66612	0.89275	0.066126
ENSG000C	SLC52A1	17:503259	0.188637	0	0.065575	0.530493	0.086543	1.7649	0.033022	0.234507	1.43101	1.34954	0.02665	2.59539
ENSG000C	WDTC1	1:2723451	1.24986	1.56416	3.63015	0.769819	8.27571	0.36541	1.69132	5.68833	1.9501	15.2987	0.37985	0.538076
ENSG000C	ZNF816-Z	19:528618	1.69875	0.692938	0.471654	0.297294	0.828294	0.798398	1.59871	0.54214	0	1.59188	0.92115	0.176022
ENSG000C	NDUFA11	19:586582	192.311	144.272	222.865	232.957	99.291	248.593	275.345	202.884	178.354	32.3596	0.9123	-0.04513
ENSG000C	CTD-2371	11:945955	9.18224	2.89733	4.83601	5.64981	3.04892	12.0168	8.82752	7.23597	15.7949	4.27075	0.5074	0.971916
ENSG000C	LBX2-AS1	2:7449751	2.90567	0.984044	0.767046	0.243964	1.27867	8.49152	2.00869	0.768416	1.58973	0.459868	0.5347	1.08514
ENSG000C	RGL2	6:3327713	33.9884	41.7076	6.76426	5.89269	5.70614	30.5256	17.5687	7.83427	4.46345	20.8231	0.89815	-0.10845
ENSG000C	GRAMD1E	11:123358	7.90767	5.39905	7.39244	7.86145	3.32434	16.0487	13.3284	14.3536	3.45314	5.50859	0.63005	0.536628
ENSG000C	NUPL2	7:2318182	55.6348	36.5827	43.0555	28.7997	37.9057	42.9158	49.6979	30.1914	43.9868	22.3338	0.9092	-0.05189
ENSG000C	HSPE1	2:1974865	118.491	113.636	172.429	174.079	100.729	273.431	234.189	184.791	128.648	64.5564	0.61725	0.337381
ENSG000C	GPR19	12:126608	2.79208	1.63828	0.699849	1.3592	0.748088	1.84429	2.74631	1.2115	3.51402	2.01272	0.4674	0.729995
ENSG000C	POLR2I	19:361111	60.0833	56.7877	104.044	106.216	48.9371	78.1977	71.5412	88.3253	68.1902	11.9188	0.71155	-0.32225
ENSG000C	PSMC3	11:474187	86.2215	56.3002	163.95	147.059	154.525	105.399	123.024	142.074	124.857	90.7665	0.79895	-0.08241
ENSG000C	TPST2	22:265219	121.423	87.9967	117.476	112.822	110.214	91.2109	150.032	56.7962	33.1802	58.074	0.041	-0.71921
ENSG000C	ULK3	15:748361	77.8808	72.574	38.0976	77.1478	34.1956	47.1963	56.4891	31.3782	23.8331	41.2037	0.04185	-0.60526
ENSG000C	FUBP1	1:7794405	39.6121	37.2714	25.7786	21.614	28.7673	38.1309	52.1495	36.9039	16.8541	44.1455	0.28595	0.449753
ENSG000C	NMD3	3:1610838	21.1014	20.3226	11.5959	14.0942	7.54297	19.8744	15.5773	13.2718	13.116	11.1571	0.886	-0.0462
ENSG000C	TRAV26-2	14:222025	4.57504	0.719377	0	2.85823	0	6.646	0.252277	0.149238	0.186436	0	0.9113	-0.25046
ENSG000C	FCGR1CP	1:1438747	0.418339	0.025621	0.086279	0	0.063434	8.15714	0.0145	1.27772	0	0	0.3187	3.78997
ENSG000C	PTPRJ-AS:	11:479805	0.435941	0.5601	0	0	0	2.41771	0.643402	0	0	0.498317	0.34535	2.25257
ENSG000C	TMEM30B	14:611875	1.19904	0.749458	1.05954	0.553053	0.628974	0	0.105348	0	0	1.18109	0.2956	-1.86675
ENSG000C	HIP1	7:7553329	2.63063	1.44534	1.34509	1.59254	0.752121	1.95039	3.70487	1.13064	2.43233	4.9983	0.369	0.797523
ENSG000C	TRIOBP	22:376816	20.4388	24.7634	12.688	4.68547	12.6244	31.0735	23.2166	16.8486	5.12776	17.3769	0.6913	0.312723
ENSG000C	ATP10A	15:256772	1.72481	12.4117	0.418605	0.565212	0.552286	4.13929	6.483	0.999185	0.336491	3.14465	0.8954	0.114887
ENSG000C	EXT2	11:440955	14.5664	15.669	10.9403	10.7229	7.49102	3.07246	11.7358	9.49526	12.1375	10.3272	0.3259	-0.37333
ENSG000C	PDCL3	2:1005626	47.1141	40.466	45.5741	51.8737	38.3111	87.9569	82.247	63.9761	75.741	20.6098	0.1231	0.560007
ENSG000C	ZNF529	19:365106	3.85648	4.87686	3.42374	3.08729	2.3338	2.43314	2.08685	4.26058	2.82191	5.88116	0.963	-0.03169
ENSG000C	RP4-614C	20:351720	1.41233	1.15249	1.41828	0	0.214084	0.900185	1.16611	1.71484	1.96252	1.62404	0.5373	0.842871
ENSG000C	STK10	5:1720420	147.09	159.796	51.7026	58.6237	68.2416	69.0911	86.2335	69.6402	40.2036	91.071	0.29125	-0.39949
ENSG000C	PAK6	15:401610	0.98441	1.15122	0.884961	0.786424	0.577875	0	0.883083	0	0.179203	0.942589	0.272	-1.00029
ENSG000C	ZNF384	12:666325	18.9754	23.0951	11.1385	14.2824	7.45785	17.1364	13.1864	9.4642	7.22924	15.0805	0.36635	-0.41549
ENSG000C	ESR1	6:1516566	0.271689	1.06975	0.272449	0.261331	0.104553	0	1.51563	0.509971	0	0.449718	0.9662	0.054984
ENSG000C	EIF2AK4	15:399341	8.78786	9.84571	4.5954	5.65995	6.96576	6.32985	5.33984	3.4754	3.06464	7.59221	0.56455	-0.4552
ENSG000C	MAFG	17:819119	5.50337	7.58679	4.87614	5.48872	7.82327	28.881	20.4005	15.9018	35.2336	24.9731	0.13865	1.90641
ENSG000C	PINK1-AS	1:2063345	0.643754	1.96997	0.598912	0.399783	0.488624	0.590987	1.03997	0.482277	0.116661	1.89246	0.9467	0.10413
ENSG000C	RP11-546.	2:1578767	0.112404	0.641931	0	0	0	3.07631	0	0	0	1.20568	0.49665	2.55185
ENSG000C	TCEA3	1:2338106	0.996641	21.5099	1.78715	2.07231	1.15583	0	0	1.77834	0.428026	0.829389	0.038	-2.97509

ENSG0000	FTX	X:7394432	6.19217	7.35155	6.95163	2.29856	7.27493	3.87533	7.3965	11.0203	1.28559	10.3051	0.8045	0.185533
ENSG0000	ST3GAL1	8:1334548	34.7584	37.1093	33.6685	24.2977	32.3811	16.7371	39.1219	35.9127	18.8505	64.4452	0.37975	-0.31294
ENSG0000	GNPTAB	12:101745	21.0027	22.2249	14.0781	18.0843	24.6812	28.2933	37.5047	14.9283	5.81469	31.6208	0.57865	0.241146
ENSG0000	HAT1	2:1717843	69.8319	53.1907	49.54	48.7066	40.9182	60.8556	65.8929	48.8323	36.8594	27.9823	0.8707	-0.06224
ENSG0000	DPH1	17:203010	31.1764	38.8836	41.9343	68.9648	30.5731	22.6921	34.6101	40.0282	32.133	38.1275	0.34275	-0.41146
ENSG0000	RASA4B	7:1024824	17.0759	15.358	18.8006	10.571	16.3622	13.8202	12.1819	11.8695	9.92302	14.2346	0.6576	-0.44745
ENSG0000	NISCH	3:5245511	23.9138	22.9042	12.6065	14.6663	10.8389	16.6891	12.9504	12.5825	11.8697	21.6087	0.6984	-0.14139
ENSG0000	JRKL	11:963899	4.28846	1.3056	1.41457	1.95041	1.37119	5.67006	3.90212	3.38378	6.33975	3.38216	0.02175	1.18418
ENSG0000	TRAPPC6E	14:391478	10.4902	8.38547	8.64439	5.11049	7.4923	5.16898	9.47929	16.8942	12.8446	19.5758	0.20415	0.55341
ENSG0000	INVS	9:1000992	5.00262	7.25038	3.15307	3.26167	3.03125	4.03706	1.51004	1.44363	0.010698	5.56517	0.1414	-0.93017
ENSG0000	SPATS2L	2:2003058	43.6597	17.1318	36.0681	22.3366	60.2531	50.3253	139.256	52.8414	38.9942	49.1818	0.0624	0.714211
ENSG0000	TP53TG1_	7:8732522	1.76844	1.09188	6.41954	2.92919	0.817233	0.990375	0	3.16772	2.58302	0	0.89255	-0.15793
ENSG0000	SNAP23	15:424912	56.9685	81.4355	67.0904	64.4966	36.4421	47.0485	53.1278	51.4703	60.9895	36.7822	0.44895	-0.24792
ENSG0000	LIG4	13:108207	5.06045	6.6925	1.81239	1.73984	1.50224	2.48027	3.5623	1.92286	0.095093	4.84285	0.5198	-0.43142
ENSG0000	FAM58A	X:1535879	22.7657	14.2487	35.0441	29.385	29.5657	13.9588	25.4323	35.3834	25.8263	9.94609	0.52405	-0.27876
ENSG0000	RAB21	12:717548	13.3447	13.6253	1.21576	0.776537	1.27692	12.6248	16.5461	1.8984	3.61994	3.5416	0.6675	0.395572
ENSG0000	MLF2	12:674799	110.77	136.979	116.311	98.2841	128.802	139.336	167.315	106.822	95.5757	86.3245	0.9791	0.008729
ENSG0000	IL21	4:1226126	0	0	0	0.546802	0	10.313	6.69987	8.12099	10.0766	2.41262	0.1665	6.05537
ENSG0000	AC004057	4:1128181	29.8071	35.2872	5.28904	7.46056	3.13145	36.4057	55.2546	6.29236	4.86669	0.458534	0.66715	0.297631
ENSG0000	SEPSECS-/	4:2516064	5.5563	3.37911	4.6414	6.06272	2.16492	3.23034	2.08861	1.72578	3.80176	2.33981	0.5946	-0.75289
ENSG0000	APOOL	X:8500382	1.94014	3.10294	0.665742	0.347669	1.53644	0.652363	0.495206	0.66949	1.1445	1.65639	0.48865	-0.87019
ENSG0000	GTF2H4	6:3090818	4.73655	5.83689	2.97017	5.59487	6.47582	3.31076	5.18122	8.88643	4.36128	5.80154	0.76175	0.242135
ENSG0000	RP11-362	4:1413194	0.370543	0.397772	0.051534	0	0.358431	2.76253	0.069543	0	0	0.04582	0.5033	1.0825
ENSG0000	TMEM147	19:355334	5.15987	8.79996	3.33246	3.37601	2.0577	6.02749	3.83324	5.18239	0	4.66464	0.93425	-0.08771
ENSG0000	HNRNPA1	17:205415	1.68343	0.607188	0.554416	0.17403	0.406706	0.159952	0.829379	1.7017	1.33672	2.15633	0.4753	0.97218
ENSG0000	ZNF606	19:579770	5.89368	14.6912	4.78034	2.52621	2.55033	0.925544	7.51237	7.4512	6.10198	6.65652	0.9395	0.045429
ENSG0000	ZXDC	3:1264376	15.4855	22.257	10.978	10.0599	7.50007	9.15599	14.5755	5.7861	4.37866	12.7714	0.2969	-0.47865
ENSG0000	OXLD1	17:816650	70.0241	75.2357	85.5195	94.7812	74.6329	68.463	53.7923	34.8895	88.2472	47.5949	0.2723	-0.51739
ENSG0000	LRFN4	11:668482	1.62561	0.796182	0.046798	0.064738	0.469248	5.30998	8.47649	1.85339	0	2.27528	0.2154	2.40237
ENSG0000	ZNF257	19:220524	1.28597	0.10858	0.040002	1.5586	1.08698	5.69933	0.480023	0.379371	1.18568	1.72234	0.46025	1.1518
ENSG0000	RP11-770.	11:118359	16.1274	21.4284	4.55128	4.97082	0.17843	10.2386	11.3686	5.6796	2.06274	0.431061	0.71725	0.670128
ENSG0000	GPR137	11:642698	11.429	9.21201	3.92577	3.20044	7.1891	7.21931	5.56035	6.70421	4.01746	6.09444	0.71115	-0.2428
ENSG0000	PLEKHB2	2:1311048	34.6648	27.5239	26.6634	34.9754	31.7607	22.0982	26.5461	34.2275	38.9021	44.787	0.89765	0.045712
ENSG0000	TMEM8A	16:370772	27.5656	41.6159	6.60554	2.69337	13.0018	21.2337	27.3567	7.63917	1.29125	29.1359	0.5637	-0.25288
ENSG0000	TRAV8-3	14:218525	12.6747	31.106	14.2007	4.51406	9.18274	13.1251	21.294	10.2154	1.52523	10.3697	0.83785	-0.15961
ENSG0000	RP11-147	11:747488	2.62625	0.701591	1.63856	0.975768	1.02515	0	1.1233	0.523863	0.395185	1.06028	0.40105	-0.90741
ENSG0000	ATG2B	14:962792	2.34172	2.67548	0.901615	1.38277	1.47538	4.24047	2.39339	2.7507	0.411023	4.52182	0.48675	0.531168
ENSG0000	GALK2	15:491060	17.9267	14.8595	21.2645	24.5671	17.3322	15.2836	17.0056	17.3929	19.6678	18.9325	0.71905	-0.1841
ENSG0000	TMEM208	16:672271	67.1092	47.6486	88.9406	110.591	69.3026	80.0273	84.8796	109.295	139.16	33.3399	0.5171	0.244137
ENSG0000	C17orf67	17:567919	1.48282	1.8166	2.46674	1.64288	2.57825	2.20218	2.09303	1.93764	0.995069	1.71619	0.8679	-0.24986
ENSG0000	SPPL2B	19:225225	11.6664	27.6794	6.22013	7.69257	12.3675	8.84059	18.2803	8.76268	11.4747	18.3446	0.99895	-0.00098
ENSG0000	SSU72	1:1541672	185.968	184.953	209.108	194.886	256.081	197.936	224.606	183.657	102.627	130.953	0.29045	-0.36649
ENSG0000	TMEM170	6:1153827	0.291423	1.43311	0.209971	0.080063	0.122679	2.10042	0.169044	0.336869	0	0.085597	0.6877	0.30898
ENSG0000	RUFY1	5:1795505	37.5329	46.2245	18.4321	16.0724	21.5218	37.1066	42.9454	12.7534	18.6291	25.4994	0.95255	-0.02172
ENSG0000	PRPSAP2	17:188357	18.8146	27.9845	16.5819	22.1005	13.6471	19.3514	8.4502	15.9444	19.5932	9.39075	0.25095	-0.4547
ENSG0000	ASB6	9:1296088	22.1102	26.9826	14.779	17.063	15.5121	8.48915	18.0251	16.9414	12.9206	23.6011	0.4627	-0.39434
ENSG0000	TSPYL5	8:9727347	0.231942	0	0.17994	0.323475	0.05442	1.50744	0.841358	0.759533	0	0.386926	0.1972	2.07257
ENSG0000	ST6GALN/	9:1278664	105.742	55.229	52.9115	66.1247	42.5545	143.063	76.1906	51.0079	85.4872	92.9085	0.23555	0.424472
ENSG0000	IGHV1-46	14:106482	0	26.587	0	0.705618	0.231151	0	0	0.239295	3723.44	0.27068	0.19295	7.50672
ENSG0000	EPHB6	7:1428550	7.78497	26.5502	3.94342	5.40296	6.75438	3.18663	3.73164	1.0443	7.28912	5.77638	0.0622	-1.04344
ENSG0000	MAD2L2	1:1167447	52.1546	36.5449	62.9005	67.2001	94.9283	101.194	125.798	99.021	43.1308	46.4358	0.458	0.253467
ENSG0000	RP5-855D	8:213185-	0.298516	0	0	0	0.261694	0	0.888771	1.3733	0	0.666197	0.2341	2.56969
ENSG0000	WDR81	17:171149	12.198	10.8532	2.31604	1.8583	5.38913	5.50162	4.79476	3.74517	4.26858	8.46781	0.9449	-0.04361
ENSG0000	INCA1	17:498770	0.161105	1.20257	0.686286	1.21515	0.545874	0.600937	0.96058	0.195418	1.20939	0.643837	0.998	0.002798
ENSG0000	RP11-180	7:1309417	0.831985	1.46442	0.151527	0	0.077096	0.448868	0.124288	0.213811	0	0.536562	0.87015	0.333187
ENSG0000	CLDN15	7:1012320	4.01434	5.6578	3.52812	1.16528	3.91957	6.45055	6.04582	2.62035	0.202675	10.0778	0.60625	0.429929
ENSG0000	AC062029	2:8853871	2.76315	2.33387	2.18048	3.24253	1.39688	1.26127	1.00387	2.58692	0	1.74764	0.6155	-0.92525
ENSG0000	WDR73	15:845978	20.1288	20.4344	18.1937	20.7027	9.64985	15.7191	18.7186	18.7698	19.0814	29.0161	0.67895	0.190253
ENSG0000	AC009245	7:1373810	0.816195	0.838126	0.945347	1.07967	0.201608	0.590592	0.979626	0.775741	0.421152	0.081706	0.7677	-0.45907
ENSG0000	FUK	16:704544	6.71838	6.22243	1.01965	2.1542	2.5357	6.34298	3.6899	3.3802	3.47097	7.10677	0.4771	0.376115
ENSG0000	CHMP5	9:3326487	76.5213	46.505	61.9379	54.5195	36.5882	115.946	127	74.6029	48.4999	23.6231	0.1141	0.545914
ENSG0000	GIGYF2	2:2326972	18.717	17.389	14.2117	12.2474	19.0823	13.8535	21.6713	13.9508	15.1872	32.622	0.93875	0.028878
ENSG0000	TRBV19	7:1426188	16.3539	50.2781	45.1801	24.241	19.6694	49.9424	43.5806	44.444	107.84	17.5452	0.07515	0.813065
ENSG0000	EMC10	19:504667	5.0862	2.95615	4.00598	2.78819	5.37708	3.05585	3.52128	5.08591	2.79327	4.50165	0.5537	-0.40718
ENSG0000	CTA-223H	22:414309	0.26932	1.49333	0	0	0.10062	1.36548	0.891661	0	0	0.716951	0.55775	0.889865
ENSG0000	RPL13AP2	13:544407	5.09424	3.30543	2.14088	2.06021	0.90417	3.37758	4.44936	0.363759	2.21075	0.210968	0.80315	-0.45072
ENSG0000	HLA-DQB	6:3265946	18.5468	10.0278	26.6377	36.0951	2.74398	96.1769	9.96356	54.8531	56.8484	1.70351	0.0072	1.14341
ENSG0000	RP11-264	16:289739	1.64221	3.37982	1.46332	1.15048	2.26144	9.93038	5.83393	0.89189	1.05516	9.64802	0.53415	0.782045
ENSG0000	DAB2IP	9:1215670	0.507341	0.032505	0.106445	0.310358	1.2823	2.26268	0.076519	0.194164	0.042361	2.29585	0.48025	0.962067
ENSG0000	GLRX2	1:1930964	17.6275	9.98132	16.3452	11.2576	18.6194	37.4557	23.037	16.8987	10.8163	5.48023	0.666	0.255912
ENSG0000	CCDC106	19:556350	1.28841	2.28991	1.10484	2.15772	1.28547	2.05627	1.08283	0.340031	0	1.0429	0.29325	-0.94919
ENSG0000	RP4-706A	1:7708898	9.50372	4.73757	9.97418	10.6983	1.8331	8.59153	8.22102	4.73463	6.14276	0.557469	0.9371	0.108983
ENSG0000	MISP3	19:140725	3.25033	2.94012	2.85328	1.07824	2.77805	5.65731	1.45678	2.53527	0	3.72518	0.96145	0.053139
ENSG0000	TBC1D8	2:1008201	0.326801	2.162	0.062226	0.092899	0.288772	0.691615	5.37181	0.386551	0	0.543201	0.4765	1.25124
ENSG0000	ZNF823	19:117212	2.70753	4.71127	1.55701	2.43379	0.871717	1.68639	2.35266	0.20864	0	1.63278	0.306	-1.1984
ENSG0000	LCLAT1	2:3044722	6.9388	13.687	3.83017	5.51466	7.54282	4.98657	6.70118	2.62311	3.78434	15.0725	0.41785	-0.41806
ENSG0000	DDX24	14:940263	150.223	164.881	113.307	85.7532	142.458	137.002	174.732	127.263	151.263	157.127	0.80005	0.123531
ENSG0000	ALDH1A1	9:7290066	0.317687	3.05665	0	0	0.033342	6.78377	0.016229	0	0	0.019506	0.50815	1.11801
ENSG0000	SLC35B4	7:1342893	3.0419	2.74078	6.46294	2.8181	5.44092	3.63971	6.84987	5.42283	7.79325	15.2508	0.4216	0.508417
ENSG0000	ARHGAP4	19:106592	353.93	553.886	126.618	152.059	143.434	171.42	195.601	129.762	111.751	201.501	0.32015	-0.44335
ENSG0000	FKBP15	9:1131509	9.90349	15.028	5.60514	4.75439	7.97247	15.0571	10.8098	5.21006	5.69702	18.5145	0.5541	0.279742
ENSG0000	CTD-2562	11:752601	0.9064	1.88551	2.3305	0.479547	3.81953	0	0	0.299332	0	2.06701	0.2258	-2.16525
ENSG0000	P4HTM	3:4891778	45.6369	44.6835	50.1077	45.8454	38.2861	50.8821	60.8698	32.5453	31.1571	49.8308	0.97285	-0.03272
ENSG0000	RP11-446	8:5605003	2.11672	1.15102	1.43651	1.19134	0.962162	1.45885	1.56519	1.1602	0.928341	0.49988	0.6574	-0.2193
ENSG0000	COMMD9	11:362692	27.4351	33.7008	40.4665	28.8249	43.3992	37.4881	23.91	30.3785	26.2832	17.0281	0.2526	-0.47605
ENSG0000	ARID3A	19:925780	0.555527	1.33793	0.636544	0.753946	1.44931	1.05244	0.582331	1.06255	1.75472	3.70037	0.231	0.700234
ENSG0000	C11orf1	11:111782	4.5648	3.31031	10.8987	7.47334	7.338	6.05062	6.67665	7.82384	8.8554	1.71184	0.8037	-0.20222
ENSG0000	RP5-827C	1:2343721	3.34949	2.4794	2.80415	3.2306	3.02862	1.17162	2.48929	1.95396	1.523	1.17406	0.49375	-0.85061
ENSG0000	KIAA1143	3:4473766	12.825	5.65073	4.83428	6.87641	4.94981	24.3288	14.4554	5.06112	3.35893	5.34498	0.9159	0.086488
ENSG0000	RP11-347	8:9481331	0.373485	2.00534	0.112052	0	0	0	0.401922	1.41524	0	0.124939	0.93645	0.165614
ENSG0000	LRRIQ3	1:7402601	0.830136	0.411174	0.167661	0.233914	0.201925	0	0	0.184082	2.01131	0.392821	0.27785	1.12205

ENSG0000C	LATS2	13:209730	0.821621	0.41892	1.86478	0.816392	2.76082	0.658538	0.652317	1.27541	4.02398	2.29981	0.6695	0.240823
ENSG0000C	SAR1B	5:1346011	27.2073	18.8064	22.5067	22.426	13.261	50.1015	32.04	28.3898	26.2575	13.3558	0.60165	0.353257
ENSG0000C	AC010547	16:714475	0.194926	1.31554	0.385959	0.52172	0.301916	0.10715	0.397239	0.158141	0.508214	0.622826	0.65955	-0.79153
ENSG0000C	RP3-475N	6:4294036	0.692846	0.193523	0.450828	0.903061	0.061073	0.765002	0	0.147168	0	0.6797	0.72375	-0.55364
ENSG0000C	RXRB	6:3319358	6.73647	9.87177	5.17132	4.32682	8.66691	7.83261	10.6044	7.43837	7.07995	12.9402	0.5834	0.363198
ENSG0000C	CTD-2192	19:126432	11.5281	12.1376	0.149862	3.30922	0.650612	5.17796	1.02632	0	0	0	0.753	0.391795
ENSG0000C	OSBPL10	3:3165788	0.06436	2.3221	0.440657	0.547981	0.23002	0	0	0	0.149849	0.17453	0.0776	-4.12781
ENSG0000C	ANK3	10:600262	4.79786	2.66299	1.53531	1.64128	1.90801	0.066698	0.259352	0.661186	0.344824	4.50267	0.1265	-0.95756
ENSG0000C	FAM185BF	7:7695983	0.339016	0.857801	0.207402	0.917956	0	0.397968	1.06612	0.555851	0.205655	0.344291	0.7281	0.573742
ENSG0000C	RBM22	5:1506907	41.3014	35.505	22.1669	22.8167	38.143	23.5531	34.0645	31.5976	21.0859	45.3675	0.68875	-0.1255
ENSG0000C	RP11-119.	4:625583-	0.240849	1.53053	0	0.2843	0	0.954835	0	0	0	0.034072	0.7082	-0.62793
ENSG0000C	ASMT	X:1615000	0.339714	0	0	0	0.03447	2.26188	2.25874	0	0	0.167898	0.1692	3.56109
ENSG0000C	ZNF805	19:572406	1.50513	2.55888	1.12114	3.23987	1.21431	3.13569	1.67097	1.84501	0	1.05973	0.6403	-0.46054
ENSG0000C	RHOQ	2:4649074	3.65355	4.95503	1.06863	3.08908	2.00111	6.38301	5.26718	3.42595	2.93609	5.71963	0.45285	0.647807
ENSG0000C	XXbac-B4	22:203207	0.781387	0.586496	0.794029	0.702179	0.265066	0	0.509956	0.843314	0	0	0.49615	-1.18308
ENSG0000C	ZSCAN18	19:580592	11.3186	21.6774	1.94046	10.9232	7.12408	7.45957	6.27625	4.99308	13.2481	14.8289	0.9968	-0.00228
ENSG0000C	MAF1	8:1440986	137.141	125.539	161.306	137.354	163.479	89.6176	129.882	112.401	92.6899	102.672	0.18685	-0.51325
ENSG0000C	RANBP9	6:1361532	10.3078	7.24119	8.74172	12.4244	11.2093	12.6604	12.4078	20.0162	2.89918	22.3336	0.56815	0.446899
ENSG0000C	TMEM120	7:7589919	29.5937	39.1102	19.7914	30.1252	21.3184	30.0944	51.0121	43.4546	51.2477	26.4001	0.2576	0.545986
ENSG0000C	RP11-351	10:431368	1.8091	3.92933	2.60988	4.11633	1.41898	0	2.75067	1.75964	4.97756	0.498873	0.69745	-0.37217
ENSG0000C	DTD1	20:185673	29.3566	27.2113	67.1119	56.383	44.2639	44.4436	59.1779	49.6262	20.0038	17.3355	0.48475	-0.32933
ENSG0000C	RPA1	17:177948	43.0337	48.2011	41.2352	45.7245	38.7808	55.475	43.2075	40.7677	31.0137	43.984	0.77665	-0.10286
ENSG0000C	PCED1A	20:283531	16.9769	13.4621	13.8983	16.1866	11.9474	8.50669	13.23	7.85484	11.6498	13.9242	0.7795	-0.25587
ENSG0000C	RP11-479	7:6564700	0.506736	0.479356	0.795018	0.898218	0.04504	1.55068	0.442218	1.48652	1.93181	0.547646	0.47965	1.13708
ENSG0000C	LMAN1	18:593278	25.6124	21.8904	11.3964	12.7121	31.1504	19.4135	18.6815	13.654	11.3159	30.8626	0.71985	-0.18489
ENSG0000C	SLC29A1	6:4421950	5.04399	8.23318	2.55578	4.48219	4.80748	19.4567	7.32275	8.89981	4.09726	5.67536	0.13385	0.834725
ENSG0000C	ZNF500	16:469650	5.11181	11.7594	3.14888	1.4375	3.64541	9.00866	8.76945	4.65394	0.938932	4.87433	0.9351	-0.07787
ENSG0000C	POC1B	12:894197	39.5504	20.8889	16.998	21.4689	18.2846	15.4975	18.8544	18.0437	12.6787	21.0168	0.2335	-0.4649
ENSG0000C	SIGMAR1	9:3463472	43.9715	38.8558	53.7876	52.868	43.9164	40.4062	38.432	50.432	58.755	28.9365	0.74165	-0.1009
ENSG0000C	H3F3AP6	4:1396657	19.459	18.3851	5.29126	5.63289	3.00438	19.2455	24.5862	3.29475	3.07502	1.43488	0.9959	-0.0069
ENSG0000C	ZNF510	9:9675586	2.57655	1.96794	1.41441	0.526196	1.31736	4.25244	2.37455	0.6239	1.22109	1.58817	0.79135	0.197258
ENSG0000C	SYAP1	X:1671963	7.62614	7.64165	10.0301	14.0549	8.77839	11.7427	7.52282	9.90821	11.367	9.25626	0.7724	-0.10729
ENSG0000C	HSD17B8	6:3320464	21.047	15.3831	46.0112	42.5777	36.4642	20.0321	19.0134	27.9733	20.3541	12.2725	0.09085	-0.7642
ENSG0000C	AGRP	16:674813	0	0	0	0	0	3.34026	0.21841	0	0	0	0.13	inf
ENSG0000C	AGTRAP	1:1173608	69.57	46.993	74.7384	80.4755	98.8423	110.83	108.937	72.6278	53.0504	34.5384	0.7855	-0.09012
ENSG0000C	PDCD6IP	3:3379835	48.2899	38.744	29.4557	23.6181	18.1069	46.725	30.1411	40.0904	23.7749	31.1105	0.70245	-0.14833
ENSG0000C	VPS13C	15:618523	16.6785	16.649	3.84093	4.98857	7.99121	14.2325	10.3499	10.0443	4.52098	18.3282	0.3245	0.431651
ENSG0000C	RP11-810	17:414316	4.08739	6.74762	2.32808	2.41693	5.94988	3.87907	2.36443	4.61133	5.69577	7.86872	0.70395	0.580738
ENSG0000C	CCNJ	10:957117	2.05427	2.32647	0.495676	0.043576	0.63461	2.25535	1.979	1.71959	0.078621	1.46948	0.7287	0.442125
ENSG0000C	BCL7C	16:308336	82.6263	45.4926	68.9066	53.8425	77.1732	79.3929	71.4605	49.1373	42.4139	16.9383	0.3193	-0.37598
ENSG0000C	SEC24A	5:1346011	5.47867	6.78985	1.95604	2.02264	1.66564	1.51733	2.90098	3.38479	4.37795	3.49063	0.88285	-0.10805
ENSG0000C	ZZZ3	1:7756241	15.9099	20.3476	12.2919	9.70109	5.89944	17.5124	8.08036	15.5398	13.8707	10.522	0.9885	0.007538
ENSG0000C	LRRCC1	8:8510714	4.14859	5.73957	1.47377	0.427902	1.55873	14.7491	2.93082	1.64405	2.37087	2.67532	0.23605	0.874445
ENSG0000C	VPS52	6:3324953	40.5719	47.4682	19.9029	23.6335	19.3547	37.0032	36.0666	31.498	22.3004	35.7049	0.8846	0.150208
ENSG0000C	NUP133	1:2294402	6.76592	10.2533	4.36899	4.53834	5.45782	4.42694	6.86455	7.06294	13.354	5.90435	0.8107	0.124315
ENSG0000C	UBA6	4:6761265	7.20941	5.30103	4.40333	4.3218	3.3283	6.43007	8.9489	5.64023	12.7019	10.9973	0.19315	0.721885
ENSG0000C	AL022341.	16:636816	1.01663	0	0	0.539548	0.593169	0	0	0.837151	0	0	0.54895	-1.29675
ENSG0000C	ZNF426	19:952322	5.85743	4.27377	6.32286	4.82044	5.33455	1.23703	4.64624	6.00916	4.15953	11.8634	0.5127	-0.38188
ENSG0000C	FAM69A	1:9283202	10.392	23.8918	9.53813	9.88423	5.30769	3.55702	14.9952	9.38808	4.16808	9.92582	0.6938	-0.46569
ENSG0000C	IGHV1-2	14:105986	0	4.58904	0	0	0.092497	0	0	0	0.116139	0.117518	0.1763	-3.54136
ENSG0000C	RP11-649.	17:751099	0.935043	0.949517	0.780422	1.86766	0.701636	0.749803	1.90132	1.61971	1.20092	2.64111	0.58335	0.693151
ENSG0000C	RP11-114	12:693483	31.2573	26.2526	30.6895	13.4037	16.8945	230.98	3.30044	15.921	28.5198	10.2864	0.37615	1.14796
ENSG0000C	AC009120	16:742967	0.234172	0.585868	0.236515	0	0.091048	1.08783	0	0	2.76619	0	0.402	1.86981
ENSG0000C	WDFY2	13:515844	4.99256	7.96858	4.53539	4.3338	2.29216	4.99686	2.62657	4.28163	9.06938	4.67832	0.86935	0.100991
ENSG0000C	RALGDS	9:1330977	71.6972	63.2134	46.698	26.3544	36.3965	69.7221	94.3638	47.6376	36.7925	73.514	0.3034	0.353344
ENSG0000C	PLCG1-AS	20:410288	1.19425	0.551916	1.11678	0.281451	0.648888	0	0.99626	0.058738	0.152447	1.34725	0.7191	-0.57648
ENSG0000C	AIG1	6:1430604	5.8788	6.25204	5.2162	7.95158	4.10652	5.01614	0	3.83264	4.86275	3.7229	0.3524	-0.82348
ENSG0000C	SNORD3C	17:191896	23.0605	0.901853	0.828036	2.4256	0.229784	0.116521	0	0.72368	4.2641	0.268469	0.7607	0.411512
ENSG0000C	STX8	17:925047	149.89	76.8309	121.113	121.936	130.73	133.583	127.593	104.762	65.4402	24.4488	0.2315	-0.45742
ENSG0000C	THYN1	11:134248	97.5184	99.7255	131.583	123.84	107.072	139.084	105.671	119.462	66.6471	40.8607	0.4946	-0.26503
ENSG0000C	HYOU1	11:119044	11.2256	3.75137	4.67825	3.31242	9.01647	4.13064	6.83244	10.9536	2.98499	14.663	0.3479	0.439507
ENSG0000C	SNAP29	22:207076	13.0255	11.0918	12.3349	9.93952	11.0946	7.62643	17.3995	10.6079	3.65816	10.1262	0.52185	-0.39333
ENSG0000C	COQ3	6:9936939	5.23334	5.81506	8.19941	10.2719	6.46076	2.18171	7.16079	3.25029	3.62773	3.56907	0.3036	-0.73525
ENSG0000C	RFNG	17:820479	10.9433	8.9109	2.57273	1.597	6.27865	7.57292	13.3336	8.52801	7.0048	8.72095	0.59965	0.536158
ENSG0000C	IFT140	16:149334	4.40972	6.13342	1.90394	2.21564	2.54346	3.47466	0.827041	1.4604	3.08763	2.74491	0.5956	-0.49527
ENSG0000C	AC005253	19:185577	2.00227	2.46588	1.46567	2.29195	1.60257	0.709121	0.344664	3.68491	0.774336	3.30117	0.91685	-0.16958
ENSG0000C	C18orf25	18:461735	8.24008	11.0222	5.57718	6.37364	4.21817	6.42035	7.64662	9.7774	8.14026	8.91634	0.73665	0.1443
ENSG0000C	NCKAP5L	12:497911	1.16951	1.59009	0.370293	0.163867	1.15633	0	0.700664	0.066783	0.73949	1.99578	0.8602	-0.26421
ENSG0000C	RNF41	12:562021	11.5379	9.69701	18.3278	15.1021	18.3323	11.574	24.0896	14.2438	16.8286	23.3257	0.9202	-0.04315
ENSG0000C	HSPA1B	6:3182773	13.0043	22.1481	10.5372	4.53566	20.6323	72.4408	58.5766	76.2504	99.6647	131.067	0.00005	3.43421
ENSG0000C	ZNF846	19:975199	4.94316	2.52824	6.88174	4.07787	4.22323	5.72053	3.25133	4.268	5.44261	3.15972	0.9627	0.033326
ENSG0000C	AGAP9	10:474962	0.460429	2.40681	0.316936	2.6798	0.773526	0.612506	4.03774	0.552262	2.37842	2.30331	0.6085	0.55991
ENSG0000C	DMXL1	5:1190063	7.76108	7.92107	4.57163	4.74316	3.06206	8.51054	4.92047	3.73011	1.50149	2.71173	0.4962	-0.38591
ENSG0000C	DNMBP	10:998755	1.64147	1.14155	0.470188	0.092427	0.807671	0.912949	0.106622	0.769966	0.868312	0.809429	0.90995	-0.08543
ENSG0000C	MAPK11	22:502637	0.168331	0	0.235469	0.019252	0.140541	0.136996	1.32565	0.516198	0.485201	1.04538	0.1243	2.61058
ENSG0000C	GLUD1	10:870504	65.9057	45.0437	35.636	31.5558	32.6521	53.5506	60.6974	41.3111	23.2393	48.9489	0.79255	0.091528
ENSG0000C	IVD	15:404057	29.6259	28.0298	39.5301	27.297	55.959	23.1244	30.8863	34.075	16.6833	57.8527	0.4892	-0.25485
ENSG0000C	RGS5	1:1631111	1.4983	0.835379	1.23456	3.08141	1.0194	3.74696	1.08579	0.085299	0	0.020941	0.5773	-0.53958
ENSG0000C	HOXB2	17:485408	21.9891	44.622	18.8373	27.5534	33.7295	19.5404	100.254	22.4598	49.4038	32.2383	0.314	0.509724
ENSG0000C	RPL4P6	22:403464	0.341243	0.312415	0.822526	0	0.78469	1.25766	0.372917	0.248622	0	0.241987	0.87445	-0.24551
ENSG0000C	SRRM2	16:273707	194.797	268.148	181.645	67.3754	392.699	226.795	214.186	151.924	130.551	549.271	0.8351	0.092465
ENSG0000C	RECK	9:3603643	6.61002	5.66399	4.06941	8.25735	4.0892	2.4181	1.68113	3.90384	0	5.59105	0.15125	-1.23356
ENSG0000C	FZD6	8:1031210	2.16667	2.95584	0.151467	0	0.033279	4.35767	0.629309	0.334306	0	0.295174	0.9882	0.020416
ENSG0000C	MTCP1	X:1550616	5.26602	3.62499	4.77871	1.83797	1.43639	6.17923	11.8546	12.0282	2.83923	2.42709	0.14395	1.77865
ENSG0000C	HIST1H3C	6:2604541	1.88091	0	0.722314	1.08351	0	2.07222	1.28789	5.50072	0	0	0.4064	1.30468
ENSG0000C	SLAMF1	1:1606080	48.7995	20.6143	53.7613	45.1345	30.5929	81.6089	49.4837	46.6224	40.3662	46.0529	0.59745	0.249469
ENSG0000C	ZKSCAN7	3:4455519	3.63112	1.51436	2.78606	1.73262	1.42458	0.035418	0.771981	1.89208	4.78798	1.68971	0.8505	-0.17606
ENSG0000C	SAMSN1	21:144852	31.8389	44.1218	20.5941	34.1177	15.7576	218.411	219.125	80.1949	110.703	34.8948	0.00005	2.21563
ENSG0000C	C1QA	1:2263650	0.30539	0.640817	0.478669	5.6098	2.79236	256.78	19.4252	11.5909	31.8758	7.37794	0.029	4.89903
ENSG0000C	TECPR1	7:9821462	17.1229	10.9205	7.3024	7.47451	9.11331	10.5278	4.98145	9.37625	4.29148	13.9116	0.39875	-0.35606
ENSG0000C	RP11-96L	1:2616951	0.180518	0	0.166578	0.481651	0.328914	0.107127	0	1.27931	0.309786	0.869957	0.24915	1.43493

ENSG0000C	RPS14	5:1504431	901.842	1044.27	2961.47	1889.78	641.418	726.77	921.589	1535.47	1282.74	99.5493	0.23975	-0.70037
ENSG0000C	RP11-119	4:625583-	0.51547	1.35482	0.996756	1.73033	0.67477	4.06552	1.37889	0.441486	1.18981	1.15644	0.7227	0.546908
ENSG0000C	HOMEZ	14:231853	3.43173	2.44017	2.00123	1.43042	1.77894	1.91788	1.62597	1.82743	0	3.11565	0.4807	-0.73355
ENSG0000C	MRPS22	3:1390054	36.8651	22.755	38.9167	30.2825	19.6437	61.8669	47.8501	38.5406	16.5	12.9179	0.74815	0.235985
ENSG0000C	CTD-2006	8:9321330	0.470547	1.14505	0.749301	0	0.342321	1.11145	2.1038	0.832495	0.057543	2.12081	0.4708	1.23236
ENSG0000C	TPRG1	3:1889417	2.27393	2.55549	0.476918	3.85828	2.21416	3.8348	7.72772	4.51221	2.55706	2.88321	0.2184	0.860152
ENSG0000C	PTK2	8:1406578	0.309151	5.02183	0.423124	0.089779	0.429492	0.104919	0	0	0.395951	0.115639	0.01525	-2.6583
ENSG0000C	TRAF7	16:215569	3.33474	2.15394	2.91035	1.7209	4.3854	4.05365	3.32134	4.90361	1.10701	5.8975	0.75555	0.17017
ENSG0000C	LNPK	2:1759238	6.65647	4.91564	3.1959	2.85522	2.59364	5.73834	3.70955	3.96262	4.70103	5.3644	0.879	0.10034
ENSG0000C	PCNX1	14:709074	6.66173	6.42685	1.48999	1.67697	2.35725	3.90083	7.92733	5.25412	2.14894	8.8733	0.3108	0.526728
ENSG0000C	TNIP3	4:1211314	4.91887	3.20978	2.84074	0.789547	3.52104	40.7035	43.7719	39.5246	13.6211	29.8015	0.00055	3.45102
ENSG0000C	MRPL37	1:5413268	39.782	37.7214	69.7504	74.0922	74.9783	31.4083	38.0491	56.0296	48.8698	49.4376	0.2491	-0.44713
ENSG0000C	RAC1	7:6374522	120.14	168.233	137.17	97.9388	206.734	228.267	191.098	162.241	92.3104	143.252	0.8677	0.072448
ENSG0000C	ADM	11:103046	1.65088	0.585014	0	1.92169	0.997017	36.7008	0.027181	0	5.60373	2.63144	0.0086	3.13638
ENSG0000C	SASS6	1:1000380	3.11296	4.10496	1.25458	0.902901	2.55084	4.66123	1.57387	0.8483	0.640839	3.10437	0.9259	-0.14149
ENSG0000C	JUN	1:5878078	409.77	68.8168	90.6868	165.363	38.4311	31.5754	28.651	65.9765	259.924	172.51	0.55645	-0.30311
ENSG0000C	RP11-74J1	13:411329	0.220963	1.09986	0.284396	0	0.161289	0.861821	1.95907	0	0.111884	0.770514	0.47765	1.19565
ENSG0000C	ALAS2	X:5500035	0	0	0	7.85929	0	0	0	0.019946	0	0	0.00275	-8.72282
ENSG0000C	C16orf86	16:676629	6.33234	4.19175	4.74539	9.97772	4.39599	9.83273	3.48783	1.60737	17.7574	2.85281	0.7454	0.304498
ENSG0000C	PPIL2	22:216522	23.8216	36.0774	9.81419	15.5538	13.7393	23.5975	28.8034	17.8891	35.6297	18.2306	0.5394	0.347314
ENSG0000C	ZNF486	19:200779	1.18223	0.607476	1.33468	0.800657	1.06845	0.018103	1.25248	1.21406	0	1.07389	0.6074	-0.66205
ENSG0000C	MTND1P2	1:585988-	1.70873	1.36827	3.15334	3.21136	4.06594	4.3719	2.36898	3.78495	6.52281	1.90876	0.8723	0.215068
ENSG0000C	TUT1	11:625596	19.2101	25.2052	15.346	16.2474	10.0623	7.39821	7.6313	10.7797	14.06	14.612	0.3918	-0.81422
ENSG0000C	TMX2	11:577125	47.4472	56.6559	78.6409	81.7464	54.6147	43.9373	61.8171	82.6063	51.2402	53.1652	0.75125	-0.15338
ENSG0000C	STIP1	11:641852	65.6291	69.6703	53.0159	67.975	81.9633	81.1771	108.904	84.9888	72.9163	82.2095	0.31975	0.303439
ENSG0000C	NSF	17:465906	19.2349	22.9494	10.4143	10.3011	5.78521	11.8925	18.5504	10.6167	8.58808	9.73431	0.92715	-0.03663
ENSG0000C	TIMD4	5:1569192	1.54204	1.62681	0.655323	0.695231	0.042396	2.29858	4.49548	2.64329	6.37859	0.850662	0.1618	2.65739
ENSG0000C	NCAPH2	22:505082	20.9956	32.9525	31.7135	32.7645	24.7678	27.0825	43.4723	45.676	28.1376	26.3873	0.5025	0.233356
ENSG0000C	AKAP8	19:153533	16.4039	20.6666	11.1499	10.7978	15.2144	10.5408	15.3253	10.2111	9.08397	17.8746	0.7538	-0.1924
ENSG0000C	MKLN1	7:1309417	20.6721	31.7797	7.91746	14.8811	4.19251	23.364	7.16892	5.31021	18.4034	7.54412	0.68485	-0.36928
ENSG0000C	POLQ	3:1214314	0.350412	1.94581	0.020285	0.012868	0.036604	1.24095	0.55164	0.050829	0	0.647705	0.94005	0.114884
ENSG0000C	TRBV6-6	7:1424695	9.27276	24.8107	7.22785	14.8047	4.81743	34.3131	15.6388	7.14967	13.2679	2.2637	0.7567	0.279787
ENSG0000C	SDPR	2:1918343	0	0	1.26735	7.01077	0.342822	0	0	0	0	0	0.08175	#NAME?
ENSG0000C	NDUFB11	X:4714221	124.02	86.2407	196.62	161.093	86.5769	170.03	165.444	189.929	136.67	15.1774	0.99305	0.004103
ENSG0000C	LINC0125	9:3373297	0	0	1.06902	1.43337	0.350368	0	1.00442	0	0	0.120945	0.4363	-1.69618
ENSG0000C	RNASEH2(	11:657119	44.6852	44.2661	62.4182	59.6395	49.7731	38.0899	51.4602	58.9114	37.1031	26.8248	0.51045	-0.38672
ENSG0000C	GDI2	10:576522	126.846	114.066	92.4989	83.7253	89.8343	128.1	114.986	113.228	85.4008	81.383	0.943	0.021578
ENSG0000C	TMEM241	18:231345	1.77356	1.4938	1.08767	2.75739	1.05571	0	3.96265	1.22392	1.74104	0.436297	0.78455	0.178459
ENSG0000C	HSPA6	1:1615054	1.84991	3.51014	1.47419	0.759014	1.46909	22.484	8.13899	6.7773	163.885	67.7408	0.00625	5.12892
ENSG0000C	RP11-284	4:1429331	0.077244	0.296114	0.183856	0.140618	0	0	0.78859	0	1.65966	0.519677	0.2103	2.17699
ENSG0000C	NARF	17:824542	64.4387	62.5476	67.1545	87.2517	57.2774	116.152	105.179	75.3691	136.763	67.2588	0.0522	0.644425
ENSG0000C	SCAF4	21:316710	24.4619	26.9033	11.6258	7.20607	10.1569	13.0698	15.3408	13.4745	9.51836	20.5949	0.62475	-0.23585
ENSG0000C	MSRB1	16:193820	16.8492	14.0832	23.5919	25.8455	21.9756	26.676	16.3049	21.616	6.93751	10.0557	0.40775	-0.37817
ENSG0000C	THEM6	8:1427272	22.8132	21.4736	24.4432	30.7598	30.8611	12.0309	22.3032	15.4248	29.8249	31.8612	0.55095	-0.20989
ENSG0000C	RP11-646	4:5638762	0.518812	0	1.32997	1.70739	0	0.117642	0	1.45492	0	0.576874	0.72665	-0.50684
ENSG0000C	TFAM	10:583850	7.95682	11.592	10.9969	14.0727	25.1795	13.0794	19.4976	14.059	16.1612	16.0258	0.7987	-0.15715
ENSG0000C	RBKS	2:2777171	3.41094	2.64861	4.84731	9.09055	2.19417	6.38353	5.42385	6.20203	4.9172	0.80092	0.91165	-0.13397
ENSG0000C	CSRP1	1:2014835	40.5267	48.2566	33.6132	49.7416	27.8496	45.8332	55.0961	46.4964	43.3193	33.9112	0.74245	0.113981
ENSG0000C	MGST1	12:163471	2.58922	1.50926	1.1404	0.248114	0	10.0404	0.411111	0.148926	0	0.048065	0.38485	0.979218
ENSG0000C	MLH3	14:750137	3.53388	7.2804	4.61888	10.7604	5.58569	4.27649	3.94016	6.79239	3.37488	8.60764	0.51205	-0.36531
ENSG0000C	ZDHHC18	1:2682670	9.4132	6.05029	2.72439	1.29852	4.23892	8.98603	11.6101	4.93009	3.41499	9.15198	0.2515	0.548653
ENSG0000C	CCL1	17:343603	0	0	0	0	0	0	13.3695	0	0	0	0.0067	inf
ENSG0000C	SLC38A5	X:4845853	15.5515	6.58776	8.9382	12.4597	8.82038	3.86336	18.6492	18.0628	7.52514	9.70997	0.7372	0.144177
ENSG0000C	BBOF1	14:738518	1.35508	1.83682	2.36319	1.37138	0.829794	2.33537	0	0.404928	5.86407	0.937458	0.5459	0.585624
ENSG0000C	RPL11P3	10:878591	0.968567	3.9189	0.079593	0.528029	0.348847	1.901	3.96724	2.11394	1.47896	1.00825	0.50205	1.00755
ENSG0000C	RP11-214	12:123762	0.678632	0.941679	0.119748	0.167212	0.504604	0.561295	0.125405	1.38144	3.24085	1.14767	0.2809	1.71666
ENSG0000C	RPL23AP5	8:213185-	2.13332	0.658704	2.35865	0.590853	1.77303	0	0.572595	3.26652	1.9834	0.446611	0.8704	-0.20485
ENSG0000C	CDYL	6:4706158	7.46076	8.18839	6.52955	8.32834	8.59058	3.70734	3.66474	5.83972	1.03436	12.7728	0.21475	-0.71392
ENSG0000C	STK3	8:9840140	2.91665	4.71453	0.838054	0.72729	1.49662	2.80874	6.26449	2.46782	5.38766	3.59397	0.0605	0.995938
ENSG0000C	LINC0093	12:895611	20.3221	16.3876	10.3688	8.85386	5.99744	8.1884	4.23173	4.5972	3.76376	3.62719	0.2854	-1.27818
ENSG0000C	PCGF6	10:103302	11.3806	8.38424	8.19869	6.45063	5.41686	6.21064	6.94831	7.79576	18.6224	5.1892	0.47775	0.347002
ENSG0000C	GCHFR	15:407640	85.0482	23.6901	155.58	154.453	92.018	115.684	90.0754	165.057	170.139	28.4233	0.91435	0.059798
ENSG0000C	PRKCDBP	11:631894	0.358568	0.734524	0	0	0	0	7.49149	4.30369	0	0.737852	0.1779	3.36349
ENSG0000C	ILK	11:660364	77.5455	88.9348	83.0143	83.5325	71.026	79.121	94.1719	90.4574	69.8732	67.2586	0.88415	-0.05682
ENSG0000C	DNAJC25-	9:1116313	13.8586	7.49599	11.0711	13.4351	8.46531	22.7271	9.12614	7.76625	14.1382	2.55643	0.99605	0.004481
ENSG0000C	RBM39	20:357036	395.312	358.444	218.793	271.072	207.143	314.427	255.876	263.956	306.34	275.266	0.65955	-0.16009
ENSG0000C	RP11-884	3:5384657	0.445579	0.246842	0.345144	0	0.722267	0	0.024105	1.16615	0	1.43615	0.8215	0.380739
ENSG0000C	SIRT1	10:678846	11.8525	15.8053	3.39823	2.2357	3.51377	4.87692	6.09925	3.54351	6.32175	7.18702	0.4871	-0.29856
ENSG0000C	MED11	17:473142	37.1847	22.0033	64.137	56.9655	53.8103	46.2257	24.4956	53.1735	24.725	23.1897	0.40095	-0.46979
ENSG0000C	PARP9	3:1225279	30.8338	33.6569	13.2864	22.2987	18.8388	38.6387	43.1129	29.1809	16.3725	44.7172	0.1495	0.647736
ENSG0000C	MAP4	3:4785068	15.8528	12.9608	7.3571	2.33846	11.702	16.501	27.9807	15.5127	5.22414	31.0166	0.01515	0.908247
ENSG0000C	AC104653	2:1138293	0.605048	2.40912	1.4531	2.48996	0	2.39025	0	1.58464	0	0	0.64625	-0.78339
ENSG0000C	RP11-111	11:764413	5.32952	6.29038	13.1453	18.5281	2.7027	13.3725	6.78931	10.8412	21.5893	3.12131	0.7391	0.287325
ENSG0000C	KIR2DS4	19:548164	0.150129	6.07933	3.78198	7.12721	0	1.97963	14.7305	0.2322	0	0	0.9018	-0.2471
ENSG0000C	RP11-640	4:3594822	0.962132	0	0.061556	0.302929	0.063306	0	3.9145	0	0	0.525993	0.5026	1.63086
ENSG0000C	EMBP1	1:1215191	10.1045	2.42019	5.1151	2.87372	4.81766	1.00778	2.009	4.23002	4.48768	2.9124	0.40625	-0.84785
ENSG0000C	RALGAPB	20:384728	7.16246	9.35106	3.45054	2.57068	3.12942	1.7234	3.15035	5.38231	3.65768	8.97127	0.64675	-0.20109
ENSG0000C	PSME2P2	13:487711	0.309804	0.050033	0.732624	1.03955	0.245283	0.416335	0.287423	0.343558	0.336345	0	0.63755	-0.84394
ENSG0000C	DHX15	4:2451744	68.5421	73.4283	24.9079	31.1247	21.7328	50.604	38.8522	42.5457	32.7242	40.7581	0.75995	-0.09579
ENSG0000C	TRMT13	1:1001331	21.6341	28.6383	22.5345	20.981	14.8631	18.202	20.2551	21.6544	11.5442	33.409	0.76265	-0.12993
ENSG0000C	PKNOX1	21:429745	6.54011	6.84695	12.5335	9.87683	11.7232	13.0011	9.17417	14.8359	12.4428	12.4901	0.87565	0.080089
ENSG0000C	ADGRE2	19:146191	2.00702	5.09667	0.263104	2.46957	0.404311	1.53491	1.53814	1.06604	1.19322	0.404622	0.03585	-1.71845
ENSG0000C	RP13-104	17:647496	3.32664	8.24209	2.77628	0.623856	3.45047	5.08582	9.40445	2.8729	5.262	11.4627	0.5968	0.938293
ENSG0000C	NOA1	4:5696336	16.2372	15.0088	16.509	10.9006	15.6405	16.5978	11.4997	14.024	10.376	10.0393	0.7152	-0.25886
ENSG0000C	VANGL1	1:1156419	1.38148	3.75954	1.25254	2.49719	0.467385	5.49169	2.32034	0.911075	2.40747	1.98795	0.5397	0.478321
ENSG0000C	NFYB	12:104117	23.6129	16.8355	19.8597	18.6194	16.6115	28.1783	27.9507	26.6425	13.2039	16.1902	0.95065	0.025555
ENSG0000C	SRP14-AS	15:400393	3.67245	3.96022	4.76353	4.37276	3.16041	9.39772	1.85065	3.11251	0	1.08473	0.6462	-0.46845
ENSG0000C	LINC0095	7:4402695	1.30977	1.18337	0.778655	0.6951	1.41319	0.532081	2.46955	1.37637	0.674985	2.93616	0.7263	0.380234
ENSG0000C	RP11-573	3:1865384	1.28066	0.134165	0.732636	0.63893	1.41687	0	0.337849	0.485082	4.91808	0.372695	0.83275	0.169162
ENSG0000C	FAM217B	20:599337	10.7484	10.3725	11.1996	8.79988	9.31674	7.41008	5.45418	5.50141	3.89855	10.9115	0.1113	-0.7807
ENSG0000C	ADRM1	20:623020	137.433	130.413	154.656	116.924	199.102	188.999	248.052	138.695	90.3726	81.3135	0.76605	-0.10084

ENSG0000	TOMM40L	1:1612259	4.31709	3.41999	1.7312	1.46735	3.06861	3.27099	3.93314	2.90742	0.321816	4.68657	0.8965	0.082545
ENSG0000	RP1-20N2	6:1434508	1.17382	2.01501	1.27338	0.601508	0.54631	2.8245	0	0.862011	0	1.52702	0.9196	-0.18805
ENSG0000	ZNF484	9:9280938	3.52629	2.97897	0.776499	0.65779	1.56194	1.58449	3.67657	2.6738	6.11351	4.20739	0.32115	0.943226
ENSG0000	RP11-867	11:663479	0.66792	0.380208	1.23663	0.353885	1.40074	0.058448	0.825683	0	0	0	0.09545	-2.71416
ENSG0000	TTC17	11:433119	34.8679	39.9011	15.2021	15.2972	12.3996	14.9304	24.6626	21.426	27.6384	29.313	0.8497	0.083915
ENSG0000	STXBP3	1:1087466	42.4466	43.5035	23.7776	25.6054	15.7417	35.7847	34.3428	28.166	23.4591	24.4723	0.9087	0.037622
ENSG0000	PCED1B-A	12:470757	376.368	361.28	709.838	820.772	519.983	376.383	390.3	449.972	448.447	106.939	0.13285	-0.69845
ENSG0000	ZNF467	7:1497641	0.870178	4.37552	0.55079	2.05148	0.586616	0.327484	0.020825	0	0	0.038119	0.0747	-4.24919
ENSG0000	ATP5C1	10:778812	250.308	184.888	212.735	251.962	133.974	276.601	287.839	203.891	263.279	40.3412	0.91405	0.035705
ENSG0000	HIST1H2A	6:2780819	2.22751	1.44229	3.00574	0.502426	1.86595	9.14112	2.12916	0.537533	0.285708	0.569862	0.74445	0.574445
ENSG0000	UBE4A	11:118359	14.5327	18.5285	6.05925	8.89401	7.26535	4.75354	6.913	8.0763	4.68232	18.778	0.6054	-0.67832
ENSG0000	DEPDC7	11:330158	0.384436	0.841167	0	0	0	6.16068	0.310874	0	0	0.086817	0.282	2.52598
ENSG0000	TRBV9	7:1423918	22.3148	30.5595	9.98223	6.22147	2.97131	43.5145	95.3514	2.03137	149.528	117.411	0.0006	2.91008
ENSG0000	RRAS	19:496352	12.1533	21.9559	2.37749	0.662947	8.06067	27.2564	18.0211	3.92761	1.72525	4.88112	0.8038	0.21709
ENSG0000	NACC2	9:1360065	1.72749	0.598616	0.682315	0.80149	2.31667	2.65216	0.342521	1.24094	0.174845	1.85672	0.78965	-0.15271
ENSG0000	HYAL2	3:5031778	5.15295	2.6466	10.7688	13.0293	7.29737	4.90968	8.36347	10.6947	5.13969	3.84163	0.70055	-0.32612
ENSG0000	GSTO2	10:104268	0.498351	0.672878	0.541826	0.516312	0.093038	0	0.257497	0.803062	0	0.118157	0.2891	-1.21769
ENSG0000	LRRC32	11:766570	0.320719	0.935953	0	0	0	1.42483	3.48056	0.127751	0.061528	0.414541	0.06505	2.72076
ENSG0000	DNMT3A	2:2522785	16.6581	21.1569	12.3167	7.9482	14.4274	8.67789	11.0241	12.7832	3.82659	13.1364	0.20855	-0.66511
ENSG0000	COL6A2	21:460980	22.2942	7.67579	7.59429	6.84648	23.8807	0	0.183015	3.41373	2.70158	5.02879	0.0406	-2.48973
ENSG0000	RP11-326	11:318639	8.30097	15.7011	16.453	16.648	4.81356	2.86894	8.74745	4.33955	2.48086	5.3222	0.2685	-1.48861
ENSG0000	C11orf95	11:637598	0.405843	1.04391	0.087108	0.619942	0.320278	0.830657	1.65801	0.33896	0	0.674642	0.61415	0.463024
ENSG0000	TMEM107	17:817323	37.742	22.8131	46.1886	33.9454	31.6581	38.4344	23.4235	16.0529	8.27971	16.7983	0.0992	-0.87596
ENSG0000	RP1-315G	X:1239592	1.22112	0.126178	1.48287	1.3582	0.814745	0.514786	0	0	0	0.209497	0.22215	-2.58955
ENSG0000	RP11-589	8:4215177	2.12791	0.838546	4.01438	2.79392	1.15119	0	0	4.29708	0	1.40699	0.4345	-0.97086
ENSG0000	AAAS	12:533074	42.034	42.6785	36.0482	29.6974	34.8738	56.4837	44.7705	28.1417	21.0884	37.0831	0.95415	-0.01849
ENSG0000	KLC4	6:4303761	5.36368	6.01244	3.84697	4.65827	4.15252	5.44947	3.16233	3.53746	1.7757	7.76461	0.95455	0.058612
ENSG0000	FTH1P2	1:2286874	10.4286	10.2607	7.65813	12.9639	3.27746	41.9711	12.1644	6.5398	22.331	1.38198	0.2744	0.844228
ENSG0000	CNKSR2	X:2137441	0.475416	2.20919	0.166317	0.561007	0.171405	0	0.034497	0.564903	0.674006	0.113162	0.97675	0.034494
ENSG0000	CDC7	1:9150089	13.4164	13.5696	3.75829	4.33676	1.78572	10.3942	9.23358	4.04424	3.94168	3.91823	0.8587	-0.08745
ENSG0000	BFSP1	20:174939	0.57196	0	1.22818	0.741221	0.092127	0	1.53911	0.079922	0.026481	0.346257	0.67235	-0.59435
ENSG0000	JUNB	19:127630	1292.81	891.566	1083.11	2153.46	970.063	305.432	303.571	535.685	839.464	511.721	0.0237	-1.30974
ENSG0000	TAS2R18	12:108249	0.89561	1.73495	0	0	0	1.54264	1.32555	0	0	0.345612	0.8131	0.479987
ENSG0000	PTPRJ	11:479805	5.85007	5.6355	9.49116	6.03354	15.4212	18.5654	9.45628	13.2293	7.09072	27.8927	0.5244	0.28276
ENSG0000	RP1-286D	1:3778557	0.496177	2.58552	0.334106	0	0.082076	1.20744	0.151928	0.446562	0	0.320124	0.781	-0.56992
ENSG0000	ZFAND3	6:3781949	1.56777	2.22906	1.12194	0.277932	1.82976	2.14192	3.15369	2.89725	4.22799	2.00709	0.06545	1.43031
ENSG0000	ARHGAP1	11:466770	27.4867	48.4878	21.1964	13.9976	29.404	14.7184	21.2118	21.3167	9.78042	40.96	0.1894	-0.44889
ENSG0000	ZNF215	11:682194	0	4.10479	0	0	0.055226	0	0.968995	0	0	0	0.4431	-2.16028
ENSG0000	CTC-510F	19:112961	1.09712	1.60999	0.552268	1.67131	0.253733	1.35616	0	0.415776	0.027903	0.343327	0.72745	-0.4996
ENSG0000	RP11-414	Y:2934405	0.379322	2.47933	0.958761	1.82471	0.634679	0.766091	2.74635	0.277091	0	0.435493	0.74745	-0.53486
ENSG0000	SMAP1	6:7066777	17.3089	20.6187	17.0007	24.1954	24.6071	23.6503	32.1813	18.7283	18.8321	19.3182	0.99765	-0.00094
ENSG0000	KLRG1	12:895004	584.344	52.1166	862.263	717.896	752.034	82.6138	7.46736	179.668	160.301	206.561	0.00005	-2.28781
ENSG0000	RP11-872	14:965008	1.36022	0.387256	1.72705	2.17537	0.265893	1.63953	1.79898	1.02592	0.037375	0.788824	0.8395	-0.28985
ENSG0000	BTN3A2	6:2636515	86.0357	10.8965	147.916	114.309	177.339	85.0631	9.2207	85.8657	83.3625	278.716	0.6041	-0.19864
ENSG0000	LINC0084	5:1808299	17.5793	15.08	13.1971	13.2503	14.9781	14.6614	18.9367	15.178	5.4959	14.8254	0.74775	-0.18886
ENSG0000	LDHAP4	9:1492133	1.97812	2.30412	1.58877	0.051321	0.431132	2.19233	4.10503	1.24198	0.055145	0.224124	0.81995	0.491758
ENSG0000	UBE2N	12:933778	62.9707	58.5647	72.4487	58.1177	38.6547	79.2273	124.959	99.1984	76.1002	35.6567	0.675	0.214745
ENSG0000	AC011247	2:3856197	0.413708	0.532119	0.369655	0	0.347397	2.30055	2.62204	0	0.432004	0	0.50525	1.2645
ENSG0000	CTD-2619	19:584015	0.375199	0.193387	0.305289	0.333896	0.403489	0.188248	0.139722	0.367707	0.451649	0.944167	0.8711	0.167518
ENSG0000	IGHGP	14:105664	0	2.0956	0	7.06301	0.841357	0	0	0	0	0.552361	0.2133	-4.04569
ENSG0000	TXK	4:4806639	70.5712	69.0434	48.8335	82.9983	71.616	27.5215	5.98998	35.8658	30.6309	63.3778	0.03015	-1.05726
ENSG0000	AC009133	16:297907	0.591109	0.844073	0.867744	1.53637	1.23921	3.63901	2.70129	0.687889	2.62001	2.59808	0.31525	1.85946
ENSG0000	IGLV4-60	22:221621	0	0	0	6.22683	0	0	0	0	0	0.146634	0.25265	-4.5157
ENSG0000	TAGAP	6:1589728	64.0425	91.0764	67.1666	90.2015	45.6013	29.0301	52.3905	47.1354	30.7341	43.0797	0.00705	-0.97089
ENSG0000	CITED4	1:4086105	16.816	31.4151	22.0287	16.8653	12.4	15.0463	10.8606	10.937	7.18892	11.3449	0.1014	-0.82765
ENSG0000	ORAI2	7:1024268	23.4088	16.5446	12.8876	13.7693	14.2285	24.6898	23.7743	22.8902	7.28566	31.9165	0.79345	0.128277
ENSG0000	CENPI	X:1010982	0.022246	1.27405	0	0	0.099506	0.598826	1.14715	0.305856	0	0.749894	0.4347	1.12319
ENSG0000	DGUOK-A	2:7392682	0.445147	1.51943	0.555482	0.878287	0	2.95876	3.71806	2.39775	0.324987	0.531698	0.20555	1.61738
ENSG0000	PFN1P4	1:1481020	0.402108	0.613442	0.328637	0.629176	0	0	0.43362	0.703458	0.146131	0	0.87525	-0.29231
ENSG0000	MAST2	1:4578698	2.94319	4.59859	1.21327	0.576958	1.79374	4.27246	4.76231	2.42358	2.61104	3.24188	0.277	0.511473
ENSG0000	IPO5P1	19:232215	1.68728	1.06817	1.20565	0.104058	0.721449	0.665626	0.812974	3.65757	3.58936	0.742988	0.4569	1.23003
ENSG0000	ADSSL1	14:104724	1.03149	1.04776	0.781667	0	0.850894	3.54582	1.75954	2.69739	0	0.266136	0.2075	1.3073
ENSG0000	RP11-727	11:627711	1.30224	0.070089	0.362071	2.6828	0.260281	1.06888	0	0.771226	0	0.249268	0.51005	-1.15994
ENSG0000	ZNF37A	10:380943	3.86631	6.31205	1.95384	0.696636	2.07478	4.34396	3.99428	3.28963	0.900012	4.26747	0.87355	0.071582
ENSG0000	TMC6	17:779592	145.37	178.018	115.509	123.764	137.376	145.049	123.508	126.473	80.9426	164.608	0.735	-0.17134
ENSG0000	FAM21EP	10:500211	0.376888	0.409294	0.281732	0.692223	0.37337	0	1.54655	0.858386	0.16479	0.216432	0.8618	0.325094
ENSG0000	TBL3	16:197203	41.5116	50.1854	34.9972	43.349	37.9838	37.1113	36.9849	24.3044	38.6288	33.3789	0.57275	-0.22944
ENSG0000	RP11-671	14:345410	24.2407	17.3126	9.66699	14.1557	12.1295	20.1721	14.3026	13.2298	6.62856	14.0853	0.8583	-0.25503
ENSG0000	TRIM52	5:1812369	20.3739	22.7127	14.4112	16.8043	15.7667	16.2323	14.043	15.6457	11.6626	18.2703	0.68205	-0.34336
ENSG0000	GIMAP8	7:1504506	22.9479	33.0361	10.0745	3.49736	10.3465	13.5493	7.51335	8.45858	2.12846	18.6324	0.0881	-0.61749
ENSG0000	CREB3	9:3569694	23.9217	16.7361	40.2923	38.8097	27.5052	16.6818	22.8438	36.6379	35.2366	17.5619	0.91275	-0.1901
ENSG0000	CD302	2:1597124	1.12569	3.82562	0.272788	0.061037	0.171307	3.68551	0.526617	0.233729	0.012331	0.348927	0.8417	-0.33893
ENSG0000	CBY1	22:386566	9.69378	5.23098	11.0889	9.92751	10.2927	14.3637	10.5878	7.40297	6.04605	8.47183	0.9225	-0.04992
ENSG0000	CTD-2293	19:370078	0.359947	0.302313	0.847131	0.484498	0.266483	0	0.285472	0.349298	0	0.871714	0.6178	-0.71903
ENSG0000	ASTE1	3:1308505	21.6228	18.0042	17.5597	17.6876	13.1991	26.4986	24.3811	12.4665	17.7055	15.1883	0.79685	0.106743
ENSG0000	RP11-15A	19:438723	5.23285	3.33645	6.40467	13.1288	12.8358	7.04284	4.81119	6.26665	3.34689	11.195	0.6646	-0.46473
ENSG0000	HIST1H2B	6:2783775	9.51748	2.4098	10.3812	5.09963	3.02743	0.827098	3.88263	5.78493	4.99686	2.08366	0.326	-0.78145
ENSG0000	MAP2K3	17:212846	65.9082	73.4332	64.7055	64.4854	62.8821	41.3382	66.2683	67.4748	68.7329	93.3354	0.9769	-0.00915
ENSG0000	SLC6A16	19:492896	1.6814	2.21812	1.85793	1.40454	1.51842	0	0.741604	0.640017	0.375784	0.865575	0.1575	-1.32586
ENSG0000	TIMM8B	11:112024	72.7784	36.6815	92.9907	83.676	38.298	103.325	90.6492	69.6363	75.2589	12.3102	0.9592	0.079012
ENSG0000	SEC11A	15:846695	102.785	67.2026	109.729	103.682	91.3347	148.012	146.7	123.717	163.551	41.172	0.2362	0.380067
ENSG0000	RP13-314	X:2377299	0	0	0.066696	0	0	1.87512	0.663546	0	0	0.163837	0.0966	4.99905
ENSG0000	HNRNPA1	19:116397	4.32152	4.92738	2.28054	2.84944	2.28743	5.62906	4.64526	1.65991	1.84573	1.16935	0.94195	0.010151
ENSG0000	MFN1	3:1793476	9.70848	10.1716	4.02024	3.76974	2.59857	6.34134	9.80768	4.47643	2.37576	6.46713	0.7772	-0.1476
ENSG0000	UTP6	17:318608	26.5563	30.2262	21.7057	18.6039	20.4907	38.3476	36.1757	26.0488	5.43772	29.2557	0.8744	-0.06565
ENSG0000	COLGALT	19:175555	12.1412	11.6725	16.9615	16.2076	10.9046	16.162	8.53696	17.1091	6.19324	21.612	0.9459	-0.02522
ENSG0000	CARMIL1	6:2527907	0.24294	1.42321	1.44018	6.36836	0.115037	1.40342	0	2.9217	0	0.029883	0.47455	-1.05582
ENSG0000	ZNF181	19:347341	13.8955	5.72547	8.05753	7.0287	4.93731	7.13334	8.34712	9.69666	7.16903	11.1276	0.9476	0.035844
ENSG0000	SLC5A6	2:2719958	17.9453	21.0085	9.04384	7.35684	10.4793	11.1709	8.13488	4.42265	5.82033	6.96254	0.5331	-0.64292
ENSG0000	CNPY4	7:1000927	11.6238	15.3518	17.8465	16.1827	12.2282	1.8803	6.10744	12.869	9.28416	17.5427	0.61	-0.61476
ENSG0000	DCAF17	2:1713174	5.55047	7.66197	2.23624	2.17033	2.3718	6.56131	2.226	2.67647	10.1279	4.88389	0.4148	0.435167

ENSG0000(	ZNF767P	7:1495471	12.3696	17.9946	6.64104	6.70688	4.26147	14.7829	22.5132	20.8675	10.5961	9.62807	0.09055	0.806773
ENSG0000(	COG8	16:691055	6.52621	5.87805	17.8725	9.55789	11.8514	10.5585	9.07214	9.9827	4.86983	10.7905	0.4992	-0.64611
ENSG0000(	C1orf174	1:3889124	17.8487	15.5348	27.3555	25.5018	18.4087	19.9349	12.5092	20.6687	11.5249	13.4476	0.2471	-0.46395
ENSG0000(	CREB3L4	1:1539590	8.8115	4.64184	8.18243	4.73593	5.3639	4.00554	3.92851	2.69009	5.00006	1.35236	0.191	-0.84493
ENSG0000(	RP11-185	3:1714606	1.93674	2.37157	1.71776	1.63126	1.11443	0	3.32223	1.99638	0	0.68151	0.66495	-0.49064
ENSG0000(	ZNF235	19:442075	3.94937	5.73416	3.94534	1.90681	1.55739	6.42025	2.55652	1.96492	6.64748	5.55072	0.3717	0.657829
ENSG0000(	RP11-887	12:936773	0.343015	1.06991	0.709117	0	0.824963	0	1.70242	0.490528	0	0.079081	0.7918	-0.46529
ENSG0000(	TRGV2	7:3834157	43.125	12.3065	128.757	126.42	82.536	27.2447	40.9885	48.4497	106.006	66.2311	0.6219	-0.45873
ENSG0000(	RPS7P11	17:465906	1.4618	1.58061	0.688666	0.845977	0.186406	1.65341	2.51261	0.336013	0	0	0.98615	0.013273
ENSG0000(	IL2RB	22:371258	93.8285	60.8267	46.9318	40.3387	118.307	129.766	188.234	112.119	55.9666	146.178	0.0645	0.657524
ENSG0000(	FIGNL1	7:5044412	4.21333	4.27576	0.689839	1.82532	1.03395	9.60506	3.77922	1.68184	0	2.79411	0.3965	0.62221
ENSG0000(	RP11-802	3:5842762	1.75566	2.08622	1.23426	4.7996	1.47695	2.2948	0.391251	0	4.45161	1.21856	0.77955	-0.37297
ENSG0000(	GRAP	17:189503	18.8032	29.679	12.5803	17.3349	10.6748	17.6855	4.18049	12.9508	6.61028	18.5076	0.27555	-0.53583
ENSG0000(	CTD-2095	17:321275	12.4626	12.9919	10.4138	10.5484	8.4805	20.1009	44.8821	18.2109	21.3404	10.9035	0.0517	1.10514
ENSG0000(	ARRDC4	15:979606	0.135018	1.1867	0.07818	0	0.064842	4.49578	0.501499	0.194618	0	0	0.29535	1.73941
ENSG0000(	CCDC28A	6:1387252	68.0798	77.4873	83.5637	64.8148	52.6978	67.0436	73.8159	49.5197	42.1641	28.1122	0.2629	-0.40161
ENSG0000(	ATXN1	6:1629911	12.9919	12.3542	10.9779	13.0347	8.08283	9.32169	13.4177	16.7628	25.5368	20.8725	0.4837	0.506646
ENSG0000(	OPA3	19:455274	6.00161	8.50915	5.55928	4.5639	10.0286	3.8484	6.84394	11.1675	9.71734	19.0677	0.5557	0.363217
ENSG0000(	URM1	9:1283713	23.4783	26.2174	26.6438	22.9836	26.0697	17.4355	38.609	22.1839	26.1431	14.5163	0.6248	-0.25657
ENSG0000(	IGKV2D-3	2:8993685	0	7.10274	1.11744	0	26.0953	0	0	0	0	0.652401	0.1288	-4.15774
ENSG0000(	TRAV14D\	14:219240	10.2612	12.0317	9.70919	3.49296	2.46725	19.7134	26.6128	5.90131	4.92596	3.47848	0.3724	0.700452
ENSG0000(	DPF2	11:653337	55.1729	83.9213	72.7164	56.6071	67.5053	74.026	76.6236	52.6097	56.9366	88.7482	0.7869	0.088446
ENSG0000(	ABHD17A	19:187680	236.577	169.86	57.1331	32.9421	185.268	166.158	212.257	68.6959	31.8571	93.9287	0.3185	-0.39381
ENSG0000(	SLC12A5	20:460089	0.614525	0.857419	0.501311	0	0.113335	0	0.741995	0.182422	0	0	0.27155	-1.31702
ENSG0000(	FAM92A	8:9321330	0.746539	1.55355	0.986034	0.08312	0.575562	1.73482	5.26494	3.02957	1.79456	4.43692	0.0382	1.82837
ENSG0000(	SQLE	8:1249984	6.76506	10.9906	2.7552	2.92248	3.05945	12.5176	16.0395	20.0175	4.48509	6.39725	0.00125	1.41707
ENSG0000(	ARMCX1	X:1015505	0.04996	0	0.655787	0.353909	0.03948	3.77002	0	0	0	0.746939	0.3755	1.74237
ENSG0000(	RP11-305	9:9111906	0.417313	0.529289	1.10035	1.98894	1.72931	0	5.0484	4.31412	1.64747	0.128897	0.64585	0.874791
ENSG0000(	STX10	19:131440	66.9418	99.2764	34.7562	39.7553	45.1049	110.859	117.251	66.5333	59.5218	54.0615	0.1694	0.505787
ENSG0000(	API5	11:433119	71.9018	73.0194	19.6567	31.2495	17.854	48.2494	38.42	20.8403	29.625	18.7731	0.28635	-0.43786
ENSG0000(	PHLPP2	16:716237	1.01581	0.631584	0.615018	0.2833	0.532095	0.368614	0.805493	1.4059	0.459858	1.6626	0.28385	0.614286
ENSG0000(	HLA-DQA	6:3274134	0	3.71129	0.124464	1.23613	1.13044	0.746694	2.41849	0.101847	3.06153	1.43726	0.43795	0.380954
ENSG0000(	IPO9-AS1	1:2016228	1.04313	0.497048	1.05378	0.814001	0.450218	0.33466	1.41253	0.533016	1.88824	0.201813	0.91685	0.12426
ENSG0000(	RP5-1165	7:4392643	0.809736	0.370747	0.382867	0	0.160233	1.87153	0.825634	0	0	1.14491	0.6014	1.10263
ENSG0000(	RAB12	18:860944	0.095726	0.32469	0.24453	0.185093	0.248885	0	1.31324	0.190441	0.427554	0.735211	0.486	1.21427
ENSG0000(	TNFRSF13	17:169229	0.062917	6.68698	1.19274	3.18264	2.87369	10.0735	2.9007	0.982834	3.35902	0.399904	0.70365	0.348677
ENSG0000(	NCDN	1:3543348	7.23756	13.7395	4.82616	4.54358	3.67673	7.60487	10.7757	6.38017	6.5269	7.76973	0.8261	0.192518
ENSG0000(	CYP2D7	22:421402	1.02085	1.50099	1.14902	0.507979	0.914482	1.63752	2.64769	1.12356	1.8652	0.061707	0.58545	0.538083
ENSG0000(	IGF2R	6:1599690	9.32762	5.11367	1.71907	1.87948	4.35328	5.71944	5.45045	2.68245	2.11458	7.10285	0.84635	-0.078
ENSG0000(	SPON2	4:1166931	34.6427	40.7413	43.3109	64.9916	72.5682	19.5988	13.1161	14.9441	24.79	14.7551	0.0002	-1.59743
ENSG0000(	DENND6A	3:5762545	5.87487	6.8421	12.2014	10.6018	11.3174	3.59762	3.83554	6.42698	6.05337	17.7379	0.43	-0.49545
ENSG0000(	CKB	14:103519	1.55982	0.63297	1.4262	2.16518	0.567407	0	2.46468	0.591385	0.264455	0.316588	0.0705	-1.45454
ENSG0000(	WDR5	9:1341322	10.5928	13.4344	9.77515	5.80225	8.62355	10.6257	13.6175	10.2526	6.81306	13.9603	0.81055	0.09993
ENSG0000(	CCNG2	4:7715715	9.75466	13.0235	4.58268	3.73589	4.47247	7.86801	7.25893	4.74842	5.65055	8.78025	0.54455	-0.25297
ENSG0000(	RP11-45A	10:131966	0.925486	1.49593	0.870987	0.93047	0.533892	0	0.863821	0.568287	1.52851	0.62045	0.9263	-0.11158
ENSG0000(	SELENOW	19:477785	113.901	127.037	210.674	193.087	115.085	118.596	154.544	191.929	194.926	28.4092	0.82265	-0.08438
ENSG0000(	FER	5:1087478	0.779658	2.05026	0.546661	0.981612	0.574773	5.81163	0.113996	0.787047	5.02225	1.76802	0.23475	1.3745
ENSG0000(	PHACTR2I	10:119726	0.119518	0	0	0	0	0.859147	0.782382	0.032041	0	2.4043	0.1216	5.2171
ENSG0000(	SLC25A28	10:996105	20.8456	25.1004	7.02908	5.01563	9.13633	28.2508	29.7669	17.1174	7.34567	23.8268	0.168	0.676447
ENSG0000(	DLEC1	3:3803920	0.914572	0.914214	0.54289	0.226961	0.53692	0	0.304678	0.164082	0.517694	0.847676	0.49545	-0.58981
ENSG0000(	FLNA	X:1543485	351.147	263.558	78.2986	59.4149	153.884	127.943	105.584	105.982	52.9545	142.529	0.6533	-0.28458
ENSG0000(	ENSA	1:1506008	32.7221	18.5205	37.5196	42.5928	35.0171	29.5226	39.921	55.9902	43.9313	24.2902	0.7569	0.131226
ENSG0000(	ZNF276	16:897071	68.4537	79.2795	49.031	34.6194	43.3427	67.482	47.7244	40.048	35.3563	73.3237	0.7254	-0.13835
ENSG0000(	FAM220A	7:6329408	2.51132	1.16094	9.64284	12.3694	8.99648	1.9683	1.52891	12.8575	11.0386	7.18964	0.89075	-0.07285
ENSG0000(	CEP135	4:5594887	7.88292	13.7731	4.17638	3.04147	4.16238	14.148	8.44971	5.85586	14.1896	7.37541	0.3373	0.557829
ENSG0000(	ANKDD1A	15:648418	0.604729	0.688937	0.437848	0.267343	0.176267	1.58153	0.864378	1.71608	2.02751	1.19367	0.14025	1.55208
ENSG0000(	SPG21	15:649630	81.4191	101.603	79.0921	70.381	73.3972	124.39	71.1672	55.8131	73.6472	53.823	0.6872	-0.12272
ENSG0000(	ZNF25	10:379495	4.92596	6.71369	2.13285	2.50501	1.40684	5.47198	2.48919	0.583812	4.1563	3.08225	0.6989	-0.19162
ENSG0000(	CTD-2506	14:811754	1.24649	0	1.64108	0.438209	0.915995	0	0.419608	0.490962	0	0	0.3069	-2.26608
ENSG0000(	COX8A	11:639746	156.788	168.786	289.924	283.21	126.977	322.677	271.339	327.707	221.532	47.9687	0.84615	0.155973
ENSG0000(	CTB-31O2	19:181524	0	0	0.447775	0.74101	0.701642	0	0	0.997712	1.17398	0	0.92425	0.273294
ENSG0000(	CREB1	2:2072396	22.7214	22.2715	17.49	13.234	14.5796	16.0341	18.9763	25.0613	26.5825	26.9769	0.888	0.070188
ENSG0000(	TRBV10-1	7:1423998	4.19775	13.7089	6.35642	11.1425	1.07979	13.8739	4.57769	5.14682	0.52785	1.47915	0.5671	-0.49439
ENSG0000(	AC104820	2:1809672	8.06647	5.22863	7.29945	8.78907	12.1333	13.3606	15.1844	13.8311	13.9979	8.34277	0.5698	0.613605
ENSG0000(	HIST1H2B	6:2611487	15.6442	5.05167	14.5656	12.055	4.43107	1.95481	6.65988	4.49786	5.50791	1.00201	0.2306	-1.44156
ENSG0000(	RP11-290	12:985298	0.823622	0.753894	0.049462	0.916994	0.472188	0.565032	0.166543	0.683077	0.158189	0	0.5803	-0.78916
ENSG0000(	ZNF627	19:115593	3.35465	2.30849	4.16487	2.85136	2.51847	3.74048	2.8943	5.10065	3.40582	2.03106	0.85075	0.153618
ENSG0000(	SLC35E3	12:687461	2.72508	2.17827	4.34454	2.79973	1.6533	1.12898	2.41696	1.8166	4.53554	3.32844	0.7999	-0.22121
ENSG0000(	SNHG12	1:2855308	6.92401	8.96481	16.0093	15.0902	11.1561	20.5094	34.6019	21.3683	3.58837	15.3817	0.40695	0.563398
ENSG0000(	HMGCR	5:7532015	10.0851	11.2264	1.90207	4.29884	2.28802	13.4802	7.30815	5.97073	5.60839	4.9867	0.28855	0.46487
ENSG0000(	HAUS1	18:460802	41.8214	31.4937	47.2479	40.8993	29.3432	40.7927	34.8374	34.1813	55.6287	14.1108	0.97295	-0.03779
ENSG0000(	ARL15	5:5388394	9.02836	9.41857	6.60066	4.47829	4.59584	5.65179	9.41816	7.59812	4.40929	10.2579	0.99925	-0.0007
ENSG0000(	bP-2189C	21:885725	1.44596	0.469489	0.285785	1.66691	1.2289	1.14381	0.202786	0.129486	0.519484	1.88176	0.80035	-0.51896
ENSG0000(	PMAIP1	18:598999	81.9036	29.3653	58.8417	151.914	33.1945	20.0013	52.004	32.1256	83.4046	13.0459	0.0342	-0.82822
ENSG0000(	DHRS7	14:600919	161.672	87.3845	213.097	174.967	207.646	157.362	156.778	164.638	196.493	138.76	0.78215	-0.1065
ENSG0000(	ZNF300	5:1508465	1.97302	0.035162	0.345905	0.450894	0.306766	0	0.228151	0.008166	0	0.162101	0.05245	-2.95603
ENSG0000(	RGS12	4:3293027	1.3323	2.63628	0.313788	0.796203	0.183509	4.84519	2.52734	1.7099	0	1.0639	0.3348	0.625368
ENSG0000(	GPSM1	9:1363274	1.24083	0.997054	1.24312	0.43544	1.13969	0	2.07532	3.00492	2.0581	2.13234	0.3481	1.00714
ENSG0000(	RP4-694B	20:256129	0.46333	0	0	0	0	1.83379	0.675804	0	0	1.00961	0.2577	3.36932
ENSG0000(	RP5-1050	17:498770	1.36714	4.01143	2.22771	1.55073	2.49666	3.68715	3.1116	0.835202	6.38014	2.03038	0.8338	0.490731
ENSG0000(	HLA-DRA	6:3243984	48.7119	329.459	81.0957	155.479	59.4238	950.921	206.05	332.197	209.322	79.0306	0.00275	1.38878
ENSG0000(	C14orf80	14:105486	16.1088	18.843	10.6934	19.6544	14.1171	21.655	18.0985	11.107	10.2895	18.6795	0.98075	-0.01792
ENSG0000(	RP11-44N	8:1227799	1.95005	0.191333	0	1.18835	0.205233	0	0.435881	1.49046	0	0	0.6886	-0.52239
ENSG0000(	TTC37	5:9546389	20.5737	23.6324	8.5327	10.0724	10.9739	16.2646	23.7499	8.95578	10.637	13.6834	0.9009	-0.05169
ENSG0000(	PCID2	13:113177	72.5259	35.0368	77.4549	57.259	69.5535	46.677	76.298	43.2839	49.6575	52.421	0.68555	-0.1995
ENSG0000(	PLAA	9:2690408	14.7431	14.1945	6.9838	5.18858	5.43686	25.603	19.5762	10.9299	9.27033	5.21786	0.33545	0.507497
ENSG0000(	AURKAIP1	1:1373729	126.507	116.169	186.36	187.144	251.746	153.236	182.125	175.786	145.766	72.0448	0.31305	-0.36118
ENSG0000(	LIMD1	3:4555539	4.0081	7.76443	1.47331	0.671924	0.815831	3.41174	4.50136	1.94072	2.73829	4.48635	0.90705	0.149587
ENSG0000(	AP001062	21:443289	1.40463	2.37504	0.3556	0.150286	0.556872	1.12567	0.697834	0.626061	1.63823	0.779431	0.9777	0.054253
ENSG0000(	TBC1D22E	6:3721217	3.87382	2.29748	1.55029	4.28888	1.77056	2.57771	1.85799	1.74653	4.15841	3.5059	0.99785	-0.00183
ENSG0000(	ZNF512B	20:639398	1.40819	1.08439	0.194589	0.282303	1.21327	0.984919	0.940804	1.34606	1.03119	1.88271	0.83085	0.55718

ENSG0000(	GMDS	6:1623805	17.3958	20.689	15.217	9.97971	21.9743	15.6675	23.6712	11.316	1.33136	20.4814	0.36085	-0.40723
ENSG0000(	PHTF1	1:1136968	21.9067	15.8368	17.4708	13.4492	15.1308	20.6623	38.6413	22.8435	28.1579	24.6453	0.44155	0.55225
ENSG0000(	RPL36A	X:1013908	342.713	322.773	654.959	447.629	269.305	299.613	422.311	362.815	244.088	36.494	0.40835	-0.63137
ENSG0000(	SMG7	1:1834608	14.5822	16.9568	4.16344	4.36063	6.40954	9.74426	10.4415	5.96127	3.82703	14.2173	0.811	-0.11206
ENSG0000(	RP11-288	6:1599690	0.657856	0.854596	0.539801	0	0.20086	0	0.576495	1.11796	0.144412	4.17639	0.4938	1.30393
ENSG0000(	SH3BP5	3:1525418	26.6108	38.77	36.6656	41.0636	86.5695	13.817	15.7566	17.8515	24.6072	46.9799	0.0403	-0.99482
ENSG0000(	RPS26P52	16:139201	0	0.401538	0	0	0	0	0	0.182194	3.83131	0	0.3988	3.70618
ENSG0000(	NDUFB9	8:1244884	189.747	168.908	338.819	343.905	139.957	202.534	215.808	237.272	228.722	32.0728	0.3493	-0.40602
ENSG0000(	JDP2	14:754236	0.334309	4.02586	0.160751	0.353555	0.080232	0	0.258273	0.412579	0	0.382327	0.0894	-2.44495
ENSG0000(	RP11-324	10:352470	0.744998	0.284776	1.20538	0.979477	0.590819	0	0.089753	0	0	1.97775	0.6462	-1.09329
ENSG0000(	NUP188	9:1289208	16.4751	21.5647	7.62872	7.16883	3.98419	7.57246	11.737	8.04826	4.37551	7.50573	0.336	-0.43138
ENSG0000(	LINC0086	10:898295	0.393509	3.50714	0.183873	0	0.069647	0	0	0	0	0.300206	0.3502	-3.38417
ENSG0000(	USP9Y	Y:1270123	9.2848	11.5934	2.7912	4.27836	2.35692	6.81146	7.84931	10.5899	2.1051	9.92141	0.22025	0.740906
ENSG0000(	TEN1-CDI	17:759415	0.568409	1.02377	1.17293	1.74803	2.31402	2.5381	0.797366	1.52474	2.5978	1.57255	0.4538	0.992672
ENSG0000(	RP11-383	8:1243013	0.092017	0.490508	0.154903	0.278231	0	1.54098	0.285842	0	0	0.31351	0.4892	1.04361
ENSG0000(	OFD1	X:1373474	67.3461	52.5922	40.5057	31.5708	50.142	55.5263	47.6307	30.6473	46.3624	89.9821	0.88875	0.057334
ENSG0000(	PAK1IP1	6:1067141	16.3374	14.3041	22.9016	21.6073	19.0087	16.5379	16.1024	21.8687	16.8784	11.9926	0.54085	-0.25009
ENSG0000(	C16orf54	16:297424	10.6105	59.6463	170.733	121.925	96.1485	2.13796	25.3937	82.7363	68.2915	94.7965	0.05235	-0.75655
ENSG0000(	GADD45G	9:8960501	7.12762	4.05551	1.2422	1.39429	0.760759	158.578	96.751	70.7815	118.083	64.1577	0.01	5.1603
ENSG0000(	TRAV13-2	14:219181	8.71836	21.1793	30.7829	13.1879	11.0696	59.1545	20.8341	14.7525	12.6839	4.36676	0.58285	0.299841
ENSG0000(	UPRT	X:7527408	13.0618	12.4975	10.9961	16.05	9.50341	12.5151	11.8322	12.2473	15.2472	7.94161	0.92135	-0.04383
ENSG0000(	AGFG2	7:1005392	9.56237	13.2827	5.99824	1.48011	8.93033	10.1432	25.551	12.9761	2.6026	13.6754	0.4515	0.393962
ENSG0000(	ZNF674	X:4649772	0.832632	1.30174	0.501084	0.999397	0.397013	0.400801	1.17139	0.920778	1.04891	1.49258	0.2518	1.30417
ENSG0000(	RP11-343	1:2066849	0.390626	0	1.69417	0.844918	0.208585	0	0	1.38094	0.992478	0	0.9238	-0.20293
ENSG0000(	RP11-66N	14:235117	5.19911	6.54098	1.77215	1.09405	0.871453	3.52094	3.53038	1.73718	0.923773	2.10617	0.75585	-0.40977
ENSG0000(	RP13-131	3:4891778	1.13	1.42375	1.12797	0	0.77488	0.148445	1.7895	2.87973	2.80922	1.12516	0.2361	1.83244
ENSG0000(	AC138645	17:465115	2.09051	3.98316	3.04429	5.09781	6.49494	5.73782	7.00183	6.74611	0	6.68094	0.74595	0.907052
ENSG0000(	CHKB-CP	22:505688	3.37578	7.2094	0.899082	1.84169	1.16198	3.3616	7.31656	0.964801	3.13185	2.27147	0.4999	0.653457
ENSG0000(	ZBED5-AS	11:108092	11.5296	20.0511	12.7837	12.7887	8.45053	16.6729	11.9434	7.17567	13.8988	5.8464	0.83045	-0.21689
ENSG0000(	ZDHHC5	11:576677	19.7149	22.415	9.4644	8.76925	5.54712	28.8752	15.2257	14.5218	4.78929	14.8561	0.7173	0.127041
ENSG0000(	UBXN8	8:3073224	8.42704	4.79085	5.39312	4.2874	4.3417	7.45957	12.7354	4.06073	11.3132	1.63146	0.44005	0.460444
ENSG0000(	VDAC3	8:4239162	140.356	147.352	164.064	167.469	102.848	176.599	146.626	99.603	179.566	71.9781	0.8126	-0.07761
ENSG0000(	NME4	16:396724	11.4866	31.3018	43.3963	30.5001	33.7419	3.54424	8.51776	10.7739	14.1613	11.5791	0.03465	-1.588
ENSG0000(	LINC0118	5:1277033	12.405	14.0617	15.5422	17.6947	11.3321	6.99634	12.0821	14.4875	12.4871	6.7952	0.4271	-0.49296
ENSG0000(	KIAA0196	8:1250462	23.6992	21.0445	9.02423	15.3767	7.18409	8.01335	12.2872	7.59759	16.0279	15.0239	0.7289	-0.15804
ENSG0000(	ANO7L1	1:1621590	0	0.217588	0.272033	0	0.13453	0	1.23197	1.77036	0	0	0.3869	2.10994
ENSG0000(	H2AFY2	10:700527	3.99581	3.44226	5.30516	3.71473	4.6156	5.92181	3.08604	7.58427	7.89537	4.95523	0.629	0.444557
ENSG0000(	RERE	1:8352396	4.89221	11.3273	4.29618	4.50422	9.1241	5.51617	7.42551	5.48393	11.9208	14.6382	0.35955	0.520279
ENSG0000(	ZNF746	7:1494727	11.6931	10.9416	6.78282	6.08568	16.0384	3.50761	9.92769	7.04708	6.51594	18.8774	0.61995	-0.21333
ENSG0000(	RNF144A	2:6912276	1.59974	5.07591	2.09541	1.59141	1.88811	0.915501	0.24583	0.627435	2.25404	3.63543	0.36675	-0.66469
ENSG0000(	RNF19B	1:3293644	15.0429	15.39	3.2646	1.97669	3.49791	9.80906	14.2295	7.84537	5.06551	7.01414	0.73545	0.184651
ENSG0000(	C16orf70	16:671099	6.55541	9.72487	5.13493	8.22843	4.51112	5.76805	9.11835	5.44497	8.48732	6.40468	0.9537	0.028505
ENSG0000(	KLF12	13:736860	18.7983	33.6404	35.4862	29.4263	100.135	9.5288	8.85478	18.0441	14.7073	60.8881	0.0749	-1.10004
ENSG0000(	BCAP31	X:1537004	193.774	183.574	214.672	170.877	259.428	242.757	160.962	154.224	133.782	116.015	0.277	-0.35735
ENSG0000(	ELK3	12:961943	24.8819	23.1676	11.1767	16.8015	19.5418	25.9651	8.78565	14.3651	23.5906	22.7949	0.91915	-0.03533
ENSG0000(	CMC4	X:1550616	18.0394	22.8131	12.6235	12.9945	13.4606	40.0712	22.5607	15.4002	3.78027	4.32979	0.97215	-0.03159
ENSG0000(	ZNF41	X:4744587	3.80005	3.86227	0.843968	1.0249	1.21853	0.564301	1.99332	1.83658	1.34135	2.73862	0.60995	-0.29549
ENSG0000(	PISD	22:313995	23.4483	24.6253	40.8208	37.8797	40.2849	27.8462	42.9764	36.6957	24.1811	49.6394	0.99945	-0.00073
ENSG0000(	RYK	3:1340653	12.761	9.34581	6.38681	3.66611	6.21891	12.776	3.8765	4.70924	6.22366	11.0129	0.88585	-0.06371
ENSG0000(	NELFCD	20:589812	55.4781	71.3887	47.3703	57.1791	53.6694	45.7389	57.0338	48.9521	36.1073	63.5519	0.5087	-0.22335
ENSG0000(	ERMAP	1:4276726	1.26424	2.89671	0.434351	0	0.301528	0.357297	1.15498	1.17801	3.60212	0.947036	0.55495	0.78822
ENSG0000(	THRAP3	1:3622441	71.8714	78.6933	91.6641	78.9408	114.908	53.6661	73.4593	57.9278	76.1719	160.269	0.552	-0.20467
ENSG0000(	PTPRA	20:283531	26.6321	33.1122	24.6169	20.0743	36.3791	24.3609	30.5622	21.4271	15.3264	44.702	0.3419	-0.41073
ENSG0000(	OARD1	6:4102691	33.798	29.8352	36.6292	39.1746	30.5819	41.0842	40.3806	40.6796	36.0257	17.2802	0.94825	0.033263
ENSG0000(	PAIP2	5:1393415	285.336	238.47	302.473	263.246	232.392	221.39	248.545	228.524	295.818	96.0526	0.4499	-0.2627
ENSG0000(	AC008746	19:544386	0.168308	0.583063	1.29582	1.98334	0	0.105783	1.2633	0	3.02265	0.122156	0.90535	0.213176
ENSG0000(	TFDP1	13:113584	50.7202	96.669	33.9915	31.5443	31.5368	95.4978	63.3356	50.1608	39.7939	31.43	0.52865	0.250565
ENSG0000(	ARF4	3:5757136	105.515	100.894	130.557	129.358	93.3069	87.1068	104.815	103.206	80.9328	71.801	0.21905	-0.3767
ENSG0000(	ZNF280B	22:224844	0.845498	0.863936	1.00283	0.849657	1.20794	0.317078	1.10118	0.811045	1.61995	2.04812	0.78345	0.224125
ENSG0000(	AC006547	22:200802	1.1166	0.894696	0.742496	0.688996	0.933879	0	3.21753	0.546211	0	1.04183	0.54275	1.40691
ENSG0000(	ZNF561	19:960467	7.48732	8.66692	6.97666	7.84656	5.72098	22.2398	16.993	9.89647	9.29276	9.6255	0.19915	0.766335
ENSG0000(	PAFAH1B3	19:422970	23.3159	30.8983	40.7341	23.3332	36.4918	40.4465	37.7687	25.0594	10.5743	7.78584	0.2682	-0.50866
ENSG0000(	ILDR1	3:1219873	0	0	0	0.142057	0.012775	2.22275	0.798959	0	0	0	0.19985	4.80928
ENSG0000(	TRMT2A	22:200802	61.8098	75.3311	34.5795	50.0071	32.4473	90.8741	69.5681	35.845	39.4388	37.181	0.88725	0.072055
ENSG0000(	MARCKSL	1:3233383	19.4961	78.9335	15.2764	9.03847	12.2212	17.5918	17.4416	13.8693	13.6476	11.3239	0.1115	-0.75535
ENSG0000(	TCP1	6:1596690	106.913	107.756	77.0784	98.6725	100.745	94.1667	113.235	101.653	115.344	92.9224	0.95245	0.031888
ENSG0000(	PRADC1P	3:3697631	0	0.278587	0.441796	0	0	0	0	0.832128	1.89036	0.090971	0.16915	2.2084
ENSG0000(	MTURN	7:3013480	2.46268	4.66549	0.849811	0.523573	0.296428	1.64859	3.31911	0.464414	0.03592	0.369848	0.50525	-0.7375
ENSG0000(	OS9	12:576939	81.8403	66.9437	77.2787	72.0063	82.6884	72.676	60.3906	68.3725	90.6462	106.146	0.7439	0.100441
ENSG0000(	PIP4K2A	10:225348	58.5596	28.7976	89.8215	22.7966	60.6478	35.4088	32.6266	53.4953	23.5246	85.1925	0.4369	-0.32352
ENSG0000(	USP14	18:158382	19.2834	22.0504	14.7975	16.1821	15.7958	12.2659	24.1892	13.9286	9.45137	21.9632	0.45085	-0.33995
ENSG0000(	HDGF	1:1567421	49.6553	55.1812	27.6064	25.7954	45.7077	43.6572	44.5312	39.6814	47.8304	60.6808	0.61865	0.187522
ENSG0000(	ZRSR1	5:1127074	1.12755	0.132339	1.00007	0	0.152949	0	2.31954	0	0	2.26558	0.83035	0.433684
ENSG0000(	C8orf46	8:6642902	1.57725	0	0.291016	2.04906	0.200345	2.00785	0	0.25413	2.96566	0.6158	0.5298	0.732575
ENSG0000(	AKR1A1	1:4555054	73.0756	61.0063	109.175	127.675	111.393	158.399	88.3626	118.478	73.9483	73.004	0.85325	0.060968
ENSG0000(	TEKT4P2	21:906836	1.55891	0.02825	1.01614	4.03455	0.205854	2.94948	0	0.275307	0.82495	0.358038	0.6672	-0.75514
ENSG0000(	COX6A1P	6:3704485	3.91751	1.60874	0.52347	2.53451	0.500066	3.05645	2.88435	0.962527	1.30979	0.232704	0.9386	-0.13874
ENSG0000(	RP11-277	1:1672198	0.480852	2.71953	0.696556	0.628873	1.72305	0	0.85663	1.44364	0	0.116878	0.4155	-1.439
ENSG0000(	NDUFA8	9:1221440	55.3543	42.2224	83.3423	63.2462	75.3424	52.5823	59.7682	52.4991	66.411	18.8866	0.29005	-0.40255
ENSG0000(	PPA2	4:1053690	48.9213	52.162	73.3036	67.7955	48.1998	45.1211	54.4137	59.0162	67.7382	16.3243	0.35495	-0.31284
ENSG0000(	C17orf58	17:679911	1.37649	0.4775	4.08706	5.55877	1.93733	11.85	3.88878	6.56363	6.37355	2.3701	0.1728	1.13012
ENSG0000(	PNO1	2:6812293	13.0057	10.5256	15.7952	13.0375	9.44153	14.9286	15.9956	15.3148	7.3749	5.92837	0.92855	-0.04163
ENSG0000(	GAPDHP3	19:464807	0	0	0.042679	0.256981	0	2.72402	0	0	0	0	0.46465	2.85858
ENSG0000(	UFSP1	7:1008887	1.70517	1.4466	2.26468	0.49987	0.93352	1.51076	2.26923	2.89711	3.05186	0.380746	0.6467	0.57037
ENSG0000(	ST3GAL6	3:9871432	0.824962	1.9607	0	0	0.04275	22.4332	0	1.52108	0	0	0.1001	3.04586
ENSG0000(	RP9P	7:3291681	1.18555	1.36525	0.595618	1.53894	0.928346	5.19379	1.28202	0.478734	1.2321	0.617826	0.63405	1.07476
ENSG0000(	ZNF440	19:118142	1.38613	2.4636	1.16326	0.958433	0.485736	3.48968	0.53237	1.61997	6.99347	1.47872	0.11975	1.10972
ENSG0000(	ACTG1P1(	X:5309370	0.557889	0.792304	0.451341	0.497719	0.236659	0	0	1.01741	0	2.76589	0.7598	0.637963
ENSG0000(	ZNF611	19:527028	7.30629	10.6898	3.96696	7.68841	4.41961	9.7066	8.44286	1.78639	6.74604	4.75511	0.84275	-0.11963
ENSG0000(	CLEC2B	12:985298	114.996	45.4364	87.8038	103.946	46.9443	107.212	108.086	89.929	68.6324	27.148	0.851	0.070795
ENSG0000(	TPCN2	11:690488	2.65334	12.7435	1.22106	4.0123	2.00772	5.05174	7.80259	4.0872	10.1058	3.98857	0.4902	0.427335

ENSG0000	WASH2P	2:1135885	1.78006	2.33333	1.15993	1.09106	1.36326	3.8241	2.22459	1.64894	1.12853	3.38907	0.52855	0.807191
ENSG0000	SLC38A7	16:586651	4.96084	6.60074	2.3933	5.0241	2.76723	8.73071	3.60691	6.04065	4.3919	8.04311	0.60145	0.23569
ENSG0000	COG1	17:731649	21.8572	25.5949	22.0856	20.2663	27.467	18.1434	16.5007	23.718	15.596	40.8128	0.9596	-0.03007
ENSG0000	LY9	1:1607960	115.19	92.5715	176.837	146.985	138.306	59.6051	79.6804	87.8639	77.819	118.397	0.06955	-0.70926
ENSG0000	AGL	1:9985008	2.55791	1.74668	0.78126	0.764804	1.46356	1.17741	1.20147	1.28551	0.227085	2.52073	0.51075	-0.34288
ENSG0000	BCOR	X:4004981	4.60172	7.60062	2.87391	3.37734	4.64168	3.00084	4.29919	8.37285	5.04047	9.15675	0.22725	0.636612
ENSG0000	SMKR1	7:1295024	10.4039	2.19882	12.4152	7.22109	10.1115	2.34572	8.40863	3.69132	4.52036	2.88353	0.12855	-1.00471
ENSG0000	MED27	9:1318601	31.4841	37.3213	27.6022	32.5264	26.1291	33.092	26.6073	30.8727	14.5034	26.9894	0.5002	-0.25067
ENSG0000	LYZ	12:693483	82.3712	36.0426	19.0034	21.0155	8.71304	522.478	6.43828	20.1323	25.7991	7.81993	0.0356	1.86974
ENSG0000	XXbac-BP	6:3072020	0	1.712	0.447639	0.401609	0.326937	0	4.55652	0.692094	0	2.37218	0.64555	0.784123
ENSG0000	ARHGEF33	2:3887596	0	0	0	0	0.010047	3.68956	0	0	0	0	0.0004	9.06879
ENSG0000	RBM33	7:1554742	37.3881	64.3379	71.0781	37.5348	97.2114	46.1384	58.4966	90.3834	33.6787	161.837	0.6839	0.192787
ENSG0000	PLS3	X:1155181	5.03297	1.22122	0	0.451244	0	8.92907	2.63672	3.31954	0	0.194004	0.3067	1.39256
ENSG0000	TIMP2	17:788529	0.173798	1.23453	0.258442	0.100803	0.219291	3.54765	0.476067	1.76225	0.074478	0.698965	0.16935	1.78215
ENSG0000	ARFGAP3	22:427965	41.054	20.9321	51.7579	58.1171	38.897	47.16	27.9325	35.6935	21.0183	24.146	0.2153	-0.47746
ENSG0000	EIF3EP1	6:7320908	0.861261	0	0	1.01603	0.275803	0	1.99936	0.649592	0	3.54607	0.56475	1.56006
ENSG0000	FAM212A	3:4980325	0.122899	0.854665	1.06443	1.66463	1.0999	0	0.826716	0.531218	0	0.106562	0.4003	-1.94301
ENSG0000	RNF2	1:1850453	10.0893	8.15513	7.85708	10.1867	8.28679	7.29654	9.272	6.8498	17.635	5.79764	0.9642	0.022659
ENSG0000	HAUS5	19:356127	13.6625	14.248	5.4343	6.78483	6.27576	13.6102	13.0731	5.75562	8.64511	7.95845	0.82175	0.107164
ENSG0000	ZNF596	8:213185-	2.17441	2.34547	2.02586	1.4243	1.09918	4.75376	2.18881	0.771582	2.67256	2.73878	0.4929	0.662492
ENSG0000	PPP2R5C	14:101761	249.542	162.63	261.488	290.575	124.692	106.289	152.304	191.191	260.118	110.402	0.2973	-0.47596
ENSG0000	FAM204A	10:118297	75.7034	44.5618	56.7847	54.6232	61.4216	75.7122	90.7191	41.9653	68.4472	22.2072	0.9476	0.032089
ENSG0000	RPL36A-H	X:1013908	12.2766	7.54275	9.3373	27.3593	1.39327	5.0741	3.43943	2.00223	8.94622	2.02747	0.544	-1.19472
ENSG0000	RNF126	19:647525	98.8721	101.044	114.158	131.269	143.562	61.6202	80.2099	79.7189	63.7131	67.4873	0.0172	-0.79449
ENSG0000	HIPK1	1:1139239	8.42994	7.17553	7.48829	4.12212	10.6583	6.85766	10.911	13.2368	3.90693	25.7155	0.2311	0.486292
ENSG0000	CTC-338M	5:1812369	8.43048	12.5297	8.34058	10.2215	5.742	10.0353	5.69373	8.55628	12.081	12.036	0.6573	0.35016
ENSG0000	PEBP1	12:118063	129.88	115.657	213.756	199.357	220.737	158.148	156.768	144.431	167.34	76.6063	0.2154	-0.40765
ENSG0000	ALOX15B	17:803901	0	0.417033	0	0	0	3.23394	0	0	0	0	0.4818	3.29109
ENSG0000	OBSCN	1:2282035	2.28021	3.82549	1.157	0.508456	1.25224	1.3052	0.908141	2.99515	1.27733	4.17716	0.6132	0.448515
ENSG0000	RP11-22B	X:5180300	0.244058	1.33651	0	0	0.034274	0	0	0	2.60639	0.350607	0.74135	1.08673
ENSG0000	AC138969	16:163103	16.5013	22.1119	4.10675	4.99737	4.98036	14.1723	14.2971	5.11368	6.29766	10.4167	0.38545	-0.47594
ENSG0000	DBT	1:1001869	3.03235	3.93465	1.11444	2.06661	1.23385	0.583943	1.38794	1.2473	3.32898	2.92989	0.3784	-0.50415
ENSG0000	PKD2	4:8800766	4.73455	4.22451	0.928269	0.661903	1.17625	2.70882	4.35999	4.27834	0.069411	2.41453	0.6949	0.200803
ENSG0000	TMEM160	19:470459	86.4156	63.0701	139.25	124.462	157.654	112.92	121.29	99.1546	108.347	37.0184	0.22655	-0.42117
ENSG0000	PRPF38A	1:5240456	21.9637	21.5272	19.1313	14.7057	20.364	27.7792	24.7161	18.153	15.4402	17.6813	0.98655	-0.00943
ENSG0000	ICOS	2:2039367	20.5435	24.0796	8.10287	13.4099	5.74582	34.2385	84.6138	36.137	72.6985	27.4765	0.00005	2.04213
ENSG0000	SPAG8	9:3579215	1.81774	2.36152	1.48681	2.22424	0.55809	0.087223	0.40907	0.923794	1.54884	0.45139	0.1275	-1.34802
ENSG0000	XPNPEP1	10:109864	26.6356	17.4455	29.4573	26.5412	28.6473	31.9157	23.8267	19.3446	20.4324	31.2016	0.85945	-0.05722
ENSG0000	CASP9	1:1545672	6.9898	5.49579	7.66123	7.43388	7.33223	5.22397	7.0352	6.49015	7.68533	7.40807	0.828	-0.11961
ENSG0000	DSTNP1	21:466351	1.62951	4.14069	0.778633	0	0.242915	1.08425	1.36612	0.127699	0	1.43424	0.7807	-0.46925
ENSG0000	ANXA11	10:801508	114.914	184.498	82.1513	66.7892	104.385	108.849	146.894	80.1833	49.0969	94.0383	0.4763	-0.23056
ENSG0000	PRKXP1	15:100553	0.953753	0.961698	0.594378	0.919755	0.078378	2.32285	0.339832	0.476322	0.478558	0.321058	0.94635	0.095856
ENSG0000	NOL11	17:677178	36.5982	45.6108	16.5819	22.5216	16.4249	30.6723	27.1814	17.3747	16.009	20.4209	0.4118	-0.28849
ENSG0000	RP11-386	1:7917917	153.932	31.2915	54.721	98.5725	14.6356	5.81258	2.83587	4.78471	41.869	4.74228	0.1437	-2.19293
ENSG0000	SATB1-AS	3:1715716	40.4173	28.0883	5.39395	6.00894	8.68964	9.30469	7.25495	5.58844	2.37289	7.16582	0.195	-1.32737
ENSG0000	YBEY	21:462863	6.80094	7.86103	9.26565	3.75855	11.0075	16.2544	4.949	9.03072	2.21465	3.37894	0.7494	-0.22672
ENSG0000	B4GALT7	5:1776000	12.2557	16.0448	12.1869	15.5397	11.4471	14.8523	17.6262	8.12248	13.9934	8.20435	0.76125	-0.12915
ENSG0000	RP11-138	17:163807	37.4956	41.7767	39.3877	40.9627	50.4672	90.6566	28.9323	34.7399	35.7747	17.3335	0.9843	0.025894
ENSG0000	PPP1R32	11:614026	1.61837	3.12335	1.75707	1.39499	1.02021	4.77868	3.83034	5.04768	6.53067	2.10475	0.08345	1.43809
ENSG0000	KDM6B	17:783990	0.898262	2.75465	1.19008	0.906196	2.44801	1.64488	3.69367	3.83375	1.89873	4.47953	0.14385	0.831028
ENSG0000	GTF2E2	8:3057831	70.5528	49.7538	49.086	48.9326	52.2234	78.4517	75.9983	39.7664	59.7488	28.2578	0.9723	0.011277
ENSG0000	SLC35F5	2:1137050	8.97751	6.94607	3.95987	5.10896	2.63192	16.4743	6.85344	6.67228	1.68525	4.3773	0.6542	0.198777
ENSG0000	LILRB2	19:542738	2.49031	18.9667	0.337435	0.656707	1.11457	31.7473	0.092716	2.60221	3.84432	1.06256	0.24215	0.877928
ENSG0000	ATAT1	6:3062684	3.73195	4.67648	6.41566	6.17174	5.53261	1.25261	4.34103	4.07729	5.62772	7.88447	0.7693	-0.21364
ENSG0000	RFESD	5:9564675	4.45114	6.60242	7.89094	9.67458	3.23801	1.78609	0.535914	5.83452	8.35674	2.86558	0.44415	-0.62075
ENSG0000	DIS3	13:727277	10.3742	10.4129	11.985	13.092	22.2146	7.12579	10.919	17.4085	10.2328	16.8661	0.7281	-0.3384
ENSG0000	GTF3C1	16:274595	65.714	18.0765	77.3519	166.509	65.148	9.12243	13.1228	41.8166	58.3522	39.7285	0.0418	-1.25084
ENSG0000	TNKS2	10:917983	14.874	13.5223	3.67387	5.05381	6.20078	9.19846	9.57525	6.07638	10.3711	9.86262	0.7379	0.118165
ENSG0000	AC000068	22:193306	0.459979	0.740228	0.388114	0.675252	0.277331	0	0.626525	0.773965	0	0.665442	0.81645	-0.31995
ENSG0000	CTD-2358	16:317005	0.797198	0.918342	0.405011	0.54001	0.500283	1.17283	0	1.30995	0	1.39774	0.74845	0.465119
ENSG0000	UBXN1	11:626764	105.475	126.733	165.63	117.281	193.431	144.835	134.166	186.144	132.032	84.594	0.8028	-0.08654
ENSG0000	RP11-104	16:894909	0.538393	1.64924	0.226666	0.336316	0.177711	0.475	0.925279	0.38415	0.579356	0.468026	0.97835	0.039529
ENSG0000	KLF8	X:5623242	0.750182	2.79163	0.829191	0	0.334191	0.287207	0.33285	0.677113	0	1.53288	0.4134	-0.97454
ENSG0000	PRR29	17:639960	7.33104	5.18976	1.8461	5.49103	3.65195	0.081531	0.421978	0.32885	1.89761	1.66398	0.024	-2.45661
ENSG0000	FIS1	7:1012394	119.142	88.4324	167.629	171.465	145.42	122.56	135.403	137.746	154.884	80.6596	0.5779	-0.19492
ENSG0000	CDKN1C	11:288321	0.305699	0.478881	1.42022	0.781158	0.93499	0	0.790535	1.2962	0.052519	0.876574	0.59915	-0.55938
ENSG0000	FXYD5	19:351547	208.873	331.333	342.304	458.078	508.859	279.949	275.798	408.412	628.437	207.181	0.9903	-0.00435
ENSG0000	PC	11:668482	1.65319	3.45801	0.778583	1.26375	1.03493	2.24074	1.63938	0.762307	0	0.965118	0.952	0.041688
ENSG0000	PAQR6	1:1562129	1.25257	0	0.195394	0.282368	0.398968	4.01669	0	0.6139	0	0.442245	0.44045	1.23977
ENSG0000	TIAF1	17:290711	1.01257	0.641587	0.126127	0.195367	0.355349	0.187923	0.837802	0.061568	0.110292	0.614756	0.6647	-0.6827
ENSG0000	RNPS1	16:225311	157.861	193.376	259.569	255.598	253.964	194.61	212.094	174.811	184.709	168.93	0.3614	-0.33134
ENSG0000	NCBP2	3:1969354	57.8537	80.7038	66.9656	71.6267	57.8912	46.5193	43.6682	77.6769	57.8064	58.4448	0.42545	-0.25648
ENSG0000	MAP1LC3I	16:870835	124.46	120.341	150.86	220.157	100.098	154.099	131.701	120.297	200.812	71.3302	0.8484	-0.06745
ENSG0000	SERBP1	1:6740780	108.415	90.5357	87.6832	86.7171	153.858	140.159	161.144	94.2772	85.166	121.858	0.9128	0.042597
ENSG0000	GEMIN2	14:391142	12.4208	5.82332	13.0889	11.7937	5.53563	24.6954	11.1804	7.50561	5.16575	3.10451	0.9927	0.00465
ENSG0000	MIS18BP1	14:452031	38.5409	37.4447	19.8856	22.1303	31.0007	48.5562	44.3931	22.4127	29.6931	57.8471	0.51445	0.291562
ENSG0000	GMIP	19:196294	48.58	41.0977	7.33875	6.98355	11.2097	43.8306	21.8826	12.5156	7.72688	22.8121	0.71275	-0.13815
ENSG0000	PACSIN2	22:427965	10.1404	16.5939	4.84369	4.43996	4.84188	13.1395	7.66228	4.01908	1.02214	3.66406	0.50835	-0.7264
ENSG0000	AKIP1	11:891113	13.355	7.40333	18.3573	13.223	11.3697	30.4243	15.6156	9.86785	10.81	8.3112	0.78835	0.131104
ENSG0000	ERGIC2	12:291491	44.2941	31.8445	58.3607	31.3259	36.2984	71.6812	58.2897	52.3767	46.7369	47.9846	0.69225	0.216474
ENSG0000	MCEE	2:7110968	22.8044	26.4078	35.2368	32.4984	9.40734	13.3288	22.4743	33.8302	40.6181	13.7621	0.9716	0.018373
ENSG0000	CTDP1	18:796798	17.9023	21.6107	6.70419	15.4601	12.04	6.42656	14.7451	7.31486	7.21699	16.916	0.2225	-0.50488
ENSG0000	DMWD	19:457647	12.1094	15.4767	1.93816	1.63994	7.2962	4.95218	13.8616	6.03514	0	7.65164	0.69655	-0.32557
ENSG0000	LPAR6	13:483037	32.4558	62.1482	10.8522	13.8765	6.16943	12.7124	18.6196	17.3669	12.7457	6.64494	0.21395	-0.68921
ENSG0000	SLC25A10	17:817033	5.76507	2.97205	2.21982	1.95783	3.98659	2.52905	5.00805	3.26316	0	4.79609	0.86255	-0.22891
ENSG0000	ABHD14B	3:5195531	40.5716	28.0376	46.8437	51.7873	50.3629	23.4936	21.7856	31.2738	21.2831	38.0772	0.20545	-0.71802
ENSG0000	UNC45A	15:909299	33.6075	32.841	20.8297	25.2932	19.1404	25.3227	32.5616	17.9695	8.24426	27.2155	0.55895	-0.2518
ENSG0000	SOD2	6:1596690	36.551	39.2627	30.9914	38.6658	33.9462	247.774	24.9133	74.9629	90.8442	36.3488	0.2482	1.30782
ENSG0000	TUBGCP6	22:502176	29.7828	34.0605	7.04051	8.44043	8.1752	14.9117	17.5274	8.48152	5.56986	14.091	0.3487	-0.38796
ENSG0000	CNPY2	12:562716	100.402	75.6822	190.857	148.834	126.818	140.672	113.018	133.288	130.82	65.8914	0.74285	-0.17835
ENSG0000	ID1	20:316052	5.80948	5.15393	5.70354	8.78666	0.56604	0	0	3.0807	3.9612	0.405619	0.30875	-1.60073

ENSG0000	TBC1D14	4:6909241	22.7033	22.9865	12.0041	12.8326	13.1384	10.2444	12.1393	10.3413	9.15568	18.5091	0.23675	-0.51143
ENSG0000	EVA1B	1:3630638	13.147	4.16663	3.45852	3.06424	4.59073	20.8891	10.0551	4.13332	7.62151	3.72605	0.6443	0.560775
ENSG0000	MOCS3	20:509588	1.7252	3.6464	2.10211	1.9708	3.16569	0.856326	1.19759	2.89174	1.15525	2.21021	0.2109	-0.68296
ENSG0000	ZNF613	19:519271	5.5956	10.7414	5.47118	4.76165	2.74991	4.84208	5.13627	3.77075	7.99907	4.19076	0.65115	-0.23593
ENSG0000	MIR24-2	19:138345	2.42401	1.48267	1.40629	47.3204	1.80261	7.73531	20.613	17.7784	25.0302	18.6279	0.30285	0.677876
ENSG0000	USP9X	X:4108563	7.76784	10.8745	2.0106	1.77866	3.31566	5.55145	5.18609	4.38623	1.34876	8.85891	0.80645	-0.09669
ENSG0000	GTF2B	1:8885293	42.8584	45.1199	40.6713	32.1724	23.8201	35.0318	46.5881	46.4003	26.3593	13.1802	0.8555	-0.06315
ENSG0000	NCAPH	2:9633578	2.46233	3.90515	0.823128	1.85674	0.975561	5.31586	8.44636	7.19123	7.22303	7.34908	0.0161	1.73987
ENSG0000	VASP	19:455065	41.9641	35.3545	24.94	7.7973	47.2209	35.5276	30.4666	35.3568	19.5991	42.0997	0.7413	0.125809
ENSG0000	KB-1125A	22:238564	0	0	0.828944	0.520385	0.22823	0	0.223118	0	0	0	0.3285	-3.11976
ENSG0000	HMGN1	21:393423	352.073	342.746	335.214	264.023	272.66	546.69	443.743	328.248	357.287	149.215	0.52365	0.228908
ENSG0000	BPHL	6:3118373	3.64249	5.73068	7.33989	9.88086	5.06698	5.15376	7.48236	8.49089	0	2.61689	0.615	-0.35209
ENSG0000	MAP3K12	12:534416	4.37714	9.67195	1.85413	2.75605	1.46416	2.25224	6.02924	1.7766	4.51244	3.3185	0.8544	-0.15423
ENSG0000	AC116366	5:1324106	1.27081	0	0.892724	2.65763	0.431803	2.04889	0.177114	1.32001	1.82166	0.608615	0.8125	0.28593
ENSG0000	RP11-887	12:936408	9.44509	7.81646	8.43479	10.4189	4.44847	11.0585	14.0202	8.52729	7.11039	3.43439	0.92255	0.144616
ENSG0000	KIT	4:5465791	0.223043	0	0.202007	0.092108	0.063531	0.919948	0	0.614677	0.979056	1.07121	0.0446	2.51338
ENSG0000	STAG2	X:1239592	30.7796	30.6756	10.3221	11.1491	31.5486	18.7759	29.0531	10.7328	13.0842	27.2661	0.56415	-0.37057
ENSG0000	CEP162	6:8412424	7.64696	5.75096	2.7075	2.51218	4.81691	9.22261	5.61858	4.68361	2.29927	10.9736	0.64505	0.285046
ENSG0000	TCF20	22:421600	1.89987	2.30341	0.375204	0.227353	0.586342	1.47856	2.4505	0.792927	0.394408	1.77956	0.7252	0.226665
ENSG0000	NFIL3	9:9140904	11.2109	7.29024	5.69881	7.82274	6.17527	38.2378	21.8401	13.7454	6.13654	6.62707	0.0124	1.17193
ENSG0000	FAM127A	X:1350323	23.3239	27.7724	48.1584	49.5331	50.0944	38.7023	42.1706	56.328	38.6973	50.3536	0.7826	0.100486
ENSG0000	WDR54	2:7442167	34.682	27.8886	68.87	66.3504	48.8998	25.4506	23.8846	42.3451	45.5497	34.0702	0.1328	-0.53577
ENSG0000	UBE2FP1	3:3705265	0.140625	0.995905	0.295942	0.552399	0.104465	0	0	0.664122	0	0.239449	0.69335	-0.79847
ENSG0000	CDK3	17:759415	2.12755	4.01588	0.734849	1.31482	0.020693	0.720948	0.93658	0.001412	3.31186	2.91241	0.9193	0.167241
ENSG0000	FUNDC2	X:1548357	80.1148	51.3728	111.804	102.338	100.87	94.9092	79.8358	91.4258	69.2578	27.3234	0.46725	-0.41257
ENSG0000	MICU3	8:1702723	0.406051	0.51987	0.087439	1.27485	0.125173	0.745014	0.094315	0.072297	0.231202	0.163848	0.0548	-1.93448
ENSG0000	NEK3	13:520336	1.26459	3.30257	0.388372	0	0.981297	0.090765	1.40164	1.02944	0	0.427772	0.6665	-0.35953
ENSG0000	ZBTB26	9:1229155	0.567902	0	0.96087	0.359722	0.564501	0.405643	0.544216	0.765854	4.4188	1.77935	0.3044	1.61276
ENSG0000	PPM1A	14:602401	11.3908	7.96092	8.21309	6.32073	8.84645	4.31295	10.2519	14.1374	4.08345	20.674	0.80915	0.155616
ENSG0000	SNHG20	17:770867	5.97914	8.88172	11.4685	20.3823	6.31042	0	6.63769	17.4578	8.08823	8.02569	0.61825	-0.42003
ENSG0000	MED7	5:1570858	16.9707	16.5369	25.5541	19.2679	14.7677	25.7949	33.4233	25.3192	31.2879	14.5956	0.73845	0.408484
ENSG0000	MSL3	X:1175815	51.3979	95.7341	52.5685	43.2216	57.7715	62.6095	55.2131	46.5886	54.5704	50.8816	0.67645	-0.13116
ENSG0000	GDF15	19:183747	0	0	0	0.123677	0	11.9166	0	0.451861	0	0.090591	0.28045	6.3194
ENSG0000	RP11-320	11:862839	0.908319	1.09976	1.11447	1.12793	0.570966	0.890488	0.60109	1.85709	0.112547	0.893476	0.90885	-0.11051
ENSG0000	KLHL34	X:2137441	0.277136	0.372793	0.626927	0	0.215558	2.01227	0.229731	1.36539	0	0.140755	0.65225	1.25621
ENSG0000	AP000648	11:901936	0.486851	1.36134	0.240476	0.194558	0.460515	0.524636	1.51479	1.62276	1.47232	1.35795	0.05805	1.2826
ENSG0000	CNTROB	17:792185	7.58368	12.841	1.60084	2.28498	2.70614	9.54237	12.4994	2.54277	2.79276	4.83573	0.76375	0.225065
ENSG0000	RP1-267D	12:120095	2.14566	3.84179	3.05561	3.7047	1.73131	0.707153	2.91114	2.35532	6.75722	2.83662	0.92715	0.11495
ENSG0000	RNFT2	12:116738	1.12526	2.76393	0.948811	0.965062	0.566938	0.567627	0.353393	0.985747	0	0.839927	0.1696	-1.16758
ENSG0000	ARL6IP5	3:6908494	328.694	317.211	262.897	287.961	302.584	383.836	386.159	292.563	314.807	162.564	0.927	-0.0306
ENSG0000	MRPL13	8:1203807	59.5919	42.0775	67.0018	55.3727	34.4525	90.954	83.0782	62.5917	29.6592	7.89478	0.83855	0.099328
ENSG0000	RNF25	2:2186638	17.8848	14.1417	24.0655	22.6095	19.9444	18.3739	19.7131	21.0487	11.6625	16.4116	0.6778	-0.19387
ENSG0000	ZSCAN29	15:433581	2.95415	4.42394	1.20305	1.50397	2.15206	3.47358	4.15048	5.65418	2.45964	3.54004	0.4412	0.506579
ENSG0000	AC008592	5:9583442	0.576061	0.251482	0.57489	0.370509	0.429103	1.03465	0	0.166043	0	0	0.49585	-1.17147
ENSG0000	TERF1	8:7300886	36.6747	35.8147	31.5723	19.0015	46.7756	49.3428	49.9196	22.952	19.3122	39.7637	0.6434	-0.18869
ENSG0000	PIK3CD	1:9651731	65.0808	97.2103	36.3771	30.2047	48.3878	44.4302	59.5711	43.1294	47.7902	69.0678	0.90445	-0.04323
ENSG0000	APBA3	19:375081	47.5633	47.4626	38.7867	37.9465	48.8284	45.7821	49.9196	43.7351	46.5293	57.3406	0.65995	0.136191
ENSG0000	GIPC1	19:144777	53.218	23.5501	14.5596	19.0121	23.2648	44.5271	33.9935	20.6034	5.72314	14.5819	0.5028	-0.30633
ENSG0000	ELMSAN1	14:737151	3.71544	4.02698	9.8823	5.51132	5.72231	3.55954	4.39418	10.9372	7.44516	16.0008	0.47215	0.298881
ENSG0000	ARPP19	15:525470	31.7234	28.6836	21.8311	19.4686	13.5388	44.0093	68.3543	32.564	46.4191	18.8308	0.1646	0.72289
ENSG0000	SNRNP70	19:490854	131.88	156.343	73.0825	74.9327	102.733	102.228	173.215	81.87	60.2995	139.868	0.98435	-0.00809
ENSG0000	LMBRD1	6:6967580	34.5538	26.6378	25.4011	20.9495	14.4758	30.3414	46.515	33.3234	25.8109	28.5304	0.17415	0.469216
ENSG0000	ZNF513	2:2737049	12.7926	14.9832	2.39279	1.08137	3.20501	15.1845	18.7414	3.86436	2.91884	7.47129	0.7424	0.410846
ENSG0000	CRYZL1	21:335039	30.4785	26.5588	42.1273	34.6625	21.8899	25.7959	30.6973	33.306	43.1704	27.5203	0.96655	0.023563
ENSG0000	CCS	11:665901	48.7115	72.6164	55.4696	56.1187	62.0859	78.8321	55.9398	47.7687	59.4441	58.855	0.9417	0.023943
ENSG0000	RP11-666	16:285537	9.74916	4.22058	9.69281	10.0159	5.61739	11.3924	6.01724	3.91735	8.05934	1.41699	0.739	-0.48638
ENSG0000	RP1-43E1	1:1921050	3.2022	1.046	0.510169	0.919392	1.09329	4.04406	3.27636	1.1386	0	0.112206	0.8929	0.222075
ENSG0000	C10orf95	10:102449	0.875844	1.19076	1.69888	0.595104	0.549736	0	0.284699	0.670488	0.110337	0.408308	0.33565	-1.4645
ENSG0000	CTD-2349	17:307092	1.80567	3.11643	1.62348	0.726533	0	0	0.850405	0.054588	0	1.42337	0.5625	-1.04039
ENSG0000	PRKAR2A-	3:4874459	0.420376	0.539492	0.314683	0.581532	0.117642	0.246659	1.31176	0.89913	0.211008	0.116078	0.8174	0.261798
ENSG0000	RP11-467	7:3994752	2.52573	0.278039	0.74869	0.102779	0.154676	0	0	0.105506	0.962143	0.23357	0.49885	-1.36496
ENSG0000	PRKRA	2:1784139	41.2879	50.646	42.5554	43.5411	32.1108	39.5788	44.6311	41.1683	49.2951	19.0581	0.75645	-0.10719
ENSG0000	PPP3CC	8:2244081	67.3538	69.5309	41.3758	68.8378	38.9206	36.3383	36.261	41.2929	37.4691	40.1103	0.08615	-0.64452
ENSG0000	RBSN	3:1507007	8.66219	10.7137	7.05544	6.93311	4.93949	6.68387	3.54626	4.72376	14.7208	9.20353	0.94825	-0.04596
ENSG0000	UBOX5	20:310691	3.48753	3.88843	4.30063	2.30633	3.62744	3.6864	2.10793	1.47726	3.88789	4.33292	0.78255	-0.23674
ENSG0000	PEX2	8:7698025	53.0934	36.217	45.4398	46.1504	30.3328	68.9145	48.1623	46.1816	35.0057	18.2709	0.93165	-0.03099
ENSG0000	MADD	11:472691	14.0676	16.113	11.7587	15.1294	16.5965	8.14267	15.4453	10.0711	15.2791	27.054	0.9143	-0.05302
ENSG0000	SLC7A5P1	16:296131	8.11289	6.36789	4.3543	5.12174	1.71744	12.0573	11.3543	11.5171	8.46958	7.74749	0.198	1.00646
ENSG0000	C5orf34	5:4348395	1.98089	3.44418	1.33634	1.48286	0.946622	0	2.36277	2.65995	0	0.427412	0.47985	-0.6459
ENSG0000	TRBV11-2	7:1424338	10.4186	37.1205	4.46772	3.18985	9.23665	23.6708	157.554	157.95	19.0459	2.51089	0.0018	2.61138
ENSG0000	MRPL30	2:9899726	14.6308	16.8706	8.18072	9.55263	9.83962	14.5869	13.1294	6.87296	1.52365	7.61872	0.7286	-0.31144
ENSG0000	CLEC12A	12:994813	7.83893	24.6704	1.57304	2.40981	5.19222	14.9149	5.75022	4.91167	0.929839	6.06434	0.7219	-0.26514
ENSG0000	MRPS15	1:3645571	65.5902	38.7676	77.1441	85.3275	79.8395	72.9637	55.6248	68.1673	61.9899	18.9431	0.3545	-0.34259
ENSG0000	CTC-543D	19:953873	0.829487	0.611771	1.54842	0.508325	0.214664	0	0	0	0	0.457094	0.317	-2.93721
ENSG0000	B3GNT7	2:2313955	0.015313	0.302239	0.667685	0.412145	0.264878	0.015418	0.783796	0.686091	0	1.35028	0.71615	0.645914
ENSG0000	EPS15	1:5135426	20.5354	17.6022	6.93005	5.76904	6.34023	16.1408	21.6297	15.0312	15.6008	16.5238	0.04995	0.686603
ENSG0000	HIST1H3A	6:2602048	11.0221	2.06518	9.75475	18.0721	3.34987	0	1.0509	1.24537	3.56441	0	0.051	-2.78381
ENSG0000	RP11-874	12:281332	0	0	0	0.899525	0	0	0	0	3.40115	0	0.6179	2.12064
ENSG0000	VPS41	7:3872296	15.5523	14.9237	10.0456	7.73012	11.0371	22.3255	18.6602	11.7753	2.88572	18.529	0.6285	-0.20094
ENSG0000	ENOPH1	4:8243056	31.0469	22.9339	22.4186	30.9064	20.1198	39.773	27.4289	32.1483	37.6813	16.5721	0.45505	0.265488
ENSG0000	AC129492	17:807263	1.08666	0	0.436885	0.281607	0.705429	4.09328	0	0.270325	0	0.593504	0.62965	0.912543
ENSG0000	SLC35B1	17:497009	35.0023	31.2093	42.8976	45.803	30.7876	45.8357	50.9983	28.9753	33.528	30.2966	0.9231	-0.05895
ENSG0000	CH507-15	21:698644	5.41672	1.17147	4.74641	1.39571	0.752447	2.92486	1.62106	2.8407	0.261385	0.451081	0.34935	-1.27657
ENSG0000	RP11-245	3:6400402	0.612441	1.63441	0.173524	0	0.102838	0	2.3742	0	0	0.629927	0.7902	0.562247
ENSG0000	ZXDB	X:5759165	1.72111	2.19956	1.21342	0.752745	1.07448	0.130272	0.636313	0.752529	0	1.88445	0.12885	-0.9938
ENSG0000	ZNF582-A	19:563936	2.98679	4.51873	2.45676	1.85262	2.60041	4.50624	1.12884	1.54889	0.115522	2.82875	0.52565	-0.53894
ENSG0000	CAPS2	12:752347	0.599027	0.424543	0.357751	1.14444	0.147572	0	0.805819	0	0	0	0.16335	-1.80818
ENSG0000	IQGAP1	15:903882	103.04	76.5994	28.7097	25.2152	30.7557	74.2616	65.1222	41.1241	50.3117	73.4787	0.32	0.389664
ENSG0000	ZNF630	X:4783690	1.92713	1.94471	0.726139	2.48362	1.25701	1.85304	1.81784	1.1855	0.23424	4.1211	0.94915	0.068234
ENSG0000	LINC0112	2:4322720	2.22834	3.17028	0.984633	0.428494	0.099323	0	1.82018	0.507601	0	0.092944	0.36055	-1.45761
ENSG0000	KLRC4	12:103637	1.17609	1.77661	1.06755	5.13228	0.042061	1.41897	27.0328	7.96986	0.908272	1.57451	0.19985	2.05471

ENSG0000C	STAT5A	17:422875	40.6893	52.2794	20.1899	27.4318	19.9406	39.2169	34.2359	32.395	20.9698	36.3983	0.96425	0.013568
ENSG0000C	TESC-AS1	12:117099	0.32497	0.22273	0	0	0.037923	3.71198	0.251098	0	0	0	0.40045	2.72595
ENSG0000C	MIR9-3HC	15:893615	1.73712	0.262865	3.12026	0.914527	1.77908	2.55422	1.777	2.20508	0	1.27153	0.89275	-0.09224
ENSG0000C	NSMCE3	15:291202	24.9325	21.579	22.0783	19.7646	19.7807	11.675	17.8143	15.341	12.3265	12.5471	0.04825	-0.64574
ENSG0000C	RP11-316	10:100150	0.320028	1.32503	0.796514	0.094092	0.547392	0.866563	0.295232	0.331965	0	0.186504	0.5187	-0.93097
ENSG0000C	BRPF1	3:9731728	14.9482	11.7446	6.62491	5.06668	5.23893	12.1508	8.11158	7.24799	4.35861	11.1468	0.888	0.058917
ENSG0000C	NALT1	9:1365461	0.139369	1.71131	0	0	0	2.06565	0.400748	0.067743	0	0.16633	0.7338	0.583658
ENSG0000C	ADCY4	14:243183	0.593562	2.16962	0.645742	0.071639	0.367273	0.893227	1.12516	0	0	0.78186	0.5861	-0.43832
ENSG0000C	ATP2A3	17:392386	52.051	47.5718	12.2643	13.0753	22.9716	32.8741	34.3621	32.9715	14.6084	59.8902	0.2756	0.344974
ENSG0000C	C5orf42	5:3710622	1.69398	2.86794	0.260449	1.2805	2.17207	1.52127	1.42506	0.544355	1.68143	3.03345	0.6257	0.292539
ENSG0000C	ARFGAP1	20:632727	16.6203	15.9705	9.88004	14.2872	14.3542	10.6603	10.4561	14.8917	23.4417	31.5944	0.204	0.534435
ENSG0000C	NSUN4	1:4634017	7.77598	7.30176	10.0112	7.31539	6.18891	5.88791	3.03683	7.8128	6.12845	6.0437	0.4852	-0.37591
ENSG0000C	P2RX1	17:389659	0.49142	3.05031	0.064612	2.16063	0.054279	2.2406	0.32962	0	0	0	0.33215	-1.31221
ENSG0000C	CSK	15:747820	69.2871	87.9853	58.6717	44.0704	92.9162	73.6117	71.3982	61.3731	35.1542	112.274	0.9574	-0.01699
ENSG0000C	FEM1C	5:1155209	2.83085	2.21744	0.719353	0.450394	1.33502	2.82624	3.30946	2.18544	5.15581	2.32614	0.03625	1.16426
ENSG0000C	RP11-85A	10:996210	1.91378	2.06391	1.49186	0.824863	1.06271	0	0	2.60515	2.13905	1.63595	0.9499	0.009381
ENSG0000C	BOD1	5:1736075	9.77272	5.96689	10.275	5.42282	9.74645	7.65763	7.25869	9.47089	10.4145	8.40555	0.9294	0.04769
ENSG0000C	SSPN	12:261200	0	0.416054	0.006609	1.88	0.217065	0	0	0	0	0.030032	0.00495	-6.74884
ENSG0000C	MARCO	2:1189421	0.311391	0.78653	0	1.44614	0.284532	64.7298	2.24456	1.94194	4.87936	3.03377	0.00595	4.49127
ENSG0000C	GATAD2A	19:193858	18.5535	19.8912	9.25853	8.41259	21.5184	11.6419	16.3966	12.9697	7.49897	30.2461	0.71395	-0.18862
ENSG0000C	IGKV2D-2	2:8994751	0	0	0.096398	5.00219	0.484398	0	0	0	0	0	0.01885	#NAME?
ENSG0000C	RRAGA	9:1904939	72.3984	75.9092	90.7117	68.5619	88.1467	64.5978	82.1733	75.2097	62.691	82.9332	0.66075	-0.15003
ENSG0000C	RHPN1	8:1433688	10.5554	10.2314	3.68762	6.69719	3.8346	14.1674	7.27652	7.89533	8.4745	9.9791	0.4087	0.43395
ENSG0000C	SERPINB9	6:2887265	13.1516	23.9528	36.7048	20.8687	22.5943	23.6302	25.8814	35.2896	16.1768	34.465	0.7866	0.087574
ENSG0000C	AC083884	7:7465766	0.068491	0.330085	0.203521	0.423116	0	0.83664	0	0.049495	1.90346	0.045103	0.4143	1.41274
ENSG0000C	METTL7A	12:509234	2.95344	7.21801	0.731511	2.14249	0.663742	0	2.31422	2.30062	3.31246	0.740645	0.4923	-0.75959
ENSG0000C	TCTA	3:4935914	27.6366	22.9244	30.7233	37.7201	21.7865	13.7405	25.7969	24.3018	35.2807	22.4449	0.8964	-0.18675
ENSG0000C	KMT5A	12:123383	13.3065	6.38438	5.74092	6.40441	6.70399	11.3219	10.3172	4.94425	4.54088	10.2988	0.9586	0.025016
ENSG0000C	ZNF30	19:349269	5.07136	4.26308	5.152	6.71261	2.43853	2.91701	2.41133	2.35075	7.11975	2.92002	0.58415	-0.33754
ENSG0000C	IQGAP2	5:7640324	53.8974	56.3577	33.3306	27.3513	35.5122	40.2578	58.4626	22.8528	17.9307	44.5529	0.5312	-0.29728
ENSG0000C	OAS3	12:112906	7.63457	7.39423	2.7288	5.16675	2.89703	5.24329	34.5796	15.1293	10.8827	8.97372	0.0905	1.50096
ENSG0000C	ASAP1-IT	8:1300521	0.391493	1.57156	0.214203	0	0.0197	0.927975	0.318414	0.040005	1.46765	0.259326	0.77795	0.592232
ENSG0000C	TCEA2	20:640498	17.0382	26.3258	27.0493	21.6649	27.5649	13.535	23.7679	11.333	9.56664	10.328	0.0619	-0.89593
ENSG0000C	PCMT1	6:1497243	94.0847	93.5994	109.217	104.527	90.876	110.297	119.047	125.007	133.01	59.9762	0.7152	0.130215
ENSG0000C	AP3S2	15:898305	8.85962	7.09798	9.54199	12.2973	8.56349	11.2946	6.64301	7.0187	10.1343	8.59066	0.9881	0.007256
ENSG0000C	KIAA0391	14:350449	63.5072	30.2061	39.7232	83.1008	48.4886	56.827	19.7312	27.2425	55.9792	30.3618	0.51095	-0.56578
ENSG0000C	RP11-5C2	7:1549284	1.94269	3.40381	0.459872	0.894131	2.83408	1.83797	1.18236	1.51539	0	6.9798	0.92865	0.164715
ENSG0000C	RP11-206	4:1529274	0.491692	0.856733	0.538166	0.386468	0.160508	0.446612	1.02224	0.184842	0.932002	0.097701	0.90075	0.212026
ENSG0000C	AC097713	2:2344383	0.530157	1.75764	2.17809	0	1.05065	0	0.680502	0	0	0	0.2335	-3.23514
ENSG0000C	ITGB7	12:531576	216.23	271.64	162.505	186.312	168.514	133.529	171.275	126.374	129.858	215.129	0.2653	-0.37118
ENSG0000C	ENO2	12:686711	9.5991	12.1324	8.94492	6.41667	9.24687	13.1626	7.72059	15.2622	6.56652	16.3559	0.7826	0.283574
ENSG0000C	ZXDA	X:5790670	2.01803	1.54803	0.851174	0.991132	1.40384	0	0.149939	1.1156	0.179633	1.70578	0.43655	-1.15094
ENSG0000C	RAB11FIP	16:425618	6.24375	6.43799	0.680181	0.264393	0.989401	9.87384	12.4164	1.79022	0.332102	2.73686	0.1727	0.87361
ENSG0000C	MED13	17:619426	2.59132	3.12188	2.59683	2.75813	2.79722	1.87063	5.8795	4.79656	2.97458	8.52378	0.1717	0.601278
ENSG0000C	DHFR	5:8062622	6.72391	10.7972	1.21234	0.856467	0.876223	6.17213	10.1032	5.27158	4.58915	2.31583	0.65445	0.432773
ENSG0000C	GTF3A	13:274245	536.679	302.653	562.778	503.457	475.163	261.786	293.052	271.888	364.554	120.872	0.01935	-0.86697
ENSG0000C	LINC0091	17:433698	1.45912	2.96942	3.09087	2.62259	0.932127	1.89948	0.137178	1.66142	0.019895	1.58949	0.22535	-1.22646
ENSG0000C	IDH3A	15:780778	32.9896	29.1715	27.2384	28.8822	20.1672	41.3422	59.326	22.8753	22.2735	25.7819	0.77005	0.103489
ENSG0000C	SCAP	3:4741369	29.9545	32.1777	9.95062	10.4894	11.3257	32.4056	27.7569	18.5431	7.64253	21.7213	0.4088	0.307284
ENSG0000C	HLA-DRB	6:3255271	72.2864	195.583	123.473	95.7626	20.8444	452.07	233.672	318.043	235.65	30.6417	0.0003	1.34118
ENSG0000C	RWDD3	1:9511733	48.1085	30.3067	43.386	46.5104	14.181	44.3911	37.6636	27.1139	25.9253	7.32527	0.6289	-0.36466
ENSG0000C	ZNF79	9:1274243	5.52778	5.13413	3.55923	5.54942	2.79069	12.9029	5.98234	3.74125	4.80043	5.13709	0.44365	0.467331
ENSG0000C	IPO7	11:938462	2.2488	1.6558	1.5955	1.77105	2.46938	2.96534	2.48479	1.8959	0.616969	4.33648	0.7283	0.161154
ENSG0000C	FGGY	1:5929663	3.67783	3.16293	5.63378	3.10647	5.38447	9.877	9.74132	5.73798	10.9807	7.53975	0.0641	0.999864
ENSG0000C	ZNF35	3:4455519	0.59466	0.829847	1.23221	0.253485	1.63849	0	0.993144	0.912177	2.68295	0.686382	0.89955	0.144475
ENSG0000C	AC005606	16:199765	0.986664	0.99888	0	0.068321	0.167211	0	0.570962	0.203995	0.411167	0.558662	0.8311	0.385666
ENSG0000C	ARID5A	2:9653674	66.9716	107.694	84.3036	77.5165	75.0893	119.023	166.057	131.597	125.992	129.909	0.02555	0.733589
ENSG0000C	SLX1A	16:301929	42.9654	57.5821	65.7592	59.7931	97.8199	76.8033	71.9877	80.8926	38.1203	42.4036	0.9763	-0.02927
ENSG0000C	MYADM	19:538545	23.2075	32.36	30.8359	31.6346	34.7233	24.2327	85.8803	98.8304	37.6098	62.0601	0.0454	0.722287
ENSG0000C	CA11	19:486379	3.59146	2.75859	2.90575	4.05859	3.3197	2.61301	0.272618	0.242561	0	1.65519	0.25035	-1.64427
ENSG0000C	NDUFAF4	6:9688931	46.2398	38.5694	47.7195	49.1568	25.1904	52.6401	43.5253	25.0754	13.6587	8.21909	0.5247	-0.45318
ENSG0000C	OGFOD1	16:564291	25.2672	16.3206	32.6267	14.3793	25.1597	30.4718	11.218	29.4427	22.2602	34.7341	0.9788	-0.01791
ENSG0000C	EVI2A	17:310084	63.4885	34.863	56.9089	40.8678	31.9614	84.1561	56.6115	78.4555	78.139	21.5067	0.526	0.500218
ENSG0000C	BIRC5	17:782141	13.6355	24.5356	0	2.35793	0.981466	15.1279	23.3047	4.11908	7.99125	2.30164	0.4536	0.540313
ENSG0000C	RP11-468	14:241361	1.03435	1.12124	1.09196	1.08514	0.452838	1.50284	0.452737	1.31006	1.66016	2.89713	0.5594	0.733626
ENSG0000C	FOXK1	7:4682308	5.98215	9.1699	4.35631	1.39749	3.3123	3.83388	3.90844	2.3526	2.33576	5.20617	0.5489	-0.52758
ENSG0000C	RP11-3D4	15:333109	0	0.130905	0	0	0.241957	0	0	0.34878	1.46677	0.291797	0.3347	2.52542
ENSG0000C	TRIM26	6:3018445	25.8191	21.8278	9.58718	7.2876	13.5653	24.4512	24.0143	16.3576	5.92689	21.0452	0.67265	0.18639
ENSG0000C	MTIF3	13:274245	99.0422	71.0978	104.398	67.6979	96.5453	113.335	113.204	68.0911	54.1582	31.469	0.7186	-0.23817
ENSG0000C	RPL24	3:1016810	259.921	224.277	402.852	311.645	166.508	303.416	373.821	337.484	254.618	36.776	0.7743	-0.1092
ENSG0000C	RP11-391	16:814451	0.140275	0	0.354885	0.187513	0.105291	1.53674	0.669662	0.226579	0	0.505851	0.40655	1.69264
ENSG0000C	RP11-159	17:579890	4.4084	6.26039	0.788809	0.85172	0.483544	1.52442	6.32182	2.03373	4.40167	3.60035	0.497	0.691573
ENSG0000C	SMAD5	5:1361295	3.15817	1.98757	3.72594	3.53092	5.36498	4.19947	4.21949	2.86327	4.55576	6.46243	0.8586	-0.09959
ENSG0000C	AGAP2	12:577237	4.92945	3.61553	4.78428	4.96243	15.0514	5.69013	3.00652	4.81786	5.37428	17.3126	0.99345	-0.00751
ENSG0000C	ZNF708	19:212911	12.9726	10.4745	2.79116	4.54916	3.72581	8.42994	6.62917	6.30958	4.35919	6.83578	0.64915	-0.21469
ENSG0000C	ISOC2	19:554529	18.4104	14.6316	32.7581	50.2449	32.312	19.4675	31.9608	42.4396	43.5356	13.6459	0.99195	-0.00467
ENSG0000C	DCTN5	16:236413	21.3202	19.1702	15.8112	4.09258	11.4237	31.7088	26.0307	22.0914	10.6396	14.154	0.8132	0.101242
ENSG0000C	PAPLN	14:732374	0.247258	0.646554	0.242992	0.366046	0.023181	2.11051	0.11559	0	0.599152	0.282522	0.3378	1.04254
ENSG0000C	ENTPD3-A	3:4031380	7.26822	4.67996	9.3656	10.8857	2.03522	11.5896	6.95533	8.15144	3.00528	1.82966	0.8596	-0.14741
ENSG0000C	MANBA	4:1026314	9.09467	11.5535	7.32175	9.17749	2.98347	14.8688	6.64887	9.24271	5.12375	4.03959	0.9952	-0.00565
ENSG0000C	RP11-712	11:951505	5.3218	8.89905	7.82744	3.97048	3.26663	5.47672	7.35277	2.68684	0	4.88963	0.56425	-0.51908
ENSG0000C	CTD-2240	19:100861	0.428438	0.704747	0.258349	0.135834	0.325959	0.214217	1.19253	0.432927	0.053924	0.756412	0.7023	0.465773
ENSG0000C	SYTL3	6:1586500	18.966	13.0187	20.8755	49.7291	24.3574	41.0984	51.1731	25.0822	34.6147	36.2881	0.14435	0.521955
ENSG0000C	PARP1	1:2263606	85.3528	91.6747	57.7898	59.9238	100.714	78.7801	68.8835	59.6129	53.3753	121.477	0.74475	-0.15032
ENSG0000C	CTA-293F	7:2032829	0.31529	0.886918	0	0	0	1.3006	2.33404	0.382015	2.7223	0	0.16595	2.70277
ENSG0000C	EIF1B-AS1	3:3980891	1.90882	5.17877	2.01805	1.92072	0.93281	8.75725	2.16852	0.985564	1.6291	1.11971	0.7001	0.356575
ENSG0000C	RP11-326	18:318291	1.03539	1.07113	0.178089	0.820445	0.46272	0.41716	0.833105	0.19159	0.515781	0.93027	0.80795	-0.32239
ENSG0000C	CTSD	11:173240	141.839	150.115	114.023	111.078	148.212	761.506	323.031	255.775	169.007	341.793	0.00075	1.31238
ENSG0000C	ERMARD	6:1697516	16.0863	16.8389	9.91907	9.9296	8.31595	9.60481	12.351	11.0604	18.1503	10.6649	0.8282	0.094699
ENSG0000C	SLC16A1-	1:1129118	5.10693	8.32402	5.17718	4.53293	3.44257	6.30881	11.3556	4.84158	7.24846	8.71143	0.5808	0.444696
ENSG0000C	SPIDR	8:4726057	63.1526	41.84	40.5227	31.0939	36.9765	18.432	19.5026	24.8761	18.2975	37.0812	0.02785	-0.82608

ENSG0000	MUC1	1:1551694	0.378098	0	0.260837	1.05887	0.149837	0.107254	0	0.578027	0	0.359599	0.5318	-0.62259
ENSG0000	C1RL-AS1	12:708020	1.68184	3.82504	1.63777	1.27202	3.80227	6.81401	1.91145	2.44017	0.255422	5.78064	0.45955	0.675784
ENSG0000	KRTCAP3	2:2744236	2.1224	1.98495	1.05434	5.89143	0.619833	0.099604	1.59639	2.37498	10.5858	0.389199	0.69025	0.583447
ENSG0000	PDHB	3:5842762	81.3757	80.7134	95.131	110.409	85.991	95.4064	86.6436	68.2162	102.882	60.4294	0.7195	-0.12196
ENSG0000	CD320	19:830212	53.6695	26.0347	59.8564	126.938	109.32	61.7533	47.793	46.6316	48.3399	37.9893	0.2374	-0.73162
ENSG0000	CR1	1:2074961	1.54852	1.25711	0.163099	1.12947	0.498854	1.23719	0.227905	0.242948	0	0.383292	0.43985	-0.84568
ENSG0000	TRBC1	7:1426707	602.033	937.898	872.467	614.382	494.515	1033.32	1034.14	1315.69	798.557	330.641	0.8232	0.379966
ENSG0000	CEP131	17:811895	5.11672	8.98089	1.75338	1.99546	3.46489	19.3389	6.07849	5.5532	0	4.31285	0.3063	0.859066
ENSG0000	METRNL	17:830796	0.56103	2.62184	3.16424	5.90291	3.13083	13.0435	42.5148	13.1079	13.731	9.59473	0.01625	2.3687
ENSG0000	HNRNPR	1:2330377	160.082	156.22	131.118	147.529	126.897	152.309	136.921	108.545	72.9477	125.184	0.4869	-0.24919
ENSG0000	MPC1	6:1663649	62.6438	56.1213	98.8953	76.6657	41.8657	82.7599	116.562	116.068	172.384	37.6994	0.0596	0.654444
ENSG0000	CTD-2213	14:312486	0.267721	0.147393	0.456384	0.321239	0.147133	0	0.287326	0.583749	0.165586	1.00133	0.73915	0.439074
ENSG0000	ATG16L2	11:728143	66.4201	48.5245	36.5479	54.1393	39.2577	52.996	29.1554	29.138	34.9029	51.1173	0.5165	-0.29834
ENSG0000	AC005540	2:1908807	3.32269	0.94989	1.41791	1.28023	3.68636	1.41862	0.123837	1.07257	1.25398	2.3694	0.30405	-1.30135
ENSG0000	LSM3	3:1412493	22.9121	16.7042	26.2686	19.6742	11.5165	32.0219	23.9811	21.6662	15.1057	3.70296	0.9527	-0.05195
ENSG0000	SFMBT2	10:715862	3.7289	2.61492	0.860373	0.975045	1.92648	8.56227	12.4499	5.39976	4.67209	8.60446	0.05765	1.78845
ENSG0000	SLC25A5	X:1194660	183.001	198.456	196.326	234.185	130.232	258.02	207.215	213.56	276.929	79.9685	0.67795	0.141045
ENSG0000	PPP2R2B	5:1465632	17.9636	10.2746	34.7269	50.2882	54.7508	12.4442	27.4824	29.928	19.0049	30.8519	0.2354	-0.52923
ENSG0000	DNAJC13	3:1324175	5.99512	4.7366	1.37854	1.15473	2.16147	2.12365	3.49508	3.12006	2.13784	5.07992	0.77515	0.13616
ENSG0000	POC1B-G/	12:894197	3.46064	0.947292	1.96034	1.72338	1.47432	0.472609	1.0049	2.94886	4.34191	1.65989	0.9686	0.049939
ENSG0000	IMPAD1	8:5695793	8.44464	5.84591	4.47196	2.67362	4.97527	10.0198	6.48665	5.36386	2.13841	6.85771	0.6539	0.193144
ENSG0000	GNG10	9:1116313	38.2141	22.4943	29.7137	34.107	17.3356	48.3149	32.4218	25.1832	14.7074	6.30219	0.82565	-0.1079
ENSG0000	COMMD1	5:1160838	57.2153	37.7193	38.4329	46.292	30.2522	59.7268	41.0224	26.2404	15.8488	14.7055	0.3374	-0.37207
ENSG0000	PDE4A	19:103911	1.22421	0.575674	2.25788	2.36458	1.72667	1.84888	6.60365	7.76298	4.50563	8.51168	0.13275	1.65117
ENSG0000	APMAP	20:249629	210.294	77.3118	326.153	273.616	259.801	99.9268	94.6011	137.377	105.809	147.992	0.00635	-1.02666
ENSG0000	ERI2	16:206102	2.13843	4.17193	0.73441	0.511742	0.738052	6.79631	3.78971	1.21429	0	0.775147	0.5165	0.687137
ENSG0000	5-Mar	10:922911	13.8413	17.6555	12.9033	8.56438	8.70431	21.1193	15.6839	16.3283	9.0989	9.32536	0.7568	0.127308
ENSG0000	ATG9A	2:2192097	21.6622	29.935	8.06517	11.4591	11.6882	19.4651	15.4613	18.1471	6.56893	19.391	0.71275	-0.13875
ENSG0000	NUDT6	4:1228267	2.1738	2.69004	3.32157	5.36235	2.9563	2.85969	2.57452	3.45293	2.76749	3.03963	0.83895	-0.21799
ENSG0000	MIR940	16:225311	4.12082	2.37679	1.73246	5.71826	1.86475	2.08307	1.04095	2.84037	0.374564	3.36775	0.61355	-0.61162
ENSG0000	DLEU1_1	13:499825	0	0	1.86804	0	2.32424	0	0	0.474088	2.0878	0	0.2194	#NAME?
ENSG0000	RP5-908M	20:623863	3.42514	4.79621	4.42242	1.72316	0.70776	8.37597	1.20824	2.43447	5.51895	1.79688	0.59595	0.416763
ENSG0000	POLR2D	2:1278435	13.3212	12.3308	21.0878	18.8677	13.9662	10.9171	14.9512	12.9649	9.80876	13.172	0.17835	-0.60146
ENSG0000	REEP4	8:2213801	24.5758	27.4638	28.9411	33.515	26.4782	32.6268	23.1263	30.524	34.4108	32.2739	0.7748	0.104764
ENSG0000	RPSAP18	1:1602157	0.531196	1.68141	0.629612	0.424074	0.236369	1.73007	0.03446	0.155472	0	0.961139	0.85625	-0.28516
ENSG0000	RP11-110	11:119065	0.149537	0.480389	0.857559	0.720292	0	0	0	0	0	0	0.0137	#NAME?
ENSG0000	EMP1	12:131967	0.213327	0.028198	0.004934	0.757235	0.27835	1.30341	1.26453	0.375153	3.00838	0	0.08315	2.31195
ENSG0000	TMOD3	15:518296	17.4844	16.1151	5.64286	7.58697	10.7395	12.3272	13.673	9.13982	18.9747	11.7105	0.66145	0.223165
ENSG0000	F7	13:113105	0.68218	0	0.168529	1.33964	0.126795	0	0	0.065354	0.016735	0.332305	0.05745	-2.98212
ENSG0000	RP13-516	17:822900	0.103286	0.792369	0.426672	0.703682	0.060579	0	0.078652	0.163244	0.315242	0.071709	0.38625	-1.58978
ENSG0000	ZBTB43	9:1268050	6.02333	2.8643	9.56992	7.73575	8.04706	5.44336	3.88599	11.6488	6.21807	13.8239	0.742	-0.16043
ENSG0000	DPM1	20:508889	57.7877	53.5831	64.8827	55.6718	41.1581	88.6321	78.4477	72.4485	75.2692	21.3577	0.5547	0.304822
ENSG0000	MRPS5	2:9508720	63.1012	57.0483	64.2118	61.9566	69.6155	67.3336	45.8416	55.7048	27.9098	64.3369	0.34455	-0.3369
ENSG0000	SMG1P1	16:223459	13.3447	25.2835	2.9581	6.36468	1.74793	11.4561	17.3109	7.37972	20.588	7.80844	0.45435	0.644141
ENSG0000	FOXN3-A!	14:891248	2.47883	0.601719	0.739957	0	1.02213	7.83505	0.814938	1.25957	0	0.303751	0.61135	0.97993
ENSG0000	NSD1	5:1771330	28.2956	33.7013	5.67952	10.3527	21.0897	24.6744	25.1975	9.65809	24.9904	34.4246	0.69575	0.210941
ENSG0000	TMEM116	12:111931	10.9267	14.2186	10.8552	8.17198	5.89071	4.84722	9.51329	13.154	26.9536	7.99746	0.419	0.390268
ENSG0000	RP11-219	15:604791	0.471773	0.305753	0	0.786366	0	0	0.34696	0.895228	2.01641	0	0.45885	1.39675
ENSG0000	RXRA	9:1343170	0.880265	3.39512	0.745017	0.261462	1.88336	0.374331	3.50745	3.42888	1.36675	4.8216	0.3195	0.710717
ENSG0000	CD58	1:1165145	60.4069	33.5044	41.2398	51.1796	15.4282	137.859	122.321	86.099	121.887	23.8773	0.00005	1.37112
ENSG0000	HSD3B7	16:309572	0.062708	0.182191	0.201627	0.275773	0.110556	11.3502	1.01879	1.08375	0	0.330436	0.10785	3.90795
ENSG0000	FADS1	11:616804	1.11508	1.49786	5.48268	2.85368	2.66824	0.020169	4.35735	10.4435	1.96916	3.55826	0.7691	0.278533
ENSG0000	IFT74	9:2690408	8.97863	12.0399	4.74447	3.35443	8.27954	13.3473	7.57913	2.2755	2.50993	9.38221	0.8712	-0.15668
ENSG0000	KCTD11	17:735188	0.686044	1.47637	1.06691	0.444333	1.50832	3.95324	1.45382	2.7396	0	2.10338	0.30085	0.829985
ENSG0000	H6PD	1:9234774	2.30571	2.34521	1.32696	1.32989	2.09151	4.60384	4.59715	1.21971	2.05093	6.12757	0.08685	0.834794
ENSG0000	QPRT	16:296629	0.916873	0.172499	0.596421	0.14615	1.03311	1.49591	1.60655	0.266827	0.942983	0.336752	0.67615	0.480902
ENSG0000	ARHGAP1	10:969980	0	0	1.71354	2.07523	5.71182	0	0.085606	0	0	5.18519	0.53415	-1.06629
ENSG0000	RPL23AP7	2:1136105	5.17717	2.4633	11.3298	7.40951	7.54971	2.30385	3.10709	16.8342	4.83282	1.59991	0.86695	-0.20779
ENSG0000	RP11-298	14:229296	2.78625	1.80666	0.298245	2.04068	0.604243	8.04119	1.41466	1.74136	0.132584	2.31218	0.39995	1.84019
ENSG0000	PHF21A	11:459293	17.7321	25.9441	9.34647	9.01568	17.0219	12.117	9.51027	12.2775	7.17864	36.9104	0.6388	-0.17272
ENSG0000	MYLPF	16:303709	1.27572	0.690308	1.71531	2.84661	0	2.93963	1.13278	0	0	0	0.60615	-0.87925
ENSG0000	ARL4D	17:433989	3.70907	4.89358	8.7153	4.52515	6.22968	2.64335	1.38271	1.63246	6.02723	6.28661	0.3815	-0.63409
ENSG0000	BORCS6	17:818833	20.9029	18.5877	21.8167	12.4662	21.979	13.3707	15.9988	16.2948	12.5486	14.3734	0.2848	-0.41015
ENSG0000	RP1-198K	20:213027	3.9516	4.20826	4.09996	7.44176	13.4659	2.7552	8.66943	2.61148	4.28527	1.26654	0.52195	-0.95946
ENSG0000	TNFAIP6	2:1513575	1.06819	0.073275	0.646957	0.029458	0.59095	21.4377	0.073587	2.28566	6.71861	2.75851	0.0615	3.71936
ENSG0000	POLR1E	9:3748593	10.6795	7.03898	18.2449	14.6661	21.5281	12.5654	8.68104	17.4582	22.8556	20.8986	0.73745	0.147527
ENSG0000	NUMBL	19:406659	1.50101	4.24152	0.230742	0.463506	0.543416	2.2818	1.84441	0.853339	0	1.66111	0.80215	0.22369
ENSG0000	GPX3	5:1510204	0.248962	1.54268	0	0	0.051509	5.54807	0.037937	0.108277	0	0.076209	0.213	1.91398
ENSG0000	JTB	1:1539590	282.433	193.318	408.719	397.585	425.766	317.827	338.228	298.729	314.891	136.597	0.395	-0.33664
ENSG0000	RP11-700	17:509349	0.321858	1.85188	0.631753	0.605561	0.040296	0	0.480259	0	0	0.332253	0.5189	-2.16944
ENSG0000	ZNF121	19:956035	2.45855	2.43822	3.70566	2.57876	2.08157	2.19111	3.18027	4.67193	1.99568	5.7667	0.61195	0.230584
ENSG0000	TRIP12	2:2297638	17.27	14.3508	4.88824	3.40218	8.45135	14.4741	12.7518	6.91983	4.52428	17.1202	0.97095	-0.01353
ENSG0000	SLC19A2	1:1694639	1.48796	1.76386	0.731515	1.34403	0.953214	1.03857	0.408088	2.9222	0.201711	1.30303	0.8678	-0.14919
ENSG0000	AC138517	5:1394707	0.600851	0.573689	0.247422	0.087492	0.202218	1.73491	0	1.10482	1.51963	0	0.34355	1.51713
ENSG0000	CTD-2369	19:102519	0	0.260606	0.160904	0.206094	0.415001	0.325054	0	0.622353	0.052833	0.412148	0.64475	0.686439
ENSG0000	RASD1	17:174944	0.27904	0.400462	1.12978	0.922115	0.076961	2.14944	11.6507	0.503726	4.17116	1.83024	0.11355	2.66464
ENSG0000	ADPRHL2	1:3608887	29.5284	25.2839	52.689	28.4125	47.3029	30.7339	44.8559	37.8331	20.4908	37.131	0.6246	-0.17808
ENSG0000	UBE2Q2P2	15:823551	3.04564	1.40545	2.49327	1.30947	4.11188	0	0.18109	1.81823	13.4501	7.5243	0.4005	1.09229
ENSG0000	WBP4	13:409319	6.75376	8.64597	6.56093	9.93781	13.7538	12.6575	18.3422	8.034	10.0616	13.9786	0.74595	0.436598
ENSG0000	CENPS	1:1042988	9.5306	4.61855	5.81586	10.7779	5.93614	25.1474	18.8184	8.30902	1.81984	3.56538	0.50715	0.535604
ENSG0000	GLB1	3:3299660	24.8114	29.7385	25.5071	41.6181	21.6359	31.9948	16.2188	25.44	34.5272	30.5755	0.73655	-0.11442
ENSG0000	RP1-257A	6:1271680	0.703285	1.76942	0.599388	1.71313	0	0.54903	0	2.19793	3.0898	0.19872	0.5378	1.00079
ENSG0000	ABHD17B	9:7186245	9.31827	17.9064	8.73282	11.0987	4.72229	10.5353	8.39438	9.05342	10.4941	13.3461	0.88245	-0.06413
ENSG0000	Telomeras	3:1697645	1.44709	0.499018	0.653284	0	1.1369	0	0	0.901566	0	0.206613	0.4928	-1.4483
ENSG0000	YJEFN3	19:195157	1.25144	3.88003	0.075088	0.074124	0.085555	7.27914	3.33434	0.234354	0	0.273084	0.39555	1.43363
ENSG0000	ATPAF2	17:179728	11.1158	10.3985	22.0538	18.6952	17.4231	18.3538	10.8696	18.8809	9.9909	12.5156	0.46545	-0.30627
ENSG0000	POLR3E	16:222973	17.8867	21.4238	29.6833	29.6217	26.0527	15.6516	18.6452	25.144	22.1049	41.9385	0.52095	-0.26608
ENSG0000	ENTPD5	14:738518	5.05085	4.02487	4.58766	3.81022	2.45488	1.81364	3.59258	1.8738	2.36464	2.52661	0.1936	-0.8872
ENSG0000	RP3-430N	22:309706	0.710423	2.90195	0.719406	0.756222	0.029346	0.680866	1.21971	1.91605	0.273536	1.45686	0.57565	0.861548
ENSG0000	RPL10AP6	3:6156156	0.28785	0.488268	1.0878	1.40745	0.272849	0.25768	0.429569	0.412536	0.605877	0	0.54955	-1.05855
ENSG0000	CAPZA2	7:1168110	66.3802	48.2136	53.4654	36.8993	26.965	88.743	94.7132	49.2806	64.3466	20.0078	0.4957	0.296392

ENSG0000(	NR2F6	19:172318	1.0121	1.11157	4.23613	2.32236	2.61871	6.05389	1.04166	2.04202	0.045193	0.543954	0.80115	-0.35942
ENSG0000(	YTHDF3-A	8:6316772	5.96806	0.749754	2.45703	1.51825	1.61473	2.67224	3.49015	0.689046	2.6141	0.290836	0.84095	-0.40442
ENSG0000(	VCPIP1	8:6662848	5.83846	3.66402	2.84824	1.47137	3.12353	3.45552	1.84817	3.49372	3.20973	4.83993	0.84625	-0.06946
ENSG0000(	HERC5	4:8845711	12.5673	11.453	6.38609	5.58644	8.07332	17.2785	38.0588	18.4892	3.07387	7.51645	0.0201	0.97482
ENSG0000(	FBXO10	9:3751089	0.353662	1.07629	0.250547	1.05242	1.05714	1.36986	0.24513	1.27515	1.58715	1.96933	0.5968	0.550252
ENSG0000(	CTD-2514	17:742135	0.27421	1.63741	0	0.083406	0	0.909768	0	0.478337	7.767	0.078429	0.17335	2.51849
ENSG0000(	RP11-100	15:379847	0	0	0	0	0.069377	2.69847	0	0	0	0	0.48285	4.52993
ENSG0000(	QTRT1	19:107014	30.7552	44.5547	64.2264	87.9962	57.4116	15.4667	20.134	45.7033	45.6987	44.3945	0.0535	-0.73312
ENSG0000(	C1orf131	1:2312237	47.7161	34.1156	42.0838	49.9962	50.8339	57.3003	30.4895	37.3014	50.7887	32.5587	0.8141	-0.08813
ENSG0000(	PDLIM2	8:2257827	128.331	99.009	162.995	207.446	170.615	97.2101	100.218	107.332	88.8949	149.053	0.11785	-0.50462
ENSG0000(	KIAA1147	7:1415292	3.63029	5.92181	1.18995	0.791244	2.04851	0.821372	2.7626	0.812409	2.52993	5.6872	0.85335	-0.18758
ENSG0000(	CCT7	2:7323341	132.714	107.209	173.759	200.641	158.457	145.337	141.992	130.549	111.258	89.4975	0.2988	-0.34515
ENSG0000(	MX2	21:413619	20.3214	35.6905	22.3344	28.2576	16.077	14.2734	104.153	38.3279	24.6012	20.8932	0.06035	0.690428
ENSG0000(	LRP1	12:570888	0.502557	1.88147	0.054096	0.231476	0.150887	5.21105	0.002304	0.075252	0.272424	0.517537	0.28935	1.54703
ENSG0000(	ADSS	1:2443526	30.8193	22.6312	24.5571	15.6174	18.0508	27.0965	35.3826	28.1199	27.9034	20.2223	0.32035	0.346399
ENSG0000(	RP4-569M	20:472092	0	1.0314	0	0	0.045956	0.782334	0.5085	0	1.29296	1.38284	0.32755	2.04444
ENSG0000(	ATAD3C	1:1449688	3.63966	0.779963	3.92867	13.7909	8.65847	3.62116	2.06143	0.875751	9.30643	2.9333	0.6251	-0.7491
ENSG0000(	AMDHD2	16:247505	24.5928	31.0021	18.2117	22.2737	14.2148	33.3887	27.5821	22.3001	21.2918	11.5532	0.9794	0.012602
ENSG0000(	CD22	19:353192	0	6.29421	0.478018	3.93688	0.652139	0.077749	0	0	0	0.469732	0.07185	-4.19879
ENSG0000(	PPP1R21	2:4844059	16.5408	14.4618	7.81726	7.54737	7.04066	9.7153	7.0457	11.5969	6.22859	18.2872	0.53	0.301965
ENSG0000(	RP11-2C2	16:306977	1.81376	1.59765	0.036937	0.327038	0.41604	0.984176	0.265642	1.07386	0.423625	1.19754	0.5814	0.788506
ENSG0000(	RP11-101	1:1930122	17.421	20.1452	6.68615	5.34636	14.1801	19.3641	17.4268	8.45738	3.12797	19.602	0.9195	0.143459
ENSG0000(	CCT8	21:290558	104.928	88.3717	79.2794	99.08	81.0433	106.543	88.7855	85.5173	132.79	56.77	0.85195	0.059968
ENSG0000(	ME3	11:864315	0.694609	3.45514	1.31724	0.24483	0.698791	0.764913	0.102473	0.348723	0	1.14783	0.04155	-1.74084
ENSG0000(	LINC0054(	13:220409	0.587409	0.390448	0.704949	1.25084	0.55945	0.154079	0	0.111176	3.77627	0.637318	0.8023	0.430078
ENSG0000(	TOLLIP	11:127437	19.4474	17.8545	27.8334	23.4998	25.7643	29.3459	22.0412	24.2749	15.9557	20.5151	0.3755	-0.32767
ENSG0000(	OR7E7P	7:9794698	0.564324	0	0.13987	1.12483	0.18711	0	0.352765	0.358458	0	0.354679	0.57605	-0.92129
ENSG0000(	SAP130	2:1279412	7.9023	10.1307	4.26213	3.91946	7.17939	1.97396	7.0756	8.45216	5.16981	10.5494	0.8221	0.105071
ENSG0000(	TRDV1	14:220960	6.90005	10.7174	5.37246	11.0439	0.620588	24.5104	239.8	13.5424	2.17411	2.9274	0.00005	2.9961
ENSG0000(	SLC35G2	3:1368186	0.148563	0.355229	0.765978	0.462982	0.249717	0.08369	1.55427	0.488344	3.9609	1.68614	0.1374	1.99365
ENSG0000(	TLR2	4:1537014	1.47348	5.46437	0.120892	0.364719	0.197844	5.91714	0.02416	0.56009	1.8722	0.577796	0.74065	0.282772
ENSG0000(	DENND5A	11:913882	5.82769	19.1037	4.0009	4.11887	2.80803	7.67773	2.55997	4.81444	1.64243	4.02119	0.20995	-0.59284
ENSG0000(	RP11-756	4:1887405	2.07023	3.28622	1.02592	0.69391	0.523559	2.82006	1.74454	1.93141	1.269	0.900263	0.6709	0.715692
ENSG0000(	AC006077	5:1349049	6.39005	5.34688	8.41018	10.0693	4.25513	6.4687	6.3224	5.04307	1.53916	5.33666	0.8159	-0.42781
ENSG0000(	SPACA9	9:1327255	1.2006	1.27247	1.14417	2.58844	1.3572	0.039921	0.728221	0	0.022399	0.816023	0.17005	-2.41635
ENSG0000(	CRTC1	19:186836	0.538595	2.2504	1.34467	2.1327	2.55863	1.55534	0.420119	1.48993	0.65231	4.37236	0.85145	-0.19877
ENSG0000(	CHCHD6	3:1267042	9.46777	6.04337	11.6262	10.9548	11.241	6.01668	13.5429	10.5073	8.99341	2.80249	0.63675	-0.28465
ENSG0000(	RP11-266	16:250317	4.49977	7.16949	5.91898	5.98092	3.87445	4.74447	2.97226	10.2478	7.05188	4.93577	0.78095	0.179549
ENSG0000(	KIFC3	16:577582	0.4183	0.722626	0.906962	0.460055	1.22121	6.50225	2.82995	1.48623	1.15987	1.30319	0.00475	1.57658
ENSG0000(	ZBTB14	18:528901	15.481	16.2745	8.55175	7.28929	6.34195	10.2135	8.38739	10.3275	2.15688	13.6404	0.37695	-0.37713
ENSG0000(	ADGRL1	19:141371	0.672077	1.27333	0.484977	3.33289	1.11923	1.07876	0.211284	0.028764	0	1.18051	0.06365	-1.89399
ENSG0000(	ZBTB32	19:357045	8.09599	16.9765	1.11556	4.48589	0.55509	6.43206	131.309	10.2791	2.93599	3.49774	0.00325	2.18964
ENSG0000(	ARHGDIA	17:818677	52.2601	37.055	40.2043	51.9281	72.3709	40.1752	57.3846	59.0351	60.5761	64.008	0.6123	0.170219
ENSG0000(	DNAJC9-A	10:731103	5.97904	5.27617	6.6215	6.8356	2.86935	1.36257	6.98145	6.09569	6.17703	3.48265	0.90555	-0.10101
ENSG0000(	TMEM189	20:500811	15.915	26.0144	15.8052	22.7839	18.4163	24.9109	19.2677	14.834	11.4004	19.6298	0.7803	-0.2229
ENSG0000(	C8orf58	8:2257827	6.45426	6.85213	3.86585	3.7062	4.035	2.2214	5.01151	7.13683	10.0923	5.26673	0.6693	0.365045
ENSG0000(	ZNF559-Z	19:932377	0.991977	0.536481	0.519966	1.73545	0.331501	0.128345	0.005884	0.511163	0	0.95763	0.23965	-0.77326
ENSG0000(	ZSCAN5A	19:562196	6.52913	4.01715	8.91806	9.1547	4.23476	1.97494	3.58655	3.50559	6.25052	6.26119	0.4139	-0.48562
ENSG0000(	FAM222B	17:287559	7.64974	8.91221	4.08249	4.23902	6.13466	8.9577	12.217	6.786	3.29405	8.32508	0.8687	0.095841
ENSG0000(	RP11-539	4:6687447	23.8653	7.15755	64.0601	62.7882	42.5505	16.8408	17.7113	17.992	34.7936	27.7111	0.0301	-0.85747
ENSG0000(	RPL23AP1	6:2972277	0.697667	2.0836	0	0	0.991136	3.53348	2.87331	0	0	1.61755	0.52345	1.24261
ENSG0000(	ZCCHC24	10:793823	0.759962	0.683071	0.452599	0	0.301058	1.39574	1.09481	0.516687	0.138323	0.796404	0.6009	0.736835
ENSG0000(	STYK1	12:106189	0	0	0	0	0.070037	1.24934	3.07938	0.71255	2.11642	0.941153	0.23895	6.73625
ENSG0000(	RP11-358	20:323550	0.080625	0.593938	0	0.141023	0	0	1.18395	0.602349	0	0.399868	0.2896	1.69198
ENSG0000(	C6orf89	6:3685482	16.0463	10.7786	8.45032	6.44345	13.5119	15.8181	12.8309	12.6763	9.99021	19.225	0.91825	0.057447
ENSG0000(	ATPIF1	1:2823610	390.551	273.55	597.911	603.15	349.997	451.768	396.26	438.862	332.083	146.966	0.36725	-0.43337
ENSG0000(	UBE3B	12:109347	12.2051	13.6927	15.3051	14.7079	17.9568	10.3746	10.1176	11.2508	4.66839	22.8164	0.37905	-0.56896
ENSG0000(	RP11-218	12:564295	3.69095	2.76479	1.72952	0.194312	1.35372	0.098225	3.91136	2.82806	1.02362	4.43868	0.816	0.32794
ENSG0000(	RAD54B	8:9437195	5.37056	4.08301	2.15968	3.5729	1.77697	2.81014	1.13675	1.73481	0	2.299	0.2794	-1.06024
ENSG0000(	TRAPPC4	11:118998	58.7764	61.2333	100.198	86.6978	109.804	108.783	121.094	108.336	85.0453	49.9986	0.9934	-0.00594
ENSG0000(	RGS9	17:651008	5.29884	2.47382	3.19414	1.38408	6.84452	21.8365	26.1284	12.3589	4.5439	8.8521	0.0015	1.88768
ENSG0000(	ARMCX2	X:1016552	0.085078	4.28674	0.263913	0	0.55981	0	0	0	0	0.657433	0.248	-3.00173
ENSG0000(	ICAM2	17:639960	177.172	157.427	287.761	409.43	339.52	36.4367	56.0868	67.1614	153.936	55.3842	0.00005	-1.86521
ENSG0000(	PSTPIP2	18:459835	4.59175	10.0654	2.83634	2.4123	1.31887	28.1233	1.16253	2.30906	1.50648	2.20494	0.33835	0.67865
ENSG0000(	RP11-472	11:435564	0.221752	2.07556	0.027781	0	0.191146	2.67961	0.714592	0.809363	0	0.223221	0.68285	0.992733
ENSG0000(	RP11-390	7:1237490	0.677875	0.455077	0.940661	1.16346	0.365629	1.61706	0.249304	0.200146	0	0.245697	0.52385	-0.70923
ENSG0000(	RPL35AP3	15:663868	0.933225	0.147963	0	0	0	3.79342	1.65019	0	0	0	0.42295	2.45514
ENSG0000(	GLUL	1:1823817	26.6094	35.6924	44.9778	27.4589	27.8055	368.135	85.002	104.088	40.2635	60.3147	0.0001	1.77362
ENSG0000(	TPD52	8:7991871	24.5977	15.1782	27.3612	23.0823	34.8076	19.5674	17.9007	17.4781	23.4385	16.7881	0.19555	-0.65671
ENSG0000(	RP11-180	17:629106	0.580444	0.882238	0.496128	1.06118	0	0	0	0	0	1.20628	0.48215	-1.46501
ENSG0000(	WSB2	12:118013	20.525	17.6761	7.17637	6.22977	22.9399	36.0256	22.061	17.0126	1.79237	25.7281	0.7636	0.214855
ENSG0000(	EIF3I	1:3220909	126.246	99.2901	216.672	183.081	148.977	126.9	145.798	173.202	197.043	62.2675	0.6313	-0.16506
ENSG0000(	SSBP4	19:184188	98.4186	86.0351	64.9374	66.2913	82.7992	61.4135	139.844	79.2026	37.9094	65.517	0.59845	-0.20452
ENSG0000(	CHN1	2:1747993	2.3384	3.77646	0.422647	0.766973	0.248148	4.34565	2.8968	2.6038	6.23859	0.976616	0.07145	1.31896
ENSG0000(	TMEM192	4:1650706	10.9364	6.38197	13.7394	10.7267	8.67595	5.09306	6.25766	4.6424	8.35377	12.7144	0.46755	-0.5111
ENSG0000(	ZDHHC3	3:4491525	23.9849	24.3093	18.7339	15.7329	20.4688	11.542	12.6456	15.5226	23.7313	22.4704	0.4956	-0.33181
ENSG0000(	TACC3	4:1721489	34.2682	71.1309	50.61	57.165	25.9563	45.2782	41.1059	39.9121	35.0487	50.0184	0.6766	-0.1315
ENSG0000(	TYMP	22:505235	58.0885	128.753	27.3327	39.9376	44.8128	620.755	848.348	259.337	225.463	138.84	0.00005	2.72749
ENSG0000(	ZMIZ2	7:4474858	7.02804	13.8382	2.03374	7.46968	4.28099	8.01409	13.276	5.00966	2.7222	6.57377	0.84635	-0.15738
ENSG0000(	ZG16B	16:283016	0.217211	1.69048	0.248799	0	0	2.44975	2.16515	0	0	0	0.50475	1.08372
ENSG0000(	AC018693	2:3879772	0.262162	0.411517	0	0	0	1.8002	0.635298	0	3.43196	0	0.2633	3.3459
ENSG0000(	DRG2	17:180878	36.3535	40.0749	33.4097	26.471	36.4747	36.3103	22.8645	31.9125	43.0462	26.009	0.8848	-0.07077
ENSG0000(	AC005154	7:3049662	8.01649	26.5332	12.3451	12.3777	8.78783	16.5592	6.3611	10.1508	6.73702	24.1662	0.75775	-0.14533
ENSG0000(	MCPH1	8:6403550	22.2994	15.257	12.2915	12.5718	7.67489	20.713	11.5633	6.34465	18.9106	12.8145	0.96885	-0.02069
ENSG0000(	CTC-523E	19:347885	1.47749	2.4611	0.541559	0	0.292568	0	0	0.627994	2.44674	0.534518	0.86765	-0.24491
ENSG0000(	RP11-142	10:225348	1.46274	2.33693	0.301947	0	0.168003	0	0.893513	0	0	0.179665	0.56515	-1.76159
ENSG0000(	FOXD2	1:4743601	0.51895	0.396996	0.281342	0.511959	0.338027	0.105301	1.00902	0.489397	0.443233	0.771432	0.38845	0.325813
ENSG0000(	SLC25A25	9:1280612	4.91514	6.03143	1.74496	2.09127	1.34689	3.56321	4.98096	2.4294	1.66839	4.97667	0.94325	0.058072
ENSG0000(	TBC1D19	4:2657643	9.53858	5.35805	5.00516	5.85048	4.57131	4.09351	4.08187	5.10479	12.8575	6.82759	0.97635	0.015614
ENSG0000(	ZFP57	6:2967239	0.24709	0.249265	0	0	5.02195	0	2.69171	0	0	6.63064	0.679	0.703499
ENSG0000(	ELAC1	18:509679	12.0871	11.1328	13.7353	16.609	8.18001	12.731	10.9133	6.76972	7.47764	2.91148	0.46585	-0.56279

ENSG0000	FANCE	6:3545236	5.47814	3.5407	4.24282	0.868997	6.14516	0.851225	4.43743	6.36019	4.37343	3.49786	0.98185	-0.01362
ENSG0000	RNF141	11:105116	9.7421	12.6834	8.02438	5.55438	5.34605	10.6795	9.045	5.26454	6.72198	3.35601	0.4287	-0.57052
ENSG0000	BTBD7	14:932375	2.96661	6.68249	2.87261	3.47662	2.73581	3.57362	4.01884	4.54595	2.65601	6.80878	0.85665	0.111055
ENSG0000	EML3	11:626022	16.5454	19.0023	6.90764	4.67988	12.3052	14.7478	17.457	18.3001	6.36494	30.9466	0.18055	0.577407
ENSG0000	RP11-550	3:1571752	1.94659	1.65135	1.149	2.30112	0.21259	0	0	0.665877	1.22794	0.724624	0.26575	-1.18811
ENSG0000	CTD-2574	16:299003	0.399523	0.339277	0.255478	0	0.383252	1.64316	0.031562	0.277958	0	0.757287	0.6728	0.772107
ENSG0000	PARP4	13:244081	22.1734	27.8848	7.61068	6.98121	6.40158	12.6227	11.5315	10.0806	2.7987	13.724	0.13525	-0.53088
ENSG0000	CREB5	7:2829932	1.57144	2.08361	0	0.674774	0.157456	0.377983	0	0	0	0	0.2317	-3.81231
ENSG0000	SELENOM	22:311047	15.4689	21.0018	28.8596	22.2664	14.0159	18.0798	23.0341	40.2733	25.478	6.60775	0.9823	0.013005
ENSG0000	DOLK	9:1289208	3.14481	4.77636	8.44891	5.12126	8.1742	5.64007	6.19454	7.73436	3.3108	9.34456	0.9489	0.060251
ENSG0000	SNX4	3:1254466	24.6382	18.4618	17.1223	21.0895	15.0787	20.2362	27.7639	14.8558	16.1417	11.4448	0.85025	-0.06538
ENSG0000	VAT1	17:430146	11.3308	13.8617	12.8739	5.07709	16.3262	33.4464	19.8953	6.94975	2.35154	21.2167	0.34505	0.399175
ENSG0000	JMJD6	17:767128	39.4006	42.3929	42.7832	42.8537	34.1654	51.413	45.3845	52.6497	65.5717	58.9088	0.16115	0.51085
ENSG0000	ZNF436-A	1:2335944	2.12178	7.74875	0.523204	1.4914	0.324535	1.87486	0.327714	1.14167	0	0.500877	0.49745	-0.85198
ENSG0000	CAPRIN2	12:307095	8.93761	12.5269	4.44828	4.71825	9.01316	10.8921	6.87393	7.40178	6.82117	13.1284	0.5461	0.262968
ENSG0000	RP11-867	11:662685	1.06017	0.770962	0.515559	1.3951	0.226862	0.424501	0.996424	0.954987	0.663501	1.38369	0.5923	0.840905
ENSG0000	RBBP7	X:1678642	101.261	105.305	89.3364	92.692	80.0032	71.1894	102.485	84.0392	100.44	69.3243	0.70765	-0.12833
ENSG0000	WAPL	10:864352	21.6436	27.8787	11.5555	8.89936	15.562	14.6979	16.5732	13.8241	6.66981	28.7622	0.60545	-0.17924
ENSG0000	NUFIP1	13:449392	2.55831	5.96308	2.63944	2.82124	3.04946	4.33336	2.06629	2.63478	2.47578	2.81948	0.71925	-0.24255
ENSG0000	LRRC8A	9:1288329	28.5549	24.1147	22.3823	15.3041	26.5862	9.7859	11.5128	11.3748	4.2912	28.5901	0.046	-0.89938
ENSG0000	THRIL	12:124993	0	0.270181	0.25263	0	1.41863	0	0	0	0	1.44793	0.3673	-2.61963
ENSG0000	UBIAD1	1:1127320	15.8419	21.4112	22.346	16.5825	17.5818	10.7707	12.2498	18.0055	22.4688	15.9074	0.64295	-0.16303
ENSG0000	RCN3	19:495276	1.06474	1.53173	0.966104	0.348781	0.262771	4.93412	1.12574	0.784892	0	0.093893	0.66225	0.605036
ENSG0000	RP11-214	12:123762	0	0	0.790763	0.902728	1.307	0	0.643324	0.853796	0	0.113306	0.5806	-1.13744
ENSG0000	RP11-146	16:306977	2.54032	1.82487	1.24317	2.38636	0.643293	0.802605	0.671498	1.92309	0	0.588837	0.22225	-1.58582
ENSG0000	PIP4K2C	12:575911	25.3939	22.0351	15.4538	14.021	9.94623	8.72184	18.263	24.1533	1.3479	16.9069	0.3374	-0.35496
ENSG0000	ABL1	9:1307139	7.75895	7.32116	2.66619	3.68321	4.08532	3.98331	8.56182	3.12009	3.94789	10.0611	0.975	0.021657
ENSG0000	SYNE4	19:360033	1.5626	2.07845	1.12078	0	0.128421	0.803631	1.9286	0.754481	1.51349	2.34437	0.45515	0.794409
ENSG0000	LINC0127	X:6334322	18.0709	13.2246	9.55493	9.56232	11.287	4.66973	1.85173	8.86741	4.06794	8.10978	0.016	-1.34465
ENSG0000	SPDYE2	7:1024824	0.980761	1.87178	0.986219	0.42659	0.295662	0.790758	0.966894	0.53402	0.239153	1.42947	0.78445	-0.35537
ENSG0000	LTK	15:415036	37.6168	1.62661	14.5415	19.9493	18.9158	18.0924	0.723175	4.56211	11.7584	23.402	0.1695	-0.59751
ENSG0000	CFLAR	2:2011161	131.021	79.1498	144.023	145.671	110.488	133.015	200.845	174.769	117.835	104.89	0.7219	0.168686
ENSG0000	SLC37A4	11:118998	9.14224	8.59657	8.86712	7.36079	7.24917	19.3599	5.33018	11.0208	4.21205	8.88741	0.80485	0.180877
ENSG0000	SMN1	5:7092502	26.0283	36.9741	27.7663	29.0391	13.8287	26.7186	31.8025	29.6158	30.6071	13.3064	0.71085	0.198692
ENSG0000	RBM8B	14:603964	0.113852	0.376295	0.25953	0.451485	0.091949	0.715878	0.444299	0.299731	0.35043	0	0.82545	0.40769
ENSG0000	SLC39A8	4:1022510	30.9536	17.8972	16.5131	23.749	14.2959	37.3836	11.0574	22.0659	22.4067	20.287	0.65885	0.160395
ENSG0000	PCYOX1L	5:1493580	11.1452	15.6623	7.44156	4.15039	4.86213	18.5996	9.21702	5.49257	0	15.5963	0.76685	0.179327
ENSG0000	RP11-620	12:579315	23.1248	12.52	25.9854	20.1651	9.00779	10.9058	11.6682	17.73	13.7664	6.3046	0.51015	-0.49026
ENSG0000	AC093495	3:1412493	4.80656	0.421946	1.79651	1.65504	0.353282	2.02641	3.33963	2.70291	1.21813	1.41813	0.1702	1.31258
ENSG0000	PRR13	12:534416	427.664	424.69	501.897	444.077	426.139	460.557	386.769	382.287	407.678	193.854	0.5054	-0.28869
ENSG0000	SPATC1L	21:461611	4.84571	17.1749	12.4501	3.77107	12.7494	10.6734	14.2786	9.02041	5.39976	4.97455	0.61255	-0.29723
ENSG0000	CHMP3	2:8650343	24.5093	35.0238	37.0288	27.9005	33.2556	50.3299	32.7218	32.8777	31.2389	27.1131	0.92625	0.049251
ENSG0000	NQO1	16:697069	7.11621	4.7889	2.29632	1.97418	2.22348	5.16226	2.17898	1.56053	8.31293	0.830154	0.86445	0.167308
ENSG0000	RNF111	15:587711	11.8773	10.2978	4.50233	3.38546	4.47917	10.2182	6.6415	8.14854	2.86082	7.58467	0.73375	0.259672
ENSG0000	FRG1	4:1899407	61.1849	50.6351	49.2717	43.5915	49.9491	71.2967	86.6892	39.5789	31.0213	16.3466	0.8173	-0.09267
ENSG0000	AC006011	2:1278435	4.91443	2.76091	3.32475	10.3835	0.370131	7.87281	0	2.4333	4.13134	0.343991	0.97425	-0.06157
ENSG0000	BRF1	14:105209	8.82666	8.40839	5.70704	7.49885	12.5411	9.06771	7.66093	5.08523	6.27753	16.8923	0.94885	-0.04324
ENSG0000	SLC35D2	9:9632070	22.1117	13.4285	8.1814	7.43188	8.31943	27.3609	40.8322	10.996	16.0384	10.6305	0.0995	0.852964
ENSG0000	HERC2P2	15:224955	18.6327	20.9491	4.358	3.81547	5.61179	12.509	8.81087	8.70313	5.89496	13.913	0.9729	0.012451
ENSG0000	SNORA81	21:316596	0	0	0	0.742321	0	0	0	1.15041	2.69924	1.17712	0.30865	3.7784
ENSG0000	TGFA	2:7044727	1.57367	1.06875	0.837626	1.31553	1.24563	0	0	1.5282	3.24026	1.03266	0.87005	0.137042
ENSG0000	SDF2L1	22:216422	71.5256	53.6741	108.674	139.982	129.297	82.2202	147.007	119.889	81.6175	33.9656	0.4483	-0.26275
ENSG0000	RP11-330	3:5222107	1.06368	2.83431	0.608788	0.889455	1.91203	0.766418	1.0947	0.676656	0	0.590717	0.37095	-1.32589
ENSG0000	ABHD16B	20:638612	2.15904	2.53688	1.5969	0.895535	0.863982	2.38312	1.1372	1.03853	0.938644	1.22253	0.8415	-0.38499
ENSG0000	RBM26-A	13:793118	1.05378	1.45253	0.725319	1.08877	0.145537	3.5734	0.131576	0.612743	2.9907	0.40631	0.5078	0.931979
ENSG0000	MYCBP2	13:769906	36.7175	65.5266	19.831	20.3145	31.4577	50.2441	85.4214	36.1634	31.5188	68.327	0.4573	0.645355
ENSG0000	CHST7	X:4657378	11.8577	14.4909	5.46537	3.29788	5.15679	14.94	8.98849	10.8103	7.08023	5.94816	0.5738	0.313649
ENSG0000	LRRC8D	1:8952483	24.8128	27.4135	10.7913	5.73694	9.93665	10.6532	8.76071	15.7991	7.25054	12.4324	0.31555	-0.56107
ENSG0000	TBC1D2	9:9819899	5.13315	4.5918	2.11108	2.45709	3.38109	10.7703	2.0803	1.79113	1.75069	5.60815	0.49205	0.424154
ENSG0000	RPSAP58	19:237629	0.755087	0.56466	0.948057	1.35992	1.4986	0.512372	0.648278	0.898934	0.193136	0.505119	0.6888	-0.82216
ENSG0000	HIST1H2B	6:2780744	2.12743	0.814167	0.403961	0	0	0	1.14632	0.446715	0	1.83689	0.788	0.392653
ENSG0000	ATRX	X:7750487	93.6138	114.447	26.1782	34.6025	46.4425	102.782	75.8552	21.752	30.3084	86.4384	0.5556	-0.21914
ENSG0000	POLR2J4	7:4392643	1.53087	1.8525	1.81835	2.10857	2.52955	1.76275	1.10467	2.42901	3.59804	4.44867	0.6871	0.478692
ENSG0000	SEC61A2	10:121296	4.94873	4.00451	3.11742	2.43387	1.742	4.19392	2.76348	8.87197	2.42118	4.80283	0.5428	0.410484
ENSG0000	TNNC2	20:458232	0.378807	0.580332	0.542557	0.31911	0.159139	1.59902	0.41761	0	0	0	0.9492	-0.00441
ENSG0000	OSBPL7	17:478073	14.9299	19.5384	5.87446	4.45678	6.10859	7.96049	17.1245	5.67761	6.69776	9.56742	0.87535	-0.06441
ENSG0000	RP11-529	16:702896	3.33517	3.57711	4.53517	4.90106	5.60473	4.75225	2.9705	2.27694	2.21567	4.08777	0.6329	-0.70836
ENSG0000	RP11-159	11:113314	1.5293	0.314633	1.0924	0.721176	0.207702	0.619839	0.140698	0.382165	1.31942	0.602236	0.89975	-0.17911
ENSG0000	NBPF15	1:1444213	4.14695	7.3657	3.1291	2.0693	2.35257	1.48987	3.90029	4.59643	5.07683	9.12008	0.3848	0.576502
ENSG0000	IMPA2	18:119810	4.77356	8.11698	2.27968	4.39548	3.14078	9.31546	3.08887	6.44725	0	1.04728	0.78895	-0.23926
ENSG0000	CHST11	12:104455	27.1078	40.7465	31.8517	51.6252	17.2882	47.596	50.4873	45.6985	70.0572	29.7864	0.69335	0.285079
ENSG0000	HINT3	6:1259567	3.32984	4.09469	5.06814	6.51192	2.94162	14.3026	5.03582	3.77014	4.74433	4.61724	0.36655	0.432367
ENSG0000	CCDC102	16:575121	13.8587	7.12621	9.49121	13.9952	15.3715	6.03958	9.19095	3.75239	1.2507	10.0055	0.14695	-0.81512
ENSG0000	OSBPL9	1:5156186	31.5117	27.5605	25.5169	34.2638	35.1123	10.8211	16.0431	14.9425	15.281	25.529	0.1741	-0.94224
ENSG0000	GTF2H1	11:183222	28.1612	33.5002	23.8816	28.9025	15.7313	46.7935	25.7563	25.4295	12.724	18.7201	0.965	-0.01737
ENSG0000	PLIN3	19:483833	15.5506	18.1404	16.1832	12.3629	15.0093	29.4278	32.7237	24.5422	20.2758	24.4376	0.0641	0.698593
ENSG0000	RP11-113	11:830720	0	0.615531	0	0	0	0	0.824064	0.49428	0	0.634979	0.29215	2.02414
ENSG0000	EDEM1	3:5122219	13.3363	17.7347	5.41384	4.52854	4.24289	10.6715	10.6106	9.08774	7.30292	10.9919	0.92115	0.052383
ENSG0000	WBP2NL	22:419987	0.644942	0.714426	0.446512	1.02846	0.441692	0.037298	0.183367	0.348344	0.400876	1.3195	0.68585	-0.45174
ENSG0000	LIN54	4:8281866	2.83961	4.91015	1.84056	1.47846	2.03811	1.00252	2.82798	2.39964	1.34859	4.94108	0.59005	-0.38092
ENSG0000	GZMK	5:5497786	670.738	51.3929	988.038	696.958	893.871	171.017	71.4882	317.581	181.957	170.754	0.00005	-1.89727
ENSG0000	SLC22A18	11:288777	12.5128	16.587	9.67487	12.0822	12.5539	40.1385	18.7965	10.0053	7.82222	16.7012	0.275	0.51731
ENSG0000	HTATSF1	X:1364970	43.2867	26.9693	33.7168	32.0383	46.5719	45.6767	33.3147	28.467	43.7423	44.7385	0.85505	0.060192
ENSG0000	SGCB	4:5202070	1.78264	5.44976	2.28713	1.64701	1.15859	2.20587	7.39677	3.65631	0.990347	2.27897	0.4627	0.421538
ENSG0000	RP11-294	17:506931	0.896555	1.0254	21.8897	12.1044	13.3004	0.221544	0.356963	8.67631	17.5393	11.5263	0.85975	-0.29928
ENSG0000	GDE1	16:195016	27.0365	20.4423	21.6769	22.9529	20.4784	53.8605	22.1572	35.8859	17.138	15.986	0.4348	0.282213
ENSG0000	TRAV18	14:220031	0.549311	0.78187	0.758582	0.780738	0.497238	0.233837	1.98085	0.727676	0	0.22449	0.9404	-0.12454
ENSG0000	CAPN15	16:527716	11.4754	13.2924	4.32467	3.10544	9.432	9.23951	10.2182	2.60187	2.54228	9.4167	0.6716	-0.26964
ENSG0000	GNB4	3:1793960	0.445098	1.693	0.12362	0.320272	0.147796	4.72973	0.898165	0.740328	0	0.436927	0.1577	1.154
ENSG0000	THBD	20:230456	0.285738	1.19848	0	0.032193	0	1.47136	0	0.74222	0.20691	0.498885	0.5364	1.19415
ENSG0000	WARS2	1:1190312	9.86349	4.97555	6.42384	5.78439	3.1587	4.28479	2.86894	4.14266	13.1074	6.80815	0.9993	0.000433

ENSG0000	SPATA5	4:1229230	2.24176	4.36546	1.23623	1.30174	1.16653	0.205715	1.46815	0.948819	1.45478	2.19698	0.2456	-0.95381
ENSG0000	GEMIN4	17:744413	11.8214	9.48373	7.14616	6.86588	7.33756	0.182274	7.94973	4.72696	1.3414	8.35919	0.21755	-1.05536
ENSG0000	FUZ	19:497669	3.30979	1.94213	6.45006	4.86188	4.59526	1.86245	1.41629	6.73463	3.57443	2.66095	0.63305	-0.35231
ENSG0000	FZR1	19:350627	5.13386	6.65671	7.09069	5.70348	10.6832	6.47464	9.68788	11.559	5.81649	20.8424	0.75775	0.227005
ENSG0000	CLECL1	12:971585	2.53898	5.72076	2.5174	1.24966	0.702588	0	2.38421	5.40803	0	0.766013	0.82955	-0.33919
ENSG0000	RNF135	17:309687	8.88889	7.63965	10.4158	14.5628	8.98391	11.3649	11.7539	4.77878	0	10.1523	0.4387	-0.49753
ENSG0000	ENTPD1	10:957117	7.30711	17.1432	0.216411	1.85805	0.49997	46.94	77.414	2.67724	19.2178	4.02258	0.0055	2.46926
ENSG0000	EIF4A3	17:801352	65.5231	79.9275	84.3683	88.7442	60.9756	65.7391	107.097	76.9068	104.769	62.0901	0.7205	0.111024
ENSG0000	RCSD1	1:1676273	113.65	109.203	72.895	71.5183	69.6798	70.0154	54.4607	38.3558	47.1146	67.8071	0.063	-0.63064
ENSG0000	ATG9B	7:1509909	6.85573	20.6329	6.11316	5.55033	4.86701	0.949591	10.7606	1.10653	10.2121	4.84405	0.49	-0.54188
ENSG0000	MCRS1	12:495391	50.3466	56.4774	86.3029	67.7902	130.781	47.9316	75.3217	81.865	54.6587	98.747	0.48705	-0.21789
ENSG0000	RP11-141	5:7021991	2.49809	4.89754	1.76907	2.17466	1.01332	2.28419	2.42487	2.38334	2.01133	2.80294	0.96465	-0.00022
ENSG0000	AAGAB	15:672010	40.532	21.5417	24.9828	41.6377	19.0266	34.7606	30.4868	34.6102	22.0607	23.1371	0.8506	-0.07539
ENSG0000	RP11-408.	9:1458879	0.730117	0.374646	0.265831	0	0.270916	4.00327	0.450631	0	0	0.439417	0.4581	1.51331
ENSG0000	ST3GAL4-	11:126283	9.01761	3.07235	19.4074	8.99344	8.36596	4.41203	4.89142	3.35869	6.35794	3.09718	0.40185	-1.19653
ENSG0000	AP2S1	19:468381	47.3786	39.7277	60.586	61.5276	76.6586	70.0659	47.1576	70.0531	42.583	29.9996	0.62795	-0.18483
ENSG0000	CELSR3	3:4863646	0.494372	2.15071	0.176329	0	0.251427	0.007832	1.7639	0.397212	0.273742	0.174071	0.9657	-0.05149
ENSG0000	DYNLRB2	16:800658	0.27605	0.899496	0.722124	0.850424	0.124792	0.289508	0	0.355079	0	0	0.2165	-1.86182
ENSG0000	AF230666.	8:1327753	0.298793	0.509993	0.09349	0.05911	0.229345	0	0.066568	0.252837	1.0244	0.584732	0.5453	0.85125
ENSG0000	XXbac-BP	6:3341966	0.840657	2.04744	2.23906	0.755484	0.338142	0	2.30805	0.605829	5.74352	1.38604	0.58345	0.922603
ENSG0000	CLCF1	11:673178	2.71161	1.16595	10.7156	3.87066	12.2412	4.27096	4.99056	2.18952	0	3.99236	0.35225	-1.25404
ENSG0000	DRAM2	1:1111173	34.6083	33.1047	33.1497	27.3424	22.1606	48.691	23.8261	37.9544	28.4261	11.8305	0.9811	0.009984
ENSG0000	AC006277	19:294903	1.07576	2.20004	0.315932	0.092758	0.452987	3.21626	0.070183	0.434495	2.26911	0.953604	0.513	0.864258
ENSG0000	HNRNPA1	16:516457	3.61326	3.83126	2.91347	1.66369	2.27516	3.07184	3.53584	1.59921	1.09008	3.03396	0.8034	-0.18668
ENSG0000	CASP10	2:2011828	4.75686	9.66748	6.75181	5.17237	6.86088	4.54941	4.22858	3.01377	11.4557	4.3152	0.42725	-0.56542
ENSG0000	RP11-18F	16:805979	0	0	0	0	0	1.04282	0	0.808775	0	0.510266	0.112	inf
ENSG0000	UGP2	2:6384093	94.694	73.2028	59.8027	53.0584	88.7851	82.4601	124.713	58.9963	82.5635	74.599	0.54325	0.192112
ENSG0000	ERBB2	17:396711	0.878565	1.13445	0.30156	0.365825	0.352731	0.438919	0.320265	1.24485	0.010121	0.601277	0.6474	-0.3945
ENSG0000	BISPR	19:174056	26.609	19.0055	22.2002	35.06	22.2836	20.1029	35.5004	23.8916	18.326	28.075	0.91515	-0.04439
ENSG0000	MRPL52	14:228298	74.8402	59.4907	62.1025	58.4433	32.818	156.224	100.889	63.5662	40.9877	9.46534	0.55755	0.361409
ENSG0000	FCGR2C	1:1615054	1.46898	9.87283	1.26657	4.43226	1.55746	13.1392	0.559641	1.5509	1.85477	0.317553	0.421	-0.67876
ENSG0000	NAXE	1:1565917	69.9396	78.6852	109.38	81.2652	108.101	79.0828	80.769	74.1785	91.9104	33.3742	0.2566	-0.37538
ENSG0000	PCSK5	9:7589064	0.046569	4.61055	0.780068	0.898262	0.077555	0	0	0.772545	0.008079	0	0.1511	-2.92748
ENSG0000	PTPRC	1:1986386	713.695	705.556	518.27	411.588	440.604	387.684	446.677	376.613	398.767	614.651	0.40085	-0.38612
ENSG0000	TMEM159	16:209331	6.58332	10.1064	18.9506	8.41539	14.7805	7.0485	6.66571	16.352	16.8869	6.33533	0.82065	-0.11411
ENSG0000	RP11-435	9:9544297	1.26613	0.859762	1.73858	0.819956	1.77303	0	0	0.339067	0.752677	0.919903	0.38245	-1.55874
ENSG0000	IFNL1	19:392963	1.23655	0	2.37311	1.50446	1.79711	0.587716	0.899546	0.3122	0.584773	0.820069	0.6349	-1.24352
ENSG0000	FASTK	7:1510765	91.7314	109.246	61.9271	57.9276	58.7983	102.098	92.2132	66.6767	42.2246	67.2063	0.79915	-0.08439
ENSG0000	TALDO1	11:747328	121.511	155.626	110.066	89.8343	145.094	204.56	142.711	113.439	92.5662	71.0388	0.9155	-0.0333
ENSG0000	TUBGCP4	15:433581	9.00936	14.5734	4.93961	3.57536	5.0071	13.2653	3.35146	8.90536	4.22497	12.7732	0.81755	0.135243
ENSG0000	ZNF275	X:1533341	2.79785	2.10799	1.84341	0.45336	2.54941	0.739801	1.20601	2.12567	2.14852	5.34691	0.77145	0.147772
ENSG0000	LTBP2	14:744981	0.450724	1.46574	0.540626	0.541978	0.123078	0	0.499604	0.039555	0.074742	0.023458	0.0401	-2.27446
ENSG0000	RNF185	22:311601	16.4395	18.7745	12.2022	9.84761	8.33496	14.0708	12.5832	10.4963	5.32216	13.6401	0.40715	-0.30538
ENSG0000	SLC2A8	9:1273971	13.371	2.27112	6.2088	7.06839	11.1142	17.2513	25.7594	16.4328	14.7556	16.2309	0.04645	1.05184
ENSG0000	GADD45A	1:6768506	36.2148	28.6898	28.976	67.025	26.8732	80.5859	84.6682	45.3474	31.826	31.2074	0.26155	0.428067
ENSG0000	F8A2	X:1553821	21.9896	35.8509	31.8205	29.461	57.7747	19.0211	36.7365	40.0949	19.7433	49.9816	0.74475	-0.63347
ENSG0000	NUTM2A-	10:872038	23.8314	21.5714	15.7212	18.9266	15.5893	42.213	27.4125	24.4614	25.4812	20.0293	0.59415	0.340565
ENSG0000	CKAP4	12:106237	3.35374	8.0826	1.81061	2.92108	4.40285	2.70964	0	1.17662	1.40683	2.1904	0.2224	-1.35738
ENSG0000	SOWAHD	X:1197586	0.562034	3.71506	0.088124	2.65041	0.331304	11.6836	1.66856	0	0	1.33808	0.7157	0.887564
ENSG0000	CTTNBP2N	1:1123961	0.204684	1.06751	0	0	0.006083	2.81815	3.61266	0.170727	0.097808	0.642956	0.1162	2.79967
ENSG0000	PHPT1	9:1368444	60.1197	38.7133	128.413	129.962	75.7754	157.836	113.482	124.915	87.2782	27.3889	0.81605	0.094126
ENSG0000	SRC	20:373446	0.521404	1.17484	0.442015	0.451676	0.538343	0.899268	0.792447	0.959988	0	0.635689	0.95425	0.054312
ENSG0000	BRD8	5:1381397	33.6788	49.1756	30.2093	36.5904	24.2981	34.4343	17.9674	27.9402	26.732	32.7107	0.3654	-0.36755
ENSG0000	IDI1	10:971145	112.659	46.3041	74.3822	120.208	58.7894	128.605	147.614	97.5523	116.559	58.1336	0.3421	0.357473
ENSG0000	PCTP	17:557509	5.09306	3.52364	3.6539	3.28799	4.15554	4.7066	9.67539	13.5023	7.1917	3.69264	0.0897	0.989341
ENSG0000	AMY2B	1:1035256	4.12735	14.6291	3.477	4.2395	2.24371	0.602524	3.15382	4.45964	1.50305	11.9606	0.79255	-0.25649
ENSG0000	BCL2A1	15:799608	16.5579	11.735	24.1919	14.2717	19.0277	63.2231	15.1275	41.5998	23.1695	18.7069	0.07065	0.867887
ENSG0000	AP000347	22:236084	0.784603	4.84315	1.42932	1.63963	0	2.11414	0.547554	0.846384	0	2.14726	0.73565	-0.46163
ENSG0000	RP11-121	16:183580	15.4938	21.2446	4.52047	5.1024	4.65605	13.7565	14.3185	6.21887	6.0895	11.3711	0.9593	-0.05017
ENSG0000	SNHG14	15:248236	16.9458	16.4662	10.394	7.95123	12.8777	6.81715	7.29937	7.30226	15.0978	16.8884	0.53085	-0.44396
ENSG0000	SLC25A38	3:3938334	46.7508	37.8763	45.9456	48.2339	40.3411	39.0639	57.0815	44.198	39.5117	34.5183	0.78435	-0.08693
ENSG0000	PPP6C	9:1251465	26.6095	27.763	13.7024	11.2158	13.57	22.5608	19.1806	16.2515	12.6355	12.1596	0.66265	-0.14472
ENSG0000	CHKB-AS1	22:505688	2.03709	0.256139	0.231676	2.70099	0.936047	2.59781	2.01465	0.488261	0.551953	0.759554	0.8847	-0.17719
ENSG0000	CTD-2545	19:504667	0	0	0	0.559074	0.506246	1.09594	1.12601	0.215873	0	0.300534	0.61415	0.959547
ENSG0000	C2orf40	2:1060632	0	1.4261	0.491963	0	0.242622	0	0.944697	0	0	0	0.48935	-1.31022
ENSG0000	NT5E	6:8544958	0.107537	6.53317	1.82724	0.274044	0.437159	0	0	0.196243	0	0	0.2477	-5.1913
ENSG0000	ULBP2	6:1499419	0.715068	0.505529	0.818147	1.30491	0.528965	0	1.42407	1.62903	2.92174	0.725314	0.57415	0.820653
ENSG0000	PNMA1	14:737117	15.4575	27.6747	30.0464	25.775	15.397	26.055	37.534	50.4738	29.2199	28.8659	0.0766	0.606238
ENSG0000	FAM131B	7:1433533	0.319543	0.148465	0.207653	0	0.441395	0.249284	1.65084	1.65466	0.237278	1.69039	0.07645	2.49028
ENSG0000	ALOX12	17:685386	0.550513	0.878386	1.10699	1.48004	0.532656	0	0.268624	0.187163	0.071361	0.231942	0.0679	-2.56001
ENSG0000	CTD-2231	19:161116	73.6878	69.4164	65.3536	54.89	99.8885	119.762	82.9268	66.6525	48.9204	52.71	0.98055	0.027125
ENSG0000	RP11-455	16:300923	0.158911	0	0	0.169694	0.25956	2.61017	0.536121	0	0	0.042139	0.2916	2.67401
ENSG0000	ZNF283	19:438272	0.620698	2.03891	0.909348	1.8567	0.948708	5.07957	0.332027	0.563583	0	1.4077	0.9918	0.008317
ENSG0000	GPA33	1:1670525	4.1664	27.8515	2.13781	4.23339	0.878191	0	1.50982	2.06397	0	2.08081	0.0873	-2.69131
ENSG0000	NLN	5:6558967	0.500717	1.11325	0.129635	0.186934	0.246328	1.11478	0.871794	0.986466	1.37258	0.375835	0.1753	1.22679
ENSG0000	ZNF777	7:1494313	10.9885	12.1452	3.65201	0.084538	4.28086	7.65469	9.39137	3.11726	0.77613	3.60394	0.4373	-0.48758
ENSG0000	CCNE2	8:9481331	3.96573	4.87609	0.374963	0.343468	0.166192	3.05317	5.28252	2.61872	0.013058	1.39445	0.80795	0.239485
ENSG0000	HARS	5:1406646	49.9077	43.5096	55.3607	64.5727	60.3782	52.3348	35.8597	40.9575	35.4989	50.6965	0.4916	-0.39504
ENSG0000	PLPPR2	19:113553	1.69923	2.01487	0.090435	0.727519	0.438442	0.86145	0.37905	0.314077	0	1.00236	0.2978	-1.19298
ENSG0000	MORC2	22:309223	13.7909	15.1384	11.5393	6.85908	11.6025	3.53339	6.8094	3.81388	8.52713	10.2414	0.1123	-0.81604
ENSG0000	GFM1	3:1586442	32.3496	29.8982	18.5757	18.6882	11.3819	23.4969	23.082	16.1152	4.2308	16.4859	0.5199	-0.2712
ENSG0000	FECH	18:575482	8.04794	6.2677	9.07664	8.77709	5.98469	9.44642	7.34394	4.95013	11.4606	5.07625	0.76565	-0.13399
ENSG0000	OXNAD1	3:1626515	41.1504	75.0025	41.673	54.3593	40.5386	72.0167	89.315	77.4527	88.8057	69.6871	0.1979	0.619113
ENSG0000	VGLL4	3:1127230	14.9555	10.1295	7.32921	9.56965	19.3075	13.6618	13.6922	8.94536	7.73363	22.5754	0.994	-0.00546
ENSG0000	SFSWAP	12:131711	11.6082	21.5625	6.35882	7.85597	15.3242	20.8756	16.2684	6.05852	6.32124	19.286	0.8924	0.058972
ENSG0000	PRKY	Y:7273971	7.44145	11.1696	8.81294	6.26865	4.44742	7.39505	9.28349	11.2965	7.53565	15.4216	0.7616	0.142957
ENSG0000	ALG6	1:6336758	21.5891	18.806	9.73665	10.9683	8.26512	19.6113	19.2915	8.9606	14.6593	12.3063	0.9061	0.047362
ENSG0000	TSTD1	1:1609952	202.315	203.887	204.545	263.789	104.44	248.866	268.184	224.972	166.509	71.5031	0.8875	-0.06151
ENSG0000	SOAT1	1:1792937	21.5577	23.1822	37.4463	11.8218	4.9107	31.0831	30.6382	28.9822	14.9862	11.6823	0.84865	0.122483
ENSG0000	RP11-973	18:124388	0.10311	0.403016	0.615712	0	0.476193	0	1.02345	0.335511	0	1.79615	0.63995	0.929291
ENSG0000	HGH1	8:1441377	23.2354	17.4651	17.1841	16.2231	19.4897	14.6039	18.3921	11.9386	20.047	19.235	0.4611	-0.30737

ENSG0000(	H2AFZ	4:9994808	306.642	351.103	354.86	300.693	320.112	497.68	438.858	338.335	309.423	141.937	0.88215	0.051384
ENSG0000(	APOLD1	12:127259	2.41098	4.04387	1.13322	1.06895	0.380549	2.022	3.21575	1.44983	4.94183	0.46907	0.49475	0.79546
ENSG0000(	CDC37L1	9:4679558	11.6935	12.1962	9.12077	6.04819	6.75929	19.3679	14.9266	9.86107	6.96469	7.2368	0.7682	0.16149
ENSG0000(	CUTC	10:996595	51.501	30.2271	54.8275	58.5214	35.415	34.0479	31.7146	30.6094	26.8481	20.9918	0.1512	-0.63545
ENSG0000(	RP11-110(	8:5189932	2.02104	4.82768	2.10021	3.10184	1.29728	0	4.30583	3.26176	0	0.08555	0.4414	-0.90043
ENSG0000(	RUVBL1	3:1280516	62.8252	51.3713	91.7205	83.2363	64.6078	34.2157	30.7751	42.746	22.2972	29.1477	0.08865	-1.16196
ENSG0000(	CTSW	11:658798	444.325	125.115	788.184	260.473	997.081	164.803	415.041	370.902	29.4031	221.306	0.03845	-1.22796
ENSG0000(	LUC7L2	7:1393394	61.3822	84.7575	22.2486	16.0634	24.8813	50.4506	45.8072	24.5519	26.8954	47.5655	0.98095	0.009263
ENSG0000(	RASSF2	20:478002	17.1635	20.5568	8.30107	9.13322	8.41892	14.8346	7.96324	8.03221	16.4347	15.3531	0.96295	-0.017
ENSG0000(	MPV17	2:2730949	94.6655	105.363	128.752	163.671	138.432	123.011	82.4839	107.736	82.3942	71.2635	0.18355	-0.44266
ENSG0000(	ZNRF2	7:3028430	25.899	17.4233	3.14318	2.62845	4.0167	32.9741	26.5933	5.22074	4.31931	3.68937	0.25955	0.645699
ENSG0000(	CXCL13	4:7751175	0	0.250976	0.028543	0	0	79.2581	1203.74	164.524	46.7696	11.2799	0.2196	12.5729
ENSG0000(	DEFB1	8:6808516	0	0	0	0	0	2.75538	0.782846	1.6805	0	0	0.0269	inf
ENSG0000(	TOMM20	1:2351093	20.6182	24.774	15.7217	17.3627	14.4471	21.0938	20.9649	17.9203	12.6913	10.2768	0.72135	-0.12588
ENSG0000(	RP11-63A	17:469231	6.93343	5.75134	5.04503	3.28814	4.35693	3.18969	6.59798	3.22344	3.79817	2.77564	0.9447	-0.12508
ENSG0000(	RP11-264I	9:1980289	0.49189	0	0.105088	0.311569	0	0.274661	0.145885	0.498689	0	0	0.5621	0.991029
ENSG0000(	KLHL7	7:2310575	15.9046	16.9914	20.9145	24.0095	10.6482	32.6219	24.7027	19.3588	21.3917	8.58589	0.8631	0.102051
ENSG0000(	SUCO	1:1725323	9.92113	7.71345	3.56298	3.34953	4.19482	9.90674	8.19052	5.31734	3.16957	8.33871	0.7244	0.193739
ENSG0000(	SLC37A2	11:125063	1.10728	3.16935	0.028192	0	0.175152	10.2451	0.34762	0.060876	0	0.137673	0.39175	1.27907
ENSG0000(	AP5B1	11:657758	5.12551	8.18096	1.1487	0.669303	3.76467	5.97201	3.59403	4.1414	0.038548	4.93925	0.93465	-0.04069
ENSG0000(	GAPDHP6	8:1005097	0.430973	1.58034	0.56669	0.818153	0.336276	0.530701	1.73429	0.921092	0	1.66728	0.84275	0.316684
ENSG0000(	ASH1L-AS	1:1553352	7.28853	9.54989	18.1324	23.6405	9.48838	12.8317	8.25648	11.8553	12.2065	8.47398	0.76585	-0.37122
ENSG0000(	C1orf228	1:4467469	12.6998	112.511	20.1419	17.7741	4.93726	16.1659	12.4828	22.3605	17.1699	9.11478	0.13075	-0.92891
ENSG0000(	SETDB1	1:1509262	8.71338	14.6463	5.84888	6.97544	4.94345	6.013	4.29606	6.71693	9.86837	12.5332	0.8899	-0.07668
ENSG0000(	NPPA-AS1	1:1176178	0.508006	0.331897	0.053231	0.639455	0.159232	0	2.26197	0	0	0.898665	0.6325	0.801749
ENSG0000(	NME7	1:1691056	16.3622	7.25832	14.3881	14.2276	14.4269	20.675	19.954	13.1281	16.1746	11.2613	0.46255	0.335415
ENSG0000(	ZFX	X:2414622	7.55125	5.09024	6.38185	4.94055	6.74178	4.75334	5.52147	3.95811	12.7194	12.5613	0.78415	0.126003
ENSG0000(	RPLP1P6	5:1517423	0.108141	0.771244	3.20729	1.03218	0.427631	0	1.08807	0	3.54556	0.107986	0.86715	-0.28836
ENSG0000(	ITGA5	12:543536	32.8052	44.1027	13.9938	12.7807	12.5082	25.5583	2.83788	15.8252	16.1492	22.0044	0.42115	-0.29845
ENSG0000(	TRMT5	14:607347	0.920942	2.433	1.08468	0.90689	2.54523	2.75913	1.217	1.34639	1.04003	2.09636	0.89675	-0.14422
ENSG0000(	SNHG16	17:765577	35.3645	24.2168	58.1239	77.0144	50.9287	22.4521	20.2004	35.1481	37.353	30.1378	0.04265	-0.75416
ENSG0000(	MMP25-A	16:304668	39.5037	35.2576	39.7105	77.3498	67.3778	20.0226	26.2152	28.1132	85.9402	63.6516	0.80945	-0.15971
ENSG0000(	SYNGR1	22:393499	13.538	12.2205	14.6875	23.9387	13.192	6.76279	32.1662	22.1687	20.4085	10.8023	0.82235	0.110904
ENSG0000(	SDE2	1:2259827	6.52043	5.81362	3.88563	6.87578	2.88253	5.67383	7.19794	5.76632	4.43429	6.10842	0.6434	0.198207
ENSG0000(	BX470102	1:1535336	0.220289	0.071137	0.595918	0.778351	0.430554	0.487334	0.837947	0.469195	0.225744	0.769195	0.77975	0.410648
ENSG0000(	ATAD2	8:1233198	9.19994	11.2671	1.8899	1.19977	2.34975	3.98043	9.28109	6.93969	4.90497	5.86345	0.34285	0.384808
ENSG0000(	CTC-550B	19:550061	0.365707	2.1191	0.341378	0.445122	0.180114	2.16543	2.05262	0.800083	0	1.23523	0.5263	0.814353
ENSG0000(	RP11-18(	12:984855	0.695594	0.793549	0.588555	0.704626	0.401206	0	0.93654	1.34824	0	0.27883	0.7654	-0.54315
ENSG0000(	LDHAP2	1:2356610	0.62304	0	0	0.204327	0.332946	0.594857	0	1.24534	0	0.129017	0.59545	0.971558
ENSG0000(	RBBP4	1:3265114	114.881	85.0173	88.0295	66.5217	59.7749	88.9665	81.112	85.2421	58.3933	73.8376	0.48445	-0.26334
ENSG0000(	GAS1	9:8694436	0.296568	1.00446	0.54333	3.12568	1.15059	0	0.050554	0.040094	1.08187	1.2585	0.3161	-1.19538
ENSG0000(	UBE2V2	8:4800839	76.2212	59.3116	81.6577	59.503	36.5025	72.3421	73.5742	53.2712	70.044	26.6845	0.662	-0.1952
ENSG0000(	CTD-2287	5:1159624	0.571836	0.913515	0.312753	0.755529	0.393263	0.711501	0.988911	0.360568	0.391261	0	0.91075	-0.2313
ENSG0000(	FLT1	13:283003	0.587388	1.46324	0.074335	0.41893	0.150248	0.09234	10.3141	3.07139	0	0.65698	0.09085	2.22722
ENSG0000(	RASAL3	19:154516	170.211	163.648	51.7538	48.6694	87.031	196.922	172.723	94.9881	44.4142	180.275	0.26245	0.380529
ENSG0000(	CTD-3075	14:751769	1.996	1.45678	2.48495	2.0435	1.0187	0.718781	0	0	0	0.35764	0.2813	-2.96836
ENSG0000(	EPM2A	6:1455015	4.95188	2.18294	2.27096	0.376526	1.43062	3.27725	1.69821	1.38163	0	2.78684	0.7092	-0.2849
ENSG0000(	ELOF1	19:115511	39.7431	48.4777	53.7859	55.541	74.6038	53.9499	69.0229	61.7214	53.2686	27.6443	0.51245	-0.20876
ENSG0000(	RP11-73(	18:291699	0.229564	0	0	0.364662	0.034235	0	0.033093	0	4.50168	0.801811	0.25825	3.1432
ENSG0000(	C2orf42	2:7014987	25.6528	17.5938	15.4156	12.7377	10.8579	14.2004	9.82545	14.2286	4.78849	14.4107	0.4964	-0.58005
ENSG0000(	FBXL4	6:9886853	2.63974	3.65058	2.35504	1.58432	1.99775	0.970038	1.56997	1.76745	1.59598	3.52047	0.21615	-0.74118
ENSG0000(	ITM2C	2:2308646	8.17145	20.7416	9.78227	25.1934	9.23758	38.1198	42.2092	112.804	109.941	176.318	0.00005	2.71317
ENSG0000(	TLN1	9:3569694	79.1958	83.3945	37.0278	13.0658	90.9602	70.0594	60.5037	48.5403	18.2576	123.367	0.5909	-0.24177
ENSG0000(	HKR1	19:373128	19.9009	27.3439	17.5671	18.1424	11.8691	22.3562	17.8081	21.3613	12.1885	22.4835	0.71405	0.149675
ENSG0000(	CRYZ	1:7470548	38.8252	30.9051	34.2849	40.0597	22.3957	33.0404	37.526	35.3705	47.7987	12.7846	0.9606	0.021672
ENSG0000(	RP11-44M	7:1005722	4.68862	9.63428	1.09536	2.73352	1.46175	3.38226	6.67155	2.18004	1.41301	2.4693	0.7332	-0.57287
ENSG0000(	NDUFS2	1:1611843	87.649	117.92	96.4028	94.8292	85.148	91.6023	78.9043	91.6035	102.747	60.9881	0.6275	-0.15186
ENSG0000(	EXO5	1:4050874	7.77995	7.02689	7.92739	10.314	6.01734	11.8564	9.73176	9.11603	10.2822	10.7736	0.4373	0.331968
ENSG0000(	DBP	19:486153	33.8951	55.5981	40.4734	23.4752	52.1289	18.1223	21.2056	20.225	16.3686	31.4414	0.3726	-0.951
ENSG0000(	DHFR2	3:9398013	3.86555	4.20107	3.02619	1.16824	1.53217	1.9743	2.14736	2.10228	3.85847	3.58092	0.93185	0.088359
ENSG0000(	ST7	7:1169524	6.9044	4.99832	5.93613	3.05307	6.23645	10.7962	9.2971	5.93806	4.46004	4.1566	0.4778	0.346129
ENSG0000(	CTC-329D	5:1403602	0.711891	0.896452	0.594487	1.86511	0.308487	0.317159	1.46069	1.02565	1.59444	0	0.9501	0.109597
ENSG0000(	ZNF626	19:205358	8.0328	3.59251	4.11673	5.50379	3.89942	9.78021	0.276911	1.24087	2.24379	1.99323	0.4737	-0.80027
ENSG0000(	PLEK2	14:673869	0	0	0	0.682444	0	2.60494	0	0	0	0	0.62295	1.47985
ENSG0000(	HDHD3	9:1133734	18.4992	12.9764	27.1081	20.3311	23.2242	7.53788	23.4379	15.7232	21.2636	11.5725	0.3671	-0.40236
ENSG0000(	G3BP1	5:1517423	91.8862	55.2771	82.6193	56.6107	70.0622	72.207	55.0856	73.7789	86.9613	83.2006	0.75595	-0.1121
ENSG0000(	TP53RK	20:465578	9.42167	11.0055	10.8674	12.1842	11.2757	7.79103	17.099	12.3191	6.65924	9.41709	0.84225	-0.08087
ENSG0000(	MICALL2	7:1428464	0.758652	2.09402	0	0.735274	0.077756	0	0.024173	0	0	0	0.22215	-7.35656
ENSG0000(	TCEANC	X:1365318	5.50109	7.89031	6.23038	4.15087	4.56581	7.93051	1.20916	3.61822	2.07481	4.10585	0.2259	-0.8053
ENSG0000(	RP11-16E	15:656113	0.248966	0	0	0.334545	0	0	0.655572	0.086853	1.56613	0.097786	0.35545	2.36491
ENSG0000(	ETV2	19:356417	0.528148	1.04193	1.53026	1.1871	1.19739	1.24431	1.4964	1.8755	0	0.077978	0.72635	-0.34484
ENSG0000(	RASA2	3:1414870	19.7559	28.2484	11.9573	11.6612	11.7832	17.3251	13.931	21.7568	13.9588	32.3819	0.88105	0.05597
ENSG0000(	CEP85L	6:1184607	13.5487	20.5587	10.8835	15.3303	14.9752	9.11659	19.3748	10.6269	14.9767	34.2704	0.79835	0.122258
ENSG0000(	UBA6-AS1	4:6770127	8.89403	10.3153	13.0182	8.07807	8.61697	12.0805	9.90085	12.6826	4.43772	13.2156	0.8765	0.083242
ENSG0000(	CATSPER2	15:437269	1.69979	2.1385	0.770025	1.90056	0.672681	0.088654	1.12423	1.75819	11.7286	1.41662	0.3147	1.21501
ENSG0000(	DTL	1:2120355	4.37546	7.36927	0.037271	2.36283	0.777579	8.31159	5.59231	3.37123	0	1.86974	0.7201	0.276297
ENSG0000(	ZNF10	12:133130	7.86196	9.82242	3.43092	5.29754	2.34074	1.1976	7.59014	2.62381	10.3362	3.76449	0.94145	0.063722
ENSG0000(	RP1-167C	20:215699	1.59773	3.45455	0.405573	0	0	3.6198	2.1611	0	0	0.050416	0.90805	0.149908
ENSG0000(	TMEM237	2:2016201	2.48492	1.60447	2.69893	2.49894	1.45072	4.03613	4.56101	5.45106	1.68014	1.58593	0.4676	0.556687
ENSG0000(	TCF19	6:3115854	9.62548	10.5922	4.85667	5.04872	5.9504	5.43858	13.0099	6.69308	2.00462	10.4208	0.8702	-0.07952
ENSG0000(	YIPF5	5:1441581	28.1737	21.8434	33.6659	26.9431	35.6178	33.987	44.3359	44.9913	34.6695	56.8947	0.32325	0.341794
ENSG0000(	VPS50	7:9323233	8.15275	7.93797	2.86629	6.55517	3.11654	8.43008	4.66703	5.66447	4.15555	5.66808	0.8089	-0.10134
ENSG0000(	CDKN2AIF	5:1344020	15.8443	22.2623	26.552	24.9815	13.4496	21.2241	25.0281	20.6995	20.2885	8.70812	0.92745	-0.04638
ENSG0000(	GLOD4	17:757096	57.9323	60.0669	81.2243	65.7653	84.5556	73.0445	58.2633	70.7382	65.6302	46.6059	0.504	-0.22888
ENSG0000(	RP11-834	12:540582	3.43922	1.37841	4.50688	2.37301	3.52046	0	1.92874	2.0796	0	2.33828	0.4354	-1.18391
ENSG0000(	GUCY1A3	4:1556667	0.354401	0	0.682671	2.16251	0.154912	0	0	0.038975	0	0	0.22	-6.17554
ENSG0000(	MPI	15:748900	27.2362	19.5446	46.9937	41.8403	38.2482	9.38803	12.5051	21.6829	25.8783	29.2	0.05305	-0.87318
ENSG0000(	MAK16	8:3337082	11.2029	7.20037	4.68025	4.08529	4.89048	8.36024	5.61816	6.33258	14.0612	5.81589	0.43285	0.479532
ENSG0000(	GCN1	12:120127	16.9875	15.7563	4.5017	3.43757	6.70974	8.37541	10.5322	4.51231	5.18754	10.39	0.5303	-0.2185
ENSG0000(	CXorf65	X:7110388	5.34777	8.00457	6.4047	6.76802	3.50043	2.76085	5.55046	5.11636	10.0654	4.41101	0.93355	0.070773
ENSG0000(	SS18L1	20:621437	1.12585	2.10004	1.08643	2.14192	0.734765	0.868143	1.67165	1.70559	0.020188	1.92503	0.5219	-0.42643

ENSG0000(	OLIG1	21:329136	0.363073	2.15663	0.326237	0	0.146449	1.32551	0	0	0	0.182785	0.96705	-0.02301
ENSG0000(	SRFBP1	5:1219619	9.80767	7.07857	6.61965	8.75765	10.3729	14.0156	15.3032	4.67021	15.5676	8.72631	0.3613	0.402536
ENSG0000(	RPUSD2	15:405692	7.51648	16.6472	16.1274	12.4631	15.0651	7.82064	10.5581	12.5829	16.5129	4.66716	0.4792	-0.4008
ENSG0000(	JMJD1C-A	10:631672	0.813436	2.57566	0.563174	0.323345	0.127731	1.25389	0.290442	0.634066	0	1.18542	0.852	-0.28261
ENSG0000(	PDE3B	11:146437	19.3247	38.1945	5.51217	11.1342	3.96497	14.7382	17.0588	10.1218	13.2506	13.6892	0.40195	-0.31573
ENSG0000(	TRBV7-7	7:1425116	0.692175	0.891105	1.05985	0	0.469941	5.76273	0.80924	0	0	0.906857	0.4233	1.31256
ENSG0000(	NCF1C	7:7515663	7.83033	32.1523	6.22912	12.8722	6.69805	42.6308	8.96714	13.5255	25.9191	6.93783	0.2344	0.642644
ENSG0000(	ZFYVE21	14:103715	11.2201	16.5303	14.125	16.3256	13.8794	26.3677	21.2699	16.8044	6.45887	11.006	0.92055	0.042546
ENSG0000(	UBE3A	15:248236	42.0575	31.0716	18.6921	20.7665	26.9234	34.0303	33.8847	17.6371	25.5962	39.6072	0.748	-0.16873
ENSG0000(	RANBP10	16:677230	8.8173	11.5971	3.48475	4.45883	5.39082	4.92864	2.30615	3.98877	2.85343	9.9166	0.3067	-0.52241
ENSG0000(	MPZL3	11:118226	8.38779	3.50913	11.8672	7.01309	7.13462	9.47824	8.21038	19.7929	10.5847	24.5851	0.0453	0.735332
ENSG0000(	EZH2	7:1488073	11.8328	20.83	5.38049	6.65858	3.47758	17.2826	21.1569	6.89548	15.5601	15.8	0.1264	0.668205
ENSG0000(	TNPO3	7:1289541	14.3351	18.4006	18.7733	11.3137	9.23858	11.7875	6.09033	21.0942	14.2245	27.6978	0.93985	0.027895
ENSG0000(	ERGIC1	5:1728342	8.89927	6.19784	12.2766	28.652	16.8677	4.92906	1.95914	10.5366	28.5121	19.21	0.6824	-0.19204
ENSG0000(	CCT4	2:6186808	106.799	77.7226	91.1211	88.5291	101.231	82.5016	104.75	84.9516	70.2643	68.1485	0.6116	-0.18526
ENSG0000(	HAGH	16:179561	38.6768	43.5276	82.4004	69.1119	79.0251	65.6038	58.0298	54.1384	60.1033	53.8768	0.62435	-0.20742
ENSG0000(	RAD1	5:3490526	15.2647	14.3138	16.1687	11.3533	11.5577	37.0358	25.9463	16.9646	5.66965	19.9353	0.363	0.602903
ENSG0000(	DEDD2	19:421985	85.2159	79.2975	140.77	148.155	113.25	65.0269	52.0349	73.4522	74.012	81.4779	0.0332	-0.75265
ENSG0000(	RBM23	14:228932	41.8268	75.7021	42.7582	33.8881	30.9398	56.9262	50.7153	32.3257	21.712	45.1382	0.56685	-0.23461
ENSG0000(	ZNF652	17:492892	10.1293	10.0842	8.46818	4.76491	16.1437	8.58953	8.07601	8.31132	6.71226	25.6188	0.47045	0.335371
ENSG0000(	PEX3	6:1434508	14.7194	12.163	10.821	14.1572	6.83715	13.1489	17.403	10.5151	7.33851	5.49948	0.9552	-0.02834
ENSG0000(	WDR59	16:748713	9.34099	9.91212	9.18527	7.36648	7.49587	23.9234	11.3998	10.0354	3.29827	11.5975	0.4907	0.370133
ENSG0000(	COX5A	15:749197	125.841	97.5608	146.908	133.73	105.932	170.262	270.691	210.985	110.202	28.0041	0.46185	0.249072
ENSG0000(	ANKRD40	17:506931	2.45695	2.74589	10.2721	4.7569	5.55255	3.23257	1.98127	8.62202	4.45575	11.7879	0.93715	0.039399
ENSG0000(	ENO1P1	1:2363482	0.702786	1.72673	0.381268	0.741844	0.641679	2.90301	2.9704	0.610822	0	1.68189	0.39645	1.83473
ENSG0000(	ZFR2	19:380402	2.17952	3.14156	5.18056	5.76442	6.24233	1.63448	1.13201	0.584797	6.49814	1.74631	0.4057	-0.861
ENSG0000(	GIMAP6	7:1506253	80.0057	71.5708	91.8096	64.9435	74.7301	44.316	34.4362	86.9523	44.2932	120.854	0.36625	-0.30441
ENSG0000(	SYNJ2BP-(	14:703250	8.61059	8.23166	13.9047	8.99573	6.10143	14.2409	11.3052	8.5763	0.972301	1.88271	0.47015	-0.82391
ENSG0000(	TSC22D3	X:1077132	574.806	857.99	422.431	1255.85	262.621	277.166	824.13	487.615	1078.07	314.354	0.8403	-0.11072
ENSG0000(	RTEL1	20:636578	3.07343	5.63187	0.774206	2.04518	1.80858	2.45282	2.82629	3.24441	3.01326	3.94723	0.63165	0.395436
ENSG0000(	RP11-421	1:1009896	0.549398	0	0.146342	0.351486	0.174467	0	1.20584	1.65044	0	0.417232	0.34155	1.66528
ENSG0000(	RP11-161	12:491847	4.41032	1.57569	0	3.95921	4.28966	4.09139	6.16003	6.53308	6.29679	6.1026	0.6037	0.667914
ENSG0000(	CD99P1	X:2609347	9.4613	6.66291	6.06389	10.5793	9.12285	4.96519	7.06016	5.47582	13.9538	5.63565	0.79705	-0.17942
ENSG0000(	VPS13B	8:9901326	10.224	18.3303	2.63528	2.29722	1.75289	8.60533	6.11004	2.95249	4.93539	7.7066	0.8988	-0.10717
ENSG0000(	AFDN	6:1678269	1.03881	2.22272	0.492161	0.327553	0.77812	6.76676	9.94687	0.751104	0.042658	2.22589	0.0164	2.00031
ENSG0000(	SDF2	17:286483	51.3464	36.3161	61.4851	54.5296	40.6236	79.7525	64.3107	59.2395	23.9603	25.1345	0.88325	-0.06991
ENSG0000(	MBTPS1	16:840537	41.6598	39.3403	20.1714	21.8449	16.3141	22.6953	19.2781	17.9049	8.24392	26.1876	0.0747	-0.60535
ENSG0000(	UBE2O	17:763894	7.26269	9.30617	3.60983	3.2146	7.32215	5.63874	4.60799	3.99919	2.02894	9.0929	0.454	-0.32318
ENSG0000(	CTD-2256	5:1035270	1.36944	0.606867	0	3.32066	1.56846	0	0.853283	0.420181	2.88684	0.48543	0.80105	-0.46508
ENSG0000(	GPN2	1:2687613	7.23454	2.2796	12.2948	6.24759	10.2089	9.58129	11.3943	7.60199	5.70073	8.12835	0.89	0.087043
ENSG0000(	OLFML2B	1:1619831	0	0.416023	0	0	0	7.3995	0	0.430337	0	0	0.33405	4.45167
ENSG0000(	ZNF45	19:439020	8.94469	4.18661	3.84041	3.26898	2.27521	1.42855	5.24364	2.06923	2.31438	3.89865	0.367	-0.47043
ENSG0000(	AC008063	2:1619922	1.51577	0	0.341117	1.48845	0.364153	1.47252	0	0.404046	2.98525	0.370249	0.68955	0.643646
ENSG0000(	CRADD	12:936773	17.8905	12.8297	31.9869	43.0245	21.5427	24.8822	37.1016	35.7062	36.3714	6.8782	0.80485	0.105169
ENSG0000(	SCML4	6:1077041	41.0411	74.2824	44.941	30.807	47.2469	55.5554	74.6196	67.7931	54.8583	152.481	0.09295	0.715752
ENSG0000(	KIAA0930	22:451903	3.51796	5.24825	0.985484	0.675775	1.83774	4.21819	4.26465	1.76637	0.226429	3.53038	0.894	-0.11637
ENSG0000(	ARIH2	3:4891778	53.7124	58.0136	88.5425	103.494	48.537	61.4301	68.6398	70.4923	89.0984	58.3117	0.8284	-0.13849
ENSG0000(	TRAPPC12	2:3379674	25.5738	31.291	8.54604	12.5695	16.6639	17.0928	17.6725	12.8076	16.3874	23.3988	0.8964	0.057483
ENSG0000(	RP11-23N	15:525778	0.910147	2.28788	0	0.392164	0	0	0.271589	1.80593	2.58477	0.420536	0.67325	0.814055
ENSG0000(	TUBA8	22:180765	1.12075	0.580076	1.81236	1.64497	1.06637	2.26444	1.42023	0	0.622416	0.107382	0.80155	-0.32157
ENSG0000(	ZNF821	16:718459	8.75031	7.53135	6.74357	6.7554	6.27332	5.5382	13.2799	14.4226	4.69256	5.31652	0.873	0.10649
ENSG0000(	LRCH1	13:465531	3.49716	3.60161	1.82652	1.92759	1.90585	1.90599	2.12036	3.5432	2.64893	5.17905	0.61945	0.256748
ENSG0000(	AMZ1	7:2679521	0.050267	0	0	0.101772	0	4.45174	6.92751	2.73217	0	0.055993	0.00505	6.21046
ENSG0000(	RP11-278	12:326791	0.401491	0.507214	0.629432	0.5907	0.255255	0.844401	1.37191	1.58439	0	1.96372	0.30025	1.3222
ENSG0000(	SUGP1	19:192760	26.848	21.1133	32.5992	25.2244	26.9203	27.1587	20.804	19.9777	15.7728	27.6607	0.56925	-0.28262
ENSG0000(	RP11-61A	16:668028	1.41546	0	0.225457	0.885324	1.07153	6.3045	0.712948	2.05862	0	0.284376	0.49865	1.23007
ENSG0000(	RP11-66(	18:860944	0.465257	0.580904	0	0	0	0.133864	1.7641	0.490361	0	0	0.60585	1.3428
ENSG0000(	PCAT6	1:2028109	1.08775	0	0.995156	0.869403	1.10393	2.05775	0.30249	3.97028	0	2.02909	0.4454	1.07442
ENSG0000(	RP9	7:3309479	22.9556	17.6238	18.2646	25.1026	26.7024	14.5809	13.7704	14.3185	23.4025	13.4107	0.26685	-0.51652
ENSG0000(	LINC0046	1:2113828	1.81812	2.95902	1.05185	1.44748	0.790219	3.851	2.09471	0.992074	2.24936	0.595979	0.7228	0.444376
ENSG0000(	UBAC1	9:1359329	21.8287	24.7012	18.8216	7.37213	22.4121	26.7808	25.0837	8.61414	9.08172	16.1588	0.68065	-0.16884
ENSG0000(	CTD-2369	19:103048	1.1514	1.19162	0.417528	1.28302	0.778667	0	1.77659	0.280598	0.848482	1.62968	0.85345	-0.22527
ENSG0000(	NPAS2	2:1008201	1.92423	3.10515	0.970729	1.96484	1.91881	0.079673	0	1.03141	0.132217	2.82287	0.318	-1.44511
ENSG0000(	LIME1	20:636578	252.68	239.704	306.927	322.026	315.038	105.465	147.395	222.797	207.56	92.1949	0.0995	-0.89586
ENSG0000(	PRRC2A	6:3162071	3.49478	5.06053	1.48325	3.61129	8.51106	3.72209	4.36616	5.56896	3.85358	12.3618	0.69965	0.255618
ENSG0000(	EIF4EBP1	8:3803034	21.9085	30.0407	28.2294	34.4594	24.8424	117.25	53.181	55.6408	45.8197	19.6193	0.01965	0.992159
ENSG0000(	STK11	19:117755	24.0856	30.4773	4.4353	3.73257	11.7659	17.5444	35.9998	12.0535	2.85568	17.5564	0.8542	0.088783
ENSG0000(	MTPN	7:1359264	31.4304	19.5678	22.1649	18.6323	61.2559	22.71	28.9535	17.5719	12.905	34.2995	0.2444	-0.5746
ENSG0000(	RP13-977.	12:132186	16.4798	19.6767	23.2938	19.9844	16.6155	7.14769	6.3743	11.9453	4.96774	7.20985	0.014	-1.35739
ENSG0000(	RMND1	6:1514047	10.7268	4.47974	9.97942	10.6091	9.15879	12.151	9.85646	8.1777	4.65661	8.16023	0.77165	-0.14445
ENSG0000(	RP11-104	9:3987393	0.881829	1.38869	1.37339	3.20068	1.4786	3.14373	1.17572	1.80484	2.52099	1.72183	0.6835	0.686857
ENSG0000(	CCDC43	17:446564	21.5879	19.742	15.7517	13.5208	17.5983	24.4202	15.4959	13.791	11.1846	13.5163	0.67915	-0.16928
ENSG0000(	MRPS33	7:1410026	55.1243	29.3916	59.0505	46.4032	29.8022	72.8843	54.6331	52.6357	37.5858	13.2334	0.94355	0.035468
ENSG0000(	SP3	2:1739064	30.757	25.6924	8.78027	8.65031	7.52678	19.1144	21.1516	15.0239	10.3526	15.412	0.8023	-0.09616
ENSG0000(	PRKACA	19:140916	2.30564	9.30866	1.93972	2.04798	3.99588	1.49941	4.13517	3.65046	3.8218	6.27495	0.9061	0.089263
ENSG0000(	LETM2	8:3838620	8.11972	3.0113	3.61447	4.82975	2.19287	9.17089	5.5682	1.68634	3.62658	3.79102	0.9951	-0.00473
ENSG0000(	CCR9	3:4582331	0.474619	3.8108	1.11686	1.4372	0.330895	0	0	0	1.76703	1.46665	0.55825	-1.04177
ENSG0000(	MRPL44	2:2239574	23.2168	34.0599	31.5553	24.3051	33.4616	39.2473	39.728	22.137	41.2477	19.8419	0.72145	0.129751
ENSG0000(	NGLY1	3:2571894	57.5203	59.6529	47.055	49.0297	25.0278	48.9572	27.4073	21.0141	36.1748	25.9864	0.15415	-0.50251
ENSG0000(	ARL4C	2:2344930	264.284	179.586	241.011	126.939	293.859	108.116	132.56	95.2419	67.6784	144.075	0.0084	-1.0838
ENSG0000(	CHD3	17:785668	27.5339	53.3806	9.99537	5.27113	21.7186	29.3148	42.0846	17.9521	4.90789	40.6593	0.6595	0.273192
ENSG0000(	DTHD1	4:3628162	3.58053	2.09361	0.556967	1.92998	1.93225	1.7932	1.3116	2.42611	0.335584	1.96978	0.66025	-0.37175
ENSG0000(	ZNF100	19:217227	2.65543	2.78042	0.622601	1.03155	0.446886	1.03646	2.49705	0.565396	1.58352	1.05395	0.7536	-0.20399
ENSG0000(	RP11-178	16:870835	0.692449	0	0	0.877787	0	0	0	0	0	0.478817	0.56905	-1.70422
ENSG0000(	TRIQK	8:9288298	3.6463	1.4718	1.62946	2.715	1.83235	0.855204	0	2.83797	2.22494	1.82458	0.19935	-0.81185
ENSG0000(	RP11-407	16:572450	0.706605	0.455025	0.439292	0.760304	0.365953	0.564791	1.26512	0.198792	0	0.095841	0.82865	-0.29726
ENSG0000(	RP11-676	11:127940	2.6593	2.49931	1.1512	0.926803	0.707822	3.99928	3.22414	0.613334	1.22436	0.209801	0.9082	0.229444
ENSG0000(	PHLDB3	19:434749	1.93758	1.09764	1.91124	1.99094	2.04774	2.83194	0.588402	3.92168	1.94927	3.67009	0.47395	0.530101
ENSG0000(	CREBRF	5:1730563	21.2001	22.4439	12.1669	15.8373	8.02788	12.5009	10.168	14.8829	31.9337	11.2197	0.9239	-0.05387
ENSG0000(	ETNK1	12:226250	17.1918	17.4297	9.44468	12.814	7.49938	11.442	14.3974	12.729	17.6693	11.1045	0.8527	0.086682
ENSG0000(	THAP3	1:6624865	40.0506	31.8989	39.9182	33.2042	37.4359	34.0312	43.2559	30.2632	22.1224	17.6466	0.5808	-0.28312

ENSG0000	ARAP1	11:726850	34.0537	45.8592	11.7736	10.6356	13.4081	38.6688	23.6393	13.8893	10.7869	29.16	0.64805	0.192783
ENSG0000	CLCC1	1:1088158	4.14283	9.5018	4.43167	2.38664	3.61007	5.60947	10.6348	8.44897	4.32225	3.59295	0.5682	0.360438
ENSG0000	PSMA2	7:4290872	174.191	136.366	159.993	168.786	126.864	222.394	315.793	199.485	199.507	51.1989	0.31845	0.446133
ENSG0000	APBB1IP	10:264382	46.3506	67.7406	69.2028	69.9887	110.852	40.6361	58.285	57.1391	81.3134	143.775	0.90435	-0.043
ENSG0000	AGA	4:1774307	20.4939	11.0882	26.9143	19.5789	12.62	12.4836	23.3355	22.193	24.4562	9.71678	0.85	0.067307
ENSG0000	TDP1	14:899549	23.1989	22.2832	20.1226	15.5023	13.2462	32.662	18.5968	14.6548	24.4273	11.6229	0.9072	-0.04514
ENSG0000	CSAD	12:531576	2.02288	3.71018	2.26815	4.33528	1.88436	2.69734	3.96299	3.39779	2.28851	4.5881	0.61575	0.302226
ENSG0000	ZBTB42	14:104800	1.04247	2.21961	0.507129	0.780987	0.612937	0	1.42322	0.939508	0	2.76559	0.9753	-0.03564
ENSG0000	MPND	19:434352	2.58525	1.9019	2.61051	1.69859	5.02626	9.05882	4.32526	2.15098	0	2.62493	0.81045	0.232577
ENSG0000	SRP68	17:760387	61.6051	59.3476	55.9906	48.2629	53.194	50.4515	53.4237	42.9017	32.5711	46.2222	0.30335	-0.47267
ENSG0000	CTA-305I2	7:1018159	0.081347	1.2716	0.189485	0	0	2.01531	1.04314	0	0	0.076273	0.6518	1.05491
ENSG0000	ZNF232	17:510554	7.50578	4.74867	9.24743	3.24194	7.9418	6.51734	6.77288	5.16197	4.96605	6.10654	0.7965	-0.18158
ENSG0000	TADA2A	17:374068	7.60719	5.99153	6.6754	8.26196	11.1991	12.6879	16.7299	10.8182	2.52846	9.8173	0.9552	0.029047
ENSG0000	LTV1	6:1435368	26.6783	26.7745	26.507	28.2272	23.4149	31.1146	29.0808	24.8548	10.2914	29.0178	0.86385	-0.12594
ENSG0000	AF129408.	21:391841	0	0.089523	0.301805	0.476961	0	0	1.08101	0	0	0	0.67455	0.794305
ENSG0000	TRRAP	7:9887793	2.38726	2.92935	1.35268	0.907165	2.40135	3.51065	3.64185	2.1394	1.84896	5.69305	0.3444	0.471485
ENSG0000	NOMO1	16:148336	21.5606	20.1223	6.50346	10.3932	7.08136	11.0012	14.8718	7.18103	3.58781	9.33502	0.25725	-0.48718
ENSG0000	RP11-26J3	8:7991871	0.534949	0.278979	0.390609	1.05017	0.045236	1.38175	0.041943	0	0.060308	0	0.6897	-0.77553
ENSG0000	IL20RB	3:1368622	0.301222	0.767176	0.295741	0.365413	0.398566	0	1.13457	0.129325	0	0.259789	0.54585	-0.80654
ENSG0000	TOR1AIP1	1:1798816	53.7589	45.5959	38.057	55.4354	41.089	60.7756	40.4535	41.7939	32.819	53.4711	0.7838	-0.08588
ENSG0000	RP11-365	1:2355476	0	0	0	0	0	0	0	0	3.07169	0	0.22405	inf
ENSG0000	CWC15	11:949626	71.9874	72.272	89.3126	74.9124	76.6167	97.971	82.4808	64.9092	59.3629	24.4773	0.426	-0.26399
ENSG0000	IFITM3	11:318639	91.2327	104.909	120.943	188.181	186.796	214.605	251.962	219.958	260.783	97.4039	0.19995	0.486226
ENSG0000	RP11-368	14:530367	1.05476	1.66417	1.26907	1.21087	0.444121	0.164097	0.876479	2.09018	0	0.733428	0.9865	0.003612
ENSG0000	PSTPIP1	15:769933	100.609	90.2361	111.904	82.0381	103.769	114.523	176.578	121.108	70.5752	94.6302	0.5279	0.199837
ENSG0000	RP11-156	13:489749	7.21877	4.67168	7.60529	5.15399	7.71483	0.312182	1.58825	4.66129	2.69614	3.33538	0.46435	-1.33956
ENSG0000	FBXO4	5:4192525	15.176	11.9634	15.6999	15.2102	9.50995	7.6593	12.8865	13.1639	10.5837	9.72629	0.46015	-0.34268
ENSG0000	KHDRBS1	1:3201382	80.5696	66.2761	71.4563	54.9001	73.7496	68.651	67.4829	85.5845	70.1329	95.8184	0.69015	0.133472
ENSG0000	EXOC4	7:1332530	20.7588	28.1738	9.40738	28.2013	6.5123	25.3245	20.8308	7.49622	13.294	10.4661	0.5235	-0.32299
ENSG0000	FUS	16:311801	78.2972	100.947	50.6539	45.7611	55.8509	94.828	94.5312	48.8548	46.1565	67.0181	0.4903	0.314466
ENSG0000	PSMA1	11:144434	210.362	202.193	165.036	205.253	110.042	210.213	215.956	151.693	240.438	70.2053	0.80415	0.104788
ENSG0000	ZNF432	19:520313	2.36414	2.99692	1.77562	2.01547	1.75638	4.93336	2.32875	2.82809	0	3.0788	0.9189	0.085439
ENSG0000	YEATS2	3:1836978	3.68099	4.41475	1.66855	2.86599	2.31853	2.76553	4.1824	4.12758	1.08602	7.62611	0.38605	0.385489
ENSG0000	RP11-553	3:1142143	1.46485	1.94406	3.10798	1.22971	0.973923	3.10074	4.10883	0.739881	0.387564	1.23419	0.98045	0.03242
ENSG0000	MLX	17:425670	34.6378	47.6109	47.7225	58.3268	36.879	38.8397	37.3968	52.0219	50.4662	34.232	0.86655	-0.06149
ENSG0000	ZNF684	1:4053156	2.87991	3.0822	7.16978	6.39741	1.94918	1.93191	4.10633	2.5691	1.64306	2.42355	0.2285	-0.78386
ENSG0000	RPL15P2	14:948008	0.787476	0.021426	1.84214	2.62556	0.521456	0.262669	0.203743	0.823637	1.89358	0.087465	0.6275	-0.81093
ENSG0000	ANKRD11	16:892676	68.4563	85.1907	39.2359	32.1044	54.8109	84.4461	78.9516	37.5251	48.2795	90.8716	0.92485	0.040639
ENSG0000	MYH11	16:156432	0.301491	2.56225	0.191689	0.07653	0.06064	1.20034	0.736189	0.343006	0.255254	0.251664	0.86545	0.191027
ENSG0000	CCDC112	5:1152671	2.60569	3.35336	4.89597	4.50956	7.13327	0.338043	1.22663	2.08558	3.08357	2.43517	0.07585	-1.22785
ENSG0000	RP11-357	1:2073215	0.533549	0	0.165397	0.219773	0.249473	0	2.39448	1.49569	0	0	0.3387	1.91308
ENSG0000	ROPN1L	5:1044128	1.61782	3.5918	1.41917	1.40934	2.64455	0	0.459528	0.054629	0	0	0.1163	-4.59662
ENSG0000	OGFR	20:627964	39.9535	38.7643	20.0204	21.3516	25.3199	54.1047	78.7837	27.9257	24.8072	40.4535	0.1846	0.574364
ENSG0000	KXD1	19:185577	46.4269	58.5835	117.357	115.704	98.1266	40.8925	63.261	110.027	106.257	50.5842	0.54895	-0.21838
ENSG0000	KLF5	13:730549	0.181773	1.14617	0.767806	0	0.214925	0	0.276131	2.12977	2.36127	0.21529	0.338	1.18513
ENSG0000	MRPL32	7:4290872	73.2504	55.486	80.3631	74.2896	48.3841	91.98	81.323	76.0817	77.0316	19.6757	0.95945	0.043679
ENSG0000	PIK3R1	5:6821571	53.0995	35.3279	32.9249	37.5478	23.7704	36.0494	29.781	21.7198	39.3334	22.9372	0.50875	-0.25121
ENSG0000	MSL3P1	2:2338654	4.01541	5.34819	3.42699	1.73989	3.1584	5.88372	2.84895	0.014305	2.04175	0.706019	0.63025	-0.42308
ENSG0000	LINC0086	8:1259223	132.046	132.251	177.841	135.646	98.0522	44.6923	40.702	55.6189	91.2579	64.1921	0.0473	-1.23176
ENSG0000	RANBP2	2:1087194	8.61571	11.0679	2.80032	3.88956	4.53701	6.39332	6.51105	5.65929	4.51214	9.95199	0.78545	0.12279
ENSG0000	UHRF2	9:6413150	16.9594	15.8018	9.09171	10.3045	7.26095	20.9255	21.129	13.1614	31.6344	22.5981	0.0213	0.832212
ENSG0000	SAP30L	5:1544459	17.5957	16.6286	2.74962	1.35103	5.83686	20.574	18.2846	8.70247	0.421938	5.09643	0.8265	0.155856
ENSG0000	IL10	1:2067676	0.094632	1.9936	0.279515	0.060916	0.09771	51.2231	239.771	66.2161	18.8908	9.1177	0.0045	7.3772
ENSG0000	HIST1H2A	6:2612414	82.565	22.6523	31.5159	45.0097	18.4921	53.9438	34.7027	21.8344	19.5318	15.4377	0.31915	-0.39263
ENSG0000	CXCL16	17:473142	1.26209	5.96505	1.93301	0.743232	1.80427	64.5914	5.78723	4.401	5.99016	3.87385	0.0415	2.70435
ENSG0000	CHMP1B	18:116889	43.1897	40.6776	45.1754	65.3941	31.6041	37.6936	28.5918	37.8746	32.9795	25.7672	0.2282	-0.49813
ENSG0000	CTC-425F	19:129385	0	0	0	0	0.416847	0	0	0.213697	0.267102	0.263218	0.7333	0.713794
ENSG0000	TOP2B	3:2517433	55.5615	66.1982	18.0194	12.9967	27.3544	28.6671	30.7877	20.084	16.9673	41.1132	0.709	-0.15295
ENSG0000	ZNF839	14:102317	5.16463	10.0355	4.14505	7.86221	4.43199	0.695077	8.19672	4.08919	1.5246	10.3484	0.868	-0.16203
ENSG0000	KCND1	X:4896137	0.794239	0.360224	0.074383	0.071254	0.240972	0.129972	0.887393	0	1.12303	0.655338	0.4227	1.2172
ENSG0000	FOXJ3	1:4217653	11.1004	15.4027	3.63521	2.82677	3.96523	6.54064	7.27799	3.26466	2.66082	7.21419	0.36335	-0.47217
ENSG0000	ARG1	6:1315731	1.89099	0.279978	0	0.251826	0	3.38088	0	0	0	0	0.742	0.619488
ENSG0000	KDM3B	5:1383525	8.1063	12.8758	4.64584	3.07386	6.86672	9.59536	6.21778	7.04508	8.33191	15.0648	0.26745	0.449898
ENSG0000	RPS27A	2:5517479	636.413	641.69	813.94	568.752	313.791	578.547	580.067	605.675	425.364	57.2896	0.3454	-0.36227
ENSG0000	SNHG15	7:4498302	55.2274	38.0862	67.3073	96.6253	28.8787	36.4082	49.7404	37.3806	52.5554	22.4356	0.2749	-0.49799
ENSG0000	SYNE2	14:638529	133.581	94.9218	32.5198	50.9119	51.8855	84.2557	73.4428	34.4056	55.5948	92.365	0.85675	-0.16732
ENSG0000	FSBP	8:9437195	4.24634	1.911	6.18775	8.65646	4.69907	1.63091	0.388336	2.58586	0.147081	2.95526	0.17655	-2.03013
ENSG0000	EML5	14:885629	0.410236	0.519023	0.09958	0.508987	0.348357	0.128218	0.319823	0.168529	0	0.935779	0.88335	-0.19778
ENSG0000	SERPINE2	2:2239751	3.37157	19.4311	0.646833	0	0.248313	12.7056	15.9383	2.41719	4.39418	1.28609	0.47635	0.690293
ENSG0000	FBXL17	5:1078590	5.02558	6.87576	0.182635	0.859959	0.506368	2.88543	6.38304	2.17772	0.057631	2.08369	0.9945	0.006713
ENSG0000	SUMO3	21:448056	39.9551	32.9016	33.5332	42.6343	39.9214	47.1068	35.9297	26.2818	34.9101	13.078	0.3967	-0.29146
ENSG0000	PFKL	21:443000	89.1963	120.466	57.292	55.6473	60.5715	79.7062	72.0773	51.0674	59.2006	74.7388	0.65395	-0.14422
ENSG0000	AP5Z1	7:4775621	39.0822	52.3015	11.4492	19.5026	18.0438	57.1708	48.488	17.3093	28.1778	18.6001	0.33235	0.363667
ENSG0000	TMEM165	4:5539595	65.2298	62.2451	43.9576	17.3858	27.9828	112.417	75.189	51.004	46.2768	36.6424	0.08095	0.592481
ENSG0000	KIF13B	8:2906727	2.27156	6.06222	2.5667	0.95183	3.96255	5.50339	3.06721	2.2301	2.41986	12.6976	0.7602	0.210314
ENSG0000	NME9	3:1381872	0.870893	0.742358	0.565024	0.905428	1.39101	2.63608	1.37651	0.210009	0	1.3583	0.69895	0.388214
ENSG0000	C9orf66	9:212823-	0.994223	2.19108	0.180257	0.09217	0.073683	4.41612	7.17236	2.01674	1.15522	0.82967	0.14205	2.41558
ENSG0000	TPRG1-AS	3:1889417	0	0.059022	0	0	0	0	0	0.159586	3.998	0	0.1901	6.56454
ENSG0000	TBCA	5:7769116	124.477	73.1922	111.966	106.196	57.5148	211.708	169.112	119.541	128.843	18.4576	0.28985	0.417085
ENSG0000	PPP4R3A	14:914576	100.143	87.2094	31.0345	39.2771	38.8008	36.4312	48.6705	47.1363	27.4864	58.1713	0.2652	-0.3779
ENSG0000	RP11-747	14:915806	1.76341	1.41041	2.2854	0.976438	1.70316	1.84395	1.69081	1.05907	1.93669	5.84626	0.8328	0.328521
ENSG0000	CGA	6:8708549	0	0	0	0	0	0	4.55884	0	0	0	0.11245	inf
ENSG0000	TM4SF19	3:1962114	0	2.6116	2.28262	0.689902	0	0	0	0	0	0	0.02225	#NAME?
ENSG0000	TPRG1L	1:3625001	12.4404	8.9717	6.64291	5.81191	9.76486	1.94582	8.17006	7.25849	5.63446	8.20555	0.3469	-0.48259
ENSG0000	EIF4B	12:530061	53.0103	60.535	48.9274	21.1927	68.444	55.7697	41.6268	53.2215	30.3061	86.9275	0.92115	0.036163
ENSG0000	SLC25A24	1:1081340	14.3165	16.0341	4.27115	5.20493	4.91682	19.3349	14.7839	10.1559	12.7013	7.4857	0.25835	0.466383
ENSG0000	SLAIN1	13:776978	18.3303	19.9698	6.92819	4.02127	8.87065	7.57007	7.29115	4.86556	4.7519	8.19155	0.08785	-0.77016
ENSG0000	HAR1A	20:630954	5.80175	2.83416	8.42327	8.26058	7.6981	4.7437	0.302892	3.02904	12.1853	3.39745	0.6338	-0.3323
ENSG0000	ZNF571	19:374971	4.49991	3.01086	2.47122	1.3254	2.14121	1.37621	0.258941	1.04723	5.34272	3.38075	0.78595	-0.29882
ENSG0000	RP11-137	10:804541	1.983	2.70307	1.56115	1.25232	0	1.36714	1.40502	0.501823	0.880706	0.195801	0.5602	-0.76531
ENSG0000	RP11-269	1:4473981	0.211894	0	0.084144	0.89438	0.328541	0	0.272314	0.456026	1.08182	0.107823	0.84905	0.309515

ENSG0000(	PCYOX1	2:7025738	5.12621	9.28271	3.69755	2.97088	3.22106	3.87023	4.41227	1.74254	6.89528	5.01574	0.72075	-0.15306
ENSG0000(	RP11-46H	12:132768	0.91776	2.10975	0.560477	0	0	2.46673	0.993546	0	0	0	0.9225	-0.1906
ENSG0000(	LINC0108!	12:121712	6.6423	20.5173	7.69262	8.52424	6.5167	4.71937	8.52297	5.37737	11.5891	10.0644	0.841	-0.15778
ENSG0000(	AVIL	12:577623	0.392743	1.00099	0.775987	0.492271	0.364214	2.01481	0.973777	0.309856	0.034107	1.71559	0.46205	0.667622
ENSG0000(	HSPE1P2	14:635970	3.97828	7.54932	2.09022	0	0	5.53114	5.64833	0.298957	0	1.30425	0.95465	0.107418
ENSG0000(	SLC16A5	17:750877	0.730941	1.52555	0.386553	0	0.206018	4.93942	1.69149	0	0	2.24898	0.1441	1.63811
ENSG0000(	ZNF600	19:527641	20.222	7.29961	12.4307	12.7608	6.82493	24.7126	9.96258	13.0199	5.44801	10.0473	0.8929	0.075821
ENSG0000(	PI4K2B	4:2516064	12.1442	13.6612	3.33811	5.99998	4.36303	5.40759	11.4403	8.30543	0.324528	5.84125	0.6466	-0.25933
ENSG0000(	ABI1	10:266976	58.0988	52.8609	41.4847	50.0146	30.0756	39.502	47.5507	36.6318	40.5043	28.1525	0.4042	-0.27688
ENSG0000(	PIAS3	1:1458456	12.1817	16.4392	11.6904	13.2318	10.4423	12.2778	16.8289	16.4055	14.4218	20.6488	0.3979	0.32818
ENSG0000(	ZNF441	19:117669	3.22801	2.5651	0.838692	2.51566	1.02626	0.848474	3.32744	1.15157	0	1.49734	0.3663	-0.67845
ENSG0000(	XXbac-B1	22:209651	0.702194	1.11041	2.72775	2.57658	1.58151	0	0	0	0	0	0.1232	#NAME?
ENSG0000(	SMTN	22:310516	2.39227	4.87707	0.162346	0	0.458354	0.669677	2.30208	2.88445	2.51536	1.01633	0.58205	0.463886
ENSG0000(	PROSER3	19:357581	3.85536	6.20818	2.14255	9.6208	4.63398	4.49014	8.00137	5.54633	7.36334	4.32662	0.8446	0.106254
ENSG0000(	BTN2A1	6:2645790	18.6408	32.4426	22.2585	13.1312	14.7123	19.2818	24.982	16.181	19.5926	26.3008	0.9299	0.03328
ENSG0000(	MAPRE1	20:328198	55.3554	54.3068	59.2971	63.6162	39.0426	54.7437	47.0282	54.6555	60.3054	55.0142	0.8636	-0.05312
ENSG0000(	PTGS1	9:1223705	0.088818	0.875816	0.363084	1.64065	0.192948	3.9377	0	0	0	0	0.84205	-0.19117
ENSG0000(	CNOT7	8:1722496	43.9748	50.5971	64.8467	65.9338	60.789	32.1476	42.137	71.648	61.3946	58.4986	0.3874	-0.40657
ENSG0000(	TNFRSF13	22:419220	0.220238	0.703657	0.696264	1.50694	1.21524	0.350662	0.39812	0.952553	1.14346	1.0625	0.7887	-0.19489
ENSG0000(	BCAS2P2	1:1003444	2.80746	4.71472	0.231541	0	0.264287	0.068151	2.3601	0	0	0.387314	0.55555	-1.2329
ENSG0000(	JAKMIP2	5:1475599	5.70584	4.24227	3.31762	3.04873	5.74944	6.30508	2.55262	3.24832	2.91093	7.65497	0.74805	-0.19157
ENSG0000(	NUP93	16:567301	32.6596	24.9076	41.3699	41.071	32.7206	20.1201	21.3109	29.7867	24.667	39.2516	0.25905	-0.36321
ENSG0000(	PINX1	8:1072376	22.2792	16.9997	25.4622	17.0431	29.9958	38.3165	21.9439	13.2326	20.6809	11.3193	0.76885	-0.13834
ENSG0000(	CCDC90B	11:831569	57.8509	44.2665	46.7571	53.6079	35.349	63.2087	79.1847	43.5299	51.0745	20.5391	0.896	0.06514
ENSG0000(	BTN3A1	6:2640223	62.0321	74.2592	56.1841	35.8218	42.6806	18.0953	55.6652	50.2137	25.9253	81.505	0.3625	-0.28215
ENSG0000(	C1R	12:708020	0.885509	0	0.555162	0.778363	1.16908	2.01267	1.08779	2.12493	0	2.13501	0.2093	1.22733
ENSG0000(	FPGT	1:7419821	5.16938	4.6466	2.46344	3.36766	1.41991	2.39783	3.17661	1.67092	3.01919	1.6085	0.5503	-0.31348
ENSG0000(	MXRA7	17:766719	17.203	9.19642	1.4162	1.23613	7.11415	31.5477	31.8473	5.84804	2.83823	6.99776	0.04875	1.13034
ENSG0000(	ARHGAP2	10:245836	2.26855	5.05225	1.11347	1.0742	1.60677	5.51398	2.21051	1.24871	4.24683	2.30825	0.48195	0.39661
ENSG0000(	CYP2E1	10:133453	1.28727	0.812801	0.327417	0.788403	1.93857	1.45984	1.43493	0.779094	3.56738	2.79822	0.5054	0.860368
ENSG0000(	STRAP	12:158823	84.2125	61.7407	89.4043	91.3944	69.5572	55.1642	64.787	76.2683	108.055	36.9099	0.7196	-0.12396
ENSG0000(	RP11-495	1:1483629	2.58642	6.96785	1.35588	2.00702	1.19267	0.344076	2.60238	1.75477	1.51233	1.15312	0.3493	-0.76794
ENSG0000(	GBP1P1	1:8940767	0.461616	0.113442	0.558105	0.627905	1.40877	2.48878	9.83696	4.08851	3.35051	2.9355	0.04245	2.81338
ENSG0000(	DENND1C	19:643607	83.9179	95.1899	83.2145	71.444	93.0224	72.7656	45.7114	64.2241	40.0708	110.259	0.2029	-0.38922
ENSG0000(	UBE2M	19:585557	29.4999	19.1164	4.03199	3.66699	6.6784	22.0085	33.3063	7.83109	1.65004	4.69401	0.972	0.02096
ENSG0000(	TMC4	19:541601	4.38256	0.979089	2.26529	2.9837	2.1257	0.896281	0	0	0	0.813481	0.10345	-2.85716
ENSG0000(	PRKCH	14:611875	199.888	210.309	158.992	152.26	143.808	115.71	131.572	119.268	64.9046	151.626	0.07245	-0.59588
ENSG0000(	HSPB11	1:5391657	75.796	56.8981	83.3526	55.792	36.9433	140.316	116.597	98.2937	54.6085	23.8772	0.2902	0.424892
ENSG0000(	INTS6L	X:1355200	13.7594	25.2182	4.99736	7.62196	8.16776	18.4891	21.9162	11.2825	8.8488	19.0386	0.26125	0.499338
ENSG0000(	CLEC11A	19:507233	1.16111	8.83521	1.58941	2.59579	0.746296	7.90378	0	0.412214	0	0.620659	0.56855	-0.76009
ENSG0000(	NUDT16L1	16:469369	93.4904	60.5193	130.809	130.451	109.747	73.7806	76.977	83.612	109.497	49.1289	0.18835	-0.44072
ENSG0000(	TNNT3	11:191956	0.296052	6.10253	0.262746	0.309119	0	0	1.74443	0	0	0.41179	0.22105	-1.7517
ENSG0000(	FKBP14	7:2992010	1.78124	0.688566	3.5143	2.33118	2.04835	0	1.77558	1.68298	2.83105	6.56496	0.9473	0.083046
ENSG0000(	CDC25C	5:1382852	0.465596	1.92981	0.057787	0	0.028806	3.15728	2.01151	0	1.34408	0.325564	0.2334	1.6297
ENSG0000(	PTTG1	5:1604218	37.1241	75.307	8.27149	18.8263	6.0616	81.7246	113.049	65.3917	72.198	11.1493	0.01115	1.34231
ENSG0000(	PDCD1	2:2418498	10.0985	12.5631	7.27955	4.71933	10.3339	118.051	190.643	86.2052	19.098	88.8214	0.00005	3.35486
ENSG0000(	SLC16A13	17:703607	2.29532	1.6515	2.74917	2.01118	2.32499	4.75049	8.96038	5.41971	0	8.10046	0.40145	1.24944
ENSG0000(	UBE2T	1:2023316	16.5155	17.2954	5.8692	5.28666	3.08762	29.3391	26.6714	16.4177	2.91939	3.40255	0.16	0.79419
ENSG0000(	HIC1	17:205415	0.463246	0.394317	1.41663	0.768024	0.246209	0.416025	2.0419	0.486834	0.441943	2.14269	0.6276	0.53234
ENSG0000(	RNASEH1-	2:3531812	4.24262	3.3304	10.3813	6.05697	4.05397	2.23434	4.09479	5.22433	2.69511	2.87205	0.5243	-0.78744
ENSG0000(	AMN1	12:316471	11.9424	16.9364	8.31442	8.97872	4.73246	10.9053	16.4416	8.40738	17.2174	5.8041	0.68735	0.201288
ENSG0000(	RP11-98D	1:1517980	0	0	0	0	0	0	0	0	3.33099	0.648532	0.00275	inf
ENSG0000(	RP11-611	12:688081	0.563934	0.178934	0	0	0	0.220318	0.619636	0	1.90091	0	0.4153	2.39379
ENSG0000(	CPA5	7:1303448	0.946877	3.71444	0.37972	0.241194	0	0.975667	0.333043	0.847022	0	0.053276	0.2101	-1.3343
ENSG0000(	HMGN5	X:8111370	1.5853	3.03722	2.00021	5.71478	4.95789	6.28644	1.44552	0.598805	0.037814	1.61109	0.4636	-0.7328
ENSG0000(	GS1-124K	7:6649370	13.6467	8.7625	10.3951	11.1851	3.73245	25.9323	6.77084	7.71176	13.6325	1.90875	0.8686	0.212704
ENSG0000(	ECE1	1:2121724	14.094	30.203	6.2882	6.87435	5.62335	5.53616	24.3614	7.39643	2.43501	13.3522	0.2709	-0.43124
ENSG0000(	PARVA	11:123771	0.855799	0	1.56873	1.0261	0.930967	0	0	0	0.011975	0.504575	0.1791	-3.49057
ENSG0000(	AC021188	2:9630726	6.49886	4.58336	5.2519	3.64813	5.66125	11.0107	27.0121	10.1287	15.1939	7.04859	0.11235	1.32975
ENSG0000(	PPME1	11:740127	13.1593	15.5194	13.111	12.2544	12.5664	18.607	6.3735	12.8146	14.8035	16.5862	0.89855	0.059206
ENSG0000(	ADM5	19:496757	0.575659	2.88214	0	0.25363	0.283768	0	0.097773	0.536146	0	1.0384	0.65715	-1.07152
ENSG0000(	RP11-147	17:681898	0.375774	1.35716	0.258646	0.063473	0.085236	0	0.299997	0.65053	0.341419	0.9098	0.75005	0.002424
ENSG0000(	HNRNPA2	7:2618992	453.417	480.094	164.046	112.691	288.357	374.572	460.31	236.16	134.392	382.971	0.90445	0.06229
ENSG0000(	METAP1D	2:1717843	2.63785	5.93324	6.08291	15.6493	6.26071	2.368	1.06133	1.56979	0.01515	5.35745	0.1259	-2.01367
ENSG0000(	ANKS1A	6:3488926	1.94461	3.54193	0.573143	0.614472	1.35689	0.289446	1.99382	1.06588	0.521268	3.24842	0.6049	-0.30455
ENSG0000(	NEFL	8:2495095	0.124041	0	0.95673	1.38532	0.056511	0	0	0	0	0	0.00105	#NAME?
ENSG0000(	TRBV2	7:1423009	8.41867	38.3422	1.87266	5.38309	2.3351	22.2385	107.704	7.66516	19.325	1.81066	0.0207	1.53895
ENSG0000(	RP11-360	8:4152920	1.71515	0.826016	5.16544	5.49959	3.56078	5.14773	6.52727	5.84019	0	4.45117	0.9582	0.072131
ENSG0000(	RP13-39P	10:777828	1.61929	0.256995	5.04551	1.01733	1.90769	3.71416	0.533108	1.92796	0.319134	2.87017	0.93415	-0.17715
ENSG0000(	ADAMTS7	15:852553	3.21918	0.939545	4.60418	4.02162	1.32165	1.98138	4.02923	3.27343	0	0.295342	0.57575	-0.67952
ENSG0000(	COA3	17:427806	79.3743	54.2612	141.886	126.99	82.708	134.343	85.2066	94.9987	100.156	25.2196	0.5714	-0.2073
ENSG0000(	RP11-129	10:117239	0.197274	0.920881	0	0.281103	0.082076	0	0.025104	0.118782	1.69873	0.941083	0.6422	1.00465
ENSG0000(	STARD7	2:9618485	79.2326	89.4208	74.532	73.5728	49.0869	50.2833	54.7035	63.5394	38.2968	59.7262	0.11495	-0.46664
ENSG0000(	RPS15AP6	1:2080629	0.873753	0	0	0	0	2.60053	1.18415	0	0	0.28565	0.3761	2.48713
ENSG0000(	LSM4	19:183062	122.959	133.736	186.116	174.799	176.788	151.143	160.191	157.994	157.832	56.7529	0.40565	-0.30196
ENSG0000(	SPAST	2:3206361	5.36642	8.26158	1.36991	2.36326	1.84961	6.86044	4.0704	2.80195	0.165579	4.27242	0.9593	-0.02715
ENSG0000(	VHL	3:1014100	19.4407	18.4799	12.999	10.5313	13.9075	14.7	12.2527	10.0639	9.20016	22.7727	0.60965	-0.19472
ENSG0000(	FAM218A	4:1649544	0.434968	0.791626	0.012233	0.483267	0.120837	3.41097	0.881227	0	0	1.15725	0.4577	1.60015
ENSG0000(	PIGHP1	15:595948	1.06169	0.674499	0.87848	2.55948	0.080462	2.75934	1.72903	0	0	1.35271	0.9454	0.101825
ENSG0000(	BZW2	7:1664613	51.4867	51.4568	38.6355	38.9846	39.223	34.423	30.5156	21.9069	30.0738	32.0293	0.12065	-0.58353
ENSG0000(	THNSL1	10:249819	3.80767	5.69799	2.38491	2.31233	1.49262	0	1.37436	1.83305	0.408347	1.56917	0.33135	-1.52203
ENSG0000(	ACO2	22:414691	59.5499	59.2884	64.9521	52.5729	59.0483	43.26	37.5288	39.9211	46.1523	54.4623	0.1783	-0.44845
ENSG0000(	TAP2	6:3281276	7.50613	11.5083	2.53122	16.7754	4.28314	8.78916	22.0086	5.79465	21.2865	10.6425	0.23985	0.623901
ENSG0000(	RASGRP3	2:3343632	1.71688	4.60742	0.607734	3.76654	0.933144	4.64349	7.09974	3.24232	0.948313	1.51672	0.595	0.400593
ENSG0000(	RP11-290	11:620190	0.206372	0	0	0	0.085156	1.27652	0.107174	0.483219	0	0.471503	0.19285	3.12734
ENSG0000(	RNF4	4:2418973	62.1162	73.2653	56.1927	52.3373	64.6874	42.9337	64.3134	41.9545	19.1399	77.1154	0.18925	-0.43306
ENSG0000(	GATA3	10:805360	55.7417	68.3753	63.9552	55.696	56.3333	28.4739	64.2572	43.9304	46.0324	67.1817	0.3364	-0.30896
ENSG0000(	LY86	6:6346464	2.06331	12.1659	2.62746	6.35553	1.65556	18.6368	2.35463	2.23406	2.2452	0.824908	0.93895	0.062709
ENSG0000(	KLHL28	14:449243	15.3649	9.06948	31.7239	26.7111	23.2721	10.4134	5.39108	18.0917	20.1542	39.6336	0.616	-0.25442
ENSG0000(	SMCO4	11:934784	6.37982	14.4398	7.89485	9.39736	9.32982	85.2864	11.4025	42.7028	32.1221	8.22705	0.00215	1.84684
ENSG0000(	RP4-583P	20:637436	0.478929	0.159609	0	0	0.188542	0.624753	0.812404	0	3.38006	0.396285	0.1133	2.72067

ENSG0000(	VPREB3	22:237527	0	3.77706	4.36072	6.45086	0.821903	0	0	0	0	0.080282	0.23055	-7.40242
ENSG0000(	SLC1A3	5:3660635	0	0	0.009274	0.639262	0	6.64815	0	0.68508	0	0.313416	0.0265	3.34218
ENSG0000(	KDM4A-A	1:4365015	0.723871	1.7369	1.3947	0	0.143333	0	1.41999	0	0.774138	0.193727	0.51085	-0.84249
ENSG0000(	RNF138	18:320183	30.0136	56.4198	18.0348	27.7186	9.72054	18.555	27.0401	17.5004	36.7561	14.5114	0.60695	-0.22271
ENSG0000(	CPT2	1:5319642	13.4934	19.2853	17.7458	13.228	11.9086	6.81836	7.13151	10.5945	22.2819	13.7262	0.64115	-0.29923
ENSG0000(	RP11-331	9:3560530	0.789961	0.812439	1.8689	2.09019	0.363361	0.732936	0.173706	1.24118	0.247515	0.586071	0.50945	-0.98561
ENSG0000(	TRIM14	9:9805673	24.512	39.1898	34.1647	20.3735	47.6326	23.201	49.0933	30.4005	25.9808	97.0618	0.70235	0.198007
ENSG0000(	CTD-2301	5:1771330	0.253245	0.235482	0.671175	0.940415	0.187444	2.22017	0.925998	0	0	0.638428	0.7445	0.640477
ENSG0000(	OSTM1	6:1080414	43.4695	18.6486	14.4394	15.8	9.07525	59.4253	28.1938	18.1337	22.4266	21.1268	0.19595	0.473299
ENSG0000(	DKC1	X:1547627	34.3219	27.7984	56.3511	46.2648	58.1695	23.1805	31.2181	37.6046	9.38673	49.1813	0.08965	-0.55492
ENSG0000(	NPIPA8	16:183179	3.37336	8.79369	2.28252	1.52768	1.36035	4.39106	6.15122	2.76065	3.52698	3.47608	0.93735	-0.11342
ENSG0000(	MUT	6:4943035	8.84889	8.07102	5.00527	4.53839	3.11149	5.60006	4.66038	6.10967	8.06131	5.32977	0.92245	0.03512
ENSG0000(	RABIF	1:2028789	13.8092	11.6576	15.8246	15.8338	14.7874	17.3922	23.3617	25.6827	10.5304	10.4259	0.47355	0.267923
ENSG0000(	SARS2	19:389152	12.0457	13.7106	16.4542	13.6996	11.7738	17.7542	10.8466	8.06464	4.55858	9.13398	0.48365	-0.49668
ENSG0000(	WNT10B	12:489653	3.46347	1.77336	4.02251	1.58588	2.42241	0	1.04514	1.50633	0	7.07101	0.77655	-0.28778
ENSG0000(	HIST1H2B	6:2627297	0.171802	0.655994	1.21964	0.345506	0.13931	0	0.663256	0.155615	5.3054	0	0.36305	1.37097
ENSG0000(	TANC2	17:630095	7.87851	1.66001	3.6803	3.84419	4.54628	4.13501	2.06877	3.6678	8.46743	4.36472	0.9258	0.089565
ENSG0000(	GBE1	3:8148969	7.29917	11.3944	4.01227	5.13344	4.24635	4.31587	4.1321	3.9549	0.047606	3.97603	0.07235	-0.94886
ENSG0000(	R3HDM2	12:572434	7.84109	13.2227	14.4183	8.666	10.6009	4.06537	9.07373	17.5922	7.1576	20.9828	0.9431	-0.03115
ENSG0000(	AC022154	19:486153	0.648718	1.38567	0.24565	0.259268	0	1.5445	1.07065	0.360221	0.629394	0.10413	0.6123	0.753853
ENSG0000(	ETS1	11:128458	122.303	124.563	57.0867	42.8922	68.5035	57.4696	69.2481	61.113	32.6507	75.9852	0.23255	-0.53291
ENSG0000(	RBM5-AS:	3:4994000	0.687578	1.9379	1.98598	0.695078	1.13067	2.13547	2.26838	0.038191	0.670519	0.705182	0.83875	-0.26923
ENSG0000(	RP11-440	4:705747-	0.245414	0.375638	0	0	0.141439	0	0.271369	0	1.75648	0.341079	0.4148	1.89829
ENSG0000(	CHMP4B	20:338113	2.36171	0.333401	1.7369	2.74029	2.9911	2.71322	1.88495	1.52109	1.51927	2.28275	0.8463	-0.14107
ENSG0000(	RP1-8B1.4	6:4609709	0.651677	1.37232	1.78084	0.418769	0.772776	0	0.709045	0.852526	0	0.390871	0.37805	-1.3495
ENSG0000(	ZNF418	19:579218	3.71263	3.58361	0	0	0.178582	2.11003	0	0	0	0.720901	0.2785	-1.67115
ENSG0000(	XRRA1	11:747488	13.6621	31.8047	5.59624	9.03271	12.6107	17.9619	12.9542	5.19326	9.14625	20.8949	0.72655	-0.15313
ENSG0000(	RP11-254	2:1012073	0.449861	0	0	0.442316	0	0	0.848583	0.746073	0	0.425115	0.32985	1.79409
ENSG0000(	TCN2	22:305766	0.733239	1.73551	0.373238	0.121274	0.264504	2.74041	1.91595	1.62657	1.54233	0.35963	0.1635	1.41415
ENSG0000(	INO80B	2:7445502	4.13916	4.40429	4.20247	1.07301	3.85445	7.0708	6.80762	3.21771	0.616173	2.16229	0.89955	0.166861
ENSG0000(	ROR2	9:9156309	3.01387	0	0.085579	0.604021	0.047246	0	0	0	0	0	0.00205	#NAME?
ENSG0000(	ITGAM	16:312599	11.8738	8.79441	2.0688	5.30896	5.38937	3.09137	0.360221	0.33148	1.91327	1.46177	0.0033	-2.18431
ENSG0000(	RP11-480	5:8333480	0.330082	0.417331	0.726435	0	0.263865	0	0	0.277904	2.43351	0	0.64675	0.799683
ENSG0000(	XXYLT1	3:1950682	2.58945	5.54692	2.37801	4.26118	3.82666	0.23424	3.24344	5.41777	3.52342	5.3339	0.93795	-0.04978
ENSG0000(	ALG8	11:781009	34.0674	40.0492	25.4704	29.8033	27.4482	28.773	43.5961	25.2917	37.0853	41.5384	0.68075	0.14506
ENSG0000(	IGHV3-30	14:106335	0	3.30948	1.3827	224.412	0.802758	0	0	0	0	1.24577	0.0465	-7.04762
ENSG0000(	FANCI	15:892439	5.22076	14.8084	1.52587	2.81192	1.25608	10.2756	6.87496	6.15933	7.58499	2.83895	0.6984	0.302485
ENSG0000(	RP11-452	17:746705	27.6142	19.2251	35.9456	52.4894	24.1934	19.9234	19.2627	24.9911	31.1757	9.97805	0.5937	-0.63572
ENSG0000(	AK1	9:1278664	14.4532	7.32112	3.51686	9.49268	4.53316	28.615	8.67746	8.27161	9.19115	23.2502	0.3008	0.984958
ENSG0000(	TOR1AIP2	1:1798399	2.26854	3.06859	5.0678	4.40447	5.33908	0.611119	3.71782	4.8567	8.17708	9.35459	0.85055	0.093565
ENSG0000(	ANKRD37	4:1853960	11.5129	8.81711	24.9451	25.9557	20.753	12.9737	16.7287	32.2502	32.8279	14.2941	0.8702	0.109845
ENSG0000(	PLAT	8:4217523	0.247137	0.449009	0.4457	0.232175	0	4.17722	1.8518	0.41532	1.42489	3.08916	0.00895	3.07143
ENSG0000(	MSTO1	1:1556097	30.2525	42.9032	20.8065	36.6684	15.9903	34.2667	16.3416	11.2057	16.7406	21.5253	0.3433	-0.56995
ENSG0000(	WEE1	11:957368	11.6579	15.2388	5.38172	2.92873	6.05791	13.2553	14.121	8.99677	3.64073	9.1699	0.423	0.386691
ENSG0000(	CHMP2B	3:8722727	28.8138	23.0825	34.6831	18.9386	35.4309	37.9588	35.4086	25.6039	26.6962	23.2607	0.9258	-0.04062
ENSG0000(	SRP54-AS	14:349208	4.26748	5.03938	8.26248	7.50375	1.50436	5.7513	2.69012	8.80212	7.60941	5.51741	0.7717	0.29528
ENSG0000(	CTC-435M	19:412624	17.5641	0	7.61805	23.3297	4.6899	5.89575	0.545978	2.60385	10.4556	0.926159	0.4303	-0.98368
ENSG0000(	MYB	6:1351813	0.955591	2.55172	0.075926	0.077321	0.061349	0.992122	2.92562	1.73206	0	0.835673	0.31615	0.946617
ENSG0000(	RP11-775	15:809995	1.07899	0.80783	0.84781	0.306122	0.125091	0	0.249984	1.49274	0	0.1594	0.7788	-0.43386
ENSG0000(	RP11-603.	12:560798	2.68906	2.82044	0.792417	0.70227	2.72823	1.78865	2.79407	1.84184	0.368751	0.808009	0.81645	-0.28831
ENSG0000(	BRICD5	16:220424	3.11039	7.25856	0.680854	3.22615	1.00042	5.64013	2.60722	0.370266	1.86565	2.16934	0.79585	-0.33834
ENSG0000(	YARS2	12:326791	17.5495	17.5576	18.0432	18.9248	18.7832	11.6314	16.2352	25.6733	17.6735	14.474	0.8254	-0.13518
ENSG0000(	MZT1	13:727083	18.7064	10.5029	11.0816	17.0116	9.23143	16.5379	18.3018	13.8694	10.8897	7.86188	0.8555	0.067841
ENSG0000(	MESDC1	15:810009	3.02761	3.0028	1.82546	1.39217	3.9156	1.32374	2.24388	1.31877	2.51623	3.8878	0.6994	-0.22292
ENSG0000(	ORAI1	12:121626	104.374	99.9736	144.019	112.07	190.415	86.7016	86.8423	97.9335	57.379	58.5622	0.00615	-0.85573
ENSG0000(	GGA1	22:376084	22.9076	25.0244	29.9667	26.141	25.7718	22.1153	32.327	46.6569	15.469	42.5563	0.4127	0.296767
ENSG0000(	RP11-631	5:6133227	1.04458	0.995322	0.615906	0.604103	1.07577	3.08768	3.22674	1.08546	0.906934	1.67842	0.41965	1.22245
ENSG0000(	AC013461	2:1730754	3.29446	2.66002	2.20169	1.01381	1.82189	2.72887	4.16936	1.40536	0.033308	3.48679	0.8252	-0.16352
ENSG0000(	FOXP1	3:7095469	36.7859	115.227	30.966	24.0812	36.7765	38.8274	47.6176	21.4945	30.4752	45.6316	0.25525	-0.44567
ENSG0000(	ZC3HAV1	7:1390257	0.5183	0	0.322359	0.184326	0.023908	0.296237	0.704584	1.29087	2.13009	1.04291	0.20455	2.47494
ENSG0000(	MFSD7	4:672435-	0.714299	1.11571	0.039171	0.293304	0.784612	6.23172	1.17672	0.078344	0	0.423484	0.32585	1.2386
ENSG0000(	SWI5	9:1282558	16.6049	15.7889	28.3879	25.6012	25.6622	20.5294	10.0569	15.7382	12.4857	7.36686	0.23855	-0.80978
ENSG0000(	OCEL1	19:172262	14.299	40.5063	19.6894	25.5132	32.7047	30.794	27.0767	20.58	28.7134	25.9175	0.98725	0.007616
ENSG0000(	LARP1	5:1547129	9.46654	14.1487	12.4758	5.47398	37.6157	8.61241	14.4275	19.8043	4.82432	59.7306	0.53585	0.261928
ENSG0000(	ABHD12	20:252480	32.5482	34.4757	22.4006	30.2242	21.7007	52.5362	32.5982	25.9513	11.1609	21.6919	0.94225	-0.03416
ENSG0000(	PKD1P6	16:149745	5.41533	6.24536	0.814653	2.01096	1.26513	2.44302	1.44467	0.160703	1.36275	1.58528	0.30985	-1.06452
ENSG0000(	PRDX2	19:127630	184.48	188.844	327.051	280.722	344.981	182.778	161.157	194.284	270.867	81.6355	0.6805	-0.59791
ENSG0000(	ATP5I	4:672435-	180.93	115.27	285.755	292.175	68.9316	312.777	221.8	192.8	122.951	18.1959	0.5611	-0.22876
ENSG0000(	GARS	7:3049662	47.5187	59.4717	37.4595	44.6674	37.9514	50.508	60.2851	50.0753	35.4619	41.527	0.7159	0.139675
ENSG0000(	PPP1R12C	19:550909	14.5942	29.9319	11.1498	7.6721	23.191	18.7165	30.7805	17.8098	16.5238	32.5426	0.24955	0.552688
ENSG0000(	FBXW2	9:1207519	11.6746	15.7188	10.3653	17.5504	8.47203	6.47484	12.0292	13.6754	18.2661	13.787	0.73465	-0.19303
ENSG0000(	UQCC3	11:626468	10.2792	7.53996	12.3608	15.8026	11.7436	15.2128	7.463	11.74	9.07058	3.41737	0.815	-0.33234
ENSG0000(	KDM5D	Y:1970386	28.3812	46.5284	12.7393	10.0105	8.67737	31.6631	15.2495	23.3326	17.1739	30.1851	0.9474	-0.02299
ENSG0000(	ANKLE2	12:132725	36.6726	50.0923	17.5981	24.9707	14.9184	23.2028	35.1918	25.9049	17.6156	28.4143	0.5094	-0.21029
ENSG0000(	SMS	X:2194057	35.2902	43.0397	28.6982	22.498	24.2361	50.7297	53.1418	33.625	53.3744	11.3212	0.27375	0.37127
ENSG0000(	RP11-697	15:902495	0	0	0	0	0	0	0.248157	0	2.70387	0	0.16285	inf
ENSG0000(	SMIM7	19:164790	51.2697	41.3451	50.0134	53.4331	35.1319	99.7763	79.7261	61.8971	53.5436	34.3641	0.50655	0.363
ENSG0000(	AC009120	16:742967	7.214	6.78613	4.65335	4.15155	4.95061	12.9311	11.3063	4.11435	6.16206	8.0511	0.5159	0.505824
ENSG0000(	NR3C2	4:1480787	1.06225	1.56585	0.806506	0.664491	1.5349	0.030158	0	0.784322	4.75798	1.57755	0.6505	0.426504
ENSG0000(	RP11-589	14:527751	1.04091	0.567426	0	0.247759	0.429466	1.27244	1.07387	1.6996	0	0.344006	0.56065	0.723643
ENSG0000(	ZC3H6	2:1122755	1.4014	5.671	1.48708	0.880272	4.70909	0.852887	3.7637	1.04831	3.91407	2.79745	0.71705	-0.32129
ENSG0000(	CTC-273B	19:485132	1.24534	1.92421	0.466668	1.26331	0.362468	0	2.6887	0.31111	0	0.613548	0.73935	-0.54732
ENSG0000(	MZF1	19:585591	13.4279	17.0426	16.2473	18.6505	14.3902	8.56874	18.3738	11.5916	6.82103	30.5336	0.8429	-0.12295
ENSG0000(	ACSM3	16:206102	1.29975	0.408034	1.80137	2.70349	1.52281	0	0.115394	1.65908	4.55411	0.742927	0.7037	-0.37164
ENSG0000(	YTHDC1	4:6831038	27.7417	26.4341	31.4207	23.0214	49.4328	29.1162	36.7203	41.049	22.7433	57.0881	0.87165	-0.11423
ENSG0000(	ZW10	11:113733	8.86736	9.82715	6.43029	4.67306	4.20947	9.0234	6.52089	4.51627	7.82756	4.9283	0.8843	-0.06301
ENSG0000(	SLCO4A1	20:626407	9.53755	3.82901	0	0.999743	0.512869	25.3629	20.408	8.49024	6.77507	4.46804	0.0131	2.34321
ENSG0000(	ALDH1B1	9:3839266	2.88238	0.963567	4.83144	2.76001	4.76645	0	7.20699	5.0651	3.39203	3.25179	0.80845	0.192725
ENSG0000(	XKR6	8:1089604	5.62123	2.99239	2.96702	0.986247	3.28144	0.235375	2.21122	1.54806	1.55572	5.24745	0.719	-0.46333
ENSG0000(	TRAM1	8:7057344	191.433	117.277	73.0181	99.3185	57.434	113.691	131.369	92.6715	121.259	60.3789	0.76685	0.097917
ENSG0000(	ARHGAP2	5:1427703	21.2336	10.0266	7.24697	5.14057	19.4505	21.4546	16.6557	7.11174	5.61551	19.8307	0.98415	0.013648

ENSG0000(	HERC2P9	15:285894	8.82035	8.4798	3.84674	3.25637	2.92641	6.38393	3.174	5.34563	5.51146	6.59818	0.59405	0.351158
ENSG0000(	AP3D1	19:210098	19.3716	20.5635	30.6883	28.6696	40.3567	17.1749	24.7415	29.0153	17.1475	59.4062	0.9449	-0.03351
ENSG0000(	ZNF321P	19:528618	5.42719	1.8533	2.51989	4.66041	1.05181	8.93301	0.955472	1.9078	4.34912	1.76454	0.8654	0.249791
ENSG0000(	RP11-562.	15:504243	0.259465	0.602417	0	0.075252	0	0.353269	0	0	2.1337	0.160212	0.4778	1.66696
ENSG0000(	WDR1	4:1007433	177.994	265.457	143.377	132.637	170.102	182.419	220.78	163.464	123.62	226.941	0.7786	-0.09045
ENSG0000(	TRIM46	1:1551694	1.14793	2.68734	0.462773	0.389178	0.973711	0	1.56747	2.30625	2.68845	2.65525	0.45095	0.785614
ENSG0000(	LARS2	3:4538850	6.24638	6.3571	1.53002	2.58033	1.24977	4.80466	3.88026	0.642702	2.59966	3.20958	0.5598	-0.32764
ENSG0000(	ISG20	15:886361	239.068	271.849	460.36	565.524	367.373	304.291	889.369	619.638	342.24	98.3199	0.81295	0.102843
ENSG0000(	TRBV14	7:1425878	2.29856	9.67621	2.67652	3.00942	1.22274	17.6608	3.91594	2.17358	0	1.41872	0.84805	0.41687
ENSG0000(	SRRD	22:264838	9.58157	13.2243	8.72008	4.43089	8.64393	14.8301	9.99248	8.83236	6.36743	10.9272	0.7904	-0.2128
ENSG0000(	FMNL1	17:451909	52.0865	72.1972	25.9337	20.5834	65.4185	96.0831	94.8456	46.7429	25.1552	99.2147	0.21575	0.631868
ENSG0000(	MTMR1	X:1506929	14.1802	20.0952	3.09505	2.16664	4.47091	5.02736	13.4054	2.54644	0.886399	7.48779	0.21175	-0.54729
ENSG0000(	PGAP3	17:396711	15.0813	17.8159	8.68426	8.65144	10.3414	9.94946	9.45543	3.43618	0.026813	10.4923	0.37245	-0.84202
ENSG0000(	ARHGAP9	12:574601	159.263	293.306	91.7442	130.868	89.8188	241.8	267.512	120.73	172.885	184.327	0.44665	0.384868
ENSG0000(	KCTD13	16:299003	18.6412	26.7127	21.5934	21.6072	21.3815	22.4081	24.1416	20.2188	43.1525	31.9966	0.361	0.384969
ENSG0000(	RNF19A	8:1001579	24.499	21.8239	14.6533	24.8032	9.94302	35.8543	44.6718	38.7648	44.891	31.4758	0.00915	0.889719
ENSG0000(	IFIT3	10:892135	1.48085	7.5177	2.64426	5.01712	1.98313	12.0942	145.128	79.3958	18.6919	16.4045	0.01145	3.86895
ENSG0000(	TFR2	7:1006204	2.08496	1.48836	1.47608	0.961962	2.10538	1.32826	1.25021	0.26157	2.05937	3.12848	0.74125	0.231826
ENSG0000(	ABI2	2:2033282	4.4214	7.63828	3.438	3.05436	2.57592	8.16263	6.64929	4.8935	2.52742	5.29581	0.46125	0.355102
ENSG0000(	RMND5B	5:1781309	9.6787	30.0455	5.04595	6.0522	5.52586	8.86375	7.24468	5.96814	7.31126	5.22231	0.43885	-0.71267
ENSG0000(	TXNDC17	17:657814	73.6692	47.3484	72.4306	66.0388	34.9024	152.7	149.562	99.6659	55.9007	14.6814	0.55005	0.574507
ENSG0000(	CFAP36	2:5551960	17.3024	14.5209	19.5784	12.734	18.7898	12.8634	11.153	20.5375	24.8537	8.53901	0.9812	0.014451
ENSG0000(	RP11-642.	8:1405201	1.19954	0.542534	0.827016	4.99856	0.653673	1.58592	4.23039	1.85446	9.55757	0.64667	0.5066	1.18371
ENSG0000(	RP11-161	12:493665	0.270544	0.781294	0	0	0	3.92032	0.098991	0	0	0	0.4648	1.93897
ENSG0000(	ARHGAP1	15:306169	5.83463	12.3465	2.91243	1.13521	0.831405	21.7805	23.3396	2.09959	8.42801	7.81028	0.01635	1.57892
ENSG0000(	SPOP	17:495988	68.8025	98.6898	72.4371	57.4482	89.7245	83.3662	78.2022	62.7818	56.2255	70.8341	0.46925	-0.2238
ENSG0000(	RAB43	3:1290875	2.25693	3.72526	2.1682	3.33762	2.1366	2.38733	1.83879	2.19365	0.109056	1.212	0.26705	-1.08679
ENSG0000(	RP3-402G	22:501859	3.12332	7.67036	2.20673	6.7623	1.24507	4.40317	4.62384	1.32926	0.09369	1.94133	0.5164	-0.92342
ENSG0000(	TOP1	20:410288	15.4365	14.4906	12.7101	10.298	34.4384	20.8059	34.1429	17.4	9.53649	31.2894	0.65905	0.240884
ENSG0000(	SAMHD1	20:368761	92.0092	98.9802	29.7472	43.1761	45.2561	70.4515	58.2537	24.8632	33.5374	44.9944	0.1741	-0.43591
ENSG0000(	PLCH2	1:2425979	7.14194	3.66999	0.453195	0.841314	1.46451	9.09175	3.81617	1.79897	0	5.53134	0.5746	0.593969
ENSG0000(	ACTR5	20:387484	8.3966	4.1052	10.6289	9.04049	6.20949	3.90753	8.12182	7.63984	10.435	9.37168	0.97815	-0.01223
ENSG0000(	RN7SL809	12:566633	0.232139	0.145284	0.321094	0.860963	0	0	0.172064	0.183055	0	0	0.42795	-1.90801
ENSG0000(	ZHX1	8:1232199	9.89245	10.6279	5.36313	6.67439	6.9831	9.67169	11.7739	8.73813	17.175	8.73814	0.40485	0.502562
ENSG0000(	PRSS53	16:310744	0.194963	0.344688	0.037138	0.117373	0.659416	0.230686	2.13207	0.408332	0.815907	0.255582	0.4295	1.09851
ENSG0000(	ZNF12	7:6663973	8.25066	10.518	4.36989	3.95287	4.30405	7.52243	10.786	9.40002	2.76183	15.2565	0.77295	0.116026
ENSG0000(	ACADVL	17:718988	257.15	251.742	144.534	197.423	126.387	375.647	295.375	159.521	155.59	153.989	0.41955	0.267499
ENSG0000(	RP11-573	3:1867817	1.39693	1.77898	0.294483	0.822771	0.14109	0.047494	0.096279	0.310946	0	0.070051	0.0816	-2.87201
ENSG0000(	DNAAF1	16:841452	2.37823	1.78722	0.187197	1.4604	1.50167	9.92838	0.868198	2.91927	6.70758	1.31596	0.02145	3.76841
ENSG0000(	CLASP2	3:3349624	9.131	9.06164	2.24604	2.79189	1.85338	6.85314	4.53177	2.27243	6.61387	5.37933	0.6706	-0.17217
ENSG0000(	IDH3G	X:1537857	64.6731	71.7328	122.275	102.148	148.164	82.4483	72.5451	105.93	97.6227	88.7231	0.6869	-0.23833
ENSG0000(	UBA52	19:185717	335.506	349.542	553.984	427.017	325.99	394.182	386.613	395.883	367.667	66.1347	0.5376	-0.3499
ENSG0000(	TPT1-AS1	13:453334	9.84551	10.1859	15.257	13.0924	7.6159	6.91333	7.92467	13.617	2.41683	9.59372	0.4202	-0.55191
ENSG0000(	REC8	14:241718	38.1708	27.0904	9.70826	11.7212	18.4694	35.4333	28.5893	15.5313	7.23439	34.1563	0.83565	0.162132
ENSG0000(	THEM4	1:1518735	40.8103	70.3221	64.96	63.5081	56.1424	38.7299	17.8547	52.3172	50.8813	50.3185	0.2989	-0.43098
ENSG0000(	SULF2	20:475019	1.60284	16.7959	0.778716	0.57142	0.032971	13.6664	0	0.571853	0.325316	0.011462	0.87295	-0.27123
ENSG0000(	LUZP6	7:1359264	0	0.679707	0.947787	0	0	0	0	0.242655	0.146835	0.625732	0.5543	0.723661
ENSG0000(	TRUB2	9:1283051	8.27278	6.89326	16.5798	15.7601	12.1608	6.35145	7.8511	9.67099	10.9719	6.19604	0.18565	-0.63121
ENSG0000(	CALM1	14:903965	1208.53	1008.97	1825.92	1549.27	2795.21	1270.3	1402.68	1170.83	1105.44	763.931	0.30065	-0.76334
ENSG0000(	UXT	X:4765179	102.134	88.884	119.383	111.353	64.713	116.282	137.434	118.447	81.8371	17.7743	0.8071	-0.08669
ENSG0000(	EDF1	9:1368621	300.544	216.816	352.655	301.307	447.133	422.385	500.714	359.795	273.11	129.713	0.87415	-0.05808
ENSG0000(	RNF115	1:1457194	5.87808	5.00057	9.9605	7.67093	6.17186	3.25042	4.7713	9.03338	7.57358	14.1728	0.43905	-0.46117
ENSG0000(	KDM4B	19:496911	8.13927	9.91327	2.81217	3.52001	3.02749	4.33325	6.05763	1.80634	4.3836	6.39677	0.5094	-0.32627
ENSG0000(	PDZK1	1:1456708	0.396715	0.869941	0.626003	0.970525	0.423102	0.318199	0.030452	0.105897	0	0.145578	0.0247	-4.03843
ENSG0000(	STK35	20:210161	3.49357	3.6802	2.28184	4.5257	2.20978	1.04022	2.82913	2.82013	1.39856	5.05192	0.2318	-0.73873
ENSG0000(	NUDT9	4:8742258	16.7013	14.621	23.2639	19.3143	23.9519	10.252	9.85541	15.9086	30.5657	16.7542	0.5259	-0.26117
ENSG0000(	TP73-AS1	1:3630766	7.53021	14.6229	6.22798	4.28151	6.09742	6.97769	6.98106	3.90107	0	7.99025	0.3349	-0.71211
ENSG0000(	YIPF1	1:5385171	27.6614	22.8937	14.7407	18.8205	19.2734	38.7272	32.2854	27.6581	27.1252	20.2371	0.2222	0.464303
ENSG0000(	IGHE	14:105597	3.08787	0.991339	0	0	0.382114	0	0	0	0	0.109687	0.1236	-5.35421
ENSG0000(	NOL12	22:376816	57.9342	56.4909	83.2483	63.0031	78.626	61.9096	65.7003	71.1076	64.7787	47.0133	0.75555	-0.17613
ENSG0000(	ZNF90	19:200779	0.564646	2.12049	1.40554	0	0.740799	0.442893	0.376809	0.463936	0	1.45739	0.3437	-0.8617
ENSG0000(	TRAF6	11:364870	4.61368	4.15623	8.37883	6.88894	4.56501	0.927002	2.34715	8.43614	3.66044	8.87188	0.09625	-0.78173
ENSG0000(	COX7CP1	13:489749	0	1.58988	1.6301	1.92316	1.60761	0.55724	0.989798	0	0	0	0.20765	-2.51488
ENSG0000(	LMBR1	7:1564721	16.6898	22.1812	9.92817	7.29919	5.32238	17.3889	28.746	11.6368	8.02057	12.45	0.733	0.17585
ENSG0000(	DFFA	1:1045652	15.0328	15.6584	21.7705	17.6891	18.6004	9.77681	17.3564	17.0741	15.5049	21.4312	0.5288	-0.28256
ENSG0000(	RP11-323	17:788529	0	0	0	1.59411	0	3.46414	0	0	1.49393	0	0.74115	1.29097
ENSG0000(	RPL17	18:494772	353.906	236.094	748.024	582.6	210.083	334.764	356.568	509.243	379.92	64.9506	0.34515	-0.46768
ENSG0000(	APOBEC3I	22:389527	4.7812	4.53139	0.36535	0.362528	1.08638	0.296954	2.73042	1.06088	0.103759	1.31434	0.55635	-1.05263
ENSG0000(	SCART1	10:133453	13.7924	2.28321	6.40456	7.12971	6.50269	7.70284	0.747188	5.33171	6.7336	17.4725	0.68595	-0.20812
ENSG0000(	MTRF1	13:411329	18.7209	22.7628	10.1459	11.5485	7.314	26.4154	20.4956	9.22696	2.45496	10.893	0.8503	-0.12536
ENSG0000(	CCT3	1:1563089	120.902	111.853	135.632	128.214	124.285	85.5664	103.573	94.8055	76.0142	71.078	0.07765	-0.54206
ENSG0000(	RP11-92F.	2:2244646	2.03308	1.27467	1.77215	1.45548	0.807206	0.426224	1.08666	1.74184	1.17303	0.917734	0.75185	-0.45628
ENSG0000(	PKI55	2:2162170	0.959435	1.43814	0.384144	0.124558	0.352431	0.994279	0.177575	0.253761	0	0.395627	0.5038	-0.82596
ENSG0000(	SP1	12:533801	2.1428	3.771	1.84798	0.846949	2.49544	3.73266	3.95084	5.57929	0.027019	7.05429	0.08815	0.728552
ENSG0000(	RUFY3	4:7070374	7.5563	15.1866	2.90552	0.652998	2.42659	5.6501	3.63992	5.34286	0.282819	3.61816	0.46165	-0.58029
ENSG0000(	TAPBP	6:3329969	151.757	203.064	220.767	136.379	222.055	135.862	162.335	243.09	146.383	339.368	0.87885	-0.05523
ENSG0000(	DDRGK1	20:319034	46.0276	39.466	39.8581	30.9357	78.572	103.262	82.0428	52.3781	34.5438	52.3175	0.2761	0.403205
ENSG0000(	TANGO2	22:200170	22.1807	19.8259	27.9338	36.4739	22.3532	21.5149	22.9531	24.7094	31.2408	35.1863	0.866	0.058045
ENSG0000(	DHDDS	1:2643228	14.0423	12.144	10.7327	8.28737	6.8942	18.1447	23.1178	10.7439	13.6172	13.3456	0.26075	0.519806
ENSG0000(	RP4-539M	22:304355	0.576527	0.137583	2.18859	5.12282	1.00708	0	0	0.720528	0.306742	0.278874	0.3174	-2.92007
ENSG0000(	FHL1	X:1361467	2.7023	4.75718	11.2005	34.7464	44.6929	4.70505	0.433054	3.25849	1.65031	23.1015	0.0028	-1.81315
ENSG0000(	MFSD11	17:767268	18.063	20.8812	12.615	22.4473	9.58639	30.0163	20.3306	11.8352	15.6237	14.5076	0.8499	0.099948
ENSG0000(	CTD-3214	19:767662	17.0711	22.9033	0	0	0.733803	56.259	14.5598	15.6501	46.3318	0.022979	0.29115	7.66615
ENSG0000(	TRAV8-6	14:219784	11.1843	31.2929	8.99146	9.76831	2.28468	18.1748	39.5683	8.21944	3.22138	6.71807	0.73125	0.226745
ENSG0000(	DOCK5	8:2518472	0.196167	1.49694	0.126445	0.075631	0.035564	0.485868	4.27845	0.555234	0.545596	0.93059	0.02465	2.00928
ENSG0000(	POLE	12:132623	5.67318	14.314	0.553453	0.438464	2.98444	7.68638	7.33165	1.35109	3.8764	6.2619	0.47355	0.418091
ENSG0000(	AC007365	2:6463162	0.793043	0.460199	0	0.523252	0	1.55058	0.451649	0.090116	0.331369	0.103605	0.79455	0.510419
ENSG0000(	NAT6	3:5027902	8.88372	10.4482	15.5394	15.7632	11.3982	2.42776	7.15756	9.12135	5.12865	11.2149	0.265	-0.79975
ENSG0000(	NDUFA1	X:1198704	257.207	140.745	532.581	475.907	174.571	324.325	300.906	430.882	345.873	65.3231	0.7262	-0.16323
ENSG0000(	USP1	1:6243629	26.3879	32.7324	20.8039	15.1611	31.9214	38.5007	29.4056	20.3489	24.7878	38.2335	0.55515	0.206047
ENSG0000(	CTB-79E8	5:1726412	2.18009	0	0	3.72727	0	5.78244	4.36048	5.0081	2.30664	0	0.4528	1.83467

ENSG0000(	AGPAT4-I	6:1611299	1.78177	3.22192	0.402465	0.125325	0.674204	2.75594	1.04847	1.46536	0	2.3522	0.8454	0.317053
ENSG0000(	RP11-378.	2:2182553	0.497377	0.319388	0.54133	0.520166	0.691633	0.12521	0.288414	1.56959	0	1.63847	0.8146	0.367708
ENSG0000(	RP11-770.	11:118436	0.519787	0	0.771752	0	0.300991	2.52271	0	0.419997	0	0.258005	0.7946	0.497746
ENSG0000(	RP11-206I	1:585988-	3.69453	2.51547	0.448287	2.52221	0.319013	2.95897	2.18451	1.2228	0.398728	0.419081	0.6498	-0.69507
ENSG0000(	TNKS	8:9555143	4.91192	7.9867	3.05768	6.45264	2.30451	5.60378	2.0187	1.85155	11.0882	8.6527	0.8444	0.130261
ENSG0000(	LIG1	19:481154	23.2817	47.8035	18.3313	19.2769	18.7246	26.1321	32.7308	24.0707	22.8403	27.5912	0.2705	0.40222
ENSG0000(	TIPIN	15:663362	7.85623	8.2166	5.88215	5.34526	4.0054	15.3276	12.5729	14.4254	11.4735	3.33819	0.1159	0.908931
ENSG0000(	C14orf159	14:908478	55.8104	44.8752	35.9008	51.2933	27.0254	42.7612	52.2173	38.658	30.8465	34.3476	0.5428	-0.20063
ENSG0000(	WASH6P	X:1559975	1.91626	2.15728	2.54378	2.14405	2.11398	5.54864	2.74372	9.75934	1.89387	2.59754	0.05575	1.32133
ENSG0000(	RP11-432I	3:1602256	0.219854	0.665848	0	0	1.13581	2.32432	1.4994	0.746673	0	0.734616	0.36225	-1.33683
ENSG0000(	STN1	10:103882	77.5496	29.7845	78.3736	108.604	60.6406	58.6463	50.6101	47.7772	77.3238	35.2483	0.26555	-0.40075
ENSG0000(	MCOLN1	19:752262	7.14515	11.3977	4.43072	4.24101	8.3408	15.3056	6.14609	7.51	8.03611	7.16781	0.7309	0.282495
ENSG0000(	RP11-58K.	11:445644	0	0.607896	0	0	0	1.3665	0.848118	0.078765	0	0.182041	0.37915	2.10002
ENSG0000(	CLDND2	19:513451	1.64797	1.05616	2.14216	1.85148	1.22893	2.01088	3.93423	3.57594	1.8531	1.2056	0.5131	0.635391
ENSG0000(	PRKAR1B	7:549196-	9.66115	15.4689	6.60733	13.3013	9.02138	3.30241	13.6274	15.7415	9.2589	11.2443	0.7119	-0.23253
ENSG0000(	CSGALNA(	10:431384	26.212	34.3638	9.68639	17.9016	6.76084	29.735	15.7433	20.3558	25.2561	16.4134	0.50035	0.233724
ENSG0000(	ABALON	20:316644	1.68763	1.4868	1.86513	1.76118	3.24016	1.18789	3.92784	0.232252	5.16844	1.37471	0.9172	0.2377
ENSG0000(	IGLV2-8	22:228226	0	23.36	1.16356	0.545553	0.285216	0	0	0	0	0.183493	0.25785	-6.38804
ENSG0000(	LINC0027(	Y:3002911	0.408579	2.22856	0.955398	1.50087	0.419626	0.528756	1.13811	0.74761	0	1.22146	0.7704	-0.44744
ENSG0000(	GTF2IRD1I	7:6662888	1.16497	0.842926	0	0.071842	0	0.479796	0.156883	0.318126	0	1.36916	0.32165	1.60968
ENSG0000(	SLC39A1	1:1539590	22.7813	25.9426	24.7892	20.7264	22.0625	43.4151	50.7456	31.7183	56.0069	31.5033	0.38215	0.864765
ENSG0000(	ZNF195	11:333672	17.3323	15.4747	14.3663	16.9808	12.5466	16.9818	16.4109	10.0773	19.1508	23.5521	0.85915	0.060532
ENSG0000(	CTD-2537	19:556541	1.29011	1.20348	0.106626	0.419011	0.745462	0	0.109817	0.120935	0	1.90059	0.8449	-0.39654
ENSG0000(	NELFA	4:1982713	22.0127	43.7579	16.6488	19.7549	24.8639	26.1563	23.9564	20.4871	22.6502	23.0728	0.71495	-0.13835
ENSG0000(	TMEM99	17:408181	20.266	12.9432	16.9794	9.48873	12.1796	33.725	5.72491	9.00315	10.5773	5.59425	0.8931	-0.20321
ENSG0000(	PTER	10:164369	10.2533	4.71949	9.24423	9.86018	6.09234	9.9092	16.7353	8.67915	8.36893	10.9867	0.758	0.158462
ENSG0000(	RP1-68D1	11:351388	1.03627	1.50631	0.203492	0.091575	0.071675	0.511019	2.93004	0.397513	0	0.65256	0.5763	0.890788
ENSG0000(	COQ10A	12:562668	26.4365	32.2266	34.1626	34.4564	19.952	5.70985	11.2689	15.6252	14.3919	22.7154	0.0164	-1.0881
ENSG0000(	CTD-2288	5:5267519	0	0	0	0	0	2.94068	3.76443	0	5.47178	0.31063	0.11375	inf
ENSG0000(	PNP	14:204689	87.3687	30.269	132.27	125.619	132.77	77.3343	34.6084	80.2726	136.285	88.2562	0.3812	-0.30871
ENSG0000(	ALCAM	3:1053669	1.92281	1.41122	0.578843	1.24151	0.926133	10.3684	0.60571	0.34317	0.139306	1.61205	0.36655	0.742207
ENSG0000(	RP11-163I	7:1023638	4.01092	6.14134	4.25463	3.23031	2.51511	0.696792	3.95788	0.784023	1.82245	5.22184	0.6841	-0.41947
ENSG0000(	AC004951	7:4392643	0.803733	0.363746	0.0338	0.147875	0.188523	0.341186	1.39502	0.082129	1.0426	0.368467	0.4705	1.37604
ENSG0000(	PRKCQ-A!	10:658041	104.158	160.775	98.6851	121.191	103.771	106.202	104.77	97.8838	89.1375	58.247	0.2712	-0.38288
ENSG0000(	DDTL	22:239669	4.88897	4.34984	3.12081	5.32583	1.72433	4.29138	9.22907	3.66991	8.06131	5.78993	0.5518	0.907291
ENSG0000(	RP11-705(	18:268147	0.844324	0.832656	0.232054	0	0.16217	0	0.523802	0.439487	4.05134	0.14601	0.56445	1.55969
ENSG0000(	NANP	20:256129	0.871459	0.97287	0.535143	0.848302	0.608266	0.31357	0.241976	1.06733	1.02087	0.774147	0.94345	-0.13769
ENSG0000(	ORC3	6:8759006	24.0909	19.0681	15.5054	18.7042	9.94676	31.6062	20.6008	10.1558	8.3447	12.9438	0.8717	-0.07936
ENSG0000(	NUDT3	6:3424637	6.41936	5.98718	4.06205	1.58548	4.93655	6.60732	7.57322	5.55687	4.49395	6.84317	0.8832	0.181769
ENSG0000(	NRDE2	14:902564	6.20621	5.64215	6.7294	8.46982	6.54579	10.3952	11.7215	3.00486	3.50874	9.03658	0.94395	-0.05863
ENSG0000(	SLC35F6	2:2676428	3.50013	1.52489	12.4703	5.90417	8.96054	5.12076	3.03896	13.2033	10.2381	14.2865	0.4709	0.385916
ENSG0000(	ANAPC11	17:818878	126.904	83.5903	199.378	190.777	104.079	188.848	190.488	151.368	131.936	40.8744	0.81185	-0.13854
ENSG0000(	ZNF337	20:256129	7.98939	11.7705	7.99237	5.0272	5.32203	6.98652	9.10804	4.57575	5.18465	11.5846	0.4199	-0.37308
ENSG0000(	NCOA2	8:7010976	4.70822	6.40207	3.99407	2.79778	3.32143	4.45431	7.15974	4.97718	1.39994	9.6766	0.50915	0.261488
ENSG0000(	AL109767.	14:991692	1.02455	0.466058	0.335481	0.750585	0	0.092655	0.631683	0.263145	0.111143	0.099914	0.5561	-1.04684
ENSG0000(	RP11-305I	3:1560377	0.224481	1.25883	0.534515	1.03889	0.680939	0	0.239655	0	2.82849	0.871762	0.92315	0.197102
ENSG0000(	DGKD	2:2333545	14.4433	21.965	7.90309	7.56278	9.71759	7.53444	11.4108	7.30611	7.15166	11.9718	0.24985	-0.40383
ENSG0000(	STIL	1:4725013	1.38331	2.77816	0.026974	0	0.208832	3.01679	2.64174	0.057272	0.011633	1.09041	0.7182	0.386182
ENSG0000(	IFT27	22:367582	9.42345	5.27964	22.1046	12.5939	17.6337	12.9164	8.27925	18.7884	8.94843	20.022	0.8774	0.084854
ENSG0000(	CTD-2245	19:531971	1.81709	1.17835	0	0.172492	0.214965	1.50311	0.016167	0	0.146158	0.230286	0.7705	0.410154
ENSG0000(	GPR141	7:3768379	0	0.624944	0	0.082288	0	4.43865	0	0	0	0	0.2788	3.03185
ENSG0000(	TRBV18	7:1426157	7.85732	22.0007	3.43738	8.08757	1.03288	42.0395	8.56832	4.99763	17.7751	9.23446	0.1727	1.13869
ENSG0000(	TRIM7	5:1811919	0.760023	0	0.322756	1.54528	1.69396	2.95731	0	0.15632	1.87383	2.50585	0.5746	0.797554
ENSG0000(	RBM12	20:356260	12.8743	13.35	5.16717	2.73423	5.12986	8.83334	9.67253	9.62625	1.88612	10.4614	0.7819	-0.28803
ENSG0000(	HLA-G	6:2982696	0	0	6.6245	0	8.26621	0	0.080047	8.41004	0.041641	6.85781	0.9919	-0.01069
ENSG0000(	S1PR5	19:105127	201.739	34.1066	422.937	298.775	452.584	11.9628	0	66.6725	39.654	85.4336	0.0026	-2.77284
ENSG0000(	PLCB3	11:642515	0.559707	0.439415	0.503632	0.326263	1.00446	0.933506	0.377075	0.897878	0.084319	1.95663	0.6258	0.450976
ENSG0000(	LINC0099(	7:1504336	0.42894	0.190139	0.666762	0.347226	0.241976	1.14887	2.56964	0.239726	3.14126	0.13913	0.31665	1.90373
ENSG0000(	DECR1	8:8993333	106.635	99.3902	106.186	104.146	86.6221	88.8773	80.3666	68.3494	134.328	40.1259	0.6116	-0.22985
ENSG0000(	C22orf34	22:494145	2.55552	2.72207	3.17566	1.31397	2.0048	2.74983	1.67737	0.931966	2.85402	1.03713	0.766	-0.30092
ENSG0000(	RP5-1074I	1:1103471	0.443282	0.806658	0.243316	0.117538	0.173647	0.114551	2.88454	0.49297	0	3.31044	0.36145	1.86664
ENSG0000(	RP11-488I	1:2471898	10.9895	5.19072	6.78415	4.37326	6.57754	12.1886	9.95424	7.66048	2.28039	13.2708	0.65495	0.340138
ENSG0000(	RBM26	13:793118	12.0373	18.8229	5.44539	4.37082	7.59378	20.5835	13.127	13.006	8.353	21.7836	0.15165	0.585226
ENSG0000(	KRT73	12:525855	2.5588	2.86085	0.834273	0.676005	0.276578	0	0	0	0	0	0.11965	#NAME?
ENSG0000(	AACS	12:125065	4.03062	5.14558	3.38661	1.70186	2.93471	5.96096	5.64566	2.58358	7.89407	2.81751	0.30725	0.549405
ENSG0000(	CCDC9	19:472559	9.38145	9.16782	10.1969	6.51457	11.81	16.8828	19.301	16.4414	16.5679	10.5924	0.09545	0.74889
ENSG0000(	RP11-319(	3:1393898	0.785089	1.57137	1.12533	0.638764	1.12221	5.79073	0.274771	2.31077	3.30984	0.646642	0.1187	1.40595
ENSG0000(	HIST1H3D	6:2619555	2.03466	0.398681	0	0	0	0	0.795352	0	0.114711	0.671554	0.46725	-2.24441
ENSG0000(	U2AF2	19:556541	6.93725	10.8635	18.6329	21.1907	59.6152	6.93266	13.0117	23.5192	10.9028	38.1556	0.53535	-0.32198
ENSG0000(	RP11-393I	6:8715115	0.809348	0.813125	0.225442	0.398118	0.215907	0.912298	1.32182	0.377987	0.918622	0.864113	0.56775	0.830165
ENSG0000(	TREM2	6:4115850	0	0.030381	0	0	0.050206	10.0957	1.74832	0.387869	3.55372	0	0.2856	7.44521
ENSG0000(	ARSB	5:7877720	6.36227	1.38729	4.31261	6.79287	4.76643	10.8036	7.40829	2.7974	0.503079	4.92205	0.76455	-0.22361
ENSG0000(	TULP4	6:1582322	17.9467	21.0276	8.91787	10.1313	19.1934	20.237	21.3605	8.8377	6.30689	22.156	0.96915	0.028992
ENSG0000(	XXyac-YX:	1:1479938	0	0.763727	0	0	0.108589	0	1.21174	1.06617	0	0.153109	0.4161	2.04306
ENSG0000(	PIEZO1	16:887064	56.8667	43.7717	18.5249	28.3457	22.5835	51.8507	38.5728	24.5005	44.8226	28.7906	0.31425	0.584523
ENSG0000(	TMEM9B	11:894729	114.029	67.0412	96.0359	77.4972	67.1222	86.6355	111.044	78.1212	67.0583	40.1866	0.5882	-0.18825
ENSG0000(	HDAC6	X:4880187	18.0702	34.4973	5.26222	12.6541	4.17057	18.1264	11.9162	7.09075	6.18291	8.90391	0.14445	-0.49928
ENSG0000(	HMGCL	1:2380188	27.3418	32.8643	31.9711	39.1403	22.6985	46.8144	20.8839	25.025	32.8219	22.8536	0.8894	-0.05212
ENSG0000(	ABT1	6:2659695	30.6339	34.7907	54.6168	54.6459	48.7404	51.3849	44.8368	37.9562	32.6541	17.322	0.3296	-0.32545
ENSG0000(	GUK1	1:2281399	438.301	254.763	657.727	650.471	464.534	523.179	549.436	524.058	467.032	155.553	0.42395	-0.25625
ENSG0000(	USP4	3:4926860	49.7113	53.3216	34.8398	32.4704	27.6367	52.2226	42.948	32.075	17.4885	45.3355	0.84495	-0.06268
ENSG0000(	FAM156A	X:5292640	6.48277	15.1303	7.83788	10.707	5.40533	12.2634	7.04419	6.44509	3.34182	10.1847	0.78525	0.202226
ENSG0000(	TDRKH	1:1517555	14.2632	7.64019	11.8126	6.81673	9.06281	10.5362	8.40251	6.75092	12.8267	9.80411	0.85815	-0.11848
ENSG0000(	XPO1	2:6147118	30.306	29.1651	9.94835	7.72411	12.1211	20.1356	24.0151	9.76807	7.17818	25.3215	0.94325	0.024738
ENSG0000(	YIPF2	19:108715	5.71074	4.0347	9.50061	13.5428	12.8306	8.42268	11.1783	18.5819	10.2374	9.75086	0.6468	0.248717
ENSG0000(	SCD	10:100347	9.69457	2.17588	3.55833	2.92777	2.53269	5.72276	2.96239	6.91403	2.21835	6.43739	0.70285	0.163577
ENSG0000(	RPS24	10:780337	445.944	287.843	726.079	580.306	440.579	373.387	474.258	474.701	411.344	85.569	0.18095	-0.47333
ENSG0000(	RBM14-RI	11:666165	10.9113	6.51737	7.73435	11.3605	8.11679	16.0828	6.88334	7.4629	8.12356	6.47273	0.97605	-0.02949
ENSG0000(	DERA	12:159111	31.3391	27.7715	20.013	21.4116	15.6356	41.918	28.1057	18.6559	12.2209	10.3253	0.8701	-0.06315
ENSG0000(	TAF3	10:781850	14.724	24.4058	9.83321	12.0747	12.2965	7.05045	14.4724	6.28019	6.95639	15.0986	0.0765	-0.6103
ENSG0000(	FEM1A	19:479168	2.70006	2.30432	1.04185	1.76519	0.711051	2.2007	1.48071	0.569831	1.90449	1.36732	0.94855	0.083128

ENSG0000C	PPDPF	20:635207	13.3863	12.8956	3.24194	6.99292	9.98548	10.5545	15.4706	5.58195	5.99783	8.10035	0.9402	-0.05166
ENSG0000C	RP11-647	2:1904083	0.245148	0	0.345636	0.825912	0.46012	0	0.812141	1.22927	0	0.31382	0.7685	0.48809
ENSG0000C	NLK	17:280417	2.13571	1.62923	1.79522	1.89488	2.03939	0.011045	1.88434	2.36326	0	4.61263	0.9068	-0.07863
ENSG0000C	CTD-2162	19:367731	0.260244	0	0.449794	0.559759	0.54741	1.23192	0	0.669555	1.85587	0.160017	0.5386	0.988924
ENSG0000C	FCGR3A	1:1615054	9.27845	64.4199	24.2692	31.5668	14.9654	87.9842	4.04039	4.02056	2.46494	3.78772	0.47325	-0.57744
ENSG0000C	TGFBR3	1:9168034	37.704	10.6822	11.0083	8.76706	12.1871	3.02904	10.6503	4.39429	2.48407	7.46429	0.00175	-1.76852
ENSG0000C	RPS7P3	1:2329506	0.119092	0	0	0.334819	0.366519	0	1.72322	0	1.67368	2.75938	0.15845	2.93263
ENSG0000C	IMPDH1P	2:2011161	6.84192	4.78901	2.29714	3.72917	1.4802	2.11067	7.54601	1.419	7.55845	1.75219	0.70645	-0.83522
ENSG0000C	NSUN6	10:181406	9.03787	11.0789	12.4361	10.3074	7.50254	3.76045	5.3485	6.64083	10.0275	12.4465	0.63885	-0.26872
ENSG0000C	NIT2	3:1003347	26.5293	26.5141	37.8103	33.8163	15.0762	35.0121	43.237	31.5782	25.886	24.6999	0.91885	0.043539
ENSG0000C	IAH1	2:9473657	37.4763	28.2361	52.2799	42.7901	39.7481	56.8911	72.4949	65.48	41.7509	36.5385	0.45505	0.312578
ENSG0000C	DGAT2	11:757584	0.904694	1.45866	0.353261	0.154713	0.412019	2.17724	1.60475	0.304515	3.49817	0.150907	0.13785	1.47215
ENSG0000C	FAM129A	1:1847907	14.9412	11.7041	9.75205	6.89309	11.6309	14.4894	17.2471	26.5139	17.3959	32.9852	0.0069	0.89945
ENSG0000C	PGS1	17:783786	42.0991	44.3033	48.1728	37.2058	46.7266	31.0607	26.6233	42.084	24.8396	64.3058	0.50535	-0.23699
ENSG0000C	CASC3	17:401403	23.9151	26.8425	10.3903	10.2566	9.13386	16.6205	25.4004	15.9323	15.8513	22.104	0.3152	0.325091
ENSG0000C	TRGV4	7:3834157	41.7433	29.6163	25.3937	87.4075	91.7809	16.6925	43.1239	17.1776	38.5193	37.6941	0.36065	-0.83465
ENSG0000C	PRKAG2	7:1515561	14.0421	24.7755	9.07715	11.147	10.9241	9.33387	21.8164	11.3804	6.38676	11.6419	0.7507	-0.13172
ENSG0000C	LINC0049	20:185673	77.6112	46.2323	96.7898	78.9982	62.9916	85.7366	82.4698	82.761	66.4123	11.7331	0.7333	-0.22152
ENSG0000C	CBWD7	9:4099224	24.7907	13.619	15.2083	19.1459	13.7434	28.4844	17.7458	13.766	22.6233	18.8915	0.8067	0.202773
ENSG0000C	MIR22HG	17:171149	7.22708	4.51432	4.01862	13.7394	3.32964	16.2513	5.67925	8.45889	18.1037	5.33733	0.3191	0.725006
ENSG0000C	UQCC1	20:353025	17.8788	16.0851	16.9263	16.7159	12.9912	13.6517	13.6203	9.11328	13.1756	16.7253	0.53345	-0.26056
ENSG0000C	CDK18	1:2055045	1.06976	0.496746	0.487391	0.959648	0.542641	0.797843	0	0	0	0.609616	0.1375	-1.77309
ENSG0000C	CFAP44	3:1132110	0.671192	2.00583	0.57871	0.938076	0.708094	0.306639	0.842968	0.243863	0	2.62998	0.28445	-0.92253
ENSG0000C	OLFM1	9:1350754	0	0	0	0	0	0	0	0	3.11383	0.109418	0.0456	inf
ENSG0000C	MMAB	12:109553	8.43195	12.5024	22.0575	22.0123	15.6534	12.2197	18.0392	18.0025	22.3089	3.91333	0.82085	-0.14496
ENSG0000C	SPARC	5:1516610	0	0	6.38567	26.6224	1.76267	16.9617	0	1.56651	0.497365	0.579234	0.34515	-1.2823
ENSG0000C	IPP	1:4562730	4.94403	7.75434	4.8211	6.06832	2.91408	3.32014	8.14146	4.25065	12.7241	4.20513	0.7889	0.286062
ENSG0000C	AC007192	19:180589	1.04382	1.73513	2.00575	4.74032	1.99425	1.12029	1.72966	1.91134	4.26246	2.67267	0.7079	0.446492
ENSG0000C	SNRPD1	18:216122	41.3608	34.5045	33.4353	29.4658	29.6746	72.64	67.3552	35.9533	20.3389	7.5076	0.8187	0.138363
ENSG0000C	RP11-385	1:2365179	0.391784	0.518073	0.720996	1.06214	0.585385	0.998496	2.83331	1.26868	0	1.45587	0.483	0.950647
ENSG0000C	ASPSCR1	17:819768	26.569	34.2715	30.6097	23.7605	28.542	42.7901	46.8555	30.6473	32.1024	21.8794	0.5347	0.222332
ENSG0000C	ZBED1	X:2219515	23.4308	20.008	14.5942	14.4896	24.3598	8.44245	25.5365	12.2221	7.30661	25.5928	0.2428	-0.52287
ENSG0000C	ZKSCAN8	6:2814190	0.509306	0.72087	0.263553	0.089466	0.914584	0.004603	1.49878	2.14218	2.00927	2.19642	0.02075	1.72001
ENSG0000C	L3MBTL2	22:412052	17.0135	23.654	22.5207	15.8419	20.4654	17.1556	17.9656	19.9283	9.88347	24.9906	0.4933	-0.22479
ENSG0000C	PARD6G-/	18:801479	0.241312	0	0	0	0	0.605016	0.811926	0	0	0.888383	0.08015	3.24221
ENSG0000C	RP11-589	8:4213946	4.29768	3.91617	2.2063	4.95963	0.726738	0	0	0.141522	8.70124	0.159781	0.7039	-0.65727
ENSG0000C	ACTR10	14:579997	68.3239	45.654	63.9077	48.6531	41.9028	61.1412	65.4699	58.0173	62.7437	24.8952	0.955	0.029126
ENSG0000C	IGBP1	X:7013344	26.6085	34.0162	38.3861	46.6619	36.1234	29.1976	18.6293	32.7206	26.3197	14.9393	0.16915	-0.52002
ENSG0000C	CTA-384D	22:505235	1.96426	6.81127	2.19394	2.04827	1.69785	5.54486	7.53726	3.14545	1.57206	3.28539	0.6532	0.513226
ENSG0000C	SACS	13:233288	3.35815	2.14505	1.30806	1.24566	1.94573	4.26847	3.77012	5.76103	3.18382	4.80618	0.1778	1.25928
ENSG0000C	RP11-488	14:767621	1.98817	1.89374	2.75585	0.615205	1.29687	2.85461	1.50851	1.08364	0	1.78865	0.85225	0.298568
ENSG0000C	PAXIP1-A	7:1550034	8.2315	10.4046	10.6294	11.2145	7.75546	6.86872	5.24531	8.96741	12.294	9.14492	0.6396	-0.2168
ENSG0000C	KRT18	12:528971	0.489945	1.16933	1.76047	1.96647	0.753945	0	3.06666	2.5244	6.03523	1.55139	0.35135	1.28595
ENSG0000C	SLC39A14	8:2236724	5.49539	3.23211	1.77796	1.9758	2.3708	2.92228	2.19852	1.78969	2.40535	3.13644	0.62135	-0.33539
ENSG0000C	UBE2D4	7:4392643	6.5189	5.14645	17.8445	8.50164	9.71396	3.56065	6.45643	6.90839	9.00685	2.09945	0.2841	-0.80959
ENSG0000C	AC062017	2:2390481	2.05222	2.59022	4.41514	3.13561	0.879054	3.27745	2.71368	1.71546	1.07268	0.439635	0.6476	-0.52508
ENSG0000C	RPE	2:2100025	17.335	14.5605	9.10066	9.33083	10.1739	19.9684	11.9334	11.911	19.0894	6.55949	0.6092	0.199801
ENSG0000C	EFNA3	1:1550637	0.471249	1.55983	1.80371	1.72023	1.42135	0	1.0398	2.17086	2.74461	1.22051	0.9214	0.159983
ENSG0000C	RP11-373	2:6088152	0.556454	1.44536	0.981623	0	1.1767	3.21895	2.69406	4.0656	0.807337	4.1041	0.28465	3.18973
ENSG0000C	BTG3	21:175936	21.4379	12.1483	18.2011	19.7692	13.8028	52.1635	66.5565	39.3656	44.3824	12.9513	0.0013	1.36159
ENSG0000C	RPL21	13:272513	1909.44	1689.96	3111.56	2126.26	829.132	1268.94	1643.82	1852.73	1517.3	135.372	0.24845	-0.55076
ENSG0000C	TCAF1	7:1438513	1.69614	1.85722	1.25922	2.15534	2.22292	2.19857	1.36518	2.42223	1.72919	2.81284	0.9708	0.039486
ENSG0000C	LSP1	11:185296	263.944	403.342	492.574	288.857	387.603	259.041	462.318	576.921	307.379	277.009	0.94155	-0.02865
ENSG0000C	PDE4DIP	1:1488081	10.5624	14.7167	3.08725	3.14884	4.32855	33.7568	12.1248	12.8073	12.3366	20.0803	0.01405	1.19786
ENSG0000C	KLHL26	19:186369	2.80705	1.26516	2.56238	1.77568	6.3054	0.458677	1.36514	5.81063	0	4.9962	0.8105	-0.19384
ENSG0000C	HIST2H2B	1:1497826	1.15225	0	2.08716	0.75251	0.908258	0.02183	0.984851	0.5342	0.306075	0.369322	0.4368	-1.13716
ENSG0000C	MMP9	20:460089	1.25098	0	0	0.844517	0.036721	4.77037	0.315878	0.028382	0	0	0.6658	1.01267
ENSG0000C	RELB	19:450014	16.1269	10.2932	4.66343	10.0057	13.4017	29.0621	35.0501	28.62	16.2852	21.4435	0.00555	1.23814
ENSG0000C	PTPN23	3:4738101	0.963022	1.58353	0.82723	0.652425	1.97615	1.89516	1.19472	2.08095	3.10207	3.85295	0.06185	1.2524
ENSG0000C	KIAA1551	12:319593	128.455	119.591	22.1286	22.3366	32.6664	132.245	107.629	54.9328	28.2646	100.316	0.2969	0.487385
ENSG0000C	NBPF10	1:1460646	1.56203	3.44479	0.829217	0.706592	1.5756	1.96443	2.30075	1.19802	1.2919	5.00204	0.906	0.147935
ENSG0000C	KLF3	4:3861270	32.5159	47.175	40.1791	61.5038	38.945	6.93853	1.93928	5.12128	11.5299	11.919	0.00005	-2.79899
ENSG0000C	STPG1	1:2430755	2.2451	2.9564	4.97442	1.79921	1.4032	2.41211	2.1575	4.82872	1.25912	2.33153	0.90115	-0.10793
ENSG0000C	SERINC3	20:444962	47.1658	45.189	53.6138	32.7341	39.7591	54.1886	45.6436	43.5329	26.6021	67.3775	0.99615	0.00176
ENSG0000C	CTD-2306	15:457026	0.717626	0	0.692548	0.310714	0.5043	0	0	1.501	0	0	0.82845	-0.31286
ENSG0000C	NICN1	3:4941677	3.67414	7.30799	2.55353	4.15273	2.56893	0.820706	2.68887	2.20751	0.050394	4.13018	0.14915	-1.42222
ENSG0000C	DIP2C	10:274189	0.719394	1.27814	1.23388	0.944873	0.891409	0	0.12803	0	0.113992	0.946232	0.0764	-2.46822
ENSG0000C	MGME1	20:179415	22.079	9.33069	20.7809	14.8874	9.25038	32.5436	17.5343	17.3189	20.1804	10.0726	0.6202	0.360993
ENSG0000C	NEDD4	15:558269	1.97217	1.78355	1.08842	1.19448	1.00056	4.69355	1.17523	0.763263	0.248983	2.41007	0.8552	-0.10922
ENSG0000C	CCDC13	3:4269266	0.48945	0.240859	0.096935	0.267706	0.100812	2.62323	0.060353	0	0	0.211521	0.3217	1.3154
ENSG0000C	IRGM	5:1508465	0.418477	1.37748	0.099936	0.338581	0.217172	0.308066	1.62591	0.706732	0	0.213672	0.8323	0.309085
ENSG0000C	ZNF493	19:213971	11.986	17.6172	7.24285	10.952	6.88677	4.59851	9.87686	17.1857	13.7873	23.3496	0.554	0.377396
ENSG0000C	LILRB1	19:546171	1.03543	16.0177	0.469606	2.77356	1.05796	33.0287	0.959487	1.9356	5.54	0.127676	0.1291	1.0202
ENSG0000C	C7orf26	7:6590016	22.3867	20.6928	11.6152	10.8165	16.1012	10.8966	19.6843	14.401	5.95486	18.7495	0.44285	-0.351
ENSG0000C	RPL32P29	14:495709	12.4558	2.06087	8.59812	4.77695	1.91145	9.12989	3.25217	7.68706	8.72169	0.141413	0.97455	-0.05062
ENSG0000C	C1orf198	1:2308371	3.13849	2.59514	1.55513	0.473919	0.972232	4.30687	2.35623	3.39557	0	2.67527	0.6126	0.462113
ENSG0000C	PPP6R1	19:552266	70.2272	68.6048	17.8603	14.8902	32.1482	51.3769	54.0773	34.2111	16.4565	60.3386	0.90485	0.039645
ENSG0000C	RP11-73M	14:103553	0	1.52986	1.1419	0	0.16305	2.64957	2.89106	0.557836	0	1.30779	0.90355	0.246282
ENSG0000C	LAMTOR1	11:719985	40.4638	46.5437	81.0118	99.3431	84.7704	38.1321	41.8591	77.9191	67.346	26.6283	0.5384	-0.47689
ENSG0000C	LA16c-39	16:144493	11.0767	9.64051	1.57555	2.88213	1.70718	2.41619	4.80679	2.30485	6.51721	2.93952	0.8246	0.348905
ENSG0000C	NLRX1	11:119166	8.54785	6.86402	3.73528	4.25118	3.86492	2.65057	5.36349	3.23059	4.33684	7.23349	0.54405	-0.29111
ENSG0000C	STK26	X:1320232	57.7928	38.353	20.6654	26.7732	20.0474	19.595	12.9755	19.2257	24.7538	18.5073	0.0324	-0.72817
ENSG0000C	FAIM	3:1386086	12.971	8.29139	12.198	9.20283	13.3166	4.22531	7.87576	11.5024	25.6833	8.6223	0.905	0.072666
ENSG0000C	DUSP6	12:893472	27.8694	55.3304	5.34868	27.5268	8.13105	35.9062	20.6245	14.2151	57.5885	55.3929	0.2767	0.517952
ENSG0000C	ZWILCH	15:664980	7.66059	7.30903	3.67213	7.91644	4.95053	10.3323	11.3555	5.34287	9.76648	5.46101	0.64105	0.401727
ENSG0000C	ECSCR	5:1394085	0.194216	0.891124	0.126185	0	0.044174	4.12534	0	1.1176	1.47281	0.344109	0.2435	2.65153
ENSG0000C	AUH	9:9121381	15.1583	11.3592	25.1397	28.5988	14.7461	17.4236	18.6548	23.4325	16.7083	10.8493	0.76395	-0.12121
ENSG0000C	ZNF563	19:123174	0.862495	9.0954	2.26524	0.415112	0.497727	2.62435	3.87501	0.251762	1.94994	0.973869	0.55095	-0.43358
ENSG0000C	AK5	1:7728205	0.544612	16.7606	2.35119	0.534429	0.640939	0	0.335467	0.06699	0	0	0.0744	-5.40109
ENSG0000C	RP11-175	12:100156	1.89282	2.40993	1.99326	0.900265	0.659054	4.53105	2.54954	0.868276	0.494375	2.95277	0.57965	0.507076
ENSG0000C	RP3-323N	10:107983	1.66102	2.60741	0	0.713318	0.099113	0.778737	1.46804	0.103227	3.42615	0	0.81835	0.433313

ENSG0000	CD300LB	17:745211	0.42991	3.64033	0	0	0.115191	0.415797	0.015869	0	0	0.058667	0.1816	-2.97268
ENSG0000	OGT	X:7153308	55.6829	73.4637	44.0813	27.4208	41.2931	44.8939	56.9815	75.7223	35.4069	153.069	0.24215	0.444335
ENSG0000	IMMP1L	11:314324	23.2436	17.5874	24.2037	21.7445	11.6924	25.2883	22.0195	19.1547	15.0968	3.43501	0.73255	-0.20309
ENSG0000	TMEM155	4:1217589	0.57927	1.0354	0.445769	0	0.01532	2.67059	0	0	0	0.756113	0.69875	0.502296
ENSG0000	SDHB	1:1701872	69.514	84.6222	122.443	112.417	68.4532	99.8959	96.1224	102.059	88.0182	43.3984	0.73995	-0.10854
ENSG0000	ZNF382	19:365106	5.69285	7.7732	0.768654	1.55749	1.4185	0.122703	7.04161	3.45457	1.44339	3.32782	0.88745	-0.12985
ENSG0000	AC005224	17:142104	0.324753	0.335903	0.850582	0	0.199617	2.0792	0.625839	0.371137	0	0.676665	0.6142	1.03228
ENSG0000	TRBV6-5	7:1424509	20.845	51.432	34.7865	41.7127	24.6802	51.5486	40.6371	21.5234	48.7114	14.4356	0.9018	0.060323
ENSG0000	AUNIP	1:2580017	1.99437	0.313448	0.289174	0.136239	0.044457	1.93604	0.263601	0.147848	0	0	0.8877	-0.26456
ENSG0000	STX17	9:9990663	13.441	12.0435	12.5245	12.46	14.0392	12.9634	8.97769	10.5919	7.45654	19.6526	0.46335	-0.27935
ENSG0000	CH17-385	1:1463218	3.23504	2.42883	8.63728	3.8678	3.9004	1.61874	2.16884	3.75319	1.59957	1.72022	0.6278	-0.94644
ENSG0000	FBXL6	8:1443522	36.6005	38.9694	18.1611	28.846	14.2901	38.9692	53.5523	21.8709	17.6917	23.5044	0.7674	0.199851
ENSG0000	CALM3	19:464807	95.885	102.214	52.5643	63.5824	67.8969	179.039	137.91	88.5234	66.5595	40.6536	0.34265	0.42496
ENSG0000	PDZD4	X:1538021	1.10818	0.275344	0.931531	0.205488	3.09674	0	0	0	0	1.35472	0.28465	-2.06852
ENSG0000	STX16-NP	20:586512	7.2989	10.478	0.390878	0.556489	0.736604	1.9015	7.36518	0.749034	0.062319	0.812354	0.5554	-0.96309
ENSG0000	CEP290	12:879800	11.7767	8.57563	12.2805	4.4369	17.6254	20.289	20.2017	6.67521	11.6455	26.8235	0.7665	0.318773
ENSG0000	CTD-2228	5:473235-	2.3184	2.56706	0.266592	0.81349	0.687283	2.86411	0	0	0	2.12266	0.80665	-0.27003
ENSG0000	PDGFD	11:103907	9.10904	2.71958	3.72071	2.78355	1.98587	0	1.00775	1.63346	0	0.983013	0.0927	-2.49757
ENSG0000	ANKRD23	2:9681624	2.46035	2.32011	1.24498	1.34115	0.995483	4.87242	1.3579	1.32423	0.366345	3.09881	0.53695	0.46571
ENSG0000	RP13-93L	9:8936078	1.86292	15.9325	0	0.245058	0	4.85076	10.7676	0	0	0.26174	0.97735	-0.06623
ENSG0000	UCHL5	1:1930122	37.004	31.2171	36.9466	30.3432	35.8135	40.6447	31.3423	23.5302	39.8827	24.8749	0.71945	-0.19567
ENSG0000	ANP32B	9:9798336	51.2166	73.0774	78.1016	48.6904	108.171	63.9115	78.0001	118.448	69.9531	71.9029	0.57075	0.184423
ENSG0000	RP11-171	2:1785211	0.361177	0.041104	0.150388	0.042534	0.163802	0.064859	0.77688	0.654565	0	0.828275	0.3549	1.71092
ENSG0000	MRPL24	1:1567373	57.5725	43.1581	128.801	120.58	135.303	64.5702	57.1874	73.217	82.5922	37.5661	0.06805	-0.67066
ENSG0000	RP11-473	16:909164	0.872356	0	0.744482	0.839701	0.49885	0	1.45091	0.881206	2.98549	0.690598	0.3704	1.17893
ENSG0000	TAF6	7:1000927	25.1582	41.6324	15.5073	9.16762	21.0035	22.9239	27.4543	15.2484	9.56366	22.2431	0.672	-0.24984
ENSG0000	KLRD1	12:102260	21.7914	15.5965	37.1522	31.7876	36.5341	19.9682	79.7656	75.2952	11.8142	17.6335	0.54245	0.266941
ENSG0000	ATF2	2:1750722	26.4812	21.4504	8.69668	9.27636	7.46744	13.8415	17.1213	11.4084	21.3873	9.58317	0.8165	0.088992
ENSG0000	ZNF483	9:1115251	1.89062	2.08347	0.772772	2.375	0.517599	2.4581	0	1.8984	0	0	0.6323	-0.74228
ENSG0000	RP11-13N	18:216508	0	1.09862	0.232558	0	0.085108	0.49377	0.434385	1.01107	0	0.093247	0.68185	0.753352
ENSG0000	RP11-815.	15:853805	0	0	0	0	0.133214	0.223187	0.600674	0.216265	0.622054	0.382499	0.2537	3.75059
ENSG0000	VMP1	17:596185	56.1042	36.4939	48.4639	40.2112	36.9204	128.017	109.308	93.2543	113.335	69.701	0.0091	1.38253
ENSG0000	NUCB1	19:489000	71.2651	50.3006	34.9551	37.6613	43.5385	58.287	67.9115	43.6867	43.1854	45.181	0.70945	0.13592
ENSG0000	HDAC1	1:3229208	106.132	119.262	100.714	75.2289	84.3215	71.5785	88.475	79.4285	63.4519	80.1223	0.17385	-0.40015
ENSG0000	LETMD1	12:510479	17.4807	27.6635	17.5716	24.7092	15.1766	11.1146	22.8648	16.3684	14.7811	29.5762	0.67705	-0.16289
ENSG0000	LRRC23	12:686711	4.23245	1.91864	7.66168	3.11047	2.66909	6.99257	4.30522	2.77186	0	2.29278	0.6174	-0.35534
ENSG0000	RPP25	15:749544	3.87973	7.33441	2.87915	4.34818	3.83414	6.00118	3.75389	2.73357	0.56229	2.51867	0.45405	-0.46378
ENSG0000	DPY19L4	8:9471970	4.7021	5.11141	4.89147	5.96783	2.16578	3.68747	1.68062	3.13305	9.92202	2.37089	0.6923	-0.30481
ENSG0000	GLCCI1	7:7636517	40.5529	39.603	26.1201	19.3715	31.7864	25.4959	32.2342	16.1593	19.356	46.9224	0.5745	-0.29408
ENSG0000	OTOF	2:2645720	0.50806	0.877876	0	0.503599	0	2.10201	8.00756	0.205719	0.873665	2.55931	0.046	2.61872
ENSG0000	NFIC	19:335956	6.17507	8.12699	9.6032	8.87145	22.3339	6.17191	6.61226	6.48083	9.81324	24.1033	0.88495	-0.0903
ENSG0000	CTB-31N1	16:215972	2.10444	1.0928	1.01008	1.23944	0.355088	4.53249	0	0	2.72493	2.10325	0.63465	0.708814
ENSG0000	SMG6	17:205415	8.36048	12.8407	4.9422	2.29114	7.52883	12.1099	8.46515	7.52354	1.22172	16.3243	0.70745	0.301965
ENSG0000	GDAP1	8:7423469	4.91186	5.73183	1.72989	2.88542	2.56527	11.4784	3.0015	1.57577	4.059	1.27039	0.93115	0.05305
ENSG0000	RP11-81A	15:798335	0.82232	1.0982	0.589852	1.41187	0.478858	1.98329	1.03926	3.08513	1.12384	3.88468	0.4558	1.28827
ENSG0000	NFYA	6:4102691	13.1284	13.6207	6.36604	11.8788	5.79818	4.3861	8.63506	3.43095	8.82165	8.42505	0.29315	-0.65032
ENSG0000	SELL	1:1696620	627.543	1171.25	194.535	115.569	160.612	97.8833	113.809	46.5887	24.2494	61.3479	0.0008	-2.71996
ENSG0000	RP11-43F	5:1594625	8.9086	9.73723	11.4234	12.0075	6.36423	8.55067	7.65089	7.58817	11.8104	5.8931	0.9212	0.047548
ENSG0000	INTS9	8:2876766	11.9375	10.0307	11.6133	12.8628	7.6421	7.27037	8.83735	12.1003	1.79736	11.3987	0.192	-0.54178
ENSG0000	IRAK4	12:437589	24.8711	19.4922	17.0755	15.1476	15.948	28.0436	23.8781	16.0659	22.4777	27.8178	0.43525	0.307633
ENSG0000	TMEM79	1:1562829	10.1822	8.798	12.1433	7.18206	7.97919	16.3859	18.1084	7.69489	6.2202	16.14	0.53455	0.445176
ENSG0000	OTUB1	11:639746	150.983	166.37	158.091	146.233	128.661	220.088	223.859	166.216	141.276	92.5815	0.74005	0.156934
ENSG0000	LINC0024	6:3079865	3.55693	1.79913	4.49484	1.72037	1.57385	1.75596	0.7288	1.11354	1.44072	1.07922	0.47705	-0.97969
ENSG0000	SLC4A1AP	2:2758296	32.9574	38.6593	34.6625	22.3277	31.1769	20.048	27.0901	21.534	17.6679	40.6981	0.61615	-0.33858
ENSG0000	DGCR6	22:189060	5.8285	2.12092	14.6742	11.7437	9.45124	4.81937	3.75027	14.802	12.5259	1.86136	0.9801	-0.02135
ENSG0000	RPAIN	17:528226	38.7461	52.5238	54.1089	45.5595	36.4337	53.4097	39.7597	34.9175	39.4167	31	0.7716	-0.14794
ENSG0000	SPATS2	12:493665	2.80329	4.72806	3.94789	1.08149	9.9355	3.29571	1.92104	3.26828	0	10.4972	0.34945	-0.65241
ENSG0000	ANKZF1	2:2192297	64.4123	130.181	43.0236	50.3435	33.6408	47.1561	65.3995	42.1866	40.1169	50.3851	0.56155	-0.35732
ENSG0000	PPT2	6:3214835	4.20591	4.96153	6.80511	6.89297	7.70701	2.92602	4.74225	3.46376	10.6751	3.88658	0.83225	-0.21906
ENSG0000	ZNF280D	15:566301	12.9193	13.0693	4.94689	7.53541	6.17645	13.8397	9.5777	6.01452	1.57526	7.70287	0.9447	-0.04361
ENSG0000	C11orf49	11:469366	21.4501	17.8992	31.0756	26.3077	37.1312	18.9098	13.0208	34.0417	11.6202	41.6911	0.70235	-0.21987
ENSG0000	OR7E38P	7:9796608	6.27795	3.69195	9.30245	7.13778	5.17802	4.50687	7.50995	8.70896	12.7712	2.42144	0.7552	0.169062
ENSG0000	ATP13A4	3:1934020	0.377716	0.651403	0.655752	0.731503	0	0	0.201043	0.159866	0.174593	0.124655	0.21915	-1.62179
ENSG0000	CTC-548K	19:144330	1.39146	2.50662	0.052051	0.069719	0.423063	0	2.83546	0.307171	1.13441	1.40941	0.69315	0.596528
ENSG0000	TSEN54	17:755160	107.987	75.0174	206.427	182.67	151.336	111.288	135.65	142.854	98.6136	180.286	0.65535	-0.14131
ENSG0000	SNURF	15:248236	13.0669	13.5937	8.46063	8.68845	6.11597	13.642	25.4395	12.2316	16.5768	4.67406	0.666	0.387296
ENSG0000	AQP3	9:3344115	117.109	54.7866	73.9973	103.003	107.199	70.7686	18.8434	57.0435	101.128	69.2389	0.11885	-0.53603
ENSG0000	COX10-A	17:137564	17.4584	30.0391	13.2201	12.1981	7.67253	17.4144	17.4399	19.7915	36.7285	29.7735	0.1896	0.755546
ENSG0000	SART1	11:659616	40.9419	44.3358	25.2009	17.3433	21.3546	32.0603	32.8777	27.4731	26.192	25.3744	0.9921	-0.00321
ENSG0000	APAF1	12:986451	4.81253	6.73907	1.30994	2.52959	2.44586	3.94598	4.60356	1.77207	3.20152	3.34142	0.86395	0.096607
ENSG0000	MDH1	2:6312138	207.353	148.355	192.606	201.05	156.436	328.94	214.253	146.271	153.29	77.3392	0.9326	0.030931
ENSG0000	ESYT2	7:1587309	32.8915	56.5343	4.64979	4.32987	8.6535	19.255	21.1754	10.0434	3.49424	17.0455	0.0771	-0.79241
ENSG0000	ILF3-AS1	19:106518	41.921	26.7568	67.6832	54.3481	29.4739	22.4469	21.2573	28.2339	41.8825	21.6491	0.07565	-0.67605
ENSG0000	RILPL1	12:123457	0.537959	0.706119	0.596962	0.098332	0.609535	1.23896	1.22439	0.863487	0	1.11579	0.6983	0.376718
ENSG0000	ABHD13	13:108218	12.432	10.5849	6.54022	8.06524	5.75241	10.8454	7.23486	8.42407	5.40075	6.71826	0.6916	-0.12712
ENSG0000	DR1	1:9314974	45.4222	33.1013	22.6348	18.4698	21.1829	25.7964	44.0266	34.4923	41.7331	20.9948	0.82945	0.188941
ENSG0000	AC009303	2:1178594	0.380768	0	0.430391	1.60408	0.341876	0	0	0	0	0.033215	0.22975	-6.28051
ENSG0000	AL450992.	1:1518418	5.68894	2.40505	6.05021	10.4866	3.46677	4.08666	2.09731	3.50848	10.5808	2.02458	0.7671	-0.26545
ENSG0000	TARBP2	12:535001	34.0908	36.1417	31.0852	35.8153	21.3886	16.0559	29.6804	27.644	13.8841	15.5852	0.19305	-0.61188
ENSG0000	CTD-2377	17:486359	0.281776	0	0	0	0	0	2.61432	0.350394	0	0.367416	0.0084	4.07643
ENSG0000	RPL13P12	17:173833	9.24124	6.69986	16.0039	24.3394	5.56693	7.41311	7.37415	7.91471	14.2304	0.560394	0.35115	-0.77593
ENSG0000	BID	22:177341	71.0867	46.6	85.298	75.5125	109.821	91.1199	81.2462	55.1403	69.2101	40.63	0.5113	-0.30125
ENSG0000	TRAF3IP2	6:1112990	12.902	17.4641	9.69423	8.28958	11.6612	7.42197	2.35438	4.99089	0	10.3134	0.20745	-1.10265
ENSG0000	DDIT4	10:722739	131.401	144.564	157.904	587.609	216.435	394.979	406.024	348.301	354.687	270.826	0.231	0.466958
ENSG0000	SNX12	X:7105633	2.62337	2.97718	2.76587	3.91592	6.83945	5.22292	3.24371	4.94964	3.58945	5.11847	0.5268	0.478055
ENSG0000	HSPE1P13	2:3898139	0	0.172182	0	0.248756	0	0.223825	0	0	5.54852	0	0.3053	3.96554
ENSG0000	METTL13	1:1717816	23.3056	26.4278	11.9801	17.7364	21.6717	12.5412	17.458	10.75	9.18828	25.1932	0.33915	-0.40029
ENSG0000	DNTTIP2	1:9386628	76.0917	55.096	80.8783	65.6383	74.4534	82.4438	87.7178	61.3237	64.6674	72.2742	0.77105	-0.0956
ENSG0000	PIGH	14:674470	30.6632	14.5764	40.3925	47.4783	24.8044	43.6652	28.685	35.7312	38.5484	15.5494	0.8699	-0.07584
ENSG0000	NEURL4	17:730699	2.85792	6.23836	1.90394	1.32675	2.03826	2.40042	1.1129	2.11782	0.78523	6.77427	0.9647	-0.04458
ENSG0000	SNRNP40	1:3125956	59.178	69.0802	70.6861	49.0764	52.6034	71.8213	70.8909	63.1888	32.1495	33.1628	0.5423	-0.18782

ENSG0000	MRC1	10:178093	0.170197	1.74938	0	0	0	15.7015	2.71114	0.143202	0	0.084384	0.16	3.31152
ENSG0000	FAM83G	17:189503	3.62899	2.69903	1.33808	1.04906	3.65477	0.729901	2.54013	1.3108	3.89505	7.67481	0.70585	0.345667
ENSG0000	POLR3C	1:1458240	28.3703	22.6267	27.7123	29.6236	16.483	30.5737	25.3638	27.1935	14.4228	12.9077	0.6007	-0.19981
ENSG0000	CTB-96E2.	17:283575	0.706776	2.27764	0.301919	0	0	1.23351	3.30159	0	0	0.630907	0.76055	0.665729
ENSG0000	PAK3	X:1109442	0.73343	1.32989	0.379573	0.559242	0.81304	2.62916	0.585726	1.26858	0.865695	4.32527	0.09985	1.4348
ENSG0000	ADTRP	6:1171205	6.02456	11.6584	3.32066	0.467898	1.77001	47.7251	24.3981	8.71017	5.80248	2.02072	0.01105	1.93533
ENSG0000	RP11-111	11:763813	0.691033	0.419822	0.734091	0.289082	0.900054	0	0.645013	0	0	0.525433	0.1465	-1.75927
ENSG0000	LINC0009	9:1340254	6.43668	6.96124	8.29791	8.84452	8.03105	4.09768	3.60878	7.5804	4.45768	8.98515	0.4206	-0.5915
ENSG0000	CHD2	15:928827	125.148	148.32	49.0001	104.443	69.9911	106.625	109.75	65.0569	129.344	144.967	0.89895	0.059111
ENSG0000	ATXN10	22:456570	79.2222	93.0823	62.6957	61.4252	77.4878	60.9512	80.0732	49.6156	63.0823	49.2215	0.19175	-0.45684
ENSG0000	HMGN2P3	16:256920	4.19425	2.453	1.70463	1.39382	0.558248	3.78845	4.7701	0.943854	1.46408	0	0.96955	0.034768
ENSG0000	EPRS	1:2199686	27.0116	22.4226	24.926	20.8173	21.7747	11.1269	16.7411	21.349	14.953	31.4112	0.49805	-0.2305
ENSG0000	ASTN2	9:1164252	1.70878	0.439755	1.7056	0.555371	2.71149	0	0	0.780847	1.92692	0.24706	0.41965	-1.18247
ENSG0000	LARS	5:1460843	37.8461	45.085	33.7607	46.5627	94.6885	32.9183	51.7953	34.5185	43.8665	83.5706	0.433	-0.49468
ENSG0000	LINS1	15:100553	33.1457	60.1412	35.6916	39.4164	28.4736	19.6629	33.5372	37.3373	44.7503	48.5863	0.67	-0.17632
ENSG0000	CTD-2574	16:298583	1.9308	0.961168	2.24866	4.69285	2.73472	1.22145	3.0918	4.44464	2.90526	2.18813	0.93075	-0.09947
ENSG0000	PLEKHF2	8:9513380	17.4455	27.6672	12.6315	17.1535	6.57414	17.8807	11.9392	10.4113	5.53403	7.06702	0.069	-0.6766
ENSG0000	RP11-552	17:758970	0.64637	0.760883	0.257709	0.515293	0.254749	0.62413	0.567907	0.149372	0.136257	0.094807	0.53875	-0.72268
ENSG0000	C1QC	1:2264362	0.095441	0	0	0	0.923578	20.5075	1.52496	3.06152	0.238144	3.12551	0.12805	4.52974
ENSG0000	EIF4A1P10	X:9177926	0.33675	0.229079	1.41999	1.36507	0.923688	0.54532	0.167573	0.680006	1.24307	0.16132	0.7823	-0.64413
ENSG0000	PDP1	8:9385780	9.65842	13.9316	3.10263	5.37905	2.87972	7.88038	4.96389	6.56592	6.47204	6.40272	0.57275	-0.21694
ENSG0000	HS2ST1	1:8691464	4.31459	5.83099	1.78265	1.1341	2.04587	3.73551	2.18338	2.20234	9.77724	1.90194	0.5584	0.491129
ENSG0000	VCAM1	1:1007197	0.074281	0.481731	0.203177	0	0	2.08223	9.80719	0.792705	0	0.358186	0.03245	4.35698
ENSG0000	PSPC1	13:196746	48.8505	61.0423	60.1586	58.2956	60.9536	26.1557	35.7109	43.133	49.4066	53.5642	0.20035	-0.50872
ENSG0000	IARS	9:9221020	31.0761	29.1965	12.6608	26.3636	7.48254	19.4095	22.9332	28.2609	21.7316	17.4017	0.55935	0.190295
ENSG0000	CTR9	11:107509	34.739	33.8521	28.9665	23.5893	28.526	36.545	16.5662	16.2183	18.1674	33.3901	0.1981	-0.39918
ENSG0000	SNAP47	1:2277285	31.4817	28.6738	46.3711	34.558	28.9888	21.7137	36.94	34.8376	37.335	18.1871	0.702	-0.17292
ENSG0000	RP11-264	16:289739	19.9187	22.7645	1.5479	13.6224	0.659108	25.5863	8.00109	1.1692	1.42895	1.21957	0.6677	-0.56399
ENSG0000	GDPD5	11:754346	6.88659	14.7348	3.6938	3.45218	7.69025	6.54513	5.87277	3.50139	12.5209	14.2039	0.3441	0.564714
ENSG0000	IQCG	3:1978890	6.39117	3.6615	4.06426	8.33815	6.16596	14.3149	18.3463	29.7522	5.32793	6.16842	0.12745	1.39217
ENSG0000	PPM1F	22:219194	5.2396	6.50703	1.20417	1.92955	2.56508	3.12146	3.1404	4.16729	3.88372	6.11421	0.8689	0.11036
ENSG0000	PARVG	22:441729	130.43	128.531	99.5633	91.3035	80.7379	191.485	147.998	90.6844	73.7883	67.8757	0.86825	0.065021
ENSG0000	IMPDH2	3:4902432	31.468	39.952	69.8075	51.6145	60.5674	26.4391	37.7923	44.8221	40.6228	35.9905	0.1339	-0.47732
ENSG0000	NPHP3-A	3:1325581	1.68748	2.21556	0.656231	0.306088	2.06462	1.61794	5.31777	0.716297	0.828347	4.41181	0.67075	0.648459
ENSG0000	AC010883	2:4322957	0.451259	1.17493	0.726439	1.66897	0.122327	0.13711	2.89706	0.653322	0	0.601695	0.97265	0.060484
ENSG0000	KDM1A	1:2301944	39.1766	44.8112	26.6608	34.4737	20.0274	17.5247	24.5308	20.0192	32.0303	22.922	0.4311	-0.41625
ENSG0000	EXTL3-AS	8:2860046	0.204997	0.360178	0.036338	0.299368	0.057803	0	0.878426	0	0	1.27184	0.5982	0.748981
ENSG0000	PARP12	7:1400237	58.9523	76.157	27.8221	35.7858	21.2224	62.6717	102.616	50.3659	40.1149	40.5235	0.18935	0.502854
ENSG0000	GMEB1	1:2866873	5.50884	4.92157	4.03432	5.45673	6.49167	10.4918	7.93136	9.95754	12.0258	10.4156	0.1511	0.803536
ENSG0000	RAB4B	19:407716	85.4371	78.6853	54.1622	73.598	74.1194	113.237	94.526	56.8972	58.4887	49.1143	0.92405	-0.0573
ENSG0000	CTD-2260	5:9588509	0.860388	0.084585	0.892964	0.728356	1.06287	0.052634	0.758175	0.201975	1.82804	2.40627	0.68145	0.523373
ENSG0000	STARD9	15:425756	0.242996	0.863823	0.055967	0.110365	0.362752	0.35504	1.13088	0.617746	0.502734	1.18269	0.06735	1.38543
ENSG0000	HIST1H2B.	6:2712589	2.15906	0	2.38378	2.10086	1.81196	1.93972	0.951153	0.165065	1.78296	1.35144	0.66065	-0.60802
ENSG0000	E2F4	16:671921	59.3563	62.7681	37.5371	42.1304	51.0087	42.6071	67.9691	42.3828	53.1364	67.9671	0.71855	0.109361
ENSG0000	PDCD5	19:325810	74.9015	68.5033	92.7185	114.506	63.8512	122.802	126.076	100.106	52.3583	20.5124	0.8782	-0.06209
ENSG0000	PTAFR	1:2814716	1.641	12.6792	0.538604	1.50236	0.366174	46.6908	4.83616	1.43794	0.032522	1.63251	0.07775	1.49415
ENSG0000	TMEM52B	12:101583	0	0.017529	0	0	0.135435	1.69945	0.426938	0	0.165431	0.26873	0.0902	2.70491
ENSG0000	SGO1-AS	3:2016059	0.312823	0.42026	0.034398	0.105483	0	1.78884	0.148858	0	0.348939	0.427346	0.09555	2.29171
ENSG0000	NDUFA4L2	12:572293	0.329092	0.489388	0	0	0	0	3.09241	0.1932	0	0	0.3058	1.8033
ENSG0000	TOM1	22:352988	26.1358	35.396	23.0972	21.5126	17.4444	43.9144	33.8262	20.7443	18.1183	26.3375	0.6451	0.162836
ENSG0000	LRRC1	6:5379449	3.22809	0.419376	2.59597	1.29649	0.559492	1.87371	0.488736	0.353739	0.093762	3.14374	0.3956	-0.67218
ENSG0000	INPP5B	1:3786069	15.0473	11.6366	6.10689	5.68806	5.99839	10.1105	7.04898	6.71112	5.48979	11.5342	0.5799	-0.22195
ENSG0000	RPL27	17:429982	451.117	311.345	627.432	523.842	123.334	428.866	475.866	385.639	302.181	33.672	0.32135	-0.37478
ENSG0000	ZNF683	1:2636163	0.682057	38.5433	5.04891	2.63637	4.22098	47.8806	468.877	162.963	35.5601	155.214	0.00005	4.09539
ENSG0000	RP11-175	1:2023486	77.9296	60.7763	26.7384	32.4412	6.62075	60.8195	72.2692	10.6776	21.7877	1.12641	0.8176	-0.28934
ENSG0000	H1F0	22:378050	0.120497	1.9952	0.123717	0.382149	0	4.64942	0.955658	0.267935	2.05507	0.061537	0.1743	1.73998
ENSG0000	GAMT	19:139708	10.8989	11.4562	30.9106	29.233	28.3813	4.44365	12.369	25.9154	11.6948	7.12187	0.06885	-0.95375
ENSG0000	PLEKHA1	10:122374	20.7555	20.2363	23.9223	17.6408	24.0082	1.28471	3.39923	10.6025	24.5225	17.9799	0.01515	-0.97525
ENSG0000	WAS	X:4867659	10.0002	9.09711	4.28603	7.2344	10.3111	6.84577	5.83248	5.13367	2.21291	11.1286	0.48525	-0.45004
ENSG0000	ZNF714	19:210821	1.86142	5.24927	0.792158	2.65835	0.876802	2.40806	1.02062	1.38858	0.743918	2.01205	0.38315	-0.62087
ENSG0000	ZNF589	3:4824109	5.54105	8.08274	5.06295	4.45861	2.00467	5.89463	5.90326	6.99279	1.69293	5.2703	0.98435	-0.01697
ENSG0000	HHAT	1:2103282	0.938716	1.16389	0.929585	1.60858	0.342582	1.88358	1.6512	1.5687	2.54711	0.634249	0.27265	0.789987
ENSG0000	CACNA1H	16:115312	0.700178	0.775679	0	0.074103	0.379421	1.57952	0.410277	0	1.75827	0.410442	0.32435	1.18615
ENSG0000	TMEM71	8:1326850	99.8238	55.8182	117.062	156.017	94.4763	34.6913	21.7158	51.2665	104.093	60.6776	0.0084	-0.95954
ENSG0000	TOX	8:5880541	15.7046	22.8792	16.3613	8.22733	12.2002	16.4119	28.2812	24.5007	26.2449	27.1066	0.0644	0.66059
ENSG0000	FCGBP	19:398633	0.152964	1.55998	0.098742	0.003486	0.025326	0.515557	1.53714	0.325812	0	0.450417	0.71245	0.375141
ENSG0000	RP11-686	17:354038	0.653198	0.420976	0	0.230872	1.068	1.70955	0.561617	0	0	0.942565	0.8227	0.353187
ENSG0000	IGFLR1	19:357180	41.2503	41.5727	105.048	84.3444	66.6293	103.29	164.597	146.253	139.579	42.7776	0.1407	0.749613
ENSG0000	UQCRC2	16:219526	124.45	119.645	101.101	111.402	82.0948	103.832	100.194	74.6854	64.9637	46.3715	0.1482	-0.49307
ENSG0000	TRBV25-1	7:1426707	3.79449	6.13161	6.36187	5.87373	3.4606	0.887079	1.70655	9.64835	1.17763	0.482389	0.59775	-0.64137
ENSG0000	TMEM191	22:185278	0.735741	2.37308	1.819	1.67213	0.613664	0.831905	3.97146	1.11118	0.519434	2.17586	0.9068	0.133388
ENSG0000	RP11-161	16:991150	0.226728	1.35371	0.566083	0	1.09813	0	0	0	0	1.12191	0.50545	-1.18051
ENSG0000	ATP5O	21:335039	176.064	167.138	234.045	223.234	126.643	206.42	245.93	189.483	200.819	28.4623	0.88995	-0.10742
ENSG0000	RP11-266	16:250855	2.68129	0.425767	5.27621	4.40971	2.02983	3.7208	1.50165	1.66373	2.93954	1.06395	0.7922	-0.55251
ENSG0000	AFAP1	4:7754089	0.658922	0.546672	0.201051	0.520447	0.222391	0	1.59466	0.752054	2.74053	1.09952	0.08055	2.00673
ENSG0000	RNASET2	6:1664093	156.704	202.858	269.264	228.316	219.107	249.156	150.54	220.604	255.867	133.326	0.73695	-0.10968
ENSG0000	RP5-1142.	16:887064	0.502858	0.597832	0.156049	0.183745	0.324378	1.64115	0.606267	1.12377	0	0.498759	0.39295	1.12722
ENSG0000	TREX2	X:1534447	0.302764	0.87524	0.598286	0.218282	0.460247	3.18786	3.20899	0.541281	0	0.794464	0.143	1.57718
ENSG0000	OCIAD1	4:4880521	98.5433	84.6112	69.8037	55.5958	58.7781	95.1031	108.916	76.724	46.06	44.8385	0.8641	0.055449
ENSG0000	RNF11	1:5123627	14.0488	9.32499	12.9829	19.3355	12.3626	11.207	10.8078	14.8708	22.4493	17.6428	0.687	0.141724
ENSG0000	CCDC102	18:686736	1.41686	1.34031	1.245	2.18911	2.85853	0.565306	0.293927	1.0847	0	1.96291	0.1751	-1.2893
ENSG0000	RP11-48B	17:366289	0.1332	0.083629	0	0.252547	0	1.00976	0.381825	0	0.268164	0.220676	0.2643	2.08891
ENSG0000	RP11-517	10:434207	1.17535	1.66434	1.52825	2.35633	2.4389	0	2.69979	0	0.451825	0.629535	0.4751	-1.42821
ENSG0000	KIN	10:775096	6.40136	12.0008	7.5356	3.25692	5.2908	9.11937	13.6575	9.92218	8.66792	3.78003	0.5425	0.382494
ENSG0000	MBOAT4	8:3009539	0.971387	3.15174	1.89173	2.34135	0.441347	0.515142	0.104125	0.122741	2.00458	1.04077	0.40215	-1.13436
ENSG0000	ATRIP	3:4844670	4.53514	2.98024	5.04449	3.64737	1.71869	5.15777	3.05247	3.87402	3.20093	5.83096	0.7692	-0.25299
ENSG0000	INAFM2	15:403252	1.10134	1.14535	0.60891	0.793459	0.612572	3.69044	0.889534	0.073529	4.04126	1.05903	0.63675	1.25263
ENSG0000	VAMP1	12:643900	29.9389	53.2763	31.0753	13.0094	13.7008	24.8435	34.211	33.0988	9.43089	33.5009	0.95135	0.047138
ENSG0000	GBP5	1:8925894	242.137	72.6548	149.319	145.871	189.736	174.219	207.48	165.938	131.934	260.936	0.67675	0.157719
ENSG0000	LINC0100	7:1049410	1.60471	0.786016	1.33185	1.42377	0.136258	0	2.72223	0.797095	0	0.230224	0.82265	-0.35201
ENSG0000	AK6	5:6935098	54.33	38.7193	51.8502	48.5353	29.4613	61.9878	57.858	37.9	58.5079	11.3805	0.8909	0.095114

ENSG0000	SUMO2P1	6:2963605	0.277986	0.176691	0.196516	0.487313	0.21297	0.234718	0.655634	0	0	0	0.728	-0.66307
ENSG0000	TINF2	14:242396	75.068	90.1466	102.334	93.4825	78.8483	77.8484	87.6141	80.7438	70.3111	80.4539	0.6234	-0.15987
ENSG0000	FAM120A	9:9344649	14.5502	15.5218	12.5866	12.0493	27.2827	13.4148	17.3315	19.3028	12.2768	44.7723	0.38115	0.462227
ENSG0000	SPCS2P4	1:2809574	7.53476	5.95301	6.0329	6.783	3.05174	10.1853	10.1971	3.13244	5.79001	2.02127	0.76715	0.22157
ENSG0000	TOP3B	22:219194	30.4221	41.0328	8.1472	13.4354	8.99231	19.7326	24.9096	12.9911	10.5956	11.7617	0.2949	-0.42671
ENSG0000	PACS2	14:105209	12.287	13.1336	2.86604	3.64188	4.00752	9.07556	7.52939	6.79015	3.3404	9.93851	0.78435	0.225091
ENSG0000	CECR5-AS	22:171375	0.692067	0	0.084977	0	0	0	1.19533	0	2.19769	0.526484	0.21195	3.09487
ENSG0000	RASA2-IT1	3:1414870	0.1057	0.69984	0	0.099959	0.077814	0.275975	0.476917	0.432964	0.228289	0.34554	0.4609	1.00439
ENSG0000	UBE2E2	3:2320301	1.55394	9.57717	0.733596	6.55163	0.472555	23.0585	1.79485	2.98449	1.40814	0.039623	0.6145	0.600251
ENSG0000	CNN2P9	6:1108582	0.418651	0.911644	0.31767	0	0.88608	0	0	0	0	0.445769	0.20445	-2.33028
ENSG0000	MKRN2	3:1248443	28.8608	24.2456	39.5901	26.7097	22.3309	15.1155	16.0574	23.5664	14.4123	21.5486	0.1895	-0.76187
ENSG0000	ANAPC1	2:1117662	12.1803	9.94873	8.93247	14.7309	7.5694	10.1219	15.2796	9.22096	7.83009	3.13628	0.8297	-0.31282
ENSG0000	RP11-2B6	9:9416628	0	0.194277	0.429395	2.93196	0.084262	0	0	0.175301	1.74131	0.470562	0.69215	-0.72647
ENSG0000	CENPC	4:6746874	23.9696	24.7933	12.2889	12.4166	12.1094	17.369	26.9314	12.0142	10.1632	22.1373	0.9934	0.003031
ENSG0000	ZFYVE27	10:977371	18.5681	18.8474	15.5777	19.5306	9.54621	8.58649	18.4583	25.1055	17.6889	19.4078	0.63955	0.176164
ENSG0000	NDUFAF1	15:413873	22.0406	20.5165	32.0158	24.3077	22.8919	36.01	19.385	23.2034	27.0079	16.875	0.95225	-0.0272
ENSG0000	SAT2	17:756187	41.159	42.458	32.4235	24.9769	29.5097	39.2678	50.9586	29.7963	22.714	18.9326	0.90255	-0.09821
ENSG0000	PSMA3	14:579997	113.196	104.522	119.457	111.272	81.4863	146.363	180.909	143.155	134.538	59.4959	0.52245	0.348483
ENSG0000	LDLRAD4	18:132174	9.09621	14.9429	6.19971	9.89964	2.718	49.8209	69.1001	30.2199	27.6949	20.2599	0.0005	1.98562
ENSG0000	SIAH2	3:1507411	42.3375	36.6073	24.3163	69.7875	25.2183	37.4589	50.8689	29.2484	56.3545	22.3602	0.9241	0.034528
ENSG0000	B4GALT4	3:1192117	20.736	21.4337	15.6259	14.2312	9.25916	11.5618	10.8882	14.5848	8.52173	9.73623	0.12235	-0.60505
ENSG0000	FCRL6	1:1598005	2.84107	31.6242	20.2737	23.4808	11.0613	8.46495	32.8134	26.4675	3.91036	15.6444	0.81135	-0.10787
ENSG0000	AEBP2	12:194040	11.9907	7.90784	8.47146	7.71645	6.45613	6.54902	9.88756	10.1296	5.05044	9.29075	0.86605	-0.07213
ENSG0000	THOC2	X:1236005	44.8576	56.8743	26.8224	24.2964	28.877	49.5534	41.9349	29.5022	33.8118	67.1526	0.28215	0.365829
ENSG0000	PPBP	4:7398703	0	0	36.1632	167.155	13.4901	0	0	0.162595	0.10081	0	0.1111	-9.62461
ENSG0000	PA2G4	12:560798	84.8446	78.4659	123.587	91.7246	139.851	117.858	149.001	131.116	83.7164	101.944	0.9174	-0.03891
ENSG0000	RP11-49K	18:471059	0.666789	1.27302	0	0	0.070795	0	2.4188	0	0	1.02139	0.71125	0.984568
ENSG0000	ZNF316	7:6637321	6.70698	9.05449	0.851388	1.06228	3.09106	1.71507	3.37434	1.63878	0	4.51432	0.2415	-0.87485
ENSG0000	CTC-490G	19:433292	1.09169	2.35748	0	0	0.450676	0	3.94124	0	0	0.300401	0.9559	0.030888
ENSG0000	HIST1H1PS	6:2619555	0.849784	0	5.56024	0.241091	0.457112	0.106746	0.456874	0.312345	0	0.250181	0.17115	-2.8093
ENSG0000	NPIPB5	16:223459	18.56	41.5584	6.65617	7.00221	4.53092	29.7143	34.6538	11.2108	14.4056	18.4633	0.4184	0.558798
ENSG0000	PILRB	7:1003077	114.301	68.8602	51.4005	45.0936	50.0428	62.5106	39.1897	21.7765	28.7287	48.5982	0.33355	-0.62961
ENSG0000	FGF7P6	9:6237412	2.78517	2.21501	5.16961	3.51959	3.60711	2.67459	0.07163	2.35704	3.64617	2.02242	0.43855	-1.55566
ENSG0000	RP11-112.	9:3574928	0.789481	0.686593	0.368825	0.155363	0.700028	0	1.32558	0.711198	0.35004	0.544057	0.82675	-0.39865
ENSG0000	RP11-465	1:2019823	1.03478	1.56815	1.49741	0.486981	0.342417	0.291142	0.370993	0.822853	0	0.616382	0.3998	-1.11739
ENSG0000	RP1-199J3	6:9957571	0.546193	0	1.51412	3.08216	0.521163	3.99143	0.670713	0.375879	0	0	0.872	-0.29453
ENSG0000	AF196970.	X:4869555	0.154446	0.411373	1.4784	1.01105	0.101313	0	0.517996	0.126974	0	0.322717	0.25505	-2.35396
ENSG0000	ARHGAP3	1:1610469	88.6917	104.073	80.7321	52.5309	120.484	59.4563	79.88	89.8715	50.7113	205.831	0.95455	-0.02215
ENSG0000	CBX5	12:542309	8.05179	10.0261	4.77034	4.49008	8.23215	4.00967	11.2825	10.0344	4.80881	10.0836	0.9364	-0.06071
ENSG0000	PAPSS2	10:876079	0.22686	0.56714	0.080508	0.193903	0.135025	5.00435	0.145674	0	0.659246	0.175144	0.17515	2.25527
ENSG0000	RP11-23P	15:418280	26.7271	16.2547	2.34141	37.4996	6.44302	18.5882	18.2816	4.33153	14.1272	24.7039	0.81645	-0.19039
ENSG0000	DHRS13	17:288977	15.2366	10.3074	19.6288	14.4511	16.8044	11.188	8.29573	15.1177	13.8804	13.8646	0.4377	-0.37198
ENSG0000	TSC2	16:203981	34.3045	39.5222	8.69964	7.96536	13.2227	35.8307	18.966	19.2929	9.54014	18.3978	0.72	0.2017
ENSG0000	WDR78	1:6681288	0.172456	1.7609	0.605073	0.13135	0.159508	3.92514	0.098443	0	0	0.038108	0.73085	0.302322
ENSG0000	TNFAIP8L1	19:463951	3.4517	6.35468	0.596672	2.04034	1.36806	2.61929	3.74007	3.13492	0.739856	3.30709	0.98125	-0.04501
ENSG0000	HMOX1	22:353803	4.32586	7.59487	0.392213	5.2739	1.3189	121.24	6.16154	2.69098	3.46156	1.79992	0.0011	2.7253
ENSG0000	MMGT1	X:1359620	3.40117	3.47515	2.75537	1.45701	2.97407	3.13815	2.93277	4.85	4.7916	5.08363	0.3834	0.562199
ENSG0000	DHX57	2:3879772	2.98354	5.2569	1.08106	1.56606	1.80026	6.37855	2.56441	2.91025	1.44673	3.15564	0.3694	0.519169
ENSG0000	TAF11	6:3479201	26.1838	26.1456	22.8275	24.6041	22.1842	25.4214	22.2051	20.1997	26.9152	17.6097	0.82315	-0.10122
ENSG0000	CD40	20:461182	0.339531	4.00672	1.89729	3.54167	2.11734	6.5799	1.81396	0.762687	0.520555	0.992608	0.7611	-0.21651
ENSG0000	AF127577.	21:148188	2.55944	0.586153	1.24283	1.51916	2.06326	0.139845	0.577951	2.20984	0	1.53124	0.67855	-0.62458
ENSG0000	NXPE3	3:1017792	8.9977	26.37	5.72023	8.94531	7.86732	12.8605	12.5998	7.3469	9.01555	15.5753	0.6767	-0.171
ENSG0000	AP2M1	3:1841746	236.797	218.566	176.706	139.116	228.638	301.104	237.822	164.128	107.819	161.959	0.8445	-0.06399
ENSG0000	VPS9D1-A	16:897071	0.425662	0.522524	2.05525	2.04768	1.9611	0	0.50618	0.973274	0	2.08681	0.41655	-1.22569
ENSG0000	PCGF3	4:705747-	9.56633	25.1395	9.79135	4.66109	10.9621	9.41994	25.9066	17.8824	15.3236	26.513	0.08835	0.754464
ENSG0000	RGL4	22:236084	45.738	33.5177	82.2634	82.0253	33.7883	78.2744	95.352	70.3285	83.1081	21.8505	0.613	0.302672
ENSG0000	TBP	6:1705543	30.9817	40.1084	42.4737	42.5154	27.112	34.649	18.5157	23.4822	31.5216	29.3198	0.1659	-0.54361
ENSG0000	ANKRD54	22:378308	14.2828	13.3339	18.1985	17.1871	16.0898	9.18683	16.8061	15.7993	17.9249	15.791	0.8714	-0.16194
ENSG0000	RPL5P34	22:427764	0.420081	0.180312	0.935772	0.965742	0.393448	0.140449	0.154183	0.363467	0.707487	0	0.5598	-1.05238
ENSG0000	6-Sep	X:1196157	79.4507	160.34	64.2292	30.1968	99.8324	120.948	118.935	53.8686	35.4167	153.764	0.90695	-0.05013
ENSG0000	WI2-1896	1:1491760	0.060175	0.096316	0	0.063007	0.054017	10.5065	0	0.80655	1.50873	0	0.17885	5.5977
ENSG0000	PRSS27	16:271241	0.870707	1.48135	0	0.10844	0	0	0.101836	0.484362	2.49207	0.377612	0.507	0.958658
ENSG0000	SLC29A3	10:713192	6.03578	6.03319	10.9919	7.09822	14.874	9.05849	7.58818	10.4638	4.00864	12.0739	0.65485	-0.21961
ENSG0000	RSBN1	1:1137618	17.3497	17.3929	14.5448	18.0775	20.4516	11.9967	9.62921	10.7645	16.0101	18.5713	0.63485	-0.54957
ENSG0000	MCM5	22:354000	35.6004	57.2802	27.5628	26.1847	31.2584	44.8688	43.4159	31.2151	19.0637	43.3341	0.6991	-0.13487
ENSG0000	PEF1	1:3161768	69.1417	53.121	113.505	101.503	93.7243	77.2619	75.7579	74.0859	90.3135	57.7984	0.45175	-0.24458
ENSG0000	RP11-147	17:681885	0.24943	0.476256	0.953069	0.066239	0.753234	0	0.385034	0.678769	1.34032	3.99002	0.51025	1.28573
ENSG0000	RP11-486	1:8407633	1.15158	2.87318	1.07673	0.488513	0.90795	1.35821	3.90126	0.797845	0.123162	0.828567	0.8147	0.16141
ENSG0000	EPHA4	2:2214180	3.21208	4.33891	2.91237	3.43233	2.69627	3.82946	0	4.46754	4.35788	6.69263	0.77925	0.216009
ENSG0000	CISH	3:5060648	45.7678	32.9491	37.9945	55.3259	87.088	77.8044	127.979	130.689	77.6094	87.0464	0.05325	0.879371
ENSG0000	EIF4EBP2	10:704043	23.8564	21.316	16.2968	4.85986	14.706	12.7243	12.3145	12.6942	8.21111	23.6798	0.3317	-0.32167
ENSG0000	IGHV3-43	14:106470	0.107301	0.067314	1.64161	8.11843	0.083369	0	0	0	0	0.107209	0.2767	-5.86593
ENSG0000	PPP3CB-A	10:734364	14.1454	12.8995	9.40308	8.8278	4.76297	4.76737	4.11184	5.29064	10.4251	4.36771	0.2828	-0.7648
ENSG0000	HIST1H2A	6:2619555	2.73301	1.73513	71.3824	29.8534	18.5113	2.02174	1.98336	34.203	11.2391	3.50879	0.55505	-1.19222
ENSG0000	LINC0100	7:1564721	2.06544	3.63546	0.144577	9.18563	2.81417	14.7339	14.538	2.0986	8.73718	4.68289	0.30465	1.15431
ENSG0000	TREM1	6:4126792	1.35417	5.13243	0.370353	0.426371	0.350463	6.34935	1.21895	0.820942	5.85342	0.726812	0.2739	1.17919
ENSG0000	RN7SL364	19:463476	0	0.143685	0.530042	0	0	0	2.07307	0	0	0.228281	0.5604	1.62966
ENSG0000	ZNF143	11:945955	19.0728	26.6472	16.0796	24.804	16.6159	20.016	25.5347	12.3948	4.15056	14.1913	0.23535	-0.46719
ENSG0000	PWWP2A	5:1600091	16.7164	27.4931	9.50683	12.2145	29.6771	11.9239	29.7531	11.5732	17.7261	23.4208	0.96505	-0.03995
ENSG0000	TOR4A	9:1372777	1.74479	0.944976	0.354079	0.579803	0.800425	1.58858	0.70352	0.157443	1.21005	1.41462	0.73645	0.253263
ENSG0000	PMS2CL	7:6710127	6.75068	7.14386	7.12733	5.61687	3.68337	6.9206	7.49409	4.99402	11.0303	7.59274	0.62425	0.414911
ENSG0000	APPL1	3:5722773	8.80386	7.32001	11.9272	9.621	30.5466	6.62203	11.4414	10.9684	9.99141	27.9524	0.50055	-0.38811
ENSG0000	NAA30	14:573905	8.93211	5.94377	1.90296	2.2059	2.69764	10.5263	4.39702	4.81011	3.04112	4.87454	0.74405	0.214683
ENSG0000	LINC0032	17:822064	12.4334	13.5713	18.0909	20.0591	22.3113	10.6061	12.726	9.60664	15.3607	11.8288	0.16375	-0.5776
ENSG0000	THADA	2:4322957	10.0512	12.9887	2.48549	3.6318	2.48381	7.2203	13.0472	10.4515	4.81423	8.4491	0.19165	0.586423
ENSG0000	CTD-3138	19:582285	1.22141	0	1.21295	0.876983	1.98274	1.00991	0.749529	0	1.38256	0.888898	0.55175	-1.33744
ENSG0000	METTL12	11:626468	12.5629	4.96	11.5177	14.9236	5.20738	0	14.9936	12.8547	16.9898	5.68908	0.91115	0.142817
ENSG0000	RAB39B	X:1552582	4.37574	3.29264	4.40014	2.47986	3.33603	6.57706	2.91317	5.79891	1.85706	5.25956	0.6102	0.247935
ENSG0000	RP11-11N	8:3299617	1.10744	0.554562	0.52776	0	0	0	0	0	4.04039	0.127722	0.4566	1.47872
ENSG0000	COPG1	3:1292496	47.8448	47.5841	32.9907	31.8341	27.8998	17.8531	24.9696	25.2699	22.7856	31.5389	0.05775	-0.6064
ENSG0000	MIR181A1	1:1988074	7.17128	11.6098	1.60567	1.44564	0.574844	11.6142	3.64368	0.881804	0.021013	1.55606	0.75805	-0.21554

ENSG0000	FARP1	13:981425	0.229255	0.191015	0.130845	0.221298	0.047069	0.540642	0.711005	0.22055	0.194708	0.71101	0.01215	1.93175
ENSG0000	TBC1D7	6:1271680	20.3884	27.254	19.7197	22.8481	13.2946	22.6059	18.898	21.3116	18.2786	12.4142	0.89655	-0.06533
ENSG0000	RP11-599	17:814047	18.6043	32.8242	52.7285	43.4138	89.0624	32.902	40.7171	30.5677	33.6554	55.3326	0.7377	-0.48884
ENSG0000	GPR150	5:9562007	2.25073	2.03806	3.41355	4.29072	3.32202	0	0	1.12446	0.583423	3.7075	0.3424	-1.29173
ENSG0000	C2orf81	2:7419856	0.102331	1.78949	1.39745	1.16345	0.835094	0	0.220133	1.05963	0	0.95588	0.3728	-1.22612
ENSG0000	SRSF2	17:767268	187.354	292.806	196.388	284.64	213.542	225.172	267.476	279.825	263.089	278.36	0.71095	0.150393
ENSG0000	TUFM	16:288424	107.534	109.289	145.193	158.653	136.509	166.234	126.202	145.092	114.575	99.6719	0.8363	-0.06852
ENSG0000	NDFIP2	13:794773	6.78142	3.33029	0.602498	0.541415	0.70949	21.2695	40.4439	25.8437	7.6181	6.07259	0.00015	3.13219
ENSG0000	FKBP9	7:3295740	0.236612	0.21807	0.02922	0	0	3.46862	0	0	0	0.2779	0.1078	3.62408
ENSG0000	AC007098	2:6267385	0	1.38821	0.058465	0.885733	0.405071	0	0	0	0	0	0.1398	#NAME?
ENSG0000	RP11-252	16:752936	0.174542	0.676768	0	0	0.261328	1.95031	0.38658	0.275932	0	0.321132	0.4683	1.3995
ENSG0000	CCNYL1	2:2077115	0.945863	1.18496	0.705435	1.40344	0.885686	0.296128	1.51621	0.065241	0	2.03041	0.4801	-0.76715
ENSG0000	DNMT1	19:101333	82.1562	126.642	79.2087	62.9777	157.786	112.03	122.193	54.693	57.299	122.797	0.5519	-0.3155
ENSG0000	TNFAIP8	5:1192686	188.86	176.171	178.478	187.966	156.009	154.241	95.238	123.916	145.989	106.045	0.14705	-0.56478
ENSG0000	SNRNP48	6:7590198	4.4851	4.77088	4.33036	6.24009	7.65976	7.40805	4.25863	5.73465	5.72149	6.32102	0.8235	-0.20654
ENSG0000	IFNGR1	6:1371974	216.956	45.4425	141.366	149.276	111.084	144.783	36.5103	92.9688	103.624	86.9368	0.1058	-0.55508
ENSG0000	ATP6V1E1	22:175921	96.6553	77.9682	117.85	104.804	102.094	105.732	115.253	89.7919	105.763	48.8808	0.7849	-0.08981
ENSG0000	SIPA1	11:656380	213.72	135.084	76.4503	116.595	85.8491	81.8015	84.9913	64.852	43.6956	92.0268	0.02345	-0.74135
ENSG0000	RPL6	12:112405	279.008	244.065	769.079	462.519	780.262	320.585	339.198	523.053	440.489	134.149	0.17015	-0.59448
ENSG0000	NDOR1	9:1372056	1.39037	0.964357	0.946806	0.533998	3.90792	0.584758	2.00636	1.74895	0.05683	2.00621	0.8904	-0.22248
ENSG0000	RP11-22B	12:927723	0.987824	4.14931	0.15065	0.82915	0.1991	0.889749	1.83432	0.597812	0	0.552684	0.5284	-0.66761
ENSG0000	MED20	6:4190535	7.05698	2.43492	6.63929	7.49325	4.38112	3.98651	5.59236	4.61104	9.5551	6.23168	0.839	0.094632
ENSG0000	FTH1P22	1:1167751	1.59833	0.162885	0.191583	0.125264	0.461914	5.07234	6.3401	3.08484	5.64661	1.31454	0.23255	3.0916
ENSG0000	VIM-AS1	10:172142	11.8365	8.41654	19.6732	15.516	22.7097	8.28144	20.1107	16.6365	22.2214	25.3777	0.8745	0.151099
ENSG0000	SRP19	5:1127074	95.4944	43.6368	84.403	67.4918	43.7935	62.8672	85.592	58.3132	56.9486	18.4974	0.77125	-0.22123
ENSG0000	RP11-582	8:2244081	1.30386	1.00677	1.00412	1.14068	0.524568	0.335476	2.42948	0.268433	0.120114	1.19165	0.85655	-0.29801
ENSG0000	ABRACL	6:1390286	309.477	214.673	316.901	290.007	147.758	398.233	257.727	323.544	315.342	77.8374	0.7573	0.102014
ENSG0000	LRRC56	11:537526	0.721719	2.25127	0.016753	0	0.206297	1.40782	1.36834	0	0	0.496329	0.97455	0.072996
ENSG0000	DESI1	22:415980	6.66466	7.68729	7.37615	5.91357	8.27561	13.4111	18.5038	14.2629	5.06759	8.35114	0.1621	0.543447
ENSG0000	TBCC	6:4274448	98.9734	108.03	155.362	131.338	117.204	118.574	166.645	134.779	80.2365	67.6717	0.56185	-0.18325
ENSG0000	M6PR	12:868159	208.708	191.2	162.762	194.97	137.741	237.874	123.073	136.918	167.159	139.637	0.56045	-0.18615
ENSG0000	FSCN3	7:1275806	1.55111	1.73553	1.07507	0.605269	0	0.386599	0.462293	0.13384	0	0.37079	0.8947	-0.17203
ENSG0000	CNEP1R1	16:500244	16.1014	13.9757	12.2066	12.0026	8.14564	23.7513	20.9116	20.0277	11.1901	9.87225	0.20665	0.471728
ENSG0000	AC092155	2:6253258	1.4591	0.468507	0.801692	0.428972	0	0.858206	0.40578	0.188278	0.084938	0.207945	0.70175	-0.63476
ENSG0000	GPR25	1:2008729	16.9732	27.4932	12.7279	23.2808	11.1242	164.954	193.645	197.789	71.7351	202.204	0.00005	3.06948
ENSG0000	IL13	5:1325414	0	0	0	0	0	0	0	7.37863	0	0	0.03335	inf
ENSG0000	STAB1	3:5249533	0.910214	3.96697	0	0	0.062327	9.62695	0	2.17834	0.111055	0.492488	0.3599	1.94065
ENSG0000	PANK4	1:2508532	28.1825	44.3093	31.8361	44.4883	22.7859	17.1164	25.256	29.9112	23.1867	34.2406	0.27055	-0.38365
ENSG0000	HSPA7	1:1616062	0.425717	1.28267	0.718323	0.437217	0.332478	5.20752	1.38348	1.49615	23.0371	8.16154	0.16385	3.41195
ENSG0000	GTPBP6	X:304528-	52.402	41.1975	51.5848	44.6013	67.3635	29.1552	54.3824	60.4406	44.8056	49.204	0.7331	-0.12253
ENSG0000	SKIL	3:1703576	3.22702	3.05874	2.0328	3.05482	1.99503	12.5081	10.4507	11.2318	13.7012	14.2228	0.0002	1.97902
ENSG0000	EMILIN1	2:2707856	1.06307	1.78307	0.446799	0.37855	0.183873	0.324432	0.788502	1.42003	2.62097	0.441951	0.653	0.903927
ENSG0000	TAF10	11:660364	138.184	114.546	68.105	32.9776	97.0994	146.261	145.071	64.5954	52.0306	53.4891	0.91135	-0.08741
ENSG0000	RP11-617	15:900013	1.09386	5.20017	1.01003	1.42487	1.67515	2.07679	2.68506	0.980644	0.518881	3.30314	0.9362	0.103628
ENSG0000	RICTOR	5:3884585	8.27811	11.7497	5.53321	5.95941	3.07516	15.8878	15.8788	6.60191	12.3967	14.0879	0.1151	0.818303
ENSG0000	RP11-345.	16:294257	27.0086	26.2697	40.5089	24.3725	17.0323	35.2288	38.7005	34.373	41.2383	14.704	0.6584	0.245632
ENSG0000	PHKG1	7:5606400	0.336122	0.591928	0.42333	1.26033	0.28654	0.516251	1.07649	0.176507	0.213615	0.559663	0.77925	-0.28246
ENSG0000	KCNJ15	21:381214	0.54696	0	0	0	0.053833	2.07312	0.43474	0	0	0	0.25895	2.01113
ENSG0000	UBXN4	2:1357416	99.4615	109.196	80.5616	85.3374	112.788	121.761	111.984	95.2341	107.283	171.681	0.47515	0.301935
ENSG0000	EMC8	16:857717	21.8068	24.9812	32.5426	32.4786	23.1064	14.3246	22.9101	24.7718	16.4071	17.8476	0.53865	-0.5297
ENSG0000	RPS4XP16	13:513357	0.543586	1.12765	1.00666	1.64343	0.156363	1.73618	0	0.068775	2.40433	1.85491	0.7885	0.425865
ENSG0000	LINC0026	7:3973363	1.984	2.30356	0.625007	0.87824	0.169908	1.15959	4.30681	1.08746	0.892921	0.54159	0.5428	0.420248
ENSG0000	HMGN1P8	3:1676832	0	0	0.321139	0	0	0	0.624528	2.16667	0	1.17144	0.2967	4.10891
ENSG0000	REEP5	5:1127074	65.879	43.1715	70.055	67.5417	55.0643	75.0463	79.3387	92.0269	69.7252	45.4069	0.6858	0.172182
ENSG0000	TUBE1	6:1120540	23.5147	15.2021	15.5076	14.4404	7.78149	19.6392	15.0607	13.7852	11.3598	10.8541	0.81995	-0.09379
ENSG0000	NELFB	9:1372551	6.06859	5.18363	3.73472	1.41784	9.8528	5.89824	4.98974	6.70388	1.58486	11.8444	0.7497	0.177261
ENSG0000	MRPL3	3:1314622	68.557	47.3862	69.9823	56.758	58.5491	59.1222	58.7511	55.403	70.994	36.3141	0.60895	-0.16047
ENSG0000	CNIH1	14:554235	25.9395	22.2401	37.1692	21.8052	18.904	55.6392	70.3614	41.8013	48.7699	7.7699	0.1955	0.554642
ENSG0000	TJP3	19:370810	3.19408	3.76784	3.78297	3.31497	1.97416	0	1.55318	0.701648	0	1.53474	0.06475	-2.14901
ENSG0000	JAK2	9:4984389	9.06879	8.12759	1.90426	2.42831	1.64484	3.12204	4.68114	1.6675	8.44464	3.41741	0.68695	-0.18153
ENSG0000	ZNF707	8:1436844	2.75212	1.68069	3.51698	5.95218	3.69208	1.87253	11.5954	7.69539	2.72439	5.49968	0.47425	0.508921
ENSG0000	ITGAV	2:1865900	1.70217	1.96577	0.231527	0.124642	0.502719	1.53754	2.09439	0.649593	0.456442	1.17551	0.2996	0.7144
ENSG0000	BLNK	10:961297	0	12.5588	1.40526	4.4186	1.65893	2.75668	0	0	3.48702	0.862499	0.331	-1.52627
ENSG0000	AHCYL2	7:1292250	2.65538	5.54676	1.03911	2.81497	1.10059	2.17951	2.61608	2.73812	1.25396	2.96247	0.83305	-0.10669
ENSG0000	OR52K3P	11:447481	0	1.40701	0	0	0	3.97332	0.209982	0	0	0	0.2517	1.61617
ENSG0000	BTBD2	19:198543	22.4922	19.8262	3.94027	4.60683	8.50829	11.2768	18.4723	6.99797	1.49016	17.1902	0.8642	-0.09322
ENSG0000	TMEM167	1:1090879	21.7521	11.4938	8.79079	28.0517	7.49429	24.1323	15.7441	10.7452	29.8685	8.15438	0.58965	0.211288
ENSG0000	GLS	2:1908807	37.0136	30.5831	13.0058	10.9107	19.9286	32.3978	31.9262	25.0931	21.1077	37.5775	0.62045	0.459968
ENSG0000	NDUFA12	12:948970	223.883	207.561	236.082	268.717	136.32	199.455	180.391	183.448	169.786	24.113	0.11935	-0.50737
ENSG0000	FBXO34	14:552627	17.612	23.5325	17.516	19.8578	22.6797	12.5978	15.9762	11.0988	17.1126	21.6505	0.1281	-0.59634
ENSG0000	FBXW7	4:1523212	20.7876	23.7815	10.0916	8.0033	15.6338	13.584	14.6706	12.3256	17.6376	25.8943	0.5883	0.219715
ENSG0000	LYPLA2	1:2379096	51.5804	53.9988	98.649	89.601	110.13	46.0299	67.8902	82.7885	51.3343	84.1846	0.2843	-0.35522
ENSG0000	HLA-DQB	6:3275609	0	1.1263	0	0.907755	0.462547	0.415463	1.20972	0	0.59574	0.433402	0.9301	-0.11762
ENSG0000	PAQR8	6:5236142	10.3002	21.1626	5.67232	3.95806	6.98882	6.26318	6.51778	6.85216	4.59853	11.4483	0.21705	-0.57743
ENSG0000	RAB4B-EG	19:407716	1.02422	0.979225	1.36051	1.9755	2.85627	1.27838	2.39363	0.91746	0	2.53501	0.67965	-0.76836
ENSG0000	CD300A	17:744664	64.2976	23.8379	199.587	122.121	82.4453	48.5569	21.5586	36.2448	28.4073	36.3572	0.0002	-1.57785
ENSG0000	LYRM4	6:5102592	8.03374	11.6836	9.69446	13.2491	5.81633	12.9099	13.2481	14.2453	11.214	3.03827	0.8941	0.093204
ENSG0000	ACTR6	12:100156	51.2375	36.0243	36.315	34.975	33.2155	48.0105	47.4028	28.1957	9.32897	20.3339	0.44505	-0.30667
ENSG0000	RPIA	2:8869164	23.0484	20.5476	14.1307	32.0823	13.1287	19.9338	26.9522	14.0255	23.5907	7.4462	0.64915	-0.1756
ENSG0000	CAPN12	19:386476	14.3568	3.33954	34.6657	34.8254	35.9109	16.7622	7.40196	13.6895	20.278	19.5045	0.3388	-0.73595
ENSG0000	PI4KA	22:207076	32.9984	38.7897	13.5519	13.301	24.0726	27.1088	17.2222	12.0999	11.8719	27.727	0.97765	0.015559
ENSG0000	PPP4R1L	20:582289	2.72287	5.17655	2.04466	3.71305	0.814251	2.69061	2.31999	0.409195	1.06016	2.75604	0.26415	-0.83873
ENSG0000	GPATCH3	1:2689048	13.442	16.1319	16.9689	14.9866	16.1215	2.35346	10.5379	12.1874	5.98972	15.4205	0.47915	-0.7592
ENSG0000	MAP6D1	3:1838158	0.788513	0.162782	2.7386	1.6531	1.11732	0	0.227926	0	0.999006	0.32545	0.11105	-2.11486
ENSG0000	RNF149	2:1012712	122.432	110.521	131.812	127.202	90.8483	165.744	131.925	144.048	158.117	107.738	0.39195	0.263705
ENSG0000	CTAGE5	14:392302	49.9719	37.8953	15.5613	18.5735	14.286	35.891	30.8112	13.6049	8.8133	22.7309	0.6442	-0.188
ENSG0000	SUMO2P1	17:488748	0.086885	0.682148	0.436406	0.269101	0.072514	0	0	0.081587	1.98632	0.279135	0.4581	1.56707
ENSG0000	DBI	2:1193669	267.525	170.044	309.776	294.706	157.466	410.201	317.603	282.069	277.431	42.164	0.7743	0.095778
ENSG0000	TADA2B	4:6909241	17.9688	17.0822	4.29279	2.55309	7.28724	17.2193	7.25051	3.26977	6.58766	8.55134	0.589	-0.4623
ENSG0000	POLR2M	15:575919	27.5889	21.0301	9.97117	13.8673	10.6731	12.8208	18.6074	14.7796	7.94274	11.127	0.18195	-0.60203
ENSG0000	TTC7A	2:4689927	18.0925	18.6025	2.97997	3.0075	4.85967	15.125	11.1132	10.1858	5.39924	8.36971	0.9089	-0.12048

ENSG0000	CTC-459F	19:276845	0.907332	0	0	0	0.519654	0	2.08628	1.41642	0	0.564581	0.4313	1.86555
ENSG0000	SNRPD2	19:456874	372.907	200.899	700.654	566.313	168.107	456.226	481.168	538.733	376.298	48.8134	0.69865	-0.15912
ENSG0000	SIGLEC7	19:511422	4.18934	3.46112	2.62703	5.14136	5.67899	8.39121	0.645903	0.454974	0	2.99646	0.451	-0.73915
ENSG0000	PDIA3	15:437269	247.424	164.478	264.255	253.416	263.938	252.495	262.759	228.722	179.009	202.412	0.74555	-0.20837
ENSG0000	AP1S1	7:1011543	1.7517	0.359804	0.891772	0.889483	2.26048	2.05894	1.30624	2.67007	1.01817	1.173	0.7516	0.357648
ENSG0000	ZNF174	16:336509	16.7745	14.4352	16.3551	11.2882	12.9771	9.2198	3.43038	5.8147	8.49549	10.9293	0.36695	-0.83263
ENSG0000	PHTF2	7:7779879	12.5758	10.0762	3.22457	5.0569	2.26284	30.1186	16.4374	9.60099	17.9175	12.0795	0.0035	1.37858
ENSG0000	SDHAP1	3:1959597	29.6009	51.8429	29.8131	36.6843	11.9	27.9796	30.5563	23.793	48.6176	14.5875	0.84845	-0.10256
ENSG0000	TAOK1	17:293520	1.86977	2.4465	1.70446	1.27577	2.477	3.00858	4.54195	2.31415	4.72419	6.86855	0.0972	0.754409
ENSG0000	CACFD1	9:1334599	1.36672	0.447739	0.569573	0.624263	1.19246	2.09282	2.05373	1.81459	0	1.33885	0.4794	0.675469
ENSG0000	PPM1M	3:5224579	62.1174	68.0529	25.1868	25.0363	24.625	62.5806	60.196	37.2396	50.4301	25.5507	0.5247	0.2138
ENSG0000	C16orf45	16:153957	0.371767	1.5451	0.708333	1.05734	0.588741	0.282714	8.44651	2.10939	3.55857	0.593196	0.10055	1.86981
ENSG0000	TOPORS	9:3254054	13.4891	16.5454	12.3915	13.1277	10.5011	9.5433	10.6055	14.1988	10.2532	15.4319	0.6651	-0.26177
ENSG0000	MFSD13A	10:102461	2.46202	2.83437	4.89787	1.7096	3.12553	1.82743	3.22338	4.89158	4.83047	2.71065	0.7908	0.243134
ENSG0000	FAM168B	2:1310478	10.4952	12.8922	7.2134	3.55088	13.0326	7.65826	9.23767	4.89059	8.09593	16.629	0.88035	-0.09173
ENSG0000	TMEM143	19:483323	14.6658	24.3841	13.8181	18.371	8.89551	11.6535	10.8095	8.23265	4.71456	9.96022	0.1027	-0.78479
ENSG0000	RPL7P9	1:9667887	37.5668	25.6607	58.0151	61.7393	15.9529	32.8596	24.4336	18.1656	36.721	1.86773	0.6057	-0.43519
ENSG0000	P4HB	17:818431	258.051	185.2	347.516	314.625	366.637	256.912	229.523	252.335	258.911	272.068	0.462	-0.23415
ENSG0000	RPS19	19:418599	1432.57	1131.21	3051.74	1832.82	1170.07	1837.74	1627.08	2028.85	1297.28	186.243	0.38165	-0.39954
ENSG0000	G6PD	X:1545313	0.851829	1.18584	6.45149	4.25489	13.4733	1.86311	1.01685	4.57797	3.32279	8.89067	0.578	-0.44459
ENSG0000	ZSCAN30	18:352410	2.78898	8.41141	2.28325	1.68295	1.26653	4.57238	2.56202	3.2135	0	5.0583	0.9071	0.105769
ENSG0000	LKAAEAR1	20:640801	0.236885	0.451354	1.13779	0	1.2472	0	0.986117	1.56103	0	0	0.69315	-0.66452
ENSG0000	RNPC3	1:1035256	18.4585	46.087	25.9514	30.7331	29.9502	18.8726	29.0147	27.5767	25.5584	40.7438	0.65305	-0.2537
ENSG0000	MED4	13:480533	71.5572	65.9053	78.8657	68.7236	58.3117	72.9821	77.1888	67.4686	62.2179	36.5158	0.69555	-0.1408
ENSG0000	ABHD1	2:2712378	0.768504	1.50088	0.74311	0.515842	1.53827	0.498271	0	0.674092	0	0.942979	0.2331	-1.26795
ENSG0000	SYNJ2BP	14:703250	8.4676	7.41789	13.1823	7.75114	8.79906	11.5048	6.75765	5.73181	2.67888	8.80638	0.49505	-0.71041
ENSG0000	PDRG1	20:318476	17.6881	12.4138	20.7461	22.6305	24.8376	15.0536	14.2224	23.3719	15.6941	17.2918	0.5556	-0.21974
ENSG0000	USF2	19:352689	20.2186	24.2285	14.4893	11.8875	27.6905	42.6746	34.7117	34.4052	24.3577	31.5306	0.07595	0.767458
ENSG0000	FBXL20	17:392526	2.31473	5.51868	4.05454	2.3481	2.38137	2.64281	2.93527	2.04296	2.80265	7.10603	0.72365	-0.23985
ENSG0000	LINC0066!	19:363130	2.76408	1.1575	0.667498	1.25621	0.826428	0	2.14082	0.125816	0	1.47821	0.39855	-0.86533
ENSG0000	KPNA4	3:1604949	5.92727	3.28805	2.90453	3.83046	4.40786	4.47912	6.06817	5.31226	4.64677	7.51991	0.2051	0.457974
ENSG0000	ZNF628	19:554759	0.213207	0.137476	0.971475	0	1.48866	0	0.385736	0.285137	0	0.667894	0.36735	-1.29134
ENSG0000	CBFB	16:670291	84.7401	74.1311	31.2879	39.2467	26.4943	82.6917	90.075	70.423	64.7405	51.2146	0.0569	0.651128
ENSG0000	SYNM	15:986489	0.837319	0.005172	0.674701	0.831191	0.037181	0.585516	0.391667	0.548911	1.59853	0.456057	0.60265	0.73112
ENSG0000	EVI5	1:9250869	0.71596	0.767639	0.030164	0.065915	0.060925	1.58244	0.418787	0.102824	0.036434	0.152147	0.97175	-0.03972
ENSG0000	RP11-35G	18:576303	0.768959	0.022025	0.223158	0.821275	0.963522	0.192183	0.153787	0.030987	0	0.676406	0.3626	-1.50086
ENSG0000	LEKR1	3:1568254	1.38977	0.445602	0.703943	0.887574	0.288855	0	0.09959	0.070059	0.887485	0.193764	0.0513	-1.5785
ENSG0000	POLD2	7:4411468	58.0718	62.2554	77.1382	78.7741	80.4872	43.1184	41.5722	49.8354	17.2199	34.4632	0.00275	-1.04745
ENSG0000	WDR7	18:565972	12.3559	11.3543	2.62425	2.71757	2.89938	10.2091	3.88038	0.682802	11.0567	5.03643	0.97275	0.026177
ENSG0000	MT-ND5	MT:12336	28.8634	34.1374	570.494	676.334	1990.45	43.9112	26.7236	627.829	733.801	1706.06	0.76255	-0.33039
ENSG0000	ZNF248	10:377753	4.60249	6.9472	3.43249	2.75651	1.74974	6.33889	3.86901	3.09263	6.19712	4.14726	0.65185	0.221894
ENSG0000	RTN4R	22:202414	8.01435	1.64106	12.9417	9.9487	17.3486	1.19153	1.54134	7.80978	8.73555	17.2777	0.45685	-0.42446
ENSG0000	LINC0089	X:1499295	3.09676	2.42891	0.805499	1.49227	0.310047	0.997486	3.54534	0.94001	0.537132	1.04727	0.4339	-0.6991
ENSG0000	CCL4L2	17:362109	67.0092	22.3932	76.4648	72.7346	30.2302	304.727	770.414	1444.58	207.947	59.3859	0.00005	3.41789
ENSG0000	ZC3H12A	1:3747455	24.021	11.5919	20.7536	41.6042	32.0421	27.4242	31.9627	44.8783	29.4801	41.6878	0.2726	0.377191
ENSG0000	IGHV2-70	14:106723	0	0	0	6.37252	0	0	0	0	1.33801	0	0.1477	-2.00008
ENSG0000	EMC9	14:241361	19.1423	12.6283	21.5417	16.5128	14.2676	54.8472	27.6195	30.6523	25.582	16.3864	0.0821	0.810555
ENSG0000	DHX33	17:528226	0.476533	1.29035	1.57921	1.13711	1.57257	1.7593	1.23057	1.83111	1.98032	2.62814	0.6119	0.499315
ENSG0000	RPS27AP1	7:1571389	1.44592	1.26971	1.1094	0.133782	0.745879	3.97496	1.31463	0.600524	2.49448	1.9799	0.3877	1.19794
ENSG0000	DIS3L2	2:2319612	21.1849	17.601	11.668	18.7382	13.7739	10.0842	14.8275	8.72519	31.9858	16.6477	0.93355	0.040728
ENSG0000	CCNC	6:9954237	48.7082	35.3471	33.3951	44.7082	25.0806	20.6895	33.2773	19.8544	37.6888	21.7937	0.1666	-0.44816
ENSG0000	SAFB2	19:558699	68.9565	100.058	62.675	46.6352	79.662	74.8363	70.7935	71.4097	38.7443	96.7847	0.94085	0.032108
ENSG0000	EPB41L5	2:1200130	1.40597	1.38988	0.44938	0.060936	0.585924	0	0.045953	0.294238	0	0.362748	0.0521	-2.51525
ENSG0000	SNAI3	16:886632	10.1291	7.05472	28.04	16.2689	39.5686	5.62517	11.0083	19.1257	11.3817	22.484	0.2954	-0.52868
ENSG0000	BAP1	3:5240101	17.0644	19.4788	11.7094	11.4136	13.7061	10.8909	14.8127	13.4964	5.01154	18.5425	0.7087	-0.16569
ENSG0000	IL1A	2:1127739	0	0	0.303178	0	0.040511	1.71962	0	0.651298	0	0.178465	0.2254	2.49772
ENSG0000	NCALD	8:1016865	44.2564	18.463	21.3458	15.1703	19.8414	7.2335	21.1105	11.1522	13.8624	17.1262	0.0543	-0.75025
ENSG0000	CENPM	22:419387	31.6379	24.2024	23.579	23.6089	7.92648	36.0231	43.3099	43.886	69.0789	6.32037	0.10285	0.917073
ENSG0000	PPP2R3C	14:350449	81.1539	41.4726	76.8772	67.4975	46.171	89.067	72.3437	55.9483	66.6102	36.9807	0.89805	0.083857
ENSG0000	RPL30	8:9786883	441.637	398.295	723.939	494.453	294.806	378.532	465.427	618.192	384.241	56.0562	0.42315	-0.30531
ENSG0000	RAB33B	4:1392661	4.86422	1.81363	3.76353	4.30853	2.54096	2.27267	1.09385	4.25872	7.27363	3.10263	0.63485	-0.47616
ENSG0000	AKAP17A	X:1591592	78.4023	102.341	43.8684	47.2761	63.0876	41.5912	54.9504	30.4078	46.0428	77.3848	0.2398	-0.39525
ENSG0000	PITPNA-A	17:151693	10.0341	5.31262	8.65532	6.55108	6.2984	12.7766	8.33769	6.92751	5.78041	2.8379	0.9842	0.032303
ENSG0000	BBC3	19:472208	5.41968	5.81294	1.84423	3.4825	3.43135	5.85863	3.40174	4.67433	4.83378	2.34327	0.926	0.073464
ENSG0000	HIST1H2B	6:2604327	1.15586	0	2.92396	1.6137	0.128679	0	0.124921	0	0.174272	0.14958	0.2028	-3.42737
ENSG0000	TTN	2:1785211	1.61532	21.7291	0.100842	0.223928	1.0192	15.928	10.2247	0.085673	2.73554	7.61729	0.49665	0.63689
ENSG0000	EIF4HP1	7:2745816	4.91745	3.54625	0.863385	1.70433	0.379931	3.87666	2.83611	0.276758	0.604875	0.148894	0.778	-0.6068
ENSG0000	GTPBP2	6:4357614	6.69627	9.04276	6.37918	4.07238	6.73648	9.8592	10.9731	9.21665	3.15377	17.737	0.3115	0.732511
ENSG0000	CLUAP1	16:336509	5.66801	10.0139	9.11096	6.41561	6.85968	2.24713	5.77442	3.74363	3.51706	6.66792	0.25065	-1.07649
ENSG0000	ZMAT5	22:297200	37.1634	33.1223	41.8369	45.6122	31.2757	37.4636	44.0984	37.2495	33.1562	12.0552	0.5476	-0.26063
ENSG0000	CTD-3137	19:184414	0.326796	0.799217	0	0.156909	0	0.547129	0.529406	0.402798	0	0.527285	0.6542	0.757095
ENSG0000	TIMM10	11:575284	27.7157	20.6301	56.7412	70.0058	21.3793	40.8441	48.3981	58.5555	41.1014	13.6074	0.91865	0.040447
ENSG0000	PLK4	4:1278808	4.93898	6.65792	0.245983	1.92856	0.652291	3.64017	6.79001	0.763019	0.210802	1.99066	0.92015	0.071137
ENSG0000	UBA1	X:4719086	54.1194	63.3097	27.7727	24.9541	30.9032	30.5254	41.2158	43.9821	18.4645	52.7762	0.72475	-0.11834
ENSG0000	FAM24B	10:122832	10.4284	14.3275	13.3841	13.2274	8.87106	21.276	11.5262	13.7767	4.44107	3.04708	0.78435	-0.22808
ENSG0000	ATP2C1	3:1308505	10.1615	10.7779	6.04749	5.48872	5.95906	18.6619	11.5438	13.5505	7.30825	15.1841	0.15325	0.848425
ENSG0000	FBXO3	11:337409	16.9855	23.5023	14.8891	18.7337	11.703	32.371	23.7729	14.8023	13.2026	16.485	0.6721	0.198527
ENSG0000	AC090587	11:385431	0.616787	0.247397	1.53568	0.382763	0.099791	0.116436	0	0.334635	0	0.134596	0.2767	-2.15144
ENSG0000	WDR47	1:1089702	6.66458	6.52517	2.84876	3.63042	2.53286	7.34384	6.64251	2.79558	6.66873	4.41069	0.4907	0.340668
ENSG0000	RP11-106	12:276963	3.02072	0	0	0.04512	2.05156	3.65914	0	0	0	1.66812	0.9877	0.01156
ENSG0000	DOK2	8:2190887	178.416	203.298	150.356	108.084	138.771	181.019	239.682	107.993	81.8695	90.1356	0.41475	-0.26586
ENSG0000	IRF2BP2	1:2346042	26.5722	25.8396	23.8638	50.5468	24.0007	25.1136	20.2371	33.7647	17.1537	35.4076	0.5256	-0.33257
ENSG0000	CDC45	22:194794	4.34352	6.30471	0.750313	1.88578	0.883511	8.2641	3.84122	5.067	2.01309	3.36801	0.36595	0.852156
ENSG0000	SLFN12	17:354110	5.06413	2.26016	0.818859	2.16439	4.93904	11.658	5.05209	2.79458	6.74645	6.30031	0.0685	1.08804
ENSG0000	MAGOH	1:5322689	28.6263	15.7557	68.2572	51.2252	47.9126	22.6629	36.5882	61.6699	49.7804	13.0514	0.7074	-0.24523
ENSG0000	TAGLN	11:117199	0.618049	2.86178	0.674258	0.625405	0.483017	2.02829	1.93595	0	0	2.59902	0.73465	0.319325
ENSG0000	WDR6	3:4891778	79.9582	96.2204	26.6929	37.8947	25.7903	54.5462	56.6508	41.1881	31.3445	62.4385	0.8712	0.122791
ENSG0000	JAGN1	3:9890553	25.7835	22.094	38.7522	27.2524	42.5424	26.7838	27.4327	32.6502	22.3056	18.4582	0.36025	-0.32556
ENSG0000	RP11-308	X:1369093	0.429987	0	0.121072	0.024699	0.342885	0	0.349608	2.09799	3.51129	0.68013	0.09235	2.65841
ENSG0000	RP11-247	22:313995	0.967498	1.12342	0.54109	0.456228	0	0	0.568055	0.596406	2.97906	0.837149	0.49905	1.0452
ENSG0000	CRCP	7:6607579	14.4363	14.3279	19.1338	14.0275	28.6127	20.1313	16.7093	12.9783	13.0618	23.5894	0.61085	-0.26773

ENSG0000	KLRC1	12:104123	54.0254	5.93998	55.5067	91.7375	56.2634	79.5263	298.067	49.9664	169.456	65.6326	0.00235	1.3548
ENSG0000	PGF	14:749418	0.084097	0	0	0	0.147784	0	0.026487	0	4.65292	0.016813	0.1187	4.9056
ENSG0000	RP4-539M	22:303319	0.788129	0.163476	0.653263	0	0	0.431033	0.939178	0.18926	0	0.188306	0.84825	0.330209
ENSG0000	ZFAS1	20:492192	24.7875	26.7223	54.4704	39.7631	11.6014	32.1201	41.9296	62.49	34.9392	6.69109	0.8274	0.172673
ENSG0000	SPRED3	19:383884	0.051993	0.322331	0.009814	0	0.037106	0	0.301069	0	2.853	0.654219	0.02515	4.16755
ENSG0000	FLJ27354	1:8952483	4.08458	13.6659	2.1964	3.74291	1.42421	2.55522	1.24722	1.55357	5.1938	0.666306	0.37105	-0.84383
ENSG0000	PREPL	2:4427545	14.1443	12.401	4.63158	3.33165	3.99584	13.755	6.39397	7.65901	6.15081	7.14344	0.88395	0.05879
ENSG0000	CAHM	6:1634130	0.578191	2.7113	0.976232	1.1492	0.657194	0.555656	0	1.11786	0	1.60412	0.689	-0.72041
ENSG0000	TMCC2	1:2052281	0.788655	0.697827	3.23527	0.91665	1.55911	0	0.716846	0.792835	0	1.95068	0.39285	-0.92942
ENSG0000	ST6GAL1	3:1869304	46.9717	81.6076	20.519	15.8652	20.6405	16.7752	22.0524	15.6955	15.719	26.1191	0.0073	-0.85149
ENSG0000	CHURC1-I	14:649143	3.64691	1.4905	5.27713	3.35446	2.30757	5.63246	4.12499	4.14617	1.19013	0.950481	0.86005	0.218894
ENSG0000	RARG	12:532105	16.6888	4.35905	12.8721	15.332	17.6038	3.7754	0.779958	10.6047	2.50391	14.9209	0.02825	-1.06927
ENSG0000	RP11-455I	16:301828	2.13312	3.59347	4.23382	7.23582	2.86787	1.271	2.17426	2.0996	5.25011	1.45808	0.6351	-0.38375
ENSG0000	SLC35A4	5:1405582	43.6837	63.9015	54.3142	46.2481	42.8105	38.8339	34.5636	35.1997	51.9512	47.7662	0.5021	-0.23591
ENSG0000	TPP1	11:661276	91.2052	111.194	54.2001	42.3136	48.3711	148.813	88.3889	43.7804	67.9949	85.3187	0.3257	0.289413
ENSG0000	HNRNPA1	13:526000	6.60393	6.30883	4.85544	4.97927	3.93292	6.40972	5.92072	3.76355	2.27875	5.96731	0.69135	-0.24275
ENSG0000	SLC39A7	6:3319358	10.0127	9.28275	5.83661	29.8269	8.38556	17.216	9.82557	8.92276	15.3803	7.52578	0.8135	-0.14898
ENSG0000	MAP3K14-	17:451909	2.97052	14.2082	4.85445	3.34174	2.12085	4.72919	6.60319	8.64496	15.9846	2.61924	0.4713	0.689483
ENSG0000	RP11-418.	1:1190312	5.76797	2.98758	3.59694	6.02632	2.81833	9.47373	4.59645	2.89219	7.16982	2.97142	0.7421	0.370798
ENSG0000	CCT6B	17:349278	0.942141	1.87817	1.00092	2.05929	0.58005	1.71429	0.154183	0.020556	0	0	0.2554	-1.93051
ENSG0000	CENPO	2:2478973	1.71086	3.49294	1.88887	1.17418	1.72072	4.24767	4.91407	1.90652	2.27361	4.68943	0.25065	0.902233
ENSG0000	RP3-424M	12:110491	1.5913	1.15259	1.48562	1.17423	0.75748	0.321418	1.04151	0.995279	2.22214	2.51944	0.8461	0.2427
ENSG0000	ALG9	11:111782	6.22492	9.02969	8.03685	7.72221	3.44723	9.44628	6.09322	11.2759	11.9868	6.20797	0.4558	0.460405
ENSG0000	TECPR2	14:102362	1.11318	1.73534	0.64619	0.677733	0.986115	1.97848	0.664284	0.902413	3.33275	1.5943	0.60105	0.439693
ENSG0000	RP4-647J2	7:4495899	2.31666	1.34358	0.944992	1.22877	3.38259	1.66574	2.32068	1.4505	4.02548	4.23861	0.46805	0.497891
ENSG0000	GYPC	2:1266559	116.258	101.428	86.0841	79.5324	109.477	106.657	100.822	95.7154	95.5582	33.2074	0.5422	-0.20897
ENSG0000	TTYH3	7:2631950	0.744401	1.53782	0.664232	0.305494	1.11749	1.82147	1.02658	3.39738	0.82339	4.29476	0.025	1.44621
ENSG0000	RP11-727I	11:627711	4.337	3.33583	8.39031	4.40312	3.41188	0	1.35485	2.42048	8.271	2.71102	0.68615	-0.58853
ENSG0000	C12orf75	12:105173	201.769	113.859	164.671	137.18	148.835	164.282	211.943	141.742	145.179	38.6498	0.7043	-0.14372
ENSG0000	U1	1:1496063	20.5106	1.20892	8.52033	40.8472	2.93095	0	0	0	18.6835	0	0.50255	-1.71289
ENSG0000	CIT	12:119685	0.542842	0.778212	0.023018	0.356833	0.067084	0.22264	0.176817	0.141828	2.57256	0.60002	0.06635	1.4126
ENSG0000	GANC	15:422084	6.93225	15.0103	7.39944	6.93262	3.83327	8.49034	6.52309	4.03968	5.19834	4.98513	0.37315	-0.56191
ENSG0000	EIF4A2P1	18:127854	0.17117	1.2781	0.584162	0.509696	0.085174	0	0	1.70522	0	0	0.79775	-0.51097
ENSG0000	GATC	12:120438	8.29134	5.32726	7.38516	3.62518	7.54128	3.51052	11.4775	7.0417	7.87938	16.0499	0.756	0.367897
ENSG0000	DNAJC28	21:334855	1.57146	0	0.976479	0.452698	0.708967	0.342865	2.59456	0	2.58609	0.831826	0.55495	0.85672
ENSG0000	BCL2L15	1:1137618	0.103331	0	0.110276	1.8004	0.034174	2.18779	0	0	0	1.13149	0.8841	-0.26745
ENSG0000	MXI1	10:110207	19.4206	28.8604	16.2214	10.707	21.6013	9.20257	6.37059	10.4896	6.4577	12.3192	0.00595	-1.20831
ENSG0000	CNIH4	1:2243568	30.0406	16.9131	19.8295	16.2356	16.4988	40.9334	39.736	22.4178	25.2333	8.4217	0.8255	0.108385
ENSG0000	ATF6	1:1617662	9.59405	10.9351	4.54289	5.26806	5.52696	8.39083	6.34832	5.87831	3.81089	9.58253	0.7329	-0.12824
ENSG0000	ARHGEF1	19:418831	260.844	499.335	95.1167	125.951	115.928	207.42	313.209	130.544	96.8146	205.313	0.6293	-0.16573
ENSG0000	SLC26A1	4:958886-	1.52334	1.16194	0.571262	0.488479	0.216784	1.89583	2.19436	1.01926	0.018394	0.865074	0.6045	0.554364
ENSG0000	PTPRF	1:4352518	0.036285	0	0	0	0	0.197096	0	2.14762	1.49595	4.90468	0.22025	7.92267
ENSG0000	CAMK1	3:9749943	5.12188	9.69625	1.15332	1.88269	1.52502	62.1781	14.5509	4.68056	5.77414	3.19761	0.09585	2.17658
ENSG0000	TTC24	1:1565797	2.45714	2.18411	1.63129	1.04447	0.755701	1.84961	3.29407	2.95065	3.22374	3.77888	0.2336	0.93993
ENSG0000	HSDL2	9:1123979	10.4969	7.22141	3.64473	5.36114	5.28056	15.8301	6.2527	3.40171	2.70076	7.82604	0.74975	0.140019
ENSG0000	COQ8A	1:2268975	35.2027	59.9939	39.7212	31.7528	30.5637	10.004	20.863	15.6178	9.99482	16.8089	0.0008	-1.35325
ENSG0000	LRRC63	13:462119	0.769074	0	0.020715	0.418642	0.131682	1.48635	1.15428	0.948307	0	0.362856	0.3592	1.75412
ENSG0000	RP4-756G	22:412052	0.033092	0.807036	0	0.018051	0.030185	2.31138	0.563161	0	0	0.145179	0.50935	1.60146
ENSG0000	RPS6P25	19:128910	2.43447	3.22286	0.739893	0.486802	0.764961	1.94877	6.80653	2.43051	0	0.660869	0.77225	0.685674
ENSG0000	MTHFR	1:1176178	3.75534	2.74468	2.17979	2.94274	2.90426	7.73808	3.30025	4.01538	4.70947	7.70046	0.20905	0.742171
ENSG0000	WDR55	5:1406646	14.783	19.8511	13.8753	9.2769	12.0699	7.6023	9.06495	11.8858	8.29301	16.0805	0.60755	-0.35466
ENSG0000	RP11-141	5:7019725	2.99257	5.79553	1.97894	1.23438	1.48351	3.13178	2.53597	3.0718	2.94601	6.22412	0.7921	0.619753
ENSG0000	SLC38A9	5:5562584	12.9909	10.4011	5.69846	8.05684	3.00186	4.74805	9.87767	14.1719	2.44894	6.88562	0.87675	-0.07653
ENSG0000	GLI4	8:1432468	21.7765	35.5706	25.1745	18.54	23.2439	11.3958	22.4046	11.3918	22.583	16.2469	0.45865	-0.53153
ENSG0000	MAU2	19:192760	23.1166	22.4072	9.38474	18.8703	11.6778	14.0468	19.2349	8.17551	14.9456	25.0971	0.8775	-0.06615
ENSG0000	AUP1	2:7452664	181.156	176.816	146.695	163.836	119.63	270.695	238.907	137.498	117.659	153.004	0.62565	0.178051
ENSG0000	XBP1	22:287945	388.523	142.711	620.364	505.82	624.425	118.594	223.631	249.292	321.891	259.907	0.0198	-0.97952
ENSG0000	YY1	14:100207	12.06	11.1602	18.1888	13.0882	22.9716	13.1621	16.0419	16.1263	10.4248	27.4664	0.6758	-0.17843
ENSG0000	UTP4	16:691055	30.4707	24.2148	22.549	25.8216	19.4333	16.6512	23.1144	20.159	27.5187	14.2783	0.7966	-0.27165
ENSG0000	ATP2B4	1:2036265	24.8812	20.4096	11.3138	3.55433	10.5331	12.2534	22.5087	14.795	9.29156	28.9262	0.1911	0.469823
ENSG0000	KDM2A	11:671192	7.79785	7.81125	8.92665	6.71654	11.5846	5.96912	7.95176	10.1862	10.0808	24.6006	0.46515	0.329545
ENSG0000	RAB2A	8:6051685	80.0924	79.7968	64.9246	53.2992	60.737	99.9158	115.547	53.517	72.6404	49.072	0.7415	0.159558
ENSG0000	ASNSD1	2:1896613	63.0011	54.9559	41.0665	32.2372	29.3062	27.9005	57.3299	31.8153	34.1302	21.6536	0.3785	-0.29544
ENSG0000	KLF11	2:1004284	3.56902	6.74291	2.04683	4.74216	4.1121	3.70664	2.57257	2.13564	0.0184	3.65671	0.1764	-0.88322
ENSG0000	LENG1	19:541377	13.1683	15.5878	33.4641	20.2865	31.1563	23.3372	15.6994	29.0271	8.83315	14.9212	0.48005	-0.36447
ENSG0000	ZNF830	17:349278	24.7613	35.3015	24.6206	26.4611	35.6128	23.1905	42.2151	26.339	24.4463	25.2977	0.8494	-0.10756
ENSG0000	ALDH4A1	1:1883959	1.60129	1.59631	0.332134	0.463184	1.01653	1.31563	2.1686	0.043247	0	0.956914	0.8608	-0.23193
ENSG0000	PIK3C3	18:419552	21.2917	10.2129	11.916	5.70879	6.44303	20.6768	16.4149	8.81387	18.4302	10.1915	0.8847	0.078694
ENSG0000	DUOX1	15:450926	0.425788	2.12458	0.401455	0.018139	0.361375	5.62417	0.084548	1.04068	3.43886	0.778968	0.03855	1.90228
ENSG0000	HPDL	1:4532690	1.14571	0.02662	2.22516	2.70408	1.65234	0	0.927059	3.4355	0	0.888424	0.63115	-0.62818
ENSG0000	MT-ND6	MT:14148-	24.4766	36.2075	331	358.1	971.591	53.0134	45.4451	312.083	370.578	726.15	0.6226	-0.22732
ENSG0000	GADD45B	19:247612	99.7558	49.893	146.019	138.011	21.9439	83.6408	104.293	137.767	178.575	16.5864	0.6086	0.187097
ENSG0000	AC002310	16:305240	5.40918	6.88253	9.72294	5.98143	3.40234	5.16598	3.02794	0.290906	12.7097	5.36549	0.86095	-0.21861
ENSG0000	RPL32P3	3:1293812	5.68244	15.2291	9.25633	9.88565	8.16636	8.6411	18.9512	12.7063	13.1694	15.3694	0.44305	0.504995
ENSG0000	IFI44L	1:7861992	4.41043	6.68084	8.35347	6.08284	6.85146	6.79957	83.8345	30.7478	8.47802	26.5772	0.00005	1.85002
ENSG0000	RP3-388M	22:438124	0.289786	0.359858	0.643015	0	0.043506	0.622653	0	0	2.279	0.936393	0.3073	1.69161
ENSG0000	RP11-437	15:928827	0.576221	2.48408	0.549616	0	2.53837	4.2982	4.59629	0.98763	0	4.11542	0.55775	0.914941
ENSG0000	FCHO1	19:177477	16.6357	25.8163	5.1757	4.38633	6.20593	15.0287	24.5164	7.68664	9.87836	18.5067	0.3944	0.325414
ENSG0000	TARS2	1:1504873	18.4388	22.8298	26.3611	26.0796	16.193	13.7188	12.4313	14.2105	20.9083	17.1232	0.06535	-0.64503
ENSG0000	SLC5A2	16:314830	0.720676	0.873993	1.47609	0	0.831133	0.541208	0	0.199629	0.17802	1.0499	0.99965	-0.00114
ENSG0000	SEC14L6	22:305227	0	0	0.307573	0	0.50627	0	0.127353	0	0	2.11575	0.5431	1.46611
ENSG0000	ATP6AP1L	5:8227946	0.180411	0	1.41681	0.06582	0.292742	0	0.072839	0.14195	0	1.19646	0.7002	-0.51401
ENSG0000	CHMP4A	14:241718	168.011	125.726	167.115	176.638	223.488	268.329	226.102	164.307	167.38	90.7812	0.95765	0.04604
ENSG0000	TNRC6C-/	17:779592	11.6495	21.5459	3.73176	4.89438	4.93169	13.1129	10.774	3.01909	2.7117	12.5012	0.79735	-0.26736
ENSG0000	ZC3H10	12:561181	2.95846	3.36262	2.43186	1.37269	1.73691	2.90927	2.04356	2.64557	0.250449	3.04793	0.8589	-0.17146
ENSG0000	GPBP1L1	1:4562730	61.7848	51.3112	16.3659	15.4093	19.7386	66.8347	47.7564	32.0785	19.7903	33.2365	0.33905	0.474108
ENSG0000	BBS2	16:564291	43.7557	38.2211	32.3565	29.7924	21.0601	15.3677	12.6607	18.6724	16.5748	14.6603	0.0694	-0.98311
ENSG0000	IFFO2	1:1883959	3.67795	1.84937	1.8959	1.35664	3.55994	3.06712	4.06105	1.63671	0.362777	3.84155	0.92865	-0.05229
ENSG0000	RPS3P2	20:384728	0.397792	0.298222	0	0	0	0.109156	3.08158	0	3.04486	0	0.2867	3.42934
ENSG0000	ABHD16A	6:3168694	34.116	40.8155	47.1163	40.2557	42.3379	44.0152	44.8374	53.5361	30.6047	40.8902	0.9099	0.036002
ENSG0000	UGDH	4:3949875	17.8699	9.28695	12.3233	19.4747	10.3474	8.45524	7.17927	8.35937	12.5237	9.27745	0.27995	-0.54548

ENSG0000	CC2D1B	1:5214209	11.4209	7.23138	3.29698	3.68049	4.60935	12.8469	6.16593	6.96612	2.96899	12.5531	0.4115	0.400343
ENSG0000	PREX1	20:486242	43.705	33.8921	15.7412	9.76956	30.6162	33.9797	38.6536	35.9851	11.7204	60.2604	0.33935	0.323827
ENSG0000	GEMIN8	X:1400827	17.1894	11.5611	15.2604	15.2615	17.6902	18.3393	15.8561	11.6142	22.5262	10.0849	0.97045	0.01835
ENSG0000	LTA	6:3157205	29.9725	22.0222	29.2093	41.5095	27.9197	70.1409	83.5929	97.0152	69.7562	27.1687	0.0009	1.21044
ENSG0000	RP11-113	12:825452	0.115447	1.04611	0.739658	0.634004	0	0	0	0.987695	3.04873	0	0.70145	0.791615
ENSG0000	NXT2	X:1095357	11.698	12.2593	6.56619	6.41711	4.22386	11.3926	9.79822	6.69574	6.22037	5.53688	0.93885	-0.03543
ENSG0000	PARP3	3:5194234	4.8483	3.1175	2.6312	5.64409	3.44824	6.82976	2.57925	5.51131	7.81731	8.48444	0.20235	0.68733
ENSG0000	CEP85	1:2623415	7.41478	11.4968	3.60847	3.18081	2.28739	4.31607	5.92071	6.25978	9.77132	5.81893	0.4894	0.342375
ENSG0000	RPL7L1	6:4287961	39.1564	29.3625	104.163	59.9803	119.208	35.3012	54.1693	76.5746	71.3155	94.82	0.66395	-0.17894
ENSG0000	HRAS	11:532241	17.6446	13.4909	10.5599	13.0136	20.3828	19.797	22.7122	20.348	13.8393	16.0879	0.6436	0.261407
ENSG0000	KIAA0101	15:641655	31.9546	44.6016	1.7393	7.59103	2.09625	56.4168	71.2835	30.9291	15.4771	3.14013	0.25545	1.08882
ENSG0000	YAF2	12:421571	13.1242	12.7773	24.4079	15.6581	31.317	19.5163	22.4509	24.2813	28.5924	22.075	0.29175	0.457351
ENSG0000	KCTD7	7:6662888	8.72638	9.00124	3.32864	3.04448	4.05304	5.94924	11.3784	6.26326	7.58127	13.1597	0.3605	0.464949
ENSG0000	LILRB4	19:546438	0.318878	1.44602	0.035635	0.824253	0.318559	46.5338	0.313717	0.858968	1.0981	0.432502	0.00065	3.99358
ENSG0000	CDK8	13:262541	2.71971	2.33077	0.568894	2.90265	2.49222	1.66989	1.19534	6.06877	2.48418	5.89414	0.4303	0.648884
ENSG0000	AP000275	21:325722	1.7561	3.32377	0.153579	0	0.534773	4.96323	2.8102	0.968783	0	0	0.7777	0.548628
ENSG0000	CELF2	10:107983	71.0959	75.0313	93.3141	65.7059	108.385	65.6254	67.3472	109.157	50.6765	189.51	0.96575	-0.02073
ENSG0000	ZNF696	8:1432896	3.92103	4.06932	1.51023	2.31713	2.36256	4.54133	0.797745	2.77916	0	3.54774	0.76335	-0.25585
ENSG0000	MED28	4:1761463	22.465	18.0874	44.7405	25.3613	21.9697	37.9052	26.1579	35.38	22.3007	8.10914	0.79465	-0.18129
ENSG0000	UBE2C	20:458125	8.77215	34.2311	0	2.33493	1.67895	48.2073	35.1847	12.778	29.2228	9.11063	0.20545	1.67763
ENSG0000	EIF2B3	1:4485052	21.9994	30.1942	26.839	37.535	25.0427	28.828	19.3164	16.4426	19.4581	17.1602	0.18745	-0.49372
ENSG0000	SLC11A1	2:2183820	5.39029	19.7421	1.39798	0.8555	0.980097	53.35	1.67589	3.23098	0.352727	1.29598	0.03675	1.22012
ENSG0000	CTD-2336	8:1755777	9.25233	15.229	4.36518	7.5177	4.16458	9.72165	8.42101	3.48954	6.60955	2.72886	0.8079	-0.34201
ENSG0000	SMYD5	2:7321422	8.65594	9.44301	6.39311	8.36182	6.3518	2.20305	6.40399	4.34615	1.50828	7.40372	0.09205	-0.85024
ENSG0000	LINC0092	15:573003	0.203973	4.30764	1.69272	4.61368	1.11273	0	0.564305	0	0.202749	0.510687	0.0352	-3.62768
ENSG0000	RP11-203	9:1279345	1.42019	7.75272	0.244249	0.038772	0.057943	0.638873	2.81049	0.434358	0	2.49177	0.80435	-0.35851
ENSG0000	RAB7B	1:2059767	0	0.513469	0	0.579617	0	2.74878	0	0.084929	1.04315	0.153598	0.3018	1.99642
ENSG0000	TMCC3	12:945671	0.742485	2.64255	0.609048	0.770262	0.335655	0	0	0.009536	0.796419	0.702191	0.1424	-1.91427
ENSG0000	LRRC37A	17:462747	2.07766	2.83286	1.17965	1.28132	1.06924	1.71988	1.92234	0.891159	0.771579	3.82815	0.9468	0.0963
ENSG0000	RP11-713	12:249934	2.02179	2.56059	0	0.811455	0.122038	0	0.261293	0.741535	0	0.126891	0.322	-1.8301
ENSG0000	B4GALT5	20:496329	1.7273	0.861134	1.43069	1.38577	3.42603	2.18755	3.6337	1.89606	1.29162	5.09258	0.36325	0.48175
ENSG0000	FNDC3B	3:1720396	5.71303	3.68807	5.22883	1.68401	6.25475	12.4531	15.7602	4.16021	3.6286	5.38139	0.21075	0.947276
ENSG0000	PROSC	8:3776259	67.1964	53.5045	52.5119	52.4965	63.9901	77.1982	48.7316	49.4632	53.129	65.0607	0.83685	0.070257
ENSG0000	C8orf76	8:1232199	27.2899	23.5356	16.4346	16.9976	17.197	57.4844	35.6944	20.3836	15.7356	8.49891	0.5147	0.423043
ENSG0000	HERPUD2	7:3563265	47.0665	38.3042	63.2267	63.96	45.2322	15.2169	30.5135	60.1626	45.3917	48.8221	0.29195	-0.37316
ENSG0000	TPT1P12	12:914883	0.985287	0	0	0	0.368748	0	0	0	3.13955	0	0.6105	1.48691
ENSG0000	E2F6	2:1144437	7.14585	2.03962	6.02258	5.53016	5.97366	13.3045	8.26068	9.32995	5.3259	9.49235	0.36985	0.514273
ENSG0000	KRT81	12:522325	0	0	0	0.421824	0.252096	0.899201	2.09738	0	0	0	0.41605	2.11064
ENSG0000	LINC0042	13:303402	14.9613	25.7687	9.25366	13.4776	16.2632	26.021	39.9529	20.8184	34.7095	25.0555	0.0739	0.935072
ENSG0000	ACVR2A	2:1478445	0.0723	0.595477	0	0.078622	0.04832	1.10208	2.61752	0	0	0.617658	0.1109	2.33768
ENSG0000	RP1-30M3	6:2466703	0.640994	0.514792	0.118413	0	0	0.411139	0.455973	0.372936	0	0.549455	0.5375	1.00939
ENSG0000	KCTD15	19:337959	0.447105	0.887082	0.696845	0.601704	0.492951	1.30224	0	0.062639	0	0.319398	0.2259	-1.36926
ENSG0000	CXorf56	X:1195381	8.54673	4.55778	9.26424	7.48251	5.27835	9.92402	3.37083	6.36586	5.40745	5.0348	0.68565	-0.29818
ENSG0000	ABCC4	13:950198	1.56458	2.30169	0.634064	0.873506	0.508598	0.42787	0.812832	0.51323	1.34257	2.8906	0.84115	-0.13881
ENSG0000	RP1-20C7	6:4302164	0	0	0.303774	0.803822	0	0	0	1.51022	0.385052	0	0.73235	0.9979
ENSG0000	PSMD14	2:1610962	53.404	45.1198	52.5188	60.4288	40.3552	75.5285	58.1851	54.2625	46.8047	25.5105	0.8503	0.093686
ENSG0000	SLC12A9	7:1008025	3.05831	3.96695	1.17091	0.906589	2.12871	5.56443	2.0468	2.11191	1.05391	5.29376	0.53115	0.42253
ENSG0000	MAP4K3	2:3924926	2.77368	5.79692	1.2374	2.64632	0.686944	3.07109	1.77249	1.56809	1.36685	2.09653	0.81395	0.145456
ENSG0000	PA2G4P4	3:1567473	0.710156	1.60746	0.282887	0.125418	0.247391	0.22785	0.497612	0.406595	0	2.12933	0.9377	0.14371
ENSG0000	CCT6P1	7:6564700	3.94463	4.72006	3.34849	4.70047	1.19524	3.87403	9.68398	4.67276	11.4928	3.16653	0.17975	1.05327
ENSG0000	LMO2	11:338585	0.595658	3.02847	0.47681	0.910727	0.889547	4.78163	0.629512	0.308375	0.192227	0.411459	0.94505	0.139389
ENSG0000	KIF4A	X:7028659	1.35311	5.2525	0	0	0.114094	6.07132	1.18296	0.570057	1.53383	0.439551	0.72205	0.7051
ENSG0000	RP11-803	2:1111959	1.67841	1.09751	0	0	0.288373	0	1.11191	0.393619	1.51589	0.433597	0.7622	0.468604
ENSG0000	SMARCAL	2:2163852	18.6109	19.3571	14.2369	11.7601	10.3993	15.3116	8.67785	8.12902	13.9106	15.2867	0.75645	-0.25167
ENSG0000	UNK	17:757764	10.7154	10.104	26.4864	32.2884	13.3946	5.53419	14.9458	29.7729	25.2658	8.83486	0.8429	-0.15647
ENSG0000	AF131217	21:284393	1.57972	0.325748	0.62549	0.535193	0.564064	0.96649	1.49073	0.205965	0.460446	0.584425	0.9493	-0.04588
ENSG0000	SEC24D	4:1187228	17.1766	13.8321	6.76816	11.2507	6.00451	4.43638	9.96274	8.61431	18.3727	8.50399	0.95365	-0.02134
ENSG0000	FBXL15	10:102402	31.2032	33	36.3732	28.5197	50.4947	51.8749	45.7592	25.1077	16.5481	16.4	0.44895	-0.4014
ENSG0000	ZNF33A	10:380106	7.14111	8.94045	4.73023	6.57143	5.93279	6.99608	8.02526	5.36593	13.8215	10.0693	0.7899	0.175537
ENSG0000	ARMCX6	X:1016151	16.455	17.793	25.1311	28.9614	24.6049	34.3913	34.4009	29.7648	41.7719	20.3167	0.40025	0.460667
ENSG0000	AC006116	19:562196	2.20891	0.124957	1.13214	2.81884	0.688663	5.80293	1.03519	1.80148	2.42511	0.094359	0.59355	0.679381
ENSG0000	ADGRE3	19:146191	0	2.04507	0	0	0.145539	8.8597	0	0	0.407713	0	0.341	2.10906
ENSG0000	RAD21	8:1166421	43.5226	60.0462	16.8696	15.5815	27.3239	44.835	54.754	36.5557	19.7005	48.6901	0.6349	0.271725
ENSG0000	CCDC15	11:124954	0.573694	1.25807	1.06147	0.545604	0.565789	1.92525	0.680829	1.34114	2.17532	1.40122	0.2743	0.87615
ENSG0000	EMD	X:1543791	5.16871	3.26573	4.30017	7.65855	4.86592	6.27005	6.06079	6.23146	9.83848	4.43952	0.59015	0.361321
ENSG0000	IGHV3-23	14:106268	0	111.156	3.51852	4.37094	2.82856	0	0	0	1.34116	0.341439	0.0056	-5.64783
ENSG0000	RP11-498	9:9715656	1.13832	0.339138	2.86225	3.32503	0.924048	0	1.67681	2.90755	0.941338	0.671524	0.7803	-0.49156
ENSG0000	AP4B1	1:1137618	26.9754	22.8646	20.7699	19.9298	20.5834	29.2145	26.4177	25.9701	16.2747	37.3646	0.66835	0.236846
ENSG0000	UHRF1	19:490307	2.48706	4.63329	0.113726	1.61095	1.37154	3.54821	0.9943	5.72248	1.54656	5.28206	0.4485	0.567955
ENSG0000	PTPN6	12:694646	240.914	205.214	129.77	173.192	169.944	176.875	154.349	116.527	39.6571	177.887	0.1837	-0.42759
ENSG0000	RPP38	10:150953	37.7396	27.5706	47.0381	25.827	38.2334	44.5415	39.0685	27.3956	10.0173	11.4634	0.47785	-0.52408
ENSG0000	PAX8-AS1	2:1132115	6.73995	13.5089	4.18801	0	7.18206	4.67261	13.6858	7.48992	0	15.5423	0.68195	0.324751
ENSG0000	SPPL2A	15:507022	48.0625	54.6183	37.6615	38.8333	20.9606	118.998	108.78	101.781	43.7542	49.9773	0.05425	0.929081
ENSG0000	WWP2	16:697623	31.2666	36.3264	11.4102	25.1282	11.7955	28.4298	22.6395	14.5407	10.0971	18.421	0.26255	-0.41368
ENSG0000	SERP2	13:443736	0	0.586375	0	0	0.123844	0	1.0448	1.10928	4.01562	0.08605	0.1537	3.58611
ENSG0000	LGALS12	11:635060	0.281055	0	0.685287	1.04913	0.055233	1.59206	0	0	0	0	0.6811	-0.65918
ENSG0000	ZNF330	4:1412208	22.8149	32.6472	26.2464	27.5267	22.8918	19.1327	23.5612	22.0452	28.7125	26.2515	0.50855	-0.21602
ENSG0000	CCDC134	22:418006	2.7484	3.19653	2.35961	0.91228	0.95034	4.26884	2.88577	2.03481	0	3.80115	0.753	0.377711
ENSG0000	MAN2A2	15:909022	25.5105	21.5188	5.95281	5.4795	7.78159	35.1196	22.0794	12.9278	5.10275	18.3614	0.4575	0.310395
ENSG0000	SLC25A32	8:1033986	16.2229	10.8124	10.7436	15.642	8.56378	8.98095	12.1273	19.4818	10.9867	12.0975	0.9343	0.047001
ENSG0000	WBP11	12:147845	31.7872	35.598	20.3205	17.9785	27.6469	21.8655	24.0739	19.5544	16.5934	29.8174	0.1671	-0.41323
ENSG0000	TPPP	5:612271-	0.18889	0.906912	0	0	0.06413	3.74364	1.66204	0.384721	0	0.13091	0.17925	2.49506
ENSG0000	RP11-526	15:100553	0.819429	0.738851	0.60024	0	0	0.6428	0.482143	0.126847	2.05951	0	0.6352	0.782407
ENSG0000	PLEKHA7	11:167773	5.18697	1.27844	4.71785	11.402	13.306	1.46958	0.663525	2.01242	8.75548	8.47582	0.42655	-0.67225
ENSG0000	AMPH	7:3838370	0.163932	1.57872	0.231477	0.271588	0.071439	1.22586	2.32843	0.408494	0.09148	0.860128	0.1616	1.2534
ENSG0000	PMS2P10	7:7528811	2.02311	1.87152	2.14573	1.50536	0.985	0.99212	1.30845	0.823523	2.49728	0.640652	0.94655	0.101425
ENSG0000	RP11-474	2:6812293	1.9146	1.51272	0.691535	0.518012	0.751859	0	0.477402	0.906445	0	1.39308	0.4608	-1.3434
ENSG0000	CRNDE	16:548451	0	0	0.28918	0	0	0	0.590713	3.40979	0	0	0.2984	4.47305
ENSG0000	TMEM50A	1:2524223	149.996	120.38	154.158	154.904	118.131	230.369	253.742	175.367	176.566	90.0476	0.49085	0.409514
ENSG0000	DIS3L	15:662784	22.5258	21.4852	13.0564	14.3269	8.0328	7.29631	14.6158	10.5397	21.0983	11.2152	0.5624	-0.20405
ENSG0000	NFATC1	18:793958	22.2595	30.5912	14.1212	15.754	12.1742	17.2401	12.5674	25.7831	21.8471	22.6098	0.59285	0.239999

ENSG0000(	ISY1	3:1290875	47.3328	42.7059	65.4535	58.3042	63.6955	62.7625	59.1211	56.3762	41.7905	49.8353	0.60015	-0.19574
ENSG0000(	PPRC1	10:102132	13.3823	17.1314	5.29857	2.737	6.65195	7.73656	7.62075	7.33453	1.22948	10.0462	0.67825	-0.21386
ENSG0000(	RP4-789D	1:3586980	0.598554	0.821647	0	0	0	0	0.592123	0	4.68693	0	0.3551	3.66875
ENSG0000(	OSCAR	19:540946	1.11389	2.26042	0	0.271822	0.533896	3.47836	0	0.140665	0	0	0.9553	-0.06163
ENSG0000(	NSRP1P1	1:7777962	0.127367	0.478348	0.096196	0.322185	0.106193	0	2.34669	1.75813	0.046424	0.834695	0.32735	2.12376
ENSG0000(	NDC80	18:257151	17.1352	21.6057	5.43799	9.52083	3.49699	14.841	18.7965	15.47	13.4643	8.18059	0.29955	0.474181
ENSG0000(	CTD-2054	15:998070	4.12451	2.21771	1.69727	2.0528	1.70143	6.95719	4.94765	2.65635	1.63909	1.41561	0.3842	1.01102
ENSG0000(	AC020571	2:1961943	0.830851	9.8365	0.296761	0.51434	0	4.69285	4.26161	4.84057	19.4485	6.35613	0.09475	1.98551
ENSG0000(	SBNO1	12:123289	11.5558	12.4284	10.5067	7.32977	10.6249	8.41268	9.75419	10.7736	7.46794	22.9461	0.9638	0.017205
ENSG0000(	LMBRD2	5:3609841	2.3073	3.8146	1.04703	1.07052	1.10509	2.09985	2.60312	1.68117	2.71896	3.50881	0.3476	0.498632
ENSG0000(	NAMPTP1	10:365217	2.09956	1.41165	0.512308	0.885836	0.332686	3.8537	4.16936	2.49823	3.17103	0.491243	0.1978	1.63792
ENSG0000(	EXOC3-AS	5:441497-	0.888238	0.347179	1.92785	1.71114	1.68343	4.47878	1.93115	0.798734	0	1.84213	0.87325	0.241629
ENSG0000(	TRBC2	7:1426707	1124.04	1802.55	2140.53	1723.99	1714.66	2039.31	2372.44	2278.25	2235.67	1147	0.7364	0.216495
ENSG0000(	RP11-876	16:108649	0.493252	1.71831	0.647902	0.102815	0.228403	4.90333	1.12703	1.07867	0.137043	0.490743	0.44415	1.2561
ENSG0000(	RP11-357	17:486359	0.143134	0.280237	0.226453	0.206451	0.471779	1.31357	12.4891	1.61045	2.12714	1.61357	0.0445	3.71682
ENSG0000(	RPS7P1	17:284557	1.44547	1.31572	1.06879	0.719661	0.277382	1.20149	3.07547	0.549487	0.531639	0	0.96855	0.054998
ENSG0000(	RP11-82C	17:295605	0.603152	1.19576	0	0.18329	0.510156	0.979847	0.594702	0.361362	0	0.248949	0.92395	-0.1357
ENSG0000(	SRGAP3	3:8980590	1.29548	1.83737	0.212347	0	0.011607	10.3975	9.07455	4.44566	3.65881	5.74512	0.0157	3.33232
ENSG0000(	MTRNR2L	3:9661718	0.053152	0.035204	0.571518	1.09032	0.36573	0.087401	0.073587	0.155711	1.47025	0.040957	0.83795	-0.30624
ENSG0000(	ACTL10	20:336570	6.17319	4.03494	8.47861	5.8832	11.4259	4.59856	4.44504	7.07714	10.47	7.36653	0.97765	0.021689
ENSG0000(	NKAPP1	X:1200362	4.09058	6.83497	6.40929	6.21061	5.96604	3.67031	3.89794	3.13584	1.71356	3.01288	0.40475	-1.05686
ENSG0000(	STIM2	4:2685767	33.1475	46.6668	19.5923	14.8553	18.5807	32.4487	36.6451	20.2376	14.3659	28.9801	0.85695	-0.07958
ENSG0000(	GAS5	1:1738632	103.809	92.3569	221.182	161.792	73.9494	104.321	117.803	174.551	148.174	53.3118	0.79055	-0.11902
ENSG0000(	PIGT	20:454160	45.3049	55.7934	58.8247	60.8063	49.2559	65.8119	39.9837	45.0626	57.7803	64.177	0.99005	0.00422
ENSG0000(	AC093323	4:6670724	30.438	36.9956	48.9786	35.0295	31.8881	16.8567	23.6476	28.2792	36.6856	24.1505	0.23485	-0.46363
ENSG0000(	C2CD4A	15:620669	0.260397	0	0.014149	0.042133	0	1.24485	1.44769	0.145982	0	0	0.1653	3.3715
ENSG0000(	DCAF6	1:1679167	11.2861	12.1897	4.26067	10.1739	5.31431	16.4958	9.59715	8.15172	12.8442	8.45722	0.5931	0.39758
ENSG0000(	RP11-505	16:840537	0.269617	0.405209	0.07081	0.461736	0.068429	4.50947	0	0.546112	0	0.439807	0.301	2.02757
ENSG0000(	MIR17HG	13:913478	0.259209	0	0.720438	0.759992	0.301947	0	1.74214	1.58258	2.24113	3.58522	0.04195	2.25847
ENSG0000(	MAFF	22:381114	5.83849	3.15246	4.58305	23.0721	3.9414	4.42492	6.36672	9.12558	21.4523	10.5828	0.70705	0.290977
ENSG0000(	DCLRE1B	1:1137618	2.1887	4.22224	1.41727	2.96149	1.51618	2.40259	2.19522	0.920838	0	2.70793	0.56465	-0.71277
ENSG0000(	PSD3	8:1852730	0.052927	0.08049	0	0.371426	0	1.44465	0	0.401173	0.035892	0.790568	0.0493	2.62541
ENSG0000(	LINC0113	1:3745487	9.61826	6.5511	13.9799	11.9293	12.7146	3.21873	4.07307	4.75795	5.47939	4.75218	0.06795	-1.31536
ENSG0000(	RP11-489	8:3006301	113.772	52.3955	25.4609	36.5282	11.8154	9.97887	20.0451	11.6666	39.2764	19.5756	0.3872	-1.12528
ENSG0000(	SNRNP20(	2:9627433	52.4019	57.1635	30.4386	22.0008	35.1438	27.7359	32.4273	46.7	19.459	64.4798	0.832	0.092338
ENSG0000(	SCARNA1	12:696494	1.32162	0.530919	1.5205	1.37278	0	1.08043	2.1203	1.53902	6.24635	0	0.47665	1.23659
ENSG0000(	TGM2	20:380335	0	0.807221	0	0	0.076613	3.11753	1.48838	0.726935	0.037923	1.27401	0.0713	2.73155
ENSG0000(	KIF20B	10:897016	8.37673	19.9732	9.12312	4.35361	8.50786	13.9047	27.9291	12.4397	9.99935	17.1789	0.6171	0.372097
ENSG0000(	ZUFSP	6:1166356	12.593	10.1057	9.87508	11.337	4.76135	17.8032	11.836	11.1543	12.8391	9.32375	0.3054	0.467814
ENSG0000(	FIP1L1	4:5337764	65.7073	47.5319	32.4455	26.9805	35.4939	38.3165	47.4446	33.5914	40.266	38.2219	0.96225	0.023386
ENSG0000(	SRD5A1	5:6633342	2.35977	2.07861	1.51358	2.18494	1.51565	2.28427	5.1493	1.37162	0.824416	2.70926	0.86795	0.149723
ENSG0000(	WASH7P	1:11868-3	1.43935	1.35176	1.27241	0.925334	0.916003	1.42712	0.880414	1.16372	1.09463	1.12682	0.92355	0.17461
ENSG0000(	HEATR6	17:600431	7.06861	4.50518	3.29285	4.64635	5.08164	5.18706	4.88546	4.64584	0.34658	6.71882	0.6882	-0.22628
ENSG0000(	AC022182	8:6091005	0.489203	2.75873	2.00487	1.15866	3.87493	0	0.528314	0	5.10505	5.06235	0.907	0.184422
ENSG0000(	RP11-89K	8:1265524	0.61108	1.00136	1.05836	0.474925	0.287961	0	0	0.100122	1.07626	0.306649	0.34015	-1.22406
ENSG0000(	NORAD	20:360456	6.46317	8.25634	3.71525	3.93629	4.85024	7.01465	7.65259	6.35729	2.73923	8.7516	0.5398	0.216069
ENSG0000(	ZNF324B	19:584516	1.16549	1.53237	0.832567	0.614226	0.87213	1.06309	0.374869	0.023079	0.058804	1.14128	0.15845	-1.62688
ENSG0000(	PRDM4	12:107732	11.7803	10.421	7.23688	9.48064	6.76716	6.26515	7.74502	4.88504	3.61084	13.5419	0.24575	-0.46024
ENSG0000(	ELK1	X:4763552	4.78146	6.83151	6.08845	5.36317	8.23505	3.92362	2.41082	3.52036	11.6403	9.22238	0.96705	-0.02286
ENSG0000(	RP11-182	10:796608	0.502997	0.099683	0.469658	0.06087	0.022921	0.273721	0.925946	1.53502	0.325249	0.416189	0.37865	1.91265
ENSG0000(	SPIB	19:503842	0.070576	7.46574	1.80894	4.00924	2.94024	0	0	0.848533	0	0.110065	0.00315	-4.10076
ENSG0000(	CLIP3	19:360145	0.170955	0.912482	0.1192	0.288968	0.094062	1.42033	0	0.560083	0	1.02177	0.48245	0.926563
ENSG0000(	FAM135A	6:7041282	2.68519	2.87313	0.879189	0.728122	0.393989	1.3906	0.89646	0.744099	0	1.36941	0.4559	-0.59241
ENSG0000(	CENPL	1:1737995	5.8547	6.54611	7.6357	5.25154	3.19494	10.9666	9.12225	6.71755	0.357087	2.96444	0.89365	0.110275
ENSG0000(	IVNS1ABP	1:1852963	107.939	106.708	55.5072	69.6214	25.8048	127.486	147.701	126.216	92.3771	58.1133	0.06555	0.639193
ENSG0000(	CORO2A	9:9812097	3.61063	7.53896	7.17252	5.13972	6.24884	6.35121	3.36204	7.37884	6.12325	9.93233	0.7765	0.110055
ENSG0000(	AC009133	16:298061	3.64171	2.75269	1.04471	2.40811	2.46418	6.93615	3.62923	5.15319	1.03337	3.72729	0.4777	0.55648
ENSG0000(	RSL24D1	15:551808	77.9418	57.3355	81.3186	89.8624	41.3138	53.4	91.9998	78.8193	77.3294	13.0621	0.8167	-0.07479
ENSG0000(	ARFGEF2	20:489218	3.77394	3.30119	1.23778	0.966212	2.25663	1.88735	1.57817	1.58828	5.23317	3.65194	0.5041	0.283216
ENSG0000(	C11orf80	11:667444	3.26154	4.91774	2.97253	3.97301	1.9152	4.65796	6.51671	4.18258	1.3555	2.27463	0.8506	0.135013
ENSG0000(	uc_338	2:1916780	0	0	0	0	0	0	0	1.16707	0.661823	0.766201	0.17655	inf
ENSG0000(	PLGLB1	2:8681533	6.03047	2.32146	0.793381	0.944475	3.71961	6.30097	4.36819	1.49717	5.75707	5.69656	0.5296	0.913623
ENSG0000(	DHCR7	11:714281	9.22382	7.78269	14.0377	15.5938	12.8468	3.0888	13.8701	16.7656	18.4886	15.9301	0.7044	0.210894
ENSG0000(	MKI67	10:128096	6.39562	15.7795	0.013117	2.63227	1.07016	14.5364	8.91843	1.23647	5.57869	7.36285	0.5395	0.449544
ENSG0000(	RP11-119	20:388823	1.90745	2.06701	1.0672	2.10003	0	2.98207	2.5488	1.98517	1.53648	0.864364	0.64275	0.584912
ENSG0000(	RHOU	1:2287350	3.70278	0.795987	1.59123	1.72162	3.92905	1.06098	0.686676	0.188983	0	3.02117	0.03735	-1.43458
ENSG0000(	FBXO30	6:1457935	0.986276	0.471804	0.413683	0.302568	0.388602	2.67303	2.16032	0.607963	0.303292	1.0797	0.3393	1.40821
ENSG0000(	CTD-2630	19:364451	1.31701	1.42444	0.680447	0.658298	0.601792	1.50734	0	0	0	0.793937	0.591	-1.11612
ENSG0000(	IL16	15:811595	258.403	324.941	246.28	256.301	182.61	163.528	135.024	103.261	151.022	204.116	0.041	-0.75909
ENSG0000(	SLC16A14	2:2300349	0.408997	0.535772	1.21223	0.141406	0.724322	0	0.485664	0.387572	3.26956	0.271813	0.73105	0.551132
ENSG0000(	ZNF165	6:2807879	0.116191	0.061959	0.381768	0.259104	0.316737	0	0	0.277676	2.81918	0.024397	0.5488	1.56724
ENSG0000(	NMI	2:1512704	91.7543	52.1725	92.8492	95.3851	97.8514	121.21	119.603	98.2109	100.061	50.8742	0.5691	0.191342
ENSG0000(	SPOCK2	10:720590	131.505	86.3733	185.228	150.488	233.96	148.016	129.075	246.819	180.7	309.49	0.6038	0.235378
ENSG0000(	GNA11	19:309440	0.176714	0.301128	0	0.243772	0.645138	0.233041	0.870775	0	5.29462	0.656492	0.0308	3.09724
ENSG0000(	SYNJ2	6:1579818	6.95199	3.28637	1.19936	6.5503	3.06938	6.12182	8.44923	1.96931	7.86563	4.05082	0.63195	0.462711
ENSG0000(	IGKV2-18	2:8912872	0	4.20742	0	0	0	0	0	0	0	0	0.00105	#NAME?
ENSG0000(	XPNPEP3	22:408570	2.4795	3.29892	3.32227	0.801333	1.36469	1.22614	1.20308	2.40935	0.203822	1.63117	0.26205	-0.80458
ENSG0000(	INO80E	16:299923	8.42837	5.22452	4.18957	3.33022	3.66998	5.83782	4.48797	5.0831	6.50055	4.25903	0.8008	0.155906
ENSG0000(	C19orf48	19:507977	17.9553	27.5104	33.7806	42.6373	21.6525	19.8123	29.8123	25.055	40.5572	12.3462	0.6442	-0.1738
ENSG0000(	ECH1	19:388154	185.892	198.349	383.706	329.672	467.67	225.23	249.343	297.591	249.554	217.409	0.2766	-0.36663
ENSG0000(	ZNF564	19:123791	6.77316	9.11861	7.63489	7.54285	3.9771	5.40389	3.03942	1.18726	0.210236	8.12435	0.35335	-1.04412
ENSG0000(	DPP8	15:654424	17.1664	16.9667	16.6343	17.2477	12.8739	9.66793	12.7655	11.3417	10.4536	22.2728	0.48505	-0.34375
ENSG0000(	RNF213	17:802608	85.8159	95.6888	25.9799	37.0348	65.5934	122.272	149.472	50.4806	49.7194	123.51	0.06565	0.761594
ENSG0000(	DUSP4	8:2933306	1.56185	2.84049	0.061998	0.823787	0.149798	9.57323	46.6151	25.6203	18.1387	10.7035	0.0012	4.35758
ENSG0000(	RP11-505	7:7636517	0.713675	2.14934	0.693212	0.928464	0.568612	0	2.05542	0	0	0	0.37525	-1.84457
ENSG0000(	CUL4A	13:113177	29.7816	24.5099	22.3746	28.161	18.0191	19.6587	21.5305	27.9002	15.8837	29.7033	0.59565	-0.25195
ENSG0000(	OPTN	10:128966	83.0052	72.4925	40.8182	42.9808	62.893	88.5895	99.2417	67.363	73.3684	63.2532	0.3213	0.336353
ENSG0000(	TELO2	16:149334	31.8941	43.2698	6.52986	14.2069	11.1157	25.0929	21.6639	10.5815	15.9039	16.3208	0.9002	-0.08937
ENSG0000(	VPS11	11:119067	22.3001	25.9073	15.8354	12.75	13.1602	23.6514	13.4008	14.208	12.7541	19.0627	0.73635	0.13734
ENSG0000(	NGRN	15:902495	37.1555	37.8828	47.2833	42.2439	42.4443	43.4473	56.9225	41.9071	28.3359	24.7844	0.75625	-0.10627
ENSG0000(	ACTN1	14:688741	8.36195	118.827	6.03129	7.72218	2.91871	25.5381	2.97395	3.39377	3.53262	3.39831	0.00055	-1.90624

ENSG0000C	FRMD8	11:653865	22.0767	25.3166	15.0175	16.9464	17.8274	21.7238	15.6869	15.7228	16.3477	22.6257	0.6776	-0.17594
ENSG0000C	RP11-526	15:100547	4.05016	2.37853	2.08656	0.802099	0.119042	1.07568	1.21428	1.94194	1.43171	0.799925	0.59765	-0.4272
ENSG0000C	NIPBL	5:3687675	10.7974	21.468	6.32372	3.97062	16.5233	14.969	19.4422	8.35132	9.97745	53.8099	0.3923	0.387053
ENSG0000C	RP11-325	7:1049410	11.3652	4.84042	23.6319	8.63246	6.20077	8.68753	15.4801	16.2642	8.47816	6.63921	0.91865	-0.07185
ENSG0000C	GFPT1	2:6931976	2.98497	1.66178	1.8557	1.3301	1.68773	1.6641	2.4771	3.86634	3.25682	4.69639	0.1086	0.665665
ENSG0000C	TET2	4:1051458	12.9225	16.1042	4.64222	2.90975	3.85815	17.2177	13.1986	2.90898	8.00019	11.6728	0.364	0.400125
ENSG0000C	MED17	11:936616	19.9214	19.7984	14.7125	8.92224	13.449	31.0527	25.0441	24.8413	11.6812	14.6408	0.5443	0.316333
ENSG0000C	TESMIN	11:687074	1.22785	3.44409	2.48209	2.44736	0.842271	1.02343	0	0.872318	1.59636	0.714464	0.2973	-1.0425
ENSG0000C	SERPINI1	3:1677352	13.7704	6.43528	8.54494	11.2326	8.6896	9.29749	8.46969	1.39237	22.2545	2.76073	0.9539	0.028505
ENSG0000C	TBC1D10A	22:302851	52.673	59.6278	54.7591	60.1762	65.9921	42.4722	46.5373	65.5059	62.9291	49.1471	0.7818	-0.09593
ENSG0000C	ZNF277	7:1122065	18.6094	13.0059	17.3068	6.50536	9.03773	10.855	13.0196	13.9964	8.61734	10.8006	0.8168	-0.11642
ENSG0000C	UQCRC1	3:4859900	108.605	113.159	148.787	147.467	115.179	122.181	102.694	135.676	128.68	77.0765	0.53715	-0.18767
ENSG0000C	IGKV2D-2	2:8995997	0	27.0974	2.55653	0.370072	0.576946	0	0	0	0	0.08486	0.1245	#NAME?
ENSG0000C	SCAF11	12:459191	50.2928	49.5624	36.2416	37.125	36.1724	60.6236	45.9516	54.2024	52.3024	86.3739	0.4171	0.270887
ENSG0000C	ADAP2	17:308973	0.484638	5.47624	0.208033	1.35648	0.345881	18.0938	1.24325	0.082926	7.40368	0.650893	0.14755	1.865
ENSG0000C	EEF2	19:397605	95.0445	79.0119	38.4535	32.2034	74.3947	52.6791	49.0216	52.5512	27.4336	101.581	0.5003	-0.25069
ENSG0000C	SOS1	2:3898139	7.4528	3.19519	1.835	2.70461	4.70965	3.52583	11.0232	4.19583	5.2431	12.4468	0.2984	0.503921
ENSG0000C	DIEXF	1:2098280	2.80361	1.98146	2.3195	1.6635	2.66249	0.80457	2.12379	2.22621	0.753579	2.98824	0.3047	-0.45294
ENSG0000C	RP1-118J2	1:3978897	0.092734	0.373904	0.039964	0	0.389011	0	0.345724	1.04668	0	1.41437	0.32765	1.6753
ENSG0000C	KIF15	3:4476171	3.11272	5.65371	0.132519	0	0.233786	7.51309	1.63267	0.492938	1.86683	1.96432	0.63845	0.589489
ENSG0000C	MTFMT	15:650015	7.26063	8.3553	11.9241	8.33378	10.5902	9.92983	7.59389	5.87935	4.59618	6.14746	0.1197	-0.80849
ENSG0000C	NINJ1	9:9312148	50.139	44.9947	117.58	75.1772	101.154	107.942	69.655	104.767	96.9696	28.4804	0.9384	0.02599
ENSG0000C	STAG1	3:1362503	11.3182	16.6855	2.78966	2.94281	3.21432	7.34126	8.48874	3.57903	1.92511	7.50839	0.5642	-0.34573
ENSG0000C	SAE1	19:470641	63.0447	83.4039	49.0693	59.5696	59.7098	71.6768	72.7167	50.6706	55.9769	57.6135	0.891	-0.04814
ENSG0000C	HAVCR2	5:1570858	6.31221	10.4042	7.85321	8.296	3.61377	71.059	100.316	23.9015	27.5515	22.929	0.02575	2.59369
ENSG0000C	CMTM2	16:665794	3.52337	1.35875	0.104404	0.419539	0.223832	0.888954	1.05908	1.50799	5.72863	0.217579	0.52295	1.02659
ENSG0000C	PDK3	X:2446522	42.0579	15.9668	41.9676	28.8506	53.984	31.8107	11.8878	27.128	22.7846	47.7596	0.307	-0.40431
ENSG0000C	A1BG-AS1	19:583451	2.08879	2.58674	2.01461	1.94453	2.70336	6.65993	4.2103	7.01842	7.26513	4.93624	0.0818	1.36078
ENSG0000C	CCDC126	7:2359737	16.3284	10.0984	7.21589	9.37117	3.69081	17.0661	8.26764	8.02416	20.7728	5.42744	0.28745	0.464763
ENSG0000C	HADHA	2:2619063	82.7144	75.976	60.4093	51.0496	52.7991	70.5907	83.7465	52.7754	46.9098	57.8218	0.9856	-0.00747
ENSG0000C	BAIAP3	16:133360	1.18314	1.38458	2.38739	0.971192	0.39964	0.01234	1.07297	0.972727	0	0.350854	0.257	-1.14189
ENSG0000C	FARSA	19:129224	60.0673	70.3993	91.2362	76.789	91.1479	33.6457	68.915	56.8304	20.4793	70.0796	0.04305	-0.68076
ENSG0000C	ZNF268	12:133130	9.24686	9.46499	3.15386	4.4328	2.1823	4.33869	5.23626	2.69773	0.632097	5.33084	0.10105	-0.90917
ENSG0000C	RP11-434	2:7376126	0.208429	0.206603	0.081132	0.923515	0.408978	0	0	0	0	0.195292	0.2357	-2.85887
ENSG0000C	RP4-563E	20:628777	4.14565	3.82417	1.78687	1.86844	2.57788	4.61939	4.65235	1.48838	1.68112	3.86338	0.8415	0.240015
ENSG0000C	NOLC1	10:102152	54.0727	40.9392	29.6118	28.3183	32.0461	32.7544	46.7164	24.8897	10.553	34.8122	0.3683	-0.327
ENSG0000C	RP11-140	16:695650	0.333956	0	0.200049	0	0	1.83986	0.118325	0	0	0.692426	0.34045	2.44297
ENSG0000C	CEBPZOS	2:3716781	11.9456	12.6389	16.8179	12.4827	9.61254	19.8911	6.504	16.3442	17.0311	8.10157	0.93295	0.080601
ENSG0000C	RP11-166	16:118516	0.32321	0.560162	0.182962	1.10067	0	2.38175	1.25926	0.598479	0	0.11507	0.6248	0.839742
ENSG0000C	HCG4	6:2979095	1.117	0.864079	0.446898	0.894884	0.853184	0	0.88798	0	2.02938	0.417024	0.9297	-0.14698
ENSG0000C	ATOH8	2:8575134	0.559505	0	2.10983	0.498782	1.6962	2.62821	0.031229	0.97531	0	0.669003	0.7824	-0.42455
ENSG0000C	RAB36	22:231453	0.101439	0.533393	0.671953	0.741184	0.453573	0	0	0.356362	2.24583	0.225151	0.753	0.312133
ENSG0000C	KIAA1324	1:1091059	3.52582	3.28981	2.8651	0.813997	1.78816	4.05329	1.01008	1.43384	3.91443	3.2233	0.8735	0.089912
ENSG0000C	RAD51D	17:349278	7.38228	13.4006	8.65478	4.12634	6.43423	7.28892	8.55352	6.34257	5.61095	8.84986	0.8238	-0.14792
ENSG0000C	CEP295	11:936616	2.57263	4.63441	0.88884	1.13492	1.9078	5.46382	2.35494	1.48515	0.499924	4.03747	0.86455	0.229476
ENSG0000C	MT-ND3	MT:10058	68.5952	73.8892	390.067	402.019	874.559	94.4238	95.7602	525.533	350.069	189.864	0.155	-0.58634
ENSG0000C	ACSF2	17:504261	2.33867	4.79048	3.45683	2.98874	2.41788	4.81915	1.70063	5.06888	6.42424	2.67254	0.3044	0.498012
ENSG0000C	CDC16	13:114234	26.3159	20.8455	14.3268	15.9218	16.0388	22.1919	27.6561	20.2643	13.0189	31.6219	0.509	0.278042
ENSG0000C	GORASP1	3:3905199	8.18801	14.9228	4.63351	5.49622	2.36211	9.4867	14.1471	5.80889	3.33298	7.32046	0.7396	0.220687
ENSG0000C	FGD5-AS1	3:1481896	16.6345	18.4305	8.90192	9.1512	11.2833	11.7291	14.6435	14.579	13.0402	15.3492	0.732	0.176279
ENSG0000C	PEX11G	19:747687	1.08072	1.37415	1.14921	2.56552	0.947752	1.48962	1.0068	3.20971	0.052203	0.512059	0.8347	-0.36384
ENSG0000C	CKS1B	1:1549746	24.7866	29.6893	26.57	26.1032	14.8495	54.5338	89.1648	39.755	47.8797	11.8237	0.0208	1.00958
ENSG0000C	PIBF1	13:727277	25.055	36.554	9.86684	7.69938	8.023	20.6887	19.0062	5.04961	9.77919	11.1262	0.56115	-0.42088
ENSG0000C	IL6	7:2272539	0	1.79829	0	1.10665	0.046443	0	0	0	3.01215	0.403759	0.75935	0.444212
ENSG0000C	TRAV3	14:217237	22.3565	59.7376	10.3314	14.9922	16.9328	25.7877	41.9269	25.8289	19.0386	20.6314	0.721	0.189231
ENSG0000C	DHX35	20:389622	3.70953	4.70095	2.88951	2.50125	3.14118	4.3504	2.87884	3.8114	1.31956	5.27594	0.9916	-0.00558
ENSG0000C	RP11-406	10:171373	1.85679	0	0.666765	0	0.243454	0	0.231177	0.736647	0	0	0.4996	-1.05601
ENSG0000C	PATL1	11:596164	2.24525	3.12852	2.1709	2.34821	2.44256	3.53765	1.80139	3.72628	0.747113	8.69945	0.34705	0.452095
ENSG0000C	TLK2	17:624586	11.8903	10.7402	5.06509	3.90608	5.99554	12.8465	6.98068	8.72578	4.73252	9.6651	0.8239	0.103542
ENSG0000C	FAM169A	5:7462740	2.44171	2.07929	1.06445	3.63455	0.682796	0.185844	0.213908	1.02694	4.22062	1.62141	0.6602	-0.55756
ENSG0000C	TADA3	3:9749943	41.7023	51.5761	21.7058	22.0839	42.1354	61.0359	49.5931	27.7753	30.4194	40.5786	0.8814	0.157775
ENSG0000C	TMEM59	1:5402668	351.632	229.354	310.484	198.452	258.168	473.739	461.2	268.456	246.454	112.271	0.9383	0.038649
ENSG0000C	ZNF862	7:1498383	3.79407	6.1232	2.82605	4.03551	2.1023	1.52449	1.03679	3.76745	3.18162	5.67783	0.31305	-0.53957
ENSG0000C	ACP2	11:472393	9.23884	13.7717	16.0095	13.7356	16.7393	37.7052	17.5178	15.2619	29.2457	20.7971	0.06945	0.785298
ENSG0000C	LL0XNC01	X:1040625	0.981121	0.395735	0.894958	0.130547	0	0	0.093983	0	0	0.834788	0.39665	-1.34407
ENSG0000C	CTD-3222	19:164790	1.08312	1.01962	0.386003	3.10099	0.338503	0	2.68651	2.7299	1.31856	1.59147	0.7832	0.45703
ENSG0000C	LRRFIP2	3:3705265	15.7625	15.9843	10.1991	4.72022	13.8571	19.042	21.3575	8.75591	13.1878	10.4754	0.64745	0.202461
ENSG0000C	NIF3L1	2:2008893	17.77	22.0395	21.3254	21.2467	15.7289	22.217	16.591	14.9229	10.9493	12.7368	0.3485	-0.384
ENSG0000C	ZNF219	14:210167	1.01317	4.22528	0.336698	0.82346	1.05308	0.44911	2.05827	0.325103	0	0.787132	0.2439	-1.0725
ENSG0000C	BABAM1	19:172318	80.5791	96.9498	140.038	125.509	133.474	67.8792	90.1305	115.375	93.87	67.93	0.19745	-0.41342
ENSG0000C	NADK	1:1751231	28.596	27.3043	7.30031	7.99252	19.7623	21.3447	17.68	6.76525	3.47209	23.2391	0.13235	-0.603
ENSG0000C	AC004381	16:206102	0.8172	2.0255	0.202855	0.868262	0.208374	2.30703	2.01325	0.941158	0	1.19179	0.62615	0.462468
ENSG0000C	PIGA	X:1531945	5.98078	2.6851	2.80787	7.62264	3.30357	2.06928	3.17031	3.88249	10.1939	3.51709	0.86575	0.084915
ENSG0000C	WDSUB1	2:1592357	13.1455	6.82786	9.34438	12.4381	10.0178	13.2736	3.84814	3.98097	5.26972	5.25607	0.1574	-0.78726
ENSG0000C	UBXN6	19:440266	4.15923	6.72877	7.41783	6.64395	18.9017	6.27922	8.07857	10.6172	2.5543	13.4454	0.95345	-0.0582
ENSG0000C	IGFL2	19:461011	0	0.688442	0	0	0	0	0	2.49069	0	0	0.52865	2.39829
ENSG0000C	LRRC75A	17:164388	1.3446	2.7396	4.08471	1.84621	4.77138	3.44244	6.84793	1.41027	0	6.02143	0.97735	0.040215
ENSG0000C	RPL23AP1	1:2122855	0	0.483841	0.114419	0.293384	0.11751	0.795415	0.470787	0	0.611844	0.462486	0.44735	1.2874
ENSG0000C	UBL4A	X:1544837	40.8474	24.37	42.2793	36.9541	38.7546	30.4207	24.7916	34.0915	20.8493	31.9006	0.1873	-0.43188
ENSG0000C	ZNF688	16:305693	28.8268	20.9222	31.5891	30.9097	36.9006	34.8927	42.3702	31.7331	26.6565	23.4294	0.83865	0.090826
ENSG0000C	SLC25A23	19:643607	7.75537	7.74821	1.05507	0.49567	3.15554	5.58513	3.3923	0.416582	2.50787	3.19615	0.8158	-0.2234
ENSG0000C	CASK	X:4151493	20.3961	18.8572	5.78548	1.93326	6.20673	12.4756	24.7835	7.33994	8.86787	7.4069	0.6734	0.263233
ENSG0000C	BCL7B	7:7353635	57.7921	61.1939	75.286	96.2533	61.6729	88.4605	75.4157	62.5096	47.6907	51.8221	0.59445	-0.17033
ENSG0000C	BCS1L	2:2186379	30.5201	43.7269	40.373	38.6325	29.0682	23.6007	36.1481	29.3231	31.3579	39.6432	0.7914	-0.12802
ENSG0000C	RP11-646	4:5639336	0	0	0.702446	0	0.235674	0	0.073974	1.23683	0	0.504661	0.7399	0.931375
ENSG0000C	PLB1	2:2845714	0.106463	1.18371	0.350582	1.11197	0.477802	1.46587	0	0.316822	0.237219	0.258213	0.6463	-0.47012
ENSG0000C	ERH	14:693801	74.486	44.3709	67.6011	139.946	56.6465	98.3354	81.75	87.5538	93.9784	18.844	0.9764	0.013631
ENSG0000C	IDH2	15:900830	74.8447	112.532	138.097	136.317	121.058	145.348	180.916	182.234	128.713	155.426	0.36165	0.348022
ENSG0000C	RP11-216	9:1367913	1.40822	0.876583	0.167075	0.503937	0.775959	0.251034	0.71687	0.425792	1.25255	1.73836	0.72925	0.450314
ENSG0000C	Metazoa_S	1:1549440	0.958024	0	1.88917	0.972424	0.194351	0	0.559793	0	0.758114	3.13759	0.59545	1.03945
ENSG0000C	KRT18P5	22:204248	0.368276	1.45215	0	0	0.262905	0.304489	0.178044	0.44983	0	1.70692	0.76765	0.562608

ENSG0000(	ARL2BP	16:572450	64.997	61.77	72.9973	108.983	62.6059	29.7496	38.3064	51.7768	86.1531	43.4091	0.07685	-0.61073
ENSG0000(	ARL17A	17:465115	5.44272	9.42785	5.302	4.86586	5.86728	12.1117	11.6088	4.87857	6.6263	10.7708	0.53445	0.726621
ENSG0000(	DPF3	14:726192	0.508921	0.439181	0.071992	0	0.106918	1.17144	1.61276	3.28613	0.140258	1.14268	0.00445	2.72234
ENSG0000(	KIF18A	11:280206	2.08006	1.06141	1.23718	0	0.204299	1.38678	7.42058	1.32313	0	1.37657	0.3412	1.29433
ENSG0000(	POLR1C	6:4350696	19.3598	29.9338	44.5314	36.8507	44.0158	22.2891	25.4334	37.7002	19.5628	33.5867	0.533	-0.40755
ENSG0000(	RAB9A	X:1368912	31.0299	12.6847	41.2091	34.3233	30.5806	16.0411	19.0794	17.9061	12.1455	6.88201	0.0204	-1.20602
ENSG0000(	HOOK3	8:4284963	10.9235	9.51587	5.73803	5.65942	9.993	25.55	10.2502	5.87344	4.49918	21.1203	0.69745	0.480907
ENSG0000(	UBTD1	10:974988	2.84251	1.00103	0.962862	1.30392	2.55611	12.8594	1.53961	5.48724	1.63468	1.98055	0.0848	1.32451
ENSG0000(	POMP	13:286591	123.773	79.9463	91.6388	92.1739	51.6401	195.506	156.553	116.174	99.4577	15.7915	0.1201	0.520177
ENSG0000(	RRP15	1:2182852	0.319309	0.490644	4.54296	4.20372	5.85353	0.464844	0.382339	3.22248	4.66331	2.51631	0.363	-0.93299
ENSG0000(	CBR4	4:1683567	12.8992	11.3983	5.68141	18.4241	2.53353	8.67243	12.7704	4.11821	29.6853	10.4863	0.63735	0.464126
ENSG0000(	KRBOX4	X:4644685	20.3529	22.1843	22.888	28.9715	11.8724	11.7213	7.24377	10.2946	12.0055	12.7824	0.03815	-0.9446
ENSG0000(	DAGLB	7:6409125	6.54361	17.4378	5.47211	3.82056	4.0653	17.0208	5.80005	4.77033	0.917621	7.49005	0.98955	-0.01631
ENSG0000(	PMS2	7:5973238	6.25793	4.06032	4.43018	3.41805	3.25556	6.29585	3.30104	1.24237	0.888498	4.0172	0.26165	-0.75563
ENSG0000(	PSD	10:102402	3.17055	5.67734	4.22425	5.32235	2.68596	5.10374	3.25184	1.37587	1.0577	0.868095	0.42245	-0.99483
ENSG0000(	TAF13	1:1090624	3.54973	3.10696	15.8923	8.47818	8.14706	9.20745	4.76445	20.6655	8.78384	9.28941	0.602	0.33517
ENSG0000(	IL4I1	19:498896	32.0807	1.76647	19.3989	57.9698	45.2009	160.756	25.813	70.4399	87.1796	106.185	0.05445	1.46308
ENSG0000(	PDAP1	7:9932589	15.48	8.33365	18.6943	12.0605	22.143	13.9352	11.5144	30.4027	5.68191	10.6862	0.7987	-0.23752
ENSG0000(	TNFRSF6B	20:636578	0.692151	0	0	0	0	6.61736	0	0	0	0.368859	0.2799	3.93734
ENSG0000(	HACD3	15:655304	21.9145	18.6665	22.1131	16.9164	30.5485	20.8197	27.4474	20.7194	6.76706	32.9906	0.59345	-0.2362
ENSG0000(	ACACA	17:370849	4.63488	6.27653	2.89125	1.95756	1.87771	7.18717	11.9624	9.09176	13.3376	7.7749	0.0417	1.69236
ENSG0000(	FKRP	19:467195	3.55578	4.81482	2.5861	3.71748	3.89442	1.20063	4.19823	3.25033	4.76243	7.89799	0.74885	0.27391
ENSG0000(	ADGRB2	1:3172711	0.063362	0.593966	0	0	0.086231	0.306538	0	0.642123	2.32366	0	0.09905	2.42853
ENSG0000(	TARBP1	1:2343913	11.8021	26.3306	9.80973	11.2555	6.88717	7.36936	19.762	9.7009	11.6296	15.3417	0.89615	0.070072
ENSG0000(	NDC1	1:5376545	4.51382	5.99748	2.44712	1.63915	1.50463	1.87308	3.39866	2.12671	1.6051	1.72127	0.2989	-0.51461
ENSG0000(	CYFIP1	15:228386	2.9877	7.28793	1.83787	1.84988	1.58652	16.1027	1.40461	1.13088	0.281961	3.48937	0.8771	0.207405
ENSG0000(	BCAR3	1:9356178	0.385185	1.15593	0.04334	0.339545	0.474051	10.4831	2.28647	2.51336	0	0.271194	0.01465	2.64004
ENSG0000(	POLK	5:7536848	10.4218	14.8867	4.0166	5.83304	4.76596	10.7881	11.3418	7.135	7.7433	7.15423	0.91675	-0.05926
ENSG0000(	AC007163	2:2006782	1.08802	0.021149	0.655186	0.230216	0.366322	0	0	0.124764	2.49966	0.025795	0.8716	0.31889
ENSG0000(	LINC0121!	3:1081258	0.991695	0.734508	0.863901	0.632655	1.24399	0.161096	0	0.083047	0.329721	0.756349	0.18035	-1.90623
ENSG0000(	AC005538	2:2338654	0.178053	0	0	0.000291	7.83E-05	0.333325	0.475019	0.56971	3.17289	0.691491	0.29455	2.93702
ENSG0000(	ANG	14:206840	0.476114	0.680539	0.537661	0	0.671185	8.08902	0	0	6.11326	0.270869	0.11985	2.63792
ENSG0000(	YTHDF3	8:6316855	17.9594	21.5077	6.45145	10.2458	8.04795	13.7942	16.3538	8.72924	13.3162	16.5226	0.73775	-0.11772
ENSG0000(	FEZ2	2:3635592	3.64558	4.56468	3.43215	1.85027	5.36657	5.74831	5.27278	3.0894	0.492077	5.10898	0.8827	0.118862
ENSG0000(	SUMO1P3	1:1602892	1.8425	0.811089	0.432125	0	0	0.774462	1.06787	0.324017	0	0	0.81835	-0.4446
ENSG0000(	IRAK3	12:661888	0.405403	1.63095	0.12358	0	0.037376	7.9211	0.044913	0	0.770306	0.155587	0.147	2.11945
ENSG0000(	TMX4	20:797734	29.4254	14.6538	19.2859	11.7409	15.9639	27.3039	34.2277	19.0534	22.7789	20.2149	0.6017	0.232948
ENSG0000(	RNASE6	14:207810	5.30107	16.3412	7.15064	13.5865	5.90974	22.3144	0.424578	3.20606	19.7046	1.50994	0.974	-0.01913
ENSG0000(	MIR210H(	11:565659	0.199536	0	0.286978	0.189507	1.55077	5.8575	0.87235	0	0	1.23794	0.25705	1.4913
ENSG0000(	ZNF433	19:119876	1.86042	2.91513	2.39054	2.33639	0.837514	0.888279	0.4556	0.80572	0	0.619114	0.0894	-1.76429
ENSG0000(	RP11-967I	12:281332	0.754837	0.40955	0.542781	0.320931	0.296977	0.864892	0.209039	0.012076	0	0.514205	0.5903	-0.71768
ENSG0000(	GNRH1	8:2541925	2.07945	0.689769	0.63477	1.24045	0.42882	5.67408	8.71681	2.06232	0	3.83241	0.25145	2.09901
ENSG0000(	RP11-348I	X:5135694	0	2.52168	0.563908	0	0.327854	0	0	0	0	0.271013	0.20055	-3.11463
ENSG0000(	DAPP1	4:9981683	9.91773	38.2391	3.90618	4.83355	4.28431	21.777	25.4529	7.04246	9.58445	10.5681	0.61185	0.279009
ENSG0000(	BIVM-ERC	13:102799	3.46127	0.119434	2.51158	2.47096	1.93066	6.60049	2.52255	1.40536	0.324653	1.20113	0.94305	-0.07378
ENSG0000(	DAZAP1	19:140756	11.5258	9.06768	13.3781	9.71657	19.4797	9.42521	7.71812	15.9705	9.21739	23.6491	0.995	-0.00402
ENSG0000(	RP11-449I	5:1462035	8.83015	12.5441	8.2662	11.0757	16.6788	9.22857	11.2821	7.93748	8.21134	17.8802	0.49195	-0.34293
ENSG0000(	NCAPD2	12:649188	18.2762	33.0666	5.84088	2.84507	6.081	12.0613	9.33008	8.88894	6.3166	13.3397	0.61045	-0.45013
ENSG0000(	RP13-20L1	17:823892	0.308054	0.26906	0.516952	0	0.081463	0.790982	0.32175	0.184463	0.561855	0.101028	0.65405	0.684645
ENSG0000(	EFNA4	1:1550637	1.53631	1.94562	3.4108	3.4804	4.85489	3.48948	0.980302	4.70953	0	4.95029	0.85115	-0.23428
ENSG0000(	SIK2	11:111602	0.169872	0.293798	0.506529	0.258318	0.570237	0.004622	0.897725	0.581837	0.10485	1.28343	0.80415	0.418025
ENSG0000(	KCTD21-A	11:781397	0.521491	2.66069	0.030092	0.358812	1.01913	2.31618	0.200641	0.177192	0	0.599929	0.6285	-0.58659
ENSG0000(	MAGEF1	3:1847103	7.00606	15.7981	7.79257	11.0546	5.96115	1.6967	10.7923	7.51446	1.1997	5.36368	0.1119	-0.85428
ENSG0000(	FRG1CP	20:285807	20.9973	16.5554	16.2145	20.3394	13.3091	12.8341	13.6717	19.9061	16.5438	5.96509	0.6923	-0.23771
ENSG0000(	CUL2	10:350085	18.6323	18.2293	7.01558	10.2293	6.52887	18.5961	16.0185	6.64466	17.0231	8.0075	0.76475	0.120597
ENSG0000(	CD79A	19:418771	3.18235	76.3749	12.7172	26.1284	11.6683	0	11.7829	2.41263	3.47444	1.39982	0.1576	-2.78247
ENSG0000(	EAF2	3:1218351	8.14471	10.3421	7.50473	18.1746	4.49013	11.507	4.7436	4.76367	26.171	3.25935	0.84025	0.123899
ENSG0000(	IGLV2-14	22:227586	0.126236	24.6001	4.49729	0.257974	2.02926	0	0	11.0879	0	0	0.61415	-0.9936
ENSG0000(	SPG11	15:445370	15.8628	16.8909	9.21056	10.5505	7.11871	9.25663	9.47407	10.1586	14.2293	25.2139	0.8114	0.134798
ENSG0000(	TRMT6	20:593723	12.478	13.1537	12.1111	13.7123	12.2889	14.5036	17.0934	13.3353	18.2139	15.8195	0.55225	0.23485
ENSG0000(	PPM1N	19:454852	2.96398	4.66638	4.25692	1.75905	3.49474	1.94668	8.39489	3.7664	0	4.58416	0.9484	0.059671
ENSG0000(	SH2B3	12:111405	7.72655	7.43911	4.32179	2.93422	5.83571	4.87116	2.0359	4.76895	2.88224	10.8112	0.698	-0.16512
ENSG0000(	AC005306	19:198543	2.25036	1.62956	1.5439	1.77397	0.517804	3.51268	5.58278	1.43171	2.9953	1.95577	0.5624	1.104
ENSG0000(	CCR8	3:3932970	0.723171	4.56041	0	0	0	1.57001	36.6321	2.05003	0	0	0.18775	3.13391
ENSG0000(	TRAK2	2:2013772	10.3165	7.40209	2.10858	3.1984	3.23395	7.04996	6.8449	4.00697	8.84761	7.21498	0.9226	0.073375
ENSG0000(	TBCD	17:827520	83.861	46.182	63.1992	104.869	58.5512	155.826	272.126	163.125	112.052	168.445	0.0001	1.42821
ENSG0000(	RP11-458.	17:406249	3.55608	7.48781	4.46687	3.89878	1.88112	0.417302	4.01878	2.75886	3.13779	1.53224	0.5678	-0.78433
ENSG0000(	LINC0050	4:1447046	0.815206	0	1.50269	1.7745	4.81997	0.417917	0	0.104451	0	0.948796	0.12525	-2.95982
ENSG0000(	MTMR4	17:584895	12.2376	9.73903	7.88441	9.71395	5.79053	5.4015	6.25343	5.27879	3.34678	10.457	0.4086	-0.58644
ENSG0000(	CCDC115	2:1303382	45.5068	59.909	121.712	101.484	86.384	47.831	61.4288	79.4144	85.3874	44.9764	0.47135	-0.36543
ENSG0000(	RP11-384I	4:1185917	2.01697	2.47713	1.04354	0.34454	1.23636	1.51916	1.35999	3.63553	0.408096	4.09768	0.2624	0.620223
ENSG0000(	FNBP1	9:1298871	63.9513	85.4373	62.2989	50.4714	85.2398	56.4905	111.804	75.6677	69.4676	136.806	0.8055	0.111975
ENSG0000(	RP11-661.	8:1435734	0.700531	0.230563	0.886314	1.13404	0	0.619595	0.373191	0.207927	0	0	0.41635	-1.4033
ENSG0000(	SNX13	7:1779076	8.15954	10.5112	2.96269	6.3841	2.02853	8.13583	3.19903	2.88938	1.43569	4.70754	0.21855	-0.66844
ENSG0000(	CPEB4	5:1738882	4.23731	3.44736	1.73018	1.11129	1.15855	1.87567	3.04328	1.99904	0.290343	3.47707	0.4912	-0.3879
ENSG0000(	DNAJB11	3:1865384	64.4618	55.1625	50.2321	57.7271	51.068	56.6319	102.101	56.4632	58.1771	45.5249	0.64555	0.189505
ENSG0000(	MLC1	22:500593	0.484183	0.312641	0.319607	0.933336	0.327798	0	0.477071	0	0	0	0.30975	-2.80413
ENSG0000(	MEF2A	15:995654	6.27034	14.8867	3.01593	2.47424	7.11574	13.2763	8.80905	3.33744	6.13489	9.30271	0.7573	0.185525
ENSG0000(	PDSS1P1	9:4984389	0.041073	0	0	0	0	0	0.32433	0	1.56644	0	0.3278	5.94063
ENSG0000(	TMSB15B	X:1039174	3.27263	3.00437	2.53218	2.14426	1.51723	5.64316	3.89499	2.23864	2.55155	2.09552	0.71905	0.358524
ENSG0000(	RP11-278	12:326791	0	0	0	0	0	0	0	0	2.5323	0	0.2145	inf
ENSG0000(	HACE1	6:1047280	5.45363	5.37946	1.3885	2.44416	1.31334	4.02914	4.10456	2.56708	3.00948	2.07967	0.5506	0.334996
ENSG0000(	CH17-262	14:106025	0	68.5438	0	0	0.314394	0	0	1.77934	0	61.9603	0.39045	0.822138
ENSG0000(	FBXW4	10:101610	9.35886	16.5403	8.79421	11.0323	11.5232	16.1124	15.5847	11.3185	13.7214	20.4915	0.2729	0.48176
ENSG0000(	ASB2	14:939341	1.66664	11.5262	1.05211	3.78391	1.16755	32.5449	49.5461	24.5367	15.288	24.0228	0.00035	3.03194
ENSG0000(	FKBP3	14:450840	91.0778	74.3457	75.2457	77.2756	61.9085	117.274	88.9754	64.0332	64.1013	41.14	0.8688	0.068197
ENSG0000(	CTD-2012	5:1594625	0.746573	1.32892	0.368589	0.865479	0.489905	0	0.563538	1.21355	0.941503	0.384924	0.90725	-0.17716
ENSG0000(	AMPD3	11:103083	7.44069	7.50937	7.03039	6.29569	6.02033	7.98614	0.253178	4.4638	6.58765	9.92006	0.4999	-0.30914
ENSG0000(	EBPL	13:496607	27.4048	28.1812	30.343	16.943	20.9128	27.3686	39.3927	20.9851	15.6597	6.02981	0.66255	-0.23739
ENSG0000(	CTB-119C	7:2615223	0.676383	0.401962	0.808245	0	0.139056	1.55455	1.25104	1.24063	2.94114	0.227587	0.39415	1.91845
ENSG0000(	AC079250	2:4769071	5.13917	3.5648	4.93426	6.24658	2.37831	3.47457	3.06062	2.75401	2.92309	0.20304	0.48935	-0.85514

ENSG0000(	RHOBTB2	8:2268765	1.97904	1.48752	0.966374	0.934666	1.3015	0.265806	1.94368	0.40963	0.877422	2.41441	0.81015	-0.24275
ENSG0000(	RAB5A	3:1994707	33.7416	34.3574	23.4812	28.7021	17.9474	16.8789	32.1816	33.6159	30.6137	19.1013	0.8133	-0.08275
ENSG0000(	PEX10	1:2391774	4.95649	5.9722	8.40512	6.97475	5.88747	3.48743	3.1082	9.21413	7.43061	10.0525	0.93825	0.109414
ENSG0000(	CTA-384D	22:505423	0.390597	0	0.208805	0.397421	0	3.10578	1.53015	0.970488	2.47629	0	0.20045	3.69092
ENSG0000(	USP47	11:118414	48.9536	47.5603	21.0768	25.8988	24.0753	41.9589	26.5731	23.1663	38.0752	37.6268	0.765	0.111974
ENSG0000(	STXBP5	6:1468419	3.13058	2.56333	1.14035	0.684183	2.31401	0.812515	2.61844	1.19916	0.295997	1.35708	0.3876	-0.49283
ENSG0000(	CCDC91	12:281332	56.0316	64.1828	54.8938	40.8451	62.4529	46.7568	72.4536	51.2111	44.8822	69.1216	0.96885	-0.01378
ENSG0000(	RP11-572(	4:1712820	7.07353	9.78815	8.42848	8.50021	7.12124	13.3268	12.0333	9.80698	11.7742	14.245	0.3035	0.58205
ENSG0000(	TMEM86B	19:552266	2.86455	6.40022	2.9124	0.551131	3.27352	1.86344	7.9433	1.35694	0.084319	8.77172	0.86105	0.201498
ENSG0000(	C1orf145	1:2282035	0.318259	1.14837	1.16841	0.529343	0	0	1.0581	0.033029	0	0.340811	0.4647	-1.29918
ENSG0000(	RFX7	15:560872	1.92592	3.07482	1.04095	0.689688	1.41268	0.783346	1.38182	2.28205	0.945047	3.28904	0.59175	0.299529
ENSG0000(	LAT	16:289739	260.661	393.174	411.707	322.593	401.912	340.53	371.988	344.928	330.937	354.119	0.7636	-0.10713
ENSG0000(	HMGA1	6:3423687	26.8549	34.5589	10.5085	10.8364	24.5048	47.6038	32.2255	25.833	15.4436	52.6195	0.1032	0.656646
ENSG0000(	SLC30A4	15:454305	0.693607	0.704924	1.04516	2.53892	0.426878	0.430722	0.85452	0.328383	1.06611	0.8332	0.4658	-0.7404
ENSG0000(	RAB3IP	12:697386	11.6261	5.31533	4.97641	7.82712	7.23385	11.704	14.3883	14.6824	12.382	6.71333	0.2108	0.657546
ENSG0000(	ANKRD17	4:7307337	7.75459	8.13535	7.85777	3.43603	10.9087	7.03092	11.5782	7.09755	4.18514	22.9429	0.68045	0.197079
ENSG0000(	RP11-563	2:3216504	2.45753	3.02486	5.55971	2.39051	2.16968	0	3.28137	2.97684	3.49395	1.53508	0.6129	-0.43664
ENSG0000(	LRRC59	17:503732	10.7129	9.24622	11.7293	14.8682	12.9514	10.2787	11.2802	11.7805	13.2411	15.4005	0.883	0.059293
ENSG0000(	C16orf74	16:856900	2.30271	8.23529	4.94243	6.9489	2.19027	0	2.37009	0.433069	2.32511	0.757652	0.10065	-2.03727
ENSG0000(	DCAF7	17:635504	22.4256	25.5013	27.9904	26.1345	29.8677	14.8479	26.1934	33.153	34.7271	47.1929	0.98075	0.016418
ENSG0000(	RP11-191	2:4844004	2.17346	2.18699	0.572496	0.475486	0.966897	1.74443	1.4454	1.19135	0	0.598771	0.8771	-0.31496
ENSG0000(	ZNF787	19:560873	1.57856	2.85156	0.813862	0.550934	1.8317	1.57304	2.76425	1.09594	2.37806	1.87001	0.74335	0.386131
ENSG0000(	MORF4L2	X:1036754	66.2696	73.9084	87.1765	94.1187	62.0111	60.2587	74.2619	57.6267	83.2623	45.6471	0.4583	-0.23093
ENSG0000(	PSMA3-A	14:579997	34.3695	47.7774	66.9975	76.3133	38.9947	36.7097	40.7627	73.1306	82.5449	80.5903	0.70245	0.194395
ENSG0000(	STOML2	9:3509977	58.662	51.4535	78.662	88.3679	72.0402	44.0523	53.7274	60.5725	70.2586	33.2331	0.22295	-0.41331
ENSG0000(	SBK1	16:282925	6.06634	8.04737	0.911413	0.610318	3.14621	2.11042	1.6697	0.864054	0.023402	2.04891	0.08055	-1.37338
ENSG0000(	KIF11	10:925932	3.78211	7.16261	0.155334	0.148355	0.353122	7.54632	4.80844	0.611744	0.00881	2.4681	0.31955	0.509587
ENSG0000(	OST4	2:2704236	423.466	276.017	631.473	636.789	294.091	556.133	493.786	575.591	440.572	84.4866	0.7175	-0.13723
ENSG0000(	DDX52	17:376097	13.3516	12.363	11.2961	6.70051	9.7395	21.2921	9.63371	7.37762	3.12381	13.128	0.406	-0.32032
ENSG0000(	FAM45A	10:119104	23.456	25.4889	24.988	23.1388	25.1643	34.7685	36.1614	21.972	44.5787	30.2718	0.2764	0.403142
ENSG0000(	RP11-379	7:3563265	0.936887	0.70813	1.18323	0.606952	0.622834	1.44492	1.28946	1.61621	0	1.95523	0.7233	0.532507
ENSG0000(	CTH	1:7041121	0	0.546345	0.686426	1.05114	0.429117	0	0	0	0	0.141866	0.24775	-4.5024
ENSG0000(	RPS6KB2	11:674284	51.1147	71.03	29.9587	43.1986	35.1814	51.9802	60.2376	36.0833	28.0488	28.0794	0.51265	-0.22069
ENSG0000(	NAA10	X:1538615	57.6757	73.6507	77.2869	70.6043	67.4406	69.5327	101.928	73.4731	58.8271	30.2489	0.8297	-0.13807
ENSG0000(	LAG3	12:677251	76.8308	19.6001	42.872	31.3137	61.6467	255.331	388.342	163.828	42.6472	131.719	0.00005	1.92668
ENSG0000(	IGHG1	14:105736	0	353.935	0.795322	1484.31	281.733	0	0	0.707824	0.274917	99.6557	0.3659	-4.54374
ENSG0000(	RLIM	X:7458521	1.90737	3.0221	1.40428	1.83969	2.27308	3.35549	2.93609	2.41589	1.48548	3.97726	0.89515	0.076435
ENSG0000(	XXbac-BP	6:3166122	0.485523	0.113255	0.859138	0.524878	0.113424	0.219924	0.835887	0.941998	0.776632	0.329384	0.5979	0.860988
ENSG0000(	TFB2M	1:2465405	21.1407	14.4438	12.0669	15.9739	9.28234	8.07277	16.3608	7.93749	16.4856	3.65497	0.40075	-0.40425
ENSG0000(	HBD	11:523267	0	0	0.990257	26.0647	0	0	0	0	0	0	0.05095	#NAME?
ENSG0000(	GRK5	10:119207	5.01251	12.1991	4.00751	3.28277	4.35674	8.59306	7.59377	2.71577	3.10812	8.11118	0.9394	0.044537
ENSG0000(	DVL1	1:1331313	4.34737	6.95901	1.49059	1.75979	3.405	7.62786	3.698	2.04017	0.638132	6.07611	0.71015	0.28418
ENSG0000(	UBE3D	6:8289239	4.21998	6.30013	3.49252	4.02687	4.35691	1.11484	1.79101	2.67418	0	3.81322	0.1679	-1.3018
ENSG0000(	IQCB1	3:1217697	22.5982	28.9255	18.6161	19.0721	9.04003	21.1269	17.676	14.21	8.37287	14.119	0.31765	-0.40154
ENSG0000(	PWP2	21:441072	11.0584	9.56873	11.7495	16.8868	9.40639	7.43272	3.45471	6.11482	13.0425	6.76672	0.25995	-0.92622
ENSG0000(	PHF3	6:6363581	17.433	28.1429	8.384	5.26929	13.6105	14.8088	14.5465	5.93438	12.9167	21.4304	0.67855	-0.17404
ENSG0000(	MED9	17:174769	8.27106	11.4083	8.15207	8.10413	8.16819	13.251	12.8174	7.35918	3.63769	8.95478	0.99645	0.001987
ENSG0000(	SNX22	15:641517	0.495411	1.92141	0.657881	1.03341	0.404879	1.08418	1.20058	0.828225	0.390398	0.747293	0.90885	-0.14174
ENSG0000(	FBXL22	15:635045	0.949884	1.12266	2.18028	1.37526	1.37961	2.51549	0	0.665929	0.340479	2.53054	0.82155	-0.2673
ENSG0000(	AC129492	17:818833	12.5211	9.43425	12.8631	17.7809	12.9022	13.4997	13.0455	8.35966	8.93019	5.57938	0.71515	-0.6385
ENSG0000(	FBLN7	2:1121383	4.57326	14.6405	1.47759	2.03755	0.413027	14.5244	9.38979	14.2673	27.3256	5.87676	0.01155	1.8045
ENSG0000(	HIGD1A	3:4269266	109.146	70.1404	89.4753	102.562	55.6167	125.357	135.781	75.6407	119.987	25.5246	0.48415	0.231263
ENSG0000(	PSMF1	20:111326	43.1413	44.2691	69.4751	51.0161	47.1651	39.1983	64.875	67.5046	64.662	50.1035	0.6892	-0.16501
ENSG0000(	RP11-326	11:307630	0.855145	2.04636	0.272766	0.676217	0.78095	1.41047	1.12693	1.06631	4.10436	0.190487	0.65635	0.662437
ENSG0000(	REPIN1	7:1503637	48.888	58.6705	26.9064	20.8672	31.6022	32.1158	22.9326	14.2822	15.0456	41.3732	0.14365	-0.55929
ENSG0000(	SUCLG2	3:6736045	38.8864	42.7411	32.8584	32.6799	26.3828	45.5858	36.4502	26.003	18.3767	24.6456	0.5138	-0.22107
ENSG0000(	CDK17	12:962782	30.758	30.3333	13.2737	21.645	12.4906	20.3733	16.2672	19.8584	12.4247	16.1666	0.37145	-0.31292
ENSG0000(	CCDC107	9:3565828	135.454	59.3705	155.517	149.816	130.193	177.432	114.339	137.507	151.204	67.7604	0.96175	-0.0181
ENSG0000(	BAZ2B	2:1593189	26.0795	25.9109	11.7876	11.6256	16.5757	15.2995	7.73532	9.43102	1.35808	18.1042	0.05095	-0.90411
ENSG0000(	RP1-102E	12:643900	6.42847	3.34156	0.744318	0.260155	1.50175	2.52015	3.9081	1.87602	0.184788	2.61885	0.98885	0.020078
ENSG0000(	RCN1	11:317847	19.0212	11.3989	9.41216	9.88067	15.4881	9.98999	9.71338	5.25025	8.87534	8.10131	0.1342	-0.61817
ENSG0000(	RP11-297	8:1179557	0.946311	1.87847	4.76349	3.03939	0.662654	0.784743	0.808893	2.85152	3.79982	0.739555	0.8066	-0.24796
ENSG0000(	PLCXD2-A	3:1116746	0.738064	0.242037	0.177114	0.3478	0.097353	0	0	0	0.122333	0.113906	0.2498	-2.32575
ENSG0000(	CNPPD1	2:2191718	41.1403	63.96	37.6856	29.0234	34.8848	34.2154	53.3026	47.4208	32.2718	54.9589	0.8468	0.074328
ENSG0000(	PRSS21	16:281717	2.03024	0.748385	1.97941	0.761414	13.8845	2.34473	2.98125	2.53417	3.36635	3.11228	0.489	-0.55568
ENSG0000(	PEBP1P3	1:1986386	0.466401	0.750311	1.39147	2.09952	0.236737	0.878332	1.11001	0.84262	3.47202	0	0.80455	0.383789
ENSG0000(	NPHP4	1:5862810	1.42463	2.07954	0.586362	0.768013	0.892919	4.5482	0.655161	1.30318	0.656118	3.07035	0.46595	0.677223
ENSG0000(	IGKV2-28	2:8922169	0	31.8681	4.22499	1.00552	1.27609	0	0	0	0	0.621517	0.12915	-4.02087
ENSG0000(	LEAP2	5:1328723	0.552711	0.899979	0.736587	0.799123	0.572521	2.78856	2.63024	1.45091	1.98783	1.50903	0.24005	1.6501
ENSG0000(	RP11-114	X:4857977	1.59391	0.087228	0.367556	0	0.278595	0	0.415191	0.3976	2.43007	0.328784	0.5889	0.883901
ENSG0000(	CTD-2636	5:4328760	0.62126	0.653427	0	0.757242	0.539764	0	0.79724	1.07457	1.91145	0.179599	0.5131	0.836831
ENSG0000(	ZNF583	19:563936	4.64274	4.17039	6.60749	4.69438	5.38501	18.564	10.1032	7.02493	5.16273	9.00649	0.19205	0.828902
ENSG0000(	RP11-20C	1:1089702	7.15728	3.24913	8.31839	8.79855	1.16313	6.19758	6.03851	4.13978	5.37361	0.451324	0.90695	0.153638
ENSG0000(	STYXL1	7:7599633	51.445	34.6259	71.5241	43.7912	37.3454	90.7355	41.3478	49.1812	54.1817	17.3629	0.93065	0.038729
ENSG0000(	CD72	9:3560530	7.29379	19.4503	16.7521	14.0284	8.21573	43.2058	13.4525	10.2863	9.2839	9.82224	0.63805	0.278074
ENSG0000(	PF4	4:7398107	0	0	10.1027	71.889	1.3034	0	0	0	0	0	0.00005	#NAME?
ENSG0000(	RP11-216	9:1285046	0.338178	1.418	0.90161	1.49941	0.310262	0	2.4992	0	0	1.18825	0.90325	-0.17606
ENSG0000(	TP53BP2	1:2237798	11.5506	9.7406	3.63491	2.84541	4.32976	5.89557	10.1173	2.88014	5.73014	4.92463	0.9222	-0.0439
ENSG0000(	IK	5:1406387	172.447	204.067	193.555	176.84	257.893	207.869	177.8	133.386	175.967	213.392	0.64285	-0.18574
ENSG0000(	RP11-79D	9:7957177	0.40559	0.451396	0.379113	0.399491	0	2.85676	0.753462	0.269932	1.57428	0	0.14975	inf
ENSG0000(	MRPS9	2:1049362	39.7067	28.4219	44.7519	43.9054	39.1828	39.2023	33.8363	19.2017	31.0583	23.6884	0.25255	-0.41582
ENSG0000(	USF3	3:1136483	2.57064	3.2315	1.25959	1.23355	1.8747	3.78711	4.16138	2.32047	0.995552	3.58943	0.2962	0.710536
ENSG0000(	CHAF1A	19:440266	6.97721	13.343	1.7396	3.79285	5.77528	9.203	7.68378	4.33018	1.80196	11.3673	0.78325	0.168346
ENSG0000(	HNRNPH2	X:1013908	56.5415	57.396	78.1986	80.7948	57.8896	54.9681	49.2061	70.5114	94.9892	57.1386	0.92375	-0.04761
ENSG0000(	ANXA6	5:1511007	89.2871	61.8302	119.437	75.5981	120.783	40.2368	56.6159	102.064	76.1664	89.9163	0.3891	-0.27275
ENSG0000(	STARD5	15:811595	6.98068	12.0173	3.11589	1.95179	3.28992	4.11601	3.26122	2.87906	3.55862	3.39819	0.96	0.045174
ENSG0000(	CAMK2N1	1:2048239	0.449684	0.279083	4.15151	1.14648	2.62993	1.17894	1.17079	2.32737	0	1.32642	0.58525	-0.6367
ENSG0000(	DTNBP1	6:1552280	27.5631	34.5064	28.4178	17.5631	41.7616	43.7183	29.6143	20.6093	29.8692	39.687	0.7846	0.110394
ENSG0000(	SUCLG2-A	3:6765469	1.75414	1.17633	0.602663	1.58783	0.580778	0	0.576133	0.379936	0	1.69276	0.43635	-1.02373
ENSG0000(	RRAGB	X:5571773	15.4283	11.472	9.86182	14.3068	5.98554	12.3617	3.52615	5.40403	14.5392	5.18221	0.2433	-0.51941
ENSG0000(	RP11-100	15:325198	1.55237	1.5542	1.89591	3.23467	1.45747	0	1.55884	0.498413	2.64346	1.48919	0.5776	-0.76143

ENSG000C	CYSLTR1	X:7827146	0.718032	8.96218	0.675317	1.78625	0.450289	2.15908	1.29039	0.083217	0.015691	1.13571	0.11865	-1.41685
ENSG000C	ALG3	3:1842304	44.3795	42.4158	82.2665	68.6727	84.7952	31.2419	47.6291	60.1716	73.0635	63.9728	0.46765	-0.242
ENSG000C	RP11-368I	16:896807	0.158543	0.312958	0.312327	0.656194	0.25562	0	0	1.07952	0	0.23477	0.88935	-0.22634
ENSG000C	WAC	10:285226	42.9596	61.8317	26.5418	17.7828	35.018	41.1074	46.9037	28.8506	23.0976	57.2771	0.8458	-0.07534
ENSG000C	DHRS11	17:365917	3.91962	2.84443	4.02536	2.89782	2.60252	7.23152	3.86882	2.72573	0.522805	4.86234	0.90725	0.098407
ENSG000C	MYD88	3:3813847	85.6886	88.2488	67.5448	82.765	54.6829	94.6977	96.6115	81.6997	68.5445	72.2529	0.6621	0.142202
ENSG000C	CELA1	12:513284	0.08349	2.30541	0.525111	0.133541	0.040411	0	0.032784	0	0	0	0.267	-6.66515
ENSG000C	OLA1	2:1740724	42.3453	26.2553	31.9978	24.9928	36.0848	51.7675	40.207	41.1026	30.627	16.6501	0.76185	0.117658
ENSG000C	IGHG2	14:105643	0	14.1873	0.145831	29.1511	96.0745	0	0	0.028148	0	4.50727	0.2522	-5.99732
ENSG000C	EBNA1BP2	1:4316417	54.7569	50.5787	65.2959	64.1624	70.0447	53.7631	72.4589	70.2016	36.5574	23.8891	0.39215	-0.29347
ENSG000C	ZNF335	20:459486	12.2583	17.5071	5.05974	3.81412	5.35259	12.6846	10.744	7.48041	3.76985	9.84037	0.97865	-0.01148
ENSG000C	RN7SL1	14:495709	78.5967	11.7763	3759.26	625.237	909.145	0	39.8964	5535.98	2933.57	703.547	0.53955	1.346
ENSG000C	C15orf57	15:404880	25.7017	20.6264	31.3576	38.517	29.1697	42.7548	38.3356	42.9895	26.8624	16.965	0.9637	-0.01778
ENSG000C	HOXB3	17:485408	0.460043	0.555021	0.928994	0.441051	0.517594	0.772394	2.11921	0.471489	0.124479	1.94623	0.33115	0.936848
ENSG000C	RP11-35G	8:1082012	1.0756	1.51739	0.921648	2.11934	0.286	0	2.15363	2.59622	0.131011	1.11517	0.9354	0.114453
ENSG000C	ERP44	9:9997917	29.417	27.8481	24.156	21.0914	23.1786	30.7373	26.12	22.8408	30.1564	16.4532	0.9913	-0.00346
ENSG000C	SLCO3A1	15:918536	28.3819	24.7619	11.3107	7.4401	10.9119	6.0756	17.0594	4.86267	3.54689	12.6768	0.02815	-1.18871
ENSG000C	ARMT1	6:1514522	27.2719	12.9825	16.3219	15.9418	12.5026	21.374	10.3608	13.7051	1.90286	13.594	0.4171	-0.46785
ENSG000C	CTC-559E	19:197573	2.083	5.43603	2.48802	0.886116	1.26846	0	0.479119	2.79522	0.422582	2.17182	0.3263	-0.99331
ENSG000C	ST8SIA4	5:1008069	11.7429	9.13861	4.25685	7.12016	3.3963	11.2504	12.5714	10.4077	7.37363	8.11443	0.58295	0.290242
ENSG000C	SRGAP2C	1:1211676	1.90621	3.1113	1.49242	0.771499	1.07179	2.37416	2.99421	1.50641	1.88363	2.87841	0.83115	0.137634
ENSG000C	IGHG4	14:105624	0	21.5605	0.038054	72.6526	26.8108	0	0	0	0	12.83	0.0667	-3.44086
ENSG000C	HCP5	6:3139978	69.5088	67.2371	51.823	35.0579	43.9242	50.4657	59.2437	55.1435	38.9746	47.5827	0.75825	-0.10344
ENSG000C	MAP3K2	2:1272987	23.3539	26.316	6.05306	2.44058	9.97007	16.4715	22.7394	7.13153	2.26338	14.5456	0.73655	-0.33484
ENSG000C	ZNF480	19:522695	12.4459	11.5788	13.5487	9.65166	7.29138	11.2571	9.0826	8.15281	6.52698	5.71034	0.49705	-0.51309
ENSG000C	SPSB3	16:177320	142.781	142.366	234.614	239.92	176.596	119.98	156.117	150.625	228.116	167.556	0.61435	-0.22712
ENSG000C	MMADHC	2:1495696	109.205	71.3654	90.4415	83.5173	54.1912	85.1369	97.7972	66.1433	77.1661	33.8683	0.6991	-0.13066
ENSG000C	CTB-131K	17:392526	1.52925	2.56161	2.06997	2.63115	2.556	6.61773	3.48221	4.86512	5.27815	4.04556	0.48905	1.08537
ENSG000C	ABCA7	19:104010	46.5893	41.997	16.1673	11.202	15.4118	16.089	22.9554	17.118	4.68831	31.4857	0.4961	-0.35055
ENSG000C	ELP2	18:361294	25.0132	27.9114	33.277	32.3241	15.9665	11.8121	16.2988	26.9912	21.507	29.7918	0.39665	-0.33647
ENSG000C	CEP70	3:1384943	6.03044	4.52547	2.83227	4.77794	3.6372	2.10457	2.66142	2.73761	3.33471	2.23078	0.156	-0.82154
ENSG000C	KANSL1	17:460299	9.6955	12.787	6.3231	6.65601	7.79536	10.9413	7.28138	9.6506	3.0202	20.9123	0.5623	0.261922
ENSG000C	TCEB1P19	1:8853751	2.87125	1.45737	0.196896	1.33524	0	3.1302	0	0.706003	0	0.252949	0.87175	-0.27678
ENSG000C	SEC23IP	10:119892	16.4146	20.6052	16.0668	13.7029	9.29348	7.4659	8.48557	11.9413	16.6659	15.1553	0.25615	-0.70191
ENSG000C	PACS1	11:660703	27.7528	51.6964	7.77232	7.15286	16.6179	16.7536	26.1886	8.87135	8.90225	24.506	0.42595	-0.35318
ENSG000C	LAMTOR5	1:1103471	147.683	122.858	236.892	245.124	132.931	223.998	242.31	274.316	171.064	51.559	0.7898	0.10203
ENSG000C	SNCA	4:8955135	0	0.66048	3.43933	17.1504	0.219702	3.71047	0	0	0	0.884361	0.1404	-2.58578
ENSG000C	FICD	12:108515	3.55308	1.4816	6.64782	3.17048	4.86449	0.072929	2.50814	8.03649	3.90513	12.2573	0.75655	0.309384
ENSG000C	ARL6IP1	16:187812	192.79	179.349	119.425	96.7642	120.461	179.851	217.881	112.06	113.904	133.25	0.8664	0.075156
ENSG000C	RP11-121I	1:2002534	0.099517	0	0	0	0	0.9444	1.03136	1.77893	0	0.086958	0.2414	5.62848
ENSG000C	SCOC-AS1	4:1402572	1.65955	0.87461	1.48949	2.21667	1.03389	0.60808	0.269031	0.151654	0.768139	0.325426	0.2012	-1.57022
ENSG000C	PTPN13	4:8659431	0.764611	0.33787	0.085307	0.581199	0.148758	1.88701	0.469709	0.910569	3.56861	2.98195	0.02045	2.50927
ENSG000C	FTH1P16	11:776599	0.42017	0.354792	0	0.20503	0.359926	1.40691	2.07536	0	0.231093	0.105644	0.38715	1.44552
ENSG000C	QDPR	4:1746026	19.5281	19.7367	31.0922	37.6015	28.4637	21.6595	17.6375	23.5319	33.6464	12.8573	0.36475	-0.36
ENSG000C	RP4-798A	1:1652069	4.55684	0.65027	1.96655	2.02104	0.805919	1.80204	0	0.112214	1.06875	0	0.3542	-1.42075
ENSG000C	TBC1D10C	11:673178	350.867	476.745	302.906	299.911	285.965	314.025	422.866	334.893	246.092	322.449	0.86625	-0.09921
ENSG000C	BPGM	7:1346468	19.3601	29.351	20.239	29.0812	15.1759	23.9802	9.15962	23.7609	18.4582	4.78805	0.23135	-0.48764
ENSG000C	TRA2A	7:2350477	34.6853	36.56	50.531	55.3001	33.0689	16.6955	33.8331	50.9508	58.8955	49.7278	0.96815	-0.01514
ENSG000C	RASGEF1B	4:8142639	0.931636	2.82312	1.54116	3.52807	3.81567	3.16014	6.23402	10.3054	7.92909	9.75021	0.0184	1.43197
ENSG000C	MCTS2P	20:315144	3.12195	4.84974	1.23107	2.23269	0.971855	2.01725	0.748778	0.678476	0	0.431851	0.2795	-1.61838
ENSG000C	CDADC1	13:492479	7.2198	4.44538	7.16477	9.64945	6.12307	8.80208	8.74097	9.80398	2.31417	3.03591	0.71165	-0.30673
ENSG000C	RP11-175I	3:1934020	0.86662	0.878663	0.987411	0.455747	1.0515	0	0	1.32103	5.09469	0.3633	0.5142	0.881491
ENSG000C	RN7SKP9C	11:167773	0	0	0	0	0	0	0	0	3.24135	0	0.1425	inf
ENSG000C	STAM2	2:1521168	5.12813	4.84238	2.38773	1.70287	1.62937	9.18178	5.84108	2.99699	3.76071	3.67363	0.2681	0.586658
ENSG000C	MRPL55	1:2281066	80.7443	84.1199	147.591	173.365	108.398	90.3479	81.2635	82.1238	88.0879	62.1954	0.09635	-0.63346
ENSG000C	ZDHHC4	7:6577433	28.8737	28.9561	25.2836	23.7253	26.369	21.3608	23.0214	17.3839	36.9137	17.9039	0.5991	-0.20909
ENSG000C	MFNG	22:374690	188.026	203.691	225.077	232.783	174.931	98.9726	143.6	140.278	136.224	152.165	0.0392	-0.62831
ENSG000C	RPS19BP1	22:395290	142.705	89.6761	152.909	137.614	195.979	218.924	202.363	139.713	97.3163	50.3152	0.67215	-0.14871
ENSG000C	PSMA4	15:785404	278.471	216.702	301.661	278.103	229.634	336.172	363.981	309.085	302.246	97.596	0.7458	0.107863
ENSG000C	SEMA6C	1:1511316	0.894029	0.888121	0.133936	0.012427	0.343861	1.12892	0.424728	0	0	0.172086	0.79515	-0.24373
ENSG000C	AC093818	2:1724273	9.53525	12.4858	8.07577	11.5797	12.1523	8.89762	8.08973	6.19671	8.24905	5.15129	0.6116	-0.49425
ENSG000C	RP5-966M	3:5281297	0.486902	1.07441	0.135866	0.249023	0.135608	1.64723	2.89168	0.401518	0.107104	0.751367	0.41235	1.31012
ENSG000C	RP11-139I	15:552029	7.20261	3.59226	4.26169	5.54994	2.89301	10.4541	12.1457	7.64809	11.1291	6.74789	0.3278	1.0863
ENSG000C	RECQL4	8:1445112	4.91951	3.35646	0.401354	1.06141	1.39669	6.32844	9.01488	1.11692	0	1.27833	0.357	0.826686
ENSG000C	DAG1	3:4946870	2.48053	1.91313	1.47165	2.61604	1.36546	0.728151	0.397415	1.56722	0.358769	1.7591	0.00465	-1.39056
ENSG000C	CTC-241N	5:1797975	0.562857	0.398667	1.32773	0.731279	0.756518	0	0.137574	0.940768	0.03954	0	0.33305	-1.74422
ENSG000C	MYH9	22:362812	209.49	227.256	25.6116	13.2379	57.8765	114.232	94.4645	45.3122	20.2067	113.747	0.23555	-0.48032
ENSG000C	MTPAP	10:303098	6.62438	4.59057	9.3506	20.9993	3.04641	9.17428	4.42306	9.87267	10.9405	4.44176	0.7081	-0.33788
ENSG000C	RFX2	19:597840	1.05144	1.6028	1.04557	1.08138	1.77748	1.74674	1.76581	1.63671	1.2643	3.04666	0.05995	1.05375
ENSG000C	CTC-246B	19:393283	5.14703	3.37098	1.58685	0	0	4.80054	1.37132	3.24758	0	0	0.9164	0.141266
ENSG000C	RP11-305I	7:1292250	4.18542	2.72524	1.5726	3.37401	0.833201	2.21027	2.77695	1.69815	0.915649	0	0.92805	0.157555
ENSG000C	ICMT	1:6181268	10.0198	9.55496	11.3743	6.02875	7.70236	4.02135	9.74465	13.7484	5.70902	13.7094	0.8528	-0.14875
ENSG000C	SNORD3B	17:190619	16.3608	3.1442	4.99686	1.70182	1.32214	0.315749	0	1.7424	3.92583	0.394955	0.41075	-1.89888
ENSG000C	RP11-46H	12:132768	4.11915	0.417471	1.94984	2.39801	0.865816	2.44903	0	1.53577	0	0	0.44895	-1.37203
ENSG000C	RP5-832C	1:1574974	1.28785	0	4.86746	1.19704	0.906736	0	0	0	0	0.406549	0.0058	-4.49718
ENSG000C	NINJ2	12:564295	15.9316	27.3914	16.6636	9.85442	15.1346	39.9418	29.8527	14.8652	28.1983	14.4039	0.4889	0.56226
ENSG000C	TMEM256	17:738053	42.2299	15.9591	40.6048	38.0484	12.9362	63.6463	32.1187	33.4607	51.3333	4.93506	0.7921	0.294567
ENSG000C	PHKB	16:471443	24.8436	22.5512	8.68958	14.787	9.57233	25.0891	11.7323	7.98792	18.9528	14.8693	0.72825	-0.14686
ENSG000C	LGR6	1:2021939	4.63429	1.39583	6.66955	5.02123	7.1133	0	0.473491	2.70463	1.1521	2.84767	0.10275	-1.91417
ENSG000C	XPO7	8:2191967	7.70915	5.48383	6.73193	5.78712	10.9698	6.07435	5.77127	6.72462	3.82273	15.3369	0.67585	-0.16517
ENSG000C	IKZF1	7:5030412	70.9593	110.581	34.626	36.496	55.778	46.9083	57.9068	42.3205	36.2153	95.6349	0.66795	-0.20656
ENSG000C	AGPS	2:1772275	8.15389	7.12069	2.44185	3.4681	2.8991	6.07715	7.5192	3.56394	4.4012	4.16794	0.74865	-0.24982
ENSG000C	ZNF155	19:439678	3.26411	6.94578	2.0772	1.23485	2.11686	0.196324	5.08038	6.80647	0	3.14267	0.88625	-0.11227
ENSG000C	SLC16A4	1:1103471	0.814136	0.621461	0.299942	0.469854	0.227316	0	0.301728	0.373582	0	1.03777	0.6384	-0.59105
ENSG000C	SRXN1	20:646614	16.1113	4.97621	8.44069	5.98888	10.1313	13.7447	10.2545	9.43846	11.4304	12.7731	0.4587	0.303733
ENSG000C	CTD-2561	17:809915	1.24936	2.03154	0.135098	3.08127	1.56414	5.59486	2.44878	0	0.339061	0.018965	0.72825	-0.50453
ENSG000C	SSBP2	5:8141302	4.9981	26.1479	8.85183	16.4849	2.4756	3.65741	0.077303	2.05958	14.5402	3.45013	0.06235	-1.39132
ENSG000C	RP11-158I	11:118791	1.18611	0.782441	1.182	0.931333	0.429055	0.062993	0.563423	0.06127	0.195527	0.573268	0.4825	-1.60157
ENSG000C	ZBTB38	3:1412287	20.1999	25.3901	19.8389	16.5596	45.115	23.8589	23.4642	9.59424	18.9394	49.789	0.5634	-0.28333
ENSG000C	NTAN1	16:149745	43.087	40.1886	32.9628	25.561	31.4288	34.5875	62.041	35.812	33.3797	10.3525	0.94025	0.056739
ENSG000C	NPAT	11:108157	18.0096	27.9111	10.4362	11.0381	14.1844	11.1984	13.4811	6.8974	9.33249	25.283	0.60185	-0.28596

ENSG0000	CASP3	4:1846276	46.5487	32.3444	23.3996	23.9256	18.0676	72.3221	60.2757	40.9287	37.7277	27.9986	0.0553	0.639488
ENSG0000	COPB1	11:144434	55.6879	55.9013	30.6916	34.8917	29.206	26.269	47.1641	42.3708	39.348	53.6743	0.97445	-0.02155
ENSG0000	CTB-89H1	5:1494921	1.92376	2.88151	5.14272	0.939781	4.41793	5.66218	8.46993	9.50853	1.69291	13.3154	0.3702	1.18278
ENSG0000	TVP23C-C	17:154360	0.591636	1.75351	0.340359	0.174297	0.630676	0.665041	1.70639	1.0763	1.36655	0.23036	0.86365	0.209061
ENSG0000	CASP2	7:1432882	7.32106	9.44619	15.7875	6.64108	8.4939	4.80156	7.67673	13.3353	5.60085	24.1614	0.8825	0.058759
ENSG0000	ITGA3	17:500559	1.74963	0.902302	0.412244	0.167378	0.291265	1.6001	1.7851	0.877554	0	2.12166	0.36075	0.76102
ENSG0000	ACYP1	14:750532	36.4422	30.8596	32.9983	27.4958	26.2885	69.7699	47.5419	36.0308	9.07437	14.873	0.88365	0.158273
ENSG0000	NRAS	1:1147044	19.6591	16.9064	4.98053	4.88397	4.91768	15.7567	15.4496	8.12001	11.8061	9.99249	0.32065	0.329223
ENSG0000	MCTP1	5:9415296	1.83398	4.5024	0.639227	0.253114	0.470571	13.8918	1.09957	2.10556	4.22264	2.00477	0.0687	1.68775
ENSG0000	KCTD9	8:2542784	2.80915	1.57423	1.993	1.13132	1.27843	3.27711	4.85421	6.60129	3.03638	8.03407	0.0005	1.58299
ENSG0000	GZMA	5:5510264	1277.46	170.282	2396.11	1610.86	1779.44	1481.21	2061.96	1801.57	1133.01	490.816	0.8071	-0.1342
ENSG0000	CHCHD4	3:1411207	14.5466	16.2172	15.394	13.6464	10.8244	10.0356	27.0858	11.1788	14.773	4.7084	0.87495	-0.06793
ENSG0000	NPTX2	7:9861729	0.023512	3.95799	0	0	0.159487	0	0	0	0	0	0.0184	#NAME?
ENSG0000	NADSYN1	11:714531	55.367	53.4853	38.2327	37.777	25.9675	46.621	47.5556	37.5735	47.8467	37.5495	0.6672	0.126041
ENSG0000	ITGB1	10:329003	112.902	196.02	28.0952	50.0994	20.3024	90.526	140.976	87.6671	78.5912	40.0489	0.7888	0.093143
ENSG0000	MYO1G	7:4496266	73.9412	86.9226	34.6644	39.5719	54.7578	46.1061	54.5018	31.4735	21.2509	52.6709	0.0674	-0.61313
ENSG0000	PSEN2	1:2268701	4.4724	1.22206	2.53488	7.58708	2.66055	2.27626	7.67421	0.994613	6.4432	5.06039	0.73345	0.1874
ENSG0000	LRRC37A1	17:469231	0.885672	1.15689	0.43112	0.230544	0.281276	0.911884	1.52635	0.089878	0.086121	0.681841	0.88555	0.193808
ENSG0000	PIGP	21:370591	28.0473	15.0481	29.0729	30.4039	28.1399	43.4279	14.2121	23.7672	35.6981	15.1076	0.91335	-0.06489
ENSG0000	CCNT2	2:1347354	15.7195	12.4183	4.85593	6.08043	9.35837	9.02345	14.6519	5.51969	6.02975	21.1651	0.8978	0.062763
ENSG0000	SREBF2	22:418312	13.1388	16.3977	2.53062	2.76821	3.70228	8.03882	9.74588	7.90641	2.639	7.4665	0.80295	-0.11725
ENSG0000	SCNM1	1:1511566	63.7644	61.029	105.418	99.026	88.7401	78.2918	67.3676	85.1823	76.6688	51.8287	0.66865	-0.26651
ENSG0000	C16orf87	16:467966	21.0528	18.7684	10.2021	16.7553	7.20194	23.8328	22.8241	9.5528	18.8624	5.51482	0.76625	0.148001
ENSG0000	ANKRD10	13:110878	54.1328	72.0586	55.7477	67.2462	33.2947	44.5707	70.2809	61.9854	79.6282	62.3407	0.6086	0.173801
ENSG0000	ZNF304	19:573508	2.42396	0.749812	1.16981	0.394682	0.833057	2.04676	2.36152	1.07123	0	2.6422	0.5642	0.433985
ENSG0000	ACY1	3:5195531	10.2032	8.36771	12.1153	16.1362	7.65245	9.85659	12.4585	10.663	9.10562	6.27103	0.84205	-0.18175
ENSG0000	MBIP	14:362985	23.0296	23.4428	12.2277	12.7412	10.2858	18.6963	23.0339	14.666	0.950462	6.7916	0.5418	-0.29593
ENSG0000	PIGCP1	11:330394	0.524387	0.474791	0.463328	0.401038	0.264859	0	0.505116	0.045176	0	0.461205	0.46835	-1.08313
ENSG0000	NDFIP1	5:1421085	22.8585	36.3963	14.5521	15.9855	14.6417	27.432	24.1336	16.3222	20.1522	12.4717	0.9655	0.018988
ENSG0000	RP11-332	2:1053340	1.54966	1.47572	1.34554	1.25559	0.82081	2.33397	1.66046	1.23476	3.49751	3.07401	0.4675	0.793792
ENSG0000	EWSR1	22:292598	207.293	198.199	243.824	217.793	161.544	217.933	181.514	222.113	172.532	226.838	0.98905	0.00535
ENSG0000	SMARCA5	4:1435134	28.768	21.3699	8.9191	12.0437	9.09962	14.0229	21.1345	12.3324	9.66215	13.3588	0.8614	-0.05547
ENSG0000	PAPD5	16:501529	3.39895	3.48617	8.14109	3.59217	9.37106	3.26296	5.66421	6.46495	1.34007	11.7985	0.601	-0.22547
ENSG0000	TBL2	7:7356893	17.003	14.5172	20.3816	10.7091	16.2458	24.5468	17.7942	14.7812	29.772	17.148	0.38565	0.368675
ENSG0000	HNRNPUL	11:626902	1.92133	3.08405	1.37979	1.22848	3.88676	4.25802	3.82861	1.5072	1.67483	4.66727	0.61795	0.469655
ENSG0000	GNG7	19:251121	0.216396	3.48663	0.319731	1.96608	0.90138	0.408226	0	0.186089	1.90854	0.272157	0.3137	-1.22161
ENSG0000	TMEM199	17:283575	18.5847	21.3298	18.4794	22.2778	11.6382	16.3995	18.2081	19.6949	10.85	12.1008	0.3444	-0.43987
ENSG0000	HOOK2	19:127630	2.53863	4.60817	10.0989	8.91671	7.9653	8.79132	3.60189	7.38297	0	11.8522	0.8212	-0.17362
ENSG0000	RP11-326	4:1844748	2.39228	3.09218	1.37612	1.64205	1.03766	6.58108	1.67122	1.80149	0.394304	3.03687	0.582	0.469931
ENSG0000	8-Mar	10:454545	4.10339	5.02827	2.69931	3.06857	5.53471	3.09076	3.36088	3.59623	1.20231	8.16301	0.57275	-0.25552
ENSG0000	SDCBP	8:5855292	93.1463	105.787	68.1503	93.7402	55.9844	263.663	95.3052	106.183	160.117	69.2039	0.0523	0.678125
ENSG0000	ORM1	9:1143230	0.126449	0	0	0.465678	0	6.0808	0	0	1.01567	0	0.3284	3.70936
ENSG0000	SAPCD2	9:1370576	1.05029	1.05357	0	0.596148	0.304539	0.887087	1.77536	0	0	2.45349	0.72095	0.853416
ENSG0000	RP11-421	6:1074332	2.10556	1.44943	0.836817	0.443511	0.580626	0.437987	2.07103	0.301833	0	0.848932	0.8051	-0.47194
ENSG0000	AC098820	2:2163852	0.487297	0.120128	0.532792	0.707867	0.064285	0.330084	0.740706	0	0	0.775436	0.9887	-0.02159
ENSG0000	MFSD14A	1:1000380	39.1343	40.8422	22.0792	45.4736	14.6505	38.6396	56.5726	32.1436	31.3537	23.6186	0.44165	0.255878
ENSG0000	CCR2	3:4635373	83.6251	8.64311	41.7676	54.7202	38.9516	46.8046	16.5318	20.2058	31.2865	25.2436	0.034	-0.75809
ENSG0000	LSM7	19:225225	83.0363	46.7574	140.261	159.404	68.9646	119.754	129.258	130.835	94.3626	24.3306	0.91165	-0.10296
ENSG0000	AC024592	19:586582	2.32939	1.39309	7.26005	4.80264	3.72494	3.93343	1.54142	3.49005	4.13999	0.648684	0.665	-0.5716
ENSG0000	COG2	1:2306424	21.1164	22.5239	14.9412	17.7333	14.5678	15.9477	16.8744	14.5468	6.79356	16.4058	0.35275	-0.34004
ENSG0000	RPF1	1:8447925	51.3372	49.4896	57.884	59.0965	48.9484	82.4034	76.5445	50.2787	28.177	16.7172	0.78565	-0.09068
ENSG0000	ATXN2L	16:288224	13.0731	13.4677	3.77226	3.96248	6.84839	10.8543	12.8159	8.36657	4.1383	16.7443	0.2559	0.652899
ENSG0000	ZSWIM6	5:6133227	1.05903	1.48271	0.581565	0.557884	0.485418	1.68271	0.248431	1.04994	0.258639	1.43976	0.7491	0.243339
ENSG0000	FOXO4	X:7109619	0.708405	1.46379	0.227733	0.635122	0.506106	0.12558	0.651638	0.136686	0	0.508526	0.31595	-1.27361
ENSG0000	ELAC2	17:129923	47.5112	39.0909	37.9712	34.6688	33.0341	29.1651	27.0104	21.6793	20.5164	36.506	0.128	-0.4842
ENSG0000	TCEAL3	X:1036074	2.69506	2.47569	3.41249	4.26614	12.4756	3.72966	2.20115	4.5722	0.484877	2.32445	0.42425	-1.04165
ENSG0000	OSM	22:302628	4.57203	0.019432	7.05146	58.0716	13.6982	25.0989	16.0328	20.1768	37.578	7.49074	0.62805	0.245504
ENSG0000	CLIC1	6:3172703	473.092	224.474	593.232	581.923	496.146	622.045	632.694	721.247	633.009	198.173	0.658	0.191143
ENSG0000	DNPH1	6:4322562	56.9898	54.697	65.5551	68.6816	49.6327	214.388	152.873	139.938	139.496	33.4156	0.0039	1.0961
ENSG0000	ARHGDIB	12:149420	2365.66	2634.84	2994.12	2483	2714.06	2447.97	2141.27	2258.05	2737.84	1052.06	0.5649	-0.30747
ENSG0000	CXorf21	X:3055882	3.23112	5.867	2.09677	1.40196	1.00395	14.3484	3.58315	6.14132	3.66429	1.73601	0.1353	1.14278
ENSG0000	TRDC	14:222025	1432.84	50.6728	41.5294	59.4966	29.9676	534.693	611.996	57.8869	0	14.1864	0.927	-0.20858
ENSG0000	RP11-399	10:890158	0.917705	1.45993	0.76168	1.14206	0.133693	0	0.967787	0.487693	0	0.481793	0.6236	-1.10347
ENSG0000	C1S	12:697089	0.195475	0.347474	0	0	0.04883	1.97982	0.644955	0.005596	1.23489	0.388407	0.0047	3.04135
ENSG0000	SLC35A5	3:1125325	11.9236	8.90975	8.46382	4.5723	3.84094	19.3495	19.7533	7.06385	7.47491	5.4246	0.605	0.451984
ENSG0000	BTBD9	6:3816845	5.90481	9.5982	2.82198	9.59709	8.70373	3.45986	14.3906	6.49797	12.9424	22.5419	0.46395	0.469609
ENSG0000	NRDC	1:5156186	86.4944	90.1626	36.5291	32.8706	33.8322	74.8424	106.428	37.7326	30.9481	85.6574	0.62275	0.294005
ENSG0000	TMEM14B	6:1074775	135.304	113.919	177.277	169.824	67.2146	137.021	130.504	158.293	156.983	29.6469	0.79035	-0.08786
ENSG0000	RP4-583P	20:636578	0.194876	0.253691	0.783021	0.405701	0.160397	0	1.96424	0.161046	0	0.406727	0.79985	0.447923
ENSG0000	RP11-196	1:1498444	1.38448	1.51899	0.545631	0.232785	0.856044	0	5.41001	1.2717	0.760816	2.94638	0.446	1.26818
ENSG0000	CARD16	11:105041	261.637	148.494	305.439	274.957	232.932	386.914	371.187	242.434	162.236	59.3208	0.96285	-0.01647
ENSG0000	AC007318	2:6520510	2.81913	2.75787	2.20411	3.77098	0.80054	3.60304	3.58493	1.33673	2.46462	0.373221	0.58705	0.925657
ENSG0000	CFAP61	20:200343	0.845048	0	0.314858	0.796889	0.232281	0	0	0	0	0.291114	0.00055	-2.98699
ENSG0000	GOLGA2P	12:100156	2.47695	2.73683	0.78583	0.656208	1.07713	2.82532	2.92028	2.60184	0.706956	4.32776	0.3334	0.763331
ENSG0000	NEK6	9:1242576	2.20567	3.40524	2.39274	1.55165	3.52399	12.0028	3.53305	2.74926	1.08393	1.86503	0.4387	0.464939
ENSG0000	SUFU	10:102503	3.44876	2.52278	0.914529	1.02582	2.03504	3.04181	1.15045	0.43702	0.10036	6.27172	0.95895	-0.03154
ENSG0000	SEPT7-AS	7:3575185	0	0.220618	0.084462	0.422335	0.137267	0.338281	0.930897	0.752241	0	0.127983	0.3791	1.43663
ENSG0000	ZBTB33	X:1202507	6.55314	4.73125	1.71915	1.38866	2.04736	2.92636	2.91254	1.5661	2.85553	4.14688	0.68435	-0.20992
ENSG0000	PYCRL	8:1436039	7.02088	4.04912	10.2718	11.9754	6.95427	6.74867	7.74284	8.41313	8.16509	7.8163	0.782	-0.13196
ENSG0000	EXT1	8:1177944	2.29223	3.81403	1.9045	1.62722	1.07253	2.11054	1.5222	3.50976	0.072652	1.71179	0.33095	-0.74836
ENSG0000	WDR27	6:1694572	9.42752	14.6337	6.88835	8.44508	5.17597	6.04005	6.93357	4.73183	6.28728	16.2761	0.8244	-0.1728
ENSG0000	PFAS	17:822482	7.19211	7.25734	2.91299	3.18157	2.10348	1.80768	7.40317	3.12961	0.361172	3.95325	0.5601	-0.48304
ENSG0000	DUSP23	1:1597809	41.3678	21.9081	67.792	45.7181	46.3575	72.8981	99.0425	81.8464	41.6194	17.3485	0.526	0.272463
ENSG0000	LPIN1	2:1167759	30.4679	35.8917	14.2646	18.422	15.4044	34.2245	29.6583	24.1688	25.4233	38.9235	0.2965	0.473338
ENSG0000	ZFP82	19:363831	4.92947	7.01676	3.60328	4.92697	2.85291	5.11857	4.12371	4.11416	1.36137	5.21471	0.5939	-0.27487
ENSG0000	QSOX1	1:1801548	5.26488	4.82683	10.9929	8.44306	15.8066	15.4226	9.59824	28.0884	17.4578	30.6861	0.02415	1.14069
ENSG0000	MTMR3	22:298831	8.83744	9.54185	3.23882	3.68753	4.25512	4.15974	4.03554	6.47518	3.33022	7.8447	0.5682	-0.33051
ENSG0000	MFAP1	15:438044	19.6915	32.1745	23.5187	16.0758	40.8263	29.8484	24.3415	20.0641	13.881	37.082	0.82015	-0.07951
ENSG0000	RAB28	4:1336135	26.1566	14.7247	9.61951	14.8572	10.0195	25.9249	22.2455	11.0084	11.8427	6.93546	0.76605	0.128245
ENSG0000	LEO1	15:518296	27.0977	31.0417	32.8354	21.0431	37.4697	55.4589	31.5059	29.9509	13.4464	40.8736	0.78765	0.150887
ENSG0000	ARHGAP1	10:969980	4.13475	4.86258	2.98373	1.67812	1.9444	4.05718	2.52214	4.37373	3.20329	5.09683	0.4225	0.444248

ENSG0000(	NUTF2	16:678067	57.6599	52.2368	76.7725	100.643	62.5988	75.9177	86.3368	86.8604	59.1213	28.6992	0.8899	-0.11815
ENSG0000(	SEMA3F-/	3:4994000	0.987272	1.61013	0.176262	1.59194	0.314159	1.80321	1.15177	0.752032	0.611074	0.285607	0.9194	-0.12105
ENSG0000(	ZFP36	19:394068	338.559	352.286	215.372	813.023	217.446	133.492	193.243	337.333	512.656	406.003	0.47815	-0.30548
ENSG0000(	PNRC1	6:8908075	318.123	342.842	201.905	335.886	283.791	129.672	212.829	193.409	258.093	178.32	0.05865	-0.65763
ENSG0000(	PDS5A	4:3982286	20.4759	16.7044	3.59674	5.6918	6.72422	10.6977	12.604	9.13486	7.41453	11.3059	0.9752	-0.01247
ENSG0000(	SLC30A5	5:6909364	36.9558	25.7024	20.9705	28.3101	14.4982	22.7226	23.6205	18.1205	19.2372	18.6431	0.51825	-0.34398
ENSG0000(	ATF6B	6:3203826	26.522	30.4164	36.7264	52.9426	34.969	26.9947	22.3948	31.5974	40.5811	49.7516	0.73655	-0.14013
ENSG0000(	BIN3	8:2260463	40.6344	56.7594	62.0244	48.7368	43.8945	57.3807	48.4538	34.4542	12.288	41.4162	0.5266	-0.4454
ENSG0000(	RNPEP	1:2019823	24.8504	31.7939	31.7085	20.4888	35.1919	23.9639	21.402	25.2269	25.2445	39.2409	0.63555	-0.1622
ENSG0000(	GAS7	17:991060	2.42269	2.14969	1.2072	0.381089	1.38156	6.0366	3.24231	2.35016	0.046601	1.67203	0.3853	0.479345
ENSG0000(	HBM	16:152686	0	0	0	6.94089	0	0	0	0	0	0	0.12005	#NAME?
ENSG0000(	PQBP1	X:4889344	70.3821	64.3562	95.6333	79.1288	113.159	118.404	106.695	89.7251	68.8048	62.7368	0.9954	0.003573
ENSG0000(	HDX	X:8431787	1.41858	2.40664	0	0.273154	0	2.36422	1.10687	0	0	0.617066	0.9923	-0.01344
ENSG0000(	CDKN3	14:543968	10.5764	15.1058	1.96084	4.17829	1.42303	19.4215	10.1148	10.8676	6.68818	1.86083	0.3253	0.809604
ENSG0000(	CTU2	16:887064	10.2977	14.6372	12.3621	9.15736	8.963	6.01022	13.2517	11.5574	6.39864	8.00067	0.7489	-0.32533
ENSG0000(	RP11-98I9	6:9939805	1.89656	2.82594	3.97237	2.29687	1.3762	0.437834	3.18571	2.61139	5.33763	1.23356	0.9353	0.11428
ENSG0000(	TMEM185	X:1495965	16.2695	19.8333	16.4811	18.8597	13.5517	9.17718	23.5835	12.6948	12.2118	17.8289	0.97705	0.015042
ENSG0000(	FAM207A	21:449400	11.5675	4.03075	4.07891	5.5233	4.5486	8.40437	9.35884	5.69902	5.48102	1.62889	0.98085	-0.02454
ENSG0000(	PTBP3	9:1122177	9.93329	8.45316	3.48118	2.73738	6.28866	7.88977	10.1162	6.11571	5.07192	13.0485	0.47455	0.262079
ENSG0000(	CEP78	9:7823606	14.4585	28.5408	8.2775	7.84084	6.82542	8.7433	9.11737	9.3433	11.6716	7.84356	0.2846	-0.61099
ENSG0000(	RP11-290	13:453334	1.83189	0.321248	0.758163	2.3105	0.721224	1.39001	3.59217	1.93375	0.575379	0.647142	0.9147	0.191054
ENSG0000(	C19orf25	19:144630	35.3262	33.3106	46.741	61.124	38.201	51.3451	53.1094	35.244	38.0861	33.0434	0.8646	-0.07275
ENSG0000(	SLC41A1	1:2057890	7.33983	6.22106	2.74412	2.74669	3.56513	3.00483	4.13141	2.78593	4.16606	5.63338	0.98285	0.014479
ENSG0000(	LINC0064	14:212000	2.06697	0.985735	1.86976	0.672884	1.32898	0	0.887801	0.301391	0.688514	1.83693	0.3864	-1.03802
ENSG0000(	RP11-201	1:1551694	0	1.36709	0.886938	0	0	0	0.516891	0	0	0.580924	0.72645	-0.90843
ENSG0000(	ZC3H4	19:470641	1.77072	1.92359	6.86828	3.92967	5.51726	0.686762	2.27995	4.34525	2.33119	10.5644	0.9823	-0.02481
ENSG0000(	DARS-AS1	2:1359066	4.44072	5.39161	3.2293	1.81449	1.4812	2.24035	0.785542	3.7178	7.28425	0.922885	0.93765	0.06558
ENSG0000(	FKBP4	12:194605	19.4914	16.7625	7.51027	7.26178	10.9065	23.4113	21.0043	10.8777	14.8629	11.6048	0.5156	0.426687
ENSG0000(	DHX34	19:473492	9.21342	12.0636	2.18628	2.58547	3.03097	7.04073	6.16851	2.85187	1.52978	5.08747	0.5458	-0.34795
ENSG0000(	KRT8P12	3:1604949	2.72018	0.96223	1.26884	1.44168	0.890986	8.76414	4.88188	4.90923	5.92426	2.73955	0.1001	1.91632
ENSG0000(	AC112721	2:2374214	0	0	0	0	0	0	0.879638	1.35707	0	0	0.0037	inf
ENSG0000(	SMIM18	8:3057831	0.124893	0.173968	0	0.204238	0	0	0.04163	0	2.61649	0	0.385	2.59178
ENSG0000(	HMMR	5:1634602	5.32429	8.41111	0	0.524177	0.279012	3.63376	8.16715	0.643559	4.38313	4.02179	0.4067	1.02548
ENSG0000(	RNLS	10:882738	2.76861	5.16315	3.15907	0.4948	2.21161	4.10895	3.13367	0.762183	1.62481	4.66076	0.9455	-0.05478
ENSG0000(	PLEKHO2	15:648418	17.1541	15.8054	3.65783	4.33394	6.27918	41.5754	25.6502	15.7595	10.5122	25.0961	0.0022	1.29783
ENSG0000(	CDK10	16:896807	105.127	127.998	59.6818	87.4636	43.5014	139.992	117.955	47.0555	49.9279	64.9409	0.7854	-0.09294
ENSG0000(	MGAT2	14:496207	18.638	12.2089	22.9209	33.2599	26.3066	19.5042	16.7634	25.6826	23.7854	25.8509	0.70535	-0.15138
ENSG0000(	MID1IP1	X:3880143	11.408	9.60061	1.71098	1.3174	2.14604	6.109	6.03534	1.98614	2.78959	0.966146	0.4991	-0.44816
ENSG0000(	OXA1L	14:227021	30.5488	45.6198	51.0966	45.0208	45.9943	46.3117	46.6061	55.0119	72.2683	44.8582	0.5818	0.188627
ENSG0000(	KRT10	17:408181	90.635	80.7237	140.135	106.068	58.8561	92.822	106.675	110.076	80.6591	23.5885	0.6962	-0.19234
ENSG0000(	UBR2	6:4256406	8.77364	8.98403	4.60315	3.63714	4.79412	6.31005	7.53169	5.7503	4.32363	9.20522	0.96315	0.016825
ENSG0000(	IGLV3-1	22:228807	0	1.67246	1.43473	0.654501	0.514008	0	0	0	0	15.5215	0.1757	2.59338
ENSG0000(	EDEM3	1:1846902	9.90506	8.31789	5.14978	2.97256	2.75925	6.21117	8.21215	4.77761	3.27045	7.00132	0.53895	0.23652
ENSG0000(	NUDCD1	8:1092409	17.9469	11.4737	12.3464	13.7553	10.4038	4.74658	7.74736	7.34042	13.944	5.86195	0.4373	-0.62296
ENSG0000(	RP11-23J9	9:9715656	1.55491	1.23731	1.13449	0.498497	0.345641	1.18975	1.27002	0.869932	0	0.869199	0.8586	-0.23868
ENSG0000(	LDLR	19:110893	6.22436	8.39677	2.91527	4.93636	2.15635	7.68188	20.7264	23.1949	9.60308	7.80785	0.00195	1.39388
ENSG0000(	FAM156B	X:5289130	7.22158	16.8466	7.52044	10.6595	5.71723	13.6013	8.68523	6.53725	3.58495	11.3872	0.67875	0.400138
ENSG0000(	IL6R	1:1544023	5.42011	13.9172	0.436383	2.1838	0.415337	14.9805	4.47873	1.75009	2.89995	4.97944	0.70255	0.220005
ENSG0000(	HLCS	21:367508	4.83761	1.04042	2.70612	4.71109	1.62869	6.99534	2.54184	6.18775	6.01544	2.81929	0.2328	0.741777
ENSG0000(	NDRG1	8:1332371	31.7876	25.1978	16.7037	15.0961	30.5307	20.4902	23.1777	15.8906	16.9517	43.057	0.9967	-0.00128
ENSG0000(	NME1	17:511535	50.2875	33.2294	65.6108	61.3548	48.4021	68.8509	94.2861	54.0304	27.1964	16.1785	0.9032	-0.06685
ENSG0000(	EID3	12:104215	7.99065	8.00311	14.6594	16.3836	10.3312	14.0188	14.1552	14.4851	11.8252	7.40458	0.94035	0.075692
ENSG0000(	EP400NL	12:132084	3.8525	5.24488	5.5936	9.87246	2.64283	4.14056	2.45972	2.53703	2.77984	4.78173	0.0763	-1.04386
ENSG0000(	ULBP3	6:1500631	0	0.725716	1.50993	0	0.678242	0	4.8994	0.904483	0	1.65611	0.4609	1.28019
ENSG0000(	DHX40	17:595655	11.3141	13.2145	7.00421	8.83951	3.99162	9.31597	5.84022	3.99256	11.3928	3.2807	0.31345	-0.40225
ENSG0000(	TRIM38	6:2596280	8.52126	10.069	6.92842	5.64665	7.04247	8.14597	11.72	7.36853	7.71919	14.2226	0.4063	0.282309
ENSG0000(	HSPB1	7:7630254	35.0291	91.8051	53.4049	27.2982	81.6411	521.758	147.688	89.0069	226.464	64.733	0.00005	1.91547
ENSG0000(	FBXO44	1:1164836	17.9819	12.2145	9.14641	10.2472	6.64102	17.2561	23.0356	10.4979	5.63107	10.4127	0.9478	0.035154
ENSG0000(	RP11-68I3	17:295605	0.607462	0.61086	0.134844	0.173314	0.075441	0.074464	1.38143	0	1.55979	0.916304	0.2836	1.63909
ENSG0000(	LL22NC03	22:474612	22.1595	0	15.3389	35.0051	22.7598	3.42297	0	9.14687	2.11816	17.3834	0.25	-1.54838
ENSG0000(	TMEM234	1:3220909	11.6158	6.59326	13.2403	14.7446	8.14085	16.5905	15.8512	15.4123	30.8499	9.00189	0.2043	0.731374
ENSG0000(	ERO1B	1:2361425	3.62725	2.47916	2.56108	3.74288	3.16495	5.4659	2.10378	3.83199	6.85788	4.45861	0.90955	0.083951
ENSG0000(	CTIF	18:485390	0.930054	0.322915	0.192111	0.92427	0.626007	0	0.260039	0	0.07491	1.04591	0.2319	-1.22281
ENSG0000(	FSD1	19:430459	9.88114	0.75402	12.3908	15.7747	22.175	10.0779	8.11964	33.1663	29.947	42.0388	0.03645	0.981053
ENSG0000(	NUP85	17:752056	34.4292	58.9257	36.0812	51.7756	33.4187	37.4726	36.5924	32.3956	37.6597	46.119	0.811	-0.07833
ENSG0000(	CCDC92	12:123762	30.3462	31.6526	20.4165	10.4211	33.0293	30.2043	41.2636	19.917	13.0256	41.5143	0.69205	0.175947
ENSG0000(	BANF1	11:659965	128.909	129.067	162.757	158.108	109.729	133.755	174.343	168.106	121.673	36.4603	0.79825	-0.12218
ENSG0000(	HIVEP1	6:1200766	2.86213	9.5029	1.56139	1.42272	0.783825	8.22835	7.52542	1.94024	7.73258	5.74214	0.1935	1.07091
ENSG0000(	RP11-701	15:248236	1.89855	0.607726	2.15651	1.28982	0	0	0	0	0	0.365236	0.3004	-2.72643
ENSG0000(	RP11-452.	4:1616050	0	0	0	0	0	0	0	0	2.47422	0	0.2033	inf
ENSG0000(	MAPRE2	18:349769	57.1777	50.5475	49.5603	52.4643	39.0499	42.0243	29.4482	48.8144	53.0781	50.5746	0.37825	-0.27008
ENSG0000(	MIS18A	21:322682	7.73422	9.55018	11.94	8.42549	9.88372	10.7331	3.81782	7.54709	4.31108	7.03546	0.2728	-0.57113
ENSG0000(	TTC19	17:159765	45.6537	44.8003	33.9042	44.6446	21.7372	43.4948	38.7893	23.3458	11.4652	26.7859	0.5939	-0.34105
ENSG0000(	GPR27	3:7167541	0.369465	2.05021	0.13313	0.604091	0	2.13877	1.46315	1.88126	0	0.501	0.5394	1.1731
ENSG0000(	LRRC57	15:424912	9.38553	6.04383	9.91185	13.0302	6.29492	13.7826	6.98895	9.70518	7.49767	12.9597	0.9928	0.009058
ENSG0000(	THAP6	4:7547903	10.6289	14.7623	8.32706	5.89665	6.17202	10.0821	8.69338	6.90966	6.25248	11.6945	0.75005	-0.16735
ENSG0000(	IL2RA	10:601068	23.7057	27.1212	0.445367	2.45119	0.49456	62.5704	187.304	13.2367	38.1668	5.40202	0.00005	2.70087
ENSG0000(	DDX11-A!	12:310207	0.140172	0.134926	1.14584	0.878985	1.16957	0	0.187262	0.091484	0	0.47407	0.2877	-2.02429
ENSG0000(	EIF3H	8:1166421	144.682	167.982	163.631	109.624	133.004	132.177	120.926	169.99	77.0775	59.5025	0.4632	-0.38878
ENSG0000(	CD1D	1:1581799	0.665323	8.40787	0.787235	0	0.162629	3.90841	0.017834	1.03459	0	0	0.43235	-0.95445
ENSG0000(	L3HYPDH	14:594290	50.2485	46.6643	23.9467	26.4111	7.40343	23.8755	64.9419	43.9967	14.9614	20.0298	0.71995	0.26313
ENSG0000(	PHACTR4	1:2836958	8.04636	5.80652	5.30837	2.48034	5.08226	3.456	5.90019	2.26683	3.65349	9.39065	0.61945	-0.24167
ENSG0000(	TCTN2	12:123671	0.352898	0	1.52669	1.79318	0.596909	0	0.011586	0.21569	2.26281	0.412162	0.6841	-0.5836
ENSG0000(	ECD	10:731103	25.3733	32.2768	23.1259	28.549	20.1541	27.0498	18.0675	24.9896	20.882	18.1131	0.60345	-0.34637
ENSG0000(	AL365181.	1:1565944	0.234575	1.47481	0.091344	0.051621	0.034157	0	0	0.304541	0	0	0.36455	-2.72711
ENSG0000(	RP11-655	11:584978	0.824118	0.927746	0.69528	0.412021	0	0.522554	0.081473	0	0.920283	0.846839	0.88775	-0.17404
ENSG0000(	JMJD7	15:418280	12.0253	5.18788	20.5891	36.3352	10.5526	6.3763	4.60647	8.28472	21.5121	6.23388	0.53665	-0.7461
ENSG0000(	PPIP5K1	15:435334	2.59113	3.19966	1.52282	0.801261	0.50368	4.34307	1.77282	1.04058	3.4417	1.58832	0.52875	0.392048
ENSG0000(	LSM12	17:440346	28.8905	23.1843	36.3848	30.8113	45.2347	42.7399	31.2505	33.3853	37.9705	29.4002	0.7559	0.171843
ENSG0000(	AP1G1	16:717289	12.8618	17.6962	2.59175	3.192	4.02966	6.52313	7.61196	10.7392	7.50483	11.8915	0.7644	0.150418
ENSG0000(	RPS3A	4:1510995	718.999	575.32	1223.26	350.538	274.213	547.438	478.653	787.541	298.03	49.2742	0.18245	-0.52489

ENSG0000	ARFGAP2	11:469366	51.1941	64.4375	58.5977	64.4775	52.6926	43.7125	56.2539	41.7416	59.626	64.3597	0.709	-0.11916
ENSG0000	MAGED1	X:5180300	21.9803	27.0993	25.673	20.9918	22.6581	11.3388	20.8566	12.6826	10.0385	19.8813	0.0482	-0.73273
ENSG0000	AD000684	19:352483	0.126378	0.16567	0.197015	0	0	0	0.590762	0	0	1.92172	0.3882	2.30586
ENSG0000	CLDN23	8:8701937	0	0.351353	0	0	0.026034	6.66552	0	0	0	0.030164	0.09345	4.21
ENSG0000	CHRNA5	15:785655	0.37731	0	0	0	0.701436	2.95509	2.71042	1.43766	1.03765	0.233632	0.0288	3.21587
ENSG0000	LYL1	19:130990	3.66934	14.3042	2.41	9.82822	3.90439	10.8768	5.58513	3.02117	3.00796	4.52661	0.64915	-0.37251
ENSG0000	AC009065	16:225311	39.5844	53.9263	60.7488	75.1383	50.6344	46.5392	54.8416	44.3541	44.2337	24.3142	0.82885	-0.44605
ENSG0000	NCR1	19:549061	1.42479	0.297026	3.25846	5.39902	2.70282	2.95587	4.71339	1.80176	2.01468	1.6729	0.8645	-0.13111
ENSG0000	CEP68	2:6505636	3.62772	10.1274	4.26395	2.36999	2.57334	1.47928	2.10723	3.93458	1.93513	5.54587	0.52945	-0.64583
ENSG0000	ZNF296	19:450714	4.68781	5.79775	6.92705	7.14061	12.4211	1.92384	5.88285	10.0423	7.97474	6.83115	0.9331	-0.12972
ENSG0000	CTB-32O4	19:292053	0	0	0	0	0	2.6009	0	0	0	0	0.16855	inf
ENSG0000	NEK11	3:1308505	0.908343	0.066759	0.42167	0	0.136544	0	0.808563	0.725075	2.87182	1.13464	0.09675	1.74982
ENSG0000	POGK	1:1668394	12.0552	7.30028	4.11133	2.27137	5.4459	6.26925	6.31591	6.19968	1.36002	8.77125	0.57725	-0.23096
ENSG0000	USP39	2:8559854	37.7662	33.071	54.4088	41.7018	41.1441	35.4468	36.3838	34.8315	27.7151	40.558	0.48515	-0.26905
ENSG0000	RP11-301	1:2338360	0.205123	0.258327	0.431685	0.574566	0.60089	0	0.450614	0.492372	0.616556	0	0.7683	-0.50476
ENSG0000	DCXR	17:820351	69.0139	75.6595	73.8714	67.6129	70.6378	72.0544	139.766	78.5314	30.5403	23.2284	0.6247	-0.17553
ENSG0000	RGS3	9:1134447	42.2811	21.9562	45.302	61.0607	54.7064	27.3387	22.223	25.8113	25.432	49.2569	0.2267	-0.4742
ENSG0000	AL365273.	10:957117	0	0	0	0	0.084013	0	0	0	0	0	0.27335	5.00817
ENSG0000	RAB34	17:287142	1.30569	1.81734	0.928148	0	0.169926	6.1805	1.48262	0.783557	0	0.829547	0.1913	1.14751
ENSG0000	MRPS24	7:4386655	74.9383	47.9533	112.467	124.088	92.3278	85.3646	107.74	91.3494	57.7676	21.4646	0.3618	-0.4773
ENSG0000	RP4-738P	20:251407	0	0.178776	0	0	0	0	0	0	3.81162	0	0.48255	4.88845
ENSG0000	HEIH	5:1808299	33.1746	31.8957	44.9016	24.8762	32.9575	48.2244	28.7695	29.9155	19.2745	22.5577	0.6505	-0.20486
ENSG0000	MRPL2	6:4303761	43.1072	50.201	63.8108	92.0009	56.9738	60.6644	47.4568	59.5432	49.3421	20.3236	0.4346	-0.37741
ENSG0000	SYMPK	19:458154	12.5548	7.09797	2.51053	3.06643	1.91212	7.0343	8.42097	4.18944	0.115002	6.073	0.8815	-0.1051
ENSG0000	GUF1	4:4462206	9.71835	11.6297	2.56452	4.83431	3.61021	7.51953	5.03901	4.00422	13.8354	5.88272	0.85575	0.122439
ENSG0000	MAP2K2	19:409032	76.5336	72.3527	114.781	87.1056	132.375	112.41	130.468	138.75	101.897	109.555	0.4478	0.237055
ENSG0000	RP11-182	10:796608	10.6007	2.84166	7.78673	6.62353	6.49079	13.4834	6.01318	8.47436	3.15916	9.68485	0.9087	0.169391
ENSG0000	MYEF2	15:481209	4.38975	9.38438	2.59015	2.36744	1.28826	1.91975	2.93058	2.72684	4.56671	2.3847	0.629	-0.34529
ENSG0000	FAM53B	10:124461	6.9182	3.29509	4.34233	1.20069	5.77116	8.38837	4.73874	7.04586	0.665401	14.4412	0.47955	0.519064
ENSG0000	TBC1D1	4:3789108	15.7825	14.4077	12.0093	17.7727	13.349	18.1038	20.7076	17.1888	25.2896	22.7789	0.28625	0.531834
ENSG0000	GFER	16:198420	13.9253	6.11792	22.1741	15.6802	18.2214	23.9192	21.1755	26.0036	19.1693	11.0366	0.7765	0.25463
ENSG0000	PAOX	10:133379	4.26906	2.97837	5.20238	6.14208	5.43615	6.90718	13.9277	6.56054	11.6335	3.87213	0.45515	0.791854
ENSG0000	BCCIP	10:125823	82.9529	79.5936	79.7951	80.5755	68.659	104.57	88.6965	63.4707	65.2104	46.2804	0.78845	-0.11116
ENSG0000	KAT6A	8:4192947	17.2331	32.7189	3.99572	4.04551	4.96307	4.77693	6.91427	2.60439	7.63952	10.8007	0.1527	-0.87354
ENSG0000	PTCD2	5:7221940	2.10163	3.91095	4.4131	2.14006	2.79954	3.33025	3.40216	2.12785	0	3.08132	0.49675	-0.54326
ENSG0000	RP11-626	16:191139	0.212972	0	0.334961	0	0.374739	3.17738	2.14758	1.74321	2.35053	1.06463	0.17455	3.47763
ENSG0000	CHST15	10:124007	1.02884	5.31245	0.13024	0.26139	0.207324	3.57168	0.639282	0.541093	0	0.114594	0.8337	-0.24101
ENSG0000	SPIRE2	16:898181	1.48457	0.602734	2.28622	2.95632	2.17995	0.041461	0	0.793438	1.87116	4.4769	0.6491	-0.46204
ENSG0000	IMMP2L	7:1106630	10.8687	13.695	12.4771	15.3108	12.7513	3.74666	6.53651	8.56782	5.31947	10.4934	0.97945	-0.03152
ENSG0000	EDC4	16:678730	36.6449	46.4839	10.2718	12.9264	14.4911	31.9261	30.7126	9.69174	8.18657	13.2772	0.3737	-0.3215
ENSG0000	GZMM	19:544033	85.9921	28.664	132.931	128.781	137.241	20.2393	38.9854	104.48	55.7292	28.5976	0.00735	-1.11275
ENSG0000	MAP3K5	6:1365570	16.9681	17.8776	4.87635	3.65015	7.69808	13.4087	9.04358	3.87912	2.23566	15.4562	0.64035	-0.19269
ENSG0000	FASN	17:820783	0.68662	0.511947	0.673838	1.09851	1.8347	0.698055	0.90435	2.39389	2.27387	3.35554	0.1125	0.910855
ENSG0000	CTC1	17:822482	33.7502	36.7858	9.94725	16.3944	11.5652	16.0373	16.863	10.0096	2.80774	28.3089	0.04375	-0.95062
ENSG0000	TCEAL1	X:1036074	11.0355	13.1678	22.8084	19.1198	17.6836	7.12449	9.65007	9.01478	0.630924	9.03084	0.03425	-1.29703
ENSG0000	TCEB3	1:2374315	25.5381	25.7711	36.317	34.4472	45.308	16.2551	19.715	29.7138	40.6718	54.6322	0.7444	-0.12159
ENSG0000	COQ5	12:120503	36.638	19.9978	39.8541	40.101	25.769	27.492	27.7116	29.536	50.5354	15.1129	0.93415	-0.06321
ENSG0000	LINC0135	1:2032871	2.2508	0.226564	2.18023	1.3015	0.12031	2.23487	1.06275	0.569641	0	0.464769	0.8337	-0.28606
ENSG0000	RP11-234	12:456220	0.257848	0.042356	0.047499	0.155831	0.340396	0.058306	0.232669	0.08096	0.162379	0.139715	0.50745	-0.93325
ENSG0000	RSBN1L	7:7769644	8.32009	7.7067	8.73681	8.35993	12.114	6.26426	11.7854	8.88656	9.0309	12.4164	0.99365	0.002734
ENSG0000	TVP23B	17:187809	47.3711	22.4022	49.161	57.566	28.9055	52.5987	53.864	38.6448	45.097	20.3959	0.92685	-0.02986
ENSG0000	POLR3D	8:2224510	10.7702	11.2343	22.5262	18.7163	17.8479	8.26849	11.3639	18.2167	10.3812	16.2808	0.4169	-0.36166
ENSG0000	SOD1	21:316596	266.993	222.552	290.414	269.836	224.548	395.991	349.16	304.018	286.993	103.612	0.6169	0.160352
ENSG0000	ZIK1	19:575715	4.8297	4.60416	3.46237	2.41899	2.50292	7.80162	0.066377	1.03714	0	3.82971	0.41235	-0.71692
ENSG0000	POLR3A	10:779692	6.11818	4.42265	3.26312	1.34191	2.56848	0.872754	4.40565	3.03868	0.608343	5.68305	0.4734	-0.31422
ENSG0000	AC109826	2:9876193	3.63059	2.61391	4.8862	7.13395	4.12208	10.307	3.50497	3.25548	2.59851	3.71701	0.9887	0.012943
ENSG0000	CTC-260E	19:200779	1.46874	1.10705	0.376068	0.814011	1.98364	1.26008	1.23363	1.70123	6.66607	2.02148	0.41065	1.11779
ENSG0000	AC108463	2:1111959	1.24996	0.630214	0	0	0.374536	0.824496	0.929287	0	1.47009	0.211699	0.61425	0.83112
ENSG0000	PHF1	6:3341039	38.3292	38.6498	21.545	17.8514	24.7923	19.4853	34.9058	37.7666	11.1544	31.957	0.9415	0.050017
ENSG0000	ZNF319	16:579946	4.09488	4.4757	2.73988	1.3523	5.3208	3.38204	1.44194	1.13136	3.25477	9.05823	0.97045	0.041684
ENSG0000	SLC25A35	17:828776	2.13309	4.63172	2.17626	3.85488	1.88814	2.02879	3.62838	3.68321	6.15359	7.61891	0.47175	0.641578
ENSG0000	AC009403	7:1554742	7.02028	4.00586	10.4594	6.65326	3.32472	8.31404	6.22762	7.7772	7.07511	7.29367	0.9253	0.096068
ENSG0000	UBE2D3	4:1027943	282.412	236.439	285.583	277.951	198.792	241.417	286.379	261.68	261.4	156.421	0.5408	-0.22553
ENSG0000	SPTSSA	14:339242	5.16759	6.1344	2.21241	3.32245	2.0513	16.0977	10.8859	1.95944	7.34547	0.892029	0.02725	1.04355
ENSG0000	KB-1027C	22:218852	2.39778	2.73041	1.00555	2.36535	3.39332	3.17393	2.50538	0.486244	1.89776	3.62335	0.83005	0.270763
ENSG0000	PRPF8	17:165062	47.4394	63.3902	14.1042	14.1663	20.0927	26.9301	19.0183	23.1571	15.244	32.4887	0.96815	-0.01819
ENSG0000	TSPAN5	4:9847036	22.1358	20.0301	16.3708	17.2006	34.2865	15.5657	36.3507	28.1358	13.207	59.6172	0.39195	0.374044
ENSG0000	TTLL3	3:9749943	12.9889	20.4553	12.4263	9.01908	6.05562	24.3293	17.5124	10.216	9.98674	16.852	0.41685	0.399667
ENSG0000	MTMR2	11:958328	11.7362	10.471	4.62494	7.61293	4.73921	10.8277	9.18361	8.35133	7.75639	8.15495	0.94465	0.027684
ENSG0000	CBFA2T2	20:334900	10.8985	18.648	25.7928	17.8082	19.1304	21.9591	12.9239	20.4282	25.7892	47.1071	0.6813	0.197942
ENSG0000	CD200R1L	3:1128157	0.101495	0.105199	0	0.017871	0	0	0	0.032427	3.05399	0	0.24005	4.4853
ENSG0000	AFF4	5:1328753	10.998	15.2831	3.12678	6.28652	5.25163	11.0492	12.6312	8.12693	7.61452	11.2347	0.61895	0.226546
ENSG0000	TNFRSF12	16:301471	1.76372	0.68216	2.30972	1.86795	2.47116	8.79422	14.7694	8.85455	0	4.18169	0.24705	1.77714
ENSG0000	INTS10	8:1981713	54.2277	49.3091	41.9966	57.7071	35.621	37.4811	44.6911	37.5289	48.2981	34.0566	0.83525	-0.06191
ENSG0000	ADAMTSL	1:1505485	2.82502	2.52587	2.40703	2.15321	0.343607	3.86756	1.6463	0.768324	2.02743	0.530417	0.75055	-0.31621
ENSG0000	GTF3C3	2:1966395	14.0538	15.7499	5.34604	9.01732	5.1874	7.78456	10.5589	7.36181	10.4105	7.64642	0.6653	-0.15822
ENSG0000	C10orf76	10:101845	10.0073	11.0005	4.55885	16.2181	4.53196	8.53839	6.6823	4.67309	3.37846	5.85982	0.4452	-0.6724
ENSG0000	SERINC1	6:1224433	73.294	57.9382	36.8513	30.6691	27.7293	59.5383	57.4634	45.106	41.6729	50.24	0.4792	0.219371
ENSG0000	RP11-552	1:1114386	1.15655	3.55564	0.701576	6.59838	1.2711	6.094	2.75782	0	2.36699	0.665368	0.96375	-0.07273
ENSG0000	C4orf32	4:1121453	1.65233	3.04663	0.537022	0.596788	0.690741	3.09171	1.65723	3.20331	4.43598	1.37266	0.01415	1.15733
ENSG0000	RPL39L	3:1871209	10.2264	6.38844	4.6821	6.20033	4.5657	22.926	27.237	18.0338	10.0797	4.27358	0.05505	1.25597
ENSG0000	PMEL	12:559541	1.08347	3.2107	0.06544	0	0	1.27189	1.24011	0.733257	0	0.229489	0.6793	-0.39165
ENSG0000	HMGB1P3	2:2304159	3.11329	1.60346	0.825803	0.542259	0.424919	1.72364	2.28678	1.2654	5.09213	2.26125	0.45	1.21057
ENSG0000	TNKS2-AS	10:917828	0.333902	0.116401	0.544151	0.974698	0	0	0.423703	0	0	0	0.30145	-2.14156
ENSG0000	NAP1L5	4:8852098	4.2757	2.47329	5.17756	5.49525	2.39157	7.50123	3.52734	2.8259	10.2668	5.63238	0.69135	0.560323
ENSG0000	KMT2E	7:1050134	167.936	218.769	217.073	121.682	166.901	181.557	192.043	170.807	164.251	319.966	0.78625	0.127171
ENSG0000	RAB2B	14:214590	9.63752	6.32053	7.62655	10.7871	5.79247	3.26462	6.27215	4.626	5.95838	7.97212	0.49135	-0.55055
ENSG0000	ZC3H14	14:885629	31.1195	37.0312	18.156	17.861	16.2402	33.8547	20.5123	17.3431	15.2794	21.8935	0.60575	-0.22984
ENSG0000	UCN	2:2728239	0.625353	1.40344	0.607988	1.14098	0.539442	2.19582	2.25902	3.9276	2.57229	1.64391	0.4851	1.59176
ENSG0000	TTC28-AS	22:278516	17.001	17.0862	17.0115	11.4635	9.88718	22.9791	25.1448	17.3089	12.0458	7.88183	0.7287	0.284597
ENSG0000	FAM160B1	10:114821	9.55708	14.0559	6.27729	6.01391	3.54786	6.74505	8.21528	7.38221	16.218	12.7077	0.5351	0.602297

ENSG0000(	ANKMY1	2:2404794	10.15	8.8089	6.88691	7.30896	6.63455	9.48696	8.73618	2.46019	8.30173	6.59328	0.79585	-0.17482
ENSG0000(	CYB5RL	1:5413268	1.1798	0.667568	1.38511	0.572121	0.671029	0.477687	1.79605	0.197658	0	2.75258	0.8483	-0.20381
ENSG0000(	AGPAT2	9:1366731	18.0629	16.4317	23.672	16.4997	27.4034	54.5395	62.271	43.1264	31.8877	26.9497	0.01075	1.00525
ENSG0000(	DDX49	19:188995	21.3888	16.7357	33.1825	38.6151	29.5594	19.3595	26.5073	26.855	21.6027	20.9317	0.76805	-0.29092
ENSG0000(	RNPEPL1	2:2404794	73.5127	60.1977	33.6482	29.693	64.4443	42.471	80.6342	41.634	25.3291	93.8293	0.94315	0.031688
ENSG0000(	CDK5RAP2	9:1203888	12.8824	21.1221	5.62294	9.45961	19.0513	20.861	26.1338	5.29145	6.47087	41.0854	0.59475	0.433546
ENSG0000(	RP3-354N	6:1191772	0.386021	0	0	0.398257	0.078541	2.0523	0.96779	0.331297	2.11892	0.691347	0.16305	2.86348
ENSG0000(	TRAFD1	12:112125	40.9162	55.0912	28.924	34.4373	22.3738	63.9375	77.7737	46.9926	46.1601	40.6784	0.0623	0.608994
ENSG0000(	THBS3	1:1551950	3.72459	4.52244	2.95118	1.54676	1.45186	0.508079	6.71297	3.74502	0.752954	4.60443	0.68075	0.336826
ENSG0000(	LA16c-40	16:566994	0.311731	0.851752	0.202399	0	0	0.642856	1.55377	0	0	1.07493	0.81475	0.459587
ENSG0000(	FBXO9	6:5300127	65.0887	75.7973	24.1516	18.4662	23.7989	50.5259	46.3439	32.9276	15.2816	22.2506	0.4866	-0.35776
ENSG0000(	BMPR1A	10:867566	0.278051	0.936888	0.305023	0.162958	0.116604	0.13888	0.612212	0.297645	0.174776	0.645664	0.81765	-0.22817
ENSG0000(	RPS26P6	8:1008957	1.35881	1.39426	2.4603	2.04491	0.9954	1.57653	2.5617	0.732172	2.10132	0	0.68465	0.665588
ENSG0000(	RPS20P14	3:1869001	0.535194	1.23322	1.66341	2.98855	0.208659	0.710907	0.415279	1.66503	2.62093	0.280424	0.97045	-0.03215
ENSG0000(	C3orf67	3:5871736	0.773824	0.644623	0	0	0	0	1.50416	0	3.09518	0.081424	0.17535	2.25725
ENSG0000(	ITIH4	3:5281297	1.93512	2.07595	0.891441	3.01992	0.985942	8.76024	7.94504	1.95693	0	2.36452	0.06255	1.77813
ENSG0000(	EDAR	2:1088944	0.064892	4.01203	0.063884	0	0.111885	0	0	0	0	0.209026	0.23975	-4.52538
ENSG0000(	RRP8	11:659507	1.50232	1.28271	19.695	18.0195	20.0039	1.74793	3.7638	22.5822	7.79528	15.2861	0.57835	-0.38286
ENSG0000(	TP53INP1	8:9481331	9.75414	16.3066	1.35578	2.53905	1.19216	10.129	5.72863	5.80014	4.03353	7.16697	0.8497	0.139629
ENSG0000(	RP11-894	3:5281297	0.111738	0.869285	0	0	0	0	1.15206	0.178207	2.12439	0.096624	0.47755	2.13221
ENSG0000(	RP11-70D	16:796058	1.31546	0.57174	0.219893	2.3748	0.361828	1.31216	3.29036	0.936001	0	1.33067	0.81545	0.483054
ENSG0000(	TCFL5	20:628162	1.46626	1.50643	1.16053	0.280396	2.21828	1.02405	0.215419	1.61913	0	3.58879	0.9711	-0.05097
ENSG0000(	TYW1B	7:7255874	4.1295	2.86736	1.66646	1.33401	1.43851	5.81512	2.17072	0.395685	0.976955	2.49932	0.79545	0.169671
ENSG0000(	ICE1	5:5420663	9.81736	7.50711	6.50305	3.88797	9.01433	4.35217	9.68249	6.35019	6.44955	8.36146	0.8417	-0.12957
ENSG0000(	1-Mar	4:1635242	0.759503	8.64082	0.123031	1.11886	0.341075	15.4569	0.987651	1.16612	0	0.512112	0.46935	0.65529
ENSG0000(	MICA	6:3139978	3.30087	4.51727	7.36892	7.06604	10.58	7.29674	3.29876	10.4654	18.8419	12.4434	0.5526	0.708316
ENSG0000(	IGLC1	22:228877	0	341.41	7.39308	4.17305	4.08904	0	0.924384	0	2.38006	1.36971	0.1806	-5.96202
ENSG0000(	TK2	16:664272	4.60256	4.99016	3.15622	6.26466	6.54525	5.743	5.87186	7.59965	2.09778	11.3566	0.5978	0.251145
ENSG0000(	CHMP6	17:809915	33.0738	32.657	41.1148	44.7833	43.4953	39.2013	39.6702	24.202	34.2676	24.0024	0.3881	-0.32002
ENSG0000(	RP5-1139	1:2282035	0.562692	0.072315	0.283988	0	0.178101	0	0	2.13615	1.7052	1.01809	0.256	2.14967
ENSG0000(	ASIC1	12:500575	0.075749	0.31242	0.144973	0	0.158884	0	1.3287	2.09121	0	1.45709	0.02665	3.13653
ENSG0000(	BTN3A3	6:2644047	55.7176	61.4309	36.168	39.508	33.2652	32.8306	34.3156	30.1487	30.3763	46.8434	0.1908	-0.38155
ENSG0000(	SARM1	17:283575	2.12156	2.86553	1.18778	0.699376	1.82958	4.05353	1.78692	2.07942	0.589292	3.57657	0.9697	-0.03389
ENSG0000(	RP11-116	8:7342000	2.2083	2.07007	0	0	0	1.46346	4.50734	0	2.14922	0	0.6074	1.02661
ENSG0000(	DISP1	1:2228150	1.97851	2.23197	1.17531	1.16159	0.353446	0.042644	0.014085	0.35372	0	0.934048	0.06685	-2.65772
ENSG0000(	AC007278	2:1024186	2.46061	0.104339	1.32951	0	0.985812	0.529013	0.028359	0.333806	0	2.01596	0.61405	-0.80961
ENSG0000(	ATL1	14:504185	0.637377	2.17795	1.87746	0.819809	1.27806	0	0	0.013734	0	0.300828	0.0005	-4.96196
ENSG0000(	RHD	1:2524223	0.083821	0.298855	0.62588	0.334931	0.171775	0.415797	0.19863	0.40349	1.85755	0.362526	0.31105	1.12101
ENSG0000(	HNRNPL	19:388154	31.5754	23.7692	8.79312	9.38816	9.55519	43.2012	36.2718	11.4782	9.65374	27.6023	0.5679	0.615103
ENSG0000(	TRIM4	7:9987598	19.1054	25.9687	19.1498	16.1221	18.264	17.9608	19.3954	14.4193	5.9907	26.141	0.5525	-0.23544
ENSG0000(	DYNLL2	17:580834	0.490358	0.30576	1.03256	0.893137	1.26053	0.240277	0.411911	0.862673	0.853567	1.12793	0.5265	-0.35128
ENSG0000(	MBP	18:769746	55.1452	58.5312	68.1124	38.7285	91.1934	42.5109	55.5766	62.1783	59.9538	110.302	0.84735	-0.11085
ENSG0000(	ZFP91	11:585791	4.58406	5.82818	2.7128	2.51955	5.56484	4.9344	4.5982	5.59888	2.56056	10.7949	0.4662	0.32248
ENSG0000(	ZMYND10	3:5034110	1.36471	1.62192	1.31674	0.857633	1.64589	0	1.74205	0.351762	0	1.29448	0.54195	-0.62763
ENSG0000(	RP13-554	12:132489	0.85214	3.27981	0.016991	0	0	0.097908	1.62718	0	0	0.516177	0.77795	-0.87465
ENSG0000(	AC002117	17:451485	1.92033	2.74759	4.16022	11.2942	2.38936	1.29033	1.90911	0.86355	9.09021	0.329258	0.41545	-1.4988
ENSG0000(	HYLS1	11:125883	6.49299	9.11499	7.07319	5.31249	8.58626	3.9563	7.07262	3.28907	0	2.58791	0.3185	-1.15665
ENSG0000(	EID2	19:395387	26.2465	30.0757	6.76479	3.90446	6.00103	18.296	27.3816	2.87903	0.311806	4.33045	0.3781	-0.46138
ENSG0000(	CUL9	6:4318217	10.6903	19.308	4.47761	3.49915	4.11313	7.86186	18.9842	2.55178	4.38096	9.29786	0.5601	0.383725
ENSG0000(	WRNIP1	6:2765413	23.7836	21.9864	23.5102	14.5145	32.3207	13.6016	27.9874	19.9147	9.46013	35.278	0.7169	-0.14925
ENSG0000(	MPHOSPH	12:123152	17.5428	15.555	9.45876	14.9066	11.6715	18.8083	14.8774	18.4281	17.0254	30.1879	0.5958	0.257489
ENSG0000(	MGLL	3:1276890	0.434781	0.72882	1.18835	1.88196	0.42416	23.0184	21.2733	7.99792	10.6405	12.2462	0.00005	3.81784
ENSG0000(	CTD-2368	19:579770	1.20809	0.320492	0.122551	0.034444	0.873649	0.124094	3.4513	0.661122	0.192802	1.77649	0.33315	1.15281
ENSG0000(	TTLL11	9:1218219	3.64149	3.42871	3.03947	3.67193	3.07497	0.139841	2.67698	1.93239	0.393154	3.53311	0.3595	-0.84836
ENSG0000(	ST6GALNA	1:7607471	0	1.34456	0	0	0.070976	1.14682	4.93865	4.08535	0.002707	0.480658	0.23975	2.98391
ENSG0000(	SLC25A22	11:790474	13.6169	11.2012	8.48194	7.81196	11.5293	8.02462	10.1765	10.8836	2.69881	17.7894	0.92635	-0.13101
ENSG0000(	LANCL3	X:3734927	0.197962	0.566478	0.119318	0	1.01396	0	0.576791	0.802981	0.00091	0.692427	0.93885	-0.11283
ENSG0000(	TMEM176	7:1507912	1.09115	0.754512	0	0	0	58.9749	1.09897	3.52606	0	0.057453	0.0138	5.13431
ENSG0000(	C21orf33	21:441336	34.1078	31.4176	34.2282	49.4926	30.8429	41.5239	35.3262	28.9327	38.4013	15.551	0.8791	-0.11008
ENSG0000(	DNAJB12	10:723328	14.3932	17.1048	11.4595	8.6041	11.9787	26.5588	25.9269	9.51158	7.14688	12.2953	0.754	0.124371
ENSG0000(	CRACR2A	12:360663	12.7679	5.03997	6.75599	21.4338	9.27948	8.3436	5.4592	8.9903	16.4759	13.0766	0.8404	-0.11995
ENSG0000(	UBAP1	9:3417900	42.553	23.7882	47.8229	40.3964	40.8818	30.1802	19.1708	28.8143	23.3179	43.7527	0.13565	-0.4992
ENSG0000(	NUF2	1:1631111	8.06105	7.52067	2.39602	1.00993	1.15657	8.87345	8.27035	5.29124	9.08452	2.12927	0.2203	0.920358
ENSG0000(	IL4R	16:273133	9.91144	42.2514	2.32667	5.79006	4.32418	16.5828	21.9607	6.51975	13.7931	9.11091	0.6766	0.195645
ENSG0000(	RP11-478	4:2269581	0.41471	0	0	0	0	2.70983	0.359079	0	0	1.2574	0.2203	3.6892
ENSG0000(	MCM3AP	21:462292	27.8369	23.916	9.97987	11.5941	11.824	19.801	14.2241	8.81583	7.63048	24.3589	0.23515	-0.48542
ENSG0000(	METTL18	1:1696620	45.3816	33.4669	37.3494	29.3613	37.9427	57.036	38.2904	41.5821	24.3965	20.1806	0.9786	0.025704
ENSG0000(	HIST1H4C	6:2610387	416.242	248.556	745.116	389.526	310.199	345.488	367.188	447.202	418.346	80.7923	0.2578	-0.38266
ENSG0000(	HP1BP3	1:2074266	107.665	112.451	64.055	70.0385	115.714	85.7155	89.3943	74.5547	57.3228	133.492	0.5239	-0.23912
ENSG0000(	TNFAIP2	14:103123	3.27129	20.0721	1.42356	2.24554	2.4403	11.4941	1.30283	7.21807	14.9462	3.46047	0.32165	1.0582
ENSG0000(	DIP2B	12:505049	2.82144	6.5319	0.69841	1.51793	1.37526	2.47494	1.92209	2.48611	6.21419	2.69546	0.40765	0.396885
ENSG0000(	XXbac-BP	6:3135387	0	4.67616	0.067069	0	0	0.148928	5.10147	0.122224	0.09974	0	0.8981	0.372446
ENSG0000(	NFKBID	19:358876	8.04181	13.8066	7.55032	12.072	6.98506	9.90021	31.2802	56.1994	0.079839	20.208	0.17785	1.21224
ENSG0000(	CD200R1	3:1129212	7.04682	12.9283	4.69736	7.50641	1.82521	2.47415	7.04772	6.89344	8.31007	5.8317	0.76405	-0.14141
ENSG0000(	SNHG10	14:955322	13.3026	17.3237	17.5223	14.0404	6.92598	13.0734	18.0019	17.1786	7.67366	12.0499	0.9609	-0.04073
ENSG0000(	TRAV8-4	14:218944	11.6072	37.1175	8.95928	5.40261	7.17698	51.2182	19.8134	23.0671	3.38802	8.99574	0.3666	0.651771
ENSG0000(	APOL1	22:362530	23.2204	12.5287	15.1211	5.92668	20.4843	24.5654	27.6123	8.51288	2.03713	27.2176	0.94075	-0.03344
ENSG0000(	NT5DC2	3:5249533	0.979719	2.16775	1.47673	0.803825	1.00672	10.8748	2.58573	10.2647	6.56864	3.09786	0.00605	2.52742
ENSG0000(	PTBP1	19:797074	83.8946	92.9619	40.2534	32.5761	92.3792	55.1848	79.2864	61.6994	40.8486	103.831	0.89135	-0.04548
ENSG0000(	ENTPD1-A	10:957117	7.35933	8.51313	5.27217	9.90886	3.21999	8.96312	3.92263	3.24555	6.04435	5.52619	0.92205	0.103787
ENSG0000(	RP11-275	15:386718	2.17406	3.41817	1.74107	4.37765	0.745938	0	0.491804	1.03048	3.11409	0.29379	0.58655	-1.07043
ENSG0000(	APOBEC3A	22:389527	1.47264	12.1068	0.776004	1.5582	3.34588	4.22845	0.228754	5.95058	6.21815	4.71399	0.7277	0.316989
ENSG0000(	UTP14C	13:520123	4.01946	2.27246	2.11235	1.64148	2.8243	2.35756	3.45155	2.901	0	7.1384	0.5631	0.541552
ENSG0000(	GNLY	2:8568517	104.926	244.86	929.391	880.24	273.169	451.548	997.488	405.057	129.687	45.335	0.3346	-0.48404
ENSG0000(	ZBTB20-A	3:1143145	0	0	0.725678	0	0	0	0	0	2.97513	0	0.59025	1.8227
ENSG0000(	ULK2	17:197708	3.99362	4.72641	1.1802	3.00816	1.50959	3.01784	2.05699	0.228212	1.35221	2.32226	0.1369	-0.75098
ENSG0000(	CIART	1:1502825	0.344381	0.539008	0.217556	0	0.361806	0	0.717098	2.11038	0.09575	0.916589	0.19795	1.50462
ENSG0000(	YME1L1	10:271101	75.113	57.7317	34.9747	32.4507	29.802	60.2824	51.6932	39.5485	38.4637	39.755	0.9894	0.004645
ENSG0000(	BCAS3	17:606774	13.7913	10.8719	14.7066	8.99285	7.31772	5.5528	6.592	7.04283	3.2907	12.953	0.1846	-0.59433
ENSG0000(	SLC17A3	6:2583306	5.37527	0	3.13352	6.84865	2.92256	1.24395	0	2.21969	2.48102	2.65399	0.3412	-1.01948
ENSG0000(	LRRC40	1:6956839	12.6721	5.36504	5.75765	6.76365	4.41775	10.999	8.6095	7.0845	6.24821	6.39623	0.71815	0.153255

ENSG0000	WSCD2	12:108129	0.33969	1.35674	0	0	0	1.793	0.956443	0	0	0	0.5694	0.871635
ENSG0000	IGKV1-16	2:8909985	0	1.34055	0	7.5277	3.1661	0	0	0	0	0.15403	0.2819	-5.58797
ENSG0000	ZFAND1	8:8170133	27.5403	27.7684	28.5007	25.9196	14.2955	36.2692	22.3242	13.113	33.7931	12.1185	0.87905	-0.05493
ENSG0000	PIF1	15:648156	2.09604	2.10424	0.758119	1.03631	0.176362	3.50088	4.35164	4.83201	0	3.78578	0.12625	1.35901
ENSG0000	OSTC	4:1086505	155.644	92.2517	192.523	191.347	77.5917	164.885	146.739	143.035	121.455	27.2931	0.5255	-0.21859
ENSG0000	SLC10A3	X:1544873	31.7564	21.4863	61.5148	63.5461	57.923	34.264	28.2962	48.4973	33.2396	45.9277	0.25635	-0.39035
ENSG0000	TSSC1	2:3188924	25.1719	16.1599	30.4267	25.1215	28.1957	20.8224	18.4243	12.8402	40.6713	22.738	0.74235	-0.12489
ENSG0000	TAMM41	3:1179044	15.306	21.9509	18.871	17.4268	15.6637	8.33587	18.6189	9.37451	19.0823	14.773	0.50425	-0.3208
ENSG0000	MTMR12	5:3222699	8.81227	5.4779	3.64243	5.17431	1.73795	0.762819	1.11667	2.54764	4.30282	4.02223	0.0942	-1.09513
ENSG0000	TPT1	13:453334	1005.67	622.015	704.255	431.06	322.328	981.172	900.485	880.527	574.866	112.682	0.6983	0.171621
ENSG0000	THAP7	22:209991	42.8659	49.0389	60.506	36.8195	48.294	43.8528	61.4664	53.6509	29.9415	20.3301	0.6113	-0.23048
ENSG0000	GTSF1	12:543536	20.4048	5.98102	18.2444	9.10333	13.6563	18.1771	1.68254	3.09073	2.31174	4.82302	0.3547	-1.31827
ENSG0000	FDPS	1:1553087	70.8514	72.4134	100.328	123.441	101.065	112.709	125.262	112.107	106.708	66.6649	0.63555	0.169956
ENSG0000	CHERP	19:164790	22.4417	27.0432	5.70226	6.34422	9.10772	15.6862	19.6802	7.60079	4.23714	16.362	0.92525	-0.08534
ENSG0000	TMEM91	19:412624	20.4215	10.8162	14.4767	16.5572	14.6105	3.9629	4.85774	6.70761	7.42016	5.53149	0.0103	-1.45921
ENSG0000	C8orf44	8:6666759	7.0663	7.18297	9.98406	8.94118	2.84182	1.01951	8.46213	7.4175	9.23611	2.19477	0.80255	-0.25509
ENSG0000	POP5	12:120578	59.0959	55.2127	88.4773	66.5718	62.5978	63.882	47.7628	43.3569	37.3761	37.1095	0.2247	-0.49297
ENSG0000	SLC9B1	4:1028689	0.950673	3.9059	1.72338	1.55618	0.374669	2.0567	1.15486	3.10706	0.56509	0.479146	0.7804	-0.26406
ENSG0000	SNRPB2	20:167148	49.0872	32.6625	60.6576	46.7866	43.4605	87.4815	104.342	54.7079	39.3992	18.1146	0.93045	0.043607
ENSG0000	RP11-448.	7:1296045	0.540048	0.722349	0.172412	0.472674	0.203747	0.760589	0.096373	0.241123	0.60213	1.27598	0.6077	0.404448
ENSG0000	RP11-147	17:681257	0.897408	4.38863	3.50791	2.6215	0.971134	0	1.55407	5.08473	3.76375	3.2776	0.83765	0.354672
ENSG0000	RPS12	6:1328144	253.939	300.252	514.446	299.65	88.1316	229.743	289.938	310.202	215.803	15.371	0.16965	-0.49147
ENSG0000	RP11-493	12:472377	0	2.59155	0	0	0	0	0	0	0	0	0.19555	#NAME?
ENSG0000	AE000661	14:222025	14.0788	1.61095	0.708354	1.85356	0.710716	10.3835	4.93755	0.090168	0	0.745442	0.75505	-0.46462
ENSG0000	NFATC2	20:513869	16.1001	14.9966	11.3455	7.86289	14.572	9.54642	21.0316	13.8026	9.4315	29.7053	0.4549	0.253652
ENSG0000	UBA5	3:1325581	26.8287	30.1039	23.1058	29.2121	19.749	23.1084	25.1417	27.4825	21.8655	17.3534	0.64525	-0.23984
ENSG0000	AC078883	2:1724273	2.65984	6.6041	0.606856	0.803315	0.869569	0.347415	0	0	0.699444	0.373743	0.1051	-2.72765
ENSG0000	MTF1	1:3780956	3.66583	4.67704	4.23221	2.11402	2.87963	1.68492	4.27738	3.38905	3.82499	6.42311	0.91135	-0.05915
ENSG0000	RP11-271	3:1137164	1.51535	1.36814	3.85516	1.33767	0.930773	0.406392	1.52773	0.741798	4.57831	2.18076	0.8827	0.21745
ENSG0000	EXOC6B	2:7217598	1.5984	0.523493	0.294983	0.753469	0.701531	0.674786	0.589369	0.809443	1.23737	1.08921	0.9328	0.060747
ENSG0000	EEF1A1P6	7:2241944	81.6373	59.0019	81.2282	78.7872	67.0987	53.1458	46.3831	46.5429	56.0644	22.3089	0.3454	-0.70069
ENSG0000	SH2D2A	1:1568062	110.125	36.1022	90.8102	94.1954	73.6718	115.173	133.952	115.464	104.842	97.9012	0.2025	0.411041
ENSG0000	RMDN1	8:8633327	25.1809	25.7021	20.5149	29.7593	17.5429	23.1878	17.3597	16.4599	28.0199	15.2794	0.7107	-0.16012
ENSG0000	SLC35B3	6:8413067	14.4995	17.2364	6.91058	11.1451	5.85307	10.3773	7.34905	8.43412	6.30801	8.89643	0.356	-0.43421
ENSG0000	HSD11B1L	19:567494	2.48766	3.52075	4.55111	4.13	1.19364	0.332239	0.931757	2.66273	3.48598	1.33693	0.5875	-0.38671
ENSG0000	S100P	4:6693068	6.43574	1.2176	1.59065	14.0613	1.07705	5.67288	0	2.9529	5.39664	0.473465	0.63335	-0.74215
ENSG0000	CDKN2B	9:2180254	0.104937	0.234434	0.009356	0	0.160158	0	1.29142	0.557823	2.23743	0.068968	0.0645	2.79157
ENSG0000	ARIH2OS	3:4891778	9.99936	4.92769	15.7353	15.8727	6.01654	12.2735	5.36588	7.39112	26.7787	5.21497	0.9042	0.16176
ENSG0000	MRPL50	9:1013876	12.4125	11.595	10.9381	8.80317	7.68706	16.3766	21.1086	8.15106	10.3913	3.02791	0.46415	0.25989
ENSG0000	ADGRE5	19:143805	194.863	152.9	41.8056	176.822	100.824	164.264	250.745	181.031	175.687	145.1	0.2294	0.403533
ENSG0000	ZNF738	19:213589	1.70383	1.59115	2.32668	3.63027	1.61405	1.30091	4.02601	3.6379	2.27128	5.95512	0.5554	0.655397
ENSG0000	PYGB	20:252480	19.5599	31.3624	8.22163	8.2541	9.61583	8.51277	17.4756	11.8202	4.60705	12.9573	0.3531	-0.48483
ENSG0000	IDSP1	X:1494769	5.00264	1.31619	3.29555	2.26697	0.372578	1.87898	2.72254	2.45388	4.07795	0.480901	0.7299	-0.60988
ENSG0000	MRPS26	20:304594	64.9586	62.3421	94.4024	96.788	102.997	65.7332	72.8243	65.395	48.3056	44.5104	0.09255	-0.57853
ENSG0000	PFN1P1	1:1716705	3.10362	5.48761	4.64055	10.1144	1.50232	3.47203	3.70339	2.56959	5.68536	0.653165	0.70795	-0.61889
ENSG0000	BICDL1	12:119989	16.4958	25.2384	7.74065	6.93947	12.4899	10.7146	26.5829	19.0755	2.20517	23.4643	0.544	0.248136
ENSG0000	RP11-767	1:4562730	0.462352	0.753704	0.326343	0	0	0	0.777485	0.090522	0.593778	0.455436	0.7483	0.512727
ENSG0000	SLC37A3	7:1402936	16.2879	30.1031	10.2893	12.7441	4.50763	5.44238	14.9708	7.59772	5.10543	12.4872	0.3317	-0.42102
ENSG0000	AP000892	11:117199	3.12288	4.44109	1.02397	1.73942	1.43693	0.384566	1.10449	1.17271	2.12176	3.0752	0.8576	0.247589
ENSG0000	CRHR1-IT	17:456203	7.59792	15.8994	10.1365	8.08576	8.9999	11.8843	6.15569	4.12968	15.8413	15.7764	0.9343	0.038479
ENSG0000	SLC9A1	1:2709881	7.96004	7.36577	1.70452	1.05344	2.66084	4.56565	5.89028	2.74964	3.52704	7.09617	0.54055	0.328623
ENSG0000	TSPAN15	10:694514	21.6682	0	17.9202	56.5904	29.479	7.60888	0.42585	2.8	27.4754	10.0049	0.05505	-1.31579
ENSG0000	ZNF345	19:367975	4.36328	8.53791	3.69245	4.0705	1.81949	2.75624	7.24765	8.20955	4.94835	3.33924	0.8031	0.141182
ENSG0000	PHF14	7:1097387	9.20393	11.2179	4.05453	3.65778	6.63851	11.1514	13.09	7.68947	1.71385	8.43231	0.75075	0.154771
ENSG0000	EIF2A	3:1505419	31.4778	35.2992	33.0866	31.7438	25.6952	30.6888	34.1099	29.4483	17.668	25.2387	0.80205	-0.20793
ENSG0000	NHLRC4	16:566994	0.651019	2.48986	0.454395	0.225178	0.587375	0	1.36689	0.450437	1.25248	0.317168	0.8594	-0.25792
ENSG0000	ALKBH6	19:360033	42.4836	54.5473	43.6292	42.3087	33.4406	64.2126	65.8284	38.0295	22.8539	31.2181	0.8064	-0.12553
ENSG0000	CDK11B	1:1632094	14.7055	20.2002	14.9422	8.70343	44.1311	16.4373	25.6403	14.127	11.9102	31.4997	0.825	-0.12903
ENSG0000	TMEM161	5:8818963	7.03421	13.7266	17.0983	9.8722	6.10942	9.78142	10.9998	9.00395	9.73681	10.1914	0.88845	-0.09513
ENSG0000	PP7080	5:441497-	18.6478	23.6748	18.8712	19.8916	12.3003	14.1816	7.69723	16.9068	0	15.1924	0.2943	-0.69528
ENSG0000	FAM103A	15:828369	27.79	24.552	31.1354	22.9123	26.9839	50.0564	30.1624	30.4264	15.2453	8.94692	0.96435	-0.03855
ENSG0000	LZTFL1	3:4582331	4.11493	6.16821	2.94573	3.95764	2.73769	3.77836	6.12039	5.79598	6.76613	8.53773	0.47485	0.384464
ENSG0000	RP13-103	17:816811	12.2213	16.3659	15.0827	21.8323	16.5223	6.24158	8.99192	9.03008	8.02031	10.6241	0.7803	-0.69231
ENSG0000	HSP90AA1	14:102080	191.424	132.118	289.29	242.414	545.304	245.622	233.87	273.544	331.819	555.532	0.9224	-0.05046
ENSG0000	SUCLG1	2:8442352	78.4001	57.9742	68.4676	60.9234	71.0788	80.7523	60.2972	57.1846	67.4393	27.0805	0.5375	-0.20684
ENSG0000	GCC2	2:1084485	103.679	125.242	61.694	58.6159	107.222	102.676	82.2879	68.7786	66.8637	136.842	0.71435	-0.16108
ENSG0000	MYO9A	15:718183	6.14198	9.12405	3.11552	3.4114	3.81322	6.44075	8.81283	4.1302	8.17123	9.79885	0.60975	0.277017
ENSG0000	LINC0090	18:745917	18.911	10.9433	27.4169	14.6346	17.0494	12.0092	18.0629	20.139	14.8857	13.3043	0.38935	-0.4277
ENSG0000	BRAF	7:1405184	8.74779	9.29617	3.26222	8.90318	3.73267	6.29007	8.15218	8.53338	8.13768	5.58508	0.9053	0.124099
ENSG0000	IL17F	6:5223668	0	0	0	0.065753	0.056012	6.01245	2.69411	104.197	154.375	1.16213	0.2474	11.0714
ENSG0000	SETD1B	12:121804	1.61154	3.69296	1.81996	0.921289	3.12978	2.1692	3.10198	2.2426	1.1994	7.48436	0.40425	0.405263
ENSG0000	PER2	2:2382316	0.746391	0.813913	1.73567	1.44084	1.79954	1.69469	0.550376	2.71365	1.82871	3.78262	0.69665	0.290381
ENSG0000	CKLF	16:665525	224.615	82.52	298.76	323.966	129.808	641.352	510.524	468.195	300.586	74.1516	0.0567	0.833318
ENSG0000	CARS2	13:110641	31.3623	57.3448	28.1256	14.8449	30.3646	29.9273	34.6013	33.7308	20.6228	22.5418	0.63525	-0.18837
ENSG0000	LINC0156	16:424582	1.77462	0.537099	2.63201	1.02843	1.68611	0	0	1.44427	3.29206	1.83498	0.9481	-0.0808
ENSG0000	CAMKK2	12:121237	8.76406	10.5906	7.93177	10.6708	10.7511	6.79651	7.44815	9.2263	5.49483	14.5552	0.56735	-0.36288
ENSG0000	TMEM201	1:9588921	1.17587	1.4449	0.623845	0.643241	0.530786	0.449324	1.0992	0.20493	0	2.33302	0.86845	-0.17973
ENSG0000	RAB6B	3:1337840	0.383543	0.657245	0.302387	2.47845	1.95819	0	0.390343	0.051209	1.38189	0.97199	0.4075	-1.14534
ENSG0000	R3HDM4	19:896502	97.1279	137.608	59.8259	86.8746	87.8817	98.1976	101.101	99.934	111.11	84.3979	0.7565	0.098933
ENSG0000	CCDC152	5:4275680	3.16565	5.34018	1.6748	3.4433	3.44517	0.651875	4.63991	1.48118	4.53149	4.15707	0.7239	-0.21175
ENSG0000	RP11-102	3:3069766	0.329679	2.35971	0.376939	0.516971	0.464055	0	0.124083	0.095844	0	1.48356	0.45935	-1.58324
ENSG0000	NR1D1	17:400190	5.78875	3.03281	9.48775	7.87902	22.0104	2.67273	0.624669	5.92164	6.80782	19.7214	0.58845	-0.54479
ENSG0000	CDAN1	15:427235	6.96235	5.36935	0.798095	1.06832	1.64152	3.41515	5.32652	1.8385	0.394953	3.54312	0.8443	0.144601
ENSG0000	TMC3-AS	15:813243	0.302377	0	0.591975	0	1.04122	0	0.888692	0	0	0	0.42825	-1.47074
ENSG0000	3-Mar	5:1268677	5.18994	3.29082	1.79668	2.42102	2.76019	9.71106	3.38858	8.78392	13.5447	6.03971	0.02385	1.55965
ENSG0000	ZNF445	3:4442112	1.16855	1.74898	1.12043	0.428407	0.620421	1.72576	0.183762	0.991983	0.07312	1.61926	0.76265	-0.25824
ENSG0000	UBR3	2:1698274	5.6955	7.7217	1.12109	1.45328	2.42387	0.442461	2.84379	3.07383	4.31522	2.76523	0.7686	-0.23295
ENSG0000	TRAV13-1	14:218688	43.926	68.8312	27.8942	33.1092	18.8979	70.498	74.8327	54.9561	42.037	17.1236	0.26525	0.534887
ENSG0000	CTD-2260	5:9681402	3.50606	4.83104	1.76953	2.04261	2.27173	4.93327	3.60827	1.35789	2.67813	3.34046	0.9228	0.140598
ENSG0000	UFD1L	22:193306	75.8269	58.8173	83.6482	88.7631	95.2988	104.418	119.987	87.5863	103.38	35.5429	0.8711	0.087069
ENSG0000	RP11-127	4:8235249	20.0612	13.6269	12.587	11.7459	9.92336	15.8668	14.4614	19.5525	25.7356	6.30169	0.836	0.298468

ENSG0000	ARHGAP2	4:8547511	0.50123	2.63362	0	0.042451	0.236998	3.85948	0	0	0	0	0.8388	-0.19277
ENSG0000	PLEK	2:6836121	148.069	54.3402	249.8	226.046	270.292	57.0864	3.4489	36.8957	21.8388	77.9274	0.00005	-2.44806
ENSG0000	RP11-464	10:736259	2.36705	1.1088	3.13539	2.90661	1.02033	0.946608	3.65952	1.31205	2.55632	3.63808	0.8064	0.205489
ENSG0000	YIF1B	19:383051	34.6714	25.7976	56.9052	71.9705	65.2158	56.6762	35.8704	56.2315	79.6697	35.3848	0.919	0.038242
ENSG0000	SNRNP27	2:6989355	77.1982	64.7577	75.4191	69.4655	63.3075	87.337	113.338	70.0196	80.5985	26.0584	0.8889	0.064277
ENSG0000	RP11-658	11:666165	7.3889	11.5415	8.96428	8.37674	6.72692	11.2878	9.83223	12.2259	13.2426	14.8341	0.414	0.766409
ENSG0000	FAM161A	2:6182485	0.843973	0.582647	1.26298	1.1653	1.92878	1.76622	0.80117	3.02712	0.014176	1.703	0.8651	0.147374
ENSG0000	APOBEC3	22:390406	195.063	45.7315	280.157	294.434	208.212	314.284	320.95	339.597	147.765	159.951	0.6126	0.195467
ENSG0000	SIAE	11:124622	0.493662	0.372917	0.371729	1.00101	0.755931	2.36439	0.151853	1.52458	1.75583	1.8434	0.2975	1.09876
ENSG0000	RP4-575N	1:1012356	1.29384	2.17593	0.415233	0.164019	0.921913	0	0	0.441239	1.16514	0	0.4349	-1.26964
ENSG0000	NONO	X:7128319	154.905	177.047	117.562	123.843	103.34	164.465	160.406	127.162	172.165	136.937	0.4246	0.250429
ENSG0000	RP11-398	10:148776	1.70441	1.30399	0.966773	0.6355	0.314322	2.8627	0.266412	0.334872	0.936543	0.496477	0.9556	0.020258
ENSG0000	LRRC42	1:5394607	5.8498	9.59028	4.08216	4.76256	6.04261	8.6574	9.34289	3.56572	5.5241	2.84309	0.93045	-0.04964
ENSG0000	PSMG1	21:391747	23.1928	18.8872	10.0951	11.4601	14.9804	25.0048	21.7641	6.41091	16.988	8.90266	0.62855	-0.23406
ENSG0000	TMEM87B	2:1120552	5.07778	2.89764	1.72671	1.66352	2.68155	9.1738	3.69073	4.48646	3.09449	8.37058	0.01145	1.13487
ENSG0000	EIF4E2	2:2325479	34.7584	43.304	80.8158	68.2032	52.7354	60.9651	55.2305	60.729	71.3983	32.5657	0.95855	-0.02431
ENSG0000	TAOK2	16:299736	6.62369	3.57874	2.90097	2.52072	5.48151	7.26588	7.05441	5.94904	4.50783	11.8944	0.133	0.712072
ENSG0000	BORA	13:727277	10.616	7.31397	5.03516	7.0996	3.01754	7.62481	9.62863	3.66439	7.2497	3.96774	0.93975	-0.07384
ENSG0000	TPI1P1	1:7669978	0.416011	0.230728	0.364313	0.356177	0.30896	0.591431	0.384948	0.344105	0.535027	0	0.96085	0.076624
ENSG0000	TERF2IP	16:756274	282.779	173.212	302.928	317.693	357.134	194.144	184.72	218.259	191.555	166.011	0.23725	-0.61418
ENSG0000	TMEM254	10:800468	0.283931	0.036819	0.082472	1.67893	0.233344	0	0.040587	0	3.88069	0.062103	0.6161	0.993967
ENSG0000	GPS1	17:820479	78.3348	83.4421	97.0167	92.8282	92.1298	35.0503	74.0521	96.4127	41.5529	54.72	0.1012	-0.60489
ENSG0000	Metazoa_	14:498536	0.199434	0.126485	1.37834	0.20207	0.610015	0.681121	0.151461	3.13787	0.823858	0.464622	0.55595	0.956593
ENSG0000	RP11-862	18:769746	0.473178	0	0	0.321149	0	0	0.088281	0	1.45123	0.069149	0.9537	-0.10419
ENSG0000	CTB-31O2	19:187487	34.4634	33.9714	44.8665	74.9123	26.7504	45.2539	51.4641	40.4271	69.0792	36.9902	0.67255	0.161586
ENSG0000	SUGT1P3	13:408222	1.40046	0.443279	0.723361	1.02907	0.820746	0	1.45085	1.66564	0	0.924649	0.7166	0.360829
ENSG0000	PMCH	12:102120	3.65227	0	0	0	0	4.18906	35.482	3.05083	2.89892	0	0.2548	4.1646
ENSG0000	RP11-395	7:1009696	13.334	3.17415	6.44336	8.79346	2.65718	7.98435	4.84986	7.91868	7.57054	0	0.9052	-0.22187
ENSG0000	C17orf96	17:386717	0.322082	0	0.267083	0.151989	0.492553	0.143068	0.613784	0.422777	1.60382	0.96586	0.51375	1.6189
ENSG0000	RMI1	9:8398071	5.34763	5.19181	1.20486	0.83361	1.34234	3.49689	3.87651	0.726674	0	3.07256	0.6551	-0.38435
ENSG0000	RP11-546	12:123152	3.43619	5.52785	9.70142	1.86283	3.76866	4.4483	4.85846	10.2161	0.1652	11.5426	0.8832	0.305853
ENSG0000	GPLD1	6:2440290	0.273568	1.62965	0.082937	0.214793	0.305168	0	0	0.032653	0.214276	0.028285	0.002	-5.37764
ENSG0000	HIST1H1A	6:2601708	2.19102	5.03081	0	0.445503	0.212086	0	5.21771	0	0	0	0.60315	-0.72612
ENSG0000	FAM21FP	10:457064	3.01172	3.36686	1.09047	2.67921	0.432859	1.78813	1.75967	3.06492	3.96475	1.18255	0.7226	0.406245
ENSG0000	ENC1	5:7462740	0.798112	1.85215	0.459816	2.71187	0.201982	0	0	0.216263	0.025923	0.104465	0.02795	-4.51021
ENSG0000	CTD-2510	17:591554	0	0.825337	0	0	0.043721	0.175352	0	0	0.270114	0	0.12985	inf
ENSG0000	MYDGF	19:463951	117.398	61.3457	256.768	247.995	177.401	133.636	146.638	160.377	226.324	45.9477	0.40885	-0.30755
ENSG0000	EDN3	20:593004	0	0	0	0	0.0744	0	0	0	2.61861	0.285078	0.33145	5.40346
ENSG0000	COX4I1	16:857717	439.995	366.841	636.366	668.385	480.621	629.891	571.329	621.421	602.86	138.134	0.7991	-0.10058
ENSG0000	LACTB2	8:7060857	8.6568	8.317	7.86437	11.0453	5.91481	7.93773	6.18716	6.1821	2.11654	4.88074	0.29795	-0.66275
ENSG0000	RP1-317E	1:2580017	1.05076	1.80186	1.23396	0.857908	0.215947	0.267819	0.160766	0.233267	0	0.268734	0.02905	-2.59597
ENSG0000	LPAL2	6:1604534	2.86941	0.680624	0.381726	2.11756	0.456437	3.51319	0.830308	0.565241	0	0.833472	0.7154	-0.40125
ENSG0000	OXSR1	3:3816508	11.2652	17.3994	3.9573	3.47907	5.12046	7.47592	6.53557	5.55384	7.49884	11.8737	0.91425	-0.04308
ENSG0000	B3GAT2	6:7066777	1.47014	1.96453	0.415587	0.276404	0.020863	0.481743	0.100987	0.368511	0.715379	0.286676	0.6286	-0.79686
ENSG0000	POLH	6:4357614	6.71434	6.65389	5.23889	5.30723	3.97057	2.99396	6.1415	4.99621	4.93567	8.15289	0.90715	-0.06243
ENSG0000	XPC	3:1412493	33.8867	39.7672	38.9769	40.5554	31.5673	26.5844	36.9935	27.4457	32.9191	44.8169	0.7988	-0.16481
ENSG0000	ZNF702P	19:528618	3.05202	2.5021	0.021394	0	0.069967	2.82146	1.15297	0.017517	0	0.705815	0.8096	-0.27304
ENSG0000	FAM19A1	3:6800421	1.3029	0	2.18671	2.21056	1.95023	0	0	0.058969	0	0.070723	0.166	-6.84413
ENSG0000	SERINC5	5:8011165	3.83894	35.1005	1.43938	2.37103	0.616525	8.06757	7.93437	3.11329	5.29578	6.29725	0.41075	-0.48246
ENSG0000	POLR2A	17:748436	18.6477	15.6992	11.3001	5.34808	22.641	11.2786	10.7858	16.2365	15.8308	32.8559	0.6693	0.167844
ENSG0000	CXCR5	11:118883	1.73189	7.75856	2.64682	1.86514	2.71119	4.61044	5.13968	2.73121	0.626542	7.59076	0.8581	0.124657
ENSG0000	PGLS	19:174687	36.1128	39.289	50.7867	40.5316	66.7663	38.4529	40.6387	37.7925	26.9421	22.4516	0.1877	-0.58478
ENSG0000	PMVK	1:1549247	64.3766	33.8244	56.7178	75.3453	76.7044	96.8939	87.0876	68.951	85.7493	37.6054	0.4092	0.261667
ENSG0000	MTA1	14:105398	3.23202	4.15921	1.14991	1.44819	3.25417	4.13819	4.63997	3.28807	0	5.1044	0.60005	0.343561
ENSG0000	YLPM1	14:747633	16.5245	28.0639	7.08244	3.74123	14.7081	15.3604	12.4827	10.2231	8.40439	28.18	0.8829	0.058214
ENSG0000	TRIAP1	12:120438	42.3188	32.5528	47.734	52.5871	35.7112	48.5775	39.5665	33.9878	40.5865	16.6273	0.8674	-0.24413
ENSG0000	STBD1	4:7621403	1.69011	0.316427	0.790242	0.427221	0.566282	0	2.01129	0.475516	0	1.23854	0.65795	0.368806
ENSG0000	TRAV19	14:220075	6.39585	20.7325	4.29555	3.83217	0.685566	26.974	10.0107	1.62936	1.06591	0.685904	0.8423	0.1398
ENSG0000	TNFRSF11	18:623252	0.186421	0.356787	0	0.196775	0	1.05227	0.661648	0.207454	0.702821	0	0.2016	1.98717
ENSG0000	TMEM141	9:1367913	26.5392	17.0626	30.2609	25.5762	16.3625	33.0279	23.4564	25.2727	13.5654	3.4231	0.63485	-0.29817
ENSG0000	DUSP7	3:5204891	6.50054	2.89311	7.98695	6.74283	10.884	7.17342	2.34732	4.28789	2.74624	21.2415	0.97895	0.013775
ENSG0000	INF2	14:104689	5.85432	13.6983	3.09441	5.66469	1.08945	16.1111	4.97158	1.50555	0	4.12176	0.96395	-0.05017
ENSG0000	RBM18	9:1222376	17.1318	13.2052	12.24	8.92613	8.93831	14.1216	8.96246	11.7986	9.87508	7.01023	0.56925	-0.24165
ENSG0000	TSR2	X:5444040	47.3264	50.7927	68.8441	50.9359	55.8278	37.1289	38.9964	47.2717	58.9955	46.0062	0.4481	-0.24931
ENSG0000	CTSA	20:458886	144.427	94.0657	131.517	120.97	388.997	230.703	189.106	172.943	155.873	391.297	0.4355	0.282065
ENSG0000	SKP2	5:3615198	11.3942	14.2169	13.4098	12.8091	8.63456	12.8448	1.82391	7.84824	12.3905	6.57199	0.2583	-0.67184
ENSG0000	TSGA10	2:9899726	3.33444	9.14184	9.04773	8.47913	8.39823	0.910429	1.44219	2.28994	1.22208	6.96813	0.03725	-1.7371
ENSG0000	NFKBIA	14:354015	368.326	198.094	354.351	1888.61	296.703	279.545	309.498	543.561	833.246	299.441	0.26355	-0.51343
ENSG0000	AC016757	2:2382245	0.746222	0.4697	1.22004	0.722001	0.153327	0	2.56975	0	0	0.681503	0.7697	-0.32241
ENSG0000	ECE2	3:1842496	4.16268	4.42389	5.86526	6.6466	5.39229	3.00101	3.43311	2.35678	0.712764	1.94713	0.07215	-1.31277
ENSG0000	RHOA	3:4935914	403.525	427.534	258.436	256.983	267.44	456.149	466.532	313.117	281.677	202.365	0.84525	0.076516
ENSG0000	MIATNB	22:266464	13.4679	7.86755	12.3026	20.2136	7.13878	19.84	14.0538	14.0907	27.718	6.36558	0.6142	0.443607
ENSG0000	APIP	11:348530	16.8265	13.983	15.1289	16.8614	11.2345	28.7437	15.7948	19.3672	10.3689	4.05843	0.91225	0.06407
ENSG0000	TMEM238	19:553792	19.2533	11.785	10.985	10.5733	19.2777	22.1309	28.4189	7.61416	5.3947	4.18214	0.58475	-0.3758
ENSG0000	IGLC2	22:229009	0	299.822	15.2708	1244.06	35.0318	0	0.196707	9.76415	6.38727	31.7909	0.00005	-4.59273
ENSG0000	BCORL1	X:1299811	0.526433	0.377424	0.307054	0.44649	1.87018	0.353446	0.512043	0.080838	0.664932	2.81351	0.8431	0.156121
ENSG0000	CETN2	X:1528269	20.4375	24.8827	19.7851	24.7255	14.0088	12.4275	11.6377	12.4982	19.9995	4.28541	0.14015	-0.6919
ENSG0000	NSL1	1:2127261	46.127	34.756	32.479	29.167	28.3021	54.629	46.4556	29.0248	30.4365	16.9575	0.8226	0.082354
ENSG0000	ABLIM1	10:114431	26.8295	78.189	26.4249	19.9348	31.0175	9.60359	11.9876	15.5916	18.818	42.2521	0.0525	-1.00713
ENSG0000	DNAJC10	2:1827160	25.5042	22.2515	5.2964	10.8133	7.41321	15.4719	16.8902	13.517	13.9264	11.0751	0.9879	-0.00603
ENSG0000	SEC24B	4:1093474	8.38908	12.5306	3.22134	3.24599	2.97739	3.94552	6.32772	6.81169	8.22434	5.95743	0.8596	0.073036
ENSG0000	FAM78A	9:1312580	14.9962	13.917	16.456	8.35187	22.3564	9.47283	12.2727	13.5896	6.44287	32.6218	0.6336	-0.18348
ENSG0000	ZNF781	19:374971	2.47777	3.07088	0.520836	1.04619	0.285936	0	0	0	0	0.312255	0.01415	-5.20724
ENSG0000	KSR1	17:274567	3.43118	3.32105	0.916697	0.83949	2.29527	1.45474	5.95566	4.20035	1.07369	5.84746	0.304	0.700036
ENSG0000	DCTPP1	16:304072	52.1829	28.7054	71.6327	50.7025	59.3609	56.5809	82.2198	63.3635	33.8603	19.1679	0.7567	-0.11962
ENSG0000	REEP2	5:1384390	0	0.08146	0.197414	0	0.123009	0	1.45241	0.490643	0	0.592241	0.04405	2.98588
ENSG0000	RP11-213	9:8580535	3.25817	3.62427	0.256409	0.216396	1.6144	2.08511	1.09984	0.138612	0.036047	2.3658	0.6387	-0.6777
ENSG0000	NDUFAF5	20:137849	9.88002	6.6633	9.32199	9.40896	5.0512	8.01701	9.41286	12.7067	9.85496	3.87331	0.8443	0.101929
ENSG0000	GSK3A	19:422301	8.52742	3.84506	3.84228	0.941291	10.5176	3.49848	4.82938	8.00166	1.84529	8.72768	0.9563	-0.03837
ENSG0000	GTF2H3	12:123602	7.71121	4.83482	5.78623	5.8159	5.53093	9.10706	4.48236	3.98203	4.5373	6.28348	0.6792	-0.37999
ENSG0000	C19orf57	19:138823	0.086927	1.65564	0.51028	0.869113	0.208721	1.79195	0	0	0.406105	0	0.8152	-0.26767

ENSG0000(	TRIB2	2:1271688	72.302	97.1055	23.8248	18.0524	25.2353	28.477	37.8852	33.7509	31.8231	51.2039	0.62895	-0.18161
ENSG0000(	DOCK9-A	13:990878	1.33055	2.55403	1.48716	2.27206	1.96304	2.41338	3.23182	2.48796	3.22333	1.15173	0.52665	0.391486
ENSG0000(	RINT1	7:1055320	17.9636	7.70181	8.05782	12.7104	6.82276	19.8778	13.4557	8.19388	5.19199	14.0987	0.797	0.106768
ENSG0000(	IRF4	6:391738-	2.23499	6.83223	0.36829	1.64938	1.32234	8.94831	17.8066	36.7421	15.2483	13.4943	0.00055	3.08147
ENSG0000(	PITPNC1	17:673772	83.5942	66.6657	133.992	95.0405	109.168	61.1616	88.5454	90.4677	39.1759	90.2067	0.2063	-0.52401
ENSG0000(	RN7SL172	3:1216313	0.700164	0.322513	0	0	0.390686	0.444789	2.17686	0.254548	4.47075	0.739653	0.1701	2.68663
ENSG0000(	AC147651	7:549196-	8.12803	5.12073	5.04484	5.44885	2.85083	21.6048	12.8148	12.4833	21.1394	6.87039	0.30395	1.55549
ENSG0000(	DKK3	11:119631	4.04283	0	10.2726	8.85074	2.48169	5.78176	0	19.4081	29.5974	8.35413	0.1217	1.22904
ENSG0000(	AP2B1	17:355747	22.6537	20.2269	8.975	10.0014	15.9969	28.1287	14.2352	20.9868	12.7861	22.2332	0.65675	0.191578
ENSG0000(	NF1	17:310084	4.117	9.95426	3.68592	3.992	4.71181	1.79947	6.26227	4.57644	8.39922	9.34937	0.6417	0.312322
ENSG0000(	AC004951	7:4392643	0	0.734474	0.447175	0.151066	0.49847	0.461292	0.07855	0.081491	0	0.159747	0.34615	-1.52027
ENSG0000(	STON1	2:4852938	0.605455	0.622244	0.160647	0.406568	0.397488	0.587205	0.019422	0.016814	1.4842	1.0419	0.7598	0.485515
ENSG0000(	LINC0113(	1:1483629	9.06041	10.5148	8.17996	4.58942	5.68174	6.75832	8.94345	11.3476	9.39139	9.26764	0.6532	0.310284
ENSG0000(	PXDC1	6:3722613	0.305485	0	0	0.819132	0.144689	6.40269	0	0.303381	2.28896	0.171996	0.1719	2.88058
ENSG0000(	CKAP2	13:524554	14.4605	24.168	4.63533	8.48594	10.3876	10.4961	17.9868	6.0994	8.54203	20.1658	0.9045	-0.05481
ENSG0000(	B4GALNT3	12:460363	3.16232	0.661544	0	0.009474	3.24776	5.9662	0.532082	0	0	6.04801	0.68945	0.641097
ENSG0000(	RP11-234.	3:1015740	7.89838	7.59249	4.83005	4.4681	1.27096	6.05616	7.02577	1.7495	2.60502	0.60951	0.6645	-0.54018
ENSG0000(	HDAC3	5:1415150	39.5507	41.2783	51.234	49.2578	45.6583	37.5656	40.1175	40.7505	45.9751	50.8349	0.9668	-0.02181
ENSG0000(	TRIP11	14:919659	38.5529	36.668	17.6534	17.2706	22.897	49.3063	41.4691	8.70072	32.9903	33.6718	0.992	-0.00833
ENSG0000(	CTC-463A	5:1421657	0.400736	1.24548	0.444054	0	0.189654	0.688978	0.847709	0.642625	0	0.886114	0.6725	0.411458
ENSG0000(	SELENOI	2:2630817	2.54082	1.87962	0.742045	1.74941	0.485531	4.22385	2.1297	1.00532	0.516912	1.96649	0.7099	0.348551
ENSG0000(	TMTC2	12:826868	0.375492	0.641134	0.840543	0.624223	0.536316	0	0	0.16691	0	1.2673	0.4471	-1.24922
ENSG0000(	ATP5J2-P	7:9932589	0.251483	0	0.577388	0.143509	0.483048	0.479361	2.76634	0.286398	0.017051	0.696998	0.714	-0.82787
ENSG0000(	CERK	22:466844	87.5791	38.3422	45.4776	48.8835	44.5021	58.6781	26.5455	35.0603	35.5463	58.8084	0.2123	-0.41295
ENSG0000(	PRKAA1	5:4075937	22.6238	25.1566	12.6378	29.1486	13.6701	9.78534	16.9021	19.2048	27.1795	17.717	0.8141	-0.14847
ENSG0000(	FAM234B	12:130442	0.398775	0.567287	0.915655	1.54558	1.43061	0.243465	0.395142	0.49831	0.050576	2.50544	0.32645	-0.84652
ENSG0000(	RP5-1112	6:1112779	0	0.478388	0	0	0.268485	0.209976	0.269531	0.796075	1.96158	0.850652	0.18415	2.5635
ENSG0000(	LINC0105	1:9461381	0.623271	0	0.804902	0.844361	0.059073	0.011402	0	2.91693	0.683535	2.05506	0.54625	0.739012
ENSG0000(	PTBP2	1:9672166	8.10268	5.25282	2.61703	0.814439	1.59067	4.97041	5.78674	6.5244	4.48913	6.7988	0.17325	0.750475
ENSG0000(	TEX10	9:1003020	13.7804	14.425	7.96538	8.64079	6.23032	7.71867	7.37346	10.4881	4.40095	10.4508	0.44425	-0.31374
ENSG0000(	ARMCX7P	X:1015975	14.6027	13.4424	19.0596	22.1578	18.5176	31.0271	27.3146	23.978	33.99	12.5203	0.54495	0.968936
ENSG0000(	TBC1D32	6:1210794	2.07154	2.54934	0.712574	1.15039	0.704172	0.63166	1.33046	0.756346	6.4447	2.00271	0.4596	0.638803
ENSG0000(	CBX8	17:797921	1.31452	2.63269	1.35713	1.31826	2.66284	6.59057	7.91645	0.492865	1.6165	1.35891	0.529	0.520347
ENSG0000(	CHD9	16:530550	63.5846	50.7498	40.9162	52.2085	27.5147	62.7371	46.388	19.8272	12.6024	44.58	0.34965	-0.41753
ENSG0000(	MRPL41	9:1375511	59.6579	46.7973	86.4751	69.8021	100.193	148.739	135.186	129.02	72.1643	21.3284	0.3734	0.305076
ENSG0000(	AGFG1	2:2274721	25.8501	21.0796	6.85449	3.22205	5.72914	20.1243	22.5781	8.08026	3.37463	12.7587	0.83975	-0.11349
ENSG0000(	FLJ20021	4:1010234	2.51807	2.3417	4.88639	4.26076	2.289	1.88589	7.06137	4.5671	0	1.61964	0.79835	-0.36788
ENSG0000(	RP11-820	18:116889	0.202433	0.824861	1.37833	0.879808	0.810755	0	0	0.409784	0.774042	0	0.2601	-2.02015
ENSG0000(	RAB1A	2:6505636	49.3238	49.0897	58.4951	82.1658	56.0656	95.1729	70.8061	49.544	47.1088	51.8225	0.90095	-0.0538
ENSG0000(	PDK1	2:1724273	4.99813	14.3659	3.76734	5.77853	7.73273	0.960624	5.86317	6.78034	12.77	7.57711	0.7539	-0.21907
ENSG0000(	CDC27	17:471177	17.2358	15.9587	16.4114	13.1649	21.2893	18.9949	22.0005	13.4439	29.1389	45.1662	0.37725	0.371553
ENSG0000(	RBMX2	X:1304019	8.99213	11.75	20.7119	18.6211	27.9093	21.48	23.3722	14.4484	15.6598	12.368	0.99245	-0.00636
ENSG0000(	PELI3	11:664350	2.06514	4.89921	0.710237	0.225396	1.65269	0	2.31631	2.16229	0	3.13465	0.8202	-0.28636
ENSG0000(	SBNO2	19:110763	1.86801	1.81708	0.536626	0.460536	2.54807	2.99435	3.19652	3.39123	1.54377	4.42955	0.0891	1.03798
ENSG0000(	DET1	15:884594	1.42556	3.49177	1.18819	0.341894	0.95092	4.74341	0.265371	0.652662	2.04592	0.513786	0.94075	0.078336
ENSG0000(	UPF3B	X:1198053	22.1011	29.7579	22.6776	21.2267	34.9255	27.4513	21.854	15.76	23.2395	31.6346	0.76295	-0.11906
ENSG0000(	KIAA0355	19:342545	1.3673	4.46547	2.14475	1.91559	1.26866	2.13338	0.791685	3.51112	0.797601	4.10903	0.73965	-0.17616
ENSG0000(	TRIM24	7:1384603	5.19399	10.8754	1.81034	2.08325	2.58769	6.17064	1.71555	4.99902	0.415364	4.57186	0.27735	-0.64079
ENSG0000(	SLC23A2	20:485235	4.68772	4.56898	2.04592	1.9312	2.48381	4.03735	3.07811	1.43591	0.593405	6.70694	0.6103	-0.23236
ENSG0000(	ARMC9	2:2311985	0.488365	2.23663	0	0	0.296505	0	0	0.906886	3.25226	0.157759	0.2475	1.43591
ENSG0000(	C1orf27	1:1863758	8.2986	9.43722	5.88355	4.72448	4.8222	14.2019	3.81786	4.46801	1.38244	6.87111	0.48625	-0.34642
ENSG0000(	AC113188	8:1181894	0.831568	0	6.39571	5.11858	3.15494	0	1.6558	3.01829	3.27589	5.15637	0.9122	-0.20812
ENSG0000(	MVP	16:298061	121.125	148.997	51.0771	81.9567	46.6754	155.97	151.269	65.7407	104.968	74.2863	0.6297	0.224783
ENSG0000(	CD8B	2:8681533	36.6152	269.979	114.886	97.2574	111.507	117.291	484.719	465.326	194.681	111.107	0.00335	1.12584
ENSG0000(	RP11-46H	12:132768	0.935172	1.39144	0.177025	0	0.093848	0.23097	1.48319	0	0	1.68722	0.7923	0.539947
ENSG0000(	SLC44A1	9:1052446	1.26916	3.31945	0.49347	0.811974	0.809282	2.06166	0.614793	0.427411	0.76836	1.8439	0.33985	-0.61473
ENSG0000(	ATP6AP1	X:1544286	84.7927	68.0841	135.284	145.853	120.681	102.459	68.8296	107.031	83.716	112.269	0.3275	-0.30766
ENSG0000(	BRMS1	11:663302	48.3308	50.2886	72.8931	86.6047	58.6808	51.6658	68.0581	66.7603	29.202	25.7236	0.2139	-0.45344
ENSG0000(	GPANK1	6:3166122	18.916	18.5561	35.1528	34.4201	42.3868	19.451	17.4627	21.3918	23.3749	22.0935	0.282	-0.6607
ENSG0000(	SRSF8	11:950671	34.6912	34.4255	47.7054	35.7054	36.0432	31.9059	22.7155	32.7049	23.2692	28.4728	0.12665	-0.49186
ENSG0000(	HIST4H4	12:147679	9.46957	7.98671	6.02569	8.61809	0.93628	0	2.13189	6.23699	2.3145	1.12788	0.13945	-1.6822
ENSG0000(	NAXD	13:110613	11.3612	26.0581	14.03	12.6928	7.51029	12.5048	21.0006	14.9665	2.549	15.785	0.7936	-0.11868
ENSG0000(	AGAP1	2:2354940	0.897255	0.370106	2.31025	2.48197	5.68099	1.42426	0	0.596935	0.021178	5.26493	0.22295	-1.37702
ENSG0000(	SIRPA	20:188812	0.278581	0.43528	0.174131	0.022156	0.083026	5.50272	0	0.120872	0.387564	0.075437	0.16	2.72051
ENSG0000(	IL1RN	2:1131072	1.79114	2.87058	0.748985	1.72382	1.9134	27.8579	24.4077	51.7223	8.12988	8.47078	0.00005	3.75024
ENSG0000(	PDSS2	6:1071525	7.6458	8.23365	11.4213	13.6378	9.1004	1.91422	8.65838	6.88922	16.6731	7.18974	0.60425	-0.29823
ENSG0000(	STRBP	9:1231094	13.9133	15.8845	6.97009	5.09884	8.87953	18.1928	15.2901	12.8574	7.92285	17.6631	0.34535	0.38108
ENSG0000(	RCC2	1:1740675	28.3718	32.6922	11.7492	10.4473	16.7818	27.9075	25.983	17.467	8.02477	28.0787	0.70815	0.130609
ENSG0000(	SCARNA1:	14:955322	2.92983	6.4176	5.22941	4.14683	1.41924	2.39042	5.09795	2.92313	5.15553	2.28662	0.91605	0.122663
ENSG0000(	ATG13	11:466028	41.5761	43.2791	34.3565	32.5555	26.7707	40.7571	29.864	29.8816	27.172	31.8528	0.3677	-0.27943
ENSG0000(	TPX2	20:317392	7.70995	19.3438	0.212194	0	0.857747	15.1486	9.90582	2.57679	6.07231	4.32668	0.3351	0.639412
ENSG0000(	UVSSA	4:1347265	2.35605	3.97348	1.94389	2.17055	1.45338	2.4617	4.10481	0.131772	2.44878	2.09655	0.76275	-0.20561
ENSG0000(	CCDC171	9:1555289	0.381832	0.241092	0.099194	0	0.36802	0.939532	1.49021	0.196168	0	0.414581	0.1222	1.56969
ENSG0000(	TBCE	1:2353673	12.4966	9.11237	11.8558	11.756	9.71866	18.2884	12.8365	10.7148	4.32958	6.09083	0.7981	-0.13061
ENSG0000(	NUP50-AS	22:451330	2.07754	1.57243	6.09846	6.68571	4.57775	5.39015	5.08569	6.86016	5.83624	2.4075	0.92705	0.08461
ENSG0000(	OSER1-AS	20:442109	8.56127	3.85833	8.82089	23.693	6.67539	8.82841	0.33453	7.36916	11.6811	1.53259	0.203	-0.74901
ENSG0000(	XYLT1	16:171017	1.04274	4.19303	0.326621	0.061136	0.730796	4.53583	3.13491	0.818399	0.521202	2.10903	0.32395	0.719809
ENSG0000(	RAB30-AS	11:830720	14.3656	20.2819	15.6993	13.4086	5.58396	27.8691	8.33919	8.34201	21.281	10.5072	0.7863	0.149495
ENSG0000(	CABP4	11:674513	0.742813	0.647295	0.112142	0.794877	0.108178	0	0.262319	0.020678	0.840288	0	0.43085	-0.80236
ENSG0000(	SRPRA	11:126202	104.448	91.573	82.2681	58.3416	62.3642	100.838	55.5094	72.0678	37.7813	74.6803	0.505	-0.2488
ENSG0000(	MBNL1-A	3:1522438	0.281611	0.457941	0.418848	0.648317	0.263833	0.227402	0.558496	0.309598	1.99632	1.04546	0.6861	-0.59405
ENSG0000(	SUDS3	12:118376	9.55187	8.75254	6.28767	2.34026	9.1216	6.64703	9.55197	7.54149	5.90191	10.9831	0.7176	0.141372
ENSG0000(	RPL28	19:553853	167.404	181.468	258.654	177.895	170.084	164.618	216.07	242.713	151.123	31.4342	0.508	-0.32082
ENSG0000(	GALNT8	12:456220	0.576917	0.982324	0.337794	0.398	0.232971	0	0	0.084046	0	0.12069	0.07195	-3.11908
ENSG0000(	SHQ1	3:7274927	13.6999	17.3788	9.11273	11.585	9.39872	9.22658	12.6846	5.41687	3.48896	7.80897	0.1177	-0.62513
ENSG0000(	TRMT12	8:1244508	6.6437	4.37198	7.50975	4.73615	5.84472	12.1292	4.93179	2.57862	5.64985	5.23749	0.9105	0.070826
ENSG0000(	COL9A2	1:4030048	2.15644	7.67154	0	0.246757	0.25291	5.40366	18.4012	0.94556	0	0.104926	0.37335	1.14147
ENSG0000(	GRIPAP1	X:4897371	32.5358	40.3905	16.3745	15.715	24.9043	35.8693	35.9954	26.429	25.6132	40.1087	0.26915	0.381598
ENSG0000(	PDDC1	11:767219	4.66062	6.87038	5.52428	4.37701	6.78969	4.50653	6.15363	6.52206	5.41791	8.96522	0.9242	0.04137
ENSG0000(	PIGO	9:3508868	16.2577	13.956	7.95482	9.45467	5.07741	13.9015	10.9507	4.96361	6.66111	10.8136	0.9079	-0.04877
ENSG0000(	CD226	18:694010	13.541	17.9203	11.5592	3.49811	8.29388	21.5185	47.2661	18.9006	11.3245	27.3555	0.03005	1.14731

ENSG0000C	R3HCC1L	10:981346	16.1992	16.9998	8.61264	11.5612	4.52342	6.13464	14.9561	9.27169	16.5643	11.0659	0.54445	0.30342
ENSG0000C	HPSE	4:8329246	2.62603	4.31417	0.961682	1.26815	1.07891	12.1172	9.25181	3.63248	3.1711	4.66071	0.08635	1.52143
ENSG0000C	RP11-77K	16:752936	0.54332	0.136987	0.776127	0.37126	0.435285	0.763782	0.339583	0.951617	3.00199	0.275688	0.24785	1.96427
ENSG0000C	XX-FW835	X:1543899	6.10354	4.42172	4.20639	4.91843	2.81644	1.42643	2.50951	0.629287	3.17846	2.96893	0.39155	-1.06847
ENSG0000C	GIMAP2	7:1506856	77.0429	56.5693	80.37	49.9512	59.5053	56.6223	43.9995	55.1463	53.0827	55.0138	0.32105	-0.31543
ENSG0000C	AP000704	21:367508	0.151562	0	0.017682	0	0.232947	0.761453	0.697365	0	1.08355	0.603853	0.1573	2.96048
ENSG0000C	KBTBD3	11:106051	8.9877	6.03341	9.30912	5.16141	2.96035	12.1626	11.428	6.26734	6.44109	1.56256	0.871	0.143968
ENSG0000C	RP11-302	12:489034	0	0	0.643567	0	1.34952	0	0.157315	0.265843	0	0	0.4029	-2.63387
ENSG0000C	PRKCZ	1:2050469	15.1729	10.4681	14.6545	12.3394	18.6566	13.2522	11.4325	17.2656	8.14953	14.5087	0.6902	-0.2248
ENSG0000C	CTD-2210	5:4357159	0.655615	0.430757	0.486826	0.03818	0.184683	1.92023	0	0.642534	0	1.38491	0.49215	1.13924
ENSG0000C	TRAPPC12	2:3379674	1.7507	2.33828	0.485032	0.466148	0.525511	3.57813	2.43675	1.59009	0.348339	0.408557	0.7039	0.554061
ENSG0000C	PSMD9	12:121888	37.3678	33.3783	74.7286	65.451	58.8338	68.1393	71.5098	77.0249	49.8384	28.0836	0.82385	-0.10703
ENSG0000C	ECSIT	19:114513	17.7759	26.1789	32.8981	25.8342	30.6315	26.0398	10.1179	21.865	10.5574	13.9922	0.0743	-0.7196
ENSG0000C	LRRC37A3	17:648543	2.72746	1.37982	0.91935	1.78789	1.06047	1.57413	2.38879	0.28466	0.973122	0.950564	0.7364	-0.26264
ENSG0000C	ZNF578	19:524535	1.98569	1.81872	0.442852	5.58275	4.03537	0.042621	0.115642	0.976712	3.68041	2.1973	0.4773	-0.89996
ENSG0000C	KCNAB2	1:5862810	24.551	15.4146	12.8557	9.50053	23.0471	20.7115	11.6725	19.9106	11.6399	34.2695	0.66495	0.150565
ENSG0000C	WDR92	2:6812293	9.56065	11.1718	5.70203	5.92154	7.52998	3.30267	6.05908	5.18099	4.65811	6.51381	0.48135	-0.52235
ENSG0000C	TRIM3	11:644861	2.69259	4.84846	1.15312	0.964654	2.34782	3.86308	3.99836	2.37561	1.65941	3.12815	0.5903	0.375279
ENSG0000C	SMPD1	11:639043	8.25743	11.5179	8.49028	10.1108	13.1283	14.8229	14.1871	11.8972	0.253717	23.6383	0.52455	0.39592
ENSG0000C	LAMA5	20:623020	0.442763	1.87706	0.226811	0.442242	0.340214	0.079259	1.19374	0.339307	2.66149	0.302976	0.5366	0.738624
ENSG0000C	IGHV3-48	14:106537	0	117.527	0	0.349467	0.353628	0	0	0	0	0.217138	0.1666	-8.21316
ENSG0000C	LRRC39	1:1001331	0.195359	0.542545	0.130744	0.085384	0.051778	0.238939	0.300274	0.115077	1.06452	0.671295	0.3734	1.24805
ENSG0000C	CRMP1	4:5711196	0	0.216222	0.047164	0	0.010109	0.596841	0.149406	3.06115	0	0.679273	0.0298	4.34719
ENSG0000C	TIGAR	12:430776	8.39815	6.35172	8.65059	9.17964	5.98134	11.4439	10.0028	11.1534	7.91349	4.42866	0.5765	0.238136
ENSG0000C	LYST	1:2356610	20.6659	14.2847	15.4887	27.1326	13.7626	25.1841	21.0477	24.7527	26.1899	23.1	0.57935	0.243897
ENSG0000C	CRBN	3:3126915	121.658	102.15	117.955	99.6148	81.4308	90.9955	85.0324	50.5416	78.2306	51.2003	0.11915	-0.55516
ENSG0000C	PPIAP11	5:8197202	0.615751	0.480535	0	0.858478	0.094086	0.859487	0.629266	0.102056	0.128437	0	0.8686	-0.26832
ENSG0000C	CYP2U1	4:1078245	1.91761	4.7898	1.46578	1.03898	0.829702	0	0.308769	0.447398	0.068894	0.501599	0.0306	-2.86766
ENSG0000C	GSDMD	8:1435532	93.5333	78.5767	99.4442	113.887	105.819	91.1405	129.796	122.503	129.637	122.686	0.3541	0.320077
ENSG0000C	SLX1A-SU	16:301929	11.1981	21.6112	10.4796	9.29953	7.1749	16.0424	16.5378	12.1275	4.32588	17.9054	0.96885	0.047667
ENSG0000C	NIN	14:507197	23.4478	25.5891	8.62564	5.25449	12.425	9.26031	13.0215	9.85393	5.42765	22.0997	0.25185	-0.49169
ENSG0000C	NREP	5:1114960	1.16811	16.9864	1.68499	1.51729	0.464751	1.78011	3.38062	0.557982	0.056064	0.081071	0.0414	-1.7415
ENSG0000C	GPC3	X:1335357	0.444195	3.28431	0	0	0	0	0	0	0	0	0.0038	#NAME?
ENSG0000C	C4orf19	4:3745394	0.183063	0.020082	0.049627	0	0.022263	0.05288	1.14622	0.415804	0.36387	0.450413	0.0322	3.46738
ENSG0000C	LFNG	7:2512528	9.51866	14.5693	8.57269	3.96714	17.3885	4.82971	8.3604	22.1806	9.09144	21.1302	0.57075	0.2652
ENSG0000C	GCNT1	9:7641984	1.8742	1.91923	0.375925	0.419409	0.695375	7.52705	11.3087	7.98122	2.04797	3.51703	0.00055	2.69205
ENSG0000C	4-Sep	17:585198	0.726676	1.45407	1.20947	1.12046	0.298062	0	2.1762	0.031741	0	0	0.06385	-1.51262
ENSG0000C	DAPK2	15:639070	1.44033	1.69288	4.35469	2.44154	1.23573	8.50583	34.9556	27.1454	10.1441	21.9941	0.0001	3.07782
ENSG0000C	CLK4	5:1786026	59.4133	65.9283	26.2406	32.4587	18.3958	38.9398	39.6388	27.6177	22.1769	31.4082	0.45495	-0.2557
ENSG0000C	CLPP	19:634376	64.0095	50.6343	70.3652	80.8588	99.2793	75.8308	68.7488	74.8203	64.9802	44.2868	0.52325	-0.21982
ENSG0000C	SLC25A37	8:2352880	4.29024	5.95658	11.0698	11.1575	7.59387	3.95407	5.49647	9.87561	3.7924	11.0418	0.5921	-0.33103
ENSG0000C	CDKN2A	9:2180254	3.9308	5.63323	10.6204	19.3878	6.0234	28.626	14.8095	16.0368	12.7368	5.55298	0.55305	0.597969
ENSG0000C	AP001258	11:591301	8.74748	11.9007	13.5444	15.3729	12.1865	11.0667	10.3642	8.68665	3.62948	12.2658	0.4916	-0.50684
ENSG0000C	RP11-723	18:359720	0.306258	1.84401	0.543224	0.114099	0.381574	0.082173	0	0.034899	0	0.341874	0.2151	-2.8552
ENSG0000C	ZSCAN2	15:845978	1.88698	2.2787	4.85228	2.39392	5.3722	4.52099	3.40425	7.41546	0.996212	7.46555	0.56995	0.382289
ENSG0000C	AMMECR1	X:1101941	5.39798	3.48898	4.55097	4.81206	4.32037	7.97616	5.97116	4.737	4.47846	6.54439	0.74425	0.196491
ENSG0000C	LCOR	10:968322	11.9208	12.6544	5.72354	6.32373	6.74115	2.13648	11.079	4.39794	9.48629	9.75939	0.625	-0.28727
ENSG0000C	RP11-405	18:750735	3.55737	2.78649	3.6929	2.9664	2.54589	0.25413	1.05542	2.33577	4.86634	2.84719	0.69115	-0.31334
ENSG0000C	BRD4	19:152355	34.4497	63.3149	5.32832	2.95277	18.863	43.1582	62.159	9.46724	6.7298	33.2438	0.9629	0.024642
ENSG0000C	WBP1L	10:102743	7.15624	9.30922	7.33461	1.76828	7.93255	7.22851	7.04897	8.39106	5.35769	16.438	0.50225	0.289015
ENSG0000C	RWDD2A	6:8306766	1.06203	3.505	1.25823	0.992651	1.4981	0	0.671846	0.546002	2.46351	1.5768	0.69935	-0.62656
ENSG0000C	ASL	7:6607579	12.9493	14.6681	20.797	23.8802	19.8233	16.0139	14.1906	12.4277	12.741	6.45526	0.26885	-0.65061
ENSG0000C	ESPL1	12:532682	0.656454	1.48526	0	0	0.024275	1.80191	0.229999	0.130589	0	1.77122	0.4195	1.08816
ENSG0000C	SLC31A1	9:1132215	2.85323	2.55521	4.59547	1.1524	3.53088	12.6562	2.78674	0.804854	1.20781	3.05405	0.75225	0.310555
ENSG0000C	RAB10	2:2603410	73.3336	70.6707	36.4685	29.0863	36.1247	85.1565	73.1848	37.947	29.2299	43.9009	0.8357	0.077173
ENSG0000C	IQSEC1	3:1289721	23.0756	28.1451	9.51828	8.09716	17.2217	16.0654	10.9585	10.904	4.72367	23.2048	0.23515	-0.44635
ENSG0000C	GAREM2	2:2617309	2.09664	1.49262	0.915319	2.13777	0.824444	2.01079	1.80927	1.69668	0.017873	3.97095	0.70675	0.317862
ENSG0000C	RP11-285	10:448110	1.37469	1.24923	0.519945	0.803746	0.768809	0	1.42535	1.05817	0	1.08429	0.8734	-0.30662
ENSG0000C	BCL9	1:1475414	1.41939	2.40975	0.044825	0.47192	0.72585	0.590441	0.702122	1.16673	2.12	0.991022	0.607	0.406106
ENSG0000C	DIRAS3	1:6770146	0	0.469554	0.021814	0	0.295724	0	4.19006	0	0	0	0.44765	2.36645
ENSG0000C	LINC0156	X:4748357	0.764851	2.45099	1.22191	0	0.553318	1.58396	0.622745	2.58635	0	1.90711	0.8004	0.440374
ENSG0000C	MIER3	5:5690004	3.68607	2.56965	0.750017	1.18875	1.45042	3.04659	4.70721	1.6688	0	3.45315	0.5957	0.342258
ENSG0000C	ACSL3	2:2228609	19.2804	13.5761	16.7939	14.7409	7.37827	18.7756	13.7997	12.3941	5.518	12.5571	0.38695	-0.35463
ENSG0000C	MAP7D3	X:1362132	6.50604	3.6512	8.09652	4.24522	9.73877	3.86462	10.4415	3.3668	2.96851	15.0012	0.9739	-0.0166
ENSG0000C	MPHOSPH	2:7113031	29.1832	37.6782	33.2091	29.1303	42.4002	48.5599	34.6825	35.7122	29.3673	42.4834	0.75205	0.107949
ENSG0000C	CRKL	22:209174	18.3231	21.7564	13.8671	9.22894	14.9367	20.5418	14.1738	16.785	7.95455	24.9215	0.97105	0.01275
ENSG0000C	PDCD7	15:651173	19.2541	22.9882	24.4661	26.6307	42.4628	19.4918	21.4175	34.98	22.5434	32.8669	0.88115	-0.05249
ENSG0000C	CD19	16:288784	0.072563	15.709	2.57999	5.59011	1.32433	0	0	0	0	0.177338	0.00065	-7.20148
ENSG0000C	CDK5RAP	20:333588	28.3679	26.0512	41.1158	42.9172	29.3288	41.5136	22.9459	43.7972	58.4007	32.6755	0.39645	0.3516
ENSG0000C	AC005523	19:479168	9.07572	9.67595	7.07717	4.61295	6.80158	14.699	13.5058	6.71663	2.64082	14.018	0.73635	0.141116
ENSG0000C	SAV1	14:504185	5.61863	7.70273	2.28465	1.15258	2.90965	2.94206	4.62676	1.14565	0.194179	4.29492	0.4657	-0.6916
ENSG0000C	DUSP10	1:2217014	15.2424	15.3939	14.9625	15.4279	11.1626	43.4237	23.5619	17.4545	36.7979	16.5011	0.0321	0.87458
ENSG0000C	VWA9	15:655787	21.0143	21.7423	24.1552	21.1874	17.0767	12.3131	17.9784	21.2106	15.5185	25.9914	0.44525	-0.26619
ENSG0000C	C1GALT1	7:7156933	48.4305	27.4958	29.9877	40.5948	52.5261	70.6999	55.9376	30.3671	26.6424	43.3512	0.877	-0.07892
ENSG0000C	MEST	7:1304860	0.858087	4.34531	2.78998	1.82632	2.13893	0	0.025203	1.73523	0.060236	1.02204	0.1183	-1.80651
ENSG0000C	METTL14	4:1186853	15.8274	11.8293	15.7172	9.77073	13.1193	24.5295	15.051	16.9996	18.871	24.3513	0.659	0.30785
ENSG0000C	TSPAN3	15:770414	20.5506	37.9724	22.6715	21.8481	21.859	21.7662	10.3929	8.04752	22.2705	7.92206	0.0654	-0.7968
ENSG0000C	PLEKHM3	2:2078212	2.20397	2.51454	1.06985	1.63886	0.553858	1.98809	1.29898	0.727983	5.72484	1.75479	0.4952	0.484523
ENSG0000C	CTD-3184	20:636272	12.853	24.9325	25.263	19.6924	13.6232	10.3354	13.5417	14.4236	17.3738	13.867	0.56255	-0.41084
ENSG0000C	ENDOG	9:1288184	12.6602	18.8353	28.3832	17.1578	47.2064	20.9895	36.4924	17.8791	17.5848	13.6224	0.67025	-0.39151
ENSG0000C	PANK3	5:1685484	2.48274	2.36464	0.734763	0.741423	1.44709	2.2053	3.78319	2.9355	1.17011	3.81917	0.0541	0.904909
ENSG0000C	APBB3	5:1405582	12.2673	16.042	8.65308	8.26229	5.37452	23.2247	7.23881	5.44336	3.05868	10.6998	0.958	-0.03544
ENSG0000C	LMF2	22:505029	35.208	38.428	17.7134	18.2745	24.7358	33.0655	36.5517	36.4173	16.0013	40.5476	0.50575	0.228923
ENSG0000C	CMC2	16:809664	80.0018	41.8832	88.9077	85.0974	39.3079	145.58	186.165	149.652	141.912	26.4182	0.0527	0.946689
ENSG0000C	FLJ13224	12:312804	2.48338	0.785727	1.0237	0.258572	1.26599	2.35588	0	0	0	0	0.49035	-1.56362
ENSG0000C	ZNF286A	17:156995	7.02312	6.87034	7.37157	3.15905	4.12683	3.16452	6.23969	6.72392	18.5498	8.76345	0.4034	0.560777
ENSG0000C	POLRMT	19:617223	30.2344	23.7169	8.82214	10.5855	20.3129	17.9356	28.4872	20.335	12.7311	31.9255	0.3881	0.292006
ENSG0000C	RILPL2	12:123410	6.24838	12.187	7.89952	7.2399	6.31058	10.1609	14.8664	12.5013	8.53443	5.73961	0.3789	0.322894
ENSG0000C	PLPP1	5:5530775	3.96698	5.52156	4.04025	4.42471	2.17005	25.4782	34.3335	24.9645	9.54599	13.8676	0.0522	2.35135
ENSG0000C	NUBPL	14:314899	3.16551	3.22676	3.6968	7.18351	4.6044	2.05878	1.95745	6.08736	4.17874	4.08366	0.60565	-0.34332
ENSG0000C	RNF169	11:747488	2.14692	1.97568	1.91108	0.829242	2.69079	0.768394	2.37262	1.72602	1.08015	2.95315	0.69835	-0.35256

ENSG0000(	LRRC2	3:4651542	0.316714	1.00382	0	0	0	0	0.788126	1.79809	0.893162	0.68774	0.26925	1.94296
ENSG0000(	AL928654.	14:105486	0.605949	0.823131	0.180396	0.18505	0.247766	0.061502	0.519227	0	0	0	0.2799	-2.25498
ENSG0000(	EPDR1	7:3768379	0.50492	2.07231	0.671125	1.34568	0.126556	0	0	0	0	0	0.12395	#NAME?
ENSG0000(	AC005795	19:415061	1.00396	0.61695	0.280572	0.95876	0.074792	0.603357	0.977375	0.08159	0.093457	0.184389	0.67855	-0.72589
ENSG0000(	RPL39	X:1197865	537.781	371.747	771.603	517.598	212.496	503.798	508.34	524.289	299.004	33.0129	0.21125	-0.43513
ENSG0000(	RP11-162.	18:767947	1.00426	1.34907	1.38933	0.198131	1.10736	2.27399	3.01192	0.846377	0	2.93738	0.6746	0.836166
ENSG0000(	KLHL20	1:1737149	12.132	12.8081	10.5496	7.26765	7.26279	16.2771	13.6649	12.7922	1.81376	14.6825	0.7124	0.195229
ENSG0000(	ESRRA	11:643055	18.6305	16.9864	6.82693	3.7896	14.0522	23.1251	26.2688	8.54728	6.40547	26.9452	0.6492	0.527333
ENSG0000(	RP11-456I	1:1617662	0.585244	0	0	1.22032	0.124879	0	0	0	0	0.035436	0.22925	-6.03084
ENSG0000(	NARS	18:576006	70.6277	87.941	56.9172	56.0043	34.011	86.5551	73.5722	56.2243	51.4127	41.1584	0.96995	0.012173
ENSG0000(	DDX60	4:1682162	14.3758	11.2073	5.19986	5.36158	5.08151	7.60977	21.5983	9.96867	4.08903	9.07654	0.1366	0.605081
ENSG0000(	LILRA4	19:543070	0	0	0	0	0.114715	0	0	3.87326	0	0	0.15295	5.10641
ENSG0000(	ARL16	17:816811	52.3267	48.3852	108.327	149.913	53.1452	75.9663	50.2513	64.9353	93.0199	84.9093	0.69275	-0.22333
ENSG0000(	RANBP1	22:200802	116.573	129.273	166.277	144.142	165.857	249.8	226.308	185.081	153.522	70.8963	0.733	0.191226
ENSG0000(	LEMD3	12:651695	15.5334	17.5866	19.8145	36.968	21.3246	3.2587	8.04029	14.052	25.4502	30.9009	0.5918	-0.51454
ENSG0000(	SKP1P1	7:6620519	1.83812	0.202212	0.568331	0.753621	0.36283	1.52669	2.19498	0.789245	0	2.03128	0.5151	0.991333
ENSG0000(	RHBDF2	17:764708	46.1561	28.3476	15.9254	18.8363	19.8388	38.3582	27.0322	18.1001	6.76353	23.4912	0.4358	-0.27258
ENSG0000(	FAM60A	12:312804	35.3542	46.8024	23.325	15.2113	28.9303	29.8542	49.2984	28.9762	18.9788	32.0556	0.66535	0.173122
ENSG0000(	SNED1	2:2409988	1.15051	4.36502	0.076224	0.326316	0.234748	0	2.05388	2.00378	0.594237	0.994169	0.96005	0.055825
ENSG0000(	KAT5	11:657119	27.9334	35.847	35.8802	41.9917	44.9506	24.1047	33.8127	44.8363	28.8358	33.6213	0.61065	-0.2549
ENSG0000(	UPK3BL	7:1024824	11.211	24.1702	24.6673	20.7834	18.5614	16.2123	13.4829	20.1012	22.2757	24.353	0.80725	-0.14465
ENSG0000(	LINC0082	8:1284052	0.411822	0	0.084289	0	0	0	1.05773	1.48236	0	0	0.2727	2.70142
ENSG0000(	WIPI2	7:5190187	29.6135	29.5661	29.6505	62.6345	42.5757	11.4377	26.1103	33.5154	25.3131	40.0464	0.11285	-0.64485
ENSG0000(	SEC24C	10:737443	38.4801	27.3872	17.1253	21.4659	18.013	17.1545	14.6886	15.8814	12.4498	21.2145	0.07365	-0.77281
ENSG0000(	SNRPEP4	19:557665	2.09144	2.14314	2.68809	6.0857	0.965055	3.14237	2.3054	3.05889	3.10713	0	0.9225	-0.18156
ENSG0000(	TESPA1	12:549480	28.1532	70.1279	28.1302	33.7077	33.1998	19.8645	16.2048	22.6114	30.4413	48.7714	0.1885	-0.46624
ENSG0000(	ZNF785	16:305693	4.70051	5.51672	3.96697	1.65219	2.43371	3.15608	2.73902	2.16	1.896	7.67681	0.979	0.030442
ENSG0000(	IGKV3-15	2:8908517	0	8.4081	0.590189	3.40674	1.92276	0	0	0	0	1.07568	0.16195	-3.03994
ENSG0000(	SPECC1L	22:242708	9.14476	14.7109	2.21651	2.88313	8.33368	10.5761	8.25621	1.85122	1.82392	9.46196	0.6267	-0.48483
ENSG0000(	IGLV10-5	22:222147	0	0	0	0	4.14813	0	0	0	0	17.9163	0.1364	2.35101
ENSG0000(	RP11-196	16:311157	0.825432	0.779054	2.99971	0.793337	1.16787	1.78341	1.93527	1.39101	2.52465	3.1413	0.5344	0.78322
ENSG0000(	EMG1	12:697089	1.15288	1.84317	14.2766	1.41272	5.79256	3.00778	1.25918	17.5133	7.74126	8.03505	0.85405	-0.29911
ENSG0000(	NRSN2	20:320312	2.7331	1.60598	1.84505	4.81865	1.51352	0	7.21119	3.40014	4.10941	2.34652	0.6443	0.423083
ENSG0000(	TDRD7	9:9741194	6.22526	3.98094	2.46746	4.03122	1.9427	0.696902	12.6103	0.610746	3.82313	5.57131	0.47895	0.336144
ENSG0000(	UAP1	1:1625615	21.9452	11.0249	22.0075	21.1045	25.6204	7.39612	8.54129	6.95226	21.8629	10.695	0.0285	-0.87907
ENSG0000(	ADGRG1	16:576106	8.58109	18.8774	3.56254	1.26623	7.65012	1.72617	6.77142	0.738834	0	2.74743	0.0112	-1.87444
ENSG0000(	NUP35	2:1831175	7.14742	5.9025	6.54493	6.55101	4.87428	3.43406	6.40729	8.84181	16.0668	7.18072	0.32985	0.46266
ENSG0000(	FAHD2CP	2:9601055	4.22085	2.52585	10.6156	7.81111	5.58063	8.85441	2.73032	6.7636	1.76518	3.33124	0.6761	-0.40927
ENSG0000(	TDRD3	13:603964	6.76011	8.47934	2.89773	1.43251	4.51402	5.4134	13.8748	5.11856	1.45304	6.98888	0.4079	0.514981
ENSG0000(	KIAA1109	4:1221523	6.68981	6.6884	1.15216	2.95396	2.66514	3.66014	5.48499	3.89936	2.58621	9.84687	0.33705	0.426464
ENSG0000(	PAGR1	16:298061	45.7465	46.5569	104.139	75.623	97.0665	31.7487	40.7466	51.8117	62.3674	55.6561	0.6116	-0.59196
ENSG0000(	LTA4H	12:959965	32.3238	39.2076	35.0451	38.4154	37.2797	80.2545	20.8233	31.7235	21.6566	28.9723	0.84165	-0.06825
ENSG0000(	TRNAU1A	1:2855308	21.0667	24.3487	29.8176	31.1313	18.1437	23.6282	34.2167	35.0593	24.9629	18.2794	0.7875	0.125607
ENSG0000(	FANCL	2:5790765	6.7395	6.48826	3.118	4.43326	4.09606	15.1721	20.4949	5.04014	1.66865	8.8117	0.1717	1.07479
ENSG0000(	ZNF264	19:571914	7.32451	6.90175	9.36807	12.1336	8.78538	9.84986	13.7072	10.3821	13.2511	3.8696	0.9754	0.033295
ENSG0000(	LYRM9	17:277567	4.27006	7.16999	10.8196	17.6975	5.61074	4.85248	3.9598	3.55637	8.60867	2.93824	0.1167	-0.97699
ENSG0000(	IER3IP1	18:471059	26.4097	17.4772	26.1676	25.2936	18.483	21.2343	23.7134	18.9253	22.7944	7.30803	0.55845	-0.26028
ENSG0000(	KCTD21	11:781397	1.11947	3.40065	1.2763	1.08557	1.13718	3.31584	0.376384	8.68182	2.86122	6.02992	0.0175	1.41067
ENSG0000(	DTX2	7:7646167	13.3359	20.2257	6.86583	6.55524	12.5604	13.3763	23.5419	10.5098	0.16366	13.5989	0.87585	0.078047
ENSG0000(	ACRC	X:7157841	2.56975	2.49469	2.55386	2.05772	1.30348	1.05785	1.57851	0.863916	1.55228	2.30635	0.4832	-0.51178
ENSG0000(	RP11-103	16:187812	2.8713	7.31844	2.76081	0.444047	5.91362	0.771962	13.4164	2.8582	0.513743	1.03757	0.68795	-0.72194
ENSG0000(	PYHIN1	1:1589307	228.654	147.837	227.408	204.439	255.109	158.056	130.218	109.44	118.302	181.009	0.0524	-0.65304
ENSG0000(	SEPSECS	4:2512001	2.41751	1.44836	0.934189	1.99302	0.875848	2.52588	3.51309	4.93126	1.06337	2.54682	0.1294	0.916821
ENSG0000(	RP11-324	10:350980	0	1.1006	0.149718	2.2176	0	0.796503	4.03441	1.36632	2.23826	0.174865	0.368	1.53369
ENSG0000(	CAAP1	9:2684068	17.5983	16.8586	10.9435	14.6299	13.4607	12.0186	23.1409	11.3788	16.4227	14.87	0.9171	0.037774
ENSG0000(	ZFPM1	16:884533	0.624027	0.926478	0.131865	0.19124	0.406705	0.174783	1.25015	0.626487	0.082379	0.42121	0.97775	0.031397
ENSG0000(	NRBF2P5	10:576522	0.717733	0.718164	0.200632	0.260685	0	2.48441	0.629888	0	0	0	0.7223	0.686192
ENSG0000(	TNFRSF4	1:1211325	55.9262	45.2626	4.96793	21.976	6.15143	295.447	775.931	107.114	81.3125	34.7733	0.00005	3.00665
ENSG0000(	STX6	1:1809131	12.0341	11.2439	6.06382	4.8695	9.35081	10.3396	9.45878	7.73468	11.134	13.8217	0.51635	0.239068
ENSG0000(	C19orf47	19:403195	3.24779	2.45502	5.88855	5.00696	8.70339	2.68181	0.817426	10.111	8.64005	7.69614	0.85925	0.180711
ENSG0000(	CTCF	16:675492	33.8812	40.3944	24.623	22.2805	38.5576	30.7107	34.6601	27.8381	26.6247	49.4345	0.94595	-0.02621
ENSG0000(	LINC0100	7:1524637	26.2383	19.9663	25.3132	18.1416	18.6507	32.4245	16.6264	20.4645	21.5887	10.1625	0.7909	-0.10969
ENSG0000(	DNAJC27	2:2494363	0.963113	4.6872	1.32658	2.07749	1.74298	0.034147	3.10385	4.32169	1.55784	3.07809	0.81125	0.158593
ENSG0000(	HCFC1R1	16:302261	24.8302	28.1295	16.5928	19.3285	23.037	30.3078	39.6535	24.1661	14.1274	15.1892	0.98285	0.015519
ENSG0000(	TBC1D20	20:435479	13.6205	15.8637	6.54663	6.76258	9.48616	10.9713	20.4411	14.946	7.08169	21.2845	0.2915	0.442016
ENSG0000(	CCL4	17:361035	118.954	51.0407	464.309	629.321	426.425	436.561	1201.75	1963.1	610.5	340.256	0.0099	1.37088
ENSG0000(	SIRT6	19:417410	5.36051	3.61456	8.32596	16.158	10.976	6.73252	4.84476	15.3239	8.33462	19.4536	0.62825	0.512363
ENSG0000(	CDCA4	14:104985	11.1344	10.9656	15.9372	16.7872	20.6863	21.9222	25.1016	22.5191	9.33773	16.3245	0.5549	0.227321
ENSG0000(	DNAJC19F	14:452900	1.27236	0.132677	0.142203	0.582839	0	0.177645	0.481059	0.159626	0.210204	0	0.63485	-0.83579
ENSG0000(	EEA1	12:927215	6.64005	8.80667	2.21375	2.01132	5.61757	2.82761	6.92899	2.25448	2.4157	11.3446	0.6512	-0.30408
ENSG0000(	TP53I3	2:2407652	1.13163	1.97999	0.128535	2.66294	0.313896	9.00051	2.4439	1.99842	6.10486	0.396855	0.15145	1.69784
ENSG0000(	TRBV6-4	7:1423808	54.2948	2.77642	140.113	153.032	67.4236	20.7711	4.04376	69.6097	70.5126	44.9208	0.1696	-0.65168
ENSG0000(	P4HA2	5:1321848	0.221822	0.737031	0.249166	0	0	4.90564	2.16973	0	1.8111	0.384639	0.001	3.31114
ENSG0000(	FAM213B	1:2586490	10.6272	17.5596	9.62728	4.87292	10.2746	17.1962	14.7948	6.93764	5.98375	15.2208	0.7432	0.139255
ENSG0000(	NTN4	12:955515	5.70073	0.54245	1.16759	7.13484	4.14333	1.50266	0	0.86841	9.59592	2.85035	0.8845	-0.08885
ENSG0000(	CTD-2319	17:601265	0.2259	1.62192	0.768062	0.60561	0.298606	3.76211	1.07909	0.799062	0.368957	0.869696	0.47575	0.919581
ENSG0000(	ATG4B	2:2415844	45.9861	59.9243	41.5233	48.5319	42.8603	83.6008	59.3331	32.7134	43.7268	57.3604	0.64875	0.178958
ENSG0000(	TRAF4	17:287439	0.554082	0.293165	1.48338	0.506049	0.571266	0.738267	0.757131	0.60142	0.718497	1.11951	0.91085	0.064925
ENSG0000(	CS	12:562716	45.1498	58.3102	24.9951	27.5629	21.5033	28.1957	36.2494	33.6875	16.3437	38.0335	0.5632	-0.27205
ENSG0000(	THOP1	19:278545	19.8548	16.3335	15.9471	15.1642	18.7443	10.6757	15.0509	14.2092	0.095172	20.4293	0.24365	-0.80756
ENSG0000(	RMI2	16:112496	8.23709	23.6721	0.657375	3.72744	2.1402	16.741	13.2859	3.52152	4.68273	1.8261	0.9101	0.163635
ENSG0000(	UBA3	3:6897521	82.945	73.236	41.337	60.3608	26.8072	60.0705	85.3476	48.3477	71.8893	43.0709	0.57275	0.242138
ENSG0000(	NFIA	1:6065963	2.47605	1.06516	1.65679	0.124012	1.18818	0	0.403068	6.70048	0.044161	1.97619	0.80835	0.244584
ENSG0000(	ADAL	15:433306	6.89877	9.77588	5.80679	5.97317	3.9439	6.46803	4.71781	4.27275	1.96687	3.63166	0.5897	-0.47567
ENSG0000(	DDX19B	16:702896	27.4454	31.3521	43.6308	36.7905	31.9248	23.4173	34.5648	26.203	47.7046	23.6439	0.77265	-0.16169
ENSG0000(	RPL23AP2	1:1937568	1.1201	0.272102	0	0	0	0.614492	1.25632	0	3.74571	0	0.15865	2.32133
ENSG0000(	VAMP8	2:8556156	281.848	157.591	327.52	408.244	280.658	369.812	298.635	327.973	301.381	84.2921	0.674	-0.1366
ENSG0000(	RP11-888I	18:910262	0.413917	0.362033	0.025507	1.22229	0.411083	1.72091	0.245472	0.271576	0	2.07169	0.71505	0.613709
ENSG0000(	WDR41	5:7721044	5.97833	4.00681	8.51849	5.3596	5.64657	1.02915	3.4929	5.25806	10.0578	8.08038	0.7773	-0.12405
ENSG0000(	RP11-105I	2:2131529	2.56784	4.17923	0.257126	0.830451	1.14359	0.426776	2.14544	0.625312	0.710539	1.168	0.8124	-0.58353
ENSG0000(	HIVEP3	1:4150636	12.3324	9.66624	8.13513	8.98961	9.33408	8.20597	4.14108	3.90618	2.10535	11.2925	0.2355	-0.84418

ENSG000C	RASSF3	12:645070	2.51337	2.56323	12.8562	6.45999	8.74763	2.15915	1.51589	6.1645	3.56822	8.33076	0.0659	-0.84154
ENSG000C	MTFR1	8:6564473	3.69879	5.53177	0.612026	1.09076	1.05333	0.491942	2.35531	2.73659	2.73298	1.91572	0.92535	0.076562
ENSG000C	LINC0034	2:9580711	1.43755	1.11859	0.725115	0.104386	0.935647	4.23128	3.65423	3.19554	0	3.56908	0.0531	1.76854
ENSG000C	PKP4	2:1584569	1.2449	2.42412	2.07537	1.1123	1.66407	1.39296	0.877824	1.2031	0.369289	3.85034	0.55495	-0.3613
ENSG000C	TMEM191	22:214664	1.50199	3.32623	1.34196	2.70492	0.631516	2.81954	3.02196	1.33618	2.85475	1.02047	0.4801	0.503429
ENSG000C	TRAF1	9:1209023	9.116	15.2088	6.82161	5.36229	4.80445	14.8962	35.3192	22.2454	17.3514	20.8178	0.00005	1.49738
ENSG000C	6-Mar	5:1035370	28.4896	36.6249	42.0719	25.1014	17.7042	32.6281	33.1237	29.4955	25.3325	45.7858	0.96985	0.020165
ENSG000C	HLA-F	6:2972277	265.496	255.052	586.208	593.275	342.879	240.565	388.626	468.27	376.298	288.175	0.50045	-0.24634
ENSG000C	ARPC2	2:2182170	703.704	662.143	1031.94	1031.64	1034.17	968.231	886.012	1009.27	1036.75	543.674	0.92745	-0.03636
ENSG000C	AC005083	7:2021757	8.67227	0.817053	7.06781	4.02085	8.5019	2.57869	5.57361	8.4232	12.3585	7.20908	0.7679	0.314691
ENSG000C	TMBIM1	2:2182703	70.0369	61.8453	55.0478	63.915	61.904	69.7717	57.9919	67.3355	79.1159	63.2338	0.89815	0.045035
ENSG000C	NBEAL1	2:2030148	2.41431	4.04934	1.66348	2.77601	2.64834	1.42546	0.877641	1.73872	11.2083	7.42436	0.4164	0.754459
ENSG000C	ORC5	7:1041263	22.9683	19.1399	16.2927	11.7325	10.1403	15.5656	15.5207	10.9668	11.6618	6.84288	0.36355	-0.34651
ENSG000C	RLN1	9:5334968	3.71113	1.32129	3.66769	1.31154	1.7753	2.56414	2.40168	1.0885	1.28206	0.328485	0.8357	-0.65101
ENSG000C	RP11-12G	13:100086	1.34795	0.711342	1.00347	0.209176	0.314882	1.15717	1.54817	0	3.4565	0.823105	0.68545	1.02687
ENSG000C	TEPP	16:579764	0.30952	0.858424	0.679306	0.301746	0.716167	0	2.58004	1.2807	0.502621	0.795508	0.43905	0.938264
ENSG000C	EHD3	2:3123433	1.22781	0.365198	0.417588	1.56306	0.658083	0	0.42653	0.425217	1.66155	0.834284	0.66745	-0.33419
ENSG000C	FMR1	X:1479119	34.2513	30.0064	5.09436	5.64835	8.32675	17.3454	20.1098	12.9975	6.64539	16.966	0.74125	-0.13488
ENSG000C	FBL	19:398344	42.4724	41.6369	66.778	40.7787	86.9201	20.0909	55.5479	58.4673	18.8028	30.4757	0.11355	-0.62757
ENSG000C	CARD9	9:1363274	0.831188	6.3734	0.584869	1.77016	0.497448	3.57781	0.571462	2.64034	0.298268	1.46142	0.8701	-0.22604
ENSG000C	GRAMD1A	19:349947	39.7248	29.7278	57.2842	67.4212	50.1594	40.5551	10.8195	48.722	45.9146	70.2181	0.5248	-0.2474
ENSG000C	GBA	1:1552344	22.0625	14.3914	18.6162	16.179	19.4694	24.6982	16.5176	12.8578	14.5924	21.4806	0.6555	-0.18002
ENSG000C	TCEANC2	1:5405358	3.74768	4.4554	1.39114	3.83138	2.0834	2.97207	4.5688	3.54571	4.10484	4.02924	0.72715	0.242864
ENSG000C	CCDC167	6:3748291	113.667	60.8375	159.355	122.217	93.4003	159.706	174.706	151.413	86.6308	21.4207	0.9516	-0.02322
ENSG000C	NR2C2	3:1481896	7.72553	9.17788	2.24664	2.75641	3.79775	3.71595	5.82216	5.00532	2.72916	6.93182	0.63065	-0.28135
ENSG000C	TNK1	17:738053	6.30168	14.8117	7.45549	12.0673	3.919	4.32197	5.01149	9.08659	12.605	8.55041	0.9344	-0.0736
ENSG000C	FBXO32	8:1234978	9.98383	15.6562	9.09049	5.27454	7.80682	4.74774	9.70792	6.83463	8.86687	9.22593	0.4788	-0.46497
ENSG000C	UBASH3A	21:424034	27.2219	38.491	42.5105	26.6464	33.2543	17.8353	44.9134	34.4399	23.2297	59.2532	0.7991	-0.08612
ENSG000C	TP53TG1	7:8732522	33.2402	29.5076	38.5907	34.173	17.7774	18.9513	19.7401	28.0432	12.5476	6.342	0.1154	-0.84736
ENSG000C	CD63	12:557253	177.826	98.5504	224.795	192.842	258.277	791.196	724.438	327.161	199.126	113.381	0.00855	1.05121
ENSG000C	NUDT15	13:480375	6.33166	6.55469	9.6988	11.669	3.98216	7.38231	7.62775	11.9177	21.1543	2.1919	0.3883	0.420645
ENSG000C	ZC3H8	2:1122115	13.9801	14.9397	14.0067	17.5548	16.9769	13.081	19.0299	20.3835	16.2581	11.3096	0.74845	0.15258
ENSG000C	GRASP	12:520069	2.20862	4.1062	0.944991	1.45529	1.26201	0	1.25351	1.6448	1.37161	1.00942	0.40155	-0.89082
ENSG000C	OGFRP1	22:421600	0.473396	0.288547	0.588655	0.933465	0.455502	0.140466	0.349031	0	0	0	0.0792	-2.99768
ENSG000C	C1QB	1:2265276	0.899074	2.24574	0.742642	0.754219	2.2254	221.869	12.8084	9.44137	40.0992	10.4814	0.0094	5.42439
ENSG000C	UBE2L6	11:575516	210.607	174.997	326.784	377.372	333.365	308.736	387.335	373.136	276.943	151.609	0.9891	-0.00511
ENSG000C	FES	15:908836	4.0233	13.3312	0.409509	2.80643	0.834989	32.5113	12.3677	4.68892	6.39243	2.91349	0.01015	1.42652
ENSG000C	PPIA	7:4479667	1551.49	1322.15	1316.19	1238.8	991.811	1558.72	1713.73	1358.41	1028.5	179.091	0.8134	-0.11159
ENSG000C	CHCHD5	2:1125842	36.0886	25.8767	48.2063	61.0743	20.8422	46.2386	37.9104	46.336	33.8347	11.5205	0.6588	-0.19997
ENSG000C	TTC39C-A	18:239927	2.02948	0	1.66507	3.91922	0.367177	0.798784	0.220714	3.72769	4.24256	0.144499	0.86305	0.279099
ENSG000C	CEACAM2	19:415451	14.3936	18.3816	10.5996	53.1164	34.0194	22.2985	21.406	6.61668	34.0323	25.8381	0.7046	-0.27195
ENSG000C	CTD-2587	19:552266	2.44339	4.34768	0.344792	0.374832	0.612495	2.11523	2.14357	0.762487	0	0.672375	0.9819	0.034763
ENSG000C	KRTAP16-	17:413076	0.840156	0	0.373462	0.977879	0.104778	0.166548	3.00895	0.797748	2.35117	1.39683	0.22405	1.70626
ENSG000C	SAC3D1	11:650409	6.18705	14.7137	6.56291	5.52366	15.5005	11.1327	11.8473	9.36959	5.68114	10.5189	0.99555	-0.00482
ENSG000C	NECTIN2	19:448461	0.119685	1.0258	0	0	0	10.8214	0.573464	1.01631	0	0.821527	0.03575	3.73648
ENSG000C	GRSF1	4:7081578	50.4807	42.9349	40.0976	27.6062	31.114	44.5521	56.1919	42.3768	23.9304	26.0513	0.79995	-0.08622
ENSG000C	TUBGCP5	15:229831	4.50589	4.86762	5.20238	4.99172	2.42689	6.11892	2.54678	6.28244	2.40917	7.94491	0.77095	0.143043
ENSG000C	CD82	11:445644	59.5224	92.5949	25.8972	42.7792	45.6034	323.871	262.823	200.824	159.922	260.997	0.00005	2.05162
ENSG000C	TMEM30A	6:7525292	33.221	30.6508	11.6049	17.9171	9.12265	34.1339	15.8918	13.751	11.8408	14.6637	0.4807	-0.23243
ENSG000C	TIGD1	2:2325479	10.7212	15.4346	7.56498	4.79905	2.54494	7.87231	4.23773	7.89789	4.15074	10.8798	0.823	-0.26211
ENSG000C	RP11-540	15:409068	0.161381	0.178721	0.144726	0.305849	0	0	0.02504	0	3.28912	0	0.1984	2.32959
ENSG000C	PRDM11	11:450958	0.875664	0.353426	1.27906	0.042633	1.09814	0.044435	0.206921	0.313748	0	1.29653	0.27435	-1.14288
ENSG000C	CTD-2526	17:804537	0.249274	0.167884	0.874462	0.3288	0.367254	0	0	0	0	0.212347	0.1878	-3.29374
ENSG000C	AIFM1	X:1301293	20.6572	21.7822	10.7747	15.7321	13.0186	24.7283	12.9086	16.3549	1.85274	10.0458	0.39875	-0.33617
ENSG000C	ACAD10	12:111686	13.6591	16.5245	7.40644	8.83524	5.54295	9.40936	5.95563	6.45606	0	9.75054	0.2873	-0.63118
ENSG000C	RPS3AP26	7:9825237	8.30005	4.81509	3.66109	1.64934	0.703788	6.14523	6.36786	2.28383	0.620917	0.084831	0.89055	-0.24912
ENSG000C	LSM14A	19:341725	83.0397	91.1717	51.5514	52.2388	48.3801	56.5967	69.505	47.9993	53.7361	51.615	0.3555	-0.32023
ENSG000C	CBX4	17:798331	10.7851	10.8647	2.34497	2.01556	5.14887	6.64571	7.55272	2.74953	2.38995	7.80826	0.9884	-0.00873
ENSG000C	RP11-705	12:961728	55.632	41.2428	49.6799	54.4353	25.3141	25.6441	45.7482	43.4078	52.2638	24.6534	0.64505	-0.16534
ENSG000C	ZNF883	9:1129971	1.09849	0.233496	0.313953	1.50996	0.799675	0	0.24219	0.184602	0	0.163387	0.3043	-2.71875
ENSG000C	GMPR	6:1623857	0.481007	0	0.720405	2.7086	0.591673	2.6972	0.976404	0.877954	0	0.07024	0.8391	-0.31351
ENSG000C	CCDC186	10:114120	22.4249	24.2698	8.11368	9.87302	13.7394	19.4549	21.6441	12.3388	6.18462	20.1955	0.82915	0.097551
ENSG000C	CHCHD1	10:737820	38.9694	24.2565	37.165	36.5492	42.4559	56.6835	51.1609	39.3379	29.3175	13.0178	0.97505	0.015232
ENSG000C	HEMGN	9:9792679	1.47267	7.40932	1.13377	3.58312	0.855707	0.954596	0	0	3.43359	0.819289	0.27415	-1.48069
ENSG000C	IPCEF1	6:1540104	33.8731	38.9104	26.2826	26.5735	23.9389	9.43889	31.6333	18.0635	28.0725	42.7676	0.4086	-0.366
ENSG000C	MLLT11	1:1510477	3.93156	4.2549	3.13419	3.28909	3.30372	4.98433	1.63218	1.67342	3.31806	4.51459	0.9074	-0.2223
ENSG000C	RP11-411	10:100486	10.9649	8.89579	13.8785	11.0112	9.59704	10.5204	7.10181	10.1652	16.4163	5.31415	0.6374	-0.42636
ENSG000C	NSMCE1	16:272249	86.0993	100.524	112.32	96.6917	111.002	100.976	93.1374	80.6332	81.9615	26.4133	0.1887	-0.43554
ENSG000C	RAB37	17:746705	45.6438	71.0237	43.9729	34.1916	20.0865	23.224	31.2595	35.5647	37.906	33.2753	0.6551	-0.34831
ENSG000C	RASA3	13:113977	55.5704	85.3497	18.6471	20.9199	17.6186	8.22091	1.91899	7.8355	4.65726	11.1401	0.00005	-2.48557
ENSG000C	RP11-83A	4:1392661	0.215623	0.384102	0	0	0	1.13841	0.294599	1.21821	0	0.077653	0.28045	2.07113
ENSG000C	OVCA2	17:203010	65.768	41.4697	70.4096	78.8565	55.5287	42.1081	80.6648	73.2606	56.9638	15.7242	0.6343	-0.22854
ENSG000C	CYB5R1	1:2029618	25.0481	23.3906	23.9873	21.5259	23.1931	55.0075	28.36	23.8634	32.621	26.1167	0.19035	0.490554
ENSG000C	VPS35	16:466561	28.719	30.4761	22.8753	10.913	21.2829	44.0345	43.9419	34.2797	15.8917	33.2761	0.7457	0.105768
ENSG000C	SPINT2	19:382440	3.33814	27.3733	6.76937	12.1675	2.83248	21.6474	2.54228	12.2208	6.47736	3.14752	0.70565	-0.21305
ENSG000C	RP11-110	11:119067	2.54421	1.16582	2.48231	3.71421	1.19333	5.01546	2.13153	2.10048	1.20362	0.205962	0.9022	-0.16638
ENSG000C	RP3-477M	1:8907392	0.496147	5.41102	0.532679	0.253366	1.65004	0	0	0	0	0.460835	0.2133	-3.29712
ENSG000C	CCT5	5:1024992	146.823	128.909	145.732	129.988	154.954	156.714	153.622	112.091	110.255	95.3027	0.4366	-0.2498
ENSG000C	MUL1	1:2049944	26.8203	18.3032	20.467	19.1698	18.0366	11.1023	20.1282	15.4754	17.2225	27.8687	0.55065	-0.20791
ENSG000C	CPTP	1:1324755	12.475	16.1361	4.38464	5.99533	6.58654	7.10573	12.1779	5.22384	4.66057	8.84689	0.5518	-0.30178
ENSG000C	ROCK1	18:209469	32.7991	36.6334	13.847	9.16031	18.506	34.4223	32.2363	18.8242	15.2199	42.4827	0.3405	0.337712
ENSG000C	BLZF1	1:1693679	25.4839	18.5285	9.24759	18.5869	6.12547	17.7742	29.8496	13.0569	9.15016	11.3648	0.79335	0.097357
ENSG000C	MAPKAPK	1:2066849	1.43888	0.829548	1.66194	1.32904	3.22852	1.43046	2.952	3.72343	3.90176	4.63454	0.2027	0.875308
ENSG000C	FBXW11	5:1718615	10.0827	10.0222	5.99175	6.46143	9.32437	8.43554	6.82622	8.36961	9.43329	12.9625	0.96375	0.022631
ENSG000C	TMEM248	7:6692122	57.3021	49.3022	51.1047	42.3163	46.8394	68.8198	46.7887	58.31	46.7865	66.383	0.9383	-0.0283
ENSG000C	SPPL3	12:120762	14.2197	18.0151	9.04592	6.616	14.0234	19.0896	19.4718	12.4273	15.2032	19.1832	0.4585	0.304153
ENSG000C	SPTY2D1	11:185887	7.89866	4.66688	3.94961	3.70945	7.24054	6.63036	6.08006	5.95941	9.04287	10.2009	0.69855	0.18347
ENSG000C	ZNF185	X:1529144	0.552114	2.85718	0.695145	1.42654	0.432806	0	0.072196	1.39689	1.76995	1.6267	0.8442	-0.21912
ENSG000C	AC018816	3:4493347	0.568879	0	0.417694	0.368301	0.13233	2.97284	0.480502	1.58266	0.592875	0.620915	0.0428	2.09972
ENSG000C	IFIT5	10:894145	5.32251	5.65854	4.143	2.53821	4.98316	8.44423	16.71	12.1539	5.86297	8.96254	0.0025	1.20544
ENSG000C	CARHSP1	16:867456	13.4381	16.477	14.6322	15.3754	13.1598	22.1883	34.0483	20.4484	20.8192	10.1325	0.7157	0.401009

ENSG0000	LINC0053	13:212981	2.8108	2.12721	2.85536	4.39218	1.31305	3.96241	6.42402	8.56577	7.51074	1.53572	0.2191	1.12091
ENSG0000	SUPT16H	14:213514	52.9326	70.9758	19.4059	18.9169	34.4481	36.5302	33.4508	25.9422	23.4503	48.2971	0.6235	-0.20022
ENSG0000	RP11-159	5:4348395	0.656659	0.104898	0.48754	0.475316	0.24589	0.708677	0.120634	0.136779	1.47034	0	0.85345	0.359604
ENSG0000	LRRFIP1P1	3:1791481	1.20126	1.81301	0.411841	0.669213	0.499083	1.41088	1.69244	0.122914	0.025683	0.656893	0.86595	-0.28672
ENSG0000	DAZAP2	12:512382	647.046	702.178	916.281	884.457	601.234	559.809	523.137	550.017	633.326	519.96	0.2445	-0.48806
ENSG0000	ZNF230	19:440029	3.65233	0.874951	4.42483	2.29367	2.5696	5.10833	5.87004	4.46549	7.33699	7.05171	0.10015	1.08834
ENSG0000	RP11-265	15:425488	1.26845	0.825563	0	0.377227	0	0.328682	0.911903	0.111639	0	0.192748	0.7865	0.382778
ENSG0000	ZNF317	19:914037	31.1745	25.0388	12.3609	26.0683	9.05807	12.0351	14.9568	15.1813	19.4458	10.1319	0.2852	-0.48155
ENSG0000	ZNF212	7:1492396	8.91441	19.6888	9.96478	4.42797	6.46264	10.2097	10.4452	4.0904	3.46421	8.0599	0.23525	-0.4903
ENSG0000	NARS2	11:782152	16.7333	16.1614	11.8835	8.60133	9.64032	16.2474	13.7833	7.31644	13.8901	9.389	0.81355	-0.09495
ENSG0000	CCDC141	2:1785211	2.43874	34.5959	0.062443	0.130827	0.143109	2.0323	10.8586	0	0	4.86307	0.51195	-1.02373
ENSG0000	CREB3L3	19:415360	0.189656	0.54487	0	0.072236	0	0.722101	0.751671	0	2.32981	0.540865	0.10945	2.79173
ENSG0000	TNFRSF1A	12:632875	44.7975	38.4241	64.5483	60.279	80.4394	49.2221	62.1671	62.0075	49.5835	129.224	0.6303	0.155118
ENSG0000	SURF2	9:1333517	28.1242	20.5316	48.3584	55.0619	51.5736	38.2706	45.9629	37.0487	60.5474	12.1375	0.8186	-0.1357
ENSG0000	CXCR1	2:2181628	1.62754	2.56716	0.579993	0.333605	0.410431	0	0	1.24499	0	0	0.0934	-1.95852
ENSG0000	ARFIP1	4:1527799	11.9922	11.854	8.16988	11.9626	7.78815	15.3422	14.9904	7.81051	1.23749	8.48477	0.7822	-0.18229
ENSG0000	SLC38A1	12:461830	90.1118	102.505	89.7166	65.5186	59.6463	62.1928	72.676	80.1304	54.4316	104.573	0.46335	-0.29538
ENSG0000	RFWD2	1:1759448	28.8655	31.6411	16.1976	16.8609	14.4246	30.3794	16.3108	15.2049	6.69965	15.858	0.75595	-0.12213
ENSG0000	TRAV6	14:217684	10.746	17.7252	14.8339	9.46963	13.7922	23.1403	17.7554	8.53043	16.2514	7.84339	0.86955	0.142984
ENSG0000	ZNF18	17:115984	13.4904	11.6577	10.6293	9.37019	6.86956	16.777	12.5475	8.11023	4.99864	14.9859	0.8797	0.07033
ENSG0000	TMEM222	1:2732214	37.3317	43.9784	56.8236	60.9971	38.7542	42.6362	42.914	45.8909	18.0334	34.8892	0.2548	-0.40656
ENSG0000	CCDC88B	11:643402	73.066	72.1056	17.6678	15.2039	27.4482	79.7227	76.8099	36.6133	23.4029	49.6992	0.24305	0.68933
ENSG0000	SOAT2	12:531035	0.92436	0	0.507421	0	0.146731	0.355232	1.13574	0.192728	0	1.15704	0.5842	0.807041
ENSG0000	COA5	2:9859930	40.6307	28.7999	25.94	22.2312	18.9318	50.0122	45.1113	28.5914	29.0038	18.8394	0.37145	0.341384
ENSG0000	MARK4	19:450714	1.51372	1.74638	1.54823	0.712919	1.9338	1.27223	3.89332	3.59835	2.47344	3.93001	0.20085	1.08634
ENSG0000	COG4	16:704805	40.3989	66.3466	23.6861	31.4377	20.0911	43.0153	19.6638	26.4864	41.7515	36.3716	0.99745	-0.00109
ENSG0000	ZNF236	18:768226	5.99631	8.49338	9.05297	7.25242	8.53224	7.53782	8.86041	7.95316	5.97024	16.7425	0.94545	0.036797
ENSG0000	MIA3	1:2226180	44.6885	54.4953	17.411	25.3967	20.7959	42.8055	29.3614	24.0453	22.5867	30.1624	0.9412	0.030957
ENSG0000	GLG1	16:744474	58.886	84.4351	29.9372	30.6592	30.5938	47.5977	50.5519	33.0794	21.0006	54.7774	0.24575	-0.38526
ENSG0000	RRP1	21:437895	1.5589	1.77423	21.3324	16.8115	19.4665	1.38483	1.7419	17.309	9.34156	15.6156	0.50225	-0.36358
ENSG0000	TMLHE	X:1554665	7.62526	10.2528	5.73873	6.52939	5.68936	11.8353	8.28692	3.56547	1.95358	4.21665	0.59315	-0.34621
ENSG0000	CCNB2	15:591050	22.021	24.9385	0.090655	0.753511	0.497774	18.5932	10.7569	2.73741	21.4108	1.73515	0.4736	0.427089
ENSG0000	SLA2	20:365734	4.45977	3.6082	55.8796	67.8853	35.6448	8.315	18.6072	95.1812	73.8031	79.3888	0.4761	0.659414
ENSG0000	RP11-408	4:6531956	1.28931	1.48111	2.98951	6.53557	3.53891	1.5938	1.21306	2.24813	3.21238	0.407126	0.4661	-0.90705
ENSG0000	GAPDHP4	2:2224245	0.048607	0	0	0	0	2.35491	0	0	0	0	0.3326	5.63108
ENSG0000	PPP1R18	6:3067638	95.6721	72.4103	52.4772	38.9235	93.2592	61.3682	90.5953	78.8706	44.5066	78.3952	0.728	0.136258
ENSG0000	DVL3	3:1841553	12.5024	15.9792	16.1056	5.35419	25.9809	13.507	22.2535	20.4207	2.27653	43.563	0.6649	0.204802
ENSG0000	EMILIN2	18:284702	17.1153	11.2216	4.33441	5.87338	5.14027	29.986	16.8773	8.46604	5.40833	7.23411	0.4349	0.532558
ENSG0000	ZNF620	3:4050599	1.26118	0.796846	0.588419	1.11847	1.28257	0	0.97303	0.05757	3.29336	1.69067	0.88945	0.203651
ENSG0000	GSTCD	4:1055526	0.626367	0.866776	0.550087	0.564894	0.581465	0.650864	0.190397	1.2387	1.81373	1.16947	0.49295	0.639808
ENSG0000	HCCS	X:1111130	22.1298	22.2477	19.456	18.874	18.7488	25.146	19.4626	18.5835	7.9085	11.2311	0.4548	-0.38959
ENSG0000	EIF3L	22:378308	77.4208	108.285	141.884	121.252	151.14	80.8715	44.0782	87.9735	97.4755	93.3145	0.11685	-0.63609
ENSG0000	SPNS1	16:289739	26.0327	45.9111	19.8609	24.7233	17.0259	31.5809	41.4182	21.1893	27.4168	36.0229	0.8134	0.211713
ENSG0000	HMGCS1	5:4328760	3.10196	2.68614	2.31091	4.54462	1.52536	5.43792	4.22265	7.9136	4.98249	5.63478	0.16565	0.792102
ENSG0000	TNFAIP1	17:283356	7.45605	6.93225	6.83011	7.19132	6.05155	3.51908	6.11025	6.54036	7.75376	8.85754	0.76145	-0.12714
ENSG0000	PAPD7	5:6713006	5.13922	9.75016	2.79318	3.80169	2.30207	2.62974	6.05054	1.51488	3.92438	6.71908	0.84615	0.088809
ENSG0000	MMP25	16:304668	2.33835	2.32979	0.49048	0.961755	0.745257	3.14173	3.15536	0.437187	0.236923	1.43937	0.8696	0.216975
ENSG0000	GCSH	16:810534	5.61716	4.82236	6.27533	6.18553	3.80853	5.32657	7.20753	1.1107	0	0.761237	0.28275	-1.14571
ENSG0000	SETD9	5:5690004	3.52446	1.06071	4.31364	3.17118	0.619574	0.121053	0.081349	1.10932	0.740869	1.52542	0.2171	-1.88783
ENSG0000	RP11-379	3:1700867	6.30812	8.56426	1.6852	5.54699	1.19257	4.62116	3.39757	2.9303	7.33123	3.27392	0.76355	0.471653
ENSG0000	S100A4	1:1535436	2199.02	773.352	2909.88	2934.08	1625.42	1990.5	1630.34	2489.84	2165.34	326.279	0.5376	-0.30077
ENSG0000	RP11-381	3:1085433	0.200201	0.19168	0.076746	0	0.081091	0	0	0	2.95453	0.393695	0.1426	2.86017
ENSG0000	CDK13	7:3995003	23.7759	27.5346	5.72906	4.36991	9.46955	15.3023	21.9453	6.54782	7.08814	14.6906	0.72375	-0.17485
ENSG0000	BEX4	X:1032150	48.2951	77.6773	59.7372	34.8447	42.9513	37.2024	22.7844	21.6892	17.7178	17.6326	0.00325	-1.18088
ENSG0000	SH3KBP1	X:1953397	113.975	161.274	132.809	69.5819	250.113	103.489	107.661	109.284	90.7052	333.923	0.75875	-0.10513
ENSG0000	EIF6	20:351720	109.881	76.2141	194.57	189.172	198.483	118.365	131.418	148.394	145.357	78.6003	0.41285	-0.30837
ENSG0000	CDC42	1:2205262	103.58	48.2544	154.21	169.971	118.44	65.5879	53.9087	122.498	194.931	56.4252	0.4594	-0.30684
ENSG0000	RAB38	11:880985	4.57271	2.74287	3.04004	1.59792	1.7134	3.70667	7.27586	6.47771	0	0.820854	0.69065	0.450805
ENSG0000	GEN1	2:1766381	2.2576	0.768134	1.14195	1.27172	0.658335	3.20971	3.33062	2.61594	0.009077	3.03493	0.3672	0.868598
ENSG0000	NUDT16P	3:1308505	0.053076	2.27507	0.429436	0	0.028702	0	0.019381	0	0	0.084398	0.0911	-4.88497
ENSG0000	CATSPER2	15:436285	1.22024	1.30146	1.11218	0.049818	0.662356	6.77456	1.94371	0.863223	3.32777	1.22448	0.06665	1.54965
ENSG0000	ZBTB10	8:8048456	1.23175	0.86231	0.595903	0.107802	0.755326	0.413348	0.381998	0.712519	0.173321	0.87962	0.44785	-0.74665
ENSG0000	FAHD2A	2:9540272	19.5552	14.4329	20.5836	29.8329	14.6887	21.5292	13.1484	10.0645	28.3571	11.506	0.60285	-0.24863
ENSG0000	PRIM1	12:567124	22.4602	33.0546	17.5465	25.4902	21.4028	26.5156	31.3545	17.1676	28.3029	21.6509	0.9723	0.034336
ENSG0000	ATP5G2	12:536327	261.444	293.709	477.173	437.322	381.369	342.989	346.602	427.622	374.064	123.573	0.47445	-0.23612
ENSG0000	IGLV1-44	22:223807	0	46.3004	3.53751	0.219445	2.31487	0	0	3.8899	0	0.412259	0.02155	-3.1691
ENSG0000	RP4-639F	1:9492756	0.527507	0.673349	0.268103	0	0	2.01749	0.766228	0	0.909216	0.175932	0.39765	1.34034
ENSG0000	KLRC3	12:104123	1.15597	3.05286	1.02992	1.91858	1.24842	3.32235	16.3105	2.35919	2.07007	2.04055	0.3691	1.53381
ENSG0000	RANGRF	17:828776	79.7355	64.7303	134.802	132.908	78.1757	83.641	93.9347	120.584	81.4239	44.3746	0.462	-0.26181
ENSG0000	KDM5B	1:2027244	6.57305	12.3991	3.75685	2.6286	4.09535	13.9102	9.73584	12.3689	3.97228	15.0198	0.04415	0.764371
ENSG0000	AXIN2	17:655285	0.365373	3.37739	0.190849	0.124828	0.051334	0	0.172589	0.240705	0	0.328846	0.02515	-2.37606
ENSG0000	GPR160	3:1700379	3.71827	8.62262	1.48834	1.24165	1.57328	2.24301	1.94802	0.904947	2.84457	1.01268	0.25305	-0.80506
ENSG0000	GNL1	6:3054137	16.068	15.2024	11.4602	7.13543	14.9227	8.49991	11.6725	13.1605	5.16412	13.1223	0.3915	-0.55867
ENSG0000	SMARCA4	19:109610	22.3648	25.6963	12.4314	10.0347	15.8938	21.9845	21.362	12.9037	11.8822	27.8328	0.4838	0.267939
ENSG0000	UBXN10	1:2018424	2.02051	0.768539	2.12261	3.54272	1.94304	0.922947	0.614159	0.158402	0.993608	0.854091	0.1172	-1.6978
ENSG0000	IL10RB	21:332299	48.3761	52.4595	34.9466	39.9048	35.0612	72.2168	47.9472	39.1382	57.5978	33.7311	0.61005	0.225541
ENSG0000	CH507-15	21:663018	2.01552	0.935657	4.89105	0.916665	1.01552	2.74649	2.15336	3.93589	0	2.73684	0.37955	0.894458
ENSG0000	RP11-327	13:517679	7.84008	3.4522	5.23543	10.825	5.0229	3.95114	2.94649	3.6878	4.19132	4.17627	0.4332	-0.77235
ENSG0000	POT1	7:1248223	13.8048	7.15503	4.56686	6.03049	3.64614	9.49042	6.61542	6.9262	2.0645	6.55806	0.8508	-0.10469
ENSG0000	RNF24	20:388823	1.86668	8.51729	3.63764	1.49606	2.6572	5.70749	9.02996	7.87793	3.02837	10.5875	0.3655	0.86777
ENSG0000	DYRK1B	19:398253	9.84712	10.4417	8.09407	8.65255	16.0305	5.82847	7.18859	7.60256	6.42925	16.6973	0.47455	-0.37684
ENSG0000	CREBL2	12:126118	18.4512	22.6651	4.05717	4.49228	7.0858	17.7742	16.4448	8.13246	3.89009	8.379	0.89835	-0.04774
ENSG0000	PPP1R2	3:1955144	61.3982	117.188	36.8879	43.2624	25.651	63.9319	71.3648	46.7801	56.8945	48.1075	0.9445	0.026288
ENSG0000	CCDC12	3:4692172	97.8788	91.0416	196.795	195.787	273.079	130.982	176.16	191.951	136.524	149.415	0.72755	-0.16649
ENSG0000	MAMLD1	X:1503614	0.259431	0.020046	0.133525	0	0.102823	1.37803	2.42256	0.779321	0	0.934223	0.055	3.1829
ENSG0000	N4BP2L1	13:324007	37.4315	48.0581	33.9556	69.7154	26.0054	38.3882	47.8979	25.8701	34.4374	21.7528	0.376	-0.50784
ENSG0000	STK40	1:3633962	27.5056	23.5728	13.6459	7.6561	18.5557	11.8045	25.6054	21.9891	7.38719	31.2229	0.99685	-0.00176
ENSG0000	TUBG1	17:425795	14.77	21.4943	12.3052	15.2938	14.9518	28.109	23.8179	13.3398	12.1274	24.6714	0.6771	0.312218
ENSG0000	AK9	6:1094879	6.93029	13.8332	4.38256	6.84016	5.4653	8.90186	6.31431	3.1488	4.58131	9.0927	0.50965	-0.45786
ENSG0000	AKAP11	13:422721	2.3136	1.89832	0.689259	1.18095	1.79078	2.90504	4.36009	0.878584	0.520588	5.37473	0.0841	0.723591
ENSG0000	RP11-119	10:796608	0.375137	0.116054	0.089552	0	0.057605	0	0.594071	0	1.23401	0.337845	0.3502	1.95432

ENSG0000(	CES2	16:669344	18.0933	26.2792	13.6443	10.0115	9.71566	9.49402	14.8664	15.0317	9.0935	15.7943	0.3176	-0.37619
ENSG0000(	BBS12	4:1227327	1.1724	0.541575	0.971485	1.07781	0.539138	0	1.90259	1.08102	0.012041	1.39643	0.95765	-0.0635
ENSG0000(	ERCC8	5:6087383	7.25818	5.19103	3.64115	4.27158	3.41685	5.51906	4.72776	5.36576	7.22038	2.86894	0.77285	0.155114
ENSG0000(	PPP2R3B	X:333962-	6.74024	25.348	2.49267	2.84828	1.18376	5.90968	12.9631	1.34566	3.82296	2.9002	0.3452	-0.6195
ENSG0000(	CTC-471J1	19:520313	4.03531	1.64916	3.18651	3.20377	1.48697	4.10044	0	1.84555	0	2.37996	0.75335	-0.64349
ENSG0000(	TPMT	6:1812831	18.3036	6.7149	15.0172	16.1843	13.2134	22.5775	17.0808	16.2441	23.9478	10.3212	0.3383	0.335924
ENSG0000(	LYG1	2:9899726	1.44632	1.02044	1.21442	1.43779	1.70493	2.07508	0.815764	1.44418	0	0.651479	0.6372	-0.52291
ENSG0000(	NPIPP1	16:149745	7.34274	3.2837	1.70101	1.91258	2.45876	6.71923	3.82581	2.94251	2.72706	3.581	0.74835	0.306926
ENSG0000(	ANKRD27	19:325970	17.982	23.5054	5.68326	5.93868	4.83931	4.03795	8.99971	8.08392	7.23761	8.6258	0.20615	-0.4919
ENSG0000(	NOXA1	9:1374233	1.29294	2.28324	0.886613	1.17505	1.04843	2.8926	1.7546	1.5913	0.798446	1.53754	0.7381	0.366346
ENSG0000(	RP4-549L2	1:1008723	0	0	0.319558	2.49538	0	0.118826	0.548339	0	0	0	0.4405	-2.34902
ENSG0000(	AC016683	2:1131573	1.0342	0.200138	0.936901	0.502709	0.315654	0	0.549726	0.155105	5.70017	0.395267	0.50285	1.36067
ENSG0000(	IGKV1D-3	2:8986248	0	95.2018	1.53079	18.6642	0.901765	0	0	0	4268.31	0.08027	0.58225	1.19877
ENSG0000(	RP11-758I	7:1004352	0	0.539685	0	0	0.437288	0.537027	0.913815	0	0	0	0.89625	-0.07922
ENSG0000(	AHSA1	14:774266	70.2861	71.4312	94.361	82.5411	111.115	69.4962	77.3993	96.3882	75.5225	90.3319	0.7631	-0.10135
ENSG0000(	ZNF677	19:532353	5.19755	15.6804	6.14801	5.92912	6.53586	3.86225	2.86394	3.19517	5.18725	4.79367	0.2179	-0.95108
ENSG0000(	CDC37	19:103911	68.23	82.475	96.883	221.329	101.424	64.9639	105.289	131.119	201.594	119.395	0.7374	0.128538
ENSG0000(	CTD-2649	16:220074	0.245776	0	0.509719	0.769204	0.532279	2.33259	0	0	0	0.253144	0.95755	0.048772
ENSG0000(	RPS3AP34	8:1253707	3.26909	3.58015	0.058249	0.474088	0	8.1196	4.17726	0.29557	0	1.76107	0.6781	1.07795
ENSG0000(	DDT	22:239669	64.8149	52.0812	89.8149	82.5483	56.0868	77.0793	62.1456	85.4267	74.7101	13.5658	0.6064	-0.25145
ENSG0000(	NAPEPLD	7:1030997	5.79055	8.9662	5.07018	4.29126	5.46344	13.4209	6.4424	5.29445	8.12251	9.39674	0.44185	0.417932
ENSG0000(	FLT4	5:1806015	5.48687	0	3.19281	3.05726	4.95597	1.89806	0.634785	2.04066	0.360869	4.57064	0.1666	-1.2072
ENSG0000(	HTR6	1:1966528	0.429598	0.206223	0.738244	0	0.995785	0	0	0	0	0.087054	0.2322	-4.71213
ENSG0000(	TRAPPC3	1:3613656	66.8693	58.0277	92.2922	118.76	95.3486	72.6233	72.7237	72.3503	87.9624	49.6793	0.37755	-0.38045
ENSG0000(	AFAP1L2	10:114294	1.69644	2.72829	1.93886	1.05549	1.18508	4.66654	4.9736	1.56017	2.68933	2.08482	0.1243	0.908246
ENSG0000(	IDH1	2:2082362	14.8999	14.009	10.7035	7.38198	7.11336	22.9737	19.4206	6.66531	17.044	14.2097	0.11505	0.653159
ENSG0000(	RAB24	5:1773011	34.3171	39.374	28.9726	37.8004	43.7623	85.7677	52.5577	22.8441	43.8779	65.9141	0.54455	0.463417
ENSG0000(	MLST8	16:220424	19.8814	18.1919	27.9783	32.2785	32.0637	17.4293	29.2624	21.4364	14.9113	26.2664	0.3559	-0.39716
ENSG0000(	RP11-66N	X:1535879	3.76277	5.84967	3.00851	4.2864	2.03606	1.45259	2.67379	1.37775	1.29403	1.3021	0.23535	-1.28703
ENSG0000(	DHCR24	1:5484963	3.61222	4.02701	0.426902	0.707452	0.396091	4.57328	9.42125	3.43081	6.86032	2.31997	0.00545	1.60609
ENSG0000(	RP1-239B	11:173806	0.150754	0.184583	0.703829	0.624151	0.895889	0	0.228305	0.664825	0	1.24005	0.55575	-0.56688
ENSG0000(	HP	16:720442	1.8076	1.47194	0	0.816784	0	26.6036	0.34995	0	0	0.305459	0.1731	2.7375
ENSG0000(	SLBP	4:1692799	76.5969	64.8537	73.5174	74.4263	62.6351	115.268	84.9182	72.4199	103.173	41.3252	0.48495	0.220875
ENSG0000(	AP000442	11:596164	0.199329	0.556201	0.21515	0.747264	0.525811	1.15275	0	0	0.202878	0.272113	0.6983	-0.57342
ENSG0000(	EMC7	15:340840	99.6371	71.0325	120.707	91.4472	94.4722	169.2	152.178	149.914	120.925	51.8916	0.2702	0.376814
ENSG0000(	TSHR	14:804769	2.09648	2.04986	0	0.575783	0	3.25899	1.81489	0	0	0.517969	0.83465	0.247208
ENSG0000(	RP4-561L2	1:9384717	1.19919	0.848615	0.244719	0.095453	0.263641	1.10758	0	0	0	0.062995	0.5035	-1.12584
ENSG0000(	GPN3	12:110452	26.1122	25.1525	30.5136	27.8541	31.522	32.9533	21.1109	18.6453	21.1265	7.78284	0.2305	-0.52552
ENSG0000(	GCA	2:1623188	10.268	20.9152	3.02849	3.60968	2.47724	31.911	13.2565	4.46559	0	1.51649	0.5114	0.42403
ENSG0000(	GFOD1	6:1335782	2.44245	6.10198	2.49757	1.00053	2.78463	4.20936	5.96903	4.36554	3.31323	10.4607	0.11705	0.943607
ENSG0000(	TCTN1	12:110614	2.57238	3.23637	1.40028	1.56619	1.06473	3.93588	1.29618	3.26451	1.92613	1.30766	0.68815	0.265293
ENSG0000(	GTPBP4	10:971145	20.5851	18.8101	22.5955	19.9146	19.8837	7.27102	28.4393	17.6359	6.78798	22.1582	0.50645	-0.47277
ENSG0000(	C3orf38	3:8805194	19.9892	11.5641	16.4882	16.3907	13.6579	9.04381	24.3662	22.5901	20.827	9.06156	0.80345	0.231218
ENSG0000(	GLYCTK	3:5225442	15.8707	16.1318	11.179	16.0614	11.2444	22.482	5.90529	6.94361	12.7096	14.6829	0.8701	-0.15538
ENSG0000(	SLC39A9	14:693801	8.46036	8.1821	2.61027	3.3367	3.41215	5.81055	8.25691	7.45114	3.67882	6.93157	0.6606	0.335917
ENSG0000(	FOXD2-AS	1:4743213	1.80865	0.287283	0.517398	0.414448	1.33835	0.58099	1.40249	1.25894	0.876717	3.91114	0.37375	0.869561
ENSG0000(	FOXN2	2:4831463	19.9194	22.5053	17.7739	17.3953	17.6326	14.7786	11.07	8.83794	15.9539	24.9111	0.48515	-0.49468
ENSG0000(	ING2	4:1835049	11.4016	18.4402	17.4932	16.8444	15.7003	26.6032	20.8648	13.5784	24.9389	11.1115	0.6387	0.230937
ENSG0000(	PNOC	8:2825006	0	3.61453	0.742698	7.84997	0.49309	0	0	0	9.8058	0.407222	0.95115	-0.11771
ENSG0000(	RP11-386	12:489951	5.68681	2.05995	1.87195	8.84519	0.369491	0.248031	0.622851	2.84027	4.61778	2.78912	0.5725	-0.76771
ENSG0000(	KLHL21	1:6590723	12.5831	7.83746	5.04311	6.43041	4.8585	13.4033	12.2463	10.3097	1.56894	9.70025	0.412	0.357303
ENSG0000(	MED21	12:270225	17.4251	17.4531	14.7646	11.435	13.6674	21.8831	19.0011	16.9477	26.2931	10.2433	0.42985	0.319589
ENSG0000(	SLC15A4	12:128793	50.7934	26.1977	16.6256	8.8864	27.5379	30.6974	16.4961	23.7118	9.11737	35.8628	0.8492	-0.07817
ENSG0000(	TRIM62	1:3314540	3.02161	4.35792	1.98077	1.48596	2.27742	0.913446	4.0275	3.37408	0.106095	6.74306	0.99035	-0.00743
ENSG0000(	CR2	1:2074542	0.407125	8.07867	0.366989	0.693202	0.099889	0	0	0	0	0	0.00005	#NAME?
ENSG0000(	NXF1	11:627921	131.34	99.2468	64.3969	72.0123	51.7284	65.3868	58.319	59.3015	74.4385	81.9682	0.4672	-0.22419
ENSG0000(	RTCB	22:323875	71.8937	72.5113	81.0844	85.0588	63.8386	68.0092	62.5438	57.0039	33.5784	70.0081	0.22165	-0.39573
ENSG0000(	GATB	4:1516705	7.69084	3.29273	11.0157	13.04	8.27997	6.66582	5.26697	3.228	4.52238	5.54883	0.09375	-0.91145
ENSG0000(	AC091814	12:994813	0.74139	0.325688	0.781459	0.100315	2.80627	0.207812	0.519981	1.02205	0	5.15448	0.8431	0.291529
ENSG0000(	EXOSC6	16:702467	7.26078	9.18089	11.2701	15.3181	8.45311	7.82245	6.60224	10.4557	10.663	7.22326	0.4251	-0.2983
ENSG0000(	ASB16-AS	17:441704	12.4915	9.70182	20.4065	10.6101	8.88965	5.6904	11.3226	8.83232	5.64117	9.99633	0.27285	-0.56589
ENSG0000(	SNHG5	6:8566094	140.506	76.1071	62.1764	80.2813	60.7966	106.328	86.1177	49.2719	84.7521	31.6721	0.69085	-0.18936
ENSG0000(	CCDC65	12:489034	36.9505	9.18348	28.7479	39.2928	27.6822	0.440955	2.67378	7.33573	13.6604	8.21416	0.03745	-2.01454
ENSG0000(	C21orf2	21:443289	33.1691	18.9926	44.001	52.6299	47.9445	14.2658	28.1593	17.8597	35.4868	22.5065	0.15665	-0.7416
ENSG0000(	HAUS3	4:2071917	38.6599	40.3534	17.3984	29.7334	12.5225	30.065	50.9323	21.5019	34.9956	24.9066	0.4487	0.268561
ENSG0000(	CDK6	7:9260492	5.80904	7.20019	2.79622	6.84158	5.34101	11.8943	11.4742	5.19643	19.9828	10.4478	0.2821	1.01254
ENSG0000(	SPATA6L	9:4490443	0.738949	1.00609	0.576142	0.280289	0	0.27956	0.908818	0.591024	0.885216	0.039705	0.96775	0.031663
ENSG0000(	RP3-488N	12:360663	1.07332	0	0.561351	0	0.072965	4.65837	1.39179	0	0	0.253618	0.4236	1.76552
ENSG0000(	KIAA1671	22:249527	0.208699	1.01182	0.762834	0.034689	0.622637	1.51884	5.37778	2.50724	1.19231	4.61258	0.0041	2.47927
ENSG0000(	ARHGEF1(	1:1753983	0.225979	1.9888	0	0.45067	0.10872	9.41503	0.616268	0.302258	0	0.135541	0.2532	2.061
ENSG0000(	RP11-644I	12:557253	5.78006	0.880325	4.75302	4.96534	7.79793	7.38643	4.21918	8.13077	5.82707	7.3687	0.78345	0.418519
ENSG0000(	TRAV1-1	14:216218	13.1872	12.2049	14.8448	13.1886	16.7563	24.4685	6.37274	22.7618	37.4757	25.3925	0.21235	0.90584
ENSG0000(	ZNF776	19:576819	4.95519	8.07924	2.4728	1.64664	1.23421	3.48878	2.34609	5.09785	0.0674	3.80722	0.79985	-0.20566
ENSG0000(	SPAG4	20:356158	0.222526	0.680026	0.741727	0.393369	0.779333	3.05546	1.18679	1.23641	0	0.511222	0.3581	0.970244
ENSG0000(	ABHD17C	15:806796	5.99626	3.28644	2.85874	3.53451	4.23858	4.10098	1.1687	2.09923	0	4.64432	0.2406	-0.86574
ENSG0000(	APPBP2	17:604431	5.15239	6.6466	2.88708	3.03796	3.66116	3.75369	4.63372	3.73302	4.03062	6.23124	0.85875	-0.10979
ENSG0000(	NFKB1	4:1025013	40.9741	45.2148	16.5746	20.3685	17.3814	31.5418	34.3713	38.0643	22.9429	30.7149	0.72595	0.11914
ENSG0000(	CTB-41I6.1	17:896589	1.70587	0.098856	0.843577	0.309249	0.59633	1.68784	1.06299	0	1.93146	1.15787	0.64465	0.824185
ENSG0000(	JAZF1	7:2773306	2.62614	3.35847	0.913951	1.70158	1.7864	2.02509	6.45653	7.14424	1.29959	12.8405	0.23865	1.3194
ENSG0000(	S100PBP	1:3277523	16.5714	8.0317	6.54238	5.18237	7.81291	11.7955	16.2885	8.50094	8.53143	15.3048	0.6185	0.342088
ENSG0000(	POLL	10:101570	10.1912	17.1438	8.62208	7.9329	9.58048	5.22448	6.88103	7.88537	9.10957	12.3275	0.64005	-0.29894
ENSG0000(	KRT18P7	14:705812	0.881736	0.779742	0.253255	0.643075	0	0	0	0	0	0	0.11365	#NAME?
ENSG0000(	USP40	2:2334755	2.91989	3.35732	1.60743	1.42733	0.850981	1.76273	2.06356	1.50262	3.60281	2.39051	0.7539	0.186406
ENSG0000(	MTND1P1	9:4984389	0.403688	1.15293	0	0.0366	0	1.51815	0.509697	0	0	0.043597	0.84625	0.514105
ENSG0000(	PDXK	21:437190	16.6645	11.865	35.7111	26.7248	33.7045	67.2644	28.2814	30.4083	30.4339	20.7629	0.69885	0.174611
ENSG0000(	ZNF446	19:546670	5.12277	6.83594	4.76936	2.48193	4.04663	7.75511	2.39323	3.76894	1.97008	6.30292	0.9781	0.023885
ENSG0000(	MT2A	16:566081	47.5387	56.0186	75.3827	111.419	180.098	84.4087	166.398	134.524	34.2914	35.9118	0.4373	-0.31674
ENSG0000(	CCDC121	2:2758296	0.881605	3.11821	0.451278	0.173533	0.82219	1.16637	0	0.668131	0	0.277441	0.41395	-1.20206
ENSG0000(	IDH3B	20:265839	101.866	146.847	120.519	153.787	104.898	92.2746	120.591	100.959	135.545	87.8363	0.52775	-0.1868
ENSG0000(	CCDC93	2:1178594	5.54895	5.84803	3.0763	3.31127	3.38764	5.25335	3.65682	4.90813	2.61767	6.80846	0.97755	-0.01178
ENSG0000(	ZNF609	15:644607	2.20226	10.5764	2.50571	0.358475	3.4787	7.35123	3.01211	0.765076	3.95632	13.3966	0.56385	0.468895

ENSG0000(	DHRS1	14:242905	15.0413	29.6801	30.0131	28.9849	15.6029	16.0136	28.3704	29.785	17.1389	22.1507	0.7935	-0.10802
ENSG0000(	MYBL2	20:436670	12.1616	23.9396	0.395969	2.56338	0.900067	10.9674	12.2954	6.45287	4.38959	7.79843	0.74415	0.1752
ENSG0000(	TOMM20F	X:7322312	0.163961	0.103403	0.478767	0.462832	0.254039	0	0	0.550734	0.160271	0	0.60825	-0.89812
ENSG0000(	CCNF	16:242939	3.48384	6.55752	0.479268	1.34144	1.07973	4.62781	3.27683	0.734472	2.62122	4.79186	0.53695	0.369724
ENSG0000(	LINC0102	5:1400728	0.47297	0.593251	0.047624	0.112792	0.670235	0	0.077257	0	2.86441	0	0.42235	1.1461
ENSG0000(	SFT2D2	1:1682259	7.96374	5.00999	13.1488	3.97294	10.4337	8.08122	9.93948	11.4559	7.10386	19.6343	0.8851	0.10631
ENSG0000(	LINC0148	12:697386	24.3771	21.6059	35.3408	37.9912	19.388	35.4031	27.0029	25.5883	30.7463	19.2603	0.9942	0.009159
ENSG0000(	RP11-91G	3:1429491	1.42648	0	1.86585	0.72417	0.650086	1.32456	1.24063	0	0	0.258116	0.53575	-1.05197
ENSG0000(	TNIP1	5:1510299	25.3667	23.1826	7.87573	12.0411	15.2683	16.8769	27.18	11.0585	14.1456	25.0777	0.6425	0.175165
ENSG0000(	ACP1	2:217729-	106.513	96.293	152.178	154.482	87.492	103.933	114.034	143.558	135.159	52.4027	0.71405	-0.12367
ENSG0000(	HSP90AB1	6:4424616	200.246	196.11	276.673	233.301	334.894	229.092	248.975	279.152	243.912	306.854	0.95055	0.022705
ENSG0000(	MTCO2P2	18:478089	0	0	3.50135	3.95194	2.73445	0	0	1.02535	1.86695	1.24162	0.6569	-0.86859
ENSG0000(	ZNF43	19:218049	3.73428	4.36551	1.38355	2.27951	1.32167	4.49589	2.62963	1.99396	2.76311	3.40595	0.85845	0.103462
ENSG0000(	YWHAZ	8:1009165	706.51	526.282	362.163	386.062	306.116	536.366	503.649	342.434	294.288	307.907	0.61535	-0.21382
ENSG0000(	AC010642	19:582285	0.307818	0.75711	0.728144	1.53788	0.355954	0	0.876627	1.98467	0.84374	1.05685	0.92265	-0.16898
ENSG0000(	ACTR3B	7:1527597	6.84648	8.03281	6.12094	4.67402	5.2893	1.8237	3.6755	4.71596	0.016928	2.93483	0.20155	-1.3215
ENSG0000(	SLC7A5	16:878300	14.0582	6.00337	3.34001	6.48932	8.40809	26.9706	56.7954	49.6007	26.1943	31.9347	0.00005	2.1775
ENSG0000(	INHBA	7:4166716	0	0	0	0.007584	0	3.69208	1.43262	0	0	0	0.3241	9.48278
ENSG0000(	FHL3	1:3799676	25.9723	9.68259	30.5686	47.1952	74.8081	24.6832	9.51866	14.4984	23.6145	32.942	0.0226	-0.89824
ENSG0000(	RP11-256	5:1382852	0	0.37654	0	0.402899	0	0	0	0	0	0	0.55975	-1.35355
ENSG0000(	RP1-34B2	6:2620215	0	0	0	0	0	0	0.909424	0	2.01284	0.221687	0.0038	inf
ENSG0000(	MEOX1	17:436403	1.55586	7.1796	1.39943	0.655089	0.382589	0	0	0	0	0.384199	0.24705	-4.93607
ENSG0000(	PLCXD2	3:1116746	2.24154	2.78712	1.12559	0.966251	1.1289	1.89081	1.66845	2.66071	4.92267	3.40572	0.2848	0.758986
ENSG0000(	SERGEF	11:177880	27.8492	15.4889	36.9577	33.1659	34.75	26.6875	25.0005	29.9472	31.7473	13.5666	0.6263	-0.19173
ENSG0000(	TRIP10	19:673792	1.34088	1.82578	1.26845	0.470454	1.5589	0.723622	3.26053	6.13795	3.48502	1.59714	0.09395	1.35446
ENSG0000(	COL11A2	6:3316268	0.015984	1.90972	0	0.052482	0.293055	0.074963	0	0	0	0.686005	0.4464	-1.56442
ENSG0000(	RP11-572	3:1129904	1.92443	2.15754	2.69589	3.93378	0.246833	0	0	0.437857	0	0	0.0082	-4.43176
ENSG0000(	PCNA	20:511495	68.7344	77.3532	53.621	67.3055	47.3863	111.029	93.4062	56.1017	63.8405	31.9464	0.5615	0.19201
ENSG0000(	PPIF	10:793474	13.0706	15.5333	13.4347	18.453	17.1182	26.5771	22.0748	42.2396	13.2647	35.836	0.01265	0.836937
ENSG0000(	HIST1H3B	6:2603164	1.92092	0.14132	0.184563	0.877558	0	1.18399	2.02394	2.07682	0	0	0.6594	0.67701
ENSG0000(	IFRD1	7:1124229	33.3806	35.5811	18.498	28.1658	13.5984	13.7554	24.3064	21.0459	14.5555	11.8064	0.124	-0.57834
ENSG0000(	TSTD2	9:9750117	5.70303	7.4168	2.77961	1.54996	1.88525	4.02304	2.96941	5.11623	2.62952	4.12592	0.9852	0.012038
ENSG0000(	ACTR8	3:5386706	11.2451	8.71104	12.5964	13.8459	10.0998	9.67491	9.40813	10.1679	9.99358	10.6055	0.6271	-0.21293
ENSG0000(	RIC8A	11:207510	63.0673	62.9821	65.5577	90.6514	58.3254	43.1984	56.8544	49.6763	47.9745	60.0167	0.1853	-0.40857
ENSG0000(	SPA17	11:124622	0.658966	1.45639	2.67031	1.57602	0.276724	5.54275	1.02248	2.1884	0	0.24897	0.8157	0.357461
ENSG0000(	RSU1	10:165906	79.6644	82.1361	101.275	101.02	124.806	98.2948	97.7324	85.098	85.0093	93.1557	0.63965	-0.21365
ENSG0000(	TAF1A-AS	1:2225579	0.664812	1.6029	1.23961	1.1195	0.492562	2.73795	0	0.867107	0	0.22099	0.6793	-0.51456
ENSG0000(	KDELC2	11:108472	3.42812	2.72827	3.6278	2.13668	4.55934	8.97131	1.56381	5.07074	0.429209	8.14729	0.45935	0.347742
ENSG0000(	RP11-373	2:2838440	0.965822	0.634637	1.19018	6.79295	0.451576	0	1.05804	1.79866	3.11267	1.97372	0.87575	0.231901
ENSG0000(	MPRIP	17:170425	46.0545	31.3487	21.9734	34.9044	41.5553	48.2045	24.8364	31.6465	41.1662	33.884	0.81665	0.162801
ENSG0000(	CTDSP2	12:578199	14.2206	21.0614	8.99716	4.97982	13.2571	9.56843	6.45708	14.904	1.7594	21.3224	0.42775	-0.28117
ENSG0000(	NUDCD2	5:1634465	19.3486	12.176	22.0062	23.4283	10.4699	20.5873	16.159	13.1185	15.6669	10.7153	0.6043	-0.28546
ENSG0000(	ABCG2	4:8809026	1.23132	0	0.265402	0.481928	0.340353	0	0.415532	0	0.274498	0.457535	0.36175	-1.17115
ENSG0000(	COL4A3BI	5:7536848	10.6486	13.8881	7.0217	11.781	9.91522	10.0211	11.9565	9.06594	7.25119	25.0088	0.754	0.182998
ENSG0000(	RP11-702	11:459293	0.121004	0.156122	0	0.315309	0	1.36679	0	0	1.08736	0	0.4015	2.10564
ENSG0000(	PAN3	13:281385	8.50028	13.48	15.1663	6.49415	7.32546	6.4829	7.6011	8.3307	9.7236	16.3024	0.53645	-0.22198
ENSG0000(	RP11-681	14:891248	2.24541	3.6339	1.48084	0.487106	1.86655	2.65884	1.08362	1.30239	0	3.65336	0.87625	0.228642
ENSG0000(	PPIAP31	10:321883	1.54331	1.66581	1.60613	3.81657	0.6803	2.21512	1.88421	1.61082	2.28578	0.12061	0.93835	-0.1495
ENSG0000(	STAG3	7:1001775	1.8083	4.11857	1.14305	0.749313	1.25881	4.51284	13.2857	10.6025	4.51398	7.92772	0.09315	2.28972
ENSG0000(	RP11-793	12:535001	3.74475	1.27283	1.31463	1.00559	2.4181	1.98807	2.40663	1.33781	0	1.62415	0.76135	-0.45807
ENSG0000(	RP1-234P	6:7528501	0.610895	1.01857	0.648524	1.45929	0.464551	0	0.25976	2.12158	2.78556	0	0.69265	0.616594
ENSG0000(	LINC0102	5:1087278	0.309523	0.817514	0.134598	1.47662	0.482984	0	0	0	0.686555	0	0.2346	-2.03959
ENSG0000(	PXN-AS1	12:120201	23.1992	19.2835	14.3641	13.3918	9.35623	5.88545	8.59351	15.7726	9.72505	5.5312	0.8469	0.159644
ENSG0000(	INPP4B	4:1420231	19.1691	17.114	9.56033	19.6284	11.3133	24.0181	22.0989	18.7829	14.2498	22.3605	0.4877	0.265577
ENSG0000(	TGFBR1	9:9910403	10.7785	7.50683	5.14977	3.37471	5.22531	5.08511	4.87712	5.76519	3.95603	7.93479	0.31825	-0.36839
ENSG0000(	CERKL	2:1815366	17.253	19.7952	7.79476	7.95417	6.28576	11.9165	15.6329	12.1407	9.85881	13.4136	0.65835	0.153628
ENSG0000(	GOT2	16:587071	60.9507	59.719	60.8439	58.2201	46.3961	46.1184	37.0165	33.5478	54.7271	42.0112	0.1679	-0.43965
ENSG0000(	BMPR2	2:2023769	1.00795	1.05125	0.582344	0.472037	1.13892	1.5452	1.95122	1.39336	0.014787	2.33209	0.3197	0.639781
ENSG0000(	HIST1H2A	6:2713304	3.77967	3.31481	2.88172	2.33787	1.08129	0.756554	2.24165	1.581	4.11525	0.131118	0.55685	-0.49911
ENSG0000(	LINC0018	21:291934	0	1.1171	0	0	0	6.90649	1.07168	0	0	0	0.20955	2.89326
ENSG0000(	TRIM47	17:758741	12.3262	8.44199	12.7743	17.4536	18.6867	6.7648	7.89662	9.92322	20.5231	14.3369	0.65035	-0.22127
ENSG0000(	FAM160B	8:2208915	25.18	30.3387	6.76557	4.98026	8.13349	38.4003	23.0343	12.2664	12.0855	13.4787	0.3708	0.346268
ENSG0000(	ERO1A	14:526399	26.3767	16.306	13.513	11.4431	12.5472	36.4382	15.8253	17.2278	21.3441	22.806	0.48525	0.308873
ENSG0000(	PRAM1	19:849005	2.13068	13.9906	0.305573	0.477515	0.56265	18.9481	1.03774	0	2.02779	1.45499	0.56705	0.647269
ENSG0000(	ANKRD46	8:1005097	12.6995	10.3154	9.72424	7.67248	7.91199	7.8447	7.01107	7.40655	15.6324	12.5276	0.9544	-0.02498
ENSG0000(	RP11-375	8:9555143	1.18601	0.313198	1.02392	2.1449	0.746681	0.337232	0.408269	0.829231	4.72109	2.2056	0.68715	0.729931
ENSG0000(	GVQW1	9:3254054	0.574879	1.44995	0.530923	0.416683	0.776931	0	1.55213	0.262113	0	0.347053	0.60835	-0.8654
ENSG0000(	AGO3	1:3593071	8.29681	10.9652	5.56398	4.02395	2.36923	5.80184	5.43559	7.39067	0.256111	11.7294	0.82385	-0.18831
ENSG0000(	FAM192A	16:571524	61.8925	90.0315	66.192	47.4877	53.0632	56.0996	81.8832	52.9122	52.0185	48.9598	0.56945	-0.20604
ENSG0000(	ACTR1B	2:9765596	9.20184	11.2734	27.9518	14.9116	19.0524	10.7801	10.9272	39.5932	18.8928	22.2583	0.41355	0.320284
ENSG0000(	FXN	9:6903525	30.0473	34.9014	42.0245	38.7788	43.0392	15.4354	19.7451	19.2072	30.1261	9.93484	0.08005	-0.96739
ENSG0000(	TBC1D9B	5:1797975	24.1797	22.4943	7.54892	7.21319	11.0188	21.0272	14.9458	11.3	0.716958	18.9336	0.938	-0.07877
ENSG0000(	C1QBP	17:528226	149.598	133.721	194.813	183.171	135.934	132.57	157.241	115.344	95.8235	37.3596	0.33745	-0.61277
ENSG0000(	C9orf69	9:1361145	47.671	35.1432	18.2234	13.4656	39.0998	33.0327	52.2994	21.4024	11.5226	34.3984	0.8159	-0.08742
ENSG0000(	CSNK1G1	15:641655	4.22233	2.42938	2.11462	1.08484	2.88662	2.06829	2.80529	2.51043	4.75143	4.57743	0.68365	0.241595
ENSG0000(	PPA1	10:702028	100.471	54.4375	116.475	91.704	109.454	177.664	155.502	118.311	76.4817	35.0773	0.49595	0.219078
ENSG0000(	DDX60L	4:1683567	12.1091	13.02	6.00746	7.30562	7.16016	11.367	20.8794	11.6886	14.9323	9.69056	0.20465	0.531677
ENSG0000(	BLOC1S6	15:455871	20.8033	17.1661	15.9876	16.4975	16.1842	13.9167	19.7757	16.4663	33.7746	18.5303	0.8431	0.120645
ENSG0000(	RHOXF1	X:1200362	0	2.29559	0.073923	0.565198	0	0	0	0	0	0	0.13155	#NAME?
ENSG0000(	TRGC1	7:3825787	254.292	16.5507	76.2453	77.2369	51.5857	70.9129	34.9308	45.5605	43.6922	35.2656	0.00525	-1.12319
ENSG0000(	CETN3	5:9039226	36.1285	23.3906	20.7661	17.9244	15.6889	39.1906	20.9216	9.16036	15.1634	3.83428	0.5989	-0.30869
ENSG0000(	SNHG4	5:1389467	0.082503	0.209331	0.472097	0.296203	0.812405	0.311963	0.063731	0	2.44264	0.436282	0.60765	0.817029
ENSG0000(	AP3M2	8:4215294	10.5477	18.1012	4.07715	9.16001	6.30099	16.8107	17.4774	15.2634	19.4597	16.98	0.00705	1.01281
ENSG0000(	MRPS23	17:578347	17.2178	8.69594	19.589	12.8384	12.9706	12.0538	15.2986	13.6486	11.4479	4.50312	0.51485	-0.53471
ENSG0000(	FTH1P5	6:5091271	0.317327	0.430511	0.432258	0.902245	0.092846	1.48054	0.159167	0.406975	1.02302	0.218089	0.80765	0.388893
ENSG0000(	PRR5	22:446685	162.579	45.5824	133.604	189.17	305.955	72.5657	81.6807	86.5684	46.4474	124.597	0.0007	-1.14487
ENSG0000(	ADIPOR1	1:2029408	66.3031	59.3686	89.3961	92.4616	77.6618	65.5012	45.6682	86.7302	59.1701	77.0708	0.36695	-0.28333
ENSG0000(	FAM109B	22:420742	1.7724	2.35258	0.571952	0.451105	0.622188	7.03026	1.99552	2.10842	0.08121	0.429138	0.1855	0.995797
ENSG0000(	IER2	19:131504	765.003	478.734	683.244	1035.48	397.997	268.554	250.689	266.609	476.976	252.875	0.00375	-1.17403
ENSG0000(	MRPL10	17:478232	68.6039	32.6243	150.384	160.185	91.3776	40.0399	48.3683	68.814	111.467	40.8413	0.04535	-0.71452
ENSG0000(	PFKFB3	10:614493	6.34487	7.38489	2.86552	6.56919	1.90773	16.8128	20.3433	19.8711	28.1117	13.1383	0.00005	1.81993

ENSG0000	RP11-686	17:354066	1.1376	0.131166	0.3208	0	1.15375	0.392102	0.997761	0.681276	0	2.80816	0.70445	0.747105
ENSG0000	KRIT1	7:9219896	22.6102	20.6167	15.1694	14.5655	22.4918	28.9208	20.1965	18.626	33.8757	29.5112	0.30135	0.498788
ENSG0000	TRMT44	4:8366281	7.59145	7.14933	6.98119	7.95359	7.28443	3.55748	5.92082	4.13467	12.1294	8.34265	0.72405	0.220702
ENSG0000	FAM124B	2:2243786	0.657915	1.16742	0.174675	0.430128	0	0	0.788306	0.771996	0	0.820686	0.9905	0.016297
ENSG0000	NUP214	9:1311255	15.0683	17.0262	5.73597	2.29619	6.29514	9.72137	10.7561	3.07038	5.6847	12.0231	0.92215	-0.03763
ENSG0000	FTH1	11:619498	1760.35	2383.21	2701.42	3697.02	2949.19	8205.74	2264.62	3125.48	6262.59	1554.35	0.54855	0.527372
ENSG0000	KBTBD6	13:411275	1.32011	0.958229	2.44443	2.27905	2.18191	3.90279	3.38818	3.51178	5.2329	5.20083	0.01955	1.07714
ENSG0000	RP11-18I1	10:102461	0	0	0	0	0.13178	2.52579	0.01819	0	0	0.08026	0.51605	1.89315
ENSG0000	LSM6	4:1461756	45.1398	43.7443	68.5907	53.4967	35.7432	68.8569	70.1149	62.3779	48.5357	14.5838	0.8027	0.095218
ENSG0000	HIST3H2B	1:2284641	0	0.669427	0.523277	0	0	1.97458	0.395085	0	0	0.137035	0.51925	0.909355
ENSG0000	PHACTR2	6:1435368	95.3013	27.1944	31.4658	16.2519	56.3167	37.6842	37.4822	14.209	9.53895	37.2552	0.19565	-0.8603
ENSG0000	SRSF11	1:7020568	145.312	181.066	148.465	137.529	224.426	183.395	187.017	150.3	127.554	268.286	0.9395	-0.02783
ENSG0000	PRDM13	6:9960672	0.399034	0	0.289552	0	0	0	0	0	4.31295	0	0.43775	2.9395
ENSG0000	FCRL5	1:1575133	0	5.58567	0.212873	0.470554	0.132582	0	0	0	0	0.042585	0.23275	-6.54452
ENSG0000	RP11-44F	16:534339	0.853305	1.11116	0.339581	1.72018	0.195395	0.646367	0.099915	0.809056	0.069987	3.90831	0.70435	0.581723
ENSG0000	NUCKS1	1:2057128	6.75929	6.81848	6.85873	5.04401	8.65842	5.16201	9.19184	8.86823	3.53485	10.5094	0.9799	-0.00909
ENSG0000	ITPR2	12:261200	5.42618	9.0036	3.39638	1.86101	3.09208	9.13196	8.67468	2.80041	7.59554	8.1714	0.75585	0.236206
ENSG0000	RP11-148	7:1510538	0.192268	0.072088	0	0.049965	0.140992	2.38883	0.152351	0	0	0.19162	0.25565	2.51162
ENSG0000	KANTR	X:5309370	1.46569	2.12906	0.453596	0.925961	0.268418	0.810583	0.66009	0.151878	0.816575	1.39927	0.8638	-0.18869
ENSG0000	TRIM29	11:120111	0	0	0	0	0	3.19247	0	0	0	0	0.11145	inf
ENSG0000	DTYMK	2:2416757	28.2467	32.4314	48.734	40.3208	54.0549	29.0733	40.5246	49.1382	18.681	44.74	0.54295	-0.25353
ENSG0000	MTDH	8:9764417	42.025	39.3911	29.0343	26.8234	51.3208	60.9402	59.3401	36.6497	28.3759	58.835	0.6322	0.194282
ENSG0000	DNPEP	2:2193735	43.828	27.7079	39.93	40.2417	40.5079	45.3357	50.4681	31.3185	34.9904	38.717	0.9247	-0.03428
ENSG0000	ZFPL1	11:650842	25.8001	27.7974	45.3834	47.465	29.273	29.0809	34.006	34.9752	41.8856	23.7328	0.87325	-0.10517
ENSG0000	CENPA	2:2676428	3.1606	8.7988	0	0.898432	0.2398	15.9103	8.99355	2.09571	3.10243	0.469746	0.4479	1.22313
ENSG0000	PRADC1	2:7322800	16.4466	11.6753	10.8784	17.4171	10.9518	16.7462	13.8583	15.8305	6.59699	6.12128	0.6628	-0.23674
ENSG0000	SMARCC1	3:4758527	19.965	21.4784	14.0216	7.64798	19.2155	11.3325	11.5706	12.9079	13.6354	34.7068	0.876	0.068218
ENSG0000	HERC3	4:8852098	133.172	110.222	175.459	164.842	91.3272	122.879	111.737	106.26	126.29	73.246	0.51695	-0.29486
ENSG0000	CTDSP1	2:2183982	16.3946	19.5932	18.4771	21.3548	28.5411	10.5802	15.2978	28.0643	18.4271	46.5841	0.66575	0.173797
ENSG0000	RP11-214	17:143010	1.45606	3.20268	0.254316	0	0.146564	3.5244	2.59792	0	1.64238	1.03739	0.66935	0.937934
ENSG0000	ZNF532	18:588625	0.637533	1.69852	0.580933	0.099372	0.62218	0	0	1.27235	0	2.57786	0.72975	0.221613
ENSG0000	NAIP	5:7096848	2.07535	3.7768	1.77218	1.16579	1.32543	3.25124	0.971442	2.17465	3.37723	3.24333	0.20515	1.15416
ENSG0000	RP1-34B2	6:2619555	5.20859	3.39632	44.7207	10.5763	16.948	10.2618	3.14432	20.0419	9.47324	1.58872	0.238	-0.9521
ENSG0000	RP11-571	12:576939	10.4418	5.98803	3.86788	11.787	3.51029	5.80145	4.68809	5.31288	8.50985	3.42029	0.87565	0.239871
ENSG0000	SUPT4H1	17:583012	81.2413	53.1654	66.1546	112.743	75.7016	92.7177	62.757	81.5341	100.82	27.8064	0.96025	-0.02484
ENSG0000	TSNAX	1:2315286	45.4864	37.7808	34.6874	34.4819	27.8844	31.4776	27.8077	29.5924	35.9188	18.9236	0.41625	-0.27449
ENSG0000	PTMS	12:674799	22.2407	7.15466	7.77439	8.76006	9.58639	33.8906	99.4928	44.6934	8.70575	14.1303	0.14295	1.69992
ENSG0000	FTH1P11	8:8143943	0.328232	0.147327	0.08366	0.304932	0.169032	0.858145	0.167499	0	1.3961	0	0.6027	1.238
ENSG0000	ALMS1	2:7338575	1.48658	1.54082	0.58063	0.353466	1.04634	0.014382	2.06393	0.870257	0.21199	3.51139	0.4759	0.479018
ENSG0000	FAAH2	X:5728670	6.42046	12.0948	2.06919	2.77319	1.82731	7.49228	4.18478	1.20886	2.82046	1.51064	0.4894	-0.44175
ENSG0000	TEX261	2:7093588	13.4049	15.051	7.6874	7.85506	7.61822	14.1654	14.0936	14.7438	8.71799	19.5126	0.41605	0.417098
ENSG0000	RNF43	17:583012	0.497918	0.611761	0.763088	0.02206	0.120644	0	0.045444	0.356436	0.023666	0.865118	0.4598	-1.01778
ENSG0000	CTD-3094	15:908397	0.577909	0.378583	0.714184	1.19747	0	6.28453	3.42042	1.87774	3.53934	0.600131	0.35155	2.46868
ENSG0000	UBL7	15:744459	56.9958	59.9014	82.2279	67.5328	62.3915	73.8348	47.0572	60.3923	49.9494	35.0653	0.2848	-0.34797
ENSG0000	HMGN3-A	6:7920124	0.640409	0.7284	0.483143	0.069759	0.269821	1.64213	0.270656	0.834805	0.435731	0.545914	0.58545	0.780817
ENSG0000	TMEM191	22:207011	10.1115	11.439	12.4528	16.6076	9.62885	9.91766	15.8864	11.1649	12.0228	6.74541	0.75555	-0.17086
ENSG0000	MICALL1	22:379056	2.53596	2.45632	1.94304	1.59003	3.80012	0.503525	2.79696	1.62282	1.18139	8.87573	0.8353	0.117008
ENSG0000	C5orf63	5:1270425	10.2119	4.78981	8.41063	10.2902	4.56216	4.93039	10.2292	4.39487	6.91382	4.78236	0.6509	-0.33468
ENSG0000	GRN	17:443450	33.6023	79.9986	33.1709	27.6697	34.0226	294.902	33.1837	36.0914	32.3882	42.3582	0.00975	1.02815
ENSG0000	CD44	11:351388	179.767	217.91	92.8393	93.0763	79.8246	318.166	188.695	184.531	169.129	149.986	0.0662	0.654822
ENSG0000	CCL2	17:342552	0	0.055691	0	0.079967	0.127129	135.887	0.947798	38.5263	14.4553	0.880689	0.04965	9.27193
ENSG0000	RP11-332	3:1296477	3.51947	2.25732	2.92318	4.28817	3.05179	2.32439	2.78831	1.51915	2.53225	2.01854	0.7455	-0.49762
ENSG0000	STARD7-A	2:9618485	3.61341	3.80565	4.86528	4.68358	3.90292	2.33422	4.45418	6.87842	1.08904	3.29416	0.7922	-0.23449
ENSG0000	CTC-435M	19:412624	0	0.611878	0	0	0	0	0	0.57297	1.56776	0.131215	0.4466	2.36657
ENSG0000	ADNP-AS	20:508889	1.21901	3.59574	3.26214	0.616457	0.857155	2.92422	0	3.76889	0.788998	0.271734	0.9825	-0.03049
ENSG0000	SPRTN	1:2313327	6.31428	11.3621	9.08655	9.29815	5.64652	9.17229	8.34649	11.7928	20.5989	9.16299	0.53375	0.472205
ENSG0000	AZI2	3:2824158	32.9002	13.0047	29.3168	16.2408	28.2122	51.3191	78.0364	35.8678	10.2012	35.5166	0.3081	0.512938
ENSG0000	ECT2	3:1727506	3.95143	4.54283	1.06889	0.921726	0.889524	6.80622	4.68399	2.36026	1.23859	6.79476	0.06665	1.03302
ENSG0000	RP11-161	16:853633	0.040171	8.26203	0.305176	0	0.321487	1.17691	0	0.07526	0	1.08913	0.2659	-1.49541
ENSG0000	RGMB	5:9876864	0.292323	2.78444	0.185337	0.356329	0.142228	0	0	0.390674	5.26919	0.050839	0.51505	0.94776
ENSG0000	B3GALT6	1:1232264	18.3927	15.5271	21.667	19.9193	24.8146	18.7496	16.7498	17.4721	8.69116	19.2819	0.41725	-0.3116
ENSG0000	MGST3	1:1656308	31.4361	25.1279	73.5612	60.2154	35.0649	76.9209	54.4531	87.8099	53.492	7.42686	0.5739	0.270262
ENSG0000	PIGS	17:284557	31.5388	28.7804	25.3634	32.4733	30.0396	26.469	25.183	23.7838	33.5279	31.0703	0.84745	-0.10522
ENSG0000	PCBD1	10:708822	14.4259	15.4229	18.262	10.3908	11.3041	44.6249	30.0325	30.9663	14.3367	10.5309	0.1123	0.817525
ENSG0000	FAM214A	15:525778	23.5763	22.4253	6.8441	10.2914	8.41464	24.3717	21.284	12.8159	20.4181	25.2996	0.14335	0.572013
ENSG0000	AP003068	11:650842	0.230215	0.102873	0.616341	0.203261	1.68659	0	0.267267	1.21306	0	0.782616	0.90415	-0.23964
ENSG0000	PTPA	9:1290948	4.34741	6.72608	8.20056	6.87636	11.1312	6.17949	5.13832	11.2037	8.62925	18.0277	0.4827	0.423757
ENSG0000	FAM86C2I	11:677852	0.798019	0.347905	0.748743	1.01142	0.669522	0	1.83346	0.375594	0	0.28031	0.5703	-0.72358
ENSG0000	WDR12	2:2028747	7.02712	9.11745	13.8536	8.96319	6.4329	8.91874	7.20362	10.1591	3.52184	7.41955	0.50975	-0.47632
ENSG0000	ZNF543	19:573205	2.87925	2.96544	2.03235	1.50736	1.64829	2.31355	1.16051	4.13555	3.76348	2.0343	0.6019	0.316974
ENSG0000	ABAT	16:867456	4.29789	2.64026	2.02853	2.63319	2.77049	6.30522	1.17837	2.29856	5.07242	8.05261	0.5398	0.46777
ENSG0000	TIMM44	19:792448	13.8317	18.8069	22.9088	20.8513	19.4469	9.95002	22.5335	27.1434	11.7705	13.0714	0.45805	-0.29921
ENSG0000	BAHD1	15:404397	1.1134	2.49175	1.33555	0.509923	2.84513	0.234364	2.38219	3.06161	1.51246	5.58322	0.35385	0.597466
ENSG0000	POR	7:7589919	32.3585	49.7546	32.1931	39.8875	22.9142	48.4091	38.2288	32.2423	33.5458	28.3671	0.7468	0.133611
ENSG0000	ZNHIT6	1:8564942	8.79335	6.61513	7.53588	3.45843	8.97769	4.44875	3.93925	3.12578	4.70335	11.6534	0.3408	-0.47035
ENSG0000	CDCA8	1:3769241	4.31304	8.47823	1.99505	1.54879	0.73059	3.53137	8.40336	1.01017	0	3.18027	0.96695	-0.03201
ENSG0000	TIGD5	8:1435734	0.358747	0.253705	0.512077	0.561159	1.38159	0.267159	1.6715	0.821932	0.413002	2.03849	0.6582	0.612849
ENSG0000	CCDC6	10:597887	2.44231	2.8632	2.00435	2.05874	2.22806	3.41373	1.7063	3.55149	0.818709	4.91059	0.76065	0.145203
ENSG0000	TRADD	16:671541	109.159	69.4698	176.831	196.687	190.962	89.0126	108.48	121.575	178.528	108.689	0.4404	-0.25805
ENSG0000	ZNF204P	6:2735782	0.380803	1.59933	1.05655	0.3867	0.45063	0	0	0.178037	3.89061	0.208135	0.87405	0.139274
ENSG0000	PIK3R2	19:181531	6.46182	6.01456	3.876	2.82404	6.92752	2.38659	2.82263	1.72124	1.87257	3.43254	0.3852	-1.05751
ENSG0000	RP11-122	1:1613994	1.11356	2.55586	0.438331	1.35397	0.758773	1.06579	2.89802	1.08732	0.570191	0.290136	0.94005	-0.12442
ENSG0000	IL1RAP	3:1905140	4.42922	2.40397	1.1303	0.854615	1.71716	1.72665	1.26655	1.2756	1.08155	1.92161	0.56645	-0.40369
ENSG0000	IGLV3-21	22:227116	0	31.5678	0	24.2908	3.16992	0	0.174541	0	0	0.08028	0.1125	-7.80029
ENSG0000	RP11-806	17:783604	0.479252	0.174227	0.116989	0	0.161552	0	0	0	2.98784	0.090968	0.3317	2.0355
ENSG0000	RP11-115	2:1578767	0.738511	0.714061	0	0	0	1.04101	1.50582	0.190704	0	0.270744	0.59875	1.31975
ENSG0000	SLC4A8	12:513512	0.311019	0.89533	0.452287	0.0402	0.110144	0.090268	2.38206	1.64131	0.187255	0.364112	0.0578	1.57225
ENSG0000	CDKN2C	1:5096074	7.34272	14.8654	7.86067	9.59725	6.32766	19.048	13.962	9.2236	1.66131	4.34498	0.89895	0.081101
ENSG0000	RP13-516	17:822283	0.783323	0.901408	0	0	0	0.774451	1.03363	0	0	1.43506	0.57815	1.09431
ENSG0000	LINC0150	9:1293322	0	0.533202	0	0	0	3.354	0	0.401439	0	0	0.3743	2.98397
ENSG0000	11-Sep	4:7694970	17.162	13.9524	10.0655	8.12412	27.8127	24.3091	44.7196	23.5494	30.3659	33.614	0.02535	0.980874

ENSG0000	RBM4	11:666165	147.496	147.004	165.241	163.989	136.081	122.659	219.96	164.171	104.003	94.2594	0.74755	-0.16448
ENSG0000	NCOA6	20:345605	6.3077	7.53533	4.0412	1.91002	7.5081	4.90331	5.31842	4.95931	7.6993	12.4637	0.46165	0.418533
ENSG0000	AP000295	21:332299	2.97169	3.18109	0.709799	1.09465	0.412205	1.29646	0.590184	1.23714	2.6625	0.308188	0.4588	-1.30733
ENSG0000	SLC16A7	12:595960	10.209	8.59746	6.04794	5.45067	8.86326	5.18258	5.10061	5.71488	4.52465	5.92733	0.3629	-0.62581
ENSG0000	LL22NC03	22:222937	0.501125	1.24961	0.777433	0.154733	0.432951	1.25513	1.643	0.190326	0	1.68769	0.7024	0.770695
ENSG0000	TMEM135	11:870378	8.2225	7.89426	2.9451	5.15315	3.39624	13.6503	10.489	4.87096	7.20206	4.35801	0.6564	0.257264
ENSG0000	ZNF654	3:8805194	6.46482	5.83667	2.88421	2.45769	2.3135	5.24403	2.55098	3.16761	3.04958	5.88037	0.88855	0.174224
ENSG0000	UST	6:1487473	2.56382	1.38009	0.722449	0.333172	1.33423	3.18312	0.236574	1.83881	0.407536	1.6421	0.7251	0.282065
ENSG0000	TRPT1	11:642066	28.5127	40.5206	28.6293	30.4588	14.161	47.879	34.2874	37.0937	36.6241	22.6722	0.5409	0.325565
ENSG0000	MSH5-SA	6:3173994	1.83312	1.04239	0.196901	0.742143	0.506253	0	2.05839	0.521836	0.927221	1.57941	0.9158	-0.15016
ENSG0000	GNAO1	16:561913	0.415225	0.129135	0.725196	0.339318	0.624004	1.50803	1.08175	1.78827	2.31285	1.70474	0.02325	2.22647
ENSG0000	DALRD3	3:4891778	14.5335	12.4259	25.6836	26.3842	22.1683	8.01121	17.6462	20.242	15.2142	19.0063	0.4081	-0.4227
ENSG0000	FLJ37453	1:1583447	4.16522	4.7689	5.28022	3.92953	2.42384	1.42989	3.43843	5.94205	2.8358	3.47019	0.8325	-0.30738
ENSG0000	C12orf65	12:123152	26.6345	30.0545	47.4983	36.9788	74.287	36.9085	55.2544	44.9746	28.5789	31.4331	0.63315	-0.2724
ENSG0000	SLMAP	3:5775544	18.4829	20.8283	9.46548	13.3039	11.3116	23.9509	12.9084	7.74594	13.9841	15.2998	0.81015	-0.11494
ENSG0000	ALDH18A	10:956059	33.5965	27.6866	22.9868	26.3665	14.9665	18.2289	8.42602	14.5692	35.5328	21.2212	0.25235	-0.4166
ENSG0000	CRYBB2P1	22:254481	15.9066	32.3061	28.2011	35.5193	23.5089	21.8905	45.9789	40.4018	21.7051	37.6712	0.67575	0.296035
ENSG0000	RP11-61K	13:198638	3.83406	4.22734	3.42051	2.27926	3.48777	3.38595	3.72308	6.27013	2.68792	1.88643	0.98775	0.011443
ENSG0000	SPON1	11:139626	3.2903	2.54163	4.40826	4.85769	3.48058	1.54026	0	1.12213	3.35338	3.927	0.1874	-0.93591
ENSG0000	LCN2	9:1281490	0.462254	0	0.755738	3.52262	0.662387	0	0	0	0	1.29569	0.33645	-1.87638
ENSG0000	TMIGD3	1:1114833	0	0.69175	0	0	0	2.27282	0	0	3.38423	0	0.1981	3.29343
ENSG0000	CCDC24	1:4399135	1.22842	0.784673	0.142241	0.912503	0.552317	0	2.3311	1.29435	0.117701	0.121233	0.9787	0.02239
ENSG0000	ST20	15:798335	11.1576	6.68843	7.14283	4.61991	6.88023	19.1849	13.9634	9.64642	17.791	4.81604	0.60225	0.734405
ENSG0000	NEIL1	15:753467	8.71631	3.72325	4.26733	5.65983	3.73053	3.27994	2.36798	2.92343	7.8914	1.64865	0.3435	-0.61812
ENSG0000	MIR155H	21:255619	4.26045	1.69322	3.05959	5.98492	2.72555	28.8553	65.7095	34.68	18.0189	13.3853	0.0005	3.21626
ENSG0000	DCLRE1C	10:148788	20.0896	21.6222	16.6926	13.1401	12.6173	18.1065	21.4637	16.1258	17.4375	27.8677	0.6373	0.178297
ENSG0000	KIAA1328	18:362977	7.57768	5.4116	10.5239	8.60413	5.00282	3.73804	6.34357	3.86859	8.38175	7.94937	0.7715	-0.31227
ENSG0000	RP11-705	12:961728	0.025877	0.030137	0.757643	4.05944	0.952367	0	2.55952	0.051296	0.142183	0.226961	0.6838	-1.12985
ENSG0000	HBA2	16:172846	0.882956	0.143739	141.812	2241.84	1.03842	3.88389	5.46302	42.7152	727.837	1.21573	0.3104	-1.40769
ENSG0000	USP3-AS1	15:635045	1.82237	2.70918	0.909344	0.851206	0.424264	0.814657	0.814421	1.23377	4.64353	0.801893	0.8022	0.206583
ENSG0000	SREK1IP1	5:6471814	18.9489	16.6569	12.0131	8.5606	14.6655	25.3828	26.6787	9.75526	2.50574	8.71596	0.8882	-0.12862
ENSG0000	TCHP	12:109900	17.3101	19.9904	9.7987	6.65879	16.4842	12.7521	13.4126	8.23685	7.86133	14.6965	0.69975	-0.3166
ENSG0000	XPR1	1:1806320	2.08237	1.95741	1.08611	1.4921	1.71085	1.18659	2.41537	0.995085	2.12916	2.26065	0.98135	-0.01432
ENSG0000	RP11-799	17:351478	0.816864	5.96868	0.284172	0	0	0	0	0	0	0	0.1518	#NAME?
ENSG0000	CTD-2017	15:648418	0.435695	4.23072	0.676523	0	0.354462	2.75076	4.3853	0.150834	0	1.27507	0.7335	0.621398
ENSG0000	TNK2	3:1958633	3.35664	5.58887	2.29086	2.50667	3.97457	7.96368	12.0173	4.5229	0	10.0834	0.2751	0.764831
ENSG0000	RPL23	17:388478	234.558	178.625	999.171	373.813	126.005	290.68	261.678	648.133	380.103	35.3264	0.5353	-0.27376
ENSG0000	TMEM203	9:1371916	77.5406	56.7971	91.313	65.6263	91.7477	58.4199	61.3416	58.2642	51.6079	58.4231	0.1882	-0.44336
ENSG0000	RP11-366	4:1649544	4.32567	1.49462	1.47084	0.908541	2.61458	2.13743	2.21419	3.13543	0	1.34548	0.9138	-0.15849
ENSG0000	ZDHHC11	5:795605-	0.298488	1.12308	0.582442	1.12682	0.034116	1.20341	0	0	2.70329	0.346986	0.68805	0.432425
ENSG0000	DCAF16	4:1780065	7.48331	6.17543	10.7499	8.66216	3.89439	10.3603	5.74077	6.13823	8.08833	8.13578	0.9779	-0.02684
ENSG0000	CTC-338	5:1811919	3.44168	1.74681	0.113441	2.76886	0.474907	7.2652	1.22285	1.30896	0.388949	0.151532	0.8739	0.248533
ENSG0000	CRIPAK	4:1391551	0.308893	2.59485	0.006807	0.08656	0.230225	0.428619	1.29263	0.070892	0.010144	0.707156	0.29815	-0.25937
ENSG0000	BRD9	5:795605-	30.5377	22.8862	31.9015	41.628	29.5974	24.0431	19.3097	23.8649	33.4351	35.9843	0.64245	-0.18219
ENSG0000	KIAA1324	7:8687690	2.39609	1.14399	1.53674	4.44426	0.434092	1.10517	0.233392	0.715075	3.19335	1.55993	0.39785	-0.57693
ENSG0000	TNFRSF9	1:7915893	0.999535	1.48057	0	0.288087	0.859464	14.1979	91.4546	15.2457	2.1448	7.1405	0.01	5.20708
ENSG0000	SCARNA1	17:770867	1.13188	0.850549	0.635004	0	0	0	0	1.20744	0	0	0.5244	-1.68914
ENSG0000	TIGIT	3:1142769	23.7636	53.2911	6.98687	5.62388	3.63286	53.3521	139.058	21.2174	8.60231	14.0012	0.002	1.42921
ENSG0000	CRY2	11:458471	6.31154	10.1218	6.66427	6.20346	5.63574	5.45416	3.08124	4.09731	7.72636	6.3689	0.91535	0.050597
ENSG0000	LASP1	17:388698	33.1552	26.2494	15.5062	8.80073	22.5825	25.7485	31.7849	24.0672	12.7154	56.7456	0.1895	0.457867
ENSG0000	RP11-300.	14:880051	1.14891	0.202915	0.987114	0.700379	1.23967	0	0.386089	0	0.547138	0.2365	0.2454	-2.46353
ENSG0000	SLC51A	3:1962114	0.728536	0.763537	0.488743	0.179296	0.513183	1.92231	0.559107	0.951287	0	1.78252	0.1827	0.965788
ENSG0000	FBXO45	3:1965541	4.76915	4.29032	2.01693	1.71431	3.10071	6.18626	5.71762	3.76608	2.08982	5.56108	0.5277	0.407442
ENSG0000	RP11-440	4:1745237	0.720863	0	2.38264	1.49754	0.602264	0	0	0.059974	2.39643	0.504575	0.7577	-0.76319
ENSG0000	CTSH	15:789210	48.8458	27.9164	68.9937	80.5716	70.5707	265.284	29.0796	113.496	187.166	62.5917	0.0099	1.04864
ENSG0000	FO538757	1:184922-	1.79528	3.55111	2.44983	1.72602	2.11645	2.72777	3.74428	4.00298	2.54806	3.6726	0.53785	0.769318
ENSG0000	CERS2	1:1509262	82.2935	85.4124	62.2804	63.6712	62.1546	69.8758	72.1057	65.0854	46.1462	68.9298	0.7433	-0.12343
ENSG0000	NHLRC3	13:390383	9.61877	11.9398	10.0678	7.96271	7.36049	8.62435	12.8094	16.076	18.7891	17.6736	0.11645	0.615978
ENSG0000	FTCDNL1	2:1997605	0.219235	0.36152	1.81812	2.58325	0.124125	3.87169	3.19184	0.55135	0	0.336615	0.7843	0.403062
ENSG0000	RP11-182.	15:845978	1.27725	0.555265	0.439436	0	1.02647	4.27133	1.56135	2.03767	0	0.2182	0.3489	3.06425
ENSG0000	PPP1R13L	19:453796	0.334795	0.925001	0.312509	1.36719	0.672288	1.40512	0.087819	0	0	2.66557	0.6436	0.575836
ENSG0000	SNAPIN	1:1536586	75.6726	63.0514	90.9605	67.8583	54.549	130.116	122.342	64.7215	69.1645	35.0036	0.7711	0.205937
ENSG0000	MTND3P1	2:1200130	0	0	0	0	0	0	1.2373	0.721194	0	0.175395	0.1498	inf
ENSG0000	ZBTB24	6:1094440	4.06817	6.41247	2.22128	2.99698	3.18922	2.76292	5.10205	2.1714	3.27697	4.86153	0.89605	-0.13236
ENSG0000	BLOC1S3	19:450714	5.66584	4.91817	22.5214	8.56016	22.3812	13.1589	5.30805	18.3872	8.28363	27.1082	0.9235	0.093617
ENSG0000	ZNF799	19:123791	4.23188	2.90878	4.22568	1.08542	1.61356	1.8807	2.51963	0.701608	0.08632	2.05399	0.4636	-1.0617
ENSG0000	RP11-816.	3:1697669	0.190484	0	0	0	0.194055	1.73042	0	0.748548	0	0.169132	0.3946	2.66082
ENSG0000	CNNM4	2:9676090	3.07998	2.95811	0.938778	1.0528	1.20635	4.42631	1.8936	2.59359	1.15194	2.61448	0.51155	0.394461
ENSG0000	PLEKHG3	14:647041	21.4903	5.62881	9.95925	9.23223	19.9077	3.03734	0.143884	1.34576	1.25958	12.736	0.00455	-2.25933
ENSG0000	NFE2	12:542309	2.69547	10.7955	2.24607	3.80164	0.477463	0	0.060076	0.504715	0	0	0.1191	-4.90774
ENSG0000	GRK4	4:2963607	1.93678	0.596966	1.51699	0.624037	1.08213	1.01856	3.65898	0.133088	4.90479	0.357459	0.3804	0.708764
ENSG0000	GGPS1	1:2351311	33.7274	35.5433	48.6644	34.1853	34.9621	24.6238	21.8807	26.4944	22.2215	16.6885	0.29255	-0.81156
ENSG0000	FNTB	14:649143	5.05202	6.69927	6.79563	7.14445	3.60858	4.06483	3.2714	1.12176	0	3.93391	0.16125	-1.4031
ENSG0000	CD84	1:1605370	8.60471	15.3388	17.9792	5.17513	4.95779	20.4651	17.9723	14.9496	7.15414	21.1857	0.41655	0.528686
ENSG0000	CDC6	17:402876	2.24816	1.72048	0.403491	0.441554	0.298412	2.57212	1.07373	1.17522	0	1.58883	0.54765	0.434304
ENSG0000	OAZ1	19:225225	844.15	764.951	1402.88	1254.83	1180.89	1333.19	1272.91	1310.01	1313.93	398.333	0.953	-0.02712
ENSG0000	RPP14	3:5830624	3.76772	4.60733	5.43533	3.35838	2.82485	4.246	2.62685	4.07496	3.0091	4.50592	0.9756	0.021063
ENSG0000	BNIP3P17	19:200779	0	0.079798	0	0	0	1.96472	1.56686	0	0	0	0.22135	5.50137
ENSG0000	KTI12	1:5202013	22.2941	16.8222	32.0447	33.4909	20.3695	19.218	11.4716	19.6334	19.9637	15.5098	0.5181	-0.52652
ENSG0000	REL	2:6088152	4.4972	6.48172	8.43632	5.71961	7.2038	4.87632	7.80879	11.7048	11.9398	14.7211	0.2972	0.504041
ENSG0000	C11orf71	11:114391	10.8463	4.87845	16.5593	8.34769	7.811	9.34181	7.81133	3.67284	14.2648	6.51234	0.86275	-0.20177
ENSG0000	DNAJC19	3:1809837	40.8457	31.8577	50.8271	53.7444	29.0182	45.29	48.2664	38.183	48.3997	13.9246	0.9034	-0.04818
ENSG0000	CELSR2	1:1092500	0.435147	0.396231	0.57354	0.422008	0.596753	0.203689	0.268374	0.106544	0	0.726813	0.26145	-1.43863
ENSG0000	FAM50B	6:3831932	28.9664	20.8675	16.5315	14.4345	30.6525	28.8389	15.9496	26.7233	19.8161	28.7779	0.7803	0.122191
ENSG0000	ZBTB1	14:643880	29.4344	29.5612	15.5989	38.0313	11.414	27.5476	29.7734	20.9264	19.4533	20.8332	0.86065	-0.10817
ENSG0000	MSMO1	4:1653276	21.537	17.1462	17.2878	16	15.4843	32.5794	47.9989	27.0027	27.4356	15.6591	0.0303	0.794906
ENSG0000	AGBL2	11:476595	6.00975	10.1028	2.39416	4.84244	1.49862	3.87188	2.94588	0.931561	2.52198	3.73712	0.55105	-0.46471
ENSG0000	RP11-424	Y:1969194	1.06253	2.27918	0.138354	0.381531	0.152755	0.312488	1.93149	0.16839	1.48664	0.665476	0.42525	0.418877
ENSG0000	CCNH	5:8726788	72.405	46.9565	52.1638	49.1559	42.0837	72.7751	60.7468	75.5975	82.9582	34.8626	0.40635	0.341874
ENSG0000	NAGK	2:7093588	38.789	55.9826	35.5971	48.7373	67.8935	79.7831	60.1711	40.47	50.063	36.5012	0.7998	0.121938
ENSG0000	TSPAN2	1:1150480	2.52867	4.53697	1.70034	2.71039	0.550724	3.51668	5.3434	5.45649	8.63137	0.891426	0.02505	1.16599

ENSG0000	RP11-109	14:730579	0.222724	1.3089	0.52079	0.779974	0.122434	0	0.0578	0.066858	0.16714	0.720791	0.27055	-1.76672
ENSG0000	C15orf41	15:365796	3.86078	6.24798	5.36285	4.07328	4.17858	2.6958	1.86645	5.5708	8.90991	8.38121	0.7908	0.200333
ENSG0000	RAB20	13:110523	0.934632	2.71341	0.122886	1.0191	0.310959	11.1825	0.051739	1.54397	0	1.13101	0.3388	1.44345
ENSG0000	SIRT3	11:215457	6.68773	10.2518	11.4086	7.26737	8.23327	2.85086	8.66478	10.745	7.20968	11.1135	0.88075	-0.10478
ENSG0000	PTGES3	12:566633	164.248	180.107	140.575	128.861	153.407	201.982	192.518	125.256	122.396	104.674	0.87975	-0.04749
ENSG0000	KIF1B	1:1021080	1.5754	2.64808	0.590322	0.524211	1.30079	2.75954	2.40402	0.890222	0	2.64189	0.7023	0.276682
ENSG0000	AMN	14:102922	0.849764	1.11334	0.223128	0.873617	0.256295	0	1.89337	2.61863	1.5378	0.301176	0.34325	0.938507
ENSG0000	TNFRSF1B	1:1216694	147.824	201.465	59.6201	67.0807	63.2373	222.983	255.294	99.2722	83.3955	140.818	0.11205	0.585125
ENSG0000	ZNF197	3:4455519	4.0685	1.98637	3.04385	0.581513	1.97704	2.19993	2.62255	3.33871	1.65125	2.58849	0.97205	0.031338
ENSG0000	UPK3A	22:452849	0	1.4551	0.07316	0.671405	0.040083	0	0	0.330111	0	0.373077	0.50735	-1.32424
ENSG0000	PGK1	X:7806518	152.801	131.707	192.113	135.275	162.241	355.964	331.852	291.304	177.2	152.399	0.49505	0.45529
ENSG0000	HADH	4:1079897	24.1709	24.1427	17.2182	20.9584	22.0519	18.9981	22.981	9.56918	14.3143	7.86736	0.15125	-0.57266
ENSG0000	BLCAP	20:374924	36.5405	45.0197	58.1166	51.8385	43.7832	8.84111	26.631	37.3745	25.7758	40.5179	0.0208	-0.79057
ENSG0000	ETV3	1:1571211	7.93713	3.04635	4.57349	22.8276	5.55419	2.23028	2.71801	5.49741	1.72378	8.26076	0.0553	-1.24312
ENSG0000	CTD-2105	19:553389	1.12682	0	2.40998	0.384629	0.152309	0	0	0.163327	0	0	0.2645	-4.372
ENSG0000	HIST1H2A	6:2714712	3.0154	4.67318	0.834168	0.341103	0.131266	3.32835	2.9561	0.896545	1.57859	0	0.98275	0.000169
ENSG0000	GPAT3	4:8353591	1.06519	2.46127	0.341119	2.06513	0.380639	2.67972	0.07285	1.13733	1.21315	1.65992	0.75895	0.270645
ENSG0000	DCUN1D2	13:113455	4.97149	7.75872	4.78193	4.15766	2.24563	8.41795	0.716379	8.32446	9.38759	6.2284	0.4416	0.426128
ENSG0000	DNAJB1	19:145147	156.603	147.995	250.27	502.343	164.408	143.007	80.8829	147.879	276.452	242.74	0.3098	-0.56325
ENSG0000	LTBP3	11:655388	19.4773	29.9636	15.4023	16.7651	28.3113	26.8002	28.3684	24.2536	7.74161	25.6245	0.86345	0.087238
ENSG0000	PCNP	3:1015740	159.612	121.325	100.664	99.8905	62.8401	111.848	127.744	92.0958	88.6352	67.2005	0.6469	-0.14648
ENSG0000	LZTS2	10:100996	9.21393	8.65391	3.53152	3.30107	4.82131	9.47004	7.00895	2.89973	3.90608	7.46466	0.901	0.057806
ENSG0000	RP11-481.	16:585154	1.40727	0.614742	0.188362	0.835771	0.784797	0	3.71724	0.42873	1.42253	0.318234	0.6599	0.673633
ENSG0000	LBR	1:2254015	110.873	106.065	38.9002	64.6791	31.3379	60.3133	50.9354	44.9234	51.4827	49.8544	0.3046	-0.33015
ENSG0000	TCAF2	7:1436209	2.23669	2.39523	1.78058	1.43432	1.61157	2.45432	2.91581	3.71458	2.15948	5.46847	0.1928	0.920452
ENSG0000	ITGA4	2:1814572	77.0956	109.006	68.042	67.8111	72.0669	52.6052	66.7435	48.5812	78.1755	90.4497	0.51655	-0.25897
ENSG0000	TREX1	3:4844670	26.5302	46.6917	38.0243	38.6358	47.7612	31.9764	91.9023	56.5704	66.5843	20.2718	0.4256	0.478658
ENSG0000	RPSAP15	X:8770334	0.736739	0.571482	0.423909	0.52843	0.074141	0.60786	0.404583	0.25727	0.195992	0	0.7157	-0.72397
ENSG0000	EIF4HP2	22:306937	0.28524	0.321765	0.74091	0.754385	0.99841	0	0.074005	0	0	2.70849	0.89525	-0.01273
ENSG0000	WDR89	14:635970	12.0292	12.8308	7.64486	3.00179	5.3021	10.9567	8.19663	7.14851	4.12887	10.8043	0.95295	-0.02728
ENSG0000	ANKRD49	11:944155	39.3194	38.7605	30.88	30.7229	25.9882	40.4303	56.5667	34.8385	37.6584	25.1084	0.8283	0.106869
ENSG0000	KIZ	20:211259	16.43	11.0505	16.235	14.5457	10.3627	13.797	13.6841	10.037	7.66982	8.47794	0.35595	-0.39174
ENSG0000	CTB-167B	7:8820511	0	0.538731	0	0.310792	0	1.4565	0.407058	0	1.42552	0.197879	0.3374	2.11603
ENSG0000	C2CD2L	11:119096	7.20489	14.4581	3.22005	2.42114	4.20945	4.63829	7.57257	6.67517	4.33646	11.6079	0.8895	0.139509
ENSG0000	SOCS1	16:112496	30.647	27.4915	32.1875	58.776	102.258	132.284	366.599	190.489	100.973	124.101	0.0009	1.60583
ENSG0000	PANO1	11:790474	0.389752	0.611168	0.361322	1.96254	0.199198	0.130082	0.66934	0.398845	0	0.710271	0.52505	-1.00078
ENSG0000	HSBP1L1	18:799645	3.46969	13.4231	8.42229	7.84337	4.73155	7.21805	1.55676	6.62427	8.93842	5.21197	0.8	-0.27095
ENSG0000	ZNF749	19:573634	17.1174	14.9043	9.41541	22.7796	10.5334	27.1477	23.1479	9.71942	16.4319	6.6429	0.94495	0.102525
ENSG0000	HHEX	10:926899	1.59023	15.3046	1.70314	3.07505	1.27944	7.94317	1.6046	1.05858	3.19249	0.456654	0.4133	-0.65206
ENSG0000	ARRDC3	5:9136872	29.9164	26.3152	18.736	10.1424	9.41134	17.5331	6.98748	13.7098	26.1503	18.8252	0.5288	-0.21118
ENSG0000	ATG14	14:553663	2.41964	2.30225	2.18611	3.08065	2.52469	1.80145	1.48174	2.37722	2.08474	3.13053	0.596	-0.28916
ENSG0000	COMMD4	15:753358	36.2581	21.1918	41.599	45.997	41.17	66.5503	41.3509	28.9319	27.4113	14.5437	0.66865	-0.18117
ENSG0000	TARDBP	1:1101234	68.7731	87.3268	78.0679	53.7957	43.4674	74.1402	65.3099	85.1243	41.8799	80.7604	0.5354	-0.28951
ENSG0000	RP11-274	7:1286536	0.69732	0.302862	0.396053	0.24319	0.379174	0.213129	1.12025	0.429981	0	0.534194	0.73035	0.511187
ENSG0000	EZH1	17:427002	13.6065	22.0793	11.4578	10.1714	14.7361	10.4725	14.6586	14.5516	22.8917	21.7182	0.56365	0.192181
ENSG0000	NDOR1	9:1372056	5.21086	5.89448	2.36216	3.03646	4.89494	3.82438	5.12075	2.65807	1.22195	6.70316	0.80215	-0.18563
ENSG0000	WDR4	21:428430	4.73529	9.89522	6.93165	4.77952	9.98701	4.15395	4.52857	6.6269	14.9019	4.34095	0.98535	0.012918
ENSG0000	RRN3	16:149745	29.7857	19.6045	10.0958	9.35665	8.86906	9.60506	16.7244	16.0171	11.1348	11.852	0.74265	-0.19295
ENSG0000	CTD-2666	19:121950	0.638656	1.87642	0	0	0	0	0	0.62291	0	1.11787	0.9547	-0.12947
ENSG0000	ALG10B	12:383165	0.568279	0.644595	0.602504	0.432842	0.136365	0.249	0.666967	0.367046	0.093348	0.440183	0.3278	-0.83009
ENSG0000	RP11-104	9:3980736	0.645301	0.393438	0.653906	2.34694	0.074412	2.75523	1.4574	0.617211	2.47725	1.19054	0.52515	0.956619
ENSG0000	ECHDC1	6:1272887	58.2497	42.2478	47.515	49.7087	39.6605	65.325	38.7049	45.8592	48.2127	33.7265	0.81445	-0.07526
ENSG0000	CATSPER3	5:1349049	0.158067	1.62223	0.09557	1.75089	0.245996	0	0	0	0	1.39384	0.49285	-1.60437
ENSG0000	ENOSF1	18:596987	24.5056	22.2019	14.5005	13.8266	12.9148	33.7277	17.1587	12.2894	7.63095	12.9489	0.9008	-0.06763
ENSG0000	BIN2	12:512810	402.578	344.427	430.651	496.913	310.108	201.812	177.243	206.124	234.246	241.19	0.01575	-0.89457
ENSG0000	ANXA7	10:733751	79.1846	77.1458	86.4231	79.7798	73.8875	86.5968	95.0731	72.9117	50.2998	61.3454	0.34635	-0.28682
ENSG0000	TUBB2B	6:3224260	1.02361	1.98087	0.765708	2.2025	1.73088	2.21254	3.49617	2.2779	6.52211	1.48784	0.49675	1.01008
ENSG0000	RAD50	5:1325414	34.1345	47.9374	14.8808	10.7499	18.5003	34.7406	28.8761	14.8754	8.99366	23.0988	0.43905	-0.31379
ENSG0000	ATP10D	4:4748528	6.60459	4.05753	3.45907	2.68743	5.02316	3.11915	11.3802	4.31338	4.74071	15.8927	0.5223	0.823247
ENSG0000	RRP1B	21:436595	1.21092	1.70691	11.2859	7.73089	19.0652	0.684577	0.832247	7.66486	8.97137	25.1459	0.72535	-0.15123
ENSG0000	EIF3FP3	2:5825143	0.832557	1.38523	0.628995	0.457248	0.639169	0.794559	0.930202	0.497898	0.748142	0.128292	0.90315	-0.26099
ENSG0000	RP11-91K	3:1334908	0.067819	0	0.114452	0.788134	0	0.316777	0.214239	0.129698	2.34677	0.964796	0.2105	2.06928
ENSG0000	ZCCHC6	9:8628773	30.8249	26.0087	7.83523	10.6235	11.7153	26.6364	23.4664	13.5497	17.8276	23.5569	0.42635	0.266358
ENSG0000	PHF11	13:494956	91.8553	82.7135	72.7262	62.5846	52.3837	81.6575	163.59	81.1985	104.868	48.7365	0.0783	0.552139
ENSG0000	PPIP5K2	5:1030859	22.0971	17.9639	7.75393	8.61214	5.78356	11.5285	11.6507	10.1275	16.8773	12.2063	0.84255	0.102264
ENSG0000	SNN	16:116684	7.75974	30.7024	2.37944	6.07222	0.937331	10.3791	3.6246	4.27204	3.32799	2.50296	0.3827	-0.93128
ENSG0000	B4GALT2	1:4397894	1.26149	0.988918	0.848779	0.690625	1.25507	0.640807	3.62013	2.3944	0.39235	0.787097	0.6275	0.488531
ENSG0000	TEX30	13:102765	31.4556	18.476	29.4447	37.1137	16.7017	35.7718	32.2068	28.2849	47.2299	6.70319	0.68955	0.18677
ENSG0000	COPA	1:1602892	53.6779	60.7856	20.6488	28.0109	15.8443	49.5472	29.8302	31.0169	18.5029	25.4107	0.69885	-0.16837
ENSG0000	VCP	9:3505606	163.875	133.321	113.558	129.68	106.443	101.429	109.464	88.2476	115.871	111.816	0.3191	-0.32399
ENSG0000	RP11-611	12:688081	4.34392	5.81427	2.58469	1.02757	2.25615	5.31056	5.33211	1.61134	2.05029	0.640047	0.815	-0.29993
ENSG0000	PRR5L	11:362962	21.0884	14.5499	21.374	10.1904	39.1955	6.8798	10.7888	10.7146	7.24194	20.6816	0.0913	-0.92444
ENSG0000	ZMYM2	13:199586	15.3296	12.8092	9.01856	9.65237	13.8118	7.99969	14.1375	15.5264	18.2857	28.9162	0.58715	0.287571
ENSG0000	FUT10	8:3337082	5.78358	4.60205	3.96459	5.75005	3.85721	3.61887	4.88357	3.16982	0.013032	2.63781	0.32365	-0.77884
ENSG0000	RP11-823	5:2134183	0.288111	0.774542	0	0	0	0.017538	0.618524	0.003466	1.28526	0.371036	0.51395	1.70594
ENSG0000	RP11-337.	9:2684068	0.637755	0.406361	0	0	0	1.92129	0.456748	0	0	0	0.6105	1.24678
ENSG0000	RP11-383	7:1390435	1.4831	4.8996	1.13644	1.99728	0.803199	2.71445	4.80603	0	4.54344	2.99371	0.62535	0.642594
ENSG0000	GLTSCR1	19:476075	0.66978	1.44536	0.672985	0.27331	1.2937	1.4547	1.18967	0.625833	1.34961	2.56573	0.5357	0.705633
ENSG0000	PAXIP1	7:1549284	2.70049	4.52498	1.47821	1.50602	2.12249	1.56339	2.60984	4.28147	0.251817	3.71397	0.94035	0.058754
ENSG0000	HEATR5A	14:312486	4.13611	2.78869	4.438	1.87356	1.14321	23.6	4.67946	2.93412	15.1734	3.08298	0.12285	1.88174
ENSG0000	C16orf72	16:909164	8.01603	4.17921	4.62117	1.87633	5.18713	5.56489	4.91929	8.07235	1.83515	11.8661	0.63805	0.241667
ENSG0000	BUD31	7:9932589	123.433	81.9203	175.392	182.239	150.387	177.371	190.487	189.689	157.121	39.0407	0.9581	0.03889
ENSG0000	ZRANB2	1:7104885	68.2038	85.536	40.6132	42.951	48.4663	71.172	75.5024	60.8137	36.7122	79.5712	0.9062	0.04886
ENSG0000	TCP11L1	11:330394	4.57204	2.75449	4.64122	6.19757	3.12365	4.61287	4.55007	6.17761	3.77006	3.25695	0.91345	-0.08591
ENSG0000	RP11-886	19:844456	0.654132	0.173712	4.02532	3.34935	3.32455	0.417895	0.634529	5.12464	3.46462	1.81117	0.97875	-0.04116
ENSG0000	KLRC2	12:104123	0.263777	0.142165	1.8511	24.0599	1.02478	0.220695	41.1393	5.70044	39.247	2.65266	0.1927	1.80965
ENSG0000	TMSB4Y	Y:1370356	0.77392	1.78846	3.12378	1.24945	1.68555	0	1.11154	2.11537	0	0.182832	0.2399	-1.36444
ENSG0000	PDIA5	3:1230670	3.04547	1.03926	5.95522	6.69512	3.76566	3.19575	2.40037	1.96618	14.2793	2.4508	0.88535	0.165272
ENSG0000	USP10	16:846999	68.6522	70.4498	40.4848	64.9366	45.9891	36.4489	39.5628	30.0858	56.7025	52.0873	0.15125	-0.45454
ENSG0000	EFHC2	X:4414788	2.0057	0.460816	1.65877	1.51975	0.815286	0	0	0	0	0.522161	0.1709	-4.04833
ENSG0000	BTG2	1:2033054	82.04	98.2505	111.263	278.672	64.6275	67.4964	44.8769	115.415	126.072	115.091	0.1773	-0.50989

ENSG0000(	ZNF558	19:880616	7.97068	6.82657	1.82257	3.05255	1.56022	6.801	2.73677	3.86607	2.59909	4.15946	0.7079	-0.19098
ENSG0000(	PALB2	16:236031	5.21768	6.87606	2.32811	2.01574	2.10192	5.41983	3.87863	1.28801	1.80756	2.51816	0.99215	0.006035
ENSG0000(	RP11-577	7:1027489	0.676603	0.049471	0.821924	2.03279	0.577966	0	0	0.804845	0	0.473081	0.3791	-1.77746
ENSG0000(	RP11-295	1:2315223	2.58538	1.13518	1.78776	5.02629	1.71904	14.29	1.27791	1.34135	0	0.516175	0.8731	0.241291
ENSG0000(	YIPF4	2:3227790	23.6594	15.9679	10.3311	11.3395	7.70684	30.9736	22.723	14.8189	13.1053	9.50522	0.4968	0.454581
ENSG0000(	SFXN1	5:1754770	20.0656	33.3733	15.9098	19.1042	14.4936	27.2701	26.1289	24.228	34.4059	31.6195	0.35635	0.421276
ENSG0000(	RP11-587	11:275068	4.61681	2.49901	6.3685	3.72455	1.79951	3.1541	4.41321	3.34464	3.10998	0	0.8652	-0.37991
ENSG0000(	IKBKAP	9:1088675	16.7887	18.4546	5.95502	6.19507	5.10674	4.09612	10.2038	6.27171	12.8557	10.3581	0.73325	-0.14722
ENSG0000(	U47924.31	12:694297	45.7052	9.27411	7.18027	3.92518	2.56234	5.81901	4.6926	6.73412	6.66263	5.54902	0.4825	-1.19754
ENSG0000(	CIB2	15:780778	1.02518	0.944918	0.148657	0.975964	2.39163	0	3.22313	1.54633	0.306647	0.810841	0.8938	0.142529
ENSG0000(	TM2D3	15:101621	53.1073	48.1233	54.9507	74.6015	60.9881	85.5674	95.8709	74.2461	61.8491	38.5095	0.47565	0.238962
ENSG0000(	TMEM35B	1:3497435	117.864	73.7241	213.077	166.438	110.208	173.779	115.648	182.093	156.671	44.7126	0.9753	-0.01355
ENSG0000(	ICAM1	19:102519	13.6607	3.92482	6.07614	17.0607	10.1654	52.5398	23.3646	19.4103	6.8428	16.9319	0.05175	1.0547
ENSG0000(	DDOST	1:2063345	140.718	101.44	202.203	210.52	145.108	136.832	148.457	193.243	165.443	98.6008	0.67145	-0.1639
ENSG0000(	ABR	17:997116	10.4763	15.43	3.3216	3.48118	7.68636	18.5051	22.0678	9.7129	1.17447	15.0829	0.3082	0.742249
ENSG0000(	NSG1	4:4348139	4.03479	7.92702	58.7568	35.3684	43.7714	0	1.03943	13.849	5.47299	10.0656	0.0277	-2.43951
ENSG0000(	RB1CC1	8:5262245	25.5127	27.0643	6.4386	11.487	7.15642	11.8392	12.5165	10.0151	20.7349	14.2009	0.78185	-0.10525
ENSG0000(	SH3GL1	19:436036	12.7346	16.5726	7.06091	8.09915	12.2719	7.47591	8.15555	10.5248	7.83609	19.247	0.9822	-0.01055
ENSG0000(	GRAMD1C	3:1138281	0	0.1106	0	0.388079	0.30459	1.50059	1.46755	2.46218	0	0.72083	0.095	2.12319
ENSG0000(	SNX24	5:1228434	6.81715	0.832414	1.15194	3.4547	2.21311	14.975	1.99326	0.687881	6.159	2.04358	0.284	0.755098
ENSG0000(	POLD4	11:673178	117.24	118.255	129.319	139.262	101.781	187.97	144.431	115.828	137.044	63.4879	0.9837	0.014244
ENSG0000(	RTF1	15:414084	13.9918	15.811	30.3884	21.1753	36.5032	24.2901	43.6051	25.6372	28.3918	32.761	0.9853	0.019034
ENSG0000(	CEP63	3:1344777	23.4833	11.3689	20.6386	12.6986	32.8808	26.6651	12.294	15.3167	3.9005	29.8291	0.6245	-0.35946
ENSG0000(	POLE2	14:496435	2.60287	5.53387	0.898922	1.11236	1.45809	4.06627	3.89081	2.38407	1.9062	1.82503	0.63855	0.369555
ENSG0000(	GSTM1	1:1096560	23.6996	3.73724	35.9428	0.608245	29.563	19.2774	1.35262	27.0439	1.9887	25.3121	0.7273	-0.44889
ENSG0000(	RPP25L	9:3461048	41.1682	41.6211	67.9747	59.7824	56.4298	45.6826	38.4718	41.838	30.3622	22.3047	0.10975	-0.64651
ENSG0000(	MRPS28	8:7991871	54.9522	37.3254	70.015	62.9487	43.481	84.8531	73.6349	39.8133	60.119	11.9497	0.97465	-0.01891
ENSG0000(	IL2RG	X:7110740	1047.52	791.578	1324.07	1108.77	1473.92	963.544	894.904	1198.58	1300.32	1474.87	0.93795	-0.04002
ENSG0000(	RP3-337H	6:4350696	1.31337	0.70365	1.52176	0.820734	0	0	0	1.10254	0.337569	0.633824	0.6003	-0.77617
ENSG0000(	RPS6KA3	X:2014991	23.5576	11.0795	8.30408	7.51528	9.49537	13.4167	7.49609	11.81	4.5778	18.8819	0.3257	-0.36045
ENSG0000(	HMGB1P6	15:710969	130.635	121.624	134.913	130.12	146.826	170.23	204.166	135.809	127.889	70.4407	0.84505	0.078487
ENSG0000(	ADNP2	18:801090	2.699	2.78231	1.89705	1.48901	2.57612	3.42329	2.16285	1.34355	1.87889	4.07675	0.9003	0.056962
ENSG0000(	SIPA1L2	1:2323979	0.69794	0.444727	0.899464	0.111133	1.21145	0.006496	0	1.03969	0.370526	1.38369	0.80485	-0.20951
ENSG0000(	NPM3	10:101781	2.13901	1.57345	14.5279	22.4901	10.4595	2.95131	1.39719	19.9058	16.6818	2.38327	0.69795	-0.25853
ENSG0000(	LINC0061	12:905558	3.85794	0.238133	0.612074	3.35707	0.795191	0.407746	0	0.817725	1.8496	1.30351	0.20805	-0.94235
ENSG0000(	EDARADD	1:2363482	1.20605	2.33703	0.360217	0.691902	0.528168	4.29288	3.69684	0.890073	0.067467	1.74038	0.2308	0.893408
ENSG0000(	PRPS1	X:1076284	15.9595	9.9553	8.58033	14.7517	12.0127	14.6843	13.8847	9.40746	10.5896	3.28764	0.7803	-0.13001
ENSG0000(	PRRC2B	9:1313940	13.0505	18.1108	5.53143	4.53891	9.20959	15.8507	12.9583	8.49396	4.0651	23.6247	0.5999	0.22696
ENSG0000(	ZNF736	7:6430745	1.17034	0.806273	1.00544	0.593673	0.751273	0.781896	0.891021	1.11187	2.35711	1.65214	0.34575	0.709292
ENSG0000(	NRG1	8:3163938	1.1431	1.7861	0.339206	0	0.021805	0	0	0	0	0	0.00035	#NAME?
ENSG0000(	NR4A2	2:1563244	6.03147	2.44337	6.21351	46.8098	1.17106	2.10942	7.25971	23.0287	28.0171	9.2895	0.81475	-0.13838
ENSG0000(	Metazoa_	16:461649	0.24065	0.632644	0	0.506809	0.202819	0	0	0	0	0.758457	0.9079	0.118702
ENSG0000(	RP11-640	17:320831	0	0	0	0	0	4.08212	0	0	0	0	0.1017	inf
ENSG0000(	CNOT11	2:1008201	83.954	79.9415	44.6528	38.3391	42.4319	50.8019	35.2571	39.5654	15.3008	51.7733	0.6286	-0.53116
ENSG0000(	LYSMD2	15:517230	54.8705	32.4384	83.3174	60.7983	60.4787	29.6092	43.0317	59.8625	51.2539	24.9863	0.18355	-0.48735
ENSG0000(	VPS39	15:421587	34.8018	39.8284	10.3327	11.4517	8.47548	19.8503	11.3195	6.93964	3.64512	17.6922	0.03605	-0.74177
ENSG0000(	AC011558	19:102658	32.6527	37.6207	33.5055	39.0872	37.9438	18.4168	13.8021	17.5912	14.1679	9.0697	0.39425	-0.9788
ENSG0000(	RPS5	19:583863	201.45	217	499.248	339.675	411.405	171.496	234.468	405.812	217.844	76.8205	0.06195	-0.65257
ENSG0000(	PPIE	1:3969218	72.9187	56.6577	106.881	106.87	97.1406	66.0019	62.1091	79.3037	93.0256	38.134	0.3647	-0.35501
ENSG0000(	GATM	15:453611	0	0	0	0.076296	0.097159	2.87419	0.859709	0.518906	0	0.077212	0.082	4.90862
ENSG0000(	RP11-568	2:6452218	0.654448	0.261724	0.208023	0.136925	0.099576	1.81479	0.430169	0	0	0.375248	0.63985	0.916775
ENSG0000(	ZNF546	19:399841	1.19369	2.4199	1.69074	0.888075	0.760369	3.9752	0.668736	0.854779	0.751586	1.9613	0.6823	-0.28736
ENSG0000(	NOB1	16:697270	25.4284	19.0999	51.8128	35.0863	59.6841	11.9357	26.8344	38.3534	23.8706	24.8109	0.1258	-0.62255
ENSG0000(	CUL1	7:1486964	40.8239	39.4362	25.7037	23.0973	18.5596	31.344	21.3112	15.7281	9.47492	22.2382	0.0466	-0.68134
ENSG0000(	AC008982	19:388154	0.823222	1.13265	0.474703	0.134841	0.689262	0.232531	0.193385	1.47189	1.97342	1.57213	0.46605	1.00591
ENSG0000(	AC009950	2:2301213	1.85752	3.90462	1.87843	3.44463	0.296796	2.6865	2.43495	2.60254	1.03578	3.61838	0.75405	0.237227
ENSG0000(	BIK	22:431107	7.34628	16.3643	9.13969	13.2199	5.61974	4.91841	8.39981	10.4854	12.9405	3.13789	0.6691	-0.30935
ENSG0000(	YRDC	1:3780294	19.2412	15.3054	38.7194	54.9678	28.4995	9.96686	23.4447	31.2032	52.4954	15.3199	0.62295	-0.24872
ENSG0000(	CEP57L1	6:1090951	7.44771	6.39536	4.13843	7.6681	6.68354	11.7425	3.40255	4.49882	6.40567	6.01477	0.94505	0.036307
ENSG0000(	SCLY	2:2379668	10.7322	9.18346	7.26977	4.29758	8.75901	9.57963	13.3746	11.0121	4.51715	7.59648	0.8859	0.099146
ENSG0000(	TNS3	7:4727515	0.049861	0.220156	0.074604	0	0.126807	3.20801	1.30659	2.10453	0.411091	1.2522	0.00145	4.0103
ENSG0000(	FGL2	7:7695983	2.53147	10.1591	3.77828	8.64145	1.35086	27.1224	3.74838	4.41789	6.7188	2.02318	0.35475	0.780713
ENSG0000(	CHMP1A	16:896444	43.9488	33.502	52.4401	70.2486	52.5649	35.248	37.6503	65.7524	38.6503	48.3672	0.62525	-0.16839
ENSG0000(	TBRG4	7:4510009	82.0029	95.4764	57.8954	62.7886	55.4541	58.936	66.7962	44.088	48.4626	40.6716	0.14395	-0.44784
ENSG0000(	VPS25	17:427734	44.628	37.0336	69.7279	62.4902	46.8669	57.3841	52.3362	55.1707	42.2069	24.4043	0.53225	-0.21149
ENSG0000(	RP11-434	3:1290875	0	0.626784	0	0.092761	0	0	0	0	1.86889	0	0.6231	1.81604
ENSG0000(	CLASP1	2:1213377	8.11474	6.49485	2.5791	1.38266	3.1103	3.96324	4.04705	5.36638	1.1594	11.2892	0.69575	0.163798
ENSG0000(	RABL2A	2:1136272	10.9887	14.738	10.636	10.7479	8.40618	10.5095	9.82669	9.47673	9.29456	9.16804	0.8793	-0.07666
ENSG0000(	RP11-848	17:310084	2.48333	1.70783	2.37712	1.8568	1.77777	2.99666	2.80272	0.054091	3.55436	1.09941	0.9434	0.063763
ENSG0000(	LMO4	1:8732846	19.8966	9.49319	7.01858	14.0233	9.6923	22.9095	22.7038	13.1935	30.9846	8.26128	0.19105	0.676852
ENSG0000(	CSF2	5:1320737	0.124954	0	0	0.397307	0	12.3924	11.7044	1.4803	5.54969	0	0.0212	5.68806
ENSG0000(	RP11-154	15:483123	0.793797	0.352361	0.619002	0.795931	0.338365	0.689906	0.654056	1.06361	0.184599	1.04239	0.55025	0.691735
ENSG0000(	CTC-524C	17:528226	2.15165	3.38361	2.66756	2.80333	1.09357	2.1701	1.01252	2.87844	0.858262	8.18045	0.981	0.03208
ENSG0000(	CBWD2	2:1134376	29.8365	27.5714	27.7159	33.8703	23.7454	35.6333	36.6055	29.5981	44.4951	19.3295	0.636	0.194535
ENSG0000(	RASSF1-A	3:5032978	4.81979	4.27676	5.50435	5.61632	2.75013	1.2391	3.74382	2.44557	2.51169	2.05193	0.3402	-1.06204
ENSG0000(	LRCH4	7:1005722	115.968	163.431	69.6198	75.7059	81.9032	85.2525	108.063	93.8761	74.6963	102.966	0.82525	-0.09449
ENSG0000(	TMEM186	16:867456	14.1563	10.4139	15.216	9.08235	14.2126	14.6015	11.9008	12.4517	12.6141	8.77259	0.888	-0.11008
ENSG0000(	C14orf166	14:519894	104.972	81.5864	85.586	73.7442	76.0934	144.128	132.211	84.7355	82.2362	31.891	0.81665	0.114072
ENSG0000(	BPIFB1	20:332734	0	0	0	0	0	0	0	0	3.8458	0.566868	0.0453	inf
ENSG0000(	C11orf65	11:108157	0.555683	0.874704	0.049942	0.788245	0.090889	0.77832	0.145097	0.618057	1.46721	0.635855	0.56905	0.681312
ENSG0000(	RP11-295	12:251952	0.414156	0.95862	0.079174	0.344153	0.176778	0	1.42141	0.299488	1.2141	0.398556	0.5284	0.965844
ENSG0000(	NAA60	16:336509	38.3579	39.179	20.5283	20.6119	22.9279	25.4321	53.5385	21.787	8.90824	41.2921	0.91955	-0.06341
ENSG0000(	RBL1	20:369963	6.50824	5.4145	1.71788	2.41237	1.64711	3.23953	7.79918	4.66319	7.82639	4.16414	0.2792	0.652003
ENSG0000(	CCDC144	17:166895	0.408	1.29346	0.345033	0.318755	0.995052	1.2434	1.25684	1.00857	0.484709	0.345724	0.7713	0.287245
ENSG0000(	XXbac-BP	6:3139428	1.16157	2.59501	3.12396	2.20179	3.40083	3.77115	0.711107	3.60831	2.9089	6.08624	0.6643	0.338305
ENSG0000(	TMEM138	11:612994	41.675	28.86	38.5693	52.1735	36.4498	69.4417	47.3048	41.1773	27.8324	34.4639	0.83715	0.148537
ENSG0000(	LIMD2	17:636224	386.653	593.515	383.283	310.527	496.537	282.072	347.989	300.448	320.547	443.326	0.31205	-0.40435
ENSG0000(	RRP7A	22:425005	1.64294	1.43697	25.4286	19.8389	20.6474	0.869884	1.43894	34.6405	18.9862	20.5429	0.8507	0.082991
ENSG0000(	RP11-424	3:4433794	0.228748	0	0.316279	0.525175	1.40055	0	0.561552	0	2.70597	0.24941	0.82315	0.505092
ENSG0000(	MT1X	16:566824	44.2365	43.437	74.0259	49.7206	59.9591	72.9181	42.6388	59.8987	18.2545	8.46582	0.278	-0.59778
ENSG0000(	TSPAN32	11:229564	47.5755	71.7227	31.737	55.3465	41.7738	26.7645	20.6406	15.8466	44.4235	23.827	0.15145	-0.82854

ENSG0000	HECTD3	1:4500253	26.8117	36.1646	10.2326	6.30717	10.6428	18.2887	17.0701	7.56698	1.72364	22.1648	0.2738	-0.40422
ENSG0000	PLEKHA8	7:3002740	1.45209	2.58498	0.823077	0.817203	1.91509	0.02113	1.22808	0.045082	0.699022	1.79339	0.31555	-1.07461
ENSG0000	YY1AP1	1:1556097	40.4386	51.9317	34.3427	26.7694	29.5057	27.8043	33.1992	23.0875	19.3399	52.5851	0.65465	-0.26308
ENSG0000	ETHE1	19:435067	32.9204	30.6005	48.8915	50.8387	54.8028	51.8483	43.5815	54.3515	72.8517	13.6677	0.7945	0.10092
ENSG0000	LGALS9	17:274567	11.0808	22.068	6.74896	5.12684	5.80785	27.0852	33.5183	13.0617	2.94893	5.32831	0.25905	0.680712
ENSG0000	ZAP70	2:9771355	169.063	168.037	260.168	265.282	259.729	135.664	119.795	232.259	193.678	274.697	0.544	-0.20965
ENSG0000	BOD1L1	4:1356873	17.4566	22.4323	4.2344	2.50295	8.50978	31.8632	23.0745	6.53603	5.19797	18.7616	0.16105	0.5734
ENSG0000	CD38	4:1577827	4.23458	18.4115	2.20208	9.3767	2.76821	22.7788	51.1476	23.9942	12.166	16.9932	0.0034	1.55537
ENSG0000	DCTN3	9:3461354	87.409	87.0167	102.169	110.366	78.0746	113.968	105.138	88.6432	74.4654	29.1102	0.5836	-0.18948
ENSG0000	KHNYN	14:244265	9.88315	10.1203	4.45661	3.42241	6.22945	7.84633	10.2614	9.0246	3.40896	11.7532	0.8107	0.15904
ENSG0000	TMUB2	17:441869	42.8026	37.3784	45.2306	59.4026	32.6768	64.519	49.2896	43.543	58.0339	52.91	0.41955	0.285056
ENSG0000	HEATR5B	2:3696838	10.0896	15.9464	1.25628	2.15889	2.58434	1.92827	5.19128	2.99499	2.05924	3.20969	0.09975	-0.80215
ENSG0000	TMEM176	7:1507912	4.88037	3.76142	0	0.047956	0.652634	156.197	1.78562	13.2992	0	0.730622	0.0332	4.30032
ENSG0000	PRMT5	14:228932	26.5696	15.7523	24.5392	27.0068	22.9131	15.6182	14.2366	18.9889	12.0516	23.6624	0.37465	-0.47301
ENSG0000	TEP1	14:203656	3.50961	3.05757	2.10211	1.09543	1.67084	10.7445	3.57564	5.58406	0.122786	4.15603	0.04705	1.33878
ENSG0000	WDR26	1:2243851	7.80847	8.10602	5.17845	3.92806	5.60131	7.50652	8.89505	6.30456	5.52793	16.4712	0.435	0.29759
ENSG0000	LRRC61	7:1502439	16.0971	19.3675	21.6747	12.3545	18.2645	27.7438	26.4668	17.3956	14.3509	16.2972	0.6378	0.196428
ENSG0000	HTRA2	2:7452664	25.2911	26.2731	14.3371	14.6666	16.0002	23.4446	31.4635	15.9264	7.59978	9.74139	0.729	-0.2053
ENSG0000	PPP1R3F	X:4926984	3.78688	5.3557	1.29856	1.52131	2.92938	3.21133	5.37664	2.68594	0.240554	3.59085	0.98735	0.01315
ENSG0000	PLD6	17:170425	1.9695	9.69609	5.04732	9.1524	12.806	0.547133	1.01016	4.47573	11.0021	11.4987	0.7407	-0.39059
ENSG0000	UBE2D1	10:583349	23.9938	24.976	15.4705	20.2299	12.6796	34.5567	29.0313	15.3952	11.4284	12.3653	0.9059	0.04034
ENSG0000	MOGS	2:7446105	54.9579	50.7987	29.2551	56.6902	33.3097	84.558	60.4577	46.526	49.258	58.0141	0.46175	0.273615
ENSG0000	RP11-109	17:354642	2.71346	2.46514	1.06238	0.463558	0.359436	1.68647	4.39022	2.29207	0	1.22734	0.68115	0.633599
ENSG0000	RP11-53B	18:132174	0.848306	0.486136	1.90001	1.83529	0.936149	0.872443	1.30184	2.31063	0	3.32788	0.79585	0.45573
ENSG0000	C22orf29	22:197567	1.53999	3.28837	2.8886	1.81117	2.76943	2.35068	1.36026	2.9794	9.2698	4.34288	0.43815	0.601871
ENSG0000	TKTL1	X:1542575	3.52448	7.24131	8.35104	10.1165	2.2098	0.505708	0	2.33614	1.47662	0.600026	0.0281	-2.543
ENSG0000	DOLPP1	9:1290810	14.8633	3.61054	26.092	26.2947	33.6759	3.7084	3.48495	11.4961	39.4586	34.4875	0.7066	-0.16773
ENSG0000	PRR34-AS	22:460494	0.778669	0.768035	3.13363	2.228	1.62062	2.61344	1.51048	3.52585	2.15341	0	0.9126	-0.16055
ENSG0000	WBP1	2:7445502	34.8033	22.5589	22.2797	33.0549	19.8072	37.6929	34.922	16.6361	34.7159	14.1667	0.78605	0.106513
ENSG0000	HIKESHI	11:863022	80.0522	46.8734	87.4667	93.2313	42.106	60.9477	80.4544	85.1807	84.6455	13.181	0.8421	-0.07858
ENSG0000	SNRPF	12:958589	28.344	28.0214	28.1518	30.9	16.8901	30.2741	37.6438	30.7405	19.0619	6.80472	0.9528	-0.02238
ENSG0000	BLOC1S2	10:100273	99.517	70.3532	91.2664	111.83	50.2368	114.613	125.806	75.4064	84.5918	15.6592	0.9783	-0.01024
ENSG0000	POLE3	9:1134072	74.7928	64.2676	86.4926	55.6125	84.513	54.5555	54.5627	55.3976	52.5101	53.0229	0.18105	-0.42059
ENSG0000	PRMT1P1	5:1771330	0.188774	1.80272	0.233582	0	0	0	0	0	0	0	0.1317	#NAME?
ENSG0000	FAN1	15:309038	6.81223	5.61511	2.25799	4.69494	1.30628	2.77533	1.22391	1.27848	1.70737	4.19396	0.21085	-0.89243
ENSG0000	RPL10P9	5:1686163	22.2785	0.614459	46.2286	1.5091	1.07723	13.9526	0.652885	29.3208	1.29091	0.285042	0.42305	-0.6201
ENSG0000	RP11-192	17:284557	3.61152	4.01448	4.11212	3.55149	6.95262	10.5498	3.46851	2.1706	10.9371	1.25229	0.6901	0.469988
ENSG0000	OAS1	12:112906	46.7252	68.7053	65.0868	64.0154	41.4658	74.6845	275.888	101.97	46.7792	46.6349	0.1295	0.851335
ENSG0000	SBF1	22:503433	17.6492	19.4539	10.2043	5.45177	14.2551	15.7129	15.0098	20.511	7.28599	32.3602	0.28865	0.644466
ENSG0000	GCOM1	15:575919	0.59239	0.593699	0.838728	0.76327	0.67472	0.320195	0.346884	0.669082	0	0.297878	0.3286	-1.10678
ENSG0000	IGKV2-29	2:8923417	0	1.19459	5.07552	3.31317	0.511196	0	0.097748	0	0	0.130434	0.26185	-5.00639
ENSG0000	RP11-97C	15:884594	9.12977	7.46586	12.3142	16.8499	5.02616	13.4373	12.1447	8.26146	6.39404	2.52328	0.8911	-0.20887
ENSG0000	RP11-678	19:215708	0.044114	0	2.24093	17.0512	3.80309	0	0	1.82712	8.53898	1.56966	0.3417	-0.9058
ENSG0000	RFLNB	17:439977	4.0106	10.6484	9.75085	6.44764	19.7294	2.41365	8.40401	15.2583	10.4795	30.9392	0.5546	0.263035
ENSG0000	RAD17	5:6935098	31.4484	24.357	12.2973	25.7574	8.26758	14.7379	8.45355	16.1925	13.3866	13.0732	0.3809	-0.57707
ENSG0000	FANCF	11:226225	8.59549	6.91933	11.3322	5.68005	8.54276	13.4245	4.75655	6.66817	8.99819	5.65914	0.67945	-0.16035
ENSG0000	KCNA3	1:1106724	68.599	63.35	29.2165	26.4865	27.6068	12.8988	28.1851	27.1479	39.8064	21.6234	0.1083	-0.57931
ENSG0000	HAUS6	9:1905314	8.68558	8.84119	7.69038	5.18659	8.61927	5.38294	4.08528	8.29403	4.21419	11.9023	0.52415	-0.2773
ENSG0000	RP11-484	11:433119	0.567372	1.79957	0.903105	0	0.40985	1.06025	2.79559	0.909457	0.142812	0	0.81925	0.365441
ENSG0000	C7orf49	7:1349865	18.9606	23.323	33.319	26.0163	23.4135	19.1688	18.9504	11.8027	19.1468	12.0165	0.29	-0.66947
ENSG0000	SENCR	11:128629	4.80227	0.772954	4.47501	6.37135	7.50845	0.87545	0.720861	1.70299	0	2.20185	0.1723	-2.22524
ENSG0000	POFUT1	20:322078	4.43957	3.13836	2.34744	1.71693	2.75869	3.25817	2.83221	2.11525	1.93674	3.30489	0.6735	-0.19625
ENSG0000	RP4-730K	1:1136968	0.231568	0.488897	0.191285	0	0.199364	1.95048	0.823706	0	0	0.533167	0.41035	1.52314
ENSG0000	CRY1	12:106991	50.3568	41.9038	26.4632	31.3173	19.7975	40.6844	22.0647	38.7565	33.3952	23.6744	0.98795	0.00699
ENSG0000	RP11-50C	15:585875	1.5288	0.65289	0.673348	1.13709	0.718457	0.386913	0.884605	0.079155	0	0.182906	0.3301	-1.55629
ENSG0000	MPRIP-AS	17:170425	0.526351	0.106231	2.15117	2.35726	0.904999	0.047904	0.157454	0.778685	2.86749	1.93881	0.98095	-0.04642
ENSG0000	RP11-800	18:790692	0.262048	0.052361	0	0	0	0	2.86892	0	0	0.072091	0.38495	3.48997
ENSG0000	PSMC2	7:1032974	67.6984	69.0269	77.6513	72.4677	56.312	93.6697	74.9933	73.4319	45.4533	49.716	0.69955	-0.18944
ENSG0000	C15orf38-	15:898305	0	0.676852	3.22383	0	3.94282	10.8555	2.13815	8.33996	0	5.00256	0.98035	-0.01428
ENSG0000	PTGES2	9:1281206	48.3807	47.8592	77.6652	66.1132	54.6885	22.2654	40.8224	50.357	21.3791	46.2257	0.03335	-0.73306
ENSG0000	RP11-355	2:6147118	11.7986	6.1043	6.13156	2.47003	2.26388	12.1533	12.0704	3.14192	0.182494	4.28389	0.99945	-0.0005
ENSG0000	EZR	6:1587657	144.861	166.613	116.744	71.9665	104.872	91.1998	174.455	141.699	135.887	131.842	0.5795	0.198126
ENSG0000	IPO8	12:306289	7.59381	9.3621	2.79074	2.98008	3.29634	1.93386	5.52158	3.18614	1.23125	6.89086	0.2333	-0.54577
ENSG0000	FAM86B3I	8:8228594	0.845762	1.01285	0.223352	0.271482	0.039753	0.142776	0.051558	0.375129	0	0.087231	0.0462	-1.82977
ENSG0000	XRCC6	22:416211	213.504	257.514	274.604	256.175	222.55	216.083	203.529	230.458	162.267	162.265	0.3106	-0.35889
ENSG0000	SENP1	12:480428	8.23095	5.17456	2.09139	2.50357	3.82453	5.5717	7.436	3.93576	1.02009	6.00798	0.949	0.0371
ENSG0000	RP11-863	8:3773476	2.38784	0	0.248373	0.765482	1.00649	0	0	0.573218	0	0	0.30845	-2.75031
ENSG0000	TUBB2A	6:3153668	2.9494	4.82818	2.36415	5.63867	4.34768	4.26251	10.2262	4.53921	13.4571	2.99403	0.4544	0.665788
ENSG0000	TRBV12-5	7:1425809	7.65293	3.28165	3.84826	1.92188	5.88158	0	2.97998	3.4757	0	1.36748	0.3536	-1.41294
ENSG0000	TLR6	4:3879067	0.732571	0.752036	0.236043	0.233017	0.26375	2.01361	0.016613	0.00246	1.81855	0.621216	0.48455	0.878903
ENSG0000	CXorf40A	X:1494769	16.6888	11.6655	18.5775	16.6081	17.099	25.6318	34.9257	20.8289	14.1688	12.0008	0.6393	0.386967
ENSG0000	FDXACB1	11:111782	4.54815	3.13676	5.3921	8.08805	4.64648	2.21994	0.84779	2.95746	10.6959	3.87333	0.68545	-0.36629
ENSG0000	CNOT4	7:1353617	13.8164	23.9216	9.71187	9.47247	17.7378	14.1581	6.45879	11.5837	10.4863	23.0197	0.53195	-0.37359
ENSG0000	RP13-582	8:1432896	0.397386	0.25335	0.682153	0	0	4.75812	0	0	0	0	0.5552	1.52914
ENSG0000	RP11-16E	15:656113	6.21829	10.6702	2.03203	6.82688	0.415707	1.37554	4.75943	2.29082	3.80836	1.75952	0.58635	-0.7365
ENSG0000	DDX56	7:4456541	49.3627	51.3626	63.5345	62.7912	54.7271	46.1781	47.3564	49.3704	43.867	54.5257	0.3706	-0.29601
ENSG0000	TEF	22:413673	0.563669	1.50797	0.471952	0.075415	0.937946	0	1.00524	0.613392	0	2.29779	0.98245	0.021261
ENSG0000	ARL4A	7:1268685	42.4381	29.8318	31.2726	91.2149	26.0323	19.2411	22.2446	23.7336	60.9083	24.8642	0.22405	-0.53532
ENSG0000	WFDC21P	17:600793	0	0.237004	0	0	0.132665	1.34368	0.563777	2.2657	0	0	0.03855	3.47442
ENSG0000	IGHV2-26	14:106301	0	1.27965	0	2.35212	0	0	0	0	0	0	0.0032	#NAME?
ENSG0000	RP11-522	9:8365996	2.12918	0.573286	0.743515	0.512907	0.622472	1.95979	0	0	2.54117	0.310887	0.90995	0.217852
ENSG0000	GAB3	X:1546752	21.2105	6.0403	23.326	18.4047	21.1123	13.4832	18.8265	20.2162	15.1078	32.1194	0.67435	-0.16315
ENSG0000	CLSPN	1:3572021	1.96657	3.78	0.26274	0.550529	1.53392	7.40192	5.52269	2.21569	1.46819	5.81074	0.06955	1.44648
ENSG0000	KCNRG	13:499825	0.16552	0.09	0.345298	0.457007	0.288555	0	0.809718	1.06475	0	0.516199	0.59645	0.799776
ENSG0000	RP11-131	12:118428	0.69978	1.82394	0.639742	0.6759	0.319012	4.00276	0	0.716694	0	0.482861	0.8608	0.28232
ENSG0000	HS3ST1	4:1139314	0	0.474365	0.009631	0.177369	0.163874	2.39598	0.795064	0.060534	0.609689	0.136316	0.0969	2.42552
ENSG0000	MMACHC	1:4549386	0.279808	0.113753	0.406874	0.250019	1.86463	0.022163	0	1.15387	0.007796	0.603286	0.4651	-1.17211
ENSG0000	LRRC37B	17:320078	9.97449	18.0128	9.5382	5.10153	6.82952	18.841	11.9756	11.1332	15.8848	18.281	0.1382	0.706003
ENSG0000	ABI3	17:492027	79.2665	63.3728	167.692	127.41	222.9	118.752	123.299	112.626	92.0051	161.407	0.60745	-0.17675
ENSG0000	TRIM35	8:2728488	8.11736	6.22931	4.78598	5.06621	5.59397	5.40275	7.35463	6.02501	7.44493	9.08936	0.5435	0.262765
ENSG0000	MRPL47	3:1795628	36.8211	34.6421	31.4502	22.4286	27.0117	60.6708	50.1001	24.9705	20.3565	5.86097	0.8466	0.111174

ENSG0000C	RSF1	11:776599	38.3392	53.545	49.8938	37.0863	83.5842	56.6058	74.1222	47.9334	25.8318	120.144	0.84435	0.074257
ENSG0000C	PTPRO	12:151077	0.328495	0.57861	0.007492	0	0.226359	4.06282	0	0	0	0.150578	0.256	1.7034
ENSG0000C	ZBTB44	11:130226	5.04894	4.22197	8.47389	6.31735	9.12569	2.54441	2.85679	5.67633	9.122	14.7659	0.8961	0.05453
ENSG0000C	SLC25A11	17:493712	72.6853	56.8462	86.3099	80.7974	88.2481	81.6282	67.1968	74.3764	74.1147	56.4557	0.64775	-0.1922
ENSG0000C	TFEC	7:1159351	0.179106	1.45181	0.056637	0.005823	0.35978	3.10333	0.418588	0.409151	0	0.101864	0.3389	1.17461
ENSG0000C	MIF4GD	17:752365	62.3029	59.4498	73.4399	70.5058	71.4891	85.5116	73.5626	72.8822	61.393	44.6959	0.9885	-0.0066
ENSG0000C	SEC16A	9:1364400	21.5104	28.2822	9.7865	4.44877	20.9729	14.8313	19.2626	13.1556	8.19864	31.908	0.7372	0.18396
ENSG0000C	TLR5	1:2231094	2.38399	6.4827	0.314405	1.2815	0.25791	3.26077	0	0.337255	0	0.050337	0.28245	-1.68493
ENSG0000C	RASAL1	12:113098	0.390396	0	0.299908	0.510416	0.505455	5.07612	0.780934	0	0.389693	0.163288	0.03165	2.35265
ENSG0000C	HIF1AN	10:100486	8.45596	7.45594	8.21763	6.14845	7.61744	8.36973	8.45186	10.0473	5.37205	13.2126	0.91535	-0.06529
ENSG0000C	MED24	17:400190	35.0312	44.8991	19.6131	22.2389	19.1926	42.1401	18.6685	24.9609	25.9529	26.4732	0.9762	-0.01136
ENSG0000C	IL17RE	3:9902611	2.31359	0.589596	0.624937	1.54525	1.88997	0.719644	0.776916	0.394341	0.655952	3.74815	0.7872	-0.28309
ENSG0000C	SLC25A34	1:1568433	0.428929	0.488176	0.215534	1.28248	0.256223	0.015878	0.024941	0.581835	0	0.369841	0.32165	-1.73403
ENSG0000C	IFNGR2	21:334028	8.8039	44.7743	5.2926	6.26057	3.34795	51.2754	3.24864	2.96119	15.6266	5.64733	0.90465	0.217828
ENSG0000C	PAPSS1	4:1075902	17.8483	21.027	17.109	15.867	11.6287	25.7064	20.3048	14.7065	15.7777	17.375	0.80095	0.08688
ENSG0000C	RTFDC1	20:564685	80.7704	80.6649	100.611	78.3271	90.3624	128.47	95.6387	95.3539	97.7999	65.4393	0.6012	0.168014
ENSG0000C	RP11-91P.	11:778211	0.117579	2.0608	0.079178	0	0	1.39095	0	0.087468	0	0.777709	0.93505	0.215534
ENSG0000C	SLC4A2	7:1510538	11.4948	6.39531	3.28546	1.87065	5.45744	9.37477	10.0022	6.86302	0	9.93855	0.5672	0.448442
ENSG0000C	IFRD2	3:5027902	50.2103	59.6473	80.7391	85.1683	54.3353	48.9312	40.3186	51.2159	58.5702	43.6552	0.244	-0.38073
ENSG0000C	AL133243.	2:3235702	0.857067	1.32922	0.43946	0.618129	0.476673	1.37565	0.08506	0.872583	1.52385	2.62759	0.6719	0.710266
ENSG0000C	DIRC2	3:1227947	2.44885	4.4744	2.06689	3.30672	2.01969	4.11463	2.21573	1.14502	6.66653	2.43169	0.8672	0.116462
ENSG0000C	NCR3LG1	11:173517	0.37394	0.383366	0.597338	0.183992	0.328188	0	0.283808	1.04166	1.23238	0.373654	0.63595	0.716265
ENSG0000C	GSR	8:3067806	36.1578	15.0852	11.9675	7.86914	12.8751	27.2819	23.62	15.5347	3.67769	12.3865	0.96505	0.015935
ENSG0000C	CUL4B	X:1205246	18.9222	23.6462	5.50199	4.1393	3.7617	12.8059	15.5671	5.89709	6.02511	7.39912	0.6479	-0.17295
ENSG0000C	RPS4X	X:7218135	171.418	170.659	373.71	224.526	259.927	150.962	151.489	210.682	214.52	49.5845	0.1064	-0.62527
ENSG0000C	MZF1-AS1	19:585591	2.5856	1.16491	1.45357	1.42258	1.03992	0.499857	0.850019	0.367514	1.52349	1.75744	0.5843	-0.62749
ENSG0000C	COPRS	17:318518	9.43352	14.448	10.8361	14.0051	23.1696	41.6528	25.8457	16.9215	15.2697	5.68336	0.3682	0.444403
ENSG0000C	MYLIP	6:1612912	28.1022	23.4146	32.5683	25.5137	35.0879	28.8308	3.36966	15.7192	6.62895	78.616	0.4067	-0.34522
ENSG0000C	CENPN	16:809664	7.71875	11.039	1.82196	3.5693	1.40305	24.7616	16.4689	2.98152	0	4.23315	0.4765	0.73072
ENSG0000C	COX11	17:548993	26.7067	34.5815	32.3727	28.6595	21.5051	34.2073	24.4819	30.1897	29.6809	14.4147	0.74205	-0.12522
ENSG0000C	ZNF490	19:123791	1.74942	2.14651	0.55541	0.13889	0.477995	1.80758	1.17322	0.861743	0.086711	0.986087	0.81985	-0.31002
ENSG0000C	COLGALT2	1:1839298	0	0.245992	0.114904	0	0.076839	0	0.777292	1.07434	0	1.03601	0.16355	2.66188
ENSG0000C	MMP17	12:131828	0	5.63091	0.344867	0	0.059446	0	0	0	0	0	0.00835	#NAME?
ENSG0000C	DNAJC5	20:638951	3.42973	5.87443	4.23847	4.5649	6.21584	6.39625	3.05817	6.37797	0.707484	11.2507	0.8725	0.070015
ENSG0000C	NIPA2	15:228386	61.2898	64.5665	44.6589	46.1631	36.9833	53.8096	48.7929	40.9512	26.4617	38.9649	0.2668	-0.38836
ENSG0000C	MED1	17:392526	6.13053	7.84678	4.88675	3.91168	6.33128	2.88422	8.03803	4.57415	5.05839	9.14142	0.75675	-0.17779
ENSG0000C	SP140L	2:2303271	31.0873	44.0789	26.7637	22.0521	28.576	36.8466	28.1092	25.3981	34.9581	43.4519	0.6511	0.161811
ENSG0000C	SKAP1	17:481334	169.821	123.014	223.769	165.869	213.214	156.477	170.973	212.286	153.367	131.556	0.7198	-0.12752
ENSG0000C	ASCC3	6:1005081	17.3706	15.7953	12.3898	10.4358	14.4743	19.7769	25.5191	18.6166	22.6328	29.4501	0.26765	0.509628
ENSG0000C	RELL2	5:1416369	14.0491	21.9792	4.22122	2.75423	3.74952	15.3713	9.17818	3.56085	5.63696	7.22815	0.9186	-0.09419
ENSG0000C	DDX42	17:637028	14.6957	16.8956	11.4534	6.24225	13.5907	14.3914	14.738	16.2666	3.38948	25.6859	0.70865	0.270909
ENSG0000C	DUSP5	10:110497	2.44678	0.701815	1.64873	4.98256	1.73895	5.08394	12.3197	5.29327	1.62398	6.98983	0.06855	1.38524
ENSG0000C	RTN1	14:595959	0.093795	7.48827	0	0.14954	0.022425	2.55659	0	0.843354	0	0	0.55285	-1.05275
ENSG0000C	TAF1A	1:2225579	5.27636	6.53257	3.03548	5.69075	3.0702	2.10592	5.13678	4.31035	3.43678	3.83703	0.68955	-0.2654
ENSG0000C	TRAPPC9	8:1397303	10.1441	8.83274	5.58393	3.31639	4.04739	4.2552	6.38521	4.19858	3.8639	6.26135	0.59855	-0.27299
ENSG0000C	UBE2Z	17:488748	27.694	29.4016	21.0663	13.9231	51.0017	27.0619	37.19	31.862	12.2357	82.9333	0.5537	0.277804
ENSG0000C	CSF2RB	22:369136	3.97733	2.41417	0.050591	0.545366	0.093542	11.0146	0.659168	1.28986	1.37777	0.175491	0.18885	1.04404
ENSG0000C	RP11-713I	11:134032	0.652348	4.0856	0.255576	0.032212	1.22138	0	0.087564	0	0	1.70525	0.34085	-1.49832
ENSG0000C	DUSP2	2:9614316	381.146	84.0183	747.136	1637.72	466.368	140.054	204.204	293.818	244.944	164.589	0.01885	-1.73584
ENSG0000C	SLC29A2	11:663479	1.67026	4.56951	0.942159	0.085516	1.30471	0.927948	1.17062	1.75397	0.096366	1.99917	0.67995	-0.39399
ENSG0000C	TTC38	22:462679	13.5534	14.6564	17.0376	14.6808	15.2017	10.0265	20.1151	11.5321	10.6833	14.1136	0.5174	-0.24233
ENSG0000C	SH2B2	7:1022850	3.88775	2.54401	0.21706	0.365786	0.486002	0.554582	1.60963	0.491056	0	0.824012	0.3752	-1.40818
ENSG0000C	KLHL23	2:1696944	1.10825	0.288674	0.702448	1.0601	0.306688	0	0.146297	0.104449	0	0.242582	0.0092	-2.71778
ENSG0000C	ARHGEF18	19:734894	14.7563	23.2268	5.95592	1.19062	8.0695	8.40895	6.58122	2.37783	2.15093	10.469	0.3326	-0.63514
ENSG0000C	CTD-2616	19:513451	0.147178	0	1.79048	1.62728	0.43447	1.93837	7.59981	1.66417	0.305114	0.640411	0.4352	1.50265
ENSG0000C	RP11-529I	4:3503596	0.318903	0.950037	0.495494	0.334309	0.056459	0.90978	1.10802	0.884755	0	0.954756	0.6684	0.869049
ENSG0000C	HSD17B10	X:5343125	67.0929	59.7157	143.015	119.033	129.515	94.6386	97.7397	125.732	121.67	45.945	0.69475	-0.13713
ENSG0000C	RBM45	2:1781124	14.7218	11.0902	11.272	7.72299	6.54895	21.5852	8.53498	10.3656	16.0649	4.36655	0.4281	0.434201
ENSG0000C	ZNF682	19:199970	1.02045	1.90915	0.525606	0.481153	0.873491	3.57749	3.38247	1.82299	0	1.61151	0.1145	1.38217
ENSG0000C	MRI1	19:137645	24.0782	34.5946	17.6522	28.4017	15.8417	33.9815	31.6902	37.5268	20.6328	34.7515	0.5781	0.363352
ENSG0000C	EEF2KMT	16:488250	10.7654	7.1914	11.4107	7.9215	13.6247	5.45724	11.0416	10.5365	7.08504	7.21645	0.56765	-0.39067
ENSG0000C	SIRPG	20:162915	117.276	152.636	183.276	129.702	179.985	249.639	151.828	127.906	99.1608	184.989	0.89485	0.050966
ENSG0000C	WDR24	16:684621	11.6506	9.07906	7.32103	6.59304	7.30429	1.19616	9.12295	8.34042	9.89138	12.2874	0.93755	-0.03983
ENSG0000C	TLR3	4:1860691	1.54283	0	0.977928	0.414181	0.838961	0	0.783461	1.03012	3.7093	2.95665	0.69675	0.429112
ENSG0000C	GYS1	19:489681	17.9604	18.9816	6.10608	7.53289	6.421	17.1905	17.6351	7.94451	4.31255	13.5696	0.72485	0.124377
ENSG0000C	IMPACT	18:244266	6.82093	6.10495	2.30704	4.70242	2.30086	10.097	4.6461	2.62213	6.33614	4.04728	0.215	0.636961
ENSG0000C	UBQLN4	1:1560353	6.1883	7.17771	3.0736	1.69481	5.09834	3.55691	5.84119	4.51993	0.194388	4.88256	0.5292	-0.33821
ENSG0000C	RAB5C	17:421131	24.0201	29.0333	40.9387	41.5724	49.9157	32.0495	15.8697	48.4185	49.7016	39.6287	0.92465	-0.03973
ENSG0000C	NUP58	13:253015	10.5462	7.22696	4.02972	3.30056	4.20459	5.18908	8.60779	5.92632	9.86978	10.5967	0.38795	0.404558
ENSG0000C	ALDOA	16:300530	672.128	676.67	1011.14	894.175	954.829	948.545	876.491	816.972	669.106	550.175	0.69805	-0.14539
ENSG0000C	RP11-475	6:1532313	32.159	19.1201	31.8909	37.5956	6.21609	25.0608	24.518	12.6793	19.4641	0.81446	0.75795	-0.34297
ENSG0000C	GCLC	6:5349734	9.08042	6.99316	2.82654	4.24436	4.47903	6.69465	8.40909	3.44684	1.86116	7.55175	0.9796	-0.01114
ENSG0000C	LINC0092(	16:664085	7.23459	5.71372	2.46929	2.24018	1.11655	8.20301	2.36342	1.01502	0	0.447784	0.40775	-0.54968
ENSG0000C	TNPO2	19:126991	1.33472	1.62966	1.71416	2.17877	1.9542	0.832064	4.28016	3.13785	0.886669	4.35379	0.3869	0.448192
ENSG0000C	TRABD2A	2:8482164	3.48451	62.7078	5.25545	2.46526	4.25282	1.60286	2.02536	1.11168	0	3.16722	0.07485	-3.18748
ENSG0000C	CXCL11	4:7601118	0	0	0	0	0.05591	2.57722	0.961409	0	0	0	0.31085	7.04827
ENSG0000C	CYTH2	19:484457	17.3786	18.9913	29.2797	29.883	27.0531	29.3004	36.9476	39.8431	27.5218	31.0222	0.65745	0.238181
ENSG0000C	CYB561	17:634323	23.35	1.46551	25.3655	23.5948	20.0234	8.03021	1.92546	13.4022	11.0297	31.5671	0.0841	-0.64699
ENSG0000C	PSMD8	19:383745	144.35	124.224	157.317	153.923	149.185	162.371	238.511	183.276	181.267	66.7028	0.64665	0.164952
ENSG0000C	AL109761.	21:177633	0.356713	0	1.82767	1.17561	0.735848	0	0	0	0	0	0.1129	#NAME?
ENSG0000C	EEF1B2P3	X:2469391	1.69075	1.6015	1.12966	0.924807	0.773493	1.69902	1.74765	0.361196	0.415616	0.161538	0.75885	-0.50652
ENSG0000C	FAM172A	5:9361806	18.2485	20.7468	8.22609	10.0798	22.7027	21.2017	23.9045	12.9201	16.4156	26.3931	0.72245	0.192951
ENSG0000C	TRAPPC1	17:792185	97.8378	107.282	77.6688	69.0349	86.1343	99.9432	102.554	75.3509	75.0106	27.4754	0.47555	-0.26732
ENSG0000C	CACUL1	10:118674	8.6634	7.36598	16.9446	13.6588	13.6478	7.13884	9.30547	12.2462	10.9485	26.1464	0.86545	-0.09088
ENSG0000C	TMA7	3:4843216	649.573	355.073	641.589	543.865	326.485	802.877	741.561	484.164	436.386	82.6048	0.91865	-0.03948
ENSG0000C	MDK	11:463807	0.628967	0.740211	1.23562	2.49659	0	7.6483	3.91252	4.40622	0.35967	1.10693	0.12885	1.76364
ENSG0000C	PIK3AP1	10:965933	48.5918	12.9772	12.8546	10.5547	12.2908	18.5343	23.8322	15.7137	8.71566	21.9403	0.64095	-0.17124
ENSG0000C	NEDD8	14:241718	225.711	155.601	371.101	429.695	187.337	268.957	276.905	328.109	300.375	60.1083	0.8233	-0.16585
ENSG0000C	ZNF829	19:367975	1.13631	1.33255	0.935603	0.717315	0.783638	1.88926	0.323492	1.08487	0.53429	2.1591	0.9315	-0.0992
ENSG0000C	HIST1H2B	6:2615814	19.2756	4.97487	23.1501	6.22373	12.5064	14.1069	9.21669	5.48302	3.83143	2.96646	0.2698	-1.09295
ENSG0000C	RP11-686	2:2023367	0.286453	0.181665	0	0	0	2.02026	0.393786	0	0	0.191685	0.17055	2.7888

ENSG0000	TSNARE1	8:1422120	7.14889	9.85702	1.71529	5.86955	2.28144	8.8461	6.39628	1.60664	7.47818	3.62416	0.8718	0.111676
ENSG0000	ARAP2	4:3594822	53.4876	25.2267	14.2835	18.6265	13.3259	38.0037	31.0057	10.8612	31.3123	39.2589	0.7871	0.115096
ENSG0000	RP11-321	6:8550549	0.615251	0.615337	0.180252	0.024718	0.103685	0.024874	0.541428	0.233967	0.992634	0.275786	0.72805	0.55874
ENSG0000	SNRNP25	16:46406-	32.3311	18.9634	18.4347	22.7121	31.6672	53.9407	30.2969	19.4285	15.1217	13.6055	0.98815	-0.00949
ENSG0000	FAM72A	1:2061861	2.63753	3.32091	1.2406	0.966039	0.060955	2.86998	1.8807	1.02489	0	0.356559	0.33135	-0.95456
ENSG0000	RP11-500	6:1761503	8.98631	5.1376	10.0538	12.6332	14.0649	14.2001	15.645	11.3421	2.34175	4.78152	0.75795	-0.29053
ENSG0000	SERTAD3	19:404408	8.90313	9.17566	18.3647	31.2296	16.7535	14.8754	17.71	24.2711	11.8306	10.14	0.65055	-0.18489
ENSG0000	WIZ	19:154199	1.58392	2.50162	1.24685	0.458162	2.39303	1.67974	2.40109	1.58702	0.200681	4.96366	0.71705	0.283623
ENSG0000	PARP11	12:379104	6.40454	5.7961	4.95567	2.52945	3.57344	11.3291	6.45523	6.33025	3.42755	5.85959	0.47055	0.51883
ENSG0000	MAVS	20:384679	3.52697	5.45972	0.973633	1.64993	1.80796	4.38232	3.49301	1.79655	0.567468	4.35573	0.9658	0.017634
ENSG0000	ASB9	X:1523528	0.877717	1.75987	0.68284	0.849444	0.678752	0	0	0	0	0	0.00005	#NAME?
ENSG0000	ATP6V0A1	17:424588	5.48324	5.08037	1.5741	1.5548	2.23281	11.3251	3.85673	3.23459	5.11402	5.31901	0.0268	1.10637
ENSG0000	YWHAH	22:319335	168.138	82.3925	226.891	179.875	215.087	211.844	156.011	199.965	178.93	171.576	0.98345	-0.00646
ENSG0000	SCARNA9	11:936616	5.36036	4.21443	0.701507	5.24855	1.19215	4.44795	6.95231	4.15063	0.648027	0.965558	0.8622	0.236214
ENSG0000	NOL9	1:6521346	4.67767	7.49604	4.26633	5.20423	3.58854	3.84343	4.02602	3.5893	3.82806	6.37083	0.50295	-0.29597
ENSG0000	RPL23AP2	19:156116	1.3321	1.50109	1.13245	2.17498	0.694566	1.03862	0.791053	1.33318	0.903949	0	0.6856	-0.82502
ENSG0000	SLC35E4	22:306356	2.18214	2.3923	0.861861	0.714494	3.04413	5.94622	5.70976	4.77401	1.35593	2.20958	0.40995	1.29739
ENSG0000	MMP24-A	20:351720	13.1535	8.81767	16.7076	10.0784	11.6812	11.8746	7.51465	12.5048	15.8359	14.7805	0.9522	0.033287
ENSG0000	C2CD5	12:224485	21.8409	18.7007	4.5768	7.60361	3.06756	11.8582	11.0105	6.72357	4.54408	7.12365	0.3422	-0.33675
ENSG0000	CTD-2516	11:723877	0.112111	0.175006	0.971938	0	0.339712	2.14892	0.307262	0	0	0.227412	0.84295	0.210853
ENSG0000	PRRC1	5:1275176	13.0773	13.8785	7.7379	12.0586	14.9702	11.1143	8.65851	8.88656	10.8181	16.1493	0.8014	-0.11413
ENSG0000	MT-ND1	MT:3306-4	375.852	321.221	722.482	626.053	1482.39	1365.01	424.898	884.946	928.039	1215.68	0.5337	0.320421
ENSG0000	FAM210A	18:136633	6.35628	4.31969	6.7535	11.7186	11.1593	9.99605	5.53484	6.06591	6.11632	7.47001	0.54095	-0.48081
ENSG0000	SFT2D1	6:1663197	86.5737	57.9564	71.2558	58.3358	42.0549	131.046	72.2971	66.11	48.2258	29.2609	0.739	0.115081
ENSG0000	DNASE1	16:361172	16.5667	25.669	9.60982	8.18772	9.05794	19.8412	21.6624	11.3585	13.2438	29.4745	0.4328	0.403423
ENSG0000	SIGLEC9	19:511249	5.8625	6.40114	0.603803	2.57393	3.08789	13.2521	0.530996	1.07411	0	1.10094	0.8231	-0.26905
ENSG0000	RP11-126	18:586700	0.510144	0	0.306822	0.652058	0.600516	0	0	0.32021	0	0.15383	0.32215	-2.08954
ENSG0000	COPS8	2:2370594	56.5567	41.3341	59.1507	64.9741	42.9902	51.9113	56.0919	46.9243	87.0362	28.4251	0.9054	-0.04199
ENSG0000	ELF4	X:1300648	8.27193	8.35357	4.71478	1.662	5.94675	4.85611	5.82158	3.78581	1.17702	11.3483	0.64435	-0.22997
ENSG0000	WTAP	6:1596690	87.7866	79.4253	100.101	117.151	66.1186	96.7356	66.3599	84.3155	132.154	54.2438	0.95285	-0.04201
ENSG0000	RAB3D	19:112961	0.765882	1.17389	0.529507	0.344209	1.02444	0.498813	0.274416	0.357106	1.28537	1.75719	0.89645	0.214644
ENSG0000	9-Mar	12:577237	7.86892	13.6854	19.2467	7.5949	20.0781	5.34464	4.92981	11.5622	11.4398	20.0448	0.65645	-0.40034
ENSG0000	APOL3	22:361403	102.878	47.0932	91.2894	59.5999	90.623	58.9627	65.2874	61.6413	52.4714	94.937	0.3415	-0.32858
ENSG0000	DBF4B	17:447086	1.05172	1.92938	1.58111	1.19697	1.66586	0.065617	1.39465	2.17807	1.27828	3.61995	0.83055	0.16246
ENSG0000	FNIP2	4:1587691	2.24065	2.88843	1.41807	0.53832	1.18552	5.1242	0.131714	0.74662	0.007715	1.42208	0.63385	-0.29747
ENSG0000	SYNRG	17:375147	47.521	39.51	27.6546	34.8346	39.1554	34.4022	36.1397	26.1977	30.1484	55.7448	0.6837	-0.20094
ENSG0000	ABCC3	17:506347	0.085273	0.132755	0.337386	0.856319	0.039162	3.49218	0	0	0	0	0.28125	1.46921
ENSG0000	RP11-114	8:2318927	1.05928	0.263191	0.328285	0	0.337697	0.243572	0.47511	0.946709	0	2.00618	0.46815	1.15601
ENSG0000	FBXO48	2:6845941	1.03668	0.564656	0.721849	0.503382	0.66335	1.27125	1.12479	0.884387	0.565397	0.692266	0.56485	0.393434
ENSG0000	EDEM2	20:351153	50.2019	26.6066	53.5163	67.9509	47.7862	30.9081	17.5051	37.5319	68.2578	31.0998	0.26695	-0.39854
ENSG0000	RP11-282	12:123152	0.238923	0.162788	0.29756	0.289606	0	0	0.634973	2.00787	0	0.879081	0.16565	1.96037
ENSG0000	RP11-536	10:589020	0.359488	0.195189	0.085814	0.330478	0.10077	0.543399	0.097334	0.040067	5.65849	0.335069	0.08415	3.67174
ENSG0000	SRF	6:4317129	5.95251	4.4476	1.46662	0.944666	4.25388	11.0953	6.50409	4.46185	1.13374	7.56103	0.14175	0.786408
ENSG0000	LINC0031	21:340732	0.053333	0.737887	0.117337	0	0.160022	1.2938	1.35301	0	3.84155	0.143966	0.072	2.67932
ENSG0000	COMTD1	10:752339	23.6585	22.2091	28.1277	31.2254	39.5028	49.9008	62.8442	42.7546	31.9473	21.9463	0.3622	0.398456
ENSG0000	RNVU1-1	1:1444125	1.54579	0.881554	17.2826	4.62057	2.57594	0	0	0	4.68412	0	0.1525	-2.3524
ENSG0000	POLA2	11:652617	7.84434	13.2355	5.92887	12.0325	7.58187	3.421	9.16127	5.80985	8.89972	13.4845	0.4814	-0.3199
ENSG0000	RP11-422	1:1539777	3.45383	4.28239	2.20472	0.067025	0.464318	2.13115	2.30959	0.811595	0	1.85043	0.39845	-0.51501
ENSG0000	XXYLT1-A	3:1950682	1.16334	2.40079	0	0.14025	0	3.33107	0.232734	0	0	0.145622	0.9561	0.113861
ENSG0000	C1orf43	1:1542067	102.944	70.8226	104.855	97.7452	78.2483	114.043	101.684	111.283	76.3473	58.1527	0.957	-0.02165
ENSG0000	SLA	8:1328669	217.118	169.354	146.342	168.477	198.051	290.685	226.708	278.011	308.305	316.808	0.072	0.645502
ENSG0000	PNRC2	1:2395910	115.399	126.891	93.8863	86.0574	87.697	102.657	86.0516	74.6968	60.4402	80.1754	0.2783	-0.32622
ENSG0000	PRPF3	1:1503214	35.3832	47.649	24.326	20.268	16.9761	17.121	28.2591	37.6598	22.847	32.622	0.9092	0.039894
ENSG0000	CCPG1	15:552029	22.2735	20.1571	15.4704	11.2381	18.1065	24.8856	37.012	14.8333	12.1209	34.2971	0.7475	0.210451
ENSG0000	RP11-254	2:1004284	0	1.30905	0	1.41689	0.741663	0	1.28315	0.347511	0.851884	0	0.7271	-0.75316
ENSG0000	TOB2	22:414334	11.7038	8.63141	7.82887	8.14002	14.5674	11.2236	8.92258	10.0995	6.26283	29.1622	0.7469	0.128243
ENSG0000	SEC1P	19:486379	0.256566	0.6345	1.9993	0	0.387868	0	0	0	0	0.460586	0.1365	-2.91221
ENSG0000	SYCP2	20:598635	1.90443	1.87004	0.95826	0.939239	2.39938	1.76268	3.72794	1.61229	2.02065	3.60001	0.3676	0.538072
ENSG0000	ANK1	8:4165321	2.17276	0.771884	1.2703	1.63199	1.73099	5.92494	0.0052	2.39124	11.1665	6.27591	0.02365	1.65985
ENSG0000	TNF	6:3157556	38.4477	8.36692	35.2675	51.6768	47.2682	81.5506	82.9118	114.245	61.9323	107.702	0.0002	1.23105
ENSG0000	RGS10	10:119499	85.1954	191.957	57.1778	64.4796	33.3853	261.134	177.885	82.913	97.6406	18.8752	0.151	0.561175
ENSG0000	RP11-75L	12:966496	29.7321	63.6999	40.6597	25.6248	29.9931	65.7808	87.7193	50.1352	28.8233	41.7613	0.80245	0.43945
ENSG0000	RNF32	7:1564721	0.796572	0.708315	0	0.349679	0.856851	0.181302	1.95922	0.030887	4.59027	0.609886	0.1641	1.45372
ENSG0000	IL27RA	19:140317	76.7943	80.3339	66.03	57.9244	56.9001	76.4512	60.7295	55.1066	44.7608	74.2699	0.6228	-0.15244
ENSG0000	C7orf55-L	7:1393394	2.10853	6.93271	3.40004	2.28107	3.57498	4.48355	20.0147	5.11204	0.335874	3.92311	0.78995	-0.59025
ENSG0000	SHC1	1:1549570	20.1762	19.6393	24.3597	35.2556	23.215	19.9656	10.33	23.0155	25.3115	35.093	0.9242	-0.05122
ENSG0000	TSEN2	3:1248443	4.67851	7.95383	10.7108	15.1947	5.68477	0	8.99309	6.75133	9.46258	13.3561	0.72285	-0.24605
ENSG0000	FLJ42393	3:1881512	0.258316	0.103416	0	0	0.018563	0	0	0.738331	3.41331	0.424638	0.25645	3.85201
ENSG0000	PIM3	22:499605	33.6581	33.2013	23.7959	38.3061	22.751	47.3596	102.411	53.7383	33.7107	27.5905	0.0581	0.675363
ENSG0000	PSTK	10:122930	8.14104	4.1794	8.22969	9.54819	9.22379	11.2041	4.90896	6.316	1.41471	3.17779	0.42765	-0.67365
ENSG0000	SLC25A36	3:1409418	25.5762	23.0781	24.0431	32.2359	21.65	21.8673	24.9396	17.1464	14.9857	34.3888	0.78915	-0.16644
ENSG0000	HPN	19:350405	0.39206	0	0.338038	0.713812	0.11155	0.98434	0.863445	0	0	0.869414	0.6518	0.450895
ENSG0000	ZNF266	19:941259	22.9334	16.0066	31.4236	44.2891	32.5282	22.3008	16.4074	22.4358	28.8357	24.6497	0.4569	-0.61433
ENSG0000	CENPP	9:9229735	4.18237	3.99201	0.97311	1.34883	2.33125	2.2669	7.00232	2.54306	0	1.56271	0.8651	-0.19929
ENSG0000	RP5-994D	1:4276726	1.95649	1.76687	1.15317	1.50029	0.092927	0	0.181179	0.288414	2.92524	0	0.69175	-0.71055
ENSG0000	SMIM8	6:8728297	8.01229	5.95208	11.833	10.0539	4.19566	3.92936	7.64984	4.50366	8.40011	3.38877	0.3419	-0.57487
ENSG0000	TRAV21	14:220525	26.1605	83.9815	26.1875	9.83477	7.57096	48.3547	91.6052	8.68432	0	4.12969	0.9349	-0.07158
ENSG0000	SCAF8	6:1547333	8.71707	6.14099	4.83573	3.54522	4.13789	4.02256	4.46568	2.80511	0.746733	8.77837	0.3531	-0.51701
ENSG0000	TCF3	19:160928	24.6545	36.3704	9.39847	3.36006	22.2539	17.7046	19.4632	14.4351	1.56752	31.6529	0.43615	-0.43803
ENSG0000	LMAN2L	2:9670592	22.6624	8.4708	16.62	22.3692	18.0517	20.2429	14.9913	16.4777	20.0212	18.5214	0.95365	-0.02159
ENSG0000	MAMDC4	9:1368444	1.12002	0.453741	0.115505	0.310084	0.380603	3.90473	1.47958	0.222874	0	0.974614	0.37735	1.27606
ENSG0000	CTD-2588	14:561173	0.655581	1.54636	0.533015	0	0.73542	0	0	0.298257	0	0	0.2579	-3.33222
ENSG0000	CWC25	17:388004	32.344	40.698	25.3034	27.2718	42.1454	17.7089	38.1858	31.9737	23.7546	55.0834	0.71335	-0.13153
ENSG0000	TNFSF10	3:1725055	55.1965	59.2473	63.6329	66.9794	76.2823	93.5919	258.089	94.8165	119.829	48.8386	0.0032	0.959447
ENSG0000	AC135048	16:309193	3.11356	0.809841	1.22104	2.20385	1.98205	2.29292	1.73298	1.17304	6.21039	1.20962	0.84415	0.299126
ENSG0000	SIRT4	12:120302	1.05176	0.304548	2.2001	3.09648	4.44616	0.929814	1.03629	0.375155	2.76639	1.08369	0.6755	-0.72358
ENSG0000	SLC39A3	19:271456	30.7581	29.6048	40.5822	38.2401	53.7465	29.8099	35.2798	38.9614	31.6154	33.9845	0.50335	-0.27299
ENSG0000	QKI	6:1634139	12.7118	9.34925	7.81976	3.08263	8.90867	20.7888	9.55792	8.36245	5.82218	15.3277	0.62415	0.273095
ENSG0000	MSL1	17:401222	15.5007	16.0174	13.9066	9.37356	26.8868	26.2871	13.8557	18.6543	8.69174	43.5024	0.4138	0.375663
ENSG0000	SLC4A4	4:7118675	2.3323	0.098116	3.02845	2.13022	2.93775	2.25559	0	1.51131	0.596896	1.40607	0.3414	-1.3447
ENSG0000	CPNE5	6:3674077	0.737311	1.67442	0.364663	3.29943	0.54945	4.39882	0	0.808246	1.94593	1.0498	0.74655	0.351648

ENSG0000	CHTOP	1:1536187	62.4952	55.483	67.6081	49.9312	65.5852	41.8466	71.5806	82.1456	68.0291	75.3081	0.70805	0.139261
ENSG0000	MCMBP	10:119829	43.4105	35.5525	30.7075	32.3196	23.5758	27.8084	32.5004	39.8008	32.888	31.5749	0.809	-0.08094
ENSG0000	UBFD1	16:235220	11.5771	8.16456	6.49666	4.78565	6.11516	5.33024	14.5282	9.36885	8.22852	10.2151	0.7277	0.18747
ENSG0000	MTERF4	2:2409988	24.0358	36.7325	24.8896	28.6619	20.7172	22.4393	26.6362	11.3446	18.7912	20.2806	0.16155	-0.53713
ENSG0000	HNRNPCP	14:583959	1.92159	1.23356	1.62966	0.083625	1.21606	2.80082	2.42637	3.0689	2.58943	1.72114	0.41045	1.11802
ENSG0000	RP11-68I3	17:295605	0.760123	0.690282	0.292963	0	0.239018	0.093673	0.796044	0.174516	0	1.1818	0.86315	0.25453
ENSG0000	SUZ12	17:319370	15.4206	16.9394	4.51215	5.10047	7.67815	12.3332	15.8772	7.51374	10.5335	17.2199	0.31335	0.369811
ENSG0000	CCR5	3:4636495	37.9577	6.50772	14.6828	9.66083	20.5631	53.214	40.0026	34.4025	15.2969	35.127	0.01045	0.928714
ENSG0000	CH17-212	14:106737	0	0	3.94776	5.21627	0	0	0	4.89188	2.87802	0	0.987	0.020415
ENSG0000	ZBTB49	4:4290250	8.38246	10.1734	8.4326	10.8413	5.4417	11.2735	6.86667	5.06538	6.4241	6.14625	0.42195	-0.37684
ENSG0000	FAM46A	6:8149143	0.870677	3.8159	2.41685	1.36995	2.85388	1.65356	0.891155	1.56373	3.45329	3.59272	0.64615	-0.23391
ENSG0000	CRYGS	3:1865384	2.15129	0.246072	1.4444	1.67174	0.467286	0.049667	2.2174	2.86597	0.182287	2.63553	0.7042	0.463066
ENSG0000	RFXANK	19:191455	36.4664	39.7845	47.3276	37.812	49.706	36.7416	29.0686	38.9472	17.0296	12.2633	0.2129	-0.65488
ENSG0000	PNKD	2:2182703	52.9883	42.8232	84.3703	69.3324	48.8284	64.4226	69.2142	66.2199	37.8698	15.994	0.704	-0.34903
ENSG0000	LINC0157	16:722833	0.188861	0.168629	0	0	0.045826	4.00092	1.38851	0.201931	1.03878	0.56084	0.03005	4.31865
ENSG0000	ANKRD6	6:8943316	1.91787	0.855708	0.338312	1.72412	0.718268	0.287684	0.04697	0.243488	0	0.952347	0.00735	-2.16173
ENSG0000	POMGNT2	3:4307923	5.88859	7.23158	8.34697	5.37132	9.49167	0	3.02434	5.49117	4.52897	10.5818	0.3985	-0.57496
ENSG0000	PLCD3	17:450233	0.285298	1.0284	0.128978	0.379557	0.321778	2.09333	0	0	0	0.189972	0.92975	-0.09157
ENSG0000	GPRC5D	12:129277	0	1.34443	0	1.8072	0.438444	0	0	0	0	0	0.01105	#NAME?
ENSG0000	AHR	7:1727983	5.01391	5.57586	0.256009	0.668926	0.599693	6.12492	6.57146	4.69173	6.26497	3.69478	0.0181	1.37016
ENSG0000	VPS8	3:1848121	19.2242	21.1353	4.7794	13.7069	5.18263	15.0093	11.0367	9.67104	11.2559	9.50042	0.6752	-0.19009
ENSG0000	MAFK	7:1530713	0.941436	1.72902	0.702704	0.485186	1.04495	1.31881	3.09456	1.85749	1.24163	1.6992	0.27285	0.839719
ENSG0000	RP11-864	11:747457	1.35471	1.50705	1.72947	1.00943	0	1.15053	1.64655	0.813509	0.459135	0	0.81015	-0.44205
ENSG0000	NEK8	17:287193	4.80922	7.42567	3.80199	5.04148	1.37226	0.496822	7.03169	4.36931	3.33918	2.84306	0.85285	-0.18133
ENSG0000	RP11-425	6:1093827	0.54698	0.367156	0.918739	2.3786	0.770137	1.89424	1.05469	2.92802	0.07473	0.544906	0.8885	0.28448
ENSG0000	AC016999	2:2384267	0.138656	0	0.473322	1.82177	0	0.132227	0	0.124311	0.454282	0.691152	0.78395	-0.5806
ENSG0000	ZNF337-A	20:256129	4.66198	4.45817	1.53197	1.95598	1.15771	2.68728	2.52869	0.121136	1.86715	1.28566	0.5926	-0.36927
ENSG0000	S100B	21:465989	4.97552	18.5719	24.3157	17.7885	33.7527	15.8134	2.27755	9.7421	0	3.17776	0.3414	-1.80837
ENSG0000	VPS4A	16:691055	49.9793	56.7006	26.7938	30.7396	29.7636	49.0071	29.0781	28.504	30.7626	22.7997	0.758	-0.23794
ENSG0000	PTMA	2:2317068	1214.47	1201.76	1973.82	1598.57	2870.7	1616.39	2006.43	2174.6	1906.38	1120.33	0.88205	-0.06998
ENSG0000	RP11-199	17:766177	0.96942	2.33749	1.49011	2.61894	3.12579	0	1.0758	3.68288	2.67166	3.77335	0.95355	0.088695
ENSG0000	UBALD2	17:762652	4.72333	4.60009	4.06181	4.97525	6.22766	6.62587	7.12201	5.57356	2.23403	3.4131	0.8466	-0.10354
ENSG0000	EML4	2:4214323	100.848	97.8056	134.84	195.371	261.687	180.401	168.484	168.09	275.898	375.342	0.4309	0.43564
ENSG0000	HSPA4	5:1330519	36.5897	32.0877	24.1012	22.6109	27.0463	32.1167	48.4971	40.2877	25.4076	27.4846	0.9405	0.032736
ENSG0000	RP11-297	16:674813	2.96776	7.51632	3.87551	4.51188	1.73955	4.20529	0.778987	0.599027	8.21521	5.32677	0.95635	0.052427
ENSG0000	RP11-73C	6:1300136	27.374	16.094	19.1382	19.9465	12.0405	7.70389	6.50737	6.99718	2.95978	8.72863	0.22605	-1.43925
ENSG0000	TNPO1	5:7279440	6.7629	5.05158	9.7393	13.031	10.2824	6.02131	3.41994	10.2795	9.40078	24.5121	0.97125	-0.01596
ENSG0000	USO1	4:7572459	36.0039	22.8465	10.9936	14.0013	11.3246	23.0677	16.6436	14.2653	13.1806	22.0317	0.9214	-0.03361
ENSG0000	SPCS1	3:5249533	157.656	153.18	162.311	175.146	120.96	196.599	232.339	187.405	209.875	57.9246	0.7924	0.204193
ENSG0000	RGS13	1:1926361	0	0	0	2.33088	0	0	0	0	0	0.208484	0.48735	-3.99331
ENSG0000	RP11-262	9:6285699	0.535704	2.23523	1.04968	1.69174	0.628769	3.75359	0.815196	0.971595	3.69064	1.09512	0.75405	0.311795
ENSG0000	RBM38	20:573841	80.3304	115.011	45.8764	62.3322	47.3941	65.4963	105.101	75.4413	66.5186	75.1715	0.76495	0.092017
ENSG0000	RN7SL2	14:498611	103.825	25.9729	2853.27	179.344	588.696	65.1558	60.1827	5071.97	825.336	326.189	0.2577	0.840145
ENSG0000	ATP5F1	1:1114386	215.099	157.499	288.263	242.633	211.307	276.007	254.604	170.941	201.663	82.6263	0.56205	-0.24882
ENSG0000	UQCRH	1:4630363	152.062	131.905	204.119	219.172	130.717	230.1	183.809	237.667	171.3	41.1591	0.99105	0.003793
ENSG0000	HMOX2	16:446167	147.486	116.057	169.174	173.466	182.581	155.467	172.491	165.318	150.762	101.725	0.71985	-0.1496
ENSG0000	UBQLN1	9:8365996	34.5456	43.9241	110.855	50.578	146.459	49.3052	54.532	66.0166	59.7271	161.41	0.6831	-0.15722
ENSG0000	RP11-53I6	18:320183	4.40144	10.6323	13.2682	3.45562	9.58259	6.37322	8.2842	8.26892	13.4148	18.2623	0.9878	-0.02652
ENSG0000	CD4	12:678685	40.9903	114.174	6.49211	4.58666	1.33543	118.391	76.315	26.1886	36.0519	46.7429	0.0183	0.991993
ENSG0000	RRM2	2:1012232	24.552	43.2909	1.7516	3.81939	3.61733	38.6313	33.2802	14.1157	12.4616	20.4107	0.3634	0.436096
ENSG0000	HFE	6:2608728	1.13599	0	0.747127	0.77121	0.231908	0	0.060557	1.7699	0	0.521556	0.69235	-0.34172
ENSG0000	HIBCH	2:1898796	29.9416	35.038	15.8558	14.7916	20.4608	36.1869	46.3095	14.5576	13.381	23.9489	0.6835	0.200239
ENSG0000	AC006128	19:153533	8.84479	15.3279	1.5214	2.99957	1.0702	12.0502	9.539	2.27961	6.46358	2.32589	0.91625	0.203487
ENSG0000	CAMSAP2	1:2007395	0.066657	1.49413	0.021175	0	0.064738	3.28964	0.327108	0.511859	0.063647	0.169052	0.2447	1.67143
ENSG0000	RBM27	5:1462035	8.101	7.03574	4.83912	2.91459	4.64647	6.54464	7.94588	3.10985	0.688364	8.13893	0.71305	0.2557
ENSG0000	EAF1-AS1	3:1538127	3.82219	0.602185	1.67279	2.87568	1.69528	0	0.188367	0.079326	0	1.84698	0.07845	-2.14859
ENSG0000	NEK7	1:1981569	59.4656	38.0564	19.4341	43.3397	11.4451	27.6122	14.2331	26.6212	28.2691	18.0417	0.0806	-0.57594
ENSG0000	TCERG1	5:1464473	38.3601	34.2874	42.9407	34.2166	49.4527	37.4948	27.257	45.473	30.824	72.2906	0.8421	-0.07068
ENSG0000	RALB	2:1202400	38.2082	28.146	18.4603	11.9799	17.7377	62.1493	39.7014	23.646	12.304	14.1275	0.3529	0.349375
ENSG0000	KYAT1	9:1288329	6.5858	14.7884	6.44137	4.50041	7.16501	23.0036	5.52026	6.63346	0	5.08735	0.9833	-0.02431
ENSG0000	UROD	1:4501214	62.609	62.4054	79.8893	108.051	77.8035	87.1684	68.0574	74.8132	76.6391	43.3054	0.5633	-0.18003
ENSG0000	ORC2	2:2009089	10.2005	6.50435	4.56755	4.8874	3.30378	3.52035	5.5272	6.20321	5.39915	8.39737	0.9492	-0.066
ENSG0000	RP11-793	12:535066	0.794281	1.12399	0.828682	0.441169	0.252489	2.78338	3.34478	0.988356	2.48052	1.93022	0.2901	1.3397
ENSG0000	SGSH	17:801699	28.141	41.8178	27.9123	33.4975	35.1116	27.2873	24.5072	18.5023	25.1099	43.6323	0.4611	-0.2561
ENSG0000	AC005786	19:350627	0.251907	1.41048	0.037179	0	0.23564	0.142906	0.050269	0.701645	1.17342	0.313504	0.72295	0.560352
ENSG0000	CFL2	14:347067	3.87559	2.48505	1.61402	3.94173	2.211	1.92115	3.97513	2.89401	9.70398	2.2735	0.3117	0.666741
ENSG0000	NUPR1	16:285327	0	0	0.022102	0	0	5.20074	0.825002	0	0.897024	0.299672	0.25575	8.30194
ENSG0000	RP11-277	12:103637	0.240255	0.411179	0.195343	0.131504	0.063481	0.357685	4.9461	1.3286	0.063407	1.2	0.0411	3.1096
ENSG0000	RCAN1	21:345131	13.722	6.53815	7.32213	1.67506	8.64323	7.54373	4.56064	4.34248	2.11319	5.53056	0.3188	-0.61079
ENSG0000	NCAPG	4:1781090	5.27212	9.15628	0	0	0.165189	6.90578	7.01631	0.610867	2.18813	2.69613	0.6679	0.459487
ENSG0000	EPS15L1	19:163552	8.60807	7.96811	5.16747	7.99393	6.41227	6.01717	4.34966	3.78895	4.79423	8.25303	0.59885	-0.33604
ENSG0000	PHC3	3:1700867	18.8601	15.7874	53.2441	43.0199	64.0337	5.97511	14.1931	43.3334	84.7982	87.8005	0.9172	0.084545
ENSG0000	GPSM3	6:3219076	42.1795	36.7993	57.5024	55.8759	102.067	21.8039	16.5907	65.9932	53.636	66.061	0.41525	-0.33208
ENSG0000	KLHL9	9:2132967	10.6974	11.3141	5.4369	3.64349	4.22552	7.98265	5.69048	9.3511	5.02207	10.2918	0.8197	0.080262
ENSG0000	CBLL1	7:1077428	29.1733	42.2443	55.3819	51.8484	86.3463	9.11775	11.3594	44.1609	62.7351	63.7375	0.18075	-0.67338
ENSG0000	BUD13	11:116748	10.7144	12.0837	11.3442	6.06338	7.93818	9.11384	14.0323	7.85437	11.9307	7.78706	0.82035	0.097554
ENSG0000	EEF1DP3	13:318468	1.43023	2.07683	0.848501	0.469929	1.90943	0	0	0	3.59654	1.81575	0.9109	0.184167
ENSG0000	RNF217	6:1249090	0.008613	0.03635	0	0	0.002492	0.091957	0	0	1.80212	0.011798	0.02605	6.15091
ENSG0000	CCDC136	7:1287907	2.92584	6.7639	1.28411	0.965456	0.906394	0.428622	3.1307	2.16618	0	2.03171	0.44795	-0.62418
ENSG0000	ZNF267	16:318737	15.1338	11.2907	5.46497	8.037	4.73225	18.6951	18.8999	10.4706	11.7191	11.8336	0.03505	0.738467
ENSG0000	SF3B5	6:1440948	190.081	124.135	306.469	375.526	210.203	226.705	229.468	259.779	268.879	44.9225	0.38235	-0.29709
ENSG0000	ARG2	14:676197	0.42132	1.07909	0.646567	0.181081	0.285238	0.030935	2.22133	0	0	0.240034	0.8525	-0.27435
ENSG0000	AC005775	19:489175	0.334419	0.402058	0.07784	0.701613	0.235553	0.083321	0.650697	0	1.56225	0.363871	0.56255	0.974634
ENSG0000	TOMM5	9:3751089	140.775	78.6782	224.952	222.212	104.67	209.203	146.084	181.539	164.219	35.0942	0.8672	-0.09537
ENSG0000	TBC1D10E	16:303554	9.23419	12.7182	5.52816	3.76817	6.36734	8.33342	9.75667	7.01513	0.115624	10.7233	0.9949	0.003394
ENSG0000	CSTF2	X:1008203	15.398	19.1753	17.7029	16.7019	15.1637	34.6808	20.9636	19.5095	8.00174	22.7691	0.49855	0.259621
ENSG0000	CHCHD2	7:5610156	388.627	260.367	454.479	436.849	271.841	446.345	450.332	480.542	403.937	72.5354	0.9939	-0.00301
ENSG0000	SLC9A3R2	16:202535	1.6766	1.70582	1.11181	0.031254	2.67884	2.68512	0	1.39748	0.062928	2.63006	0.8477	-0.18219
ENSG0000	RP11-442	10:722739	0	0	0	0.35734	0.411776	0	0	0	0	1.90378	0.5685	-1.2684
ENSG0000	FPR2	19:517451	4.80556	6.36017	0.012551	0	0.491054	15.9423	0.712941	0	0	0.18322	0.78985	0.47148
ENSG0000	AC013474	2:1266559	0.692934	1.60752	0.106466	0	0.17755	0	0.88402	0	2.05109	0	0.8312	0.452438

ENSG0000	TIFA	4:1122745	10.7977	11.6613	14.7021	10.9015	10.1078	21.0183	6.64273	22.4193	18.9543	9.31605	0.7291	0.262517
ENSG0000	PHC1	12:868159	14.0878	21.7295	3.34436	2.93029	3.49592	17.0221	11.3966	7.35953	8.07557	13.1505	0.6693	0.335491
ENSG0000	CTD-2303	17:185112	0.575763	1.33349	0.472359	0.095673	0.447625	0	1.8528	0.732447	0.583601	1.17915	0.75325	0.322966
ENSG0000	WASH1	9:14520-3	2.44455	1.65633	2.30922	1.49287	1.85582	4.47459	1.43417	4.35424	1.16458	3.39885	0.5363	0.706526
ENSG0000	ZNF326	1:8999511	7.85378	8.88903	3.48632	4.00975	7.18504	11.0199	4.90133	2.74815	5.26113	9.26965	0.9376	-0.04977
ENSG0000	MAPKAPK	3:5060648	29.1851	30.3183	38.1385	39.6095	41.8324	33.5565	51.6326	47.4579	31.7436	40.8603	0.71345	0.215842
ENSG0000	SGPL1	10:708159	5.05964	4.19578	7.14073	2.34268	3.80108	6.24768	5.87761	7.58729	2.97698	9.82925	0.2635	0.408029
ENSG0000	USP35	11:781397	0.931909	0.155295	0.411293	1.05448	0.556103	1.75694	0.107757	0.071123	0.421948	0.880056	0.81195	-0.2504
ENSG0000	KLHDC9	1:1610983	0.142548	0	0.676015	1.24778	0.538445	0	0	0.598847	1.39359	1.15242	0.6483	0.507349
ENSG0000	FCGR1B	1:1210873	0.464036	0.073571	0.21519	0.298211	0	18.527	0.034278	2.02797	0.016486	0.435061	0.0201	4.44775
ENSG0000	RP11-479	10:328872	0.644793	1.45942	0.208811	0	0.122514	0	2.33845	0.417249	0	1.3738	0.72635	0.780956
ENSG0000	LY96	8:7399135	11.4512	9.78647	0.494119	2.12837	0.526502	76.0721	8.08427	7.81867	15.6454	2.43918	0.01935	2.33689
ENSG0000	IL2	4:1224514	0	0.265505	0	0	0	0	1.39483	1.72132	2.1501	0.243095	0.23905	4.8123
ENSG0000	PCBP1-AS	2:6996008	17.966	21.5396	20.2404	24.1149	13.7082	19.4847	24.1997	23.1084	20.4645	20.3985	0.88145	0.077005
ENSG0000	ZNF827	4:1456801	1.95419	2.38026	2.63128	1.28743	2.15183	1.04956	0.993073	2.18575	1.04841	8.93687	0.4903	0.347381
ENSG0000	RP5-1000	X:7791065	0.01943	2.22943	0.247233	2.06556	1.23896	0.172016	1.82385	0.714528	2.08644	0.562264	0.85915	-0.311
ENSG0000	MECR	1:2919287	11.3423	9.11097	22.5016	17.0622	14.2889	13.4653	11.5935	11.1075	16.8399	5.5858	0.44515	-0.38538
ENSG0000	EPB41L4A	5:1121424	3.8045	4.92272	11.7756	11.9784	4.05764	7.53212	4.80656	8.39347	7.89903	0.923178	0.3474	-0.79865
ENSG0000	SCGB3A1	5:1805901	0	1.91866	0.683597	0.522643	0.917484	0	0	0.603478	0	0.100804	0.32225	-2.29219
ENSG0000	USP49	6:4177017	0.424866	1.13645	0.543955	0.13008	0.1564	0.384113	0.015049	1.39448	0	0.814073	0.90295	0.152919
ENSG0000	SETD6	16:585154	11.8907	20.4641	6.50144	12.1175	10.7925	4.55999	11.2641	5.04926	4.47405	12.6815	0.1959	-0.75263
ENSG0000	ELMOD2	4:1405241	7.11607	7.97207	3.31357	3.56959	2.36345	6.33893	4.85065	7.43726	7.69643	4.83461	0.4687	0.366923
ENSG0000	COPS3	17:172468	61.2323	63.7449	49.6145	51.9231	42.6403	73.955	57.318	72.7136	60.2133	24.9204	0.59465	0.153178
ENSG0000	UCP2	11:739746	576.426	621.984	617.319	685.858	616.516	479.513	261.992	254.408	575.506	445.784	0.0931	-0.65875
ENSG0000	KIF2A	5:6230616	86.8577	82.7116	60.1554	44.5274	119.882	62.3526	57.981	36.9326	15.7458	70.6562	0.11485	-0.86228
ENSG0000	CAMSAP1	9:1358084	1.53649	1.11802	1.10329	0.625076	1.7	4.92957	2.01441	1.53309	0.411475	3.71686	0.10475	0.879413
ENSG0000	TTTY14	Y:1887250	2.43643	4.71164	1.43873	0.181865	1.59447	4.20971	0.763885	1.09073	0	0.809489	0.6387	-0.53749
ENSG0000	SH3GLB1	1:8670456	21.5959	22.7301	24.7411	17.7312	18.2035	61.2169	43.2016	30.4539	20.1752	17.667	0.37295	0.469831
ENSG0000	ZNF671	19:576819	12.0042	14.1458	11.1905	10.4965	7.95545	6.68602	4.59965	10.1523	8.90781	11.2756	0.40075	-0.45413
ENSG0000	ODF2	9:1284551	39.3172	39.8211	19.7456	16.7934	18.5656	25.2944	24.4154	13.2033	14.7516	23.708	0.1966	-0.42934
ENSG0000	MTERF1	7:9169200	15.3028	19.4848	12.77	6.47408	11.5465	7.46889	14.276	11.6022	21.9918	16.0947	0.7727	0.128617
ENSG0000	PAQR4	16:296394	5.64287	9.33396	0.795269	0.470261	1.93256	5.89907	12.3895	5.64263	0	5.84245	0.4783	0.679069
ENSG0000	STX18-AS	4:4348139	2.85228	4.64456	4.2998	1.99509	1.37824	0	0.638271	1.31662	3.26123	1.3505	0.38985	-0.9536
ENSG0000	PLAGL1	6:1439402	1.49597	5.77861	0.957692	2.08674	2.09156	2.3557	7.16507	2.20481	3.39531	6.4296	0.10275	0.85447
ENSG0000	RBM25	14:730579	43.9243	63.7154	43.5874	27.4328	65.1778	79.8983	78.6963	43.3559	47.669	96.7926	0.5184	0.279788
ENSG0000	RP11-395	2:1136579	0.494644	0.773198	0.23393	0.547372	0.12368	0.938693	1.23504	0.205953	0.357389	0.143255	0.8119	0.436952
ENSG0000	KPNA1	3:1224162	12.2692	17.1817	10.0798	5.54498	6.57271	27.4917	13.9273	8.24716	2.38035	16.914	0.65845	-0.21463
ENSG0000	UBE4B	1:1003283	5.33346	6.03616	3.96941	4.10317	5.33355	5.97851	5.68744	9.3724	7.46009	7.87373	0.29345	0.557592
ENSG0000	DNAAF5	7:549196-	4.11702	3.2892	8.21198	8.71241	7.71839	1.80989	2.71938	7.21812	3.55186	11.8133	0.7166	-0.24606
ENSG0000	SRPK1	6:3580529	20.5055	22.6418	10.9212	9.47642	25.2385	15.7575	26.3962	14.9323	17.7446	39.254	0.86945	0.103831
ENSG0000	HIRA	22:193306	6.70924	6.86391	2.18342	2.2516	2.53646	1.21559	3.73183	1.27919	0	4.30609	0.45625	-1.04996
ENSG0000	MTA2	11:625932	8.50494	7.67268	3.84675	6.88982	6.95173	6.07215	4.93696	6.19111	8.44519	7.32953	0.9681	0.016743
ENSG0000	ZGLP1	19:103048	2.02535	5.64604	1.61454	1.14434	0.753915	1.77773	7.81477	0.750972	3.58198	2.26488	0.5856	0.634098
ENSG0000	RP11-803	2:1111959	0.645676	0.717061	0.172701	0.101666	0.099854	2.75401	2.42396	0.712586	0.235516	0.729744	0.23155	1.73985
ENSG0000	IQCH-AS1	15:672010	1.53253	0.312726	0.729984	0.93161	0.488417	0.087967	1.11937	0.052613	0.436252	0.674884	0.42	-0.88472
ENSG0000	TAB3	X:3082744	4.26821	5.12308	2.28497	1.44182	2.06036	6.93052	1.83425	3.35136	0.752664	5.03148	0.86195	0.105536
ENSG0000	GPATCH1	19:330808	8.3327	7.72224	9.60952	6.1011	5.09737	2.79543	6.7767	6.24572	7.69128	9.67444	0.68825	-0.19818
ENSG0000	TXNIP	1:1459924	1105.11	1915.47	935.179	739.951	1579.64	522.337	683.68	715.401	461.797	589.795	0.10055	-1.05587
ENSG0000	PDCD11	10:103396	6.54378	7.64814	9.44843	8.48086	14.567	4.78827	4.61638	6.54193	3.33848	19.1299	0.27675	-0.43062
ENSG0000	RP1-100J1	21:290242	0.104974	0.197028	0.169916	0.21016	0.16025	0.478718	0.081007	0.276894	0.328353	0.315649	0.5223	0.820874
ENSG0000	AC005154	7:3049662	0	0.643017	0	0	0	0	0	0	2.30768	0.383076	0.5021	2.52318
ENSG0000	RAB31	18:970816	1.69758	13.7451	1.504	6.81303	3.11816	25.6326	0.067634	3.63913	6.84048	3.45065	0.5585	0.489071
ENSG0000	TMED9	5:1775921	136.775	66.2713	229.718	371.188	230.413	132.59	94.804	194.013	240.324	110.619	0.2629	-0.41656
ENSG0000	CCDC138	2:1087867	3.73387	8.1477	1.79558	2.75081	1.59923	1.41786	3.84538	1.57648	0	1.43249	0.20775	-1.11354
ENSG0000	YWHAB	20:448856	248.874	309.588	182.159	190.771	347.574	268.041	332.131	226.684	238.618	304.494	0.9662	-0.02351
ENSG0000	TAF1B	2:9843353	9.3238	9.89111	7.44498	10.7557	9.71986	8.05958	14.0871	10.0452	14.8201	13.7751	0.6014	0.228044
ENSG0000	PDZD11	X:7028659	35.2794	29.0549	49.7972	52.5367	55.3085	58.8954	59.0393	57.9171	68.2963	29.4859	0.4497	0.287356
ENSG0000	DOCK2	5:1696372	53.2497	51.5157	14.3796	10.4385	13.6935	29.0063	39.2071	19.8131	15.3556	25.4543	0.92785	-0.03553
ENSG0000	MDP1	14:241718	16.7938	17.0844	28.8111	25.7528	11.9586	25.6351	18.4288	20.2359	12.8328	10.6942	0.77335	-0.16554
ENSG0000	STAC	3:3638034	0.120211	0.679025	0	0	0	1.51342	0.869749	0.389668	0	0	0.31625	1.65093
ENSG0000	INSIG2	2:1180884	12.878	14.61	7.37937	8.8678	5.50164	10.5093	14.9988	8.10936	3.26819	8.57675	0.857	-0.07834
ENSG0000	IMPDH1	7:1283922	46.5157	40.2966	27.5555	66.5753	41.1141	34.9865	29.1008	50.4523	16.1388	36.9562	0.1435	-0.49218
ENSG0000	XPOT	12:644043	19.8775	19.208	6.2861	8.94195	4.7283	8.06916	22.3701	12.8669	5.3524	11.0607	0.8311	-0.07455
ENSG0000	BRCA2	13:323154	2.14029	8.95785	0.207146	0.052015	0.44493	3.84516	11.9256	1.09379	3.96588	1.94184	0.2865	1.13655
ENSG0000	RORA	15:604791	90.3377	36.7788	41.0714	58.6874	74.8151	72.7835	49.5821	32.4623	100.19	96.5602	0.7524	0.144595
ENSG0000	RALGAPA	20:203895	5.69737	6.44563	1.49429	1.54703	1.84534	3.07773	2.6885	3.3409	4.28642	5.95628	0.631	0.239319
ENSG0000	ALDH3A2	17:196481	12.6227	19.9054	9.9983	9.20382	8.32573	14.2111	13.9935	6.30179	6.2863	9.41597	0.31815	-0.44527
ENSG0000	RP11-325	16:569320	0.457306	0.682461	0	0.159643	0	0.530736	0.060054	0.500208	2.27729	0.080376	0.39225	1.68798
ENSG0000	RP11-798	17:455067	1.01797	1.6518	0.825976	1.75472	1.26662	1.10099	2.23379	1.56736	1.18027	0.89408	0.2477	-1.33608
ENSG0000	FRMD6	14:514545	0	0	0	0.073779	0.028521	1.9521	0.346554	0.197962	0	0.05991	0.00095	4.22053
ENSG0000	RP4-545C	7:1441860	7.85881	5.66556	4.83413	4.53337	2.31334	5.76233	1.76578	2.59694	2.39168	0.977277	0.4596	-0.56957
ENSG0000	C6orf1	6:3424637	45.2864	31.9646	86.3527	82.8742	69.0258	84.1712	72.5241	99.4527	106.138	35.4756	0.6436	0.282786
ENSG0000	ATAD5	17:308319	3.46466	6.26157	1.0649	1.70152	1.80383	7.95137	5.02873	1.96755	1.07192	3.43553	0.51675	0.379276
ENSG0000	METTL7B	12:556815	0.072517	0	0	0	0	10.2431	0	0	0	0	0.3766	7.15504
ENSG0000	CCDC122	13:438239	0.618338	0.853651	0	0	0.171684	1.94111	1.73184	0	0.795829	1.84941	0.02525	2.96557
ENSG0000	PPP1R26P	13:483037	0.102934	0.621805	0.106391	0.421287	0.281374	1.39466	1.43254	0	0	1.64256	0.4689	1.40384
ENSG0000	RP11-564	10:122879	0	0.247755	1.0496	0.139232	0.693408	0	0.132502	0	0	0.631557	0.3971	-1.76761
ENSG0000	INO80	15:409788	4.26252	6.23915	4.48528	3.99822	5.04873	3.34647	3.57859	4.18839	0.845823	7.62274	0.9083	0.058126
ENSG0000	U2AF1L4	19:357180	38.3192	55.4797	60.7627	58.6666	44.1616	47.1541	54.8152	60.7653	55.2096	42.707	0.9869	0.006229
ENSG0000	COG3	13:454648	24.8984	16.0183	10.2579	28.2512	7.31666	14.0783	10.973	11.0478	24.3769	15.1622	0.86555	-0.13176
ENSG0000	RHBDD1	2:2268355	7.92398	9.41757	6.09547	3.38145	2.87015	10.2396	5.4163	5.41815	0	10.1355	0.7489	-0.16887
ENSG0000	SLC16A11	17:704162	2.2084	1.8967	2.89107	0.883117	1.45204	0	1.38094	1.39282	0	0.048302	0.2122	-1.84736
ENSG0000	RP11-90C	1:2036265	0.351156	0.230737	0.41056	0	0.068102	0.964658	2.69966	0	0	0.819855	0.2742	2.08984
ENSG0000	PRR11	17:591554	2.70938	3.49123	0.976331	0.460223	0.835713	2.07033	2.61245	0.264476	0.040152	3.9988	0.53205	0.549309
ENSG0000	AL358852.	6:1496581	2.41391	0.971329	1.69337	0.777012	0.164735	1.59435	0.254643	0.292515	1.23693	0.4443	0.7017	-0.59133
ENSG0000	MLLT6	17:387024	18.7805	21.0088	16.4211	6.74954	29.9399	31.1373	24.1304	17.0467	7.59309	51.9613	0.28315	0.416942
ENSG0000	BCL6	3:1876982	2.47442	1.49665	1.99971	0.985456	0.814349	2.41186	0.949223	3.15963	1.26947	3.17122	0.4901	0.438383
ENSG0000	BMP1	8:2216473	1.67763	0.448684	0.640977	1.49483	1.79452	0	2.60386	1.15466	0	1.33057	0.383	-0.64894
ENSG0000	ZNF622	5:1645151	44.0081	53.265	56.4435	51.4322	52.9164	53.3922	37.3748	34.9382	41.3181	40.0791	0.30895	-0.33455
ENSG0000	CYBA	16:886432	1142.61	679.676	1867.95	1716.59	1503.38	1603.74	1434.37	1711.2	1676.93	459.479	0.82175	-0.09849
ENSG0000	CNP	17:419667	15.7215	24.5502	27.1504	19.8142	24.0333	23.9653	46.201	44.1231	15.0293	30.2012	0.51795	0.365527

ENSG0000	CSDE1	1:1147169	170.05	169.246	102.599	134.181	85.6183	112.97	92.6257	109.969	145.304	140.479	0.60805	-0.16389
ENSG0000	NDUFV2-/	18:910262	6.01241	9.37768	3.11259	14.9885	3.32834	9.82188	11.8729	2.64935	16.4734	5.88647	0.745	0.350005
ENSG0000	ZBED5	11:108092	28.9248	44.2707	13.5712	12.7267	18.0754	23.0101	22.524	22.8284	15.0121	35.7707	0.8172	-0.08111
ENSG0000	RP11-307	1:1549570	6.88766	8.78503	7.97131	5.65938	4.56678	9.24084	8.04867	2.6115	2.04594	5.00384	0.8037	-0.38312
ENSG0000	TRIM28	19:585440	61.4696	93.9499	38.9594	30.5799	72.0635	40.7897	59.3013	57.617	21.409	79.3598	0.9682	-0.01403
ENSG0000	MLLT3	9:2034166	14.8812	34.8281	8.34792	8.12056	10.2059	18.6907	15.9618	9.8577	12.2087	16.7738	0.7523	-0.16087
ENSG0000	NT5C	17:751099	92.0256	79.5378	94.1543	123.983	89.0156	126.236	152.381	97.6361	83.0287	82.7752	0.87925	0.056747
ENSG0000	SGCE	7:9458522	0	0.287143	0.21364	0	0.347985	0	0	2.28712	0	0.133987	0.45715	1.18639
ENSG0000	PFKP	10:306633	33.6997	33.4264	48.2219	35.5814	38.0356	73.3361	75.5461	62.1566	37.8402	43.9437	0.195	0.520587
ENSG0000	ZNF74	22:203941	6.35693	4.31975	2.6114	4.55765	5.4742	4.13467	0.25468	1.75044	3.68093	5.33143	0.3653	-0.6119
ENSG0000	STXBP4	17:548993	4.04502	3.15852	1.19537	0.434736	0.63626	6.9795	2.60904	1.16912	4.30267	2.05585	0.43365	0.827445
ENSG0000	ASXL2	2:2573375	5.02544	7.01671	0.912614	0.646785	1.7628	2.8513	5.97166	2.7612	0.422079	6.36876	0.8342	-0.12263
ENSG0000	CTC-471J	19:521130	0.229473	0.445574	0.165268	0.371202	0.713541	0.088088	0.335983	1.28555	0	0.191206	0.9071	0.179962
ENSG0000	PCIF1	20:459346	58.4457	69.1811	66.13	82.0232	49.9362	42.3391	56.4346	43.559	47.2262	44.7918	0.37435	-0.49369
ENSG0000	RP11-382.	15:831074	0.493426	1.66836	0.627795	0.54371	0.211626	1.64509	1.54005	1.77148	0.007529	0.149544	0.43745	0.6417
ENSG0000	HIST1H3H	6:2781006	11.2766	3.49356	6.72266	7.45236	2.524	4.22474	0.81284	4.83983	10.6907	1.37522	0.51005	-0.45986
ENSG0000	KMT2B	19:357180	2.56855	3.16471	2.04213	1.76368	3.71706	8.56197	5.83977	2.28463	5.1991	4.87953	0.225	0.958039
ENSG0000	RN7SL737	12:934674	0.013826	0.240714	0	0	0.570389	1.61357	0.095438	0.146211	0	0.71214	0.528	2.58156
ENSG0000	CCL24	7:7581166	0	0	0	0	0	2.1008	0.542596	0	0	0	0.08325	inf
ENSG0000	IL26	12:679894	0.684593	0	2.04728	0.464058	0.166925	4.12749	9.13832	4.42407	18.175	0.632503	0.1124	3.37286
ENSG0000	GARNL3	9:1272242	1.48651	1.20622	0.496762	0	0.07994	0.238365	0.659575	0.187553	0	2.20856	0.6844	0.302825
ENSG0000	TMEM164	X:1100026	2.3811	1.73423	2.42985	2.08535	2.33437	1.94429	4.10648	1.99334	1.22874	4.42453	0.6919	0.226942
ENSG0000	PHB	17:493707	118.293	66.011	129.76	130.598	199.054	143.74	111.312	94.6244	97.3261	53.346	0.24085	-0.39119
ENSG0000	GLUD1P3	10:737305	3.40082	7.57386	7.94943	8.37747	3.2982	0.974137	2.97578	5.03197	9.75804	7.80008	0.80345	-0.19093
ENSG0000	AL163953.	14:531690	1.03335	0.903613	0.13884	1.20931	0.084279	1.28367	0.562013	0	0.371156	0.111296	0.67385	-0.4575
ENSG0000	OXSM	3:2571894	11.712	12.1444	13.0725	14.8571	11.7917	10.769	11.9366	3.63992	15.4851	8.78971	0.76095	-0.27439
ENSG0000	GTF2IP1	7:7518538	38.7678	40.0233	13.8727	11.7981	17.4248	26.9611	21.2297	16.5696	6.47223	28.291	0.43135	-0.47943
ENSG0000	DYRK1A	21:373654	4.57427	5.25961	4.72418	3.76082	6.20601	3.88166	3.85344	4.09477	2.2729	12.2358	0.7338	-0.18253
ENSG0000	AC005104	2:2412272	0.88154	1.33521	0	0	0	1.32794	2.09183	0	0	0.170267	0.71495	0.81211
ENSG0000	MSH2	2:4740296	22.505	28.3494	8.95902	15.2837	7.79737	19.6086	17.2725	9.49135	9.11262	14.4111	0.55805	-0.21228
ENSG0000	PLPP2	19:281039	15.5552	0	5.36124	4.53095	17.2162	7.13765	5.21141	4.17878	6.71527	6.40578	0.5227	-0.59736
ENSG0000	FDX2	19:103048	11.0704	9.70845	15.5296	14.4297	14.6048	17.4654	16.2354	20.3243	14.3947	10.7261	0.8016	0.201704
ENSG0000	LIMS4	2:1103863	9.19834	11.6155	11.0498	10.1797	9.86119	16.8373	7.4432	4.65776	4.94249	16.0525	0.98315	-0.03646
ENSG0000	DCUN1D3	16:206102	0.49885	0.655215	0.580742	0.34003	0.2941	0.134487	0.816127	0.815034	0.022864	0.413811	0.78995	-0.34995
ENSG0000	SNX2	5:1227749	70.5489	84.5418	52.9743	56.3015	44.0024	82.2351	36.53	37.4468	57.3368	23.4752	0.22795	-0.38551
ENSG0000	PDLIM4	5:1321848	0	0	0	0	0	2.04118	2.37819	2.23529	0	0	0.00005	inf
ENSG0000	RDH5	12:556815	1.23513	4.67096	1.11747	4.37628	1.19428	2.86552	7.96473	2.15803	3.52853	4.58687	0.49755	0.68142
ENSG0000	RP11-463	12:122865	0.611924	0.766608	0.388236	1.21476	0.141896	0.364237	0.406436	0.384655	0.529831	0.877008	0.6439	-0.53341
ENSG0000	CABIN1	22:240111	8.19611	9.18083	6.58159	5.30113	7.64483	13.9725	10.3626	9.93241	13.3089	21.5243	0.0987	0.815919
ENSG0000	GBAS	7:5588727	34.0661	42.151	37.4827	35.8853	22.0845	40.3789	25.2967	19.4929	29.9152	13.0171	0.24005	-0.43628
ENSG0000	MEAF6	1:3749257	130.449	114.491	142.597	137.347	113.537	119.827	152.863	92.1362	102.2	52.1065	0.29845	-0.34805
ENSG0000	RP4-671C	22:441729	5.4709	5.57865	1.71864	3.57582	0.837049	4.20689	2.84381	2.20121	2.73989	2.04229	0.9483	0.07786
ENSG0000	ZFAND6	15:800595	67.298	78.768	102.602	95.4722	70.5386	106.353	97.689	75.1306	108.608	46.7046	0.9045	0.041755
ENSG0000	SRSF9	12:120438	214.279	211.816	342.661	306.114	344.617	234.029	260.044	298.708	209.376	252.652	0.69695	-0.22703
ENSG0000	TNIP2	4:2741647	48.3984	34.3118	45.3595	67.0447	54.1344	73.2997	115.407	79.4059	38.4646	52.5615	0.18645	0.440038
ENSG0000	MIPEPP3	13:212981	0.651835	0	1.19445	1.12649	0.521554	0	0.335533	0.757378	0	0	0.30785	-1.65202
ENSG0000	RP11-756.	18:132174	0.726173	3.96292	0	0.076655	0	0	1.21846	0	0.667645	0.662555	0.76305	-0.61891
ENSG0000	KRT2	12:526445	0.097812	0.9049	0.682458	0.80846	0.126287	0	0	0	1.17927	0	0.72555	-0.89712
ENSG0000	TUFT1	1:1515403	1.02502	0.745702	0.879071	0	0.311644	0	0.302859	0.722682	10.8009	1.03098	0.20625	2.33342
ENSG0000	ATXN3	14:919659	17.7362	24.5755	12.2479	11.8402	12.867	25.5855	31.1021	18.6061	18.4043	24.5537	0.30195	0.50681
ENSG0000	TMEM134	11:674617	63.9377	57.0454	122.257	61.73	77.4646	91.9518	96.2195	82.1137	74.6264	26.3762	0.68305	-0.18721
ENSG0000	RRP7BP	22:425536	0.870898	1.17713	20.8882	15.7775	10.2115	0.990057	1.14617	21.1957	8.18352	28.7739	0.6459	0.330256
ENSG0000	MED26	19:164790	3.7978	3.51209	1.60451	1.36534	3.00107	2.45082	3.06871	3.04808	2.39575	5.86932	0.719	0.360921
ENSG0000	NONOP2	2:6093681	0.35469	0.135003	0.186169	0	0.219377	0	0.69027	1.82293	0	1.14595	0.31705	2.03667
ENSG0000	TEX12	11:112024	0.529843	0.070615	1.42754	0.634672	0	0	0	0	2.89664	0.072346	0.9683	0.067927
ENSG0000	CSNK1E	22:382906	23.4739	16.834	19.7254	19.8452	27.8787	19.6627	32.7831	10.1257	6.23559	25.7708	0.39505	-0.40459
ENSG0000	MOCS2	5:5309567	34.8531	30.8101	27.0543	21.6937	23.5312	43.6581	27.0865	28.3275	22.5042	9.84984	0.9155	-0.04165
ENSG0000	MOV10	1:1126731	34.8226	46.5604	30.2889	16.2181	28.7262	24.6629	46.6092	29.6678	22.2268	42.5404	0.4449	0.268206
ENSG0000	VDAC2	10:752101	91.939	94.3669	114.978	101.813	100.072	109.116	120.735	91.0268	90.7444	53.0835	0.67175	-0.13126
ENSG0000	PFN2	3:1497611	0.609708	1.47726	0.369534	1.04741	0.801741	0	0.464938	1.14966	0	0.573995	0.27885	-0.95648
ENSG0000	LONRF1	8:1272189	4.00785	2.97088	1.0744	4.55069	0.990363	0.059933	8.97082	2.54292	0.135981	2.74732	0.7375	0.211872
ENSG0000	TMEM181	6:1585364	8.10828	7.84036	3.5593	3.91662	3.69028	6.10823	9.67078	4.34758	3.45579	9.74755	0.45955	0.296562
ENSG0000	FRG1EP	20:294801	1.43533	1.29177	0.275509	0.089522	0.212575	0.736696	0.536652	0	0.104293	0	0.48485	-1.10728
ENSG0000	MKL1	22:403464	12.0544	12.8494	6.67945	7.70623	19.2359	11.7298	10.5154	11.5688	10.7347	16.9057	0.98405	0.015473
ENSG0000	CMTM1	16:665525	2.97047	1.08013	1.43121	1.6461	3.47969	5.15675	4.16968	3.36567	6.51419	5.17661	0.35815	1.17299
ENSG0000	GALNT7	4:1731319	12.115	11.3097	2.5812	7.01017	2.70295	6.6155	4.12931	4.63517	6.40993	4.76004	0.279	-0.44217
ENSG0000	SMCR8	17:183153	0.769492	0.663357	0.311159	0.240237	0.97038	0.892442	0.342252	0.702942	1.21514	1.16909	0.6453	0.525531
ENSG0000	GPAA1	8:1440825	69.3736	68.5258	36.8146	40.9521	48.2206	80.6585	71.7335	43.2197	27.6702	53.073	0.9367	-0.02461
ENSG0000	FAM162A	3:1223841	22.1115	20.0146	43.6371	26.0514	18.4318	31.91	37.6791	42.0615	30.6534	6.20804	0.98255	0.012448
ENSG0000	AC109333	17:499794	0.895513	1.50617	0.65371	0	0.693253	1.71401	1.22188	0	1.2508	2.47695	0.59535	0.932078
ENSG0000	ACSS2	20:348447	3.41022	9.11438	2.65117	0.874429	0.869624	5.00151	3.8605	0.923748	0	0.890275	0.52345	-0.45116
ENSG0000	CA5BP1	X:1567493	10.0717	10.3121	15.8658	9.38592	20.1492	9.02544	16.5715	10.6324	11.5577	10.7995	0.76725	-0.24376
ENSG0000	SEC61B	9:9922206	208.123	121.236	304.874	277.724	171.48	246.566	250.711	233.58	204.799	55.2734	0.61215	-0.17545
ENSG0000	GAS8	16:900048	0.700164	1.40342	1.23578	1.54667	0.803051	2.1575	0.199869	1.34615	4.06231	3.60618	0.06195	1.34703
ENSG0000	MRPL40	22:193306	46.5451	36.6514	73.4398	64.078	43.2512	58.4288	46.4702	40.8672	17.7987	9.86281	0.5243	-0.65916
ENSG0000	PRPF40A	2:1526515	79.759	93.3306	112.855	91.7531	96.9129	88.1176	114.867	102.248	75.7333	148.505	0.9887	0.005595
ENSG0000	CES4A	16:669885	4.31404	9.90493	1.57859	1.37693	1.40541	8.62417	1.84527	1.16728	9.73385	2.23755	0.2817	0.642502
ENSG0000	LILRA6	19:542007	2.18119	6.12279	0.278298	0.258053	0.164311	9.57483	0.199162	0.732359	0	0.102362	0.85095	0.392579
ENSG0000	ACADS	12:120725	20.6496	22.2076	38.867	39.2364	30.6857	29.1175	24.5949	32.4782	19.5697	23.1531	0.4686	-0.28108
ENSG0000	ZBTB7B	1:1550026	2.11951	3.00242	0.799002	0.269255	0.721457	1.9153	1.19638	0.846954	0.813713	2.74668	0.83785	0.139689
ENSG0000	TRIM41	5:1812224	1.67623	2.14585	1.07711	1.54841	2.67677	4.06995	1.23797	2.96252	0.169702	4.90341	0.46165	0.490374
ENSG0000	RP11-527.	2:9899726	0.373147	1.27831	0.269033	0	0.236773	0	0.635786	0.061731	0	1.25135	0.98225	0.008079
ENSG0000	ZBTB21	21:419868	1.73773	2.15827	1.20997	1.32351	1.09697	3.18722	0.809491	2.58771	0.480146	3.62069	0.97575	0.025376
ENSG0000	DOPEY1	6:8306766	3.84046	3.54671	0.287109	0.193858	0.966619	1.79359	5.12858	1.80554	0.215879	4.2937	0.4105	0.726016
ENSG0000	TOX2	20:439148	3.66252	8.36964	1.11549	0	0.591498	6.76413	16.3643	13.6675	0.541951	12.6344	0.07005	2.13156
ENSG0000	TRAM2	6:5236142	2.33193	1.92479	1.06126	0.401247	1.30453	1.81005	0.578089	2.97831	0.636293	2.16685	0.85735	0.196811
ENSG0000	KIAA0513	16:850277	1.25159	1.50732	0.924205	1.33827	0.994211	2.88136	2.86665	2.12891	2.08047	6.2035	0.0602	1.31082
ENSG0000	STXBP1	9:1275793	0.777645	1.80064	0.591044	1.75862	0.435466	0.015282	0.570903	2.58562	0.073388	1.26813	0.94095	-0.08878
ENSG0000	ROCK2	2:1117976	7.87084	11.0886	1.55518	0.599081	2.03765	12.7042	5.78265	1.19448	0.150423	5.41462	0.98345	-0.01481
ENSG0000	NXT1	20:233469	30.6898	27.7736	72.7324	104.213	52.4578	24.218	54.3267	50.5581	54.9168	16.0175	0.1239	-0.58484
ENSG0000	ZFC3H1	12:716094	26.552	50.2985	19.2979	12.7605	31.4974	39.0927	57.83	20.8202	18.0317	66.4285	0.59715	0.306055

ENSG0000	LINC0017	20:640343	0.248036	1.86424	0	0.135846	0.263481	1.40258	8.14441	2.20866	0.607293	1.0161	0.08305	2.57825
ENSG0000	MARK2	11:638389	35.4878	40.5447	7.32798	5.391	16.9254	32.4766	26.1601	11.5021	3.41019	27.5596	0.68355	-0.17789
ENSG0000	TRGV5	7:3834157	25.7269	7.96842	68.5888	53.0412	41.568	12.88	41.2828	37.2049	26.5683	17.2361	0.7298	-0.62372
ENSG0000	TRIM34	11:524808	8.44766	16.1591	6.71179	10.2689	5.16964	6.08924	6.01804	7.41848	13.8144	7.80418	0.93195	-0.04645
ENSG0000	MED13L	12:115953	3.12311	4.56749	0.551172	0.461166	1.16302	1.72129	1.95406	1.69152	3.12189	2.80923	0.8205	0.154222
ENSG0000	RP11-50D	4:1277818	11.1385	8.89328	8.65314	10.1239	1.54006	8.37078	9.13032	3.7285	5.06651	0.054536	0.94075	0.124897
ENSG0000	C11orf21	11:229564	46.2551	26.989	19.2673	19.7058	15.2176	12.8558	12.8425	15.3612	8.88274	18.3984	0.04235	-0.94983
ENSG0000	HERC4	10:679218	16.1424	22.7102	5.99714	5.24269	5.44613	12.3002	11.1922	10.6217	6.75474	14.7871	0.7768	0.126893
ENSG0000	RP11-715.	16:227588	0	0	0	0.254605	0	1.25169	0	0	1.35303	0.257781	0.1622	3.57259
ENSG0000	RHEB	7:1514660	71.2985	40.5703	68.1838	76.8553	58.124	100.982	90.0507	84.2817	71.9811	27.7108	0.65735	0.180751
ENSG0000	TGFBRAP1	2:1052643	9.49985	9.73482	4.17132	1.48557	4.72644	5.03103	3.35176	2.25816	2.72797	8.91241	0.1364	-0.76805
ENSG0000	RP4-669L	1:357382-	1.6457	4.22527	0.68918	0.556246	0.394057	1.63905	2.53948	0.838472	0.63083	2.64634	0.07505	1.17333
ENSG0000	SHISA8	22:419095	0.321479	0.716238	0.073484	1.04988	0.253027	0	0	1.98685	0	0.502811	0.9273	0.134424
ENSG0000	HLTF	3:1490301	15.0533	11.0745	8.78838	7.8439	18.1517	10.0328	11.4843	8.64928	15.7541	19.5383	0.91645	-0.07253
ENSG0000	PPIL1	6:3685482	16.6086	12.0715	18.662	18.7597	15.344	23.4039	18.7073	19.0682	46.4487	7.40412	0.50385	0.466109
ENSG0000	KDM2B	12:121400	5.97734	6.38468	3.39777	4.09343	5.72673	5.08742	15.5581	7.81758	6.87009	10.828	0.22065	0.800777
ENSG0000	KIAA1958	9:1123799	0.522746	2.64416	0.220433	0	0.026548	0	0.050577	0	0	0.23856	0.0974	-3.93542
ENSG0000	CYB5A	18:742508	26.8919	13.6386	35.4904	27.2129	28.2666	36.2672	16.0694	22.4049	19.0713	7.76276	0.20925	-0.69385
ENSG0000	XPNPEP2	X:1297389	0.844272	1.7066	1.07042	0.499043	1.18874	0	0	0.505218	2.1804	0.343522	0.60435	-0.61139
ENSG0000	RNF139-A	8:1244624	2.39716	9.86967	5.23864	4.32811	0.748545	3.17085	1.71474	4.72363	5.68362	7.69385	0.94175	-0.04639
ENSG0000	CHAD	17:504261	0.959185	0.982574	0	0.424275	0.089014	0	0	0	0	0	0.01805	#NAME?
ENSG0000	ELK4	1:2055688	3.76534	5.3304	2.76809	2.28225	5.16998	3.59989	4.58213	5.12134	3.35654	6.90595	0.7994	0.135739
ENSG0000	MRPS34	16:177188	97.181	88.0702	158.631	149.273	184.44	109.302	142.891	125.881	103.484	70.8281	0.23925	-0.40684
ENSG0000	SCARF2	22:204248	0.970897	0.537043	0.364652	0.522638	0.3721	0	1.43324	0	0	1.37836	0.977	0.03672
ENSG0000	CTD-2576	16:171017	2.22551	0.979714	2.60857	2.37139	1.39238	10.659	7.35933	9.61186	3.68734	3.31097	0.32865	1.76837
ENSG0000	RAB40C	16:589356	12.2805	13.2698	9.43092	7.24634	9.06349	6.79801	8.90488	6.70264	13.064	10.1532	0.77345	-0.14512
ENSG0000	LLNLR-28	19:999795	3.78674	3.78543	1.75612	2.10475	1.46224	2.42681	8.08414	3.50896	1.64077	3.20862	0.83125	-0.25916
ENSG0000	SPG20	13:363016	7.69247	15.7159	4.96679	2.51941	3.30993	10.4609	0.013331	2.66603	0.074693	2.28418	0.0859	-1.20917
ENSG0000	CTC-429P	19:164790	1.68451	5.07529	6.67718	4.59106	2.06814	1.98621	2.47067	7.35071	1.53104	7.45541	0.98835	0.027041
ENSG0000	PGGT1B	5:1152040	6.08122	5.08785	3.21332	3.37301	1.92606	7.8964	6.9082	3.11801	0.186012	3.72687	0.7328	-0.19619
ENSG0000	AC005593	5:1317974	3.6436	0.589258	3.76756	1.57843	0.542592	0	0	1.71728	0	0.125013	0.25155	-2.36117
ENSG0000	AC141586	16:260334	2.10612	4.48371	2.85204	1.78892	2.58981	2.26473	4.21312	1.79759	3.02259	5.09203	0.90965	-0.06855
ENSG0000	ATG101	12:520692	17.9434	11.7933	24.8495	16.3536	36.1376	24.5355	25.4711	40.1791	23.4704	26.2347	0.4501	0.307295
ENSG0000	ZNF573	19:377358	3.31369	8.40327	3.95589	3.47033	3.20761	1.92591	2.16134	1.21077	1.11738	5.70532	0.16545	-0.97637
ENSG0000	ABHD17A	1:1481020	1.87519	0.896474	0.107206	0.127667	0.48299	1.01308	0.889692	0.088366	0.198825	0.352566	0.729	-0.57822
ENSG0000	AC008440	19:538545	0.274641	0	1.14554	0	0.485035	0.30159	1.40065	3.18471	1.11844	0.780746	0.37	1.58095
ENSG0000	AC007285	7:2992010	0.585219	0.196995	0.082024	0.704505	0.334972	0	0	0.094345	0.103374	0.226121	0.2346	-1.92431
ENSG0000	ZNF250	8:1448764	5.53773	4.57039	10.153	4.57458	5.04848	4.06332	9.67862	10.468	8.12722	7.23532	0.54095	0.302155
ENSG0000	CRYL1	13:204036	16.3193	21.7198	17.4484	21.315	14.0887	40.4356	22.9563	14.4309	6.68742	11.5239	0.98485	-0.00823
ENSG0000	ZNF419	19:574877	6.47629	5.63441	6.37433	5.45792	4.71836	6.65269	4.79491	5.18614	11.8178	6.50469	0.64525	0.265102
ENSG0000	PDCD10	3:1676832	118.729	65.687	78.7713	87.905	49.3362	120.267	134.017	66.1254	79.1758	22.3599	0.7295	0.115639
ENSG0000	RP13-582	8:1432468	0.368432	1.31105	0	0.210466	0.548749	0	0.553555	0.136664	0	1.949	0.60855	-1.21853
ENSG0000	BNIP3	10:131966	10.9936	17.8116	14.4751	15.9985	10.8276	13.4274	5.72466	5.95922	8.53375	6.98696	0.0805	-0.90639
ENSG0000	PES1	22:305766	30.7192	25.7298	8.16329	10.8938	14.9422	17.1658	19.955	11.8981	3.70044	18.6305	0.38995	-0.34982
ENSG0000	TTC7B	14:905245	0.540636	0	0	0.546083	0.245701	4.48801	0.185354	0.529711	3.06646	1.05173	0.02115	3.09527
ENSG0000	CCNT2-A	2:1347354	1.33606	0	1.64545	0.667416	0.422898	0	0	0.717642	0	0.266933	0.2736	-1.84842
ENSG0000	DNAJA3	16:442580	43.7234	46.0667	53.5702	43.176	27.5862	35.218	39.3403	28.1262	38.0745	34.1468	0.5545	-0.26262
ENSG0000	RAD52	12:912076	3.19462	5.52245	4.7372	6.92313	2.47245	0.140819	3.72093	3.03379	1.23185	5.42264	0.36845	-0.80245
ENSG0000	ASAP1	8:1300521	8.69072	10.8154	2.72977	1.87885	5.18739	10.8282	7.84362	11.0338	1.59928	14.6338	0.2132	0.556831
ENSG0000	ZNF718	4:124479-	2.67318	1.41927	1.33919	1.57942	1.55636	1.81782	2.12337	1.42238	1.98295	2.59356	0.8892	0.099773
ENSG0000	ZNF655	7:9954630	67.3746	69.9842	82.1596	117.18	59.5494	36.2584	62.9889	60.5393	92.3155	50.0033	0.3147	-0.39404
ENSG0000	PITHD1	1:2377840	41.5744	33.0087	52.65	57.8804	59.0232	33.3056	43.8034	50.1117	71.4266	43.1704	0.89215	-0.04637
ENSG0000	MAP1LC3	12:116548	3.79455	4.30862	5.85019	6.59801	2.13571	4.93233	4.80911	4.23545	6.05045	1.44346	0.91335	-0.07943
ENSG0000	PLEKHG1	6:1505998	1.26492	4.10406	0.157398	0	0.099735	2.57041	3.10678	0.955498	0.936049	1.935	0.37545	0.934781
ENSG0000	RP11-670	17:569141	4.78553	1.88515	5.3226	6.98524	1.99231	9.66564	5.38502	4.48907	9.12998	1.79862	0.759	0.550054
ENSG0000	TAF8	6:4205051	9.43999	16.9128	22.7763	22.2613	5.08254	8.49739	7.61356	11.6423	14.0199	12.3984	0.5553	-0.51208
ENSG0000	LPIN2	18:291699	40.5985	35.1959	39.2021	55.0743	86.3759	43.3187	54.7669	59.1942	90.555	101.603	0.65085	0.389272
ENSG0000	F12	5:1774021	1.08155	0.527731	0.939416	2.77454	1.46129	0	0	0	0	0.337189	0.06265	-4.55835
ENSG0000	SLU7	5:1604016	43.5659	44.9413	66.3817	59.1921	64.0373	45.6814	56.5906	57.4969	68.484	65.8725	0.9719	0.013371
ENSG0000	AP000347	22:236084	2.75193	2.38192	0.957425	2.35542	0.572909	4.16467	2.86565	1.20137	0.454937	4.00193	0.8848	0.141414
ENSG0000	RP11-347	2:1961335	1.47795	0.952211	0.828133	0.791749	0.294315	0.535859	1.8035	2.05989	3.00258	2.62383	0.2963	1.24262
ENSG0000	BATF3	1:2126864	0.236019	3.63927	0.193132	0.450456	0.818245	5.44222	29.9263	4.6146	6.83187	2.14787	0.04115	3.21698
ENSG0000	CLCN6	1:1176178	2.59781	5.10766	3.3278	2.245	4.78162	1.43769	3.89345	6.77854	1.36998	7.7114	0.9765	-0.02151
ENSG0000	HSBP1	16:838078	22.2838	17.1468	22.3753	19.733	9.06687	54.3933	41.2713	36.1401	32.501	7.766	0.23015	0.879794
ENSG0000	RPS10-NU	6:3424637	17.7462	4.71497	4.02923	1.38991	0.948736	8.10243	6.4991	5.41017	11.8152	0.736413	0.7519	0.447688
ENSG0000	RNF167	17:493712	51.1145	48.0319	66.5549	55.8611	91.7536	85.13	62.055	77.0549	47.7648	62.0087	0.8509	0.100856
ENSG0000	RP11-545	6:1457935	2.05464	3.40169	3.00321	0.192429	0.396174	4.94637	3.43892	1.38211	0	1.65158	0.92955	-0.07122
ENSG0000	ZNF737	19:205358	3.73734	5.90262	1.41584	1.62224	1.62086	3.04758	4.20125	1.77538	0	4.45227	0.8873	-0.10605
ENSG0000	RP11-265	15:424912	11.6251	8.37111	3.91533	20.2289	0.678608	3.37761	2.11539	1.59031	21.4068	1.03849	0.74175	-0.48233
ENSG0000	BANP	16:879492	22.4353	26.7723	21.9165	27.8243	28.5503	23.4842	30.5817	20.3453	13.1172	28.8425	0.654	-0.18894
ENSG0000	STRN	2:3684363	2.97411	3.67047	3.05599	0.971721	5.61733	2.27075	4.41088	2.72889	1.98247	5.94226	0.6371	-0.3123
ENSG0000	HGF	7:8169900	0.180982	2.24725	0.004868	0	0.428369	2.44621	0	0	0	0	0.8004	-0.34731
ENSG0000	ZMYM1	1:3505978	5.12951	4.99893	3.22027	2.00242	4.67004	4.37046	2.14793	3.33946	6.74254	3.95601	0.8938	0.070523
ENSG0000	ARRDC3-/	5:9136872	2.24821	1.73587	0.866699	1.15437	0.117127	0	1.0051	0.255323	0.652812	0.665317	0.44795	-1.06661
ENSG0000	MRPL53P1	1:1124665	1.60477	3.31501	0.145779	0	0	1.74227	5.06596	1.13168	0	1.12107	0.53795	1.18252
ENSG0000	ARV1	1:2309789	22.7363	18.4275	19.132	21.2705	12.3353	27.4276	19.7632	21.1655	32.7797	10.0642	0.4989	0.273146
ENSG0000	RP11-178	16:870835	1.57064	1.38016	1.54627	4.00035	0.560313	0	0.68377	0.158701	0	0	0.1719	-3.79106
ENSG0000	AURKA	20:563693	4.51647	7.96008	0.378802	0.97376	1.31606	1.54191	4.34745	3.33153	6.75066	3.45885	0.35425	0.66217
ENSG0000	PLD1	3:1716004	4.65167	0.206739	1.61718	6.62302	2.53921	3.23528	0.756726	4.02854	5.77197	5.19387	0.98735	-0.0126
ENSG0000	DBR1	3:1381610	7.52677	9.55009	8.4393	8.75017	5.80995	3.76325	8.44228	7.48251	1.30113	9.51897	0.30085	-0.4566
ENSG0000	IER5	1:1810887	63.0158	16.8791	31.6457	49.4763	23.0396	20.2062	30.7805	30.6546	18.1177	22.2825	0.13285	-0.49448
ENSG0000	HSF5	17:584201	1.31115	2.06532	0.411686	0.043144	0.315132	0	0	0	1.68252	0.400395	0.54875	-0.72995
ENSG0000	IDI2-AS1	10:971145	2.37845	1.40997	1.09717	3.31643	0.424051	1.60001	0.931209	1.3784	3.84801	1.68264	0.96205	-0.05027
ENSG0000	UQCRB	8:9622591	114.482	62.4753	100.909	90.7756	47.5198	149.656	148.27	98.8239	98.5219	13.5898	0.4868	0.268241
ENSG0000	MCTP2	15:942315	7.64743	1.95535	2.32405	4.75562	2.46852	6.11049	4.2163	2.40465	4.23211	3.96524	0.9767	-0.02022
ENSG0000	IGKV3-11	2:8902717	0	227.257	6.08003	4.501	1.77379	0	0	0	0	0.482203	0.103	-7.95313
ENSG0000	SLC23A3	2:2190753	0.47246	1.13749	0.33206	0.06144	0.16123	0.37094	0.894592	0.378886	1.71865	0.451691	0.39785	0.861762
ENSG0000	RP11-91A	1:5202013	0.228413	0.356884	0.469716	0.068433	2.32893	0.283397	0.179039	1.5636	0	0.930887	0.7207	-0.54661
ENSG0000	CHRNA10	11:366558	0.28694	0.190137	0.356973	0	0.6195	2.43991	1.69483	1.57065	0	0.649555	0.19055	2.00564
ENSG0000	RASGRP2	11:647269	75.7046	183.034	74.9781	62.1364	78.6387	31.1308	26.0505	25.7356	41.2293	33.9071	0.0049	-1.46471
ENSG0000	TMTC3	12:881422	1.30615	1.26129	0.726996	1.1109	0.60929	1.00713	0.847331	0.617885	1.54532	1.67046	0.8871	0.093613

ENSG0000	HLA-A	6:2994125	912.838	955.043	1056.06	808.189	1383.85	898.169	1777.63	1303.81	736.255	1036.19	0.8853	0.073812
ENSG0000	ATP11A	13:112690	2.6923	5.08891	1.27556	1.02502	2.49344	1.71703	2.36422	1.19774	4.64295	5.24802	0.61715	0.302302
ENSG0000	SH2B1	16:288465	3.30276	4.00192	3.1112	3.2411	4.45176	2.7821	1.8692	3.0547	0.469387	8.46393	0.9507	0.038472
ENSG0000	RP11-717	21:555363	0.295236	0.973097	3.21759	0.662599	0.539766	1.9193	1.45317	1.96997	0	1.58739	0.9886	-0.0104
ENSG0000	MEGF6	1:3489919	2.35488	8.96518	0.386239	0.839049	0.236875	1.20717	1.36615	0.969934	0	0.769149	0.19025	-1.30837
ENSG0000	DGCR8	22:200802	1.98286	5.14481	4.55042	2.37946	3.68644	1.04527	6.50538	5.5172	1.65645	9.6687	0.74175	0.298711
ENSG0000	PLXNA3	X:1544582	1.04874	1.26555	0.677283	0.408666	1.93737	3.19402	3.2852	1.70302	0.613672	3.13	0.20305	0.931858
ENSG0000	C20orf24	20:365734	174.409	124.214	149.137	237.926	129.055	258.969	212.865	144.844	218.027	78.1791	0.8134	0.13448
ENSG0000	CAMTA2	17:496799	24.7713	24.7588	14.3266	14.6833	31.3828	39.6919	46.3133	60.4695	27.2934	41.2124	0.0559	1.20137
ENSG0000	PPP1R16A	8:1444779	5.10301	5.24097	3.55933	2.70107	2.69022	4.97837	11.2666	4.46385	3.10672	5.78021	0.3517	0.555085
ENSG0000	RP11-767	1:4562730	1.09027	0.669408	0.108376	0.065769	0.231974	3.9173	3.48631	0	0	0.430038	0.31095	1.85034
ENSG0000	PFDN5	12:532952	373.512	276.264	717.499	562.409	266.287	461.444	428.775	561.11	435.97	104.565	0.73635	-0.12402
ENSG0000	ITGB2-AS	21:448859	47.0823	77.8176	7.86179	18.9898	8.62292	56.1444	19.2494	6.16072	10.705	14.7249	0.71555	-0.54362
ENSG0000	PET117	20:181378	19.9244	18.2419	32.0005	32.321	25.6669	15.9858	13.4141	20.175	14.8131	10.5543	0.098	-0.85417
ENSG0000	U2AF1L5	21:648462	64.7154	73.1555	71.3126	61.2806	112.663	61.7005	111.56	69.3457	51.7463	54.2725	0.81	-0.23254
ENSG0000	AC005255	19:147923	0.897194	0	0.277338	0.384023	0	1.36683	2.94775	0.295183	1.20525	0	0.37695	2.23875
ENSG0000	KLF3-AS1	4:3861270	3.39841	4.24454	3.63745	1.66295	1.531	2.68752	0.017552	0.082384	0.338024	0.295373	0.11045	-2.22
ENSG0000	PLAGL2	20:321925	2.27515	5.15565	1.67363	2.38961	3.33686	2.69438	4.95329	4.87604	2.90476	7.75193	0.2608	0.543829
ENSG0000	EARS2	16:235220	6.1121	5.54923	7.73148	8.94933	5.00031	0.015933	6.38786	4.47674	6.74294	8.36549	0.36815	-0.61045
ENSG0000	PARP15	3:1225776	16.0857	20.9638	6.78788	15.5187	10.4242	7.05029	17.4767	11.2486	7.23385	23.1091	0.7188	-0.15354
ENSG0000	SMYD3	1:2457493	21.8677	17.7711	23.7055	29.3042	18.0437	23.6475	26.601	22.4953	3.46362	13.1621	0.5255	-0.27171
ENSG0000	MTR	1:2367952	5.1999	7.28798	3.12483	1.7595	5.10059	5.93116	4.17952	5.77861	0.688743	12.0822	0.7365	-0.18154
ENSG0000	WDR46	6:3327713	42.3983	43.1433	62.1828	55.9357	55.9019	32.6321	56.6569	53.5451	20.1822	45.4307	0.5364	-0.26716
ENSG0000	FAM111B	11:591071	5.05801	5.49839	0.296274	1.06653	1.0942	7.56844	5.04603	1.35728	0	3.23842	0.929	-0.09023
ENSG0000	RP11-502	15:675410	1.81689	1.66212	1.55507	0.537635	0.966082	3.04274	0.847379	1.70463	1.57355	0.550551	0.4093	0.254725
ENSG0000	PRRT3	3:9945541	1.56209	2.63471	2.50732	1.65583	1.28003	2.11667	2.83681	1.41996	0.894803	1.66096	0.68325	-0.3069
ENSG0000	PIGBOS1	15:552029	33.6726	18.0117	45.9227	38.246	43.672	32.4214	20.4974	20.7325	27.4005	19.1726	0.52315	-0.6296
ENSG0000	NEURL2	20:458886	0.129279	0.40845	0.494511	0.671641	0.854774	6.04319	2.01402	0.632057	0	0.438695	0.42825	1.14186
ENSG0000	CENPJ	13:248822	1.84649	3.2049	0.958208	1.25897	1.89984	4.24217	1.75086	1.31044	0.361338	4.74715	0.5177	0.366982
ENSG0000	UBE3C	7:1571389	11.3327	11.5345	5.21475	3.54598	5.09341	8.56984	11.2758	6.48522	4.158	11.6506	0.63805	0.178159
ENSG0000	RP11-16E	8:6321598	0.134252	0.376543	0.36112	0	0.230546	0.311698	0.01764	0.347534	0	1.86806	0.68505	0.777292
ENSG0000	ACTG1P2(	1:2732214	0.970844	1.69567	0.477099	0.564229	0.993285	0.51614	0.973959	0.088117	0.313189	3.0648	0.7833	0.403221
ENSG0000	RP11-128	20:220721	0	0	0	0.144974	0.118977	0	0	0	2.37117	0	0.50885	3.16226
ENSG0000	SAMD1	19:140878	8.04715	18.9435	2.99308	2.1004	10.5108	13.2538	13.5103	7.57764	1.50178	14.7978	0.49495	0.349621
ENSG0000	GABARAP	17:724001	185.167	223.504	216.638	201.667	452.607	360.187	334.499	208.552	237.938	140.637	0.9928	0.004053
ENSG0000	MEX3C	18:511745	11.1819	6.96254	5.241	5.3827	6.66101	2.4727	3.72508	7.46821	1.69869	10.0909	0.28485	-0.50959
ENSG0000	VAPA	18:991400	134.606	110.741	122.63	78.6389	99.226	150.409	161.525	92.5271	82.9124	56.6046	0.7472	-0.17346
ENSG0000	CD37	19:493316	64.3464	89.1058	99.7868	72.3766	158.396	66.8253	75.5306	108.455	102.446	176.397	0.7827	0.091834
ENSG0000	CCNG1	5:1634375	53.8075	50.0166	35.1037	28.2565	27.0481	44.5074	33.5644	34.4618	28.9574	26.3089	0.72505	-0.11391
ENSG0000	LINC0128	X:3930495	0.219786	3.91322	0	0.645038	0	3.36138	0.358742	3.44259	6.588	0.186318	0.2403	1.64854
ENSG0000	ESAM	11:124752	0.240258	1.26049	2.09432	2.20863	0	0	0	0	0	0.353196	0.03335	-4.14864
ENSG0000	FYTTD1	3:1976713	26.6353	21.2103	18.7612	16.9424	12.4442	19.5382	17.8421	12.5197	11.8809	16.8254	0.2983	-0.50812
ENSG0000	SETP9	1:1606701	1.33165	0	0.115126	0.672001	0.633328	0	0.177787	0	0	0.223227	0.2709	-2.72007
ENSG0000	ZFP14	19:363344	2.2557	5.10346	4.3879	4.32278	3.00157	3.99336	5.48914	3.55695	3.72546	2.97275	0.9852	0.026271
ENSG0000	BST2	19:174029	128.765	96.3257	176.582	195.245	222.499	272.373	489.597	356.948	178.13	86.9322	0.0565	0.663091
ENSG0000	EEF1A1	6:7351574	2813.43	2269.1	3452.47	2167.88	3949.47	1909.26	1778.81	2538.15	2026.22	1444.29	0.5981	-0.6152
ENSG0000	SH3GL1P3	17:681257	1.10165	1.90592	1.14153	0.316097	1.28425	0.190875	1.40508	0.915405	0	0.976087	0.5076	-0.9932
ENSG0000	STRN4	19:467195	30.9959	36.1694	4.95665	3.85751	8.32273	24.3485	25.5942	9.87369	8.47107	15.7909	0.99295	-0.00557
ENSG0000	NECAP1	12:808221	28.2262	43.0316	39.3926	39.4459	21.2316	43.9991	40.6383	32.6706	48.1622	55.626	0.4752	0.325023
ENSG0000	CENPW	6:1263401	8.46553	10.3781	1.44772	3.35457	0.829007	9.72262	11.53	5.7181	12.5536	1.06852	0.33345	0.789986
ENSG0000	RNF165	18:463268	0.782253	0	0.37186	0.691002	0.436697	0	0	0	0.169132	0	0.28165	-2.90826
ENSG0000	CTD-3025	8:6559184	0.210792	1.09526	1.36406	0	0.434979	0	1.80656	1.79412	0	3.99984	0.46025	1.33149
ENSG0000	RP11-114	15:779760	0.294244	0.750164	0.138087	0.461991	0.520118	1.08711	1.40761	1.39731	1.77579	0.994241	0.13895	2.0302
ENSG0000	TLR7	X:1286708	0.020105	0.65965	0.082902	0.196423	0.021524	2.86841	0	0.01652	0	0.009115	0.5341	1.06406
ENSG0000	NPTN	15:734431	15.932	19.8782	8.01742	12.6376	8.97829	29.4044	37.0486	15.4004	6.80878	13.6452	0.0835	0.681859
ENSG0000	RSG1	1:1623169	2.31579	0.725945	2.38252	0.451836	2.15939	0.812017	0.610062	0	0	3.07314	0.39445	-0.91078
ENSG0000	RP4-591C	20:638612	1.00307	2.00511	0.482774	0.753588	0.449165	1.14631	0.533333	0.442152	2.23987	1.4821	0.6502	0.375046
ENSG0000	RP11-448	4:1006808	0.630169	3.56639	0.742907	0.59107	0.425001	0.503997	2.18034	2.24095	0.229259	1.41317	0.8869	0.113949
ENSG0000	AC005682	7:2285417	0.506634	0.593367	0.984958	0.360923	0.526921	0.0367	0	0.27593	0	1.13865	0.3926	-1.15349
ENSG0000	RP11-290	12:760254	3.32287	0.893439	7.68269	10.3938	2.42359	9.50648	19.6534	19.0118	15.6021	8.05069	0.57395	1.04164
ENSG0000	STAU1	20:491133	31.3702	30.532	12.6858	16.5743	23.2953	33.9022	32.0292	23.3286	11.0832	36.6772	0.4148	0.287794
ENSG0000	C1QTNF6	22:371258	3.07137	2.06326	2.66302	1.3957	0.927178	5.34364	2.00491	1.62474	1.71157	4.544	0.505	0.52552
ENSG0000	AGBL5-AS	2:2704236	0.112543	0	0.094427	0.236545	0	1.05528	0	0.400661	0	0.120401	0.39335	1.79411
ENSG0000	NOP56	20:265214	131.678	142.666	145.156	135.531	162.062	138.836	122.467	95.6878	100.05	116.074	0.2227	-0.34608
ENSG0000	PUM2	2:2024869	16.0909	16.2046	7.46176	6.45893	9.52137	11.5045	10.5197	10.6076	10.9701	22.0289	0.74595	0.114212
ENSG0000	CTD-2357	17:786173	1.59034	1.92377	1.19393	0	0.102512	3.48723	4.45515	6.78345	0	0.872945	0.1355	1.79782
ENSG0000	RPS6KA4	11:643591	4.24434	4.21681	0.275502	0.434491	1.01255	1.84724	3.0421	1.06654	1.70382	1.26052	0.82035	-0.18822
ENSG0000	ALG5	13:369497	44.4478	24.6679	59.2142	55.6074	29.1539	38.9093	45.3303	40.6628	49.9417	11.7056	0.84505	-0.16064
ENSG0000	ARMC5	16:314567	8.0736	4.47982	1.39273	2.959	4.92164	3.65291	9.35132	4.82068	0.667666	6.1291	0.75245	0.268845
ENSG0000	CLN6	15:680541	2.4774	1.03855	4.45038	13.9049	6.09706	7.08246	7.76506	12.1094	21.2665	14.7637	0.29975	1.08441
ENSG0000	SSB	2:1697919	124.589	120.164	148.057	118.475	193.601	193.715	152.772	134.661	105.204	111.606	0.8561	-0.06483
ENSG0000	CCDC18-/	1:9314974	41.5419	120.073	41.781	41.2073	28.4998	34.8646	60.0727	42.9653	30.7413	63.3215	0.45055	-0.38281
ENSG0000	CHST14	15:404709	0.988386	1.18096	1.89988	1.61233	2.69433	1.82741	1.75747	1.34939	1.48431	1.67418	0.9462	-0.08985
ENSG0000	SHMT2	12:572293	38.5926	53.7254	33.0394	30.0201	31.3218	43.7808	60.7535	46.3006	25.4714	28.3941	0.8586	0.062378
ENSG0000	KLF7	2:2070741	1.62386	8.11431	0.51424	0.303311	0.097713	2.10156	1.90069	1.43298	0.377061	0.7566	0.2102	-1.00521
ENSG0000	UBL3	13:297643	14.6323	21.4369	5.59953	8.27177	6.30365	8.45548	11.3905	10.7264	17.6349	14.2742	0.5373	0.235457
ENSG0000	ANKRD26	10:269919	2.99324	10.5116	2.62867	1.0663	2.12507	5.89418	4.92862	1.29062	3.2174	4.82613	0.93015	-0.04106
ENSG0000	TSPAN31	12:577237	33.4303	14.4071	49.7083	45.5177	33.9845	28.2392	25.0508	38.1666	49.1978	31.3305	0.9066	-0.07301
ENSG0000	NECTIN1	11:119608	0.181235	0.240991	0.082981	2.73313	1.31162	0	0.136165	0.938861	0	1.54584	0.49765	-0.88443
ENSG0000	RBM19	12:113816	10.3107	18.2402	15.4039	12.5954	6.62859	13.5575	11.5393	11.5423	14.4668	16.5735	0.6677	0.218816
ENSG0000	PSEN1	14:731364	22.2164	26.8849	13.2183	11.8014	8.374	38.1698	23.6974	18.987	16.1945	17.5511	0.3142	0.358675
ENSG0000	TRNT1	3:3126915	20.8935	24.102	17.2699	27.7421	13.8111	27.368	18.3445	15.0559	28.6124	20.6325	0.8432	0.139246
ENSG0000	PHYH	10:132777	12.8297	8.4861	12.4858	17.6038	12.7395	20.8337	7.32658	12.3519	14.0692	8.23098	0.85345	-0.0926
ENSG0000	ATR	3:1424492	11.0883	11.4207	3.7821	3.78183	4.59191	8.26963	7.40694	7.21393	9.83875	10.9323	0.22905	0.616005
ENSG0000	VPS45	1:1500672	33.465	31.8288	18.2292	21.5378	14.3371	17.3585	26.8288	22.2615	14.6512	25.6995	0.93955	-0.02625
ENSG0000	PABPC4	1:3956081	12.3542	28.294	14.6881	8.30371	20.698	17.0481	9.97257	17.7166	6.4267	29.3025	0.84	0.083925
ENSG0000	HIST1H2A	6:2781435	2.52784	2.00321	1.98052	0.839285	1.71216	2.26578	2.8557	0.295768	0.084523	0.100625	0.3568	-1.54753
ENSG0000	RP11-127	16:480320	0.795669	1.24897	0.715669	0.332781	0.163225	2.29236	1.41945	1.04659	0	1.47225	0.5097	0.923813
ENSG0000	RP11-69E	1:3949164	4.39012	5.16377	3.035	7.17609	1.96333	6.01918	4.65738	5.66111	5.55626	3.50724	0.691	0.345529
ENSG0000	AP1S2	X:1582580	47.4951	52.4176	31.835	24.6027	26.3608	71.8535	46.4276	28.6692	22.324	10.333	0.9784	0.008917
ENSG0000	TUBB6	18:123076	9.47589	5.3024	4.52615	5.83636	1.94424	30.183	4.34811	3.45341	4.98639	2.76692	0.27695	1.18956

ENSG0000C	RP11-156I	17:469231	5.96189	6.52058	1.51007	1.34794	2.68295	5.35934	4.18479	2.14713	6.45488	1.34099	0.872	0.139465
ENSG0000C	ACLY	17:418669	41.1037	32.2214	14.8821	21.3869	13.9881	21.2583	28.5848	13.4491	8.62911	27.7631	0.3219	-0.34884
ENSG0000C	EIF4A1	17:756187	271.162	266.961	1046.19	852.869	1033.27	460.657	286.478	784.972	681.272	505.21	0.38355	-0.427
ENSG0000C	AC114730	2:2418443	0	0.167765	0	1.11723	0.096889	1.31259	0.770567	2.39381	0	0.396594	0.27885	1.75924
ENSG0000C	RP5-890E	17:480451	0.375764	0.481868	0.106865	0.420093	0.380079	1.09627	0.257721	0.404875	2.36453	0.47588	0.19105	1.44341
ENSG0000C	AC012360	2:1052643	0.681117	0.924745	0.489775	0.403397	0	0	0.896285	0.456439	0	0	0.597	-0.99589
ENSG0000C	CAT	11:344389	71.265	46.5209	52.2814	53.6253	44.0181	51.8107	25.6794	29.5381	36.7645	42.1625	0.0823	-0.54775
ENSG0000C	CDKL1	14:502375	3.12873	3.70072	4.65638	3.09677	3.38621	6.0896	15.4987	4.98533	8.54589	5.73016	0.1354	1.45601
ENSG0000C	GALNT3	2:1657475	12.6799	6.45273	6.59736	3.97554	5.50443	2.09674	11.2327	4.36066	2.88065	6.87179	0.42265	-0.37355
ENSG0000C	DND1P1	17:455858	2.84513	1.52629	1.03248	2.23625	3.3353	1.80683	3.24069	1.41655	2.13307	3.80953	0.53775	0.107097
ENSG0000C	TLDC1	16:844764	2.80902	10.6494	2.06895	2.59462	2.28872	8.78899	7.34801	2.18563	1.51096	7.2404	0.58505	0.293274
ENSG0000C	RP11-727	11:628068	0.210399	0.136297	0.132328	0.788216	0	2.10366	0.158283	0.755161	0	0.449129	0.44245	1.63735
ENSG0000C	KCTD10	12:109347	11.8819	13.7891	17.8038	7.03334	8.73559	11.7592	6.64976	13.0218	15.9575	12.7557	0.9452	-0.03564
ENSG0000C	DOCK4	7:1117261	0.047359	0.196779	0	0	0	2.3656	0	0.150204	0	0	0.1124	3.58126
ENSG0000C	TADA1	1:1668565	13.7128	11.473	17.2687	21.4919	10.0932	21.516	13.4434	5.52858	7.19774	8.17058	0.22825	-0.51108
ENSG0000C	IL21R	16:274021	10.0142	25.0763	10.3122	8.73867	18.1748	17.4901	53.5795	37.7367	8.34615	36.9158	0.03425	0.826217
ENSG0000C	ZKSCAN1	7:1000155	16.2582	14.8741	11.8824	12.9689	9.90813	8.35223	13.0074	9.30931	17.5068	26.4005	0.9913	-0.0069
ENSG0000C	PSMB10	16:678932	279.163	230.83	543.359	389.509	561.725	413.988	444.178	369.365	298.678	226.564	0.44965	-0.28648
ENSG0000C	MMS19	10:974583	25.1056	44.9807	12.5589	21.8814	12.3008	18.7551	13.9241	15.7577	29.1177	21.8601	0.85475	-0.073
ENSG0000C	BTBD6	14:105209	31.9432	19.3539	30.1821	21.292	32.1732	43.7095	41.9955	37.0887	40.7345	32.2468	0.3188	0.486372
ENSG0000C	RP11-527	17:439144	0.540405	0.804398	0.904768	0	0.4692	1.696	0	0	0	0	0.65505	-0.85554
ENSG0000C	9-Sep	17:772577	48.8376	77.761	211.78	103.777	534.65	53.9023	52.0655	253.749	94.9071	527.983	0.8785	-0.07792
ENSG0000C	RP13-188	X:5590812	1.31519	2.42194	1.65131	1.76323	1.53345	0.94963	7.27573	1.72209	0	1.64045	0.5507	0.328518
ENSG0000C	DDIT4L	4:9994808	0	0	0.038415	0	0.067275	0	2.91095	1.19225	1.77474	0.36597	0.1082	5.93894
ENSG0000C	SCUBE1	22:431972	0.019574	0	0.040427	0	0	0.94986	9.35859	2.95908	6.44135	8.15296	0.08855	8.95781
ENSG0000C	FAM161B	14:738518	4.02482	3.45613	1.01197	1.76529	1.802	2.18933	1.80754	2.5477	0.341878	2.15456	0.94925	-0.11257
ENSG0000C	RP11-565	17:925047	0.359311	0	0.793714	0.488674	0.084259	0.468573	0.175116	0.437945	0.85526	0.738197	0.77585	0.523087
ENSG0000C	PLCL2	3:1680265	25.3506	27.2673	7.17907	7.89038	8.70303	2.40774	10.4298	2.24969	3.23359	10.8302	0.00245	-1.32378
ENSG0000C	ST3GAL2	16:703759	6.88666	11.3895	2.84824	2.40822	4.50922	8.8449	6.64438	3.0656	0.334731	6.2259	0.50655	-0.3302
ENSG0000C	ZNF557	19:706944	3.11695	1.54161	3.07088	2.23893	2.87033	1.5385	3.70115	3.15942	1.73699	6.6881	0.86255	0.123028
ENSG0000C	RNF145	5:1591574	8.92066	6.79877	9.42952	12.8203	6.09582	8.08146	9.51174	13.7526	15.3876	17.8872	0.2868	0.45933
ENSG0000C	GIMAP1	7:1507165	54.391	56.7356	66.2175	36.9035	96.0953	31.8365	30.8551	35.3844	28.8773	57.7055	0.2552	-0.84324
ENSG0000C	CLPX	15:651482	14.0015	13.7666	9.13724	8.75194	8.79555	20.818	10.5813	7.24404	4.3804	10.4631	0.4552	-0.38951
ENSG0000C	MYL6B	12:561507	4.03248	6.43758	1.3529	1.31177	1.5932	12.1722	10.3194	3.39904	7.39809	3.57777	0.098	1.5248
ENSG0000C	RN7SKP56	6:1257811	0	2.31403	0.292921	0	0.152109	0.845965	1.77377	0	3.64308	0.603427	0.50815	1.47995
ENSG0000C	COX5B	2:9764603	229.67	160.104	272.008	254.522	106.58	329.676	248.775	234.765	188.201	32.643	0.92855	-0.03027
ENSG0000C	TMEM126	11:856285	72.6313	40.721	74.9835	78.9268	43.5061	83.3253	116.658	93.8714	125.612	30.2905	0.1325	0.599212
ENSG0000C	SERPINB9	6:2854656	0.994189	0.42501	1.34749	6.38677	2.96752	0	1.78412	1.30253	0	1.3812	0.241	-1.55117
ENSG0000C	B3GNTL1	17:827520	8.43372	13.8706	11.0056	14.3897	17.0149	23.1909	18.6643	10.9777	15.4488	14.8524	0.76235	0.302778
ENSG0000C	RPL7P23	5:7721044	1.95134	1.45916	3.98144	4.31103	1.01893	2.79993	1.08973	1.61609	2.16527	0	0.6959	-0.65863
ENSG0000C	C19orf73	19:491143	2.99771	1.0022	5.10623	3.17078	1.57215	0	0.273163	2.13893	4.4562	1.17302	0.8091	-0.58075
ENSG0000C	ZNF544	19:582285	10.1914	9.67927	11.1369	13.7298	6.82284	9.6729	8.94961	6.74487	5.02823	9.40875	0.3853	-0.47008
ENSG0000C	CBX1	17:480700	9.92964	9.72705	11.2321	10.9004	22.8144	12.4356	7.88276	13.0673	7.81963	14.2246	0.48235	-0.32042
ENSG0000C	RPL3	22:393128	1020.66	1048.93	2559.64	1512.18	2733.86	681.041	655.075	1299.39	1052.89	762.409	0.1062	-0.96577
ENSG0000C	LINC0088	3:1834476	7.83002	2.66434	8.30056	9.65099	5.85278	12.3004	3.34786	10.9844	10.4938	4.41464	0.7368	0.250226
ENSG0000C	ZNF425	7:1491027	1.6009	0.883636	0.55015	0	0.471477	0	1.55101	0.312943	0.724447	1.12623	0.856	0.280883
ENSG0000C	TRBV6-7	7:1424878	0.43809	0	1.26058	0	0	3.69639	0	0	0	0.27748	0.37705	1.20662
ENSG0000C	RP11-890	11:130866	0.624658	0.392317	0.527177	0.191989	0.880667	0	1.45049	0.800506	0.582119	1.942	0.3098	0.749415
ENSG0000C	GS1-124K	7:6620519	1.01751	0.744527	0.167305	0.861288	0.037401	1.63081	0	1.92573	0.472748	1.17084	0.5094	1.14139
ENSG0000C	STK32C	10:132207	2.55873	7.06746	3.092	6.1264	7.52292	5.51482	5.75872	4.95032	10.1726	10.6395	0.3623	0.623283
ENSG0000C	AC002116	19:360033	10.2638	16.8276	10.4845	12.6493	4.46473	15.9726	12.1445	8.10916	5.58244	7.56491	0.7784	-0.23537
ENSG0000C	CBWD4P	9:6528791	10.5902	6.83238	7.80428	8.73459	5.45776	13.1322	9.56639	6.44901	8.31604	6.53986	0.6153	0.982959
ENSG0000C	LYPD3	19:434607	0.858405	4.73437	1.73113	0	0.876551	0	0.826199	0	5.39853	0.956571	0.94465	0.104828
ENSG0000C	ZMYM5	13:198234	25.0681	32.7339	30.0456	28.2499	24.0622	27.4697	32.9102	25.9589	33.6096	39.0224	0.84985	0.085179
ENSG0000C	RAD9A	11:673178	4.57171	3.66256	2.10322	2.29718	5.02787	8.30588	6.87226	2.82287	1.54388	4.96621	0.64605	0.347299
ENSG0000C	COL6A1	21:459817	0.272112	1.47642	0.59016	0	0.669706	0	0	0	0	0.829869	0.3861	-1.48582
ENSG0000C	BUB1	2:1106376	4.39107	11.5728	0.294589	0.149177	0.446162	8.06103	5.49099	1.72323	8.88709	5.84786	0.19335	1.18824
ENSG0000C	CUL5	11:108008	13.3599	14.5772	13.5975	27.0301	20.8505	17.9542	16.6379	14.9544	6.93965	32.8115	0.5544	-0.38252
ENSG0000C	RIPK1	6:3063990	10.108	11.1762	3.81517	7.05347	5.81823	3.85161	7.84628	4.66085	5.7444	8.11039	0.3116	-0.41324
ENSG0000C	ISY1-RAB	3:1290875	2.15949	4.06752	0.182082	0.116727	1.92414	1.04287	2.40391	0.114704	0.01901	2.05998	0.52565	-1.25471
ENSG0000C	RP11-640	3:3682315	0.81837	1.72811	0.863809	0.185899	0.352221	1.74116	0.156744	0.469135	1.71889	1.45756	0.49945	0.47233
ENSG0000C	NFE2L1	17:480483	5.06676	3.65811	1.81099	1.33037	2.09735	6.34007	3.29255	1.62667	4.7387	5.10517	0.1465	0.682617
ENSG0000C	RP11-109	4:4270610	0.299874	0.398967	0	0	0.155702	0.107627	2.00072	0.440518	0	1.09673	0.25625	2.27717
ENSG0000C	LINC0039	13:308032	0.917712	1.13317	1.77586	1.15702	0.578216	0	2.64967	2.38312	1.55314	0.407506	0.8732	0.345045
ENSG0000C	KAT6B	10:748255	15.3092	14.5651	6.7749	7.87013	9.48289	11.4836	13.474	6.91835	15.4456	14.0111	0.78125	0.132714
ENSG0000C	MCM10	10:131615	2.24842	3.59717	0	0.407737	0.78254	4.02013	1.96837	1.0157	0	3.53865	0.82415	0.257446
ENSG0000C	XPO5	6:4350696	8.00886	8.4226	4.74658	5.56755	4.21971	7.84465	5.6491	5.85064	1.9154	5.64535	0.8185	-0.18114
ENSG0000C	CHAF1B	21:363197	0.870006	2.35628	1.04628	1.09045	0.971975	0.966477	0.984379	2.17228	0.010495	2.2851	0.9992	-0.00088
ENSG0000C	MRPL46	15:884594	57.1875	41.9241	62.4261	58.6286	63.7746	45.0551	68.2422	40.7686	51.234	22.5114	0.52685	-0.35208
ENSG0000C	CXXC5	5:1396472	76.0334	21.967	102.03	109.972	213.146	14.8039	16.1739	14.0478	13.8479	48.5524	0.00005	-2.43658
ENSG0000C	RBM43	2:1512479	5.11933	2.12061	2.76761	3.00089	1.46683	2.83664	3.04241	3.29138	0.790646	1.28474	0.67425	-0.44463
ENSG0000C	ECI2	6:4021266	45.0175	39.0276	71.22	47.5474	62.9499	43.8763	51.4477	66.5681	22.0249	30.3554	0.5676	-0.39058
ENSG0000C	PRMT3	11:203875	10.1449	9.9509	4.83968	5.66035	3.59628	6.29921	8.12338	9.49779	7.03715	5.88432	0.7116	0.184267
ENSG0000C	RPS6KA5	14:908478	14.1537	14.795	8.96595	10.5698	9.04079	6.8208	17.2227	8.69924	15.1455	11.8321	0.96	0.032952
ENSG0000C	ITGAX	16:313551	0.601771	5.97855	0.653165	1.61902	0.938912	5.66596	2.84033	0.877044	2.13125	0.619561	0.6498	0.307376
ENSG0000C	TIMP1	X:4756109	28.3048	41.5875	39.1337	67.5798	51.8389	115.236	23.5015	72.3501	148.547	41.7212	0.24565	0.834953
ENSG0000C	ACKR3	2:2365677	6.22047	4.7797	10.3018	12.9494	4.99076	6.46493	0.041764	1.22641	7.8589	3.15664	0.0599	-1.08475
ENSG0000C	LINC0063	X:1027691	2.3223	4.89452	2.49588	1.07374	0.887707	2.42221	4.91892	0.915942	0	3.28438	0.8746	0.179798
ENSG0000C	STT3B	3:3153263	73.1577	70.373	33.8634	41.6213	32.4017	56.482	37.2693	49.259	39.8548	67.9481	0.833	-0.08228
ENSG0000C	PTOV1-A	19:498182	1.12333	1.6853	1.04138	0.218999	1.40216	0.623914	2.61938	0.887973	0.359947	2.71462	0.70365	0.344042
ENSG0000C	FAM227B	15:491060	9.69383	7.71957	7.31096	7.5951	5.55391	0.7521	5.16195	6.49809	4.1865	2.44807	0.20985	-0.91037
ENSG0000C	RNASEH2	19:127630	21.0546	19.279	26.9246	19.5505	36.5901	22.6273	24.7047	19.5837	33.8673	24.8533	0.99725	-0.00364
ENSG0000C	GEMIN6	2:3875153	5.09658	4.97484	7.35119	5.4937	4.12546	7.78512	7.19829	5.13003	3.75149	2.24319	0.79055	-0.2143
ENSG0000C	NR1H3	11:472393	5.07291	2.67834	3.09916	4.11731	2.38791	64.0054	3.13589	4.349	8.10861	1.99443	0.0019	2.15064
ENSG0000C	PAPOLG	2:6075622	13.3222	8.11394	5.77548	4.77569	4.98391	5.0731	8.23491	6.10009	8.28124	7.44454	0.69435	-0.1567
ENSG0000C	CALU	7:1287392	19.9196	17.057	18.4057	14.6752	18.4098	21.4184	20.5047	12.5285	9.66401	22.2502	0.6991	-0.17258
ENSG0000C	C6orf25	6:3171859	0.072484	0	1.43695	8.53896	0.584966	0.163627	0	0	0.381942	0	0.03805	-4.39964
ENSG0000C	HSP90AB4	15:585875	0.092633	0.673333	0.154329	0.109574	0.258578	0	0	0	1.82553	0.656428	0.6015	1.07911
ENSG0000C	RIMKLB	12:868159	2.55659	8.42994	3.82931	3.52562	2.65956	6.04902	0	2.02287	4.26123	2.71101	0.4081	-0.58793
ENSG0000C	SMC4	3:1602256	42.4548	89.5322	37.269	32.6242	34.6287	116.624	133.79	57.0062	47.2677	84.4705	0.14585	0.717398
ENSG0000C	WDR48	3:3905199	23.9749	24.927	11.4447	12.9038	12.2824	18.8994	14.2202	12.3098	13.2879	20.9303	0.6125	-0.19097

ENSG0000	C6orf106	6:3458728	8.07942	8.86547	3.76693	2.64806	8.11704	2.9518	6.80664	3.70294	5.16646	8.82814	0.678	-0.18632
ENSG0000	GAPDHP1	X:3978713	0.191391	1.96986	0.462918	0.343204	0.131562	0.44442	1.14605	0.544736	0.462864	0	0.9079	-0.21173
ENSG0000	PLXDC1	17:390570	3.01899	50.8222	9.83325	4.52287	4.21637	6.02661	26.5071	21.4985	11.3925	14.1501	0.57765	0.233851
ENSG0000	AC006978	7:3028430	6.39091	11.7238	2.94935	3.21783	7.33424	3.74408	10.4254	5.57914	0	16.329	0.90265	0.135456
ENSG0000	TMEM256	17:738053	77.614	98.469	59.6336	55.3289	67.772	35.7264	45.9107	30.4511	48.9046	31.4915	0.033	-0.85264
ENSG0000	NUP98	11:366558	24.4087	27.5837	7.86537	7.91967	7.66146	14.3013	13.5483	16.3772	12.7404	16.9447	0.8006	0.102508
ENSG0000	TNFRSF25	1:6424787	253.281	190.883	268.46	298.813	227.85	300.688	128.859	369.028	287.062	321.689	0.7156	0.136144
ENSG0000	ZNF346	5:1770226	11.7799	16.7511	9.37685	10.5402	11.0572	7.35889	15.3692	7.49792	14.7865	10.2852	0.84525	-0.08068
ENSG0000	SPEG	2:2194348	0.973324	1.90427	0.42799	0.16772	0.301274	0.598968	0	0.402362	0	0.097049	0.09725	-1.40304
ENSG0000	CAST	5:9588509	41.6551	41.7536	25.8781	45.5958	85.6382	48.6449	56.1864	41.1171	54.4824	91.6596	0.75345	0.140098
ENSG0000	LINC0066	19:276845	3.85678	8.53738	3.73007	5.4038	3.26667	4.96793	8.12316	3.64575	8.19272	3.63869	0.67045	0.319466
ENSG0000	USP41	22:203388	1.36394	1.50502	1.84809	1.83854	2.39375	1.85597	14.3876	3.17692	2.05539	0.575243	0.37905	-1.85829
ENSG0000	SLC5A3	21:340732	0.901258	1.90125	0.703336	0.695113	0.894298	0.830963	3.07881	0.982565	1.11191	3.56076	0.5749	0.81414
ENSG0000	C10orf54	10:713969	117.304	140.411	142.983	143.377	153.052	77.1168	54.5938	83.7894	55.0675	81.3971	0.01435	-1.20126
ENSG0000	ATP6V1G1	9:1145877	293.67	213.729	284.8	339.066	192.137	307.426	260.117	195.628	290.211	57.8646	0.41455	-0.26195
ENSG0000	PHLDB2	3:1116746	2.27716	1.6695	1.51099	0.766884	2.01816	1.07333	2.18813	0.668002	1.24802	3.00952	0.91375	-0.08506
ENSG0000	JHDM1D-.	7:1401772	1.77982	3.95882	1.47865	1.05905	0.854613	3.87942	2.68366	1.53004	0	3.22075	0.7071	0.308104
ENSG0000	ANKMY2	7:1659977	14.0423	7.29963	7.29641	9.10683	8.33471	13.9538	15.5294	10.3653	9.03899	7.06722	0.47305	0.296293
ENSG0000	QSOX2	9:1362063	0.868072	0.903723	1.57094	1.12617	2.62817	1.65948	2.14035	2.47849	0.76253	3.50671	0.4657	0.410611
ENSG0000	SMG5	1:1562492	20.2354	20.7174	6.632	6.76319	6.74487	15.323	19.1366	13.2457	9.64767	15.7987	0.4535	0.26811
ENSG0000	ZNF625-Z	19:120922	0.123165	0.591498	1.34019	0.842393	0.871078	9.81E-05	1.61736	1.12037	0.086675	1.84106	0.35635	-1.93148
ENSG0000	OXR1	8:1063594	21.1955	20.5434	6.77984	12.5255	6.89393	15.8764	12.0553	6.34531	5.70747	10.0023	0.22905	-0.44541
ENSG0000	STK16	2:2192297	4.83957	6.02436	4.09175	19.6476	5.50103	5.70807	1.47927	7.36161	5.14135	4.59522	0.297	-0.82726
ENSG0000	RNVU1-4	1:1209131	10.7898	0	1.51675	14.9212	3.11484	0	0	0	0	0	0.1001	#NAME?
ENSG0000	RAB33A	X:1301716	12.5004	22.9424	17.2939	9.92698	15.9381	42.9048	39.6323	46.282	7.58247	13.7316	0.0794	0.819856
ENSG0000	NSMF	9:1374475	11.4727	11.6464	4.14475	2.26363	6.08926	3.51261	9.87507	7.77622	4.04663	11.155	0.88395	-0.0635
ENSG0000	C17orf49	17:685386	91.2908	86.6373	86.3668	96.7079	87.1018	198.85	156.125	106.632	145.546	39.0525	0.4295	0.605254
ENSG0000	B3GNT5	3:1831780	0.415284	0.262436	0	0.680555	0.128154	3.99334	0.800259	1.12611	0.022621	1.17318	0.04035	1.55776
ENSG0000	LIMK1	7:7408293	2.9171	1.89102	3.4207	4.37579	7.0136	2.70439	4.43703	10.3265	4.20625	24.7738	0.03095	1.15348
ENSG0000	RABL6	9:1367913	9.90068	9.80558	5.01644	6.2323	11.5724	7.7048	10.4483	7.40775	4.17723	14.927	0.8233	0.139994
ENSG0000	FBLN5	14:918694	10.3796	13.4646	9.3214	14.2075	8.41408	7.24473	2.23148	5.93907	15.9473	3.57194	0.18075	-0.63396
ENSG0000	CALM2P2	10:701472	1.18236	1.55384	2.44072	1.77728	1.34487	1.5782	2.3371	1.77674	0.715358	2.41002	0.8883	0.186326
ENSG0000	TMEM200	6:1303657	0.664227	0.468305	0.524836	1.3338	0.335715	6.21248	6.31624	4.22552	3.76945	3.70611	0.00695	2.90433
ENSG0000	LGALS2	22:375702	5.00118	5.72168	1.73282	0	2.27726	39.7101	1.54965	0.974555	4.62745	2.04688	0.2381	1.6818
ENSG0000	MRPS35	12:277107	40.6012	44.608	37.1231	45.269	29.5316	36.2063	37.156	26.9179	27.3305	14.2218	0.1388	-0.4919
ENSG0000	SZT2	1:4338988	5.06322	7.03644	1.98488	2.95028	2.98591	3.02086	4.81545	1.38011	2.03909	5.85525	0.7027	-0.24493
ENSG0000	NAPA-AS	19:474842	2.89834	0.209332	1.3465	4.00284	1.03406	1.63129	1.70533	0.634673	0	0.341079	0.26495	-1.4043
ENSG0000	POLR1D	13:276207	82.2058	50.3843	70.2674	59.9652	38.7609	83.1452	60.1604	73.3369	64.5062	32.0175	0.89175	0.05661
ENSG0000	FADS2	11:616804	0.769566	2.57389	0.498438	0	0.260075	2.19214	0.54019	3.93456	1.54793	0.364726	0.3175	1.22263
ENSG0000	FRG1BP	20:303773	5.48364	3.37912	3.53311	2.68684	5.99085	5.83268	1.29985	2.02746	1.9746	3.38624	0.5636	-0.58752
ENSG0000	IGLV2-23	22:226977	0	43.357	0.150473	0	0.297217	0	0	0.541504	0	0	0.26205	-5.89459
ENSG0000	ROM1	11:626022	1.48617	1.34467	0.943534	2.43987	0.727305	0.854875	3.19848	3.41255	2.23801	1.3822	0.6086	0.614072
ENSG0000	IL9R	X:1559975	0.135139	1.38938	0.011504	0.216089	0.011737	3.19374	4.91946	8.19423	2.45688	2.67266	0.0013	4.12995
ENSG0000	PTTG2	4:3789108	0.711564	1.15443	0.235569	0.100815	1.1094	4.76637	3.99979	1.50445	0	1.83953	0.2702	1.79246
ENSG0000	MRPL58	17:750126	35.7538	26.8968	50.3635	41.1982	43.7874	43.0831	28.0796	35.5517	30.9014	9.97912	0.30185	-0.47843
ENSG0000	PDCD6	5:271620-	124.382	111.454	183.428	176.308	136.023	174.967	114.944	140.456	120.155	50.046	0.3509	-0.32327
ENSG0000	ADNP	20:508889	7.10882	6.6183	7.58138	5.34174	7.81366	6.37178	5.33368	5.99954	4.23519	14.7001	0.8267	-0.13701
ENSG0000	RP11-810	11:619677	2.1999	1.9452	3.13597	3.75781	0.999057	0.194897	1.10895	1.40382	0.306552	1.61675	0.37245	-1.41114
ENSG0000	ZNF701	19:525276	5.11125	7.69744	6.23901	6.00901	3.47913	3.01039	4.97915	4.60769	4.54926	5.97323	0.31745	-0.5451
ENSG0000	RP11-471	17:119983	1.56791	0	0	0.70397	0	1.30888	0	0	1.53896	0.64543	0.55295	1.16497
ENSG0000	GUSB	7:6596068	58.9366	68.2417	54.9557	79.7392	50.3745	77.0005	79.1496	65.5661	57.0577	61.0006	0.7631	0.095007
ENSG0000	SPTLC3	20:130089	2.56034	2.12685	1.61755	0.613833	0.476562	0	0	0	0	1.10801	0.2616	-3.46917
ENSG0000	ITM2B	13:482331	418.039	482.508	398.026	274.89	325.864	544.145	337.922	364.355	378.764	203.655	0.78255	-0.24503
ENSG0000	EGLN1	1:2313637	18.4287	14.275	7.5958	8.87774	7.44947	21.6734	7.032	7.14208	7.36191	8.09385	0.98685	-0.0061
ENSG0000	SETP17	11:862947	0.406028	0	0	0	0	3.16075	0	0	0	0.251979	0.0941	3.23794
ENSG0000	HIST1H2A	6:2583306	3.39342	0	1.28699	3.29872	1.06312	1.34563	0	0	0.950249	0.180323	0.2648	-2.27018
ENSG0000	CSNK1G3	5:1235120	18.2816	9.40113	7.75571	12.1732	7.47993	8.98035	11.1561	12.259	1.23001	9.42339	0.37035	-0.53664
ENSG0000	RP5-1198	1:4399135	0	0	0.37297	0	0	1.64502	0	0.989562	0	0	0.00345	2.71311
ENSG0000	KANSL2	12:486534	30.0441	30.7679	46.6282	47.5216	30.9668	32.399	50.1305	45.4741	39.9558	41.7949	0.7293	0.120149
ENSG0000	SPICE1	3:1134427	4.55552	7.09272	1.49443	3.29642	1.30013	2.59363	1.32971	1.15106	1.36949	2.70343	0.0403	-1.15027
ENSG0000	TMEM144	4:1581244	0.148494	0	0	0	0	2.96607	0	0	0	0	0.12245	4.34614
ENSG0000	POT1-AS1	7:1248223	0.141277	0.133233	0.199139	0.098382	0.010446	0	0	0.449515	1.45966	0.023211	0.28215	2.05126
ENSG0000	DUSP22	6:291629-	12.7904	21.1687	13.8684	9.42138	8.79609	9.29941	32.0579	18.1649	11.9615	15.0928	0.4514	0.338939
ENSG0000	PIN4P1	15:438707	0.565085	0.818272	0.264356	0	0.274235	0.156009	0.142486	0	0	0.685154	0.86845	-0.32383
ENSG0000	TUBB4A	19:649431	5.90798	2.48846	14.0314	5.34939	13.2321	1.39119	0	4.09328	5.05797	3.8464	0.06505	-1.41848
ENSG0000	CCNB1	5:6916700	17.973	26.4116	2.76249	4.00311	2.40686	15.7243	22.638	3.52209	6.78947	5.0831	0.89315	0.076293
ENSG0000	NUP88	17:528226	68.8895	46.5946	40.1107	54.1237	36.8948	33.9304	25.9503	29.9752	21.7239	41.6288	0.20365	-0.75052
ENSG0000	FBXW4P1	22:231797	0.266211	0.418792	0.98135	0.521464	0.731192	0	0.07271	0	0	0.750084	0.4749	-1.94164
ENSG0000	RSPH3	6:1589728	3.40061	3.50077	1.64687	2.00866	0.770102	1.72011	2.99296	0.037881	4.23108	1.28281	0.8102	-0.25334
ENSG0000	TMEM126	11:856479	42.6178	20.8846	61.0649	38.8508	30.0226	58.175	57.1469	49.4142	26.7498	9.21536	0.94455	0.030278
ENSG0000	GSTO1	10:104235	95.7045	78.4405	103.512	105.486	82.11	266.674	181.183	143.711	104.51	22.8535	0.11505	0.545673
ENSG0000	ZBTB48	1:6580000	19.5199	20.3741	18.59	19.3768	15.0372	13.3746	18.5087	13.3781	14.3017	13.9172	0.80305	-0.11021
ENSG0000	UBL7-AS1	15:744612	6.72827	8.31772	6.34682	11.8897	4.5632	8.1922	7.29487	5.46131	12.1352	5.63075	0.9335	-0.05519
ENSG0000	NME1-NM	17:511535	14.629	5.57451	20.1644	4.60711	14.86	9.69503	11.2822	9.18941	0	2.96067	0.6233	-0.96729
ENSG0000	RBM3	X:4856801	68.3518	83.9514	151.739	184.629	103.473	42.9381	148.44	160.733	214.258	71.0598	0.75315	0.108282
ENSG0000	SPNS3	17:443368	4.69172	4.42114	9.31655	7.63611	4.64105	4.46554	17.4227	10.2079	14.264	3.7294	0.42645	0.674845
ENSG0000	CTTN	11:702706	0	0.128893	0.45702	0.55945	0.041087	0.030975	2.43097	0.349731	0	0.351462	0.19265	1.46859
ENSG0000	CTNS	17:363646	5.79885	7.77191	3.87085	2.7877	3.86912	13.0596	2.13417	3.21739	8.17158	3.75691	0.4045	0.420485
ENSG0000	WDR35	2:1991025	1.82341	0.508374	0.844614	0.456993	0.47646	2.16005	0.840845	0.404079	0.425804	0.846232	0.76855	-0.21423
ENSG0000	RP11-875	8:2268765	3.37739	3.62928	4.94652	6.30033	7.36761	3.55031	2.08985	5.41354	9.83092	6.01612	0.93235	0.134837
ENSG0000	PARP2	14:203430	18.8156	16.2036	16.0177	15.1106	10.4941	18.0594	15.7353	10.2452	22.4739	9.09339	0.97685	0.01122
ENSG0000	SGF29	16:285537	37.0695	59.9783	61.1929	49.927	58.0898	42.4642	61.4935	63.9104	57.3627	22.9386	0.7132	-0.13272
ENSG0000	SEC22C	3:4254796	25.2213	19.1041	27.2587	27.5526	16.9008	12.4864	24.9624	22.1719	59.3668	16.7958	0.79585	0.261642
ENSG0000	RP11-43D	12:104455	1.22791	2.79373	0	0	0	6.38053	3.79291	0.291972	3.6251	0.753314	0.32875	2.11592
ENSG0000	COX19	7:897900-	16.2777	19.1588	17.8356	17.7648	23.0555	14.1614	21.1268	25.0572	11.8472	26.2479	0.91065	-0.05327
ENSG0000	RP11-298.	10:124917	2.18259	1.43573	0	0	1.11474	1.30655	0	0.302348	0	0.682156	0.56425	-1.22432
ENSG0000	KIAA0430	16:153957	9.99171	13.7423	6.70302	5.26862	9.38886	8.58093	10.7163	7.52061	4.74261	18.8462	0.83045	0.10793
ENSG0000	EXOC7	17:760387	40.5527	47.544	19.0566	21.9149	19.067	30.2577	24.3273	17.1838	20.1097	36.858	0.4711	-0.35958
ENSG0000	RP11-426	16:290537	1.36118	1.38045	0.306007	1.88952	1.09921	4.7191	3.89756	1.24072	0	2.37899	0.5178	0.979578
ENSG0000	RP11-701	4:1847555	0	0	0	0	0	0	0	0	2.41758	0.088287	0.17355	inf
ENSG0000	LINC0023	14:101634	15.96	3.01272	1.8814	6.34999	3.16778	24.0825	4.63709	9.35801	7.84374	2.62854	0.38675	0.685114

ENSG0000	MKL2	16:140713	3.14733	6.87135	2.05333	1.58987	3.79622	2.00908	3.35821	2.66669	0.240482	1.74095	0.33035	-0.88587
ENSG0000	CEP104	1:3812080	13.7204	15.5792	19.7744	15.5746	19.5644	21.3464	8.29822	9.80169	9.87497	30.4183	0.3408	-0.41094
ENSG0000	JMJD4	1:2277285	13.7438	13.7538	13.4092	19.3171	15.2804	14.7628	10.254	11.7081	14.9452	12.3998	0.804	-0.12089
ENSG0000	CTD-2325	19:800872	0.278497	0.090176	0.945713	1.60119	0.106643	0	0	1.35759	0	0	0.50695	-0.99581
ENSG0000	AC067945	2:1910215	1.75611	0.600032	0.284939	0.638204	0.392111	2.55551	2.33742	1.90897	6.79016	0.580826	0.08115	2.22099
ENSG0000	UBAP2	9:3392169	7.87894	14.6814	3.90002	3.84064	5.12052	5.90088	6.59025	7.44516	5.82259	9.95166	0.82945	0.098778
ENSG0000	RP11-351	12:422384	0.756267	0	0.539931	3.38014	0	0.23734	0.106437	0.20742	0	0.973771	0.5985	-1.11017
ENSG0000	RP11-143	17:731649	1.70653	2.30314	0.763854	0	0.513991	0.604614	2.43194	0	2.23425	0.463711	0.8628	0.26014
ENSG0000	ZNF138	7:6479438	11.2003	11.8595	4.99271	5.5454	3.67931	7.88942	4.73648	1.96549	1.72971	4.68318	0.17225	-0.85218
ENSG0000	WNT11	11:761863	1.73599	0	3.97224	19.3305	2.58259	0	0	3.73312	11.7047	1.6138	0.69225	-0.54453
ENSG0000	GS1-124K	7:6649104	3.12085	1.12121	1.24836	2.17346	1.80697	1.69754	1.10208	3.17106	1.36081	3.41639	0.8491	0.240134
ENSG0000	KMT2C	7:1521349	10.654	11.3779	3.02709	2.24939	7.46151	10.3275	12.9266	4.03593	4.64839	17.4885	0.7089	0.184601
ENSG0000	GPSM2	1:1088158	2.17763	3.17592	0.761232	0.548477	0.12255	18.768	5.68585	0.586714	5.36979	1.76432	0.0432	2.1295
ENSG0000	MTATP6P1	1:585988-	110.182	106.551	214.032	277.902	353.024	189.66	103.653	247.749	381.28	215.749	0.84825	0.121044
ENSG0000	TIGD6	5:1499607	1.25112	3.8878	1.14506	0.926711	1.35812	2.40759	0.779743	3.43593	2.22665	1.14025	0.7637	0.27298
ENSG0000	RAD18	3:8619399	5.79228	8.40196	2.22747	2.47237	3.44502	6.4582	5.29659	5.90859	5.42051	5.35464	0.76225	0.166394
ENSG0000	PSPN	19:637243	0.22571	0.295816	0.364782	0.02947	0.422714	0.546083	0.566658	0.583599	0.282059	0.495582	0.3365	1.25312
ENSG0000	HERC6	4:8837873	13.4045	7.88919	9.36991	11.4853	3.39542	4.27232	27.8109	17.1576	10.0817	7.13324	0.0865	0.664303
ENSG0000	COBLL1	2:1646536	0	1.38124	0.03059	0	0.77593	0.006893	0	0	0	0.055634	0.07335	-5.80599
ENSG0000	TRDV2	14:222025	577.436	14.8796	6.40209	7.95177	6.19154	170.577	7.41289	2.8274	0	3.03963	0.5568	-1.67713
ENSG0000	DICER1-A	14:950862	3.19016	5.78107	6.11255	6.11548	1.60582	6.62647	1.27583	3.98324	0.230489	3.00297	0.58215	-0.61549
ENSG0000	RAP2A	13:974342	3.89141	2.81598	1.59192	0.984445	2.9291	2.88364	4.23473	2.80032	2.66291	3.97076	0.361	0.41534
ENSG0000	RPN1	3:1286199	143.322	86.148	159.879	132.239	148.128	148.056	151.109	165.475	111.51	117.23	0.98145	-0.00841
ENSG0000	KIF1BP	10:689887	6.87578	7.41142	7.28724	8.51703	6.16526	3.37445	8.35063	6.66601	3.47465	7.54558	0.6069	-0.23749
ENSG0000	RP11-133	5:1774833	0.606258	0.770716	0.124895	0.156046	0	2.33096	0	0	0.329443	0	0.70015	0.685572
ENSG0000	TBC1D15	12:718397	29.8278	29.0832	22.3634	25.55	20.2872	30.7345	20.828	22.689	24.15	23.0232	0.81605	-0.07592
ENSG0000	PNPT1	2:5563426	18.0817	17.6524	7.98647	13.4806	5.14889	17.0153	26.5582	17.8281	10.4718	8.29615	0.2402	0.417168
ENSG0000	WDR86-A	7:1513416	22.667	3.86326	52.7088	46.7028	16.2538	5.74035	20.0967	36.2326	56.536	6.21084	0.8996	-0.16451
ENSG0000	RP11-440	2:9552510	1.60487	0.114273	2.19471	0	0.904024	3.05174	2.96495	4.07351	4.6996	1.45513	0.32255	1.76501
ENSG0000	CNOT6L	4:7771338	13.3649	11.8386	12.487	6.4946	22.7564	11.3159	19.2946	22.8828	11.7848	33.5812	0.63945	0.242451
ENSG0000	NAA35	9:8594114	10.1841	13.0747	7.4764	6.69627	6.31368	7.96272	8.41674	7.82805	8.44189	12.6004	0.93015	-0.03529
ENSG0000	CYFIP2	5:1572601	176.276	183.017	88.7458	115.945	114.602	71.4048	87.8373	82.6646	59.9777	107.008	0.30915	-0.46907
ENSG0000	CYP2R1	11:148774	16.3476	12.9059	13.7481	10.7588	13.966	22.7973	23.4042	21.0449	7.14066	11.9487	0.60875	0.240178
ENSG0000	SCOC	4:1402572	43.5158	34.7433	30.6973	31.3215	17.861	15.0451	23.5019	17.4959	12.4051	7.87092	0.0252	-0.9415
ENSG0000	THAP2	12:716094	9.85784	10.7067	8.09848	9.52544	10.4122	13.9858	10.9868	5.77223	2.40484	6.35858	0.7549	-0.3775
ENSG0000	RP11-380	13:260947	1.30603	1.13619	1.48346	1.42699	0	1.70207	0.722691	0.707132	0	0.514863	0.65925	-0.64478
ENSG0000	SAMD8	10:750944	3.11548	4.96927	2.73373	2.09998	1.43939	4.95371	2.44977	2.28416	5.38694	3.45278	0.81725	0.147338
ENSG0000	RPL23AP5	10:102503	0.138285	1.6616	0	0	0	0.600846	0	0	1.80514	0	0.8198	0.724789
ENSG0000	MKKS	20:104010	21.718	18.5028	16.2748	10.9744	9.03266	29.6332	21.0933	12.0003	10.2582	8.30601	0.7883	0.12795
ENSG0000	FAM174A	5:1005353	12.6365	7.35881	18.4165	22.8689	14.4532	12.4043	13.3237	10.7576	14.3308	4.60475	0.2586	-0.53517
ENSG0000	MANEA	6:9557754	5.33376	4.11113	1.41766	2.5866	1.45226	6.31186	3.30177	3.43834	7.55781	3.15681	0.11325	0.772112
ENSG0000	PRSS30P	16:283016	0.750967	0.759708	0.695605	0.260313	5.91551	1.99977	2.01031	0.4381	0	1.03023	0.37735	-1.04582
ENSG0000	TCAIM	3:4433794	10.025	10.2066	6.40462	7.52648	4.40589	12.4913	10.3184	7.80841	7.54547	6.09458	0.78345	0.136723
ENSG0000	CNDP2	18:744958	92.9072	82.9442	94.6486	102.73	84.3486	67.4752	74.1411	90.9132	75.1475	87.023	0.44275	-0.22997
ENSG0000	RP11-544	1:1493456	0.833942	1.27117	2.13843	0.391124	1.2817	1.24684	0.380541	0.653708	0.61537	0.592943	0.713	-0.72215
ENSG0000	CISD3	17:387302	76.3919	34.5331	108.039	116.102	89.3514	80.0551	58.2711	77.0461	44.5897	25.5028	0.0919	-0.73223
ENSG0000	TNNI2	11:183898	0	0.60773	0.29009	0.470266	0.073077	4.05157	1.49395	0.482158	0	0.397835	0.0812	2.31412
ENSG0000	CLN5	13:769906	5.46649	19.018	2.12141	1.95865	1.29361	23.7593	21.0132	12.3464	9.65571	6.25286	0.09175	1.29245
ENSG0000	CD93	20:230793	0.787649	2.3499	0.163377	0.073591	0.194196	0	0.345915	0.367242	0.031574	0.444936	0.33705	-1.59103
ENSG0000	COL18A1	21:454051	4.69106	15.9411	0.032152	0.229593	0.205544	1.84464	4.51584	1.27826	0.378357	1.16789	0.44105	-0.96178
ENSG0000	RP11-837.	12:109900	8.24025	3.62834	1.35687	4.32631	0.351874	0	2.99934	1.11152	0.555035	1.9235	0.4951	-0.97401
ENSG0000	AC002116	19:360145	0.515719	0.339443	0.67549	0.373837	0.558183	0.159095	1.90047	0.162002	0	0	0.76465	-0.48978
ENSG0000	ZNF641	12:483371	9.41014	9.2553	3.25905	4.26747	6.82929	5.70233	6.99149	4.83023	2.27933	13.864	0.60275	-0.25994
ENSG0000	RP11-223	4:1420231	0.468108	2.1482	1.27942	0	0.40309	1.62286	2.22013	1.2003	0	0.543149	0.83345	0.333737
ENSG0000	LINC0147	2:1860328	0.979293	3.42488	0.466325	0.144444	0.332018	0	0.209087	0	2.52898	0.538751	0.70495	-0.48637
ENSG0000	STAM-AS	10:176412	0.172876	0.520472	0.222616	0.662758	0.186286	2.59518	0.230282	1.09096	0.807873	0.684062	0.3285	1.5532
ENSG0000	DNAJC25	9:1116313	4.3961	4.71214	3.33749	3.09679	3.04278	5.06248	5.70823	4.66714	1.72598	2.23794	0.8851	0.158453
ENSG0000	ACTR3C	7:1502439	3.12623	4.28125	2.8128	5.03279	2.51223	8.67186	6.30863	4.1476	6.1438	1.64024	0.4764	0.704706
ENSG0000	RP11-138.	7:1309302	3.19475	6.50072	0.804625	1.43596	0.794801	5.74231	9.00012	2.38479	11.8683	3.74072	0.0456	1.50104
ENSG0000	TAPT1-AS	4:1616050	0.81605	4.70052	0.98919	0.144476	0.673431	0.231446	0.594765	0.238253	1.12378	1.15252	0.3677	-1.10428
ENSG0000	CMB9-22	11:654552	1.62786	0.800071	1.29616	0.7687	1.66946	5.5874	4.1435	4.4537	1.06919	1.7356	0.1866	1.48621
ENSG0000	TRMT11	6:1259864	17.6834	11.4636	12.0495	14.2431	13.156	17.1538	17.908	8.87785	11.5153	12.4363	0.8915	-0.05042
ENSG0000	TKFC	11:612994	15.076	24.2122	17.2709	11.9133	11.9485	4.71489	32.4288	15.2936	17.5853	13.1953	0.9439	-0.04721
ENSG0000	TRAV35	14:222025	6.18858	14.5532	1.39838	0.364858	0.292398	13.6639	17.3174	0.312616	0	0.177447	0.82505	0.457065
ENSG0000	ELF1	13:409319	80.2396	70.9356	70.5317	61.0172	84.8603	55.7394	104.63	79.0482	70.5236	81.7152	0.8716	0.05421
ENSG0000	IFT122	3:1294309	3.6937	1.9767	2.31772	2.19241	1.60882	4.80166	3.88107	2.97707	0	2.54352	0.70725	0.253345
ENSG0000	SGK3	8:6666759	7.95422	4.07188	10.0871	11.9329	6.8113	8.5728	5.41229	6.30312	11.0868	9.58926	0.83915	0.089381
ENSG0000	RAB11B-A	19:836328	2.76763	1.09732	4.87289	1.61392	3.4136	0.989041	1.84256	2.99385	1.64167	0.663797	0.5428	-0.78882
ENSG0000	ZNF619	3:4047711	1.79947	2.82456	1.00776	1.01152	1.18701	0	1.16035	1.04714	1.26282	2.3614	0.66245	-0.36148
ENSG0000	RP11-422	1:1539590	0	0	0.388559	0	0.134484	0	2.06333	0	0	1.2034	0.4119	2.49833
ENSG0000	CTD-2017	14:101761	0.400741	1.04275	0.472967	0.309018	0.306545	0.902918	0.751639	0.508336	0.214304	0	0.91865	-0.15178
ENSG0000	FERMT3	11:642066	232.082	209.988	189.881	241.733	179.132	158.291	160.079	143.358	182.56	189.13	0.50105	-0.24795
ENSG0000	EEF1DP2	9:9280938	1.16832	0.710112	0.894439	1.05191	1.18473	3.243	0.226087	0.836344	0	3.61775	0.77315	0.509869
ENSG0000	RP11-274	7:1286670	0.266977	1.86396	0.019438	0	0.349789	1.11434	0.707574	0.186114	0.101264	0.630523	0.90185	0.216186
ENSG0000	HMGB1P3	2:5386406	0.612665	0	0.112152	1.33744	0.831467	0.402729	0.359464	0.385279	0	0.590375	0.69645	-0.59643
ENSG0000	SEMA7A	15:744092	0.700835	0.535387	0.132216	0	1.39045	0.753597	2.02185	1.2875	0.079715	2.7719	0.3146	1.03934
ENSG0000	IDNK	9:8362304	11.1252	9.47382	19.3273	19.9539	11.8679	18.7282	32.9321	10.9391	15.8244	6.61849	0.72895	0.203192
ENSG0000	COPS7A	12:672374	37.1287	33.819	49.5751	55.5219	33.5412	42.8092	34.2814	42.7123	27.3199	38.7372	0.55005	-0.20866
ENSG0000	AC114271	19:103048	0.487174	0.638655	0.418283	0.254116	0.595547	0.589098	0.648797	0.058811	0.148387	0.652636	0.7969	-0.30395
ENSG0000	HCLS1	3:1216313	336.779	335.388	290.306	245.917	293.437	332.376	299.047	270.542	217.153	342.886	0.9262	-0.03057
ENSG0000	PIGV	1:2678747	18.9417	9.94411	21.0817	12.9239	18.3141	2.64711	7.16212	7.76709	19.5318	13.0375	0.07755	-0.73215
ENSG0000	ATRIP	3:4844670	8.11981	3.72847	6.06122	10.9558	5.83386	4.17793	4.20497	3.75608	6.81225	5.02592	0.9976	0.004406
ENSG0000	FADS3	11:618735	3.58405	4.21182	6.70775	2.71285	4.42211	4.83871	8.73644	7.073	6.41113	4.83746	0.5121	0.444214
ENSG0000	DGKQ	4:958886-	32.136	18.0203	4.51309	3.79619	5.36169	7.66638	15.2785	7.94496	2.74294	8.57796	0.2313	-0.66513
ENSG0000	CDK4	12:577237	46.8883	34.6159	49.1872	41.766	60.8939	69.4563	49.7997	43.8166	34.8994	41.2014	0.87015	-0.08302
ENSG0000	CCDC25	8:2773331	15.3929	16.432	18.5945	16.6969	20.5661	9.20773	14.9442	11.0029	17.0622	17.4912	0.51455	-0.35002
ENSG0000	LINC0109	4:7864590	0	0	0	0	0	3.39765	0	0.594043	0	0	0.0436	inf
ENSG0000	S100A5	1:1535371	1.00806	0	0.695633	0.993599	0.600075	0	0.495108	0	0	0.182557	0.32215	-2.26216
ENSG0000	RECQL	12:214376	68.9915	57.9442	18.2855	31.7242	21.8706	59.3074	37.9729	24.9694	19.813	31.5518	0.47595	-0.27402
ENSG0000	RP11-521	14:105398	0.236436	0.022797	0	0.275781	0.088635	1.75058	1.08864	0	0	0.414147	0.2446	2.06953
ENSG0000	NDE1	16:156432	22.2686	32.1619	11.8496	15.8718	18.5333	15.8486	20.9513	11.1439	14.5429	31.1781	0.6111	-0.21389

ENSG0000	RPS6KA1	1:2652976	79.9673	80.3942	50.7595	50.4935	60.8342	63.6989	48.3945	40.4341	59.5139	56.6721	0.36535	-0.29896
ENSG0000	NOD1	7:3042452	10.7311	10.8117	5.43479	10.7025	5.17238	7.16475	9.4305	2.43157	6.40742	8.60694	0.3012	-0.47479
ENSG0000	SNHG19	16:215479	2.9937	2.11546	4.07612	1.02424	1.32197	4.97491	1.38598	2.07876	2.82351	0.926613	0.9657	0.068539
ENSG0000	ZCCHC18	X:1040992	5.63449	5.08306	9.54192	10.4303	5.30081	12.5082	6.14087	15.7888	8.34543	8.93014	0.53975	0.486559
ENSG0000	TAF1D	11:936616	33.0084	36.6525	26.612	35.5582	26.8638	30.6843	34.3235	39.1269	15.6825	47.9384	0.8927	0.063268
ENSG0000	MRPL1	4:7786251	38.4592	25.5297	23.5219	28.7224	30.3387	27.3404	34.6882	32.8672	25.2817	7.9012	0.78405	-0.11252
ENSG0000	UBB	17:163807	950.557	728.884	2022.51	2226.3	1645.64	1446.39	1126.85	1468.87	1763.95	484.78	0.59795	-0.30291
ENSG0000	ELMO2	20:463645	72.5494	49.7123	35.6695	45.0607	29.2564	34.6412	40.4755	23.9433	22.5544	44.5938	0.31275	-0.33673
ENSG0000	PRKCB	16:238360	61.3348	100.332	40.838	18.8333	31.4224	34.1782	35.2909	21.1451	22.0639	56.0867	0.06165	-0.71552
ENSG0000	HNRNPA1	12:679894	0.267423	0.873981	1.2623	0.409675	0.449709	0	0	0	0	0.331691	0.317	-3.32065
ENSG0000	ITFG1	16:471443	45.3515	46.9781	26.9324	30.1772	19.8635	50.9976	46.8592	22.1969	22.1266	27.5155	0.8779	-0.06992
ENSG0000	SMIM24	19:347398	0	0	1.16766	2.43598	0.059903	0	2.48316	0	0	0	0.6115	-0.87255
ENSG0000	RARA	17:403091	9.77577	9.54791	9.11345	17.035	18.4356	10.729	10.2447	12.798	14.8612	25.8751	0.7786	0.127877
ENSG0000	RP4-756H	7:6655380	17.5098	12.1991	19.6417	21.2882	11.3442	10.1307	11.9928	11.9242	27.5051	4.13475	0.75645	-0.24852
ENSG0000	TNFRSF14	1:2549919	71.1843	97.708	71.9393	98.1896	78.3382	77.6866	71.5713	95.4309	127.028	76.0851	0.68	0.135198
ENSG0000	TRBV12-2	7:1424408	0	0.133584	0	2.6508	0.154819	0	0	0.164393	1.72186	0	0.74215	-0.43141
ENSG0000	ADORA2A	22:242708	45.1561	33.9977	69.3591	67.4078	55.8092	38.4979	81.1643	63.77	37.68	68.869	0.94275	-0.02621
ENSG0000	ATXN7L2	1:1094834	7.61625	9.94758	9.56504	5.50284	7.47733	1.14091	5.30551	2.83402	4.05713	6.1021	0.1598	-1.04521
ENSG0000	TRAF3	14:102777	13.7773	8.33273	4.25328	13.7511	9.29083	20.8043	17.5084	19.7633	16.7399	21.4986	0.17785	0.655179
ENSG0000	ZNF81	X:4783690	1.55243	1.95046	1.05344	2.21589	1.37974	0	0.188679	0.439298	0	1.64411	0.0885	-2.74302
ENSG0000	AXIN1	16:287439	4.75479	3.54686	9.95018	8.81556	14.8881	2.57305	2.88477	5.03189	6.31101	10.2771	0.17825	-0.63774
ENSG0000	KPNA3	13:496993	14.0519	14.0885	6.30337	7.25423	13.0188	10.8238	7.15551	5.79592	11.4135	16.1739	0.5336	-0.21785
ENSG0000	ING1	13:110641	21.1194	19.3833	11.318	15.3086	17.2083	10.5883	14.9006	16.8446	15.5743	8.24868	0.64405	-0.48115
ENSG0000	GCDH	19:128910	10.8854	15.8136	39.9901	40.8652	35.2398	6.77128	22.3802	30.6118	40.848	23.278	0.62155	-0.20926
ENSG0000	CITF22-1A	22:499002	0.790388	1.26854	1.16866	0.358614	0.913015	1.21503	1.44989	0.996328	0.845397	3.91304	0.85805	0.31524
ENSG0000	SIGLEC17F	19:511422	1.64855	1.00398	5.16473	2.28392	5.75883	2.02787	1.10365	1.2498	0	0	0.2088	-2.0427
ENSG0000	TRIM5	11:566355	7.60878	4.51479	8.47568	8.0594	10.2331	14.2217	7.38517	8.48	3.39677	13.8495	0.85925	0.145827
ENSG0000	MNAT1	14:607347	13.7184	16.7048	11.3117	28.0217	12.2545	20.4905	47.0693	22.2946	15.4914	12.1056	0.6149	0.263939
ENSG0000	TMEM128	4:4235541	24.2244	24.5845	43.7539	42.4091	25.6069	25.2664	28.7662	34.3111	44.3142	16.8328	0.7569	-0.1201
ENSG0000	CCDC130	19:137317	58.8001	70.542	73.3724	71.1404	53.0842	59.4309	82.7367	91.5865	61.3372	66.1916	0.73595	0.106749
ENSG0000	TSPAN17	5:1766473	24.729	19.6155	19.1235	13.1984	13.1245	23.8429	37.7177	22.3891	26.5545	23.4422	0.14885	0.570917
ENSG0000	POLR3K	16:46406-	21.9335	22.503	35.4546	26.2455	22.7749	27.389	45.4716	26.3027	23.3865	5.65461	0.9525	-0.02779
ENSG0000	BOLA1	1:1498875	29.7829	33.626	48.2676	32.1769	46.7289	34.5947	24.7487	28.2552	13.4438	21.1737	0.1541	-0.71035
ENSG0000	APOL2	22:362262	15.5886	10.3307	38.1098	15.2126	25.4381	20.5989	13.9131	38.4423	11.8483	47.1625	0.5202	0.232431
ENSG0000	RFWD3	16:746213	3.23432	6.6101	3.47189	4.27572	1.82874	1.23784	4.83718	9.16148	4.06802	4.98788	0.5966	0.265866
ENSG0000	IGKV4-1	2:8888539	0	5.88191	2.27092	1549.31	1.49185	0	0	0.438047	0	3.64853	0.0038	-8.31567
ENSG0000	CD68	17:756187	4.37613	43.5377	0.698861	1.45155	2.05834	266.87	5.82675	5.05485	7.6875	2.02612	0.32145	2.60704
ENSG0000	RNF146	6:1272666	25.9363	19.3212	10.1991	20.4123	9.50967	15.0047	9.1953	13.1547	24.9651	17.4574	0.8532	-0.07017
ENSG0000	SNX33	15:756361	0.859623	1.17466	0.279172	0.316174	0.217496	0.299815	0.651736	0.315391	0	0.571864	0.564	-0.74712
ENSG0000	CTB-75G1	17:366340	0.537853	0.828713	0.280147	0.774279	0.14371	0	0	0.604394	0	0.690937	0.68265	-0.66789
ENSG0000	TMEM255	X:1202586	1.07135	0	0.050437	0	0.57784	0	0	0	0	0.501453	0.44675	-1.78771
ENSG0000	RP11-514	6:1664093	0.463243	0.111714	0.813121	0	0.906226	0	0.675807	0	0	0.125867	0.4379	-1.6561
ENSG0000	FAM53A	4:1617914	0	2.45658	0.505412	0.390093	0.154368	0	0	1.02016	0	0.667509	0.46725	-1.04646
ENSG0000	SPAG7	17:495922	95.3737	67.8831	176.92	150.534	202.417	135.149	142.176	145.807	125.71	119.221	0.77415	-0.09374
ENSG0000	RP11-452	10:973324	0.30246	0.432942	0.619527	0.300384	0.202847	0	0.170624	0	0	0	0.20495	-3.76756
ENSG0000	RFFL	17:349278	10.0615	21.0018	8.46969	10.3923	6.34146	21.7184	18.7271	13.624	21.7075	15.2524	0.35395	0.586681
ENSG0000	SENP3	17:756187	4.39364	5.54465	0.37845	0	0.540639	11.2365	11.2156	0.90659	0	1.06411	0.363	1.53749
ENSG0000	PELI1	2:6409265	9.23602	24.1951	2.71687	5.64295	2.00219	11.1041	16.6899	5.84599	2.97237	4.34667	0.6118	-0.24103
ENSG0000	TRAV12-1	14:218412	15.9724	32.336	18.3583	21.108	10.5155	23.4568	66.9974	19.7993	17.003	12.4262	0.4053	0.570751
ENSG0000	ACOT1	14:734784	6.19826	5.10422	11.4795	8.38293	8.59622	10.8851	8.11636	9.13122	2.76669	5.28666	0.8971	-0.19102
ENSG0000	SLC43A3	11:573867	28.7896	36.3208	11.205	15.8903	21.0197	46.52	45.6946	16.6293	13.5146	41.413	0.13405	0.513932
ENSG0000	HOMER3	19:188995	2.51246	3.81857	0.916767	0.253487	0.467591	9.00794	0.867409	0	0.119562	0.066049	0.9162	0.212989
ENSG0000	DYRK3	1:2066346	0.236835	0	0.422076	0.059172	0.202416	0	0.260442	1.26347	1.13927	1.51279	0.1193	2.1041
ENSG0000	HDAC7	12:477060	26.6384	28.821	10.2169	9.71824	16.2832	16.4759	22.0466	13.5161	5.30071	35.5567	0.6743	0.172227
ENSG0000	CLNS1A	11:775149	31.9594	35.3157	50.9031	37.7616	38.9964	46.215	54.3937	44.7227	41.079	30.9118	0.8421	-0.0814
ENSG0000	PHRF1	11:576485	15.9119	12.6662	3.99325	2.57094	7.67847	4.76762	10.0199	7.81198	0.121356	13.1707	0.37185	-0.38722
ENSG0000	FPGS	9:1277945	37.3961	34.3364	43.3713	57.9142	43.3233	44.3698	36.2668	53.4519	26.2872	48.4489	0.67625	-0.1388
ENSG0000	BATF	14:755224	81.241	59.2367	100.114	64.7605	113.613	218.401	456.83	270.647	119.138	67.9229	0.0002	1.26503
ENSG0000	MFSD12	19:350627	24.9628	34.3711	6.09299	1.60646	13.2972	30.4862	27.7227	10.8815	4.65038	14.7217	0.9065	0.114573
ENSG0000	ZFAND2B	2:2191952	66.1471	72.9361	74.1523	82.2395	62.4864	87.4797	82.2813	68.6565	56.6712	53.5815	0.7506	-0.10843
ENSG0000	PHF7	3:5241065	2.24069	5.61483	4.52757	4.61552	1.11017	8.94586	1.29583	3.34011	0	2.69198	0.851	-0.1871
ENSG0000	TMEM9	1:2011347	21.149	18.2788	30.9599	28.2528	16.9204	30.9408	15.0965	18.4741	34.217	15.4135	0.9311	-0.03457
ENSG0000	NT5C3B	17:418251	10.2378	8.9164	13.1243	15.3095	21.1108	17.5258	11.8988	15.0823	10.4652	11.6343	0.8995	-0.05934
ENSG0000	FKBP7	2:1784636	1.62822	0.815499	2.29301	2.8913	2.49393	8.95787	1.50408	1.91455	1.5053	0.71097	0.78845	0.224887
ENSG0000	RP11-188	3:4328651	0.333835	1.51125	0.176753	0.080807	0.240831	0.350439	0	0.132298	0	0.147714	0.3059	-1.8837
ENSG0000	MRPS6	21:340732	78.061	138.678	142.521	68.7569	120.329	127.367	217.245	134.572	141.453	40.5175	0.58235	0.221433
ENSG0000	PIGX	3:1966396	17.3248	10.9476	16.1584	19.3268	17.2378	13.5502	8.81326	12.537	16.7122	12.8226	0.24135	-0.50548
ENSG0000	RPL35A	3:1978890	250.905	230.358	344.652	269.791	100.702	320.409	358.14	268.546	179.745	25.095	0.84225	-0.0932
ENSG0000	CTD-3222	19:164790	0.537467	1.38902	0.049042	0.309038	0.126283	0.157176	1.07595	0.051741	0.123234	0.727754	0.98265	-0.03371
ENSG0000	ADCY7	16:502461	47.3689	34.5139	13.1302	38.8611	38.0878	29.2226	27.8747	17.7917	30.3206	51.6271	0.66785	-0.27246
ENSG0000	RP11-261	13:207287	0	0	0	0	0	0	1.66166	0.376392	0	0.105258	0.08195	inf
ENSG0000	PGRMC2	4:1282652	5.19327	3.90659	3.79049	3.9577	6.33848	4.89429	7.34305	4.13974	7.28555	5.72426	0.5755	0.26868
ENSG0000	KPNA2	17:680355	31.2356	55.7142	18.3539	32.8285	23.1293	48.6036	42.9371	29.1321	42.5838	34.3032	0.31015	0.335561
ENSG0000	RP11-577	7:1024824	9.83537	14.5249	30.3017	24.1628	22.8005	11.9531	12.1279	30.854	11.8768	23.9078	0.92885	0.134781
ENSG0000	GOLGA3	12:132768	3.97212	3.97224	3.28779	2.24183	3.80894	3.22048	5.19767	1.97253	2.19488	9.33114	0.78665	0.198744
ENSG0000	GRK6	5:1774021	168.323	189.795	56.9379	69.1145	95.146	76.1098	80.4095	42.3616	45.6813	90.7952	0.0297	-0.83599
ENSG0000	AC010894	2:1745471	0.428306	0.183514	0.228632	0	0.115433	0	0.323311	0.390917	0.759011	0.461203	0.45165	1.24959
ENSG0000	RNF219	13:778954	9.60365	7.01341	5.16783	3.1379	5.06963	7.5473	7.6932	8.96036	2.17548	9.16701	0.63495	0.216144
ENSG0000	RP11-666	17:765273	0.118727	0.072039	0.923938	0	0.481653	1.45467	0	0.523624	1.411	1.02714	0.2122	1.54333
ENSG0000	RP11-119	11:678864	0.265062	0.396517	1.02107	3.2641	0	0.764835	1.1945	0.302604	2.99759	0.826032	0.8931	0.250707
ENSG0000	C18orf54	18:543243	0.853771	0.786018	0.138822	0.430347	0.304155	2.27555	0.320049	1.07611	1.24142	2.2745	0.06055	1.51862
ENSG0000	CLSTN3	12:712907	3.66091	2.8667	2.03974	2.69916	3.79139	4.5448	2.23704	5.9878	4.58786	13.4506	0.068	0.943471
ENSG0000	RP1-122C	6:8964249	0.770211	0.165641	0.298415	1.84499	0.696201	0	1.64057	0.768921	0.419347	0	0.80175	-0.46722
ENSG0000	IGLV1-47	22:223577	0	3.56612	2.82187	0.258792	1.37048	0	0	0	0	1.58401	0.29915	-1.737
ENSG0000	RP11-252	16:742967	5.04935	6.35363	3.40704	2.81591	1.52667	5.22855	2.2877	2.2864	0.889134	2.47339	0.50655	-0.48788
ENSG0000	LINC0065	20:627741	0.717357	0.238876	1.46264	0.625998	0.280647	0	0.374068	0.321568	0	0.841286	0.5203	-1.00917
ENSG0000	SNTB1	8:1203807	6.75855	6.73255	2.82155	0.988467	3.1746	6.84798	2.73243	3.10796	2.03655	4.93881	0.8213	-0.24376
ENSG0000	NBPF14	1:1485313	6.7325	11.4608	6.84697	11.1362	8.47974	12.0329	10.9613	8.06394	16.0909	12.7378	0.6142	0.45621
ENSG0000	MAP2K6	17:694146	10.7118	9.51061	8.56595	9.63768	8.36558	4.77557	3.04045	5.28352	9.75463	9.73115	0.39265	-0.47301
ENSG0000	TRAV24	14:221053	4.98098	16.6997	2.87805	0.83209	0	18.4217	38.4829	0.35121	0	0.400566	0.6479	1.14447
ENSG0000	TMEM154	4:1526186	6.53198	16.0258	4.07212	3.80267	4.20817	6.94593	14.1626	3.43566	9.77442	8.2591	0.9536	0.047836

ENSG0000	CLASRP	19:450390	7.09446	9.98476	2.70949	4.05565	4.12525	7.78212	10.4409	4.34517	1.85347	5.0134	0.9029	0.083011
ENSG0000	PTCRA	6:4291598	0	0	1.03045	3.14501	0.289586	0	0	0	0	0	0.011	#NAME?
ENSG0000	TCF7	5:1341147	69.9447	209.359	27.5058	16.4424	47.3915	0.847587	10.1158	11.5922	7.81018	41.4425	0.0227	-2.40681
ENSG0000	AF196972.	X:4850652	1.07649	0.357135	0.945345	1.96491	0.604533	0	0	0	0	0	0.0402	#NAME?
ENSG0000	GNAI2	3:5022629	396.707	496.572	162.943	43.4252	312.133	433.224	344.16	142.419	84.8503	308.393	0.61825	-0.19969
ENSG0000	PHF23	17:723502	21.5554	21.5879	13.8189	7.99754	21.2521	20.5447	19.4304	15.5223	1.74578	25.5618	0.77345	-0.12833
ENSG0000	ANKRD44	2:1969670	0.666881	1.01037	0.191136	0.098549	0.082303	0.268807	1.17945	1.27073	2.66625	1.41504	0.17475	1.91244
ENSG0000	RP11-358	16:780994	0.553049	0.480982	3.70711	4.3473	1.25563	0.805078	0.064671	1.88958	0.611768	2.70789	0.65865	-0.9201
ENSG0000	CTD-2540	19:332999	0.966582	0.182334	0	0.076987	0	1.35894	0	0.202571	1.15049	0.384729	0.3223	1.88524
ENSG0000	TMEM81	1:2050831	1.38844	1.49629	1.32851	0	1.1022	0.426768	1.25111	1.16806	0	2.78677	0.9259	0.048555
ENSG0000	ZNF775	7:1503637	25.6151	25.1017	15.5171	10.7648	16.5199	15.3776	12.8718	9.25709	12.0164	14.9599	0.63835	-0.46442
ENSG0000	IPO4	14:241718	11.4645	8.82647	7.61966	11.7332	9.79134	5.22707	7.13281	9.04262	6.30342	6.06118	0.5785	-0.40679
ENSG0000	RP11-879	18:615857	0	0	0.48757	2.29219	0	0	0	0	0	0	0.0673	#NAME?
ENSG0000	RP11-493	12:473416	1.57248	4.40248	1.01921	2.83209	0.296623	2.38278	1.06471	0	2.43071	0.929068	0.8414	-0.40902
ENSG0000	CLDND1	3:9849760	255.49	189.27	123.229	182.087	59.046	224.47	173.913	104.389	169.229	50.0846	0.7135	-0.11686
ENSG0000	LINC0089	22:202063	0.493495	0.103404	0.245935	0.228135	0.266209	2.12111	0.498454	0.137315	0.266387	0.488951	0.50345	1.28537
ENSG0000	SCNN1D	1:1280435	1.02386	0.671094	0.377728	0.193957	0.263142	3.80659	0	0	0	1.00886	0.52775	0.899922
ENSG0000	TTC32	2:1989664	18.0771	45.1863	24.2396	31.3374	18.908	15.2121	38.9443	29.1583	15.6883	20.7623	0.66605	-0.21887
ENSG0000	CCDC59	12:822236	56.0366	44.4603	59.5259	60.2737	49.1992	53.603	68.1442	43.3521	42.0903	19.8497	0.60235	-0.32103
ENSG0000	ABTB1	3:1276729	77.3583	89.4346	38.3757	32.478	33.2184	43.2278	50.5009	33.1163	30.6838	30.7096	0.13075	-0.5188
ENSG0000	RABEP1	17:528226	30.0626	20.5092	12.4105	16.3399	23.4278	22.0753	18.6296	7.95562	30.2054	20.7673	0.9294	-0.06041
ENSG0000	RP11-677	11:124739	0.362335	0.612812	0.610909	4.33615	0.158735	0	0.133515	1.62196	3.12209	0	0.9643	-0.06583
ENSG0000	SLC7A1	13:295094	1.97239	2.70411	1.73976	0.30702	1.46737	3.42315	3.56386	4.23889	0.064148	3.67983	0.15835	0.799409
ENSG0000	RP11-169	3:1127230	0.590478	0.896689	0	0	0	0	1.05012	0	0	1.20362	0.6858	0.969543
ENSG0000	CUL3	2:2244646	39.6103	44.3645	19.2032	18.7055	16.0051	21.6958	27.3072	18.225	17.1331	31.9769	0.63455	-0.18546
ENSG0000	FRY	13:320253	0.860432	0.720171	0.353176	0.616509	0.386504	0	0	0.53092	0	0.47262	0.20295	-2.03625
ENSG0000	ZNF343	20:246163	2.57792	3.47378	1.35461	0.718034	1.60431	1.58003	2.70431	1.63891	0.428639	3.50899	0.97675	-0.03249
ENSG0000	DIDO1	20:628777	26.8888	51.8284	27.2905	19.7939	22.0307	23.5731	31.3222	28.9723	19.917	33.6426	0.8618	-0.0767
ENSG0000	CXorf40B	X:1499295	26.7655	20.1548	36.8012	40.8662	45.0077	38.4597	47.7223	27.356	22.0359	23.0735	0.6878	-0.18377
ENSG0000	CTD-2165	5:1466180	0.450329	0	0.040199	0	0.225299	2.0922	0.165347	0.015374	0	0.439321	0.4137	1.50702
ENSG0000	SLC12A6	15:342249	3.82645	15.6422	0.908425	1.14532	1.06253	2.48929	6.70356	1.83824	1.36235	3.69586	0.67005	-0.49402
ENSG0000	NEMF	14:497676	44.2908	40.8468	47.0623	30.6135	44.9231	58.2026	58.4245	35.8037	31.875	77.2939	0.9376	0.038826
ENSG0000	GSPT1	16:118516	36.6403	43.1573	35.889	31.8501	40.1747	61.5319	51.7769	52.6428	55.7062	45.6557	0.40545	0.310653
ENSG0000	AD000671	19:357180	4.54264	4.94842	11.2286	4.43445	7.24959	10.8197	12.1853	17.9382	12.6433	3.47356	0.6405	0.660618
ENSG0000	ALS2CL	3:4666899	1.70987	9.81392	0.12079	0.605693	0.085862	0	0.120346	0.945385	0	0.069013	0.02905	-3.25771
ENSG0000	C11orf71	11:114391	3.62699	4.37881	2.97098	3.84669	1.74099	2.73821	3.94584	0.20231	1.45736	1.44546	0.5715	-0.74544
ENSG0000	RP11-385	17:154360	0	0.506868	0	0.255039	0.199177	0	1.72807	1.24843	1.95094	0	0.20185	2.45537
ENSG0000	PLXDC2	10:198162	1.87395	2.00303	0.372168	0.496056	0.535424	13.7705	0.43989	0.05254	1.18477	0.640825	0.2169	1.4627
ENSG0000	SDAD1P1	8:2637925	1.61612	0.459356	1.37021	0.743169	0.404942	1.70684	0.357547	0.313077	0	0.913751	0.62005	-0.80934
ENSG0000	COQ10B	2:1974534	43.6492	36.6236	43.5968	46.9541	23.1468	35.8096	38.8439	36.1746	30.0539	22.4768	0.3695	-0.28489
ENSG0000	RP3-508I1	22:387057	0.714842	1.10566	0.260371	0.315991	0.348157	1.64162	4.67454	3.65802	3.22796	1.0531	0.0483	2.4639
ENSG0000	GPR174	X:7917097	4.34375	3.39164	1.92077	0.840866	1.42487	1.39487	5.54235	2.97369	0	4.83642	0.60685	0.40482
ENSG0000	TPRN	9:1371916	0.817281	1.10543	0.164561	1.09871	0.82196	0.079694	2.09608	0.83265	0.350131	1.92518	0.6713	0.605227
ENSG0000	LINC0062	1:1471727	0.400203	0.727743	0.819324	0.752197	0.074014	0.144165	0.57307	0	0	0.671553	0.57675	-1.18537
ENSG0000	EIF3K	19:386190	168.777	147.968	276.079	221.349	438.314	198.898	189.743	239.343	232.452	133.651	0.2761	-0.37401
ENSG0000	PDK2	17:500947	20.7078	32.5087	34.184	28.3206	28.0919	12.3728	13.6611	14.6282	23.1666	19.3847	0.0818	-0.85404
ENSG0000	DNAJC1	10:217565	62.9032	25.3114	59.7024	52.2498	126.208	54.4337	63.7669	63.6387	61.9046	116.576	0.82895	0.084233
ENSG0000	SERF1B	5:7002524	12.1106	13.9977	29.2783	30.9001	16.0497	5.54236	15.9406	18.7307	7.90991	5.71513	0.6053	-0.47173
ENSG0000	PEG13	8:1397303	0.583985	0.478255	0.55968	0.802778	0.22565	0	0	0.007542	0	0.660823	0.424	-2.39838
ENSG0000	FRAT2	10:973324	32.46	33.0664	32.6497	18.9914	32.1275	31.8205	26.3838	14.5182	7.58012	15.4118	0.08595	-0.69649
ENSG0000	THAP1	8:4283667	20.3268	20.7039	13.0467	10.3411	10.0703	17.2546	15.2767	15.8804	3.14864	9.3354	0.51595	-0.26268
ENSG0000	DSTYK	1:2051425	3.06919	5.25268	5.00803	2.00791	3.55916	3.23535	2.83295	3.18302	2.53653	6.63628	0.57465	-0.22966
ENSG0000	KLHL2	4:1650706	11.8354	10.6548	4.13414	4.77428	2.85693	15.178	13.5219	5.92312	2.96478	6.2806	0.55825	0.337254
ENSG0000	POLI	18:542683	12.5711	15.8768	8.35255	16.0577	4.6763	7.12778	12.5208	9.69382	11.4546	14.216	0.9045	-0.06064
ENSG0000	HNRNPA1	18:320183	9.08295	11.2294	4.69165	5.2288	3.819	9.55742	10.2922	4.12744	3.65037	1.85567	0.95465	-0.08967
ENSG0000	C18orf8	18:235034	44.6884	35.0174	31.8091	39.9231	37.0165	61.0592	38.8974	41.4624	52.9734	45.7286	0.27125	0.417998
ENSG0000	ZBTB25	14:643880	14.1467	19.2001	19.0906	12.7627	11.7032	7.49572	8.53406	20.3393	23.2897	19.7032	0.8619	-0.13738
ENSG0000	PCGF5	10:911630	29.0744	26.4155	21.6615	45.515	22.1975	28.7806	44.9747	27.9806	29.7636	13.4983	0.98985	-0.00738
ENSG0000	NAPA	19:474842	110.464	122.951	141.727	126.038	146.192	119.125	196.845	147.301	133.642	103.752	0.82635	0.066967
ENSG0000	LINC0099	7:1131006	81.4323	45.2537	98.2129	62.4337	49.4133	102.422	92.3638	71.13	103.349	18.6518	0.55695	0.207323
ENSG0000	IL17RC	3:9917073	4.46502	3.91756	2.24115	4.04535	2.5658	2.25685	1.72779	3.09032	0.552795	1.57312	0.14885	-1.00548
ENSG0000	PIK3R4	3:1306789	15.3329	10.274	4.67684	4.76807	6.53033	3.56501	3.84012	4.39011	4.39993	10.1836	0.9883	-0.00981
ENSG0000	RPL35	9:1248534	573.815	328.73	810.771	603.504	399.056	528.621	679.144	553.813	365.227	71.0677	0.25015	-0.39533
ENSG0000	NOL7	6:1361532	97.3853	100.816	116.618	99.2411	160.408	129.582	157.196	111.94	100.512	43.6959	0.7391	-0.13452
ENSG0000	RAPGEF5	7:2211823	0	0.183357	0	0	0.139179	0	1.5007	0	1.82862	2.76758	0.09165	4.82404
ENSG0000	SNRPGP1	1:2053360	2.0969	0.908614	0.989479	7.58744	1.07047	2.48263	2.21376	1.8452	2.9546	0	0.82075	-0.3735
ENSG0000	IL17RA	22:170849	8.98117	16.9423	7.39817	5.41112	7.06138	8.33449	9.66226	7.33814	5.63127	13.915	0.68855	-0.18944
ENSG0000	HLA-F-AS	6:2972277	2.66043	2.09624	2.1851	1.08751	1.20416	1.99053	1.64952	1.31825	4.19449	1.72117	0.71825	0.304129
ENSG0000	EMC3-AS	3:9958716	0.291257	0.110965	1.81825	2.30996	0.694319	1.24845	1.1953	1.45632	1.73151	2.38774	0.9365	0.098663
ENSG0000	USP54	10:734364	0.220405	1.29276	0.659019	0.340929	0.593021	0	0.327497	0.450331	0.067614	0.923397	0.11575	-1.2116
ENSG0000	BCL9L	11:118883	3.89982	4.78174	1.12133	1.26048	4.26275	3.19195	1.91713	1.10799	1.2962	6.90892	0.9936	0.006246
ENSG0000	UXS1	2:1060933	53.0598	76.4223	39.2501	39.2247	36.2156	55.6076	52.3701	33.2161	33.4844	30.7252	0.3298	-0.32561
ENSG0000	RP11-860	12:277798	0	0	0	0.220984	0.236943	0.132641	0	0	0	0.20446	0.7117	-0.76764
ENSG0000	EIF3D	22:365108	88.8927	88.6806	168.115	116.304	154.842	84.2429	68.3344	131.35	120.597	114.005	0.3945	-0.26662
ENSG0000	LINC0095	10:130063	9.81958	6.1654	18.334	11.882	3.47846	2.12978	3.7314	7.35616	7.33541	1.42687	0.2737	-1.17066
ENSG0000	CBR1	21:360345	33.6474	40.3168	67.6629	54.7786	71.7752	47.0866	55.4293	45.0171	36.037	31.3325	0.34755	-0.37573
ENSG0000	KNOP1	16:195552	14.1612	20.8671	27.3758	18.3207	46.4099	25.718	24.2727	17.7058	9.20294	23.7944	0.51165	-0.44475
ENSG0000	GBP3	1:8900666	14.6574	17.3687	38.0083	32.6494	37.6836	25.6843	32.1709	44.5475	20.7267	71.4229	0.53785	0.226985
ENSG0000	LRPPRC	2:4388650	16.4695	12.5191	9.69337	7.75652	15.3646	11.1843	12.9254	14.0993	12.4056	24.2121	0.9456	-0.03402
ENSG0000	CDC40	6:1101801	11.4423	12.124	9.13152	9.53337	13.7268	11.2788	8.91858	10.733	11.4212	13.7002	0.56635	-0.24231
ENSG0000	CTD-2651	15:450926	0.335736	0	0.474987	0	0.470086	0	0.690725	0.794834	1.13988	0.211825	0.51675	1.13605
ENSG0000	CAB39L	13:493086	3.8308	5.32995	3.13726	2.14592	1.26592	3.07748	3.87982	1.7479	0.474259	2.35699	0.4211	-0.58213
ENSG0000	RP11-667	7:1162092	5.23455	3.81224	3.00134	9.84289	3.3542	0.901348	1.37785	1.57325	2.86645	0.77828	0.2043	-1.70668
ENSG0000	PTOV1	19:498182	35.1862	54.5988	55.5772	31.5492	84.2455	34.2529	34.1612	60.4447	37.5687	51.1695	0.6395	-0.26234
ENSG0000	USP32	17:601790	5.53242	10.1665	1.1965	1.4597	1.09931	7.92573	4.0791	2.46077	0.49036	2.9429	0.79815	-0.14246
ENSG0000	STMN1	1:2588418	183.319	411.397	94.6026	103.858	149.809	382.261	351.542	162.66	158.643	41.9678	0.5247	0.241052
ENSG0000	TCEAL9	X:1033564	0.386121	2.41802	1.04062	0.653979	0.142196	14.1546	2.33757	0	0	0.170147	0.2383	1.85483
ENSG0000	PIGZ	3:1969463	0	1.01865	0.56345	0.610656	0.39132	0	0	0	0	0.937685	0.492	-1.32724
ENSG0000	PHKA2	X:1889029	7.79715	7.85609	3.2856	1.67347	5.25206	3.3266	5.05537	8.57397	2.18952	11.389	0.6073	0.286806
ENSG0000	E2F1	20:336756	3.63321	5.61518	0.801499	0.89466	0.789218	7.63039	7.3755	2.28034	1.1504	2.58403	0.2174	0.883625
ENSG0000	PLBD2	12:113298	1.55737	1.90895	5.6196	5.16512	5.03004	1.84	2.33598	7.84617	0.264858	8.63897	0.85535	-0.18534

ENSG0000C	CTSZ	20:589951	0.891199	1.47206	0.498102	0.691816	1.49793	8.00493	0.423825	0.779806	1.97756	2.87782	0.30435	1.53194
ENSG0000C	RP11-108	11:111782	0	0.772724	0.278662	0.094758	0.385289	0	0.351461	0.400115	0.651581	0.065815	0.815	0.386318
ENSG0000C	SNX29P2	16:292912	0.475585	2.93912	1.46002	1.43511	0.742978	0.825831	1.74625	0.032382	0	1.15017	0.72325	-0.4623
ENSG0000C	RP11-977	12:562716	6.4052	6.33693	6.49704	8.23566	2.67966	6.08455	6.15168	2.7414	4.5687	1.2312	0.65375	-0.48685
ENSG0000C	CISD1	10:582690	30.3743	16.896	21.8122	17.1346	31.2646	32.3063	35.593	14.6998	12.7455	12.3502	0.73225	-0.20932
ENSG0000C	RP11-961	17:171149	0.325871	0.815061	0	0.469101	0	0	0	0.009501	0.473555	0	0.8886	-0.20701
ENSG0000C	HOTAIRM	7:2709299	8.83552	8.63393	15.7302	24.9215	5.60072	1.3853	10.7981	10.2662	15.4929	2.87475	0.2833	-0.96284
ENSG0000C	IGHV3-74	14:106810	0.425645	28.1555	2.28569	0.171382	0	0	0	0	0	0	0.1246	#NAME?
ENSG0000C	C19orf44	19:164790	2.92435	1.22332	1.13728	0.197605	0.635728	0.613129	0.116595	0.882654	0.554416	0.427442	0.56865	-0.60626
ENSG0000C	RNF214	11:117232	14.5112	28.0124	4.32003	4.66176	2.70349	12.4241	14.6212	4.65642	3.00417	7.06525	0.53415	-0.35128
ENSG0000C	RP11-499	4:1022510	0.08842	0.173333	1.69469	1.85677	0.581379	2.54657	0	0	0	0.264766	0.6193	-0.90405
ENSG0000C	TNFRSF10	8:2268765	9.23425	9.84598	10.0628	8.6662	3.00482	4.31525	3.3081	13.9375	8.70274	8.94932	0.84675	-0.09343
ENSG0000C	GNE	9:3619085	1.90032	0.923916	2.83626	1.17931	1.90235	1.75614	0.466151	3.54681	0.790384	3.0026	0.936	-0.09015
ENSG0000C	RP11-169	16:469739	0.358215	0.682595	0.86826	0.345575	0.464667	1.40909	0	0	0	0.349787	0.6573	-0.69252
ENSG0000C	AC125232	2:8703181	9.27451	14.5176	12.303	10.1575	3.36359	5.10329	11.7507	11.6726	9.56688	6.82462	0.75395	-0.18261
ENSG0000C	TRAV41	14:222025	3.75445	12.5961	1.12365	18.1412	0.845254	4.79263	8.82579	1.63239	0	0	0.47595	-1.33294
ENSG0000C	FAM3A	X:1545061	30.3299	29.2464	18.9013	21.5966	19.2746	35.0197	32.0235	26.3936	17.6744	17.8795	0.8606	0.073395
ENSG0000C	NSDHL	X:1528309	9.57222	5.80264	14.7029	10.6165	14.9114	8.23024	13.0995	13.2841	2.42826	7.43291	0.3898	-0.42904
ENSG0000C	RP11-477	14:104815	0.859668	2.42369	0.406684	1.20501	0.188588	5.01455	2.41467	1.41045	0.475587	1.06686	0.50675	1.18548
ENSG0000C	DPH2	1:4396999	27.0792	26.3874	34.7746	48.0617	23.8096	22.297	26.2088	32.5731	31.1219	24.957	0.452	-0.2522
ENSG0000C	MTERF3	8:9623939	32.0944	25.9163	26.1208	31.4724	14.1575	42.5083	32.3173	21.9055	30.0062	17.2933	0.61865	0.201646
ENSG0000C	PORCN	X:4850896	10.5853	8.30169	18.0094	15.3517	28.4356	7.88703	11.2573	18.1295	10.4702	16.2028	0.3518	-0.48622
ENSG0000C	PHKG2	16:306977	52.0626	59.5816	75.54	73.0578	61.4702	44.5587	46.8859	52.9107	46.6654	53.1285	0.4633	-0.4124
ENSG0000C	LRRC8B	1:8952483	2.09065	7.86028	2.21573	0.63609	0.985003	3.7997	1.75623	3.24164	3.01502	2.87884	0.98485	0.021938
ENSG0000C	EPHA1-AS	7:1433810	0.013456	2.97364	0.050236	0.305774	0.021878	1.09069	2.37938	6.68738	3.906	2.71967	0.1448	2.58419
ENSG0000C	PLPP6	9:4490443	3.83082	8.13079	2.8429	4.43364	2.32142	0.256149	6.10548	5.80494	5.11659	6.4875	0.7818	0.151873
ENSG0000C	GAA	17:801015	10.1958	16.6481	11.6918	15.869	24.7109	15.6203	7.79327	20.0048	7.42702	23.6156	0.6055	-0.30315
ENSG0000C	MS4A7	11:603348	1.12763	10.2423	1.28697	1.72631	2.46632	77.3389	3.63756	3.00471	10.8345	4.37715	0.00005	2.67232
ENSG0000C	RTN4	2:5497218	22.7019	30.1316	15.1934	16.1835	18.6163	15.4116	13.2433	14.2453	24.2354	22.1773	0.61075	-0.18516
ENSG0000C	ABCE1	4:1449671	53.617	35.876	25.4219	23.1284	23.5448	24.7335	34.8397	16.0684	32.9465	26.9781	0.49605	-0.26053
ENSG0000C	SLC25A44	1:1561939	8.52824	9.54372	9.55042	3.38798	5.39155	7.79036	9.67202	9.56588	3.29248	13.3398	0.7986	0.101981
ENSG0000C	AC108488	2:3531812	1.97948	3.21164	1.6922	2.59288	0.8849	5.30624	0.866718	1.44633	7.16832	0.452795	0.54645	0.640821
ENSG0000C	ZNF577	19:518713	7.07264	5.73184	1.50904	3.2556	1.14951	6.68731	0.734806	2.41689	3.24912	3.10276	0.792	-0.14054
ENSG0000C	SLC25A43	X:1193990	2.89703	4.22435	1.08657	2.56581	3.09274	10.1405	7.08673	4.67946	12.0382	8.10918	0.0027	1.57956
ENSG0000C	RP11-793	12:534416	2.22065	3.24056	1.31616	0.385808	1.98075	0.815157	2.63918	1.30694	3.45887	1.80123	0.77035	0.36345
ENSG0000C	LIMD1-AS	3:4555539	4.79542	5.14554	2.77844	1.68122	2.26895	9.31727	2.24487	2.82222	0	2.68933	0.93345	-0.11502
ENSG0000C	TMCO4	1:1968221	11.1179	12.8262	7.38018	10.7747	9.49929	4.43528	9.70629	7.52112	7.65173	9.72502	0.3806	-0.39148
ENSG0000C	SGMS1	10:503055	13.9796	15.9435	6.32075	11.0658	9.40713	16.5628	30.1685	17.2474	16.8356	14.6613	0.0776	0.86522
ENSG0000C	CDCA5	11:650662	4.66789	7.15551	0.948298	1.61961	0.598269	6.5139	16.1565	1.17998	3.03889	3.69556	0.1063	1.09321
ENSG0000C	CXorf38	X:4062692	14.2542	9.39923	8.08848	11.4055	12.46	14.6978	9.27727	7.57235	10.3188	16.859	0.9503	-0.02866
ENSG0000C	MSRA	8:1005426	1.23907	0.832662	0.897431	0.962807	1.59865	0.410089	2.15367	4.09548	0.324927	1.63745	0.4653	0.718049
ENSG0000C	AMIGO2	12:470757	7.82616	15.6861	7.34475	4.75209	5.42937	8.24623	19.4692	7.29232	3.25084	12.1833	0.84815	0.182976
ENSG0000C	FCGR2A	1:1615054	6.05845	30.8132	0.367187	1.84397	0.762875	81.7577	1.41621	4.46094	2.34391	1.01079	0.35135	1.23342
ENSG0000C	CLTB	5:1763654	75.2392	71.8771	89.9864	79.6406	126.306	135.484	131.342	114.635	102.681	72.6853	0.61225	0.266407
ENSG0000C	MT-ND4L	MT:10469	494.033	284.009	1560.24	3464.72	3612.86	577.063	272.314	1179.98	3005.22	1652.81	0.70495	-0.40775
ENSG0000C	FGFBP2	4:1596024	4.49471	74.6743	80.2859	68.6878	42.2716	2.16743	1.65843	3.44666	0.055676	2.58758	0.00005	-4.82662
ENSG0000C	RPS3	11:753994	743.045	768.943	2089.57	1921.73	886.2	504.849	637.753	1332.37	1553.08	185.236	0.23815	-0.59235
ENSG0000C	RPL21P93	10:288989	6.49026	4.21664	2.35267	1.96458	0.724665	3.49428	3.96603	0.602319	0.692877	0	0.48715	-1.42255
ENSG0000C	MUTYH	1:4532916	29.3455	32.4718	33.1932	50.5063	19.8183	33.6187	32.3322	24.7046	34.8215	33.9552	0.93105	-0.03709
ENSG0000C	KIAA0232	4:6781374	11.6744	6.23048	3.21353	4.77403	5.5512	13.9499	10.7743	3.78264	9.24958	10.7034	0.58735	0.488728
ENSG0000C	RP11-505	16:841170	2.69754	2.63378	1.89277	3.3026	1.49651	2.22649	1.37667	1.11994	5.55075	1.6872	0.89035	0.100747
ENSG0000C	KLHL36	16:846485	12.0595	15.3944	17.2082	23.2756	15.6554	9.56528	7.66126	21.3169	18.4503	29.9297	0.9575	-0.02621
ENSG0000C	PPP1R15A	19:488723	246.377	75.9453	127.229	303.54	43.4202	29.7696	42.7055	116.578	273.149	167.188	0.55695	-0.26207
ENSG0000C	SCARNA2	1:1091001	0	7.68E-05	0.730778	0.01848	0.865373	0	3.95859	2.71227	0	5.86E-05	0.68535	1.11002
ENSG0000C	ZMYM3	X:7123962	5.63254	4.11459	4.3692	5.43046	6.53454	2.28533	4.35968	4.09367	4.95339	5.69355	0.4701	-0.49376
ENSG0000C	MSTO2P	1:1557458	4.4484	6.93629	3.76868	7.23873	3.11581	5.00523	3.52774	1.65512	2.65469	2.96608	0.8632	-0.26151
ENSG0000C	RP5-894A	7:1415292	1.28863	11.0313	1.72946	2.26763	2.5987	3.50949	2.48018	5.58318	0	7.87597	0.9351	0.136027
ENSG0000C	CHD4	12:657008	30.4981	39.7064	20.1757	11.1753	45.8188	22.4653	16.6166	22.3113	13.2929	54.4074	0.61325	-0.18844
ENSG0000C	ARHGAP4	X:1538615	134.379	152.208	57.5225	87.7147	61.7793	90.5293	105.474	62.1402	101.785	92.2973	0.864	0.058824
ENSG0000C	MB21D2	3:1927968	0.214936	0.439009	0.210399	2.55234	0.160417	2.21857	0.74551	0.316341	2.78541	0.197746	0.45645	0.795184
ENSG0000C	PXK	3:5833287	29.519	21.2731	18.6949	21.9816	26.3207	17.3779	24.6354	21.7997	20.1649	37.0227	0.84925	-0.08007
ENSG0000C	MIEN1	17:396711	88.7256	83.4387	153.747	163.899	104.775	145.058	139.92	148.476	154.378	51.0269	0.96565	0.016442
ENSG0000C	RP11-140	6:3458728	15.3007	11.7673	11.1924	12.7798	4.17231	31.3536	12.9174	5.63308	6.90144	1.25359	0.9883	-0.02852
ENSG0000C	AASDH	4:5633828	7.16055	9.26602	5.1041	4.50366	4.31466	7.56946	5.39013	3.56461	0.675477	6.32904	0.2313	-0.71381
ENSG0000C	ZNF668	16:310564	10.6547	8.41036	16.6861	8.24303	15.947	11.9375	7.62619	13.3245	10.9449	21.0636	0.6311	0.230511
ENSG0000C	IFI27L1	14:940812	6.92875	7.82592	4.71005	7.03585	6.09624	16.312	17.639	13.8253	15.6566	2.70305	0.106	1.01821
ENSG0000C	TRBV12-4	7:1425637	9.63418	31.9236	13.2928	15.3544	2.90167	26.2765	37.9849	5.54996	47.4982	4.62492	0.2098	0.841876
ENSG0000C	TMPO-AS	12:985129	1.67906	1.89943	1.62181	0.959123	1.07459	6.18856	2.46879	2.15497	0.703008	1.44539	0.6237	0.599043
ENSG0000C	C5orf17	5:2395134	1.57162	0.03717	0.224174	0	0.092216	1.63532	0	0	0.845118	0	0.652	0.914676
ENSG0000C	BAMBI	10:286773	0.256167	0	0.051096	0.71872	0	0	0	1.42054	3.87908	0.865567	0.22505	2.72476
ENSG0000C	ALOX5AP	13:307134	292.995	113.686	547.569	376.933	307.202	401.097	646.544	823.545	589.527	192.887	0.0905	0.661924
ENSG0000C	7SK	6:5299561	13.0349	0.000183	24.7722	7.08699	0	22.1701	19.3938	30.5317	50.557	0.00031	0.40095	1.09467
ENSG0000C	RP11-326	11:333191	0.378303	0.810622	0.151831	0.697607	0.384231	0	0.433079	0.655182	0.10475	0.192514	0.6851	-0.68068
ENSG0000C	CYP4F22	19:155084	2.39072	0.275201	1.62514	1.28265	1.8806	1.27141	4.30153	1.74726	1.06144	4.94866	0.46995	0.652897
ENSG0000C	GTPBP8	3:1129904	15.1959	26.76	18.7721	13.6538	18.306	28.7289	27.4989	22.1741	3.68737	14.3558	0.9617	-0.0363
ENSG0000C	RP11-166	16:118516	0.534543	0.159235	0.024504	0.613706	0.107358	0.060452	0.413792	0.369646	1.39903	0.339414	0.5191	1.02267
ENSG0000C	ZNF644	1:9091529	25.5363	27.2531	13.9731	15.6723	19.5105	18.4931	32.1223	15.6227	21.8368	41.5706	0.68855	0.226947
ENSG0000C	TRGV7	7:3833504	18.1275	2.79733	54.796	24.1835	24.5274	7.49324	16.9257	29.0045	14.0513	19.5408	0.75165	-0.27504
ENSG0000C	DOK3	5:1774833	2.06331	14.0953	0.529849	2.56944	0.908673	31.4114	0.060256	1.55755	5.52376	1.13284	0.29595	1.11978
ENSG0000C	LAPTM4A	2:2003264	97.6595	77.5637	97.1564	89.9516	67.2364	139.722	136.223	96.6211	134.273	42.0568	0.3288	0.31598
ENSG0000C	RP11-732	11:668589	0.587425	0.822704	0.571516	0.130947	0	0.838111	1.97763	0.538832	0.453802	1.05661	0.44815	1.44071
ENSG0000C	ADD3	10:109940	171.429	201.349	59.1075	64.496	89.1886	71.502	43.3841	29.499	28.0536	58.2965	0.00005	-1.56535
ENSG0000C	MT1L	16:566174	1.39689	0.112159	0.581412	2.34655	1.70663	3.47782	0	0.475983	3.976	0.195872	0.84415	0.307425
ENSG0000C	WIPI1	17:682591	11.7907	9.17541	9.74292	13.5652	9.88668	13.2802	20.0683	10.0033	40.516	14.5451	0.3171	0.906058
ENSG0000C	MT-CO2	MT:7585-	2862.64	2335.76	5143.49	6604.43	9543.32	3845.39	1849.38	3589.99	7250.93	3800	0.83795	-0.43764
ENSG0000C	TRAV10	14:218254	9.60704	17.1116	19.2569	20.8572	18.5638	10.4937	22.3548	7.85841	7.78039	16.2056	0.72495	-0.30276
ENSG0000C	SH3D21	1:3630638	1.14961	0	0.220477	0.697898	0.429743	0.179518	1.37422	0	0.017023	0.751527	0.72415	-0.40436
ENSG0000C	RP11-425	11:463964	2.23894	1.39101	0.965319	0.657092	0.178346	2.50133	2.78524	0.996081	0.526504	0.354402	0.7297	0.569515
ENSG0000C	RP11-167	11:739949	1.87729	1.16927	0	0.561071	0.087909	0	0.056943	0	0	0.066382	0.1815	-4.73817
ENSG0000C	LINC0112	2:9766421	1.33106	6.02043	1.21372	0.456042	0.917084	0.724392	0.810043	1.45453	1.30378	1.34024	0.0221	-0.76916
ENSG0000C	ZNF780A	19:400282	4.65874	5.11376	18.0286	9.9715	6.80181	3.61119	7.60728	18.5637	2.10684	4.63827	0.61675	-0.4749

ENSG0000	AC079807	2:4769552	2.3829	0.91969	0.674722	1.83442	0.090632	0.207562	1.74742	1.46739	0	0.438249	0.67625	-0.55179
ENSG0000	FMO5	1:1471727	1.30992	4.9318	0.467463	1.24346	0.233246	0.784364	0.949225	0.658371	0	0.440766	0.14465	-1.32265
ENSG0000	TRAPPC2	X:1371224	18.6945	23.6607	26.2724	15.9244	16.0306	20.8073	12.9479	22.8652	17.7523	22.4144	0.76875	-0.10462
ENSG0000	RASGRF2	5:8094769	6.98453	4.28362	4.49482	4.39061	4.37036	0.652842	0.13591	2.61158	3.11819	5.83497	0.1484	-1.17239
ENSG0000	JOSD2	19:505059	21.7086	9.36823	29.7397	36.0438	25.4408	23.0652	25.5775	38.5173	32.6983	11.2294	0.9453	0.03463
ENSG0000	ZC3H18	16:885703	12.0234	16.7043	9.77215	4.14112	13.4252	11.4044	11.5813	9.84181	1.41814	20.186	0.877	-0.07275
ENSG0000	FRYL	4:4849736	21.1058	22.9123	9.96527	19.1789	20.1145	22.2891	19.9251	10.0376	31.8849	40.7231	0.40415	0.538111
ENSG0000	ENPP2	8:1195570	0	1.49379	0.052624	0	0.079598	13.598	0	0.05153	0.016368	0.458584	0.1114	2.97689
ENSG0000	IL15RA	10:589020	23.8186	7.95604	37.6347	42.2322	34.0223	33.727	55.9435	31.1263	24.1044	32.9488	0.81935	0.133024
ENSG0000	NFKBIZ	3:1018279	29.6934	38.1908	11.1517	85.2065	10.2103	12.717	13.7422	29.5894	42.4343	16.8496	0.0327	-0.86512
ENSG0000	ANAPC7	12:110372	19.8397	22.1421	21.3666	17.403	15.105	30.2034	33.2934	22.668	6.78211	22.6357	0.86755	0.134319
ENSG0000	BZW1P2	3:1158023	12.0516	6.79509	0.973327	3.52399	0.574234	9.55356	6.07493	1.1224	2.4556	0.271511	0.43035	-0.05099
ENSG0000	GADD45G	19:129458	62.8422	39.2275	90.7621	70.0495	99.4267	74.5482	85.0724	75.1166	53.0943	17.3694	0.59435	-0.30959
ENSG0000	TMSB4XP4	9:1283406	0.226147	0.946166	0.309308	0.211946	0.32149	0.671108	1.15914	0.466068	2.57694	0.503056	0.2793	1.4525
ENSG0000	FAM159A	1:5263334	4.68791	3.11229	6.03533	4.36208	3.62244	4.30643	3.32775	2.61614	1.3883	4.22483	0.6166	-0.4679
ENSG0000	ZBTB34	9:1268606	0.562136	0.680212	0.57434	0.352494	1.19785	1.17629	0.287481	1.33027	1.75512	2.35714	0.26705	0.833277
ENSG0000	ZEB1-AS1	10:311878	6.20773	4.67085	4.20361	6.72166	2.20861	2.15864	3.73904	4.81371	3.39291	3.44661	0.4764	-0.60465
ENSG0000	DBNL	7:4404463	190.649	144.107	218.325	250.819	196.519	172.325	117.699	137.165	198.63	162.485	0.27425	-0.38817
ENSG0000	SRSF7	2:3874359	351.433	370.018	282.49	482.32	198.586	462.943	583.416	333.204	355.934	339.231	0.57845	0.225491
ENSG0000	TAB2	6:1492179	6.69542	12.1093	4.29688	4.79544	6.38234	7.00078	13.7472	11.5162	4.50195	19.0588	0.13035	0.641152
ENSG0000	INTS12	4:1055526	31.0257	32.7322	30.0205	25.9597	24.4722	14.0102	27.8872	18.4034	22.3431	16.9385	0.28855	-0.48308
ENSG0000	RP11-592	15:710969	0.542899	0.281426	0.640475	0.505626	0	0.092651	0.285336	0.273505	0.372304	0	0.5271	-0.98137
ENSG0000	RP11-799	17:352314	6.6986	10.6058	6.91873	8.05216	2.85423	4.65115	5.56588	4.23821	8.23998	3.17759	0.49835	-0.33347
ENSG0000	SMAP2	1:4034484	180.649	324.298	60.8206	43.8433	77.9916	97.2883	80.546	95.0551	67.2247	88.1359	0.10715	-0.54421
ENSG0000	PRR14	16:306507	15.692	19.5199	11.228	10.1425	20.4576	15.7577	26.6103	14.0134	11.9765	22.5031	0.49195	0.285487
ENSG0000	POU2F1	1:1672208	3.85006	8.66041	3.06713	2.0566	4.75213	2.01764	1.57089	4.16099	2.22959	7.43788	0.56655	-0.46281
ENSG0000	SCARNA1(	12:649188	0	1.34203	0.310096	0.429966	1.26805	3.33337	0	0	0	0	0.7789	-0.51602
ENSG0000	AHSG	3:1865384	0	0	0	0	0	0	0	0.386448	5.55041	0.031916	0.12715	inf
ENSG0000	TK1	17:781740	29.1285	41.2703	3.52443	11.0899	7.4312	31.4879	31.3771	21.6648	12.5126	10.8118	0.6323	0.224022
ENSG0000	VKORC1L1	7:6587326	2.57623	2.6444	1.43978	1.08281	1.58683	3.13799	3.4366	2.18129	0.955226	3.62245	0.4757	0.47776
ENSG0000	STRIP1	1:1100315	24.6779	21.48	23.3983	15.4067	14.7192	13.9901	17.9584	15.5789	15.4385	24.0898	0.5854	-0.18439
ENSG0000	ZCRB1	12:422384	31.5602	37.2621	50.3017	67.899	56.9816	45.8091	69.5235	54.149	59.9621	18.6061	0.9793	-0.0126
ENSG0000	ZNF567	19:366876	11.3901	11.3043	6.59374	8.77805	6.43096	4.0601	7.1583	4.1948	4.9753	6.56266	0.11605	-0.72951
ENSG0000	IGHV3-11	14:106116	0	2.01632	0	12.0555	0.17124	0	0	0	0	0.436862	0.1252	-4.42091
ENSG0000	ANKRD36	2:9711349	18.451	29.7714	7.57335	11.2455	6.84409	24.8463	27.6108	10.0615	12.5332	31.7582	0.7014	0.23225
ENSG0000	LRRC29	16:672071	1.94929	2.29182	1.51376	0.659811	1.5308	2.27705	1.56672	0.041919	5.11807	2.3944	0.41545	0.692963
ENSG0000	POLR2E	19:106592	116.224	96.5629	241.576	174.141	247.882	136.909	170.874	171.345	121.648	101.763	0.6114	-0.47921
ENSG0000	POLG2	17:644777	13.421	22.7852	12.8582	10.2638	9.44793	12.9295	16.8538	13.8405	2.9754	15.2547	0.699	-0.18577
ENSG0000	PITPNM2	12:122983	2.1232	1.23733	1.55662	3.76392	0.957654	0.254363	0.825341	1.06449	0.074432	0.515264	0.0286	-1.97282
ENSG0000	RP11-178	18:244893	0	0	0	0	0	0	0	0.667683	6.13861	0.241978	0.0035	inf
ENSG0000	SDC3	1:3086946	0.129392	0	0	0	0.019867	5.42887	0	0	0	0.546066	0.2406	5.49172
ENSG0000	HDAC11	3:1347698	2.26694	0.857666	0.979743	0.408677	1.91421	0.309784	2.97056	2.04511	3.10807	2.35123	0.19015	1.11655
ENSG0000	NPM1P49	X:4743873	0	0	0	0	0	0	0	0	2.23572	0	0.00105	inf
ENSG0000	ING3	7:1209507	21.8384	18.3774	25.9123	46.6186	17.764	33.6179	23.7871	20.412	23.8496	18.484	0.5943	-0.23466
ENSG0000	AC105760	2:2370594	0.485933	0.365699	0.189521	0	0.116492	0.892069	0.514351	1.19068	2.33063	0.670376	0.09445	2.37466
ENSG0000	MTMR9	8:1128349	5.36211	5.87148	4.50376	4.34244	2.76712	8.0213	5.24908	5.76853	3.05316	9.05486	0.6871	0.229548
ENSG0000	AC079305	2:1772275	4.2825	0.954939	0.566394	1.21686	0.309124	0.373865	0.918303	0	1.20976	0	0.37705	-1.55168
ENSG0000	CH17-224	14:106088	0	0	0	12.4862	0	0	0	0	0	0.147841	0.2532	-4.9094
ENSG0000	MCEMP1	19:767662	2.09016	2.00244	0.563851	0.142702	0.209617	50.6512	4.34675	3.9163	0	0.342977	0.0951	3.42895
ENSG0000	GABPB1-A	15:502753	14.2876	21.3703	19.3788	17.3244	16.4358	14.8799	15.4061	16.5784	23.7863	23.3059	0.9577	0.055124
ENSG0000	SLC25A45	11:653674	14.0033	26.3417	34.7651	51.8923	27.034	18.1013	15.1985	21.6201	39.3492	33.8912	0.60525	-0.39594
ENSG0000	NDUFC1	4:1392661	102.894	102.679	148.75	111.335	74.759	140.671	139.317	156.342	68.142	20.182	0.8483	-0.14018
ENSG0000	TYK2	19:103505	100.807	105.274	17.9491	26.7783	23.4298	73.2893	61.2725	26.559	18.934	50.3275	0.7791	-0.10812
ENSG0000	ZNF449	X:1353447	2.25425	3.87415	0.852127	1.36248	0.790248	1.11368	1.2204	0.870505	0.022742	0.914484	0.11125	-1.10079
ENSG0000	TRBV4-1	7:1423131	18.1067	46.1098	21.4	17.9401	8.1329	37.7851	49.7251	5.09293	2.66835	12.8907	0.9582	0.03732
ENSG0000	KNTC1	12:122527	6.8092	10.9549	0.461637	3.26048	1.53962	8.12943	6.33327	1.79707	1.56956	7.81049	0.4901	0.469418
ENSG0000	ALDH3B1	11:680085	0.78215	3.65668	0.654761	0.861131	0.293131	9.55409	0	0	0.180209	0.097815	0.64795	0.611712
ENSG0000	APOBEC3(	22:390140	118.312	82.0466	249.549	198.827	331.724	227.092	222.957	301.787	164.743	417.302	0.50815	0.293804
ENSG0000	KB-1396H	8:1016865	1.78169	1.14432	0	0	1.32292	0.076593	2.71551	0	0	1.28865	0.9783	-0.05189
ENSG0000	RP4-740C	1:2321252	0.216495	0	1.00173	0.188641	0.220408	0	0	1.66644	0	1.64147	0.5686	1.19257
ENSG0000	BFSP2-AS	3:1333999	0.119557	0.117098	0	0	0	0	0.240497	0.948191	1.13209	0.054325	0.21605	3.65695
ENSG0000	TFG	3:1007093	28.6188	33.5836	39.04	45.6215	42.1437	44.7987	29.8148	32.6955	36.8522	38.4704	0.78585	-0.09638
ENSG0000	SLC8A1	2:3978645	0.012919	0.908307	0.153066	0.027827	0.09258	1.71412	0.67591	0.267354	0.688327	0.001689	0.15475	1.43861
ENSG0000	TRMU	22:463308	31.5943	38.9138	30.177	21.96	16.6051	39.7162	34.3541	29.734	23.1505	22.7689	0.83235	0.088478
ENSG0000	AP001505	21:449788	0.502198	0.160154	0.101439	0.38826	0.592705	0	0	0.318087	0	0.13297	0.3514	-1.70222
ENSG0000	ATP6V0A2	12:123712	9.5478	7.01033	8.10291	8.48642	5.20682	9.82007	10.7028	10.5616	6.83993	12.2211	0.7912	-0.12132
ENSG0000	MDM2	12:688081	8.64968	10.9897	7.11174	3.58564	6.29742	6.98607	20.7283	8.3032	5.04879	13.6507	0.55795	0.36725
ENSG0000	CTBP2	10:124917	1.75143	1.39809	1.12062	0.378089	2.41316	3.73061	0.600639	0.923013	0.218342	1.89243	0.5731	-0.48398
ENSG0000	RP11-686	2:2023749	1.09073	1.67972	2.94444	0.445442	0.340845	0	0.620339	3.3218	4.23487	0.518402	0.803	0.537625
ENSG0000	ZBTB17	1:1594186	30.7083	34.9743	15.663	10.6441	12.4579	55.0946	29.1955	14.8396	7.70061	18.7938	0.40205	0.292204
ENSG0000	RAB6A	11:736756	92.5001	73.4857	42.988	43.9742	65.5431	74.5706	69.5379	50.6813	59.1484	61.6215	0.8783	-0.05192
ENSG0000	N6AMT1	21:288721	5.79353	6.50578	6.12355	6.8025	3.27659	4.90489	1.76493	4.82256	7.47425	1.55155	0.6561	-0.33196
ENSG0000	MYH3	17:106285	0.075628	3.56487	0.301686	0.704106	0.296293	1.25561	1.07511	0.871934	0	1.18599	0.6411	0.415674
ENSG0000	TSPAN13	7:1675353	0.21514	3.28562	0.727024	1.37853	0.22386	1.42732	5.38281	0.500404	2.39598	0.239013	0.4996	0.774703
ENSG0000	HNRNPK	9:8396808	458.655	342.169	390.973	500.671	259.37	336.497	292.668	362.283	347.244	218.461	0.35645	-0.35703
ENSG0000	AC114730	2:2417345	0	0.613171	0	0.389322	0.042256	0.195714	0	0.66872	0.544232	0	0.73345	0.738079
ENSG0000	DOCK9	13:987934	9.01781	13.4619	4.71099	6.17326	4.82401	1.6487	3.1747	5.18472	2.07216	14.2715	0.469	-0.33278
ENSG0000	MAPK1IP1	14:550516	34.372	42.4231	46.75	40.4299	34.5756	31.4783	41.6334	48.6535	49.0165	56.9506	0.9646	-0.01497
ENSG0000	TIMM8AP	2:1620773	1.44065	0.184883	2.78405	3.73268	0.461255	4.57796	2.02494	0.994483	3.03836	0	0.8945	0.226475
ENSG0000	PIK3IP1-A	22:312815	0.947204	7.79699	0.096773	1.52396	0.318953	0.271264	2.32268	0.963339	1.52938	1.45211	0.7616	-0.39896
ENSG0000	PATZ1	22:312815	11.9776	6.98736	3.92866	3.07778	7.44636	7.60764	4.47706	2.31	0.0346	10.735	0.6764	-0.42178
ENSG0000	NME6	3:4824109	21.1136	13.552	55.0621	45.134	49.2992	17.3001	16.3832	37.3337	60.0862	45.2129	0.8527	-0.09398
ENSG0000	AGAP4	10:458255	3.16002	7.24318	2.56008	1.18708	2.05366	1.77578	1.84287	3.25911	0.792317	6.97093	0.58635	-0.37546
ENSG0000	PBRM1	3:5249533	44.8361	39.323	23.8932	27.5674	24.456	28.2677	38.1443	18.9777	35.5252	50.8009	0.9952	0.002896
ENSG0000	COG8	16:691055	13.3836	12.4828	5.67992	10.1123	7.2895	4.33824	3.74395	3.24259	4.38805	4.69039	0.38895	-1.12129
ENSG0000	RPL36AL	14:496185	356.862	265.013	678.365	768.581	459.454	446.637	584.366	558.919	455.684	76.8654	0.41135	-0.29047
ENSG0000	NFE2L2	2:1772275	57.6878	70.9595	64.9097	64.9122	67.2414	85.1197	47.9048	51.0916	53.5126	64.7043	0.7203	-0.13118
ENSG0000	SSBP3-AS	1:5413268	0.955187	1.23653	0.624311	0.07831	0.294842	0.362374	0.316528	0.823453	0	2.07598	0.9098	0.181991
ENSG0000	PROCR	20:351720	7.80433	1.96449	6.25987	10.0054	5.55293	12.3396	1.24135	7.06279	2.67109	1.6628	0.78435	-0.40684
ENSG0000	MECP2	X:1540215	18.3925	15.0972	11.2648	13.3526	15.1962	9.75489	13.1839	10.8972	10.983	15.8204	0.39425	-0.48193
ENSG0000	LACC1	13:438239	0.699705	0.293567	0.216466	0.119269	0.276365	3.7424	0.40321	0	0.351375	0.679582	0.2591	1.57301

ENSG0000	TP53BP1	15:433581	6.02331	7.64788	1.99751	1.37564	1.73822	5.51953	5.99116	3.66915	7.43376	5.57952	0.41645	0.434362
ENSG0000	FCGR3B	1:1616231	9.97692	4.3734	0.613663	0.811842	0.409001	4.6682	0.116813	0.518729	0	0.547467	0.15325	-1.57605
ENSG0000	DTD2	14:312486	7.08807	5.53822	3.07721	5.18429	3.72381	2.81763	3.86591	5.31072	7.09852	4.22208	0.98315	0.020783
ENSG0000	FOXRED1	11:126202	13.3208	14.8515	21.4536	25.6576	13.4399	13.9957	16.4486	13.7472	20.1791	9.66482	0.75835	-0.3221
ENSG0000	GPR55	2:2309073	0.460567	1.90476	0	0.711522	0.059897	0	0.066836	3.43901	0	2.77632	0.47955	1.04781
ENSG0000	CNNM2	10:102918	2.65369	1.90311	2.00161	4.43111	1.09537	0.280059	0.511822	1.60461	0.748575	1.91264	0.22935	-1.39323
ENSG0000	CFAP157	9:1275793	0.320832	0.07335	0.527875	0.757018	0.52443	0.036307	0.84407	0.377677	0	0.44903	0.61535	-0.64686
ENSG0000	BARD1	2:2147256	2.28561	2.50642	1.39828	1.72758	3.4949	6.84619	5.62461	8.1767	2.17568	9.26848	0.02205	1.16414
ENSG0000	SETBP1	18:446801	1.0779	1.73127	0.215165	0.250075	0.051066	0.289604	3.14276	0.608877	0	0.638748	0.771	0.397647
ENSG0000	FAM219B	15:748900	19.7176	22.6345	20.9646	16.4538	10.6748	16.9156	15.4787	21.2379	19.3048	22.5516	0.6601	-0.23812
ENSG0000	METTL16	17:240556	7.25305	7.76574	6.04466	3.32876	4.28625	2.8691	7.76631	5.03827	6.18173	5.73583	0.6936	-0.19377
ENSG0000	RPS27	1:1539907	2090.54	1647.27	4340.5	3030.2	1341.77	1683.5	1991.82	2195.1	1623.74	162.339	0.0661	-0.75208
ENSG0000	SMIM12	1:3471273	33.5395	31.7884	55.3604	35.9004	54.2757	42.6522	49.0113	56.2952	46.2397	23.3537	0.8151	-0.1025
ENSG0000	SP4	7:2142803	6.98864	5.90388	6.2196	2.6801	4.76314	2.32066	5.0675	3.84594	0.793164	7.11671	0.2307	-0.51703
ENSG0000	SLC1A7	1:5308717	0.088321	1.80407	0.447873	0.792302	0.016565	0	0	0	0	0	0.0001	#NAME?
ENSG0000	TRPS1	8:1154084	1.61438	2.32991	0.394081	0.190871	0.257466	2.62699	2.67637	0.989289	6.81711	3.1482	0.0455	1.68711
ENSG0000	THRA	17:400190	4.64981	5.89038	2.38034	2.3247	6.03703	5.16502	1.05532	0.995572	0.873682	4.8801	0.4759	-0.76036
ENSG0000	RNF10	12:120503	101.734	118.623	51.127	71.1914	47.3784	70.4735	80.8289	53.7667	60.1516	62.1753	0.59255	-0.19197
ENSG0000	NACC1	19:131181	0.58009	0.709349	0.18956	0	0.967316	0.096901	0.657834	0.043509	0.112377	0.359364	0.34565	-1.18174
ENSG0000	ITCH	20:343632	14.173	6.70446	7.18909	6.88913	8.12137	6.40583	4.27502	4.20568	3.47124	9.43673	0.77925	-0.13884
ENSG0000	FKBP11	12:489034	168.443	36.2831	307.239	342.753	203.004	114.573	119.804	305.163	320.094	124.237	0.74675	-0.15716
ENSG0000	CKAP2L	2:1127214	1.00607	1.77242	0	0.008872	0.070549	3.05445	1.11792	0	2.0111	1.26618	0.2246	1.44977
ENSG0000	XXbac-B4	22:177876	0.388404	1.37724	0.105191	0.16797	0.67344	0	0.516312	0.014162	1.74181	2.01124	0.75785	0.71685
ENSG0000	NAV1	1:2016228	0.005166	1.23697	1.27912	0.026318	0.100863	2.56303	0.134141	0.152808	2.60783	0.047186	0.5144	1.02843
ENSG0000	SOS2	14:501171	9.04346	6.39309	1.96982	1.90525	2.9001	3.92872	2.18124	3.39421	0.22732	7.32237	0.24075	-0.53151
ENSG0000	TRIB1	8:1254303	7.80448	24.0146	0.090461	3.77793	0.131648	7.56326	19.8397	7.26737	0.472173	2.77047	0.77435	0.147013
ENSG0000	RACGAP1	12:499671	6.55754	8.65338	1.40119	7.92465	3.674	11.9791	5.82364	5.7038	7.70229	6.90447	0.22715	0.484151
ENSG0000	DNHD1	11:649725	1.85345	4.10188	1.04545	0.649089	0.990244	2.31301	2.40794	0.91551	3.2152	2.04976	0.29225	0.620031
ENSG0000	PAK4	19:391257	0.441366	0.455229	1.41846	0.520642	1.02691	2.08402	0.233429	1.93165	0.371464	3.46787	0.38105	0.815829
ENSG0000	PHYHD1	9:1289208	0.621148	0.40207	1.13434	1.07632	0.840595	0	0	0	0	0	0.001	#NAME?
ENSG0000	AGAP5	10:736448	0.149032	2.61759	0.46516	0.257445	0.84417	0.128579	1.05802	0.184909	0.014971	2.43296	0.38775	1.37616
ENSG0000	CH507-14	21:631843	6.07902	1.0085	3.82808	1.53789	0.806676	2.7997	2.20888	3.68424	1.82865	0.354138	0.8052	-0.28001
ENSG0000	ANKRD36	2:8876580	0.069111	5.18606	0	2.11427	0.349451	0.029991	0	0	0	0.017419	0.24375	-7.4686
ENSG0000	PDCD2L	19:343594	7.29906	13.1691	15.5298	11.5405	12.1237	10.0623	8.41524	15.4178	9.04398	4.49763	0.81	-0.35191
ENSG0000	ZNF511	10:133278	39.4188	26.288	59.4563	56.1807	47.7399	51.5161	54.0044	40.8464	39.9361	19.7414	0.697	-0.21366
ENSG0000	ZNF487	10:434368	6.84729	5.69592	9.34756	11.2695	3.84534	1.85294	2.09555	2.11911	3.58575	2.46842	0.11965	-1.65481
ENSG0000	COPE	19:188995	208.56	208.817	329.307	273.076	305.291	249.169	298.276	295.988	270.661	104.418	0.6376	-0.15979
ENSG0000	RP11-799	18:239927	8.24811	4.37548	62.8432	33.5251	20.7044	8.97243	10.3068	35.9744	33.8047	31.4251	0.99565	0.008973
ENSG0000	PRNP	20:468623	105.72	45.8472	81.6561	118.046	67.4142	110.747	110.924	99.6354	123.188	80.8321	0.3819	0.283799
ENSG0000	RP4-800J2	20:573841	3.22696	1.39002	3.66693	5.2122	1.82025	0.201136	2.63389	3.06693	3.25554	1.61742	0.761	-0.45886
ENSG0000	PRDX3	10:119167	138.394	145.843	109.281	106.088	69.2007	209.651	189.784	94.7426	117.127	34.8002	0.52985	0.200682
ENSG0000	TPK1	7:1444519	12.1618	14.8553	9.17586	10.0331	6.91926	9.07231	9.16512	4.6123	0.679852	5.34925	0.25035	-0.89548
ENSG0000	LRRC41	1:4624768	29.2609	24.129	28.9208	30.0237	28.346	33.1891	21.8994	26.4114	10.4429	33.6129	0.5898	-0.20691
ENSG0000	EIF2B1	12:123602	47.6154	52.8385	35.798	52.5267	35.3342	44.8188	38.6242	24.1223	34.4094	37.7193	0.42905	-0.31024
ENSG0000	PDP2	16:668785	1.05687	0	0.849001	1.42282	0.687694	0.149719	0.730767	0.747802	0	1.98454	0.43805	-0.84594
ENSG0000	MCUR1	6:1378655	6.06581	5.45982	4.01377	4.13047	3.02085	12.2078	10.9806	7.3768	3.48096	2.23308	0.50915	0.415363
ENSG0000	MORC2-A	22:309223	2.04457	0.074286	7.51113	9.63342	7.16898	0	0.042658	1.38176	4.50079	4.85461	0.40795	-1.33643
ENSG0000	GATS	7:1001775	4.85226	3.74926	2.12963	0.52752	3.30581	1.60821	1.77599	2.41059	0.201308	3.29515	0.45275	-0.77163
ENSG0000	GP6	19:550061	0.462205	0.401325	0.014374	1.80562	0	0	0.05365	0.231968	0	0	0.0528	-2.86183
ENSG0000	RP11-6N1	17:478912	1.76516	1.50172	4.55175	8.33402	2.46673	0.688502	1.86453	3.19889	1.22523	0.717392	0.42335	-1.32025
ENSG0000	HNMT	2:1379640	0.433289	5.43202	0.027054	0	0.117944	24.8744	0.315495	0	0	1.75898	0.17625	2.3147
ENSG0000	CLCN7	16:144493	27.1709	25.8639	4.47026	6.91283	9.92169	24.4262	27.2493	15.5391	5.68907	17.0832	0.9484	0.025613
ENSG0000	PDSS1	10:266976	5.06799	5.75302	3.15499	4.67148	2.29289	7.4438	7.73471	2.76735	5.79221	3.15412	0.6583	0.391838
ENSG0000	UBXN10-/	1:2018424	4.89815	0.603506	8.46101	5.34423	2.60137	0	0.390203	4.45859	10.1814	0.204602	0.84045	-0.41902
ENSG0000	GABARAP	12:102124	23.1819	10.4078	18.9379	18.0681	20.9525	14.6934	49.3773	30.1874	29.0956	33.903	0.0433	0.701701
ENSG0000	AP000866	11:124766	0.162576	0.480497	0.23516	0.130445	0.765527	0	2.10012	1.05015	3.6727	1.68853	0.2753	2.18944
ENSG0000	bP-21264	21:932501	4.37361	1.55472	1.52374	1.15669	0.312425	3.23928	1.45434	1.57431	0.11153	0.866088	0.90065	0.137705
ENSG0000	WARS	14:100333	31.9677	61.1854	35.8618	31.37	26.2876	131.729	127.568	86.3079	48.4167	36.2776	0.00075	1.11719
ENSG0000	SLC11A2	12:509794	9.15014	20.4312	5.79091	10.1898	3.70791	9.94307	11.0721	4.46412	11.2509	6.77401	0.56245	-0.21082
ENSG0000	LY6G5B	6:3166122	2.00455	4.58194	1.49063	1.71759	0.658473	4.57328	4.31015	1.71874	2.75996	5.09643	0.35075	0.798348
ENSG0000	SNAI3-AS	16:886632	2.25602	2.15122	2.76459	2.90325	2.24816	3.96936	0.225848	1.42101	3.75899	3.43166	0.9415	0.067305
ENSG0000	RER1	1:2391774	60.7515	61.8863	60.7514	48.951	54.8869	97.6758	100.197	82.5425	70.9798	48.5069	0.3028	0.410397
ENSG0000	C6orf120	6:1697021	9.09228	10.4856	5.90802	3.81544	5.79507	10.5352	8.13739	7.48034	5.79013	8.80186	0.7446	0.233267
ENSG0000	CBFA2T3	16:888748	0.365596	2.31842	0.03041	0.03384	0.307154	0	0	0	0	0.14575	0.23035	-3.9801
ENSG0000	HCFC2	12:104064	11.8651	8.61699	6.54394	7.97835	5.75538	12.1368	9.53983	4.15196	2.23885	10.9134	0.71245	-0.17261
ENSG0000	RP11-148	8:1149400	1.66204	0	0	2.04327	1.56174	3.17387	0	0	3.71259	1.81122	0.7129	0.711594
ENSG0000	SLC43A2	17:156926	1.98271	5.44192	1.52504	0.665767	1.04523	4.64237	2.37142	0.426642	0.221792	2.40895	0.65535	-0.35044
ENSG0000	H3F3A	1:2260607	576.678	722.694	615.905	527.641	635.798	741.476	925.171	565.526	480.806	217.57	0.7019	-0.14691
ENSG0000	NPM1	5:1713871	267.332	221.546	360.231	359.255	247.463	353.351	409.397	355.888	378.116	91.2003	0.82515	0.091628
ENSG0000	PLLP	16:572450	0.097005	8.3607	0.05772	1.67596	0.800394	0.326468	0.181947	0.405771	0.831096	0.449868	0.2815	-1.84665
ENSG0000	RP11-286	11:614026	24.5399	23.5555	30.0268	30.8294	21.7212	26.9811	24.0771	20.7041	9.58258	6.64673	0.51495	-0.59339
ENSG0000	HIST1H4H	6:2627760	2.94494	1.16097	4.88358	7.27173	1.01412	8.49732	2.91301	3.0344	5.67698	1.00845	0.8127	0.256191
ENSG0000	FYCO1	3:4591789	7.9751	2.15009	4.60317	3.32061	5.05219	6.77689	8.06242	6.63012	7.09151	14.9969	0.37135	0.834898
ENSG0000	EIF4H	7:7417424	151.132	168.367	136.296	114.432	102.72	150.558	132.215	116.92	97.2571	138.484	0.7766	-0.0925
ENSG0000	AHCY	20:341945	10.4156	18.3399	8.67501	12.6436	9.70822	15.5755	11.485	10.9641	7.47792	8.72109	0.6865	-0.16809
ENSG0000	ZNF444	19:561325	18.4859	28.3804	13.8349	8.97192	18.4513	32.8286	21.0173	14.0107	14.6211	12.2466	0.8597	0.080503
ENSG0000	AHSP	16:315278	0	0	0	8.06936	0	0	1.07441	0	0	0	0.49335	-3.33805
ENSG0000	RAN	12:130871	220.754	236.187	244.595	246.638	233.443	288.479	334.859	284.493	217.764	115.759	0.9672	0.01356
ENSG0000	MAP3K10	19:401917	1.80369	0.140654	0.592116	0.086152	1.07676	5.24231	1.0481	1.28668	0.290796	1.81863	0.1245	1.28778
ENSG0000	ZNF274	19:581830	28.268	26.2995	18.3821	20.5544	9.9465	14.6288	15.524	17.329	19.9963	17.7762	0.532	-0.24058
ENSG0000	SHB	9:3751089	0	0.499172	0	0	0	0	0.336295	0.591231	2.04798	0	0.33905	3.2551
ENSG0000	ZCCHC7	9:3712053	16.6106	14.5178	20.6483	24.2628	17.4265	20.232	13.6976	16.8332	13.9544	16.9037	0.63895	-0.18275
ENSG0000	AC022182	8:6091005	1.29469	1.0972	0.231807	0.65791	0.276301	0.088875	1.03939	0.078648	1.28331	1.19148	0.78025	0.442407
ENSG0000	ZNF140	12:133079	22.6883	15.7922	11.114	16.0957	11.06	17.3124	16.1422	10.9572	4.07288	15.1783	0.4752	-0.28919
ENSG0000	RP11-115	11:126355	0	0	0	0	0	0	0	0	3.58248	0	0.0522	inf
ENSG0000	SRSF3	6:3659435	203.656	161.098	215.772	208.633	160.613	208.046	219.61	188.335	165.957	151.227	0.71605	-0.18978
ENSG0000	ZMYND12	1:4243032	0.455677	0.098842	1.42891	0.77936	0.140972	0	0	0.087916	0	0	0.0777	-5.10262
ENSG0000	TIPARP-A	3:1566718	2.52241	1.54943	8.21095	5.18504	0.815695	2.01824	0.481967	6.23967	3.63582	1.17856	0.82325	-0.32049
ENSG0000	LY6E	8:1429817	183.821	179.185	139.437	140.027	160.28	185.118	642.972	237.082	103.394	98.1847	0.2167	0.481047
ENSG0000	EPB41L3	18:539238	0.259341	6.45724	0.036634	0	0.087639	4.53871	0.227527	0.391306	1.5577	0.420183	0.651	0.329175
ENSG0000	IGKV3-20	2:8914257	0	17.0006	11.3087	40.9528	253.307	0	0	0	0	0.638136	0.0629	-8.6924

ENSG0000	CD2AP	6:4747778	8.0763	9.90434	2.39008	4.65116	4.37752	9.55537	3.95323	4.34865	3.73702	5.9754	0.74575	-0.14266
ENSG0000	RP11-756	4:1887383	0.850458	0.810616	0.336868	0.025598	0.088058	1.30871	1.03924	0.182763	0	0.027005	0.8022	0.405517
ENSG0000	GALM	2:3866590	62.5164	39.0466	66.7339	81.0443	52.6876	129.99	110.34	70.8577	64.0897	80.4884	0.1087	0.514636
ENSG0000	IL12RB1	19:180589	42.3126	17.2139	48.3319	66.3658	47.5795	38.387	37.0386	63.0371	45.8728	50.5134	0.92535	0.044367
ENSG0000	AC015849	17:355747	0.377257	0.161619	0.199281	0.52749	0.291534	0	0.292283	0.106301	0	0.121965	0.34945	-1.61807
ENSG0000	ZNF497	19:583451	0.539788	2.08691	0.645467	0.666165	0.439649	0.685128	0.652246	0.501485	0.085307	0.460633	0.4662	-0.96215
ENSG0000	NPDC1	9:1370072	57.1801	17.8845	37.4748	34.4352	63.1909	43.3774	20.1593	30.9002	32.4699	26.2021	0.3706	-0.52247
ENSG0000	INTS4P1	7:6514052	1.06149	2.30694	1.71875	2.25281	2.04254	0.950748	1.24462	1.03244	1.51838	2.70007	0.987	0.028554
ENSG0000	NOP9	14:242998	1.56238	0.536402	1.0294	0.501523	1.71257	0.546866	1.65895	1.70387	0.540833	3.03684	0.80225	0.298743
ENSG0000	TRAV1-2	14:216428	206.414	24.3799	684.9	610.785	528.997	117.897	33.2221	607.775	687.091	505.291	0.79475	0.102898
ENSG0000	COG5	7:1071689	16.8614	22.1034	8.54428	9.81021	5.99936	15.3272	15.0601	12.5132	9.52972	13.5587	0.82995	-0.12365
ENSG0000	RP11-144	10:474962	1.64711	14.065	1.22015	4.07707	0.829996	0.595779	8.74903	0.573327	4.52199	2.66226	0.94485	-0.09232
ENSG0000	MTMR10	15:309038	5.53114	8.13604	1.2058	1.12934	1.75221	4.28267	2.73095	2.69685	5.872	2.97308	0.66255	0.231973
ENSG0000	HIST1H4K	6:2783121	12.8333	4.85569	15.2862	14.4696	6.02505	11.4038	13.6589	5.6019	16.3231	1.64004	0.93585	-0.1158
ENSG0000	MOB4	2:1974865	24.7215	20.5736	26.4275	23.7104	14.3381	22.0951	39.4639	32.9392	16.4935	11.2127	0.8848	0.106462
ENSG0000	MIS12	17:547125	16.1339	14.3726	12.345	21.9762	10.1389	10.493	16.7127	13.8114	8.7263	10.7005	0.7716	-0.28316
ENSG0000	PRC1	15:909660	9.08761	9.38249	2.58936	2.71383	0.788576	6.1954	19.8356	7.99155	29.2989	13.9053	0.0037	1.97987
ENSG0000	ABHD10	3:1119790	18.4126	20.7886	21.6316	21.8949	10.8136	17.6824	13.0242	9.92006	24.7305	10.3316	0.2711	-0.41031
ENSG0000	MRPS31P5	13:521677	2.99276	4.1421	4.39083	10.3946	4.66282	2.27261	2.84042	11.2054	0.975075	3.97862	0.45865	-0.4255
ENSG0000	ZNF286B	17:186350	1.95416	2.76593	2.4309	1.44698	1.68322	0.61239	1.56499	1.53579	4.64343	3.10787	0.6752	0.340159
ENSG0000	MEF2C	5:8850754	0.830864	8.5417	1.33873	3.87498	1.05262	3.70916	0	0.132587	1.44652	1.07085	0.0392	-1.51808
ENSG0000	ANKRD39	2:9681624	29.8792	19.8647	48.4494	41.8158	30.6233	41.7503	26.8058	34.628	22.9314	25.1974	0.65665	-0.27389
ENSG0000	RP11-849	18:790692	0.209359	0.621898	0	0	0.113592	0	0.764536	0.153356	5.24538	0.743984	0.1495	2.99542
ENSG0000	SPINK2	4:5680985	0.567343	4.12943	2.63105	2.05307	0	1.34474	1.40008	0.439349	3.19102	0	0.6114	-0.5482
ENSG0000	RP1-199J3	6:9954237	1.78129	1.19102	0.199067	0.83458	0.545994	3.33221	1.53293	0.675045	2.30951	2.87569	0.3573	1.26644
ENSG0000	RP11-456	17:481334	0.423806	2.02703	0	0	0.129848	0	0.392176	0	0.364574	0.250249	0.59155	-1.15482
ENSG0000	SNW1	14:777080	72.1252	94.4597	87.1111	77.8095	114.789	87.7398	81.7488	76.0344	85.9778	78.892	0.75615	-0.12149
ENSG0000	CASC8	8:1272898	5.80207	0.231186	8.36778	6.55611	2.70365	0	0	0	0	0	0.0084	#NAME?
ENSG0000	CGRRF1	14:545098	17.7619	11.1745	24.2684	24.6733	8.75054	17.7463	12.2393	15.4527	8.26101	5.0677	0.17805	-0.57576
ENSG0000	FAM118A	22:453089	37.8695	86.4944	13.9951	19.5873	7.45268	73.835	61.7929	12.9085	17.4248	16.1636	0.7624	0.130504
ENSG0000	B3GALNT2	1:2354499	3.13335	7.9255	1.09391	0.893978	1.12224	0.517452	1.11478	1.22093	5.87514	1.81377	0.5814	-0.3866
ENSG0000	SNX15	11:650141	5.79736	4.04419	3.18169	2.37562	4.96166	4.56502	5.7097	5.54719	3.64012	4.4348	0.82415	0.271676
ENSG0000	ZCCHC8	12:122471	19.4916	20.5069	10.8008	9.18306	7.50649	13.7673	21.8011	14.0356	7.06086	16.1871	0.5715	0.22973
ENSG0000	RP11-378	2:2183982	0.759991	0.977551	0.997583	1.21409	0	0.338896	0.209889	0	3.12026	0.156426	0.87645	-0.26948
ENSG0000	P2RX4	12:121209	25.3185	21.7706	21.9626	37.703	24.1532	61.8324	24.3187	25.2238	40.3098	20.7707	0.34415	0.354764
ENSG0000	GORAB	1:1704604	10.6219	14.5631	7.29089	6.36729	6.97987	4.38769	8.54137	5.07681	12.0639	7.35782	0.92245	-0.05806
ENSG0000	RP11-423	12:108778	0.291033	0.384051	0.313724	0	0	0	0.269373	0.366519	3.52389	0.282302	0.3646	2.37863
ENSG0000	GATSL3	22:302851	9.22879	23.2161	19.7101	13.6474	18.0474	14.9006	7.98057	11.0621	9.37343	12.7938	0.5633	-0.51778
ENSG0000	PPM1G	2:2738119	61.969	60.3714	62.4293	36.7565	63.4003	78.0473	83.2828	58.8653	32.5406	56.5397	0.79375	0.08377
ENSG0000	XCL2	1:1685407	4.91359	2.45576	15.6241	3.18868	3.35488	25.4101	111.257	100.08	30.482	10.9286	0.0001	3.14575
ENSG0000	HIST1H4F	6:2624042	2.64656	0	3.99697	3.60267	0.464161	9.15916	2.1706	0	2.1655	0	0.9415	0.115146
ENSG0000	B4GALT1	9:3310408	5.41428	4.22075	59.6865	42.1796	47.6111	1.00724	2.47236	28.7242	24.7121	50.9688	0.07765	-0.78119
ENSG0000	MOB2	11:146945	37.5603	28.8206	16.1818	15.6803	18.3721	18.6533	24.8812	11.3257	14.9232	9.40732	0.13195	-0.6194
ENSG0000	RP3-508I1	22:387057	1.43027	0	0	3.1883	0	0	0	0	0	0	0.87705	-0.24144
ENSG0000	SREK1	5:6613997	26.9725	38.0577	24.6746	19.5649	38.1462	43.7669	36.1599	22.28	30.0473	58.1827	0.63095	0.225262
ENSG0000	UQCC2	6:3362036	34.9818	24.6231	41.8822	40.3035	14.6772	52.5199	54.423	38.4929	43.1906	13.2246	0.7591	0.246873
ENSG0000	CCL23	17:360130	0	0	0.20287	0	0	4.61554	2.51409	2.0786	0	0.149551	0.2522	5.22798
ENSG0000	EIF5A	17:730699	168.772	110.775	286.832	252.37	291.103	101.193	114.697	247.69	183.161	127.989	0.35025	-0.54287
ENSG0000	ANAPC2	9:1371747	4.81752	5.85174	11.289	24.8445	9.64582	8.28617	5.6196	5.17073	18.2306	18.4857	0.9303	-0.04346
ENSG0000	DCUN1D4	4:5184299	11.0384	8.14559	4.48601	3.62924	3.22384	6.98381	7.23416	5.85814	2.44315	6.1771	0.54785	-0.24354
ENSG0000	USP36	17:787873	21.9004	17.3815	13.7274	14.1179	10.1633	49.6342	21.4607	21.6194	35.591	26.5906	0.0842	0.664905
ENSG0000	GZF1	20:233621	2.74124	2.23352	0.933315	3.42438	1.45604	3.63964	1.2987	2.77807	1.85244	1.60942	0.91345	0.06679
ENSG0000	VIPAS39	14:774266	6.60296	9.28124	10.2111	7.27963	5.4585	6.7813	2.66458	4.18181	0.554114	8.25044	0.3784	-0.81101
ENSG0000	RP11-651	3:1497611	2.40141	2.01937	1.62574	1.333	0.435879	1.86463	1.48922	0.73935	2.3125	0.320825	0.912	-0.16567
ENSG0000	HAUS2	15:425488	9.39933	9.23574	8.26202	13.2373	6.19207	14.3246	8.19297	5.51971	7.57	8.69923	0.7666	-0.17207
ENSG0000	ADAM20P	14:704258	0.321209	0	0	0	0.026726	1.91852	0	0.584269	0	0	0.2573	3.1399
ENSG0000	CACNA2D	12:179195	20.3639	4.99318	10.9386	17.4481	4.93416	17.7324	11.9365	12.1071	11.3707	6.61059	0.962	-0.02656
ENSG0000	RP3-486I3	6:1162541	0.374777	2.16342	0.117974	1.11595	0.459803	0	0.111787	0	0	0.073963	0.2386	-5.2301
ENSG0000	CX3CR1	3:3926349	16.227	26.5721	28.1438	15.2719	61.84	1.14964	1.94988	0.404031	0	3.06723	0.00815	-4.79326
ENSG0000	KPNA5	6:1166811	3.06457	7.58956	1.97652	3.10357	1.99154	3.87683	4.07598	1.13303	1.30495	3.4527	0.3889	-0.66083
ENSG0000	RP1-151F	6:1629911	1.87531	1.74399	1.14047	1.34529	1.78735	1.42047	2.9643	1.76671	1.92059	1.98168	0.71285	0.356751
ENSG0000	BBS4	15:726861	13.3871	9.23645	8.12366	13.5148	7.18717	14.6796	14.0793	6.52268	7.79553	9.51734	0.7015	-0.15008
ENSG0000	EXOSC1	10:974361	48.8736	44.1504	71.6286	66.9656	52.3201	79.1825	68.0168	74.1822	60.7173	27.5503	0.7973	0.097917
ENSG0000	PDE1B	12:545493	1.03542	0.897412	0.414347	0	0.278695	4.25925	2.52349	1.12127	0.233911	0.73131	0.0757	1.77354
ENSG0000	PLCD1	3:3800749	15.5918	12.0703	20.1728	16.3843	12.7035	9.02935	9.86371	16.2097	47.6038	13.8941	0.2065	0.473534
ENSG0000	CPT1A	11:687546	3.54959	4.07341	1.34995	1.34221	4.92986	4.66777	4.45574	3.64637	0.888172	6.50683	0.62445	0.263585
ENSG0000	RP4-647C	14:732374	0.207436	1.34543	0.346764	0.279898	0.173233	0	0	0	2.91739	0.337005	0.74075	0.736772
ENSG0000	GSG2	17:371462	0.308809	1.18747	0.106988	0	0.034844	1.08144	4.62755	1.1015	0.394166	1.55103	0.1865	2.53585
ENSG0000	RP11-100	15:723982	5.44782	1.46601	0.453207	0.11739	0.369655	1.36539	0.236419	0.116458	2.56834	0.421639	0.77025	-0.54202
ENSG0000	ZNF672	1:2488382	15.871	20.3338	8.06289	10.745	13.4928	12.4287	17.0865	13.1666	11.9407	20.0515	0.69685	0.144225
ENSG0000	ZC4H2	X:6491580	3.99256	2.82197	3.55382	4.83678	3.55062	3.02829	0.625069	2.18516	3.00095	1.15958	0.16805	-0.97372
ENSG0000	ERCC4	16:139201	5.51753	8.28545	11.6043	6.79817	9.5898	9.55831	2.56588	9.30726	1.99706	16.0483	0.49655	-0.46955
ENSG0000	SNORD3A	17:191880	277.941	25.0832	16.3371	7.03272	1.81879	9.77861	5.51461	9.73839	14.9738	0.815703	0.161	-2.25997
ENSG0000	RP11-841	14:526399	6.55166	3.85975	11.5325	1.77232	4.85008	4.32229	2.88162	8.05034	5.70816	4.64488	0.93405	-0.11727
ENSG0000	RP11-761	15:811595	1.60358	2.4761	0.972264	0.261215	0	0.705089	0.398629	0.107506	0	0.361802	0.2928	-1.62068
ENSG0000	KIR2DL1	19:547244	0.029505	1.65323	1.15808	5.69095	0.327958	0.205117	4.2807	0.040848	0	0.073237	0.51375	-1.22392
ENSG0000	ZNF91	19:233049	8.96679	9.43881	6.23336	2.95534	5.24401	9.46726	5.66604	5.60504	8.4261	10.5382	0.58255	0.235625
ENSG0000	ELF2	4:1390157	21.4998	21.1782	19.0146	27.7923	21.7294	19.912	18.6629	12.5914	20.9483	22.8707	0.50345	-0.31389
ENSG0000	PLP2	X:4917192	49.4152	63.1289	41.5873	65.2829	85.7252	122.436	219.642	314.291	216.195	122.094	0.00005	1.7289
ENSG0000	WDFY4	10:486848	0.112819	3.06894	0.180188	0.159176	0.019568	2.00646	0.640746	0	0	0.069577	0.9432	-0.10811
ENSG0000	ARHGEF3	3:5672741	119.25	107.237	51.999	68.6337	75.1537	66.1096	62.8074	62.2669	65.4381	116.633	0.62175	-0.18866
ENSG0000	CTD-2233	19:469186	0.637768	0.4256	0.221512	0.029057	0.087779	2.60893	0.582547	0.33071	0.260893	1.80363	0.2195	1.95354
ENSG0000	KLF2	19:163248	156.268	365.028	116.28	154.997	305.864	24.4979	18.2852	33.8527	47.6575	45.0158	0.00005	-2.64632
ENSG0000	MAML2	11:959765	2.04375	6.82486	0.576605	1.02486	1.16586	1.56695	2.21231	0.98119	0.863182	1.6911	0.4277	-0.49821
ENSG0000	CTD-2521	19:174056	0.612475	0.39559	0.118072	0.303173	0.121182	0.613393	0.922885	0.329058	0	0.208271	0.7418	0.453045
ENSG0000	PTGER2	14:523143	81.783	25.6799	76.6907	156.543	98.7381	15.9087	22.8692	26.2827	33.6672	32.061	0.00005	-1.82284
ENSG0000	CENPK	5:6551776	31.8724	60.6502	17.6922	25.0444	9.60059	20.0578	37.9674	17.9739	12.1189	11.9395	0.3041	-0.4047
ENSG0000	TOLLIP-AS	11:130976	4.02627	6.86464	6.20384	5.16205	7.54086	4.47374	7.43476	5.14052	1.31193	2.12785	0.3359	-0.6993
ENSG0000	RP11-326	4:1845064	1.55522	0.103019	0.583972	0.310757	0.666182	0	0.224963	0.207031	2.26786	0.991499	0.82275	0.396953
ENSG0000	REEP3	10:631672	6.54449	4.89543	8.51952	6.51566	6.17258	6.62609	5.06109	11.9281	16.5767	10.9663	0.415	0.525456
ENSG0000	UNC5CL	6:4102691	1.24571	1.06048	0.600716	0.416409	0.618744	0.781277	1.14355	0.624673	0.162504	1.22596	0.96085	-0.06252

ENSG0000(	RP4-778K	7:1050134	1.55007	1.75949	1.21467	1.73972	0.778427	2.87773	1.58423	0	0	0.413092	0.6621	-0.6354
ENSG0000(	SPHK2	19:486153	8.48903	12.2786	7.92024	11.127	6.16528	4.97618	9.00072	8.70277	2.83053	10.3449	0.4377	-0.54166
ENSG0000(	ZNF593	1:2616951	18.6013	17.0115	27.4058	31.059	18.0787	16.7539	46.7881	36.0065	21.0777	3.97028	0.95565	-0.03351
ENSG0000(	SAP25	7:1005722	3.52727	15.5743	0.500986	0.530156	0.065556	6.32528	3.0024	0.05283	2.97138	0.535277	0.743	0.344417
ENSG0000(	DLD	7:1078909	46.1131	37.4273	40.0119	44.9524	34.3311	60.47	37.5944	36.7765	24.2736	24.8381	0.56295	-0.20597
ENSG0000(	CREM	10:350980	42.4168	20.4023	72.2075	155.225	54.5653	197.724	344.472	255.333	409.076	42.2067	0.00005	1.75657
ENSG0000(	SCARB1	12:124776	1.85087	2.43273	1.15564	1.43516	1.71871	17.2656	1.41112	1.64411	0	2.67251	0.1154	1.40135
ENSG0000(	MCCC2	5:7158728	17.2361	22.8982	9.82429	14.6946	5.76214	17.3862	12.9971	7.39883	8.97505	12.1029	0.35645	-0.3513
ENSG0000(	ARL2	11:650141	40.0014	36.0961	76.3915	58.5478	95.0151	67.4738	72.3911	56.0991	20.3196	35.0808	0.2912	-0.43085
ENSG0000(	RBMS3	3:2834914	0.977767	4.22852	0	0	0.025734	0	0	0.143352	0	0.442728	0.04555	-2.58297
ENSG0000(	GINM1	6:1495662	15.8174	13.3775	11.683	19.7391	11.6489	18.6216	15.2897	13.3846	26.3843	8.45927	0.6583	0.165369
ENSG0000(	FAM65B	6:2479732	200.771	255.209	105.434	160.636	116.871	23.4527	10.4847	19.2514	41.7599	49.9274	0.00005	-2.50802
ENSG0000(	CUEDC2	10:102423	64.4223	51.1915	71.7709	58.294	66.657	75.2664	76.9798	68.0131	43.3489	35.1604	0.74475	-0.10868
ENSG0000(	POLR3GL	1:1459113	106.513	80.979	130.717	120.945	181.168	81.252	84.3043	106.767	97.5184	137.398	0.67885	-0.29474
ENSG0000(	RPL4	15:664980	365.961	431.252	742.583	442.314	755.691	468.55	358.718	543.192	430.511	158.153	0.31615	-0.41588
ENSG0000(	LAMB2	3:4912111	0.264351	0.913933	0	0	0	2.6539	0	0	0.164233	0	0.2006	2.44994
ENSG0000(	ZNF383	19:370078	6.46171	5.19871	7.02275	5.88065	5.04155	5.26173	7.91604	6.25034	17.0893	7.22573	0.5421	0.520506
ENSG0000(	RHOF	12:121712	66.6989	63.2744	98.7009	89.6639	122.684	77.0441	82.3794	73.439	75.3064	115.287	0.7035	-0.13918
ENSG0000(	WSB1	17:272940	58.1134	49.1677	91.4348	80.7866	45.0436	61.6761	52.1716	74.4358	100.55	79.0836	0.43925	-0.30987
ENSG0000(	FYN	6:1116603	393.04	403.117	395.716	277.613	248.486	345.103	438.963	305.543	257.742	296.399	0.63395	-0.16505
ENSG0000(	COL6A4P2	3:1302733	0.933091	3.55916	0.225868	0	0.145304	0	2.03294	0	0	2.58686	0.4887	-0.92474
ENSG0000(	LINC0113(	1:2032987	0.715969	0.026037	1.04839	0.850163	0.174865	0.397773	0	0.129789	2.84643	0.160284	0.66815	0.712105
ENSG0000(	HDAC4	2:2390481	7.63136	13.6688	2.13436	6.55774	3.31525	14.7401	4.72968	4.89898	7.14289	4.14659	0.8097	0.123491
ENSG0000(	GINS1	20:254077	0.781718	2.13075	0.061852	0.272005	0.21721	0	1.13528	1.42969	0	0.795815	0.9722	0.049054
ENSG0000(	C21orf58	21:463001	0.772119	3.85318	0.356089	0.342457	0.347399	3.07	3.64761	0.778723	0.300273	2.54902	0.32105	1.00244
ENSG0000(	RMDN3	15:407358	26.4033	19.1227	46.5332	41.8643	36.0352	26.5401	28.4459	21.8029	10.6481	31.3171	0.0712	-0.6286
ENSG0000(	NPIPB14P	16:699762	3.50196	9.0106	0.658884	3.67786	0.236226	1.52097	1.97075	0.912313	0.754456	0.393048	0.10795	-1.70387
ENSG0000(	FAM66B	8:7301610	0.525374	1.4605	0.237845	0.541454	0.335301	0	0.312069	0.008128	0.162702	0.375378	0.28795	-1.88513
ENSG0000(	FAM208A	3:5655716	21.2032	17.0014	10.9788	4.36022	8.81609	19.1797	19.4326	11.2844	19.1436	21.0061	0.33415	0.651825
ENSG0000(	SMDT1	22:420796	88.3739	103.112	122.775	166.318	46.3814	99.4608	104.489	119.974	142.722	14.2875	0.79365	-0.15411
ENSG0000(	RP11-5C2	7:1549284	0.648007	0.928124	0.116748	0.13909	0.888828	0	0	0.1239	0	1.9393	0.8758	-0.27068
ENSG0000(	GFPT2	5:1803006	0.4852	0	2.05628	1.84414	0.725538	0	1.53371	0.328177	1.96667	1.175	0.84995	0.220277
ENSG0000(	CD180	5:6717961	0.131623	1.02274	0.243689	0.902711	0.152797	1.00946	0	0	0	0.021	0.30625	-1.3937
ENSG0000(	RP11-629(	11:112959	0.07816	0	0.180087	0	0.22141	1.92983	0.478171	0	0	0	0.317	2.13399
ENSG0000(	CARM1	19:108715	2.59323	1.17206	1.04467	0.65607	2.71042	3.01963	1.50982	1.81516	1.24289	3.14938	0.85675	0.151531
ENSG0000(	VDAC1P8	6:1434508	1.4348	2.80248	1.91245	1.02558	0.398738	5.12781	1.98059	0.655837	5.06936	2.29363	0.2268	1.01583
ENSG0000(	CYB5B	16:694245	76.8521	69.1045	91.3608	74.8687	64.8555	81.2076	81.7729	66.0052	78.1891	48.5272	0.62925	-0.17147
ENSG0000(	EXOG	3:3849612	9.30059	15.0642	8.90583	15.7216	5.40273	13.8619	12.9999	10.301	20.8309	14.5608	0.71025	0.431681
ENSG0000(	ATP1A3	19:419665	0.53549	0.158833	1.28327	1.4291	2.13923	0.055858	1.29545	0.854095	1.69789	2.58339	0.80065	-0.18553
ENSG0000(	GSTK1	7:1432440	316.883	430.979	306.668	346.536	338.046	482.858	603.944	382.299	265.736	256.618	0.64445	0.157218
ENSG0000(	NETO2	16:470777	9.98725	3.02224	1.21407	1.76413	0.73389	18.7005	16.8016	4.37432	0.928157	5.99814	0.01455	1.63556
ENSG0000(	KEAP1	19:104861	39.0175	23.9957	37.87	45.0332	30.632	23.5219	39.1195	25.6984	32.0562	37.5954	0.51175	-0.20827
ENSG0000(	PPP2R1B	11:111602	15.9168	14.3278	6.20091	12.0676	6.21485	11.3527	5.87782	6.92839	5.05344	7.64179	0.05485	-0.84562
ENSG0000(	GRK3	22:255648	1.83658	1.72037	0.081638	0.147907	0.082373	2.92806	0.244671	0.34254	0	0.396494	0.85085	0.239494
ENSG0000(	ZNF836	19:521130	7.02803	8.32097	10.7488	5.86748	7.45119	5.94636	8.74908	5.8929	0	7.907	0.62725	-0.57001
ENSG0000(	LINC0112(	1:585988-	4.02913	7.47651	6.32714	5.37022	4.53007	0.325411	2.49157	6.21407	5.61887	6.10876	0.69505	-0.30147
ENSG0000(	HNRNPA1	3:3933497	1.64018	2.8303	0	0	0	2.48161	43.0003	2.43988	0	0	0.03415	3.56113
ENSG0000(	BRI3	7:9825237	6.72903	4.06786	16.5089	16.948	20.6763	11.4418	10.6431	25.1118	16.752	9.71677	0.8853	0.102873
ENSG0000(	FAM206A	9:1089341	10.7394	13.6137	11.4924	13.8259	4.70675	12.2177	3.13766	8.08504	6.32768	6.5198	0.21455	-0.57747
ENSG0000(	TSG101	11:184558	67.2761	46.7234	86.4709	59.3688	57.9979	86.8092	108.593	83.5105	53.5042	43.6773	0.73215	0.116607
ENSG0000(	RP11-244(	1:3497435	1.96653	1.86286	6.75475	7.86114	3.31467	1.9915	2.38154	2.98771	0.019737	3.70751	0.32945	-1.25897
ENSG0000(	BAD	11:642698	59.9144	31.1445	80.5442	92.2521	76.4873	54.1596	63.3361	54.8537	62.0195	23.3609	0.22385	-0.49184
ENSG0000(	RP11-839(	11:113278	0.085452	0	0.185268	0.676652	0	0	0.395253	1.87872	0	0	0.49565	1.35662
ENSG0000(	DANCR	4:5271240	37.1493	46.6607	35.2524	65.6758	40.9046	38.403	37.2343	42.5282	32.7814	20.7027	0.4043	-0.32439
ENSG0000(	DAP3P2	2:1714914	0	0.466896	0.883414	0.442659	0.325989	0	0	0	0	0	0.0182	#NAME?
ENSG0000(	AC010967	2:5272267	1.90958	0	0	0.804628	0.303894	0	0	0	0	0	0.0237	#NAME?
ENSG0000(	SLC39A11	17:726459	8.4242	13.835	11.762	11.89	10.1755	33.5753	17.215	16.8285	12.7713	10.6192	0.1696	0.618879
ENSG0000(	ADAR	1:1545820	62.7258	73.4026	66.0191	34.0435	51.8153	44.7659	73.6721	62.0781	37.0712	127.071	0.95865	0.023491
ENSG0000(	GOLT1B	12:215017	28.0066	19.8921	12.7757	12.7211	5.81329	46.0169	42.4166	29.5113	35.6331	12.5643	0.00155	1.22017
ENSG0000(	BIRC6	2:3235702	14.9983	12.1969	3.79012	4.44817	6.23761	9.11954	8.28843	5.96776	7.70564	13.8138	0.49915	0.281683
ENSG0000(	TMEM177	2:1196791	8.29067	11.2535	13.6	13.9075	10.8676	6.92532	5.93598	9.21215	18.9357	5.10723	0.64355	-0.25338
ENSG0000(	CDK19	6:1106099	0.694522	1.12123	0.958622	0.523667	1.43765	1.88239	2.27567	1.39822	0.520792	3.3002	0.18005	0.924499
ENSG0000(	RPSA	3:3940668	201.346	164.599	332.619	264.409	321.729	181.207	177.056	267.597	175.03	95.993	0.09965	-0.58358
ENSG0000(	COQ9	16:574281	23.4173	27.1187	28.3729	24.7532	20.7642	27.8446	22.4482	24.7281	39.4245	15.5549	0.8547	0.118047
ENSG0000(	RNF31	14:241433	22.2435	25.5641	23.9533	21.6501	42.7211	23.6869	19.2348	17.9614	13.2446	50.8764	0.6154	-0.3131
ENSG0000(	GREM2	1:2404895	0.63122	0	0.66919	0.635596	0.698017	0	0	0	0	0.266543	0.45515	-3.68731
ENSG0000(	NUB1	7:1513416	40.4155	34.4119	37.4842	38.6811	70.2415	39.3467	56.1473	30.2925	19.5653	45.3461	0.41125	-0.36678
ENSG0000(	RP11-27I1	9:1207935	1.48101	1.77779	0.192201	0.622009	0.878747	0	0	0.372307	0	0.212741	0.26025	-2.7195
ENSG0000(	ICK	6:5300127	2.59764	2.90763	1.33111	0.976095	1.69891	1.43369	1.83707	1.37362	0.89572	1.88224	0.77285	-0.34578
ENSG0000(	PHAX	5:1266002	79.5782	79.174	95.1991	83.4834	106.156	78.6124	62.1259	50.3755	37.3979	72.9831	0.15685	-0.63845
ENSG0000(	SLC26A6	3:4862572	14.2863	21.6869	4.95846	8.30397	4.46235	15.9279	12.4318	10.3643	6.60751	8.31796	0.85525	0.080075
ENSG0000(	RB1	13:483037	15.2043	15.843	3.37271	4.71832	3.75045	10.2165	5.80775	6.46496	5.84701	7.29191	0.6402	-0.26575
ENSG0000(	HAGLR	2:1761731	0	0	0	0	0	1.56625	0	1.03188	0	0.025821	0.11685	inf
ENSG0000(	RP11-214(	17:143010	2.92125	7.41906	0.423719	0	0.39171	1.4258	1.37509	0	0	0.356137	0.41905	-1.78637
ENSG0000(	RP11-554.	2:5554729	12.9934	9.60185	16.1429	11.9216	7.93646	7.67643	9.76781	9.18913	10.422	8.94501	0.81945	-0.31342
ENSG0000(	STAG3L4	7:6729560	13.9517	12.4383	10.7071	21.9696	7.88228	8.07296	11.5408	12.3298	12.4014	8.04848	0.53765	-0.33678
ENSG0000(	SEH1L	18:129471	11.1623	8.4291	7.79563	7.90435	3.59485	12.1163	10.7921	8.64917	7.43674	4.99692	0.7565	0.125171
ENSG0000(	TMEM267	5:4344425	15.4603	10.9171	12.2954	14.4129	4.78893	14.5421	14.9852	12.3997	17.8311	7.8846	0.5698	0.247344
ENSG0000(	IL18RAP	2:1024186	118.686	9.56179	144.354	265.264	68.7259	46.6078	65.7994	126.072	101.818	102.075	0.1625	-0.49978
ENSG0000(	CD200	3:1123024	1.90824	3.71971	0.32836	0.411275	0.242761	5.65955	2.61012	2.77849	2.64925	4.41427	0.15935	1.57062
ENSG0000(	RP11-228(	9:1278150	0.343268	0.457062	0	0	0.197094	0.768019	0	0.16329	1.20827	0.381734	0.3787	1.54931
ENSG0000(	KB-1460A	8:1011668	0.185178	0.909269	0.12781	0.264763	0.206762	0	1.13222	0.585381	0	1.14172	0.7725	0.713893
ENSG0000(	ASXL1	20:323583	13.4395	23.1096	8.22996	6.19256	11.6187	26.3575	21.8674	12.2955	11.4403	23.8487	0.12715	0.694655
ENSG0000(	RCL1	9:4792868	11.3779	8.6159	13.887	12.5382	9.5945	13.6307	8.18213	15.6999	15.0019	8.4795	0.7866	0.106258
ENSG0000(	TMEM260	14:564883	12.4016	11.3495	7.05316	5.22305	4.90391	3.57642	5.65621	7.48814	7.96132	11.4701	0.77945	-0.11803
ENSG0000(	MTHFD2L	4:7411417	4.98962	6.62148	3.12298	7.17787	1.4178	12.4516	1.19459	1.70019	8.24757	1.09942	0.96635	-0.0263
ENSG0000(	BMT2	7:1128191	5.37411	7.14738	1.10268	2.50248	1.35313	4.16236	1.42586	3.17933	0.211674	3.96032	0.4873	-0.36591
ENSG0000(	RP11-497(	5:7046224	0.324333	1.01226	0.906378	0.257915	0.702125	1.30844	0.011625	1.40283	0	1.10102	0.8937	0.201708
ENSG0000(	TRDV3	14:222025	5.15012	3.25492	4.39801	0.368789	0	1.29347	6.57541	5.00443	0	1.19317	0.858	0.273808
ENSG0000(	KRAS	12:251952	13.666	20.1487	26.9996	7.25671	19.0314	15.9329	26.4774	34.8224	21.6643	47.5908	0.41325	0.565072
ENSG0000(	IGHV1-24	14:106276	0	0.539219	0	0.592541	0.469794	0	0	0	0	0.462138	0.4922	-1.11257

ENSG0000(	TBPL1	6:1339521	63.3724	36.5422	101.194	62.5144	108.38	76.3568	45.997	52.0949	34.2129	43.1972	0.09675	-0.75836
ENSG0000(	PSME2	14:241433	528.63	364.472	795.77	808.721	614.689	838.008	801.239	707.734	620.1	254.667	0.97645	0.016598
ENSG0000(	C2orf49	2:1053340	10.3831	2.86337	6.83359	2.30278	8.91396	9.08861	15.2831	8.31182	7.5698	4.35115	0.65795	0.424897
ENSG0000(	RCN1P2	13:453334	0.498313	1.04664	0.250342	0.20694	1.06224	0.499761	1.44215	0.704093	0.369747	0.709818	0.7989	0.416455
ENSG0000(	NCL	2:2314535	217.54	219.781	168.15	100.861	297.953	192.249	248.055	138.622	60.0165	214.808	0.36885	-0.45626
ENSG0000(	ENY2	8:1092409	54.2459	33.4754	56.5492	41.8983	29.587	69.707	75.1223	52.2835	50.5949	19.4875	0.7693	0.212023
ENSG0000(	KLHDC8B	3:4917161	1.09343	2.10877	2.13785	0	1.48353	17.3587	0	1.5463	0	0.648455	0.30275	1.4246
ENSG0000(	SRA1	5:1404018	34.9196	32.8575	63.3786	70.9034	38.6372	47.6017	46.922	50.5898	51.4982	19.1823	0.74765	-0.23411
ENSG0000(	DDX3X	X:4133334	99.9002	95.656	31.6978	36.895	26.4881	58.8563	63.7883	58.1953	50.3306	61.0616	0.88275	0.045792
ENSG0000(	ACTG1	17:815099	999.497	1284.59	1181.36	997.401	1412.79	1374.83	1470.76	1617.46	1218.54	1266.57	0.76285	0.154232
ENSG0000(	ZNF528	19:523978	6.12881	4.70625	6.1736	3.85603	4.7055	6.31326	10.7252	4.46112	6.43274	4.83034	0.7544	0.238924
ENSG0000(	PTMAP5	13:816899	0.760339	0.852038	0.953586	0.831052	0.401186	1.08334	1.37444	0.162601	0.394692	0.261478	0.8898	-0.23722
ENSG0000(	RGS5	1:1631111	4.60313	4.61528	2.01939	4.60663	0.725408	5.99503	2.85325	1.01655	0	2.03194	0.6598	-0.37479
ENSG0000(	SAR1A	10:701472	14.4097	8.24992	62.5254	39.2493	33.7323	17.2637	18.4102	49.2425	33.2733	46.1538	0.9449	0.028987
ENSG0000(	ATP1A4	1:1601515	0	0	0	0	0	0	2.8079	0	0	0	0.1145	inf
ENSG0000(	CDH2	18:279509	0.725518	0	0.503184	0.289826	1.39251	0	0	0	0	0.659499	0.3193	-2.53359
ENSG0000(	THOC1	18:158382	38.024	34.921	18.7262	17.24	15.9193	56.5093	37.8255	19.3295	18.5386	21.1686	0.3707	0.421212
ENSG0000(	RNMT	18:136633	11.9297	18.8517	19.0513	18.5279	27.5475	19.8292	36.496	13.1401	21.4942	25.1918	0.782	0.178714
ENSG0000(	RP3-425C	6:1224367	1.14366	2.00651	0.690612	0	0.929177	1.31603	2.25512	2.44857	0	1.02794	0.3811	0.67385
ENSG0000(	SNAPC2	19:792031	15.343	12.5472	21.8702	12.723	23.0254	9.91067	20.6511	37.4396	17.3462	21.1194	0.49325	0.315497
ENSG0000(	TXLNB	6:1391351	0.527523	0.438546	0.103611	0.079073	0.235084	1.61476	0.256152	0.639847	0	0.386018	0.439	1.12962
ENSG0000(	SLC46A2	9:1127508	0.954676	4.12763	0.050834	0	0.131237	0	0	0.083757	0	0.007108	0.0944	-6.62387
ENSG0000(	ARMCX3	X:1016227	25.6974	22.5907	25.6657	16.8306	25.6444	36.3634	25.6052	31.1557	24.9218	39.731	0.48665	0.261
ENSG0000(	DVL2	17:722534	14.6279	19.5083	6.26345	11.2761	7.33743	10.1931	10.5556	6.70409	13.9882	10.7451	0.77795	-0.10918
ENSG0000(	RP11-481	17:511774	1.10623	0.520103	0.536888	0.424748	0.355367	3.12041	0.672708	0.137447	0.552615	0.968451	0.66995	0.801052
ENSG0000(	SLFN13	17:354350	4.35089	2.5425	4.00801	2.69365	2.44396	3.35128	4.78589	4.88844	3.50672	7.17488	0.3565	0.490471
ENSG0000(	SERPINA1	14:943767	24.9919	87.5075	2.58191	2.98195	8.25924	374.91	16.1315	19.0844	8.13709	11.8946	0.003	1.76354
ENSG0000(	COX18	4:7305236	4.16906	1.09626	2.2014	2.21857	2.53604	5.63107	2.49004	4.3352	1.0155	4.99128	0.5202	0.492849
ENSG0000(	PXYLP1	3:1412287	5.39424	16.4546	3.04723	2.02396	3.87074	1.80095	8.53179	2.74	5.50178	4.48998	0.6642	-0.39061
ENSG0000(	RPLP1	15:694527	363.185	313.795	882.941	652.23	422.798	427.629	406.47	970.082	702.767	115.531	0.8718	-0.06728
ENSG0000(	MTSS1	8:1244884	6.80719	16.3611	4.61822	8.89334	8.33906	2.23491	4.41091	6.05475	8.77758	8.74972	0.5641	-0.60699
ENSG0000(	RHOG	11:382697	115.763	140.905	144.503	146.527	237.666	143.325	158.496	138.54	121.179	173.959	0.67795	-0.13642
ENSG0000(	PRKD2	19:466476	36.9941	51.0879	15.152	15.8696	26.8703	36.8955	61.7615	34.0044	10.7309	53.0258	0.20325	0.640244
ENSG0000(	ZNF302	19:346776	17.9294	17.9047	18.6313	21.0754	10.2717	13.9362	9.9327	10.5917	9.02361	22.7145	0.3844	-0.35827
ENSG0000(	RIC3	11:801924	3.51369	12.5788	0.504833	3.70817	1.27251	4.92418	9.93032	0.575769	3.60409	3.87798	0.83465	0.158016
ENSG0000(	RP11-120	17:682591	2.10789	3.14446	0.915528	0.786513	0.384422	0.244299	0.128302	1.513	0.186989	1.28998	0.5209	-0.79886
ENSG0000(	PLCL1	2:1978047	2.20967	4.73929	0.233912	0.118362	0.973553	3.77857	1.18146	1.40933	0.615799	2.3551	0.83365	0.13838
ENSG0000(	IPO11	5:6230616	9.46313	7.18419	1.54407	4.39206	1.35894	3.91806	7.44641	3.33126	3.23921	2.76195	0.9397	-0.07138
ENSG0000(	CCZ1	7:5898724	31.4641	25.6781	11.6883	11.3747	13.9615	24.317	28.7139	13.1952	15.5666	13.2725	0.9101	-0.0621
ENSG0000(	TMCO6	5:1406387	15.3162	20.1966	15.0739	20.5213	7.35329	8.25656	14.464	14.9765	15.0322	10.8262	0.7126	-0.26726
ENSG0000(	C2orf69	2:1999112	5.87656	8.10492	5.17492	4.19582	5.10648	8.18629	6.677	6.87161	6.61581	10.4382	0.5526	0.327469
ENSG0000(	SGPP2	2:2224245	0.672013	0.700983	0.039023	0.179741	0.153965	0.51732	1.95136	0.617715	0	0.59613	0.72265	1.03714
ENSG0000(	CDK12	17:394615	4.6061	6.61914	7.0787	4.80168	13.264	7.74302	6.15624	7.59996	4.30154	21.4833	0.54805	0.244899
ENSG0000(	LYPD5	19:437959	0	0	0.077686	0	0	2.14292	0	0	0	0.498287	0.3239	4.2407
ENSG0000(	USP15	12:617082	96.8761	117.127	38.5425	58.2508	41.122	93.5877	111.423	49.5891	73.829	71.4432	0.5873	0.237515
ENSG0000(	C17orf62	17:823892	121.224	139.372	111.278	123.023	106.11	152.571	198.061	109.771	95.7592	134.309	0.6413	0.154218
ENSG0000(	TYW1	7:6698767	15.719	16.5121	11.7886	17.1315	10.624	6.62279	12.8449	12.2336	9.99732	14.3567	0.45835	-0.48955
ENSG0000(	CDK20	9:8796644	2.74722	3.28144	2.05847	2.33507	2.66235	1.79909	3.65101	1.81309	5.90058	1.73161	0.7058	0.281394
ENSG0000(	EXOC1	4:5585361	16.3113	17.2625	8.66815	7.43495	6.10064	18.3738	12.7589	11.552	4.24001	11.894	0.8259	0.088392
ENSG0000(	INPPL1	11:722237	10.198	11.3603	3.05958	2.54884	5.68688	9.90904	10.5249	8.20136	3.61536	12.2307	0.17845	0.569157
ENSG0000(	IGLV1-51	22:223032	0	4.89127	2.41365	0	0.454049	0	0.110267	0	0	0.14882	0.11385	-4.50111
ENSG0000(	AC016734	2:6312138	0.988076	0.586822	0.655848	1.9919	0.29903	1.24776	1.35362	0.914826	2.49069	0	0.73555	0.43229
ENSG0000(	VPS51	11:650842	64.7865	69.4535	70.9126	49.11	80.8668	55.7635	52.9762	59.1173	36.7621	61.8876	0.3172	-0.36348
ENSG0000(	RP11-331	9:3560530	1.24891	1.85949	0.069188	0.43987	0.066489	0.767392	2.16405	0.446659	0	0.08581	0.9526	-0.11323
ENSG0000(	ILF2	1:1536586	144.295	113.678	175.082	166.563	159.958	163.412	146.309	150.051	117.98	90.3018	0.5864	-0.2125
ENSG0000(	OCIAD2	4:4888501	123.042	131.032	148.7	120.578	85.4553	210.051	168.158	114.799	221.109	43.2561	0.43675	0.291092
ENSG0000(	CSNK1G2	19:194114	63.0309	72.0319	15.8001	11.8562	26.9591	36.6597	82.8231	23.1382	20.5061	27.6232	0.67935	-0.1529
ENSG0000(	AC055764	3:1977912	0.389216	0.33773	0.181744	0.127519	0	0	0.195501	0.519688	0.936202	0.21909	0.4918	1.15311
ENSG0000(	NPEPPS	17:475229	20.9907	24.5876	15.0325	14.1159	23.1783	23.5225	15.4774	27.3064	14.1862	46.614	0.54885	0.275126
ENSG0000(	PCYT1A	3:1962114	12.75	16.2702	9.0373	13.5578	9.01309	11.1017	12.4973	8.13015	20.1767	18.7228	0.695	0.18216
ENSG0000(	CH17-373	1:1452811	95.5391	12.6121	13.6055	15.2088	4.54396	1.20491	1.3537	2.19553	16.6528	0.983681	0.16555	-2.31722
ENSG0000(	CTC-459F	19:276845	7.41406	6.43805	8.32741	7.51016	9.97212	12.3841	14.6253	8.76054	20.1093	23.7788	0.18825	1.03955
ENSG0000(	NEDD8-M	14:241718	0.314023	0.602112	0.545852	0.18997	0.215976	0.221934	0.561033	0.236	1.30528	0.218348	0.50135	1.42056
ENSG0000(	AGO4	1:3580817	5.61479	2.63797	2.86274	1.84135	3.07751	1.44054	3.73887	3.33176	1.47154	5.91308	0.68145	-0.17431
ENSG0000(	TKT	3:5322470	35.9358	41.6978	38.4395	30.2429	48.6433	55.2719	46.3941	54.2951	23.4117	41.1535	0.98375	-0.00723
ENSG0000(	PODXL2	3:1276291	2.86263	5.9525	1.62876	1.93248	2.17411	3.3199	3.43976	1.66149	0.518639	2.80682	0.6297	-0.38665
ENSG0000(	CPSF6	12:692395	32.0198	40.4608	29.0857	26.2954	36.6793	13.6582	31.2977	39.262	39.5984	44.305	0.75455	-0.12679
ENSG0000(	PCOLCE2	3:1428159	0	0	0	0	0	6.27863	0	0	0	0	0.11165	inf
ENSG0000(	MPZL1	1:1677211	1.90109	8.03453	0.720096	1.97431	1.20128	6.76097	0.348322	1.89597	3.28438	2.63478	0.90345	0.083832
ENSG0000(	TLE2	19:299763	4.33635	12.0625	1.13426	1.44512	3.61377	0	0	1.28218	0.764078	3.28308	0.0507	-1.85507
ENSG0000(	TTC5	14:202565	10.4975	8.9572	21.0245	19.6426	12.87	15.2373	16.907	13.0567	13.0509	14.5452	0.7122	-0.1503
ENSG0000(	PRF1	10:705973	701.716	220.091	870.377	768.07	1404.86	213.075	664.921	610.32	266.04	574.755	0.0634	-0.91684
ENSG0000(	CORO6	17:295605	0.965592	1.42243	0.616564	0	0.690343	1.11034	0.520861	0.172923	0	1.56386	0.91185	-0.09982
ENSG0000(	ACAT1	11:108008	31.7304	33.7053	40.0713	32.894	32.2574	53.3772	48.7138	37.2953	29.9407	20.6658	0.7151	0.17395
ENSG0000(	ACAA2	18:497821	130.898	66.9483	133.074	193.766	185.123	93.6585	100.488	94.3835	106.638	87.2319	0.10295	-0.61007
ENSG0000(	B3GLCT	13:311999	7.43299	1.93442	4.45041	3.31625	2.81833	4.65692	1.38984	3.1708	0.181582	3.47679	0.1979	-0.70083
ENSG0000(	PHLDA1	12:760254	3.49782	1.38227	3.53191	9.75604	2.28524	16.7282	30.1376	16.266	6.9916	6.7432	0.00005	2.13761
ENSG0000(	ZMYND11	10:134464	30.5469	24.405	8.3283	8.02523	12.2108	15.5399	14.6295	15.3675	9.9806	19.2265	0.58235	-0.19146
ENSG0000(	PTCD3	2:8610618	60.9276	84.3477	29.2457	37.1177	15.6402	54.0644	44.8075	32.1912	19.6548	25.9205	0.3089	-0.45952
ENSG0000(	PATL2	15:446657	14.1823	28.0459	20.9272	15.8918	8.86679	16.0508	24.2359	13.8676	9.25564	16.3506	0.98205	-0.0121
ENSG0000(	ATM	11:108157	30.2645	34.5091	15.7904	15.2258	12.9411	14.7639	17.3141	12.0918	11.69	24.7584	0.3122	-0.46135
ENSG0000(	ALKBH5	17:180878	53.6099	46.5597	30.6578	28.5998	30.4623	31.2299	34.9443	46.9841	29.222	52.7778	0.8691	0.073183
ENSG0000(	RP11-787	6:1089864	0.768868	0.606806	0.427586	1.15012	0.526506	0	0.241614	0.635079	0	0.336829	0.3538	-1.30479
ENSG0000(	NUDCD3	7:4437912	21.5792	21.1442	16.4496	14.7731	13.5042	13.3478	25.6276	12.6568	15.6026	20.0439	0.3325	-0.4029
ENSG0000(	CDIP1	16:451067	8.2921	9.07359	5.25966	4.08397	8.46696	10.909	12.9407	6.47607	3.52931	16.2759	0.3288	0.478903
ENSG0000(	RP11-121	11:902011	0.157931	0.182344	0.247784	0	0.026667	1.49067	0.83537	0.366176	0	0.639715	0.19215	2.33181
ENSG0000(	ASH1L	1:1553352	23.1679	45.3778	7.78331	6.11601	5.73843	26.8238	35.163	10.1842	13.9973	18.0564	0.87975	0.097454
ENSG0000(	STK36	2:2186638	9.23514	12.1704	0.820798	2.2615	2.11957	3.02573	7.44324	3.36992	0	3.71663	0.5568	-0.49916
ENSG0000(	EIF2AK3	2:8853871	10.2581	16.0526	3.70044	5.47864	2.35716	4.54035	2.72276	2.94967	7.22004	6.15331	0.2396	-0.50958
ENSG0000(	AC061992	17:783157	1.33148	0.35557	0.270028	1.80493	0.591889	1.3761	0	0.219345	0	0.233647	0.46255	-1.23619
ENSG0000(	TMPRSS6	22:370654	0.235385	0.010854	0.233839	0	0.185585	0.98411	3.35886	0.307195	0	0.528954	0.05855	2.78464

ENSG000C	INPP5A	10:132537	2.70424	5.50341	0.703864	1.09893	0.904009	3.53152	4.0805	1.88116	0.322066	4.35588	0.6429	0.362836
ENSG000C	CORO7-P.	16:431476	6.94977	5.17301	0.571225	5.88002	2.29578	4.30209	5.72161	2.67819	6.2978	1.76487	0.90835	0.136658
ENSG000C	OCM	7:5879826	0.39913	3.02451	0.706793	0.393561	0.308313	0	0.447337	0	0	0.777866	0.3891	-1.80026
ENSG000C	C4orf33	4:1288096	5.92313	3.35456	8.45559	3.56846	3.39901	17.195	10.468	7.30647	2.61829	3.05448	0.616	0.492909
ENSG000C	FUT8	14:654105	6.45305	14.2943	5.5815	7.1582	5.89001	15.943	21.7953	7.47128	17.7487	15.2032	0.0439	0.965465
ENSG000C	TGFB1	19:412624	35.8743	19.7067	5.72669	3.13656	22.0899	10.8692	19.2558	6.09365	4.407	13.3629	0.63075	-0.64624
ENSG000C	SNHG6	8:6687074	52.1436	49.3841	84.7835	56.3358	34.6705	58.7455	53.9528	79.3995	60.727	12.6361	0.94245	-0.03326
ENSG000C	PLEC	8:1439151	13.0339	37.1731	6.15166	3.39859	33.7608	16.7657	35.5504	29.7719	7.64708	86.0032	0.0263	0.876377
ENSG000C	CCR1	3:4616360	22.5055	10.5499	28.3144	20.6603	54.8194	31.2831	32.2581	29.5519	11.7169	49.7416	0.9247	-0.03527
ENSG000C	DICER1	14:950862	6.79464	9.19542	3.86355	3.13894	4.84644	7.84572	6.18164	5.06506	5.14415	11.1981	0.64115	0.184367
ENSG000C	CLEC2D	12:966496	103.957	207.737	144.638	112.173	68.6907	234.096	297.552	214.923	209.213	123.189	0.08705	0.680402
ENSG000C	TOMM20F	17:354642	18.3386	4.3514	4.26616	12.8315	6.24121	26.0279	17.8623	16.23	19.7397	5.55539	0.5792	0.947205
ENSG000C	VTI1A	10:112446	24.1117	29.6141	10.1964	20.5231	12.4174	17.3499	27.2231	11.648	19.5808	25.9208	0.9884	-0.00704
ENSG000C	TTLL1	22:430385	8.73068	6.30225	13.3468	18.6483	15.7116	5.51766	6.26543	7.79843	11.2903	8.81349	0.1693	-0.68609
ENSG000C	BCAS1	20:539367	0.09666	0.315352	0.391822	0.467851	0	1.48144	0.41932	1.70205	0.519349	0.55212	0.1155	1.74249
ENSG000C	DPY19L2	12:635589	0.472926	0.023973	0.241108	1.40231	0.238954	2.46058	0	2.51665	0	2.32366	0.24775	1.35475
ENSG000C	BACE1-AS	11:117232	0.78008	1.25375	1.74464	0.297547	0.861106	0.36809	2.769	1.02454	0	3.62311	0.6972	0.631851
ENSG000C	SPIN1	9:8838841	9.31643	8.67469	3.32967	1.60672	3.61752	5.31567	7.01745	5.04152	3.70668	9.9234	0.71675	0.143194
ENSG000C	CRIM1	2:3635592	1.64387	0.755793	0.789107	0.36227	1.17119	3.06117	1.22313	2.7529	0.934075	2.24777	0.09925	0.940983
ENSG000C	RP11-15A	19:439678	0.484421	0.041411	0.535398	0	0.16885	2.06972	0.03791	0.250392	0	0.067393	0.68965	0.955806
ENSG000C	LDHA	11:183943	379.331	336.93	320.17	373.198	291.774	597.051	677.274	606.468	591.379	353.04	0.10495	0.669461
ENSG000C	RP11-2H8	12:718397	0.30575	1.44051	0.150981	1.19516	0	0	0.849851	0.350601	2.4268	0.445781	0.75565	0.539771
ENSG000C	PYCR1	17:819323	2.21629	3.76386	0.312111	2.06299	0.70944	1.74492	8.60522	4.10952	8.56847	0.666078	0.02905	1.53471
ENSG000C	SWSAP1	19:113681	6.46622	7.86521	13.4015	8.65061	9.00721	12.6605	3.95447	9.24823	8.69383	6.30932	0.6514	-0.22134
ENSG000C	PPP1CC	12:110719	75.8903	121.808	52.0651	57.8567	51.017	86.4011	73.1446	63.9937	72.5246	75.5249	0.8965	0.039218
ENSG000C	ENOX2	X:1306233	10.5818	10.6246	7.26309	7.8662	9.31835	6.53005	7.56034	4.46964	16.9719	10.0689	0.91615	-0.06485
ENSG000C	POFUT2	21:452639	4.98181	4.9246	0.959519	2.04869	1.2225	4.47089	3.23596	1.09461	1.69434	3.34855	0.7385	-0.20949
ENSG000C	TMEM43	3:1412493	43.1649	46.0956	35.4249	45.7982	22.2071	39.1237	68.4772	50.6369	39.6429	44.3535	0.53525	0.315144
ENSG000C	RP3-402G	22:502009	0.110581	0	0.139369	0	0.310028	0	1.7513	0.060795	0	0	0.89315	0.226832
ENSG000C	RP5-965G	20:252480	1.38147	0.349221	0.075859	0	0.758625	0.603407	0.958776	0.761086	0	0.39339	0.9813	0.001991
ENSG000C	CDKAL1	6:2053445	7.1109	18.6062	7.46813	9.5319	4.44796	1.45871	9.72911	5.10065	7.88256	8.87034	0.30325	-0.45742
ENSG000C	PTPN7	1:2021470	102.365	113.948	102.1	91.7764	90.9446	212.268	274.754	264.985	176.791	325.289	0.00085	1.31163
ENSG000C	PPP1R10	6:3060039	34.2219	18.5224	29.2964	14.0468	24.8711	14.4678	17.9793	20.5139	11.4961	28.4629	0.2398	-0.80516
ENSG000C	FAM167B	1:3224723	0.54911	0.209068	0.244912	0.285257	0.49598	0	1.10394	0.660517	1.30482	0.836585	0.57815	1.14996
ENSG000C	ZC3H7A	16:117505	38.2833	62.4837	13.3099	25.8624	12.916	40.4335	21.7443	12.8706	36.8647	17.2002	0.98265	0.00814
ENSG000C	MBNL2	13:972214	18.3014	15.7302	6.19273	6.20547	5.01468	11.7218	4.70435	7.1682	10.8129	7.64203	0.6986	-0.16143
ENSG000C	AC005740	5:1419435	0.18574	0	0	0	0.085082	2.23135	0	0	0	0	0.45355	3.03206
ENSG000C	RP11-7F1	8:6734397	0.456476	0.028753	0.190131	0	0.260708	0	2.03673	0.18392	0.041286	0.244642	0.35155	1.49357
ENSG000C	KLHL35	11:754223	0.143517	0.849291	0.468542	0	0.164518	0	1.06997	0.612614	0	0.923963	0.60335	0.6747
ENSG000C	RP11-106	15:722411	2.80605	5.66054	1.6805	3.81427	1.20817	3.35938	2.2664	1.18018	3.97168	4.26374	0.99185	-0.01754
ENSG000C	SLC15A3	11:609131	0.262351	1.28708	0.522756	0.534793	0.64317	5.5414	0.670288	1.2653	3.72064	1.01502	0.13785	1.91947
ENSG000C	PEX6	6:4296075	10.0117	1.02195	1.71547	2.96286	6.89705	14.4488	0.537852	2.4635	1.70086	12.7965	0.49505	0.388073
ENSG000C	IFT52	20:435909	17.407	14.7091	17.6134	11.8105	15.5251	14.8707	20.514	11.1188	2.88464	3.03358	0.19995	-0.63623
ENSG000C	KCNQ1	11:244468	1.24111	10.6122	0.341936	1.91428	0.548381	4.10719	1.10407	0	0	0.688333	0.27555	-1.42954
ENSG000C	MAP3K8	10:304339	32.861	3.90165	49.2873	81.5221	19.3628	13.515	26.602	43.9596	27.6409	16.9884	0.2735	-0.64217
ENSG000C	SUGP2	19:189908	18.1343	19.9921	10.0962	14.681	14.5179	19.519	16.5012	14.491	17.4835	36.7001	0.29285	0.43693
ENSG000C	PEAK1	15:771006	3.49093	2.71666	1.47447	2.20918	3.14502	1.96369	0.984	1.15474	0.892823	4.19329	0.6159	-0.3906
ENSG000C	RP11-120	18:625230	0.575037	0.499491	0.43517	0.777502	0	0.169025	0	0	0.421415	0.349928	0.58985	-0.91737
ENSG000C	HSPA1A	6:3181546	27.0205	52.7037	28.9275	12.7045	56.8339	155.034	112.001	145.656	213.184	308.695	0.00005	2.47937
ENSG000C	NUP155	5:3728813	7.94207	7.5738	1.80624	3.75146	2.14626	3.21447	5.45181	5.37842	3.94819	4.84924	0.879	-0.08966
ENSG000C	TTPAL	20:444758	3.58771	2.63851	5.10361	2.95293	4.77008	3.85519	3.27359	4.49229	4.46477	9.4335	0.97415	0.014152
ENSG000C	LY6G5C	6:3167668	9.05404	6.10182	8.11262	7.66318	8.53429	17.0479	7.01583	9.60062	16.0728	6.94908	0.3774	0.585131
ENSG000C	PIP4K2B	17:387656	6.29435	6.43145	2.35615	2.04716	4.44447	2.25081	3.34759	3.65065	1.37525	10.0706	0.9274	-0.05096
ENSG000C	LILRA1	19:545729	1.15297	5.72835	0.21475	0.456836	0.230357	5.18621	0	0.236035	0.102882	0.813225	0.80175	-0.28666
ENSG000C	CFL1P5	5:6928017	1.01354	0	0.173882	1.76623	0.269705	0	1.0168	0	0	0	0.4665	-1.93619
ENSG000C	PGAP2	11:366558	12.5071	12.6422	8.52467	10.7595	10.7081	9.88952	7.58533	4.17441	12.3791	10.3407	0.5456	-0.37513
ENSG000C	AAK1	2:6945799	89.6574	155.63	105.836	161.179	120.374	109.133	133.768	67.9111	95.1911	121.735	0.73735	-0.28139
ENSG000C	TREML1	6:4114934	0	0.072068	2.32025	10.8585	0.496959	0	0	0	0	0	0.00855	#NAME?
ENSG000C	NCSTN	1:1602892	52.019	58.6317	39.6059	63.0655	41.0134	52.4955	32.4241	33.455	40.8424	47.5942	0.64665	-0.19939
ENSG000C	LMBR1L	12:490965	30.7493	50.5294	8.61524	29.4608	6.26546	28.6408	19.2447	13.6987	36.678	6.87835	0.5735	-0.19811
ENSG000C	ATRN	20:347103	1.89569	2.72413	0.618754	0.305663	0.502822	0.525474	3.42726	1.45924	1.50245	1.39253	0.7141	0.258623
ENSG000C	ATP11C	X:1397263	2.16852	2.53937	1.17268	0.981974	2.19914	3.61119	3.09208	1.07213	7.85488	4.3524	0.02075	1.17415
ENSG000C	APOE	19:449057	0.081611	1.55096	0.394178	0	0.065166	1656.24	25.6023	12.3795	59.6869	0.963015	0.00235	9.90329
ENSG000C	ANKRD19I	9:9280938	0.619608	0.823772	0.690899	0.174595	0.918088	1.59643	0.092708	0.209997	0.076308	1.42036	0.99275	0.013865
ENSG000C	SLC50A1	1:1551353	38.3811	29.4328	34.9935	49.1578	33.3072	58.3822	55.7287	34.1381	54.5873	21.5834	0.4959	0.249531
ENSG000C	S1PR4	19:317234	308.632	324.447	244.264	168.992	398.659	150.688	203.283	160.826	164.426	177.932	0.0185	-0.76294
ENSG000C	DEDD	1:1611005	20.6011	34.1199	32.2175	29.7697	30.9022	26.8325	36.4698	32.9772	25.4512	26.7491	0.8087	-0.10809
ENSG000C	ZNF586	19:577696	11.3099	15.2586	12.5325	9.7738	7.97268	5.55347	8.13083	13.6368	4.96134	9.74909	0.53395	-0.45838
ENSG000C	RP11-107	17:493707	0.963134	1.58263	2.89728	2.59329	0.345001	4.98008	1.88788	0.426258	1.89601	0.514729	0.65	-0.62775
ENSG000C	RP13-580	17:443368	15.8998	8.95061	17.5147	16.4651	30.6646	10.0986	15.2187	18.3424	13.1443	30.9797	0.92415	-0.05703
ENSG000C	TMEM44	3:1945840	1.02651	1.02168	0.621576	0	0.368018	0	3.82369	4.90604	0.328526	2.54229	0.0834	1.81399
ENSG000C	SIPA1L1	14:712927	34.7789	15.7548	13.3698	39.9837	26.0601	20.8966	24.2657	23.9332	35.5965	41.1084	0.57515	0.25788
ENSG000C	TRBV5-7	7:1425200	0.494269	0	0	0	0	0	0	2.11027	10.063	0	0.12595	5.07961
ENSG000C	GPR82	X:4151493	0.159634	1.1449	0	0.019716	0.022719	1.2707	3.02812	5.22903	2.30924	3.97843	0.0248	3.72817
ENSG000C	CCDC146	7:7695983	6.40992	4.4073	5.9403	2.01217	8.23044	7.20928	4.26262	7.40727	5.00542	10.0329	0.83355	0.187517
ENSG000C	HLA-DPB1	6:3306456	19.1792	58.7179	104.568	80.2074	111.593	156.901	98.9055	156.81	100.399	100.965	0.2586	0.601339
ENSG000C	GLRX3	10:130136	75.8257	46.9971	66.6517	70.013	50.1294	104.639	87.428	66.9072	56.9905	28.1514	0.7822	0.093369
ENSG000C	STK17B	2:1961335	27.4762	37.8653	31.1741	30.1216	20.8006	45.6769	63.5284	77.5141	74.6356	71.8992	0.0022	1.02643
ENSG000C	RBM28	7:1282976	17.9927	22.2961	11.6924	10.0617	15.2389	17.7737	21.297	15.2196	1.6084	24.8842	0.9098	-0.06759
ENSG000C	PQLC3	2:1115519	58.8573	38.3394	54.8035	48.2514	37.8775	65.8705	45.7537	69.3756	78.4355	37.1651	0.2371	0.384277
ENSG000C	DBN1	5:1774566	5.57871	8.20108	5.41941	5.89519	9.84695	7.74568	31.4261	7.75801	2.61978	11.3308	0.18695	0.609275
ENSG000C	EIF3B	7:2251769	103.8	94.7588	46.3008	35.1168	56.5574	91.2844	69.5464	36.8289	46.511	63.6606	0.61775	-0.16149
ENSG000C	PWWP2B	10:132397	2.94835	2.99271	3.50994	1.29472	3.53842	1.37563	8.38093	1.72814	6.30245	3.75419	0.4302	0.571519
ENSG000C	PKN2	1:8853751	7.88535	8.5813	2.27403	3.37594	4.56523	8.80371	6.36671	4.26044	2.71986	5.17706	0.9068	-0.06044
ENSG000C	BEND3P1	10:506559	1.29392	1.31998	0.133848	0.635168	0.17593	1.44196	0.68228	0	0.018153	0.413297	0.70785	-0.52028
ENSG000C	RCBTB1	13:495319	8.78441	7.2001	1.45349	3.66093	1.45761	5.44463	4.08421	2.43503	3.5111	6.04344	0.85755	-0.08264
ENSG000C	EEF1A1P1	5:4348395	1.00823	0.676163	1.70428	1.60409	0.973263	0.175426	0.587557	0.761147	1.24761	0.388567	0.65645	-0.75386
ENSG000C	ARFRP1	20:636578	47.928	66.1731	77.0036	62.9648	88.0773	47.6599	63.7742	61.7622	54.2808	65.8464	0.7424	-0.25412
ENSG000C	RP11-205	8:3785861	1.95813	2.68971	1.81624	0.540751	2.06569	1.35762	1.91001	2.66553	6.98849	3.97838	0.4256	1.77697
ENSG000C	RUFY2	10:682826	4.77203	6.52857	4.08433	3.502	5.76072	2.83628	3.89016	4.8227	6.69299	13.6723	0.6474	0.289099
ENSG000C	RP11-580	17:475229	0.803961	1.56033	3.49369	2.33243	0	0	0	1.46992	0.352686	0.217314	0.32805	-1.98657

ENSG0000C	SFMBT1	3:5290357	6.03366	9.03259	7.52703	7.54519	6.91997	8.81371	6.74921	5.50043	14.0109	12.768	0.59875	0.236696
ENSG0000C	KDM5C	X:5319132	4.35367	5.23597	2.1078	4.95468	2.94215	3.27867	3.55452	3.19774	16.0254	7.69576	0.064	1.07067
ENSG0000C	CD7	17:823148	76.6747	70.474	19.6734	17.6699	40.8557	79.788	202.928	38.6875	39.4539	44.9161	0.0658	0.69756
ENSG0000C	RPL4P1	14:217502	2.86415	2.69264	3.48574	4.966	1.59049	0.182401	3.17311	0.457173	2.78127	4.86968	0.50505	-0.50034
ENSG0000C	RIC1	9:5629024	4.6435	5.86245	0.982448	1.52143	1.03727	2.20641	2.96327	0.798647	2.58997	1.93172	0.75585	-0.28213
ENSG0000C	ANAPC4	4:2537721	38.2167	31.034	17.7355	18.6626	12.2911	15.265	29.8914	18.8456	23.2325	24.3311	0.98915	0.005175
ENSG0000C	EFHD1	2:2325479	0.193972	1.42826	0	0.896117	0.293171	0	0.165104	0	0	0.838992	0.36115	-1.49648
ENSG0000C	TCTEX1D2	3:1962114	22.8166	15.6815	21.8332	21.3263	11.341	29.5171	17.8174	23.1695	32.213	2.7972	0.8588	0.176375
ENSG0000C	TSPOAP1	17:583012	31.9041	7.79151	3.84777	6.72588	9.10938	26.3156	13.9293	5.48569	9.30713	14.276	0.74105	0.251906
ENSG0000C	LMNB2	19:238977	9.11078	12.9827	7.27192	5.26385	7.55211	18.6925	16.5648	9.47352	8.30202	9.82509	0.3601	0.571329
ENSG0000C	TOP1MT	8:1433043	5.04641	15.392	5.74892	3.65583	5.56958	17.5982	8.62139	7.78664	5.18169	5.58031	0.4593	0.381036
ENSG0000C	BMS1P4	10:736448	0.634774	1.33736	0.828907	0.744437	0.314753	1.61279	1.75705	0.36934	3.42996	0.3368	0.73545	0.45118
ENSG0000C	KIR3DL3	19:547244	0	0	0	0	0	1.74982	1.63038	0	0	0	0.17335	inf
ENSG0000C	FAM219A	9:3439818	0.366149	0.433972	0.561798	0.307205	0.825983	0.526517	0.757501	0.652335	1.87211	1.16969	0.34565	1.04041
ENSG0000C	SLC44A2	19:106024	120.634	167.673	80.0071	76.731	80.9687	63.1269	57.6421	55.9674	55.1411	89.9124	0.0155	-0.74416
ENSG0000C	MDS2	1:2358149	0.170176	3.22817	0.382662	0	0.176148	0	0	0.064325	0	0.152188	0.1893	-4.5356
ENSG0000C	RP11-109	11:117135	6.8889	4.88059	2.6368	5.95851	1.66032	5.01279	2.67028	1.68823	1.81532	1.78167	0.71755	-0.86934
ENSG0000C	PICK1	22:380563	8.41863	12.3561	5.56318	8.30246	5.84724	6.21282	7.2887	5.76259	3.37908	9.30995	0.45845	-0.39076
ENSG0000C	RPS23P8	X:7096296	2.6087	2.53627	4.35855	4.39899	1.49944	3.6365	1.73183	2.59575	2.73529	0	0.79235	-0.5497
ENSG0000C	MAN2C1	15:753467	64.9297	97.7276	31.0406	39.8914	19.2836	57.1642	45.4101	28.1573	29.5404	33.831	0.5491	-0.24283
ENSG0000C	CEP57	11:957689	38.9026	46.471	29.3204	34.5676	33.6866	35.9501	33.6303	39.9029	32.8579	37.6637	0.77525	-0.11702
ENSG0000C	TRAT1	3:1088226	142.746	164.251	112.722	110.135	80.0714	75.1775	48.0445	127.432	139.777	39.203	0.1714	-0.43468
ENSG0000C	RP11-798	4:1733637	1.27706	12.1155	4.26558	2.99677	2.17672	4.80415	7.21833	9.36569	5.27153	3.18607	0.7251	0.465545
ENSG0000C	YY2	X:2183963	1.26063	1.09293	0.093169	0.101347	0.411159	2.05122	0	0.224001	0.113483	0.557244	0.8691	0.318836
ENSG0000C	PIGF	2:4649074	21.0914	22.2255	21.2635	22.2894	7.8582	24.6193	27.0509	15.5076	14.4563	5.49	0.8627	-0.10949
ENSG0000C	RP11-734	12:894197	0	0.486449	0.338803	0	0.563591	0.618402	1.63739	0	0.275218	0.457572	0.7066	0.808977
ENSG0000C	PMPCB	7:1032974	51.0695	76.4571	59.3709	71.2562	44.0832	58.5418	63.5008	57.9772	62.1301	59.0983	0.95165	-0.03613
ENSG0000C	RP11-138	7:1309364	1.49835	1.64161	4.38625	3.15881	0.607412	0.373467	2.45748	6.24886	8.09719	4.37301	0.1074	0.877997
ENSG0000C	BAZ1A	14:347527	20.9101	32.2356	14.9023	16.8206	17.2577	26.9617	30.8752	23.3113	32.1468	46.5613	0.0712	0.576888
ENSG0000C	PRDM2	1:1370019	18.129	21.39	10.8613	10.8364	14.6731	14.6802	14.2564	12.9662	8.61816	24.5633	0.94205	-0.03458
ENSG0000C	RP11-372	12:194605	0	0	0.929544	1.43525	0.338371	0	0	0.764449	0.340713	0.137784	0.59915	-1.02284
ENSG0000C	F11R	1:1609952	1.85952	2.97242	1.01648	1.86322	2.19003	1.91932	1.6616	1.80352	0.907499	3.52663	0.99035	0.015508
ENSG0000C	SLC35A1	6:8740798	34.478	23.7796	25.9643	29.134	14.9999	22.0864	28.8298	12.263	18.7872	9.86511	0.20595	-0.45206
ENSG0000C	SLC18B1	6:1327693	9.91532	10.4235	7.21741	4.83321	5.6072	5.75174	11.282	6.61303	0.346715	6.96439	0.5134	-0.30046
ENSG0000C	NAPG	18:105259	11.6103	13.4295	5.42574	5.80687	6.15438	21.7636	7.26808	7.39677	16.6693	6.98746	0.38325	0.406176
ENSG0000C	ZDHHC16	10:974461	18.0286	24.4797	17.0071	18.9366	20.129	26.0539	17.81	14.1888	22.5657	14.6582	0.7952	-0.10221
ENSG0000C	LZTR1	22:209651	28.9038	26.3797	5.97081	6.48889	5.78706	27.2687	17.746	13.3457	5.13543	10.2765	0.9659	0.01732
ENSG0000C	CMTM6	3:3239167	70.2582	42.451	53.4321	51.3836	56.7378	80.0662	75.5898	90.4913	88.1133	73.8299	0.13895	0.558317
ENSG0000C	CALML4	15:680541	6.91456	3.33283	1.72362	1.99271	1.84999	21.7072	5.21425	2.99134	15.466	4.75945	0.09405	1.79758
ENSG0000C	UPP1	7:4808862	53.3661	39.3967	87.2567	88.3534	92.2072	104.029	34.9626	106.61	109.374	36.8956	0.83775	0.080941
ENSG0000C	RRN3P1	16:217961	13.8036	11.4047	19.5523	15.1538	4.90679	27.3434	12.8193	20.1002	10.6561	6.85389	0.66725	0.255802
ENSG0000C	SF3A3	1:3795697	43.8139	45.7019	59.9754	52.3053	45.6279	48.5461	42.6961	37.9518	24.5337	45.2422	0.2074	-0.38104
ENSG0000C	CCDC71L	7:1063722	1.28545	3.49102	2.82869	1.34558	4.59216	3.6798	2.852	3.71991	2.64058	5.46165	0.8617	0.250976
ENSG0000C	LMLN	3:1979601	1.17939	0.768388	0.455449	0.62398	0.515705	1.90665	0.644292	2.08462	0.832986	2.06085	0.10975	0.996909
ENSG0000C	HEXIM1	17:451485	7.90982	7.89524	7.75064	5.69821	7.07704	8.20938	8.08407	7.28512	11.4963	11.6555	0.53255	0.28513
ENSG0000C	RPL18AP3	12:104215	25.9906	16.3155	58.5113	76.1623	13.8559	12.1412	14.9852	23.0027	33.0744	1.30987	0.5092	-0.87576
ENSG0000C	METRN	16:715114	12.3279	3.53283	7.8538	2.57237	12.6834	6.13672	18.5205	5.00612	0.237352	2.65599	0.3137	-0.79362
ENSG0000C	IL24	1:2068974	15.02	33.6869	8.69526	8.69063	9.5423	6.34041	5.80008	5.36149	6.13075	9.67431	0.00815	-1.18301
ENSG0000C	KCMF1	2:8497109	14.5375	9.07158	6.64279	4.59838	5.04272	13.9265	14.969	6.74964	2.2409	6.25476	0.99565	0.004101
ENSG0000C	EMP3	19:482964	326.401	322.06	365.492	336.488	350.546	278.744	395.574	336.826	262.935	97.1842	0.3113	-0.3286
ENSG0000C	RP11-398	6:7320908	1.62659	1.59736	1.31736	0.820194	1.47383	0.468542	1.50213	1.40028	0.713221	4.83761	0.74505	0.334779
ENSG0000C	INPP5K	17:149457	56.4729	70.2507	78.0848	94.3586	65.3915	28.5127	48.4829	55.6029	39.8372	62.6517	0.0227	-0.65511
ENSG0000C	RSPH9	6:4364504	0.625349	0.391348	0.902638	0.769015	0.283668	0.530586	0.068592	0.365372	0.817117	0.295108	0.68525	-0.67802
ENSG0000C	PHF20L1	8:1327753	49.4754	43.351	42.0628	32.5686	44.8754	61.7122	55.4309	35.5036	33.837	60.1781	0.72635	0.13561
ENSG0000C	THAP7-AS	22:209991	2.73775	5.40765	5.68252	1.67754	3.35132	0.072845	3.59576	2.97362	1.94434	1.56741	0.5467	-0.94412
ENSG0000C	AC013264	2:1969670	8.98018	10.2456	6.52342	4.07177	2.70612	2.11972	1.10812	7.02061	0.000196	6.03542	0.16715	-1.39016
ENSG0000C	NT5C2	10:102918	25.6899	18.057	14.5521	18.0129	7.56863	13.0432	15.9722	14.2254	9.46147	11.6126	0.2125	-0.40111
ENSG0000C	SORD	15:450231	1.05788	2.93072	2.75331	1.24545	2.60339	1.71568	0.922322	4.41767	1.23188	3.44662	0.8977	-0.10301
ENSG0000C	AL162151.	14:989733	3.03578	16.8556	14.5073	23.3311	9.46119	15.3347	7.51904	7.74539	14.3827	0	0.823	-0.38575
ENSG0000C	MPO	17:582698	0.062522	1.1381	0.378073	1.35281	0	0.984509	0	0	0	0	0.43365	-1.6224
ENSG0000C	PFKFB2	1:2070343	0.289687	0.5531	0.070693	0.183462	0.299775	1.68289	0	0.385615	3.03389	0.165386	0.025	2.55758
ENSG0000C	CTD-2574	16:298061	0.521637	0.648872	1.00661	1.43775	0.392389	0	1.33098	0.117117	0.514818	0.387423	0.63395	-0.67219
ENSG0000C	IWS1	2:1274362	39.2688	38.3846	34.5357	25.0816	59.0063	41.6603	49.5527	27.934	21.1377	74.1166	0.76355	-0.10126
ENSG0000C	SLC26A4	7:1076539	0.043436	0	0.076598	0	0.006882	0	0	0	1.36544	0	0.2407	4.58235
ENSG0000C	EGR1	5:1384654	5.2421	16.9619	2.33424	4.85477	1.4036	3.48885	3.40751	52.225	16.8561	18.6147	0.0036	1.81879
ENSG0000C	SETDB2	13:494443	13.9541	10.4416	4.66518	4.23604	3.72084	11.28	7.16934	3.13853	4.13541	9.02176	0.58885	-0.29994
ENSG0000C	WDR91	7:1351838	5.6721	10.2832	3.70156	3.08342	2.6181	4.23327	5.18068	3.06069	7.79769	6.67025	0.7492	0.133986
ENSG0000C	BCAP29	7:1075795	25.0518	17.6898	18.848	18.4412	27.5725	41.9128	47.3796	8.78573	18.7823	12.5017	0.64895	0.206311
ENSG0000C	RPL12	9:1274476	228.952	297.056	590.128	420.436	217.225	209.045	361.453	422.952	298.18	49.1627	0.24185	-0.42702
ENSG0000C	ATP5J	21:256392	221.357	164.418	282.479	226.895	162.114	315.775	284.946	246.955	231.991	37.8747	0.8914	0.058641
ENSG0000C	LACTB2-A	8:7060857	4.47367	3.59165	2.93114	4.72558	1.62657	4.018	3.42486	0.795493	0.01199	0.848864	0.59085	-1.02426
ENSG0000C	BCAT1	12:248100	0.447747	1.05843	0.045471	0	0.075473	4.21198	2.43703	2.13994	0.242605	0.82744	0.0363	2.36255
ENSG0000C	MRPS2	9:1354951	37.8033	41.2863	33.9707	40.0845	55.3557	35.5929	25.1539	24.4388	11.6054	19.995	0.023	-0.90537
ENSG0000C	COPS9	2:2401265	187.112	90.2925	298.819	202.183	72.9241	267.209	229.247	215.911	151.664	22.894	0.9243	-0.03469
ENSG0000C	MFSD4B	6:1112593	1.75724	3.56425	0.707889	0.304466	0.400723	1.5738	2.46864	0.495281	0.411925	1.20487	0.9111	-0.06025
ENSG0000C	C1QTNF3	5:3398617	2.49082	0.869786	2.06288	1.68221	0.51465	0	0.018888	1.7564	0	2.58285	0.51285	-0.99634
ENSG0000C	SIVA1	14:104753	117.039	85.1402	153.681	153.777	78.0861	173.883	218.494	187.475	156.593	26.0732	0.5101	0.248091
ENSG0000C	SC22CB-1	22:320394	0.478125	0.101855	0.708569	1.06463	0.246843	0.42334	0	0.406795	0.490614	0	0.58185	-0.96929
ENSG0000C	TTC28	22:278516	0.153802	0.736516	0	0.003176	0.012631	1.59988	0.152048	0	0	0.220829	0.3631	1.54641
ENSG0000C	RP11-697	17:376097	1.12096	1.36895	0.517803	0.926748	0.237102	0.399528	0.685623	0.399988	0	0.606356	0.8975	-0.15772
ENSG0000C	RP11-114	12:693265	0.293146	0.050418	0.592901	0.53071	0.170898	0	0	0.915539	1.42106	1.42203	0.1648	1.03421
ENSG0000C	KHK	2:2708674	5.02672	5.13393	5.16617	3.47584	5.06641	10.8404	7.04599	3.83049	3.16861	4.02234	0.86075	0.094978
ENSG0000C	PGM2L1	11:743303	4.6025	11.5112	2.13984	1.42429	2.25207	10.8459	15.6356	2.63864	2.08683	6.3165	0.3812	0.743057
ENSG0000C	SNRPD3	22:245404	84.815	60.0006	87.257	95.8937	61.0083	133.551	110.733	71.5689	71.6146	24.8877	0.96705	-0.02324
ENSG0000C	THUMPD1	16:206102	37.5441	40.1978	41.4657	33.4773	34.6008	24.3499	22.048	29.1902	24.5961	51.4501	0.29085	-0.45388
ENSG0000C	KRCC1	2:8802720	45.75	22.7879	35.7679	38.0669	34.3576	32.2051	40.3431	26.013	37.5204	16.8355	0.56	-0.20748
ENSG0000C	TMEM5	12:635589	15.7521	11.6554	18.877	17.7779	12.1959	18.2262	18.6957	17.8358	19.3259	15.9425	0.5944	0.255654
ENSG0000C	TRIM22	11:566355	137.575	138.129	135.464	140.833	83.785	141.126	236.525	235.99	149.629	126.33	0.2551	0.420959
ENSG0000C	LRRC46	17:478316	0.445411	0.806687	0.551663	0.729737	0.459146	2.88852	2.23075	0.42575	0.149785	0.379042	0.35305	0.986964
ENSG0000C	TTK	6:8000388	1.77131	1.82875	0.116281	0	0.175246	5.01743	3.08179	0.661479	0.335927	1.44715	0.07985	1.57634
ENSG0000C	KLHL8	4:8716010	7.93922	6.45416	3.60323	2.77157	2.46577	1.68553	3.11053	2.07633	6.02127	3.53426	0.4494	-0.40177

ENSG0000	PRKDC	8:4777310	14.4586	18.4423	6.34963	8.50017	16.1917	12.1189	13.2883	6.74568	8.98441	16.7169	0.76585	-0.17675
ENSG0000	BCRP3	22:246329	0.321167	0.855221	0.229116	0.16375	0.682306	1.01511	0.261403	1.17625	0	0.09583	0.83225	0.227958
ENSG0000	WDR61	15:782505	52.5507	56.6602	74.1256	75.9882	51.2084	55.4413	56.8051	45.911	60.9531	19.5145	0.37395	-0.37558
ENSG0000	MANEA-A	6:9557518	2.16971	2.89818	0.850085	1.12455	0.998175	0	2.04239	0.834927	1.07914	1.55644	0.5589	-0.42411
ENSG0000	IQSEC3	12:66764-	0	0.793719	0.03182	0	1.8156	0	2.02679	0	0	0.572221	0.97815	-0.03858
ENSG0000	IGHV2-70	14:106770	0	0.080635	0.099279	96.7756	0	0	0	0	0	0	0.00005	#NAME?
ENSG0000	CACNA1C	12:194605	3.77035	3.40406	7.02367	11.2242	3.93877	0	2.63688	3.74633	0	1.64905	0.24585	-1.85601
ENSG0000	RP11-452.	1:2465664	0.383653	0.471514	0	1.13015	0	0	1.18041	0.982931	1.52861	0	0.5124	1.32982
ENSG0000	MYL12A	18:324748	1995.94	1429.26	2443.07	2669.46	1575.26	1890.99	2117.09	1980.99	1772.69	419.416	0.58495	-0.2994
ENSG0000	ST6GALNA	9:1279078	10.8681	5.76369	11.9126	12.5886	21.7782	18.7666	14.7373	12.5132	9.64688	20.4467	0.67865	0.187327
ENSG0000	PRSS35	6:8351253	8.24985	0	17.2401	10.2623	6.40423	2.52988	0	1.97242	15.0627	8.13237	0.224	-0.68666
ENSG0000	ZNF793-A	19:374971	0.926146	0	0.102593	0	0.236136	0	0.975225	0	4.92601	0.191752	0.10915	2.57178
ENSG0000	KDELR2	7:6409125	34.7265	31.9496	46.8021	30.8026	21.0219	45.7176	41.9295	43.7442	26.0484	22.1475	0.91395	-0.04121
ENSG0000	RTTN	18:700037	1.94765	1.86429	1.03006	0.45835	0.750609	2.3673	0.91764	1.21818	0.986669	1.67534	0.58665	0.378943
ENSG0000	C12orf66	12:641863	7.6965	4.74633	8.09914	10.7535	5.00184	0	1.41377	4.5274	9.77984	4.75382	0.37375	-0.96395
ENSG0000	SRGN	10:690881	405.939	222.389	662.663	1056.34	430.483	839.704	899.25	1346.65	1634.03	186.109	0.1565	0.786387
ENSG0000	DDIAS	11:828235	1.47878	5.6103	0.20771	0.378477	0.09716	0	1.53276	0	0	1.15615	0.3222	-1.41907
ENSG0000	PAFAH1B1	17:259320	12.5709	15.8118	11.4734	8.87545	10.3659	11.3632	16.485	13.7816	13.0135	19.6737	0.9828	-0.01164
ENSG0000	CTD-2547	14:912582	1.16573	1.29229	0.774381	1.25032	0.92653	3.26482	0.779075	1.24735	4.33872	3.93129	0.56475	1.41655
ENSG0000	LINC0085	3:1024802	4.25025	1.53823	1.74258	2.51286	2.78601	2.05951	1.93944	2.15858	1.9786	8.84491	0.71845	0.358554
ENSG0000	AC009506	2:1593189	0.858832	0	0	0.920885	0	6.99797	0.301536	0.742071	0	0	0.49865	1.99226
ENSG0000	C17orf100	17:665171	4.22658	2.64951	3.48261	1.62807	3.20244	6.6145	4.72757	3.03631	4.88081	3.02845	0.492	0.538394
ENSG0000	CEP44	4:1742366	7.08947	5.50347	2.53612	0.92059	5.28744	12.9147	4.22835	4.67354	5.10645	6.49194	0.3423	0.623905
ENSG0000	AC079922	2:1126418	2.33059	0.630723	0.950922	4.11624	1.93747	1.23948	1.63339	2.30792	0.653536	1.32832	0.47235	-0.56547
ENSG0000	SSR4	X:1537857	247.609	111.589	502.821	509.881	320.856	308.267	293.36	355.961	274.306	95.3503	0.3928	-0.41905
ENSG0000	CAP2	6:1739321	0.528523	0	0.249382	1.45879	0.451469	0	0	0	0	0.979013	0.3484	-1.69325
ENSG0000	C3	19:667770	0	0	0.433249	0	0	8.65791	0	0.008586	0	0.336684	0.09925	3.22952
ENSG0000	TMEM214	2:2703290	35.1204	23.4555	22.7384	13.3539	26.0482	15.5114	28.5707	20.9165	11.0435	34.7505	0.7538	-0.10049
ENSG0000	NAB2	12:570888	1.42373	1.78243	2.69612	0.602954	1.80142	6.50669	7.3828	11.136	4.33974	7.93912	0.0404	2.147
ENSG0000	HMGN2P5	15:296993	5.38693	9.80847	3.20519	3.20937	0.863896	15.1626	14.2594	2.24858	4.21098	0.759137	0.7718	0.692127
ENSG0000	LINC0048	9:9111906	0.068384	0.13721	0.056859	0.280894	0.350879	0.314734	1.89497	0.868876	0.589302	0.144564	0.1639	1.94042
ENSG0000	SSR3	3:1565401	22.9326	15.4187	26.4861	21.0172	17.8038	69.8237	43.2595	38.84	33.9259	19.5643	0.23345	0.617308
ENSG0000	LAMTOR4	7:1001489	199.638	192.707	334.406	299.216	156.121	309.273	232.008	246.922	252.699	60.262	0.69715	-0.14048
ENSG0000	RP11-286	11:614026	0	0.437877	1.19135	2.82955	0.316041	0	0	0.137153	3.54143	1.4238	0.94615	0.131602
ENSG0000	PROS1	3:9387303	0	0	0.041382	0	0	3.57373	0	0	0	0	0.4754	6.11314
ENSG0000	ARHGAP2	5:1427703	0	0	0.120838	0.158619	0.624975	0.291355	0.762247	0.13406	2.02858	0.136423	0.48775	1.51051
ENSG0000	SPN	16:296629	116.588	125.121	69.8615	36.5975	115.464	72.1056	109.636	67.4572	23.3427	115.85	0.3763	-0.32326
ENSG0000	RPS7	2:3575204	266.097	200.994	455.862	295.947	224.644	269.596	363.817	347.476	214.699	36.7069	0.3856	-0.31754
ENSG0000	NQO2	6:2987966	12.332	5.34034	36.8079	32.6689	26.4735	14.8975	5.10186	22.3336	20.971	9.56108	0.397	-0.69458
ENSG0000	BCL2L1	20:316644	24.5736	7.75062	16.9606	24.2839	31.7833	9.7645	21.2236	26.6725	29.284	29.6422	0.8055	0.094647
ENSG0000	DHX32	10:125823	11.196	8.28601	7.17148	7.71993	6.54286	3.09565	3.61065	5.40044	12.8368	5.43198	0.51675	-0.48443
ENSG0000	VPS29	12:110491	126.586	118.802	182.476	185.232	89.5508	187.503	186.221	177.79	172.52	145.703	0.36615	0.304815
ENSG0000	LAMP2	X:1204278	40.9853	41.4757	20.0915	17.2653	17.2497	89.6384	38.2713	23.0252	17.1764	21.0057	0.3308	0.387326
ENSG0000	EIF4E3	3:7167541	21.8835	13.9493	12.7075	4.15657	12.9108	21.3236	23.3936	16.076	12.908	23.7821	0.64645	0.17007
ENSG0000	IGFBP4	17:404436	16.5124	7.70505	22.8652	20.4344	16.0684	5.10033	3.25821	16.5946	11.2645	14.1467	0.12885	-0.70619
ENSG0000	DSCR9	21:372085	0.6155	0.528958	1.14842	0.121047	0.036083	0.322073	0.547885	0.250172	0.455664	0.593093	0.78995	-0.31795
ENSG0000	BAG5	14:103553	8.87238	11.6715	8.73147	8.70371	12.5899	7.97817	14.7386	10.8185	12.5957	23.3684	0.9053	0.075843
ENSG0000	PSAP	10:718162	305.28	536.459	264.825	318.226	276.915	1127.38	171.853	185.268	294.706	255.426	0.64925	0.246554
ENSG0000	UBE2I	16:130554	68.2627	80.3175	165.989	160.924	126.416	75.5793	98.4	153.11	121.086	92.0812	0.4975	-0.27065
ENSG0000	RIOK1	6:7268305	23.2132	18.4434	24.3068	26.563	23.9055	18.1498	20.7261	19.1179	16.3544	23.5133	0.66625	-0.3248
ENSG0000	TYMS	18:596987	23.4592	34.8996	1.43683	3.24668	6.5525	47.2348	23.3902	11.8731	3.99512	15.5997	0.47025	0.596626
ENSG0000	CFP	X:4762421	2.0645	14.6497	1.07662	0.771435	1.63621	6.31713	1.44102	1.11934	1.04382	1.32774	0.48525	-0.61103
ENSG0000	PUS1	12:131924	7.82087	7.89616	10.6823	10.8001	15.7499	10.2846	12.1276	11.2994	7.6278	14.492	0.96445	-0.02347
ENSG0000	PJA1	X:6916085	35.5522	23.6431	29.071	29.462	36.9326	10.007	21.1418	24.8421	11.2951	23.108	0.05475	-0.76418
ENSG0000	RPA4	X:9668466	0.619153	1.66134	0.590553	0.178125	0.086877	1.43892	0.154804	0.61359	0.039182	0.73828	0.96725	-0.06579
ENSG0000	MAF	16:795858	18.4932	6.23083	21.5304	18.3375	20.508	24.547	20.6673	25.7793	23.4768	42.6532	0.339	0.427585
ENSG0000	NUDT5	10:121296	60.0151	47.2876	53.837	53.4341	42.8633	101.281	81.7505	69.3886	60.879	31.5899	0.4877	0.371009
ENSG0000	BYSL	6:4192118	8.27125	2.93967	10.0263	7.9601	8.67278	11.0213	6.65933	7.42966	1.21781	7.02995	0.52835	-0.32452
ENSG0000	PPP1R7	2:2411060	104.588	81.682	91.5298	97.0456	137.967	94.4334	127.505	91.5102	95.4927	63.3462	0.9076	-0.04392
ENSG0000	EMC4	15:342249	118.959	114.617	221.143	222.536	146.325	153.585	139.528	156.322	152.736	53.7217	0.32455	-0.37197
ENSG0000	KBTBD8	3:6699830	1.17832	1.42732	0.425651	0.410026	0.583705	4.44486	1.55135	0.804679	0.102135	1.1303	0.28645	0.689432
ENSG0000	RAB3GAP	1:2201482	9.98594	8.06989	3.60991	3.59117	2.98876	8.4358	7.05224	6.74789	5.36914	10.1036	0.24445	0.421705
ENSG0000	NUMB	14:732751	31.2278	47.9141	16.0489	20.6468	9.91028	39.3062	32.4695	12.9091	28.2768	29.276	0.5098	0.245453
ENSG0000	SCARNA1	12:649188	0.380216	1.55182	0	0	0	0	0	0	0	0.5971	0.86815	0.306583
ENSG0000	ARMC6	19:189908	27.9654	22.5501	28.3691	34.5394	23.0761	15.4887	18.9796	16.9674	16.9639	19.364	0.2664	-0.64761
ENSG0000	KPTN	19:474751	4.32068	4.55625	1.97607	4.82312	2.43271	7.0508	4.71246	4.19391	1.19974	2.6484	0.84625	0.130454
ENSG0000	LCP2	5:1702324	185.317	151.935	54.87	54.5185	60.345	194.998	159.668	102.951	77.7435	126.825	0.4279	0.310021
ENSG0000	PTPRCAP	11:674355	934.146	470.735	1386.7	980.637	1337.88	911.393	1196.82	1236.66	768.752	413.821	0.58925	-0.23915
ENSG0000	RASA4CP	7:4402695	10.1949	12.8366	8.4647	7.56884	10.8617	5.47299	7.69253	9.20163	6.44906	9.17276	0.6638	-0.38627
ENSG0000	RP13-297	X:1732583	0.515144	2.09608	3.19941	3.9805	0.846755	1.20969	2.08565	0.766438	0	0.413002	0.25385	-1.38883
ENSG0000	TRIM27	6:2890300	43.9747	43.1236	36.5938	40.6897	32.4071	25.5126	25.237	27.3552	20.235	30.7389	0.11475	-0.50845
ENSG0000	CPNE1	20:356260	141.008	127.078	290.256	200.801	233.012	119.347	110.136	192.308	157.904	180.04	0.36815	-0.35495
ENSG0000	FADD	11:702031	18.6796	15.8421	14.6915	6.27972	14.3725	20.6202	27.592	12.5616	11.4604	10.9754	0.6203	0.220003
ENSG0000	MITF	3:6973943	0.206121	0.862406	0	0.507323	0	3.59581	0.045806	0	0	0	0.4429	1.0815
ENSG0000	CTB-113I	5:1340051	1.5426	2.64239	1.79321	2.73918	0.642203	2.28135	0.875864	1.17042	1.44658	0.897851	0.8691	-0.34998
ENSG0000	TREML2	6:4119027	1.70317	3.20785	0.378468	0	0.816306	3.68371	0	0.630576	0	0.70743	0.75875	-0.23455
ENSG0000	HCRTR2	6:5510645	0	0	0	0	0	2.93546	0	0	0	0	0.11955	inf
ENSG0000	ATP8B4	15:498582	0.663382	1.54041	0.328047	0.245666	0.162411	4.19311	11.1912	2.55521	0	0.721258	0.01255	2.62312
ENSG0000	NDUFS5	1:3902631	181.719	155.763	298.52	327.783	106.938	374.958	398.149	327.671	281.783	37.2027	0.3252	0.347146
ENSG0000	TPM1	15:630426	7.14731	11.37	5.39405	9.78629	6.83022	7.66445	10.9567	10.5111	9.30855	9.6553	0.54475	0.283863
ENSG0000	RP4-605C	12:501119	2.87886	5.97566	4.70646	8.14165	3.48746	5.21413	3.95583	1.31843	0.115471	1.0002	0.2402	-1.2266
ENSG0000	MIEF1	22:394994	9.60929	8.26812	13.6228	5.24579	12.2135	5.99731	6.60985	9.90391	11.9707	19.4352	0.9213	0.040589
ENSG0000	MAP2K4	17:119983	18.5478	12.4628	9.451	12.2382	12.793	16.4733	11.5377	17.6879	12.4545	21.3035	0.78565	0.10258
ENSG0000	CHD6	20:414021	36.5609	63.2668	43.8558	35.6123	68.6108	61.9845	69.5137	28.7053	31.3282	48.2833	0.83255	-0.13226
ENSG0000	CCRL2	3:4640716	8.20313	7.74694	7.0369	8.32371	6.12983	43.0217	19.3041	12.987	10.566	18.7153	0.0084	1.34294
ENSG0000	ZNF470	19:565675	0.904664	1.95961	0.482279	0.285302	0.287767	3.17598	0.805016	0.895513	0	0.851733	0.50955	0.688284
ENSG0000	TRIM11	1:2283936	23.8348	22.0251	15.7436	12.2327	19.0904	3.82846	17.6886	18.7634	9.2627	31.1653	0.83745	-0.08259
ENSG0000	ZNF354C	5:1790604	1.61452	1.32401	0.191088	0.139891	0.365711	0.243656	0.538254	0.418901	0	1.08113	0.53095	-0.85094
ENSG0000	CD96	3:1112927	154.003	89.7803	134.745	148.922	101.27	177.612	206.497	176.764	210.41	202.292	0.31155	0.491959
ENSG0000	MIIP	1:1201946	54.546	62.8043	45.1935	46.7116	49.9325	56.7203	105.342	59.3476	70.0688	52.6869	0.284	0.372799
ENSG0000	WASH3P	15:101961	5.82957	9.27758	6.09683	8.33925	3.11484	10.9454	9.61247	5.38832	5.29148	3.79794	0.91955	0.098984

ENSG0000C	MTERF2	12:106955	24.5039	18.5519	17.1757	14.1522	7.71507	10.4689	7.69754	12.5572	6.81089	8.82693	0.253	-0.76758
ENSG0000C	FRMD4A	10:135869	0.419362	1.7033	0.471507	0.05105	0.319518	0.149813	4.29174	0.402169	0.565635	1.08648	0.2245	1.2347
ENSG0000C	B4GALT1-	9:3310408	2.92476	0.575113	1.82861	1.63782	1.14472	2.8029	1.20347	0	0	0	0.6228	-1.12728
ENSG0000C	TMEM110	3:5281297	6.64572	4.26861	4.91532	5.11578	7.54335	11.1096	22.3097	10.9057	4.70156	6.54973	0.17335	0.824973
ENSG0000C	MRPL11	11:664350	33.9146	26.7785	42.7273	42.3565	30.4587	39.7526	42.3028	43.2485	28.4971	7.71003	0.73375	-0.23187
ENSG0000C	RP1-28O1	1:2096613	1.43706	0.213105	1.61634	1.00926	0.554545	0	1.51674	0	0	0	0.43125	-1.76833
ENSG0000C	LMNA	1:1560825	18.3672	41.6232	4.3053	21.4687	2.34152	54.4289	105.071	100.298	187.899	10.5873	0.00005	2.40146
ENSG0000C	PNN	14:391748	65.9281	89.0607	60.5977	52.1462	89.5086	78.6015	87.4548	61.4022	51.3263	127.646	0.8399	0.061479
ENSG0000C	RAP2B	3:1531622	5.36744	5.68356	12.5297	8.09202	9.10193	3.74913	2.42714	5.71393	5.91778	7.49864	0.0286	-0.84112
ENSG0000C	BCL11A	2:6045116	0.18984	2.38204	1.6731	1.37383	0.720643	0.342173	0	0	0.62307	0.226626	0.04715	-2.74023
ENSG0000C	ANTXR2	4:7990114	21.4188	13.2923	1.11538	10.9687	4.86573	21.336	13.0705	5.10667	3.75392	7.24194	0.43065	-0.43221
ENSG0000C	DDX28	16:679873	12.1681	24.2415	27.1558	19.6309	25.5673	12.0468	7.54714	8.68625	6.08567	12.4666	0.3907	-1.25849
ENSG0000C	HEXB	5:7462740	81.2115	48.8679	59.2456	53.8967	44.8384	210.568	68.5455	54.1063	45.4893	38.71	0.22725	0.51126
ENSG0000C	RFXAP	13:368192	5.85193	7.55938	2.11935	2.73905	3.22513	0.406934	8.04814	6.2242	4.16824	5.05862	0.7361	0.206015
ENSG0000C	RBPMS	8:3038211	0.384818	0.702796	0.312238	0.015165	0.335279	0.481727	0.324718	0.685038	0.245078	0.72071	0.50705	0.484799
ENSG0000C	FUT4	11:945438	1.49547	1.50552	1.1766	1.19312	2.82995	1.47623	0.636094	2.58641	0.974151	4.28598	0.75225	0.192361
ENSG0000C	KIF3C	2:2592659	0.432029	0.595939	0.914342	0.009184	0.735601	0.169091	0.298711	1.24973	0.24074	1.74351	0.57525	0.391617
ENSG0000C	KRT17P6	17:205125	0.434956	0.733355	0.038549	0.167072	0.031828	0.355763	1.29326	0.42977	0.04536	0.404918	0.5003	0.922219
ENSG0000C	SSSCA1	11:655704	44.2585	37.4287	95.5115	97.1277	49.6826	65.931	60.8636	82.9012	47.4913	19.4146	0.7471	-0.35129
ENSG0000C	CTD-2538	19:326870	1.03212	0.896121	2.93531	1.39481	2.08016	0	1.97604	1.20997	0	0.333881	0.49565	-1.36859
ENSG0000C	SNRPE	1:2038616	105.198	93.9254	142.148	142.442	51.3614	118.008	135.785	125.122	106.097	24.7745	0.94285	-0.02722
ENSG0000C	SLFN12L	17:354642	17.6495	8.04661	15.1376	19.2622	12.2338	15.592	27.3895	17.5347	27.2679	20.3384	0.1479	0.563886
ENSG0000C	SAP30L-A	5:1539901	1.98955	1.80286	0.395935	1.12268	1.34311	3.91641	3.28864	1.56594	2.89803	4.21498	0.19545	1.34875
ENSG0000C	ZCCHC14	16:874062	0.893595	2.20778	1.36079	0.557643	1.72684	0.495195	0.572513	0.615419	1.30999	2.14459	0.44585	-0.53062
ENSG0000C	C17orf50	17:355747	0	0.232958	0	0.922344	1.42627	0	0	0.863278	0	1.00088	0.7665	-0.55924
ENSG0000C	RP11-519	15:454305	0	0	0	0	0	3.33018	0.460801	0.165478	0	0.383259	0.0003	inf
ENSG0000C	GABPA	21:256392	12.1866	10.2673	2.76366	3.26644	3.76335	7.3249	8.03838	8.67754	3.386	7.84963	0.8988	0.12896
ENSG0000C	HPCAL4	1:3967864	1.18627	1.48743	0.150556	0	0.193451	0	1.909	0	0	0.37277	0.835	-0.38445
ENSG0000C	RP11-159	17:579890	0.649624	1.72746	0.724553	1.99759	0.322047	5.08902	2.25854	0.613608	1.35234	0.607774	0.55215	0.936159
ENSG0000C	RP11-305	10:303028	0.192671	0.762165	0.195114	0	0.218124	0.886559	0	0.231059	1.83798	0.460125	0.48175	1.29121
ENSG0000C	HLA-L	6:3023403	0.962829	0.876495	0.477238	0	0.49573	1.09806	1.54539	0.806452	0.164123	0.497682	0.8194	0.271466
ENSG0000C	AK2	1:3298695	58.175	50.763	77.0719	51.3186	59.1753	68.8049	83.4529	71.5245	37.6962	35.05	0.69585	-0.17469
ENSG0000C	ABCA3	16:227588	1.52575	1.49441	1.60754	1.48453	3.23796	3.12697	2.69486	2.43419	3.87359	5.17431	0.5396	0.602863
ENSG0000C	FNDC3A	13:489749	7.2344	8.62509	2.62595	3.65298	3.50225	6.45544	3.70725	5.00142	2.50715	7.16327	0.4184	-0.3362
ENSG0000C	PRR19	19:422970	0.143645	0.023503	0.731422	0	0.743231	0	2.01989	0.757729	0	0.047685	0.80865	0.384991
ENSG0000C	FTH1P10	5:1735369	0.371849	0.250303	1.14898	1.70781	0.678671	1.01426	0.699029	1.0909	1.44787	0.594388	0.7819	0.4498
ENSG0000C	RP11-186	17:756187	4.93873	5.80105	2.1944	9.74543	3.17548	6.77222	3.15865	6.16083	7.38479	6.37931	0.91005	0.145284
ENSG0000C	FH	1:2414976	23.759	17.0403	23.085	17.0707	15.7575	28.4945	10.9189	11.9278	17.9463	10.5839	0.49425	-0.27881
ENSG0000C	LONP2	16:481649	11.9149	17.0516	6.47724	7.12225	8.38095	8.74879	9.98902	5.46268	5.00932	18.1247	0.386	-0.43749
ENSG0000C	GPATCH4	1:1565944	34.8406	38.7091	60.2581	38.2883	113.017	26.5351	30.7489	33.8619	23.9337	78.5323	0.0852	-0.63004
ENSG0000C	MAP2K5	15:675427	11.7851	11.9413	5.99263	5.25014	7.77857	4.80265	9.16193	7.22354	13.0696	10.1076	0.8565	0.081711
ENSG0000C	ADAMTS1	15:999702	0.267484	0.372663	0.100367	0.439887	0.248526	0.948511	0	1.66961	0	3.09368	0.08505	1.9305
ENSG0000C	MAGED2	X:5480759	80.8151	93.7038	120.005	89.999	93.6978	75.2593	141.872	119.515	110.05	78.0778	0.606	0.163149
ENSG0000C	CCND2	12:424876	38.819	41.2782	13.2578	9.93666	23.9703	33.7655	53.5474	22.5684	14.9996	41.9327	0.2209	0.400068
ENSG0000C	PARGP1	10:458540	3.15245	6.5082	1.15424	1.09292	0.549966	1.62182	0.864698	1.33248	0.203123	1.34744	0.3197	-1.08492
ENSG0000C	HYI	1:4338988	2.75138	3.89544	1.55859	2.92535	3.81492	6.12655	1.06196	4.98495	0.237484	0.636322	0.7133	-0.31006
ENSG0000C	JMY	5:7923618	4.10881	3.77602	2.75599	1.29182	3.64117	3.24782	4.83221	3.98624	3.64296	6.11717	0.2114	0.454922
ENSG0000C	EHD4	15:418280	3.07462	5.68067	3.46743	3.82402	3.17266	5.09122	6.90467	7.34952	10.4502	5.41377	0.2536	0.875886
ENSG0000C	FBXL16	16:684621	0.990248	3.2888	0.279753	0.145458	1.14653	2.21863	2.73053	1.94108	1.37437	3.03527	0.4192	1.0251
ENSG0000C	BRAP	12:111642	15.6516	15.2387	15.8029	16.0314	10.8765	14.6226	9.18015	11.1536	8.91154	10.4606	0.09635	-0.65207
ENSG0000C	KCNK6	19:383198	2.91519	5.37216	2.54187	2.31546	1.84887	5.8624	4.41586	7.5075	5.29912	10.8571	0.01375	1.1562
ENSG0000C	ACAD11	3:1325581	10.9596	7.05211	4.73441	11.7951	1.51249	7.97682	5.62061	5.08243	3.39388	7.382	0.8413	-0.15106
ENSG0000C	RP11-139	4:1574061	4.99676	4.43777	12.0511	7.01969	9.57229	0.806011	1.60736	10.1395	7.58577	1.39768	0.315	-0.74869
ENSG0000C	NRAV	12:120469	1.56155	0.39117	0.874059	0.455944	1.06655	5.89458	1.34602	1.23907	0	0.686581	0.50235	0.988867
ENSG0000C	AGPAT5	8:6708356	11.2846	10.2972	6.65303	8.45953	3.79213	10.1136	9.52505	7.90657	14.3411	8.1162	0.4855	0.284125
ENSG0000C	BTBD1	15:828369	48.6424	39.6724	13.4911	34.5036	7.42831	38.9452	31.0572	23.4979	21.7047	10.8627	0.8709	-0.07386
ENSG0000C	ZNF184	6:2745074	11.5544	8.02153	4.7335	5.29839	2.75378	4.24226	9.62848	1.90518	5.77714	6.24518	0.64875	-0.21261
ENSG0000C	RPS15A	16:187812	546.915	385.241	625.616	301.052	121.281	574.888	584.26	421.437	251.568	31.2772	0.90325	-0.10281
ENSG0000C	DNAH1	3:5231631	3.09474	3.60906	2.12647	0.856536	1.18526	2.27384	2.07681	0.654973	0.466853	3.3618	0.6541	-0.33241
ENSG0000C	MYCBP	1:3883819	31.0329	23.8336	27.3713	30.4502	13.6163	42.7451	35.7038	23.8556	17.8715	7.08093	0.8779	0.064656
ENSG0000C	NDUFB1	14:921161	120.958	78.6658	117.214	116.118	33.7596	145.777	108.397	122.011	69.0111	15.1222	0.9669	0.024686
ENSG0000C	ZC3H12D	6:1494467	3.62247	10.7893	0.803761	0.575498	0.101457	10.8037	9.67453	6.07944	9.29077	3.84104	0.04495	1.5767
ENSG0000C	KCTD18	2:2004889	10.3332	12.2383	8.03606	9.43994	7.09089	5.59014	5.57397	6.59501	3.15636	10.226	0.23655	-0.65374
ENSG0000C	ATF7	12:535066	9.131	10.4582	9.83467	14.2533	6.32928	9.26494	15.7102	17.1819	18.7199	7.50209	0.4313	0.726557
ENSG0000C	TWF1	12:437937	22.3721	9.31025	6.91194	10.9917	4.0308	6.62734	19.2316	11.3022	6.05306	11.09	0.64505	0.192958
ENSG0000C	CTF1	16:308966	0.216927	0.536058	0.375962	1.18399	0.242273	0.665487	0.40835	0.24033	0	1.60573	0.90785	0.196908
ENSG0000C	RCOR3	1:2112583	18.7042	25.1628	9.85393	9.8531	9.69272	16.898	15.7201	15.6039	29.3184	18.3202	0.17565	0.478686
ENSG0000C	SNX14	6:8550549	46.9322	25.4153	16.6997	25.5251	11.4056	24.6298	21.8533	22.4113	32.1305	22.6475	0.67625	0.300896
ENSG0000C	PXMP2	12:132687	13.1062	11.496	9.37929	7.73756	19.0632	15.0539	13.319	12.7971	13.1993	5.46105	0.91545	-0.0845
ENSG0000C	CPSF1	8:1443731	32.8767	34.5802	12.356	9.13505	17.2154	14.8444	17.4005	19.5339	6.96486	23.4652	0.7753	-0.14033
ENSG0000C	VMAC	19:590484	3.12832	2.1815	6.41718	9.18348	5.58159	1.05593	1.93852	6.28407	2.85913	1.97173	0.48	-0.94728
ENSG0000C	HIST1H2B	6:2621516	1.72548	2.93079	42.9961	5.25855	9.27877	0	2.28611	1.73061	3.32567	3.16856	0.00045	-2.65309
ENSG0000C	AC004076	19:573634	0.368851	0.021173	0.167843	0.974906	0	1.19023	0	0.384632	1.47612	0.19639	0.4962	1.09782
ENSG0000C	BCL3	19:447477	29.8876	17.3151	12.0833	13.9128	29.5228	63.4143	121.1	47.3108	47.981	89.8749	0.00005	1.72593
ENSG0000C	RP11-369.	10:800468	0	0.070581	0.897928	0.810372	1.12126	1.7557	0.259778	0.369076	1.11141	0.274263	0.20815	inf
ENSG0000C	GOLGA1	9:1248783	6.80984	13.4898	2.10088	1.74893	3.76759	6.37813	4.4655	3.74449	0.249651	7.96255	0.6782	-0.2688
ENSG0000C	TRGV5P	7:3834157	6.24393	0.116861	1.48405	5.64483	0.72815	4.01894	0.587397	2.90927	0.922833	1.06589	0.89015	-0.2524
ENSG0000C	TJP2	9:6912126	4.18702	11.0998	0.011169	0.854033	0.286878	1.65751	1.20225	0.798222	10.322	0.920046	0.89445	0.101826
ENSG0000C	SEC22A	3:1230670	15.114	13.2775	14.9667	11.4327	10.4604	24.8715	16.4586	23.2564	11.4748	7.61649	0.5982	0.32639
ENSG0000C	RIPK3	14:243183	32.6315	39.2614	25.9387	37.9458	23.8267	34.2465	46.1701	28.8151	28.4573	38.637	0.69485	0.148663
ENSG0000C	ARSK	5:9555507	6.33968	3.96357	10.4208	9.31654	5.29235	5.11879	4.04221	5.51594	9.80113	3.67422	0.38455	-0.4706
ENSG0000C	CLNK	4:1048639	0.411693	0.863062	0	0	0.015922	1.00324	12.4165	1.94513	0	4.76981	0.03775	3.82001
ENSG0000C	TRAV16	14:219904	2.62512	11.7846	44.6186	13.3025	3.50846	12.4134	29.5477	12.9772	0	9.08471	0.72185	-0.27648
ENSG0000C	CTB-47B1	5:1572601	1.50688	5.26756	1.0906	0.774265	0.862775	3.34604	1.58909	1.6763	2.56767	3.60918	0.68285	0.427833
ENSG0000C	GTPBP3	19:173349	7.55847	9.54957	16.0916	16.5934	12.1653	8.06448	5.44197	13.3746	6.56111	12.081	0.27175	-0.48979
ENSG0000C	IL18	11:112024	0.070834	0.070119	0.042803	0.033592	0.316192	28.5354	0	0.247347	1.38551	1.04606	0.0165	5.68884
ENSG0000C	PSMD6	3:6400402	93.7778	77.4246	78.081	84.0384	69.5671	112.064	83.4506	65.3694	71.6754	46.3112	0.83075	-0.07659
ENSG0000C	ZDHHC7	16:849741	25.1846	30.4375	11.2679	11.1201	11.9271	12.5502	10.5605	9.84438	13.9177	23.336	0.2475	-0.40754
ENSG0000C	GPD1L	3:3210568	12.6439	8.84952	5.42267	5.48134	4.93486	2.83934	4.66162	8.28737	7.07232	9.10254	0.6352	-0.18613
ENSG0000C	CACYBP	1:1749991	88.3602	90.7756	98.5357	74.522	103.855	207.99	183.677	91.6016	81.0005	67.1631	0.59265	0.310053
ENSG0000C	PRR13P5	19:399432	36.2322	37.8499	61.5685	67.8183	36.4771	35.0805	37.2402	36.9574	38.1204	7.44383	0.40085	-0.61752

ENSG0000(	SPTSSB	3:1613447	3.01168	0.542468	0.043883	1.81846	0.9079	0	0	0	0	0.233191	0.24155	-5.14078
ENSG0000(	SCO1	17:106724	14.0884	14.4351	13.7059	11.2499	12.2539	12.8247	14.913	14.7705	13.2811	8.56236	0.73655	-0.17979
ENSG0000(	ANPEP	15:897848	2.3657	2.64306	0.180584	0.071685	0.296522	11.346	0	0	0.109	0.08452	0.48285	0.837593
ENSG0000(	CASP8AP2	6:8982989	19.8677	30.2661	23.3108	14.6519	30.9917	26.6915	19.7848	13.3752	18.1854	43.7861	0.74645	-0.1515
ENSG0000(	CYP27B1	12:577623	0.976175	0	0.79251	2.00406	0.43598	2.62766	0	0.635483	0	1.8151	0.95885	0.078791
ENSG0000(	NSUN5P2	7:7287936	10.8324	30.7239	10.7648	16.4518	11.7844	27.9837	26.199	20.7815	12.7975	16.7561	0.47325	0.44235
ENSG0000(	SUV39H1	X:4869555	5.29381	9.74329	6.26732	5.48288	6.52137	4.23301	6.18807	9.37301	7.3778	3.37983	0.95565	0.028131
ENSG0000(	RALA	7:3962348	25.9197	38.1845	12.0855	8.04029	15.6117	41.3859	38.4854	29.657	17.2192	16.8295	0.1282	0.588424
ENSG0000(	ANAPC15	11:719985	50.895	61.629	57.6122	79.2693	36.2125	65.7947	44.8955	39.3869	52.7749	12.411	0.61925	-0.41242
ENSG0000(	FKBP5	6:3557358	70.0458	116.029	20.9183	24.1571	13.1907	66.6594	25.9482	78.0264	55.4926	61.4563	0.40535	0.296177
ENSG0000(	CH17-340	X:1544243	3.19099	3.29524	8.04284	3.50218	4.88191	23.176	7.5787	6.60271	5.44846	1.3452	0.29815	0.75033
ENSG0000(	TRAV12-2	14:218878	20.175	59.256	9.56349	4.09437	8.35609	35.0093	32.7064	11.532	34.119	10.4131	0.55005	0.427557
ENSG0000(	IQCC	1:3220038	1.39646	1.08379	0.80345	2.25489	1.26386	1.62903	1.56001	1.14151	0	2.79891	0.9857	-0.02289
ENSG0000(	ZNF831	20:591233	9.57615	13.6216	6.70004	7.01347	5.04735	8.00237	9.66708	5.40695	4.06924	7.75911	0.68785	-0.24961
ENSG0000(	RP11-151	15:445370	0	0	0.203412	0	0.072384	0	0.902192	0	2.29454	0.334325	0.2649	3.5142
ENSG0000(	MGAT1	5:1807847	103.589	97.9071	89.7031	85.8785	94.4847	193.392	101.421	100.226	54.3328	142.149	0.4208	0.26751
ENSG0000(	POLR3F	20:183833	8.80016	5.72568	8.11251	6.91219	6.11717	7.77118	4.87048	7.68421	15.5551	4.20422	0.66625	0.210708
ENSG0000(	EPN1	19:556752	4.6675	6.67663	0.518432	0.638189	1.2405	6.95251	5.83879	0.633392	0.910985	1.64403	0.99545	-0.00495
ENSG0000(	SMAD1	4:1454811	1.06672	0.265178	0.696377	0	0	4.61961	3.96683	1.52845	0	0.883666	0.0493	2.414
ENSG0000(	WBSCR22	7:7368096	61.9439	77.2245	114.001	114.197	93.9073	88.8495	77.4267	97.1812	85.089	39.9705	0.4418	-0.28202
ENSG0000(	LINC0157	15:928827	131.497	122.861	66.2292	31.3406	27.5666	92.9309	145.388	57.2004	54.2673	24.5752	0.83115	-0.14679
ENSG0000(	RP11-523	13:196746	0	0.710903	0.452809	0.204409	0.164222	2.41218	0.339774	1.04898	0	0.383936	0.43805	1.44934
ENSG0000(	TRBV5-1	7:1423206	22.6444	82.7086	9.54829	8.24896	6.35454	61.0009	48.1022	8.76107	26.3562	30.7205	0.25865	0.589775
ENSG0000(	GDF11	12:557432	0.689091	1.55988	0.386511	0.377088	1.08018	1.0937	1.35763	0.922253	0.42844	1.73795	0.92485	0.143304
ENSG0000(	SSSCA1-A	11:655684	3.64674	0.931021	5.25693	4.37734	2.08975	2.75381	1.42613	5.4418	2.9247	1.84717	0.9131	-0.12101
ENSG0000(	ZNF169	9:9425931	2.5779	1.43327	2.15626	4.1436	2.41058	1.92519	2.78468	1.28274	13.4268	5.66075	0.12515	0.976489
ENSG0000(	AD000864	19:358876	4.52431	1.55698	3.81577	2.60984	1.89833	4.58352	3.73914	5.19605	2.27094	3.8289	0.7571	0.399534
ENSG0000(	DLST	14:748818	3.05632	1.36975	2.11202	2.74261	1.45866	3.53296	5.54044	1.5709	0.383004	1.71459	0.8155	0.151724
ENSG0000(	ERCC3	2:1272572	48.3284	47.881	31.3913	42.7625	26.1868	27.135	31.5978	27.4767	31.2824	31.1445	0.18415	-0.43206
ENSG0000(	STK39	2:1679540	26.1696	17.0513	6.78155	3.00966	11.7352	17.1016	8.72192	6.68417	6.5997	15.9039	0.71275	-0.15263
ENSG0000(	TRIM39	6:3023403	9.74154	9.7264	6.737	8.08833	8.38015	7.78818	5.49139	5.72472	4.01933	15.0543	0.637	-0.32367
ENSG0000(	TRIM16L	17:186979	0.446293	3.32111	0.61915	0.63148	0.660133	0.589257	0.110602	0.188189	0.058463	0.46814	0.0017	-2.17343
ENSG0000(	BEST4	1:4478358	0.23946	0.408678	1.13681	0.436752	0.450754	1.39048	0	0	0.14408	0.684292	0.8578	-0.32865
ENSG0000(	PBX3	9:1257473	5.69177	7.35501	1.91517	3.68433	2.66695	8.28271	3.91652	2.51394	4.99792	5.02888	0.5864	0.306884
ENSG0000(	AF127936.	21:148188	0.771144	1.03106	0.663055	0.470434	0.551648	0.108213	0	0.289068	0.801893	0.626775	0.8647	0.202135
ENSG0000(	SART3	12:108515	23.8676	29.2136	15.7762	18.5914	25.8041	23.6916	24.2131	25.7453	8.9273	32.5724	0.7883	-0.09333
ENSG0000(	RDH10	8:7329026	1.97335	2.01028	0.684166	0.893021	0.8179	5.41561	2.43654	1.20496	0	1.8163	0.58655	0.799708
ENSG0000(	BBS1	11:664350	10.5663	10.8682	8.5709	8.05797	6.71558	14.4833	13.388	2.3735	5.63413	8.27976	0.9378	0.055098
ENSG0000(	ZNF225	19:440943	2.13695	2.46797	1.46481	1.33115	0.992176	1.18828	2.03858	0.849112	0.113349	2.65315	0.6866	-0.38667
ENSG0000(	NOP58	2:2022657	118.234	121.3	154.772	141.17	148.751	177.893	171.676	145.099	134.195	156.382	0.5342	0.181459
ENSG0000(	BDP1	5:7145561	23.3468	41.9622	13.8532	18.5423	22.5894	37.8521	44.9807	17.5008	20.9208	49.7545	0.3654	0.36029
ENSG0000(	RAB11B	19:836328	70.3397	81.4403	42.3411	33.5912	52.7001	63.4646	75.9979	62.2412	46.9012	56.0068	0.7624	0.106086
ENSG0000(	BANK1	4:1014112	0.816583	15.886	1.90516	8.52035	2.52811	3.07819	0	0.378709	0	2.74464	0.1988	-1.74565
ENSG0000(	RP11-29B	13:102593	1.21139	0	0.559051	0.43982	0.263821	0	1.16056	0.854325	0.75231	0.18481	0.76385	0.508842
ENSG0000(	CAV1	7:1165247	0.38203	0.531994	0.0136	1.30644	0.443722	0	0	0	2.12411	0.585082	0.7932	0.328282
ENSG0000(	CCNI	4:7704715	112.696	114.518	110.769	69.6854	198.8	120.984	114.169	142.103	91.0184	213.583	0.5995	0.179012
ENSG0000(	IL1RL1	2:1023115	1.17364	0.582994	0.661311	0.411979	0.385915	0.800117	1.00745	0	0	0.65032	0.6691	-0.47506
ENSG0000(	TMEM44-.	3:1945840	6.60694	2.67833	6.16021	3.92582	1.71211	9.16951	2.1578	8.99923	4.33923	1.89221	0.8107	0.337835
ENSG0000(	BRWD3	X:8067085	1.35599	1.1477	0.538467	0.13943	0.903506	0.912536	0.678814	0.689684	0.553526	2.63687	0.9994	-0.00049
ENSG0000(	MYOM1	18:306680	0.458812	1.31666	0.05393	1.2796	0.230228	1.8171	1.1483	0	0	1.38844	0.5014	0.688895
ENSG0000(	TP53	17:766177	10.9766	11.6626	17.0503	10.2164	19.6286	8.29391	4.7708	9.86456	6.74962	21.54	0.3202	-0.51623
ENSG0000(	AL162151.	14:991584	0.098225	0.129186	0.080361	0	0	0.089437	0.451919	0.348147	1.05397	0.576341	0.22445	3.3002
ENSG0000(	SF3B6	2:2406758	136.651	88.0018	140.085	119.014	95.8667	185.559	181.54	132.039	106.264	22.2851	0.79515	0.088432
ENSG0000(	LA16c-43	16:161232	0.123851	0.065208	0.27279	0.085354	0.108994	0.150631	0.337212	0.540996	1.11194	0.716011	0.16675	2.21822
ENSG0000(	RP11-25K	8:5880541	11.1006	16.2003	8.98557	12.0587	10.0154	15.2121	18.7042	19.8345	0.858124	13.3255	0.87245	0.234136
ENSG0000(	ZNF761	19:534319	5.54076	3.98822	1.69336	4.90079	2.22525	4.83098	2.1992	3.72427	3.99303	2.74129	0.81815	-0.18502
ENSG0000(	P3H4	17:418019	3.72425	3.0096	8.45772	3.54304	10.4245	2.82744	4.77137	2.48856	4.10054	6.13954	0.43605	-0.51365
ENSG0000(	RP5-1159	7:7552461	0.402977	1.02963	2.83006	2.64133	1.42649	0	0.169943	0	0.518673	2.07676	0.58585	-0.7714
ENSG0000(	PALM2-AI	9:1096407	0	0	1.70817	1.09682	4.55876	4.34693	4.44773	2.11653	0.738806	5.89991	0.88005	0.152173
ENSG0000(	LMNB1	5:1267766	13.7886	15.2924	2.33877	8.11063	2.42603	18.8407	20.7854	7.3255	1.72385	6.88377	0.77595	0.18571
ENSG0000(	ZBTB4	17:745936	5.50137	7.00831	4.14831	1.34972	7.40276	6.83112	4.10694	5.01192	1.9285	12.0027	0.74505	0.143973
ENSG0000(	C7orf61	7:1004566	0.672376	0.314613	0.04946	0.245658	0.641321	0	2.25976	0	0	0.198481	0.94335	0.121295
ENSG0000(	ZNF160	19:530666	18.7916	16.2529	15.3993	23.7251	6.31654	10.6396	9.28055	10.4999	19.4673	11.9587	0.51215	-0.3265
ENSG0000(	HOPX	4:5664798	128.613	20.2431	119.666	191.76	91.8843	118.288	371.143	160.829	232.62	40.1073	0.0745	0.690046
ENSG0000(	C6orf3	6:1112990	0.813059	1.69552	0.867425	0.414343	0.45319	1.39526	0.74408	2.01148	0	1.29385	0.8409	0.323263
ENSG0000(	TAF5L	1:2295931	1.95677	0.840491	0.342802	0.811876	0.558585	1.69825	1.20153	0.371072	0.264551	1.55969	0.93185	-0.11504
ENSG0000(	ARHGAP1	10:318054	4.94387	8.95081	1.97179	3.44011	3.2665	3.23497	3.80559	2.09529	6.55748	4.8796	0.9804	-0.01437
ENSG0000(	LEPROTL1	8:3009539	105.063	149.502	143.67	111.381	81.9075	114.178	183.546	156.702	160.207	61.0127	0.8776	0.09322
ENSG0000(	CTD-2561	17:810179	0.535331	1.82697	0.506222	0.466738	0.52549	0.113471	0.487498	0.160237	0	0.207537	0.34015	-1.52859
ENSG0000(	METTL4	18:253752	14.1751	17.0326	6.87882	7.38848	5.51677	11.5312	12.0102	7.48493	8.14771	6.45082	0.5743	0.253952
ENSG0000(	PARG	10:498182	11.4755	17.2136	4.41366	6.42965	3.13756	8.2959	6.06815	7.81933	5.16959	6.70598	0.5641	-0.28111
ENSG0000(	S1PR2	19:101333	0.746084	0.556759	8.87828	3.79566	7.23094	2.08074	0.504431	10.0078	6.38451	14.8556	0.63335	0.584077
ENSG0000(	WDPCP	2:6312138	3.52037	3.63572	3.52576	7.21639	2.09885	5.06661	3.45407	0.398909	10.6299	2.36878	0.99965	-0.00026
ENSG0000(	SSNA1	9:1371886	98.2112	75.431	123.168	143.307	110.069	154.899	155.082	148.56	120.107	45.663	0.7559	0.101282
ENSG0000(	RP11-343	16:691055	9.3913	25.2406	20.5523	1.83428	24.0851	39.4246	33.292	18.5316	1.15391	19.2354	0.883	-0.26452
ENSG0000(	HM13	20:315144	109.997	87.7082	114.897	106.554	128.614	101.878	152.289	119.524	144.402	95.9463	0.64985	0.139773
ENSG0000(	UHRF1BP1	6:3479201	1.12867	0.709914	0.91298	0.432656	0.778716	0.230841	0.579881	0.419387	0.010063	1.49365	0.4984	-0.76066
ENSG0000(	RP11-347	12:121712	3.75981	4.12929	6.10427	1.87921	2.73043	1.39972	2.6186	4.51389	3.43698	4.7639	0.83145	-0.30996
ENSG0000(	ZNF136	19:121630	4.86343	6.82369	5.72309	6.67599	3.67688	3.2194	3.97572	3.81078	5.82055	5.35224	0.47065	-0.38961
ENSG0000(	RNF170	8:4284963	7.91785	7.63503	10.5424	10.4951	6.20151	8.15227	7.72135	8.72885	11.9914	12.143	0.9439	0.047916
ENSG0000(	RPL7P1	5:1500532	40.8915	26.6996	31.527	34.182	9.3484	33.7299	25.0737	11.7357	21.9876	1.15782	0.7595	-0.33652
ENSG0000(	ZSCAN16-	6:2812179	3.96906	3.2155	3.97441	3.78139	2.82504	3.67277	2.98907	2.49782	5.28496	2.50013	0.94455	-0.0787
ENSG0000(	HLA-K	6:2992598	0.464761	0.823184	0.206732	0.659958	0	0.038763	1.31792	0.039578	0	0	0.64075	-0.88607
ENSG0000(	CTD-2184	15:521151	1.70992	1.53692	2.6156	1.1433	0.545564	0.505272	3.65346	1.46909	0.195612	0.42702	0.981	0.037856
ENSG0000(	IGHV6-1	14:105939	0	0	1.18873	12.8374	0.182377	0	0	0	38.163	0	0.51315	1.61738
ENSG0000(	F2RL1	5:7681893	0.995078	0.625273	0.109565	1.18736	0.129962	0	0	0	0	0.536843	0.4395	-3.29953
ENSG0000(	SNRPEP2	9:6716494	0.293982	0.189994	0.646701	1.40407	0.238593	1.01473	0.450488	0	0	0	0.5195	-1.19655
ENSG0000(	RP11-217	15:893350	0.35039	2.34445	0	0.990105	0.668503	0	0.964568	0	0	1.2601	0.61175	-0.9733
ENSG0000(	RP11-421	17:511774	0.725253	0.462046	0.393158	0.465606	0.139449	0	2.83491	0	0.660564	0.61335	0.5785	1.0645
ENSG0000(	APEX1	14:204551	77.8438	83.3522	136.318	117.348	102.042	64.4888	82.4666	69.419	109.881	78.5317	0.3651	-0.35584
ENSG0000(	PIGB	15:552029	17.8625	13.7618	12.0624	16.6271	7.71569	16.3024	15.8912	10.8812	3.27913	14.7495	0.94105	0.064665

ENSG0000	MCUB	4:1095602	125.613	186.738	144.937	120.814	64.4066	81.8165	64.4898	85.9145	116.089	44.9786	0.07445	-0.67666
ENSG0000	NUDC	1:2689048	81.8542	86.2641	121.851	103.547	165.94	88.9032	90.0201	105.217	89.0079	94.4111	0.4858	-0.24501
ENSG0000	ZNF24	18:353322	16.4465	19.4446	29.0745	18.5174	28.7635	6.02641	15.6458	21.3848	16.3305	37.6326	0.2697	-0.59493
ENSG0000	FAM30A	14:105917	0	1.7056	1.57287	4.06635	1.24994	0	0	0	0	0.027247	0.23815	-9.28908
ENSG0000	DDX27	20:492192	44.7051	60.2187	43.5263	38.0671	41.5309	29.011	43.7109	34.4205	39.0398	40.8176	0.6082	-0.29107
ENSG0000	UBXN11	1:2627917	35.7581	52.5562	38.3431	35.3418	17.9968	88.2457	35.1577	40.6863	48.6967	20.2262	0.47065	0.449318
ENSG0000	IFT22	7:1013133	4.74537	2.14942	5.19012	6.55823	6.92948	6.44185	11.8538	8.88684	6.52237	1.84756	0.5986	0.420914
ENSG0000	ZNF2	2:9516543	3.15494	4.20393	5.30118	2.16176	2.84809	10.733	1.50624	3.44456	7.68363	3.88346	0.4589	0.496107
ENSG0000	ALDH6A1	14:738518	5.62235	4.66484	8.69742	3.21144	6.1836	7.74178	4.05746	6.56154	4.53759	8.58257	0.98865	-0.01015
ENSG0000	RP11-342	2:7564678	1.30082	0.886051	0.461179	0.628247	0.870404	0	0.091455	0.51911	0	1.32979	0.52365	-1.1606
ENSG0000	TRGV9	7:3831701	160.187	6.27466	60.4618	48.1206	27.7119	41.4499	22.0908	24.7245	12.658	10.7832	0.0013	-1.39311
ENSG0000	RN7SKP15	11:132766	0.20781	0	0	0	0	0	0	0.522297	0.850686	0.428128	0.2908	3.93617
ENSG0000	APOC1	19:449057	0	0.339194	0	0	0	455.369	5.76001	3.2591	32.6085	1.93408	0.0033	10.5025
ENSG0000	SLC2A4RG	20:636578	2.26295	2.52388	8.75139	5.25709	11.0607	1.31436	4.38923	15.9131	9.1752	12.3082	0.59015	0.558831
ENSG0000	RP11-473	16:315673	0.578189	0.375167	0.749447	0.082508	0.351446	0	0	1.72518	1.29711	0.968151	0.5594	1.06257
ENSG0000	ZNF182	X:4783690	7.5239	8.32062	1.81496	2.10944	1.98459	10.056	5.675	3.76302	1.10966	3.60754	0.79205	0.159
ENSG0000	SPATA1	1:8449832	0.528621	1.40121	0.587268	1.39746	1.69037	2.00674	5.77053	0	3.32995	1.39779	0.35335	1.06933
ENSG0000	HAMP	19:352807	0	0	0	0	0	1.65704	0	3.52291	0	0.107746	0.0087	inf
ENSG0000	PPP1R37	19:450714	1.26195	1.92618	0.672349	0.89352	1.86403	1.27778	0.631547	1.09155	0	1.18432	0.367	-0.88486
ENSG0000	RAC2	22:372252	672.398	626.631	1063.33	856.99	2164.87	552.138	745.963	1093	683.405	1141.96	0.43825	-0.39086
ENSG0000	AC005253	19:185577	0.927007	0.309087	0.809064	0.812398	0.404586	0	0.399114	0.888199	1.01429	0.349732	0.73315	-0.56813
ENSG0000	RPL7AP6	14:698541	1.57332	1.79343	1.37772	1.84729	0.662258	2.21789	1.3389	1.14598	0.847079	0.082275	0.8382	-0.45474
ENSG0000	DNAJA4	15:782505	10.6876	14.2537	4.78013	3.15457	3.88036	17.7151	8.74303	5.61583	5.78436	14.0117	0.53415	0.4711
ENSG0000	CATSPERE	14:915806	1.91805	0	0.433202	0.60629	0.342079	0	0.100049	0.392975	5.02919	0.442678	0.29025	1.41166
ENSG0000	EPHX2	8:2749077	5.47733	73.5492	4.55068	1.2638	5.02713	6.95242	33.6381	1.55258	0	3.91131	0.1551	-1.07881
ENSG0000	IRAK1BP1	6:7886747	0.88277	1.17624	0.288599	0.210755	0.379615	1.33097	0.562479	0.221725	3.17287	0.341212	0.51725	0.77018
ENSG0000	PINLYP	19:435430	2.42142	1.16031	3.29976	2.29843	0.907507	2.12748	8.47204	7.78428	0	0.809798	0.51395	0.848513
ENSG0000	MYBBP1A	17:449739	20.023	22.3146	7.36066	7.18004	7.86327	4.37	11.2467	6.01515	2.05502	9.88365	0.1242	-0.81861
ENSG0000	PDXDC1	16:149745	27.5998	15.137	7.45878	7.65176	5.6539	29.9869	9.02343	7.7718	8.05741	13.5205	0.94875	-0.04229
ENSG0000	ZNF263	16:326374	28.5909	28.4768	29.4584	27.3927	20.1864	18.9103	14.5747	21.9324	29.6913	22.3958	0.4519	-0.3335
ENSG0000	LPXN	11:585268	170.167	117.095	279.154	232.098	289.927	143.926	141.384	223.41	249.961	273.383	0.7502	-0.10528
ENSG0000	NUDT7	16:777224	15.7206	11.8087	11.9627	14.577	10.8617	17.7604	13.0504	15.6637	22.3776	9.58561	0.65925	0.293107
ENSG0000	PLEKHA4	19:488370	1.03201	0	0.92711	0	0.673128	0.036532	0.608986	1.69515	0	1.87368	0.4575	0.773725
ENSG0000	RFC1	4:3928745	18.1183	21.2026	9.54844	6.02431	21.0732	19.5504	26.8744	10.2404	7.7098	27.8639	0.89325	-0.08038
ENSG0000	KLC2	11:662572	6.14998	4.21376	6.23996	4.13827	8.88335	4.42314	11.3113	8.63635	7.79553	14.9636	0.26115	0.570702
ENSG0000	AMDHD1	12:959432	0.688892	0.16438	0.096529	0.473272	0.856836	0.583517	1.45604	0.641414	0	0.780587	0.7556	0.332475
ENSG0000	BST1	4:1560453	2.37162	2.78159	0.382038	1.70264	0.128043	14.1278	0.724202	0	0	0.402803	0.5473	0.991972
ENSG0000	DHX8	17:434838	13.8041	14.7464	9.75548	10.8959	8.12152	13.584	7.00018	12.8613	3.25304	13.3714	0.63475	-0.20025
ENSG0000	FAM20C	7:182934-	0	0.431643	0	0	0	3.25795	0	0.118466	0.624344	0.136761	0.2886	3.44943
ENSG0000	C19orf38	19:108365	1.90003	5.39909	0.600953	0.097845	0.612263	0	0.139411	0.241077	0	0.63893	0.1195	-2.92058
ENSG0000	RP11-530	12:795749	0.58061	0.626044	0.050942	0.862215	0.056659	0.130846	1.27109	0.118011	2.05909	0.062098	0.62215	0.95728
ENSG0000	DNAJC11	1:6624865	14.2686	16.6221	11.2489	8.95006	7.77183	14.9611	10.9345	6.35366	6.35048	11.2075	0.3277	-0.46122
ENSG0000	FCGR1A	1:1497826	0.924365	2.63928	0.249292	0	0.492773	70.7073	1.70496	2.49555	10.1936	0.906613	0.0027	4.37626
ENSG0000	PYCARD-/	16:312014	2.7653	0.978773	1.30875	3.68012	1.4164	0.786359	1.64958	1.51863	2.17603	0.479903	0.66705	-0.53982
ENSG0000	NR3C1	5:1432779	22.7259	31.669	18.0099	16.5209	19.8311	21.528	35.2075	38.0204	27.7001	50.5115	0.117	0.581683
ENSG0000	PHIP	6:7886747	12.8661	16.9887	5.3283	5.41467	7.57505	11.2682	11.8243	6.43683	6.66851	15.9817	0.85535	0.061347
ENSG0000	PIGN	18:620435	12.2681	6.97884	2.66246	5.21128	1.89288	4.85273	3.66875	4.87098	9.77698	2.91611	0.7673	-0.12649
ENSG0000	DPCD	10:101570	2.47429	1.68284	1.88517	2.49925	1.68093	10.9237	0.146414	1.35535	0	1.04007	0.85635	0.266263
ENSG0000	TSEN34	19:541899	19.8619	32.9304	42.5921	33.8677	39.2717	37.8804	40.3074	32.5752	20.7833	16.0942	0.5619	-0.23062
ENSG0000	CD48	1:1606701	145.337	75.6946	212.395	208.263	154.791	220.51	240.561	236.969	136.99	70.1475	0.6708	0.154789
ENSG0000	PNPLA7	9:1374599	0.674484	2.16802	0.493531	0.143379	0.22759	0.011267	1.47991	0.805633	0	0.474756	0.6481	-0.45541
ENSG0000	FAAH	1:4639426	0.878715	4.21415	0.81798	0.443908	1.22565	3.37782	1.78697	1.43802	0	0.639862	0.89285	-0.12755
ENSG0000	RIMS3	1:4062067	0.746096	0.063241	0.598771	2.23023	2.11206	4.32752	0.6212	2.59794	0.393978	7.42373	0.42555	1.07574
ENSG0000	SNF8	17:489293	98.0063	88.3979	112.104	107.63	96.722	136.545	123.294	111.444	61.3499	59.1226	0.6203	-0.19486
ENSG0000	SPAG1	8:1001579	6.4933	2.57307	1.55341	0.793607	1.07379	6.25937	4.2319	2.1651	1.37484	2.53809	0.5891	0.630385
ENSG0000	TC2N	14:915806	221.882	84.2883	173.913	195.398	102.88	83.2014	70.6146	84.9376	157.002	113.564	0.256	-0.61891
ENSG0000	ERLIN2	8:3773476	3.4097	3.00971	2.73067	5.40185	1.32795	2.34089	3.06072	2.10264	5.12322	1.50826	0.76655	-0.20931
ENSG0000	FGFR1OP	6:1669991	18.1222	11.6715	10.4408	7.12382	10.9329	24.9679	25.4902	21.4217	16.8872	11.2493	0.56195	0.734857
ENSG0000	TMEM175	4:932386-	48.1887	70.0958	30.3624	35.7982	31.013	44.4815	61.8545	18.5328	17.5902	47.8092	0.39275	-0.32019
ENSG0000	ZNF473	19:499764	2.89806	3.51461	2.31349	1.1948	1.68129	3.29241	2.49925	4.0972	1.78213	3.26231	0.77325	0.232817
ENSG0000	CTD-2006	19:119250	0.173035	0	0.195172	0.29045	0	0	0.010891	0	0	0.471042	0.296	1.60069
ENSG0000	ICE2	15:604196	24.5551	18.46	7.4019	11.3012	7.75197	13.8799	22.207	12.2808	10.2947	13.1352	0.8462	-0.09816
ENSG0000	PTGFRN	1:1169100	0.525211	1.31193	0	0.007982	0.166765	3.18535	0	0.219386	0	0.267048	0.64285	0.874045
ENSG0000	PARPBP	12:102120	4.54412	4.10806	1.53305	1.75861	0.322994	14.4145	3.07746	1.59238	0.111229	1.6665	0.3378	0.775943
ENSG0000	LRRC45	17:820187	12.9761	16.4324	5.1829	3.72469	6.61251	20.1282	19.2498	4.92923	2.8892	12.5701	0.7162	0.322999
ENSG0000	RP11-458	7:6690285	0.470605	1.16905	1.0987	0.63341	0.629576	0.124034	0.322919	1.26639	0.997441	2.82179	0.4808	0.487372
ENSG0000	KNL1	15:405940	1.66844	2.64914	0.067139	0.08668	0.30014	4.59212	2.77725	0.511875	0.386328	1.18994	0.2212	1.07862
ENSG0000	HMGB3	X:1509805	3.6097	8.70975	0.246818	2.17028	1.29623	9.03678	8.02246	4.85046	12.5274	1.3585	0.29165	1.19371
ENSG0000	SFXN3	10:101031	7.34264	3.68302	9.98417	14.3072	7.68418	6.97574	6.31468	16.197	11.985	15.4801	0.36755	0.380049
ENSG0000	PTDSS1	8:9626171	42.7878	45.3817	42.9148	43.9407	44.3109	43.321	57.5862	49.5168	36.4553	57.1643	0.9049	-0.0397
ENSG0000	TLR1	4:3879067	10.4877	8.64044	4.26909	5.12365	5.58373	12.2494	0.132448	1.69412	4.23761	5.87557	0.2002	-0.59658
ENSG0000	FAM133DI	2:1573797	0.293305	1.40004	0.503965	0	0.621952	0.121312	3.19934	0.367228	1.93537	0.232698	0.50645	1.10695
ENSG0000	ARHGEF1(	8:1755777	0.026928	25.8818	0	0	5.25806	0	4.55304	0.383495	0.156187	5.56289	0.3459	-1.42793
ENSG0000	FAM132A	1:1242445	0	0.286972	0	0	0	0	1.58738	1.16144	0	0	0.361	3.11108
ENSG0000	NUDT14	14:105140	7.39781	7.02153	9.79647	5.93508	2.95515	49.5841	35.2766	34.2156	17.1098	6.97333	0.0016	2.09378
ENSG0000	RP11-635	18:633277	4.27351	2.67034	4.71437	2.09957	4.30394	0	5.10554	3.70323	3.40714	0.743641	0.68735	-0.58836
ENSG0000	HEATR1	1:2365179	6.30308	5.71435	2.00368	2.81432	2.32163	3.36897	12.3962	6.45559	3.28295	5.51947	0.3842	0.697855
ENSG0000	SCAMP1	5:7836058	15.7301	12.7108	9.08965	15.8775	9.68398	6.99649	11.4047	8.98776	11.1531	11.1426	0.188	-0.7825
ENSG0000	CAPN10	2:2405867	38.8703	36.403	16.4311	23.0692	16.3715	26.9358	31.0071	21.6539	18.8997	28.6495	0.9259	0.040918
ENSG0000	C6orf47-A	6:3165829	1.83285	1.80863	0.404113	2.03649	0.684069	0.557904	0.474125	0.187371	0.700795	0.35215	0.7608	-0.43295
ENSG0000	DNASE2	19:128752	27.9997	18.9334	60.8989	67.0842	51.6068	81.5099	26.432	45.3636	53.4852	38.2654	0.98485	-0.00688
ENSG0000	KIF21B	1:2009693	4.11935	3.91536	3.35102	1.18966	5.53085	4.48309	6.36044	5.10957	1.76203	11.6848	0.1239	0.580188
ENSG0000	ZNRF1	16:748713	11.7475	15.6534	5.46675	3.99053	14.8472	85.0395	100.106	96.0649	35.512	115.173	0.002	2.85641
ENSG0000	TUBA4A	2:2192297	112.558	257.918	113.304	195.553	154.339	114.955	209.163	171.249	149.483	134.953	0.72165	-0.13746
ENSG0000	ZBTB37	1:1738632	0.743402	1.52035	0.276839	1.40297	0.853682	0.078402	2.89085	1.3844	0.16555	1.38024	0.98965	0.015244
ENSG0000	GPR18	13:992007	32.7358	36.9931	45.5624	45.0692	55.4971	56.6505	49.0574	54.4225	24.6055	66.3574	0.91905	0.136071
ENSG0000	POLR3G	5:9045820	1.59743	0.770508	1.21619	0.581665	0.444925	0	1.07841	0.209641	0	0.801308	0.2028	-1.47514
ENSG0000	RP11-108	10:101060	0.385239	0.379522	0.370401	0.379529	0.0429	0	2.86605	0.451481	0	0.294033	0.50015	1.16767
ENSG0000	AC091814	12:993657	4.43788	2.29837	0	0	4.02615	4.39593	1.17248	0	0	4.64253	0.93085	-0.08669
ENSG0000	DNAL1	14:736448	2.39123	0.768598	1.80713	0	1.00228	0.057659	1.86235	5.36808	0.021827	0.914429	0.63475	0.52259
ENSG0000	PSMB8-A	6:3284071	185.343	171.252	295.385	294.412	135.17	174.825	147.266	213.499	172.961	112.692	0.68155	-0.32304

ENSG000C	GLDC	9:6532463	0	1.05928	0	0.36572	0.009515	0	1.41043	1.44071	1.0103	0.460855	0.19825	2.03415
ENSG000C	UTP20	12:101280	0.865881	2.75552	0.807005	0.605759	1.16807	0.559414	1.14852	1.00696	0.387841	2.11137	0.31725	-0.57448
ENSG000C	C7orf43	7:1001489	4.94391	6.87403	6.83028	7.67129	7.5491	2.70329	6.63223	9.25532	4.76761	16.1726	0.72935	0.312712
ENSG000C	DHX36	3:1542725	18.2159	20.0084	8.73966	8.14672	13.2816	16.9217	26.6608	14.5109	13.0211	21.9799	0.3242	0.365616
ENSG000C	QRSL1	6:1066295	9.39327	11.7572	9.25073	9.89916	5.61203	7.95727	9.11848	8.15646	16.8331	9.4023	0.70175	0.15885
ENSG000C	UHMK1	1:1624972	10.3805	10.6425	8.00519	6.22227	9.69834	5.98705	7.44734	10.0293	7.97883	18.316	0.50775	-0.26512
ENSG000C	GTF2I	7:7465766	52.4584	58.0404	28.5094	36.7418	52.0837	32.272	35.7176	42.0674	23.8385	82.2102	0.46165	-0.42408
ENSG000C	ZACN	17:760387	8.48676	7.23035	0.093166	0	0.297734	3.49311	4.75727	0.130004	0	0.358263	0.32675	1.55658
ENSG000C	RP11-342	17:619426	0.390715	0.229936	0.132501	0.18091	0.171348	0	0.027067	0.019238	3.23147	0.375082	0.33695	1.90529
ENSG000C	RP11-750	11:473831	0.517853	1.58947	0	0.215756	0.677242	1.69472	0	1.05773	1.36665	0.378353	0.59605	0.831605
ENSG000C	PIK3CA	3:1791481	8.18682	10.3626	1.43975	0.669729	1.58479	1.10148	6.18254	1.33604	1.67681	3.26447	0.1625	-0.74336
ENSG000C	SAMD10	20:639741	14.9531	16.4144	5.28747	4.31334	9.54091	14.9408	23.3116	9.92777	10.1157	21.3544	0.2114	0.53827
ENSG000C	LHFPL2	5:7848521	0.209102	1.2116	0.063377	0.254532	0.042228	4.27633	0.633266	0.553315	0.038756	0.204953	0.08875	1.60152
ENSG000C	MORF4L1	15:787592	184.149	170.469	122.741	105.164	106.797	194.848	206.955	96.0381	93.4167	45.2763	0.6406	-0.14655
ENSG000C	RP11-174	12:147625	0.083503	1.03902	0.202781	0.596979	0.082753	0.700559	0.638309	0.208277	0.506839	0.584525	0.6028	0.381588
ENSG000C	DCTN2	12:575207	47.1757	48.2696	40.0844	31.2755	40.562	58.2112	62.4559	63.0073	33.3296	42.7182	0.44445	0.24609
ENSG000C	AC011933	17:753180	0.502595	1.76612	0.187203	0	0.19588	0	1.32265	0	0	0	0.6254	-1.07249
ENSG000C	PAN3-AS1	13:281368	1.0327	0.375708	1.37373	0.281503	0.431923	0	1.05513	0.540181	0.049167	0.086176	0.62165	-0.92361
ENSG000C	CARNS1	11:673178	0.475385	1.64745	1.15741	0.819757	1.29443	0.150603	0.239765	1.77558	0	0.637191	0.49655	-0.92127
ENSG000C	EIF2S2	20:339830	47.8177	39.9588	36.7331	49.3682	46.5201	73.2145	67.7515	49.2483	39.1296	21.366	0.80695	0.164684
ENSG000C	RP11-158	3:1715716	2.24413	3.42888	0.238044	0.247319	0.271302	0	0.837666	0	3.00365	3.35449	0.88405	0.237479
ENSG000C	C14orf2	14:103912	180.188	124.986	245.829	202.062	121.409	256.803	199.109	180.53	161.676	44.7371	0.76605	-0.10509
ENSG000C	CMPK2	2:6840569	8.41199	10.7332	3.33582	11.798	4.17394	6.6459	76.9036	19.663	3.52423	6.37646	0.09275	1.50221
ENSG000C	ABHD8	19:172921	7.60405	4.09806	8.19862	6.05957	8.54438	6.83158	4.16645	6.5533	6.40352	6.75434	0.9125	-0.19796
ENSG000C	C12orf76	12:109999	16.625	11.669	18.5549	12.2309	10.3825	9.3225	14.6915	20.6436	17.1224	10.1746	0.91685	0.075318
ENSG000C	MSC	8:7182816	8.92188	3.71564	37.5475	11.6017	18.7609	14.4157	47.0765	122.312	58.8583	42.3349	0.00025	1.79957
ENSG000C	PPP2R5A	1:2122855	48.3782	28.3283	26.8461	24.8913	39.8399	18.9314	27.751	30.3714	18.1803	42.6586	0.46585	-0.25035
ENSG000C	MALSU1	7:2329873	17.9913	20.0052	32.5615	11.5517	17.5239	46.0271	37.0826	30.7401	14.2588	6.05127	0.88735	0.101176
ENSG000C	JAML	11:118193	245.381	159.687	374.413	389.603	241.997	240.665	341.606	408.583	594.695	359.787	0.32515	0.407258
ENSG000C	RAC1P2	4:4672382	4.4136	4.97268	0.898714	0.796041	0.710375	5.90061	6.6284	1.34084	0.672629	0.515232	0.83825	0.404517
ENSG000C	EXOSC7	3:4491525	31.53	27.1318	71.2485	53.1104	59.1926	33.2321	36.0709	43.1721	37.3724	25.1687	0.36805	-0.52859
ENSG000C	IL9RP3	16:14380-	0.243333	0.368318	0.026133	0.062002	0.026698	0.854632	0.692747	0.953391	0.278593	0.131263	0.4285	1.77811
ENSG000C	RP11-73M	14:103553	0.495799	0.742831	0	0	0.128363	0.432949	1.56118	0.132635	0	0.610081	0.6168	0.881536
ENSG000C	DZANK1	20:183833	0.811535	1.22253	1.54714	2.43395	0.241646	1.4037	0.095294	1.62051	0.039822	0.810331	0.7188	-0.39702
ENSG000C	SH3GLB2	9:1289208	26.0901	21.4233	5.57488	3.50555	8.45075	42.5089	24.04	7.06215	5.68762	16.1507	0.84905	0.148375
ENSG000C	RP11-496	11:537526	0.720009	0.345278	0.022206	0	0.077297	0.568787	1.81544	1.22225	0	0.439741	0.1381	2.20653
ENSG000C	CYP51A1	7:9211215	5.82683	6.33658	3.95874	3.89125	3.08694	8.56391	17.8576	15.1886	17.3985	6.3415	0.001	1.51392
ENSG000C	CASS4	20:564121	1.5381	5.56569	1.76141	1.72013	0.81641	6.64133	4.80174	7.045	3.1204	4.24187	0.08305	1.21168
ENSG000C	GALC	14:877104	43.4659	6.15433	12.3772	24.8303	12.6942	16.6789	3.33474	7.78493	7.36515	9.86204	0.0017	-1.11389
ENSG000C	RNASE4	14:206840	0.339719	1.27589	0.860396	0.425003	0.223158	1.52203	0.791921	0.241169	7.72572	0.651544	0.1368	1.72607
ENSG000C	RP1-101K	6:1529705	3.16975	4.47509	3.62649	10.4296	2.25631	3.77001	0.77934	5.58635	4.25736	6.46387	0.8711	-0.29984
ENSG000C	GPR15	3:9849760	2.31073	2.25593	1.42999	0	0.092293	5.81631	0	0	0	1.1373	0.9335	0.147478
ENSG000C	RP11-423	5:1778094	0.076418	1.04659	0.153793	0.2708	0.514972	0	0	0	0	0.580393	0.214	-1.56638
ENSG000C	UBALD1	16:460888	5.83807	4.87296	3.66245	1.67119	5.25563	3.08274	6.80366	3.29511	2.23854	3.62122	0.8387	-0.14078
ENSG000C	NIPSNAP1	22:295058	32.1598	24.5692	30.7372	26.5883	25.5765	15.1621	18.1899	14.5444	26.6564	18.1077	0.19215	-0.58576
ENSG000C	PCYT2	17:818878	33.9883	25.8383	32.6313	24.509	27.9989	32.8688	35.134	37.4406	12.9159	27.2784	0.71185	-0.21475
ENSG000C	PRSS36	16:311389	2.26702	1.80192	0.359354	0.772494	0.337065	1.79815	1.18652	0.725404	3.53791	0.520021	0.71605	0.358227
ENSG000C	TBC1D17	19:498182	8.6631	11.3183	7.70344	26.444	22.1564	5.25794	6.32113	7.70596	16.7515	23.3599	0.70455	-0.31899
ENSG000C	SLC16A8	22:380781	0.182175	0	0	0	0.160159	0	1.78157	1.54283	0	0.336482	0.23505	4.35331
ENSG000C	TRGV3	7:3834157	39.2692	29.4047	39.5706	63.9415	90.4501	41.6395	46.5344	15.7961	50.1306	60.9505	0.76995	-0.33284
ENSG000C	CCDC174	3:1465174	17.1608	26.6322	36.8497	15.3384	65.8273	45.6422	39.6577	33.1491	23.9681	60.979	0.885	-0.05817
ENSG000C	C2orf15	2:9899726	1.79802	1.77527	1.7858	1.48315	2.25472	0	0	0	0	0.394971	0.19325	-4.74515
ENSG000C	USP3	15:635045	21.6007	44.6062	14.7566	17.0707	18.1653	24.383	24.9063	21.9259	26.4623	32.0848	0.7262	0.128258
ENSG000C	ING5	2:2416870	12.2723	17.3697	11.1115	20.657	14.4315	25.3883	19.9983	17.2783	18.8342	14.7444	0.64145	0.234817
ENSG000C	PHOSPHC	17:492233	2.08057	0.273518	0.423658	0.389902	0.401904	0	0.699223	0.109871	0	1.07988	0.30055	-1.11641
ENSG000C	TOMM7	7:2281262	98.0771	67.058	148.115	128.288	83.7402	97.0995	103.07	108.609	130.836	13.3517	0.50575	-0.2389
ENSG000C	HMG20A	15:774204	21.3976	16.3153	28.7101	24.6811	12.9987	13.0739	14.5322	10.5407	0.083678	21.3737	0.06745	-0.97348
ENSG000C	DHRS4	14:239387	18.6956	22.0615	39.609	31.8638	46.9828	20.3889	19.147	21.9787	14.9254	15.4797	0.21315	-0.86123
ENSG000C	GUSBP1	6:5791978	1.57159	2.48745	1.18337	2.64199	1.06499	0.452451	3.21025	1.98812	0	0.778326	0.67565	-0.61064
ENSG000C	FAM199X	X:1041666	5.55494	5.67059	2.42226	1.93002	2.36601	1.75105	5.25773	2.71075	1.11254	4.17886	0.4352	-0.44634
ENSG000C	RUVBL2	19:489934	49.0135	38.641	62.6567	52.5707	78.7585	61.8389	55.9281	45.4509	43.5277	43.7086	0.39635	-0.27611
ENSG000C	PRPF39	14:450840	22.3717	31.2173	6.18135	8.11571	5.25023	19.4938	23.2873	7.91644	9.32845	7.99647	0.9452	0.04221
ENSG000C	COL6A3	2:2373240	0.246967	0.628918	0	0	0.058113	2.03989	0.384326	0.490861	0	1.64929	0.074	2.36462
ENSG000C	CTD-2331	19:528618	0.24663	0	0.641592	0	0	0	0	0.37208	0	0	0.64895	-0.93019
ENSG000C	PIGK	1:7708898	20.1344	18.9968	11.4692	7.719	11.0392	13.218	21.5709	11.1861	14.3868	10.1352	0.9783	0.014236
ENSG000C	RP11-968	12:542309	1.12965	1.80795	0.229412	0.224899	0.52447	1.00963	3.39774	1.62716	0.882224	0.744664	0.39605	1.10376
ENSG000C	ZNF501	3:4472959	1.56198	0	1.48709	0.689564	1.28693	2.33137	0.098316	3.68728	1.90485	2.1403	0.36125	1.1501
ENSG000C	RPS26P39	10:123171	0.217452	1.83378	0	0.215222	0	1.92188	0.850813	0.188414	0	0	0.7931	0.364739
ENSG000C	FAM63B	15:587711	11.8463	12.1409	24.5281	13.1853	18.896	14.4992	12.9099	17.2266	20.6927	31.1965	0.93215	0.087315
ENSG000C	PCF11	11:831569	14.9388	18.4848	14.2744	35.2215	20.1398	10.3748	14.4972	20.2843	47.8567	49.6749	0.81615	0.175297
ENSG000C	MSN	X:6558837	330.77	267.876	73.1515	34.2677	83.7807	182.986	152.132	98.573	64.0279	134.55	0.60445	-0.21333
ENSG000C	AMFR	16:563614	17.2791	20.4318	14.0573	17.6444	14.1993	10.4569	20.59	15.0127	13.6241	25.1184	0.8081	-0.08897
ENSG000C	HCG4P11	6:2972277	5.34887	6.50864	1.19809	4.62843	0.74192	4.04943	3.88357	2.88498	8.25399	3.08589	0.5448	0.781888
ENSG000C	CDKN2AIF	4:1834445	37.549	28.6017	35.7767	34.205	19.9787	17.0276	27.6416	25.1284	31.8656	20.9875	0.36835	-0.31483
ENSG000C	RMRP	9:3565775	0.262665	0.661475	0	0	0	4.0789	1.87795	0	3.5385	0	0.25855	4.17534
ENSG000C	COX7A2L	2:4233354	142.783	86.6788	198.49	164.571	104.773	156.025	145.035	140.842	206.649	39.3664	0.92565	-0.03273
ENSG000C	BFSP2	3:1333999	2.77177	0.928441	0.658986	0.150703	0.235011	3.28023	7.48848	3.87893	0	0.826495	0.22265	1.81299
ENSG000C	RGS6	14:719324	0.262456	0.240047	0	0.363489	0.158223	3.97643	0.233352	1.07684	0	0.395512	0.0411	2.35142
ENSG000C	SLC2A14	12:781251	0.389661	0.276419	1.65692	0.332122	0.858024	0.4319	0.502323	2.79829	0.524679	2.96155	0.3174	0.970062
ENSG000C	TIMM22	17:997116	16.0588	16.2512	26.5476	28.1009	21.6546	10.9776	16.4192	16.3132	18.2805	12.3468	0.2272	-0.60492
ENSG000C	SMG1P3	16:214022	21.8056	27.0031	4.65513	8.29502	1.52127	21.8859	23.4676	9.46734	21.2205	4.67782	0.5299	0.600967
ENSG000C	CXorf23	X:1991285	5.33037	4.17933	0.780244	0.659531	1.47591	2.3921	3.19428	2.04333	0.301348	5.60091	0.9922	-0.00565
ENSG000C	COPZ2	17:480261	1.85703	1.9464	2.22246	0.591699	4.95211	2.05832	5.59737	1.22681	0	1.53511	0.7235	-0.35137
ENSG000C	SERF2	15:437269	1445.95	940.313	1937.87	1945.84	736.843	2384.25	1778.68	1438.33	1648.93	300.849	0.9942	0.006132
ENSG000C	VWA7	6:3176558	0.893709	0.03603	0	0.59068	0.638105	0.277213	1.44386	0	2.58766	0.776879	0.6049	0.754248
ENSG000C	C16orf46	16:810534	0.487371	0.218387	1.12186	0.756319	0.223623	0	0.54945	0.833335	0	1.35169	0.89385	-0.15538
ENSG000C	AC002467	7:1077428	9.39816	5.92792	5.63918	6.74308	4.67589	6.08201	4.68557	4.26193	6.188	1.08474	0.59075	-0.53425
ENSG000C	MT-CYB	MT:14746	47.3263	46.3531	1585	1478.39	4150.45	54.2621	45.1401	2279.13	2473.13	3489.75	0.93785	0.070923
ENSG000C	TYW5	2:1999112	4.49476	2.22135	3.15941	3.28336	2.26191	3.4665	4.4182	7.85113	4.23637	4.52697	0.4631	0.660036
ENSG000C	RNF14	5:1419435	19.1292	20.2478	19.8721	18.1799	11.9637	21.06	16.8141	27.3961	20.0063	13.4187	0.57785	-0.21389
ENSG000C	FAM35A	10:870504	14.352	12.7233	5.46145	8.69061	4.24231	18.0753	13.1159	11.6414	7.67312	8.79925	0.6312	0.433901

ENSG0000(	SLC25A42	19:190639	17.332	19.5497	8.00162	5.34897	8.20303	11.8841	10.9543	6.06223	2.8188	16.7973	0.6087	-0.24859
ENSG0000(	EIF5A2	3:1708884	2.51792	2.47495	2.77585	0.777896	1.7824	3.20852	6.28967	3.55257	1.61133	2.02778	0.6507	0.293577
ENSG0000(	FANCC	9:9509905	1.38483	1.25019	0.642774	1.22557	0.669463	1.73286	0.977311	0.555531	2.13677	1.6865	0.5898	0.334814
ENSG0000(	FUT11	10:737443	13.5103	6.30701	32.7321	12.2226	30.6787	5.81889	8.24778	8.02437	4.94142	19.0496	0.35165	-1.13918
ENSG0000(	HDC	15:502419	0	2.47895	0	0	0.206493	0	1.95326	0	0	0.020406	0.78195	-0.47776
ENSG0000(	AAR2	20:362364	19.5925	25.082	30.9509	26.0789	28.5964	12.1104	22.3445	16.9549	27.545	21.6141	0.2845	-0.38387
ENSG0000(	NIPAL2	8:9818983	1.44415	2.64992	1.18126	0	0.036539	3.52921	0.264457	0.036946	0.689025	0.058289	0.96035	-0.06027
ENSG0000(	SDS	12:113392	0	0.115516	1.14107	0	0	21.5565	0	1.20771	3.23084	0	0.0795	3.73679
ENSG0000(	PNISR	6:9939805	125.089	193.497	124.82	111.155	115.386	162.956	141.169	109.535	118.371	209.677	0.9558	0.021624
ENSG0000(	NR4A3	9:9982185	0.607611	0.915727	0.169861	1.68074	0.057335	0	2.24872	5.51194	3.3124	1.25072	0.2632	1.88816
ENSG0000(	DNAJB9	7:1084704	35.0402	18.4782	41.0417	71.4171	33.0983	26.5365	34.9029	24.2892	47.0641	28.1245	0.52955	-0.32649
ENSG0000(	MRPL28	16:367383	70.1966	69.8633	84.928	95.0155	72.5836	86.2198	84.0719	80.2325	54.3304	33.032	0.39815	-0.29365
ENSG0000(	ARRDC2	19:180011	42.2469	63.1856	31.8859	52.9596	22.883	33.8634	29.5187	35.5224	34.4912	31.7375	0.42345	-0.26295
ENSG0000(	ZBTB2	6:1513641	18.085	16.0653	12.4178	12.7627	13.9408	11.2132	9.2809	13.6738	8.76611	21.5786	0.3924	-0.31657
ENSG0000(	PPTC7	12:110533	7.38326	7.93639	4.15815	5.68993	6.02877	2.10708	3.73995	6.07467	11.0045	10.3642	0.9069	0.042796
ENSG0000(	TLE6	19:297744	0	0	0	0	0.031226	2.09038	0	0	5.8945	0	0.30175	8.11152
ENSG0000(	TBCK	4:1060415	17.1559	18.8868	7.93737	5.33998	4.91944	10.2829	13.76	5.29193	1.99822	12.7887	0.77425	-0.19622
ENSG0000(	NAGS	17:440045	0.521255	0.17122	0.896903	0.309877	1.84603	2.28631	0.564246	2.59117	4.16172	0.601046	0.30445	1.53087
ENSG0000(	DPP3	11:664350	29.24	21.9305	21.3632	34.7683	18.054	19.2403	27.1011	30.6254	26.8441	30.9748	0.815	0.121017
ENSG0000(	IGHV3-72	14:106790	0	0.593436	0	0	0	0	0	2.21993	0	0	0.3861	2.47924
ENSG0000(	NF2	22:296035	6.63101	11.1298	7.20989	3.81694	9.24969	6.91529	7.44144	8.77396	4.36509	15.2523	0.79695	0.10545
ENSG0000(	RP5-943J3	1:8952483	2.96289	7.62556	3.02902	3.58506	2.62315	4.74446	2.74136	1.19189	0	1.15932	0.47805	-1.05998
ENSG0000(	RP5-1157	3:5222107	1.15695	3.64257	2.36631	0.573398	0.989838	0.039814	3.94189	0.616414	0.145985	0.572079	0.5996	-0.76219
ENSG0000(	RP11-415	3:1626515	1.81138	2.75579	1.92208	1.37348	1.93805	2.37816	3.55145	2.04586	0	5.78334	0.6379	0.589779
ENSG0000(	C14orf169	14:734784	22.3178	24.1479	19.0011	16.0919	16.6303	21.5953	17.4037	13.9102	4.76617	18.2343	0.32525	-0.41421
ENSG0000(	RP11-764	17:443952	0.807322	1.04491	0	0	0	0	1.05622	1.27688	0	1.26585	0.50295	1.4021
ENSG0000(	ASPHD2	22:264292	5.75608	6.82397	4.0936	2.86112	4.71925	2.46447	8.1379	3.48411	1.61836	6.36718	0.8391	-0.15913
ENSG0000(	CD244	1:1608301	32.1746	9.59897	30.3335	31.5052	55.8085	16.9337	33.3366	21.6052	5.98665	39.3095	0.08205	-0.6039
ENSG0000(	PROCA1	17:287031	1.77342	1.46244	2.71166	0.299456	2.76096	0.174462	2.34397	2.82522	0	3.62589	0.9287	-0.10086
ENSG0000(	MED16	19:867629	54.1278	64.6174	31.892	50.9747	37.1155	39.46	34.6085	29.9432	37.3481	38.8064	0.32665	-0.31753
ENSG0000(	UBN2	7:1392303	0.982412	0.953656	1.0243	0.151742	2.25531	0.781046	0.69531	2.15126	0.801808	3.98527	0.72105	0.265703
ENSG0000(	RBM12B	8:9321330	4.17737	3.93441	2.31649	2.28404	4.60391	0.524192	4.32655	5.454	5.96693	9.06878	0.8136	0.152414
ENSG0000(	BAG2	6:5708684	2.36033	1.48395	1.37995	1.93353	1.53145	1.11375	2.99091	1.16041	0.820903	1.17041	0.83445	-0.22647
ENSG0000(	SRP54	14:349208	49.03	68.2529	51.3598	37.4021	37.6957	62.7776	51.6158	42.9145	46.887	42.5168	0.92335	0.03115
ENSG0000(	RP11-126	1:1513406	2.71578	1.61015	1.75765	1.76168	0.749372	2.24504	1.08757	0	2.26336	0	0.6578	-0.61273
ENSG0000(	RUBCNL	13:463419	0.078342	2.85387	0.599751	0.733475	0.928176	1.77682	0	1.95351	0	0.540934	0.72815	-0.36242
ENSG0000(	AC090587	11:385431	0.279725	0.640566	0.870462	0	0.109571	1.74928	1.35743	0	0.905923	0.458725	0.5422	1.17705
ENSG0000(	COG6	13:396556	15.7877	8.3453	3.77469	4.74419	4.31477	5.68395	8.62584	4.67025	1.65143	6.16114	0.16705	-0.55978
ENSG0000(	RP11-337	1:1471727	0.4481	0	1.13476	1.97546	1.29198	2.00825	0	0	0	2.18715	0.7708	-0.54043
ENSG0000(	C7orf25	7:4290872	3.09557	1.11269	2.60427	0.206072	1.47549	4.21782	2.64804	3.91458	0	3.03956	0.5983	0.542052
ENSG0000(	CTA-292E	22:288006	0.415857	0.572916	0.678578	1.30302	0.838728	0	0.483763	0.760366	0	0	0.38405	-1.67086
ENSG0000(	HELB	12:663025	10.392	15.0236	3.50144	3.8125	3.36369	10.5958	8.52096	7.73217	4.19396	11.8575	0.7388	0.164434
ENSG0000(	ILVBL	19:151074	15.1968	21.2565	17.7463	32.72	19.5488	8.92954	25.2447	26.0366	22.9787	19.8282	0.67235	-0.15749
ENSG0000(	RP11-4B1	18:769746	0.430772	1.59645	0.024171	0.056487	0.012453	0.109413	0.395163	0.122983	2.13608	0.885701	0.6457	0.907735
ENSG0000(	HMCES	3:1292788	46.0141	55.8272	51.9947	35.8268	52.616	44.5574	51.6038	47.2162	36.4357	58.2948	0.77475	-0.09663
ENSG0000(	DDX59	1:2006238	28.5733	18.3297	16.721	15.5535	14.2442	29.6033	21.4327	23.003	17.3046	18.2803	0.5096	0.226279
ENSG0000(	CD247	1:1674306	227.405	191.425	291.591	289.367	259.626	222.274	182.218	232.567	197.822	174.757	0.2905	-0.34929
ENSG0000(	TICAM1	19:481593	7.14812	6.04363	0.89075	3.75622	3.52021	3.14045	4.84125	2.19492	1.01395	4.49152	0.4009	-0.48964
ENSG0000(	EPG5	18:456476	4.26397	3.80568	2.533	3.24151	6.12764	3.17896	2.77437	5.46644	4.6943	13.4668	0.3674	0.409235
ENSG0000(	PPP2CB	8:3077445	5.37504	7.48662	9.33455	3.3312	10.4035	6.93335	4.55076	7.23766	5.21801	9.69278	0.92485	-0.05329
ENSG0000(	CTD-3001	19:476075	2.64572	6.12282	2.53423	1.41669	2.21729	5.60344	12.8617	3.38679	11.65	1.65784	0.46845	1.22847
ENSG0000(	RP11-796	12:919849	12.9256	19.3337	28.4016	19.8915	14.274	4.30561	6.37496	7.22322	18.0423	5.36899	0.1866	-1.1197
ENSG0000(	RP1-292L	14:709066	0.777999	0.41197	0.800926	0.361586	0	0.435838	1.50614	0.941829	0	0.585498	0.67455	0.719733
ENSG0000(	ZNF124	1:2471219	4.53759	4.27035	6.70291	6.68232	5.37308	7.90437	3.96943	8.07731	0	9.10917	0.93785	-0.05097
ENSG0000(	SVIL-AS1	10:294094	4.66394	1.51594	3.52382	10.6491	7.59244	9.93779	0.472187	5.26493	11.7553	5.17499	0.8006	0.184834
ENSG0000(	EFL1	15:821302	11.3544	13.6062	4.51818	6.34516	4.56593	6.56968	7.42675	4.60925	9.32545	5.44327	0.6945	-0.1626
ENSG0000(	ARAP1-AS	11:726850	8.08127	10.8696	2.25368	7.6356	4.72908	10.6709	3.1299	2.02736	5.11246	3.50577	0.90505	0.199823
ENSG0000(	ZNF542P	19:562196	3.59923	2.15219	1.98124	3.73693	3.08844	0.324633	0.780466	2.78419	5.04659	0.547493	0.54495	-0.56971
ENSG0000(	NLRP1	17:549942	71.8557	131.688	41.4581	36.134	50.2073	40.3621	31.1417	46.7449	33.8731	80.8287	0.4056	-0.47278
ENSG0000(	FCN1	9:1349058	50.1005	203.972	3.77838	6.18487	6.8695	93.5586	1.7091	3.09362	10.2815	2.78427	0.0408	-1.20283
ENSG0000(	TBL1XR1	3:1770193	12.4081	16.5066	7.49153	3.7042	6.36967	19.2723	15.7855	15.566	10.0792	30.8572	0.0035	0.924738
ENSG0000(	RP5-1068	20:187945	4.11239	6.88286	6.80565	5.73421	3.41454	3.37458	2.64499	2.94461	0.105931	2.78653	0.075	-1.23216
ENSG0000(	RP11-24J1	15:918536	0	0.523416	0	0	0	0	0.023887	0	0	0	0.36505	-2.05721
ENSG0000(	EXOSC5	19:412624	34.3045	20.4896	66.4607	60.9087	49.4009	21.1413	30.4748	32.9476	54.7465	13.7206	0.69175	-0.61029
ENSG0000(	NDUFA5	7:1235369	115.095	81.3771	107.206	95.9604	55.005	84.9925	89.0769	80.663	109.258	21.1118	0.58995	-0.17627
ENSG0000(	PPP1R13B	14:103733	3.30914	2.98035	2.00855	2.45672	4.066	2.48555	1.35	1.02868	6.56285	7.92766	0.95775	0.039377
ENSG0000(	CFDP1	16:752936	65.6124	64.5781	60.5857	35.5877	82.0998	101.629	62.2797	44.4464	58.5015	62.0533	0.86085	0.081949
ENSG0000(	AURKB	17:820473	8.09746	30.3319	0.033867	1.50285	1.14213	17.4591	30.8707	8.08946	5.83866	9.18532	0.28275	0.874703
ENSG0000(	CEP350	1:1799547	22.6217	25.9669	6.65883	5.1776	11.5386	15.1207	17.2081	8.57772	11.1602	17.9932	0.8266	-0.12535
ENSG0000(	ZCCHC10	5:1329969	36.9997	34.7171	34.7295	37.3234	25.9489	45.2462	44.9376	36.1689	42.66	10.1143	0.61215	0.222203
ENSG0000(	PMF1	1:1562129	43.9495	59.166	93.041	119.844	81.1035	91.953	119.154	104.599	105.016	39.4933	0.62645	0.176796
ENSG0000(	CYCS	7:2512009	59.4812	40.5774	106.518	111.271	53.0312	63.7564	102.884	95.8877	82.2887	33.2031	0.666	-0.26601
ENSG0000(	RP11-83A	4:1392661	4.76184	6.93095	3.19324	2.39316	3.96473	7.47362	4.38269	2.86149	2.27305	3.97778	0.8767	-0.1339
ENSG0000(	PDF	16:691055	19.5691	21.4876	21.807	18.2691	25.3213	22.1646	18.7997	14.9482	6.65668	13.1526	0.63065	-0.61779
ENSG0000(	OSBPL8	12:763517	54.3018	81.9532	28.9823	48.246	162.266	93.0961	83.9505	23.0849	51.3258	165.213	0.87615	0.117185
ENSG0000(	TMEM121	14:105526	1.90678	5.18938	8.76973	1.97153	2.24276	3.57084	10.6716	9.84983	2.24149	1.27106	0.72025	0.325
ENSG0000(	ANKFY1	17:414316	11.297	12.5205	3.88249	4.86069	3.98911	6.9853	9.44677	4.33631	6.8825	13.5104	0.9166	0.046314
ENSG0000(	ATP6V0B	1:4397448	136.548	111.623	171.437	214.251	131.201	231.558	157.285	169.244	233.617	67.8173	0.6826	0.147202
ENSG0000(	FBXO38	5:1483839	17.4907	11.4642	3.95023	7.32883	4.26795	9.54525	8.07037	9.99089	6.3154	11.6263	0.9405	0.02914
ENSG0000(	AC004893	7:9887793	1.2213	1.79957	1.22741	3.36904	1.77603	2.03964	2.81079	1.26247	0	2.94528	0.8779	-0.15903
ENSG0000(	VSTM1	19:540408	0	4.36362	0	0	0	0	0	0	0.210879	0	0.4791	-3.49691
ENSG0000(	IFT57	3:1081608	18.3483	10.4341	19.5509	17.156	13.7395	9.28353	9.94161	15.0135	13.3291	14.8579	0.18445	-0.58624
ENSG0000(	RP11-417	12:131894	0.943284	2.4336	0.71473	0	0.148392	1.21448	1.04409	0	0	0.119538	0.76415	-0.87964
ENSG0000(	TMEM256	17:738053	0.607519	0.57725	0.978361	0.363555	0.806322	0.214812	0.08273	0.533505	0.235066	3.39471	0.74495	-0.37453
ENSG0000(	ANLN	7:3632422	1.08925	4.85413	0	0	0	4.16442	2.69288	0.649541	0	0.413848	0.70935	0.49864
ENSG0000(	AKAP1	17:570850	10.4975	15.2274	12.4099	9.28586	8.66145	4.83967	13.7748	4.29758	2.94789	8.43798	0.09925	-0.65623
ENSG0000(	MAP3K11	11:655977	12.9913	11.8983	9.28763	8.67635	15.0871	14.7008	9.85338	12.2872	5.39861	21.7622	0.53335	0.296153
ENSG0000(	CTD-2021	2:1517889	11.2959	15.3491	41.9037	16.966	55.7382	21.2467	32.8006	24.6667	33.3301	49.5486	0.4291	-0.42677
ENSG0000(	LIPA	10:892135	34.2357	46.0463	27.3396	32.1525	22.507	72.5126	28.491	28.8932	30.3208	26.554	0.76675	0.175668
ENSG0000(	CCDC86	11:608359	16.2546	9.24978	18.0879	18.1022	19.3841	15.2156	5.94283	15.9072	7.62502	10.2186	0.2075	-0.58086
ENSG0000(	S100A9	1:1533578	302.773	276.643	41.2614	191.322	50.7261	432.431	12.3395	169.669	125.724	40.7788	0.79075	-0.10427

ENSG0000	LAP3	4:1757719	46.4411	49.0222	49.278	52.1363	45.1808	128.655	174.16	74.6073	60.0204	28.5232	0.0108	0.884658
ENSG0000	C2orf27A	2:1317223	1.09861	0.055526	0	1.58059	0.348783	1.28321	0.619906	0	0.37098	0.914444	0.90165	0.1574
ENSG0000	PEX26	22:180765	3.86792	8.50936	3.53242	5.31912	3.88653	3.14597	9.07055	5.03908	2.98846	4.89093	0.8721	-0.21096
ENSG0000	RP13-942	12:123289	1.34201	1.95586	1.95167	0.151988	0.44105	1.97922	1.65384	0.325051	0.47726	1.97823	0.9107	0.141291
ENSG0000	EEF1E1	6:7881516	48.8959	27.8972	55.2276	61.6592	19.9119	43.4599	48.8662	62.8315	61.842	14.4531	0.8379	0.161137
ENSG0000	CCL20	2:2278138	8.00311	2.16803	8.55844	14.9771	4.26768	124.023	201.652	188.864	59.0404	23.1797	0.00105	4.02182
ENSG0000	HELZ2	20:635580	4.22533	5.5814	0.570828	1.62293	0.465481	7.14414	41.2485	9.53353	6.67067	7.58522	0.02165	2.44323
ENSG0000	CSNK2B	6:3166122	102.117	113.643	143.296	147.771	148.772	123.847	162.887	159.034	163.224	80.5197	0.88805	0.051277
ENSG0000	ADCK1	14:778000	2.99406	7.04946	3.44148	3.26919	4.70608	1.60432	1.08094	3.28825	3.08351	3.76161	0.31	-0.69262
ENSG0000	WDR70	5:3737921	18.0712	17.161	19.7887	18.2205	11.5185	12.9572	18.599	12.4363	18.467	7.56139	0.54115	-0.27967
ENSG0000	DHX16	6:3064703	34.9584	47.4098	18.5036	21.6007	17.2136	31.3065	28.9783	22.7148	18.7733	27.0704	0.8262	-0.07879
ENSG0000	LAIR2	19:544978	77.8011	37.5167	1.60462	31.0281	10.0645	250.219	112.188	2.68386	0.207717	2.52686	0.1451	1.1761
ENSG0000	AC010226	5:1155786	0.283816	1.63186	0.054987	0.288081	0.375596	0.725927	2.44368	1.41101	4.73362	0.738967	0.11125	2.26984
ENSG0000	AP003068	11:651776	0.734228	0.359887	0.589544	0.732441	0.361577	0	0.147249	0.534359	0.974574	0.294314	0.7999	-0.35087
ENSG0000	ASB3	2:5353267	19.8981	16.0433	15.2967	13.1432	12.1744	5.08323	15.4499	14.285	8.07502	16.5098	0.6084	-0.38507
ENSG0000	MCAT	22:431322	7.46199	6.16519	8.73148	10.2517	9.62034	9.38519	7.27866	10.6384	7.88454	3.28154	0.8221	-0.15921
ENSG0000	HEATR3	16:500464	3.71676	3.92915	1.70944	2.57721	1.21387	1.86461	2.84509	0.769466	0.544644	4.57728	0.50815	-0.59815
ENSG0000	RPS3AP6	15:597683	6.78634	4.42851	5.93096	3.9014	1.27078	5.54503	3.88702	3.25658	1.73739	0.087256	0.8961	-0.25246
ENSG0000	ARSD	X:2903969	2.85983	2.18297	1.42181	0.523331	2.29811	4.27033	3.91494	2.22342	4.74475	2.58211	0.43755	1.02315
ENSG0000	ERAP1	5:9588509	35.8172	34.6662	21.5236	36.5772	51.9476	16.6806	18.9804	29.1202	31.1273	51.1279	0.4039	-0.57492
ENSG0000	NPTXR	22:388184	0.332805	0.150869	0.35486	0.180452	0.232951	0.307712	0.722981	0.772636	0.103296	0.679606	0.3878	0.928782
ENSG0000	MCM9	6:1188134	12.9175	11.6223	4.25359	6.54542	5.14141	4.45739	13.8317	5.57649	8.78741	9.46131	0.9563	0.038159
ENSG0000	RPL13	16:895606	1440.23	1303.91	3238.11	1887.39	2286.48	1086.4	1220.95	2189.26	1261.52	275.279	0.05875	-0.84696
ENSG0000	AC005037	2:2009089	0.557344	1.12799	0.370685	0.764324	0.256707	2.19788	1.05567	1.43912	2.65654	0	0.31395	1.32674
ENSG0000	KLHL25	15:857593	1.75142	1.34125	0.270017	1.70055	0.765846	1.23817	0.671874	2.11876	8.71602	2.07576	0.03035	1.5667
ENSG0000	RP11-452	1:2228150	12.411	7.52135	5.74035	10.5091	3.79799	13.054	14.9806	4.76837	11.8455	4.2724	0.6402	0.45387
ENSG0000	EBP	X:4852115	69.8135	43.9387	125.255	126.096	95.9345	61.2085	80.3346	117.296	150.305	40.9944	0.89445	-0.05043
ENSG0000	TMC8	17:779592	178.242	234.098	24.2019	27.6829	47.0295	118.269	97.0859	31.9047	15.0143	99.4711	0.3391	-0.61223
ENSG0000	TNFAIP3	6:1378236	119.082	69.5171	57.5174	299.536	27.1964	101.483	97.0431	125.31	259.856	136.834	0.70805	0.163043
ENSG0000	GID4	17:180392	3.02532	2.6677	1.19629	3.02249	1.63422	2.93858	1.38797	1.98051	1.80287	3.18687	0.8822	-0.07766
ENSG0000	SLC17A5	6:7359337	4.38755	1.94528	3.76444	3.11373	3.11503	3.52974	4.14488	4.67255	2.99006	4.54051	0.7283	0.186454
ENSG0000	TRAV8-1	14:217972	5.05009	15.8076	4.47302	8.52531	6.84784	4.51765	51.6238	5.29353	8.51981	14.6831	0.2377	1.04353
ENSG0000	RP11-43N	19:474842	1.21137	1.09648	0.452524	1.54292	0.430129	1.13839	2.76615	2.97543	0.048385	0.949773	0.6215	0.657669
ENSG0000	TXN2	22:364670	78.3671	55.1602	123.925	140.207	141.988	101.341	89.8518	83.4194	139.577	61.076	0.55775	-0.20558
ENSG0000	HSP90B1	12:103841	336.939	239.691	181.857	206.208	286.673	297.998	276.323	164.905	171.397	226.13	0.49915	-0.24904
ENSG0000	DCAKD	17:450233	6.17118	5.88243	8.11934	5.77288	8.78412	6.28529	9.10114	10.2626	4.68305	9.31954	0.9238	0.061998
ENSG0000	FAM117B	2:2026351	3.62119	10.9606	2.98078	3.2805	2.90624	1.18298	0.712674	0.865128	0.995789	1.42784	0.00105	-2.22773
ENSG0000	LGMN	14:927038	16.6648	17.1834	1.0879	5.39226	0.599245	51.0744	6.68416	15.2886	33.051	2.84512	0.00235	1.50229
ENSG0000	EME1	17:503732	2.85765	0	0	0	0.284068	0	0.374826	0	0	1.5656	0.68885	-0.60711
ENSG0000	IFI35	17:430067	69.0337	50.0664	108.877	114.463	111.415	128.058	295.969	174.571	79.0475	63.4937	0.09145	0.579702
ENSG0000	WRB	21:393802	18.7453	20.8882	16.8964	20.1157	11.6631	18.1305	21.8696	13.2788	22.3441	5.46275	0.8103	-0.09577
ENSG0000	SMG1P5	16:302675	19.4198	15.0702	12.7628	11.4539	5.59302	22.1322	17.7995	17.0831	19.2999	8.44795	0.51695	0.379837
ENSG0000	H3F3AP4	2:1745471	5.7074	6.90464	3.1139	3.40071	2.39182	5.78034	9.234	1.60252	3.0876	0.251256	0.898	0.198334
ENSG0000	OSGEP	14:204464	28.629	32.5143	42.5396	44.7005	28.0211	49.8251	41.3867	40.7269	35.0109	29.6137	0.7662	0.101239
ENSG0000	RP11-196	1:1498444	0.523846	0.62235	0.325697	0.277399	0.308638	0.048349	2.6024	1.47114	0.883273	1.92868	0.01195	1.75684
ENSG0000	ACOT8	20:458338	36.9052	27.8595	30.6073	33.4269	29.1134	42.4668	34.8638	48.454	29.3906	12.4791	0.9772	0.012267
ENSG0000	CXXC1	18:502823	87.2964	122.835	39.0766	42.4303	42.956	53.4686	101.019	48.2674	7.3676	44.5848	0.13235	-0.50176
ENSG0000	GLE1	9:1285046	24.1393	17.0692	12.0723	17.3139	9.60236	18.5359	17.8875	16.115	11.236	19.1566	0.91945	0.036067
ENSG0000	SIRPB1	20:151675	1.5877	5.85743	4.24345	0.248287	4.24476	13.2562	1.47926	1.70602	3.36028	5.28836	0.51135	0.529669
ENSG0000	RP11-430	1:2046030	0.449031	1.16765	0	0.764843	0	0	0.879043	0.254057	0	0.837716	0.95615	-0.09017
ENSG0000	TMED4	7:4457789	62.9478	38.3396	115.238	94.0534	108.434	63.2571	36.6484	80.9445	76.9462	91.1778	0.3688	-0.32181
ENSG0000	C1orf112	1:1696620	3.35184	4.91748	1.65851	1.05054	0.985818	3.11887	3.10301	0.633464	0	1.97393	0.46755	-0.5574
ENSG0000	GATAD1	7:9244745	7.03299	7.79854	7.09314	4.50999	5.61641	3.22361	6.20013	8.61605	1.73489	14.1693	0.999	-0.00122
ENSG0000	ITSN1	21:335039	0.183119	3.64028	0.220867	0	0.520806	0.125155	0.703915	0.00496	0	0.228334	0.1179	-2.63581
ENSG0000	SLC7A8	14:231252	0.138873	0.044303	0	0.339809	0.309983	0	0	0.727552	0.115864	7.44479	0.0594	3.00688
ENSG0000	NDUFA10	2:2398924	69.9945	58.6593	83.5937	82.2521	63.3644	56.7528	79.3978	54.2822	56.1202	35.237	0.2886	-0.41566
ENSG0000	IGLC3	22:229063	0	96.1764	7.35932	439.378	7.59131	0	0.701925	2.57847	2.15049	11.1076	0.06125	-4.91729
ENSG0000	AIMP1	4:1060415	34.0052	24.2313	42.2482	28.5398	39.8285	66.8863	57.0284	34.3377	32.2983	19.1733	0.9491	0.037683
ENSG0000	ARMC7	17:751099	3.63174	2.93635	17.8849	17.9304	20.2771	1.07025	3.18979	21.5759	20.3618	21.8618	0.8799	0.15239
ENSG0000	AC009473	7:7636517	1.62502	0.124013	0.428674	2.34572	0.330905	0	1.0065	3.61369	0	0.259741	0.8923	0.277498
ENSG0000	RP11-483	1:1081340	0.978473	2.03142	1.87814	0.750398	0.6433	2.08306	0	1.16095	0	0.142553	0.51955	-1.23653
ENSG0000	EXD3	9:1373068	7.9178	6.12167	5.14215	7.65169	5.45799	9.63593	9.85402	4.46293	9.25013	8.36654	0.798	0.215027
ENSG0000	LTBR	12:634684	0.683799	1.68214	0	0.952164	0.3818	4.87638	0	0	1.15408	0.427888	0.38455	0.904659
ENSG0000	UQCR10	22:297673	193.743	146.474	294.288	267.863	172.556	294.327	164.338	203.581	181.26	33.5781	0.30525	-0.36858
ENSG0000	STAU2	8:7342000	21.1402	21.6566	13.5582	15.0517	18.4046	24.9252	25.2286	13.6907	13.1384	37.7278	0.77305	0.125489
ENSG0000	TRBV1	7:1422991	1.42322	0.885124	0.378634	0	2.31728	0.456198	1.03483	1.35644	0	0.251413	0.64405	-0.72599
ENSG0000	FAIM2	12:498668	0.228662	0.237956	0	0	0	1.69145	4.8162	0.676607	0	0	0.1158	4.19085
ENSG0000	SLC25A12	2:1717843	9.92406	11.4342	6.5553	11.1168	6.03668	15.5071	10.925	9.04299	3.44841	10.796	0.91585	0.073625
ENSG0000	ABCB6	2:2192097	2.35721	5.60439	1.23902	4.17021	1.8223	4.22225	3.22104	0.343496	1.98506	3.36336	0.7923	-0.21608
ENSG0000	ALOX12-A	17:685386	10.0749	6.04151	11.6433	14.2375	8.57266	8.33048	8.74785	8.83821	7.0307	6.6046	0.437	-0.53244
ENSG0000	TRAV8-5	14:219030	0	0.982656	0.132191	0.334692	0.547469	0.441305	0.516531	0	0	0.168396	0.59205	-0.92655
ENSG0000	AZIN1	8:1028263	30.1114	36.1595	16.6424	18.9309	17.6998	57.8937	28.9911	25.8665	20.4896	16.6806	0.55525	0.199353
ENSG0000	CTC-205M	5:1807847	0.559466	1.16281	1.51863	3.2846	0.774039	2.51195	0.827926	0.496703	0	1.05708	0.57425	-0.78391
ENSG0000	RP3-449C	22:382906	4.17462	8.25802	1.60348	2.7793	1.97198	16.9852	8.29185	4.41756	17.6278	5.02571	0.1153	1.53895
ENSG0000	ABCB1	7:8750363	69.9349	6.41273	28.4941	27.077	21.4969	25.8202	10.9872	40.6061	28.1042	36.3644	0.73145	0.130909
ENSG0000	SLC46A1	17:283575	0.476012	1.39728	0.836141	0.763688	2.04024	1.98822	1.61572	1.25304	1.77706	3.34849	0.38445	0.959836
ENSG0000	PPARA	22:461505	2.01407	1.74164	1.03476	1.30261	1.61452	2.88506	2.8384	1.22257	1.08187	3.20533	0.9379	0.051138
ENSG0000	FASLG	1:1726590	35.9833	6.08702	47.2441	31.453	38.7564	29.4723	103.431	84.9685	38.2413	80.1136	0.0031	1.0202
ENSG0000	OAF	11:120210	2.46981	8.65816	3.84209	4.29501	2.56809	3.91117	1.4494	0.179962	0	0.80806	0.14855	-1.9162
ENSG0000	WHSC1	4:1871423	6.72213	10.4244	2.87361	1.30601	3.1352	7.544	10.617	7.92299	1.61791	10.9103	0.32515	0.519933
ENSG0000	C19orf53	19:137645	152.172	109.758	176.257	212.668	126.596	224.391	177.075	155.993	134.217	34.2481	0.66505	-0.23828
ENSG0000	CTD-3137	19:184414	1.7914	0.983989	0.421044	2.74196	1.2515	0.241056	0.772213	1.24502	0.693917	0.801903	0.53435	-1.08038
ENSG0000	ZNF525	19:533306	5.73507	3.80807	4.38854	5.55327	3.45923	8.75396	1.96724	1.87036	1.42517	5.99652	0.7925	-0.25296
ENSG0000	OSTF1	9:7508854	130.047	72.6038	141.394	176.821	127.827	169.572	157.236	145.291	247.629	82.2249	0.3741	0.285727
ENSG0000	C14orf1	14:755748	23.2582	15.6519	28.5745	22.3294	27.0469	22.6178	28.8991	20.6724	22.9621	11.8856	0.71585	-0.16354
ENSG0000	SNRPA1	15:101281	92.514	115.249	103.049	119.882	89.8941	104.121	110.68	120.83	98.2141	54.4546	0.7679	-0.10686
ENSG0000	RP11-366	4:1650123	0.935191	0.331057	0.348633	0.375261	0	0.332289	1.02892	0	0	0	0.76755	-0.45566
ENSG0000	HMGN4	6:2653840	127.262	103.735	97.772	93.6778	87.6202	95.4302	98.856	77.0542	117.687	52.8513	0.53645	-0.20084
ENSG0000	RP11-510	16:820350	0.422854	0	0	2.12786	0	0	0.24666	0.272354	0	0.377494	0.55235	-1.31118
ENSG0000	MRPS17	7:5588727	8.92385	8.85908	14.0328	10.116	9.69034	17.8972	12.5284	15.5916	15.7311	1.81064	0.70105	0.309267
ENSG0000	PSME4	2:5386406	7.86997	10.0077	6.24958	5.58153	8.02846	2.44399	11.9017	11.374	8.1924	13.3739	0.63675	0.278887

ENSG0000	MED22	9:1333383	7.66387	8.15756	3.77988	3.3432	4.36209	5.51687	5.64111	4.78304	0.484141	7.99976	0.74605	-0.19603
ENSG0000	EID2B	19:395309	4.96563	7.76221	8.30886	11.4144	5.15766	4.2398	8.48409	6.61337	15.5263	3.64894	0.905	-0.07173
ENSG0000	ARHGEF4	2:1308309	0.564125	3.21264	1.4932	2.00435	0.613433	0	2.47233	1.97229	0.198856	0.574338	0.2843	-0.79128
ENSG0000	LEMD2	6:3377120	33.3168	32.7205	17.0708	17.9707	22.0066	21.3898	37.0651	22.3321	14.471	31.6549	0.8092	0.093653
ENSG0000	DGCR14	22:191302	8.16397	13.6426	12.089	8.86609	13.9952	23.6931	8.1464	9.99036	3.41581	15.2553	0.8519	-0.10265
ENSG0000	C19orf68	19:481706	0.961139	0.894219	0.269716	0.066374	0.679951	0.21232	0.443202	0.099424	0.230331	1.55063	0.9661	-0.05173
ENSG0000	RBM15B	3:5139126	11.0119	12.9291	7.03114	5.19284	7.75642	5.37696	5.52178	6.00567	1.271	12.3706	0.3983	-0.54371
ENSG0000	YPEL2	17:593316	5.82685	27.6681	5.18208	2.33207	6.30538	7.95336	16.2076	6.69885	6.18117	14.425	0.96105	0.029322
ENSG0000	RP11-298	14:229296	2.2513	0.56799	0.568437	0.180308	0.239137	0	0	0	0	0.403121	0.11635	-2.68746
ENSG0000	CD69	12:975248	778.309	189.506	542.068	2231.81	243.008	210.501	270.405	603.091	1172.7	299.999	0.42315	-0.59309
ENSG0000	RP11-571	12:577623	0.882426	0	0.50405	0.683516	0.276786	0	0	0	0	0.400506	0.3158	-2.2311
ENSG0000	ZNF789	7:9947284	5.06623	7.94483	11.7822	10.14	4.31789	4.877	7.9886	2.98943	3.70713	6.70119	0.51415	-0.58122
ENSG0000	KLHDC4	16:876005	41.6913	23.7354	26.5964	35.0152	39.3094	31.8585	23.1034	21.2903	39.6727	42.5942	0.8798	-0.05458
ENSG0000	CD46	1:2077520	80.7922	71.8559	81.8965	78.4978	118.418	68.4642	66.1967	93.3706	73.2662	156.091	0.8305	-0.10069
ENSG0000	ZNF395	8:2834558	5.17318	6.7925	3.46992	2.62761	8.33053	2.6523	5.49969	1.43638	2.62823	5.00796	0.103	-0.891
ENSG0000	RP4-758J1	1:1399521	22.9606	19.4795	51.5472	37.9693	34.7985	9.24099	18.0032	17.2089	12.3008	35.0922	0.28495	-0.90311
ENSG0000	ABHD5	3:4328651	8.03283	10.6296	6.15636	4.73663	3.96074	12.7034	6.80839	2.10181	4.09014	4.97205	0.92455	-0.08948
ENSG0000	DLGAP1-A	18:349603	21.3323	12.2967	14.1135	11.9798	11.2128	27.053	18.4995	8.48711	8.51191	6.61792	0.6712	-0.23349
ENSG0000	KATNA1	6:1495948	20.5015	22.177	17.829	17.6514	18.6345	25.1786	37.5875	16.6728	24.4935	22.7788	0.37615	0.346406
ENSG0000	TRBV20-1	7:1426266	83.1288	111.228	215.176	143.637	207.71	98.9826	112.949	198.844	151.77	63.1993	0.4498	-0.28169
ENSG0000	TIMM17B	X:4889344	70.5781	56.6584	113.445	94.564	94.5674	73.7179	73.9342	118.48	119.303	38.2419	0.91555	-0.04988
ENSG0000	AP001372	11:744917	3.92506	6.27039	3.44634	2.34027	1.96038	1.20178	0	2.63329	0	1.25155	0.4192	-1.5083
ENSG0000	CCDC66	3:5655716	33.5025	39.2938	27.069	33.2847	24.5054	27.3774	23.9224	17.5209	32.8183	38.8222	0.64135	-0.21663
ENSG0000	RP5-867C	17:471177	1.19503	2.30183	0.061724	2.27053	0.195061	0	2.23332	2.70425	2.95309	1.95621	0.6299	0.877899
ENSG0000	ITGB3	17:472538	0	0	0.247213	1.52788	0	0	0	0	0	0	0.0189	#NAME?
ENSG0000	ACAD8	11:134253	25.9182	30.2994	26.3369	32.1893	16.3401	17.4282	22.4495	29.3183	29.9309	18.2429	0.7439	-0.11245
ENSG0000	CTD-2313	5:5497786	1.60289	1.18182	9.89992	14.033	3.73655	1.5813	0.851602	4.87292	3.79356	0.715686	0.2162	-1.40703
ENSG0000	RPL38	17:742035	276.301	280.954	367.165	261.849	107.661	360.856	370.265	250.081	157.021	23.8937	0.6401	-0.23867
ENSG0000	SEPT7P2	7:4572377	17.4243	17.4479	10.4128	12.3662	10.1704	19.8778	15.0011	14.8341	9.22764	20.3707	0.6491	0.183586
ENSG0000	CSNK2A2	16:581295	8.67985	9.9915	8.3713	1.70216	6.76766	3.79406	7.84375	7.14687	1.15779	11.4569	0.53995	-0.26554
ENSG0000	ARPC3	12:110372	547.837	454.626	494.263	480.06	321.988	772.056	708.68	471.69	556.245	127.968	0.59605	0.19322
ENSG0000	WDR13	X:4859004	28.8986	23.8692	22.6023	37.9796	27.9034	19.471	16.5967	26.0123	22.3024	21.2039	0.18975	-0.47193
ENSG0000	PLGLB2	2:8774808	1.66573	1.71893	0.268364	0.529212	0.416286	1.27024	1.08158	0.431402	1.63018	1.62661	0.907	0.202794
ENSG0000	ERI1	8:9002146	10.3308	11.7459	3.51972	4.69356	2.67161	11.8712	16.2925	5.73442	8.21574	7.46011	0.11695	0.653219
ENSG0000	CHCHD10	22:237630	1.81908	1.11489	1.13503	3.13101	2.20863	2.75272	3.45182	4.19982	3.21215	1.2328	0.69985	0.556567
ENSG0000	MCF2L2	3:1831780	1.77529	2.66102	0.623568	0.916457	0.450179	4.78781	4.32761	2.34743	0	3.5904	0.25955	0.935487
ENSG0000	RPS2P32	7:2349047	2.00061	4.03741	1.40987	1.7747	2.21197	5.95398	3.24564	0.206349	0	1.49199	0.62515	-0.18797
ENSG0000	PEX11A	15:896643	0.989243	1.98561	1.6568	2.17023	1.34244	1.57716	1.07119	0.934958	0	0.469617	0.3032	-1.14255
ENSG0000	CAPZA1	1:1124665	238.923	178.11	185.789	115.917	119.147	262.061	283.454	208.564	141.428	194.666	0.2864	0.367113
ENSG0000	MRPL27	17:503678	53.8081	44.2743	68.0878	76.4743	41.1088	85.0617	96.4564	65.7184	50.2186	17.138	0.83385	0.094979
ENSG0000	RAB42	1:2858932	0	0	0	0	0	21.7612	0	0	0.032113	0.64835	0.14555	inf
ENSG0000	CDC14A	1:1003444	41.8586	38.1865	10.5713	16.1208	9.78888	17.009	30.0161	16.7386	15.3643	14.8776	0.53925	-0.28121
ENSG0000	C9orf85	9:7191150	15.3575	13.2057	20.5741	13.5625	16.7423	18.9673	9.7302	12.6361	11.4792	6.87749	0.3843	-0.4709
ENSG0000	MUC12	7:1009696	1.67198	0.697712	0.582543	2.6544	0.077709	0.476542	1.35453	0.511955	0.543406	0.316427	0.50825	-0.91744
ENSG0000	CECR1	22:177187	24.2327	57.1889	20.7577	16.8989	14.5883	28.9414	19.0342	25.8091	17.6417	32.4314	0.67865	-0.13471
ENSG0000	LMTK2	7:9810688	1.82166	2.54303	1.02605	0.577385	1.2622	0.771583	1.06308	1.93861	1.10301	3.25242	0.8683	0.079345
ENSG0000	SLAMF6	1:1604850	93.7497	62.9727	57.689	48.0938	36.9793	41.023	48.5777	52.9134	41.9483	59.5662	0.34185	-0.30674
ENSG0000	RP11-339	9:1283051	0.543514	0.059179	0.232712	0.566424	0.226495	0	0	0	0	0	0.0725	#NAME?
ENSG0000	RP11-493	12:472377	0.493737	0.944153	1.02547	2.51251	0.244736	0	0	1.16437	0	0	0.42385	-1.86039
ENSG0000	TMED10	14:750532	101.679	66.997	106.967	92.0648	87.9037	85.6203	86.071	96.7489	78.2568	59.9923	0.6075	-0.27247
ENSG0000	CD99L2	X:1507663	5.32159	3.36294	2.56443	2.18206	2.51282	6.9022	3.70976	3.76289	1.55524	5.28848	0.503	0.384944
ENSG0000	DNAJC7	17:419667	31.3497	45.8244	46.3958	55.2517	75.9061	36.6634	50.8812	34.3336	36.6766	53.3191	0.4807	-0.34934
ENSG0000	CCR10	17:426788	2.29794	6.24556	0.371467	0.984943	0.038355	0.828083	1.62273	0.518064	13.1221	0.408903	0.2688	1.21124
ENSG0000	ELP6	3:4749563	21.942	14.3986	29.0024	29.2973	22.5379	23.6658	28.357	29.1314	16.154	22.1894	0.8889	-0.05007
ENSG0000	FBXO8	4:1742366	20.0558	13.0994	20.6062	23.0421	19.2975	12.7474	16.2348	14.5396	30.4835	9.62514	0.6998	-0.20405
ENSG0000	SLC3A2	11:628561	140.037	94.5916	214.978	234.601	183.581	304.465	313.632	293.845	249.817	209.473	0.06375	0.600113
ENSG0000	KMT5C	19:553398	4.57256	2.45549	1.48736	1.31107	1.41267	4.94628	4.06838	1.64354	0.961472	4.52576	0.4383	0.486329
ENSG0000	RP11-360	17:673772	1.93169	2.64679	0	0	0.075227	0.24145	1.18122	1.27934	0	0.329282	0.83405	-0.40385
ENSG0000	C20orf96	20:270862	0.375878	0.555781	0.807328	0.82566	1.08778	0.066805	0.291917	0.621062	0	0.802853	0.422	-1.14578
ENSG0000	PRDX6	1:1734772	71.5974	78.8449	79.4514	79.5483	89.3327	86.5542	93.8161	95.3521	87.1423	33.3947	0.9407	-0.02725
ENSG0000	EFCAB13	17:472538	0.586178	2.88987	0.875966	2.70615	0.939336	0.04414	0.893525	1.28636	3.06747	1.38406	0.6615	-0.34992
ENSG0000	SLC9A3R1	17:746705	377.613	410.51	470.925	427.384	486.096	265.95	316.18	264.303	308.128	337.622	0.1449	-0.54843
ENSG0000	TXNDC16	14:524305	1.78496	3.75378	0.20443	1.85154	0.485898	0.133136	1.36436	0.232277	0.009165	0.719209	0.24805	-1.48738
ENSG0000	ZNF773	19:574877	5.99188	8.41866	12.0811	4.16947	8.19111	8.56097	5.09791	6.41637	8.34557	10.2967	0.91275	-0.06974
ENSG0000	RPL7AP30	4:1127880	0.074133	0.297952	1.23867	1.9112	0.444037	0.465543	0.055402	0.688448	0.77068	0	0.54925	-1.01529
ENSG0000	RP11-585	12:754806	1.25903	0	0.388453	0.74508	0.830679	0	0	0	0	0.807968	0.3604	-2.2361
ENSG0000	CASP5	11:104994	0	1.89984	0	0	0.426274	2.66834	0	0.04353	0	0.39324	0.894	0.200608
ENSG0000	LRP11	6:1497243	0.112281	0.069598	0.231533	0	0.025445	2.10275	0.063057	0.500524	0.016979	0.282171	0.1451	2.53291
ENSG0000	HDDC2	6:1251190	50.5103	69.0568	51.6236	44.7698	38.0608	67.075	62.6843	47.1859	52.8974	15.45	0.8944	-0.04829
ENSG0000	TTC33	5:4051233	12.1381	10.7335	7.55646	7.41487	5.61504	13.9976	9.79799	4.75975	11.588	3.89893	0.9772	0.035997
ENSG0000	SNX16	8:8179182	3.49317	4.23658	0.653549	4.41386	1.55388	2.36167	0.779711	3.54213	4.78255	1.50059	0.7213	-0.21586
ENSG0000	ZNF468	19:527974	15.2842	8.56474	5.47779	8.72993	3.42736	6.32729	9.70898	5.76038	3.34688	4.96901	0.30455	-0.54794
ENSG0000	DDX41	5:1775115	40.4016	54.6281	55.3507	64.8679	47.082	66.3092	29.6863	32.7602	42.903	41.15	0.345	-0.30559
ENSG0000	GAB2	11:782152	0.159631	1.61108	0.018811	0.013505	0.165684	0.313239	1.09875	0.668366	0.186445	0.638219	0.80675	0.293529
ENSG0000	RP11-61L	18:947500	0.641393	0	0.602905	0.241433	0.712449	1.13072	0	0.869598	0	0.505646	0.97375	0.051115
ENSG0000	RP11-563	1:1941494	1.111	0	0.78412	0.984004	0.414784	3.96407	0	0.141275	1.07821	1.25706	0.6963	0.87689
ENSG0000	SPSB1	1:9292879	3.50064	5.27288	0.372351	1.06607	1.38039	10.2604	11.303	5.90458	2.81777	15.732	0.0169	1.74802
ENSG0000	ANO8	19:172921	0.175296	0.72324	0.303422	0.664101	0.880639	0	0.30135	0.924604	0	3.43289	0.96375	0.060445
ENSG0000	SEC31A	4:8281866	70.6606	69.6409	31.5725	46.0529	25.8238	48.5157	29.7856	32.2772	49.7274	38.3457	0.74965	-0.1168
ENSG0000	RP11-78J	13:526000	4.90102	3.71743	4.81902	6.59061	2.62494	3.83076	1.644	4.07164	1.34606	2.93411	0.63725	-0.66638
ENSG0000	CSTF3	11:330394	17.7989	36.9506	24.1882	27.3495	19.9043	26.1922	43.3421	39.727	30.008	26.4817	0.5282	0.405414
ENSG0000	SLC25A29	14:100207	36.315	16.3573	39.4419	18.0467	81.387	10.743	21.466	22.3627	15.2393	45.9862	0.18285	-0.78043
ENSG0000	PADI2	1:1706676	0.782598	4.04692	0	0	0.009036	0.240369	0	0.100742	0.011111	0.026397	0.1137	-3.19701
ENSG0000	OTUD1	10:234394	14.0224	26.999	5.15769	4.74536	4.29529	10.1371	12.277	7.26778	4.18848	6.47527	0.4235	-0.37153
ENSG0000	DTWD2	5:1188373	1.10276	0.666385	0.605317	0.20268	0.454867	0	0.798403	0.284599	1.75057	0.718945	0.91285	0.173616
ENSG0000	JAG2	14:105140	0.28789	0.933332	0	0	0	9.32828	10.5169	3.32177	0.455709	5.09154	0.0039	4.72443
ENSG0000	MPP1	X:1547786	7.12444	19.3448	7.45784	10.5434	5.68957	38.4877	10.0585	4.74264	11.9365	4.96292	0.3368	0.451504
ENSG0000	FAM200A	7:9954630	5.68423	6.95805	3.94335	1.01369	4.16821	13.9723	3.88621	2.1556	2.86392	3.6402	0.8954	0.20706
ENSG0000	RPL18A	19:178598	990.247	860.675	3030.15	2253.62	1529.67	688.228	779.72	1831.55	1595.3	190.14	0.1235	-0.78832
ENSG0000	MAP3K7	6:9051357	21.8152	16.8496	16.6024	16.8088	14.2858	16.9882	9.86412	15.1515	12.5348	22.1067	0.4282	-0.27985
ENSG0000	GOLPH3L	1:1506462	11.5005	11.6384	7.5923	8.93793	7.23696	8.02217	4.03087	6.65862	2.22135	9.80238	0.1921	-0.58995

ENSG0000	FCHO2	5:7295598	1.91263	0.050696	0.762982	0.433546	0.754364	2.1794	0.75539	0.072824	0	1.46732	0.9833	-0.02317
ENSG0000	ANKRA2	5:7355233	27.1746	32.3194	23.9428	20.7056	13.8477	33.1777	30.8779	18.5989	13.0592	20.6167	0.94135	0.029213
ENSG0000	AC142472	17:451467	0.661981	0.998736	0.732942	0.451572	0.30464	0.306233	0.136644	0.367843	0	0.551153	0.60535	-1.18315
ENSG0000	ATXN7L1	7:1056050	10.9326	21.151	4.29447	6.52574	5.30043	17.8295	7.34716	6.87138	2.89102	10.4698	0.63345	-0.29935
ENSG0000	DNAJC27-	2:2494363	2.90706	1.71135	1.93893	4.56702	0.134336	5.97815	0.371229	1.92133	1.24348	0.160252	0.98225	0.035531
ENSG0000	CTD-3065	8:1440780	2.49076	2.09278	2.97567	4.25922	0.532111	6.58141	1.49414	3.57075	0	1.51984	0.9943	0.008263
ENSG0000	RP1-20B2	1:1740675	0.672671	5.58676	2.36944	3.15026	3.35234	2.87399	3.98401	1.89706	5.42978	0.342618	0.9697	-0.06276
ENSG0000	HCK	20:320521	7.85394	30.5721	1.45992	2.41098	1.98101	82.1797	2.98156	3.21589	9.16715	2.04534	0.08645	1.23274
ENSG0000	EIF1	17:416888	1076.11	855.726	909.285	1175.76	770.026	888.82	954.438	1236.54	1110.38	637.322	0.99645	-0.00211
ENSG0000	TRO	X:5492046	0.167521	1.38864	0.591708	0	0.103837	0.010034	1.88811	1.29299	0	0.644093	0.30645	0.830323
ENSG0000	SAT1	X:2378317	280.002	350.729	233.485	236.673	120.024	769.323	720.35	442.957	462.647	210.605	0.00505	1.09926
ENSG0000	ZNF772	19:573634	2.32328	2.1597	0.425006	0.162875	0.30936	4.59124	0.668401	0.588999	0.055409	0.649678	0.85725	0.242211
ENSG0000	SNX6	14:345567	116.704	70.4202	75.4216	69.6694	84.5967	104.152	120.311	63.3045	79.5533	49.9737	0.964	-0.014
ENSG0000	WDR77	1:1114386	21.4484	27.7208	20.6601	29.8487	19.1781	16.0304	15.5763	11.1806	17.0434	15.4338	0.50195	-0.73269
ENSG0000	RPL23A	17:287193	1213.18	1174.62	3180.56	2254.87	1128.33	864.274	1111.4	1986.78	1589.73	183.367	0.3626	-0.71422
ENSG0000	RP11-347	16:303554	0.513101	1.03555	0.137789	1.04915	0.470295	1.78737	0.081271	0.389358	0	0.364314	0.8501	-0.35761
ENSG0000	WWP1	8:8633327	15.434	15.7286	9.31169	9.65941	7.02116	7.80421	8.77906	9.27116	12.1474	12.0337	0.7522	-0.14064
ENSG0000	ZNF32-AS	10:436288	0.5773	1.31971	0.407048	0	0	0.700812	0	0.893805	0	0.408423	0.96905	-0.0709
ENSG0000	FAM122C	X:1347695	6.19355	4.25283	6.0779	4.33946	2.91478	5.51653	6.26316	4.8573	0	3.66849	0.57245	-0.41308
ENSG0000	CHSY1	15:101175	8.43406	9.29073	3.14903	2.54972	3.743	3.43322	7.95705	3.03843	3.82442	4.89076	0.73975	-0.16244
ENSG0000	IGLV3-25	22:226867	0	7.37933	1.27107	1008.56	0.107359	0	0.302013	0	0	0.134767	0.1125	-10.9591
ENSG0000	RNU2-2P	11:628322	16.3843	0.816332	3.08198	5.79491	0	0	1.67595	0.571128	19.7888	2.67233	0.6151	1.10366
ENSG0000	THRA1/BT	17:481334	3.14062	11.7951	1.36774	1.18439	0.949066	18.6377	18.4799	2.28099	0.758455	9.04627	0.29305	1.57007
ENSG0000	MSANTD4	11:105995	4.98103	3.73133	3.22145	4.39052	4.48558	4.71038	7.2207	8.51485	0	6.81071	0.56835	0.390612
ENSG0000	EGR3	8:2268765	0	0.290719	0.05841	0	0.047482	0	1.75276	3.6387	0.026657	1.44857	0.0417	4.07111
ENSG0000	MINPP1	10:875048	11.6146	5.78211	9.01175	10.9706	7.33643	14.6718	17.9758	6.3416	0.488926	8.91873	0.92095	0.04869
ENSG0000	ARID2	12:457296	5.49025	7.72095	4.43208	4.19723	3.30016	5.99038	6.5247	3.96886	4.02314	6.83032	0.63315	0.199012
ENSG0000	EFCAB7	1:6344076	7.68153	6.79421	2.30271	5.55754	2.02661	4.85999	2.00278	3.46463	3.98952	4.31503	0.7843	-0.3106
ENSG0000	BRAT1	7:2537876	36.9462	53.2561	19.1846	19.7485	18.1747	27.0733	37.6554	22.3469	12.8659	26.6713	0.4628	-0.27235
ENSG0000	TRAV17	14:219975	20.7884	29.3802	7.02154	5.27015	3.45324	35.8724	76.0173	10.6611	4.2988	2.37665	0.1642	0.983342
ENSG0000	RP11-477	2:5386406	0.760236	0.995312	0.560065	0.636471	0.64802	1.0465	1.39253	0.587885	0	1.68406	0.7661	0.369247
ENSG0000	BTRC	10:101354	4.01623	4.42691	3.18566	4.12371	2.95839	2.03868	0.948043	1.56268	2.79302	5.24938	0.05555	-1.07106
ENSG0000	FKTN	9:1055581	1.4664	1.71992	0.286075	1.97758	0.329412	0	1.26991	0.698672	0.505097	1.95205	0.7989	-0.24818
ENSG0000	ALG2	9:9921642	24.611	18.7539	33.1459	24.8296	26.0801	24.2865	23.5618	21.4791	16.4822	16.1585	0.27095	-0.42377
ENSG0000	RPS2P55	X:4093498	1.98956	1.45412	2.24562	2.02775	0.960603	1.16604	1.07633	0.916163	1.63666	0.168328	0.7387	-0.66539
ENSG0000	RBPJ	4:2616345	36.6948	47.2437	49.5038	35.385	50.901	195.117	267.979	187.355	119.429	184.67	0.00005	2.0011
ENSG0000	EXOSC4	8:1440780	19.7661	17.2564	37.4262	39.6981	33.4832	27.0281	25.976	27.9292	26.6676	15.4036	0.46625	-0.33087
ENSG0000	NELFE	6:3195208	64.3676	56.6986	111.359	93.9462	97.3188	83.5935	74.6233	77.5216	75.8903	43.6591	0.48015	-0.30083
ENSG0000	TRMT1	19:131049	53.8495	55.0784	28.3874	48.8277	33.0122	43.0059	53.2325	25.559	29.59	36.6821	0.4212	-0.26825
ENSG0000	RP11-876	16:108649	0.537533	0.863254	0	0	0.568221	1.16848	0.510165	0.426565	0.255156	1.75546	0.47005	1.15359
ENSG0000	PMM1	22:415607	28.3959	23.8323	24.7563	35.6962	26.2628	36.507	41.0932	38.8312	37.2095	24.6471	0.35005	0.363436
ENSG0000	ABCB9	12:122920	0.266094	0.893653	0.451044	1.50539	0.078948	1.94649	0.340181	0	5.97925	0.823399	0.1339	2.31956
ENSG0000	CMAS	12:220461	37.2806	33.8434	33.9331	25.2006	30.452	41.0495	37.973	27.2591	6.67125	24.2079	0.5212	-0.25372
ENSG0000	NME2	17:511535	165.141	81.7434	181.723	203.863	113.659	155.778	169.873	174.503	122.931	31.644	0.55035	-0.2738
ENSG0000	AL513523.	1:1537280	5.64428	7.98841	2.16995	2.21249	4.44576	7.66624	4.88432	4.25136	7.88797	5.73137	0.55445	1.23608
ENSG0000	TOX4	14:214590	19.3453	22.873	29.4361	38.6814	28.7795	27.7447	29.4091	43.3699	46.0082	36.947	0.9292	0.049644
ENSG0000	VASH1	14:767621	0.238159	1.27004	0.562026	0.106507	0.153974	6.52804	2.23873	1.01895	2.99148	3.37663	0.01335	2.50568
ENSG0000	ACER1	19:630614	0.406397	0.193486	0.919497	1.11652	0.594264	1.41902	0	0	0	0	0.46135	-1.40997
ENSG0000	USMG5	10:103367	181.011	113.348	197.258	145.391	39.069	250.549	231.381	134.374	146.205	14.0952	0.6248	0.268627
ENSG0000	FOSL2	2:2838440	10.9713	6.21669	9.1373	16.6017	10.9778	8.90016	14.2117	10.563	9.36199	12.5974	0.6177	-0.17531
ENSG0000	BCKDHA	19:412624	40.4862	51.6066	83.3316	82.5649	62.4698	36.8137	47.7676	47.0404	52.3976	40.0209	0.3409	-0.56142
ENSG0000	CBX3P2	18:265216	1.51343	1.00068	0.483288	1.52835	0.750847	0.037057	0	1.04473	3.30637	0.066058	0.9806	0.035432
ENSG0000	RPL5	1:9283202	377.2	373.905	634.129	494.177	519.261	244.682	270.125	466.094	397.343	88.7972	0.08495	-0.68595
ENSG0000	ACBD3	1:2261446	18.6521	20.3956	8.72699	5.38081	17.0121	19.7086	13.842	11.5121	4.92111	17.0293	0.76715	-0.10637
ENSG0000	SH3YL1	2:217729-	12.6177	22.9932	15.9758	17.4536	10.5076	6.20108	16.2568	8.29827	18.5727	7.43031	0.4657	-0.43674
ENSG0000	RPL14	3:4045729	300.638	298.86	409.993	304.275	434.026	325.819	285.571	357.426	253.14	49.8642	0.1743	-0.49117
ENSG0000	OSBPL3	7:2479653	17.4868	7.54599	6.13997	9.20097	5.5726	12.6851	11.8639	8.43153	1.12945	14.7068	0.70765	-0.15995
ENSG0000	C14orf28	14:448972	3.1666	2.62307	3.12184	2.74162	3.05925	4.997	4.05053	1.81296	0	1.94623	0.639	-0.43862
ENSG0000	RP11-758	7:1004352	1.47516	0.913595	1.27173	0.506165	0.745582	2.80115	2.2406	0.413733	0.498663	0.235705	0.8974	0.144712
ENSG0000	LRRC75A-	17:164388	138.625	147.455	194.174	181.764	52.2287	144.166	142.465	178.632	182.168	22.5101	0.8898	-0.05887
ENSG0000	RP11-77H	16:867456	1.23615	1.70334	1.03004	1.09355	1.16691	0.035658	0.722888	1.46095	3.22376	1.89967	0.72115	0.355914
ENSG0000	IER3	6:3074292	3.83369	14.4956	10.0097	20.9589	10.7086	15.0801	8.65167	24.4771	36.6689	15.4669	0.15335	0.781319
ENSG0000	KDM5A	12:280128	32.1431	38.7058	23.1739	21.4103	24.3731	17.7254	36.4783	27.2914	17.0745	53.1988	0.8583	-0.08031
ENSG0000	TXNL4A	18:799645	67.8881	47.7181	68.5628	56.5345	49.2512	77.1456	113.861	84.4253	72.0873	15.5488	0.8063	0.125626
ENSG0000	LINC0011	1:585988-	2.8899	2.29064	2.79837	4.01113	3.08833	6.25637	4.16272	3.24538	8.53399	10.7399	0.3576	1.13564
ENSG0000	TRIM32	9:1164252	4.96822	6.66052	6.42969	10.5922	7.05758	1.66131	4.06539	5.92878	6.5081	8.83635	0.2694	-0.4687
ENSG0000	CXCL3	4:7403658	0.246955	0.034217	0.027152	0.317286	1.43027	1.46282	0.083089	0.642016	5.94937	2.76608	0.17015	2.26968
ENSG0000	ARSA	22:506227	21.5141	33.6415	77.6805	95.5337	121.929	53.5148	31.2761	69.137	71.6942	148.956	0.8063	0.095001
ENSG0000	CTD-2035	5:4301441	1.35291	0.35215	3.62973	3.90429	7.91312	0	1.21394	3.62579	0	4.54061	0.55915	-0.97319
ENSG0000	TMEM167	5:8305284	33.2373	21.223	27.1679	24.9093	9.93266	85.1168	48.9242	57.0373	89.4556	9.96169	0.0813	1.11818
ENSG0000	TRBV27	7:1426707	17.7456	65.5617	11.9238	9.54924	15.2402	33.176	148.232	41.3338	1.25884	12.527	0.5082	0.92307
ENSG0000	RP1-134E	6:1060454	1.90387	2.71913	1.69788	11.5315	4.30998	4.71676	5.37085	8.06125	9.39028	2.75742	0.6504	0.523035
ENSG0000	CTB-133G	19:734894	20.1195	34.0868	3.49426	6.46356	5.95613	4.46961	0.770637	0.60526	3.08691	2.06287	0.12255	-2.54227
ENSG0000	RP11-111	2:9899726	0.606052	0.652129	0.170255	0.41657	0.039399	0.033601	1.13096	0.033863	0.255341	0	0.9828	-0.0344
ENSG0000	SELENBP1	1:1513643	0	0	0	4.84529	0	0	0	0	0	0	0.11235	#NAME?
ENSG0000	ATP6V0C	16:247505	18.9039	19.1725	9.55777	7.72775	20.4247	22.7185	25.367	18.4636	10.7755	18.5054	0.74845	0.235716
ENSG0000	DCTN1	2:7419856	50.5412	49.3857	18.1883	11.4693	22.2675	19.5412	22.0473	19.1615	5.17407	31.6547	0.3248	-0.5233
ENSG0000	BTBD3	20:118907	0.067942	0.541862	0.038646	0.273789	0.007524	2.69161	0	0	0.042355	0	0.24885	1.45964
ENSG0000	TIPARP	3:1566718	8.5741	6.86688	15.2535	24.2277	10.7375	6.88891	6.89752	5.78062	15.2254	15.5911	0.2109	-0.61647
ENSG0000	IGLV6-57	22:221957	0	0.480273	0	8.03143	0.544787	0	0	0	5.62042	0	0.75595	-0.49751
ENSG0000	INTS3	1:1537280	13.1342	14.1891	2.48284	1.21099	3.8633	14.6979	7.36387	3.72435	1.23707	11.6196	0.9499	0.043743
ENSG0000	CD24	6:1069698	0.129133	1.10699	0.360191	0.949712	0.538553	0	0	0	0	0.314254	0.24645	-3.86943
ENSG0000	TCOF1	5:1503576	33.0862	27.7315	15.5968	9.02433	18.8273	18.2525	15.5639	13.9036	8.72158	17.206	0.1494	-0.54201
ENSG0000	YWHABP2	11:184558	0.439554	0	0	0	0.065095	0	0.91397	0	2.46858	0.236257	0.2797	3.17284
ENSG0000	MFSD6	2:1904083	19.1383	20.2971	5.40602	5.40875	3.65883	26.741	25.6262	14.9717	11.1109	12.8507	0.28875	0.757956
ENSG0000	TNFSF13	17:754889	2.37732	16.3821	0.171007	3.55426	0.524513	38.3564	0.618504	2.30483	0.090831	1.39648	0.6385	0.861559
ENSG0000	RP11-102	5:3416469	4.68654	6.91211	1.76606	3.47877	0.702438	0.958271	0.963325	2.01363	1.22428	1.92232	0.32055	-1.49982
ENSG0000	SDR42E1	16:819879	1.19465	2.54352	2.47845	1.4198	1.40337	0.237268	1.05089	0.849755	0.62522	1.61774	0.08165	-1.34556
ENSG0000	ZPR1	11:116773	25.004	17.4905	22.4235	15.5383	26.4625	22.32	24.2304	28.9186	27.4429	26.4884	0.6954	0.139981
ENSG0000	ZNF592	15:847486	3.23039	5.35193	0.95563	1.14296	2.5345	3.66243	1.94414	3.65292	1.91425	7.31678	0.70495	0.200522
ENSG0000	RP11-486	16:117974	1.90044	6.42362	6.8136	1.14684	1.70255	2.86194	4.28617	5.21446	3.30697	3.44707	0.84275	0.13882

ENSG0000	ZMAT2	5:1406646	25.3811	14.8772	29.7202	28.6845	33.0182	27.4763	28.0672	24.8599	25.6112	20.7494	0.9247	-0.07979
ENSG0000	UBXN7	3:1963476	5.0578	5.83011	3.0931	2.32469	3.86027	4.6867	3.19284	5.87229	3.45964	7.49439	0.96545	-0.02235
ENSG0000	RPS15	19:143835	610.822	565.749	1491.05	1077.73	899.058	678.948	848.51	1075.15	674.026	139.187	0.1551	-0.53577
ENSG0000	SUGT1P2	3:3268514	0.119257	0.080843	0.197795	0	0	0	2.02733	0.14657	0	0.75337	0.2208	2.92268
ENSG0000	FOXD1	5:7344482	0.174466	0	0	0	0	1.18405	1.40716	0.255806	0	0.414464	0.25075	4.02826
ENSG0000	PABPC1P3	X:7458308	0.268182	0.424107	1.1022	1.49938	0.838723	2.50317	2.66775	2.4559	3.67639	1.4521	0.54355	1.5559
ENSG0000	CSF1	1:1099102	3.59522	1.59941	6.10447	7.15339	20.0149	89.2854	78.6333	60.8608	16.3737	226.223	0.00005	3.15862
ENSG0000	CBR3-AS1	21:361317	1.50446	1.18322	1.05943	1.05869	0.542578	0.069015	0.265164	0.13108	0.662336	0.146862	0.0119	-2.1338
ENSG0000	L3MBTL1	20:435076	4.2777	6.19322	0.661695	1.1495	1.12593	0.915881	1.12557	0.351741	1.23655	2.96385	0.15475	-0.95675
ENSG0000	C3orf58	3:1439717	10.8948	14.1458	5.0014	6.30136	9.67713	12.3142	4.55506	8.16047	6.75687	10.1365	0.52215	-0.2702
ENSG0000	RPL4P4	3:1852829	0.696002	0.643779	0.520502	0.417927	0.299154	0.615625	0.447003	0.336141	0.092003	0.08287	0.58115	-0.74779
ENSG0000	MORN1	1:2321252	1.20161	3.61363	2.25377	0.907155	1.20644	3.6483	1.68954	1.52389	0	1.09625	0.9191	-0.084
ENSG0000	ZNF92	7:6537379	17.017	11.0734	5.69568	6.48017	5.29936	6.73492	5.0451	5.99755	0.925678	6.1693	0.0645	-0.88126
ENSG0000	PSMB5	14:230165	36.7081	41.9378	58.9895	56.7369	70.0882	49.4994	44.5086	56.7355	47.5054	21.641	0.4667	-0.2506
ENSG0000	MT1F	16:566576	20.1829	18.6564	38.5701	16.6099	31.8043	38.2502	22.6423	36.0422	2.42105	12.2262	0.53725	-0.32959
ENSG0000	PSMA8	18:261338	3.6051	0.68197	1.74548	2.6991	0.873486	0.192418	0.864829	0	0	0.453224	0.06855	-2.65415
ENSG0000	ZC3H15	2:1864861	88.579	67.7168	68.6671	51.8966	97.676	110.457	88.0288	58.4353	31.4376	47.3627	0.44655	-0.25232
ENSG0000	C18orf21	18:359720	25.0437	16.2047	29.1516	19.3676	23.3598	35.4408	27.8511	27.0591	25.7953	20.0185	0.5997	0.250854
ENSG0000	WEE2-AS1	7:1417043	0.480161	0	0.194014	0	0.349438	2.74713	0	0	0	0	0.4677	1.23282
ENSG0000	VCL	10:739951	14.831	12.3525	6.35826	5.41211	7.33053	0	0.145374	2.47317	2.19469	5.07786	0.0764	-2.22928
ENSG0000	HSPA2	14:643880	0.374751	1.04201	0.071078	0.111273	0.488044	0.075327	2.51575	3.27216	3.67522	1.00121	0.11325	2.48407
ENSG0000	TMEM38A	19:166611	1.09958	1.85306	0	0.499375	0.106475	0.905835	0	0	0	0	0.39515	-1.61443
ENSG0000	PSMG3-A	7:1567329	2.10639	1.01183	2.04028	0.589367	1.15212	3.64123	1.95782	0.925472	3.24938	0.787705	0.5249	0.568728
ENSG0000	CDKL2	4:7557800	0.615868	0.440681	0.197776	1.38577	0.422662	2.9445	3.2834	1.64414	0	1.33235	0.213	1.62649
ENSG0000	NLRP3	1:2474161	0.651293	2.73101	0.156415	0.641332	0.137602	1.32827	0	1.51185	6.39969	3.46064	0.13395	1.74899
ENSG0000	SNAPC5	15:663868	51.1718	53.2832	90.9377	71.7764	52.5579	67.1678	59.4483	65.2359	39.12	17.7379	0.53375	-0.39445
ENSG0000	LRFN1	19:393065	5.0505	4.61441	1.13023	0.312186	2.48783	0	2.0586	0.99094	0.444179	3.41496	0.5822	-0.77694
ENSG0000	SIK3	11:116835	22.1033	13.8517	3.72377	2.32063	4.46268	7.08006	13.1391	7.26535	1.65995	11.2671	0.8291	-0.10696
ENSG0000	MIDN	19:124855	8.01055	5.99142	13.6177	7.61856	38.4456	7.07038	15.064	20.8062	4.25367	44.3196	0.96015	0.03633
ENSG0000	HNRNPA3	5:1539901	0.065173	1.2786	0	0	0	2.99751	0	0	0	0	0.7544	1.17295
ENSG0000	ACVRL1	12:519069	0	0.049823	0	0	0	2.55578	0	0	0	0	0.45245	6.63567
ENSG0000	SEC24B-A	4:1093474	1.01025	2.03879	1.06624	3.10097	0.984771	0	0.654112	2.92201	4.74552	1.09908	0.8505	0.276594
ENSG0000	SF3A2	19:210098	14.3343	18.7405	25.6013	20.6052	19.1158	15.6988	17.9323	19.3518	8.35203	9.72765	0.4476	-0.59883
ENSG0000	RP11-355	9:1229376	0.307884	1.80757	0.02579	0.689351	0	2.2484	0	0	0	0.567717	0.98555	-0.01906
ENSG0000	MIER2	19:301443	3.19783	3.83266	2.37677	1.37575	4.28801	3.01686	4.49831	3.00555	2.56243	5.5766	0.6855	0.314814
ENSG0000	ACIN1	14:230585	9.04628	12.008	9.37304	5.23999	22.0916	8.2695	13.6555	12.971	6.3059	27.9865	0.6945	0.317172
ENSG0000	ZNF148	3:1252255	23.9206	21.7601	8.34151	4.34913	11.55	12.9951	11.6179	9.87832	10.1904	18.1638	0.56975	-0.27478
ENSG0000	CITED2	6:1393718	96.143	43.35	95.8326	88.4651	72.106	19.794	12.159	43.2455	77.3232	36.543	0.03175	-0.98807
ENSG0000	KLHL22	22:204248	7.06103	10.9619	3.18418	2.71491	4.79202	3.33161	4.67608	2.21441	3.35101	5.91148	0.15975	-0.70912
ENSG0000	SETD5	3:8980590	15.5628	12.0308	13.4098	4.25991	17.6139	11.6182	16.0394	19.2408	14.8939	38.6679	0.1094	0.716729
ENSG0000	NFXL1	4:4759399	6.9458	3.63934	2.04292	4.17511	1.992	5.51257	4.63026	1.11779	0.255259	3.5345	0.7775	-0.19858
ENSG0000	CRTC3	15:905299	15.2491	24.0036	7.48141	7.07165	13.8179	20.8931	8.78058	12.0676	7.82108	18.0435	0.78645	-0.10522
ENSG0000	IGKV1-8	2:8899240	0	5.26943	0	0	0.34656	0	0	0	0	0	0.00135	#NAME?
ENSG0000	ZNF582	19:562196	5.67299	3.92346	2.82561	2.45496	1.92444	4.6437	4.08986	0.701734	0.859955	1.95205	0.5416	-0.58039
ENSG0000	TMEM156	4:3896674	66.9589	34.1948	57.2529	66.3175	39.2082	53.0876	37.5937	28.9311	64.0045	34.3796	0.542	-0.34574
ENSG0000	FBXW5	9:1369404	144.919	137.633	98.247	99.802	115.257	135.472	145.591	85.5814	69.5753	121.105	0.6042	-0.17203
ENSG0000	ZNF44	19:121950	10.3512	10.1816	16.5694	13.9843	12.2713	6.27392	11.3945	9.83623	15.8767	16.5126	0.51045	-0.26296
ENSG0000	SLC2A11	22:238564	3.18784	2.25147	2.77383	1.78321	3.24456	4.79205	1.65809	0.940266	1.33777	4.20987	0.64535	-0.39789
ENSG0000	ZNF80	3:1142143	0.121464	6.45213	2.51327	1.37179	1.75923	0	5.32364	0.55931	1.10042	1.04029	0.61025	-0.65083
ENSG0000	RNF216P1	7:4973987	20.3685	28.6663	26.3368	22.3371	30.5404	25.1426	31.738	19.4167	20.3222	21.9388	0.6751	-0.20665
ENSG0000	TRAV30	14:221684	1.4698	4.53443	0	0.174402	1.55044	4.19365	4.42976	4.46923	0	0.356011	0.60945	0.922607
ENSG0000	AC245100	1:1483629	7.7048	10.3179	11.2763	17.3529	9.94676	4.08611	7.31254	15.3594	9.39145	4.42226	0.67345	-0.49304
ENSG0000	SLC30A7	1:1008960	6.89456	9.40337	3.30561	2.27236	2.80037	7.55852	6.9706	5.20967	7.40144	8.9848	0.1435	0.603458
ENSG0000	CHPT1	12:101696	8.13585	7.86957	9.72235	2.11434	8.86438	18.6655	11.4606	8.55096	3.13984	8.96674	0.47755	0.392082
ENSG0000	PLEKHG2	19:394125	2.15816	8.85387	0.488566	1.56531	1.02165	4.35664	6.29038	3.55109	6.25833	3.6876	0.219	0.979637
ENSG0000	HMGB1P5	15:585875	0.644552	1.03701	0.470777	0.884168	0	0.645994	0.162613	0.456276	0	0.749065	0.7547	-0.44147
ENSG0000	TMEM106	7:1221124	18.4752	19.4798	16.4379	16.1008	8.28117	6.97357	14.8125	12.7021	10.3039	12.1262	0.2356	-0.49941
ENSG0000	RPS28	19:830212	247.171	172.18	296.984	86.075	38.4515	250.859	161.868	196.538	59.5741	13.6119	0.7599	-0.39021
ENSG0000	ALYREF	17:818878	40.8535	40.6387	3.28988	2.79582	6.90026	62.6289	63.375	6.96956	2.12347	6.161	0.75795	0.475881
ENSG0000	LSM11	5:1577436	0.588009	0.672129	0.377619	0.115136	0.599971	1.01695	0.998242	0.061732	0.432947	1.42173	0.4795	0.59749
ENSG0000	CISD2	4:1028689	41.6848	29.3593	36.0506	34.2263	22.622	93.0647	80.1241	45.9509	55.8179	8.42483	0.06485	0.89491
ENSG0000	SERTAD1	19:404215	15.7278	12.0827	11.2565	24.2237	7.94309	33.1171	19.1852	22.378	23.1643	7.87797	0.2166	0.516196
ENSG0000	CST7	20:249492	816.938	166.981	1886.41	1351.58	1632.02	481.787	674.191	1026.46	639.848	323.889	0.02125	-1.00051
ENSG0000	CRIP2	14:105472	11.0224	44.8704	31.6913	16.0532	14.0391	2.43392	22.5402	61.428	28.7447	4.42721	0.7358	0.167984
ENSG0000	GNPAT	1:2312412	35.1585	42.3974	26.1748	20.6078	23.363	24.0106	19.4693	26.7735	24.8045	31.382	0.5486	-0.19237
ENSG0000	KCTD2	17:750325	2.67691	2.48706	1.7615	0.885934	2.52459	2.20151	4.70702	2.28996	4.12265	5.70287	0.47865	0.85706
ENSG0000	GALNT1	18:355811	41.7573	31.4811	45.1376	34.5634	56.7606	51.121	38.4392	43.6809	43.479	76.6758	0.6818	0.148155
ENSG0000	CHML	1:2415321	1.69841	2.44033	0.47158	1.40994	1.42539	3.02385	1.11404	0.241488	0.866122	1.35807	0.8644	-0.19725
ENSG0000	CDK9	9:1277856	32.4237	38.0941	30.23	24.9507	22.6257	40.285	43.8893	19.4978	12.8569	23.4215	0.54135	-0.21453
ENSG0000	NBR2	17:430442	1.90707	4.0453	2.64961	1.52199	1.98605	4.8889	6.86344	2.71701	2.13028	3.21598	0.41065	0.713291
ENSG0000	XAB2	19:761952	11.1455	2.32479	13.0151	11.1805	14.7157	11.5696	2.65865	17.0857	6.1212	18.6311	0.97235	-0.01374
ENSG0000	HDAC8	X:7232951	20.0155	14.3054	18.2841	25.3358	13.751	26.286	22.2419	14.1286	16.9387	15.1301	0.866	0.068074
ENSG0000	FBXO6	1:1166412	28.3002	9.5292	36.8327	57.0045	45.9402	42.2041	70.9139	45.5351	42.1219	19.2697	0.671	0.162535
ENSG0000	ESPN	1:6424787	5.77385	2.71988	2.9498	5.58737	2.20976	5.53814	1.66857	3.59573	4.93478	1.73811	0.96435	0.030661
ENSG0000	NAA20	20:200171	58.0334	44.6805	66.4434	74.7445	46.2012	77.8373	70.7971	60.3989	54.7888	22.1326	0.9742	-0.01115
ENSG0000	OLFM2	19:985371	2.45126	3.4804	3.14179	2.22281	7.32944	3.64659	3.618	3.37247	4.34506	17.7529	0.23115	0.734662
ENSG0000	STX18	4:4348139	39.1728	27.1928	37.9968	39.6916	29.4762	42.9439	24.868	15.5477	11.0005	25.7192	0.20225	-0.6367
ENSG0000	CHN2	7:2897996	13.6226	3.87627	8.37603	8.54668	8.43995	9.24655	3.66231	7.90581	6.13927	7.56226	0.86345	-0.11273
ENSG0000	IGFBP7	4:5696336	1.02075	5.17218	0.70115	6.11558	3.05768	5.23835	0.196384	0.26353	0	0.30368	0.4515	-1.56585
ENSG0000	MSH6	2:4769552	9.59869	10.7963	3.51361	4.3954	4.67157	9.39553	5.41504	7.60121	4.61909	7.31026	0.6169	-0.31037
ENSG0000	ZSCAN9	6:2822488	0.84838	0.076793	1.89018	2.3613	2.88525	0.784927	3.86214	3.33667	7.27584	5.42641	0.09285	1.34139
ENSG0000	RP11-87F	4:1763082	0.129493	0.272077	0.211665	0.536114	0	0	0	0	0	0.256261	0.34225	-2.42418
ENSG0000	EPOR	19:113772	5.52807	6.74145	12.0108	11.7814	6.96109	19.4428	8.1873	7.49862	1.91262	4.92548	0.791	-0.18089
ENSG0000	RP11-231	18:744958	0.234939	1.13052	0.431922	0.111018	0.720644	1.8358	0	0.750434	0	2.23679	0.53635	1.03221
ENSG0000	HIBADH	7:2752544	28.4841	30.8832	34.8575	33.1719	24.5132	28.0166	11.7829	14.9618	13.8813	16.7378	0.0149	-0.96118
ENSG0000	TXNL4B	16:720442	9.85411	9.67142	9.16802	6.7656	6.07847	5.93069	9.68336	11.6645	7.05382	7.39808	0.99965	0.000423
ENSG0000	KMT5B	11:681548	30.1546	36.1487	21.6769	14.0225	18.8676	15.9249	27.4207	19.6124	15.8998	30.3112	0.56615	-0.22306
ENSG0000	EXOSC10	1:1106661	48.0271	61.8144	38.677	39.6189	33.9701	47.8403	58.8087	33.1248	14.3432	41.9093	0.2937	-0.33098
ENSG0000	MAPK6	15:518296	4.54309	3.81453	1.06251	1.11801	1.34276	5.59348	3.82065	3.05491	5.54211	3.66598	0.40735	0.953442
ENSG0000	TNKS1BP1	11:572996	1.2703	2.21987	0.407857	0.181595	0.477354	1.72967	1.03586	0.581232	0	1.36126	0.8661	0.214795
ENSG0000	CTD-2342	19:113681	1.17776	0.712916	1.75862	1.51006	0.367083	0.079887	0	0.315686	1.4977	1.92008	0.87025	-0.27918

ENSG0000	ATF3	1:2125653	0.322177	0.268699	0.196456	4.95414	0.20607	1.08064	1.53118	3.21635	13.8856	0.687531	0.09265	1.85673
ENSG0000	CTNNBL1	20:376939	44.8575	51.0033	48.0361	38.3284	54.9995	39.8933	49.8917	35.5368	41.2127	39.6954	0.61565	-0.19934
ENSG0000	USP38	4:1431849	10.3646	7.59805	2.64396	4.31932	3.28558	2.78725	5.26745	5.94567	5.53444	6.64968	0.64205	-0.21708
ENSG0000	RPS26P3	9:8314245	2.32589	3.5278	2.83445	3.24049	1.11136	2.89772	3.02751	1.41434	2.43572	0	0.76125	-0.50297
ENSG0000	DMPK	19:457647	4.67995	3.37549	0.823202	1.15321	2.6599	8.33866	8.65995	3.17975	0	6.97944	0.2275	0.985976
ENSG0000	ZNF66	19:207763	2.11133	3.41501	0.590116	3.20296	0.965988	0.214799	2.42876	0.766448	2.4331	0.156499	0.6119	-0.70763
ENSG0000	CTD-2659	19:127630	2.39733	1.15374	1.77855	3.45127	0.952934	0.98039	0.979076	1.45945	4.79991	0.19731	0.8112	-0.30058
ENSG0000	NFATC2IP	16:289508	16.0963	22.1605	13.2879	15.4632	15.7151	26.3485	25.7643	14.5287	15.6458	41.2114	0.24515	0.485559
ENSG0000	ZDHHC13	11:191170	12.8135	18.7747	9.87683	11.3911	4.81445	16.0213	17.2028	14.2724	7.52162	13.3272	0.5318	0.246005
ENSG0000	ATP1A1-A	1:1163726	2.5965	4.78986	5.48177	3.58331	2.65939	3.82241	1.64478	0.490564	6.78282	2.11395	0.82085	-0.28496
ENSG0000	CD59	11:336982	88.71	46.7117	49.8377	44.9402	77.1979	105.509	67.5364	58.2464	82.0013	33.6772	0.7727	0.145176
ENSG0000	LINC0056	13:462964	0.591991	0.478686	2.10377	0.428817	1.26151	0	0	0.917861	2.37124	0.098152	0.75495	-0.4736
ENSG0000	TBC1D31	8:1230025	49.3888	14.9051	22.3481	21.9357	17.41	18.8005	8.8984	16.754	17.2295	23.3219	0.3973	-0.53328
ENSG0000	CCDC189	16:306977	1.50946	0.068168	1.56903	2.42445	1.18943	3.1936	2.35926	2.91583	0	1.49305	0.6236	0.441916
ENSG0000	ZNF765	19:533306	5.45138	4.15093	2.77482	2.40196	1.46279	5.61653	2.18571	1.96144	0.552114	3.18447	0.50115	-0.46354
ENSG0000	RABGAP1I	1:1741152	56.1523	51.9963	34.7993	38.5572	34.6624	44.0599	49.6081	53.3613	45.7669	72.9328	0.23585	0.426049
ENSG0000	JUND	19:182797	630.571	462.318	35.739	29.4502	55.9966	274.032	610.61	67.7639	43.4002	60.1855	0.30985	-0.47932
ENSG0000	CCDC142	2:7447195	19.9136	18.1957	9.55325	5.4264	7.78733	29.3606	11.8349	3.61093	3.80966	14.6414	0.9655	0.032622
ENSG0000	IGHV4-39	14:106421	0	134.697	0.932479	289.042	1.94861	0	0	0	0.14453	0.429764	0.0732	-8.90429
ENSG0000	RASSF1	3:5032978	80.7392	85.1493	91.4177	115.793	48.8577	62.7727	111.341	82.0472	88.7134	56.0078	0.81635	-0.07928
ENSG0000	MED25	19:498182	11.249	16.56	2.25159	2.32562	2.8512	8.16408	11.7359	3.87351	4.21986	5.31479	0.95685	-0.04073
ENSG0000	SLC30A9	4:4199047	14.8379	17.9205	10.338	9.69285	10.3094	5.88969	12.96	10.0529	17.497	12.31	0.46755	-0.5104
ENSG0000	LY75	2:1597124	13.1015	15.064	4.66339	4.10452	6.6334	15.239	16.3731	9.58799	4.50066	11.8667	0.57605	0.398938
ENSG0000	GTF3C6	6:1109585	114.194	95.4342	107.846	95.2012	129.977	181.974	125.262	119.188	111.94	33.2605	0.9257	0.030587
ENSG0000	CHD1L	1:1471727	33.0294	25.9262	10.4888	14.7129	12.3791	20.3342	19.9789	11.2015	9.73079	16.1344	0.54355	-0.25763
ENSG0000	CD40LG	X:1366481	32.8394	19.0349	51.5008	53.9169	43.8509	49.0307	3.4027	21.0097	28.9927	58.9342	0.21685	-0.42306
ENSG0000	RP11-521	14:105398	0.666855	0.026253	0	0	0	1.21459	0	0	0	0.173269	0.4282	1.59575
ENSG0000	KIAA0368	9:1113606	9.93208	9.99732	3.67288	1.3386	6.17889	2.04487	10.8976	4.55526	2.43405	6.79365	0.3173	-0.41004
ENSG0000	PDLIM5	4:9445185	13.431	15.1142	11.4162	15.3561	7.53281	24.3293	15.062	13.0663	24.6374	11.2973	0.57475	0.433937
ENSG0000	PSMG3	7:1567329	37.5571	29.0291	99.8516	108.695	58.8342	56.4067	37.2098	72.1732	78.7145	49.3815	0.62985	-0.194
ENSG0000	N4BP2L2	13:324007	195.974	254.614	146.039	157.661	115.468	192.086	263.672	156.745	186.301	248.52	0.6252	0.243436
ENSG0000	RNF13	3:1497611	57.8292	43.4989	69.7581	73.4655	70.0731	90.1996	60.4562	58.4464	51.7362	57.463	0.7974	-0.08637
ENSG0000	FAM168A	11:734004	1.28704	1.65766	0.942377	0.836481	1.41966	3.79778	1.37359	0.697273	2.0335	2.72889	0.23495	0.795855
ENSG0000	PSMA6	14:350449	191.062	113.146	185.16	179.119	147.121	251.428	383.602	216.535	202.271	52.6144	0.35165	0.478253
ENSG0000	ASCL2	11:226849	2.65881	6.69797	2.16464	2.04004	5.45126	6.92345	3.80933	1.07589	0	0.715801	0.512	-0.72427
ENSG0000	LILRA5	19:543070	0.258934	1.04545	0.329828	0	0.454895	0.027536	0.094085	4.86514	0.956782	0.383888	0.3078	1.90309
ENSG0000	CASP7	10:113679	26.1111	20.9938	20.9011	27.0243	15.8519	27.6688	24.3697	21.3068	29.6929	20.063	0.6979	0.132291
ENSG0000	ZBTB20	3:1143145	12.7794	36.8298	10.9619	9.50626	7.96121	6.3654	22.667	11.9517	21.6787	15.6174	0.8259	-0.10334
ENSG0000	POLM	7:4407224	15.655	24.5178	11.0449	10.4332	8.49912	9.36926	11.3883	7.30038	10.2843	19.0245	0.52615	-0.2913
ENSG0000	NCKAP1	2:1829091	1.57141	1.06754	0.525166	1.78715	0.686743	0	0.303295	0.282965	0.1626	1.02042	0.1321	-1.67751
ENSG0000	RAF1	3:1248443	51.7809	48.3265	34.737	48.5093	23.9398	50.9828	37.7698	37.6009	36.4526	43.8594	0.9625	-0.01859
ENSG0000	RP4-676L	8:2935241	0	0.19353	0	0	0	0	0	0	3.06207	0	0.13665	4.50567
ENSG0000	GK	X:3065335	3.48122	7.3858	0.434948	2.32399	0.992219	38.5888	13.8997	4.01231	3.84928	4.09646	0.00045	1.99997
ENSG0000	NEU3	11:749881	0.873708	2.13772	0.40651	3.76479	0.860407	1.86568	4.97131	1.47744	1.0204	2.09228	0.6893	0.360215
ENSG0000	RRP9	3:5193342	0.221565	0.334971	22.7687	16.0731	20.5427	0.094638	0.372787	18.6606	17.6693	10.7094	0.6054	-0.27392
ENSG0000	RSC1A1	1:1561749	0.399405	1.33775	0.009496	0	0.145005	0.118094	0.983189	0.106954	0	0	0.74425	-0.5832
ENSG0000	AVPI1	10:976774	1.84623	0.548005	4.16432	7.60119	4.82307	5.76119	3.50306	5.13796	15.3939	2.53666	0.2564	0.791944
ENSG0000	LRRC69	8:9110183	0.459766	1.89715	1.24759	0.146054	0.325849	0.182125	1.60251	0	0	0.617927	0.51025	-0.67603
ENSG0000	PCGF1	2:7450504	14.8235	13.6597	18.5684	18.0362	13.7446	20.2707	18.0102	23.5015	9.92928	9.97362	0.9249	0.044919
ENSG0000	LINC-PINT	7:1309417	60.9408	35.1317	40.388	79.0033	18.6002	70.858	76.3195	90.5698	182.303	74.6278	0.0346	1.0838
ENSG0000	CEP192	18:129913	7.77504	8.23561	1.33892	2.66363	2.80205	7.68228	6.04508	2.78114	3.25578	7.14658	0.36475	0.591125
ENSG0000	KLHDC7B	22:505480	2.53812	2.01171	0.86222	0.978884	1.7501	2.53022	5.19589	2.72558	0	2.29748	0.4929	0.591441
ENSG0000	CTD-3138	19:582285	1.9636	0.778207	2.25076	1.01722	1.38929	3.11861	3.03791	1.42626	4.28207	0.733865	0.64245	0.765061
ENSG0000	NOC4L	12:132144	16.9317	23.68	19.4608	21.0746	22.5063	15.1135	20.0375	19.9611	6.07796	17.7948	0.3337	-0.44208
ENSG0000	IRF2	4:1843877	64.7369	54.5229	71.5022	50.8936	88.8848	85.5089	78.9459	63.2071	55.0379	80.7389	0.88535	0.045538
ENSG0000	B3GNT4	12:122203	0.115366	1.91024	0.93196	0.393411	0.09217	0.493614	0.036979	0.41528	0.846257	0.577011	0.8293	-0.26746
ENSG0000	EEF1A1P1	1:9644692	0.556847	0.471278	0.537226	0.807844	0.365318	0.226304	0.160658	0.190186	0.512015	0.157175	0.62415	-1.11137
ENSG0000	AC068134	2:2319612	0.3303	0.472062	0.648457	0.166299	0.486364	0	0.650139	0.339611	2.25299	1.09441	0.4058	1.30456
ENSG0000	BMP2K	4:7877634	1.10797	2.94142	0.114246	0.110383	0.421088	2.78534	1.6651	0.609357	2.04848	1.80352	0.60555	0.477909
ENSG0000	FAM86HP	3:1300818	0.286068	0.846455	0.273231	0	0	6.72904	0.173544	0.077522	0	0.589355	0.0627	2.49033
ENSG0000	RP11-7F1	14:770245	0.979086	1.68797	0.399751	0.615612	0.404567	0.984051	0.987197	0.47316	1.46856	1.2947	0.73965	0.54819
ENSG0000	SLC4A7	3:2737272	9.70754	8.64238	5.81201	3.90156	6.67695	3.38226	8.69842	7.30401	6.72271	17.2502	0.73315	0.143204
ENSG0000	DUSP16	12:124742	12.8598	5.10077	14.3325	11.2095	8.74374	17.7196	15.1767	31.5281	20.6211	16.6465	0.02765	1.06584
ENSG0000	ARSG	17:682591	4.79261	6.93249	5.51062	3.41124	3.11012	4.44147	7.64687	4.5828	4.3005	7.41256	0.89565	0.095721
ENSG0000	IPO5	13:979536	12.9622	15.4651	5.49984	8.75147	6.10531	12.4855	7.67956	10.5016	3.3903	11.811	0.41765	-0.29237
ENSG0000	RP11-85B	17:924466	0	0	0.295338	3.00745	0	0	0	0	0	0	0.00695	#NAME?
ENSG0000	CNOT8	5:1548184	101.857	106.717	98.119	97.6268	79.458	63.6294	74.0862	63.1724	142.404	59.583	0.4733	-0.21944
ENSG0000	ZNF664	12:123762	6.56441	3.99861	4.73354	6.18538	3.68058	8.66453	5.94087	3.0469	5.46518	4.40321	0.99215	0.00826
ENSG0000	RAB23	6:5708684	0.258264	0.900643	0.090184	0	0.014185	1.30074	0.972823	0.436097	0	0.274632	0.4075	1.32518
ENSG0000	ATP1B1	1:1691056	0.356926	0.443738	1.27838	0.250526	0.262826	0	5.72739	1.05754	1.76436	0.121549	0.2805	1.51783
ENSG0000	ZNF83	19:525885	28.5078	35.5413	17.3363	17.9769	10.0363	26.3377	14.1254	13.2532	25.9665	24.1823	0.92865	-0.03346
ENSG0000	CTC-444N	19:572672	0.593377	0.656825	0	1.03675	0.400432	0	0.02373	0.13387	0	0.890352	0.45055	-1.54407
ENSG0000	GNMT	6:4296075	0.36215	1.91971	0.718542	0.208088	0.036375	0	1.11934	0.262088	0	0.605207	0.75735	-0.77527
ENSG0000	RPP40	6:4994731	8.20565	7.73349	9.16142	8.49944	7.28028	8.5906	10.1174	4.15562	7.35371	4.96195	0.6653	-0.29423
ENSG0000	DPP9	19:467522	28.8806	21.153	14.3569	15.1252	13.4291	21.9769	16.8448	19.2725	15.3211	19.7949	0.8696	-0.05471
ENSG0000	C1orf54	1:1502653	2.38227	2.0954	1.49508	2.82067	2.00151	4.02303	0.812626	1.99341	3.9789	2.00725	0.6977	0.456773
ENSG0000	SMCHD1	18:265573	34.4893	54.5265	9.6476	16.4037	13.8833	26.1114	41.3388	14.8489	15.2313	25.0165	0.98535	-0.00793
ENSG0000	RP11-196	16:310744	1.26028	0.972553	1.21705	2.30635	2.2449	0.498822	1.83975	2.55726	0.500683	2.58202	0.9897	0.017836
ENSG0000	LA16c-36	16:170618	0.866972	0.683958	0.629462	0.100198	0.079993	0	0	0.17012	0	0.415858	0.3824	-1.59193
ENSG0000	IGFBP2	2:2166328	0.289633	0	0	0	0	10.126	1.21347	0.422878	2.60222	1.52193	0.2446	6.10914
ENSG0000	HPS4	22:264292	11.0339	9.08079	8.8373	7.95256	4.81633	5.55114	12.2214	6.4174	7.48383	11.605	0.88545	-0.07361
ENSG0000	CXCL9	4:7591365	0	0	0	0	0	32.1475	1.17568	0.185343	0	0	0.05335	inf
ENSG0000	SETD3	14:993977	44.3785	26.123	19.9954	34.536	26.4623	27.5347	15.4406	9.50887	16.7878	19.4463	0.0329	-0.77467
ENSG0000	C12orf10	12:532952	20.5801	19.7383	36.9865	21.8862	38.6083	17.8496	16.9103	31.7974	17.3992	14.3179	0.57885	-0.51142
ENSG0000	AGBL5	2:2704236	6.93381	8.29971	5.28953	7.68045	4.21492	4.42394	2.30346	3.1962	4.56834	4.13269	0.36675	-0.85814
ENSG0000	IFI16	1:1589999	120.835	137.935	100.253	93.4511	186.586	173.51	232.866	140.555	120.664	196.359	0.40575	0.365396
ENSG0000	TRIM52-A	5:1812369	13.0279	9.98216	20.5431	26.5499	16.9999	13.6457	11.7214	16.8786	13.7836	6.73252	0.53235	-0.50587
ENSG0000	WDR83	19:126432	18.49	16.4878	27.3888	17.3817	24.3017	24.1237	18.562	23.305	13.915	18.2656	0.9043	-0.1123
ENSG0000	TMEM41A	3:1852829	9.84882	8.26086	17.4554	19.5443	21.3131	10.1358	10.4734	11.22	7.15661	17.5844	0.2624	-0.62955
ENSG0000	MKNK2	19:203746	26.4987	17.4205	8.2821	7.9634	21.3266	23.4229	11.7911	8.29167	6.19534	34.0355	0.936	-0.03407
ENSG0000	ME2	18:508790	44.2162	28.657	28.8132	25.7589	32.2476	54.2641	30.9584	22.8737	15.6288	47.6805	0.92015	-0.03928

ENSG000C	CH17-262	4:124479-	0.266437	0.416341	0.142116	0.103236	0.320911	1.86665	0.43799	1.46098	0	2.43324	0.16865	2.39707
ENSG000C	THUMPD3	3:8980590	13.5072	7.85208	17.9573	16.5159	16.6979	21.0886	6.56671	8.73479	12.1666	16.3704	0.81215	-0.17967
ENSG000C	IFITM2	11:307630	527.698	571.367	1083.58	1513.17	1538.88	490.07	1124.15	1146.53	1137.75	681.257	0.7167	-0.32187
ENSG000C	RINL	19:388678	30.3602	22.5514	29.8111	27.1287	37.1201	36.4475	40.3193	28.9238	27.6339	39.3731	0.7617	0.112878
ENSG000C	TRIO	5:1414370	0.525273	0.976476	0.030155	0.959683	0.393399	0	0.087626	0	0	0.131752	0.07645	-3.4715
ENSG000C	LEF1-AS1	4:1080475	0.528848	13.5261	0.568171	0.83962	0.136504	1.13882	0	0.304331	0.136161	0	0.01795	-3.00098
ENSG000C	SLX1B	16:294257	44.2678	61.2381	65.6937	61.2277	98.9348	79.0952	73.0345	81.0775	38.5923	45.7261	0.97395	-0.04651
ENSG000C	ARL5A	2:1517889	33.7831	19.3887	18.8861	31.9463	10.9817	14.5503	13.1006	9.5787	10.7969	11.2382	0.0365	-0.93593
ENSG000C	SLC39A10	2:1955759	10.793	11.7539	5.61308	4.04726	5.47292	10.357	22.7719	11.6263	8.71213	10.0477	0.1991	0.736582
ENSG000C	MIB1	18:217049	1.24109	2.05691	0.519086	0.422757	0.974179	0.658898	1.12292	1.3218	1.00945	1.42902	0.9746	0.016411
ENSG000C	PABPN1	14:231853	35.6176	44.8121	15.6327	6.54232	25.3083	47.022	57.3349	25.5477	7.90546	26.401	0.6063	0.240548
ENSG000C	BSPRY	9:1133495	0	0.754403	0.58453	1.58669	0.414922	1.95703	3.28553	0	0	0.347199	0.7437	0.638194
ENSG000C	GPR84	12:543536	0	0	0	0	0.197081	8.52664	0	0.996023	0	0.113374	0.23495	5.39092
ENSG000C	PHC1P1	12:554117	3.14473	3.65272	0.281747	0.31672	0.151452	3.00588	0.918363	0.744719	0.592456	0.551948	0.6834	-0.29922
ENSG000C	ITPK1-AS1	14:929369	0.867277	2.89491	0.041455	0	0	2.52722	1.91022	0.363687	0.058197	0.04119	0.85225	0.520737
ENSG000C	PCMTD2	20:642556	32.4739	47.1812	25.3248	27.9451	16.9745	19.3549	17.5885	20.5364	14.238	24.3878	0.038	-0.76545
ENSG000C	ELL	19:184414	21.004	10.26	8.67473	6.78525	11.3882	26.7142	20.6273	11.0872	7.12954	22.7272	0.2906	0.408205
ENSG000C	SLC36A4	11:931441	8.45393	5.47915	10.1077	3.65423	3.93328	18.2029	10.7954	7.96659	11.749	3.99749	0.36055	0.551975
ENSG000C	EIF4EBP1P	14:213851	0	0	0	0	0	0	0.565778	0	1.77907	0	0.148	inf
ENSG000C	YIF1A	11:662685	41.5254	42.6106	70.7375	63.6498	69.8933	84.3388	85.7052	83.0597	67.0705	28.4655	0.82195	0.171064
ENSG000C	RAB11FIP2	10:118004	6.20203	7.31384	1.84089	0.804916	6.77352	5.22004	10.94	5.26398	1.90642	6.48076	0.90005	0.099159
ENSG000C	PLEKHA2	8:3890123	2.76748	3.09675	4.26448	3.34433	6.24093	5.17161	7.05259	6.24826	12.1987	10.3999	0.042	0.824917
ENSG000C	OXCT1-AS	5:4173006	0.267016	0.110151	0	0.176991	0	0.507159	0.600639	0	0.344953	0.079984	0.3922	1.63897
ENSG000C	RABGAP1	9:1229408	8.94128	12.5957	1.48091	2.36771	1.74348	4.4118	3.83907	1.56074	0.216928	4.37293	0.0734	-1.0261
ENSG000C	RP11-295	15:491060	1.92278	1.1491	0.514416	0	0.540664	0	0.493893	0.427542	0	0.146309	0.34815	-1.5649
ENSG000C	NEDD9	6:1117345	29.6163	22.4644	16.103	28.4345	16.4603	29.4328	30.5137	24.0664	26.0822	20.9483	0.5925	0.183284
ENSG000C	RP11-430	1:2046171	0.45717	1.1306	0.193913	0.198714	0.40002	1.21654	0.071908	0.066774	0.237048	0.609082	0.9447	-0.1055
ENSG000C	TAF1C	16:841452	16.7471	22.6069	3.73234	8.11626	6.87437	13.5435	12.5859	6.93332	8.11931	12.9231	0.903	-0.04687
ENSG000C	USP45	6:9943237	7.28192	3.29365	2.58341	4.56598	3.01716	9.29196	1.27982	4.34719	3.93221	3.77734	0.9623	0.03159
ENSG000C	CDC20	1:4335895	9.46171	13.7812	0	0.563113	0.542603	9.30107	3.9757	1.63391	5.7131	3.79615	0.8351	0.146957
ENSG000C	MLLT10	10:215134	4.37284	3.96643	2.95905	3.32315	5.17407	3.0167	3.33655	7.08468	7.79582	15.719	0.054	0.860553
ENSG000C	ESM1	5:5497786	1.73997	0	0.372987	0.266717	0.543169	0.395456	0.127792	0	0	1.70061	0.84335	-0.34
ENSG000C	LINC0114	1:4501639	0.578941	0.108038	0.199823	0	0.289048	0	0	0.541621	1.49806	0.498809	0.55095	1.30986
ENSG000C	KB-1208A	8:9786883	1.54864	2.75613	2.53575	2.65924	0.512436	0.113758	1.58601	1.93069	0.652517	3.82933	0.83185	-0.35631
ENSG000C	MRPL45	17:382970	17.185	12.143	22.573	19.3887	17.1157	18.2371	11.7544	13.7299	14.1713	6.53533	0.2365	-0.48338
ENSG000C	LUC7L	16:188968	72.6485	149.856	48.8209	52.4693	35.0434	85.616	73.333	58.0767	46.6377	69.8453	0.86285	0.058904
ENSG000C	EPM2AIP1	3:3698504	5.73846	7.11516	1.63797	1.1834	1.05619	5.20759	3.26279	3.88073	2.74757	6.80361	0.9118	-0.05934
ENSG000C	RPSAP19	1:1016395	1.39879	0.886639	0.919959	0.496398	0.329712	1.18819	1.63108	0.458765	0.050003	0.04478	0.87265	-0.28958
ENSG000C	MYOF	10:933064	0.238586	3.07208	0	0.043819	0	4.43756	0.068391	0.061386	0	0.179737	0.6372	0.741798
ENSG000C	RP11-344	9:1292824	0.196931	0	0.081657	0	0.076836	3.32942	0	0	0	0	0.15025	2.90136
ENSG000C	PLD3	19:403195	33.1706	33.8887	36.7673	25.7245	36.8152	48.673	44.1901	35.9522	47.6273	40.0628	0.2826	0.390211
ENSG000C	SCAF1	19:496421	1.21545	0.843259	0.859843	1.1687	2.21434	0.835231	2.05582	1.96989	0.574993	3.65297	0.70585	0.247559
ENSG000C	CTD-2095	17:321412	0.515907	0.097256	0.314025	0	0.093633	0	0.869243	0.386337	0.219831	1.86969	0.2751	1.706
ENSG000C	OIP5-AS1	15:412839	30.5659	24.0309	20.0333	24.8827	17.4425	51.0052	41.7296	24.0293	30.2759	32.8943	0.45435	0.46349
ENSG000C	GXYLT1	12:420818	2.53128	1.63626	0.761165	1.52183	1.08535	0.67427	1.37784	3.40734	0.575171	2.12095	0.89755	0.114098
ENSG000C	TAPBPL	12:643900	74.5451	39.2129	70.7679	83.9678	87.4739	75.9232	51.4002	53.8657	68.5826	68.5114	0.8419	-0.13559
ENSG000C	AC074366	2:3866590	0	0	0.202685	1.67878	0	0	1.68184	0.793943	0.986166	0.048497	0.53175	1.28931
ENSG000C	VPS37A	8:1722496	31.4657	26.6532	29.3719	18.5317	27.6439	14.8676	36.1909	16.4397	20.4946	24.6682	0.44735	-0.44721
ENSG000C	FAHD2B	2:9708358	14.457	4.09147	21.4947	24.0442	12.5072	16.5896	9.63185	16.039	15.5582	8.79534	0.7077	-0.1915
ENSG000C	MANEAL	1:3779380	0.433703	0.976058	0.532532	0	1.11506	0.676816	4.39822	0.277367	0	1.46823	0.53565	0.902644
ENSG000C	KLHDC10	7:1300705	1.25171	2.18537	0.70753	0.413484	1.16157	2.46245	1.1386	1.10685	0.771701	3.40612	0.44815	0.426113
ENSG000C	IGLV8-61	22:220986	0	0.461737	0	0	1.71784	0	0	0	0	0.117991	0.2577	-3.87755
ENSG000C	FGD2	6:3700564	0.695963	3.0036	1.6173	1.62237	0.502847	6.92196	0.21095	1.47469	0.682565	1.51667	0.59485	0.362623
ENSG000C	GART	21:335039	38.101	37.3659	25.7228	30.752	19.3872	22.0258	28.26	37.2187	36.2614	31.4572	0.9118	0.074862
ENSG000C	GPR180	13:946019	2.52896	1.31336	0.509806	0.793083	0.789944	2.0057	2.25927	1.34509	0.256083	1.62074	0.4186	0.383937
ENSG000C	TTC21A	3:3905199	0.411201	3.27229	0.795938	0.404094	0.177746	0.62571	4.23419	1.22418	2.48913	0.511982	0.28775	0.963157
ENSG000C	CD160	1:1457194	70.9867	4.01351	68.7179	48.5928	45.3929	9.14926	15.348	20.3917	10.6244	7.34203	0.01985	-1.79458
ENSG000C	COX16	14:703250	46.0881	21.9116	61.9168	62.0912	24.629	55.632	48.9167	36.2758	61.0864	6.16479	0.98125	-0.01413
ENSG000C	CATSPERG	19:383357	1.27922	0.734252	1.30959	2.9888	2.49869	0.612319	1.57886	1.13289	0	2.69299	0.3614	-0.80104
ENSG000C	C8orf33	8:1450523	19.5633	26.9177	20.8702	21.0154	14.687	19.4449	22.1938	24.5605	9.05894	13.738	0.61035	-0.22486
ENSG000C	NAGLU	17:425361	22.3778	19.3226	7.78264	8.37028	7.10707	9.15649	13.7421	3.24827	6.63378	14.9263	0.69115	-0.38428
ENSG000C	GPRC5C	17:744248	0.136002	0	0.343003	0.494316	0.03824	0	0	0	4.63874	0	0.2615	2.4581
ENSG000C	CHCHD7	8:5621168	56.7596	66.875	53.6559	55.2012	34.4792	55.9464	67.1233	62.4177	61.8691	20.8353	0.99505	0.002335
ENSG000C	SLC39A13	11:473831	32.2368	48.3214	24.3743	22.1657	20.0385	11.3307	41.8516	25.67	13.4478	29.7253	0.38985	-0.2862
ENSG000C	HOOK1	1:5981478	0.594045	5.39158	0.315408	0.240646	0.902571	1.46494	0.225085	0.519843	0	1.93692	0.35985	-1.09638
ENSG000C	FBP1	9:9460313	2.06175	7.54313	2.6299	3.80848	6.45018	31.9681	3.67606	0.87565	3.28187	5.72514	0.1094	0.898862
ENSG000C	ZNF256	19:579408	2.13546	2.97294	2.23638	3.70186	1.44066	0.148899	2.27768	3.2219	13.8024	6.83109	0.12025	1.09367
ENSG000C	LINC0061	14:967973	0.553071	0.194073	0.630982	0.381306	0.076658	1.02169	0	0	0	0.287891	0.6393	-0.65998
ENSG000C	NEK1	4:1693932	6.76795	5.98535	5.29015	4.79795	5.18948	7.40745	7.28865	3.31208	5.32638	12.7124	0.8196	0.102482
ENSG000C	CNNM3	2:9681624	22.6662	25.8073	11.0498	5.2964	12.9548	15.7372	11.5815	9.59743	5.38138	20.9693	0.69315	-0.23234
ENSG000C	UQCR11	19:157359	202.19	111.216	438.602	411.243	148.58	240.254	238.724	283.012	314.214	46.4139	0.52265	-0.28342
ENSG000C	SENP7	3:1013242	15.9391	13.2671	5.54469	5.14067	6.03154	20.5839	10.5869	9.22367	14.9885	17.9274	0.0821	0.652539
ENSG000C	RP4-669P	22:420855	0.413906	3.0347	0.595634	0.219035	0	0	0.745876	0	0	0.318281	0.4593	-1.8223
ENSG000C	POM121C	7:7534401	11.7159	15.2905	14.2799	7.03737	32.9152	9.47353	6.34219	17.005	4.53283	39.8727	0.69185	-0.19522
ENSG000C	NAGPA	16:488250	20.352	20.8565	23.3891	14.6463	23.2948	17.3129	19.5022	15.4787	10.5049	25.4984	0.5582	-0.30521
ENSG000C	MCF2L-AS	13:112894	0.634679	4.5164	3.89742	1.1168	1.45404	0	0	0.771178	0	0.094058	0.36475	-3.64512
ENSG000C	RNASEK-(	17:685386	25.6209	22.9262	36.8743	56.704	50.2977	34.4953	40.1746	48.2449	57.7671	10.7681	0.7919	0.154611
ENSG000C	NUDT8	11:676279	5.33582	1.548	2.19781	11.465	5.1444	0	6.16816	3.59249	3.9873	2.88049	0.6048	-0.73596
ENSG000C	RP11-212	7:1284558	2.98796	2.86099	0.957521	0.734335	0.965793	6.50115	1.21258	2.86524	1.13057	0.407895	0.8525	0.3478
ENSG000C	CASZ1	1:1063660	1.73224	1.77687	1.58587	1.01111	2.01471	1.54162	2.70023	0.880251	0	4.66378	0.91585	-0.0752
ENSG000C	IMP4	2:1303382	62.8944	67.8501	80.7288	90.3179	78.9169	78.7117	62.7099	81.0535	61.694	39.6073	0.38795	-0.34778
ENSG000C	LAT2	7:7419965	3.30505	22.3543	2.30174	12.4701	2.95372	18.6134	30.6599	2.01307	5.18168	2.3729	0.5076	0.363712
ENSG000C	SLC35E1	19:164790	7.66062	9.7197	4.52619	5.49001	7.7869	7.6925	11.1375	9.87791	17.494	22.4133	0.2342	0.780263
ENSG000C	FCAR	19:548742	0.710753	0.551169	0	0.907405	0	4.26395	0	0.211222	0	0.708659	0.3203	1.19401
ENSG000C	TRIT1	1:3984102	8.03627	11.4144	9.98806	9.65459	7.43621	8.33106	11.0272	6.99083	6.09426	5.36911	0.57375	-0.26615
ENSG000C	PLSCR1	3:1465151	46.5302	32.5684	43.4331	55.3492	35.3227	117.109	179.359	127.187	98.306	47.9459	0.00005	1.35963
ENSG000C	HMGB2	4:1733316	336.857	519.347	211.344	240.4	146.849	549.864	479.911	240.371	380.988	105.868	0.3541	0.347801
ENSG000C	NHLRC2	10:113854	4.41816	3.1197	3.36893	6.0991	3.1354	7.07463	7.63173	4.70632	4.53186	9.37551	0.6843	0.389132
ENSG000C	AC007246	2:3924926	2.5872	3.45464	3.87376	2.80692	2.59697	9.74546	1.33457	1.68331	4.81883	3.05947	0.65765	0.47227
ENSG000C	FAM118B	11:126202	16.0468	13.5274	15.3552	16.3685	13.6467	14.8186	13.8461	11.9745	21.6584	10.5787	0.95015	-0.05329
ENSG000C	RP11-996	12:291491	0.848153	1.34776	0.565765	0.076381	0.065111	2.70035	1.62049	0	0	0.447195	0.6276	0.797632

ENSG0000	AC015849	17:358689	1.2041	0.20703	0.82759	4.25489	2.13788	0.199586	1.90456	2.44139	0.552623	0.628482	0.74285	-0.386
ENSG0000	PGLYRP2	19:154686	0.471039	0.227029	0.755558	0.075146	0.707086	4.85677	2.3934	2.19016	2.13586	0.801324	0.07335	2.41489
ENSG0000	GMPR2	14:241718	55.6421	57.21	61.0134	57.7924	43.1718	40.9616	34.4753	34.3695	42.7595	25.9399	0.19135	-0.59605
ENSG0000	POLD1	19:503842	18.0594	21.8947	6.34821	8.45292	8.32453	16.4238	15.4362	8.62198	7.14279	15.2088	0.97075	0.015459
ENSG0000	SSFA2	2:1818918	2.93201	3.55745	1.53308	0.314763	1.9619	8.90607	2.76674	4.62517	0.47892	7.77678	0.05045	1.05949
ENSG0000	AC092580	2:7671603	0.355606	0	1.48056	0.119724	0.275373	2.62819	0	0.708026	0	0.120257	0.7947	0.358566
ENSG0000	TFCP2	12:510936	7.65156	8.69026	3.6976	5.98269	7.19998	11.6868	7.28151	5.82204	2.84419	11.6647	0.77015	0.123058
ENSG0000	PKD1P6-N	16:149745	2.9638	3.01993	0.220391	1.36974	1.03508	2.88369	2.48893	1.0105	0.096954	1.63144	0.948	-0.06651
ENSG0000	RP11-380	3:8980590	0.996049	0.18951	0.601092	0.174338	0.210186	0	0.335673	0	0	0.194472	0.18595	-2.32909
ENSG0000	EIF2S1	14:672411	60.6193	49.8799	66.5795	47.1216	41.4529	82.8215	83.1451	44.8987	22.839	22.0517	0.58925	-0.29105
ENSG0000	NDUFV2	18:910262	83.7591	71.2029	130.628	113.466	69.4311	176.964	136.754	161.112	153.283	35.0502	0.59525	0.446976
ENSG0000	IGHV4-59	14:106627	0	7.21821	0	13.9606	0.653504	0	0	0	0	0	0.09305	#NAME?
ENSG0000	TBCCD1	3:1865384	17.785	7.68708	19.5232	23.4801	12.0405	8.60142	11.7549	5.04872	25.2464	11.5287	0.6347	-0.37119
ENSG0000	CALY	10:133278	0.025366	0	0.419831	0	0.465485	0	0.348486	0.687627	0.995522	0.516342	0.25315	1.41003
ENSG0000	AC004980	7:7654935	4.72307	3.1149	3.58088	2.03618	4.93337	2.36391	2.53858	4.92272	1.98798	4.17791	0.6023	-0.48344
ENSG0000	RP13-270	18:306680	1.46036	2.48761	1.88872	0.940516	2.17399	1.90224	2.03169	1.20469	0.667933	1.96309	0.84765	-0.26863
ENSG0000	FAH	15:801524	3.4405	1.76598	3.65394	1.95618	3.23355	20.8884	16.9195	4.91398	1.9985	4.33838	0.01035	1.71321
ENSG0000	ACOX3	4:8366281	5.96506	11.5493	9.19825	8.03799	8.62799	4.35884	7.68478	5.0369	9.61383	8.7517	0.67325	-0.27996
ENSG0000	RTEL1-TN	20:636578	1.75723	1.55582	0.06957	0	0.208213	1.88143	1.07839	0.117401	0	0.528178	0.7477	-0.45734
ENSG0000	ANKRD42	11:831569	3.00599	2.52629	2.49514	1.55791	1.54828	0.304028	0.27092	0.589524	0.016761	2.14007	0.1176	-1.83809
ENSG0000	TMEM150	2:8559854	11.3436	8.31362	16.4442	18.8122	15.1425	22.6803	9.62941	7.79883	0.827077	12.1661	0.5729	-0.55993
ENSG0000	RP11-61L	7:1436209	7.97276	6.84404	8.81622	5.50823	3.73004	7.64569	11.983	10.4335	8.70817	5.96747	0.8963	-0.1097
ENSG0000	HADHB	2:2619063	68.2005	73.2262	87.8941	65.8424	58.8963	89.2864	78.0809	49.1029	62.4772	38.9112	0.77465	-0.14877
ENSG0000	PARK7	1:7954290	258.942	197.68	297.359	331.164	263.997	438.803	352.876	349.694	343.344	71.6598	0.5743	0.194801
ENSG0000	ITPKB	1:2266316	17.4102	32.6034	7.50803	4.04872	7.52039	6.49056	9.31878	9.09317	3.50338	14.7912	0.1457	-0.64755
ENSG0000	IL4	5:1326739	0.418703	0.922779	0	0	0	0	3.59662	2.34094	0	0	0.3515	2.27402
ENSG0000	DDX47	12:127259	43.1046	55.2809	62.1964	51.8494	38.0683	56.5796	54.3916	53.8584	36.7799	31.3659	0.76465	-0.10606
ENSG0000	HACL1	3:1556070	30.7499	22.6377	24.2093	18.8107	15.3371	25.2714	30.6167	15.0466	22.2586	12.1594	0.90585	-0.04852
ENSG0000	C1orf52	1:8524995	29.1993	18.7342	29.2139	23.2642	15.5851	33.6152	27.3049	20.2984	17.1671	11.5132	0.5805	-0.27508
ENSG0000	RP11-33C	2:2190693	0.722586	0.278406	1.16928	0.406821	0.216488	0	0	0.346391	2.98455	1.02731	0.596	0.832209
ENSG0000	NPIPA5	16:153636	7.17343	13.2359	2.43023	2.35096	4.39318	8.90849	9.93731	3.59833	2.20354	10.0605	0.9561	0.119155
ENSG0000	RPL29P14	5:1494921	0.15036	1.62851	0	0.298436	0.121444	0	0.120014	0.657191	0	0.309036	0.80285	-0.57405
ENSG0000	DEAF1	11:644232	31.7553	43.0237	42.4239	43.0668	45.8238	16.3797	31.1863	27.1095	35.4062	29.9505	0.2471	-0.49705
ENSG0000	PCNX2	1:2329506	4.11747	8.96292	3.29758	0.981297	4.03213	6.62196	7.98233	5.40159	6.23345	11.1193	0.12745	0.796147
ENSG0000	FANK1	10:125896	1.53985	4.90163	0	0	0	6.94197	7.64831	1.74408	0.899251	0.256059	0.20765	1.58818
ENSG0000	DCTN4	5:1507084	4.164	9.03533	1.756	2.89827	2.16164	2.78344	5.26529	4.5604	1.37297	4.84459	0.82	-0.09976
ENSG0000	SHMT1	17:183153	12.8123	16.7373	18.923	9.51369	13.6272	15.9421	14.5532	10.2366	12.3122	15.7208	0.6631	-0.19016
ENSG0000	RP11-493	12:472377	0.364558	1.08993	1.01671	0	0	2.11503	1.43909	0.305665	1.39323	0	0.5742	1.01359
ENSG0000	FLVCR1-A	1:2128521	7.6959	0.925909	19.6842	8.07356	5.32605	14.304	1.30333	16.0162	7.49034	4.87648	0.9998	-0.00021
ENSG0000	DBNDD2	20:453619	8.36638	8.20139	9.09413	7.51086	16.6571	16.0527	11.4351	10.6862	6.76824	6.03878	0.9842	0.023815
ENSG0000	MAP3K13	3:1852829	12.0106	15.3152	23.0476	22.9993	27.4749	14.196	9.15753	19.0108	28.1828	6.90111	0.61265	-0.42707
ENSG0000	GP1BB	22:197144	0	0	6.47461	29.0376	3.64312	0	0	0	0	0	0.49725	-10.5968
ENSG0000	GNPTG	16:135192	55.7911	53.5085	89.6836	70.422	75.4974	100.722	86.3283	64.0414	61.5694	43.9248	0.88105	-0.05657
ENSG0000	MAPK14	6:3602767	31.544	35.6222	11.1064	16.3269	13.4359	19.8527	17.4249	21.2438	11.076	22.8528	0.17135	-0.46084
ENSG0000	ZDHHC21	9:1461107	1.35125	1.92967	0.734106	0.824195	0.688854	1.4365	2.46229	0.620967	0.677793	1.81639	0.5669	0.353774
ENSG0000	PSMB9	6:3284071	353.725	239.135	645.507	609.362	619.33	528.046	707.844	665.306	577.284	303.364	0.955	0.096028
ENSG0000	DPY19L2P	7:1031751	0.559108	0.013217	0.595137	1.42802	0.477974	0	0.365391	0.289904	0.276687	0.408386	0.34055	-1.24849
ENSG0000	AF011889.	X:1494769	2.66038	4.1441	1.14306	1.78674	0.363927	5.21532	4.42405	3.79607	0.969961	2.07436	0.93135	-0.09423
ENSG0000	KBTBD11	8:1971396	0.724795	1.84015	0.040786	0.01001	0.166515	0.227591	0.342119	0	0	0.39968	0.51105	-1.54104
ENSG0000	SIRPB2	20:144216	1.04965	4.68917	0.108867	0	0.349958	7.18343	0.010984	0.442747	0	0	0.829	0.408018
ENSG0000	RPL9P9	15:823551	279.905	202.756	465.368	316.073	204.735	276.565	300.882	343.28	257.981	34.6768	0.436	-0.29924
ENSG0000	CNIH3	1:2243851	1.75886	1.7712	1.32941	0.928423	0.715932	2.73564	1.39079	2.50911	1.86174	0.060173	0.65055	0.438261
ENSG0000	LINC0051	17:723231	0	0.415735	0	0	0	6.14088	1.08146	0	0	0.72345	0.23105	4.17717
ENSG0000	C9orf3	9:9472670	5.36418	6.14617	2.04496	4.56317	3.35328	3.44735	0.982969	7.53272	10.9319	4.39551	0.538	0.352701
ENSG0000	ASAH1	8:1805599	44.2137	56.721	41.936	48.8235	34.9442	190.066	30.339	29.1655	33.7626	20.3927	0.4125	0.308039
ENSG0000	ORC1	1:5237282	1.29688	1.83881	0.125696	0.611403	0.360236	2.54955	1.6034	2.12659	0	0.574536	0.55485	0.742713
ENSG0000	ARAF	X:4756109	55.6667	58.6986	32.4182	40.5197	39.7561	44.8018	52.9704	45.5296	24.8164	52.0238	0.82245	-0.09771
ENSG0000	AP001437	21:373654	1.54725	0.902327	0.560706	0.300971	0.21862	0	0	0.753715	0	0.430415	0.6047	-1.07575
ENSG0000	METAP1	4:9899561	23.8322	17.7299	20.5485	23.8211	15.4954	15.1912	10.4257	13.1605	12.4794	20.3465	0.18335	-0.43622
ENSG0000	RP11-18H	4:1521007	87.2468	53.15	64.8311	59.322	101.23	26.6733	12.2925	23.9061	44.4048	43.7265	0.0015	-1.17515
ENSG0000	TRAF3IP2-	6:1112990	3.3522	3.48013	3.49692	7.18288	1.90718	5.05641	4.38944	5.79394	2.95789	0.930798	0.9445	-0.05955
ENSG0000	MTCO1P1	1:585988-	10.0749	17.3796	99.3169	150.782	372.57	33.3356	17.5924	78.254	161.044	249.916	0.626	-1.21066
ENSG0000	SMURF2	17:644996	3.52477	5.32151	1.9181	1.72478	2.45767	2.70184	6.01642	2.68597	0.902309	14.0918	0.55665	0.508709
ENSG0000	KLRK1	12:103637	153.285	47.1862	274.683	266.933	162.18	43.7658	261.768	380.83	213.536	100.301	0.54445	0.231085
ENSG0000	RP11-693	11:104901	145.949	18.0084	112.874	189.874	150.975	107.717	38.9368	105.069	68.9742	100.136	0.56695	-0.34044
ENSG0000	EFR3A	8:1319040	19.3567	19.0568	4.17974	7.44007	6.18598	8.76971	8.93441	7.76153	8.37954	12.4833	0.33435	-0.35929
ENSG0000	CTD-2020	17:451909	0.568858	2.97683	0.677578	0	0.054239	1.70042	0.686495	0.293774	1.15872	1.46142	0.83965	0.328841
ENSG0000	NAA25	12:112026	7.70318	6.34913	4.07978	3.93245	3.53995	1.59719	10.0987	9.83666	3.18398	8.30945	0.60355	0.263948
ENSG0000	ACD	16:676449	37.0194	39.3366	37.8319	40.7608	43.7139	40.104	41.1169	32.3429	30.5262	38.1565	0.7017	-0.18671
ENSG0000	IGKV1-5	2:8894730	0.277838	42.1607	0.386048	1228.24	1.14763	0	0	0.658324	0.413232	0.273394	0.0196	-9.61579
ENSG0000	RPAP3	12:476612	26.0191	32.2496	17.9862	20.6841	14.8238	35.6382	23.1234	11.5336	9.9328	12.1359	0.37025	-0.40223
ENSG0000	LINC0067	17:389251	0.729127	1.38137	0.357949	0.22823	0.580172	2.50899	1.19069	1.67284	0.012406	1.43123	0.1923	1.06815
ENSG0000	XYLT2	17:503460	10.6266	7.98089	7.84459	10.2895	4.32822	5.13527	3.8127	4.2186	4.00793	8.55776	0.12645	-0.73521
ENSG0000	ZNF519	18:140574	1.77323	3.8635	1.23857	0.045243	0.70366	3.9339	1.76056	0.901647	8.63937	2.08646	0.08185	1.25537
ENSG0000	ILKAP	2:2381704	52.5433	62.5624	35.6222	44.1612	26.7082	74.5889	73.1243	45.5725	39.5246	21.4973	0.5698	0.195133
ENSG0000	MED29	19:393913	21.0453	21.0414	29.4495	18.3797	20.1448	18.2012	19.6892	25.9249	19.6432	26.4165	0.6819	-0.14828
ENSG0000	POU3F1	1:3804461	0.649329	1.6255	0	0.098493	0	1.6954	1.94261	0.316093	0	0.112987	0.68365	0.67802
ENSG0000	TJAP1	6:4347752	11.8668	11.1329	10.3867	8.86452	18.332	9.11653	11.2959	11.3792	8.33152	23.6248	0.81835	-0.0973
ENSG0000	KCNJ2	17:701669	0.087058	0	0	0.008331	0.041936	1.55174	0	0.677404	0.028063	0.408869	0.14235	4.02225
ENSG0000	TMEM205	19:112961	53.4929	54.18	63.5931	72.1441	76.2599	93.1333	67.6231	65.745	49.3053	34.1139	0.78935	-0.12894
ENSG0000	RP11-327	16:506936	1.87845	1.43136	0.509143	0.47886	0.415859	3.86307	3.2093	2.28016	0.347568	0.773705	0.20175	1.2299
ENSG0000	HPGDS	4:9429853	0	0.290135	0.241593	0.123624	0.049204	1.72777	2.86435	0.838324	0	0	0.1029	2.90731
ENSG0000	FKBP1A	20:130990	20.6769	16.0093	107.981	107.225	106.29	33.8698	24.1078	153.239	151.613	80.1235	0.69755	0.170203
ENSG0000	CMTM7	3:3239167	42.1817	94.577	48.6198	44.3968	39.3453	75.2463	74.8594	64.0615	78.309	54.3004	0.5544	0.347693
ENSG0000	RP11-611	12:688081	0.973023	2.26878	0.422437	0	0.223404	0.47317	2.73232	1.35923	0.294261	0.784157	0.8727	0.27561
ENSG0000	RP11-136	12:118063	0	0	0	0	0	0.312089	2.71239	0	0	0.765518	0.0696	inf
ENSG0000	KLHL12	1:2028910	14.3498	12.0127	11.2448	8.66567	6.3146	5.95967	10.4591	11.1978	10.075	11.8832	0.82365	-0.08226
ENSG0000	C11orf84	11:638133	0.855572	0.220651	0.283991	0.094514	0.276307	2.20984	0.523669	0.92925	0.100537	0.691349	0.32495	1.39149
ENSG0000	RNF125	18:320183	9.91241	8.85728	11.5367	9.56554	9.07328	1.73979	7.40737	9.02823	23.1428	12.7919	0.841	0.143373
ENSG0000	TMEM97	17:283190	2.95881	3.83955	3.29615	5.15461	3.99181	0.907084	1.32297	5.74171	6.40371	3.12363	0.8319	-0.22406
ENSG0000	CERS6	2:1684558	6.72809	5.77416	2.2922	1.10683	2.85329	3.78228	4.95806	3.16818	1.70993	2.59834	0.665	-0.2011

ENSG0000	RN7SL846	10:119726	0	0	0	0	0	0	0.136574	0	1.56666	0	0.18365	inf
ENSG0000	VAV1	19:677271	14.8712	14.9604	15.2351	19.1626	24.3704	19.4717	20.2902	20.6287	19.1366	36.3884	0.4442	0.284606
ENSG0000	JAK3	19:178247	38.153	59.628	28.9471	17.5502	32.3988	42.462	28.4009	46.6098	34.6088	77.5175	0.35175	0.296293
ENSG0000	POLR1A	2:8602021	1.114	1.41602	0.845143	0.396688	1.46279	0.450303	1.4348	2.36269	0.379493	2.02074	0.94765	0.054638
ENSG0000	RPL9P7	X:2383335	7.58873	5.18337	8.23151	5.94637	4.2631	6.85682	6.90024	5.32479	3.9944	1.65872	0.9875	-0.00594
ENSG0000	RP11-295	15:491060	0.161295	0.101446	0.185984	0	0.857929	1.97619	1.11812	0	0	0	0.71875	0.854772
ENSG0000	KCTD3	1:2155673	0.699494	0.494648	0.219544	0.143714	0.145656	0	0.362474	0	2.88948	0.340749	0.494	1.1173
ENSG0000	ADRB2	5:1488252	33.9944	12.831	70.8126	54.2218	58.3507	5.57709	6.91011	19.6361	29.3116	26.3858	0.0003	-1.47224
ENSG0000	DHRS12	13:517679	18.3039	17.9433	40.0475	32.6563	24.322	12.9561	21.2266	22.0527	9.31856	9.65703	0.13505	-0.97458
ENSG0000	IRAK2	3:1016486	9.11952	2.04669	7.1438	4.24069	7.08662	3.93084	4.39662	4.02115	4.84672	7.42828	0.50345	-0.34019
ENSG0000	SRSF9P1	21:362951	0	0.528186	0	1.47006	0.470442	0	1.38791	0	0	0.372464	0.6082	-0.72083
ENSG0000	ITGAL	16:304726	130.895	109.766	50.1743	48.7087	71.5582	86.8983	62.6456	47.5335	34.2143	83.4901	0.21675	-0.39373
ENSG0000	IKBKG	X:1545313	26.7643	20.7321	16.8189	22.1121	22.7727	20.3273	30.6918	20.5162	19.9264	21.6675	0.77275	-0.19311
ENSG0000	ABHD14A	3:5195531	1.99729	2.57967	3.94261	9.61675	3.36578	1.9222	3.1386	1.08722	0.042309	3.29678	0.1355	-1.36126
ENSG0000	CDK11A	1:1702729	36.6832	46.8197	27.2967	27.2596	57.1447	33.6399	51.5964	19.9616	30.9792	45.9521	0.77925	-0.14925
ENSG0000	YPEL5	2:3014694	109.187	100.897	95.8567	204.431	64.5138	123.728	133.978	95.2071	200.63	61.1026	0.9128	0.040506
ENSG0000	RP5-1065.	1:1090879	0.147024	0.304436	0.700466	0.704902	0.062548	0	0	0.328269	0	0.186147	0.3849	-1.91168
ENSG0000	CTPS2	X:1658800	2.86849	2.27654	1.38609	2.35302	1.18205	4.94273	0.610576	0.764279	0.225593	1.27617	0.7747	-0.22842
ENSG0000	ZNF177	19:932377	0.571452	1.422	0.69727	0.480011	0.238117	3.17437	0.383197	0	0	0.64893	0.99645	-0.00583
ENSG0000	EML2	19:456069	22.0675	16.3999	6.03858	16.5178	6.45747	27.2744	27.0378	12.2415	26.1522	8.29251	0.10765	0.625451
ENSG0000	LYPLA2P2	19:787944	0.07465	0.049621	1.02001	1.54625	1.36829	0.36912	0.124954	0.4398	0.995663	0.495946	0.68055	-0.75184
ENSG0000	MORC3	21:363197	30.9636	30.0025	6.49904	9.52758	7.04472	17.8257	18.0589	11.7037	4.59501	11.5483	0.46785	-0.26424
ENSG0000	FBLN2	3:1354913	0.313554	5.99699	0.393843	0.247762	0.117102	0	0	0	0	0.268766	0.2588	-4.16377
ENSG0000	SRP14	15:400357	460.031	467.755	713.117	605.026	437.951	691.503	721.255	609.837	623.25	112.969	0.98505	-0.00729
ENSG0000	RP11-182	1:6474509	1.77879	2.28885	0	0	0	0	0.300117	0	0	0.458179	0.5098	-1.71189
ENSG0000	TUBB	6:3072020	66.7378	148.295	81.7903	78.0614	112.829	117.617	112.955	117.531	108.025	180.32	0.28125	0.358212
ENSG0000	AC159540	2:9741616	13.0311	22.3733	4.81788	6.75559	12.7283	23.0468	28.4811	7.76843	6.84266	52.3824	0.11315	1.15973
ENSG0000	CHPF2	7:1512324	23.6389	17.0056	3.21475	3.71043	5.54045	15.4956	13.4167	6.70824	4.07862	11.4078	0.98915	0.007684
ENSG0000	RP11-313	7:7287936	1.12527	1.77524	0.464559	1.82784	0.066184	2.1876	2.95352	3.37624	1.3298	0.267383	0.51625	0.993969
ENSG0000	DNAJA1	9:3302521	102.222	61.8215	54.3213	62.1533	48.6472	98.2261	112.157	64.1759	83.91	39.8953	0.2437	0.378888
ENSG0000	AOAH	7:3651294	74.7552	59.7311	57.6199	71.2257	28.9749	37.9687	52.3272	46.5469	78.3388	55.5257	0.8263	-0.0703
ENSG0000	CD70	19:658318	4.42215	3.36102	3.86456	4.84816	1.45228	20.1735	11.5599	12.9789	2.23012	0.980506	0.05035	1.28629
ENSG0000	PSMD5-A	9:1208154	9.29119	3.55113	0.681268	5.94064	12.7782	2.14225	1.54779	0.495546	3.57577	17.8765	0.8194	-0.421
ENSG0000	RP11-649	14:496207	19.3356	8.13742	6.30653	4.14393	5.1183	7.21948	6.73482	3.40937	10.2613	11.2403	0.87695	0.134938
ENSG0000	RP11-231	5:6086645	0.698911	2.44839	0.901405	0	0.483999	0	1.05714	0.654928	0	0.143782	0.47675	-1.14688
ENSG0000	VWA5A	11:124115	5.222	9.91174	2.91776	3.46079	1.10114	7.33791	3.95473	5.46726	10.4901	2.60777	0.34825	0.522848
ENSG0000	IPPK	9:9229735	6.24374	7.29732	6.61158	8.42225	5.67784	7.68128	4.46241	11.324	8.04434	9.91845	0.75795	0.235636
ENSG0000	PAG1	8:8096780	17.3068	21.6207	8.81792	8.03906	11.9669	10.8959	18.9461	20.0247	13.3447	24.1285	0.55175	0.184741
ENSG0000	RP11-20I2	4:1166931	2.09862	2.33036	0.431871	0.268175	0.841146	4.93862	0.711499	0.566168	2.12032	0.277761	0.7403	0.552973
ENSG0000	ATP6V1C2	2:1072164	0.630223	0.606884	0.11743	0.340233	0.48011	0.846134	0.974055	0.135026	0.112143	1.10307	0.70305	0.476626
ENSG0000	RAP2C-AS	X:1322030	0.172546	1.89961	0.960013	1.00979	0.101786	0.81062	0.541516	0.999834	0.011715	0.747243	0.6751	-0.55487
ENSG0000	ETNK2	1:2041310	1.70549	0	1.05418	2.8046	2.25211	0	0.628226	2.96222	2.62477	0.930364	0.9623	-0.05231
ENSG0000	VKORC1	16:310744	62.8327	55.675	93.2144	83.69	116.164	78.71	89.7419	101.662	31.3724	42.7714	0.4213	-0.35094
ENSG0000	UBE2R2	9:3375046	10.4316	13.5534	6.24349	3.50019	8.76078	7.91089	8.13192	7.87887	5.86309	12.0439	0.81485	-0.09297
ENSG0000	SUMF1	3:3700813	16.0825	13.6826	16.4046	26.1606	16.1519	6.82053	14.4225	15.8431	20.0228	14.3167	0.44625	-0.32562
ENSG0000	PDCD2	6:1705752	59.1635	42.4236	40.7924	40.3713	37.3739	68.5948	64.4217	40.3593	34.5743	21.6347	0.69365	-0.1905
ENSG0000	IPO9	1:2016228	10.6313	14.5274	5.82508	4.90879	8.80142	15.0355	12.2178	7.4229	3.2677	11.4276	0.9766	-0.01615
ENSG0000	CD151	11:818901	11.807	5.85694	1.86314	2.37266	3.35475	14.7589	11.8623	7.29348	1.51657	2.86096	0.5951	0.532162
ENSG0000	GPRASP1	X:1025991	2.15436	4.41888	0.751425	0.382882	1.0748	0.340246	0.272713	0.391969	0	2.01648	0.18485	-1.71434
ENSG0000	RPTOR	17:805448	4.47123	8.39673	1.54627	5.36698	2.61494	0.671443	2.54692	0.387602	5.20263	1.58245	0.18695	-1.1161
ENSG0000	SCYL2	12:100267	13.5515	14.8558	3.91681	4.89631	5.8347	11.1233	7.00582	6.8033	6.06084	8.923	0.5965	-0.19785
ENSG0000	EPHB4	7:1008025	0.425414	0.984636	0.031659	0.859128	0.214226	0	0	0.382052	0	0.262577	0.069	-1.79601
ENSG0000	LLNLR-30	19:138352	0	0.07459	0	0	0	0	0	0	0.543596	0	0.4945	4.7047
ENSG0000	ABCC10	6:4342736	19.6007	17.6366	7.00373	6.97208	5.80402	13.1382	8.33372	5.20528	8.87612	12.939	0.94065	0.035739
ENSG0000	NDUFS3	11:474659	60.4416	50.3285	80.5497	76.2454	69.0372	81.4386	66.6513	60.3216	63.622	28.8914	0.8405	-0.13397
ENSG0000	ARHGAP1	2:1430913	247	395.005	258.44	235.015	182.581	228.92	283.823	156.264	257.822	151.314	0.34535	-0.2861
ENSG0000	SULT1A1	16:286051	6.32617	9.16336	11.3457	7.91859	4.03728	7.55333	4.14752	2.64994	3.72541	3.15608	0.231	-0.9044
ENSG0000	TRAV23D	14:220864	7.92182	3.5652	5.2336	5.15034	1.40551	12.2687	9.91471	3.83205	0	1.71933	0.75725	0.256571
ENSG0000	RP11-430	15:521151	2.86494	5.0706	4.47037	2.10949	1.11388	0.499088	3.90946	2.90323	1.20568	1.05578	0.48025	-0.72244
ENSG0000	RIOK3	18:234528	42.7656	45.3357	15.8694	27.1874	12.0703	44.6117	21.2816	19.3347	48.3187	19.964	0.43635	0.253729
ENSG0000	TCTE3	6:1697401	1.99035	2.87956	1.21836	0.770802	0.562769	0	5.17067	1.77927	9.38685	3.66677	0.1006	1.50817
ENSG0000	WASH7P	12:14521-	2.13603	1.54285	1.02301	1.56767	2.31907	1.64824	2.57235	1.29363	2.06132	4.25021	0.52665	0.445522
ENSG0000	TM9SF1	14:241718	22.7553	24.7557	20.7296	24.467	18.1374	15.6644	29.6437	29.9614	24.0068	32.5535	0.75795	0.214408
ENSG0000	WDR45	X:4907115	80.8798	66.3503	107.992	114.221	80.1096	56.3851	99.6802	72.9767	90.0322	56.3234	0.4069	-0.32363
ENSG0000	CSF1R	5:1500532	2.49743	22.4838	0.414415	0.448385	0.746248	38.7038	0.017399	2.04033	0.414525	0.218527	0.4796	0.875608
ENSG0000	ZNF569	19:374111	3.12323	4.34073	2.70233	1.49936	1.80053	1.78753	2.71298	2.10998	1.30824	1.22457	0.6112	-0.5401
ENSG0000	N4BP2L2-	13:324007	0.408791	0.427477	0.157833	0.067362	0.351429	0	0.826036	0.521457	0.035024	1.68007	0.5164	0.981613
ENSG0000	ARID4A	14:582983	28.0974	37.6127	27.9337	10.7015	27.4904	38.2102	54.9172	26.1236	24.646	62.9055	0.4221	0.400338
ENSG0000	CCDC74B	2:1301296	0.222429	0.092549	0.034083	0.007319	0.417398	1.93425	1.30106	2.22626	1.81481	0.213139	0.0158	3.76118
ENSG0000	RBM41	X:1070644	15.4854	12.8642	7.95299	9.18434	8.6513	8.10114	10.7271	6.26476	10.3294	11.5106	0.51355	-0.3461
ENSG0000	OMA1	1:5692920	43.9087	35.2744	24.1757	14.6777	22.0803	19.2746	22.5977	18.3766	33.7152	17.8636	0.5027	-0.23837
ENSG0000	KIF18B	17:449247	0.59349	1.11508	0	0	0.096734	0.900853	0.754535	0	0.075201	0.801785	0.7782	0.438862
ENSG0000	RP11-10K	4:1731319	1.0481	1.26146	0.448196	0	0.320616	0	0.100604	0.023139	0	0.473721	0.09505	-2.49175
ENSG0000	RP11-532	6:708591-	1.94494	0.423237	2.94794	0.649048	0.423492	0.776057	1.45866	3.60599	1.73578	0.475025	0.59675	0.413439
ENSG0000	GAPT	5:5849143	0.141827	5.09717	2.00927	4.4404	0.929095	3.46173	0.017061	1.10128	0	0.09342	0.10785	-1.62259
ENSG0000	EPHA10	1:3771387	0	0	0	0	0	0	0	0.010124	4.7388	0	0.04375	inf
ENSG0000	ATL2	2:3829487	14.3972	21.4549	4.36706	7.08729	3.84204	14.5149	10.6547	7.51245	10.7514	5.2659	0.84305	0.079542
ENSG0000	TRIM61	4:1649544	2.42332	0.782939	2.06089	0.842925	1.66494	2.00653	1.89878	1.70024	0.682753	1.19471	0.96495	-0.0811
ENSG0000	THOC3	5:1759069	61.9373	53.9424	45.1924	44.3319	36.5838	49.6629	56.654	27.8216	18.4879	20.7624	0.2317	-0.44478
ENSG0000	ZNF890P	7:5121238	0.739902	0.838019	0.379515	0.223194	1.24909	2.02897	0.072214	0	0	2.9546	0.65875	0.640382
ENSG0000	PPIAP22	21:188577	10.5096	9.59976	3.30702	3.18125	1.66635	11.9573	14.0242	2.67699	2.82123	0.230646	0.79745	0.170781
ENSG0000	ZNF154	19:576819	6.95519	5.78937	0.643325	2.72016	0.465894	1.77403	0.175957	1.84353	0.043557	1.104	0.19325	-1.73177
ENSG0000	PRKX	X:3604369	14.2208	13.7833	12.3794	7.09444	8.78499	10.3423	17.3341	25.6926	12.2881	17.2254	0.4154	0.343284
ENSG0000	AC007383	2:2061148	0.119691	0	0.185088	0	0	0	0.482075	0.68251	2.34361	0	0.1878	2.61707
ENSG0000	TMEM53	1:4440519	4.94169	4.09116	5.42057	9.51561	5.57512	8.65587	5.67753	12.315	10.9056	3.60366	0.5873	0.433701
ENSG0000	BORCS5	12:123570	16.6469	20.0169	26.9584	12.9868	24.9548	13.069	24.3004	19.5443	15.251	11.4147	0.53605	-0.47975
ENSG0000	TRAV25	14:221123	4.70819	13.3634	0.405899	2.4012	1.02016	19.3328	17.3903	1.47124	0	0.259321	0.67725	0.794133
ENSG0000	AC093484	17:164145	0.104075	0.336035	0.847053	0.661344	0.421169	0	0.407019	0.96533	0	0.106301	0.6685	-0.68618
ENSG0000	RPS10	6:3424637	328.148	281.968	800.175	610.34	328.247	279.169	338.825	574.604	401.87	51.3986	0.30545	-0.60469
ENSG0000	MIR3911	9:1275793	0.2516	0.151	1.17627	0.590688	0.692682	1.74912	1.23089	0.806086	6.45568	1.02486	0.27415	1.47124
ENSG0000	TCIRG1	11:680390	275.531	276.426	69.1722	136.317	77.027	504.907	310.685	134.15	114.53	154.299	0.1492	0.509285

ENSG0000	HNRNPUL	11:626902	1.25088	0.05991	0	0	1.28244	0.536457	2.52706	0	0	1.50696	0.8294	-0.43389
ENSG0000	HIST1H2A	6:2783775	2.06587	0	0	0	0	1.49768	0	0.101247	0	0.516939	0.93935	0.076728
ENSG0000	GEMIN5	5:1548874	4.48408	5.64928	1.96489	2.27967	3.03142	4.015	2.05806	2.18899	2.62915	4.21188	0.539	-0.27265
ENSG0000	TSPYL1	6:1162541	8.17787	6.49806	48.4441	33.939	46.1737	5.08263	10.1214	36.9139	23.5346	64.6381	0.7481	-0.14543
ENSG0000	NDUFAF3	3:4891778	82.8793	80.7447	153.519	138.68	150.081	102.916	89.7572	89.5216	108.052	45.067	0.54045	-0.52711
ENSG0000	GSKIP	14:962792	39.406	26.3107	28.7402	38.7099	12.9376	49.5106	32.1877	37.8788	61.8846	13.0186	0.2742	0.517304
ENSG0000	CDCA3	12:683995	3.16081	10.2548	1.15418	0.841278	0.633291	17.3659	9.71228	6.52485	2.82657	2.99983	0.04515	1.49467
ENSG0000	CYB5D1	17:785668	1.39143	2.65209	1.07466	0.299512	0.785886	1.47719	1.87269	2.16628	0.127303	2.23072	0.81105	0.268125
ENSG0000	LOXL2	8:2329718	0.663444	0.832076	0.577297	0.066974	0.35209	0.012698	0.72388	0.137106	0	0.955773	0.4861	-0.66492
ENSG0000	RILP	17:164614	1.66014	1.44489	1.35579	7.50157	0.823529	14.5071	2.11009	2.54112	3.17821	0.959431	0.36695	0.79886
ENSG0000	CMIP	16:814451	0.687052	0.501447	0.56288	0.305672	1.3034	0.192744	2.49126	1.04464	1.10518	2.18647	0.2172	0.936185
ENSG0000	CAMLG	5:1347385	25.8595	24.6038	43.4046	30.1257	33.0555	22.3117	28.6475	23.8411	35.0239	12.3387	0.35785	-0.41086
ENSG0000	CLIC2	X:1552762	0.166589	1.95817	0.01206	0	0	1.63682	0.013078	0.440052	3.29007	0	0.3474	1.68154
ENSG0000	RPL11	1:2369177	202.415	199.652	437.265	291.802	277.657	207.83	272.226	313.588	194.23	66.3031	0.1842	-0.46151
ENSG0000	FBXO27	19:389907	0.407828	0.32502	0.290493	0.522213	0.445412	0	1.27985	0.239695	0	1.74844	0.51735	0.794662
ENSG0000	GON7	14:931849	33.3846	12.2532	27.1454	21.675	12.3532	28.6448	32.4966	31.5615	7.0072	5.11711	0.9686	-0.03954
ENSG0000	RN7SL200	6:3458728	0.562041	0.351738	0	0	0.140351	0	0.141693	0	5.41176	0	0.41085	2.61691
ENSG0000	SENP5	3:1968678	4.27775	6.63858	6.82111	4.67226	5.82468	2.65444	6.0606	7.48501	4.33305	8.18648	0.72415	-0.15372
ENSG0000	DLG1	3:1970425	22.4533	19.7875	3.89413	4.32071	4.86666	9.66112	9.31672	4.98484	7.84547	16.285	0.4435	-0.31487
ENSG0000	LYSMD4	15:995654	4.28944	7.85362	4.55516	3.75549	2.1195	5.04922	7.99584	1.87375	10.4544	4.62722	0.4523	0.619418
ENSG0000	SLC12A4	16:679397	2.77726	5.60851	1.45391	1.20128	1.71587	3.88161	3.25314	1.01747	0.059944	2.94562	0.567	-0.3531
ENSG0000	RP11-664	12:379104	3.57527	25.9233	19.8342	31.5306	1.4697	6.91723	1.07941	6.53998	7.94625	2.00238	0.1772	-1.63605
ENSG0000	SRSF10	1:2396480	87.1446	98.1303	98.6538	95.5383	78.857	84.916	110.458	89.3628	103.077	105.176	0.83795	0.074244
ENSG0000	DDB2	11:472144	30.3166	31.876	29.47	30.3266	23.2704	38.807	35.7412	37.2973	36.3987	16.7435	0.34715	0.379471
ENSG0000	GFI1	1:9247476	21.9779	12.5107	31.9745	33.1539	19.6564	26.8645	50.2131	25.9815	23.4783	44.0462	0.21155	0.440722
ENSG0000	RAD51-AS	15:406867	12.2368	25.8387	19.7728	15.434	9.64078	8.86746	21.6884	22.5241	22.5176	30.5399	0.516	0.36749
ENSG0000	SHISA4	1:2018886	0.149047	1.53479	0.551094	0	0.532973	3.69286	0.508816	0.2703	0	0.35016	0.6502	0.660996
ENSG0000	P2RY8	X:1462571	118.245	116.4	80.2624	74.0063	72.0744	35.8868	17.6077	26.4226	42.6459	50.1228	0.00005	-1.42136
ENSG0000	RP11-404	1:1606701	1.05381	0.505045	1.32777	0.849238	0.798799	2.52166	3.08312	1.62798	1.24363	1.3179	0.1112	1.13293
ENSG0000	ZNF20	19:120922	1.9129	1.98282	1.43451	1.74617	0.413859	7.09601	1.54581	0.052476	3.31946	0.21539	0.1753	0.994343
ENSG0000	PIAS2	18:468032	8.38894	8.90242	6.90727	7.58856	9.41205	3.3344	2.99412	1.0517	2.73835	7.69657	0.01105	-1.56204
ENSG0000	UBE2F	2:2379668	58.9391	47.8547	47.9362	65.6868	61.6623	83.211	54.4026	38.6525	47.5276	18.0202	0.4522	-0.28747
ENSG0000	CTD-3035	17:626260	0.1422	0	0.293122	0	0	0	0	0	2.45056	0	0.5144	2.75045
ENSG0000	RP11-134	7:1349865	0.616235	0.68159	0.141931	1.06309	0.40673	0.049762	1.94882	0.133349	0	0.917713	0.99295	-0.00449
ENSG0000	EPAS1	2:4629366	0.049815	0.473681	0.256617	1.64103	0.067954	0	1.3953	2.10007	1.71197	0.53768	0.35445	1.25135
ENSG0000	CACNA2D	3:5034732	1.90111	0.091455	1.28697	1.29177	1.56556	0	0.370933	0.133337	0	0.718678	0.109	-2.39434
ENSG0000	RP11-499	10:181406	0.869527	5.49032	4.07633	1.38259	2.50978	1.22136	4.61772	2.48663	3.45712	4.72339	0.90415	0.224763
ENSG0000	UBBP4	17:222029	0.66029	0.63618	1.54244	0.852425	2.82733	1.12995	1.06449	1.61108	1.75174	1.31221	0.9522	0.078227
ENSG0000	G3BP2	4:7564278	97.0088	60.5994	54.8713	73.0754	52.872	56.2472	84.736	72.2955	52.8851	57.4823	0.80025	-0.09083
ENSG0000	RP11-278	12:326791	0.451971	0.942031	0	0.915823	0.393972	2.05239	1.66436	1.29718	0	0.822494	0.37205	1.35362
ENSG0000	DPM3	1:1551398	40.5742	21.0609	56.3766	52.5004	19.9441	61.9717	40.307	44.9757	27.8158	5.49785	0.7626	-0.17797
ENSG0000	ID3	1:2355791	13.0026	90.24	7.48529	5.0638	5.6645	56.8651	41.9145	26.0347	8.24895	9.62449	0.5235	0.330546
ENSG0000	MIR1302-	9:14520-3	1.11022	1.94863	0.439809	0.160904	0.121382	1.81877	1.59127	0.630687	0.072731	0.781199	0.7493	0.520179
ENSG0000	CICP14	7:1286559	1.08741	0.198721	0.15614	0.171816	0.245569	0.440567	1.00021	0.456904	0	1.18819	0.7772	0.59658
ENSG0000	IGHV3-15	14:106153	0	2.84543	0	0	0.185215	0	0	0	3.96854	0	0.55395	0.754784
ENSG0000	ARMC8	3:1381872	28.5512	23.7854	31.8438	33.7636	21.9202	19.6362	28.7696	31.2325	20.8163	26.0508	0.44395	-0.31641
ENSG0000	MRAS	3:1383476	0.038751	0.458655	0	0	0.04746	3.59761	0	0	0	0	0.26215	3.18775
ENSG0000	FGFRL1	4:1009935	1.47247	2.65345	0.567414	0.298793	1.69741	0.997572	1.97599	4.41725	0	5.23659	0.2422	0.928
ENSG0000	COQ2	4:8326153	9.4744	8.62227	7.26373	11.8177	5.9232	3.82461	9.35713	6.80561	14.2183	7.33866	0.9542	-0.0249
ENSG0000	MRPS36	5:6921775	27.3551	16.7186	27.4126	23.7814	14.8167	31.6628	22.1373	28.6576	23.6007	4.61386	0.81125	0.099185
ENSG0000	CCL18	17:360642	0.323932	0	0	0	0	65.1788	1.71323	1.92196	31.3763	5.40693	0.2186	8.43787
ENSG0000	CTSC	11:882935	304.67	100.227	244.693	272.532	229.271	532.189	392.344	236.978	160.244	149.159	0.40305	0.307148
ENSG0000	CD81	11:232874	48.9177	22.5367	56.8374	32.9558	101.15	41.9879	49.122	67.7399	16.8749	58.1211	0.4986	-0.24632
ENSG0000	USP12	13:270661	12.8329	7.08674	4.95538	12.7	3.20339	11.436	9.816	9.13398	0.602685	5.04295	0.79915	-0.21989
ENSG0000	PITPNB	22:278516	39.8188	42.0652	26.4512	30.5857	16.4074	33.4763	49.08	25.4508	27.3863	31.2247	0.75735	0.103856
ENSG0000	SNX17	2:2737049	101.833	105.876	147.071	125.963	139.331	100.852	104.069	104.382	134.649	107.873	0.53605	-0.19786
ENSG0000	NABP2	12:562220	1.80872	0.916188	26.537	20.8111	23.5402	3.17392	1.98412	25.9717	31.2812	18.9292	0.8141	0.108189
ENSG0000	RSRC2	12:122503	88.5358	80.2406	64.6257	54.6404	88.9198	108.228	99.2785	42.6063	52.3035	92.2313	0.8614	-0.06397
ENSG0000	RASA4DP	7:1026818	11.7104	11.3205	10.7325	7.68368	13.9854	9.38957	8.94549	9.97292	6.38809	12.9308	0.4857	-0.48775
ENSG0000	PFKM	12:480428	4.26345	2.30313	1.3565	4.42504	2.09384	12.6284	5.07767	3.00794	0.016521	2.88964	0.2688	0.675296
ENSG0000	PRMT2	21:466351	148.605	206.61	230.041	211.117	254.723	96.926	124.929	129.21	133.824	146.48	0.07	-0.77128
ENSG0000	ENDOD1	11:950898	3.40504	1.44006	3.54002	3.03515	4.62621	4.13542	11.6092	2.76328	4.26453	7.34008	0.0969	0.806295
ENSG0000	ZFAND2A	7:1152070	41.2053	20.8046	42.6211	73.8192	29.1396	65.9936	35.2161	48.5508	48.1947	9.69293	0.90355	-0.05239
ENSG0000	APBA2	15:288844	1.82508	33.0847	4.05154	1.92165	2.15979	4.57199	6.39763	9.3028	6.41961	14.2273	0.99495	0.003644
ENSG0000	SNUPN	15:754632	28.9258	34.8629	37.0135	28.9018	34.1886	43.5347	30.517	26.7999	49.762	22.172	0.79085	0.132961
ENSG0000	ZNF32	10:436288	48.8558	31.142	55.6735	54.4412	34.2266	26.7634	29.653	37.0115	54.7118	16.2064	0.2148	-0.44917
ENSG0000	CCL3	17:360728	15.5957	15.9977	70.3113	71.5958	54.3803	251.577	371.112	530.017	160.199	68.8026	0.00005	2.5462
ENSG0000	MKRN3	15:235656	0.892292	0.791242	0.168953	2.76791	0.427593	0	0.520966	0.253967	4.13194	0.799026	0.91685	0.186121
ENSG0000	RP3-395M	1:2546464	14.467	18.5111	2.5668	3.5005	4.78134	10.575	10.5409	4.8419	5.32761	3.00644	0.6882	-0.35537
ENSG0000	CIITA	16:108649	1.31147	7.65411	0.508655	0.995201	0.403717	5.29819	1.00942	2.74027	0.004295	0.62875	0.7296	-0.3389
ENSG0000	RPL26	17:836556	332.434	222.755	565.239	368.411	155.467	356.29	351.479	463.041	275.36	35.0178	0.68295	-0.15919
ENSG0000	CTD-2035	5:4301441	0.545739	1.79499	0.285968	0	0.343195	0.101643	0.20429	0.239011	0	1.06207	0.6592	-0.82654
ENSG0000	RP3-465N	1:2524223	0.36251	0.472454	0.507627	1.39018	0.959657	1.86111	0.893698	0.643977	3.16628	0.129763	0.55025	0.773721
ENSG0000	PLA2G12A	4:1097099	6.51333	7.01475	3.31389	10.1918	6.99216	10.5672	7.82728	4.17247	5.3377	2.15119	0.52045	-0.53582
ENSG0000	ATP6V1E2	2:4649074	5.11673	11.1381	2.58527	4.30226	5.28172	1.20207	4.48839	1.91123	0.537735	5.38123	0.314	-0.94782
ENSG0000	RP11-483	2:1745453	0.675138	0.611109	0.35999	0	0.131313	0	0.160844	0.953073	0	1.28487	0.73635	0.554904
ENSG0000	TATDN2	3:1024802	6.6478	4.36285	6.09507	3.57596	8.5752	6.44185	9.4092	5.34048	3.83195	16.4751	0.6748	0.435729
ENSG0000	ZADH2	18:751951	5.06128	7.16899	5.82518	10.3818	6.01512	1.81006	2.26684	3.40412	0.734173	7.05742	0.02175	-1.7163
ENSG0000	PLA2G4B	15:418280	7.92529	13.7304	4.54709	2.5665	3.37902	9.872	4.85924	3.93482	2.75932	8.27964	0.8575	0.236678
ENSG0000	NCOA1	2:2449191	27.635	26.4617	5.91004	3.46343	11.087	8.66818	14.8265	4.71376	1.93666	16.5843	0.06555	-0.73599
ENSG0000	NDUFV3	21:428796	17.2223	17.499	21.1137	14.1892	23.9658	8.8023	13.4325	11.4051	9.89552	11.9264	0.2635	-0.73446
ENSG0000	LINC0044	13:482965	2.74788	0	0.140877	0	0.330011	0.414561	0	0	0	0.464818	0.27455	-3.22986
ENSG0000	CSNK2A1	20:473590	19.8486	21.9374	15.1556	10.6856	12.4931	12.5883	18.1126	20.2995	7.52957	15.9608	0.55205	-0.2564
ENSG0000	HMGB1	13:304567	560.453	584.347	706.147	711.526	1027.1	756.105	856.14	767.706	662.468	607.686	0.92805	-0.04291
ENSG0000	CLK3	15:745984	89.0245	114.752	102.892	98.1198	76.9012	72.1093	65.7373	76.8318	63.4331	75.4382	0.2056	-0.43426
ENSG0000	RP11-403	16:665525	2.30466	0.9078	0.923892	2.07366	1.58813	23.4354	7.91997	6.96968	4.8064	8.7927	0.05355	2.78715
ENSG0000	NCKAP1L	12:544977	25.6733	27.5323	9.45601	24.3707	10.9873	21.5011	17.1732	16.4597	20.6778	25.7506	0.67685	-0.17903
ENSG0000	UPK2	11:118883	0.098905	0.273879	0.271751	0.620848	0.560493	0	0	0	1.4334	0.640664	0.8553	0.352853
ENSG0000	IGHG3	14:105769	0	12.7787	0.104819	61.6264	6.7386	0.069478	0	0	0	108.959	0.61755	0.339858
ENSG0000	LACE1	6:1082948	1.67057	0.786363	1.03463	1.28998	2.31041	0.844693	2.20324	1.80071	2.81374	1.07486	0.7329	0.304775
ENSG0000	NOM1	7:1569497	6.0882	3.30285	3.40715	3.44134	5.1737	3.87052	3.07639	2.11977	2.82806	7.01748	0.6206	-0.24815

ENSG0000	RN7SL443	20:439148	0	1.89387	0	0	0	0	1.57559	0.425598	2.47034	0.471795	0.58045	1.75539
ENSG0000	NBR1	17:430442	49.7933	47.8048	19.0847	14.1905	14.9299	44.6625	20.9891	22.8974	9.31439	26.6746	0.4399	-0.28143
ENSG0000	RP11-356	11:112270	3.58038	2.22723	2.79331	6.98539	2.20835	4.44738	0.458622	1.18613	7.54882	2.45368	0.77315	-0.24017
ENSG0000	CTDSPL2	15:444272	16.2472	12.3009	14.7259	9.45887	20.7926	15.1398	6.67457	15.3178	9.23212	22.3876	0.6368	-0.27343
ENSG0000	GRAMD3	5:1263601	11.9544	11.9156	7.6618	7.82312	7.87952	6.67171	10.3035	11.9901	4.54533	7.74841	0.67715	-0.17445
ENSG0000	ZNF28	19:527974	12.9147	8.97821	6.04673	6.04621	2.72435	9.34764	13.4266	6.54737	3.18921	4.3435	0.5405	0.548524
ENSG0000	MAP4K5	14:504185	20.398	16.5746	16.8134	23.6935	20.5039	31.0271	19.7971	5.66243	12.6242	37.9926	0.97045	-0.02139
ENSG0000	LINC0054	13:299359	0.599001	1.78611	0.068754	0.662231	0	0	0	0	0	0.187997	0.1191	-3.83022
ENSG0000	AC005785	19:153533	1.47037	1.41419	0.152489	0.055778	0.097501	0.288359	0.551197	0.501682	0.173499	0.058309	0.5336	-0.92015
ENSG0000	MIGA2	9:1290366	27.3943	22.3234	8.43562	6.31903	7.27253	12.6357	15.3177	11.0778	11.8752	15.6686	0.93175	-0.03401
ENSG0000	RP11-587	12:918773	0.240101	0	0.234246	0	0	0	0	0	4.0736	0	0.0842	3.20708
ENSG0000	CDC23	5:1381397	18.2021	27.9201	9.03805	12.0823	6.80435	18.9573	12.8063	8.64872	9.96167	11.048	0.6387	-0.23815
ENSG0000	RP4-584D	7:1503637	0	0.288702	0	0	0.162497	0.497475	0	0.089838	1.28181	0.667344	0.26575	2.61696
ENSG0000	RP11-517	5:1263601	0.313744	0.482015	0.05698	0.313659	0.39159	0	0.058872	0	2.00691	0.436958	0.65755	0.704719
ENSG0000	STK19	6:3196980	15.5719	8.81896	15.9148	14.5024	13.0564	7.27364	18.8491	19.8539	11.7696	12.3379	0.91365	0.074722
ENSG0000	CLC	19:397312	0	0	1.41914	1.20954	5.44237	0	0	0	0	0	0.03095	#NAME?
ENSG0000	LRRK2	12:401860	0.428206	3.41254	0.048667	0.004882	0.042443	2.55102	0	0	0	0.045518	0.8305	-0.24655
ENSG0000	CEPT1	1:1111173	17.1159	26.8277	15.4017	12.7339	10.3528	34.5338	14.4256	18.154	10.294	17.787	0.57975	0.306775
ENSG0000	SLC35C2	20:463459	45.3036	56.0727	47.3321	43.7141	48.164	31.662	32.5893	29.4472	37.7965	33.0413	0.09655	-0.5566
ENSG0000	NT5DC3	12:103770	0.382945	0.645569	0.140969	0.246205	0.453254	1.38974	0.482574	0.561808	0	0.710124	0.3523	0.671136
ENSG0000	EEF1D	8:1435734	501.003	383.884	683.995	494.617	744.299	447.669	409.068	568.981	354.968	179.867	0.15975	-0.55469
ENSG0000	FGF14-AS	13:101452	0.531232	3.35336	1.80276	0.867255	1.30419	2.98271	0	0.187501	0	0.376738	0.60035	-1.24837
ENSG0000	LAPTM4B	8:9777505	7.44053	3.09285	22.3087	8.58744	33.5305	13.5809	11.0165	19.5634	23.1799	22.3235	0.98945	0.008839
ENSG0000	SLC2A13	12:396261	1.24138	1.3017	0.413811	0.54389	0.36553	0.217181	2.15502	1.1469	0	0.296194	0.8685	0.194742
ENSG0000	GOSR2	17:469231	43.0511	33.8312	33.7627	36.3185	27.4732	57.1434	29.9683	31.7659	16.027	25.3962	0.6686	-0.19998
ENSG0000	C1orf35	1:2281007	29.9868	30.0392	43.2751	26.5349	75.5274	61.842	44.3663	32.4815	30.7884	39.7632	0.76265	-0.12859
ENSG0000	ATF4	22:395196	96.975	77.1691	88.3062	143.305	68.1317	147.126	208.041	189.165	124.172	70.0309	0.0572	0.648008
ENSG0000	KHDC1	6:7320908	4.10265	3.46094	5.07067	2.93663	2.33499	0	3.16193	3.21937	0	3.22575	0.42795	-0.81911
ENSG0000	ZNF784	19:556207	0.214402	0.518766	0.321584	0.188977	1.08326	0.447036	0.459675	0.375528	0.065651	0.346737	0.77275	-0.58763
ENSG0000	RP11-996	12:291491	0.49823	0	0.640756	0	0.290344	0.869081	0.790137	0.379867	0.059234	0.765365	0.4921	0.963205
ENSG0000	ATP5B	12:566381	538.617	483.14	501.768	653.45	462.697	464.344	503.142	446.229	537.984	308.952	0.69075	-0.14027
ENSG0000	KRTCAP2	1:1551694	205.046	143.493	311.98	293.831	193.188	293.534	286.843	338.545	233.187	63.798	0.97995	0.008472
ENSG0000	EEF1A1P1	1:1941494	1.03335	1.5922	0.317394	0.110326	0.95209	2.4677	0	0.560743	0.079774	1.13532	0.97855	0.03226
ENSG0000	EEF2K	16:222062	1.2304	1.86135	0.669086	0.852657	0.894487	1.30751	1.85154	0.792245	0	1.95864	0.58145	-0.43742
ENSG0000	TRAC	14:222025	1385.44	1833.46	1972.28	1291.33	1711.54	2020.08	2107.3	1922.24	1583.53	1467.68	0.79895	0.12538
ENSG0000	AKT3	1:2432560	0.449207	2.08709	0.416485	0.028546	0.177597	0.071399	2.91485	1.02144	0.476527	2.44292	0.302	1.09103
ENSG0000	ZNF808	19:525276	6.71104	4.65067	3.46269	3.7116	2.65644	8.30642	5.36769	0.853007	1.18235	2.64146	0.7283	-0.25451
ENSG0000	C2CD3	11:740127	7.21654	5.5284	3.24684	4.15795	3.44965	5.32396	4.35732	2.80049	2.30973	6.22207	0.96545	0.030759
ENSG0000	PTPMT1	11:474659	39.4561	29.0051	53.179	48.1721	39.8986	59.1485	38.1523	28.4788	35.8396	9.94508	0.65265	-0.36791
ENSG0000	MTAP	9:2180254	10.7437	7.04069	14.5606	10.7559	9.31611	29.35	6.57862	8.19211	10.2295	12.4735	0.9424	0.041191
ENSG0000	ZNRD1	6:3000101	70.633	44.1692	82.4155	62.514	56.1391	125.383	92.9696	100.557	70.6231	39.8304	0.37235	0.37303
ENSG0000	PDXDC2P	16:699762	5.97319	12.9254	2.08388	5.8077	1.2355	4.90058	9.86363	3.61433	4.0517	2.54473	0.7521	-0.17377
ENSG0000	CHM	X:8586117	6.90645	6.01747	2.50021	2.81312	2.92607	6.79704	3.75414	1.0244	3.20004	2.75894	0.98025	0.015315
ENSG0000	PLXNC1	12:941487	12.6312	7.66373	2.73937	2.8008	4.24843	6.67333	2.59061	6.21177	7.49825	9.44791	0.7367	0.139796
ENSG0000	RAD51	15:406867	7.16451	6.34943	0.325131	3.35361	2.55502	11.3363	16.7793	6.81041	7.03678	4.5321	0.13505	1.19873
ENSG0000	DTX2P1-U	7:7695983	1.22346	2.26698	0.667441	0.851296	1.60809	1.36136	1.99626	1.18193	1.89966	2.28361	0.57245	0.53925
ENSG0000	NDST2	10:737855	27.9718	20.1643	16.6815	22.1787	9.64585	23.16	20.9363	18.1188	22.7048	20.2911	0.99365	-0.00551
ENSG0000	RAD54L	1:4624768	2.8148	3.37373	0	0	0.246627	5.53866	2.86944	0.019251	0	2.98372	0.3876	1.18394
ENSG0000	RP11-295	9:1327255	1.00939	0	1.79989	0.953546	1.73849	0	0.425529	0.117648	0	1.51224	0.5068	-1.60757
ENSG0000	GK5	3:1421575	3.81129	8.05725	4.15719	4.34491	2.75525	3.53341	2.75812	1.80884	1.48417	4.39299	0.24745	-0.84288
ENSG0000	GABBR1	6:2955551	1.59611	5.95519	0.366339	1.42074	0.687309	4.74812	0.267201	0.192029	0.322981	1.65745	0.45925	-0.52761
ENSG0000	HLA-H	6:2988729	89.1328	92.9088	176.249	86.9537	189.881	82.5865	144.544	212.191	90.0655	170.49	0.96725	-0.01636
ENSG0000	SLC25A46	5:1102892	24.9518	19.3242	9.58606	11.6745	6.80889	22.4059	14.9901	12.2029	12.4743	9.79287	0.9952	-0.00229
ENSG0000	VAMP4	1:1717001	21.8827	13.2958	14.9934	12.712	10.6759	51.8643	23.397	19.1984	14.9223	5.90523	0.40915	0.536823
ENSG0000	PMF1-BGI	1:1562129	5.71804	4.6888	11.2468	5.11558	11.8203	10.0192	10.6193	7.51572	3.92452	6.18363	0.93215	-0.11876
ENSG0000	FNTA	8:4284963	115.881	84.5881	104.991	100.766	71.4554	63.8165	83.3907	79.3181	60.6775	48.866	0.21085	-0.50382
ENSG0000	RP11-81K	12:750209	0	0	0	0	0	0	0	2.10061	0	0	0.1218	inf
ENSG0000	RHEBL1	12:490646	8.88911	2.85486	12.0163	21.4913	10.704	5.95708	5.00031	4.81185	6.45764	6.87869	0.08405	-1.04262
ENSG0000	PLEKHM1	17:454358	8.48852	8.42564	3.17661	3.28779	5.71323	7.56025	7.82799	3.65729	4.68896	8.26427	0.94465	0.027837
ENSG0000	C11orf74	11:365107	0.649694	4.1254	0	1.18094	0.308032	4.34523	0	0	0	0	0.6733	-0.57222
ENSG0000	RP3-370M	22:399010	5.64935	5.4583	5.22991	2.85166	2.31991	12.2745	5.52189	1.59351	1.71124	6.05318	0.8182	0.31555
ENSG0000	RP13-20L	17:824542	1.23098	0.400551	0.866248	0.215509	0.980933	0.90545	1.10258	0	0	0	0.25305	-2.04325
ENSG0000	SNX20	16:506662	58.0992	61.6221	59.6266	46.6664	47.5645	55.7132	47.5112	50.7419	56.8512	69.5103	0.76755	0.102939
ENSG0000	ENO1	1:8861001	557.569	553.12	625.239	721.784	876.065	716.742	655.104	689.328	508.861	521.326	0.7083	-0.14763
ENSG0000	IGLL5	22:228877	0	66.2499	3.08346	13.8313	4.10724	0	0.105769	0	10.3374	0.089616	0.20155	-2.39349
ENSG0000	NAA50	3:1137164	35.1624	48.7747	27.7605	19.1949	22.2825	47.5667	69.7402	49.348	20.3804	37.8228	0.463	0.286482
ENSG0000	UEVLD	11:185296	10.2085	1.63892	5.47371	6.56768	4.43571	10.0733	7.46874	6.20165	5.19405	9.60746	0.33175	0.43985
ENSG0000	AP000936	11:116835	1.68015	1.89105	8.68818	10.016	1.31383	0.738617	4.03473	5.08211	4.4078	1.81483	0.80885	-0.62509
ENSG0000	IRF9	14:241433	148.164	208.973	110.679	143.778	91.4209	223.417	318.338	167.767	105.873	134.806	0.5132	0.471407
ENSG0000	DISC1	1:2315286	1.2519	6.14013	0.581772	0.02852	0.141273	0	2.72487	0.830009	2.45077	0.361129	0.8587	-0.26089
ENSG0000	RUNX2	6:4480931	18.6893	6.15858	11.0511	13.5951	23.9556	10.2627	7.45475	12.5446	24.7818	38.3095	0.6887	0.186887
ENSG0000	BRD1	22:497732	19.5293	22.9556	7.01094	5.67755	5.77789	18.1903	15.4474	9.93865	6.87772	13.5963	0.6798	0.184429
ENSG0000	USP13	3:1796527	6.71634	2.23334	21.9832	21.979	17.2684	5.67811	4.15398	7.32486	10.2275	11.3799	0.07005	-0.9194
ENSG0000	RRM2B	8:1022045	8.00289	3.38701	1.7968	2.98607	1.56741	8.6154	4.10949	2.90932	4.08979	3.67501	0.314	0.537961
ENSG0000	POP7	7:1007060	30.4596	26.5872	55.7379	53.141	57.2778	46.5894	29.5344	51.0574	45.5232	21.8126	0.50055	-0.27525
ENSG0000	TYSND1	10:701379	21.2171	29.5227	14.5121	16.0031	16.3437	17.7741	16.461	16.6498	15.1214	13.8195	0.5008	-0.2533
ENSG0000	C8orf59	8:8517722	92.2656	85.9949	89.8995	76.992	36.5023	125.883	124.351	105.15	87.8534	18.7609	0.5052	0.305036
ENSG0000	FGFR1OP2	12:269383	97.6486	62.5955	43.342	60.7091	40.3039	82.4492	72.218	62.953	69.2587	44.7277	0.5897	0.192207
ENSG0000	MOSPD1	X:1348876	15.2331	14.2168	12.8338	13.4175	7.76039	12.6684	7.55848	11.3603	10.0521	4.42111	0.4219	-0.45077
ENSG0000	ARPC4-TT	3:9749943	2.70106	9.05598	1.7308	2.15967	1.18252	5.01469	5.38344	1.23556	2.03801	1.96429	0.86285	-0.17271
ENSG0000	RP11-616	15:442887	0.886583	0.053571	0.228306	0.546292	0.071981	1.4842	1.3629	0	0	1.03306	0.53835	1.06813
ENSG0000	TESK2	1:4532916	2.25042	1.02686	1.78783	0.866091	1.41175	1.93258	3.39817	2.4588	0.314846	2.07338	0.74595	0.459395
ENSG0000	LYPLA1	8:5404636	69.3852	55.4653	44.0987	55.1667	32.6359	57.8959	46.0081	45.2565	48.7953	36.3993	0.742	-0.09282
ENSG0000	C19orf60	19:185717	202.592	204.217	235.714	300.928	205.972	286.764	292.259	204.085	188.449	131.828	0.7645	-0.18758
ENSG0000	LUCAT1	5:9130302	0.50161	0.123849	0.204893	0.169256	0.063619	2.08456	0.360905	0.697023	0	0.188563	0.36525	1.65733
ENSG0000	FLYWCH1	16:291193	18.062	14.1722	7.85778	6.8067	10.3378	17.487	14.4605	7.36467	9.79472	14.4322	0.91205	0.059109
ENSG0000	STK4	20:449664	56.6443	86.9437	101.157	83.4096	106.621	71.6073	93.4563	85.1381	135.303	217.013	0.5626	0.310134
ENSG0000	ZFP41	8:1432468	3.46715	4.65666	0.612248	0.199405	1.11627	0.716953	1.27579	1.01543	1.67156	2.86632	0.94755	-0.07018
ENSG0000	RP11-488	1:2471872	12.0831	14.4537	16.3012	11.9231	9.08104	15.7617	17.842	25.8058	20.7889	31.034	0.0603	0.760668
ENSG0000	NUSAP1	15:413326	17.4684	54.3836	2.58552	4.43723	3.09207	44.3431	59.4012	20.2946	34.0573	24.1677	0.02015	1.40563
ENSG0000	ANXA1	9:7315175	563.268	274.124	1230.94	1091.45	790.07	332.542	616.339	923.042	1056.04	355.781	0.6258	-0.24109

ENSG0000	IGKC	2:8881118	0.751054	813.101	45.1774	5296.59	491.304	0	0	1.84429	10883.2	53.7026	0.67625	0.88562
ENSG0000	EIF3M	11:325837	145.87	129.5	182.645	122.339	143.376	166.912	186.108	195.194	186.691	103.266	0.92175	0.036832
ENSG0000	FARP2	2:2413562	2.9308	2.43334	0.894334	1.42737	0.668119	2.21758	1.81798	1.25479	0.664087	1.84047	0.6693	-0.28357
ENSG0000	C8G	9:1369452	0.915271	0.980093	0.568032	1.03465	0.224618	0	1.08884	0.539394	0	0	0.30725	-1.61036
ENSG0000	RNPS1P1	4:1137197	1.68386	1.0856	0.460089	0.856017	0.118551	1.33404	1.10345	0.064831	0.513172	0.540804	0.8978	-0.24987
ENSG0000	FBXO42	1:1624683	12.3934	15.4355	6.03598	9.01104	7.25279	6.696	8.33128	8.71879	6.15539	8.23388	0.25515	-0.51536
ENSG0000	CHID1	11:867859	26.5843	21.0553	49.9244	50.8978	44.5746	26.2925	27.6479	41.7145	31.9381	34.7897	0.41585	-0.29023
ENSG0000	NDUFC2-I	11:780157	1.05726	0.12465	2.03966	0	2.42105	0.444713	5.15471	8.71601	0	0.000974	0.75465	-0.39667
ENSG0000	SIN3B	19:168293	5.07728	3.78116	3.85272	5.81382	5.9973	7.33327	3.67585	5.04259	7.27101	5.3603	0.77895	0.138545
ENSG0000	FAM198B	4:1581244	1.2965	1.76303	0.015001	0	0	2.09325	0.008571	0.009109	0	0.009075	0.7401	-0.49303
ENSG0000	TRUB1	10:114938	7.94548	7.87073	5.59579	8.14457	3.86175	5.31247	6.73896	5.7019	4.57154	3.09621	0.5005	-0.3939
ENSG0000	AC012146	17:510554	18.7296	14.9833	21.6851	18.1968	9.41014	23.0389	15.2512	9.85662	25.8272	3.20099	0.9083	-0.12715
ENSG0000	UTS2	1:7784319	94.4282	6.63693	66.947	110.572	5.319	88.4358	11.1053	51.1513	89.702	2.18114	0.86295	-0.1336
ENSG0000	MARVELD	10:977131	0.775801	0.367815	0.132356	0.997061	0.348051	1.32332	0	0.653011	0	0.019125	0.73765	-0.48822
ENSG0000	ARMC2	6:1088484	4.40376	4.71459	2.14902	1.70948	2.3857	5.99445	5.02662	3.22487	3.27818	1.37653	0.8765	0.176907
ENSG0000	SRP9P1	10:917983	5.52462	2.98221	0.224393	0.289603	0	2.77581	2.63346	0	0.305048	0	0.73245	-0.6675
ENSG0000	SMPD4	2:1301513	24.7313	34.4624	8.78571	6.43655	10.7562	14.9892	17.8301	9.2889	3.57929	16.8009	0.7658	-0.30546
ENSG0000	GS1-124K	7:6651155	0.476007	1.00115	0.656771	0.077014	0.276706	0.35784	1.06337	0.127523	0.83562	0.889503	0.63915	0.362636
ENSG0000	MED18	1:2832900	6.47626	12.83	4.26892	2.12076	7.98422	12.4805	10.7814	9.25418	9.96896	12.7783	0.2255	0.694066
ENSG0000	UPF1	19:188319	21.8067	20.7266	3.49391	2.31578	7.06597	11.2033	13.5259	5.70982	7.92673	17.9637	0.6501	0.207587
ENSG0000	A2M-AS1	12:906517	7.96836	0.02904	1.66203	2.40087	3.36614	6.5279	0.043198	3.0765	0.132911	9.06441	0.84435	0.172975
ENSG0000	RP11-949.	3:4941677	8.47618	11.815	3.07223	2.18648	1.82635	6.77186	6.36413	2.03346	0.989635	3.09644	0.35155	-0.656
ENSG0000	BASP1	5:1706559	4.04211	4.92705	0.807798	2.99439	2.54802	30.1388	2.04716	6.20922	7.16109	2.58804	0.02555	1.6658
ENSG0000	RP4-548D	7:1512324	0.683717	0.358693	0.011686	1.06914	0.141155	0	0.069521	0.771097	0.962325	0.762193	0.87985	0.371206
ENSG0000	ADPRH	3:1195792	1.21066	2.05627	0.384839	0.0777	0.258713	9.00295	4.73717	1.99275	1.07694	2.47508	0.03275	2.34327
ENSG0000	C19orf24	19:127543	49.7727	47.2381	86.5144	81.908	109.988	54.6228	93.322	86.0185	85.4572	31.4895	0.567	-0.20472
ENSG0000	NUP62CL	X:1071234	0.702062	2.4867	0.276125	0.513743	0	0	1.32529	0.702392	0	0.164587	0.62365	-0.63833
ENSG0000	ZNF251	8:1447209	8.46575	9.71154	4.04405	3.18354	2.80755	9.89693	5.9849	2.19362	3.29341	5.98896	0.96595	0.037664
ENSG0000	USP48	1:2167829	35.1967	33.5755	24.8442	27.7831	21.31	18.1984	33.7039	23.0012	30.0984	28.6444	0.78045	-0.09491
ENSG0000	NTAN1P2	8:8633327	0.38218	0.264536	0	0	0.198874	1.98054	0.347451	0.085089	0.294833	0.092234	0.35375	1.73756
ENSG0000	NBPF8	1:1204363	3.73493	7.56012	1.57437	1.03784	3.395	4.7568	3.57373	2.36088	3.0716	10.1901	0.5249	0.377424
ENSG0000	ZCCHC3	20:296967	3.83744	4.02257	1.29898	3.16877	6.71273	5.33089	7.16253	4.83655	2.92838	8.14544	0.21985	0.634678
ENSG0000	SLC30A1	1:2115715	3.55451	2.10969	1.04774	1.03918	1.25086	2.67241	0.186486	0.80136	0.01631	1.79425	0.31905	-0.78225
ENSG0000	XRCC3	14:103553	4.32206	6.1362	1.28278	1.10675	2.91676	7.12135	6.34636	1.41673	0.230794	4.69603	0.9855	-0.0152
ENSG0000	CCAR1	10:687210	47.6612	54.4102	37.7138	27.7859	35.4483	68.6578	54.8705	32.9709	12.8794	61.099	0.90345	-0.04129
ENSG0000	ATP9B	18:790692	13.0272	19.747	7.05015	9.77481	8.60404	9.67342	11.9775	6.55966	6.50456	11.8796	0.74495	-0.14119
ENSG0000	LINC0089	X:1494769	3.52139	3.32645	0.859049	2.07169	1.21411	3.17901	5.23232	1.91002	3.05323	1.43408	0.4338	0.553532
ENSG0000	LINC0029	11:330394	1.02681	0.452832	0.619305	0.276231	0.887853	2.33287	1.30138	1.28913	0.554632	3.08554	0.4493	1.29945
ENSG0000	GTF2H2	5:7103266	11.3672	7.28851	9.5615	9.72748	6.28436	5.89163	7.57608	12.3073	12.7749	9.91991	0.59945	0.355604
ENSG0000	ASCC2	22:297886	58.7501	67.802	71.9932	75.9804	47.8056	64.1827	62.6878	38.9454	65.7054	69.2564	0.9317	-0.04015
ENSG0000	ATP23	12:579415	8.97083	10.3877	10.5892	13.3615	3.30288	7.49712	9.03268	7.81311	11.4571	1.85743	0.7414	-0.20172
ENSG0000	CYB5D2	17:414316	6.02944	3.88881	12.0558	15.4053	8.08654	2.44754	6.43242	8.47015	14.0745	5.73291	0.77	-0.29359
ENSG0000	WDR83OS	19:126432	105.045	90.8981	155.653	187.149	87.188	123.002	115.759	135.11	138.697	24.9478	0.78725	-0.2179
ENSG0000	RP3-428L	6:1609903	1.8566	0.260371	0.13799	0.177802	0.593791	0.492652	0.22316	0.249275	1.01684	0.306081	0.803	-0.34018
ENSG0000	ZFP62	5:1808476	10.7083	14.9164	6.98292	10.1605	5.87398	8.03492	10.4594	9.64233	9.83153	5.72254	0.9645	0.038954
ENSG0000	STAG3L1	7:7534401	12.3363	28.8175	15.5076	11.8882	7.57598	15.833	21.9665	14.2671	21.4327	24.0228	0.7082	0.358963
ENSG0000	SHOC2	10:110919	31.3431	25.5932	12.7923	21.0832	20.4262	16.5754	20.7064	16.2052	18.3106	14.7546	0.57035	-0.32701
ENSG0000	ZMAT1	X:1018822	3.05741	8.39542	2.93238	2.27853	4.59353	0	1.26588	1.52534	1.37418	11.0832	0.38185	-0.70381
ENSG0000	RPLP2	11:790474	277.455	399.463	560.839	378.695	327.317	293.51	363.003	396.739	243.809	42.2938	0.17805	-0.63273
ENSG0000	NTRK2	9:8466855	0	0	0	0.439486	0.079632	0.04024	0	0.605312	5.40281	1.31791	0.09295	3.96333
ENSG0000	TUBGCP3	13:112485	12.7836	18.6605	10.6112	10.2908	7.34456	9.83781	10.7588	4.17551	17.7151	10.0413	0.83505	-0.08892
ENSG0000	ZBTB45	19:585135	2.94959	2.92471	2.67277	1.34969	3.33399	1.56161	2.63083	1.92832	0.515881	5.1699	0.79455	-0.23736
ENSG0000	TRIM13	13:499825	16.2916	15.6157	20.1441	17.3352	15.3785	22.7681	24.849	38.4944	3.47125	26.8613	0.5944	0.347285
ENSG0000	TBL1X	X:9463294	4.86906	11.9298	2.4078	2.24952	4.43347	8.17223	4.97782	3.90655	4.17766	8.07222	0.99635	-0.00193
ENSG0000	RND1	12:488571	0.182491	0	0.143594	1.10783	0.321143	0	0.172241	0.517758	2.52923	0.485317	0.378	1.20736
ENSG0000	ANXA2R	5:4301441	49.8348	26.9356	105.132	97.7164	71.8338	39.8172	51.4942	101.605	82.4112	50.3673	0.81875	-0.13273
ENSG0000	BTG1P1	12:913508	19.3122	0.72536	11.9081	12.5209	9.19589	3.92419	0	2.40029	0	5.73462	0.18685	-2.1469
ENSG0000	U1	1:1464099	2.8187	0	2.06355	10.3569	0	0	0	0	0	0	0.0189	#NAME?
ENSG0000	SLC1A5	19:467195	21.2565	13.5358	10.0434	14.4162	13.4319	31.3917	45.9049	59.2426	26.882	34.8637	0.0074	1.49483
ENSG0000	ISYNA1	19:184188	12.1096	17.1602	11.2423	9.09058	15.3654	6.03993	13.0619	17.9498	7.62486	10.1454	0.5616	-0.38473
ENSG0000	PGPEP1	19:183405	3.89556	3.89582	3.55111	7.23231	4.36486	9.58723	6.80482	3.17248	8.85768	3.18875	0.68225	0.320279
ENSG0000	BLMH	17:302387	19.0991	22.1054	20.919	8.91887	17.1308	25.9144	16.9457	29.6289	20.1295	27.0034	0.15485	0.498256
ENSG0000	TMEM216	11:613916	18.2573	13.2702	35.4114	35.0532	17.021	24.9513	18.5703	22.9547	16.7179	10.0597	0.3294	-0.39315
ENSG0000	NAP1L4P1	1:1165145	0.310552	0.639356	0	0.137485	0.040252	0.399565	0.221791	1.59419	1.05612	1.2522	0.243	2.18314
ENSG0000	MILR1	17:644490	0.54153	1.47645	0.127671	0	0.748605	15.1148	0.05017	1.07089	0	0.878943	0.0761	2.50127
ENSG0000	DDX23	12:488297	62.7211	74.2801	39.6963	28.2673	32.4415	67.05	41.7132	47.9703	19.6053	55.9804	0.94775	0.01975
ENSG0000	ADAM8	10:133262	83.6343	43.7098	17.5725	25.2792	31.9837	59.1631	70.5451	42.8885	27.1041	59.2787	0.33375	0.308685
ENSG0000	PANK1	10:895794	0.151315	0.952714	0.086506	0	0.065969	0	0	1.43409	0	0.694629	0.53365	0.886185
ENSG0000	FASTKD2	2:2067377	19.567	17.491	11.6163	14.936	9.72815	19.9677	17.7522	12.9663	12.345	10.1239	0.85525	0.073123
ENSG0000	ISCU	12:108562	153.91	194.111	315.962	251.663	196.209	254.303	231.568	268.95	361.366	82.4367	0.7554	0.111401
ENSG0000	GAS6	13:113759	0.282342	5.16783	0.401546	1.35882	0	11.0833	1.22712	1.18717	8.31056	0.196376	0.16925	1.87266
ENSG0000	SOBP	6:1074899	0.060126	0.287171	0.048167	0	0.028825	0	1.33045	0	0.829764	0.279556	0.123	2.6942
ENSG0000	TRAV22	14:220705	7.34385	10.2443	8.82796	0.255543	0.584072	25.5473	11.7169	8.2522	2.13821	3.11935	0.35155	0.95619
ENSG0000	PDE12	3:5755627	6.56519	9.87772	5.14661	3.75639	4.11456	4.44954	6.24621	5.18906	2.10247	5.10634	0.24945	-0.6935
ENSG0000	LEF1	4:1080475	78.3129	587.415	43.5289	32.9673	25.8335	10.9777	14.2277	13.964	15.1465	20.7387	0.00005	-3.08517
ENSG0000	NPRL3	16:77006-	6.65589	7.06247	5.2376	3.67469	8.13017	4.16623	5.55126	4.14832	3.03192	9.05774	0.6886	-0.31482
ENSG0000	RP11-101	14:101948	1.87688	2.14227	4.8365	2.60125	1.84149	6.00006	0	2.38482	0	1.24736	0.62975	-0.56653
ENSG0000	SMIM10L1	12:108249	8.60393	9.66297	5.57129	7.6708	7.52328	8.89189	11.1477	5.45045	3.93102	2.53244	0.61685	-0.20685
ENSG0000	BBIP1	10:110869	26.6142	23.8883	27.8261	18.8437	6.36324	11.1865	26.6226	21.2535	9.2783	9.10514	0.6829	-0.28364
ENSG0000	TRBV21-1	7:1426369	8.45808	8.23538	5.73902	6.09976	4.69429	10.2872	4.334	8.95049	0	2.6977	0.4766	-0.195
ENSG0000	CIAPIN1	16:574281	38.2597	41.632	40.9709	52.5784	37.782	39.102	38.7117	27.977	45.8364	27.4326	0.5149	-0.33175
ENSG0000	ZNF547	19:573634	1.87485	2.06291	1.37982	0.97797	0.888346	3.14717	0.848676	2.48524	4.22551	2.1846	0.5271	0.811885
ENSG0000	RUNX1	21:347878	11.8385	19.1985	21.6089	15.9168	42.5408	5.70867	6.40707	16.7213	15.2356	51.9974	0.6737	-0.3294
ENSG0000	PARN	16:144357	37.8127	32.4895	27.5693	20.6697	13.8016	25.29	13.9053	24.0494	19.784	20.9736	0.3579	-0.30324
ENSG0000	XXbac-BP	6:3119519	7.29189	7.61663	19.21	16.1746	5.7854	3.7694	8.10308	10.2749	8.75863	13.6426	0.34355	-0.40657
ENSG0000	MGAT5	2:1341199	6.85012	5.75341	2.47278	3.9492	3.63703	11.8129	9.03139	3.27393	4.72711	18.1062	0.1134	0.966315
ENSG0000	FZD3	8:2849420	0.479685	0.637817	0.714574	0.681967	0.270568	0	0.602138	0.104365	2.02776	0.082677	0.92775	0.107961
ENSG0000	EIF2AK2	2:3708445	16.7724	10.0491	10.876	10.4539	12.5669	18.786	46.8504	28.2227	20.1432	23.8848	0.057	1.0363
ENSG0000	SLC26A11	17:801699	7.8537	9.19662	4.69154	3.40033	6.67786	9.38928	9.99925	8.28631	4.03413	10.5906	0.69805	0.363539
ENSG0000	C16orf91	16:141974	29.3036	17.9401	65.7601	62.628	47.775	42.1135	32.6096	49.6353	48.5109	15.5923	0.4727	-0.29977
ENSG0000	AXL	19:412192	0.105748	0.175085	0.842925	1.12783	0.098367	2.88253	0.222905	0.970735	1.30408	0.520884	0.1153	1.22188

ENSG0000	ROBO3	11:124865	2.85562	9.90649	4.31816	0.620537	3.79811	0	0.676565	1.76864	0	4.65123	0.08915	-1.56375
ENSG0000	AP003419	11:673178	26.3487	19.0231	4.03208	11.4565	3.8838	19.4581	5.80983	5.82225	5.21642	3.73636	0.8064	0.68945
ENSG0000	WBP2	17:758456	79.4173	101.499	63.4603	63.3976	80.5517	87.0534	66.5688	67.2727	84.4392	100.976	0.79665	0.079538
ENSG0000	RBM14	11:666165	47.6813	66.8017	31.2074	34.9935	35.4798	24.2991	30.3888	34.5002	16.2751	41.5404	0.40885	-0.54532
ENSG0000	TPI1	12:686711	410.008	371.057	535.164	480.645	554.09	854.427	685.766	707.419	537.089	306.324	0.41175	0.336784
ENSG0000	RCBTB2	13:484889	7.08904	13.2388	4.31974	6.10396	3.42597	7.57503	3.23136	1.37219	24.3643	3.1189	0.6196	0.237365
ENSG0000	INTS6	13:513357	19.4304	17.1229	4.69426	1.38452	5.95653	9.09522	12.6841	6.79927	3.38972	11.2077	0.73595	-0.16082
ENSG0000	RP11-325	5:1383158	0.794002	0.493778	0.426478	0.799091	0.473672	1.82932	0.618932	0.530064	0.014965	0.481347	0.62845	0.068362
ENSG0000	RP4-583P	20:636578	10.2332	20.1029	18.5813	16.7597	22.4945	12.3477	18.1349	15.8165	9.62749	10.8212	0.80265	-0.39839
ENSG0000	SSC4D	7:7638933	0	0.856568	0.07084	0.192071	0.016878	1.75546	0	0	0	0.672846	0.6163	1.14379
ENSG0000	USP20	9:1298272	50.0108	69.7887	15.5074	22.2091	17.3465	29.5661	46.2994	32.1787	30.5818	47.8549	0.67655	0.344462
ENSG0000	DLEU1_2	13:499825	1.57649	0.259437	1.75779	2.21568	3.96496	0.531339	2.88445	0	3.06207	0.160528	0.691	0.940218
ENSG0000	ZC3H13	13:459544	27.2888	30.5531	34.0275	17.1039	37.9041	28.9963	21.5353	18.9219	14.3456	48.4376	0.44035	-0.41363
ENSG0000	RP11-500	15:774204	6.28333	3.86943	2.7342	2.86248	2.2618	0.776504	1.4793	2.34759	0	3.85424	0.74935	0.596573
ENSG0000	AHDC1	1:2753403	0.673222	1.23391	0.301283	0.404657	0.462899	0.297574	0.373321	0.529518	0.042948	0.806891	0.3597	-0.99418
ENSG0000	ZNF841	19:520313	3.71041	3.21942	1.44211	1.30901	1.35082	2.92993	0.601908	0.931297	3.49163	1.22646	0.8189	-0.24382
ENSG0000	GZMB	14:245957	90.1819	162.622	329.529	451.202	171.741	1336.04	5137.09	1800.94	223.72	326.235	0.0529	2.64494
ENSG0000	GNG4	1:2355476	0.150451	0	1.13258	0	0	0.062616	8.8757	0	3.68265	0	0.2265	3.19067
ENSG0000	THOC7	3:6374182	96.3902	77.3031	79.7717	75.3088	62.138	107.877	71.122	76.4013	59.5061	20.4282	0.60195	-0.20148
ENSG0000	ERRFI1	1:8004403	0.160948	0.121883	0.229924	0.013243	0.180582	0.613664	0	0.882121	1.68344	0.039532	0.21875	1.83285
ENSG0000	BPTF	17:678255	16.3576	30.105	12.1058	7.20198	20.2453	26.8821	25.8972	15.7636	20.5639	45.4561	0.1791	0.49952
ENSG0000	RIDA	8:9810234	14.4455	10.5845	17.3656	22.0146	13.0294	16.1801	9.67073	11.8069	12.3584	3.47002	0.31625	-0.50349
ENSG0000	LINC0150	9:6854103	0.186798	1.83826	0	1.1329	0	0	0	0.143486	0	0	0.2723	-3.86408
ENSG0000	ERLIN1	10:100150	5.70685	11.8308	3.30872	2.99465	4.04776	6.55811	4.6663	3.73175	4.5293	7.88164	0.9317	-0.05439
ENSG0000	ICA1L	2:2027731	1.99679	1.47662	2.0058	1.69315	1.05043	0.016001	1.03208	0.089768	1.99531	1.55684	0.30155	-0.75988
ENSG0000	RPL13AP5	10:967502	13.301	6.79069	5.74685	3.30641	2.64484	10.2944	11.4696	2.41834	1.46992	0.654104	0.33165	-0.43607
ENSG0000	RP11-61J	1:2125578	1.24645	1.29511	0.62273	0	1.0589	0.543365	0	0.474211	0.130137	0.301039	0.5296	-1.47617
ENSG0000	LCP1	13:461259	894.12	576.936	340.051	293.72	268.994	572.338	369.387	387.997	279.389	451.104	0.7746	-0.15767
ENSG0000	FNBP4	11:477165	46.9125	72.2941	33.7432	33.3495	38.0546	40.4619	56.475	34.1579	42.3094	89.7096	0.6275	0.167858
ENSG0000	TOR1B	9:1298031	9.69876	7.82626	13.235	14.9532	10.3574	6.11815	6.59776	13.3975	12.8707	20.9699	0.8391	0.129874
ENSG0000	GLRX	5:9570124	201.526	100.981	213.079	238.581	165.084	160.684	123.536	91.7122	109.256	32.128	0.0133	-0.842
ENSG0000	CHST12	7:2403559	46.8427	27.6586	111.265	100.135	130.023	19.4391	97.3064	80.2447	53.9182	80.0658	0.21495	-0.48118
ENSG0000	SMPD2	6:1093902	11.0633	11.3598	7.93968	12.8917	10.1368	8.20803	7.83735	10.5661	15.9275	10.8008	0.8144	0.109976
ENSG0000	SEC62	3:1699393	130.871	129.561	132.354	116.222	217.647	223.094	225.728	117.623	170.695	117.039	0.7786	0.134741
ENSG0000	SESN2	1:2825952	6.97671	4.12433	22.306	20.2414	21.647	4.46494	4.14939	7.03499	10.7495	17.5619	0.04795	-0.82031
ENSG0000	CENPBD1	19:585591	12.157	11.2895	15.6741	11.3284	12.8804	12.4283	10.0249	11.5513	11.8587	8.63193	0.72855	-0.20521
ENSG0000	MPHOSPH	13:196336	31.1353	47.0446	41.5993	35.02	63.4325	51.6373	61.4579	39.2499	34.4486	74.3658	0.49985	0.217401
ENSG0000	EAF1	3:1538127	21.9486	5.0018	16.3212	23.3286	10.1853	3.93236	2.50064	11.7503	8.89091	12.8707	0.11275	-1.04168
ENSG0000	IST1	16:718459	99.0244	98.3861	120.025	125.787	111.641	99.0899	94.9622	103.135	103.177	142.063	0.78845	-0.09155
ENSG0000	HSF2	6:1223995	12.9894	19.422	10.9626	9.30027	8.59439	13.0369	7.84117	6.91846	7.64147	5.59792	0.1959	-0.59308
ENSG0000	KBTBD2	7:3286817	13.126	13.1746	10.4226	18.3166	5.50699	13.0684	14.6145	14.4345	6.07814	9.34798	0.4001	-0.29389
ENSG0000	UFSP2	4:1853960	26.4007	24.2865	28.1308	29.2215	19.8235	15.2322	17.2345	24.5107	34.1461	10.4844	0.45125	-0.32299
ENSG0000	GTF2IP20	1:2239513	1.82885	4.57199	1.62146	0.4973	0.905988	1.86143	0.913898	1.13549	2.44022	2.61057	0.9602	-0.03882
ENSG0000	SPEF2	5:3561784	6.1516	5.45899	3.04182	4.4487	6.31539	1.94493	6.74748	7.00178	6.38669	8.25926	0.72305	-0.23492
ENSG0000	AURKC	19:572308	0.262861	0	1.86372	0.511875	0.032098	0	0.327042	1.48319	0	0.646585	0.95875	-0.07027
ENSG0000	RP11-697	8:4726057	1.84676	2.1895	0	0.610335	0.186868	0.522354	1.97211	0	0	1.1439	0.89465	-0.24438
ENSG0000	RP11-505	16:838991	0.182222	0.113831	0	0.466053	0	1.67583	0.532264	0	1.31764	0.199184	0.6518	0.580848
ENSG0000	RP11-464	10:736448	1.59647	2.84657	1.44055	1.33445	0.733593	1.52889	0.728304	1.0384	1.27488	3.12384	0.70675	-0.45902
ENSG0000	ZNF282	7:1491954	1.16412	1.0852	1.17691	1.01291	2.08912	2.41968	6.14675	2.66147	0.669457	5.7837	0.06045	1.31179
ENSG0000	RPPH1	14:203430	0.756639	0.161079	3.7189	0.255151	0.446713	0	0.665699	0.609832	0	0	0.29145	-2.29714
ENSG0000	SLC12A7	5:1050375	5.83362	9.72466	2.68565	4.00241	6.16866	3.98808	1.36558	1.1562	3.54706	7.9199	0.17325	-0.60653
ENSG0000	MRPS12	19:389152	69.4149	50.423	92.4858	122.056	79.2142	63.0211	81.2923	73.6319	57.0139	17.7714	0.1904	-0.60475
ENSG0000	PMS2P6	7:7309365	4.23832	5.00479	5.86593	9.86228	4.85195	6.09944	7.51752	6.47551	7.24899	4.92442	0.8088	0.511226
ENSG0000	SLC9A8	20:498127	4.82776	6.88817	5.755	5.46236	6.31796	3.56429	6.66806	6.91439	10.7187	16.9786	0.268	0.493754
ENSG0000	ZNF207	17:322661	111.043	121.274	76.8571	83.2289	77.1399	75.135	95.1202	92.3014	86.3585	105.428	0.88855	-0.04978
ENSG0000	DDB1	11:612994	55.9757	54.3349	22.2437	29.8508	22.6186	40.7495	25.1585	25.0032	23.7021	45.8171	0.8576	0.090744
ENSG0000	APOBEC2	6:4102691	0.748901	1.44941	0.283781	0	0.141684	1.20205	2.1655	0.213291	0.766146	0.875133	0.4857	1.06543
ENSG0000	ZBTB46	20:637436	0.384388	0.369512	0.151509	0.089507	0.096211	0.030617	0.442392	0.361653	0.654623	0.344138	0.3099	1.21399
ENSG0000	LTBP4	19:405928	17.1533	28.7349	10.29	10.6042	14.9107	21.3488	18.3912	11.3781	17.6424	19.9466	0.41495	0.310668
ENSG0000	MS4A4A	11:602805	1.39617	1.33661	0	0	0.176269	53.6364	2.57281	3.29885	0.172766	0.531182	0.0168	4.46362
ENSG0000	NOC2L	1:923927-	49.0743	54.1461	29.1276	41.9159	28.738	51.6942	54.2993	32.1847	16.1772	37.3442	0.9169	-0.04901
ENSG0000	PARS2	1:5475689	1.79025	2.91967	6.51957	2.04895	4.6477	1.54099	1.7023	3.73136	0	3.68979	0.2239	-0.85852
ENSG0000	HACD2	3:1234908	9.2528	14.4059	1.98634	1.20891	3.75768	7.34242	5.45624	2.55031	0.78221	7.5179	0.3117	-0.43105
ENSG0000	Metazoa_	7:1520251	3.514	5.46895	0.923904	5.57433	0.436239	0	0	0.246982	1.37678	0	0.1692	-2.897
ENSG0000	MRPL4	19:102519	31.5712	28.6145	46.0673	37.5086	47.0692	30.3844	43.3861	44.0375	19.1947	34.8369	0.7122	-0.20987
ENSG0000	PVRIG	7:1001775	182.806	94.3508	472.372	356.014	589.713	151.012	120.093	253.2	188.543	283.845	0.03405	-0.80863
ENSG0000	MTND2P2	1:585988-	47.2317	27.5859	77.0322	88.0595	116.681	70.355	29.4029	96.9548	84.0297	61.0406	0.99075	-0.01932
ENSG0000	ZBTB8OS	1:3246505	34.6593	33.2639	62.5035	56.5493	33.0052	49.1293	64.7525	62.3713	53.3569	17.1289	0.6746	0.177004
ENSG0000	ODC1	2:1043996	33.2276	36.4087	35.1979	55.7597	27.5049	60.1886	60.0616	50.6071	41.6851	38.0772	0.25945	0.360033
ENSG0000	PARD6A	16:676609	11.0058	7.97788	10.374	8.81681	16.4105	31.971	13.002	7.48174	19.1485	3.25035	0.5255	0.363113
ENSG0000	CTD-2062	5:1395264	0.665616	1.98376	0.118948	0	0	0	1.8177	0	0.340705	0.132261	0.964	-0.05358
ENSG0000	DGKZ	11:463329	46.0436	53.4801	16.1736	17.5204	38.5708	19.5693	27.8361	22.6108	12.7506	41.4907	0.1903	-0.53155
ENSG0000	CABLES1	18:231345	0	0	0	0	0.035453	1.88947	1.89627	0.197635	0.112289	0.072549	0.00005	6.7095
ENSG0000	SLC16A6	17:682591	1.66862	1.53176	0.999738	0.11305	0.107393	0.731433	1.71216	1.7284	0.160538	3.21794	0.5577	0.691327
ENSG0000	PLEKHO1	1:1501491	33.1843	40.8545	34.9424	26.7945	33.1515	96.715	65.9003	40.9859	36.4065	49.4446	0.0359	0.722751
ENSG0000	EPS8L1	19:550720	2.04266	0	2.91638	4.29816	2.54034	1.28672	1.74896	5.27361	5.10737	4.59393	0.3405	0.651313
ENSG0000	PSIP1	9:1542270	60.539	107.209	44.3009	37.2357	104.389	57.8591	91.1281	38.5041	27.8529	73.2083	0.37305	-0.39246
ENSG0000	NRIP1	21:148188	15.1125	4.91252	6.92757	6.85359	10.0239	7.46192	1.03618	2.96628	17.097	12.1207	0.7225	-0.15847
ENSG0000	SUPT7L	2:2758296	22.5306	21.6248	28.6545	21.2379	21.1836	36.1357	21.2515	21.8033	13.6659	31.8699	0.94585	-0.06093
ENSG0000	TRMT10C	3:1015618	30.1535	29.6139	35.0015	37.2681	30.9999	25.7253	30.8557	33.9061	27.474	17.1765	0.46645	-0.28137
ENSG0000	U1	1:1203549	2.46711	0	1.90152	15.5974	0	0	0	3.92909	4.6797	0	0.7379	-0.52507
ENSG0000	WWC3	X:1001556	6.682	2.92665	11.1651	5.2507	7.03277	4.38772	3.65192	8.82215	3.71905	12.8557	0.7752	-0.12576
ENSG0000	CECR5	22:171375	33.2051	38.3843	44.5763	48.0551	40.3484	34.1068	29.123	18.9697	27.8564	21.179	0.08015	-0.63437
ENSG0000	ADPGK	15:727513	60.9736	49.6524	63.698	67.46	43.2465	64.3461	51.4936	42.8744	66.9869	61.6191	0.7958	0.086466
ENSG0000	RP11-307	9:9433247	0.022267	0.447647	0.072036	0.183122	0.075593	2.04078	0.640615	0	0	0.116546	0.38415	1.70035
ENSG0000	RAI1	17:176814	3.10204	1.95528	1.43364	1.30203	1.46918	1.17924	1.74649	1.57178	0.027902	4.02337	0.95975	0.043152
ENSG0000	ZNF227	19:442075	14.1645	12.1243	6.27361	3.06821	3.66643	8.26186	9.42552	5.5329	8.62165	8.70762	0.7996	0.117508
ENSG0000	CAPN3	15:422084	2.35286	3.43437	1.59425	2.2266	0.522581	0.816444	0.995101	2.55913	0	2.50658	0.78495	-0.23718
ENSG0000	AGGF1	5:7702925	16.0636	19.7547	12.8713	8.28591	16.1305	13.5079	14.9789	9.71426	7.81016	33.6377	0.6684	-0.25979
ENSG0000	NKPD1	19:450714	0.239541	0.46115	0.144456	0.920576	0.084513	0	0	0	0	0	0.11255	#NAME?
ENSG0000	AC016722	2:4689927	0.271826	1.02543	0.959783	0.85528	0.595965	0.365046	0.273952	0	5.10523	0.6637	0.5334	0.894395

ENSG0000(	TTF1	9:1323755	10.0466	16.0766	10.3348	9.91428	14.2635	22.0269	17.1568	8.94974	14.8141	17.3233	0.3851	0.319904
ENSG0000(	HECTD2	10:914092	1.69727	1.03511	0.571093	1.29341	0.252585	3.13431	0.802508	1.0647	1.32484	0.953122	0.49925	0.687912
ENSG0000(	HIST1H2A	6:2603317	2.01234	0.714598	0.780434	0.752603	0.14396	0	1.57701	0	0	0.202233	0.36845	-1.46255
ENSG0000(	LINC0088	3:1944026	0.575221	1.12885	0.157304	0.269839	0.527841	0.871051	1.38114	0.264425	0.222085	0.355618	0.9627	-0.05902
ENSG0000(	RP11-371	7:1327848	5.58018	5.90694	6.8174	7.04142	4.33095	3.28933	2.97789	2.69422	4.51945	0.836544	0.4847	-0.98246
ENSG0000(	RP1-30M3	6:2466703	0.754769	1.10011	0.511485	0.400551	1.29329	0	0.993927	0.053505	0	1.78146	0.6885	-0.60406
ENSG0000(	IGKV2-30	2:8924478	0	49.1816	3.31642	0	222.27	0	0	0	0	1.20814	0.016	-7.39929
ENSG0000(	PLBD1	12:145036	8.06521	16.6257	0.494754	0.852794	0.244014	19.2947	0.218322	0	0.095317	0.416285	0.56365	-0.39681
ENSG0000(	RPS26P47	13:100089	3.36117	2.7663	1.59685	3.86647	0.84911	3.67916	4.40776	0.577567	4.03312	0.470708	0.98565	-0.02737
ENSG0000(	SUZ12P1	17:307092	21.4158	10.1863	3.76065	3.92138	3.40981	15.4886	16.8424	5.9715	4.14293	6.84412	0.726	0.309115
ENSG0000(	GAPDH	12:653392	1141.92	1188.03	1018.8	863.439	1486.44	2209.13	1912.12	1613.56	1471.72	823.855	0.36145	0.498092
ENSG0000(	NIPAL3	1:2430755	23.7392	51.7514	21.6006	24.2577	15.1954	23.1376	28.495	13.4597	31.7537	26.8212	0.7494	-0.17922
ENSG0000(	MAN1A1	6:1191772	49.2712	13.2704	9.72574	12.6384	17.8854	21.4723	24.6187	12.1256	16.3803	29.408	0.7708	0.100491
ENSG0000(	HACD1	10:175890	3.86768	9.04509	0	0.184494	0.185196	9.47285	12.9806	0	0	0	0.48095	1.02049
ENSG0000(	GPR89B	1:1479283	25.4057	23.4481	22.4196	19.9232	16.7096	23.3032	24.0295	12.6192	18.5211	18.2962	0.8419	-0.1006
ENSG0000(	ZFP3	17:507824	0.985147	1.10652	0.194357	0.179091	0.319903	2.27063	1.10417	1.5167	1.20634	1.51005	0.03065	1.51538
ENSG0000(	ZNF48	16:303781	3.05825	1.56892	1.50844	0.989612	1.99729	0	1.09168	3.42589	0.171195	2.78847	0.82	-0.26735
ENSG0000(	DENND4A	15:656113	10.6707	11.1054	3.95944	5.04916	4.34298	7.80259	9.04819	9.34117	9.97797	12.0178	0.3633	0.46383
ENSG0000(	GYG1	3:1489913	54.5668	16.8208	95.2277	64.7011	86.8387	43.41	51.9265	105.554	52.4857	56.581	0.75155	-0.11143
ENSG0000(	SLC48A1	12:477060	3.18393	2.4064	2.65351	2.98907	2.86589	2.84276	3.75064	3.45902	6.49158	3.66262	0.6171	0.688769
ENSG0000(	RP11-429	10:957117	1.87979	0.620385	0.917024	0.735624	0.438116	0.508546	0.064545	0.92009	0	0.245994	0.5235	-1.19965
ENSG0000(	AZIN2	1:3308110	1.0644	0.498879	3.41398	2.52176	2.61338	0	11.6657	7.01454	0	1.90395	0.28715	0.841608
ENSG0000(	SF3B4	1:1499233	59.443	66.5637	44.1137	32.6408	55.4927	51.1753	79.1132	48.447	35.5301	69.3217	0.78605	0.093076
ENSG0000(	UBAC2	13:992007	119.976	100.612	112.495	126.319	131.081	80.5802	104.196	101.158	81.7869	121.076	0.70175	-0.31369
ENSG0000(	C9orf64	9:8393831	5.11378	4.98403	5.24537	4.13909	3.37544	7.79279	5.46995	1.4737	5.52968	5.72073	0.87515	0.099816
ENSG0000(	GPR183	13:992007	129.601	195.036	192.543	257.155	110.602	126.717	258.131	175.587	267.736	91.0733	0.929	0.045987
ENSG0000(	GPX1	3:4935717	142.21	129.227	171.321	208.146	144.013	606.664	177.733	196.295	261.338	37.6605	0.1209	0.555913
ENSG0000(	RP4-738P	20:251956	3.43231	3.10175	1.35964	2.42016	0	0.268267	0	0	0	0	0.3006	-2.45877
ENSG0000(	TAF9B	X:7812974	14.3935	16.0276	6.25718	5.39965	3.76542	6.83308	9.82412	6.48621	7.59177	6.06666	0.67105	-0.17307
ENSG0000(	GALK1	17:757213	26.2796	14.8229	30.6678	40.134	29.1595	23.3183	24.8042	27.7495	33.1999	21.9993	0.9489	-0.02786
ENSG0000(	CEP19	3:1966396	6.6278	9.6167	5.301	5.99123	8.71286	8.17604	2.45888	5.09093	2.72257	9.51108	0.61595	-0.45613
ENSG0000(	IGF1R	15:986489	0.514927	1.75434	0.535129	0.238106	0.691889	0.465732	0.01715	0	0.320694	1.13281	0.07245	-1.3337
ENSG0000(	ARL13B	3:9398013	5.28796	4.22083	5.47894	7.27832	7.27916	6.96053	7.51141	6.06341	7.76871	10.8493	0.7593	0.2126
ENSG0000(	CPT1B	22:505688	2.68746	8.43252	0.685478	0.848173	1.97014	5.55105	18.5807	1.73306	4.66215	7.62468	0.1384	1.35611
ENSG0000(	POP4	19:296040	103.965	72.073	108.596	84.9931	74.0175	113.093	108.699	85.0397	91.3224	37.2664	0.90105	-0.05435
ENSG0000(	RP11-446	12:463836	2.0455	0.483282	1.75098	0.763918	0.753793	4.45158	0.403759	0.218715	3.71545	0	0.78485	0.61394
ENSG0000(	PEX5	12:716667	5.70042	3.23005	0.676818	5.67295	2.17111	2.8483	1.44469	1.91774	5.99203	3.17238	0.9117	-0.05846
ENSG0000(	PTPN18	2:1303560	42.7062	39.0204	24.7272	19.3744	44.8689	43.0445	32.7925	37.5152	19.1214	45.461	0.9277	0.032271
ENSG0000(	BCKDK	16:311061	18.0645	25.4357	32.637	25.6918	31.1231	29.626	23.3791	28.6878	35.8194	28.6848	0.75845	0.117773
ENSG0000(	CYP2D6	22:420855	0.379367	1.57086	0.219016	0.390956	0.030581	0.17894	0.642091	0.083986	0.446779	2.09863	0.7587	0.532348
ENSG0000(	FAM229B	6:1120875	0.515815	1.3752	1.36577	1.45308	0.6998	1.80997	1.22296	0.673486	0	0.816545	0.6618	-0.70086
ENSG0000(	AP000254	21:316596	1.82842	2.99025	2.86955	1.92787	0.891914	2.69968	0.35138	3.99485	0.124317	4.12528	0.8208	0.333757
ENSG0000(	UBC	12:124911	874.656	572.422	841.92	1058.75	589.206	637.464	653.31	728.223	845.72	408.459	0.5723	-0.25854
ENSG0000(	NRBF2	10:631332	39.7209	39.5795	36.9201	35.9486	31.562	66.2967	34.7372	34.3903	30.37	14.9164	0.961	-0.01701
ENSG0000(	BTN2A3P	6:2642139	0.543482	1.1692	0.883778	1.49318	1.21651	1.8886	0.573957	0.94677	0	2.21858	0.9327	-0.08895
ENSG0000(	SH3RF3	2:1091293	0.389541	1.55326	0	0.306367	0.156186	0	0.286929	0	0	0.021671	0.3727	-3.03271
ENSG0000(	KLF10	8:1026487	14.835	34.9022	11.1583	12.377	8.77064	23.3343	25.1581	33.048	21.9809	27.8303	0.02305	0.790463
ENSG0000(	MIR3142H	5:1604682	0.073479	1.94199	0.0253	0	0.054161	1.22917	6.41587	0.53973	2.46099	1.18704	0.078	2.66634
ENSG0000(	ERP27	12:149140	3.87628	8.46452	5.55398	3.70006	2.1861	1.5036	7.80296	3.53529	4.41819	5.02366	0.97145	-0.02047
ENSG0000(	MAIP1	2:1999112	13.3738	9.13821	14.0465	11.6736	8.14779	8.9341	17.8773	12.323	12.8425	2.78631	0.99155	-0.00967
ENSG0000(	FUBP3	9:1305789	24.5423	25.1544	8.95836	9.53045	6.95773	27.4668	14.2693	11.1841	10.5025	12.3675	0.9936	0.003048
ENSG0000(	TMEM150	19:553128	0.376842	1.88967	0	0	0	2.54353	0.846023	0	0	0	0.6617	0.74378
ENSG0000(	TCEA1	8:5396655	100.883	94.6969	41.1603	52.7866	43.4559	92.494	88.1144	41.6743	57.5131	34.2811	0.96325	0.014627
ENSG0000(	SLC2A6	9:1334710	2.88087	5.22511	1.49972	1.89219	3.01045	7.78853	6.50534	5.29919	1.01227	3.23514	0.36335	0.683254
ENSG0000(	PTPRM	18:756678	6.6832	3.17011	3.77801	2.73436	7.71573	4.66329	0.592873	2.37837	4.72923	8.93312	0.88435	-0.08283
ENSG0000(	ATP6V0E2	7:1498676	0.932383	6.72689	0.812907	0	0.143913	3.11576	0.667797	1.47369	0	1.33473	0.8708	-0.26541
ENSG0000(	SUSD6	14:696115	6.14847	5.27307	14.0274	12.901	19.2875	8.50272	7.3151	27.7682	19.5889	47.2736	0.0873	0.698938
ENSG0000(	IFT20	17:283190	76.9534	49.6698	76.2615	76.7382	49.8269	105.226	77.9648	59.4757	76.8649	30.1398	0.87455	0.072743
ENSG0000(	PAXIP1-A	7:1549284	7.25302	6.78404	6.54377	4.64719	6.77457	6.23938	5.25539	3.83877	0.497079	7.79072	0.5524	-0.59802
ENSG0000(	RP11-380	12:123762	0	0	0	0	0.216136	0	0.067632	0	2.83468	0.597599	0.34205	3.9716
ENSG0000(	PFN1P2	1:1204322	0.407827	0.510382	0.412497	0	0.008642	1.9567	2.11427	0.067364	0	0.857414	0.2997	1.96196
ENSG0000(	DTX3	12:576046	12.4288	4.87868	4.04256	2.06491	6.70475	4.10802	6.49381	3.27027	1.00798	8.72475	0.5568	-0.3527
ENSG0000(	RP11-350	8:3838236	11.2305	4.86523	14.22	5.60856	3.80976	0.522625	3.45685	3.92938	15.0402	1.30532	0.4857	-0.53493
ENSG0000(	P4HA1	10:730072	18.6689	8.99453	7.12367	11.2711	6.11476	27.0918	6.69801	10.761	11.5141	13.7213	0.37045	0.356026
ENSG0000(	SSR1	6:7268305	93.8679	82.5316	43.2462	35.4968	41.7475	88.2892	83.9911	49.7567	61.5039	54.7751	0.90315	0.048877
ENSG0000(	LINC0125	4:3842066	0.701013	0.672037	7.15164	3.79187	0.439727	0	0	0	0	0.075875	0.2653	-7.2924
ENSG0000(	CENPX	17:820187	29.7849	33.1284	87.0123	74.3303	40.3159	42.0723	56.8719	91.0073	31.7022	33.2372	0.84255	-0.10033
ENSG0000(	RBCK1	20:407497	94.3957	91.4907	107.093	85.7189	113.388	128.477	175.206	150.038	47.5903	107.969	0.57785	0.218742
ENSG0000(	NUP43	6:1497243	1.7999	4.86945	2.33819	0.789256	1.13547	3.46179	3.68505	4.21508	3.25799	3.83566	0.4115	0.800829
ENSG0000(	THAP9	4:8281866	2.07648	1.49043	0.542329	1.04126	1.06847	1.90953	1.34854	1.27176	0	0.450235	0.70925	-0.38353
ENSG0000(	RP3-476K	1:2643228	0	0	0	0.229651	0	1.12253	0.817407	0	0	0.427717	0.2477	3.28327
ENSG0000(	IFIT2	10:892135	7.56743	10.6119	7.32043	5.2572	6.54116	6.67981	35.4885	34.1944	4.60823	17.181	0.29555	1.3574
ENSG0000(	IKBKE	1:2064704	28.123	48.1594	23.1553	26.1577	12.2094	31.556	25.2698	13.0394	21.4398	17.8073	0.4955	-0.32301
ENSG0000(	ATP5G2P3	2:1969670	1.84427	2.82278	0	0	0.230728	0	0.556086	0.621945	0	0.294802	0.4714	-1.38992
ENSG0000(	FAS	10:888797	58.896	44.7347	24.9416	41.9104	13.1058	71.8589	74.8362	53.0332	25.4683	24.7969	0.39495	0.340604
ENSG0000(	DUSP3	17:437661	2.87867	3.91708	2.32971	2.27638	2.85959	14.5747	3.62797	2.37419	4.75614	4.77078	0.0734	0.977807
ENSG0000(	ADAM22	7:8793414	0.30173	0.453478	0.43852	0.39447	0.536294	0	0.073128	0.247417	0.946909	0.393604	0.7872	-0.2948
ENSG0000(	TMEM40	3:1273352	0	0	0.926254	4.29797	0.245646	0	0	0	0	0	0.0057	#NAME?
ENSG0000(	RGPD1	2:8681533	3.34876	5.33404	0.356349	0.62902	0.221798	2.32242	8.31405	2.80374	2.96187	1.05401	0.51915	0.922572
ENSG0000(	CTD-2349	17:307092	0.610448	0.400342	0.173946	0.233354	0.090486	0.212107	0.661309	0.375376	0	0.298702	0.83625	0.288125
ENSG0000(	GPR108	19:667770	99.1833	61.9112	108.7	76.6586	107.66	137.769	95.9524	84.931	65.7698	106.761	0.8125	0.067863
ENSG0000(	AAED1	9:9663957	4.68511	3.25743	6.97787	7.32642	6.84577	6.02347	9.0098	13.854	9.30102	5.08973	0.3835	0.395031
ENSG0000(	CMTM8	3:3223867	2.5194	4.89867	1.69276	1.32413	2.02113	2.24769	2.94704	4.24858	0	3.73856	0.9308	0.118247
ENSG0000(	CFD	19:859642	6.27747	17.9944	10.3278	5.40024	11.1659	28.0991	4.17157	6.01615	22.1591	6.01753	0.54435	0.417379
ENSG0000(	PUS7	7:1054396	4.85948	6.27509	2.20243	2.84419	1.59318	0.394891	4.90842	0.351484	4.57292	1.83596	0.34775	-0.61699
ENSG0000(	ALDOC	17:284557	18.8087	21.1992	31.0693	23.8528	33.0989	42.2815	15.9389	30.1079	64.8484	28.2513	0.61125	0.54879
ENSG0000(	RP1-257I2	22:420855	1.22469	0.686312	0.965095	0.69804	0.637693	1.09039	0.4141	0.909683	0	1.13723	0.98355	-0.02673
ENSG0000(	SIGLEC14	19:516119	0.312917	1.62976	0.038409	0	0.010762	3.14281	0	0.514428	0	0	0.5977	1.02684
ENSG0000(	HEMK1	3:5055802	9.12047	9.72437	19.3521	22.4509	7.55661	4.88644	8.22851	14.5298	8.94081	6.25844	0.35	-0.70243
ENSG0000(	RP11-53B	18:132174	0.431157	1.62871	0	0	0	2.98621	1.19874	0	0	0.132445	0.6487	1.11763
ENSG0000(	PLEKHG5	1:6424787	1.29266	1.07661	0.2901	0.764182	1.07243	0.021508	0.465638	0.501997	0	1.25912	0.3449	-1.14601

ENSG0000(	PLCB1	20:807725	17.1025	5.7664	7.38738	11.4608	8.98953	0	0.017385	5.9731	14.1269	10.0427	0.4122	-0.51392
ENSG0000(	GPAM	10:112149	1.33646	0.913041	0.579428	0.43301	0.573215	0.826805	0.220115	1.00939	0	1.26397	0.77975	-0.28949
ENSG0000(	FTH1P7	13:226960	1.07455	1.20719	0.823159	1.73344	0.564966	4.61202	1.64292	0.327672	2.05416	0.457962	0.7648	0.619573
ENSG0000(	CTNNAL1	9:1089341	3.87591	3.09197	2.07216	1.60747	0.867256	4.90064	5.61747	4.43007	4.27943	3.71671	0.20925	1.40673
ENSG0000(	RP11-110(	12:520692	11.1594	4.47746	7.78353	7.60155	12.0411	9.80497	9.38898	8.66638	7.74693	7.57497	0.8944	0.126583
ENSG0000(	TWSG1	18:933476	2.56925	1.45804	1.49464	2.04072	1.29697	3.47343	3.72077	4.014	1.89203	1.39867	0.18485	0.684189
ENSG0000(	KPNA6	1:3210803	5.27199	6.16998	8.39646	4.36989	7.23664	6.24699	4.80386	9.68268	6.24342	19.4723	0.50105	0.253881
ENSG0000(	SUPV3L1	10:691802	22.5089	22.3315	14.4201	17.0469	9.41634	12.7379	13.478	9.46324	33.7284	9.10199	0.90575	0.0461
ENSG0000(	ATP5D	19:124174	126.347	119.501	191.848	145.467	208.952	151.989	183.288	168.317	120.197	78.1745	0.38665	-0.32237
ENSG0000(	ZNF48	16:303781	1.57922	1.21798	0.949191	0.791057	3.15792	0	1.24874	3.41763	0	2.52959	0.7567	-0.47003
ENSG0000(	RP3-395M	1:2549919	10.944	6.98383	7.804	7.22891	7.443	13.1351	10.3705	12.3249	8.49045	13.4265	0.4805	0.491693
ENSG0000(	MRPL35P3	10:741511	1.13549	0.371371	0.437489	0	0	0	1.44191	0.137819	0	0.863502	0.77795	0.566714
ENSG0000(	RP11-195I	9:3463813	1.28897	0.486512	1.16932	0.069215	0.843613	3.26289	5.33704	1.10642	0	0	0.3598	1.16319
ENSG0000(	ZKSCAN3	6:2832469	1.11622	3.47399	0.973397	1.09093	1.01372	0.00837	0.086945	1.27253	0	2.01494	0.2318	-1.34221
ENSG0000(	VPS72	1:1511566	41.9422	41.3974	59.8671	41.1836	49.6968	33.2287	51.5121	41.2967	39.3411	20.9637	0.4834	-0.37614
ENSG0000(	PPP2R1A	19:521900	59.2007	56.8879	75.786	62.8149	76.9814	63.0894	112.4	83.7971	56.4434	100.746	0.68545	-0.13807
ENSG0000(	FLCN	17:170425	2.71851	7.25865	1.52261	4.10362	1.98243	4.50415	5.6059	2.36281	4.10397	4.47255	0.7716	0.221835
ENSG0000(	ACSL1	4:1847555	11.8483	12.6628	3.15059	5.88102	1.75323	26.0129	9.7147	11.2371	14.9264	6.58028	0.0041	1.33386
ENSG0000(	FOXO3B	17:186350	2.24749	3.24138	2.3325	1.45685	3.31503	0.518482	1.18911	1.46876	0.555906	4.73204	0.7811	-0.51366
ENSG0000(	CRLS1	20:600608	26.8664	18.4675	26.4754	16.8938	18.7839	25.4017	34.0304	26.0862	29.3534	9.97419	0.6678	0.21852
ENSG0000(	NDUFA4	7:1093195	105.992	75.4482	123.054	123.823	41.1936	151.308	122.026	108.634	101.132	17.0897	0.69675	0.129377
ENSG0000(	ZNF350	19:519491	13.9555	20.7922	16.9986	20.8931	10.5934	20.5363	16.4179	11.9506	22.9487	12.1217	0.9374	-0.02954
ENSG0000(	C12orf43	12:120941	15.6404	11.2916	24.139	14.5457	19.8334	18.0845	9.29038	5.4112	4.5143	12.1454	0.0393	-1.02671
ENSG0000(	ASS1P1	6:2479732	2.41143	5.14987	0.535995	0	0.254411	7.60952	8.15188	2.27296	2.04821	1.83339	0.42335	1.44569
ENSG0000(	HHLA3	1:7035480	12.7144	11.6039	16.9983	10.1109	25.3349	8.8919	6.52695	10.6081	4.64361	9.26034	0.2466	-0.98578
ENSG0000(	CDCA2	8:2545899	1.90265	8.51987	0	0	0.132375	3.84814	4.00164	0.052939	0	3.28142	0.8404	0.295216
ENSG0000(	PRKAR1A	17:682591	121.231	110.817	53.4754	53.8373	32.8746	109.572	109.696	73.5425	61.2667	61.0999	0.8634	0.058144
ENSG0000(	RP11-110I	11:118998	0.598716	0.319259	0.074376	0.099604	0.158501	0.341421	1.05334	0.08135	1.67262	0.444502	0.25835	1.74329
ENSG0000(	BET1P1	7:1507165	0.543649	3.66708	0.667296	2.36055	0.321332	1.27011	1.70059	0.993944	1.07943	0.69043	0.828	-0.27816
ENSG0000(	FASTKD5	20:310691	10.3675	19.9842	8.33566	5.30875	7.86833	14.6416	9.33752	13.2317	7.00524	12.2127	0.7728	0.123531
ENSG0000(	FAM129C	19:175233	0.162035	6.10649	1.25409	0.739416	0.815789	0	0	0	0	0.18884	0.2216	-5.45961
ENSG0000(	ANAPC13	3:1344777	72.9509	73.6	64.183	47.8481	47.2984	76.0394	72.2283	62.7735	43.7491	22.9413	0.821	-0.09816
ENSG0000(	FAM21C	10:457064	23.337	29.9189	11.5564	12.0361	14.7307	12.8597	18.9297	10.7459	8.70292	22.7374	0.5933	-0.20551
ENSG0000(	RP4-753P	X:1297799	3.47137	1.04712	1.44149	1.99774	1.20849	1.48276	0.129486	0.137114	3.35192	0.830368	0.76715	-0.38396
ENSG0000(	IL10RA	11:117986	174.563	242.183	108.52	114.089	74.6235	99.4153	161.157	71.1073	93.1564	109.143	0.19135	-0.49796
ENSG0000(	AMIGO3	3:4967400	0	0.53603	0.159644	1.05668	0	0.950015	0	0	0.088384	0.257783	0.8545	-0.58886
ENSG0000(	S100A6	1:1535336	576.052	278.84	976.913	1039.55	463.668	592.282	555.687	1133.51	1017.13	136.975	0.97425	0.012217
ENSG0000(	SNRPC	6:3475740	150.741	147.623	290.773	291.592	133.172	228.612	161.525	190.148	164.648	34.4676	0.21815	-0.44672
ENSG0000(	ITPR3	6:3362036	6.16058	5.93807	4.04808	2.59869	6.82142	3.19747	9.54157	4.98064	3.67365	14.9566	0.5155	0.350993
ENSG0000(	CENPF	1:2146031	7.0668	16.2714	0.438878	0.134437	0.236191	22.4606	21.7937	1.8115	2.49359	5.88643	0.2424	1.18767
ENSG0000(	TRABD	22:501859	69.2825	110.443	60.8064	63.0375	67.8476	92.8406	134.745	112.438	94.138	113.755	0.1225	0.525328
ENSG0000(	HDAC9	7:1808694	12.1275	8.72618	2.77779	4.80589	1.8247	3.93377	2.16834	4.04351	7.32205	2.30791	0.52225	-0.40675
ENSG0000(	ASH2L	8:3810524	22.1748	31.9031	22.2106	20.8108	14.268	29.9565	16.5765	21.323	6.63683	18.3345	0.63455	-0.29769
ENSG0000(	HIST1H2A	6:2783775	1.6516	0.634496	1.4289	0	0.539116	2.21765	3.42604	0	0	0.612555	0.84805	0.336792
ENSG0000(	SLC25A14	X:1303398	19.2801	22.0457	15.0454	21.1031	6.23656	16.4288	8.08614	16.0744	9.98508	13.8097	0.55485	-0.31315
ENSG0000(	CPNE7	16:895757	27.9685	1.51983	51.2393	42.3927	28.1342	57.7782	49.7983	114.5	96.3908	51.2406	0.0183	1.23421
ENSG0000(	MUM1	19:135471	9.39624	10.9154	5.89028	5.61978	5.73323	14.0553	15.1428	6.59493	1.86414	16.0421	0.35255	0.490509
ENSG0000(	C21orf62-	21:327720	1.69804	2.57145	4.73896	4.22871	2.95415	5.91548	2.77771	5.36982	0.78021	1.15215	0.84395	-0.14206
ENSG0000(	METAP2	12:954735	25.0823	25.5193	24.1419	31.5879	29.4932	33.5537	30.2931	22.3636	22.854	27.4058	0.8225	0.090301
ENSG0000(	RP11-338I	12:123712	0.526809	1.57742	0.492203	1.14466	0.147218	0	0.539097	1.17045	4.21689	2.43763	0.41415	1.37556
ENSG0000(	CAMKK1	17:386031	1.00203	1.30091	0	0.539588	0.366275	1.42337	0.654263	0.994392	0.379665	0.493661	0.7598	0.365745
ENSG0000(	PRICKLE3	X:4917526	3.06291	1.56716	0.562328	1.3564	1.57992	1.21144	2.96102	1.50499	1.07829	1.63996	0.9552	0.048896
ENSG0000(	HEBP1	12:129277	7.12641	18.8313	7.38401	4.50828	11.6192	14.3782	8.15629	10.1926	8.26209	3.10376	0.83105	-0.1444
ENSG0000(	CTC-301C	19:493316	2.48493	0	0.977863	1.35395	0.980424	3.01513	1.10259	0.635423	0.204501	0.221378	0.852	-0.28626
ENSG0000(	SELP	1:1695888	0.236547	1.63607	0.026508	0.783863	0.125783	0	0.080308	0.385485	0	0	0.17115	-2.61617
ENSG0000(	IGLV3-9	22:228190	0.209824	0.692052	1.23372	0.388153	0.497451	0	0.888192	0.358838	0	0	0.37785	-1.44833
ENSG0000(	FAM98C	19:384031	24.9153	24.7719	22.2806	22.2388	30.0024	23.0905	23.8626	30.5855	18.0119	18.2986	0.7903	-0.11421
ENSG0000(	TOB1	17:508622	61.9821	40.3475	53.5249	83.9636	40.154	17.8403	22.1222	36.3779	83.3575	35.339	0.12245	-0.54957
ENSG0000(	C1orf147	1:2064704	0.739011	1.75458	0	0	0.016334	0.893628	0.946636	0	0	0.632968	0.9878	-0.02995
ENSG0000(	PAM	5:1027539	22.7333	14.7519	10.8382	10.9629	10.0894	16.6643	21.2114	9.19963	16.6343	15.4639	0.62285	0.23245
ENSG0000(	PIN4	X:7218135	14.6559	15.7715	24.1741	24.8857	21.0614	19.2113	25.5449	16.9148	20.4581	8.37156	0.90055	-0.17135
ENSG0000(	COX7C	5:8661790	349.331	223.255	429.785	368.562	186.674	366.771	400.274	395.094	283.644	47.2746	0.8322	-0.07439
ENSG0000(	CCDC51	3:4843216	13.963	10.7523	15.2782	8.76043	12.3243	22.184	12.6325	9.78176	15.4184	10.3283	0.76635	0.233161
ENSG0000(	REST	4:5690787	71.2447	84.4366	52.9024	25.9977	52.8317	96.6773	72.5425	32.2221	30.9481	110.875	0.76975	0.136195
ENSG0000(	DDAH2	6:3172703	30.0308	12.2616	23.6394	30.7653	47.6216	61.9628	47.2979	50.2249	25.5297	34.6862	0.4256	0.543489
ENSG0000(	HRH4	18:244606	0.101346	0.173678	0.008472	0.020235	0	0.218925	0	0.592315	1.02833	0.329444	0.1228	3.14142
ENSG0000(	UBA2	19:344283	34.9683	36.6569	11.7571	6.33545	20.3233	41.1068	33.893	17.2191	8.03956	20.2565	0.9117	-0.04604
ENSG0000(	PARP16	15:652344	19.4959	20.4326	17.4229	24.638	20.6734	10.0913	10.153	14.1995	9.22821	25.1646	0.0968	-0.66646
ENSG0000(	RP11-33B	4:1194547	23.0102	27.0579	23.1481	19.6713	7.83738	25.0691	11.8017	8.39177	37.655	11.1506	0.86595	-0.0957
ENSG0000(	ARAP3	5:1416534	0.218755	0.616227	0.066277	0.024579	0.156981	0.561643	1.39086	0.077006	0	0.707753	0.2038	1.40883
ENSG0000(	RP11-301(	2:8614393	2.34442	0.980122	2.24705	1.64957	0.610807	0	1.12163	4.46642	2.65027	0.826966	0.76455	0.400231
ENSG0000(	AMZ2P1	17:649665	13.1517	9.83712	13.1547	20.7247	5.86655	12.5177	12.0629	12.0799	9.61288	4.8616	0.46835	-0.3937
ENSG0000(	NFS1	20:356260	9.68495	15.7663	17.3194	13.0604	11.6363	9.31408	8.7333	11.2077	14.4891	15.2675	0.7067	-0.19644
ENSG0000(	CDC42P6	4:2269291	1.12311	0.649445	0.206975	0.756192	0.364685	0.800151	0.794134	0.076529	0.913292	0.163757	0.95625	-0.08956
ENSG0000(	RN7SL329	8:1250242	0.356052	0	0	0	0	0	0.680223	0	1.80747	0.318085	0.2414	3.60227
ENSG0000(	TMEM147	19:355334	62.5112	56.064	142.534	137.917	138.302	81.2102	91.6916	135.767	151.054	56.8617	0.82875	-0.08175
ENSG0000(	CTD-2006	17:745998	0.199931	1.71123	0.870431	0	0.229288	0	0	0.093192	0	0.015204	0.2044	-4.51005
ENSG0000(	RP11-176I	14:312486	0.774037	0.469595	0.713803	0.449969	0.587066	0.071758	0.975392	0.270087	1.43594	0.45257	0.9195	-0.11808
ENSG0000(	LSM1	8:3810524	63.4723	37.5069	76.5662	82.5515	53.7963	69.4811	69.1793	71.1658	52.9211	15.9674	0.70835	-0.20423
ENSG0000(	WDHD1	14:549389	4.63616	4.61974	1.6867	1.82571	1.41636	6.45201	2.65642	1.85673	2.77864	5.76648	0.7905	0.199775
ENSG0000(	SDHDP6	1:2524223	1.19087	1.62943	1.01956	0.658502	0.654833	2.33947	0.703726	0.882368	0	0.434733	0.8392	-0.25558
ENSG0000(	TST	22:370108	2.79817	1.19483	1.2192	3.41396	2.35479	4.95462	0	1.86266	3.65438	0.624637	0.99525	0.009815
ENSG0000(	USP16	21:290242	31.9425	32.0232	25.6332	23.1792	32.7523	51.179	41.0007	36.2579	26.163	42.5718	0.2596	0.379636
ENSG0000(	SLC2A5	1:9035106	0	0.273536	0	0.116433	0.18989	4.51584	0	0	2.0113	0.012632	0.06765	3.60851
ENSG0000(	PACSIN1	6:3446606	1.04424	1.07014	0.734322	0.38472	2.36628	0.627162	0	0	0	0.838015	0.1279	-2.6205
ENSG0000(	PAFAH1B2	11:117144	7.26677	7.60561	14.7871	7.50415	9.83776	7.79215	7.73332	10.8851	6.22925	16.1992	0.71535	-0.13673
ENSG0000(	NDUFB2-/	7:1405184	1.05654	0.677936	1.94652	1.89116	0.757344	0	0.744146	0.678794	0.528781	0.854656	0.3328	-1.21608
ENSG0000(	MPST	22:370108	22.3267	13.7793	24.7352	28.2877	32.9939	38.3431	48.1202	22.0302	18.4216	8.98238	0.92565	-0.04395
ENSG0000(	TMEM86A	11:186931	0.227975	0.378234	0.889956	0.224582	0.486191	1.52973	0	0.453704	1.57447	0.392548	0.5044	0.817147
ENSG0000(	HCG18	6:3023403	45.787	24.6974	30.0659	35.0382	36.9428	28.6046	24.905	18.2679	13.5923	34.8934	0.18205	-0.65395
ENSG0000(	RPL22L1	3:1708648	28.6299	10.7599	35.1936	28.1781	9.53204	34.1803	33.7035	37.4903	16.5037	2.76606	0.81675	0.20668

ENSG0000	ASCC1	10:720960	20.9817	25.127	11.7448	19.4245	11.3247	12.5821	18.761	13.6474	10.1916	16.0411	0.65725	-0.34366
ENSG0000	IL17A	6:5218638	0.423758	0	0.019421	0	0	11.9474	106.763	76.6724	4.51846	8.46465	0.0064	9.32168
ENSG0000	CTD-2012	16:675492	1.27826	1.77398	0.190952	0.31036	0	3.99436	0	0.788477	1.7283	0.599598	0.49515	1.17979
ENSG0000	SNTB2	16:691055	7.10297	6.09517	17.2849	11.415	29.5163	9.16665	20.9959	20.6306	9.99604	53.1699	0.6248	0.39823
ENSG0000	NDUFB5	3:1796046	58.216	57.3428	76.7221	84.8099	41.1307	79.0338	78.4903	90.6332	106.276	17.969	0.665	0.156254
ENSG0000	DDX54	12:113149	25.167	27.3676	23.6548	32.4715	31.1955	21.9142	32.2607	21.194	21.0627	33.1143	0.70755	-0.13519
ENSG0000	TMEM218	11:125096	31.4474	21.563	28.6697	41.4371	23.2847	28.2914	17.3461	33.5071	40.8212	24.466	0.9221	-0.03723
ENSG0000	RP4-655J1	1:1164237	0.626735	0.081881	0.354491	0.529103	0.194422	4.33603	0.864796	0.071526	0	0.120353	0.16965	1.43288
ENSG0000	LEPROT	1:6542058	7.40837	9.12014	10.2827	8.88933	5.61409	18.8397	11.7873	10.4819	2.98827	9.45064	0.63665	0.179752
ENSG0000	MRPL20	1:1399521	120.319	87.3323	174.446	164.534	169.211	205.482	158.306	140.38	95.8838	63.8482	0.6238	-0.23537
ENSG0000	CRIPT	2:4649074	21.5719	14.6887	22.0809	15.6092	11.9437	28.4013	27.7033	19.6424	18.9799	3.08033	0.82855	0.154217
ENSG0000	BCL2L11	2:1111193	5.75677	8.85422	3.12447	5.23029	8.76335	8.50584	13.4269	11.944	3.7534	10.7021	0.53105	0.349157
ENSG0000	CH507-9E	21:507345	14.3047	13.0578	16.965	19.9971	14.3145	9.4505	5.51867	8.5558	15.9134	11.7703	0.2891	-0.65908
ENSG0000	VAPB	20:583891	15.8716	12.7986	7.33075	7.19499	7.46143	9.19401	16.061	11.2813	19.1567	9.47021	0.4884	0.319086
ENSG0000	ZSCAN16	6:2812179	14.4374	9.26605	17.2571	14.1574	11.4254	6.23165	10.2315	13.9543	10.5647	5.70924	0.3719	-0.48178
ENSG0000	AC107081	2:6186808	0.186558	1.46333	0.170257	0.462699	0.186985	0	0	0.275243	0.087579	0.515277	0.4685	-1.36567
ENSG0000	FAM13A	4:8870978	2.61618	8.49558	1.74471	2.73781	2.22074	0.045247	1.15078	1.94404	0.420789	2.61541	0.0353	-1.43502
ENSG0000	CD2BP2	16:303507	26.1846	34.9702	36.1637	30.9463	40.6016	44.835	48.987	55.8029	17.0952	38.3562	0.8372	0.088904
ENSG0000	DUSP8	11:155404	0.367778	0.585869	0.859367	0.071166	1.0349	0.239824	0.802977	0.686921	0.110002	0.635247	0.85425	-0.33808
ENSG0000	TOMM34	20:449100	19.1462	11.2075	22.3806	20.976	11.2155	16.2439	14.7513	21.6926	13.1385	14.8318	0.81915	-0.10283
ENSG0000	NABP1	2:1916780	32.7477	38.9878	23.415	29.4161	18.0922	58.9794	34.0995	49.2274	90.0271	45.7909	0.01825	0.978406
ENSG0000	AP4S1	14:308745	5.30941	3.31999	3.83751	1.69634	2.68352	6.80595	8.76341	5.86866	6.62757	3.48601	0.40285	0.678729
ENSG0000	RP1-178F1	1:1536187	0.201055	0.3833	0.674301	0.500444	0.203861	0	0.065971	0	0	0.088743	0.113	-3.69551
ENSG0000	RIF1	2:1514098	10.2202	9.98858	4.17527	5.01146	5.28239	7.69056	11.4646	8.77264	8.02663	18.8848	0.6652	0.254646
ENSG0000	NPIPB11	16:293813	1.7922	4.67979	0.821668	1.07671	0.726705	4.04551	3.46976	1.27141	1.80852	2.95416	0.92055	0.2181
ENSG0000	FLOT2	17:288793	103.578	116.233	66.5959	63.7954	76.6647	81.48	82.4371	54.6668	60.473	67.8271	0.2728	-0.33948
ENSG0000	TMEM67	8:9375484	1.76299	3.96301	1.06858	3.70698	0.764745	0.685762	5.94775	0.486098	4.09384	0.94285	0.61115	0.354473
ENSG0000	P2RX7	12:121132	7.07131	7.52202	3.30313	2.4422	5.73986	8.87401	7.79718	3.88712	3.68667	12.7122	0.70645	0.177244
ENSG0000	CHPF	2:2195389	4.92233	3.51009	4.6178	5.0115	6.99015	2.60406	6.19638	9.89679	6.0717	14.3307	0.3125	0.559647
ENSG0000	FAM84B	8:1265524	5.41108	7.37176	3.04043	2.55531	3.65466	0	1.74372	4.07188	4.97168	2.26786	0.3869	-0.70828
ENSG0000	C3AR1	12:805830	22.1537	4.37639	26.6125	24.5038	22.5513	53.809	27.7863	36.4684	45.3294	50.0829	0.01085	0.985556
ENSG0000	LIPC	15:579534	2.02939	1.42446	11.9624	0.603302	2.37322	2.27458	1.122	2.08447	3.06434	0.42693	0.38545	-0.97093
ENSG0000	NEO1	15:730517	4.70705	0.153994	2.51056	1.55348	2.82201	0.402331	0.474083	1.72789	1.58892	4.59668	0.62125	-0.32156
ENSG0000	FAM172BF	3:1015218	0.147225	0.752948	0.280046	2.69944	0.378474	4.12747	2.08246	1.54516	0	0.843909	0.6551	0.939857
ENSG0000	PEPD	19:333869	75.5197	71.8085	81.2639	84.93	83.0929	68.4635	47.6747	54.9639	58.9211	60.9426	0.1086	-0.50971
ENSG0000	RP11-403,	18:239577	0.475499	0	0.150295	0.718926	0.324918	0	0	1.50924	1.96501	0.309802	0.4817	1.30878
ENSG0000	DCAF1	3:5139126	8.78426	9.0842	10.448	4.47836	14.144	5.10792	5.76114	13.5192	4.88625	27.1172	0.87855	0.075622
ENSG0000	BEND5	1:4853285	0.182559	4.48165	0.558288	2.43784	0.406136	0	0	0	1.76347	0	0.2007	-1.92042
ENSG0000	PYCR2	1:2258862	31.6972	42.8875	31.2384	22.4645	31.6621	21.1018	28.3844	25.144	19.8823	29.8546	0.33035	-0.36997
ENSG0000	RARS	5:1684864	24.6912	24.8895	24.2408	25.6899	23.1222	30.789	24.4092	21.0196	41.4245	28.3254	0.5376	0.231007
ENSG0000	CTD-2583	19:577696	2.71574	0.745882	2.66252	2.15969	0.659792	0.915607	2.13952	1.23262	0.719663	1.74668	0.9342	0.070449
ENSG0000	RP11-225	10:103452	0.476613	0.305188	0	0.065759	0.225227	0	1.78133	0.777146	0	0.069303	0.5021	1.24047
ENSG0000	UPK1A-AS	19:356665	0	0	0	0	0	0	0	0	4.00135	0	0.21455	inf
ENSG0000	DDHD1	14:530367	10.4495	13.0973	4.69899	5.30174	3.97197	9.65124	9.52321	7.87608	9.50241	12.3467	0.3375	0.483058
ENSG0000	RPS2P44	16:485387	0.624607	0.932558	0.312834	0	0.272187	2.50072	3.35158	0	0.338602	0.201856	0.37995	1.50967
ENSG0000	KDM8	16:272034	3.42737	8.12489	4.70817	4.9948	3.58505	1.75166	2.71186	3.44184	5.71545	5.63865	0.549	-0.29718
ENSG0000	POLDIP3	22:425837	47.9413	52.5701	23.0471	32.1366	18.3593	36.331	34.081	24.8009	29.507	34.8052	0.8422	0.064278
ENSG0000	HAUS4	14:229296	8.49826	12.2914	12.776	18.7751	7.67881	19.1303	8.98619	15.0373	14.7585	7.6362	0.78975	0.119242
ENSG0000	TRAV20	14:220405	10.0557	20.6717	14.0781	4.53196	0.705481	15.9761	22.106	9.32786	0	3.22696	0.93735	0.057085
ENSG0000	GUSBP5	4:1435594	2.40175	1.00728	3.57709	0.744241	1.95115	1.56792	1.41504	1.34921	2.73423	3.02472	0.98165	0.021913
ENSG0000	PFDN2	1:1611005	76.9269	50.4963	140.841	134.181	132.939	139.321	149.408	134.325	98.5393	32.5403	0.91835	-0.06256
ENSG0000	AC092171	7:5431334	0.458558	0.218031	1.35644	0.213302	1.10438	0	0.241866	0.702071	0.366625	4.57475	0.69935	0.656058
ENSG0000	GET4	7:816614-	40.2216	48.7711	53.6004	50.5494	44.4264	47.9613	72.6831	53.719	26.7594	48.2139	0.94215	-0.03276
ENSG0000	TFE3	X:4902872	4.65048	4.77056	1.696	1.14564	2.0136	3.718	3.99292	2.51291	2.47761	9.52764	0.2313	0.716382
ENSG0000	RP11-419	8:1253707	3.85185	1.91825	2.34344	5.94281	1.3212	2.55682	2.88792	1.82491	5.11797	0.850062	0.9033	-0.16891
ENSG0000	U2AF1	21:430929	61.8426	67.4788	70.3793	60.3961	109.358	58.9671	107.501	69.5063	52.6047	55.9687	0.8326	0.16922
ENSG0000	MORN4	10:975843	3.29512	0.427265	8.9334	7.18837	0.737911	4.69766	2.17649	2.97439	0	0.477776	0.57425	-0.87293
ENSG0000	METTL5	2:1697919	54.1925	35.1649	58.5789	54.2752	35.4015	57.3802	48.5369	44.6976	63.1427	17.8896	0.9428	-0.05396
ENSG0000	CPNE3	8:8651442	20.4343	17.9692	14.3061	13.1839	49.9585	21.11	12.7202	12.5684	19.0839	39.3329	0.51055	-0.39279
ENSG0000	RAP2C	X:1322030	17.5251	10.4472	11.7523	11.3262	10.0115	24.1575	21.1932	16.3579	10.9076	18.5426	0.1931	0.551194
ENSG0000	GUCD1	22:245404	16.414	24.1161	12.585	17.1718	13.1269	6.96031	13.2711	13.5185	11.3095	19.7151	0.5926	-0.32483
ENSG0000	CCDC22	X:4923546	23.0927	17.5175	20.4439	24.1123	21.1204	20.0163	21.2905	21.7622	11.3686	21.0388	0.75515	-0.21519
ENSG0000	C16orf59	16:246007	0.956164	4.98774	0.288936	0.521176	0.913163	14.3627	4.17065	2.05891	0	1.40215	0.11305	1.63072
ENSG0000	ST3GAL4	11:126355	6.41258	2.34973	7.21566	13.7654	8.4286	7.98369	10.8474	7.02225	4.78566	2.26436	0.5353	-0.32928
ENSG0000	TRAV9-1	14:218115	0	0	0.38492	1.52317	0.13622	0	0	0	0	1.32836	0.86635	0.233383
ENSG0000	SKA3	13:211535	2.12896	3.80806	0.164186	0.213581	0.287433	5.10684	5.10899	0.569352	3.97894	2.08392	0.0715	1.33725
ENSG0000	WDR45B	17:825197	36.5766	29.6406	37.526	46.9452	24.9685	19.9173	35.3177	28.3605	28.7016	33.5732	0.3764	-0.32543
ENSG0000	DPP4	2:1619922	85.407	15.5053	50.0749	66.5457	33.9955	45.3308	7.65293	85.5031	53.6423	38.3152	0.70705	-0.12874
ENSG0000	RP1-40E1	6:3063990	9.86291	4.15871	3.0997	4.43286	3.03507	4.94303	6.23377	6.71128	0	3.343	0.83715	-0.36723
ENSG0000	SSH2	17:295605	20.4432	32.5925	12.0498	22.7801	15.1554	10.3569	12.121	11.1333	28.4303	19.5545	0.5986	-0.38111
ENSG0000	DFFBP1	9:3254054	0.33791	0.778252	0.551111	0.178024	0.098172	0	0.10719	0.148528	0	0.261357	0.27515	-1.78916
ENSG0000	CCDC78	16:720580	1.97222	0.842625	0.419056	0.97079	0.988141	1.86125	2.07695	4.29979	0	0.46135	0.4044	0.68035
ENSG0000	TRAV36DV	14:222025	9.15929	13.6751	1.39022	2.06866	0	13.2909	10.9386	1.00689	0	0.465532	0.96745	0.075986
ENSG0000	MSC-AS1	8:7182816	2.68202	0.447004	5.73282	1.27832	3.28661	5.79701	3.07046	7.15039	12.0904	6.87231	0.1689	1.29795
ENSG0000	RSPRY1	16:571861	22.7265	19.8123	24.0627	25.0274	20.8128	21.442	19.3567	17.6559	19.9926	20.1642	0.2951	-0.35227
ENSG0000	RP5-967N	20:595065	0.850107	1.12615	0.23146	0	0.249311	0	0	0.420749	0	0.862023	0.8239	-0.39922
ENSG0000	KIAA1429	8:9448769	9.2308	9.2625	4.72929	3.36697	7.71703	7.05473	5.8353	6.17342	5.63527	12.3047	0.61045	0.288115
ENSG0000	SERPINB1	6:2832331	46.7203	56.485	47.9811	42.7098	40.6868	58.6892	83.651	46.5466	38.2889	32.82	0.68805	0.12365
ENSG0000	DDX1	2:1559117	50.2946	61.474	46.7692	38.5775	35.9462	33.7861	26.0714	34.3508	18.4465	37.6423	0.061	-0.61567
ENSG0000	RIOK2	5:9708907	14.0971	15.4469	15.1348	10.0572	8.8767	11.8252	20.5725	8.87372	7.22877	8.1768	0.40245	-0.36023
ENSG0000	TPCN1	12:113195	3.24862	15.3104	2.02357	1.34704	1.69286	6.34379	3.41715	3.98426	0	5.87707	0.4988	-0.39652
ENSG0000	EXOSC8	13:369497	57.3388	76.4795	84.5737	82.7354	52.4395	61.3107	63.7813	69.3142	59.6142	41.0411	0.5309	-0.30259
ENSG0000	LRRN3	7:1106630	1.35683	47.9229	1.39052	0.683249	0.257324	1.00115	0.90922	0.346624	2.32196	1.12212	0.02435	-3.0984
ENSG0000	SPIN2B	X:5711855	23.5429	14.5739	16.9692	15.1468	24.9385	9.54583	11.3074	10.4603	16.2782	13.9519	0.34955	-0.55506
ENSG0000	UBE2V1	20:500811	148.169	169.815	205.186	143.28	228.22	167.407	153.222	142.087	135.616	195.801	0.39305	-0.29016
ENSG0000	OR2A1-AS	7:1441860	14.0892	9.87936	5.57112	5.05784	2.38892	7.33893	2.98659	3.18346	2.05875	1.83503	0.065	-1.39028
ENSG0000	LINC0127	20:502674	1.02457	10.7809	0.339775	2.06205	2.71257	39.7472	2.69169	4.13043	3.56768	3.20217	0.0622	1.68656
ENSG0000	RNF220	1:4440519	50.5081	64.6615	34.3932	40.0235	31.8865	35.9573	55.7389	28.4608	28.6907	25.5779	0.2587	-0.40038
ENSG0000	ARNT	1:1508097	7.65826	13.4702	3.27459	1.88125	4.60595	4.03221	5.69058	8.45888	13.5515	8.04354	0.31025	0.465279
ENSG0000	VARS2	6:3090818	7.20415	6.30614	1.35513	0.731425	2.70197	4.43212	4.35002	2.03009	0.431215	2.97493	0.7991	-0.23508
ENSG0000	LAMP3	3:1831222	1.04282	3.40622	0.311413	0.359249	0.560372	0	36.8273	14.0033	0	2.11315	0.0214	3.24135

ENSG0000	ZNF571-A	19:374971	1.28322	1.16605	0.089338	0	0.211046	0	0	0.178098	0	0	0.00035	-3.75162
ENSG0000	EBLN2	3:7288807	2.09382	2.57969	2.67128	1.81529	0.717292	1.81621	3.38523	2.42841	3.74305	4.02423	0.712	0.626711
ENSG0000	ARHGEF9	X:6363496	4.4521	4.43513	4.02894	1.12675	2.4551	14.3622	9.27077	4.30838	7.81466	6.36953	0.05165	1.17948
ENSG0000	SRD5A3	4:5534610	4.43714	3.81863	5.49382	3.15196	6.62746	4.96362	2.24783	4.43744	1.79101	2.95697	0.3137	-0.65175
ENSG0000	EIF1AX	X:2012451	16.1343	10.828	32.3433	11.9733	46.0031	18.5552	26.0443	25.8548	25.7518	26.8863	0.72365	-0.24938
ENSG0000	TSNAXIP1	16:678067	0.447594	0.513791	0.156718	0	0.047417	0	0.301589	0	2.72074	0	0.40675	1.67075
ENSG0000	ENDOV	17:802608	8.4541	6.98458	8.57494	11.5189	6.94143	10.7562	23.5576	15.9909	10.7435	19.7861	0.2716	0.775022
ENSG0000	TCAP	17:396641	0.760898	2.84609	0	0	0.075489	0.67713	1.16121	0	0	0.028025	0.5505	-1.1313
ENSG0000	HDAC5	17:440767	18.5739	20.9442	9.51667	9.78411	9.17338	16.0555	7.74074	12.877	4.48025	14.1079	0.3781	-0.33841
ENSG0000	EPHA1	7:1433810	0.480165	8.49102	0.710973	0	0.48612	9.28437	5.30725	10.7631	8.32707	17.8072	0.029	2.72716
ENSG0000	SETX	9:1322613	20.9308	20.6762	8.18095	6.96633	13.0928	15.7225	20.2542	9.99158	7.44089	26.619	0.73335	0.144062
ENSG0000	FBXO7	22:324746	102.378	81.5803	113.126	127.004	93.0464	77.569	114.315	97.0137	95.6083	105.022	0.68545	-0.13831
ENSG0000	SRI	7:8820511	134.864	101.837	195.691	173.934	176.766	177.085	205.917	213.223	142.046	74.2798	0.94145	0.027798
ENSG0000	NSUN5	7:7330251	36.5229	48.882	44.7469	35.4186	47.5348	39.1796	39.0361	40.1601	21.5277	53.463	0.6377	-0.17899
ENSG0000	AARS2	6:4427046	6.59362	3.06741	4.28883	2.35114	2.74004	2.66856	4.43235	2.57323	5.44963	3.09223	0.8543	-0.08547
ENSG0000	BTBD8	1:9208030	1.1739	0.155907	0.46586	0.227896	0.316717	0.425333	1.35873	0.344561	0	0.756012	0.86195	0.183797
ENSG0000	MBOAT7	19:541734	9.01403	13.3847	19.6508	14.755	25.2856	11.0896	9.48307	29.8945	22.5911	34.2384	0.2729	0.432012
ENSG0000	UBLCP1	5:1592630	37.9348	34.8423	30.5094	31.4428	26.7907	27.0057	40.2825	25.2065	20.1556	20.8329	0.4266	-0.26704
ENSG0000	APOPT1	14:103553	33.5783	26.2218	32.3304	28.2565	28.3372	57.2258	46.9979	37.2872	21.7746	4.09924	0.93675	-0.05455
ENSG0000	CCSAP	1:2293194	6.49318	1.65329	2.02977	2.02986	1.16655	6.05997	2.21761	1.73116	2.2644	2.57576	0.649	0.237413
ENSG0000	RP11-119	10:686984	1.00974	0.798234	0.591692	0.719755	0.659819	0.82687	1.34365	1.11072	0	2.1106	0.46765	0.494861
ENSG0000	AATF	17:369488	69.0501	67.0339	59.2792	50.0627	66.3095	100.588	83.7303	45.2194	30.4058	72.5503	0.8255	0.069215
ENSG0000	RRS1	8:6642902	15.0635	15.8147	29.2227	14.7202	35.7107	10.9668	14.9192	24.1612	3.13239	20.9734	0.4334	-0.64746
ENSG0000	CAPNS1	19:361395	21.05	19.2406	32.6004	19.1946	82.8777	21.9707	33.3144	48.2437	10.8072	48.3672	0.6878	-0.1864
ENSG0000	ZNF607	19:374971	1.37377	0.914385	0.340935	0.329847	0.284075	0.235008	1.03418	1.12715	0	1.09365	0.73035	-0.36125
ENSG0000	TMPO	12:985129	36.8424	48.5997	11.8858	15.215	12.4079	27.8882	31.922	20.5465	17.4751	20.7	0.95175	0.021933
ENSG0000	SVBP	1:4276726	27.4192	24.661	18.4112	21.6323	12.6398	35.2683	35.3055	37.2642	14.8007	6.29094	0.68895	0.320956
ENSG0000	IQCH	15:672010	0.145486	0	0.090634	0.127458	0.459925	0.525185	0	0.404236	1.30938	0.548869	0.03435	2.45074
ENSG0000	AC009299	2:1610962	1.90722	0.62172	0.074239	0	0.124889	3.15427	3.95175	0	0.086262	0.199627	0.6075	1.13033
ENSG0000	CRTAP	3:3311397	11.3534	16.6297	24.3845	12.8032	23.427	17.0383	12.9943	13.7088	10.1347	24.4233	0.2968	-0.47581
ENSG0000	TMEM204	16:149334	22.675	74.1396	99.0745	69.8372	76.229	20.8443	33.5857	40.8333	38.1376	58.9908	0.0889	-0.81077
ENSG0000	NEFH	22:294802	2.7724	1.70588	0.984377	0.589216	1.41968	5.3428	0.868471	0.81362	0.020936	1.00188	0.9047	0.09029
ENSG0000	SUSD4	1:2232208	3.93333	7.85933	2.57472	5.48341	4.07422	0	0	0.045259	0.361085	0.832426	0.02055	-4.3939
ENSG0000	NPTN-IT1	15:734431	0.79881	2.30641	0.68951	0.063274	0.332386	0.019834	0.424842	0.817892	0.976214	2.72891	0.89285	0.301733
ENSG0000	SIAH2-AS	3:1507411	1.01391	0.584551	0.213229	0.522304	0	0.587464	0.587919	0.11459	1.06732	0.280729	0.77835	0.379026
ENSG0000	SPTBN1	2:5445628	17.7168	29.7423	9.94294	3.68527	7.77035	5.32077	5.93398	6.16955	7.8933	10.1385	0.03405	-0.91022
ENSG0000	RP11-804	3:5066998	1.76265	1.31072	0.573048	2.26661	0.826484	1.79609	1.62308	3.87468	0	1.40862	0.78365	0.361263
ENSG0000	HIST2H2A	1:1498869	16.4594	13.9374	17.6266	6.91028	12.1461	15.7019	6.51529	3.10091	3.09718	0.473034	0.367	-1.46348
ENSG0000	GORASP2	2:1709284	72.4984	65.5949	81.5325	83.6101	77.9488	36.4074	53.3011	45.4912	66.1721	76.8305	0.1175	-0.48762
ENSG0000	OSBP	11:595743	18.9523	24.1618	4.03318	2.78721	3.83097	10.5016	14.501	5.08884	6.74774	10.1444	0.71935	-0.14886
ENSG0000	DDX21	10:689561	17.967	15.3345	14.3263	15.4752	17.103	14.5668	21.3652	19.6465	18.3693	26.7088	0.38885	0.301175
ENSG0000	HNRNPD	4:8235249	226.527	320.53	80.9241	44.2046	143.909	186.549	233.886	92.8845	64.8103	196.475	0.8393	-0.08034
ENSG0000	REM2	14:228831	1.18746	2.42909	0.974637	1.34115	0.409165	0.576173	0.770898	0.196794	0	0.200057	0.3147	-1.9658
ENSG0000	RLN2	9:5299867	5.57266	2.78195	4.09226	3.27559	1.69333	5.81215	2.99708	2.17628	5.27335	2.18565	0.8784	0.138207
ENSG0000	H1FX	3:1293147	105.874	96.3471	32.6663	27.5833	43.6528	93.6761	75.6424	45.7423	25.9578	42.5117	0.6392	-0.17989
ENSG0000	EXOC6	10:928347	6.03905	7.26105	3.08086	3.21292	2.13353	9.55191	9.92726	3.02187	3.95871	5.61506	0.28425	0.580958
ENSG0000	GTSE1-AS	22:462951	1.04917	0.055216	0.267288	0	0.176231	0.58176	0	1.82343	0	0.306092	0.4185	1.25362
ENSG0000	DPM2	9:1279345	31.0348	32.9134	72.8655	71.0788	76.1063	35.1458	45.0027	58.8112	53.5555	42.7211	0.71695	-0.3226
ENSG0000	SIDT2	11:117178	3.22544	16.4895	3.09907	1.6652	5.18638	6.59315	3.17634	4.31668	4.22522	12.7379	0.62575	0.253821
ENSG0000	FAM3C	7:1213488	25.5927	12.0457	24.8585	18.607	18.522	24.8281	26.6804	29.7996	11.6229	19.3054	0.6017	0.184569
ENSG0000	ETFBKMT	12:316471	0.392688	0.8179	0.422685	0.933047	0.168851	0.847111	0.11867	0.694992	2.99245	0.124077	0.3813	1.02944
ENSG0000	BDH2	4:1030198	40.9339	24.7353	25.6251	20.8079	24.905	42.4118	34.852	18.8484	18.4538	15.2498	0.8424	-0.10685
ENSG0000	ZNF141	4:337813-	4.65964	5.24727	2.08515	2.63059	2.06761	2.83087	3.27077	0.891978	0.28182	5.16705	0.546	-0.4663
ENSG0000	CHD8	14:213851	7.57456	9.4172	3.38048	4.31793	5.20212	3.49781	4.11801	3.89275	3.55596	7.15097	0.5656	-0.31195
ENSG0000	TPM2	9:3568199	14.4185	9.62045	1.34282	0.817641	2.57399	5.93759	1.47224	1.2474	0	4.54183	0.2769	-1.11698
ENSG0000	FBXL5	4:1560453	61.6752	51.3932	49.6259	37.9478	32.9442	31.6883	36.44	28.5362	25.5086	34.4365	0.0963	-0.59709
ENSG0000	CRELD1	3:9933821	47.1168	42.0429	33.6644	36.9423	26.2793	26.3423	38.7921	21.957	17.4524	33.2333	0.17265	-0.48781
ENSG0000	TRAF3IP1	2:2383204	3.01632	3.55731	0.566935	0.910077	2.47147	4.63331	4.44542	1.25628	0.208922	4.2071	0.81085	0.172549
ENSG0000	C21orf91	21:177633	29.5413	16.1423	20.7025	25.6623	18.401	26.1279	20.837	20.6306	19.9399	15.4265	0.7213	-0.19236
ENSG0000	ZNF37BP	10:425135	6.64032	12.1898	6.98282	9.53491	3.57232	4.94099	7.55863	11.7769	8.23877	6.16724	0.955	-0.04575
ENSG0000	CYTH3	7:6161775	13.2166	3.54482	5.00921	4.01848	3.38444	4.251	0.38742	2.94381	7.44452	5.74499	0.4101	-0.39686
ENSG0000	TBC1D2B	15:779760	6.48312	12.5013	1.76234	1.73449	3.17279	11.66	16.1826	9.09107	8.54085	23.3207	0.0007	1.55729
ENSG0000	SLC38A2	12:463581	44.7823	55.7976	8.368	19.9741	10.3189	27.7032	35.3999	22.9234	25.3493	21.0388	0.94505	-0.02038
ENSG0000	EDRF1	10:125683	16.3171	12.1107	5.10463	9.85005	4.30914	13.25	9.98578	3.97403	9.29598	8.6533	0.52375	0.29979
ENSG0000	SEMA4B	15:901606	8.13348	13.5697	4.18737	3.86501	4.08664	5.12028	5.73969	1.77467	0.209748	6.39148	0.2053	-0.8446
ENSG0000	RP11-44N	8:1227799	5.41105	1.38648	2.99556	5.21334	1.0545	0	0.928432	1.35388	0	0	0.4151	-3.00292
ENSG0000	LRP8	1:5322689	2.31475	3.42428	1.16552	0.563939	0.529473	1.54495	4.87002	2.11348	2.8658	2.18082	0.2901	0.775009
ENSG0000	ARRDC1-/	9:1376056	21.8393	18.424	26.3597	25.7918	20.4246	25.6676	20.9872	30.3078	28.7811	21.8367	0.86535	0.179966
ENSG0000	BMS1P1	10:467536	2.96146	3.49608	1.5399	0.282497	0.30849	5.06177	1.14034	0.879688	0.182618	1.20183	0.9491	-0.10112
ENSG0000	CMC1	3:2824158	219.05	53.8176	221.471	174.795	67.2549	95.646	83.4497	113.584	58.967	17.3079	0.22765	-0.97316
ENSG0000	MTHFD2	2:7419856	14.0839	12.0307	13.7219	13.9387	10.6907	30.7799	55.716	43.2903	17.2529	17.4886	0.1766	1.23033
ENSG0000	CCER2	19:389089	0.439145	0.93721	0	0.123364	0.173321	3.42137	0	1.51551	1.06422	0.608549	0.17815	2.21468
ENSG0000	TIRAP	11:126283	6.13261	3.59459	5.97886	3.65476	3.48053	9.48628	4.08857	6.47295	0.023943	7.26398	0.8975	0.121942
ENSG0000	C1orf216	1:3571387	8.60708	5.12593	7.17208	7.66082	4.73978	11.9014	9.92745	12.0664	6.76602	9.20326	0.2135	0.525905
ENSG0000	AP5M1	14:572005	31.5368	27.5149	16.827	29.794	14.9279	11.1919	17.8763	15.0463	12.301	18.8611	0.1985	-0.84403
ENSG0000	CLDN7	17:724001	1.04663	1.46459	2.73066	4.5622	2.92757	0	2.11305	4.41126	6.29874	1.85671	0.89535	0.116675
ENSG0000	CCDC191	3:1139479	1.48903	4.35807	2.8243	1.45298	2.49434	0.996337	1.53692	3.02651	7.13349	5.71135	0.6018	0.459864
ENSG0000	C9orf78	9:1298272	161.95	141.204	239.987	205.659	244.476	149.622	199.581	181.393	197.089	158.59	0.5813	-0.20897
ENSG0000	RP11-88E	13:112686	1.58812	1.30124	0.925268	0.305969	0.446811	1.2111	1.48665	1.30577	2.5233	0.447461	0.32995	0.742929
ENSG0000	ARRB1	11:752641	2.42125	5.74938	5.63471	1.18027	4.37444	15.1288	2.34136	1.07712	1.3344	2.07471	0.91955	0.073232
ENSG0000	CWC22	2:1799448	25.1007	25.569	18.7692	14.2826	15.9587	10.5155	14.3826	13.2458	20.0259	21.065	0.3098	-0.38926
ENSG0000	CDC34	19:531711	46.022	45.5978	47.0704	22.1887	64.5181	54.6037	64.3139	27.7165	13.0106	34.6109	0.312	-0.36085
ENSG0000	KNSTRN	15:403827	5.46634	11.5037	8.34199	4.34579	4.53136	5.42811	13.3423	1.24889	4.21951	4.49338	0.6648	-0.27682
ENSG0000	NPRL2	3:5034732	47.5532	50.4663	72.8345	67.8879	58.5943	45.2163	39.5628	41.8966	46.3382	38.1723	0.2959	-0.54924
ENSG0000	UCKL1	20:639398	35.1584	33.6455	46.7853	55.8205	41.1542	24.4617	30.8519	46.3867	22.8108	41.9163	0.24775	-0.41858
ENSG0000	RP11-196	18:793958	1.30048	3.82313	0	0	0	5.44187	0.590588	0	0.714457	0	0.8544	0.491577
ENSG0000	GTF2IRD2	7:7479614	9.55316	15.6549	16.4997	14.3169	10.6409	17.2713	12.8246	18.7903	14.7088	9.77411	0.84495	0.210401
ENSG0000	SDCBP2-A	20:130990	4.96679	5.60943	2.39583	4.27045	2.05581	2.84993	4.85772	2.99183	10.1557	3.17056	0.7143	0.426572
ENSG0000	GCNT2	6:1049222	1.29456	2.44296	0.411465	2.77682	0.508872	2.32633	3.41294	1.47189	0	2.34384	0.8874	0.120915
ENSG0000	DNAAF2	14:496251	20.1544	13.6219	34.767	21.7999	19.621	33.1168	23.7614	17.741	17.2591	14.5542	0.74705	-0.12686
ENSG0000	KCNAB3	17:792185	0.934869	1.15494	0.825595	0.087779	0.313013	0	0.84094	0.364177	0	0.611058	0.42095	-0.86341

ENSG0000	NDEL1	17:841313	50.9085	56.6191	80.2238	49.8045	91.1821	25.8104	42.542	46.8691	44.3921	67.6803	0.05035	-0.58962
ENSG0000	MSR1	8:1610787	0.146482	0.398761	0.029405	0	0.042282	6.96486	0.443242	0.51602	0.598533	1.33385	0.0009	3.73019
ENSG0000	AL133243.	2:3235702	1.05007	1.60895	0.190809	0.811347	0.123311	1.6415	1.2419	0.894039	1.07982	2.13569	0.4723	0.937766
ENSG0000	TSKS	19:497397	0.575277	2.95525	1.09715	0	0.365303	0	2.06811	0.268258	0	0.319504	0.4421	-1.09892
ENSG0000	HCG4P7	6:2988729	2.23917	0.266764	0.486644	1.23208	0.68123	0.380224	1.59592	0	0.583144	0.40669	0.65655	-0.8693
ENSG0000	TMEM56-	1:9511733	1.68485	0.67902	0.329183	0.871621	0.223241	0.579168	0.783007	0.517441	0	0.249733	0.57205	-0.7461
ENSG0000	SMC1A	X:5337414	17.5773	18.3253	6.21489	3.68079	19.0033	20.6278	20.1764	6.93063	14.4284	31.6224	0.78195	0.170035
ENSG0000	RP3-510H	22:352988	0.740305	0.237728	0.136349	0.180239	0.136858	0	0.665864	0	0	0	0.54485	-1.21353
ENSG0000	ADPGK-A	15:727513	1.45556	0.834833	0.673407	1.3601	0.865422	0.028508	0.120255	0.983699	2.99322	0.6704	0.8423	-0.26048
ENSG0000	CLTA	9:3619085	82.499	82.4954	79.6298	45.8672	105.619	153.452	136.98	86.0301	56.7256	40.2968	0.6327	0.1783
ENSG0000	RP11-981	12:895611	1.19906	3.07701	0.488509	0.311415	0.087092	1.35902	0.798143	1.59835	2.59311	1.31385	0.61	0.688658
ENSG0000	ABC13-47	13:113953	0.736985	4.13109	1.09934	0.395022	0.734416	0.082078	0.030017	0.283244	0.07615	0.01495	0.0163	-3.64904
ENSG0000	SSRP1	11:573256	19.8832	17.8799	21.1652	17.9885	66.5421	22.7479	20.665	27.3784	29.5988	58.5762	0.64275	0.214123
ENSG0000	FAM50A	X:1544441	78.7976	48.0751	54.7658	45.2034	91.8382	114.883	73.8622	62.3339	29.4265	49.164	0.93885	-0.0261
ENSG0000	ARNTL2	12:273328	0.757953	1.23506	0	0.453034	0.00469	2.68344	2.46537	0.396067	0.539578	0.320693	0.18245	1.41971
ENSG0000	ZNF180	19:444744	5.49045	4.89472	3.73329	3.50207	2.87033	2.71424	5.93729	3.909	2.11615	3.09921	0.83175	-0.10683
ENSG0000	FANCB	X:1484340	1.99137	2.40935	1.26694	0.708231	0.821879	2.77158	0.55519	0.315067	0.159476	0.505928	0.59435	-0.69988
ENSG0000	IFITM4P	6:2975072	0	0	0	0.118583	0	0	0	0	1.50039	0.36106	0.2427	4.31078
ENSG0000	RP11-686	17:354642	1.6308	0.495672	0.948648	1.01607	1.80269	2.51586	0.76759	0.78695	0	1.76976	0.9931	0.000756
ENSG0000	FRG1HP	9:4099224	28.6707	25.4641	41.9875	29.2969	47.3322	34.5606	24.3375	44.0383	41.5707	50.0499	0.75105	0.180284
ENSG0000	NINL	20:254527	0.573657	1.03258	0	0.277912	0	1.69848	2.63525	1.08151	0.042336	1.14617	0.1163	1.99429
ENSG0000	RP11-535.	18:223478	0.92109	0.772278	2.31437	2.79575	2.12483	0	0	0	2.58554	1.1495	0.5422	-1.0894
ENSG0000	SYCE1L	16:771908	1.28702	1.86865	0.966488	1.66687	0.877347	2.49839	3.58982	0.808505	0.991429	0.10889	0.94195	0.081526
ENSG0000	LINC0096	3:1956580	28.0244	72.8007	18.596	14.0031	10.9096	24.0008	46.5866	21.9633	17.4774	24.112	0.87705	-0.05227
ENSG0000	RP11-603	15:222789	13.3115	3.72599	11.3663	5.73249	3.41581	20.372	0.706859	2.33907	8.23152	3.90495	0.87145	-0.01446
ENSG0000	MAP9	4:1551737	8.58236	4.70385	8.66186	4.29416	10.9235	5.93306	6.33513	3.55881	1.75082	12.1943	0.40505	-0.57725
ENSG0000	STARD4	5:1114960	5.79718	8.42809	2.22309	3.62222	1.66771	6.21177	8.21817	8.86451	5.18057	3.56193	0.15235	0.653481
ENSG0000	U2SURP	3:1429491	74.0622	81.9299	53.614	46.3205	100.899	76.2417	92.5388	46.3062	49.0472	96.1425	0.76305	-0.14727
ENSG0000	PI4KAP1	22:185336	4.41736	22.2656	5.05542	2.90757	2.6883	3.50604	17.347	8.25448	2.6263	8.14241	0.8989	0.099724
ENSG0000	NEAT1	11:654227	25.6664	48.9556	24.2909	18.2762	16.0575	113.769	80.6072	37.9202	26.6777	64.518	0.3262	1.26383
ENSG0000	TVP23C	17:154360	14.7158	8.82854	8.94784	13.0135	5.16567	13.4389	10.9093	11.5222	17.5457	5.5707	0.34095	0.670095
ENSG0000	WNT1	12:489784	4.20949	1.22347	8.45694	3.58614	7.94857	3.70719	2.18054	8.70624	1.87116	8.68591	0.83105	-0.15601
ENSG0000	MPZL2	11:118253	0.584439	4.09088	0.588471	1.58844	0.095185	2.68764	0.242317	0.746274	0	0.548543	0.47495	-0.75195
ENSG0000	RP11-468	14:241718	2.92221	1.63933	1.4754	2.39973	1.55747	1.0171	0.94653	1.53795	1.98936	2.17819	0.9814	-0.02741
ENSG0000	C19orf54	19:406937	4.36757	7.1657	1.82858	2.80553	1.97092	3.00337	2.67028	1.69721	3.74406	4.94519	0.89685	0.101636
ENSG0000	PSKH1	16:678932	10.6154	9.2656	2.23721	5.66182	5.43573	7.15056	8.85482	4.21178	3.31623	8.14621	0.95215	0.093493
ENSG0000	MT1G	16:566667	0	0	0.381745	0	0	20.0291	0	5.21053	0	0	0.331	5.65697
ENSG0000	MICU2	13:214926	70.4761	56.6682	52.2466	40.2873	38.7008	71.8622	70.8551	64.4772	54.5321	36.0426	0.45765	0.229188
ENSG0000	AC002306	19:195617	0.677022	0.348693	0.430357	1.19652	0.258404	0.645396	0.307602	0.415904	0	0.299253	0.5188	-0.86623
ENSG0000	CMSS1	3:9981783	16.6626	10.385	23.242	21.6746	21.0018	3.45194	25.6866	14.6854	7.17199	8.82397	0.22785	-0.76292
ENSG0000	SFXN4	10:119140	13.4697	11.2313	11.4761	10.1097	7.70177	22.9524	19.4007	19.1226	9.46977	5.36776	0.341	0.483371
ENSG0000	RP11-902	14:611875	5.57005	5.19286	6.76674	4.14744	3.78443	5.48753	7.10282	5.83376	8.71757	2.03966	0.87065	0.176417
ENSG0000	RASGRF2-	5:8094769	1.36184	0	0.583236	2.40651	0.297132	0	0.094781	0	0	0.352562	0.11375	-2.94096
ENSG0000	ZNF814	19:577696	10.8281	7.02519	10.1436	13.5661	9.25867	2.97546	5.51579	6.94419	11.2955	6.03606	0.3965	-0.69487
ENSG0000	AREL1	14:746534	10.4113	7.51957	3.24475	4.84373	4.21809	8.50153	5.8361	4.11356	2.24771	8.78786	0.76865	-0.10702
ENSG0000	NCF1B	7:7322062	5.41304	12.8124	5.85892	6.94456	5.40255	27.564	5.61773	7.40661	10.9014	4.87383	0.24125	0.813686
ENSG0000	CTB-25B1	19:144630	0.242072	1.62746	2.72246	0	1.8055	0.46192	0.56289	0.598512	2.54179	2.56092	0.9333	0.145661
ENSG0000	AP1M1	19:161975	94.5305	139.835	111.986	94.102	111.474	58.9914	94.3067	73.8292	58.1454	94.1743	0.0824	-0.58575
ENSG0000	TRGC2	7:3823957	255.814	62.0232	415.204	305.664	308.694	99.3764	187.661	248.022	211.836	207.668	0.14135	-0.54501
ENSG0000	BLOC1S5	6:7881516	24.5056	15.7691	23.0852	17.7646	17.3255	16.6671	21.8927	15.92	27.4954	21.2144	0.87935	0.100571
ENSG0000	LINC0117	7:3028430	0.237827	1.31481	0.730383	0	0.382884	1.73131	1.05714	0.8178	0.994222	1.66527	0.41825	1.27575
ENSG0000	RP11-84C	4:8911928	0.224546	0	0	0	0	0	1.4537	0	0	0.873994	0.19335	4.06455
ENSG0000	RP11-707	8:6564473	2.93692	2.25083	2.72613	1.26806	2.14491	0.229088	4.20029	10.395	4.71692	7.50121	0.32115	1.49971
ENSG0000	MRM2	7:2234230	4.23435	5.73291	41.6815	35.4919	39.9923	7.5074	3.35797	26.924	47.8077	30.551	0.7803	-0.13148
ENSG0000	TGFBR3L	19:791614	1.10557	0	1.30934	0.397093	2.27298	1.24312	6.24559	0.857857	1.25591	1.52184	0.52205	0.811528
ENSG0000	RP11-478	4:337813-	1.08345	0.35644	0	0.866691	0	0	1.7188	0	0	0	0.78255	-0.62967
ENSG0000	RP11-10N	8:9321330	0.984626	1.21075	0.412384	1.32058	0.200582	0	1.86287	1.98336	1.35923	1.3694	0.8109	0.413316
ENSG0000	AP1B1	22:293276	9.87199	10.1342	6.56187	8.0601	9.40993	12.4522	7.99083	12.0961	3.44646	18.2017	0.5881	0.222672
ENSG0000	BUB1B	15:401610	2.54991	3.64133	0.211877	0	0.110815	2.30455	3.62646	0.701157	0.591569	1.62226	0.35395	0.790461
ENSG0000	OSGEPL1-	2:1896742	0.110776	0.571683	0.320597	0.595666	0.174957	3.27424	0.484592	0.174546	1.2604	0.830696	0.1393	1.71725
ENSG0000	TMEM161	5:8818963	10.5121	8.41752	5.97608	16.3922	2.70845	10.2948	12.1573	6.73178	7.35601	6.27915	0.992	0.005129
ENSG0000	FABP3	1:3136562	0	0	0	0	0	4.48791	0.086664	0	0.707931	0	0.0098	inf
ENSG0000	BTD	3:1556070	9.71452	11.3791	11.9397	8.02234	13.9494	8.35561	9.02342	15.7126	8.78787	4.70073	0.6605	-0.2723
ENSG0000	ZBTB7A	19:404436	45.168	42.2265	26.3016	11.6155	61.5189	27.2823	59.2558	34.0605	19.1672	70.4656	0.78065	0.115138
ENSG0000	RPS27AP1	16:610553	6.21422	5.72638	9.76451	8.84839	2.93855	6.35296	8.14916	5.38375	8.47691	0.251864	0.868	-0.20391
ENSG0000	RABEPK	9:1252005	14.9886	15.4904	21.6183	19.092	15.5359	14.8112	16.3952	11.6821	16.6279	8.00373	0.42065	-0.36519
ENSG0000	PDE5A	4:1194547	0.55638	1.41897	0.497282	0.525019	0.592434	1.62957	0.441911	0.683297	2.00237	0.777964	0.5156	0.523285
ENSG0000	OTUD7A	15:314753	0.503365	0.500333	0.457053	0.985251	0.384198	0.564067	0.201936	0.479462	0.510076	1.60806	0.75655	-0.28618
ENSG0000	BOP1	8:1442621	40.7746	32.0389	29.9881	22.0787	33.2399	24.8881	41.6027	26.2678	20.3492	26.8882	0.53415	-0.22079
ENSG0000	LETM1	4:1811478	9.40994	9.96078	9.15457	6.14203	8.83155	8.02852	7.57988	8.71178	6.0597	13.2479	0.8288	-0.09448
ENSG0000	NLE1	17:351215	2.15426	6.22761	3.63805	1.41739	6.45351	1.91486	3.41326	4.21672	3.74775	7.28107	0.91435	0.0669
ENSG0000	NMB	15:846551	4.9677	3.39743	8.11184	8.16862	11.5229	9.38423	6.55932	10.0316	61.3795	1.38268	0.0452	1.46395
ENSG0000	CERCAM	9:1284117	1.27993	0.254793	0.667902	3.12752	1.34361	4.27961	1.62449	1.41976	0.03952	0	0.87005	-0.14441
ENSG0000	PRMT6	1:1070566	9.78405	9.03191	15.9022	14.7742	22.3705	12.5508	11.4446	13.0116	27.0942	26.4933	0.5508	0.235303
ENSG0000	TSSK6	19:195124	1.99449	1.41445	1.66423	0.541114	0.434065	0	4.96038	1.46309	3.8288	3.02801	0.4434	1.12166
ENSG0000	MIER1	1:6692489	23.0114	14.2146	14.4379	15.5012	52.0266	20.8064	31.8364	26.0055	19.8187	57.1437	0.62595	0.231105
ENSG0000	ACAP3	1:1292375	9.5412	12.1796	3.97303	6.76142	5.19906	7.84453	15.5802	8.19374	6.75062	8.18568	0.79465	0.230038
ENSG0000	IL15	4:1416365	5.2213	2.06435	2.23524	7.26093	3.99019	10.1109	0.747921	0.83081	2.37036	2.10783	0.59115	-0.41694
ENSG0000	C20orf27	20:375350	27.6359	26.9981	37.491	32.0137	55.8131	29.0334	27.1982	24.6358	17.7621	25.2763	0.12905	-0.57111
ENSG0000	RP11-466	11:167773	28.71	23.4987	10.9377	11.6738	3.88438	20.7616	27.5096	5.52026	4.54543	0.240473	0.7966	-0.43475
ENSG0000	ANKRD13	11:672885	44.9363	50.9773	26.3125	26.7046	46.474	36.638	64.811	27.8899	19.337	68.1897	0.57615	0.206742
ENSG0000	COLQ	3:1538127	76.7575	2.75757	53.2574	82.8651	45.4513	7.09104	0.492032	13.1815	25.3937	27.3927	0.0189	-1.81033
ENSG0000	EXOC2	6:485132-	23.315	24.2262	11.3785	12.3623	8.92453	9.77096	17.2116	15.4477	10.4284	13.4487	0.45065	-0.27553
ENSG0000	NAT1	8:1817047	6.50589	6.44106	6.1862	8.56841	4.0695	13.6803	5.88698	4.51963	9.83454	2.91757	0.70895	0.230897
ENSG0000	HSPH1	13:311349	40.991	50.145	19.5696	13.3208	29.4264	101.761	61.1032	35.7558	33.9642	62.2577	0.00895	0.885591
ENSG0000	LANCL1	2:2103247	12.4418	15.8062	4.05752	4.40453	2.99578	4.72454	6.49951	5.3471	0	6.22728	0.19605	-0.78019
ENSG0000	HOMER1	5:7937263	1.26615	0.489301	0.47997	0.195759	2.27051	1.05311	0.773793	0.554756	0.095331	2.02501	0.84965	-0.18931
ENSG0000	RAVER1	19:103048	6.77075	6.12967	3.12326	3.43308	8.28218	2.75361	4.06047	6.72906	2.35465	17.122	0.8967	-0.13546
ENSG0000	MKLN1-A	7:1309417	0.512172	1.71963	0.254628	0.965838	0.37322	0.593001	0.705201	1.47106	1.67297	0.37048	0.5996	0.451717
ENSG0000	IGLV7-43	22:223950	0	9.9613	2.1809	0	0.462033	0	0	0	0	0	0.00905	#NAME?
ENSG0000	CTLA4	2:2038677	23.1625	42.6401	2.22591	3.12565	0.389066	157.848	276.093	94.0821	28.8138	3.57396	0.00005	3.04474

ENSG0000(	LINC0095⁴	2:1986885	2.94941	9.58385	2.39501	2.62273	1.07759	5.95293	11.6818	1.53357	14.7028	3.24918	0.06545	1.08661
ENSG0000(	ZNF362	1:3325654	16.494	12.8663	8.87313	8.32412	30.1356	10.8071	7.27504	9.07677	2.03066	18.8114	0.2399	-0.87838
ENSG0000(	COPS5	8:6696409	93.1221	76.355	98.1458	96.6898	71.0638	85.696	94.9205	89.9851	69.8274	51.9098	0.783	-0.1231
ENSG0000(	SFPQ	1:3517637	119.947	145.389	49.8632	69.2228	77.248	98.5857	159.931	82.7796	62.0442	132.705	0.3834	0.316929
ENSG0000(	CST3	20:236267	11.9483	118.99	23.8056	11.0038	21.3609	107.761	8.16529	30.6235	53.1218	21.8424	0.36955	0.408957
ENSG0000(	TP53I13	17:295605	85.3475	44.1148	44.989	66.7162	34.1129	115.891	122.638	71.3401	80.5674	74.2754	0.2483	0.669023
ENSG0000(	METTL9	16:215972	61.9507	49.6801	65.5186	60.7956	69.7845	59.5705	83.3815	85.0772	81.2694	35.2186	0.83755	0.072972
ENSG0000(	CDC26	9:1132215	91.1827	68.7837	139.207	113.888	116.629	103.312	109.485	113.058	105.096	46.2238	0.70615	-0.18248
ENSG0000(	CFAP54	12:964895	0.163466	0.924597	0.076941	0.099005	0	0	1.68554	0.798835	0.831063	0.371295	0.20855	1.87984
ENSG0000(	AC018755	19:515945	3.90867	4.33854	2.18956	1.357	3.3516	0	2.73581	1.5141	5.26196	0.910285	0.82765	-0.44461
ENSG0000(	BAG4	8:3810524	4.6994	3.78118	5.68326	3.95221	9.73834	3.45258	3.72844	3.53441	5.30519	11.1917	0.8036	-0.27749
ENSG0000(	AP1G2	14:235117	231.359	277.284	92.0385	124.935	70.6452	183.282	197.798	81.8293	110.252	81.8393	0.4518	-0.22509
ENSG0000(	TNFRSF10	8:2308435	2.02737	0.456115	0.228984	0	0	1.35335	0	0.705906	0.133622	0.39119	0.9428	-0.09113
ENSG0000(	HNRNPLL	2:3856197	39.9308	27.3304	41.8995	28.338	23.1464	52.3473	61.8019	63.1466	49.9735	30.7776	0.12875	0.599223
ENSG0000(	AKAP9	7:9194086	95.2082	166.926	59.3908	61.4261	73.8239	174.863	152.946	46.1159	97.2647	158.083	0.3057	0.44549
ENSG0000(	SFR1	10:104122	12.96	6.31697	19.5854	9.63404	8.05772	6.61888	12.1023	9.53675	9.45511	3.61823	0.437	-0.43174
ENSG0000(	ITGB2	21:448859	678.948	649.468	537.247	464.639	748.988	472.446	305.515	286.222	204.641	494.143	0.059	-0.81695
ENSG0000(	SDHAP2	3:1956580	0.317368	0	0.461472	0.748153	1.30716	0.989369	0.205869	0.630689	2.92617	0.924576	0.54835	1.42767
ENSG0000(	C5AR1	19:472900	0.970049	2.30744	0.019988	0.048662	0.362729	3.99171	0.460979	0.175269	0.400185	0.875412	0.74875	0.702233
ENSG0000(	ABHD3	18:216508	63.9843	61.8023	45.6333	53.602	25.0428	68.2798	49.5438	60.0415	80.225	53.673	0.2295	0.378472
ENSG0000(	SSBP3	1:5413268	10.2923	11.7277	9.13872	6.06925	17.1885	3.17684	5.23146	5.36857	8.18655	9.98871	0.4529	-0.6889
ENSG0000(	ABCD4	14:742854	24.578	36.273	33.6618	25.2097	24.5661	26.7568	26.3371	20.3317	33.4964	33.4838	0.97095	-0.01118
ENSG0000(	RPL17-C1	18:494772	22.5084	13.4104	0.922923	1.01277	0.460335	8.57189	0.305637	0.239777	12.3609	0	0.99285	-0.01521
ENSG0000(	TMEM8B	9:3581295	1.43953	1.31081	1.08267	2.53606	1.13125	5.56773	3.61143	1.36243	1.13123	2.7245	0.41515	0.756171
ENSG0000(	ACTA2	10:888797	16.3255	7.04452	10.2754	8.02216	6.52593	13.2549	24.7784	22.848	10.3405	24.4033	0.31295	0.943818
ENSG0000(	SDF4	1:1216907	96.9335	100.446	107.915	102.154	115.432	123.402	175.199	146.361	117.188	97.0515	0.40585	0.26342
ENSG0000(	RYBP	3:7237182	1.08896	1.66124	0.952919	1.03483	1.93766	0.795437	0.661527	1.97634	1.28342	4.86291	0.5369	0.288847
ENSG0000(	RN7SKP18	7:1122065	1.68097	0.489751	0.167315	0.21609	0.141529	0.390486	0.953623	1.36662	0.235992	0.048924	0.88685	0.230802
ENSG0000(	RP11-345	1:1659324	0.605077	0.223516	1.5544	1.3943	0.603814	0.280964	0.349044	0.445616	1.52672	0.645511	0.42025	-1.59934
ENSG0000(	ATP5A1	18:460802	281.161	292.899	409.052	375.708	360.04	254.933	259.862	286.549	315.823	215.492	0.2895	-0.41443
ENSG0000(	RP1-313I6	6:2807879	5.15003	2.92174	3.66242	5.16923	0.878558	0.163696	1.83789	1.58231	1.45403	4.92298	0.7397	-0.6689
ENSG0000(	TYROBP	19:359044	95.7067	89.7903	191.394	136.288	101.664	347.408	18.5143	85.1273	93.0559	35.4988	0.6441	-0.18151
ENSG0000(	RP11-817	15:753467	4.72069	2.75079	3.66582	1.5731	2.41316	0	0.603608	2.08796	2.92517	1.32628	0.55925	-0.92261
ENSG0000(	TAB1	22:393997	1.38105	1.6414	1.64477	3.19549	2.29984	1.75398	1.24898	2.58606	1.61076	5.7842	0.56125	0.417583
ENSG0000(	CPSF3	2:9423567	26.3751	28.0789	18.2075	17.9474	12.7752	32.0883	17.4918	16.7418	10.7585	15.0768	0.5664	-0.18911
ENSG0000(	RIPK2	8:8975774	9.05228	12.0497	9.60581	10.8247	8.33949	14.9906	3.36115	3.52506	13.7946	3.57279	0.3801	-0.40745
ENSG0000(	APEH	3:4967400	83.193	75.6106	85.3497	92.7644	104.041	42.0005	57.9839	63.4934	71.9092	93.4263	0.3164	-0.4079
ENSG0000(	RP4-680D	1:1552681	0.343443	1.57134	0.97356	0	0.975628	0	0.808637	0	0	0	0.2357	-2.64144
ENSG0000(	SNX30	9:1127508	1.36735	2.36498	0.517375	0.235027	0.710416	0.803102	0.167221	1.59729	1.03955	1.44893	0.8116	-0.13524
ENSG0000(	NDUFAF2	5:6094512	26.8274	14.3575	35.4991	36.5018	31.3426	21.9474	36.3903	41.9712	9.96055	8.14211	0.546	-0.32948
ENSG0000(	DYRK4	12:456220	21.8632	12.1597	44.1692	41.6449	19.7226	9.16409	26.5834	29.9332	17.7987	9.3242	0.4848	-0.55597
ENSG0000(	UROS	10:125784	44.8841	33.3811	60.4554	74.293	55.8706	43.7011	33.4326	45.57	54.7241	16.5051	0.15785	-0.48998
ENSG0000(	CCDC125	5:6928017	2.01349	0.503569	0.860653	2.28121	2.90111	3.51631	1.40053	2.69144	3.59036	2.85241	0.48625	0.692731
ENSG0000(	TAX1BP3	17:366289	10.6852	20.8499	15.3248	14.732	17.3281	26.7268	24.1526	21.6955	12.7559	10.8611	0.8154	0.24771
ENSG0000(	U3	17:585198	7.42611	0	1.92474	0.443404	0	0	0	0	0.94325	0	0.38825	-2.95436
ENSG0000(	MVB12B	9:1263268	3.81732	1.31266	4.43771	4.10708	4.89367	6.36272	8.96971	11.2521	7.16335	14.8918	0.00475	1.21955
ENSG0000(	APTX	9:3297260	22.7659	19.7017	31.2062	36.5306	15.4293	29.1914	20.3286	22.7488	24.7725	16.2747	0.6952	-0.15405
ENSG0000(	CNOT2	12:697386	51.231	58.0202	38.5444	53.5416	42.534	49.8288	45.0465	39.354	41.0017	42.0874	0.76055	-0.09217
ENSG0000(	LCMT2	15:433236	1.00495	1.59903	2.09319	0.99944	1.03391	3.16662	3.45399	2.5118	0.020213	2.54013	0.58775	0.545127
ENSG0000(	RP11-98J2	5:6987527	4.72077	11.7719	2.70583	2.31358	2.0549	3.77486	3.47752	1.1925	2.24408	4.08835	0.8071	-0.58075
ENSG0000(	RRAS2	11:142779	59.3221	12.3	30.7181	52.0231	43.3325	29.826	31.2729	14.0925	24.5342	18.0267	0.06215	-0.75068
ENSG0000(	ST14	11:130159	0.59679	3.67396	0.131254	0.050038	0.099254	10.0787	0	1.32938	0	0.037747	0.62885	1.42074
ENSG0000(	AKIRIN2	6:8767507	54.0272	48.7573	50.5592	40.4735	54.6673	43.1269	77.6318	62.7609	68.7709	59.8716	0.3192	0.327175
ENSG0000(	PFN1	17:494565	3758.03	4079.16	5013.47	4658	4525.94	5123.27	4026.27	4021.4	4405.62	1855.56	0.72895	-0.20985
ENSG0000(	SMIM15	5:6115770	20.6971	13.6086	10.6631	9.53053	10.4412	22.3017	20.6293	15.0677	14.046	13.55	0.33665	0.36709
ENSG0000(	RP11-117	1:5336666	1.35449	5.06605	2.37827	1.26505	2.38775	5.63754	0	0	3.94376	3.65398	0.79565	0.136864
ENSG0000(	LRP10	14:228716	25.4562	24.8843	13.5058	11.3425	22.8537	18.7143	27.9026	34.0455	8.50901	67.2639	0.462	0.289399
ENSG0000(	RPS6KC1	1:2130512	5.58516	9.31764	2.25888	1.85441	1.33094	7.07478	3.87745	3.20012	0.223162	6.3042	0.8317	0.118406
ENSG0000(	MST1	3:4967400	2.17165	2.3003	0.08703	1.8984	0.910049	0.367717	0.674731	0	1.2898	1.15942	0.2034	-1.04821
ENSG0000(	CAND2	3:1279647	0.382658	0.908137	0.184419	0.819851	0.147104	0	0.51188	0.342245	0.104437	0.561413	0.60265	-0.80778
ENSG0000(	FAAP20	1:2050469	48.3879	46.7681	57.1818	60.1634	54.7917	53.7885	70.0845	60.3899	52.8213	18.3216	0.76355	-0.16443
ENSG0000(	RP5-884G	1:9996838	0.771689	0.89254	0.854011	0	0.234956	0	0	0.612821	0	0	0.4035	-1.99371
ENSG0000(	CALCOCC	17:488309	63.9223	78.8137	54.9219	53.4361	51.7529	84.4677	95.5588	61.2403	73.0917	71.751	0.50525	0.229732
ENSG0000(	DGCR11	22:190362	0.458655	0.919724	0.437034	0.361268	0.223759	0	0.304334	0	1.30693	0.692687	0.98585	0.024447
ENSG0000(	RP11-197	12:122920	3.79222	5.25214	2.53302	1.65318	1.24896	3.58775	2.92143	4.43898	0	6.85206	0.894	-0.25068
ENSG0000(	AKAP10	17:199043	26.4311	34.3884	7.30449	10.4928	5.24799	23.7645	15.1266	8.37987	11.0849	11.0167	0.4632	-0.32811
ENSG0000(	TNFSF13B	13:108251	11.9002	8.47343	16.757	17.1684	16.6736	64.281	16.5929	35.3888	44.8311	37.4015	0.0014	1.41591
ENSG0000(	IL10RB-AS	21:332299	1.68357	3.72478	0.342975	0.001286	0.622911	3.54871	0.514712	0.241229	1.10794	1.54796	0.73425	0.512223
ENSG0000(	MCRIP1	17:818223	69.6152	69.2593	69.8875	73.417	62.1632	88.2577	119.329	112.919	66.7211	39.1244	0.45885	0.251311
ENSG0000(	UTP23	8:1166421	22.4924	19.4625	27.936	24.4444	44.8056	27.405	29.0283	17.1844	40.408	30.2634	0.7324	-0.30541
ENSG0000(	PML	15:739946	25.0395	31.8719	8.00639	7.20659	13.4574	25.6777	40.835	13.7085	7.05163	23.8175	0.5021	0.294046
ENSG0000(	MOAP1	14:931821	40.3802	45.5038	72.6413	105.073	42.2125	22.1792	27.5496	44.5614	72.4584	41.2258	0.13545	-0.53623
ENSG0000(	RAB22A	20:583096	13.7092	10.7896	12.346	9.61038	11.3697	12.7821	12.6675	13.7944	7.96338	8.59677	0.7285	-0.10792
ENSG0000(	WHAMMF	15:287019	1.03656	8.0498	0.917695	0.416675	0.211079	0	0	0.408513	0	0.569962	0.11365	-3.48992
ENSG0000(	CCT6A	7:5605162	102.553	99.0053	86.9551	95.2605	91.4761	129.14	107.291	82.5122	66.7303	61.63	0.745	-0.09431
ENSG0000(	RP11-763	1:1444181	0.785412	0.642362	3.25753	1.39264	1.46056	0.370439	2.9909	4.27053	3.97756	2.77089	0.3672	-1.97424
ENSG0000(	RP11-677	11:124766	0.526549	1.76238	0.281929	0.425995	0.188587	0	0.251704	0.167408	0	0.827612	0.3256	-1.3948
ENSG0000(	HSPA5	9:1252348	253.641	126.981	198.74	233.518	152.767	217.516	207.929	145.6	143.773	161.666	0.7007	-0.1421
ENSG0000(	TBX1	22:197567	0	0.033141	0.132963	0	0.048792	0.511391	1.14474	0.780711	0	0.372319	0.00755	3.65903
ENSG0000(	POMGNT1	1:4618868	20.5431	24.3032	13.6599	9.00538	13.9109	23.0867	13.8156	13.9752	8.35823	14.4096	0.7213	-0.12961
ENSG0000(	SOS1-IT1	2:3898139	0.940279	0	0.299953	0	0.52842	0	0.759328	1.84663	0	0.777991	0.545	1.00743
ENSG0000(	SELENOK	3:5388440	143.88	62.1105	256.062	321.747	123.662	152.434	203.672	224.222	292.826	40.309	0.7942	0.113801
ENSG0000(	MSH3	5:8062622	10.0169	13.2357	3.38161	6.06308	5.77309	9.68916	10.1453	3.45576	6.30102	11.6838	0.72995	0.163724
ENSG0000(	RP11-305	12:651695	3.38415	2.62807	2.76075	7.05306	1.33063	1.17565	0.06935	2.44974	4.14684	1.82891	0.6168	-0.76713
ENSG0000(	C20orf197	20:600559	1.04959	0.934888	0.194099	2.24539	0.423314	0	0	0	0	0.745906	0.01875	-3.22037
ENSG0000(	SLC25A3	12:985935	126.449	118.401	135.685	156.751	134.256	132.859	206.649	178.981	117.063	94.1511	0.81	0.079943
ENSG0000(	TGIF2-C2(	20:365734	0.541405	0	0.735872	1.38351	1.63562	0	1.87516	0.304215	0	0	0.7034	-0.73969
ENSG0000(	STARD10	11:726850	14.5802	8.49829	6.62779	14.3772	8.61276	8.82933	16.6397	8.99954	5.07644	3.3727	0.6828	-0.43521
ENSG0000(	NCLN	19:318556	16.7729	15.1421	12.0255	16.3461	19.452	16.0878	37.1992	18.555	18.3118	24.2136	0.374	0.382846
ENSG0000(	HBQ1	16:180452	0.22522	0	0	0.845817	1.24931	0	0	0	0	0	0.0057	#NAME?
ENSG0000(	IGFBP3	7:4591224	0.773511	0	0.78924	0	4.27136	0	0	0.691221	0	0.324687	0.11515	-3.06395

ENSG0000(	ZNF526	19:422202	3.3243	4.95058	3.13483	1.40536	2.90159	2.30548	3.14745	3.18622	0	5.54421	0.6455	-0.2947
ENSG0000(	EIF2B4	2:2732584	52.2276	48.4108	41.7573	61.3329	36.6151	49.5474	52.2094	37.1064	56.399	33.1199	0.9573	-0.02485
ENSG0000(	BNIP1	5:1731441	11.8713	15.8163	18.4065	19.8483	12.783	5.8809	19.6118	11.2097	22.5418	5.23802	0.59105	-0.28703
ENSG0000(	BCAS4	20:507948	7.09903	12.355	14.8057	35.3797	8.93699	13.8775	20.4227	25.9289	8.48509	5.43658	0.7356	-0.16835
ENSG0000(	FBXO31	16:870835	8.49005	9.38614	12.1398	7.73134	11.303	5.89444	19.6641	6.1092	4.81487	22.5921	0.8361	-0.18135
ENSG0000(	ATP5L	11:118359	191.635	125.35	348.829	308.804	133.167	259.527	263.885	273.219	267.298	34.5115	0.90345	-0.07404
ENSG0000(	SNU13	22:416739	115.673	134.71	205.263	190.165	145.187	139.047	153.784	143.919	141.979	43.8843	0.2085	-0.42209
ENSG0000(	RP11-452	16:314567	2.19038	3.13894	1.01731	0.631306	1.04523	1.04236	1.62957	0.193904	2.20095	2.73377	0.87115	0.231493
ENSG0000(	NEURL1	10:103479	0.767041	1.42362	0.974492	2.04487	1.07332	2.52363	1.58413	0.490452	1.47573	1.12929	0.88055	0.117305
ENSG0000(	RP11-6N1	17:479415	4.56639	2.64802	5.34922	2.47888	0.914661	4.59006	0.695407	3.10811	3.13506	0.312245	0.9697	-0.04216
ENSG0000(	TGS1	8:5569642	9.93165	12.0525	11.3748	14.8325	18.1605	11.412	10.8607	11.46	10.7184	19.1848	0.78245	-0.12053
ENSG0000(	PPP1R11	6:3006670	77.4544	63.81	61.4927	56.1148	66.4275	70.2529	68.831	64.437	48.7961	81.9858	0.97485	0.010592
ENSG0000(	PRKAR2A	3:4874459	7.0584	7.91258	7.87861	4.46253	5.225	5.03144	8.7211	3.08439	5.9112	15.3158	0.9927	-0.0054
ENSG0000(	MRPL9	1:1517555	71.9527	59.3281	86.1608	87.075	74.3725	97.1432	79.7475	66.4664	60.7332	39.5364	0.63695	-0.17529
ENSG0000(	HOXB7	17:486072	0.179862	0	0.734833	0.345675	0.664181	1.79011	1.77392	0.148046	0	0	0.5998	0.709842
ENSG0000(	USP6NL	10:114539	0.48267	2.29197	0.266325	0.636126	0.392378	2.84386	0.21239	0	0.041105	0.128053	0.6866	-0.46793
ENSG0000(	ARFGEF1	8:6696409	14.3133	18.6296	5.65718	3.07836	9.11073	17.064	11.2505	5.44795	8.99797	16.0847	0.6531	0.372587
ENSG0000(	SLC2A3	12:791922	66.0015	47.681	60.5342	95.279	29.5459	70.7913	64.3161	83.0282	100.412	82.3059	0.38325	0.29799
ENSG0000(	TRAPPC2L	16:888137	49.1755	45.5218	68.5025	86.3248	40.1551	75.1792	66.1108	62.9578	52.4107	16.9445	0.7808	-0.13502
ENSG0000(	GMEB2	20:635876	6.6058	7.22215	4.18392	2.16223	3.08665	5.53311	1.39542	4.17192	4.7876	6.394	0.937	-0.04312
ENSG0000(	RPGR	X:3734927	9.64499	2.90772	3.89648	4.48814	2.0068	8.63505	13.6279	5.59063	0.52952	7.32146	0.49675	0.64171
ENSG0000(	SMC5-AS:	9:7003392	0.412457	0	0.145719	0.308738	0.050024	0	0.987479	0	2.75618	0.062059	0.4461	2.17727
ENSG0000(	GHDC	17:421887	8.28631	3.42176	0.420587	0.381076	1.41787	6.65948	6.49814	2.42692	2.21838	2.47886	0.3182	0.58423
ENSG0000(	RP2	X:4683693	5.35371	5.46346	2.38304	6.74147	1.76562	6.66809	3.6859	4.12484	1.56541	2.98671	0.67305	-0.21317
ENSG0000(	SAP30	4:1733637	16.9315	19.352	1.97372	0.939057	1.89471	32.0392	25.4311	6.86914	5.04872	1.85028	0.28345	0.910735
ENSG0000(	DUSP18	22:306356	5.98708	7.70939	10.0947	3.78803	6.64034	5.28089	11.6851	14.6458	12.3147	16.9492	0.13105	0.742307
ENSG0000(	RP4-751H	7:1498383	1.25934	2.89362	1.03105	0.756013	1.56992	0	0.385152	0.61762	0	2.39167	0.53275	-0.93197
ENSG0000(	TTI1	20:379830	17.3224	14.4123	7.28367	7.29051	4.57156	18.0853	4.15232	7.98014	7.39205	7.63926	0.67205	-0.16366
ENSG0000(	PCOLCE	7:1005722	8.71586	2.507	6.47108	5.81869	5.5992	3.34896	4.20276	2.12276	4.19794	5.45655	0.319	-0.70729
ENSG0000(	CTD-3185	15:663868	1.75152	1.72025	3.30469	4.66497	2.51694	1.38599	0.37257	2.31251	0.045332	1.97815	0.40345	-1.31791
ENSG0000(	8-Sep	5:1327474	1.60295	2.55843	2.05041	2.06188	4.00338	2.06436	0.502341	3.95734	2.80657	5.31739	0.6172	0.375121
ENSG0000(	AC006129	19:415451	143.709	145.062	208.773	220.486	114.936	159.584	95.908	149.56	150.266	73.5727	0.3901	-0.34677
ENSG0000(	NCBP2-AS	3:1969426	78.0998	63.384	174.601	213.613	91.1481	91.2611	77.8212	101.832	58.5959	51.3399	0.0285	-0.79455
ENSG0000(	RP11-468	14:241433	2.57752	4.07863	0.56279	0.999605	0.372063	3.2551	2.24061	1.21795	0.571041	2.56221	0.52235	0.925431
ENSG0000(	TSTD3	6:9943267	5.55204	0.685768	5.43209	6.997	5.1225	4.74008	4.04825	4.97425	3.62224	2.7782	0.89505	-0.18504
ENSG0000(	PIAS1	15:680541	34.479	43.9938	61.4343	29.074	37.4227	31.515	41.1762	30.2445	22.2293	68.085	0.17295	-0.50514
ENSG0000(	DZIP3	3:1085896	14.1926	9.38777	9.45969	5.48101	5.53622	5.1312	16.9882	18.7564	15.0227	12.3785	0.24495	0.538974
ENSG0000(	RP11-761	3:4076154	1.73235	2.2021	3.19431	2.04256	0	2.09066	1.87915	0.314717	0	0	0.4588	-1.24333
ENSG0000(	RP11-452	2:1644928	5.00681	3.00028	0.669413	0.551713	0.211667	5.13578	4.48019	0.229249	0.304657	0	0.90935	0.201613
ENSG0000(	SLX4IP	20:104353	1.8578	0.951286	1.44198	1.01178	1.51834	3.18699	1.77093	1.4795	1.77057	2.73536	0.268	0.59683
ENSG0000(	C15orf53	15:386965	1.60989	3.84846	0	1.13526	0	10.948	2.64907	1.78128	4.80612	2.36103	0.2716	1.86568
ENSG0000(	AC017116	7:4404463	0.330945	0.805909	0.493764	2.14325	1.27861	0.779981	0.804904	1.50351	1.19236	2.41928	0.72375	0.404626
ENSG0000(	MED8	1:4338391	35.2463	32.3553	41.7529	35.9523	37.2918	53.4099	39.3555	48.6546	32.388	22.3715	0.88915	0.05339
ENSG0000(	CMTM5	14:233768	0	0	0.74214	2.52724	0.137341	0	0	0	0	0	0.00645	#NAME?
ENSG0000(	RPS10P3	9:8801629	0.307302	0.39725	1.66314	1.78503	0.396402	0.369953	0.686281	0.869315	1.49277	0	0.9296	-0.14066
ENSG0000(	MAN1A2	1:1173674	6.90656	5.0152	19.7729	15.4001	18.9764	5.17082	11.4539	14.4705	14.5019	27.7515	0.924	-0.09453
ENSG0000(	FAM43A	3:1946865	22.147	9.18082	6.82445	12.44	14.1134	8.08022	1.67575	7.42033	10.1408	14.4136	0.33315	-0.43566
ENSG0000(	SLC35E2	1:1702729	10.7307	9.6703	7.71338	8.24811	5.54732	5.22173	4.7191	5.37804	17.5886	4.02167	0.846	0.21785
ENSG0000(	GKAP1	9:8373942	5.56551	9.50967	2.01196	1.10927	4.6478	1.76868	1.3413	1.20431	0.910128	1.86637	0.1643	-1.47008
ENSG0000(	ZNF518B	4:1043987	5.69939	6.15853	2.01567	1.49988	2.10084	5.55455	3.42427	3.30145	3.4973	5.81175	0.556	0.321209
ENSG0000(	ARF4-AS1	3:5757136	0.774301	0.356607	1.42226	3.10502	0.862276	2.42872	1.21276	0.972625	0.900168	0	0.6987	-0.46393
ENSG0000(	RP4-734P	20:246163	0.143889	0.638305	0.115303	0	0.365975	0.144708	0.573776	0.311786	0	0	0.7985	-0.53883
ENSG0000(	TMEM50B	21:334028	38.2281	36.6262	30.885	40.9722	16.6873	39.8387	39.2179	30.2418	53.6708	26.0807	0.5412	0.243388
ENSG0000(	PAQR7	1:2585961	0.151309	0.037645	1.20159	0.462573	0.503009	0.038199	0	0.465029	0	0.524724	0.53705	-1.44207
ENSG0000(	RAMP1	2:2378588	0	0.152621	1.30066	3.11598	0.258895	0	0.8908	1.57639	1.87145	0	0.97205	-0.05312
ENSG0000(	PPIL4	6:1495047	21.823	20.6553	12.5034	10.3983	18.9709	25.469	35.372	17.552	11.7748	19.1502	0.22235	0.44189
ENSG0000(	FCHSD1	5:1416369	16.4051	10.0427	6.97474	12.7546	7.0283	14.3488	11.1585	11.843	12.2659	12.8299	0.7721	0.147324
ENSG0000(	DENND1A	9:1233561	3.55218	2.71645	2.31208	2.18415	6.53384	4.43917	2.89259	3.04725	9.17787	16.1228	0.1602	0.751713
ENSG0000(	EMC6	17:366289	71.0789	39.9759	106.772	95.5142	103.211	84.0142	103.155	98.243	56.0829	24.429	0.63925	-0.33456
ENSG0000(	PLA2G4C	19:480478	0.545854	1.89641	1.65194	5.71907	3.69911	8.97622	4.17387	0	5.19827	3.25153	0.26185	0.772434
ENSG0000(	IFIT1	10:892135	0.773354	13.613	5.14287	7.07952	3.36232	13.037	166.735	69.8664	3.34539	6.3671	0.13255	3.15796
ENSG0000(	POLR3B	12:106357	2.83827	3.08035	1.35373	0.860405	0.858931	1.32846	1.51851	1.50279	0.052413	0.907877	0.5042	-0.72354
ENSG0000(	TRAV8-2	14:218465	15.0204	44.0884	29.628	18.3924	14.7218	32.7498	50.9671	27.5064	38.5908	12.3587	0.3907	0.416903
ENSG0000(	RP11-79C	2:1990202	0.49264	1.04043	0.747747	0.858535	0.091362	0	0	0.313645	0	0.35947	0.3128	-1.96534
ENSG0000(	ITFG2	12:194605	42.0367	57.5813	41.8016	41.9558	19.5511	45.2827	39.4384	25.9019	30.2502	37.2082	0.76995	-0.13595
ENSG0000(	KDM4A	1:4365015	5.55439	6.84704	3.00481	2.73151	3.43011	2.40005	3.95329	6.20187	6.45504	9.07366	0.36465	0.409369
ENSG0000(	NHEJ1	2:2190753	11.7655	22.9417	27.2665	17.0526	39.137	14.8088	16.3	12.1083	16.0628	38.4241	0.44625	-0.336
ENSG0000(	CFH	1:1966518	1.09334	0	1.28897	3.16957	2.54672	1.86989	0	0.169982	0	1.76472	0.3759	-1.54974
ENSG0000(	IGSF8	1:1600913	36.5788	43.4543	16.9956	26.121	18.6224	18.0067	25.5914	16.8188	11.3153	19.2368	0.08395	-0.64977
ENSG0000(	XXbac-BP	6:3023403	3.29453	3.40787	1.06631	0.583099	1.13689	4.50029	0.770674	1.5325	0	2.31681	0.9866	-0.00938
ENSG0000(	ABLIM2	4:7965309	0.893605	0.057987	1.08701	1.03842	1.22413	0	0	0	0	0.121011	0.22215	-5.39123
ENSG0000(	DCAF4	14:729263	11.8613	6.91165	7.74702	12.5865	13.0182	23.5528	10.7506	8.94549	7.6714	18.0261	0.62845	0.243406
ENSG0000(	EXOC5	14:572005	17.0943	19.3757	7.6729	5.2754	11.2611	32.2555	18.4173	9.96692	14.5823	15.6238	0.72595	0.21604
ENSG0000(	STX11	6:1441505	29.0435	28.9622	18.1098	40.302	33.8297	34.1939	38.0513	40.3226	23.3754	20.6909	0.963	0.016302
ENSG0000(	FAAP24	19:329722	4.47783	6.05577	4.35982	1.06597	2.8605	9.08076	1.26673	4.3408	0	2.97691	0.81305	-0.22165
ENSG0000(	SIKE1	1:1147694	26.6927	21.9173	27.3131	22.5639	27.8137	31.8879	38.2146	30.8271	33.8271	23.8053	0.42585	0.31489
ENSG0000(	AMACR	5:3398617	5.48326	2.03802	6.06646	5.34609	5.16324	13.5057	2.66321	2.34246	2.05498	3.09924	0.57625	-0.46565
ENSG0000(	KHDRBS3	8:1354574	0	0	0	0	0	0	0	0.262482	1.28004	0.821221	0.00545	inf
ENSG0000(	OTULIN	5:1466466	10.1959	8.95637	14.877	12.6366	18.188	22.3941	32.2712	20.804	27.8972	30.8799	0.18655	0.884756
ENSG0000(	LINC0146	12:626017	8.49884	1.93684	4.24696	3.22382	4.51245	7.54298	4.71269	3.77232	3.0206	0.650983	0.80555	-0.36593
ENSG0000(	AC072062	2:2148102	0.871512	0	0.017571	0	0.058266	2.99813	0	0	0	0.111736	0.3268	2.02028
ENSG0000(	HMGXB4	22:352574	7.96622	12.3326	9.47844	8.71596	18.4131	15.1325	17.7295	12.2117	26.3598	29.1764	0.2069	0.735223
ENSG0000(	BAX	19:489548	101.568	52.7	37.2628	89.5204	37.0408	154.788	84.6137	73.8545	76.4766	33.554	0.36375	0.364652
ENSG0000(	GSTM2	1:1096560	16.5325	46.6923	15.0195	6.71617	9.68671	40.394	11.2154	9.93708	8.09126	8.3325	0.80165	-0.29861
ENSG0000(	FRMD5	15:438707	0.494975	1.57317	0.068086	0	0.072364	2.41468	0.472272	0.603109	0	0.05249	0.531	0.644248
ENSG0000(	SNX19	11:130875	15.3588	15.0886	4.99212	8.67635	4.19622	4.79003	5.82768	7.33615	4.02339	10.3089	0.11215	-0.65792
ENSG0000(	ARSJ	4:1139002	3.19271	0	2.0918	1.19847	0.769384	0	0.632676	1.58131	0.173977	2.32611	0.38405	-1.11752
ENSG0000(	CCDC30	1:4243032	2.53114	1.71096	1.00656	1.10997	0.649816	1.32696	0.595015	0.724619	3.97118	1.22417	0.86125	0.159342
ENSG0000(	PRTFDC1	10:248486	1.38309	0.626047	1.46804	0	0.0815	0	1.22393	0.038768	0	1.04846	0.64995	-0.61799
ENSG0000(	NUP50	22:451638	38.6418	38.9749	13.9587	11.4544	15.878	25.5529	24.7317	21.9651	13.6385	29.4763	0.9473	0.020995
ENSG0000(	C15orf40	15:828369	28.6743	26.142	49.348	35.5861	37.2625	35.8429	29.5484	34.1538	46.3015	13.0201	0.70645	-0.2275

ENSG000C	TET1	10:685606	3.68357	2.30596	0.585279	0.325075	0.790976	2.91035	2.12601	0.271135	0.044574	1.55146	0.81605	-0.1243
ENSG000C	VOPP1	7:5543605	13.4889	25.6316	51.1414	32.4564	102.52	15.6694	27.4969	75.5858	30.8285	156.471	0.9809	-0.01419
ENSG000C	ALG14	1:9497440	3.1227	4.17522	0.962872	2.14161	1.28236	0.555091	1.68211	0.613016	1.03248	0.427264	0.02365	-1.91404
ENSG000C	FAM171A1	10:152116	5.5836	6.19477	4.17054	3.94569	3.09117	0	0	1.33371	0	1.8148	0.1258	-2.99991
ENSG000C	NIT1	1:1611005	10.0718	19.6425	17.3193	22.9067	19.0012	17.7027	8.90693	19.0311	12.2505	17.5206	0.64075	-0.2927
ENSG000C	AC002398	19:357180	0.539237	0.282328	0.677911	0.48322	0.328938	0.640789	0.41203	0.748146	0.343248	0.529052	0.90905	0.129177
ENSG000C	CENPV	17:159765	16.3833	10.5082	20.3473	24.5871	14.4473	15.7291	10.7291	15.0728	11.2573	8.37073	0.52135	-0.5183
ENSG000C	PSMC3IP	17:425670	3.15596	3.65359	3.49614	2.01672	2.24634	7.85785	7.02395	5.9953	0	3.31463	0.4261	0.709141
ENSG000C	PGRMC1	X:1192362	16.496	15.1307	14.779	20.6107	13.1464	16.2425	13.5809	16.7109	20.3276	9.98435	0.8663	-0.06628
ENSG000C	ZNF404	19:438723	2.79458	2.46555	0.45713	1.20273	0.41979	0.044384	1.34826	1.25098	0	1.85151	0.4931	-0.86096
ENSG000C	OTUD3	1:1988251	0.84397	0.787887	0.586	0.779799	0.601543	0.577231	0.921081	0.757817	0.007752	1.70984	0.4812	0.639769
ENSG000C	MBLAC1	7:1000927	2.68782	1.81271	7.83265	1.99796	3.59865	7.0111	3.14367	4.84523	6.63405	3.11448	0.70525	0.45353
ENSG000C	ELP4	11:315097	20.5628	23.6622	20.2324	19.1271	14.4518	17.0945	13.1241	9.0594	6.92579	4.9223	0.03015	-0.96105
ENSG000C	TNFRSF8	1:1206337	0.933355	3.10318	0	0.103436	0	0.288773	21.9708	0.039896	0	1.0349	0.3299	2.43366
ENSG000C	RP11-93H	13:526513	2.26609	0.977495	1.14172	6.35044	1.16675	0.302977	9.44568	4.02041	9.77117	2.23055	0.12555	1.21011
ENSG000C	RP11-521	14:105209	0.3448	0.431262	0.255305	0	0.109838	1.33284	0.016388	0.140098	0	1.31326	0.50715	1.24176
ENSG000C	RP1-193H	17:695513	0.729129	1.523	0.083507	0.706729	0.513215	3.05516	0.769504	0.920156	0	1.65887	0.27385	0.768175
ENSG000C	MSI2	17:572558	2.80373	4.23584	3.99027	1.50548	5.75956	5.82557	5.10401	9.19487	6.02512	6.70064	0.14235	0.794972
ENSG000C	COMMD8	4:4745079	80.774	31.3962	64.6988	86.0856	41.2016	83.7623	97.9059	69.084	96.8011	10.7271	0.3355	0.312282
ENSG000C	CTD-2116	15:641655	0.331446	2.97187	0	0	0.108699	0.691295	1.09304	0.193379	0.392804	0.961442	0.966	-0.06228
ENSG000C	CEP164	11:117232	17.3171	17.4091	17.483	9.6699	24.1678	11.9997	24.6278	11.1488	13.1943	20.4408	0.8338	-0.19909
ENSG000C	DCK	4:7090232	100.279	91.3119	43.4477	70.1046	29.8003	64.8215	62.6003	48.9071	52.4109	39.2298	0.4903	-0.23193
ENSG000C	LGALS1	22:376756	145.264	151.388	30.2772	69.4401	37.1852	315.503	295.324	194.349	210.413	40.6388	0.00065	1.28412
ENSG000C	CTD-3131	19:174687	3.72726	4.08694	3.8906	5.44882	3.26962	9.0372	4.92649	4.91023	2.91875	6.78639	0.69405	0.345655
ENSG000C	CTC-444N	19:572613	2.27025	1.59645	1.06376	1.39776	2.59088	7.97369	2.29184	2.67067	0	4.61442	0.2476	0.914294
ENSG000C	ZNF281	1:2002534	11.2194	16.2031	7.01818	6.96473	6.78449	21.4325	8.99837	8.42015	8.13766	16.5046	0.66655	0.170173
ENSG000C	GOLGA7B	10:978502	0.135122	0.628184	0.222527	1.13856	0.28862	0.142112	0.579821	0.631298	0.884287	2.00432	0.3268	0.931548
ENSG000C	TFRC	3:1960271	14.8355	23.8366	3.1427	5.30709	1.68224	32.5514	27.9193	12.6777	24.9926	8.51295	0.00185	1.3092
ENSG000C	HMGN3	6:7920124	159.828	140.216	166.875	148.059	128.542	141.551	147.574	107.378	84.362	44.9726	0.13845	-0.51846
ENSG000C	ATP6V1A	3:1137468	13.2983	12.9899	12.6931	12.1606	19.7404	33.3478	16.6426	16.4668	8.72135	35.1052	0.3745	0.352984
ENSG000C	OASL	12:121019	9.41472	10.7589	16.3916	15.4146	14.2191	61.6923	244.487	101.848	38.3311	43.1353	0.00005	2.68381
ENSG000C	POU2F2	19:420861	8.63925	14.364	4.41715	4.27515	4.93931	8.66452	9.83016	6.39677	5.01784	10.7147	0.84265	0.102063
ENSG000C	PLCB2	15:402781	50.2794	70.5614	18.5746	14.7355	26.8774	50.5169	25.9042	25.7461	12.1211	49.6042	0.8842	0.06517
ENSG000C	MPP5	14:672411	0.80994	0.362244	0.722122	1.4559	0.742147	0.043266	0.404213	1.53079	0.75494	1.8508	0.96335	-0.05079
ENSG000C	ARGLU1	13:106541	142.413	184.581	64.7697	63.8946	54.3425	120.062	124.653	86.8708	58.6784	118.619	0.79315	-0.11281
ENSG000C	ZNF771	16:304072	6.17758	2.73325	7.14192	2.04729	5.71857	4.24973	6.67402	2.68211	4.00071	5.66648	0.8153	-0.26797
ENSG000C	FBXO41	2:7325468	1.90031	2.23174	0.201332	0.844204	0.795947	1.6982	2.2988	0.872161	0.184986	1.49551	0.78355	0.16998
ENSG000C	ZSCAN26	6:2826700	18.0019	17.7237	20.4997	12.631	13.842	12.7563	12.5535	10.0828	7.40333	13.2853	0.16115	-0.63369
ENSG000C	ITGAE	17:371462	60.0977	45.5742	76.1671	72.5816	29.7376	128.939	120.803	132.361	150.789	99.4024	0.00955	1.17191
ENSG000C	RPL10P19	8:8961199	1.54732	0.664509	1.45094	4.45242	0.614262	5.36373	2.15849	0	0	2.04715	0.96745	-0.07501
ENSG000C	APOBR	16:284563	3.47257	3.34638	2.2535	0.447297	7.03479	9.10254	5.41342	1.93278	0.349266	13.0483	0.56245	0.811085
ENSG000C	PTP4A3	8:1413899	18.0244	18.696	7.53422	4.59805	4.44939	20.2484	28.2629	6.08886	2.31985	4.27964	0.92535	0.062231
ENSG000C	NFAM1	22:423804	0.332937	2.52949	0	0	0.071466	2.72051	0.006435	0	0	0	0.7891	-0.51667
ENSG000C	MDN1	6:8964249	7.67772	10.1662	2.40507	3.18845	5.49114	5.37025	6.71882	2.3934	3.0967	7.51569	0.7877	0.293053
ENSG000C	NID2	14:519894	0.213996	0.77786	0.145068	0.733556	0.083339	0.552651	0.419608	0	0	0.104044	0.51445	-0.95355
ENSG000C	ZC3HC1	7:1299532	14.9311	15.4142	21.4461	13.3861	19.7423	30.1944	14.3475	12.5588	9.30011	14.1948	0.7014	-0.15875
ENSG000C	E2F5	8:8517722	2.69058	5.0108	1.58825	1.89739	1.24305	1.16781	2.09854	0.894408	0.01892	1.56115	0.24525	-1.2136
ENSG000C	DDX55	12:123602	28.0511	35.1925	47.4736	22.9113	39.4143	35.4716	26.4541	34.7198	15.0625	46.2993	0.55435	-0.31877
ENSG000C	AC010761	17:286483	0	0.558633	0.133633	0.23591	0	0	0.545579	0.741429	0	0.765526	0.39135	1.58775
ENSG000C	RP11-567	2:1478445	0.140277	0.54439	0	1.02339	0.590894	0	0.195359	0.104125	0	0.627819	0.5804	-1.00737
ENSG000C	TAX1BP1	7:2773306	135.51	112.226	104.928	86.8663	114.293	132.507	134.842	83.1368	115.728	139.951	0.85215	0.065812
ENSG000C	RP11-574	10:736448	0.448609	1.13045	0.586306	1.12734	0.19858	0.081609	0.068815	0.44203	0.243556	0.90097	0.397	-1.07269
ENSG000C	LST1	6:3158612	86.4297	27.4176	69.0708	170.131	54.937	66.3959	6.78023	41.5618	112.122	27.9557	0.30435	-0.72598
ENSG000C	C11orf68	11:659168	35.548	39.5028	17.8429	7.98329	23.7633	42.712	42.627	13.0293	12.0521	21.029	0.95175	0.024541
ENSG000C	RIBC1	X:5337414	0.255809	1.77608	0.946805	0.750813	0.410238	0	0	0	0	0.516301	0.1268	-3.0918
ENSG000C	SPINT1	15:408358	1.17869	3.64181	0.213081	0.49257	0	0.759694	4.07294	0.204209	0.025402	0.134696	0.5763	-0.55315
ENSG000C	TAF7	5:1412592	156.293	97.7731	131.277	135.601	147.777	109.267	111.918	117.432	135.039	136.332	0.6638	-0.14043
ENSG000C	PTTG1IP	21:448495	66.8638	62.2659	46.2915	58.9576	52.5026	103.647	41.3444	39.2461	49.208	56.1029	0.9604	-0.01563
ENSG000C	CHP1	15:412308	4.48041	4.31646	28.3246	17.5381	21.1027	3.3879	6.69947	33.7697	28.4969	30.1068	0.43585	0.305573
ENSG000C	RP11-96H	12:463836	2.46402	2.29929	1.37122	1.61288	0.340735	0.530989	0	0.613856	0.354685	0	0.21425	-1.92727
ENSG000C	NUP205	7:1355579	8.70162	15.8863	3.89041	3.66799	5.51186	3.46066	6.51918	7.47925	0.2673	9.66286	0.96515	0.031297
ENSG000C	SLC10A7	4:1462539	8.73561	10.2119	2.22789	5.12599	1.6067	10.4861	4.71608	7.72189	4.90564	2.29361	0.66175	0.260175
ENSG000C	NELL2	12:445082	5.55457	41.0353	5.57522	11.3803	1.25879	2.61916	6.54302	15.2991	7.19927	12.5517	0.63355	-0.22914
ENSG000C	SUN3	7:4798714	0.427228	1.00024	0.491121	0.454698	0.091956	0	0	0.65515	0	0.098443	0.37135	-1.20266
ENSG000C	CTD-2270	16:233884	2.15441	2.24171	2.65121	3.07389	2.9426	0.416402	3.56483	2.42	5.61994	0.669537	0.9615	0.046267
ENSG000C	HMGN2	1:2647244	544.243	821.216	562.838	506.982	388.512	742.162	625.494	598.2	551.513	300.385	0.99795	-0.001
ENSG000C	SBF2-AS1	11:975476	0.995165	0.879602	0.616576	0.205059	0.583111	3.66277	1.80904	1.3456	0.71373	0.289649	0.21365	1.27553
ENSG000C	UBE2J1	6:8932662	31.4201	20.1583	16.2361	28.8046	12.5665	21.8884	17.6	15.5697	23.567	12.8889	0.4318	-0.25613
ENSG000C	RP11-541	10:103877	1.43056	0.451896	1.04985	0.697743	0.960027	0	0	1.75273	0.767384	2.21122	0.96775	-0.02792
ENSG000C	CCR6	6:1669991	38.0782	14.8701	48.1233	42.6752	39.5725	27.2112	20.2238	66.9091	111.286	94.7577	0.09925	0.718404
ENSG000C	SUV39H2	10:148788	2.75644	5.50712	2.84157	2.56855	2.04865	0.393777	8.26785	1.89752	0	1.31738	0.4666	-0.65421
ENSG000C	AC006547	22:200170	1.15812	0.43313	0.682081	0.646924	0	0.668135	0.51741	0.61167	0.574111	0	0.81115	-0.33811
ENSG000C	RARRES3	11:635368	245.789	139.903	401.903	304.497	301.167	248.307	425.678	307.952	308.512	71.9996	0.797	-0.09126
ENSG000C	FAM49A	2:1654945	3.4287	8.59974	3.05903	2.42887	3.11936	6.93552	3.36528	3.54855	7.08394	1.85379	0.4851	0.378933
ENSG000C	SRP72	4:5646691	50.9956	45.9369	36.8821	41.6711	42.8388	49.9223	57.3102	33.8401	44.517	40.0809	0.9331	-0.03335
ENSG000C	RBM10	X:4714221	22.0277	26.3778	12.5223	6.91374	19.8787	15.589	16.6618	7.32253	11.2279	24.202	0.8682	-0.18159
ENSG000C	WDR18	19:984270	45.0654	52.2799	74.7531	71.8186	87.6957	37.7824	72.0088	35.7622	30.863	56.3008	0.0858	-0.59645
ENSG000C	ISG20L2	1:1567218	15.8269	12.1175	23.109	14.3121	17.6325	16.6137	16.2286	16.9648	14.4517	26.004	0.9783	0.010326
ENSG000C	H2AFY	5:1350277	113.912	137.761	99.3391	91.2372	121.37	188.569	107.741	97.0708	88.1563	80.3018	0.87695	-0.05241
ENSG000C	RPS23	5:8197202	429.9	458.736	760.832	487.539	219.078	467.701	430.634	560.058	352.231	44.7962	0.3498	-0.42974
ENSG000C	RP11-603.	12:561181	1.25363	2.6389	0.415274	1.84995	0.569605	3.07534	1.07933	0.506695	0.120725	0.382962	0.6645	-0.53419
ENSG000C	AC074289	2:6409265	1.5369	11.5147	0.151206	0.236456	0.555508	3.07038	7.21176	0	9.5621	0.569901	0.6294	0.75192
ENSG000C	S100A10	1:1519829	449.35	554.868	378.463	429.504	337.725	515.344	565.663	509.951	574.481	98.5076	0.8251	0.07998
ENSG000C	MRPL17	11:668078	21.0515	21.7509	23.7098	22.8396	21.1788	26.251	33.4462	20.8167	10.9464	12.6895	0.6718	-0.20607
ENSG000C	EIF2B5	3:1841340	67.4489	96.5543	37.7533	36.1364	29.9949	52.9271	57.5789	30.9322	39.3238	33.0412	0.38975	-0.26094
ENSG000C	AC018755	19:516119	0.536392	1.73616	0	0	0	5.68819	0	0.870518	0	0	0.5496	1.60414
ENSG000C	SLC6A6	3:1440257	6.64141	5.56947	2.31379	2.88143	2.73216	5.29274	7.79253	6.80124	7.35359	11.5355	0.24725	0.834997
ENSG000C	CD300LF	17:746705	0.248599	6.19244	0.200767	0	0.400297	16.1449	0	0.169689	0	0.071836	0.6619	1.30526
ENSG000C	RP11-10L	4:1027943	1.65649	1.96001	3.08944	2.44525	1.27836	0	1.74808	1.05649	0	0.387943	0.2389	-1.67028
ENSG000C	TAOK3	12:118149	54.0237	64.5904	55.9658	54.3522	82.5847	66.9554	64.2422	38.9235	39.8622	90.1037	0.46305	-0.28808
ENSG000C	SERP1	3:1505419	138.463	80.3071	118.728	137.157	99.415	115.486	86.4149	92.15	93.2697	76.8143	0.49625	-0.38337

ENSG0000	RP11-264I	17:606774	0.76417	0.247712	1.06867	0.38557	0	0	0.601287	0.681217	0.475416	0	0.89495	-0.18002
ENSG0000	DDIT3	12:574601	25.6816	16.9442	62.9948	108.234	25.4118	119.157	158.516	128.13	70.5599	24.9533	0.3169	0.969334
ENSG0000	ALOX5	10:453741	5.08862	22.0688	1.43181	3.55855	1.44706	38.8886	1.77308	6.15139	1.88098	0.39419	0.32375	0.593189
ENSG0000	CAB39	2:2307128	62.1202	56.8591	14.2327	16.1446	12.7048	52.183	41.2455	28.7987	24.2194	26.8549	0.52455	0.1974
ENSG0000	HTT	4:3074680	2.90614	8.87723	1.80863	1.03162	3.39083	7.86435	4.77481	3.13078	2.24793	6.821	0.3758	0.438493
ENSG0000	RHBDD3	22:292598	17.0969	21.2998	12.778	11.2024	16.2879	14.6018	35.5108	12.8059	11.0475	24.7221	0.6906	0.300407
ENSG0000	AC145124	8:1219446	1.04588	3.77473	1.20007	2.37972	1.69124	2.60996	3.02985	1.44979	1.15226	2.52281	0.9705	-0.05164
ENSG0000	RP11-768	12:119334	0	0.696235	0.240512	1.25263	0	0	0	0	0	0	0.098	#NAME?
ENSG0000	NKAP	X:1198958	5.12204	7.12205	7.07844	4.9584	9.5881	9.95419	10.142	5.06725	3.8002	4.76632	0.87195	-0.06105
ENSG0000	PET100	19:762973	73.9693	53.0205	111.934	125.293	25.8165	135.762	114.452	72.8918	54.5452	13.0851	0.89505	-0.08921
ENSG0000	RP11-634I	7:1393394	2.53924	0	2.82887	1.37135	0.786999	2.73899	3.12411	1.93496	0	0.310415	0.9245	-0.1319
ENSG0000	WHSC1L1	8:3822521	58.4259	101.207	48.8514	36.8742	80.6751	88.1016	109.129	58.2956	54.7532	93.012	0.8608	0.092585
ENSG0000	RP11-69M	12:808038	0.999749	0	0.90041	1.38797	0.274872	0	1.35396	0.698152	2.03557	0.14853	0.86465	0.30761
ENSG0000	GANAB	11:626248	58.4684	48.8623	45.2532	16.9579	53.315	22.883	24.3711	62.6656	28.4321	69.2431	0.8472	-0.06431
ENSG0000	CHTF8	16:691055	61.0603	50.7101	34.1396	57.4874	36.6272	52.8934	35.159	28.0923	41.3829	39.2015	0.64215	-0.29334
ENSG0000	DPH6	15:351817	8.09627	17.4465	17.5685	11.987	11.7801	8.40351	8.82783	12.6738	5.68731	4.96048	0.44915	-0.51216
ENSG0000	TRBV29-1	7:1426707	14.7504	49.8405	31.4959	7.3379	14.1453	42.0348	92.1149	5.72909	6.24108	10.6571	0.82555	0.292052
ENSG0000	NSUN5P1	7:7534401	17.9544	41.8947	14.0075	19.3597	8.37925	33.2613	25.2075	26.9389	18.312	24.2713	0.4663	0.539201
ENSG0000	RP11-482I	12:106357	1.53473	0.450532	1.23208	0	0.154439	0	0.438501	0	0	0.253656	0.2981	-2.20234
ENSG0000	DNAL4	22:387057	11.2309	7.97627	12.9868	13.7261	15.9132	10.5885	8.23089	10.8263	13.2159	7.15265	0.82935	-0.33491
ENSG0000	KLRF1	12:982748	36.2528	1.62581	43.0517	100.665	43.6185	1.69162	0.543679	2.78093	11.8589	2.65888	0.00005	-3.52128
ENSG0000	KLC1	14:103553	59.2897	72.2016	59.8811	69.7131	68.1754	80.3372	81.0864	65.6636	58.4951	110.119	0.5345	0.250142
ENSG0000	FAM120C	X:5406832	4.10823	3.58754	3.20233	1.34214	2.64869	2.85022	1.04875	0.869795	0.240968	2.69999	0.49985	-0.98443
ENSG0000	RP11-817	15:753467	0.694841	0.783649	0	0	0.284214	0	0	0.624122	1.2603	0.372096	0.7372	0.643298
ENSG0000	CMPK1	1:4733379	118.322	85.5263	51.2351	54.8589	48.602	96.1878	97.7657	63.9331	60.7253	45.6507	0.8754	0.04639
ENSG0000	ZSWIM7	17:159765	28.9809	33.7542	45.69	40.5866	25.6565	16.3773	40.6578	30.258	43.0367	11.585	0.68715	-0.30623
ENSG0000	ARPC5	1:1836208	263.709	354.475	279.128	209.49	182.244	330.729	286.8	236.233	255.953	88.2584	0.4629	-0.32956
ENSG0000	RP11-160I	17:191119	11.3064	9.71334	2.53599	0.467075	0.346711	0.461134	0	1.54864	1.11996	0.02465	0.38475	-2.56413
ENSG0000	COA4	11:738726	35.217	25.3845	55.6056	51.4014	33.8937	43.26	66.7203	53.8527	39.4505	13.3162	0.9414	0.033731
ENSG0000	CENPU	4:1846496	6.96537	11.4993	2.23707	4.64866	3.26831	13.2647	12.1912	2.83033	7.06317	3.47565	0.56545	0.532799
ENSG0000	AC009404	2:1178339	0.723134	1.14319	0.218505	0	0.158782	0.478009	0.099398	0.445537	1.74874	0.543401	0.57805	0.80959
ENSG0000	SDHAF2	11:614026	64.5728	50.4707	122.622	111.065	82.0169	61.5815	62.1327	85.0186	62.1008	34.5868	0.22645	-0.59385
ENSG0000	CHAMP1	13:114314	2.02343	4.53675	2.2682	6.80639	4.27581	6.70362	5.13514	4.32696	5.61578	2.86831	0.83475	0.241177
ENSG0000	NPIPB13	16:302229	2.73736	5.54503	0.762594	1.12613	0.820131	4.17629	5.10897	1.42187	2.17814	2.433	0.62425	0.462097
ENSG0000	TAF4	20:619354	2.29674	2.00349	0.698629	1.20062	2.00597	2.99837	2.98125	2.00686	0.043102	2.82713	0.5556	0.415433
ENSG0000	MAST4	5:6659636	5.41056	16.239	1.13825	2.09272	1.75725	17.9346	14.8787	4.94511	15.3653	3.95465	0.01065	1.364
ENSG0000	PLXND1	3:1295551	17.3528	2.00339	12.441	13.7045	19.4722	10.8884	3.48511	20.0185	11.1176	39.2991	0.3483	0.462208
ENSG0000	APLF	2:6846756	1.4959	2.28894	1.33249	1.74079	0.831577	0	0.472253	0.224705	0.820518	0.297646	0.0423	-2.19224
ENSG0000	AC000068	22:193306	0.377912	0.08224	0.410413	0.52304	0.6188	0	0	0	0	0.372071	0.3481	-2.09296
ENSG0000	OGFOD3	17:823892	8.81676	14.5366	15.9872	20.6672	24.9059	20.644	24.9671	21.284	17.4857	11.1632	0.96585	0.033964
ENSG0000	RPS7P10	13:216284	0.248986	0.242335	0.477826	0.494301	0.182546	0.328013	0.4713	0.215546	0.25664	0	0.79305	-0.4791
ENSG0000	EXOC8	1:2313327	1.81766	3.31344	1.33752	0.937176	1.62456	0.099505	1.50273	1.40572	0.48701	3.90049	0.68465	-0.39605
ENSG0000	RP11-216I	9:4010582	2.51086	3.40081	1.00218	0.770443	2.11532	1.59172	1.91811	1.89803	3.10703	1.73595	0.79455	0.196439
ENSG0000	BAG3	10:119651	1.93753	18.0257	3.12234	7.66376	3.94961	16.2105	4.91129	7.39061	12.026	15.3894	0.10985	0.84054
ENSG0000	RP11-973I	18:127246	2.01306	1.59037	2.66617	1.71459	0.390675	0.035443	0.532413	0.728526	2.26231	1.49716	0.55525	-0.68084
ENSG0000	WDR62	19:360548	0.728347	1.60026	0.072498	0.118126	0.132905	1.8185	1.90282	0.078322	0	0.265976	0.6614	0.567945
ENSG0000	ZRANB3	2:1350522	2.74646	2.95329	1.81767	1.0116	0.80949	0.058938	3.56492	1.6492	1.41345	0.798513	0.7916	-0.25571
ENSG0000	ZNF226	19:441650	17.608	17.393	22.2645	18.7583	8.38015	13.8261	19.1532	22.746	15.9311	11.8368	0.8304	-0.12858
ENSG0000	MTMR8	X:6426808	2.43345	0.698524	1.05646	0.962795	1.85386	2.94939	0.882908	2.39664	0.868596	1.34886	0.7932	0.173735
ENSG0000	CCL28	5:4337664	1.86076	3.40787	2.98852	2.98505	2.09213	0.77743	4.39164	1.38697	0	1.04254	0.4286	-0.83909
ENSG0000	ETS2	21:388053	4.89575	7.7193	3.14981	2.26496	3.81098	5.79638	4.83833	5.80106	4.48944	4.90924	0.5494	0.288256
[truncated: 2,337,195 more chars]
